# DNA barcode reference library for Iberian butterflies enables a continental-scale preview of potential cryptic diversity

**DOI:** 10.1038/srep12395

**Published:** 2015-07-24

**Authors:** Vlad Dincă, Sergio Montagud, Gerard Talavera, Juan Hernández-Roldán, Miguel L. Munguira, Enrique García-Barros, Paul D. N. Hebert, Roger Vila

**Affiliations:** 1 Institut de Biologia Evolutiva (CSIC-Universitat Pompeu Fabra), Passeig Marítim de la Barceloneta 37, 08003, Barcelona, Spain; 2 Biodiversity Institute of Ontario, University of Guelph, Guelph, N1G 2W1, Ontario, Canada; 3 Institut Cavanilles de Biodiversitat i Biologia Evolutiva (ICBiBE) - Universitat de València, Carrer Catedràtic José Beltrán 2, 46980, Paterna, Spain; 4 Department of Organismic and Evolutionary Biology and Museum of Comparative Zoology, Harvard University, Cambridge, MA 02138, USA; 5 Faculty of Biology and Soil Science, St Petersburg State University, 199034 St. Petersburg, Russia; 6 Universidad Autónoma de Madrid, Department of Biology, Campus Cantoblanco 28049, Madrid, Spain

## Abstract

How common are cryptic species - those overlooked because of their morphological similarity? Despite its wide-ranging implications for biology and conservation, the answer remains open to debate. Butterflies constitute the best-studied invertebrates, playing a similar role as birds do in providing models for vertebrate biology. An accurate assessment of cryptic diversity in this emblematic group requires meticulous case-by-case assessments, but a preview to highlight cases of particular interest will help to direct future studies. We present a survey of mitochondrial genetic diversity for the butterfly fauna of the Iberian Peninsula with unprecedented resolution (3502 DNA barcodes for all 228 species), creating a reliable system for DNA-based identification and for the detection of overlooked diversity. After compiling available data for European butterflies (5782 sequences, 299 species), we applied the Generalized Mixed Yule-Coalescent model to explore potential cryptic diversity at a continental scale. The results indicate that 27.7% of these species include from two to four evolutionary significant units (ESUs), suggesting that cryptic biodiversity may be higher than expected for one of the best-studied invertebrate groups and regions. The ESUs represent important units for conservation, models for studies of evolutionary and speciation processes, and sentinels for future research to unveil hidden diversity.

Evaluations of cryptic species (those overlooked due to their morphological similarity) are gaining momentum^1^, fuelled by recent advances in molecular techniques. Several studies suggest that cryptic species may be surprisingly common in certain taxonomic groups^2^,^3^,^4^, but the actual fraction of biodiversity that they represent in different lineages is subject to debate^1^,^5^,^6^. This mainly stems from the fact that ascertaining the incidence of cryptic species across the diversity of life is a challenging, time-consuming task. Yet, the need to gather comprehensive information about overlooked species is higher than ever, not only because of implications for various fields of research^7^, but also because planetary biodiversity is decreasing at an alarming rate and informed conservation decisions require accurate data. A necessary first step is documenting intraspecific diversity, which in itself is also relevant for the study of evolution, ecology, biogeography and conservation of biodiversity^1^,^8^,^9^.

Short DNA sequences have been used for species identification for more than 20 years^10^, but DNA barcoding^11^, the use of sequence variation in a short, standardized DNA marker to assess biodiversity, has only gained broad adoption within the past decade. Although this approach is not sufficient to verify the specific status of taxa, it provides an overview of variability in the mitochondrial genome which is typically accompanied by similar patterns of divergence in the nuclear genome. With comprehensive geographical sampling, this exercise can highlight potential cases of cryptic species that can subsequently be investigated in more detail. The accumulation of more than three million DNA barcode records (648 base pair sequences of the mitochondrial gene cytochrome *c* oxidase I, *COI*) (http://www.boldsystems.org/) has created the need for algorithmic approaches for species delimitation, approaches which generate results that are particularly useful for phylogenetic community ecology or for groups lacking a strong taxonomy. The Generalized Mixed Yule-Coalescent (GMYC) model^12^,^13^, the Automatic Barcode Gap Discovery (ABGD)^14^, the Poisson Tree Process (PTP)^15^, and the Barcode Index Number system (BIN)^16^ represent four of the most popular approaches. Among these methods, GMYC usually generates the highest number of putative species (i.e. GMYC entities) compared to morphospecies^17^,^18^,^19^. As a consequence, it was adopted to reduce the chance of overlooking potential cryptic species. Based upon phylogenetic and coalescent theory, GMYC defines statistically significant clusters and reports probabilities for the delimitations as a measure of robustness. Thus, resulting entities can be graded according to their GMYC support and minimum inter-entity genetic distance, so that decisions on research and management can be taken objectively. Once phylogenetic significant units have been identified, these can be studied in more detail by targeted studies using multiple data sources, expanding the operational criteria used for species delimitation. It is important to stress that units recovered by GMYC should be regarded as genetically diverged lineages that reflect various evolutionary processes and, in some cases, represent potential cryptic species^20^.

The Lepidoptera, one of the four most diverse insect orders, are arguably the most intensively studied group of invertebrates and more than one million barcode records have been so far generated for this group (http://www.boldsystems.org/). European butterflies in particular are the focus of monitoring schemes and intensive research on distribution, population dynamics, ecology and biogeography, and represent a flagship group for insect conservation efforts^21^,^22^,^23^. Nevertheless, they have been the focus of relatively few DNA barcoding studies^24^,^25^,^26^,^27^,^28^, all with rather limited geographical coverage. Even more surprising, no comprehensive study is available for the Mediterranean region, although it was a major glacial refugium, a fact reflected by the high species richness and endemicity of the modern fauna^29^,^30^.

We assembled a DNA barcode dataset for all 228 species of Iberian butterflies, with an average of 15.4 specimens/species from various geographic regions, including mainland Spain, Portugal, Andorra and the Balearic Islands (Supplementary Fig. 1). Comprising about 50% of the European butterfly fauna (sensu^22^), the Iberian Peninsula is a butterfly diversity hotspot with a fauna representative of the Mediterranean region^31^. We subsequently merged these sequences with all other public records for European butterflies on the Barcode of Life Datasystems (BOLD)^32^ (www.boldsystems.org), creating a dataset with 5782 DNA barcodes from 299 species (ca. 60% of the European butterfly fauna) (Fig. 1). We then employed GMYC to identify phylogenetically significant units and highlight deeply diverged lineages which represent potential cases of cryptic species that require further study (Fig. 2). Thus, we present the first continental-scale estimate for the incidence of cryptic diversity in European butterflies. These results provide important direction for future research, and have broad implications for the biogeography, conservation, taxonomy and ecology of European butterflies.

## Results

### DNA barcoding performance

For the Iberian dataset we obtained 3502 *COI* sequences derived from 228 currently recognized species, with an average of 15.4 specimens per species (Supplementary Fig. 1, Supplementary Table 1). Only one species (*Azanus jesous*) was represented by a single individual. According to our evaluation (see the Methods), DNA sequences for 93.9% of the 228 species were monophyletic, 2.6% were para-/polyphyletic and 3.5% shared barcodes. All species that shared barcodes belonged to the family Lycaenidae: *Polyommatus fabressei - P. fulgens*, *Pseudophilotes baton - P. panoptes*, *Cupido lorquinii-C. minimus* (because of taxon *carswelli*), and *Lysandra caelestissima* - *L. albicans* (due to a potential hybrid between the two species) (Supplementary Figures 2 and 3, Supplementary Table 2). Two of the six Iberian butterfly families barcoded (Pieridae, Papilionidae) showed 100% monophyletic species, while, at the opposite pole, 86.5% of the Lycaenidae were monophyletic.

**Figure 1:**
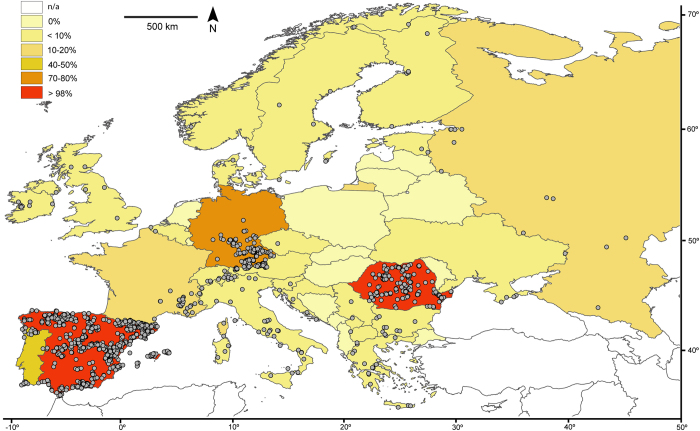
Map of Europe illustrating the sampling effort and taxon coverage (percentage of the species present in each country with DNA barcode records). The distance scale corresponds to longitudinally-oriented distances only. The map was generated using Quantum GIS 1.8.0 based on a map from Natural Earth.

The European dataset consisted of 5782 sequences with representation for 299 species, with an average of 19.3 specimens per species. Compared to Iberia, this dataset included more geographic and taxon coverage (71 additional species) (Fig. 1, Supplementary Table 1). DNA sequences for 84.6% of the 299 species were monophyletic, 8.0% were para-/polyphyletic and 7.4% shared barcodes. Only the family Papilionidae showed 100% monophyletic species while the most notable decreases in monophyly occurred in the Nymphalidae (83.7% versus 96.8% for Iberia) and the Pieridae (88.6% versus 100% for Iberia) (Supplementary Figures 2 and 4, Supplementary Table 2).

### Patterns of genetic diversity in European butterflies

**Figure 2:**
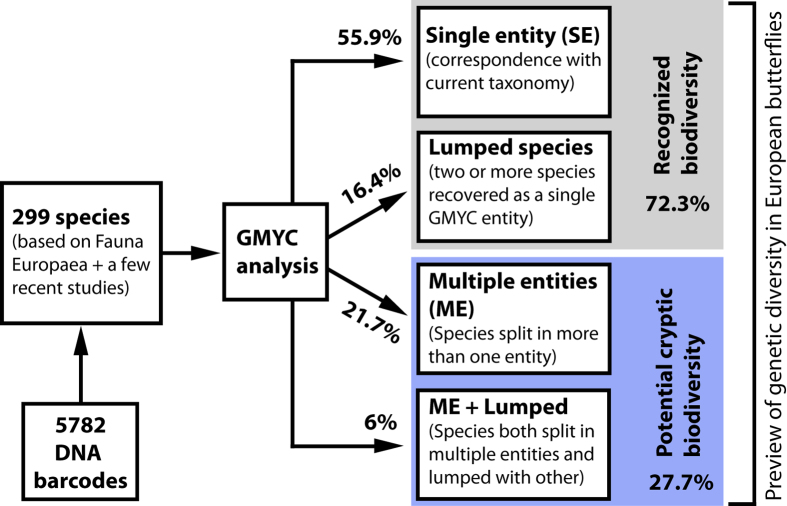
Workflow used to assess recognized biodiversity and potential cryptic biodiversity in European butterflies, and percentages obtained in each category.

**Figure 3:**
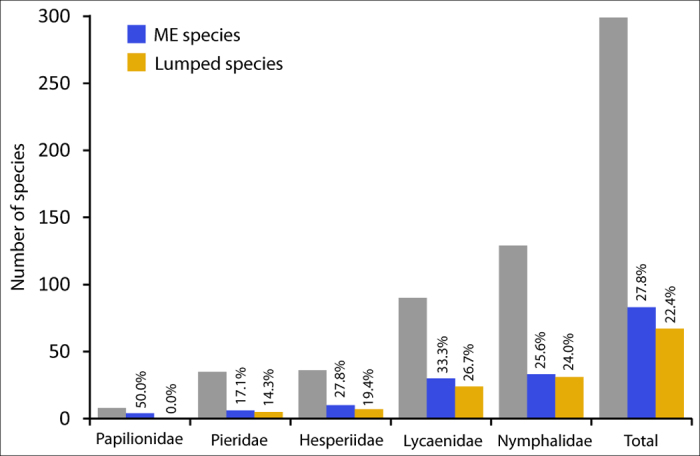
Species distribution by family and the number of species recovered as multiple entities (ME) by the ST-GMYC analysis, or lumped with another species. Percentages of ME and lumped cases are shown above the bars. The single representative of the Riodinidae (*Hamearis lucina*), not illustrated in the graph, but counted in the total assessment, was recovered as a single entity.

The single-threshold GMYC (ST-GMYC) generated a higher score compared to the multimodel one (MM-GMYC) based on Akaike’s Information Criterion (AIC), and the subsequent analysis of GMYC entities was based on the ST-GMYC model. Depending on the method used and the taxon studied, the estimated values for haploid effective population size (N_e_) varied between 4 × 10^4^ and 1.8 × 10^7^, with an average of 1.6 × 10^6^, and the speciation rate (SR) was 0.0455 when considering species based on Fauna Europaea and 0.0473 when considering GMYC entities. Such low values for SR compensate for the rather high N_e_ and their product (N_e_ x SR) ranged from 1.8 × 10^3^ to 8 × 10^5^, with an average of 7 × 10^4^, falling within the range found to be optimal for GMYC performance (see^33^ for details). Because regionally oriented biodiversity surveys typically include very genetically divergent species and a small proportion of closely related species compared to taxon oriented surveys, they should result in datasets with parameters more adequate for GMYC analyses. The ST-GMYC analysis of the full dataset of 1971 haplotypes representing 299 species recovered 55.9% (167 species) as single entities (SE), 16.4% (49 species) were lumped with another species, 21.7% (65 species) were split into two to four entities (ME) and 6% (18 species) were split into multiple entities, but some of these entities were lumped with other species. Overall, 27.7% (83 species) involved ME, indicating potential cryptic biodiversity (Figs 2, 3). These 83 species generated 64 more GMYC entities than the number of recognized species (363 entities versus 299 species, representing an increase of 21.4% compared to the recognized species) (Supplementary Figures 5 and 6, and see Supplementary Table 3 for further details on the GMYC results).

Cases of ME were detected within five of the six butterfly families, but the sole representative of the Riodinidae, *Hamearis lucina*, was recovered as a single entity. The highest percentage of ME was found in the Papilionidae (50.0%) followed by the Lycaenidae (33.3% of the species) (Fig. 3).

**Figure 4:**
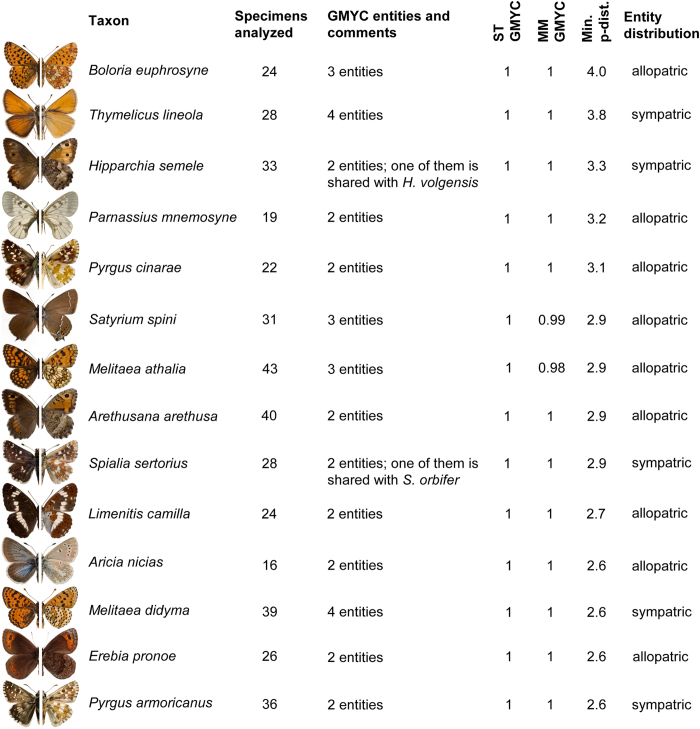
Fourteen species split into multiple ST-GMYC entities with a minimum genetic distance of at least 2.5%. Representative specimens (not drawn to scale) of each species are shown in dorsal view (left side) and ventral view (right side). The ST-GMYC and MM-GMYC supports for entity differentiation are shown. The sympatry/allopatry relationship among conspecific entities is shown based on a 50-km distance threshold. If a species was split in more than two entities, the data presented refer to the entity that was genetically most distant to the others. Specimen photos by R. Vila.

For the 83 species with ME, the minimum p-distance to the nearest conspecific entity (or containing conspecifics) varied from 0.3% to 4.0%, and averaged 1.5% (Supplementary Table 4). Fourteen of the ME species displayed a minimum inter-entity p-distance of more than 2.5% and five of these species included entities detected in sympatry (Fig. 4).

**Figure 5:**
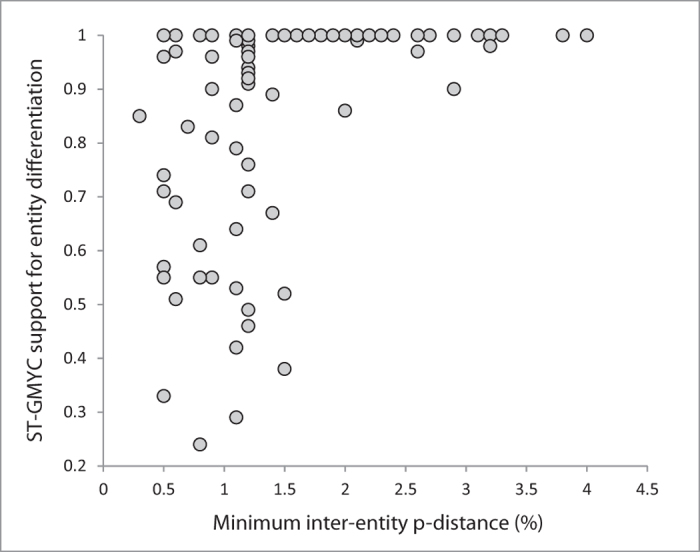
Bivariate scatter plot illustrating the relationship between minimum genetic divergence (%) among conspecific entities and the ST-GMYC supports for entity differentiation. ST-GMYC supports rose abruptly over 1.5% divergence, but high support values were also achieved for some p-distances under 1%.

The ST-GMYC and MM-GMYC entity supports were very variable (between 0.24–1.00 for ST-GMYC and between 0.25–1.00 for MM-GMYC) for entities displaying up to 1.5% genetic divergence. When divergence was higher than 1.5% (the average value for all the MEs), both supports rose abruptly, usually exceeding 0.95 (only two cases between 0.85 and 0.9) (Fig. 5, Supplementary Fig. 7).

### The case of ***Iphiclides podalirius***

**Figure 6:**
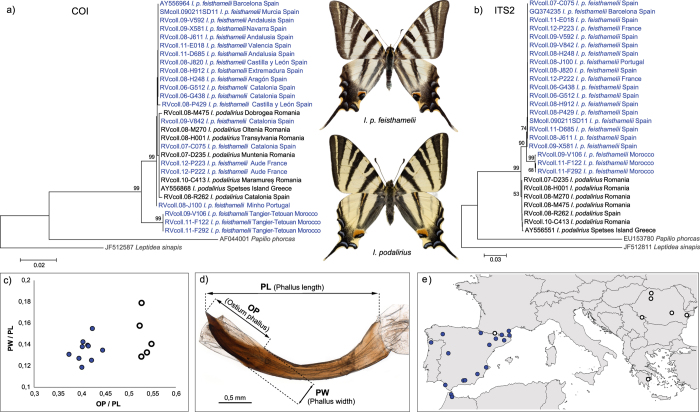
The taxa *Iphiclides p. podalirius* (in black) and *I. p. feisthamelii* (in blue) are differentiated morphologically and genetically, but in Europe they share DNA barcodes due to an apparently fixed mitochondrial DNA introgression from *podalirius* into *feisthamelii* in the Iberian Peninsula. (**a**) Neighbor-joining tree based on DNA barcodes of *podalirius* and *feisthamelii*: only the Moroccan specimens form a distinct clade. (**b**) Neighbor-joining tree based on nuclear *ITS2* sequences of *podalirius* and *feisthamelii*: with the exception of one specimen from the northern Pyrenees, all specimens from Iberia, south-western France and Morocco form a distinct clade composed of *feisthamelii*. (**c**) Results of linear morphometrics for elements of the phallus (phallus width, phallus length and ostium phallus). The morphological separation between *podalirius* (black circles) and *feisthamelii* (blue dots) is in agreement with the nuclear marker (*ITS2*). (**d**) Phallus of *Iphiclides* indicating the measurements performed. (**e**) Sampling localities for the sequenced *Iphiclides* specimens and taxon assignment based on morphology and the nuclear marker *ITS2*. The map was generated using Quantum GIS 1.8.0 based on a map from Natural Earth. Specimen photos by R. Vila.

The GMYC analysis for *I. podalirius* included specimens from Iberia and south-western France belonging to the subspecies *feisthamelii*, which has also been suggested to represent a distinct species by some authors^34^,^35^. It also included specimens of nominotypical *podalirius* from Romania (Supplementary Table 5). All these specimens were recovered as a single highly supported GMYC entity (support 1 for both the ST- and MM-GMYC and maximum within entity p-distance = 0.5%), without support for *feisthamelii* as an independently evolving lineage (Supplementary Figures 5 and 6). When the dataset was extended with additional samples from southern Greece (one specimen), north-eastern Spain (one specimen) and northern Morocco (three specimens), we found that the three north African specimens were recovered as a distinct *COI* lineage as illustrated in a neighbor-joining tree (2.1% minimum p-distance to the European specimens) (Fig. 6a). Data from one nuclear marker (*ITS2*) showed that the Iberian and south-western France specimens clustered with those from Morocco and were distinct from the Romanian and Greek individuals (Fig. 6b). Morphological differences matched the pattern revealed by the nuclear marker (Fig. 6c,d, Supplementary Table 6).

## Discussion

Our results showed that a very high proportion (93.9%) of Iberian butterfly species were recovered as monophyletic, indicating that they can be unambiguously identified to a species level using DNA barcodes. When the geographical and taxon coverage was extended across much of Europe (Fig. 1), the overall levels of monophyly decreased to 84.6% (Supplementary Fig. 2, Supplementary Table 2). Many of the paraphyletic species are likely to be diagnosable as well since they displayed well-diverged lineages with respect to their nearest neighbour (e.g. *Erebia pronoe*, *Spialia sertorius*) (Supplementary Fig. 4). Some of the few cases where resolution is only possible to a triplet or quartet, such as species in the genus *Lysandra*, are known for a relatively high frequency of introgression events^36^. Studies assessing the effects of increased geographical coverage on DNA barcoding are scarce for large faunas. In Eurasian butterflies, for example, only one study has addressed this issue with a dataset on central Asian species^37^. In contrast to our findings, increasing geographical coverage in central Asian butterflies had little effect on the ability of DNA barcodes to discriminate species. This discrepancy could be caused by the difference in sampling between the above-mentioned study (2.9 specimens/species) and the current study (19.3 specimens/species). Furthermore, the complex local orography in the Mediterranean region, especially in the Iberian Peninsula, in conjunction with complex patterns of population isolation and reconnection throughout the Pleistocene, has created a mosaic of genetic diversity in this area (e.g.^38^,^39^). For example, we found that some boreo-alpine taxa such as *Aricia nicias*, *Erebia pandrose* and *Pyrgus andromedae* display more sequence divergence at COI between populations in the Alps and the Pyrenees (separated by ca. 450 km) than between Scandinavia and the Alps (separated by over 1200 km).

Overall, the resolution delivered by the DNA barcode library now available for Iberia and Europe is high enough for numerous applications because most specimens can be identified to a species or at least to a species-pair. Features such as the ability to identify preimaginal stages and to document food webs or host-parasitoid interactions highlight the advantage of rapid DNA-based assessments. Furthermore, the establishment of a comprehensive DNA barcode library for Iberian butterflies covers a key region in the Mediterranean and, together with the data available for regions such as Romania^25^ and Germany^26^ provides the basis for the development of an identification system targeting particular geographical areas, maximizing identification success. This capacity for DNA-based identifications is a valuable resource to support implementation of the new European biodiversity strategy focusing on the main drivers of biodiversity loss^40^.

In addition to creating a tool for DNA-based specimen identification, the Iberian dataset coupled with GMYC analysis permitted the first large-scale assessment of genetic patterns and potential cryptic diversity in European butterflies. Although European butterflies are among the best-studied invertebrate groups and considerable efforts have been devoted towards revealing hidden diversity, recent studies continue to report new cryptic species^41^,^42^,^43^,^44^. Conversely, some previously accepted species have been shown not to deserve their taxonomic status^45^. The ST-GMYC analysis revealed that 83 (27.7%) species in our dataset, including representatives from the five main European butterfly families, were split into two to four entities (Fig. 3, Supplementary Table 3, Supplementary Annex 1), that were mapped to facilitate the visualization of spatial genetic patterns (Supplementary Annex 2). In absolute terms, the GMYC analysis recovered 64 more entities than the number of species currently recognized by Fauna Europaea. Thus, the high number of species split in multiple entities by the GMYC model and the complex patterns detected in some cases that were both split and also lumped with another species, suggest that much research is needed even for one of the best studied invertebrate groups such as European butterflies.

The ST-GMYC model also revealed some potential cryptic species with low levels of genetic differentiation (e.g. as low as 0.3% in the case of *M. athalia*), with high support (Fig. 5, Supplementary Fig. 7, Supplementary Table 4). At the opposite pole, 14 ME species displayed high levels of genetic divergence (more than 2.5%) between conspecific ST-GMYC entities (Fig. 4) and thus represent particularly likely cases of overlooked taxa that require further study. The ST-GMYC model also recovered as distinct entities well-confirmed cryptic species that have been overlooked for centuries, such as the triplet *Leptidea sinapis*, *L. reali* and *L. juvernica*^42^ as well as *Polyommatus icarus* and *P. celina*^41^. However, the same analysis also lumped a number of recognized species, several of which had, and some still have, debated taxonomical status (e.g. *Pieris napi - P. bryoniae*, *Polyommatus ripartii - P. nephohiptamenos*, *Erebia medusa - E. polaris*, *Kretania pylaon - K. sephirus* etc.) (Fig. 2 and see Supplementary Table 3 and Supplementary Annex 1 for further comments).

It is also worth noting that a series of the ME cases included populations that were sometimes treated as distinct species by various authors. Interestingly, our results for some of these cases did not always fully correspond with current taxonomic views, often highlighting more complex situations than previously recognized (see Supplementary Annex 1 for a brief discussion of each case). Such examples are: *Arethusana arethusa* (due to taxon *boabdil*), *Coenonympha pamphilus* (due to taxon *lyllus*), *Melitaea athalia* (due to taxon *celadussa*), *Hipparchia hermione* (due to taxon *genava*) and *Cupido minimus* (due to taxon *carswelli*).

Obviously, conclusions cannot be drawn based on a single genetic marker, but these overall patterns highlight the novelty of the results provided by the genetic survey as an alternative view to traditional (mainly) morphology-based taxonomy and call for more detailed studies to clarify the reasons behind a strong or, conversely, a lack of clear differentiation in mitochondrial DNA. The case study on *Iphiclides* (Fig. 6) further demonstrates that estimates of cryptic biodiversity are dependent on geographic and gene coverage and that more cases will likely emerge with increased sampling.

While species are the standard currency employed to measure biodiversity and to develop conservation measures, the recognition of biological species is, as exemplified above, complicated by operational issues and by introgressive hybridization. As an alternative, the concept of conserving genetic diversity is receiving growing attention in biodiversity studies^8^. Extinction is not only the loss of species, but is also preceded by the erosion of genetic diversity, which can result in the loss of potentially useful traits (e.g. resistance to parasites). Thus, even if many of the GMYC entities detected by our analysis do not represent cryptic species, they could still be Evolutionary Significant Units (ESUs)^46^ that can be highly relevant for fields such as phylogeography and nature conservation. For example, as shown by^47^, the effects of climate change can be much more accurately assessed when based on patterns in mitochondrial DNA instead of morphospecies. The present data will aid the prioritization for conservation of populations that collectively represent most of the genetic diversity in each taxon. As well, it will aid the selection of genetically similar populations for reintroductions, avoiding the risk of mixing potentially incompatible lineages. A recent study on European butterflies has also highlighted the unique properties of the cryptic fraction of butterfly diversity involving original qualitative aspects (influence on the beta-diversity turnover) that can affect ecological and biogeographical studies^7^. Such results underline the necessity to thoroughly document cryptic diversity at both intra- and interspecific levels. Overall, the presence of both deeply diverged lineages and cases of lumping suggest interesting historical and evolutionary processes that, through further research, may illuminate the functioning of natural populations and species.

European butterflies also include numerous model taxa for biogeography^38^,^48^,^49^, ecology^21^ and speciation^50^ and are intensively used as bioindicators and as flagship group for invertebrate conservation efforts^22^,^51^,^52^. As a consequence, any change in their taxonomy and any improvement of our knowledge will have consequences for both research projects and conservation policies. As^53^ noted, superficial taxonomic decisions may jeopardize an intensively studied system such as European butterflies. Given the alarming rates of global biodiversity loss^54^,^55^ and the limited resources available, the exploration of biodiversity through large-scale molecular approaches such as DNA barcoding combined with automated methods of ESU delineation can provide valuable guidelines for future efforts.

Studies assessing levels of potential cryptic diversity in speciose groups on a large geographic scale are rare and the results are not directly comparable due to unequal sampling efforts and different methods employed. Nonetheless, a study on North American birds found that about 2% of the 643 species in the dataset contained lineages displaying at least 2.5% divergence^56^. By comparison, 4.7% of the species in our dataset matched this criterion (Fig. 4), suggesting a considerably higher proportion of potential cryptic diversity in European butterflies. Another study on freshwater fishes from the Mediterranean area reported up to 64 potential cryptic species detected as GMYC entities in a dataset of 498 species^57^. This finding matches the increase of 64 GMYC entities identified in our dataset compared to currently recognized species, which represents 12.9% compared to the 21.4% that we obtained. Obviously, there is a critical need for more large-scale studies that, besides improving knowledge on biodiversity, can provide accurate answers to the ongoing debate about the global proportion and distribution of cryptic diversity^1^,^5^,^6^.

DNA barcoding combined with automated methods for species delineation represents a cost-effective, rapid approach that is a powerful first step for exploring cryptic biodiversity across wide geographical areas. This is especially true for poorly studied taxonomic groups and for those with few diagnostic characters, such as numerous invertebrates.

## Methods

### Sampling and collection data

We gathered over 10,000 specimens that provided coverage for the 228 butterfly species belonging to the six families that occur in the Iberian Peninsula (Spain, Portugal and Andorra, including the Balearics) according to Fauna Europaea. To provide a good representation of intraspecific genetic variability, these samples were collected from hundreds of localities across the Iberian Peninsula (Supplementary Fig. 1, Supplementary Table 1) and a subset of these specimens was selected for barcode analysis (see the Results). Butterfly bodies were stored in 2 ml polypropylene tubes with 100% or 96% ethanol and kept at −20° C, while the wings were detached from the body and kept in glassine envelopes as vouchers. A small percentage of the *COI* sequences (usually highly localized species) were obtained from dried specimens, most of which were less than ten years old.

The Iberian dataset was expanded to 5782 sequences by including records from several other regions of Europe (Fig. 1, Supplementary Table 1). Romania and southern Germany have been intensively analysed in previous studies^25^,^26^, while the other regions of Europe have seen less investigation. Because one source project (“Lepidoptera of Central Asia” - LOWA) contained both European and Asian specimens, we filtered the data by setting the southeastern limit of Europe to 50° long. E and 43.5° lat. N, between the Black Sea and the Caspian Sea, just north of the Caucasus Mountains. The European dataset was compiled by adding 363 complementary sequences from various European countries that were produced either specifically for this study, or were available from previous publications by the authors. We also used previously published DNA barcode data from the following projects publicly available on BOLD: “Butterflies of Romania” (EZROM, 1363 sequences)^25^, “Lepidoptera of Romania – 2GenBank” (EOEME, 18 sequences)^58^, “Fauna Bavarica - Lepidoptera Rhopalocera” (FBLRH, 406 sequences)^26^, European arctic-alpine Lepidoptera (DATASET-AALE1, 55 sequences)^28^, “Lepidoptera of the Alps 3” (PHLAC, 8 sequences)^27^ and “Lepidoptera of Central Asia” (LOWA, 67 sequences)^37^. We preferentially used DNA barcodes from public projects on BOLD because this platform aids both the verification of specimen identifications and an assessment of the quality of the electropherograms. We used sequences that were greater than 400 bp long to allow the inclusion of 407 bp-long amplicons obtained with a primer set often used when the standard Lepidoptera primers failed to recover a full barcode, usually because of DNA degradation in older specimens (25 and references therein). Five sequences from already published BOLD projects were reedited: *Aglais urticae* (EZROM597-08), *Brenthis hecate* (EZROM-288-08), *Boloria napaea* (GWOSA776-10), *Boloria thore* (PHLAB292-10) and *Carcharodus lavatherae* (EZROM311-08). One *Eumedonia eumedon* (FBLMW307-10) and one *Polyommatus dorylas* (GWORA2454-09) from published BOLD projects were not included in the analyses because inspection of electropherograms revealed their poor quality. All data have been uploaded to the public dataset “Iberia-Europe” (DS-IBEUR, dx.doi.org/10.5883/DS-IBEUR) on BOLD at www.barcodinglife.org.

### Sequence analyses, phylogenetic inference and Generalized Mixed Yule-Coalescent (GMYC) models

DNA extraction, amplification and sequencing of the *COI* fragment followed standard protocols for Lepidoptera^25^ (and references therein). A 658-bp fragment of *COI* was targeted for amplification using the primers LepF (5′-ATTCAACCAATCATAAAGATATTGG-3′) and LepR (5′-TAAACTTCTGGATGTCCAAAAAATCA-3′). Samples that did not produce a PCR product with the primers LepF and LepR were amplified with the primers LepF and Enh_LepR (5′-CTCCWC CAGCAGGATCAAAA-3′), which amplify a 609-bp fragment of *COI*. If this approach failed, we used two primer combinations that amplify shorter overlapping fragments: LepF + MH-MR1 (5′-CCTGTT CCAGCTCCATTTTC-3′) (307-bp amplicon) and MH-MF1 (5′-GCTTTCCCACGAATAAATAATA-3′) + LepR (407-bp amplicon). Sequences were edited in CodonCode Aligner 3.0 or in Geneious Pro 4.7.5 (Biomatters, http://www.geneious.com/) and assembled using the latter.

The use of the Bayesian software BEAST for phylogenetic inference, as well as the removal of identical haplotypes, have been shown to optimize GMYC analysis^18^,^20^. Thus, the European dataset was collapsed to 1971 haplotypes under a conservative approach so that, where applicable, sequences that were shorter than 658 bp and/or included ambiguities were assigned to multiple potential haplotypes. BEAST 1.7.4^59^ was used to reconstruct a reference ultrametric phylogenetic tree. Four independent chains were run for 15 million generations. The substitution model was set to GTR + I + G with four gamma rate categories. A strict clock and coalescent tree priors were used. Values were sampled every 10% of the run length and convergence was inspected in Tracer v.1.5^60^. The GMYC method was applied to the resulting tree using the “splits 1.0–11” R package^61^. The single-threshold (ST-GMYC) (Supplementary Fig. 5) and the multimodel (MM-GMYC)^62^ (Supplementary Fig. 6) approaches were evaluated, and GMYC support values based on the Akaike’s Information Criterion (AIC) were calculated for tree nodes following the approach described by^63^.

The suitability of our dataset to fit GMYC models was evaluated according to previous findings based on empirical^20^ and simulated datasets^13^,^33^. On one side, the sampling strategy followed guidelines for regional biodiversity surveys designed to capture maximum intraspecific divergences and to maximize taxon coverage. In general, species in our dataset are represented by similar numbers of specimens (the average number of specimens per species in the European dataset was 19.3, and 75.3% of the species were represented by at least ten specimens), typically including phenotypically differentiated populations from a variety of habitats. On the other side, because the accuracy of the GMYC models is dependent on the scaling parameters for both the speciation and coalescent parts, estimates of speciation rate (SR) and haploid effective population size (N_e_) were used as a test for data suitability as suggested by simulation studies^33^. N_e_ and SR were estimated for both the currently recognized species according to Fauna Europaea and for the resulting GMYC cluster delimitations. For N_e_ calculations, π for synonymous sites, and slow (0.0075 per site per Myr) and fast (0.0115 per site per Myr) substitution rates reported for *COI* in invertebrates were used for those species represented by at least 10 individuals. For SR calculation, we used the R routine bd.ms in the “geiger” package considering an age of 110 Myr for the Papilionoidea tree root^64^.

For the case study on *Iphiclides podalirius podalirius* and *I. p. feisthamelii*, we used 23 *COI* sequences from the European dataset and sequenced three other specimens from northern Morocco (using the DNA barcoding protocols described above). A nuclear marker (the internal transcribed spacer 2 - *ITS2*), was sequenced for all 26 specimens analysed for *COI*. Two specimens of *I. p. podalirius* from Greece and Spain possessed both *COI* and *ITS2* sequences in GenBank and these records were added to the dataset (Supplementary Table 5). All 28 *Iphiclides* specimens analysed (including *ITS2* sequences produced for this study) were included in the dataset “Iphiclides” (DS-EUIPHI, dx.doi.org/10.5883/DS-EUIPHI) publicly available on BOLD at www.barcodinglife.org.

A 699–728 bp fragment at the 5′ end of *ITS2* was amplified by polymerase chain reaction using the primers ITS3 (5′-GCATCGATGAAGAACGCAGC-3′) and ITS4 (5′-TCCTCCGCTTATTGATATGC-3′)^65^. Double-stranded DNA was amplified in 25 μl volume reactions: 14.4 μl ultra-pure (HPLC quality) water, 5 μl 5X buffer, 2 μl 25 mM MgCl_2_, 0.5 μl 10 mM dNTP, 0.5 μl of each primer (10 mM), 0.1 μl Taq DNA Polymerase (Promega) and 2 μl of extracted DNA. The typical thermal cycling profile was: 95 °C for 45 seconds, 51 °C for 60 seconds and 72 °C for 60 seconds, for 40 cycles. *ITS2* sequence editing and alignment was done using Geneious Pro 4.7.5. MEGA 5^66^ was used to produce neighbor-joining (NJ) trees for *COI* and *ITS2* and to perform bootstrap analysis (100 replicates). *COI* and *ITS2* sequences for *Papilio phorcas* and *Leptidea sinapis* from GenBank were used as outgroup (Supplementary Table 5).

All sequences obtained in this study have been deposited in GenBank (see Supplementary Tables 1 and 5, as well as the DS-IBEUR and DS-EUIPHI public datasets on BOLD).

### DNA barcoding performance assessment

To facilitate the visualization of genetic distances, bootstrapped NJ trees (built with MEGA 5, see above) were produced using uncorrected p distances^67^,^68^. The NJ trees were based on unique haplotypes: 1264 haplotypes for the Iberian dataset, and 1971 haplotypes for the European dataset. The performance of DNA barcodes in species identification at Iberian and European scales was assessed based on the following categories: (1) monophyletic, (2) para- or polyphyletic and (3) species sharing DNA barcodes (Supplementary Figures 2–4).

### Morphology examination

Strong effort was made to ensure the correct morphology-based identification of each specimen. Genitalia were examined where external morphology was considered insufficient for reliable identification. For the case study on *Iphiclides*, we performed linear morphometrics of the male genitalia. Genitalia were prepared for all 15 specimens that were also sequenced for *COI* and *ITS2* (Supplementary Table 6), according to the following protocol: maceration for 20 minutes at 100 °C in 10% potassium hydroxide, dissection and cleaning under a stereomicroscope and storage in 0.5 mL tubes with 70% ethanol. The genitalia were slightly pressed under a cover slip and were photographed in a thin layer of 50% ethanol under a Leica Z16 APO macroscope equipped with a Leica DFC500 digital camera for photomicrography. Measurements were based on digital photographs using the ImageJ software and three elements of the genitalia were measured: ostium phallus length (OP), phallus width (PW) and phallus length (PL). The resultant measurements are provided in Supplementary Table 6.

### Defining species and potentially cryptic taxa

Although European butterflies are among the best-studied invertebrates, recognition of species is not always straightforward, as it has been based on different approaches for various taxa. Some species have been defined based on their external morphology, while detailed morphological, molecular, and behavioural analyses have been needed in other cases^69^. In this study, 299 species were recognized based on the checklist of the Fauna Europaea Project^70^ (www.faunaeur.org), which gathers widely accepted views of the scientific community, with a few additions according to recent publications (see Supplementary Annex 1). We then compared the 299 species with the entities recovered by the ST-GMYC analysis and classified each of them into one of three main categories (see Fig. 2 for a schematic representation of the workflow): 1) Single entity (SE): species that were recovered as a single entity, indicating correspondence with current taxonomy. 2) Lumped: specimens of two or more species that were recovered as a single ST-GMYC entity. 3) Multiple entities (ME): species that were split in two or more ST-GMYC entities, which represent potential cryptic biodiversity.

For species involving ME, entities were considered as sympatric if they were separated by less than 50 km. To facilitate the visualization of spatial genetic patterns, the ME cases are shown on maps in Supplementary Annex 2.

## Additional Information

**How to cite this article**: Dincă, V. *et al.* DNA barcode reference library for Iberian butterflies enables a continental-scale preview of potential cryptic diversity. *Sci. Rep.* **5**, 12395; doi: 10.1038/srep12395 (2015).

## Supplementary Material

Supplementary Information
