# Supplementary material for: DNA barcode reference library for Iberian butterflies enables a continental-scale preview of potential cryptic diversity
Source: Sci Rep. 2015 Jul 24;5:12395. doi: 10.1038/srep12395 (PMC4513295; doi:10.1038/srep12395)
Supplement: Supplementary Information [file srep12395-s1.pdf]

Supplementary Information

DNA barcode reference library for Iberian butterflies enables a continental-scale preview of potential cryptic diversity

Vlad Dincă, Sergio Montagud, Gerard Talavera, Juan Hernández-Roldán, Miguel L. Munguira, Enrique García-Barros, Paul D. N. Hebert & Roger Vila

---

Table of contents

Supplementary Figure 1 | Iberian sampling 2

Supplementary Figure 2 | DNA barcoding performance 2

Supplementary Figure 3 | NJ tree of Iberian samples 3

Supplementary Figure 4 |NJ tree of European samples 7

Supplementary Figure 5 | ST-GMYC 12

Supplementary Figure 6 | MM-GMYC 14

Supplementary Figure 7 | MM-GMYC supports vs. p-distance 16

Supplementary Table 1 | Samples used in the study 17

Supplementary Table 2 | DNA barcoding performance 134

Supplementary Table 3 | ST-GMYC results 145

Supplementary Table 4 | Species involving ME 159

Supplementary Table 5 | *Iphiclides* specimens analysed 166

Supplementary Table 6 | *Iphiclides* morphometry 168

Supplementary Annex 1 | Discussion of split or lumped taxa 169

Supplementary Annex 2 | Maps of species with ME 197

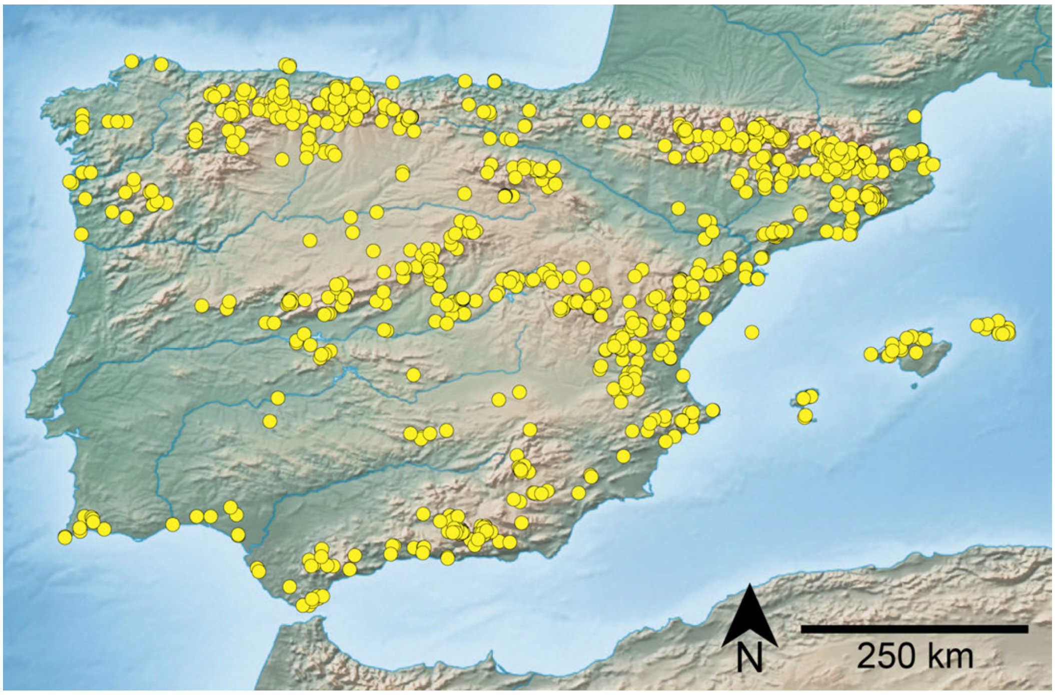

Supplementary Figure 1 | Map of the Iberian Peninsula (Spain, Portugal and Andorra) displaying the sites from where specimens were sampled and successfully barcoded. The map was generated using gvSIG 2.2.0-2313 (http://www.gvsig.org), based on a relief map from Natural Earth (free vector and raster map data at www.naturalearthdata.com).

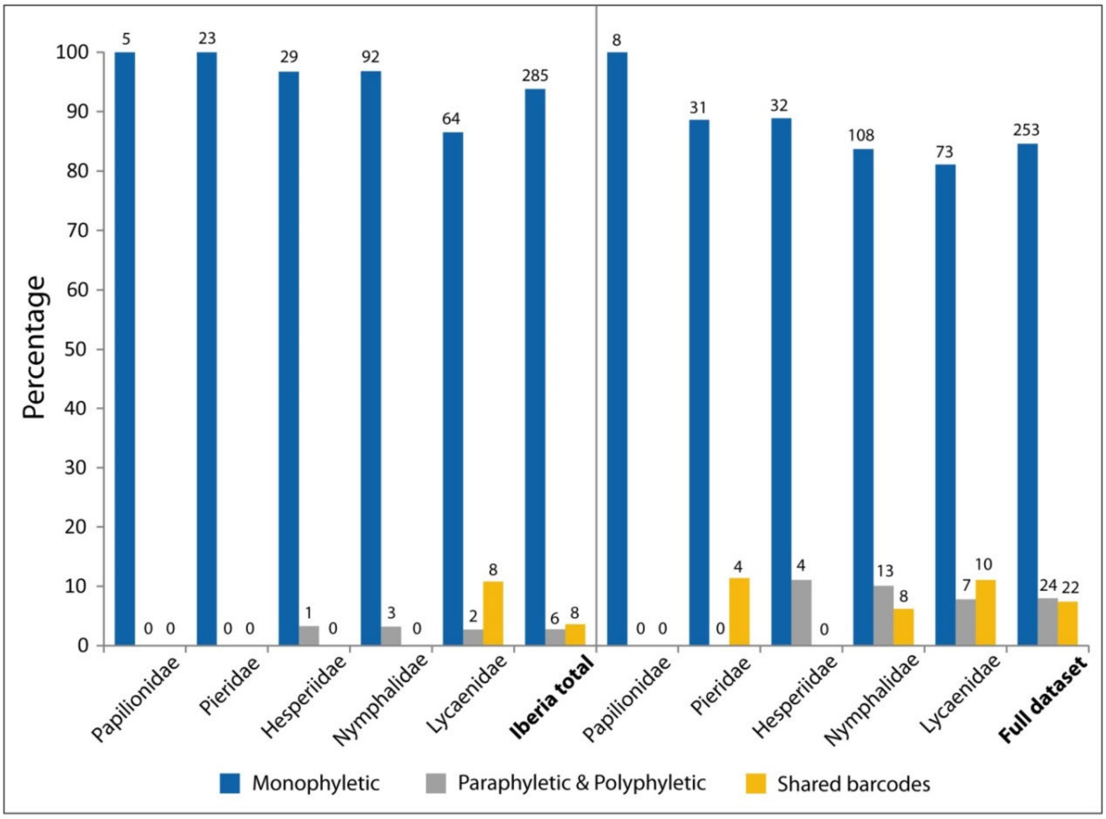

Supplementary Figure 2 | Comparative DNA barcoding performance based on taxon clustering in a neighbour-joining tree for the Iberian dataset and the full dataset. Overall identification success decreases when the number of taxa and geographical scale are increased. The values above the bars represent the total number of species for each partition. The single representative of the Riodinidae family (*Hamearis lucina*), was monophyletic.

Supplementary Figure 3 | Neighbor-joining tree based on 1264 haplotypes inferred from 3502 barcoded specimens (228 species) of Iberian butterflies. Bootstrap support values (> 50%) based on 100 replicates are indicated.

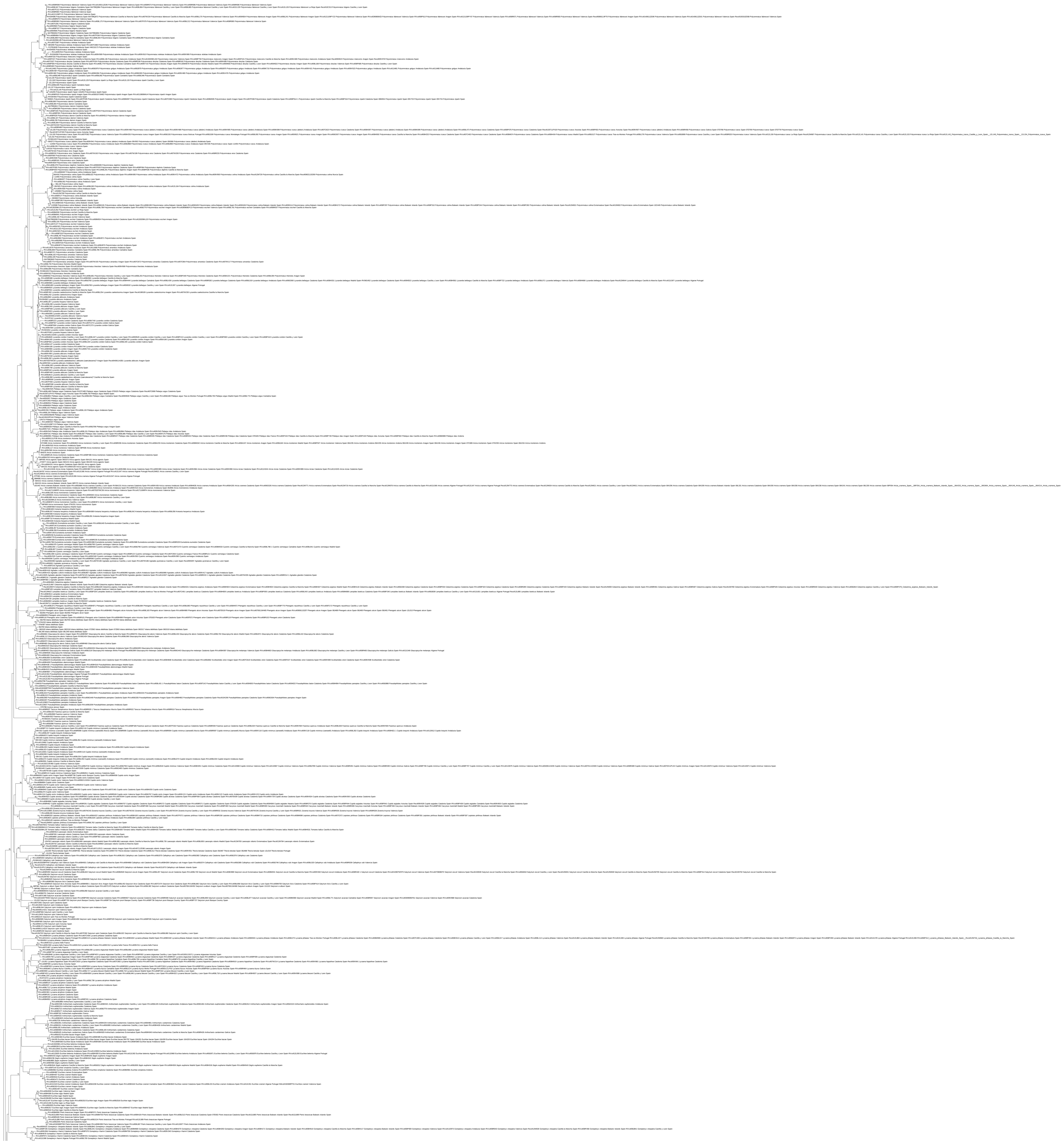

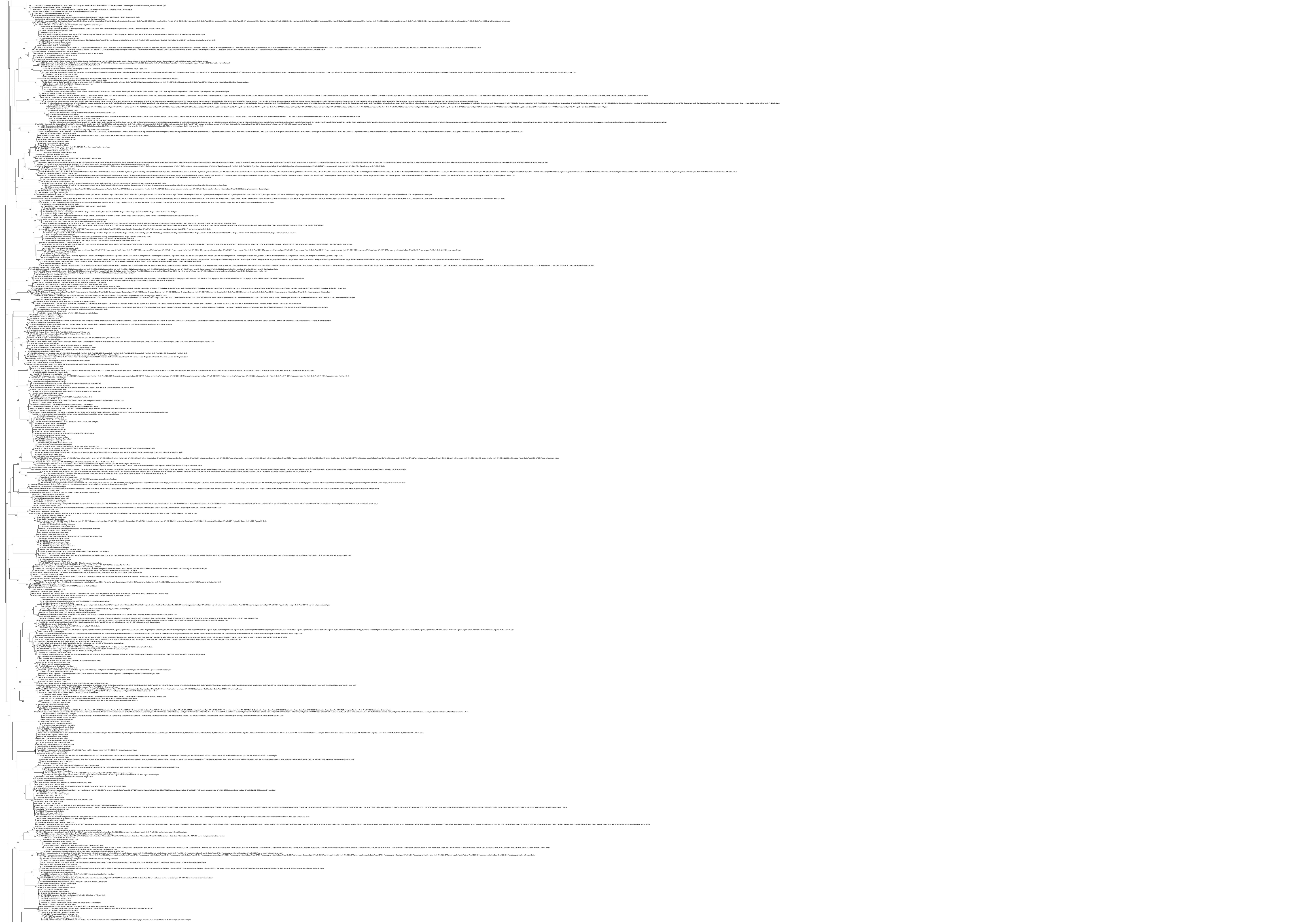

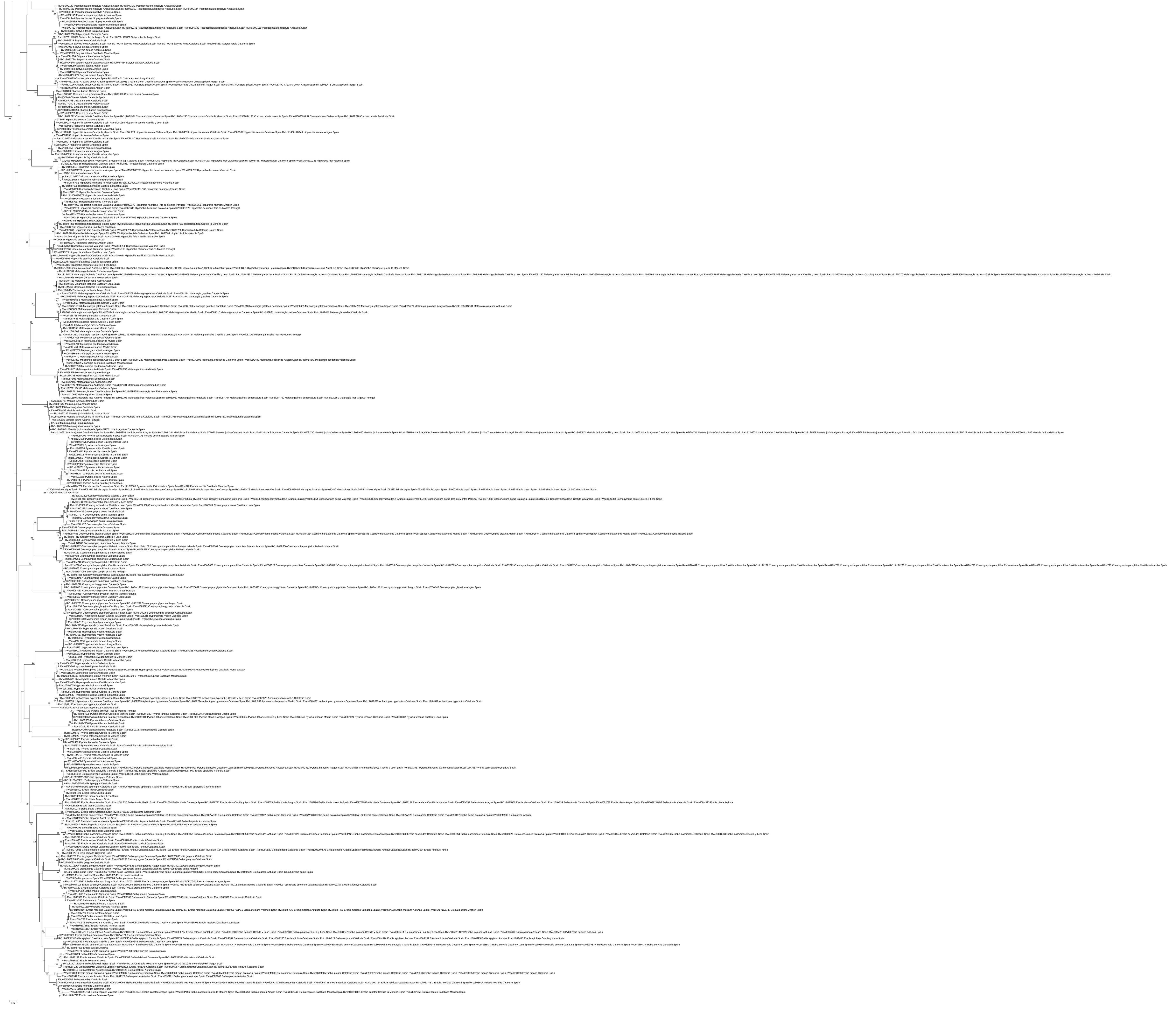

Supplementary Figure 4 | Neighbor-joining tree based on 1971 haplotypes inferred from 5782 barcoded specimens (299 species) of European butterflies. Bootstrap support values (> 50%) based on 100 replicates are indicated.

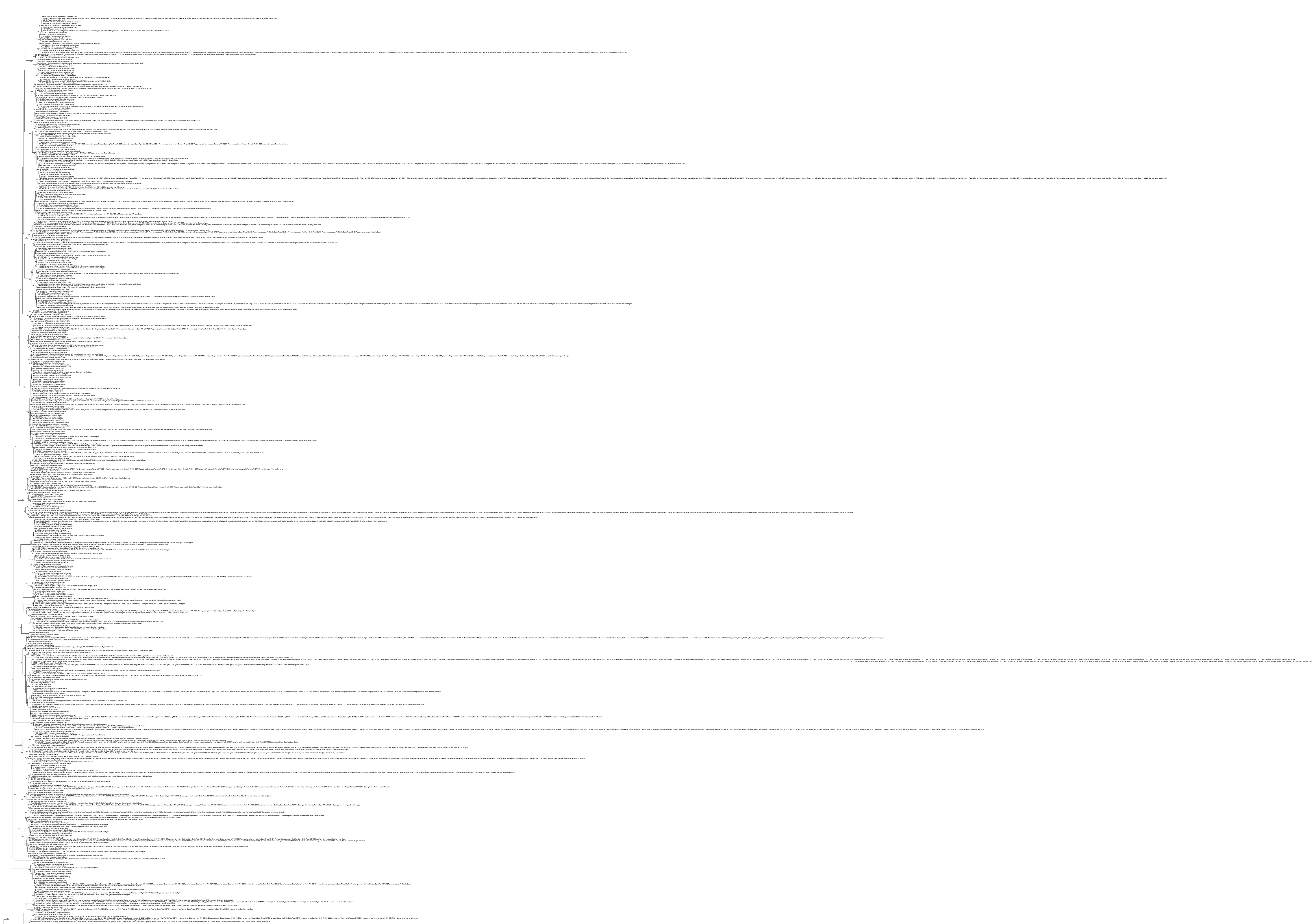

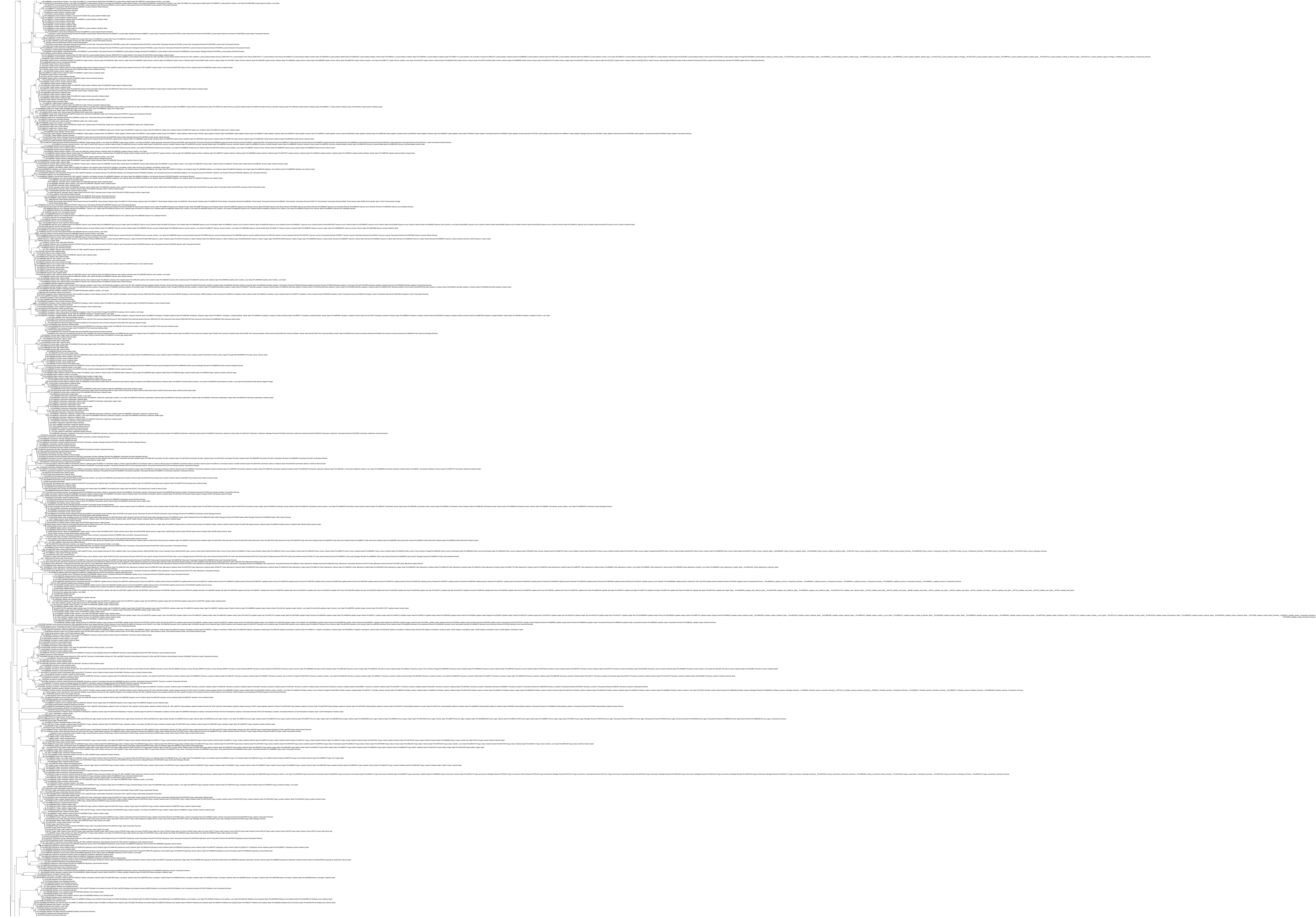

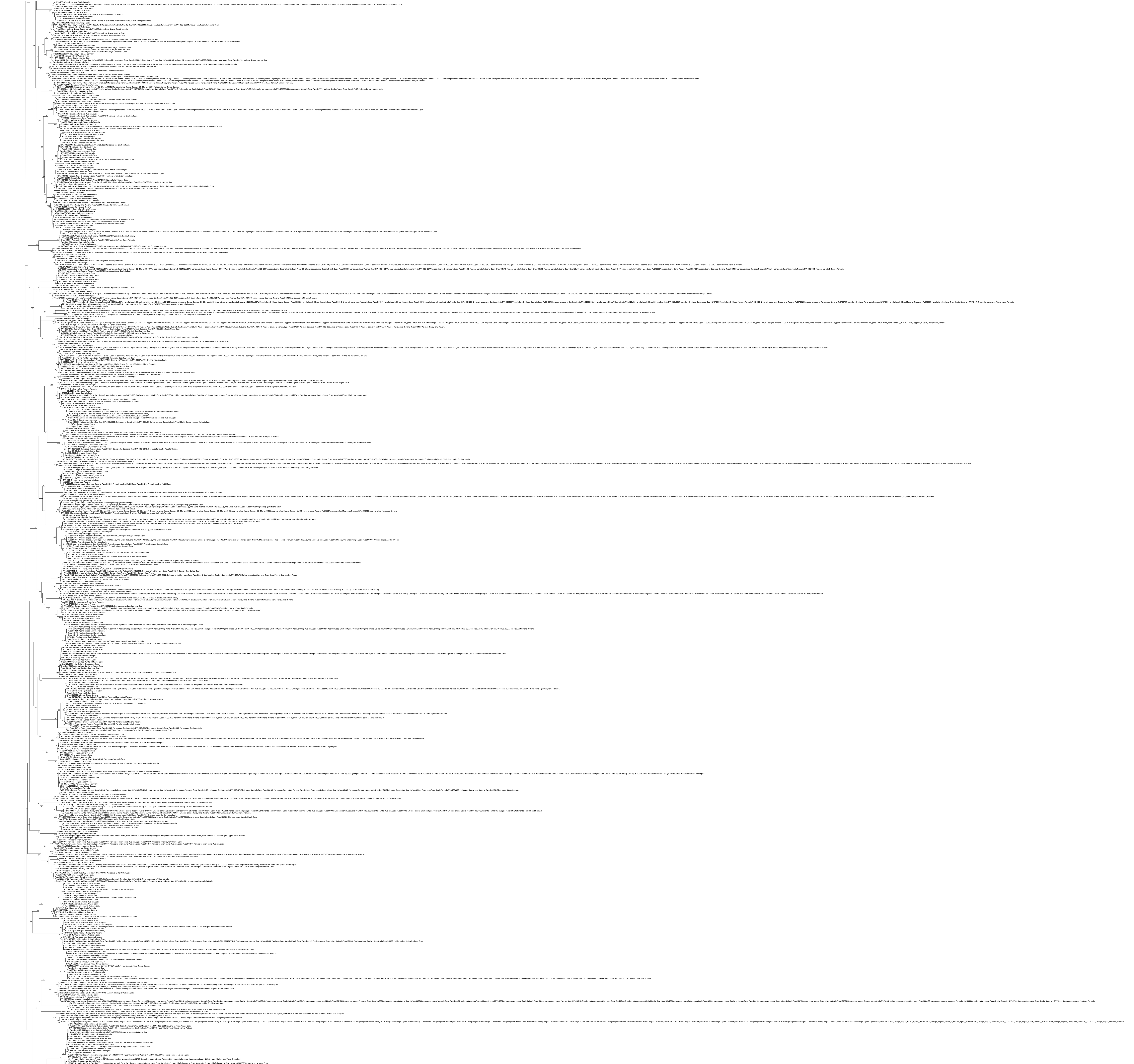

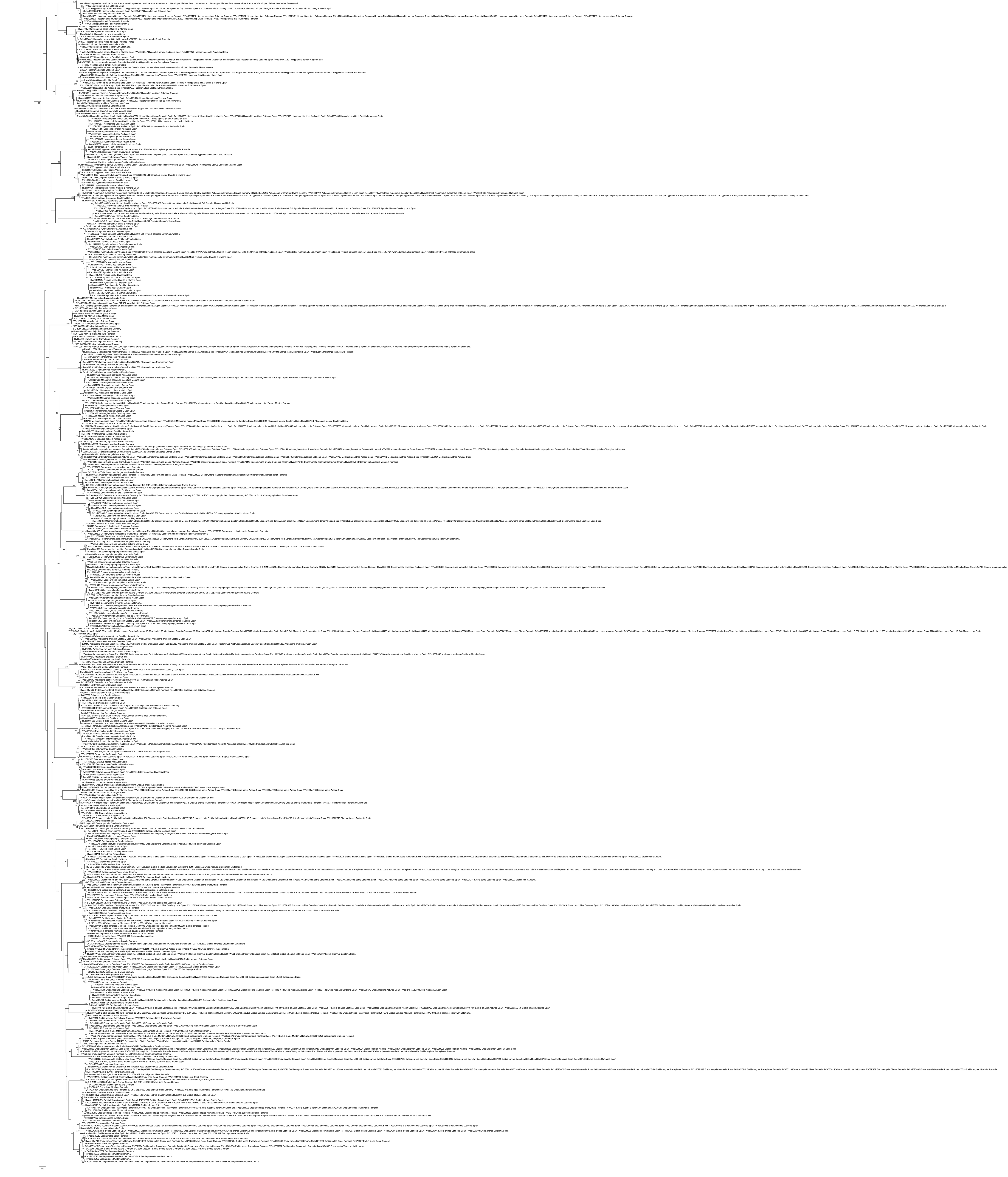

Supplementary Figure 5 | Entities recovered by the single-threshold Generalized Mixed Yule-Coalescent model (ST-GMYC) based on 1971 haplotypes inferred from 5782 European butterfly specimens (299 species). GMYC supports for entity differentiation are indicated. The entity names correspond to those in Supplementary Table 3.

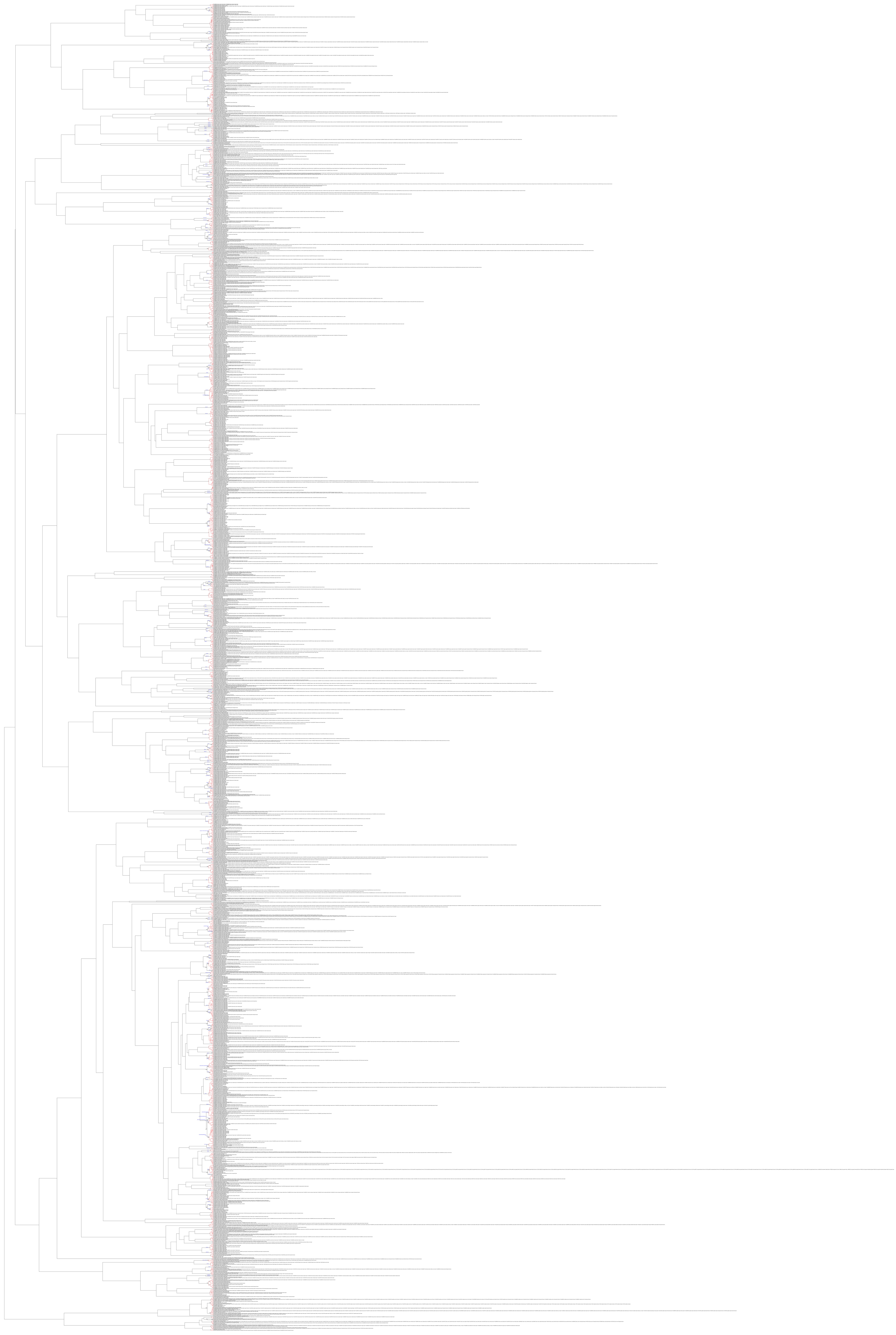

Supplementary Figure 6 | Supports for entity differentiation produced by the Multimodel Generalized Mixed Yule-Coalescent analysis (MM-GMYC) based on 1971 haplotypes inferred from 5782 European butterfly specimens (299 species).

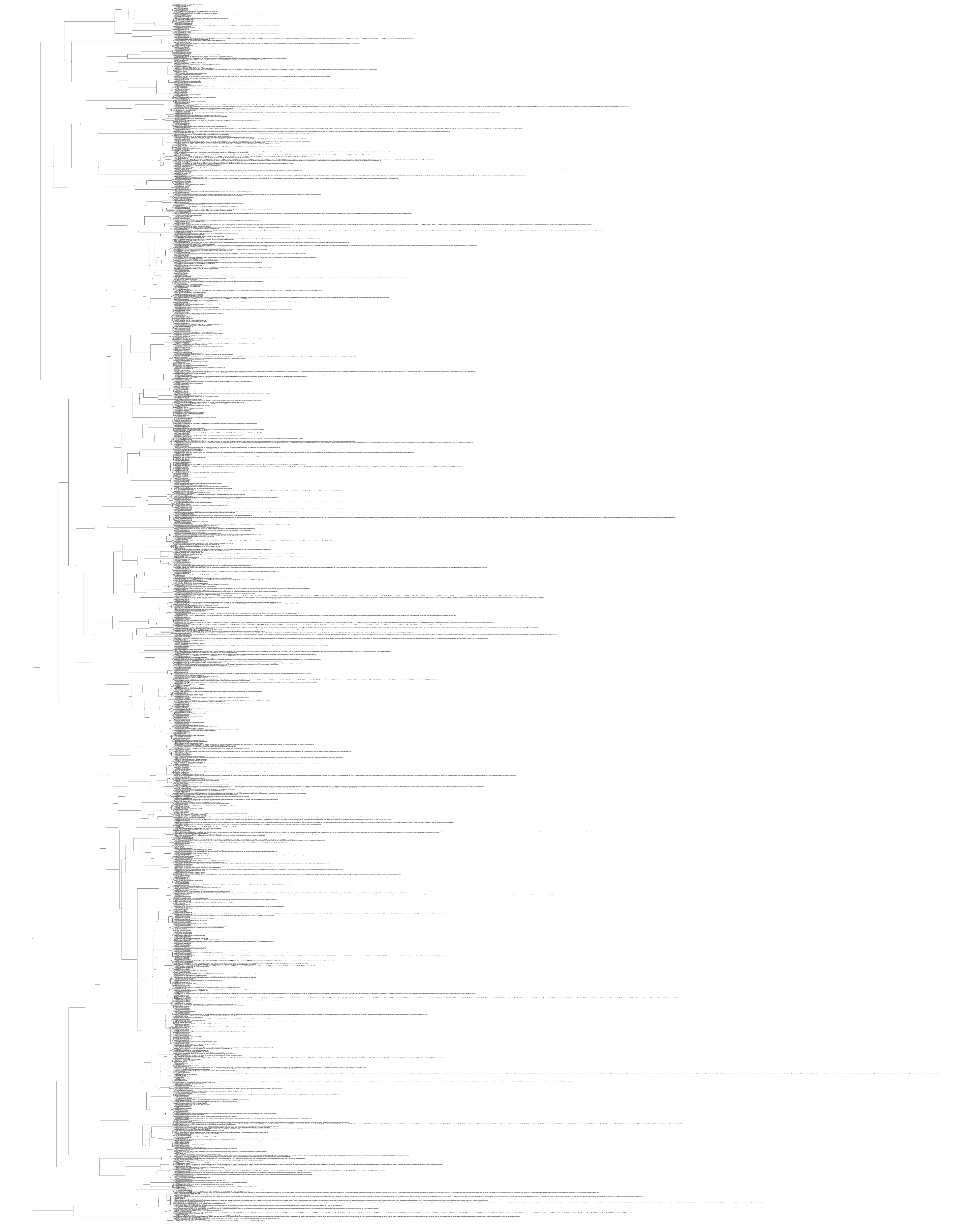

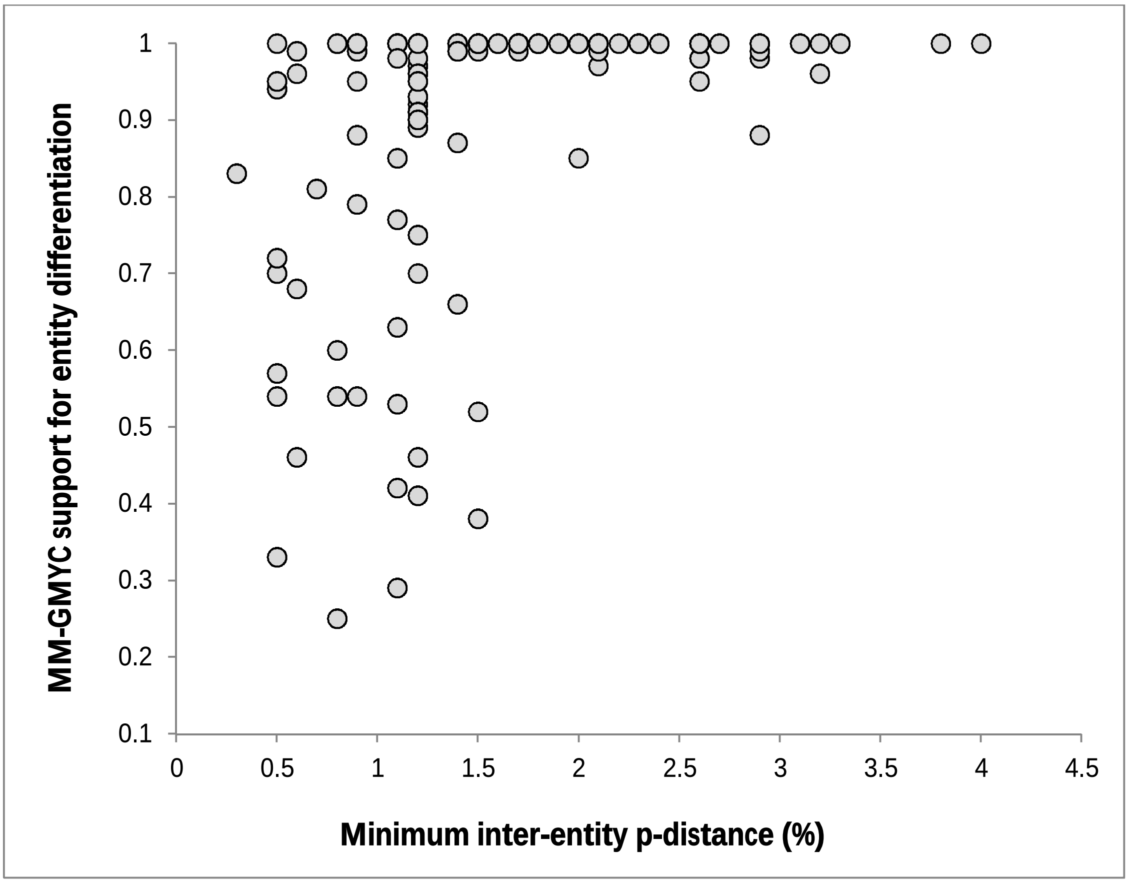

Supplementary Figure 7 | Bivariate scatter plot illustrating the relationship between minimum genetic divergence (%) among conspecific entities and the MM-GMYC supports for entity differentiation.

Supplementary Table 1 | List of 5782 European specimens included in this study. Samples are ordered by genus, species, country and region.

GenBank accession number	BOLD Process ID	Sample ID	Used in the Iberian dataset	Family	Genus	Species	Exact Site	Region	State/Province	Country	Alt. (m)
GU707096	FBLMU443-09	BC ZSM Lep 27093		*Nymphalidae*	*Aglais*	*io*	Schoengeising, Jaegereinfang b. Jexhof	Oberbayern	Bavaria	Germany	570
HQ003941	EZROM217-08	RV-06-K655		*Nymphalidae*	*Aglais*	*io*	Baciu forest (Cluj-Napoca)	Cluj	Transylvania	Romania	530
HQ003942	EZROM218-08	RV-06-K659		*Nymphalidae*	*Aglais*	*io*	Baciu forest (Cluj-Napoca)	Cluj	Transylvania	Romania	530
HQ003943	EZROM330-08	RV-08-A008		*Nymphalidae*	*Aglais*	*io*	Cluj-Napoca, Borhanci	Cluj	Transylvania	Romania	480
HQ003944	EZROM1026-08	RVcoll.08-M530		*Nymphalidae*	*Aglais*	*io*	Tismana monastery	Gorj	Oltenia	Romania	230
HQ003945	EZROM1027-08	RVcoll.08-M640		*Nymphalidae*	*Aglais*	*io*	Scorota (Retezat Mts.)	Hunedoara	Transylvania	Romania	1560
HQ003946	EZROM1025-08	RVcoll.08-M493		*Nymphalidae*	*Aglais*	*io*	Cota 2000 m (Bucegi Mts.), cabana Miorita	Prahova	Muntenia	Romania	2000
FJ663678	LOWA167-06	2005-LOWA-167		*Nymphalidae*	*Aglais*	*io*	Osyno vill.	Sebezh distr	Pskov	Russia	150
FJ663677	LOWA783-06	2005-LOWA-783		*Nymphalidae*	*Aglais*	*io*	Osyno vill.	Sebezh distr.	Pskov	Russia	150
HM901297	EZSPC1096-10	RVcoll.08-L935	x	*Nymphalidae*	*Aglais*	*io*	Puerto de La Morcuera		Comunidad de Madrid	Spain	1766
KP870896	EZSPM574-12	RVcoll.08-L836	x	*Nymphalidae*	*Aglais*	*io*	Oteruelo del Valle, Rascafria		Comunidad de Madrid	Spain	1146
GU676242	EZSPN911-09	RVcoll.08-L848	x	*Nymphalidae*	*Aglais*	*io*	Pinilla del Valle		Comunidad de Madrid	Spain	1100
GU675773	EZSPM241-09	RVcoll.08-R448	x	*Nymphalidae*	*Aglais*	*io*	Padron, Picarana-Rio Tinto	A Coruna	Galicia	Spain	30
KP870771	EZSPC444-09	RVcoll.07-E605	x	*Nymphalidae*	*Aglais*	*io*	Roc Fite, Canoves	Barcelona	Catalonia	Spain	400
GU669794	EZSPC446-09	RVcoll.08-L515	x	*Nymphalidae*	*Aglais*	*io*	El Cafe, pla de la Calma, El Brull, Osona	Barcelona	Catalonia	Spain	1192
GU675680	EZSPC447-09	RVcoll.08-R146	x	*Nymphalidae*	*Aglais*	*io*	Turo de L`Home, Montseny	Barcelona	Catalonia	Spain	1643
GU669795	EZSPC448-09	RVcoll.08-R287	x	*Nymphalidae*	*Aglais*	*io*	Masia del Plans, Vidra, La Garrotxa	Barcelona	Catalonia	Spain	1200
KP870332	EZSPM571-12	RVcoll.08-H222	x	*Nymphalidae*	*Aglais*	*io*	Vallforners, Canoves, Valles Oriental	Barcelona	Catalonia	Spain	600
KP870786	EZSPM572-12	RVcoll.08-H225	x	*Nymphalidae*	*Aglais*	*io*	Vallforners, Canoves, Valles Oriental	Barcelona	Catalonia	Spain	600
GU676170	EZSPN994-09	RVcoll.08-M063	x	*Nymphalidae*	*Aglais*	*io*	Budia, La Alcarria	Guadalajara	Castilla-La Mancha	Spain	1006
GU676309	EZSPN848-09	RVcoll.08-L692	x	*Nymphalidae*	*Aglais*	*io*	Candelario	Salamanca	Castilla y Leon	Spain	1300
GU676241	EZSPN918-09	RVcoll.08-L862	x	*Nymphalidae*	*Aglais*	*io*	Abejar	Soria	Castilla y Leon	Spain	1155
KP870911	EULEP045-14	08-H029		*Nymphalidae*	*Aglais*	*urticae*	Căldarea Lala (M-ții. Rodnei)	Bistrita-Nasaud	Transylvania	Romania	
HQ003948	EZROM597-08	RV-07-D352		*Nymphalidae*	*Aglais*	*urticae*	Racos	Brasov	Transylvania	Romania	460
HQ003947	EZROM283-08	RV-07-E645		*Nymphalidae*	*Aglais*	*urticae*	Dealul Istrita, Breaza	Buzau	Muntenia	Romania	720
HQ003952	EZROM001-08	RV-06-M855		*Nymphalidae*	*Aglais*	*urticae*	Cluj-Napoca dealul Feleacului colonia Borhanci	Cluj	Transylvania	Romania	480
KP870497	EULEP134-14	10-C378		*Nymphalidae*	*Aglais*	*urticae*	Filpea-Gălăuţaș	Harghita	Transylvania	Romania	
HQ003950	EZROM002-08	RV-07-D548		*Nymphalidae*	*Aglais*	*urticae*	Rohia	Maramures	Maramures	Romania	360
HQ003949	EZROM754-08	RVcoll.08-M491		*Nymphalidae*	*Aglais*	*urticae*	Cota 2000 m (Bucegi Mts.), cabana Miorita	Prahova	Muntenia	Romania	2000
HQ003951	EZROM003-08	RV-07-C204		*Nymphalidae*	*Aglais*	*urticae*	Varful Vanturarita Mare (Buila-Vanturarita)	Valcea	Oltenia	Romania	1810
HM901624	EZSPC769-10	RVcoll.08-L703	x	*Nymphalidae*	*Aglais*	*urticae*	Manzanares el Real		Comunidad de Madrid	Spain	908
GU675710	EZSPM139-09	RVcoll.08-P651	x	*Nymphalidae*	*Aglais*	*urticae*	Valle (Valle-Zurea), Lena		Asturias	Spain	662
GU676685	EZSPN360-09	RVcoll.08-H339	x	*Nymphalidae*	*Aglais*	*urticae*	Puerto de Morcuera, Miraflores		Comunidad de Madrid	Spain	
GU676323	EZSPN835-09	RVcoll.08-L657	x	*Nymphalidae*	*Aglais*	*urticae*	San Martin del Pimpollar, Piscifactoria Rasquilla	Avila	Castilla y Leon	Spain	1270
KP871021	EZSPC076-09	RVcoll.08-P217	x	*Nymphalidae*	*Aglais*	*urticae*	La Molina	Barcelona	Catalonia	Spain	1622
KP870295	EZSPC092-09	RVcoll.07-E603.1	x	*Nymphalidae*	*Aglais*	*urticae*	Castellar de n`Hug	Barcelona	Catalonia	Spain	1300
HM901799	EZSPC964-10	RVcoll.190608FT45	x	*Nymphalidae*	*Aglais*	*urticae*	La Vega, Vilafranca	Castellon	Comunidad Valenciana	Spain	1124
GU675664	EZSPC077-09	RVcoll.07-C631	x	*Nymphalidae*	*Aglais*	*urticae*	Meranges	Girona	Catalonia	Spain	1555
KP870424	EZSPC078-09	RVcoll.08-R180	x	*Nymphalidae*	*Aglais*	*urticae*	Vallter 2000, Setcases, Ripolles	Girona	Catalonia	Spain	2537
JN274547	EZSPC1309-10	RVcoll.130209KL69	x	*Nymphalidae*	*Aglais*	*urticae*	Ruta del Veleta,	Granada	Andalusia	Spain	
HM901605	EZSPC750-10	RVcoll.08-L143	x	*Nymphalidae*	*Aglais*	*urticae*	Laguna Seca, Hueneja	Granada	Andalusia	Spain	2276
HM901793	EZSPC958-10	RVcoll.100608DS47	x	*Nymphalidae*	*Aglais*	*urticae*	Acceso Veleta, Monachil	Granada	Andalusia	Spain	2737
KP870419	EZSPM615-12	RVcoll.11-D824	x	*Nymphalidae*	*Aglais*	*urticae*	Canada Real de la Zubia, Monachil	Granada	Andalusia	Spain	1350
KP870417	EZSPM651-12	RVcoll.11-I470	x	*Nymphalidae*	*Aglais*	*urticae*	Road to Veleta (Sierra Nevada)	Granada	Andalusia	Spain	2725
KP870996	EZSPM652-12	RVcoll.11-I472	x	*Nymphalidae*	*Aglais*	*urticae*	Road to Veleta (Sierra Nevada)	Granada	Andalusia	Spain	2725

GenBank accession number	BOLD Process ID	Sample ID	Used in the Iberian dataset	Family	Genus	Species	Exact Site	Region	State/Province	Country	Alt. (m)
KP870560	EZSPM653-12	RVcoll.11-I473	x	*Nymphalidae*	*Aglais*	*urticae*	Road to Veleta (Sierra Nevada)	Granada	Andalusia	Spain	2725
KP870910	EZSPM654-12	RVcoll.11-I474	x	*Nymphalidae*	*Aglais*	*urticae*	Road to Veleta (Sierra Nevada)	Granada	Andalusia	Spain	2725
GU676840	EZSPN091-09	RVcoll.06-A020	x	*Nymphalidae*	*Aglais*	*urticae*	Mecina Bombaron, Alpujarras	Granada	Andalusia	Spain	1150
GU676834	EZSPN098-09	RVcoll.06-A037	x	*Nymphalidae*	*Aglais*	*urticae*	Barranco de los Lagartos, Cadiar	Granada	Andalusia	Spain	860
GU676423	EZSPN727-09	RVcoll.08-L134	x	*Nymphalidae*	*Aglais*	*urticae*	Laguna Seca, Hueneja	Granada	Andalusia	Spain	2276
GU677030	EZSPN184-09	RVcoll.08-J882	x	*Nymphalidae*	*Aglais*	*urticae*	Villaobispo de las Regueras	Leon	Castilla y Leon	Spain	
KP870657	EZSPC079-09	RVcoll.08-L484	x	*Nymphalidae*	*Aglais*	*urticae*	Bassa d`Arres, Vall d`Aran	Lleida	Catalonia	Spain	1501
GU675776	EZSPM260-09	RVcoll.08-R474	x	*Nymphalidae*	*Aglais*	*urticae*	Folgoso do Caurel, Estr. Ferramulin-Visuna Km. 18-20	Lugo	Galicia	Spain	850
HM901637	EZSPC784-10	RVcoll.08-L982	x	*Nymphalidae*	*Aglais*	*urticae*	Navafria, Los Reajos	Segovia	Castilla y Leon	Spain	2051
GU676191	EZSPN967-09	RVcoll.08-L981	x	*Nymphalidae*	*Aglais*	*urticae*	Navafria, El Nevero	Segovia	Castilla y Leon	Spain	2130
KP870899	EZSPM761-12	RVcoll.030111FB20	x	*Nymphalidae*	*Aglais*	*urticae*	Fuente del Pinarico, Valdelinares	Teruel	Aragon	Spain	1712
KP870637	EZSPM783-12	RVcoll.070211PL18	x	*Nymphalidae*	*Aglais*	*urticae*	Acceso Javalambre, Puebla de Valverde	Teruel	Aragon	Spain	1817
KP870928	EZSPM784-12	RVcoll.070211PL40	x	*Nymphalidae*	*Aglais*	*urticae*	Acceso Javalambre, Puebla de Valverde	Teruel	Aragon	Spain	1817
KP870696	EZSPM806-12	RVcoll.090611SZ73	x	*Nymphalidae*	*Aglais*	*urticae*	Fuente del Pinarico, Valdelinares	Teruel	Aragon	Spain	1712
KP870662	EZSPM820-12	RVcoll.140410KV21	x	*Nymphalidae*	*Aglais*	*urticae*	Ascenso a Penarroya, Alcala de la Selva	Teruel	Aragon	Spain	2016
KP871096	EZSPM822-12	RVcoll.140410KV47	x	*Nymphalidae*	*Aglais*	*urticae*	Ascenso a Penarroya, Alcala de la Selva	Teruel	Aragon	Spain	2016
HM401002	EZSPM550-10	RVcoll.08-P083.1	x	*Lycaenidae*	*Agriades*	*glandon*	Estany Rodo, Pessons			Andorra	2380
HM901686	EZSPC845-10	RVcoll.08-R217	x	*Lycaenidae*	*Agriades*	*glandon*	Port de la Bonaigua, Pallars Sobira	Lleida	Catalonia	Spain	2338
HM400995	EZSPM537-10	RVcoll.08-R214.1	x	*Lycaenidae*	*Agriades*	*glandon*	Port de la Bonaigua, Pallars Sobira	Lleida	Catalonia	Spain	2338
HM400996	EZSPM538-10	RVcoll.08-R215.1	x	*Lycaenidae*	*Agriades*	*glandon*	Port de la Bonaigua, Pallars Sobira	Lleida	Catalonia	Spain	2338
HM400997	EZSPM539-10	RVcoll.08-R216.1	x	*Lycaenidae*	*Agriades*	*glandon*	Port de la Bonaigua, Pallars Sobira	Lleida	Catalonia	Spain	2338
HM401000	EZSPM548-10	RVcoll.07-W204	x	*Lycaenidae*	*Agriades*	*glandon*	Col de Vilac, Vall d`Aran	Lleida	Catalonia	Spain	2334
HM401001	EZSPM549-10	RVcoll.07-W209	x	*Lycaenidae*	*Agriades*	*glandon*	Col de Vilac, Vall d`Aran	Lleida	Catalonia	Spain	2334
KP871072	EZSPM634-12	RVcoll.11-H025	x	*Lycaenidae*	*Agriades*	*glandon*	Port de la Bonaigua, Tuc de la Cigalera	Lleida	Catalonia	Spain	2200
KP871020	EZSPM636-12	RVcoll.11-H027	x	*Lycaenidae*	*Agriades*	*glandon*	Port de la Bonaigua, Tuc de la Cigalera	Lleida	Catalonia	Spain	2200
GU676977	EZSPN247-09	RVcoll.07-W123	x	*Lycaenidae*	*Agriades*	*glandon*	Tuc de la Cigalera, Vall d`Aran	Lleida	Catalonia	Spain	2330
HM871929	LEFIC060-10	MM03344		*Lycaenidae*	*Agriades*	*optilete*	Kiiminki	Ostrobottnia borealis pars australis	Northern Ostrobothnia	Finland	
HM871930	LEFIC061-10	MM03345		*Lycaenidae*	*Agriades*	*optilete*	Kiiminki	Ostrobottnia borealis pars australis	Northern Ostrobothnia	Finland	
GU688455	GWORO798-09	BC ZSM Lep 30490		*Lycaenidae*	*Agriades*	*optilete*	Zellwies Koenigsdorf / TOEL		Bavaria	Germany	
FJ664077	LOWA296-06	2005-LOWA-296		*Lycaenidae*	*Agriades*	*optilete*	Tamengont		St. Petersburg	Russia	
FJ664076	LOWA297-06	2005-LOWA-297		*Lycaenidae*	*Agriades*	*optilete*	Tamengont		St. Petersburg	Russia	
FJ664075	LOWA757-06	2005-LOWA-757		*Lycaenidae*	*Agriades*	*optilete*	Tamengont	Oranienbaum distr.	St. Petersburg	Russia	
GQ129011		VL-01-B424		*Lycaenidae*	*Agriades*	*optilete*	Tamengont		St. Petersburg	Russia	
HQ968289	PHLAB1026-10	TLMF Lep 01826		*Lycaenidae*	*Agriades*	*optilete*	Val Bever, Alp Suvretta		Graubunden	Switzerland	2100
GU676984	EZSPN237-09	RVcoll.08-J811	x	*Lycaenidae*	*Agriades*	*pyrenaicus*	Lagos de Covadonga		Asturias	Spain	
HM901722	EZSPC882-10	RVcoll.09-T143	x	*Lycaenidae*	*Agriades*	*pyrenaicus*	Subida a la Laguna de las Verdes, Cabrillanes	Leon	Castilla y Leon	Spain	1520
JN276899	EZSPN1083-11	Rvcoll.09-X297	x	*Lycaenidae*	*Agriades*	*pyrenaicus*	Puerto de San Isidro	Leon	Castilla y Leon	Spain	1700
JN276900	EZSPN1084-11	Rvcoll.09-X298	x	*Lycaenidae*	*Agriades*	*pyrenaicus*	Puerto de San Isidro	Leon	Castilla y Leon	Spain	1700
GU676957	EZSPN268-09	RVcoll.07-W185	x	*Lycaenidae*	*Agriades*	*pyrenaicus*	Camino de Wamba, Puerto de San Isidro	Leon	Castilla y Leon	Spain	1700
GU676769	EZSPN269-09	RVcoll.07-W186	x	*Lycaenidae*	*Agriades*	*pyrenaicus*	Camino de Wamba, Puerto de San Isidro	Leon	Castilla y Leon	Spain	1700
HM901731	EZSPC892-10	RVcoll.09-V415	x	*Lycaenidae*	*Agriades*	*zullichi*	San Juan (Sierra Nevada)	Granada	Andalusia	Spain	2760
HM901732	EZSPC893-10	RVcoll.09-V417	x	*Lycaenidae*	*Agriades*	*zullichi*	San Juan (Sierra Nevada)	Granada	Andalusia	Spain	2760
GU676084	EZSPM471-09	Rvcoll.09-V416	x	*Lycaenidae*	*Agriades*	*zullichi*	San Juan (Sierra Nevada)	Granada	Andalusia	Spain	2760
GU676085	EZSPM472-09	Rvcoll.09-V418	x	*Lycaenidae*	*Agriades*	*zullichi*	San Juan (Sierra Nevada)	Granada	Andalusia	Spain	2760
GU676082	EZSPM473-09	Rvcoll.09-V414	x	*Lycaenidae*	*Agriades*	*zullichi*	San Juan (Sierra Nevada)	Granada	Andalusia	Spain	2760
GU676486	EZSPN659-09	RVcoll.08-J957	x	*Lycaenidae*	*Agriades*	*zullichi*	Acceso Veleta, Monachil	Granada	Andalusia	Spain	2737
GU676468	EZSPN677-09	RVcoll.08-J989	x	*Lycaenidae*	*Agriades*	*zullichi*	Acceso Veleta, Monachil	Granada	Andalusia	Spain	2737
GU676469	EZSPN678-09	RVcoll.08-J990	x	*Lycaenidae*	*Agriades*	*zullichi*	Observatorio Veleta, Dilar	Granada	Andalusia	Spain	2768
HQ003953	EZRMN410-09	RVcoll.07-D031		*Papilionidae*	*Allancastria*	*cerisy*	Canaraua Fetei (Baneasa)	Constanta	Dobrogea	Romania	20
GU688476	GWORO780-09	BC ZSM Lep 30472		*Pieridae*	*Anthocharis*	*cardamines*	Fischerhaeuser Isarauen / MUC		Bavaria	Germany	482

GenBank accession number	BOLD Process ID	Sample ID	Used in the Iberian dataset	Family	Genus	Species	Exact Site	Region	State/Province	Country	Alt. (m)
HQ957207	GWORA2444-09	BC ZSM Lep 30656		*Pieridae*	*Anthocharis*	*cardamines*	Auenzell near Saulburg	Niederbayern	Bavaria	Germany	440
HQ957206	GWORA2443-09	BC ZSM Lep 30655		*Pieridae*	*Anthocharis*	*cardamines*	Sudelfeld, Arzmoos	Oberbayern	Bavaria	Germany	1200
HM391816	FBLMU458-09	BC ZSM Lep 27108		*Pieridae*	*Anthocharis*	*cardamines*	Duggendorf, Girnitztal	Oberpfalz	Bavaria	Germany	375
HQ003958	EZROM757-08	RVcoll.08-M213		*Pieridae*	*Anthocharis*	*cardamines*	3 Km SW of Lunca Corbului	Arges	Muntenia	Romania	235
HQ003960	EZROM756-08	RVcoll.07-D315		*Pieridae*	*Anthocharis*	*cardamines*	Racos	Brasov	Transylvania	Romania	460
HQ003955	EZROM006-08	RV-07-D096		*Pieridae*	*Anthocharis*	*cardamines*	Dealul Istrita, Breaza	Buzau	Muntenia	Romania	540
HQ003961	EZROM007-08	RV-07-D203		*Pieridae*	*Anthocharis*	*cardamines*	Dealul Istrita, Breaza	Buzau	Muntenia	Romania	540
HQ003954	EZROM004-08	RV-06-K525		*Pieridae*	*Anthocharis*	*cardamines*	Badeni	Cluj	Transylvania	Romania	440
HQ003956	EZROM005-08	RV-06-K531		*Pieridae*	*Anthocharis*	*cardamines*	Badeni	Cluj	Transylvania	Romania	440
HQ003959	EZROM755-08	RVcoll.07-D157		*Pieridae*	*Anthocharis*	*cardamines*	Dumbraveni forest	Constanta	Dobrogea	Romania	80
HQ003957	EZROM758-08	RVcoll.08-M355		*Pieridae*	*Anthocharis*	*cardamines*	Valea Belchia (Gheorgheni)	Harghita	Transylvania	Romania	1010
HQ003962	EZROM008-08	RV-07-D924		*Pieridae*	*Anthocharis*	*cardamines*	valea Otasaului, Barzesti, Buila-Vanturarita	Valcea	Oltenia	Romania	515
GU676663	EZSPN381-09	RVcoll.08-H428	x	*Pieridae*	*Anthocharis*	*cardamines*	Campo Real		Comunidad de Madrid	Spain	731
GU675838	EZSPM230-09	RVcoll.08-R425	x	*Pieridae*	*Anthocharis*	*cardamines*	Santiso, Embalse Portodemouros	A Coruna	Galicia	Spain	265
GU675835	EZSPM231-09	RVcoll.08-R426	x	*Pieridae*	*Anthocharis*	*cardamines*	Touro, Lana	A Coruna	Galicia	Spain	280
GU676693	EZSPN346-09	RVcoll.08-H311	x	*Pieridae*	*Anthocharis*	*cardamines*	Casillas, Casillas	Avila	Castilla y Leon	Spain	
KP870830	EZSPC081-09	RVcoll.06-H861	x	*Pieridae*	*Anthocharis*	*cardamines*	Vallforners, Canoves, Valles Oriental	Barcelona	Catalonia	Spain	650
KP870255	EZSPC082-09	RVcoll.08-H229	x	*Pieridae*	*Anthocharis*	*cardamines*	Vallforners, Canoves	Barcelona	Catalonia	Spain	610
GU676897	EZSPN492-09	RVcoll.08-H929	x	*Pieridae*	*Anthocharis*	*cardamines*	Hervas, Carretera Hervas-La Garganta	Caceres	Extremadura	Spain	1007
KP871150	EZSPC083-09	RVcoll.08-J351	x	*Pieridae*	*Anthocharis*	*cardamines*	Cantallops, Alt Emporda	Girona	Catalonia	Spain	225
GU676453	EZSPN694-09	RVcoll.08-L026	x	*Pieridae*	*Anthocharis*	*cardamines*	Casillas de Rojas, Gueejar Sierra	Granada	Andalusia	Spain	1733
JN276237	EZSPN1040-11	Rvcoll.08-H549	x	*Pieridae*	*Anthocharis*	*cardamines*	Chillaron del Rey, La Alcarria	Guadalajara	Castilla-La Mancha	Spain	710
GU676675	EZSPN369-09	RVcoll.08-J895	x	*Pieridae*	*Anthocharis*	*cardamines*	Vega de Espinareda	Leon	Castilla y Leon	Spain	
KP871089	EZSPC084-09	RVcoll.08-L420	x	*Pieridae*	*Anthocharis*	*cardamines*	Rius de Cerdanya	Lleida	Catalonia	Spain	1273
KP871157	EZSPC085-09	RVcoll.08-M900	x	*Pieridae*	*Anthocharis*	*cardamines*	Bassa d`Arres, Vall d`Aran	Lleida	Catalonia	Spain	1500
GU676512	EZSPN630-09	RVcoll.08-J728	x	*Pieridae*	*Anthocharis*	*cardamines*	Rincon del Sardo, Castielfabib	Valencia	Comunidad Valenciana	Spain	1381
GU675958	EZSPM026-09	RVcoll.08-P202	x	*Pieridae*	*Anthocharis*	*euphenoides*	Courbassil	Languedoc-Roussillon		France	1390
GU676689	EZSPN350-09	RVcoll.08-H321	x	*Pieridae*	*Anthocharis*	*euphenoides*	Cebreros, Cebreros	Avila	Castilla y Leon	Spain	
JN276236	EZSPN1034-11	Rvcoll.06-G464	x	*Pieridae*	*Anthocharis*	*euphenoides*	Sallent, Bages	Barcelona	Catalonia	Spain	320
JN276244	EZSPN1075-11	Rvcoll.09-V695	x	*Pieridae*	*Anthocharis*	*euphenoides*	Meranges, La Cerdanya	Girona	Catalonia	Spain	1744
GU676843	EZSPN090-09	RVcoll.06-A019	x	*Pieridae*	*Anthocharis*	*euphenoides*	Barranco de los Lagartos, Cadiar	Granada	Andalusia	Spain	860
GU676448	EZSPN696-09	RVcoll.08-L029	x	*Pieridae*	*Anthocharis*	*euphenoides*	Casillas de Rojas, Gueejar Sierra	Granada	Andalusia	Spain	1733
GU676624	EZSPN415-09	RVcoll.08-H533	x	*Pieridae*	*Anthocharis*	*euphenoides*	Gualda, La Alcarria	Guadalajara	Castilla-La Mancha	Spain	730
GU676536	EZSPN605-09	RVcoll.08-J312	x	*Pieridae*	*Anthocharis*	*euphenoides*	Barranco de Valcuerna, Candasnos	Huesca	Aragon	Spain	210
GU676590	EZSPN455-09	RVcoll.08-J880	x	*Pieridae*	*Anthocharis*	*euphenoides*	San Esteban de Valdueza	Leon	Castilla y Leon	Spain	
GU676791	EZSPN144-09	RVcoll.06-G514	x	*Pieridae*	*Anthocharis*	*euphenoides*	Coll de Comiols, Pallars Jussa	Lleida	Catalonia	Spain	
GU675837	EZSPM262-09	RVcoll.08-R477	x	*Pieridae*	*Anthocharis*	*euphenoides*	Folgoso do Caurel, Alto de Visuna	Lugo	Galicia	Spain	1330
GU676600	EZSPN441-09	RVcoll.08-H625	x	*Pieridae*	*Anthocharis*	*euphenoides*	Benadalid	Malaga	Andalusia	Spain	790
GU676487	EZSPN643-09	RVcoll.08-J779	x	*Pieridae*	*Anthocharis*	*euphenoides*	El Masegar, Moscardon	Teruel	Aragon	Spain	1531
GU676519	EZSPN625-09	RVcoll.08-J715	x	*Pieridae*	*Anthocharis*	*euphenoides*	Rincon del Sardo, Castielfabib	Valencia	Comunidad Valenciana	Spain	1381
GU707196	FBLMU109-09	BC ZSM Lep 25619		*Nymphalidae*	*Apatura*	*ilia*	Eching, Obere Isarau	Niederbayern	Bavaria	Germany	400
HM393229	GWORA2515-09	BC ZSM Lep 30727		*Nymphalidae*	*Apatura*	*ilia*	Fuerstenzell, Altenmarkt	Niederbayern	Bavaria	Germany	460
HQ565466	GWOSA731-10	BC ZSM Lep 35742		*Nymphalidae*	*Apatura*	*ilia*	Ruselgebiet, Vord. Bay. Wald, Dattinger Berg N-Seite	Niederbayern	Bavaria	Germany	800
HM391818	FBLMU463-09	BC ZSM Lep 27113		*Nymphalidae*	*Apatura*	*ilia*	Grassau, Kendlmuehlfilzn	Oberbayern	Bavaria	Germany	535
HM391819	FBLMU464-09	BC ZSM Lep 27114		*Nymphalidae*	*Apatura*	*ilia*	Schweinfurt	Unterfranken	Bavaria	Germany	285
HQ003963	EZRMN371-08	RVcoll.08-M101		*Nymphalidae*	*Apatura*	*ilia*	Oraseni Vale (forest)	Botosani	Moldavia	Romania	165
KP870745	EULEP186-14	11-J883		*Nymphalidae*	*Apatura*	*ilia*	Pădurea Hoia	Cluj	Transylvania	Romania	
HQ003966	EZROM009-08	RV-06-M872		*Nymphalidae*	*Apatura*	*ilia*	Cluj-Napoca, dealul Feleacului, Gheorgheni forest	Cluj	Transylvania	Romania	600
HQ003967	EZROM010-08	RV-06-M898		*Nymphalidae*	*Apatura*	*ilia*	Cluj-Napoca, dealul Feleacului, Gheorgheni forest	Cluj	Transylvania	Romania	600

GenBank accession number	BOLD Process ID	Sample ID	Used in the Iberian dataset	Family	Genus	Species	Exact Site	Region	State/Province	Country	Alt. (m)
HQ003965	EZROM759-08	RVcoll.06-M893		*Nymphalidae*	*Apatura*	*ilia*	Cluj-Napoca, dealul Feleacului, Gheorgheni forest	Cluj	Transylvania	Romania	600
HQ003964	EZROM760-08	RVcoll.08-M661		*Nymphalidae*	*Apatura*	*ilia*	Gheorgheni forest, Cluj-Napoca	Cluj	Transylvania	Romania	600
KP871074	EULEP142-14	10-C420		*Nymphalidae*	*Apatura*	*ilia*	Lăpuș-Prislop	Maramureș	Maramureş	Romania	
FJ663265	LOWA861-06	2005-LOWA-861		*Nymphalidae*	*Apatura*	*ilia*			Belgorod	Russia	150
FJ663264	LOWA862-06	2005-LOWA-862		*Nymphalidae*	*Apatura*	*ilia*			Belgorod	Russia	150
HM901712	EZSPC872-10	RVcoll.09-T123	x	*Nymphalidae*	*Apatura*	*ilia*	Torayo, Tineo		Asturias	Spain	320
HM901714	EZSPC874-10	RVcoll.09-T125	x	*Nymphalidae*	*Apatura*	*ilia*	Onon, Cangas del Narcea		Asturias	Spain	375
KP870641	EZSPC086-09	RVcoll.08-L449	x	*Nymphalidae*	*Apatura*	*ilia*	La Llavina, Montseny, Valles Oriental	Barcelona	Catalonia	Spain	500
GU669637	EZSPC640-09	RVcoll.08-L381	x	*Nymphalidae*	*Apatura*	*ilia*	Vallforners, Canoves, Valles Oriental	Barcelona	Catalonia	Spain	650
KP870478	EZSPM587-12	RVcoll.08-P960	x	*Nymphalidae*	*Apatura*	*ilia*	Vallforners, Canoves, Valles Oriental	Barcelona	Catalonia	Spain	430
KP871015	EZSPC087-09	RVcoll.08-R194	x	*Nymphalidae*	*Apatura*	*ilia*	Setcases	Girona	Catalonia	Spain	1350
GU676755	EZSPN279-09	RVcoll.07-W211	x	*Nymphalidae*	*Apatura*	*ilia*	Bisaurri, Bisaurri	Huesca	Aragon	Spain	1350
JF847976	EZSPN027-09	RVcoll.09-T050	x	*Nymphalidae*	*Apatura*	*ilia*	Garos, Val d`Aran	Lleida	Catalonia	Spain	1100
HM393227	GWORA2513-09	BC ZSM Lep 30725		*Nymphalidae*	*Apatura*	*iris*	Fuerstenzell, Altenmarkt	Niederbayern	Bavaria	Germany	460
HM393228	GWORA2514-09	BC ZSM Lep 30726		*Nymphalidae*	*Apatura*	*iris*	Kleinwissing	Niederbayern	Bavaria	Germany	355
HQ565465	GWOSA729-10	BC ZSM Lep 35740		*Nymphalidae*	*Apatura*	*iris*	Ruselgebiet, Leopoldshochwald	Niederbayern	Bavaria	Germany	700
KP870877	FBLMX206-11	BC ZSM Lep 50417		*Nymphalidae*	*Apatura*	*iris*	Teisendorf	Oberbayern	Bavaria	Germany	520
HM393226	GWORA2512-09	BC ZSM Lep 30724		*Nymphalidae*	*Apatura*	*iris*	Leak near Regensburg, Thiergarten	Oberpfalz	Bavaria	Germany	330
HQ003969	EZROM765-08	RVcoll.08-M606		*Nymphalidae*	*Apatura*	*iris*	Vidraru Lake, pensiunea Vidraru	Arges	Muntenia	Romania	900
HQ003974	EZROM011-08	RV-06-M869		*Nymphalidae*	*Apatura*	*iris*	Cluj-Napoca, dealul Feleacului, Gheorgheni forest	Cluj	Transylvania	Romania	600
HQ003973	EZROM012-08	RV-06-M870		*Nymphalidae*	*Apatura*	*iris*	Cluj-Napoca, dealul Feleacului, Gheorgheni forest	Cluj	Transylvania	Romania	600
HQ003972	EZROM761-08	RVcoll.06-M871		*Nymphalidae*	*Apatura*	*iris*	Cluj-Napoca, dealul Feleacului, Gheorgheni forest	Cluj	Transylvania	Romania	600
HQ003971	EZROM763-08	RVcoll.08-M591		*Nymphalidae*	*Apatura*	*iris*	Gheorgheni, Valea Belchia	Harghita	Transylvania	Romania	1000
HQ003968	EZROM764-08	RVcoll.08-M595		*Nymphalidae*	*Apatura*	*iris*	Gheorgheni, Valea Belchia	Harghita	Transylvania	Romania	1000
HQ003970	EZROM762-08	RVcoll.08-M553		*Nymphalidae*	*Apatura*	*iris*	4 Km NW of Podeni	Mehedinti	Oltenia	Romania	730
HM901713	EZSPC873-10	RVcoll.09-T124	x	*Nymphalidae*	*Apatura*	*iris*	Viboli, Ponga		Asturias	Spain	750
KP870551	EZSPM844-12	RVcoll.150111HJ81	x	*Nymphalidae*	*Apatura*	*iris*	Rascafria		Comunidad de Madrid	Spain	1150
KP870715	EZSPM854-12	RVcoll.300511SB98	x	*Nymphalidae*	*Apatura*	*iris*	Rascafria		Comunidad de Madrid	Spain	1150
KP870989	EZSPM855-12	RVcoll.300511SB99	x	*Nymphalidae*	*Apatura*	*iris*	Rascafria		Comunidad de Madrid	Spain	1150
JF848006	EZSPN063-09	RVcoll.09-V716	x	*Nymphalidae*	*Apatura*	*iris*	Yesero	Huesca	Aragon	Spain	1160
KP870700	EULEP079-14	08-P964	x	*Nymphalidae*	*Apatura*	*iris*	Camí Reiau, Artíes, Vall d'Aran	Lleida	Catalonia	Spain	1120
KP870711	EULEP214-14	12-L037	x	*Nymphalidae*	*Apatura*	*iris*	Salt deth Pitx, Vall d´Arán	Lleida	Catalonia	Spain	1570
KP871061	EZSPC088-09	RVcoll.08-L533	x	*Nymphalidae*	*Apatura*	*iris*	Barranc de Riu Major, Gerri de la Sal, Pallars Sobira	Lleida	Catalonia	Spain	600
HM901757	EZSPC919-10	RVcoll.09-V892	x	*Nymphalidae*	*Apatura*	*iris*	Arros, Vall d`Aran	Lleida	Catalonia	Spain	906.4
JF847980	EZSPN031-09	RVcoll.09-T066	x	*Nymphalidae*	*Apatura*	*iris*	Sant Joan de Toran, Vall d`Aran	Lleida	Catalonia	Spain	990
KP870299	EZSPM666-12	RVcoll.12-L279	x	*Nymphalidae*	*Apatura*	*iris*	Serra do Courel, Alto do Couto	Lugo	Galicia	Spain	1400
HQ003976	EZROM013-08	RV-07-D010		*Nymphalidae*	*Apatura*	*metis*	Canaraua Fetei (Baneasa)	Constanta	Dobrogea	Romania	20
HQ003975	EZRMN376-08	RVcoll.08-M779		*Nymphalidae*	*Apatura*	*metis*	2 km E of Smardan	Tulcea	Dobrogea	Romania	5
HQ003977	EZROM014-08	RV-07-E441		*Nymphalidae*	*Apatura*	*metis*	2 km E of Smardan	Tulcea	Dobrogea	Romania	5
HQ003978	EZROM701-08	RV-07-F565		*Nymphalidae*	*Apatura*	*metis*	2 km E of Smardan	Tulcea	Dobrogea	Romania	5
HQ003979	EZROM702-08	RV-07-F566		*Nymphalidae*	*Apatura*	*metis*	2 km E of Smardan	Tulcea	Dobrogea	Romania	5
KP870619	EULEP198-14	12-L009	x	*Nymphalidae*	*Apatura*	*iris*	Viegas, Somiedo		Asturias	Spain	
KP870337	EULEP199-14	12-L010	x	*Nymphalidae*	*Apatura*	*iris*	Puente a Argul, Pesoz		Asturias	Spain	
HM393201	GWORA2479-09	BC ZSM Lep 30691		*Nymphalidae*	*Aphantopus*	*hyperantus*	Ruderting	Niederbayern	Bavaria	Germany	430
GU655027	FBLMT907-09	BC ZSM Lep 25467		*Nymphalidae*	*Aphantopus*	*hyperantus*	Diessen am Ammersee, Alte Ammer	Oberbayern	Bavaria	Germany	535
HM393200	GWORA2478-09	BC ZSM Lep 30690		*Nymphalidae*	*Aphantopus*	*hyperantus*	Leak near Regensburg, Thiergarten	Oberpfalz	Bavaria	Germany	330
HQ003982	EZROM335-08	RV-08-A013		*Nymphalidae*	*Aphantopus*	*hyperantus*	Apuseni Mts., Rimetea (Piatra Secuiului)	Alba	Transylvania	Romania	750
HQ003980	EZROM354-08	RV-08-A032		*Nymphalidae*	*Aphantopus*	*hyperantus*	Apuseni Mts., Rimetea (Piatra Secuiului)	Alba	Transylvania	Romania	750
HQ003984	EZROM766-08	RVcoll.08-M514		*Nymphalidae*	*Aphantopus*	*hyperantus*	Apuseni Mts., Rimetea (Piatra Secuiului)	Alba	Transylvania	Romania	800

GenBank accession number	BOLD Process ID	Sample ID	Used in the Iberian dataset	Family	Genus	Species	Exact Site	Region	State/Province	Country	Alt. (m)
KP870307	EULEP043-14	08-H021		*Nymphalidae*	*Aphantopus*	*hyperantus*	Poiana Santivan, Popesti (jud. Cluj)	Cluj	Transylvania	Romania	
HQ003985	EZROM015-08	RV-06-M894		*Nymphalidae*	*Aphantopus*	*hyperantus*	Cluj-Napoca, dealul Feleacului, Gheorgheni forest	Cluj	Transylvania	Romania	600
HQ003981	EZROM333-08	RV-08-A011		*Nymphalidae*	*Aphantopus*	*hyperantus*	Cluj-Napoca, dealul Feleacului, Gheorgheni forest	Cluj	Transylvania	Romania	600
HQ003986	EZROM016-08	RV-06-M901		*Nymphalidae*	*Aphantopus*	*hyperantus*	Stana	Salaj	Transylvania	Romania	500
HQ003983	EZROM570-08	RV-07-C351		*Nymphalidae*	*Aphantopus*	*hyperantus*	Sunatori, valea Colbul	Suceava	Moldavia	Romania	800
GU676023	EZSPM082-09	RVcoll.08-P402	x	*Nymphalidae*	*Aphantopus*	*hyperantus*	Cotillos		Cantabria	Spain	1105
GU676210	EZSPN949-09	RVcoll.08-L939	x	*Nymphalidae*	*Aphantopus*	*hyperantus*	Rascafria		Comunidad de Madrid	Spain	1150
KP870632	EZSPC090-09	RVcoll.08-R269	x	*Nymphalidae*	*Aphantopus*	*hyperantus*	Masia del Plans, Vidra, La Garrotxa	Barcelona	Catalonia	Spain	1200
KP870518	EZSPC091-09	RVcoll.08-R192	x	*Nymphalidae*	*Aphantopus*	*hyperantus*	Setcases	Girona	Catalonia	Spain	1350
KP870667	EZSPC093-09	RVcoll.08-P094	x	*Nymphalidae*	*Aphantopus*	*hyperantus*	Sanavastre, Riberes del Segre, Cerdanya	Girona	Catalonia	Spain	1100
HM901644	EZSPC792-10	RVcoll.08-P093	x	*Nymphalidae*	*Aphantopus*	*hyperantus*	Sanavastre, Riberes del Segre, Cerdanya	Girona	Catalonia	Spain	1100
HM901682	EZSPC840-10	RVcoll.08-R193	x	*Nymphalidae*	*Aphantopus*	*hyperantus*	Setcases, El Ripolles	Girona	Catalonia	Spain	1321.7
HM901750	EZSPC912-10	RVcoll.09-V812	x	*Nymphalidae*	*Aphantopus*	*hyperantus*	Vilallonga de Ter, Ripolles	Girona	Catalonia	Spain	1143.6
HM901596	EZSPC741-10	RVcoll.08-J859.1	x	*Nymphalidae*	*Aphantopus*	*hyperantus*	Posada de Valdeon	Leon	Castilla y Leon	Spain	
HM901673	EZSPC825-10	RVcoll.08-P770	x	*Nymphalidae*	*Aphantopus*	*hyperantus*	Ocejo de la Pena	Leon	Castilla y Leon	Spain	1265
HM901674	EZSPC826-10	RVcoll.08-P774	x	*Nymphalidae*	*Aphantopus*	*hyperantus*	Ocejo de la Pena	Leon	Castilla y Leon	Spain	1265
KP870938	EZSPC089-09	RVcoll.08-M931	x	*Nymphalidae*	*Aphantopus*	*hyperantus*	Vilamos, Vall d`Aran	Lleida	Catalonia	Spain	1178
HM901650	EZSPC800-10	RVcoll.08-P375	x	*Nymphalidae*	*Aphantopus*	*hyperantus*	Arros, Vall d`Aran	Lleida	Catalonia	Spain	906.4
GU688508	GWORO779-09	BC ZSM Lep 30471		*Pieridae*	*Aporia*	*crataegi*	Machtlfing Starnberg		Bavaria	Germany	
HM393180	GWORA2447-09	BC ZSM Lep 30659		*Pieridae*	*Aporia*	*crataegi*	Kleinwissing	Niederbayern	Bavaria	Germany	355
JF415668	GWORK512-09	BC ZSM Lep 21842		*Pieridae*	*Aporia*	*crataegi*	Diessen	Oberbayern	Bavaria	Germany	600
GU676579	EZSPN564-09	RVcoll.08-J105	x	*Pieridae*	*Aporia*	*crataegi*	Ribeira de Varzea (EN311), Varzea Cova, Fafe		Minho	Portugal	500
HQ003990	EZROM769-08	RVcoll.08-M206		*Pieridae*	*Aporia*	*crataegi*	3 Km SW of Lunca Corbului	Arges	Muntenia	Romania	235
HQ003987	EZROM298-08	RV-07-E660		*Pieridae*	*Aporia*	*crataegi*	Dealul Istrita, Breaza	Buzau	Muntenia	Romania	380
HQ003992	EZROM017-08	RV-06-M826		*Pieridae*	*Aporia*	*crataegi*	Cluj-Napoca, dealul Feleacului, Gheorgheni forest	Cluj	Transylvania	Romania	600
HQ003993	EZROM018-08	RV-07-D461		*Pieridae*	*Aporia*	*crataegi*	Catcau	Cluj	Transylvania	Romania	255
HQ003991	EZROM767-08	RVcoll.06-K660		*Pieridae*	*Aporia*	*crataegi*	Cetatea Fetei (Cluj-Napoca)	Cluj	Transylvania	Romania	450
HQ003989	EZROM768-08	RVcoll.07-D450		*Pieridae*	*Aporia*	*crataegi*	Catcau	Cluj	Transylvania	Romania	255
HQ003988	EZROM770-08	RVcoll.08-M385		*Pieridae*	*Aporia*	*crataegi*	Garboavele forest	Galati	Moldavia	Romania	90
GU676649	EZSPN392-09	RVcoll.08-H448	x	*Pieridae*	*Aporia*	*crataegi*	Puerto de Pozazal		Cantabria	Spain	1026
GU676314	EZSPN836-09	RVcoll.08-L660	x	*Pieridae*	*Aporia*	*crataegi*	San Martin del Pimpollar, Piscifactoria Rasquilla	Avila	Castilla y Leon	Spain	1270
JN827862	EZROM706-08	RV-06-H886	x	*Pieridae*	*Aporia*	*crataegi*	Campus UAB, Bellaterra	Barcelona	Catalonia	Spain	150
GU675665	EZSPC094-09	RVcoll.07-C400	x	*Pieridae*	*Aporia*	*crataegi*	Cardedeu, Valles Oriental	Barcelona	Catalonia	Spain	190
JN827864	EZSPC211-09	RVcoll.08-H294	x	*Pieridae*	*Aporia*	*crataegi*	Borreda, Bergueda	Barcelona	Catalonia	Spain	850
GU676869	EZSPN461-09	RVcoll.08-H670	x	*Pieridae*	*Aporia*	*crataegi*	Grazalema, Puerto de los Alamillos	Cadiz	Andalusia	Spain	887
JN827863	EZSPC215-09	RVcoll.08-J386	x	*Pieridae*	*Aporia*	*crataegi*	Estanys de la Jonquera, La Jonquera, Alt Emporda	Girona	Catalonia	Spain	180
GU676465	EZSPN681-09	RVcoll.08-L003	x	*Pieridae*	*Aporia*	*crataegi*	Carretera al Veleta, Gueejar Sierra	Granada	Andalusia	Spain	1266
GU676524	EZSPN618-09	RVcoll.08-J892	x	*Pieridae*	*Aporia*	*crataegi*	San Esteban de Valdueza	Leon	Castilla y Leon	Spain	
JN827865	EZSPC224-09	RVcoll.08-L482	x	*Pieridae*	*Aporia*	*crataegi*	Bassa d`Arres, Vall d`Aran	Lleida	Catalonia	Spain	1501
GU675853	EZSPM254-09	RVcoll.08-R466	x	*Pieridae*	*Aporia*	*crataegi*	Celanova, Vieiro de Abaixo	Ourense	Galicia	Spain	817
GU676905	EZSPN500-09	RVcoll.08-H949	x	*Pieridae*	*Aporia*	*crataegi*	Candelario, Sierra de Candelario	Salamanca	Castilla y Leon	Spain	1300
HM901209	EZSPC1005-10	RVcoll.08-H342	x	*Pieridae*	*Aporia*	*crataegi*	Sierra de Javalambre, Alpuente	Valencia	Comunidad Valenciana	Spain	1100
GU655026	FBLMT893-09	BC ZSM Lep 25453		*Nymphalidae*	*Araschnia*	*levana*	Neustadt/Donau, Plattenberg	Niederbayern	Bavaria	Germany	370
GU707092	FBLMU447-09	BC ZSM Lep 27097		*Nymphalidae*	*Araschnia*	*levana*	Diessen, Huebschenried	Oberbayern	Bavaria	Germany	650
HQ004000	EZROM021-08	RV-06-M926		*Nymphalidae*	*Araschnia*	*levana*	Dealul Istrita, Breaza	Buzau	Muntenia	Romania	600
HQ003997	EZROM022-08	RV-07-D599		*Nymphalidae*	*Araschnia*	*levana*	Teregova	Caras-Severin	Banat	Romania	385
KP871126	EULEP173-14	11-J322		*Nymphalidae*	*Araschnia*	*levana*	Gheorgheni, Dealul Feleacului, Cluj-Napoca	Cluj	Transylvania	Romania	550-650
HQ003998	EZROM019-08	RV-06-K528		*Nymphalidae*	*Araschnia*	*levana*	Badeni	Cluj	Transylvania	Romania	440
HQ003999	EZROM020-08	RV-06-K530		*Nymphalidae*	*Araschnia*	*levana*	Badeni	Cluj	Transylvania	Romania	440

GenBank accession number	BOLD Process ID	Sample ID	Used in the Iberian dataset	Family	Genus	Species	Exact Site	Region	State/Province	Country	Alt. (m)
KP870236	EULEP107-14	09-V628		*Nymphalidae*	*Araschnia*	*levana*	Gheorgheni, Valea Belchia	Harghita	Transylvania	Romania	1000
HQ003996	EZROM772-08	RVcoll.07-D566		*Nymphalidae*	*Araschnia*	*levana*	Spini	Hunedoara	Transylvania	Romania	240
HQ003994	EZROM571-08	RV-07-C348		*Nymphalidae*	*Araschnia*	*levana*	Sunatori, valea Colbul	Suceava	Moldavia	Romania	800
HQ003995	EZROM771-08	RVcoll.07-C999		*Nymphalidae*	*Araschnia*	*levana*	Otasau Valley, Buila-Vanturarita	Valcea	Oltenia	Romania	515
FJ663284	LOWA778-06	2005-LOWA-778		*Nymphalidae*	*Araschnia*	*levana*	Osyno	Sebezh distr.	Pskov	Russia	150
FJ663283	LOWA779-06	2005-LOWA-779		*Nymphalidae*	*Araschnia*	*levana*	Osyno	Sebezh distr.	Pskov	Russia	150
KP870249	EZROM355-08	07-E609	x	*Nymphalidae*	*Araschnia*	*levana*	St. Maria de Besora	Barcelona	Catalonia	Spain	860
HM901511	EZSPC1377-10	RVcoll.09-X300	x	*Nymphalidae*	*Araschnia*	*levana*	Can Jorda, Santa Pau, GARROTXA	Barcelona	Catalonia	Spain	550
KP870217	EZSPC243-09	RVcoll.08-P060	x	*Nymphalidae*	*Araschnia*	*levana*	PEIN, Riberes de l`Alt Segre, Cerdanya	Girona	Catalonia	Spain	1100
KP871134	EZSPC244-09	RVcoll.08-P061	x	*Nymphalidae*	*Araschnia*	*levana*	PEIN, Riberes de l`Alt Segre, Cerdanya	Girona	Catalonia	Spain	1100
KP870269	EZSPC245-09	RVcoll.08-P062	x	*Nymphalidae*	*Araschnia*	*levana*	PEIN, Riberes de l`Alt Segre, Cerdanya	Girona	Catalonia	Spain	1100
GU676119	EZSPM404-09	Rvcoll.09-V810	x	*Nymphalidae*	*Araschnia*	*levana*	Vilallonga de Ter, Ripolles	Girona	Catalonia	Spain	
GU676120	EZSPM405-09	Rvcoll.09-V811	x	*Nymphalidae*	*Araschnia*	*levana*	Vilallonga de Ter, Ripolles	Girona	Catalonia	Spain	
HQ004007	EZROM023-08	RV-06-V702		*Nymphalidae*	*Arethusana*	*arethusa*	Padurea Craiului Mts., Suncuius	Bihor	Transylvania	Romania	370
HQ004001	EZROM660-08	RV-06-V709		*Nymphalidae*	*Arethusana*	*arethusa*	Padurea Craiului Mts., Suncuius	Bihor	Transylvania	Romania	370
HQ004005	EZROM773-08	RVcoll.06-V707		*Nymphalidae*	*Arethusana*	*arethusa*	Padurea Craiului Mts., Suncuius	Bihor	Transylvania	Romania	370
HQ004003	EZROM774-08	RVcoll.06-V710		*Nymphalidae*	*Arethusana*	*arethusa*	Padurea Craiului Mts., Suncuius	Bihor	Transylvania	Romania	370
HQ004006	EZROM957-08	RVcoll.06-V708.1		*Nymphalidae*	*Arethusana*	*arethusa*	Padurea Craiului Mts., Suncuius	Bihor	Transylvania	Romania	370
HQ004008	EZROM024-08	RV-07-E414		*Nymphalidae*	*Arethusana*	*arethusa*	8 Km S of Babadag (Babadag forest)	Tulcea	Dobrogea	Romania	115
HQ004002	EZROM598-08	RV-07-E432		*Nymphalidae*	*Arethusana*	*arethusa*	3 km E of Slava Rusa (Babadag forest)	Tulcea	Dobrogea	Romania	130
HQ004004	EZROM775-08	RVcoll.07-E431		*Nymphalidae*	*Arethusana*	*arethusa*	3 km E of Slava Rusa (Babadag forest)	Tulcea	Dobrogea	Romania	130
HM901792	EZSPC956-10	RVcoll.09-X570	x	*Nymphalidae*	*Arethusana*	*arethusa*	Sorauren		Navarra	Spain	530
KP870423	EZSPM579-12	RVcoll.08-P937	x	*Nymphalidae*	*Arethusana*	*arethusa*	Caranga		Asturias	Spain	540
KP870871	EZSPM582-12	RVcoll.08-P940	x	*Nymphalidae*	*Arethusana*	*arethusa*	Cabo Penas, Luanco		Asturias	Spain	65
KP870380	EZSPN1117-11	Rvcoll.10-C316	x	*Nymphalidae*	*Arethusana*	*arethusa*	Cabo de Penas, Luanco		Asturias	Spain	
KP870834	EZSPN1094-11	Rvcoll.10-A008	x	*Nymphalidae*	*Arethusana*	*arethusa*	La Herguijuela	Avila	Castilla y Leon	Spain	1560
KP870689	EZSPN1095-11	Rvcoll.10-A012	x	*Nymphalidae*	*Arethusana*	*arethusa*	Sierra de Gredos	Avila	Castilla y Leon	Spain	1650
KP870373	EZSPC197-09	RVcoll.06-G545	x	*Nymphalidae*	*Arethusana*	*arethusa*	Can Rovira, El Brull, Osona	Barcelona	Catalonia	Spain	830
KP870920	EZSPC198-09	RVcoll.06-G565	x	*Nymphalidae*	*Arethusana*	*arethusa*	El Sola, El Brull, Osona	Barcelona	Catalonia	Spain	800
KP871146	EZSPC267-09	RVcoll.08-R155	x	*Nymphalidae*	*Arethusana*	*arethusa*	Montvi de Baix, Moianes	Barcelona	Catalonia	Spain	800
KP870730	EZSPM850-12	RVcoll.170410YM76	x	*Nymphalidae*	*Arethusana*	*arethusa*	Huelamo	Cuenca	Castilla-La Mancha	Spain	1400
GU676873	EZSPN465-09	RVcoll.08-H679	x	*Nymphalidae*	*Arethusana*	*arethusa*	Huelamo, Rio Jucar	Cuenca	Castilla-La Mancha	Spain	1237
HM901779	EZSPC942-10	RVcoll.09-X057	x	*Nymphalidae*	*Arethusana*	*arethusa*	Barranc de Montmalus, La Cerdanya	Girona	Catalonia	Spain	1340
HM901530	EZSPC1396-10	RVcoll.09-V107	x	*Nymphalidae*	*Arethusana*	*arethusa*	W.Sierra Nevada, barranco de las viboras	Granada	Andalusia	Spain	1800
HM901531	EZSPC1397-10	RVcoll.09-V108	x	*Nymphalidae*	*Arethusana*	*arethusa*	W.Sierra Nevada, barranco de las viboras	Granada	Andalusia	Spain	1800
HM901543	EZSPC1441-10	RVcoll.09-V153	x	*Nymphalidae*	*Arethusana*	*arethusa*	W. Sierra Nevada, barranco de las viboras	Granada	Andalusia	Spain	1900
HM901544	EZSPC1442-10	RVcoll.09-V154	x	*Nymphalidae*	*Arethusana*	*arethusa*	W. Sierra Nevada, barranco de las viboras	Granada	Andalusia	Spain	1900
GU676355	EZSPN792-09	RVcoll.08-L351	x	*Nymphalidae*	*Arethusana*	*arethusa*	Sierra Nevada	Granada	Andalusia	Spain	
KP871148	EZSPC818-10	RVcoll.08-P699	x	*Nymphalidae*	*Arethusana*	*arethusa*	Villanueva de Alcoron, ctra. CU2101 km 10	Guadalajara	Castilla-La Mancha	Spain	1358
GU675735	EZSPM105-09	RVcoll.08-P440	x	*Nymphalidae*	*Arethusana*	*arethusa*	Villanueva de Alcoron, ctra. CU2101 km 10, Alto Tajo	Guadalajara	Castilla-La Mancha	Spain	1358
HM901595	EZSPC740-10	RVcoll.08-J829.1	x	*Nymphalidae*	*Arethusana*	*arethusa*	Compludo	Leon	Castilla y Leon	Spain	
JN278892	EZSPN1115-11	Rvcoll.10-C314	x	*Nymphalidae*	*Arethusana*	*arethusa*	Abelgas de Luna	Leon	Castilla y Leon	Spain	1400
JN278893	EZSPN1116-11	Rvcoll.10-C315	x	*Nymphalidae*	*Arethusana*	*arethusa*	Abelgas de Luna	Leon	Castilla y Leon	Spain	1400
KP871168	EZSPC202-09	RVcoll.06-V774	x	*Nymphalidae*	*Arethusana*	*arethusa*	Coll de Boixols, Abella de la Conca, Pallars Jussa	Lleida	Catalonia	Spain	1365
KP871070	EZSPC252-09	RVcoll.08-P333	x	*Nymphalidae*	*Arethusana*	*arethusa*	Perves, Pallars Jussa	Lleida	Catalonia	Spain	1071
HM901651	EZSPC801-10	RVcoll.08-P427	x	*Nymphalidae*	*Arethusana*	*arethusa*	Pena de la Cruz, Bejar	Salamanca	Castilla y Leon	Spain	1380
HM901652	EZSPC802-10	RVcoll.08-P428	x	*Nymphalidae*	*Arethusana*	*arethusa*	Pena de la Cruz, Bejar	Salamanca	Castilla y Leon	Spain	1380
GU675728	EZSPM098-09	RVcoll.08-P426	x	*Nymphalidae*	*Arethusana*	*arethusa*	Pena de la Cruz, Bejar	Salamanca	Castilla y Leon	Spain	1380
HM901665	EZSPC816-10	RVcoll.08-P617	x	*Nymphalidae*	*Arethusana*	*arethusa*	Albarracin	Teruel	Aragon	Spain	1250
KP870621	EZSPM764-12	RVcoll.040611HZ07	x	*Nymphalidae*	*Arethusana*	*arethusa*	Fuentes Calientes	Teruel	Aragon	Spain	1273
GU676394	EZSPN756-09	RVcoll.08-L233	x	*Nymphalidae*	*Arethusana*	*arethusa*	Ruta de la Sima, El Vallecillo	Teruel	Aragon	Spain	1531
KP870352	EULEP253-14	12-Q447	x	*Nymphalidae*	*Arethusana*	*arethusa*	Grado del Pico	Segovia	Castilla y León	Spain	1260
KP870388	EULEP254-14	12-Q448	x	*Nymphalidae*	*Arethusana*	*arethusa*	Grado del Pico	Segovia	Castilla y León	Spain	1260
GU688471	GWORO811-09	BC ZSM Lep 30503		*Nymphalidae*	*Argynnis*	*adippe*	Nantesbuch Bd. Heilbrunn / TOEL		Bavaria	Germany	
GU707091	FBLMU446-09	BC ZSM Lep 27096		*Nymphalidae*	*Argynnis*	*adippe*	Inning am Ammersee, Martinsberg b. Inning	Oberbayern	Bavaria	Germany	585

GenBank accession number	BOLD Process ID	Sample ID	Used in the Iberian dataset	Family	Genus	Species	Exact Site	Region	State/Province	Country	Alt. (m)
JF415670	GWORK514-09	BC ZSM Lep 21844		*Nymphalidae*	*Argynnis*	*adippe*	Diessen	Oberbayern	Bavaria	Germany	600
JF415669	GWORO720-09	BC ZSM Lep 27656		*Nymphalidae*	*Argynnis*	*adippe*	Oberschleissheim, MW 20	Oberbayern	Bavaria	Germany	480
GU707127	FBLMU403-09	BC ZSM Lep 27053		*Nymphalidae*	*Argynnis*	*adippe*	Laaber, Endorfmuehle	Oberpfalz	Bavaria	Germany	420
GU676561	EZSPN582-09	RVcoll.08-J141	x	*Nymphalidae*	*Argynnis*	*adippe*	Ponte Pedrinha, Beca, Boticas		Tras-os-Montes	Portugal	730
HQ004013	EZROM026-08	RV-06-M950		*Nymphalidae*	*Argynnis*	*adippe*	Dealul Istrita, Breaza	Buzau	Muntenia	Romania	600
HQ004014	EZROM027-08	RV-07-C968		*Nymphalidae*	*Argynnis*	*adippe*	Baile Herculane (Pecinisca)	Caras-Severin	Banat	Romania	220
HQ004011	EZROM776-08	RVcoll.07-C913		*Nymphalidae*	*Argynnis*	*adippe*	Teregova	Caras-Severin	Banat	Romania	385
HQ004012	EZROM025-08	RV-06-M882		*Nymphalidae*	*Argynnis*	*adippe*	Cluj-Napoca, dealul Feleacului, Gheorgheni forest	Cluj	Transylvania	Romania	600
KP871142	EULEP133-14	10-C373		*Nymphalidae*	*Argynnis*	*adippe*	Filpea-Gălăuţaş	Harghita	Transylvania	Romania	
HQ004009	EZROM608-08	RV-07-D509		*Nymphalidae*	*Argynnis*	*adippe*	Cheile Babei	Maramures	Maramures	Romania	265
HQ004010	EZROM572-08	RV-07-C347		*Nymphalidae*	*Argynnis*	*adippe*	Sunatori, valea Colbul	Suceava	Moldavia	Romania	800
GU675705	EZSPM133-09	RVcoll.08-P643	x	*Nymphalidae*	*Argynnis*	*adippe*	Valle (Valle-Zurea), Lena		Asturias	Spain	662
KP870958	EZROM357-08	07-E611	x	*Nymphalidae*	*Argynnis*	*adippe*	Vallforners, Canoves	Barcelona	Catalonia	Spain	650
KP870687	EZSPC279-09	RVcoll.08-R279	x	*Nymphalidae*	*Argynnis*	*adippe*	Masia del Plans, Vidra, La Garrotxa	Barcelona	Catalonia	Spain	1200
JN272495	EZSPN1047-11	Rvcoll.08-L177	x	*Nymphalidae*	*Argynnis*	*adippe*	Font del Regatxal, Vilafranca	Castellon	Comunidad Valenciana	Spain	1361
JN272496	EZSPN1060-11	Rvcoll.08-P610	x	*Nymphalidae*	*Argynnis*	*adippe*	Mogorrita, Tragacete	Cuenca	Castilla-La Mancha	Spain	1748
GU676874	EZSPN468-09	RVcoll.08-H689	x	*Nymphalidae*	*Argynnis*	*adippe*	Huelamo, Rio Jucar	Cuenca	Castilla-La Mancha	Spain	1237
KP870872	EZSPC268-09	RVcoll.08-R163	x	*Nymphalidae*	*Argynnis*	*adippe*	Llanars, Ripolles	Girona	Catalonia	Spain	1119
HM901764	EZSPC927-10	RVcoll.09-V939	x	*Nymphalidae*	*Argynnis*	*adippe*	Sierra de la Sagra	Granada	Andalusia	Spain	1745
GU676215	EZSPN938-09	RVcoll.08-L901	x	*Nymphalidae*	*Argynnis*	*adippe*	San Andres del Rey	Guadalajara	Castilla-La Mancha	Spain	1018
GU676527	EZSPN621-09	RVcoll.08-J819	x	*Nymphalidae*	*Argynnis*	*adippe*	Compludo	Leon	Castilla y Leon	Spain	
KP870608	EZROM356-08	07-E610	x	*Nymphalidae*	*Argynnis*	*adippe*	Refugi dels Rasos	Lleida	Catalonia	Spain	1880
KP870913	EZSPC282-09	RVcoll.08-R307	x	*Nymphalidae*	*Argynnis*	*adippe*	Planes de Son, Pallars Sobira	Lleida	Catalonia	Spain	1589
JN272499	EZSPN1099-11	Rvcoll.10-A823	x	*Nymphalidae*	*Argynnis*	*adippe*	Boixols, Alt Urgell	Lleida	Catalonia	Spain	1225
JN272502	EZSPN1107-11	Rvcoll.10-B511	x	*Nymphalidae*	*Argynnis*	*adippe*	Coll de la Creu, Ports de Tortosa	Tarragona	Catalonia	Spain	1120
JN272503	EZSPN1108-11	Rvcoll.10-B512	x	*Nymphalidae*	*Argynnis*	*adippe*	Coll de la Creu, Ports de Tortosa	Tarragona	Catalonia	Spain	1120
HM901512	EZSPC1378-10	RVcoll.09-X507	x	*Nymphalidae*	*Argynnis*	*adippe*	2 km NW El Vallecillo	Teruel	Aragon	Spain	1450
JN272504	EZSPN1110-11	Rvcoll.10-B543	x	*Nymphalidae*	*Argynnis*	*adippe*	El Valecillo	Teruel	Aragon	Spain	1490
GU676585	EZSPN559-09	RVcoll.08-J079	x	*Nymphalidae*	*Argynnis*	*adippe*	Teresa, Caroig	Valencia	Comunidad Valenciana	Spain	
GU688470	GWORO810-09	BC ZSM Lep 30502		*Nymphalidae*	*Argynnis*	*aglaja*	Zellwies Koenigsdorf TOEL		Bavaria	Germany	
GU707194	FBLMU111-09	BC ZSM Lep 25621		*Nymphalidae*	*Argynnis*	*aglaja*	Lalling, Durchfurtherschuss	Niederbayern	Bavaria	Germany	735
HQ565468	GWOSA735-10	BC ZSM Lep 35746		*Nymphalidae*	*Argynnis*	*aglaja*	N Lalling	Niederbayern	Bavaria	Germany	800
GU655025	FBLMT912-09	BC ZSM Lep 25472		*Nymphalidae*	*Argynnis*	*aglaja*	Kallmuenz, Kirchenberg	Oberpfalz	Bavaria	Germany	390
GU707115	FBLMU414-09	BC ZSM Lep 27064		*Nymphalidae*	*Argynnis*	*aglaja*	Kallmuenz, Kirchenberg	Oberpfalz	Bavaria	Germany	390
JF860019	PHLAC443-10	TLMF Lep 02478		*Nymphalidae*	*Argynnis*	*aglaja*	Ritten/ Obergruenwald		South Tyrol	Italy	1750
HQ004020	EZROM029-08	RV-06-M932		*Nymphalidae*	*Argynnis*	*aglaja*	Dealul Istrita, Breaza	Buzau	Muntenia	Romania	600
HQ004021	EZROM030-08	RV-06-M936		*Nymphalidae*	*Argynnis*	*aglaja*	Dealul Istrita, Breaza	Buzau	Muntenia	Romania	600
KP870389	EULEP041-14	08-H011		*Nymphalidae*	*Argynnis*	*aglaja*	Cluj-Napoca, Dealul Feleacului	Cluj	Transylvania	Romania	
HQ004019	EZROM028-08	RV-06-M881		*Nymphalidae*	*Argynnis*	*aglaja*	Cluj-Napoca, dealul Feleacului, Gheorgheni forest	Cluj	Transylvania	Romania	600
HQ004018	EZROM031-08	RV-07-D935		*Nymphalidae*	*Argynnis*	*aglaja*	11 km south of Ranca	Gorj	Oltenia	Romania	940
HQ004015	EZROM600-08	RV-07-D517		*Nymphalidae*	*Argynnis*	*aglaja*	Rohia	Maramures	Maramures	Romania	360
HQ004016	EZROM777-08	RVcoll.07-D496		*Nymphalidae*	*Argynnis*	*aglaja*	Cheile Babei	Maramures	Maramures	Romania	265
HQ004017	EZROM778-08	RVcoll.07-D526		*Nymphalidae*	*Argynnis*	*aglaja*	Rohia	Maramures	Maramures	Romania	360
KP870537	EULEP193-14	11-J955		*Nymphalidae*	*Argynnis*	*aglaja*	Baia Mare	Maramureș	Maramureş	Romania	
GU676277	EZSPN879-09	RVcoll.08-L782	x	*Nymphalidae*	*Argynnis*	*aglaja*	San Andres		Cantabria	Spain	972
GU676163	EZSPN999-09	RVcoll.08-M082	x	*Nymphalidae*	*Argynnis*	*aglaja*	Oteruelo del Valle		Comunidad de Madrid	Spain	1148
KP870242	EZSPC248-09	RVcoll.08-P222	x	*Nymphalidae*	*Argynnis*	*aglaja*	La Molina	Barcelona	Catalonia	Spain	1622
KP870385	EZSPC266-09	RVcoll.08-R150	x	*Nymphalidae*	*Argynnis*	*aglaja*	Turo de L`Home, Montseny	Barcelona	Catalonia	Spain	1643
GU676425	EZSPN721-09	RVcoll.08-L110	x	*Nymphalidae*	*Argynnis*	*aglaja*	Puerto de las Cabrillas, Portell de Morella	Castellon	Comunidad Valenciana	Spain	1301
KP870544	EZSPC208-09	RVcoll.07-E637	x	*Nymphalidae*	*Argynnis*	*aglaja*	Meranges-Guils	Girona	Catalonia	Spain	1555

GenBank accession number	BOLD Process ID	Sample ID	Used in the Iberian dataset	Family	Genus	Species	Exact Site	Region	State/Province	Country	Alt. (m)
HM901240	EZSPC1036-10	RVcoll.08-J217	x	*Nymphalidae*	*Argynnis*	*aglaja*	Campos de Otero, Sierra Nevada, Gueejar Sierra	Granada	Andalusia	Spain	2200
HM901402	EZSPC1202-10	RVcoll.09-V428	x	*Nymphalidae*	*Argynnis*	*aglaja*	San Juan (Sierra Nevada)	Granada	Andalusia	Spain	2760
GU675764	EZSPM227-09	RVcoll.08-R419	x	*Nymphalidae*	*Argynnis*	*aglaja*	Cabillanes, Huergas de Babia	Leon	Castilla y Leon	Spain	1220
GU676516	EZSPN622-09	RVcoll.08-J854	x	*Nymphalidae*	*Argynnis*	*aglaja*	Posada de Valdeon	Leon	Castilla y Leon	Spain	
KP870393	EZSPC236-09	RVcoll.08-M928	x	*Nymphalidae*	*Argynnis*	*aglaja*	Vilamos, Vall d`Aran	Lleida	Catalonia	Spain	1178
KP870221	EZSPC256-09	RVcoll.08-P360	x	*Nymphalidae*	*Argynnis*	*aglaja*	Perves, Pallars Jussa	Lleida	Catalonia	Spain	1071
GU676304	EZSPN851-09	RVcoll.08-L698	x	*Nymphalidae*	*Argynnis*	*aglaja*	Candelario	Salamanca	Castilla y Leon	Spain	1300
HQ004023	EZROM033-08	RV-07-E465		*Nymphalidae*	*Argynnis*	*laodice*	Dumbrava Vadului, Vad	Brasov	Transylvania	Romania	495
HQ004022	EZROM032-08	RV-06-M971		*Nymphalidae*	*Argynnis*	*laodice*	Stejarenii	Mures	Transylvania	Romania	550
HQ004024	EZROM779-08	RVcoll.08-M653		*Nymphalidae*	*Argynnis*	*laodice*	Stejarenii	Mures	Transylvania	Romania	550
HQ004025	EZROM780-08	RVcoll.08-M654		*Nymphalidae*	*Argynnis*	*laodice*	Stejarenii	Mures	Transylvania	Romania	550
KP870789	EZROM358-08	07-E612	x	*Nymphalidae*	*Argynnis*	*niobe*	road Sainte-Leocadie to Puigmal	Languedoc-Roussillon		France	1550
GU688468	GWORO812-09	BC ZSM Lep 30504		*Nymphalidae*	*Argynnis*	*niobe*	Samerberg (RO) Frasdorf		Bavaria	Germany	960
HM393220	GWORA2504-09	BC ZSM Lep 30716		*Nymphalidae*	*Argynnis*	*niobe*	Sudelfeld, Arzmoos	Oberbayern	Bavaria	Germany	1200
HQ004032	EZROM034-08	RV-06-M980		*Nymphalidae*	*Argynnis*	*niobe*	Apuseni Mts., Rimetea (Piatra Secuiului)	Alba	Transylvania	Romania	750
HQ004026	EZRMN337-08	RVcoll.08-H017		*Nymphalidae*	*Argynnis*	*niobe*	Dealul Sf. Pavel (Cluj-Napoca)	Cluj	Transylvania	Romania	450
HQ004031	EZROM035-08	RV-07-D499		*Nymphalidae*	*Argynnis*	*niobe*	Cheile Babei	Maramures	Maramures	Romania	265
KP870945	EULEP139-14	10-C407		*Nymphalidae*	*Argynnis*	*niobe*	Ţibleş	Maramureş	Maramureş	Romania	
HQ004027	EZROM599-08	RV-07-D052		*Nymphalidae*	*Argynnis*	*niobe*	Babadag forest	Tulcea	Dobrogea	Romania	115
HQ004028	EZROM781-08	RVcoll.07-D049		*Nymphalidae*	*Argynnis*	*niobe*	8 Km S of Babadag (Babadag forest)	Tulcea	Dobrogea	Romania	115
HQ004029	EZROM782-08	RVcoll.08-M473		*Nymphalidae*	*Argynnis*	*niobe*	Babadag forest (10 Km S of Babadag)	Tulcea	Dobrogea	Romania	115
HQ004030	EZROM783-08	RVcoll.08-M427		*Nymphalidae*	*Argynnis*	*niobe*	8 Km S of Babadag (Babadag forest)	Tulcea	Dobrogea	Romania	115
HM901284	EZSPC1083-10	RVcoll.08-L736	x	*Nymphalidae*	*Argynnis*	*niobe*	Manzanares el Real		Comunidad de Madrid	Spain	934
HM901726	EZSPC886-10	RVcoll.09-T149	x	*Nymphalidae*	*Argynnis*	*niobe*	Puerto de La Morcuera		Comunidad de Madrid	Spain	1578
GU676251	EZSPN904-09	RVcoll.08-L823	x	*Nymphalidae*	*Argynnis*	*niobe*	Puerto de la Morcuera, Rascafria		Comunidad de Madrid	Spain	1438
GU676325	EZSPN825-09	RVcoll.08-L637	x	*Nymphalidae*	*Argynnis*	*niobe*	Hoyocasero, Venta Rasquilla	Avila	Castilla y Leon	Spain	1224
KP870263	EZSPC247-09	RVcoll.08-P220	x	*Nymphalidae*	*Argynnis*	*niobe*	La Molina	Barcelona	Catalonia	Spain	1622
KP870266	EZSPC242-09	RVcoll.08-P050	x	*Nymphalidae*	*Argynnis*	*niobe*	La Valira (near Urus), Cerdanya	Girona	Catalonia	Spain	1300
HM901401	EZSPC1201-10	RVcoll.09-V426	x	*Nymphalidae*	*Argynnis*	*niobe*	San Juan (Sierra Nevada)	Granada	Andalusia	Spain	2760
HM901417	EZSPC1218-10	RVcoll.09-V531	x	*Nymphalidae*	*Argynnis*	*niobe*	Loma de San Francisco (Sierra Nevada)	Granada	Andalusia	Spain	2315
GU676491	EZSPN655-09	RVcoll.08-J951	x	*Nymphalidae*	*Argynnis*	*niobe*	Fuente Albergue de San Francisco, Gueejar Sierra	Granada	Andalusia	Spain	2221
GU676417	EZSPN730-09	RVcoll.08-L138	x	*Nymphalidae*	*Argynnis*	*niobe*	Laguna Seca, Hueneja	Granada	Andalusia	Spain	2276
GU676930	EZSPN528-09	RVcoll.08-H998	x	*Nymphalidae*	*Argynnis*	*niobe*	Vega de Gordon	Leon	Castilla y Leon	Spain	1050
KP870818	EZROM359-08	07-E613	x	*Nymphalidae*	*Argynnis*	*niobe*	Planes de Son, Son	Lleida	Catalonia	Spain	1440
KP870763	EZSPC233-09	RVcoll.08-M901	x	*Nymphalidae*	*Argynnis*	*niobe*	Bassa d`Arres, Vall d`Aran	Lleida	Catalonia	Spain	1500
KP870428	EZSPC264-09	RVcoll.08-R114	x	*Nymphalidae*	*Argynnis*	*niobe*	Salt de Pitx, Vall d`Aran	Lleida	Catalonia	Spain	1570
KP870309	EULEP177-14	11-J354		*Nymphalidae*	*Argynnis*	*pandora*	Breaza, Dealul Istriţa	Buzău	Muntenia	Romania	400-650
KP870222	EULEP178-14	11-J355		*Nymphalidae*	*Argynnis*	*pandora*	Breaza, Dealul Istriţa	Buzău	Muntenia	Romania	400-650
HQ004035	EZROM786-08	RVcoll.08-M436		*Nymphalidae*	*Argynnis*	*pandora*	1 Km NE of Gura Dobrogei reserve	Constanta	Dobrogea	Romania	40
HQ004036	EZROM784-08	RVcoll.08-M273		*Nymphalidae*	*Argynnis*	*pandora*	4 Km SW of Cazanele Mari (around Plavisevita)	Mehedinti	Oltenia	Romania	110
HQ004038	EZROM036-08	RV-07-D069		*Nymphalidae*	*Argynnis*	*pandora*	Greci massif, izvorul Esitul Sufletului	Tulcea	Dobrogea	Romania	145
HQ004037	EZROM037-08	RV-07-D071		*Nymphalidae*	*Argynnis*	*pandora*	Greci massif, izvorul Esitul Sufletului	Tulcea	Dobrogea	Romania	145
HQ004033	EZROM661-08	RV-07-D073		*Nymphalidae*	*Argynnis*	*pandora*	Greci massif, Macin Mts.	Tulcea	Dobrogea	Romania	120
HQ004034	EZROM785-08	RVcoll.08-M404		*Nymphalidae*	*Argynnis*	*pandora*	Macin Mts. (3 Km S of Jijila)	Tulcea	Dobrogea	Romania	230
HM901577	EZSPC722-10	RVcoll.08-H469	x	*Nymphalidae*	*Argynnis*	*pandora*	Soto del Real		Comunidad de Madrid	Spain	1165
HM901578	EZSPC723-10	RVcoll.08-H471	x	*Nymphalidae*	*Argynnis*	*pandora*	Soto del Real		Comunidad de Madrid	Spain	1165
HM901579	EZSPC724-10	RVcoll.08-H482	x	*Nymphalidae*	*Argynnis*	*pandora*	Colmenar Viejo		Comunidad de Madrid	Spain	848

GenBank accession number	BOLD Process ID	Sample ID	Used in the Iberian dataset	Family	Genus	Species	Exact Site	Region	State/Province	Country	Alt. (m)
GU676646	EZSPN401-09	RVcoll.08-H470	x	*Nymphalidae*	*Argynnis*	*pandora*	Soto del Real		Comunidad de Madrid	Spain	1165
HM901801	EZSPC966-10	RVcoll.210207TM10	x	*Nymphalidae*	*Argynnis*	*pandora*	Pista Banyeres-Biar, Biar	Alicante	Comunidad Valenciana	Spain	938
KP871058	EZROM744-08	RV-06-H898	x	*Nymphalidae*	*Argynnis*	*pandora*	El Polell, Parc Natural del Montseny, Valles Oriental	Barcelona	Catalonia	Spain	750
KP870703	EZSPM933-12	Rvcoll.12-M667	x	*Nymphalidae*	*Argynnis*	*pandora*	Rio Jandula, Mestanza	Ciudad Real	Castilla-La Mancha	Spain	407
GU669688	EZSPC579-09	RVcoll.07-C047	x	*Nymphalidae*	*Argynnis*	*pandora*	Puig Neulos, Alt Emporda	Girona	Catalonia	Spain	1110
KP870682	EZSPM658-12	RVcoll.11-I494	x	*Nymphalidae*	*Argynnis*	*pandora*	SE of Cumbres Verdes (La Zubia)	Granada	Andalusia	Spain	1400
GU676503	EZSPN645-09	RVcoll.08-J826	x	*Nymphalidae*	*Argynnis*	*pandora*	Palazuelo de Torio	Leon	Castilla y Leon	Spain	
GU669639	EZSPC642-09	RVcoll.08-L375	x	*Nymphalidae*	*Argynnis*	*pandora*	La Mussara, Plans del Panxo	Tarragona	Catalonia	Spain	850
KP870256	EZSPM898-12	Rvcoll.12-M620	x	*Nymphalidae*	*Argynnis*	*pandora*	Tordesillas, El Montico	Valladolid	Castilla y Leon	Spain	697
HM393218	GWORA2502-09	BC ZSM Lep 30714		*Nymphalidae*	*Argynnis*	*paphia*	Laberveinting, Hart	Niederbayern	Bavaria	Germany	360
HM393219	GWORA2503-09	BC ZSM Lep 30715		*Nymphalidae*	*Argynnis*	*paphia*	Reit im Winkl	Oberbayern	Bavaria	Germany	700
HQ004041	EZROM787-08	RVcoll.08-M545		*Nymphalidae*	*Argynnis*	*paphia*	Valea Feregarilor - Poiana Musuroaie	Caras-Severin	Banat	Romania	320
HQ004040	EZROM788-08	RVcoll.08-M546		*Nymphalidae*	*Argynnis*	*paphia*	Valea Feregarilor - Poiana Musuroaie	Caras-Severin	Banat	Romania	320
KP870278	EULEP085-14	08-P971		*Nymphalidae*	*Argynnis*	*paphia*	Cluj-Napoca, Dealul Feleacului	Cluj	Transylvania	Romania	
KP870784	EULEP172-14	11-J316		*Nymphalidae*	*Argynnis*	*paphia*	Gheorgheni, Dealul Feleacului, Cluj-Napoca	Cluj	Transylvania	Romania	550-650
HQ004043	EZRMN340-08	RVcoll.08-H027		*Nymphalidae*	*Argynnis*	*paphia*	Faget (Cluj-Napoca)	Cluj	Transylvania	Romania	680
HQ004044	EZRMN341-08	RVcoll.08-H028		*Nymphalidae*	*Argynnis*	*paphia*	Faget (Cluj-Napoca)	Cluj	Transylvania	Romania	680
HQ004042	EZROM038-08	RV-07-D972		*Nymphalidae*	*Argynnis*	*paphia*	4 km west of I.L. Caragiale	Dambovita	Muntenia	Romania	270
HQ004039	EZROM789-08	RVcoll.08-M589		*Nymphalidae*	*Argynnis*	*paphia*	Gheorgheni, Valea Belchia	Harghita	Transylvania	Romania	1000
GU675685	EZSPM144-09	RVcoll.08-P656	x	*Nymphalidae*	*Argynnis*	*paphia*	Valle (Valle-Zurea), Lena		Asturias	Spain	662
GU676257	EZSPN901-09	RVcoll.08-L817	x	*Nymphalidae*	*Argynnis*	*paphia*	Puerto de la Morcuera, Rascafria		Comunidad de Madrid	Spain	1249
GU676203	EZSPN955-09	RVcoll.08-L949	x	*Nymphalidae*	*Argynnis*	*paphia*	La Quintana		Cantabria	Spain	967
KP870783	EZROM360-08	07-E600	x	*Nymphalidae*	*Argynnis*	*paphia*	Vallforners, Canoves	Barcelona	Catalonia	Spain	650
KP870599	EZROM361-08	07-E601	x	*Nymphalidae*	*Argynnis*	*paphia*	Vallforners, Canoves	Barcelona	Catalonia	Spain	650
GU676880	EZSPN476-09	RVcoll.08-H910	x	*Nymphalidae*	*Argynnis*	*paphia*	Hervas, Carretera Hervas-La Garganta	Caceres	Extremadura	Spain	1007
GU677009	EZSPN210-09	RVcoll.07-F063	x	*Nymphalidae*	*Argynnis*	*paphia*	10 km S. of Morella	Castellon	Comunidad Valenciana	Spain	900
HM901220	EZSPC1016-10	RVcoll.08-H676	x	*Nymphalidae*	*Argynnis*	*paphia*	Herreria de los Chorros, Tragacete	Cuenca	Castilla-La Mancha	Spain	1270
HM901323	EZSPC1123-10	RVcoll.08-P605	x	*Nymphalidae*	*Argynnis*	*paphia*	Piscifactoria, Laguna de Una, Una	Cuenca	Castilla-La Mancha	Spain	1219
KP871177	EZSPC241-09	RVcoll.08-P047	x	*Nymphalidae*	*Argynnis*	*paphia*	La Valira (near Urus), Cerdanya	Girona	Catalonia	Spain	1300
KP870787	EZSPC281-09	RVcoll.08-R303	x	*Nymphalidae*	*Argynnis*	*paphia*	Tapis, Macanet de Cabrenys, Alt Emporda	Girona	Catalonia	Spain	575
HM901493	EZSPC1357-10	RVcoll.09-V931	x	*Nymphalidae*	*Argynnis*	*paphia*	Sierra de la Sagra	Granada	Andalusia	Spain	1745
GU676496	EZSPN651-09	RVcoll.08-J840	x	*Nymphalidae*	*Argynnis*	*paphia*	Posada de Valdeon	Leon	Castilla y Leon	Spain	
KP870415	EZSPC260-09	RVcoll.08-P372	x	*Nymphalidae*	*Argynnis*	*paphia*	Perves, Pallars Jussa	Lleida	Catalonia	Spain	1071
KP870303	EZSPC230-09	RVcoll.08-M682	x	*Nymphalidae*	*Argynnis*	*paphia*	Farena, Alt Camp	Tarragona	Catalonia	Spain	580
JN272501	EZSPN1103-11	Rvcoll.10-A877	x	*Nymphalidae*	*Argynnis*	*paphia*	Refugi del Caro, Ports de Tortosa	Tarragona	Catalonia	Spain	1090
JX678130		07-C290		*Lycaenidae*	*Aricia*	*agestis*	Wervik		West Vlaanderen	Belgium	20
JX678128		RE-04-C216		*Lycaenidae*	*Aricia*	*agestis*	Pegsdon Hills, Hitchin		Hertfordshire	England	
JX678129		RE-04-C219		*Lycaenidae*	*Aricia*	*agestis*	Pegsdon Hills, Hitchin		Hertfordshire	England	
JX678122		MAT-99-Q929		*Lycaenidae*	*Aricia*	*agestis*	Mende		Lozère	France	780
JX678124		07-W056		*Lycaenidae*	*Aricia*	*agestis*	La Chasse du domaine des Puits de Rians, Rians		Var	France	458
JX678134		LD-386		*Lycaenidae*	*Aricia*	*agestis*	Corsica dint. Pietralba		Corsica	France	
JX678135		LD-387		*Lycaenidae*	*Aricia*	*agestis*	Corsica dint. Pietralba		Corsica	France	
GU688531	GWORO799-09	BC ZSM Lep 30491		*Lycaenidae*	*Aricia*	*agestis*	Garching-Hochbrueck MuenchenL		Bavaria	Germany	486
GU687043	GWORL191-09	BC ZSM Lep 18597		*Lycaenidae*	*Aricia*	*agestis*	Hanau, Grossauheim	Darmstadt	Hesse	Germany	110
HQ563538	FBLMW287-10	BC ZSM Lep 37388		*Lycaenidae*	*Aricia*	*agestis*	Dorngrund	Mittelfranken	Bavaria	Germany	250
HQ957267	GWORL223-09	BC ZSM Lep 18629		*Lycaenidae*	*Aricia*	*agestis*	Ehingen, Hesselberg	Mittelfranken	Bavaria	Germany	560
GU687007	GWORL231-09	BC ZSM Lep 18637		*Lycaenidae*	*Aricia*	*agestis*	Markt Nordheim, Ulsenheim, Dorngrund	Mittelfranken	Bavaria	Germany	360
GU687009	GWORL235-09	BC ZSM Lep 18641		*Lycaenidae*	*Aricia*	*agestis*	Neustadt/Aisch, Dorngrund	Mittelfranken	Bavaria	Germany	300
GU686997	GWORL251-09	BC ZSM Lep 18657		*Lycaenidae*	*Aricia*	*agestis*	Rennertshofen, Trugenhofen	Oberbayern	Bavaria	Germany	430
HQ563549	FBLMW309-10	BC ZSM Lep 37410		*Lycaenidae*	*Aricia*	*agestis*	Koednitzer Weinleite	Oberfranken	Bavaria	Germany	385
GU687000	GWORL245-09	BC ZSM Lep 18651		*Lycaenidae*	*Aricia*	*agestis*	Dietfurt/Altmuehl, Zell b. Dietfurt/Altmuehl	Oberpfalz	Bavaria	Germany	455

GenBank accession number	BOLD Process ID	Sample ID	Used in the Iberian dataset	Family	Genus	Species	Exact Site	Region	State/Province	Country	Alt. (m)
JF415674	GWORL247-09	BC ZSM Lep 18653		*Lycaenidae*	*Aricia*	*agestis*	Velburg, Velburg	Oberpfalz	Bavaria	Germany	510
GU686995	GWORL249-09	BC ZSM Lep 18655		*Lycaenidae*	*Aricia*	*agestis*	Dietfurt/Altmuehl, Zell b. Dietfurt/Altmuehl	Oberpfalz	Bavaria	Germany	455
HM903321	GWORL273-09	BC ZSM Lep 18679		*Lycaenidae*	*Aricia*	*agestis*	Regensburg, Brandlberg	Oberpfalz	Bavaria	Germany	400
JF415676	GWORL279-09	BC ZSM Lep 18685		*Lycaenidae*	*Aricia*	*agestis*	Regensburg, Brandlberg	Oberpfalz	Bavaria	Germany	380
GU687047	GWORL189-09	BC ZSM Lep 18595		*Lycaenidae*	*Aricia*	*agestis*	Mainz, Mainzer Sande	Rheinland-Pfalz	Rhineland Palatinate	Germany	130
GU686998	GWORL253-09	BC ZSM Lep 18659		*Lycaenidae*	*Aricia*	*agestis*	Monheim, Warching	Schwaben	Bavaria	Germany	480
GU686992	GWORL255-09	BC ZSM Lep 18661		*Lycaenidae*	*Aricia*	*agestis*	Monheim, Warching	Schwaben	Bavaria	Germany	480
GU686994	GWORL257-09	BC ZSM Lep 18663		*Lycaenidae*	*Aricia*	*agestis*	Noerdlingen, Holheim, NSG Ofnethoehlen	Schwaben	Bavaria	Germany	500
GU686988	GWORL259-09	BC ZSM Lep 18665		*Lycaenidae*	*Aricia*	*agestis*	Noerdlingen, Holheim, NSG Ofnethoehlen	Schwaben	Bavaria	Germany	500
HQ957269	GWORL260-09	BC ZSM Lep 18666		*Lycaenidae*	*Aricia*	*agestis*	Hoppingen, Rollenberg b. Hoppingen	Schwaben	Bavaria	Germany	460
GU686990	GWORL262-09	BC ZSM Lep 18668		*Lycaenidae*	*Aricia*	*agestis*	Hoppingen, Rollenberg b. Hoppingen	Schwaben	Bavaria	Germany	460
GU686984	GWORL263-09	BC ZSM Lep 18669		*Lycaenidae*	*Aricia*	*agestis*	Hoppingen, Rollenberg b. Hoppingen	Schwaben	Bavaria	Germany	460
GU686985	GWORL264-09	BC ZSM Lep 18670		*Lycaenidae*	*Aricia*	*agestis*	Hoppingen, Rollenberg b. Hoppingen	Schwaben	Bavaria	Germany	460
HQ957270	GWORL265-09	BC ZSM Lep 18671		*Lycaenidae*	*Aricia*	*agestis*	Hoppingen, Rollenberg b. Hoppingen	Schwaben	Bavaria	Germany	460
GU686986	GWORL266-09	BC ZSM Lep 18672		*Lycaenidae*	*Aricia*	*agestis*	Hoppingen, Rollenberg b. Hoppingen	Schwaben	Bavaria	Germany	460
GU686980	GWORL267-09	BC ZSM Lep 18673		*Lycaenidae*	*Aricia*	*agestis*	Hoppingen, Rollenberg b. Hoppingen	Schwaben	Bavaria	Germany	460
GU686981	GWORL268-09	BC ZSM Lep 18674		*Lycaenidae*	*Aricia*	*agestis*	Syrgenstein, Staufen	Schwaben	Bavaria	Germany	740
GU686982	GWORL269-09	BC ZSM Lep 18675		*Lycaenidae*	*Aricia*	*agestis*	Bissingen, Thalheim/Umg.	Schwaben	Bavaria	Germany	476
GU686983	GWORL270-09	BC ZSM Lep 18676		*Lycaenidae*	*Aricia*	*agestis*	Bissingen, Thalheim/Umg.	Schwaben	Bavaria	Germany	476
JF415675	GWORL271-09	BC ZSM Lep 18677		*Lycaenidae*	*Aricia*	*agestis*	Hoppingen, Rollenberg b. Hoppingen	Schwaben	Bavaria	Germany	460
GU687032	GWORL205-09	BC ZSM Lep 18611		*Lycaenidae*	*Aricia*	*agestis*	Ochsenfurt, Kleinochsenfurt	Unterfranken	Bavaria	Germany	225
GU687034	GWORL207-09	BC ZSM Lep 18613		*Lycaenidae*	*Aricia*	*agestis*	Ochsenfurt, Kleinochsenfurt	Unterfranken	Bavaria	Germany	225
GU687028	GWORL209-09	BC ZSM Lep 18615		*Lycaenidae*	*Aricia*	*agestis*	Karlburg, Rammersberg	Unterfranken	Bavaria	Germany	279
GU687030	GWORL211-09	BC ZSM Lep 18617		*Lycaenidae*	*Aricia*	*agestis*	Randersacker, Randersacker Sonnenberg	Unterfranken	Bavaria	Germany	265
GU687024	GWORL213-09	BC ZSM Lep 18619		*Lycaenidae*	*Aricia*	*agestis*	Randersacker, Randersacker Sonnenberg	Unterfranken	Bavaria	Germany	265
GU687026	GWORL215-09	BC ZSM Lep 18621		*Lycaenidae*	*Aricia*	*agestis*	Randersacker, Randersacker Sonnenberg	Unterfranken	Bavaria	Germany	265
GU687020	GWORL217-09	BC ZSM Lep 18623		*Lycaenidae*	*Aricia*	*agestis*	Triefenstein, Kalmuth, Homburg-Lengfurt	Unterfranken	Bavaria	Germany	230
GU687022	GWORL219-09	BC ZSM Lep 18625		*Lycaenidae*	*Aricia*	*agestis*	Triefenstein, Kalmuth, Homburg-Lengfurt	Unterfranken	Bavaria	Germany	230
GU687016	GWORL221-09	BC ZSM Lep 18627		*Lycaenidae*	*Aricia*	*agestis*	Triefenstein, Kalmuth, Homburg-Lengfurt	Unterfranken	Bavaria	Germany	230
GU687011	GWORL225-09	BC ZSM Lep 18631		*Lycaenidae*	*Aricia*	*agestis*	Roettingen, Roettingen, Weinbergsbrachen E von	Unterfranken	Bavaria	Germany	290
JF415671	GWORL227-09	BC ZSM Lep 18633		*Lycaenidae*	*Aricia*	*agestis*	Roettingen, Roettingen, Weinbergsbrachen E von	Unterfranken	Bavaria	Germany	290
JF415672	GWORL229-09	BC ZSM Lep 18635		*Lycaenidae*	*Aricia*	*agestis*	Karlburg, Rammersberg	Unterfranken	Bavaria	Germany	279
GU687008	GWORL233-09	BC ZSM Lep 18639		*Lycaenidae*	*Aricia*	*agestis*	Buch, Bucher Berg, Steinachtal	Unterfranken	Bavaria	Germany	300
GU687003	GWORL237-09	BC ZSM Lep 18643		*Lycaenidae*	*Aricia*	*agestis*	Randersacker, Randersacker Sonnenberg	Unterfranken	Bavaria	Germany	265
GU687005	GWORL239-09	BC ZSM Lep 18645		*Lycaenidae*	*Aricia*	*agestis*	Ochsenfurt, Kleinochsenfurt	Unterfranken	Bavaria	Germany	225
JF415673	GWORL241-09	BC ZSM Lep 18647		*Lycaenidae*	*Aricia*	*agestis*	Ochsenfurt, Kleinochsenfurt	Unterfranken	Bavaria	Germany	225
HQ957268	GWORL243-09	BC ZSM Lep 18649		*Lycaenidae*	*Aricia*	*agestis*	Ochsenfurt, Kleinochsenfurt	Unterfranken	Bavaria	Germany	225
JX678144		150308PP94		*Lycaenidae*	*Aricia*	*agestis*	Kamilari	Iraklio	Crete	Greece	58
JX678121		LR-08-D160		*Lycaenidae*	*Aricia*	*agestis*	Mt. Chelmos (Aroania), Kalavrita, Ski Station		Peloponessos	Greece	1700
JX678127		LR-08-D242		*Lycaenidae*	*Aricia*	*agestis*	Parnassus Mt.		Parnassus Mt.	Greece	784
JX678137		LD-621		*Lycaenidae*	*Aricia*	*agestis*	Bosco della Ficuzza	Palermo	Sicily	Italy	
JX678138		LD-622		*Lycaenidae*	*Aricia*	*agestis*	Bosco della Ficuzza	Palermo	Sicily	Italy	
JX678118		07-D876		*Lycaenidae*	*Aricia*	*agestis*	Carpineto Romano, Monti Lepini, Roma		Rome	Italy	590
JX678119		07-E047		*Lycaenidae*	*Aricia*	*agestis*	P.N. La Sila		Calabria	Italy	1230
JX678120		LD-626		*Lycaenidae*	*Aricia*	*agestis*	Esperia		Frosinone	Italy	
JX678131		07-E227		*Lycaenidae*	*Aricia*	*agestis*	Mompantero Vecchio, Susa Valley		Torino	Italy	1340
HQ004051	EZROM793-08	RVcoll.08-M519		*Lycaenidae*	*Aricia*	*agestis*	Apuseni Mts., Rimetea (Piatra Secuiului)	Alba	Transylvania	Romania	850
HQ004054	EZROM048-08	RV-07-E536		*Lycaenidae*	*Aricia*	*agestis*	3 km S of Fundata	Arges	Muntenia	Romania	1190
HQ004052	EZROM790-08	RVcoll.08-M373		*Lycaenidae*	*Aricia*	*agestis*	Buciumeni forest (Radacinesti)	Bacau	Moldavia	Romania	180
HQ004049	EZROM606-08	RV-07-E466		*Lycaenidae*	*Aricia*	*agestis*	Dumbrava Vadului, Vad	Brasov	Transylvania	Romania	495
HQ004045	EZROM042-08	RV-06-K666		*Lycaenidae*	*Aricia*	*agestis*	Cluj-Napoca dealul Bogomaia Fanatele Clujului	Cluj	Transylvania	Romania	480
HQ004046	EZROM043-08	RV-07-C131		*Lycaenidae*	*Aricia*	*agestis*	Cluj-Napoca dealul Bogomaia Fanatele Clujului	Cluj	Transylvania	Romania	480

GenBank accession number	BOLD Process ID	Sample ID	Used in the Iberian dataset	Family	Genus	Species	Exact Site	Region	State/Province	Country	Alt. (m)
HQ004047	EZROM044-08	RV-06-K652		*Lycaenidae*	*Aricia*	*agestis*	Baciu forest (Cluj-Napoca)	Cluj	Transylvania	Romania	530
HQ004050	EZROM601-08	RV-06-M830		*Lycaenidae*	*Aricia*	*agestis*	Cluj-Napoca, dealul Feleacului, Gheorgheni forest	Cluj	Transylvania	Romania	600
HQ004055	EZROM041-08	RV-07-D155		*Lycaenidae*	*Aricia*	*agestis*	Dumbraveni forest	Constanta	Dobrogea	Romania	80
HQ004048	EZROM039-08	RV-07-E399		*Lycaenidae*	*Aricia*	*agestis*	Picior de Munte	Dambovita	Muntenia	Romania	260
HQ004053	EZROM605-08	RV-07-D523		*Lycaenidae*	*Aricia*	*agestis*	Rohia	Maramures	Maramures	Romania	360
HQ004056	EZROM040-08	RV-07-E419		*Lycaenidae*	*Aricia*	*agestis*	2 km E of Greci village (Macin Mts.)	Tulcea	Dobrogea	Romania	130
JX678125		08-H854		*Lycaenidae*	*Aricia*	*agestis*	Orovac (Kosmaj)			Serbia	264
JX678132		LR-08-D714		*Lycaenidae*	*Aricia*	*agestis*	Jagnjenica		Radeče	Slovenia	260-300
JX678151	EZSPC189-09	RVcoll.06-A100	x	*Lycaenidae*	*Aricia*	*agestis*	Coll Formic, Osona	Barcelona	Catalonia	Spain	1300
JX678152	EZSPC190-09	RVcoll.06-A218	x	*Lycaenidae*	*Aricia*	*agestis*	Font de`n Vila, Matagalls, V. Oriental	Barcelona	Catalonia	Spain	1550
JX678148	EZSPC193-09	RVcoll.06-A251	x	*Lycaenidae*	*Aricia*	*agestis*	El Puig, P.N. Montseny, V. ORIENTAL	Barcelona	Catalonia	Spain	1064
JX678064		06-A220	x	*Lycaenidae*	*Aricia*	*agestis*	Can Liro, St. Pere de Vilamajor, Vallès Oriental	Barcelona	Catalonia	Spain	329
JX678145		06-A101	x	*Lycaenidae*	*Aricia*	*agestis*	Coll Formic, El Brull, Osona	Barcelona	Catalonia	Spain	1200-1400
JX678063		06-A213	x	*Lycaenidae*	*Aricia*	*agestis*	Montjoi, Roses, Alt Empordà	Girona	Catalonia	Spain	70
JX678071		06-G573	x	*Lycaenidae*	*Aricia*	*agestis*	Sta. Maria del Mont, La Garrotxa	Girona	Catalonia	Spain	1110
JX678072		08-P055	x	*Lycaenidae*	*Aricia*	*agestis*	Estoll, Fontanals de Cerdanya, Cerdanya	Girona	Catalonia	Spain	1110
JX678140		07-Z077	x	*Lycaenidae*	*Aricia*	*agestis*	Gorg de l'Olla, Campdevànol, Ripollès	Girona	Catalonia	Spain	840
JX678146		06-A222	x	*Lycaenidae*	*Aricia*	*agestis*	Darnius, Alt Empordà	Girona	Catalonia	Spain	183
JX678149		06-A201	x	*Lycaenidae*	*Aricia*	*agestis*	Closes del Tec, Alt Empordà	Girona	Catalonia	Spain	1
JX678154		LR-08-D668		*Lycaenidae*	*Aricia*	*anteros*	Kalotina		Dragoman	Bulgaria	600-700
JX678153		08-H850		*Lycaenidae*	*Aricia*	*anteros*	Debelo Brdo			Serbia	1052
JX678053		KA-472		*Lycaenidae*	*Aricia*	*artaxerxes*	Thrislington		Durham	England	
JX678054		KA-475		*Lycaenidae*	*Aricia*	*artaxerxes*	Thrislington		Durham	England	
JX678078		07-F518		*Lycaenidae*	*Aricia*	*artaxerxes*	Karilatsi mõhnastik		Polva	Estonia	
JX678105		09-X285		*Lycaenidae*	*Aricia*	*artaxerxes*	Col d'Allos, GR-56 south		Alpes-de-Haute-Provence	France	2250
JX678106		09-X296		*Lycaenidae*	*Aricia*	*artaxerxes*	Entraigues, le Vallonnet, Savoie		Savoie	France	1400
HQ563572	FBLMW335-10	BC ZSM Lep 37436		*Lycaenidae*	*Aricia*	*artaxerxes*	Arnstadt, Behringen		Thuringia	Germany	435
HQ563574	FBLMW337-10	BC ZSM Lep 37438		*Lycaenidae*	*Aricia*	*artaxerxes*	Wipfratal, Kettmannshausen		Thuringia	Germany	450
HQ563576	FBLMW339-10	BC ZSM Lep 37440		*Lycaenidae*	*Aricia*	*artaxerxes*	Ilmtal, Dienstedt		Thuringia	Germany	380
GU687036	GWORL201-09	BC ZSM Lep 18607		*Lycaenidae*	*Aricia*	*artaxerxes*	N Vordergundhoefle		Bavaria	Germany	
HQ563581	FBLMW347-10	BC ZSM Lep 37448		*Lycaenidae*	*Aricia*	*artaxerxes*	Altoberndorf, Boller Halde	Freiburg	Baden-Wuerttemberg	Germany	540
HQ563569	FBLMW332-10	BC ZSM Lep 37433		*Lycaenidae*	*Aricia*	*artaxerxes*	Berchtesgadener Alpen, Trischuebel	Oberbayern	Bavaria	Germany	2000
GU687038	GWORL203-09	BC ZSM Lep 18609		*Lycaenidae*	*Aricia*	*artaxerxes*	Aschau/Ch., Kampenwand	Oberbayern	Bavaria	Germany	1450
HQ563541	FBLMW290-10	BC ZSM Lep 37391		*Lycaenidae*	*Aricia*	*artaxerxes*	Blankenburg (Harz), Almsfeld	Sachsen-Anhalt	Saxony-Anhalt	Germany	430
JF415677	GWORL197-09	BC ZSM Lep 18603		*Lycaenidae*	*Aricia*	*artaxerxes*	Kemptner Kopf, Mindelheimer Huette	Schwaben	Bavaria	Germany	2000
GU687042	GWORL199-09	BC ZSM Lep 18605		*Lycaenidae*	*Aricia*	*artaxerxes*	Oberstdorf, Oytal	Schwaben	Bavaria	Germany	1025
JX678090		LR-08-D303		*Lycaenidae*	*Aricia*	*artaxerxes*	Timfristos Mts., Karpenissi		Timfristos Mts.	Greece	
JX678100		LR-08-D232		*Lycaenidae*	*Aricia*	*artaxerxes*	Parnassus Mt.		Parnassus Mt.	Greece	1750
KP870636	EULEP166-14	11-I597		*Lycaenidae*	*Aricia*	*artaxerxes*	Pondel village		Aosta	Italy	890
JX678055		KA-48		*Lycaenidae*	*Aricia*	*artaxerxes*	Sunndal			Norway	
JX678056		KA-51		*Lycaenidae*	*Aricia*	*artaxerxes*	Sunndal			Norway	
HQ004065	EZRMN213-08	RVcoll.08-M552		*Lycaenidae*	*Aricia*	*artaxerxes*	Valea Feregarilor - Poiana Musuroaie	Caras-Severin	Banat	Romania	800
HQ004062	EZROM045-08	RV-07-E380		*Lycaenidae*	*Aricia*	*artaxerxes*	Arjana Mountain	Caras-Severin	Banat	Romania	1455
HQ004059	EZROM791-08	RVcoll.08-M627		*Lycaenidae*	*Aricia*	*artaxerxes*	Scorota (Retezat Mts.)	Hunedoara	Transylvania	Romania	1560
HQ004058	EZROM792-08	RVcoll.08-M628		*Lycaenidae*	*Aricia*	*artaxerxes*	Scorota (Retezat Mts.)	Hunedoara	Transylvania	Romania	1560
HQ004063	EZROM046-08	RV-07-E580		*Lycaenidae*	*Aricia*	*artaxerxes*	Jepii Mici (Bucegi Mts.)	Prahova	Muntenia	Romania	1800
HQ004064	EZROM047-08	RV-07-E582		*Lycaenidae*	*Aricia*	*artaxerxes*	Jepii Mici (Bucegi Mts.)	Prahova	Muntenia	Romania	1800
HQ004060	EZROM049-08	RV-07-E576		*Lycaenidae*	*Aricia*	*artaxerxes*	Jepii Mici (Bucegi Mts.)	Prahova	Muntenia	Romania	1800
HQ004061	EZROM050-08	RV-07-C201		*Lycaenidae*	*Aricia*	*artaxerxes*	towards Varful Ciucas (Ciucas Mts.)	Prahova	Muntenia	Romania	1760
HQ004057	EZROM602-08	RV-07-E579		*Lycaenidae*	*Aricia*	*artaxerxes*	Jepii Mici (Bucegi Mts.)	Prahova	Muntenia	Romania	1800
JX678083		AD-00-P228		*Lycaenidae*	*Aricia*	*artaxerxes*	Kamyshinsky		Volgograd	Russia	
JX678085		NK-00-P868		*Lycaenidae*	*Aricia*	*artaxerxes*	Karabi Yaila		Crimea	Russia	
JX678091		AD-00-P194		*Lycaenidae*	*Aricia*	*artaxerxes*	Tatinki		Tula	Russia	120

GenBank accession number	BOLD Process ID	Sample ID	Used in the Iberian dataset	Family	Genus	Species	Exact Site	Region	State/Province	Country	Alt. (m)
JX678052		KA-216		*Lycaenidae*	*Aricia*	*artaxerxes*	Coupar Angus			Scotland	
JX678058		KA-198		*Lycaenidae*	*Aricia*	*artaxerxes*	Coupar Angus			Scotland	
JX678089		08-H866		*Lycaenidae*	*Aricia*	*artaxerxes*	Debelo Brdo			Serbia	
JX678080		08-H813		*Lycaenidae*	*Aricia*	*artaxerxes*	Duvberg		Härjedalen	Sweden	
JX678081		08-H821		*Lycaenidae*	*Aricia*	*artaxerxes*	Skutskär		Uppland	Sweden	
JX678082		08-L317		*Lycaenidae*	*Aricia*	*artaxerxes*	Örnsköldsvik, Angermanland		Angermanland	Sweden	
JX678087		08-H827		*Lycaenidae*	*Aricia*	*artaxerxes*	Närsholm		Gotland	Sweden	
JX678088		08-H840		*Lycaenidae*	*Aricia*	*artaxerxes*	Skoge		Gotland	Sweden	
JX678036		LD-415		*Lycaenidae*	*Aricia*	*cramera*	Monte Albo, Nuoro		Sardinia	Italy	
JX678040		LD-365		*Lycaenidae*	*Aricia*	*cramera*	Sardegna Gennargentu dint. Broncu Spina		Sardinia	Italy	
JX678041		LD-366		*Lycaenidae*	*Aricia*	*cramera*	Sardegna Gennargentu dint. Broncu Spina		Sardinia	Italy	
KM459142	EZSPM667-12	RVcoll.12-L547	x	*Lycaenidae*	*Aricia*	*cramera*	Porches, Lagoa		Algarve	Portugal	
KM517850	EZSPM668-12	RVcoll.12-L566	x	*Lycaenidae*	*Aricia*	*cramera*	Espiche, Lagos		Algarve	Portugal	
KM459144	EZSPM891-12	Rvcoll.12-M610	x	*Lycaenidae*	*Aricia*	*cramera*	Medellin	Badajoz	Extremadura	Spain	240
KM517860	EZSPM892-12	Rvcoll.12-M611	x	*Lycaenidae*	*Aricia*	*cramera*	Medellin	Badajoz	Extremadura	Spain	240
JX678027	EZROM707-08	RV-06-A215	x	*Lycaenidae*	*Aricia*	*cramera*	Vall d`Horta, Valles Occidental	Barcelona	Catalonia	Spain	400
JX678023		06-A200	x	*Lycaenidae*	*Aricia*	*cramera*	Montseny d'Amunt, Montseny, Vallès Oriental	Barcelona	Catalonia	Spain	1100
JX678025		06-A208	x	*Lycaenidae*	*Aricia*	*cramera*	Vallforners, Cànoves, Vallès Oriental	Barcelona	Catalonia	Spain	650
JX678030		08-L330	x	*Lycaenidae*	*Aricia*	*cramera*	Montseny d'Amunt, Montseny, Vallès Oriental	Barcelona	Catalonia	Spain	1100
JX678031		08-L331	x	*Lycaenidae*	*Aricia*	*cramera*	Montseny d'Amunt, Montseny, Vallès Oriental	Barcelona	Catalonia	Spain	1100
JX678044		06-A238	x	*Lycaenidae*	*Aricia*	*cramera*	Can Ferriol, Sant Feliu de Llobregat, Baix Llobregat	Barcelona	Catalonia	Spain	223
JX678048		06-G664	x	*Lycaenidae*	*Aricia*	*cramera*	El Burguès, Coll Formic, Montseny	Barcelona	Catalonia	Spain	1010
JX678049		06-A149	x	*Lycaenidae*	*Aricia*	*cramera*	Els Hostalets de Balenyà (Osona)	Barcelona	Catalonia	Spain	575
JX678050		06-A214	x	*Lycaenidae*	*Aricia*	*cramera*	Vall d'Horta, Sant Llorenç Savall	Barcelona	Catalonia	Spain	380
KM459146	EZSPM1022-12	Rvcoll.12-M771	x	*Lycaenidae*	*Aricia*	*cramera*	7 km SE Navalvillar de Ibor	Caceres	Extremadura	Spain	729
KM517854	EZSPM1025-12	Rvcoll.12-M775	x	*Lycaenidae*	*Aricia*	*cramera*	Hospital del Obispo, Navalvillar de Ibor	Caceres	Extremadura	Spain	1010
KM517848	EZSPM1036-12	Rvcoll.12-M787	x	*Lycaenidae*	*Aricia*	*cramera*	Carrascalejo	Caceres	Extremadura	Spain	636
HM901563	EZSPC706-10	RVcoll.08-H635	x	*Lycaenidae*	*Aricia*	*cramera*	El Gastor	Cadiz	Andalusia	Spain	400
JX678039		07-F082	x	*Lycaenidae*	*Aricia*	*cramera*	4 km E. of Olocau del Rey	Castellón	Comunidad Valenciana	Spain	1005
KM459145	EZSPM912-12	Rvcoll.12-M634	x	*Lycaenidae*	*Aricia*	*cramera*	Lagunas de Ruidera, Ossa de Montiel	Ciudad Real	Castilla-La Mancha	Spain	795
KM517863	EZSPM943-12	Rvcoll.12-M678	x	*Lycaenidae*	*Aricia*	*cramera*	Rio Jandula, Mestanza	Ciudad Real	Castilla-La Mancha	Spain	407
KM517853	EZSPM967-12	Rvcoll.12-M719	x	*Lycaenidae*	*Aricia*	*cramera*	Alrededores San Lorenzo de Calatrava	Ciudad Real	Castilla-La Mancha	Spain	749
HM901611	EZSPC756-10	RVcoll.06-G526	x	*Lycaenidae*	*Aricia*	*cramera*	Meranges, La Cerdanya	Girona	Catalonia	Spain	1744
JX678032		08-P054	x	*Lycaenidae*	*Aricia*	*cramera*	Estoll, Fontanals de Cerdanya, Cerdanya	Girona	Catalonia	Spain	1110
HM901562	EZSPC705-10	RVcoll.09-V439	x	*Lycaenidae*	*Aricia*	*cramera*	Loma de Lastonares (Sierra Nevada)	Granada	Andalusia	Spain	2050
JX678150		06-A012	x	*Lycaenidae*	*Aricia*	*cramera*	Mecina Bombarón, Alpujarras	Granada	Andalusia	Spain	1150
KM459120	EULEP147-14	10-C442	x	*Lycaenidae*	*Aricia*	*cramera*	San Rafael	Ibiza	Balearic Islands	Spain	
GU676447	EZSPN703-09	RVcoll.08-J884	x	*Lycaenidae*	*Aricia*	*cramera*	El Burgo Ranero	Leon	Castilla y Leon	Spain	
HM901608	EZSPC753-10	RVcoll.06-A205	x	*Lycaenidae*	*Aricia*	*cramera*	Tremp, P. JUSSA	Lleida	Catalonia	Spain	660
JX678024		06-A206	x	*Lycaenidae*	*Aricia*	*cramera*	Tremp, Pallars Jussà	Lleida	Catalonia	Spain	660
JX678034		08-P272	x	*Lycaenidae*	*Aricia*	*cramera*	S'Albufera	Mallorca	Balearic Islands	Spain	3.5
JX678035		08-P254	x	*Lycaenidae*	*Aricia*	*cramera*	Santa Maria, Mallorca	Mallorca	Balearic Islands	Spain	181.3
JX678026		06-A209	x	*Lycaenidae*	*Aricia*	*cramera*	Llimpa, Maó	Menorca	Balearic Islands	Spain	
JX678033		06-A210	x	*Lycaenidae*	*Aricia*	*cramera*	Llimpa, Maó, Menorca	Menorca	Balearic Islands	Spain	
JX678028		06-A241	x	*Lycaenidae*	*Aricia*	*cramera*	La Morera de Montsant, Priorat	Tarragona	Catalonia	Spain	750
JX678043		08-M690	x	*Lycaenidae*	*Aricia*	*cramera*	Farena, Alt Camp	Tarragona	Catalonia	Spain	580
KM517855	EZSPM899-12	Rvcoll.12-M621	x	*Lycaenidae*	*Aricia*	*cramera*	Tudela de Duero	Valladolid	Castilla y Leon	Spain	713
JX678045		06-A253	x	*Lycaenidae*	*Aricia*	*cramera*	Rivas-Vaciamadrid		Comunidad de Madrid	Spain	
JX678059		06-A234	x	*Lycaenidae*	*Aricia*	*montensis*	Pic dels Pessons, Encamp		Andorra	Andorra	1400
JX678060		06-A235	x	*Lycaenidae*	*Aricia*	*montensis*	Comapedrosa			Andorra	1800
JX678061		06-A239	x	*Lycaenidae*	*Aricia*	*montensis*	Sorteny			Andorra	1800

GenBank accession number	BOLD Process ID	Sample ID	Used in the Iberian dataset	Family	Genus	Species	Exact Site	Region	State/Province	Country	Alt. (m)
HM901632	EZSPC777-10	RVcoll.08-L842	x	*Lycaenidae*	*Aricia*	*montensis*	Pinilla del Valle		Comunidad de Madrid	Spain	1100
KP870720	EZSPM773-12	RVcoll.050111LP38	x	*Lycaenidae*	*Aricia*	*montensis*	PR-23 de Fuente De, Camaleno		Asturias	Spain	1093
HM901567	EZSPC710-10	RVcoll.08-R145	x	*Lycaenidae*	*Aricia*	*montensis*	Turo de L`Home, Montseny	Barcelona	Catalonia	Spain	1643.5
HM901604	EZSPC749-10	RVcoll.08-L117	x	*Lycaenidae*	*Aricia*	*montensis*	Puerto de las Cabrillas, Portell de Morella	Castellon	Comunidad Valenciana	Spain	1301
JX678066		08-P608	x	*Lycaenidae*	*Aricia*	*montensis*	Mogorrita, Tragacete	Cuenca	Castilla-La Mancha	Spain	1748
KP870448	EULEP119-14	09-X375	x	*Lycaenidae*	*Aricia*	*montensis*	Sierra de la Sagra	Granada	Andalusia	Spain	1670-1820
JF848012	EZSPN075-09	RVcoll.09-V919	x	*Lycaenidae*	*Aricia*	*montensis*	Sierra de la Sagra	Granada	Andalusia	Spain	1750
JF848015	EZSPN078-09	RVcoll.09-V560	x	*Lycaenidae*	*Aricia*	*montensis*	Collado de las Sabinas, Sierra Nevada	Granada	Andalusia	Spain	2175
HM901641	EZSPC788-10	RVcoll.08-M042	x	*Lycaenidae*	*Aricia*	*montensis*	Gualda, La Alcarria	Guadalajara	Castilla-La Mancha	Spain	730
HM901551	EZSPC692-10	RVcoll.06-A247	x	*Lycaenidae*	*Aricia*	*montensis*	Val de Barbarisa	Huesca	Aragon	Spain	1800
JX678062		06-A249	x	*Lycaenidae*	*Aricia*	*montensis*	Cañon de Añisclo	Huesca	Aragón	Spain	900
JX678165		06-A246	x	*Lycaenidae*	*Aricia*	*montensis*	Puerto de la Pez	Huesca	Aragón	Spain	1800
GU676495	EZSPN658-09	RVcoll.08-J843	x	*Lycaenidae*	*Aricia*	*montensis*	Posada de Valdeon	Leon	Castilla y Leon	Spain	
JX678147	EZSPC191-09	RVcoll.06-A219	x	*Lycaenidae*	*Aricia*	*montensis*	Espot-St. Maurici, P. SOBIRA	Lleida	Catalonia	Spain	1700
JX678051	EZSPC192-09	RVcoll.06-A233	x	*Lycaenidae*	*Aricia*	*montensis*	St. Maurici, P. SOBIRA	Lleida	Catalonia	Spain	1700
JX678070	EZSPC262-09	RVcoll.08-P398	x	*Lycaenidae*	*Aricia*	*montensis*	Salt de Pitx, Vall d`Aran	Lleida	Catalonia	Spain	1570
JX678069	EZSPC272-09	RVcoll.08-R208	x	*Lycaenidae*	*Aricia*	*montensis*	Port de la Bonaigua, Pallars Sobira	Lleida	Catalonia	Spain	2338
KP870528	EULEP017-14	07-C688	x	*Lycaenidae*	*Aricia*	*montensis*	Serra de la Gritella, Prades, Baix Camp	Tarragona	Catalonia	Spain	1100
KP870555	EULEP018-14	07-C689	x	*Lycaenidae*	*Aricia*	*montensis*	Serra de la Gritella, Prades, Baix Camp	Tarragona	Catalonia	Spain	1100
KP870261	EULEP019-14	07-C692	x	*Lycaenidae*	*Aricia*	*montensis*	Serra de la Gritella, Prades, Baix Camp	Tarragona	Catalonia	Spain	1100
HM901676	EZSPC829-10	RVcoll.08-P927	x	*Lycaenidae*	*Aricia*	*montensis*	Ayora, La Hunde	Valencia	Comunidad Valenciana	Spain	
JX678065		07-W250	x	*Lycaenidae*	*Aricia*	*montensis*	Saliencia		Asturias	Spain	1100
JN276878	EZSPC1265-10	RVcoll.070207MC36	x	*Lycaenidae*	*Aricia*	*morronensis*	Acceso Aitana 2,Confrides	Alicante	Comunidad Valenciana	Spain	1402
JN276882	EZSPC1276-10	RVcoll.171106RP5	x	*Lycaenidae*	*Aricia*	*morronensis*	Acceso Aitana 1,Confrides	Alicante	Comunidad Valenciana	Spain	1342
HM901473	EZSPC1301-10	RVcoll.171106RP4	x	*Lycaenidae*	*Aricia*	*morronensis*	Acceso Aitana 1,Confrides	Alicante	Comunidad Valenciana	Spain	1342
HM901601	EZSPC746-10	RVcoll.08-H973	x	*Lycaenidae*	*Aricia*	*morronensis*	Hoyocasero, Venta Rasquilla	Avila	Castilla y Leon	Spain	1224
GU676916	EZSPN513-09	RVcoll.08-H974	x	*Lycaenidae*	*Aricia*	*morronensis*	Hoyocasero, Venta Rasquilla	Avila	Castilla y Leon	Spain	1224
GU676361	EZSPN785-09	RVcoll.08-L328	x	*Lycaenidae*	*Aricia*	*morronensis*	Serrat dels Tossals, Bergueda	Barcelona	Catalonia	Spain	1500
JN276885	EZSPC1315-10	RVcoll.130209KL8	x	*Lycaenidae*	*Aricia*	*morronensis*	Penyagolosa,Vistabella del Maestrazgo	Castellon	Comunidad Valenciana	Spain	
JF848013	EZSPN076-09	RVcoll.09-V516	x	*Lycaenidae*	*Aricia*	*morronensis*	road to Veleta, Sierra Nevada	Granada	Andalusia	Spain	2640
JF848014	EZSPN077-09	RVcoll.09-V556	x	*Lycaenidae*	*Aricia*	*morronensis*	Collado de las Sabinas, Sierra Nevada	Granada	Andalusia	Spain	2175
GU676467	EZSPN676-09	RVcoll.08-J988	x	*Lycaenidae*	*Aricia*	*morronensis*	Acceso Veleta, Monachil	Granada	Andalusia	Spain	2737
JX678017		08-J956	x	*Lycaenidae*	*Aricia*	*morronensis*	Acceso Veleta, Monachil	Granada	Andalusia	Spain	2737
JX678013		07-W251	x	*Lycaenidae*	*Aricia*	*morronensis*	Torrestío	León	Castilla y León	Spain	
HM901568	EZSPC712-10	RVcoll.09-X040	x	*Lycaenidae*	*Aricia*	*morronensis*	Pic del Moixero, La Cerdanya	Lleida	Catalonia	Spain	2000
HM901572	EZSPC717-10	RVcoll.09-X041	x	*Lycaenidae*	*Aricia*	*morronensis*	Pic del Moixero, La Cerdanya	Lleida	Catalonia	Spain	2000
HM901606	EZSPC751-10	RVcoll.08-L889	x	*Lycaenidae*	*Aricia*	*morronensis*	Abejar	Soria	Castilla y Leon	Spain	1111
GU676229	EZSPN931-09	RVcoll.08-L887	x	*Lycaenidae*	*Aricia*	*morronensis*	Abejar	Soria	Castilla y Leon	Spain	1111
JX678014		08-P669	x	*Lycaenidae*	*Aricia*	*morronensis*	Gamoniteiru, Sierra de Aramo, Quirós		Asturias	Spain	1712
HM871928	LEFIC059-10	MM03341		*Lycaenidae*	*Aricia*	*nicias*	Kiiminki	Ostrobottnia borealis pars australis	Northern Ostrobothnia	Finland	
HM873047	LEFID248-10	MM06112		*Lycaenidae*	*Aricia*	*nicias*	Tornio	Ostrobottnia borealis pars borealis		Finland	
HM401003	EZSPM551-10	RVcoll.09-V884	x	*Lycaenidae*	*Aricia*	*nicias*	Banys de Tredos, Vall d`Aran	Lleida	Catalonia	Spain	
HM401004	EZSPM552-10	RVcoll.09-V885	x	*Lycaenidae*	*Aricia*	*nicias*	Banys de Tredos, Vall d`Aran	Lleida	Catalonia	Spain	
HM401005	EZSPM553-10	RVcoll.09-V886	x	*Lycaenidae*	*Aricia*	*nicias*	Banys de Tredos, Vall d`Aran	Lleida	Catalonia	Spain	
HM401006	EZSPM554-10	RVcoll.09-V887	x	*Lycaenidae*	*Aricia*	*nicias*	Banys de Tredos, Vall d`Aran	Lleida	Catalonia	Spain	
HM401007	EZSPM555-10	RVcoll.09-V889	x	*Lycaenidae*	*Aricia*	*nicias*	Banys de Tredos, Vall d`Aran	Lleida	Catalonia	Spain	

GenBank accession number	BOLD Process ID	Sample ID	Used in the Iberian dataset	Family	Genus	Species	Exact Site	Region	State/Province	Country	Alt. (m)
HM401008	EZSPM556-10	RVcoll.09-V890	x	*Lycaenidae*	*Aricia*	*nicias*	Banys de Tredos, Vall d`Aran	Lleida	Catalonia	Spain	
KP870773	EZSPM641-12	RVcoll.11-H244	x	*Lycaenidae*	*Aricia*	*nicias*	Banhs de Tredos, Vall d`Aran	Lleida	Catalonia	Spain	1875
KP870378	EZSPM642-12	RVcoll.11-H245	x	*Lycaenidae*	*Aricia*	*nicias*	Banhs de Tredos, Vall d`Aran	Lleida	Catalonia	Spain	1875
KP870982	EZSPM643-12	RVcoll.11-H246	x	*Lycaenidae*	*Aricia*	*nicias*	Banhs de Tredos, Vall d`Aran	Lleida	Catalonia	Spain	1875
JX678099		08-H801		*Lycaenidae*	*Aricia*	*nicias*	Skutskär		Uppland	Sweden	
HQ968283	PHLAB1019-10	TLMF Lep 01819		*Lycaenidae*	*Aricia*	*nicias*	Val Bever, Alp Suvretta		Graubunden	Switzerland	2100
HQ968284	PHLAB1020-10	TLMF Lep 01820		*Lycaenidae*	*Aricia*	*nicias*	Val Bever, Alp Suvretta		Graubunden	Switzerland	2100
HQ968285	PHLAB1021-10	TLMF Lep 01821		*Lycaenidae*	*Aricia*	*nicias*	Val Bever, Alp Suvretta		Graubunden	Switzerland	2100
JF860269	PHLAC716-10	TLMF Lep 02751		*Lycaenidae*	*Aricia*	*nicias*	V. Laschadura/ Zernez		Graubunden	Switzerland	2100
KP871115	EULEP030-14	07-D788	x	*Lycaenidae*	*Azanus*	*jesous*	Algeciras	Cádiz	Andalusia	Spain	
HQ565469	GWOSA737-10	BC ZSM Lep 35748		*Nymphalidae*	*Boloria*	*aquilonaris*	NSG Todtenau, Dornerau	Niederbayern	Bavaria	Germany	
HM391820	FBLMU469-09	BC ZSM Lep 27119		*Nymphalidae*	*Boloria*	*aquilonaris*	Ruhpolding, NSG Roethelmoos	Oberbayern	Bavaria	Germany	850
GU686888	GWORL401-09	BC ZSM Lep 22303		*Nymphalidae*	*Boloria*	*aquilonaris*	Egerndacher Filz	Oberbayern	Bavaria	Germany	570
JF415679	GWORR674-10	BC ZSM Lep 32170		*Nymphalidae*	*Boloria*	*aquilonaris*	Zellwies, Koenigsdorf	Oberbayern	Bavaria	Germany	600
HQ004066	EZROM794-08	RVcoll.08-M523		*Nymphalidae*	*Boloria*	*aquilonaris*	Molhasurile de la Izbuce, Apuseni Mts.	Bihor	Transylvania	Romania	1200
HQ004067	EZROM795-08	RVcoll.08-M524		*Nymphalidae*	*Boloria*	*aquilonaris*	Molhasurile de la Izbuce, Apuseni Mts.	Bihor	Transylvania	Romania	1200
HQ004068	EZROM796-08	RVcoll.08-M525		*Nymphalidae*	*Boloria*	*aquilonaris*	Molhasurile de la Izbuce, Apuseni Mts.	Bihor	Transylvania	Romania	1200
HQ004069	EZROM797-08	RVcoll.08-M526		*Nymphalidae*	*Boloria*	*aquilonaris*	Molhasurile de la Izbuce, Apuseni Mts.	Bihor	Transylvania	Romania	1200
HQ004070	EZROM798-08	RVcoll.08-M527		*Nymphalidae*	*Boloria*	*aquilonaris*	Molhasurile de la Izbuce, Apuseni Mts.	Bihor	Transylvania	Romania	1200
JF415680	GWORR671-10	BC ZSM Lep 32167		*Nymphalidae*	*Boloria*	*dia*	Garching-Hochbrueck	Oberbayern	Bavaria	Germany	487
GU707295	FBLMV675-09	BC ZSM Lep 28655		*Nymphalidae*	*Boloria*	*dia*	Regenstauf, Kreuth	Oberpfalz	Bavaria	Germany	510
HQ004072	EZROM850-08	RVcoll.06-N012		*Nymphalidae*	*Boloria*	*dia*	Apuseni Mts., Rimetea (Piatra Secuiului)	Alba	Transylvania	Romania	850
HQ004076	EZROM093-08	RV-06-M957		*Nymphalidae*	*Boloria*	*dia*	Dealul Istrita, Breaza	Buzau	Muntenia	Romania	550
HQ004077	EZROM094-08	RV-07-C954		*Nymphalidae*	*Boloria*	*dia*	Baile Herculane (Pecinisca)	Caras-Severin	Banat	Romania	220
HQ004074	EZROM091-08	RV-06-K536		*Nymphalidae*	*Boloria*	*dia*	Badeni	Cluj	Transylvania	Romania	440
HQ004075	EZROM092-08	RV-06-K538		*Nymphalidae*	*Boloria*	*dia*	Badeni	Cluj	Transylvania	Romania	440
HQ004073	EZROM851-08	RVcoll.07-C423		*Nymphalidae*	*Boloria*	*dia*	Valea Mare	Dambovita	Muntenia	Romania	225
HQ004081	EZROM855-08	RVcoll.07-D922		*Nymphalidae*	*Boloria*	*dia*	Pestera Muierii, Baia de Fier	Gorj	Oltenia	Romania	640
KP870230	EULEP135-14	10-C381		*Nymphalidae*	*Boloria*	*dia*	Filpea-Gălăuţaş	Harghita	Transylvania	Romania	
HQ004071	EZROM849-08	RVcoll.08-M354		*Nymphalidae*	*Boloria*	*dia*	Valea Belchia (Gheorgheni)	Harghita	Transylvania	Romania	1010
HQ004080	EZROM853-08	RVcoll.07-D562		*Nymphalidae*	*Boloria*	*dia*	Spini	Hunedoara	Transylvania	Romania	240
HQ004079	EZROM852-08	RVcoll.07-D504		*Nymphalidae*	*Boloria*	*dia*	Cheile Babei	Maramures	Maramures	Romania	265
HQ004078	EZROM854-08	RVcoll.07-D909		*Nymphalidae*	*Boloria*	*dia*	Otasau Valley, Buila-Vanturarita	Valcea	Oltenia	Romania	515
KP870402	EZROM709-08	RV-06-H866	x	*Nymphalidae*	*Boloria*	*dia*	Vallforners, Canoves	Barcelona	Catalonia	Spain	650
KP870413	EZSPC196-09	RVcoll.06-G467	x	*Nymphalidae*	*Boloria*	*dia*	Font Pomereta, El Brull, Osona	Barcelona	Catalonia	Spain	1210
KP870801	EZSPC249-09	RVcoll.08-P223	x	*Nymphalidae*	*Boloria*	*dia*	La Molina	Barcelona	Catalonia	Spain	1622
KP870457	EZSPM795-12	RVcoll.070611MH85	x	*Nymphalidae*	*Boloria*	*dia*	El Pueyo de Jaca, Panticosa	Huesca	Aragon	Spain	1779
HM901675	EZSPC827-10	RVcoll.08-P776	x	*Nymphalidae*	*Boloria*	*dia*	Besande	Leon	Castilla y Leon	Spain	1237
GU676364	EZSPN789-09	RVcoll.08-J889	x	*Nymphalidae*	*Boloria*	*dia*	Aviados	Leon	Castilla y Leon	Spain	
KP870558	EZSPC218-09	RVcoll.08-L416	x	*Nymphalidae*	*Boloria*	*dia*	Rius de Cerdanya	Lleida	Catalonia	Spain	1273
KP870466	EZSPC253-09	RVcoll.08-P334	x	*Nymphalidae*	*Boloria*	*dia*	Perves, Pallars Jussa	Lleida	Catalonia	Spain	1071
HM901633	EZSPC778-10	RVcoll.08-L853	x	*Nymphalidae*	*Boloria*	*dia*	Villaverde del Monte	Soria	Castilla y Leon	Spain	1100
GU676245	EZSPN914-09	RVcoll.08-L854	x	*Nymphalidae*	*Boloria*	*dia*	Villaverde del Monte	Soria	Castilla y Leon	Spain	1100
GU676237	EZSPN923-09	RVcoll.08-L870	x	*Nymphalidae*	*Boloria*	*dia*	Abejar	Soria	Castilla y Leon	Spain	1097
GU676336	EZSPN810-09	RVcoll.08-L505	x	*Nymphalidae*	*Boloria*	*eunomia*	Refugi de Riu dels Orris, per sota del (vall del Madriu), Escaldes-Engordany			Andorra	2180
HM875823	LEFIG143-10	MM13982		*Nymphalidae*	*Boloria*	*eunomia*	Kempele	Ostrobottnia borealis pars australis		Finland	
HM875824	LEFIG144-10	MM13983		*Nymphalidae*	*Boloria*	*eunomia*	Kempele	Ostrobottnia borealis pars australis		Finland	
JF853660	LEFIJ544-10	MM17169		*Nymphalidae*	*Boloria*	*eunomia*	Kiiminki	Ostrobottnia borealis pars australis		Finland	
GU655024	FBLMT910-09	BC ZSM Lep 25470		*Nymphalidae*	*Boloria*	*eunomia*	Diessen am Ammersee, Vilgertshofener Forst	Oberbayern	Bavaria	Germany	708

GenBank accession number	BOLD Process ID	Sample ID	Used in the Iberian dataset	Family	Genus	Species	Exact Site	Region	State/Province	Country	Alt. (m)
JF415682	GWORK308-09	BC ZSM Lep 21543		*Nymphalidae*	*Boloria*	*eunomia*	Koenigsdorf, Weitfilz	Oberbayern	Bavaria	Germany	600
JF415681	GWORR675-10	BC ZSM Lep 32171		*Nymphalidae*	*Boloria*	*eunomia*	Zellwies	Oberbayern	Bavaria	Germany	600
JX034677	ODOPE249-11	BC ZSM Lep 53120		*Nymphalidae*	*Boloria*	*eunomia*	Lamerdingen, Kleinkitzighofen	Schwaben, Ostallgaeu	Bavaria	Germany	600
JX034619	ODOPE250-11	BC ZSM Lep 53121		*Nymphalidae*	*Boloria*	*eunomia*	Lamerdingen, Kleinkitzighofen	Schwaben, Ostallgaeu	Bavaria	Germany	600
FJ664012	LOWA758-06	2005-LOWA-758		*Nymphalidae*	*Boloria*	*eunomia*	Tamengont	Oranienbaum distr.	St. Petersburg	Russia	
FJ664014	LOWA292-06	2005-LOWA-292		*Nymphalidae*	*Boloria*	*eunomia*	Osyno	Sebezh distr.	Pskov	Russia	
FJ664013	LOWA293-06	2005-LOWA-293		*Nymphalidae*	*Boloria*	*eunomia*	Osyno	Sebezh distr.	Pskov	Russia	
GU676265	EZSPN891-09	RVcoll.08-L802	x	*Nymphalidae*	*Boloria*	*eunomia*	Brana Vieja		Cantabria	Spain	1408
GU676266	EZSPN892-09	RVcoll.08-L803	x	*Nymphalidae*	*Boloria*	*eunomia*	Brana Vieja		Cantabria	Spain	1408
GU676267	EZSPN893-09	RVcoll.08-L804	x	*Nymphalidae*	*Boloria*	*eunomia*	Brana Vieja		Cantabria	Spain	1408
GU676268	EZSPN894-09	RVcoll.08-L805	x	*Nymphalidae*	*Boloria*	*eunomia*	Brana Vieja		Cantabria	Spain	1408
HM901612	EZSPC757-10	RVcoll.09-V672	x	*Nymphalidae*	*Boloria*	*eunomia*	Meranges	Girona	Catalonia	Spain	1555
HM901618	EZSPC763-10	RVcoll.07-C632.1	x	*Nymphalidae*	*Boloria*	*eunomia*	Meranges	Girona	Catalonia	Spain	1555
GU677019	EZSPN198-09	RVcoll.07-C679	x	*Nymphalidae*	*Boloria*	*eunomia*	Meranges	Girona	Catalonia	Spain	1555
GU669634	EZSPC646-09	RVcoll.07-C025	x	*Nymphalidae*	*Boloria*	*euphrosyne*	Puigmal	Languedoc-Roussillon		France	2050
GU669635	EZSPC647-09	RVcoll.07-C026	x	*Nymphalidae*	*Boloria*	*euphrosyne*	Puigmal	Languedoc-Roussillon		France	2050
GU669630	EZSPC652-09	RVcoll.07-C646	x	*Nymphalidae*	*Boloria*	*euphrosyne*	Col de Puymorens	Languedoc-Roussillon		France	1825
JN272497	EZSPN1072-11	Rvcoll.09-V303	x	*Nymphalidae*	*Boloria*	*euphrosyne*	Porte Puymorens	Languedoc-Roussillon		France	1825
JF415684	GWORR669-10	BC ZSM Lep 32165		*Nymphalidae*	*Boloria*	*euphrosyne*	Isarauen, Fischerhaeuser	Oberbayern	Bavaria	Germany	482
JF415683	GWORR670-10	BC ZSM Lep 32166		*Nymphalidae*	*Boloria*	*euphrosyne*	Starnberg, Machtlfing	Oberbayern	Bavaria	Germany	715
KP870400	PHLAC362-10	TLMF Lep 02397		*Nymphalidae*	*Boloria*	*euphrosyne*	Ritten/ Obergruenwald		South Tyrol	Italy	1750
KP870661	EULEP086-14	08-P972		*Nymphalidae*	*Boloria*	*euphrosyne*	M-ţii Trascăului, Rimetea, Piatra Secuiului	Alba	Transylvania	Romania	
KP870533	EULEP044-14	08-H024		*Nymphalidae*	*Boloria*	*euphrosyne*	Corongiş (M-ţii. Rodnei)	Bistrita-Nasaud	Transylvania	Romania	1500
HQ004086	EZROM857-08	RVcoll.07-D314		*Nymphalidae*	*Boloria*	*euphrosyne*	Dumbrava Vadului, Vad	Brasov	Transylvania	Romania	495
HQ004085	EZROM095-08	RV-07-D221		*Nymphalidae*	*Boloria*	*euphrosyne*	Dealul Istrita, Breaza	Buzau	Muntenia	Romania	550
HQ004084	EZROM096-08	RV-07-D234		*Nymphalidae*	*Boloria*	*euphrosyne*	Dealul Istrita, Breaza	Buzau	Muntenia	Romania	550
HQ004083	EZROM301-08	RV-07-E663		*Nymphalidae*	*Boloria*	*euphrosyne*	Cluj-Napoca, dealul Feleacului, Gheorgheni forest	Cluj	Transylvania	Romania	600
HQ004082	EZROM614-08	RV-06-N006		*Nymphalidae*	*Boloria*	*euphrosyne*	Gheorgheni, Valea Belchia	Harghita	Transylvania	Romania	1010
HQ004089	EZROM856-08	RVcoll.08-M343		*Nymphalidae*	*Boloria*	*euphrosyne*	1 Km E of Gheorgheni	Harghita	Transylvania	Romania	900
HQ004087	EZROM859-08	RVcoll.08-M645		*Nymphalidae*	*Boloria*	*euphrosyne*	Scorota (Retezat Mts.)	Hunedoara	Transylvania	Romania	1560
HQ004088	EZROM858-08	RVcoll.07-D486		*Nymphalidae*	*Boloria*	*euphrosyne*	Cheile Babei	Maramures	Maramures	Romania	265
HM901719	EZSPC879-10	RVcoll.09-T137	x	*Nymphalidae*	*Boloria*	*euphrosyne*	San Andres de Parana, Lena		Asturias	Spain	765
HM901747	EZSPC909-10	RVcoll.09-V790	x	*Nymphalidae*	*Boloria*	*euphrosyne*	Valle del Tena, Camino embalse Respomuso, Sallent de Gallego	Huesca	Aragon	Spain	1650
GU676966	EZSPN258-09	RVcoll.07-W162	x	*Nymphalidae*	*Boloria*	*euphrosyne*	Vallibierna	Huesca	Aragon	Spain	1800
HM901720	EZSPC880-10	RVcoll.09-T138	x	*Nymphalidae*	*Boloria*	*euphrosyne*	Cercanias de Aralla de Luna	Leon	Castilla y Leon	Spain	1178
KP870241	EZSPC225-09	RVcoll.08-L492	x	*Nymphalidae*	*Boloria*	*euphrosyne*	Bassa d`Arres, Vall d`Aran	Lleida	Catalonia	Spain	1501
KP870707	EZSPC226-09	RVcoll.08-L493	x	*Nymphalidae*	*Boloria*	*euphrosyne*	Bassa d`Arres, Vall d`Aran	Lleida	Catalonia	Spain	1501
HM901781	EZSPC944-10	RVcoll.09-X132	x	*Nymphalidae*	*Boloria*	*euphrosyne*	Pleta amagada, Vall d`Aneu, Pallars Sobira	Lleida	Catalonia	Spain	
HM386890	LEFIA746-10	MM04100		*Nymphalidae*	*Boloria*	*napaea*	Enontekioe		Lapland	Finland	
HQ570285	LEFIC094-10	MM03407		*Nymphalidae*	*Boloria*	*napaea*	Enontekioe	Lapponia enontekiensis	Lapland	Finland	
JN272537	LEFIJ543-10	MM17168		*Nymphalidae*	*Boloria*	*napaea*	Enontekioe	Lapponia enontekiensis	Lapland	Finland	
HQ565492	GWOSA776-10	BC ZSM Lep 36642		*Nymphalidae*	*Boloria*	*napaea*	Ammergauer B., Ahornsattel	Oberbayern	Bavaria	Germany	1500
KP870926	EULEP182-14	11-J433		*Nymphalidae*	*Boloria*	*napaea*	Passo Campolungo		Ticino	Switzerland	2350
GU669636	EZSPC648-09	RVcoll.07-C027	x	*Nymphalidae*	*Boloria*	*pales*	Puigmal	Languedoc-Roussillon		France	2150

GenBank accession number	BOLD Process ID	Sample ID	Used in the Iberian dataset	Family	Genus	Species	Exact Site	Region	State/Province	Country	Alt. (m)
HM901775	EZSPC938-10	RVcoll.09-X039	x	*Nymphalidae*	*Boloria*	*pales*	Vallée d´Eyne (Orri de Baix), Pyrénées-Orientales	Languedoc-Roussillon		France	2051
KP870922	FBLMX200-11	BC ZSM Lep 50411		*Nymphalidae*	*Boloria*	*pales*	Oberstdorf, Edmund-Probst-Haus	Schwaben	Bavaria	Germany	1900
HQ004094	EZROM802-08	RVcoll.08-M611		*Nymphalidae*	*Boloria*	*pales*	3 km above cabana Capra (Fagaras Mts.)	Arges	Muntenia	Romania	1830
KP870530	EULEP024-14	07-D690		*Nymphalidae*	*Boloria*	*pales*	M-ţii Bucegi	Prahova	Muntenia	Romania	1900
HQ004096	EZROM053-08	RV-07-E454		*Nymphalidae*	*Boloria*	*pales*	Babele (Bucegi Mts.)	Prahova	Muntenia	Romania	2140
HQ004095	EZROM054-08	RV-07-E575		*Nymphalidae*	*Boloria*	*pales*	Saua Dichiului (Bucegi Mts.)	Prahova	Muntenia	Romania	1560
HQ004090	EZROM663-08	RV-06-M965		*Nymphalidae*	*Boloria*	*pales*	Bucegi mountains, Cota 2000	Prahova	Muntenia	Romania	2000
HQ004091	EZROM799-08	RVcoll.06-M989		*Nymphalidae*	*Boloria*	*pales*	Bucegi Mts. (cota 2000 - cab. Miorita)	Prahova	Muntenia	Romania	2000
HQ004092	EZROM800-08	RVcoll.06-M996		*Nymphalidae*	*Boloria*	*pales*	Bucegi Mts. (cota 2000 - cab. Miorita)	Prahova	Muntenia	Romania	2000
HQ004093	EZROM801-08	RVcoll.07-D650		*Nymphalidae*	*Boloria*	*pales*	Bucegi mountains, Cota 2000	Prahova	Muntenia	Romania	2000
HM901715	EZSPC875-10	RVcoll.09-T126	x	*Nymphalidae*	*Boloria*	*pales*	Teverga, Vega Socellares		Asturias	Spain	1780
HM901716	EZSPC876-10	RVcoll.09-T127	x	*Nymphalidae*	*Boloria*	*pales*	Teverga, Vega Socellares		Asturias	Spain	1780
GU676125	EZSPM411-09	Rvcoll.09-V831	x	*Nymphalidae*	*Boloria*	*pales*	Refugi d`Ulldeter, Ripolles	Girona	Catalonia	Spain	
GU676123	EZSPM412-09	Rvcoll.09-V832	x	*Nymphalidae*	*Boloria*	*pales*	Refugi d`Ulldeter, Ripolles	Girona	Catalonia	Spain	
GU676124	EZSPM413-09	Rvcoll.09-V833	x	*Nymphalidae*	*Boloria*	*pales*	Refugi d`Ulldeter, Ripolles	Girona	Catalonia	Spain	
GU676126	EZSPM414-09	Rvcoll.09-V834	x	*Nymphalidae*	*Boloria*	*pales*	Refugi d`Ulldeter, Ripolles	Girona	Catalonia	Spain	
GU676121	EZSPM415-09	Rvcoll.09-V835	x	*Nymphalidae*	*Boloria*	*pales*	Refugi d`Ulldeter, Ripolles	Girona	Catalonia	Spain	
GU676122	EZSPM416-09	Rvcoll.09-V836	x	*Nymphalidae*	*Boloria*	*pales*	Refugi d`Ulldeter, Ripolles	Girona	Catalonia	Spain	
KP871059	EZSPM789-12	RVcoll.070611MH21	x	*Nymphalidae*	*Boloria*	*pales*	Ibon de Asnos, Panticosa	Huesca	Aragon	Spain	2060
KP870260	EZSPM794-12	RVcoll.070611MH78	x	*Nymphalidae*	*Boloria*	*pales*	Ibon de Asnos, Panticosa	Huesca	Aragon	Spain	2060
KP870355	EZSPM831-12	RVcoll.140711ZG08	x	*Nymphalidae*	*Boloria*	*pales*	Ibon de Asnos, Panticosa	Huesca	Aragon	Spain	2060
KP870576	EZSPM833-12	RVcoll.140711ZG20	x	*Nymphalidae*	*Boloria*	*pales*	Ibon de Asnos, Panticosa	Huesca	Aragon	Spain	2060
KP870435	EZSPM840-12	RVcoll.140711ZG55	x	*Nymphalidae*	*Boloria*	*pales*	Ibon de Asnos, Panticosa	Huesca	Aragon	Spain	2060
JN827872	EZSPC269-09	RVcoll.08-R201	x	*Nymphalidae*	*Boloria*	*pales*	Port de la Bonaigua, Pallars Sobira	Lleida	Catalonia	Spain	2338
JN827871	EZSPC270-09	RVcoll.08-R202	x	*Nymphalidae*	*Boloria*	*pales*	Port de la Bonaigua, Pallars Sobira	Lleida	Catalonia	Spain	2338
JN827870	EZSPC271-09	RVcoll.08-R203	x	*Nymphalidae*	*Boloria*	*pales*	Port de la Bonaigua, Pallars Sobira	Lleida	Catalonia	Spain	2338
HM901689	EZSPC848-10	RVcoll.08-R237.1	x	*Nymphalidae*	*Boloria*	*pales*	Port de la Bonaigua, Pallars Sobira	Lleida	Catalonia	Spain	2338
HQ968300	PHLAB1037-10	TLMF Lep 01837		*Nymphalidae*	*Boloria*	*pales*	Val Bever, Alp Suvretta		Graubunden	Switzerland	2100
HQ968301	PHLAB1038-10	TLMF Lep 01838		*Nymphalidae*	*Boloria*	*pales*	Val Bever, Alp Suvretta		Graubunden	Switzerland	2100
HQ968302	PHLAB1039-10	TLMF Lep 01839		*Nymphalidae*	*Boloria*	*pales*	Val Bever, Alp Suvretta		Graubunden	Switzerland	2100
KP870348	EZSPC651-09	RVcoll.07-C641	x	*Nymphalidae*	*Boloria*	*selene*	Col de Puymorens	Languedoc-Roussillon		France	1825
GU669632	EZSPC654-09	RVcoll.07-C659	x	*Nymphalidae*	*Boloria*	*selene*	Col de Puymorens	Languedoc-Roussillon		France	1825
GU669607	EZSPC675-09	RVcoll.08-M969	x	*Nymphalidae*	*Boloria*	*selene*	Col de Puymorens	Languedoc-Roussillon		France	1825
GU669608	EZSPC676-09	RVcoll.08-M970	x	*Nymphalidae*	*Boloria*	*selene*	Col de Puymorens	Languedoc-Roussillon		France	1825
HM393213	GWORA2494-09	BC ZSM Lep 30706		*Nymphalidae*	*Boloria*	*selene*	Grandsberg	Niederbayern	Bavaria	Germany	850
HM391821	FBLMU471-09	BC ZSM Lep 27121		*Nymphalidae*	*Boloria*	*selene*	Traunstein, Pechschnait	Oberbayern	Bavaria	Germany	695
JF415687	GWORK309-09	BC ZSM Lep 21544		*Nymphalidae*	*Boloria*	*selene*	Koenigsdorf, Weitfilz	Oberbayern	Bavaria	Germany	600
JF415686	GWORR667-10	BC ZSM Lep 32163		*Nymphalidae*	*Boloria*	*selene*	Garching-Hochbrueck	Oberbayern	Bavaria	Germany	487
JF415685	GWORR668-10	BC ZSM Lep 32164		*Nymphalidae*	*Boloria*	*selene*	Geretsried, Babenstuben	Oberbayern	Bavaria	Germany	
GU676580	EZSPN565-09	RVcoll.08-J106	x	*Nymphalidae*	*Boloria*	*selene*	Ribeira de Varzea (EN311), Varzea Cova, Fafe		Minho	Portugal	500
GU676555	EZSPN588-09	RVcoll.08-J151	x	*Nymphalidae*	*Boloria*	*selene*	Ponte Pedrinha, Beca, Boticas		Tras-os-Montes	Portugal	730
HQ004097	EZROM615-08	RV-07-D313		*Nymphalidae*	*Boloria*	*selene*	Racos	Brasov	Transylvania	Romania	460
HQ004103	EZROM101-08	RV-07-C902		*Nymphalidae*	*Boloria*	*selene*	Teregova	Caras-Severin	Banat	Romania	385
HQ004100	EZROM097-08	RV-06-K635		*Nymphalidae*	*Boloria*	*selene*	Baciu forest (Cluj-Napoca)	Cluj	Transylvania	Romania	530
HQ004101	EZROM098-08	RV-06-K642		*Nymphalidae*	*Boloria*	*selene*	Baciu forest (Cluj-Napoca)	Cluj	Transylvania	Romania	530
HQ004099	EZROM099-08	RV-07-C421		*Nymphalidae*	*Boloria*	*selene*	4 km west of I.L. Caragiale	Dambovita	Muntenia	Romania	270
HQ004102	EZROM100-08	RV-07-D973		*Nymphalidae*	*Boloria*	*selene*	4 km west of I.L. Caragiale	Dambovita	Muntenia	Romania	270
HQ004105	EZROM860-08	RVcoll.08-M223		*Nymphalidae*	*Boloria*	*selene*	2 Km E of Moreni	Dambovita	Muntenia	Romania	290
HQ004104	EZROM861-08	RVcoll.08-M344		*Nymphalidae*	*Boloria*	*selene*	1 Km E of Gheorgheni	Harghita	Transylvania	Romania	900
HQ004098	EZROM573-08	RV-07-C336		*Nymphalidae*	*Boloria*	*selene*	Radauti	Suceava	Moldavia	Romania	375

GenBank accession number	BOLD Process ID	Sample ID	Used in the Iberian dataset	Family	Genus	Species	Exact Site	Region	State/Province	Country	Alt. (m)
FJ663335	LOWA756-06	2005-LOWA-756		*Nymphalidae*	*Boloria*	*selene*	Tamengont	Oranienbaum distr.	St. Petersburg	Russia	
GU676319	EZSPN831-09	RVcoll.08-L649	x	*Nymphalidae*	*Boloria*	*selene*	Hoyocasero, Pinar de Hoyocasero	Avila	Castilla y Leon	Spain	1254
JF848005	EZSPN062-09	RVcoll.09-V690	x	*Nymphalidae*	*Boloria*	*selene*	Meranges	Girona	Catalonia	Spain	1750
GU676365	EZSPN790-09	RVcoll.08-J893	x	*Nymphalidae*	*Boloria*	*selene*	Vega de Espinareda	Leon	Castilla y Leon	Spain	
HM901706	EZSPC865-10	RVcoll.09-T082	x	*Nymphalidae*	*Boloria*	*selene*	Vall d`Hurno, Vall d`Aran	Lleida	Catalonia	Spain	1800
HM901696	EZSPC855-10	RVcoll.08-R445	x	*Nymphalidae*	*Boloria*	*selene*	Tui, Rio Penedo	Pontevedra	Galicia	Spain	30
GU675766	EZSPM240-09	RVcoll.08-R444	x	*Nymphalidae*	*Boloria*	*selene*	Tui, Rio Penedo	Pontevedra	Galicia	Spain	30
GU676900	EZSPN497-09	RVcoll.08-H943	x	*Nymphalidae*	*Boloria*	*selene*	Candelario, Sierra de Candelario	Salamanca	Castilla y Leon	Spain	1300
GU676258	EZSPN874-09	RVcoll.08-L765	x	*Nymphalidae*	*Boloria*	*selene*	Valsain	Segovia	Castilla y Leon	Spain	1400
HM386883	LEFIA739-10	MM04093		*Nymphalidae*	*Boloria*	*thore*	Enontekioe		Lapland	Finland	
HM386884	LEFIA740-10	MM04094		*Nymphalidae*	*Boloria*	*thore*	Enontekioe		Lapland	Finland	
HQ570286	LEFIC095-10	MM03408		*Nymphalidae*	*Boloria*	*thore*	Enontekioe	Lapponia enontekiensis	Lapland	Finland	
GU688473	GWORO851-09	BC ZSM Lep 30543		*Nymphalidae*	*Boloria*	*thore*	Laubau		Bavaria	Germany	
HM391823	FBLMU475-09	BC ZSM Lep 27125		*Nymphalidae*	*Boloria*	*thore*	Ruhpolding, Eschelmoos	Oberbayern	Bavaria	Germany	950
JF415688	GWORK566-09	BC ZSM Lep 21896		*Nymphalidae*	*Boloria*	*thore*	Wettersteingebirge, Kreuzeck-Stuibensee	Oberbayern	Bavaria	Germany	1600
JX034684	FBLMX199-11	BC ZSM Lep 50410		*Nymphalidae*	*Boloria*	*thore*	Oberstdorf, Oytal	Schwaben	Bavaria	Germany	1025
HQ968293	PHLAB1030-10	TLMF Lep 01830		*Nymphalidae*	*Boloria*	*thore*	Chur, Pagig		Graubunden	Switzerland	1400
HQ968294	PHLAB1031-10	TLMF Lep 01831		*Nymphalidae*	*Boloria*	*thore*	Alt St. Johann, Gmeinenwies		Sankt Gallen	Switzerland	1500
HQ968295	PHLAB1032-10	TLMF Lep 01832		*Nymphalidae*	*Boloria*	*thore*	Alt St. Johann, Gmeinenwies		Sankt Gallen	Switzerland	1500
JX034685	PHLAB292-10	TLMF Lep 01092		*Nymphalidae*	*Boloria*	*thore*	Tiefencastel S/ Salouf, Got Grond		Graubunden	Switzerland	1650
JF860244	PHLAC686-10	TLMF Lep 02721		*Nymphalidae*	*Boloria*	*thore*	Riefawald/ Vals		Graubunden	Switzerland	1800
HM391822	FBLMU472-09	BC ZSM Lep 27122		*Nymphalidae*	*Boloria*	*titania*	Bergen b. Traunstein, Hochfelln	Oberbayern	Bavaria	Germany	1670
HM393214	GWORA2496-09	BC ZSM Lep 30708		*Nymphalidae*	*Boloria*	*titania*	Sudelfeld, Arzmoos	Oberbayern	Bavaria	Germany	1200
JF415689	GWORR672-10	BC ZSM Lep 32168		*Nymphalidae*	*Boloria*	*titania*	Samerberg, Lederstuben	Oberbayern	Bavaria	Germany	750
KP870710	FBLMX197-11	BC ZSM Lep 50408		*Nymphalidae*	*Boloria*	*titania*	Oberstdorf, Oytal	Schwaben	Bavaria	Germany	1025
HQ004106	EZRMN428-09	RVcoll.09-V681		*Nymphalidae*	*Boloria*	*titania*	Valea Belchia (Gheorgheni)	Harghita	Transylvania	Romania	1000
HQ004107	EZROM055-08	RV-06-N010		*Nymphalidae*	*Boloria*	*titania*	Gheorgheni, Valea Belchia	Harghita	Transylvania	Romania	1010
HQ004108	EZROM803-08	RVcoll.08-M601		*Nymphalidae*	*Boloria*	*titania*	Gheorgheni, Valea Belchia	Harghita	Transylvania	Romania	1000
HQ004109	EZROM804-08	RVcoll.08-M602		*Nymphalidae*	*Boloria*	*titania*	Gheorgheni, Valea Belchia	Harghita	Transylvania	Romania	1000
HQ004110	EZROM805-08	RVcoll.08-M603		*Nymphalidae*	*Boloria*	*titania*	Gheorgheni, Valea Belchia	Harghita	Transylvania	Romania	1000
HQ004111	EZROM806-08	RVcoll.08-M604		*Nymphalidae*	*Boloria*	*titania*	Gheorgheni, Valea Belchia	Harghita	Transylvania	Romania	1000
KP870950	EULEP025-14	07-D710	x	*Hesperiidae*	*Borbo*	*borbonica*	Algeciras	Cádiz	Andalusia	Spain	
KP870285	EULEP031-14	07-D799	x	*Hesperiidae*	*Borbo*	*borbonica*	Algeciras	Cádiz	Andalusia	Spain	
KP870416	EULEP222-14	12-L074	x	*Hesperiidae*	*Borbo*	*borbonica*	Benalup	Cádiz	Andalusia	Spain	10
KP870215	EULEP223-14	12-L075	x	*Hesperiidae*	*Borbo*	*borbonica*	Benalup	Cádiz	Andalusia	Spain	10
KP871028	EULEP224-14	12-L076	x	*Hesperiidae*	*Borbo*	*borbonica*	Benalup	Cádiz	Andalusia	Spain	10
KP870814	EULEP225-14	12-L077	x	*Hesperiidae*	*Borbo*	*borbonica*	Benalup	Cádiz	Andalusia	Spain	10
KP870782	EULEP226-14	12-L078	x	*Hesperiidae*	*Borbo*	*borbonica*	Benalup	Cádiz	Andalusia	Spain	10
KP871176	EULEP227-14	12-L079	x	*Hesperiidae*	*Borbo*	*borbonica*	Benalup	Cádiz	Andalusia	Spain	10
KP870452	EULEP228-14	12-L080	x	*Hesperiidae*	*Borbo*	*borbonica*	Benalup	Cádiz	Andalusia	Spain	10
KP870338	EULEP229-14	12-L081	x	*Hesperiidae*	*Borbo*	*borbonica*	Benalup	Cádiz	Andalusia	Spain	10
HQ004117	EZROM058-08	RV-06-M960		*Nymphalidae*	*Brenthis*	*daphne*	Dealul Istrita, Breaza	Buzau	Muntenia	Romania	600
HQ004116	EZROM060-08	RV-07-C912		*Nymphalidae*	*Brenthis*	*daphne*	Teregova	Caras-Severin	Banat	Romania	385
HQ004114	EZROM810-08	RVcoll.08-M548		*Nymphalidae*	*Brenthis*	*daphne*	Valea Feregarilor - Poiana Musuroaie	Caras-Severin	Banat	Romania	350
HQ004120	EZROM056-08	RV-06-M834		*Nymphalidae*	*Brenthis*	*daphne*	Cluj-Napoca, Baciu forest	Cluj	Transylvania	Romania	530
HQ004119	EZROM057-08	RV-06-M873		*Nymphalidae*	*Brenthis*	*daphne*	Cluj-Napoca, dealul Feleacului, Gheorgheni forest	Cluj	Transylvania	Romania	600
HQ004113	EZROM809-08	RVcoll.08-M503		*Nymphalidae*	*Brenthis*	*daphne*	Badeni	Cluj	Transylvania	Romania	490
HQ004115	EZROM808-08	RVcoll.08-M452		*Nymphalidae*	*Brenthis*	*daphne*	Canaraua Fetei (Baneasa)	Constanta	Dobrogea	Romania	20
HQ004118	EZROM059-08	RV-07-D978		*Nymphalidae*	*Brenthis*	*daphne*	4 km west of I.L. Caragiale	Dambovita	Muntenia	Romania	270
HQ004112	EZROM607-08	RV-06-M910		*Nymphalidae*	*Brenthis*	*daphne*	Stana	Salaj	Transylvania	Romania	500
GU676246	EZSPN907-09	RVcoll.08-L831	x	*Nymphalidae*	*Brenthis*	*daphne*	Oteruelo del Valle		Comunidad de Madrid	Spain	1152
KP870262	EZROM708-08	RV-06-H895	x	*Nymphalidae*	*Brenthis*	*daphne*	Vallforners, Canoves	Barcelona	Catalonia	Spain	650
KP870740	EZSPC228-09	RVcoll.08-L518	x	*Nymphalidae*	*Brenthis*	*daphne*	El Cafe, pla de la Calma, El Brull, Osona	Barcelona	Catalonia	Spain	1192

GenBank accession number	BOLD Process ID	Sample ID	Used in the Iberian dataset	Family	Genus	Species	Exact Site	Region	State/Province	Country	Alt. (m)
KP870238	EZSPC229-09	RVcoll.08-L519	x	*Nymphalidae*	*Brenthis*	*daphne*	El Cafe, pla de la Calma, El Brull, Osona	Barcelona	Catalonia	Spain	1192
HM901619	EZSPC764-10	RVcoll.08-L511	x	*Nymphalidae*	*Brenthis*	*daphne*	El Cafe, Pla de la Calma el Brull, OSONA	Barcelona	Catalonia	Spain	1192
HM901588	EZSPC733-10	RVcoll.08-H908	x	*Nymphalidae*	*Brenthis*	*daphne*	Hervas, Carretera Hervas-La Garganta	Caceres	Extremadura	Spain	1007
HM901589	EZSPC734-10	RVcoll.08-H920.1	x	*Nymphalidae*	*Brenthis*	*daphne*	Hervas, Carretera Hervas-La Garganta	Caceres	Extremadura	Spain	774
GU676956	EZSPN475-09	RVcoll.08-H909	x	*Nymphalidae*	*Brenthis*	*daphne*	Hervas, Carretera Hervas-La Garganta	Caceres	Extremadura	Spain	1007
GU676195	EZSPN971-09	RVcoll.08-L991	x	*Nymphalidae*	*Brenthis*	*daphne*	Una	Cuenca	Castilla-La Mancha	Spain	1201
KP870357	EZSPC246-09	RVcoll.08-P069	x	*Nymphalidae*	*Brenthis*	*daphne*	Torrent de Ridolaina, Cerdanya	Girona	Catalonia	Spain	1060
GU676130	EZSPM408-09	Rvcoll.09-V825	x	*Nymphalidae*	*Brenthis*	*daphne*	Vilallonga de Ter, Ripolles	Girona	Catalonia	Spain	
HM901636	EZSPC781-10	RVcoll.08-L902	x	*Nymphalidae*	*Brenthis*	*daphne*	San Andres del Rey	Guadalajara	Castilla-La Mancha	Spain	1018
KP870768	EZSPM786-12	RVcoll.070611MH06	x	*Nymphalidae*	*Brenthis*	*daphne*	El Pueyo de Jaca, Panticosa	Huesca	Aragon	Spain	1779
KP870971	EZSPM792-12	RVcoll.070611MH67	x	*Nymphalidae*	*Brenthis*	*daphne*	El Pueyo de Jaca, Panticosa	Huesca	Aragon	Spain	1779
KP870891	EZSPM839-12	RVcoll.140711ZG48	x	*Nymphalidae*	*Brenthis*	*daphne*	Balneario de Panticosa	Huesca	Aragon	Spain	1642
GU676137	EZSPN1025-09	RVcoll.08-M958	x	*Nymphalidae*	*Brenthis*	*daphne*	Forcat	Huesca	Aragon	Spain	1039
KP870706	EZSPC254-09	RVcoll.08-P345	x	*Nymphalidae*	*Brenthis*	*daphne*	Perves, Pallars Jussa	Lleida	Catalonia	Spain	1071
HQ004128	EZROM813-08	RVcoll.08-M516		*Nymphalidae*	*Brenthis*	*hecate*	Apuseni Mts., Rimetea (Piatra Secuiului)	Alba	Transylvania	Romania	850
HQ004123	EZROM186-08	RV-07-D344		*Nymphalidae*	*Brenthis*	*hecate*	Racos	Brasov	Transylvania	Romania	460
HQ004124	EZROM288-08	RV-07-E650		*Nymphalidae*	*Brenthis*	*hecate*	Dealul Istrita, Breaza	Buzau	Muntenia	Romania	525
HQ004122	EZROM062-08	RV-07-C970		*Nymphalidae*	*Brenthis*	*hecate*	Baile Herculane (Pecinisca)	Caras-Severin	Banat	Romania	220
HQ004121	EZROM061-08	RV-06-M850		*Nymphalidae*	*Brenthis*	*hecate*	Cluj-Napoca, dealul Feleacului, Gheorgheni forest	Cluj	Transylvania	Romania	600
HQ004125	EZROM609-08	RV-07-D543		*Nymphalidae*	*Brenthis*	*hecate*	Rohia	Maramures	Maramures	Romania	360
KP871027	EULEP013-14	06-M911		*Nymphalidae*	*Brenthis*	*hecate*	Stana, pǎd. Rotunda	Salaj	Transylvania	Romania	500
HQ004126	EZROM811-08	RVcoll.08-M433		*Nymphalidae*	*Brenthis*	*hecate*	3 km E of Slava Rusa (Babadag forest)	Tulcea	Dobrogea	Romania	130
HQ004127	EZROM812-08	RVcoll.08-M461		*Nymphalidae*	*Brenthis*	*hecate*	Babadag forest (3 Km E of Slava Rusa)	Tulcea	Dobrogea	Romania	130
HM901287	EZSPC1086-10	RVcoll.08-L839	x	*Nymphalidae*	*Brenthis*	*hecate*	Oteruelo del Valle		Comunidad de Madrid	Spain	1152
HM901288	EZSPC1087-10	RVcoll.08-L840	x	*Nymphalidae*	*Brenthis*	*hecate*	Oteruelo del Valle		Comunidad de Madrid	Spain	1152
GU676248	EZSPN909-09	RVcoll.08-L838	x	*Nymphalidae*	*Brenthis*	*hecate*	Oteruelo del Valle		Comunidad de Madrid	Spain	1152
GU676211	EZSPN951-09	RVcoll.08-L943	x	*Nymphalidae*	*Brenthis*	*hecate*	Rascafria		Comunidad de Madrid	Spain	1150
KP870617	EZROM362-08	RVcoll.07-E630	x	*Nymphalidae*	*Brenthis*	*hecate*	Cal Farmico, Moror	Lleida	Catalonia	Spain	920
KP871051	EZROM363-08	07-E631	x	*Nymphalidae*	*Brenthis*	*hecate*	Cal Farmico, Moror	Lleida	Catalonia	Spain	920
JN272500	EZSPN1100-11	Rvcoll.10-A831	x	*Nymphalidae*	*Brenthis*	*hecate*	Coll de Faidella, Boixols, Pallars Jussa	Lleida	Catalonia	Spain	1230
HM901272	EZSPC1069-10	RVcoll.08-L220	x	*Nymphalidae*	*Brenthis*	*hecate*	Ruta de la Sima, El Vallecillo	Teruel	Aragon	Spain	1531
HM901273	EZSPC1070-10	RVcoll.08-L238	x	*Nymphalidae*	*Brenthis*	*hecate*	Ruta de la Sima, El Vallecillo	Teruel	Aragon	Spain	1531
GU676385	EZSPN759-09	RVcoll.08-L237	x	*Nymphalidae*	*Brenthis*	*hecate*	Ruta de la Sima, El Vallecillo	Teruel	Aragon	Spain	1531
HM393212	GWORA2493-09	BC ZSM Lep 30705		*Nymphalidae*	*Brenthis*	*ino*	Fuerstenzell, Altenmarkt	Niederbayern	Bavaria	Germany	460
JF415690	GWORR666-10	BC ZSM Lep 32162		*Nymphalidae*	*Brenthis*	*ino*	Lindenrain, Geretsried	Oberbayern	Bavaria	Germany	610
HQ004134	EZROM065-08	RV-07-D345		*Nymphalidae*	*Brenthis*	*ino*	Racos	Brasov	Transylvania	Romania	460
HQ004132	EZROM815-08	RVcoll.07-D309		*Nymphalidae*	*Brenthis*	*ino*	Racos	Brasov	Transylvania	Romania	460
HQ004129	EZROM816-08	RVcoll.07-D318		*Nymphalidae*	*Brenthis*	*ino*	Racos	Brasov	Transylvania	Romania	460
HQ004130	EZROM817-08	RVcoll.07-D346		*Nymphalidae*	*Brenthis*	*ino*	Racos	Brasov	Transylvania	Romania	460
HQ004136	EZROM063-08	RV-06-M865		*Nymphalidae*	*Brenthis*	*ino*	Cluj-Napoca, dealul Feleacului, Gheorgheni forest	Cluj	Transylvania	Romania	600
KP870375	EULEP140-14	10-C414		*Nymphalidae*	*Brenthis*	*ino*	Gheorgheni, Valea Belchia	Harghita	Transylvania	Romania	
HQ004135	EZROM064-08	RV-06-M906		*Nymphalidae*	*Brenthis*	*ino*	Stana	Salaj	Transylvania	Romania	500
HQ004131	EZROM814-08	RVcoll.06-M899		*Nymphalidae*	*Brenthis*	*ino*	Stana	Salaj	Transylvania	Romania	500
HQ004133	EZROM807-08	RVcoll.08-M478		*Nymphalidae*	*Brenthis*	*ino*	Babadag forest (3 Km E of Slava Rusa)	Tulcea	Dobrogea	Romania	130
HM901802	EZSPC967-10	RVcoll.210207TM83	x	*Nymphalidae*	*Brenthis*	*ino*	Font del Regatxal, Vilafranca	Castellon	Comunidad Valenciana	Spain	1394
GU676396	EZSPN744-09	RVcoll.08-L171	x	*Nymphalidae*	*Brenthis*	*ino*	Font del Regatxal, Vilafranca	Castellon	Comunidad Valenciana	Spain	1361
GU676876	EZSPN471-09	RVcoll.08-H699	x	*Nymphalidae*	*Brenthis*	*ino*	Huelamo, Rio Jucar	Cuenca	Castilla-La Mancha	Spain	1237
KP871129	EZSPC205-09	RVcoll.07-C670	x	*Nymphalidae*	*Brenthis*	*ino*	Sant Marti de Puigcerda	Girona	Catalonia	Spain	1145
KP870504	EZSPC206-09	RVcoll.07-C675	x	*Nymphalidae*	*Brenthis*	*ino*	Sant Marti de Puigcerda	Girona	Catalonia	Spain	1145

GenBank accession number	BOLD Process ID	Sample ID	Used in the Iberian dataset	Family	Genus	Species	Exact Site	Region	State/Province	Country	Alt. (m)
KP870563	EZSPC237-09	RVcoll.08-P011	x	*Nymphalidae*	*Brenthis*	*ino*	Torre de Riu, Ribera d`Alt, Cerdanya	Girona	Catalonia	Spain	1180
HM901768	EZSPC931-10	RVcoll.09-X005	x	*Nymphalidae*	*Brenthis*	*ino*	Vallsabollera, La Cerdanya	Girona	Catalonia	Spain	1560
KP870408	EZSPM788-12	RVcoll.070611MH13	x	*Nymphalidae*	*Brenthis*	*ino*	Panticosa	Huesca	Aragon	Spain	1214
GU675953	EZSPM086-09	RVcoll.08-P409	x	*Nymphalidae*	*Brenthis*	*ino*	Buron	Leon	Castilla y Leon	Spain	1116
GU676221	EZSPN837-09	RVcoll.08-J835	x	*Nymphalidae*	*Brenthis*	*ino*	La Una	Leon	Castilla y Leon	Spain	
KP870898	EZSPC234-09	RVcoll.08-M913	x	*Nymphalidae*	*Brenthis*	*ino*	Bassa d`Arres, Vall d`Aran	Lleida	Catalonia	Spain	1500
KP870861	EZSPC261-09	RVcoll.08-P384	x	*Nymphalidae*	*Brenthis*	*ino*	Arros, Vall d`Aran	Lleida	Catalonia	Spain	906
HM901704	EZSPC863-10	RVcoll.09-T068	x	*Nymphalidae*	*Brenthis*	*ino*	Sant Joan de Toran, Vall d`Aran	Lleida	Catalonia	Spain	980
HM901756	EZSPC918-10	RVcoll.09-V883	x	*Nymphalidae*	*Brenthis*	*ino*	Banys de Tredos, Vall d`Aran	Lleida	Catalonia	Spain	
GU676231	EZSPN925-09	RVcoll.08-L874	x	*Nymphalidae*	*Brenthis*	*ino*	Abejar	Soria	Castilla y Leon	Spain	1097
KP871041	EULEP243-14	12-N703	x	*Nymphalidae*	*Brenthis*	*ino*	El Valecillo	Teruel	Aragón	Spain	1526.7
KP870879	EZSPM763-12	RVcoll.030111FB83	x	*Nymphalidae*	*Brenthis*	*ino*	Fuente del Pinarico, Valdelinares	Teruel	Aragon	Spain	1712
KP870356	EZSPM807-12	RVcoll.090611SZ94	x	*Nymphalidae*	*Brenthis*	*ino*	Fuente del Pinarico, Valdelinares	Teruel	Aragon	Spain	1712
KP870510	EZSPM817-12	RVcoll.130711PX83	x	*Nymphalidae*	*Brenthis*	*ino*	Solano de la Vega, Alcala de la Selva	Teruel	Aragon	Spain	1501
KP870330	EZSPM818-12	RVcoll.130711PX89	x	*Nymphalidae*	*Brenthis*	*ino*	Solano de la Vega, Alcala de la Selva	Teruel	Aragon	Spain	1501
GU676392	EZSPN753-09	RVcoll.08-L225	x	*Nymphalidae*	*Brenthis*	*ino*	Ruta de la Sima, El Vallecillo	Teruel	Aragon	Spain	1531
HM902068	FBLMU378-09	BC ZSM Lep 27028		*Nymphalidae*	*Brintesia*	*circe*	Regenstauf, Kreuth	Oberpfalz	Bavaria	Germany	520
GU676567	EZSPN576-09	RVcoll.08-J123	x	*Nymphalidae*	*Brintesia*	*circe*	Seculca, Beca, Boticas		Tras-os-Montes	Portugal	790
HQ004144	EZROM067-08	RV-07-E381		*Nymphalidae*	*Brintesia*	*circe*	Dobraia (surroundings)	Caras-Severin	Banat	Romania	640
HQ004142	EZROM822-08	RVcoll.08-M541		*Nymphalidae*	*Brintesia*	*circe*	Valea Feregarilor - Poiana Musuroaie	Caras-Severin	Banat	Romania	500
HQ004145	EZRMN343-08	RVcoll.08-H039		*Nymphalidae*	*Brintesia*	*circe*	Dealul Sf. Pavel (Cluj-Napoca)	Cluj	Transylvania	Romania	450
HQ004143	EZROM066-08	RV-06-V716		*Nymphalidae*	*Brintesia*	*circe*	Cluj-Napoca, Hoia forest	Cluj	Transylvania	Romania	450
HQ004137	EZROM610-08	RV-06-V717		*Nymphalidae*	*Brintesia*	*circe*	Cluj-Napoca, Hoia forest	Cluj	Transylvania	Romania	450
HQ004139	EZROM818-08	RVcoll.08-M468		*Nymphalidae*	*Brintesia*	*circe*	Babadag forest (10 Km S of Babadag)	Tulcea	Dobrogea	Romania	115
HQ004138	EZROM819-08	RVcoll.08-M469		*Nymphalidae*	*Brintesia*	*circe*	Babadag forest (10 Km S of Babadag)	Tulcea	Dobrogea	Romania	115
HQ004140	EZROM820-08	RVcoll.08-M488		*Nymphalidae*	*Brintesia*	*circe*	Macin Mts., Greci (Esitul Sufletului)	Tulcea	Dobrogea	Romania	200
HQ004141	EZROM821-08	RVcoll.08-M489		*Nymphalidae*	*Brintesia*	*circe*	Macin Mts., Greci (Esitul Sufletului)	Tulcea	Dobrogea	Romania	200
KP870990	EZSPC217-09	RVcoll.08-J416	x	*Nymphalidae*	*Brintesia*	*circe*	El Puig, Montseny, Valles Oriental	Barcelona	Catalonia	Spain	1064
GU669628	EZSPC658-09	RVcoll.08-L383	x	*Nymphalidae*	*Brintesia*	*circe*	Masia dels Plans, Vidra, Garrotxa	Barcelona	Catalonia	Spain	1365
GU669604	EZSPC679-09	RVcoll.08-L384	x	*Nymphalidae*	*Brintesia*	*circe*	Cardedeu, bosc de Can Morato	Barcelona	Catalonia	Spain	180
KP870490	EZSPM956-12	Rvcoll.12-M707	x	*Nymphalidae*	*Brintesia*	*circe*	Alrededores San Lorenzo de Calatrava	Ciudad Real	Castilla-La Mancha	Spain	749
GU676872	EZSPN466-09	RVcoll.08-H683	x	*Nymphalidae*	*Brintesia*	*circe*	Huelamo, Rio Jucar	Cuenca	Castilla-La Mancha	Spain	1237
KP871113	EZROM727-08	RV-07-C035	x	*Nymphalidae*	*Brintesia*	*circe*	Gombren, Ripolles	Girona	Catalonia	Spain	980
HM901400	EZSPC1200-10	RVcoll.09-V425	x	*Nymphalidae*	*Brintesia*	*circe*	Loma de Lastonares (Sierra Nevada)	Granada	Andalusia	Spain	2050
HM901492	EZSPC1356-10	RVcoll.09-V929	x	*Nymphalidae*	*Brintesia*	*circe*	Sierra de la Sagra	Granada	Andalusia	Spain	1745
HM901291	EZSPC1090-10	RVcoll.08-L905	x	*Nymphalidae*	*Brintesia*	*circe*	Gualda	Guadalajara	Castilla-La Mancha	Spain	730
GU676173	EZSPN985-09	RVcoll.08-M025	x	*Nymphalidae*	*Brintesia*	*circe*	Gualda, La Alcarria	Guadalajara	Castilla-La Mancha	Spain	730
GU675865	EZSPM372-09	RVcoll.08-J856	x	*Nymphalidae*	*Brintesia*	*circe*	Bercianos del Real Camino	Leon	Castilla y Leon	Spain	
KP870605	EZSPC231-09	RVcoll.08-M694	x	*Nymphalidae*	*Brintesia*	*circe*	Farena, Alt Camp	Tarragona	Catalonia	Spain	580
GU676586	EZSPN560-09	RVcoll.08-J088	x	*Nymphalidae*	*Brintesia*	*circe*	Sinarcas, Sinarcas	Valencia	Comunidad Valenciana	Spain	
HM901319	EZSPC1118-10	RVcoll.08-P460	x	*Lycaenidae*	*Cacyreus*	*marshalli*	Tres Cantos		Comunidad de Madrid	Spain	730
HM901321	EZSPC1121-10	RVcoll.08-P468	x	*Lycaenidae*	*Cacyreus*	*marshalli*	Tres Cantos		Comunidad de Madrid	Spain	730
GU675741	EZSPM113-09	RVcoll.08-P462	x	*Lycaenidae*	*Cacyreus*	*marshalli*	Tres Cantos		Comunidad de Madrid	Spain	730
GU675804	EZSPM219-09	RVcoll.08-R406	x	*Lycaenidae*	*Cacyreus*	*marshalli*	Oviedo, Casco urbano		Asturias	Spain	200
GU675806	EZSPM202-09	RVcoll.08-R300	x	*Lycaenidae*	*Cacyreus*	*marshalli*	Cantallops, Alt Emporda	Girona	Catalonia	Spain	225
GU676999	EZSPN222-09	RVcoll.07-F308	x	*Lycaenidae*	*Cacyreus*	*marshalli*	Viladrau, Osona	Girona	Catalonia	Spain	
GU675987	EZSPM045-09	RVcoll.08-J814	x	*Lycaenidae*	*Cacyreus*	*marshalli*	Ponferrada	Leon	Castilla y Leon	Spain	
GU675988	EZSPM058-09	RVcoll.08-P284	x	*Lycaenidae*	*Cacyreus*	*marshalli*	Genova	Mallorca	Balearic Islands	Spain	123
GU675985	EZSPM059-09	RVcoll.08-P285	x	*Lycaenidae*	*Cacyreus*	*marshalli*	Genova	Mallorca	Balearic Islands	Spain	123
GU675803	EZSPM206-09	RVcoll.08-R324	x	*Lycaenidae*	*Cacyreus*	*marshalli*	Es Teix, Valldemossa	Mallorca	Balearic Islands	Spain	494
HM901390	EZSPC1190-10	RVcoll.09-V334	x	*Lycaenidae*	*Cacyreus*	*marshalli*	Ametlla de Mar, Baix Ebre	Tarragona	Catalonia	Spain	187
GU676532	EZSPN614-09	RVcoll.08-J375	x	*Lycaenidae*	*Callophrys*	*avis*	Turo del Fumet, La Floresta, Valles Occidental	Barcelona	Catalonia	Spain	315

GenBank accession number	BOLD Process ID	Sample ID	Used in the Iberian dataset	Family	Genus	Species	Exact Site	Region	State/Province	Country	Alt. (m)
GU676533	EZSPN615-09	RVcoll.08-J376	x	*Lycaenidae*	*Callophrys*	*avis*	Turo del Fumet, La Floresta, Valles Occidental	Barcelona	Catalonia	Spain	315
GU676522	EZSPN616-09	RVcoll.08-J382	x	*Lycaenidae*	*Callophrys*	*avis*	Turo del Fumet, La Floresta, Valles Occidental	Barcelona	Catalonia	Spain	315
GU676346	EZSPN808-09	RVcoll.08-L500	x	*Lycaenidae*	*Callophrys*	*avis*	Sot de Mas Forts, Sant Pere de Vilamajor, Valles Oriental	Barcelona	Catalonia	Spain	480
GU676347	EZSPN809-09	RVcoll.08-L501	x	*Lycaenidae*	*Callophrys*	*avis*	Can Roviretes, Sant Esteve de Palautordera, Valles Oriental	Barcelona	Catalonia	Spain	320
HM901462	EZSPC1287-10	RVcoll.100607MP29	x	*Lycaenidae*	*Callophrys*	*avis*	Font de la Piqueta,Espadilla	Castellon	Comunidad Valenciana	Spain	709
GU688440	GWORO785-09	BC ZSM Lep 30477		*Lycaenidae*	*Callophrys*	*rubi*	Garching-Hochbrueck Muenchen		Bavaria	Germany	486
GU688437	GWORO786-09	BC ZSM Lep 30478		*Lycaenidae*	*Callophrys*	*rubi*	Geretsried /TOEL Lindenrain		Bavaria	Germany	
HQ004148	EZROM825-08	RVcoll.08-M215		*Lycaenidae*	*Callophrys*	*rubi*	3 Km SW of Lunca Corbului	Arges	Muntenia	Romania	235
GU669660	EZROM1049-09	RVcoll.07-D298		*Lycaenidae*	*Callophrys*	*rubi*	Dumbrava Vadului, Vad	Brasov	Transylvania	Romania	495
HQ004147	EZROM824-08	RVcoll.07-D292		*Lycaenidae*	*Callophrys*	*rubi*	Dumbrava Vadului, Vad	Brasov	Transylvania	Romania	495
HQ004151	EZROM070-08	RV-07-D207		*Lycaenidae*	*Callophrys*	*rubi*	Dealul Istrita, Breaza	Buzau	Muntenia	Romania	540
HQ004152	EZROM071-08	RV-07-D230		*Lycaenidae*	*Callophrys*	*rubi*	Dealul Istrita, Breaza	Buzau	Muntenia	Romania	540
HQ004146	EZROM823-08	RVcoll.06-M944		*Lycaenidae*	*Callophrys*	*rubi*	Dealul Istrita, Breaza	Buzau	Muntenia	Romania	600
HQ004149	EZROM068-08	RV-06-K552		*Lycaenidae*	*Callophrys*	*rubi*	Badeni	Cluj	Transylvania	Romania	440
HQ004150	EZROM069-08	RV-06-K553		*Lycaenidae*	*Callophrys*	*rubi*	Badeni	Cluj	Transylvania	Romania	440
HQ004153	EZROM072-08	RV-07-D469		*Lycaenidae*	*Callophrys*	*rubi*	Catcau	Cluj	Transylvania	Romania	255
HQ004154	EZROM1046-09	RVcoll.07-D180		*Lycaenidae*	*Callophrys*	*rubi*	Sipote	Constanta	Dobrogea	Romania	90
GU675782	EZSPM281-09	SMcoll.150308PP64	x	*Lycaenidae*	*Callophrys*	*rubi*	La Serrella - Aitana, Confrides	Alicante	Comunidad Valenciana	Spain	1178
GU675877	EZSPM359-09	RVcoll.08-R039	x	*Lycaenidae*	*Callophrys*	*rubi*	Arenal de Almorxo	Alicante	Comunidad Valenciana	Spain	477
KP870394	EZROM710-08	RV-06-G432	x	*Lycaenidae*	*Callophrys*	*rubi*	El Burgues, Coll Formic, Montseny	Barcelona	Catalonia	Spain	1000
KP870259	EZSPC201-09	RVcoll.06-H868	x	*Lycaenidae*	*Callophrys*	*rubi*	Vallforners, Canoves, Valles Oriental	Barcelona	Catalonia	Spain	650
KP871091	EZSPC213-09	RVcoll.08-J374	x	*Lycaenidae*	*Callophrys*	*rubi*	Vallgrassa, Parc Natural del Garraf	Barcelona	Catalonia	Spain	285
KP871065	EZSPC214-09	RVcoll.08-J385	x	*Lycaenidae*	*Callophrys*	*rubi*	Turo del Fumet, La Floresta, Valles Occidental	Barcelona	Catalonia	Spain	315
GU676457	EZSPN690-09	RVcoll.08-L020	x	*Lycaenidae*	*Callophrys*	*rubi*	Casillas de Rojas, Gueejar Sierra	Granada	Andalusia	Spain	1733
GU676640	EZSPN407-09	RVcoll.08-H501	x	*Lycaenidae*	*Callophrys*	*rubi*	Duron, La Alcarria	Guadalajara	Castilla-La Mancha	Spain	725
KP870964	EZSPC219-09	RVcoll.08-L428	x	*Lycaenidae*	*Callophrys*	*rubi*	Rius de Cerdanya	Lleida	Catalonia	Spain	1273
KP870933	EZSPM988-12	Rvcoll.12-L873	x	*Lycaenidae*	*Callophrys*	*rubi*	Rafael Vell, Mao, Menorca	Menorca	Balearic Islands	Spain	
KP870570	EZSPM989-12	Rvcoll.12-L874	x	*Lycaenidae*	*Callophrys*	*rubi*	Rafael Vell, Mao, Menorca	Menorca	Balearic Islands	Spain	
KP871047	EZSPM990-12	Rvcoll.12-L875	x	*Lycaenidae*	*Callophrys*	*rubi*	Rafael Vell, Mao, Menorca	Menorca	Balearic Islands	Spain	
KP870678	EZSPM991-12	Rvcoll.12-L876	x	*Lycaenidae*	*Callophrys*	*rubi*	Rafael Vell, Mao, Menorca	Menorca	Balearic Islands	Spain	
GU675781	EZSPM247-09	RVcoll.08-R459	x	*Lycaenidae*	*Callophrys*	*rubi*	Oia, Pedra Rubia-Mougas	Pontevedra	Galicia	Spain	40
GU676505	EZSPN635-09	RVcoll.08-J746	x	*Lycaenidae*	*Callophrys*	*rubi*	Ruta de la Sima, El Vallecillo	Teruel	Aragon	Spain	1531
GU676679	EZSPN337-09	RVcoll.08-H269	x	*Lycaenidae*	*Callophrys*	*rubi*	Mequinenza	Zaragoza	Aragon	Spain	129
GU655023	FBLMT405-09	BC ZSM Lep 24965		*Hesperiidae*	*Carcharodus*	*alceae*	Kelheim, Lehnberg	Niederbayern	Bavaria	Germany	395
HQ004161	EZROM829-08	RVcoll.08-M220		*Hesperiidae*	*Carcharodus*	*alceae*	3 Km SW of Lunca Corbului	Arges	Muntenia	Romania	235
HQ004156	EZROM305-08	RV-07-E667		*Hesperiidae*	*Carcharodus*	*alceae*	Dealul Istrita, Breaza	Buzau	Muntenia	Romania	525
HQ004157	EZROM300-08	RV-07-E662		*Hesperiidae*	*Carcharodus*	*alceae*	Cluj-Napoca, dealul Bogomaia, Fanatele Clujului	Cluj	Transylvania	Romania	480
HQ004165	EZROM073-08	RV-07-D139		*Hesperiidae*	*Carcharodus*	*alceae*	Dumbraveni forest	Constanta	Dobrogea	Romania	80
HQ004158	EZROM826-08	RVcoll.08-M261		*Hesperiidae*	*Carcharodus*	*alceae*	road E70, (Gura Vaii) Portile de Fier	Mehedinti	Oltenia	Romania	50
HQ004163	EZROM077-08	RV-07-E531		*Hesperiidae*	*Carcharodus*	*alceae*	Slaveni	Olt	Oltenia	Romania	65
HQ004155	EZROM611-08	RV-07-E523		*Hesperiidae*	*Carcharodus*	*alceae*	2 km N of Cilieni	Olt	Oltenia	Romania	50
HQ004164	EZROM074-08	RV-07-E428		*Hesperiidae*	*Carcharodus*	*alceae*	3 km E of Slava Rusa (Babadag forest)	Tulcea	Dobrogea	Romania	130
HQ004159	EZROM827-08	RVcoll.08-M413		*Hesperiidae*	*Carcharodus*	*alceae*	Dealul Consul	Tulcea	Dobrogea	Romania	80
HQ004160	EZROM828-08	RVcoll.08-M430		*Hesperiidae*	*Carcharodus*	*alceae*	Babadag forest (10 Km S of Babadag)	Tulcea	Dobrogea	Romania	115
HQ004162	EZROM837-08	RVcoll.08-M402		*Hesperiidae*	*Carcharodus*	*alceae*	Macin Mts. (3 Km S of Jijila)	Tulcea	Dobrogea	Romania	230
GU676827	EZSPN107-09	RVcoll.06-A254	x	*Hesperiidae*	*Carcharodus*	*alceae*	Laguna de San Juan, Chinchon		Comunidad de Madrid	Spain	
GU676992	EZSPN229-09	RVcoll.07-W032	x	*Hesperiidae*	*Carcharodus*	*alceae*	Armada, Lena		Asturias	Spain	

GenBank accession number	BOLD Process ID	Sample ID	Used in the Iberian dataset	Family	Genus	Species	Exact Site	Region	State/Province	Country	Alt. (m)
HM901274	EZSPC1071-10	RVcoll.08-L283	x	*Hesperiidae*	*Carcharodus*	*alceae*	Plano de Petracos, Castell de Castells	Alicante	Comunidad Valenciana	Spain	497
GU676172	EZSPN984-09	RVcoll.08-M024	x	*Hesperiidae*	*Carcharodus*	*alceae*	Alicante	Alicante	Comunidad Valenciana	Spain	11
KP870581	EZROM711-08	RV-06-H853	x	*Hesperiidae*	*Carcharodus*	*alceae*	Vallforners, Canoves	Barcelona	Catalonia	Spain	650
KP870719	EZSPC194-09	RVcoll.06-G440	x	*Hesperiidae*	*Carcharodus*	*alceae*	El Prat de la Collada, El Brull, Osona	Barcelona	Catalonia	Spain	835
KP870226	EZSPC195-09	RVcoll.06-G462	x	*Hesperiidae*	*Carcharodus*	*alceae*	Sallent, Bages	Barcelona	Catalonia	Spain	320
KP871152	EZSPC232-09	RVcoll.08-M714	x	*Hesperiidae*	*Carcharodus*	*alceae*	Delta de Llobregat	Barcelona	Catalonia	Spain	2
GU676605	EZSPN433-09	RVcoll.08-H611	x	*Hesperiidae*	*Carcharodus*	*alceae*	El Gastor	Cadiz	Andalusia	Spain	400
HQ562785	EZSPN209-09	RVcoll.07-F061	x	*Hesperiidae*	*Carcharodus*	*alceae*	10 km S. of Morella	Castellon	Comunidad Valenciana	Spain	900
KP870860	EZSPM936-12	Rvcoll.12-M670	x	*Hesperiidae*	*Carcharodus*	*alceae*	Rio Jandula, Mestanza	Ciudad Real	Castilla-La Mancha	Spain	407
KP870857	EZSPC203-09	RVcoll.07-C498	x	*Hesperiidae*	*Carcharodus*	*alceae*	Meranges	Girona	Catalonia	Spain	1555
KP870414	EULEP026-14	07-D759	x	*Hesperiidae*	*Carcharodus*	*alceae*	Salobreña	Granada	Andalusia	Spain	
GU676634	EZSPN413-09	RVcoll.08-H530	x	*Hesperiidae*	*Carcharodus*	*alceae*	Gualda, La Alcarria	Guadalajara	Castilla-La Mancha	Spain	730
GU676800	EZSPN136-09	RVcoll.06-G495	x	*Hesperiidae*	*Carcharodus*	*alceae*	Barranco de Valcuerna, Candasnos	Huesca	Aragon	Spain	210
GU676757	EZSPN281-09	RVcoll.07-W216	x	*Hesperiidae*	*Carcharodus*	*alceae*	Laguarta, Laguarta	Huesca	Aragon	Spain	1065
GU675870	EZSPM368-09	RVcoll.08-J837	x	*Hesperiidae*	*Carcharodus*	*alceae*	La Una	Leon	Castilla y Leon	Spain	
GU676806	EZSPN126-09	RVcoll.06-A315	x	*Hesperiidae*	*Carcharodus*	*alceae*	Sierra de Ayllon, Montejo de Tiermes	Soria	Castilla y Leon	Spain	
GU675878	EZSPM360-09	RVcoll.08-R026	x	*Hesperiidae*	*Carcharodus*	*alceae*	Sant Vicent, Lliria	Valencia	Comunidad Valenciana	Spain	
GU676941	EZSPN537-09	RVcoll.08-J023	x	*Hesperiidae*	*Carcharodus*	*alceae*	Regajo, Sinarcas	Valencia	Comunidad Valenciana	Spain	
GU676946	EZSPN545-09	RVcoll.08-J046	x	*Hesperiidae*	*Carcharodus*	*alceae*	Utiel, Utiel	Valencia	Comunidad Valenciana	Spain	
GU676703	EZSPN332-09	RVcoll.08-H257	x	*Hesperiidae*	*Carcharodus*	*alceae*	Mequinenza	Zaragoza	Aragon	Spain	129
JN277556	EZSPN1046-11	Rvcoll.08-L174	x	*Hesperiidae*	*Carcharodus*	*baeticus*	Font del Regatxal, Vilafranca	Castellon	Comunidad Valenciana	Spain	1361
KP870838	EZSPM955-12	Rvcoll.12-M706	x	*Hesperiidae*	*Carcharodus*	*baeticus*	Alrededores San Lorenzo de Calatrava	Ciudad Real	Castilla-La Mancha	Spain	749
KP870220	EZSPM121-09	RVcoll.08-P607	x	*Hesperiidae*	*Carcharodus*	*baeticus*	Piscifactoria, Laguna de Una, Una	Cuenca	Castilla-La Mancha	Spain	1219
KP870603	EZSPN1031-11	Rvcoll.06-A270	x	*Hesperiidae*	*Carcharodus*	*baeticus*	Sierra de los Barrancos, Cuenca	Cuenca	Castilla-La Mancha	Spain	1447
GU676778	EZSPN158-09	RVcoll.06-J003	x	*Hesperiidae*	*Carcharodus*	*baeticus*	Casa de la Herreria, Huelamo	Cuenca	Castilla-La Mancha	Spain	1270
HM901811	EZSPC976-10	RVcoll.09-V453	x	*Hesperiidae*	*Carcharodus*	*baeticus*	Loma de Lastonares (Sierra Nevada)	Granada	Andalusia	Spain	2050
HM901824	EZSPC989-10	RVcoll.09-V456	x	*Hesperiidae*	*Carcharodus*	*baeticus*	Puerto del Lobo, Pista de las Alpujarras	Granada	Andalusia	Spain	1950
HM901804	EZSPC969-10	RVcoll.08-L911	x	*Hesperiidae*	*Carcharodus*	*baeticus*	Gualda	Guadalajara	Castilla-La Mancha	Spain	730
GU676796	EZSPN141-09	RVcoll.06-G506	x	*Hesperiidae*	*Carcharodus*	*baeticus*	Sierra de Alcubierre	Huesca	Aragon	Spain	600
KP870820	EULEP257-14	12-Q527	x	*Hesperiidae*	*Carcharodus*	*baeticus*	Camarassa, La Noguera	Lleida	Catalonia	Spain	438
GU676344	EZSPN806-09	RVcoll.08-L470	x	*Hesperiidae*	*Carcharodus*	*baeticus*	Ivars de Noguera	Lleida	Catalonia	Spain	374
KP870664	FBLMX123-11	BC ZSM Lep 37604		*Hesperiidae*	*Carcharodus*	*floccifera*	Grassau, Egerndacher Filz	Oberbayern	Bavaria	Germany	530
HQ004166	EZROM075-08	RV-07-D306		*Hesperiidae*	*Carcharodus*	*floccifera*	Dumbrava Vadului, Vad	Brasov	Transylvania	Romania	495
HQ004168	EZRMN310-08	RVcoll.08-M794		*Hesperiidae*	*Carcharodus*	*floccifera*	Feleacu, Cluj-Napoca	Cluj	Transylvania	Romania	700
HQ004170	EZROM325-08	RV-08-A003		*Hesperiidae*	*Carcharodus*	*floccifera*	Cluj-Napoca, dealul Bogomaia, Fanatele Clujului	Cluj	Transylvania	Romania	480
HQ004167	EZRMN347-08	RVcoll.07-D614		*Hesperiidae*	*Carcharodus*	*floccifera*	Canaraua Fetei (Baneasa)	Constanta	Dobrogea	Romania	20
HQ004169	EZROM613-08	RV-07-D016		*Hesperiidae*	*Carcharodus*	*floccifera*	Canaraua Fetei (Baneasa)	Constanta	Dobrogea	Romania	15
HQ004174	EZROM839-08	RVcoll.08-M454		*Hesperiidae*	*Carcharodus*	*floccifera*	Canaraua Fetei (Baneasa)	Constanta	Dobrogea	Romania	20
HQ004171	EZROM830-08	RVcoll.08-M583		*Hesperiidae*	*Carcharodus*	*floccifera*	Gheorgheni, Valea Belchia	Harghita	Transylvania	Romania	1000
HQ004172	EZROM831-08	RVcoll.08-M588		*Hesperiidae*	*Carcharodus*	*floccifera*	Gheorgheni, Valea Belchia	Harghita	Transylvania	Romania	1000
HQ004173	EZROM832-08	RVcoll.08-M605		*Hesperiidae*	*Carcharodus*	*floccifera*	1 Km E of Gheorgheni	Harghita	Transylvania	Romania	900
KP870961	EZROM712-08	RV-07-F301	x	*Hesperiidae*	*Carcharodus*	*floccifera*	Viladrau	Barcelona	Catalonia	Spain	720
GU676750	EZSPN282-09	RVcoll.07-W218	x	*Hesperiidae*	*Carcharodus*	*floccifera*	Casa de la Herreria, Huelamo	Cuenca	Castilla-La Mancha	Spain	1270
GU676751	EZSPN283-09	RVcoll.07-W219	x	*Hesperiidae*	*Carcharodus*	*floccifera*	P.K. 43 CM-2106, Huelamo	Cuenca	Castilla-La Mancha	Spain	1237
GU675663	EZSPC204-09	RVcoll.07-C626	x	*Hesperiidae*	*Carcharodus*	*floccifera*	Meranges	Girona	Catalonia	Spain	1555
GU676756	EZSPN280-09	RVcoll.07-W215	x	*Hesperiidae*	*Carcharodus*	*floccifera*	Laguarta, Rio Alcanadre	Huesca	Aragon	Spain	1216
KP870699	EZSPC221-09	RVcoll.08-L436	x	*Hesperiidae*	*Carcharodus*	*floccifera*	Baltarga, La Cerdenya	Lleida	Catalonia	Spain	1121
KP870395	EZSPC265-09	RVcoll.08-R118	x	*Hesperiidae*	*Carcharodus*	*floccifera*	Salt de Pitx, Vall d`Aran	Lleida	Catalonia	Spain	1570

GenBank accession number	BOLD Process ID	Sample ID	Used in the Iberian dataset	Family	Genus	Species	Exact Site	Region	State/Province	Country	Alt. (m)
HM901772	EZSPC935-10	RVcoll.07-W293	x	*Hesperiidae*	*Carcharodus*	*floccifera*	Val de Barrados (Subida Salt del Pitx), Vall d`Aran	Lleida	Catalonia	Spain	1190
HQ004178	EZROM833-08	RVcoll.08-M521		*Hesperiidae*	*Carcharodus*	*lavatherae*	Apuseni Mts., Rimetea (Piatra Secuiului)	Alba	Transylvania	Romania	850
HQ004177	EZROM834-08	Rvcoll.08-M711		*Hesperiidae*	*Carcharodus*	*lavatherae*	Rimetea, Piatra Secuiului	Alba	Transylvania	Romania	850
HQ004175	EZROM304-08	RV-07-E666		*Hesperiidae*	*Carcharodus*	*lavatherae*	Suatu	Cluj	Transylvania	Romania	380
HQ004176	EZROM311-08	RV-07-E673		*Hesperiidae*	*Carcharodus*	*lavatherae*	Cluj-Napoca, dealul Bogomaia, Fanatele Clujului	Cluj	Transylvania	Romania	480
HM901386	EZSPC1186-10	RVcoll.09-T176	x	*Hesperiidae*	*Carcharodus*	*lavatherae*	Rio Duje, Sotres		Asturias	Spain	1216
KP870251	EZROM713-08	RV-06-G459	x	*Hesperiidae*	*Carcharodus*	*lavatherae*	La Sala, El Brull	Barcelona	Catalonia	Spain	835
HM901406	EZSPC1207-10	RVcoll.09-V475	x	*Hesperiidae*	*Carcharodus*	*lavatherae*	Sierra de la Sagra	Granada	Andalusia	Spain	1745
GU676217	EZSPN940-09	RVcoll.08-L912	x	*Hesperiidae*	*Carcharodus*	*lavatherae*	Gualda	Guadalajara	Castilla-La Mancha	Spain	730
GU676160	EZSPN996-09	RVcoll.08-M071	x	*Hesperiidae*	*Carcharodus*	*lavatherae*	San Andres del Rey, La Alcarria	Guadalajara	Castilla-La Mancha	Spain	1018
GU676820	EZSPN117-09	RVcoll.06-A289	x	*Hesperiidae*	*Carcharodus*	*lavatherae*	Rio Guarga, Laguarta	Huesca	Aragon	Spain	1140
KP870247	EZSPC220-09	RVcoll.08-L435	x	*Hesperiidae*	*Carcharodus*	*lavatherae*	Baltarga, La Cerdenya	Lleida	Catalonia	Spain	1121
KP870647	EZSPC259-09	RVcoll.08-P365	x	*Hesperiidae*	*Carcharodus*	*lavatherae*	Pas de Perves	Lleida	Catalonia	Spain	1246
KP870223	EZSPC263-09	RVcoll.08-R111	x	*Hesperiidae*	*Carcharodus*	*lavatherae*	Salt de Pitx, Vall d`Aran	Lleida	Catalonia	Spain	1570
HM901770	EZSPC933-10	RVcoll.06-A255.1	x	*Hesperiidae*	*Carcharodus*	*lavatherae*	Sierra de Ayllon, Grado del Pico	Segovia	Castilla y Leon	Spain	
HM901207	EZSPC1003-10	RVcoll.08-H286	x	*Hesperiidae*	*Carcharodus*	*lavatherae*	Els Motllats, Vilaplana, Baix Camp	Tarragona	Catalonia	Spain	980
KP871147	EZSPC210-09	RVcoll.08-H285	x	*Hesperiidae*	*Carcharodus*	*lavatherae*	Els Motllats, Vilaplana, Baix Camp	Tarragona	Catalonia	Spain	980
HM901227	EZSPC1023-10	RVcoll.08-J017	x	*Hesperiidae*	*Carcharodus*	*lavatherae*	Los Herreros, Cortes de Pallas	Valencia	Comunidad Valenciana	Spain	690
HQ004182	EZROM080-08	RV-07-D132		*Hesperiidae*	*Carcharodus*	*orientalis*	Dumbraveni forest	Constanta	Dobrogea	Romania	80
HQ004184	EZROM665-08	RV-07-D119		*Hesperiidae*	*Carcharodus*	*orientalis*	Dumbraveni forest	Constanta	Dobrogea	Romania	80
HQ004187	EZROM838-08	RVcoll.08-M437		*Hesperiidae*	*Carcharodus*	*orientalis*	Gura Dobrogei reserve	Constanta	Dobrogea	Romania	90
HQ004179	EZRMN331-08	RVcoll.08-M784		*Hesperiidae*	*Carcharodus*	*orientalis*	Garboavele forest	Galati	Moldavia	Romania	90
HQ004185	EZROM835-08	RVcoll.08-M386		*Hesperiidae*	*Carcharodus*	*orientalis*	Garboavele forest	Galati	Moldavia	Romania	90
HQ004186	EZROM836-08	RVcoll.08-M391		*Hesperiidae*	*Carcharodus*	*orientalis*	Garboavele forest	Galati	Moldavia	Romania	90
HQ004180	EZROM078-08	RV-07-D055		*Hesperiidae*	*Carcharodus*	*orientalis*	Babadag forest	Tulcea	Dobrogea	Romania	115
HQ004181	EZROM079-08	RV-07-D074		*Hesperiidae*	*Carcharodus*	*orientalis*	Greci massif, izvorul Esitul Sufletului	Tulcea	Dobrogea	Romania	145
HQ004183	EZROM664-08	RV-07-D059		*Hesperiidae*	*Carcharodus*	*orientalis*	8 Km S of Babadag (Babadag forest)	Tulcea	Dobrogea	Romania	115
KP870456	EULEP157-14	11-D637	x	*Hesperiidae*	*Carcharodus*	*tripolina*	Nave, Monchique		Algarve	Portugal	
KP870876	EULEP158-14	11-D638	x	*Hesperiidae*	*Carcharodus*	*tripolina*	Marmelete, Monchique		Algarve	Portugal	
KP870816	EULEP159-14	11-D639	x	*Hesperiidae*	*Carcharodus*	*tripolina*	Caldas de Monchique, Monchique		Algarve	Portugal	
KP870479	EZSPM1012-12	Rvcoll.12-L058	x	*Hesperiidae*	*Carcharodus*	*tripolina*	Marmelete, Monchique		Algarve	Portugal	400
KP871114	EZSPM673-12	RVcoll.12-L544	x	*Hesperiidae*	*Carcharodus*	*tripolina*	Porches, Lagoa		Algarve	Portugal	
KP870382	EZSPM671-12	RVcoll.12-L054	x	*Hesperiidae*	*Carcharodus*	*tripolina*	Poligono Industrial Campamento, La Linea, Cadiz	Cadiz	Andalusia	Spain	20
KP870837	EZSPN1096-11	Rvcoll.10-A015	x	*Hesperiidae*	*Carcharodus*	*tripolina*	Arroyo del Prior, Algeciras, Cadiz	Cadiz	Andalusia	Spain	50
GU676920	EZSPN517-09	RVcoll.08-H982	x	*Hesperiidae*	*Carcharodus*	*tripolina*	La Linea de la Concepcion, Sierra de la Carbonera	Cadiz	Andalusia	Spain	200
GU676923	EZSPN518-09	RVcoll.08-H983	x	*Hesperiidae*	*Carcharodus*	*tripolina*	La Linea de la Concepcion, Sierra de la Carbonera	Cadiz	Andalusia	Spain	200
HM393209	GWORA2489-09	BC ZSM Lep 30701		*Hesperiidae*	*Carterocephalus*	*palaemon*	Hausmetting	Niederbayern	Bavaria	Germany	370
GU655022	FBLMT881-09	BC ZSM Lep 25441		*Hesperiidae*	*Carterocephalus*	*palaemon*	Schoengeising	Oberbayern	Bavaria	Germany	560
HQ563564	FBLMW324-10	BC ZSM Lep 37425		*Hesperiidae*	*Carterocephalus*	*palaemon*	Koednitzer Weinleite	Oberfranken	Bavaria	Germany	385
JF859976	PHLAC400-10	TLMF Lep 02435		*Hesperiidae*	*Carterocephalus*	*palaemon*	Ritten/ Obergruenwald		South Tyrol	Italy	1750
HQ004189	EZROM082-08	RV-07-D244		*Hesperiidae*	*Carterocephalus*	*palaemon*	Dumbrava Vadului, Vad	Brasov	Transylvania	Romania	495
HQ004192	EZROM840-08	RVcoll.07-D290		*Hesperiidae*	*Carterocephalus*	*palaemon*	Dumbrava Vadului, Vad	Brasov	Transylvania	Romania	495
HQ004190	EZROM083-08	RV-07-D589		*Hesperiidae*	*Carterocephalus*	*palaemon*	Teregova	Caras-Severin	Banat	Romania	385
HQ004191	EZROM666-08	RV-06-K682		*Hesperiidae*	*Carterocephalus*	*palaemon*	Badeni	Cluj	Transylvania	Romania	440
HQ004193	EZROM841-08	RVcoll.08-M339		*Hesperiidae*	*Carterocephalus*	*palaemon*	1 Km E of Gheorgheni	Harghita	Transylvania	Romania	900
HQ004194	EZROM842-08	RVcoll.08-M340		*Hesperiidae*	*Carterocephalus*	*palaemon*	1 Km E of Gheorgheni	Harghita	Transylvania	Romania	900
KP870642	EULEP023-14	07-D557		*Hesperiidae*	*Carterocephalus*	*palaemon*	Rohia	Maramures	Maramureş	Romania	360
HQ004188	EZROM081-08	RV-07-D556		*Hesperiidae*	*Carterocephalus*	*palaemon*	sat Rohia	Maramures	Maramures	Romania	360
GU676997	EZSPN225-09	RVcoll.07-W027	x	*Hesperiidae*	*Carterocephalus*	*palaemon*	Armada, Lena		Asturias	Spain	
GU676994	EZSPN226-09	RVcoll.07-W028	x	*Hesperiidae*	*Carterocephalus*	*palaemon*	Armada, Lena		Asturias	Spain	
GU676995	EZSPN227-09	RVcoll.07-W029	x	*Hesperiidae*	*Carterocephalus*	*palaemon*	Armada, Lena		Asturias	Spain	

GenBank accession number	BOLD Process ID	Sample ID	Used in the Iberian dataset	Family	Genus	Species	Exact Site	Region	State/Province	Country	Alt. (m)
HQ562786	EZSPN228-09	RVcoll.07-W030	x	*Hesperiidae*	*Carterocephalus*	*palaemon*	Armada, Lena		Asturias	Spain	
KP870406	EZSPC209-09	RVcoll.07-W142	x	*Hesperiidae*	*Carterocephalus*	*palaemon*	Bassa d`Arres, Vall d`Aran	Lleida	Catalonia	Spain	1568
KP870774	EZSPC235-09	RVcoll.08-M918	x	*Hesperiidae*	*Carterocephalus*	*palaemon*	Arros, Vall d`Aran	Lleida	Catalonia	Spain	906
GU688457	GWORO793-09	BC ZSM Lep 30485		*Lycaenidae*	*Celastrina*	*argiolus*	Fischerhaeuser Isarauen / MUC		Bavaria	Germany	482
GU707100	FBLMU439-09	BC ZSM Lep 27089		*Lycaenidae*	*Celastrina*	*argiolus*	Diessen, Huebschenried	Oberbayern	Bavaria	Germany	650
JF415691	GWORK518-09	BC ZSM Lep 21848		*Lycaenidae*	*Celastrina*	*argiolus*	Diessen	Oberbayern	Bavaria	Germany	600
GU686877	GWORL416-09	BC ZSM Lep 22318		*Lycaenidae*	*Celastrina*	*argiolus*	Oberschleissheim, MW 20	Oberbayern	Bavaria	Germany	480
HQ004200	EZROM088-08	RV-07-E384		*Lycaenidae*	*Celastrina*	*argiolus*	Motel Dumbrava (Cerna Valley)	Caras-Severin	Banat	Romania	290
HQ004203	EZROM084-08	RV-06-K579		*Lycaenidae*	*Celastrina*	*argiolus*	Badeni	Cluj	Transylvania	Romania	440
HQ004202	EZROM085-08	RV-06-K581		*Lycaenidae*	*Celastrina*	*argiolus*	Badeni	Cluj	Transylvania	Romania	440
HQ004196	EZROM846-08	RVcoll.08-M444		*Lycaenidae*	*Celastrina*	*argiolus*	Canaraua Fetei (Baneasa)	Constanta	Dobrogea	Romania	20
HQ004201	EZROM086-08	RV-07-D959		*Lycaenidae*	*Celastrina*	*argiolus*	Valea Mare	Dambovita	Muntenia	Romania	225
HQ004199	EZROM087-08	RV-07-D977		*Lycaenidae*	*Celastrina*	*argiolus*	4 km west of I.L. Caragiale	Dambovita	Muntenia	Romania	270
HQ004197	EZROM843-08	RVcoll.07-C418		*Lycaenidae*	*Celastrina*	*argiolus*	4 km west of I.L. Caragiale	Dambovita	Muntenia	Romania	270
HQ004198	EZROM844-08	RVcoll.07-D573		*Lycaenidae*	*Celastrina*	*argiolus*	Spini	Hunedoara	Transylvania	Romania	240
HQ004195	EZROM845-08	RVcoll.07-D932		*Lycaenidae*	*Celastrina*	*argiolus*	Otasau Valley, Buila-Vanturarita	Valcea	Oltenia	Romania	515
GU675745	EZSPM135-09	RVcoll.08-P645	x	*Lycaenidae*	*Celastrina*	*argiolus*	Valle (Valle-Zurea), Lena		Asturias	Spain	662
GU677052	EZSPN354-09	RVcoll.08-H327	x	*Lycaenidae*	*Celastrina*	*argiolus*	Ambite, Ambite		Comunidad de Madrid	Spain	
GU675789	EZSPM249-09	RVcoll.08-R461	x	*Lycaenidae*	*Celastrina*	*argiolus*	Ames, Novais	A Coruna	Galicia	Spain	160
JN827873	EZSPC200-09	RVcoll.06-H841	x	*Lycaenidae*	*Celastrina*	*argiolus*	Vallforners, Canoves, Valles Oriental	Barcelona	Catalonia	Spain	650
JN827877	EZSPC280-09	RVcoll.08-R285	x	*Lycaenidae*	*Celastrina*	*argiolus*	Masia del Plans, Vidra, La Garrotxa	Barcelona	Catalonia	Spain	1200
KP870882	EZSPM1030-12	Rvcoll.12-M780	x	*Lycaenidae*	*Celastrina*	*argiolus*	Merendero de los Horcones, Navatrasierra	Caceres	Extremadura	Spain	664
GU676891	EZSPN486-09	RVcoll.08-H923	x	*Lycaenidae*	*Celastrina*	*argiolus*	Hervas, Carretera Hervas-La Garganta	Caceres	Extremadura	Spain	940
GU676333	EZSPN819-09	RVcoll.08-L628	x	*Lycaenidae*	*Celastrina*	*argiolus*	Algeciras, Arroyo del Prior	Cadiz	Andalusia	Spain	50
KP870270	EZSPM939-12	Rvcoll.12-M673	x	*Lycaenidae*	*Celastrina*	*argiolus*	Rio Jandula, Mestanza	Ciudad Real	Castilla-La Mancha	Spain	407
JN827875	EZSPC240-09	RVcoll.08-P034	x	*Lycaenidae*	*Celastrina*	*argiolus*	La Valira (near Urus), Cerdanya	Girona	Catalonia	Spain	1300
GU676844	EZSPN092-09	RVcoll.06-A021	x	*Lycaenidae*	*Celastrina*	*argiolus*	Mecina Bombaron, Alpujarras	Granada	Andalusia	Spain	1150
GU676629	EZSPN408-09	RVcoll.08-H504	x	*Lycaenidae*	*Celastrina*	*argiolus*	Duron, La Alcarria	Guadalajara	Castilla-La Mancha	Spain	725
KP870854	EZSPM1045-12	Rvcoll.12-N000	x	*Lycaenidae*	*Celastrina*	*argiolus*	Sant Llorenc de Balafia, Ibiza	Ibiza	Balearic Islands	Spain	90.96
GU675961	EZSPM080-09	RVcoll.08-J815	x	*Lycaenidae*	*Celastrina*	*argiolus*	Compludo	Leon	Castilla y Leon	Spain	
JN827874	EZSPC212-09	RVcoll.08-J338	x	*Lycaenidae*	*Celastrina*	*argiolus*	Coll de Comiols, Pallars Jussa	Lleida	Catalonia	Spain	1130
GU676042	EZSPM038-09	RVcoll.08-P243	x	*Lycaenidae*	*Celastrina*	*argiolus*	Puig d`en Galileu	Mallorca	Balearic Islands	Spain	1071
GU675964	EZSPM055-09	RVcoll.08-P279	x	*Lycaenidae*	*Celastrina*	*argiolus*	S`Albufera	Mallorca	Balearic Islands	Spain	4
KP870962	EZSPM1001-12	Rvcoll.12-L887	x	*Lycaenidae*	*Celastrina*	*argiolus*	Sant Antoni de Ruma, Ferreries, Menorca	Menorca	Balearic Islands	Spain	
KP870701	EZSPM994-12	Rvcoll.12-L880	x	*Lycaenidae*	*Celastrina*	*argiolus*	Rafael Vell, Mao, Menorca	Menorca	Balearic Islands	Spain	
GU676729	EZSPN309-09	RVcoll.08-H116	x	*Lycaenidae*	*Celastrina*	*argiolus*	Na Vermella, Mao	Menorca	Balearic Islands	Spain	
GU676712	EZSPN325-09	RVcoll.08-H196	x	*Lycaenidae*	*Celastrina*	*argiolus*	Es Grau, Mao	Menorca	Balearic Islands	Spain	
GU675790	EZSPM270-09	RVcoll.08-R487	x	*Lycaenidae*	*Celastrina*	*argiolus*	Lobios, Camino de Sa a Portela de Homen	Ourense	Galicia	Spain	1100
JN827876	EZSPC251-09	RVcoll.08-P323	x	*Lycaenidae*	*Celastrina*	*argiolus*	La Riba, Alt Camp	Tarragona	Catalonia	Spain	335
GU675747	EZSPM175-09	RVcoll.08-R040	x	*Lycaenidae*	*Celastrina*	*argiolus*	Pena Judia, Chulilla	Valencia	Comunidad Valenciana	Spain	288
JN264880	EZSPC1334-10	RVcoll.130209KL7	x	*Nymphalidae*	*Charaxes*	*jasius*	Cadalso		Comunidad de Madrid	Spain	
HM901320	EZSPC1119-10	RVcoll.08-P463	x	*Nymphalidae*	*Charaxes*	*jasius*	Candeleda	Avila	Castilla y Leon	Spain	450
KP870282	EZSPC844-10	RVcoll.08-P464.1	x	*Nymphalidae*	*Charaxes*	*jasius*	Candeleda	Avila	Castilla y Leon	Spain	450
HM901827	EZSPC992-10	RVcoll.07-F534.1	x	*Nymphalidae*	*Charaxes*	*jasius*	Granja Viader, Cardedeu, Valles Oriental	Barcelona	Catalonia	Spain	195
HM901828	EZSPC993-10	RVcoll.07-F535	x	*Nymphalidae*	*Charaxes*	*jasius*	Granja Viader, Cardedeu, Valles Oriental	Barcelona	Catalonia	Spain	195
GU675777	EZSPM204-09	RVcoll.08-R314	x	*Nymphalidae*	*Charaxes*	*jasius*	Els Refugis del Montseny, St. Pere de Vilamajor, Valles Oriental	Barcelona	Catalonia	Spain	630
GU676065	EZSPM420-09	Rvcoll.09-V843	x	*Nymphalidae*	*Charaxes*	*jasius*	Coll de Banyuls, Alt Emporda	Girona	Catalonia	Spain	
GU675954	EZSPM040-09	RVcoll.08-P248	x	*Nymphalidae*	*Charaxes*	*jasius*	Santa Maria	Mallorca	Balearic Islands	Spain	181
GU675778	EZSPM209-09	RVcoll.08-R329	x	*Nymphalidae*	*Charaxes*	*jasius*	Coanegra, Sta. Maria del cami	Mallorca	Balearic Islands	Spain	125
GU675775	EZSPM210-09	RVcoll.08-R330	x	*Nymphalidae*	*Charaxes*	*jasius*	Coanegra, Sta. Maria del cami	Mallorca	Balearic Islands	Spain	125
KP871071	EZSPM1008-12	Rvcoll.12-L898	x	*Nymphalidae*	*Charaxes*	*jasius*	Sant Antoni de Ruma, Ferreries, Menorca	Menorca	Balearic Islands	Spain	
GU676853	EZSPN079-09	SMcoll.020808CB85	x	*Nymphalidae*	*Charaxes*	*jasius*	Embalse de Benageber, Benageber	Valencia	Comunidad Valenciana	Spain	559

GenBank accession number	BOLD Process ID	Sample ID	Used in the Iberian dataset	Family	Genus	Species	Exact Site	Region	State/Province	Country	Alt. (m)
KP871075	EULEP175-14	11-J337		*Nymphalidae*	*Chazara*	*briseis*	Suatu	Cluj	Transylvania	Romania	350-400
HQ004207	EZROM089-08	RV-06-V673		*Nymphalidae*	*Chazara*	*briseis*	Suatu	Cluj	Transylvania	Romania	380
HQ004208	EZROM090-08	RV-06-V675		*Nymphalidae*	*Chazara*	*briseis*	Suatu	Cluj	Transylvania	Romania	380
HQ004204	EZROM667-08	RV-06-V674		*Nymphalidae*	*Chazara*	*briseis*	Suatu	Cluj	Transylvania	Romania	380
HQ004206	EZROM847-08	RVcoll.06-V672		*Nymphalidae*	*Chazara*	*briseis*	Suatu	Cluj	Transylvania	Romania	380
HQ004205	EZROM848-08	RVcoll.06-V676		*Nymphalidae*	*Chazara*	*briseis*	Suatu	Cluj	Transylvania	Romania	380
HQ004209	EZROM958-08	RVcoll.06-V677.1		*Nymphalidae*	*Chazara*	*briseis*	Suatu	Cluj	Transylvania	Romania	380
GU676205	EZSPN957-09	RVcoll.08-L954	x	*Nymphalidae*	*Chazara*	*briseis*	San Andres		Cantabria	Spain	988
KP870519	EZROM714-08	RV-06-V746	x	*Nymphalidae*	*Chazara*	*briseis*	Coll de Torn, Saldes	Barcelona	Catalonia	Spain	1910
KP870354	EZSPC216-09	RVcoll.08-J400	x	*Nymphalidae*	*Chazara*	*briseis*	Matagalls, Montseny, Valles Oriental	Barcelona	Catalonia	Spain	1550
HM901826	EZSPC991-10	RVcoll.07-F080.1	x	*Nymphalidae*	*Chazara*	*briseis*	4 km E. of Olocau del Rey	Castellon	Comunidad Valenciana	Spain	1005
GU676747	EZSPN287-09	RVcoll.07-W240	x	*Nymphalidae*	*Chazara*	*briseis*	Casa de la Herreria, Huelamo	Cuenca	Castilla-La Mancha	Spain	1270
HM901502	EZSPC1368-10	RVcoll.09-X060	x	*Nymphalidae*	*Chazara*	*briseis*	Barranc de Montmalus, La Cerdanya	Girona	Catalonia	Spain	1340
KP870822	EZSPC238-09	RVcoll.08-P015	x	*Nymphalidae*	*Chazara*	*briseis*	Torre de Riu, Ribera d`Alt, Cerdanya	Girona	Catalonia	Spain	1180
KP871098	EZSPC239-09	RVcoll.08-P026	x	*Nymphalidae*	*Chazara*	*briseis*	Talltendre, Cerdanya	Girona	Catalonia	Spain	1571
HM901334	EZSPC1134-10	RVcoll.08-P716	x	*Nymphalidae*	*Chazara*	*briseis*	Alquife	Granada	Andalusia	Spain	1282
GU675722	EZSPM127-09	RVcoll.08-P622	x	*Nymphalidae*	*Chazara*	*briseis*	San Andres del Rey	Guadalajara	Castilla-La Mancha	Spain	1018
KP870808	EZSPC258-09	RVcoll.08-P363	x	*Nymphalidae*	*Chazara*	*briseis*	Pas de Perves	Lleida	Catalonia	Spain	1246
KP870747	EZSPM767-12	RVcoll.040611HZ50	x	*Nymphalidae*	*Chazara*	*briseis*	Fuentes Calientes	Teruel	Aragon	Spain	1273
GU677047	EZSPN755-09	RVcoll.08-L231	x	*Nymphalidae*	*Chazara*	*briseis*	Ruta de la Sima, El Vallecillo	Teruel	Aragon	Spain	1531
HM901478	EZSPC1318-10	RVcoll.130209KL92	x	*Nymphalidae*	*Chazara*	*briseis*	Cardete,Camporrobles	Valencia	Comunidad Valenciana	Spain	
HM901485	EZSPC1339-10	RVcoll.130209KL91	x	*Nymphalidae*	*Chazara*	*briseis*	Cardete,Camporrobles	Valencia	Comunidad Valenciana	Spain	
KP870627	EZSPM679-12	RVcoll.12-L035	x	*Nymphalidae*	*Chazara*	*prieuri*	Sierra de Caldereros, Hombrados, Guadalajara	Guadalajara	Castilla-La Mancha	Spain	1280
KP871045	EZSPM680-12	RVcoll.12-L036	x	*Nymphalidae*	*Chazara*	*prieuri*	Sierra de Caldereros, Hombrados, Guadalajara	Guadalajara	Castilla-La Mancha	Spain	1280
JN278863	EZSPC1305-10	RVcoll.130209KL2	x	*Nymphalidae*	*Chazara*	*prieuri*	Teruel	Teruel	Aragon	Spain	
JN278864	EZSPC1325-10	RVcoll.130209KL33	x	*Nymphalidae*	*Chazara*	*prieuri*	Albarracin,Albarracin	Teruel	Aragon	Spain	
HM901655	EZSPC805-10	RVcoll.09-X324	x	*Nymphalidae*	*Chazara*	*prieuri*	Albarracin	Teruel	Aragon	Spain	1300
KP871166	EZSPM674-12	RVcoll.08-J472	x	*Nymphalidae*	*Chazara*	*prieuri*	Prieuri Valley, Albarracin A-1512 km 25	Teruel	Aragon	Spain	1200
KP870410	EZSPM675-12	RVcoll.08-J473	x	*Nymphalidae*	*Chazara*	*prieuri*	Prieuri Valley, Albarracin A-1512 km 25	Teruel	Aragon	Spain	1200
KP870545	EZSPM676-12	RVcoll.08-J474	x	*Nymphalidae*	*Chazara*	*prieuri*	Prieuri Valley, Albarracin A-1512 km 25	Teruel	Aragon	Spain	1200
KP871013	EZSPM677-12	RVcoll.08-J475	x	*Nymphalidae*	*Chazara*	*prieuri*	Prieuri Valley, Albarracin A-1512 km 25	Teruel	Aragon	Spain	1200
KP870889	EZSPM678-12	RVcoll.08-J476	x	*Nymphalidae*	*Chazara*	*prieuri*	Prieuri Valley, Albarracin A-1512 km 25	Teruel	Aragon	Spain	1200
KP870526	EZSPM768-12	RVcoll.040611HZ54	x	*Nymphalidae*	*Chazara*	*prieuri*	Fuentes Calientes	Teruel	Aragon	Spain	1273
KP870895	EZSPM826-12	RVcoll.140611ZG87	x	*Nymphalidae*	*Chazara*	*prieuri*	Puerto de Sant Just, Escucha	Teruel	Aragon	Spain	1421
HM393203	GWORA2481-09	BC ZSM Lep 30693		*Nymphalidae*	*Coenonympha*	*arcania*	Thurasdorf	Niederbayern	Bavaria	Germany	440
KP870462	FBLMX208-11	BC ZSM Lep 50419		*Nymphalidae*	*Coenonympha*	*arcania*	Schneizlreuth, Unterjettenberg/Oberjettenberg	Oberbayern	Bavaria	Germany	650
HM910008	GWORT462-10	BC ZSM Lep 32148		*Nymphalidae*	*Coenonympha*	*arcania*	Ismaning, Fischerhaeuser	Oberbayern	Bavaria	Germany	482
HQ004211	EZROM103-08	RV-06-M941		*Nymphalidae*	*Coenonympha*	*arcania*	Dealul Istrita, Breaza	Buzau	Muntenia	Romania	600
HQ004210	EZROM104-08	RV-06-M954		*Nymphalidae*	*Coenonympha*	*arcania*	Dealul Istrita, Breaza	Buzau	Muntenia	Romania	600
HQ004214	EZROM866-08	RVcoll.08-M568		*Nymphalidae*	*Coenonympha*	*arcania*	Breaza, dealul Istrita	Buzau	Muntenia	Romania	600
HQ004212	EZROM105-08	RV-07-C930		*Nymphalidae*	*Coenonympha*	*arcania*	Baile Herculane (Pecinisca)	Caras-Severin	Banat	Romania	220
HQ004213	EZROM102-08	RV-06-M843		*Nymphalidae*	*Coenonympha*	*arcania*	Cluj-Napoca valea paraului Micus	Cluj	Transylvania	Romania	700
HQ004217	EZROM864-08	RVcoll.08-M442		*Nymphalidae*	*Coenonympha*	*arcania*	Canaraua Fetei (Baneasa)	Constanta	Dobrogea	Romania	20
HQ004218	EZROM865-08	RVcoll.08-M457		*Nymphalidae*	*Coenonympha*	*arcania*	Esechioi forest	Constanta	Dobrogea	Romania	135
HQ004216	EZROM863-08	RVcoll.07-D569		*Nymphalidae*	*Coenonympha*	*arcania*	Spini	Hunedoara	Transylvania	Romania	240
HQ004215	EZROM862-08	RVcoll.07-D491		*Nymphalidae*	*Coenonympha*	*arcania*	Cheile Babei	Maramures	Maramures	Romania	265
HM901523	EZSPC1389-10	RVcoll.09-X571	x	*Nymphalidae*	*Coenonympha*	*arcania*	Sorauren		Navarra	Spain	530
GU675707	EZSPM138-09	RVcoll.08-P649	x	*Nymphalidae*	*Coenonympha*	*arcania*	Valle (Valle-Zurea), Lena		Asturias	Spain	662
GU676252	EZSPN905-09	RVcoll.08-L824	x	*Nymphalidae*	*Coenonympha*	*arcania*	Puerto de la Morcuera, Rascafria		Comunidad de Madrid	Spain	1540

GenBank accession number	BOLD Process ID	Sample ID	Used in the Iberian dataset	Family	Genus	Species	Exact Site	Region	State/Province	Country	Alt. (m)
GU676253	EZSPN906-09	RVcoll.08-L828	x	*Nymphalidae*	*Coenonympha*	*arcania*	Oteruelo del Valle		Comunidad de Madrid	Spain	1146
KP871165	EZSPC222-09	RVcoll.08-L445	x	*Nymphalidae*	*Coenonympha*	*arcania*	La Llavina, Montseny, Valles Oriental	Barcelona	Catalonia	Spain	500
KP871121	EZSPC250-09	RVcoll.08-P224	x	*Nymphalidae*	*Coenonympha*	*arcania*	La Molina	Barcelona	Catalonia	Spain	1622
GU676884	EZSPN480-09	RVcoll.08-H915	x	*Nymphalidae*	*Coenonympha*	*arcania*	Hervas, Carretera Hervas-La Garganta	Caceres	Extremadura	Spain	774
GU676426	EZSPN722-09	RVcoll.08-L113	x	*Nymphalidae*	*Coenonympha*	*arcania*	Puerto de las Cabrillas, Portell de Morella	Castellon	Comunidad Valenciana	Spain	1301
KP870387	EZSPC199-09	RVcoll.06-G574	x	*Nymphalidae*	*Coenonympha*	*arcania*	Sta. Maria del Mont, La Garrotxa	Girona	Catalonia	Spain	1110
GU676911	EZSPN506-09	RVcoll.08-H964	x	*Nymphalidae*	*Coenonympha*	*arcania*	Laguarta, Rio Alcanadre	Huesca	Aragon	Spain	1216
GU676029	EZSPM088-09	RVcoll.08-P412	x	*Nymphalidae*	*Coenonympha*	*arcania*	Buron	Leon	Castilla y Leon	Spain	1116
GU675949	EZSPM306-09	RVcoll.08-J853	x	*Nymphalidae*	*Coenonympha*	*arcania*	Posada de Valdeon	Leon	Castilla y Leon	Spain	
KP870290	EZSPC227-09	RVcoll.08-L495	x	*Nymphalidae*	*Coenonympha*	*arcania*	Bassa d`Arres, Vall d`Aran	Lleida	Catalonia	Spain	1501
KP870640	EZSPC255-09	RVcoll.08-P347	x	*Nymphalidae*	*Coenonympha*	*arcania*	Perves, Pallars Jussa	Lleida	Catalonia	Spain	1071
GU675847	EZSPM266-09	RVcoll.08-R481	x	*Nymphalidae*	*Coenonympha*	*arcania*	Lobios, Cela	Ourense	Galicia	Spain	650
HM901238	EZSPC1034-10	RVcoll.08-J192	x	*Nymphalidae*	*Coenonympha*	*dorus*	Seculca, Beca, Boticas		Tras-os-Montes	Portugal	790
GU676549	EZSPN594-09	RVcoll.08-J181	x	*Nymphalidae*	*Coenonympha*	*dorus*	Serra do Larouco (topo), Padornelos, Montalegre		Tras-os-Montes	Portugal	1520
KP871094	EZSPM904-12	Rvcoll.12-M626	x	*Nymphalidae*	*Coenonympha*	*dorus*	Sotuelamos, El Bonillo	Albacete	Castilla-La Mancha	Spain	890
GU677003	EZSPN216-09	RVcoll.07-F077	x	*Nymphalidae*	*Coenonympha*	*dorus*	10 km S. of Morella	Castellon	Comunidad Valenciana	Spain	900
GU676032	EZSPM018-09	RVcoll.08-P018	x	*Nymphalidae*	*Coenonympha*	*dorus*	Talltendre, Cerdanya	Girona	Catalonia	Spain	1571
GU676129	EZSPM457-09	Rvcoll.09-V429	x	*Nymphalidae*	*Coenonympha*	*dorus*	San Juan (Sierra Nevada)	Granada	Andalusia	Spain	2760
GU676127	EZSPM458-09	Rvcoll.09-V508	x	*Nymphalidae*	*Coenonympha*	*dorus*	Sierra de Alfacar	Granada	Andalusia	Spain	
GU676216	EZSPN939-09	RVcoll.08-L908	x	*Nymphalidae*	*Coenonympha*	*dorus*	Gualda	Guadalajara	Castilla-La Mancha	Spain	730
KP870212	EZSPM596-12	RVcoll.10-C389	x	*Nymphalidae*	*Coenonympha*	*dorus*	Villafeliz de Badia	Leon	Castilla y Leon	Spain	1145
JN278894	EZSPN1118-11	Rvcoll.10-C317	x	*Nymphalidae*	*Coenonympha*	*dorus*	Villafeliz de Badia	Leon	Castilla y Leon	Spain	1145
JN278872	EZSPN1037-11	Rvcoll.07-F014	x	*Nymphalidae*	*Coenonympha*	*dorus*	Valldarques, Coll de Nargo, Alt Urgell	Lleida	Catalonia	Spain	975
GU676345	EZSPN807-09	RVcoll.08-L472	x	*Nymphalidae*	*Coenonympha*	*dorus*	Ivars de Noguera	Lleida	Catalonia	Spain	374
KP870559	EZSPM595-12	RVcoll.10-C388	x	*Nymphalidae*	*Coenonympha*	*dorus*	Valbuena de Pisuerga, PP4140 Km 10,9	Palencia	Castilla y Leon	Spain	902
KP870900	EZSPM597-12	RVcoll.10-C390	x	*Nymphalidae*	*Coenonympha*	*dorus*	Valbuena de Pisuerga	Palencia	Castilla y Leon	Spain	902
KP870550	EZSPM598-12	RVcoll.10-C392	x	*Nymphalidae*	*Coenonympha*	*dorus*	Valbuena de Pisuerga, PP4140 Km 10,9	Palencia	Castilla y Leon	Spain	902
JN278895	EZSPN1120-11	Rvcoll.10-C319	x	*Nymphalidae*	*Coenonympha*	*dorus*	Valbuena de Pisuerga.	Palencia	Castilla y Leon	Spain	902
HM901822	EZSPC987-10	RVcoll.07-C696	x	*Nymphalidae*	*Coenonympha*	*dorus*	Collet de la Tina, La Mussara	Tarragona	Catalonia	Spain	240
GU677014	EZSPN201-09	RVcoll.07-C694	x	*Nymphalidae*	*Coenonympha*	*dorus*	Serra de la Gritella, Prades	Tarragona	Catalonia	Spain	1100
HM901514	EZSPC1380-10	RVcoll.09-X516	x	*Nymphalidae*	*Coenonympha*	*dorus*	2 km NW El Vallecillo	Teruel	Aragon	Spain	1450
GU676388	EZSPN762-09	RVcoll.08-L243	x	*Nymphalidae*	*Coenonympha*	*dorus*	Ruta de la Sima, El Vallecillo	Teruel	Aragon	Spain	1531
GU676954	EZSPN549-09	RVcoll.08-J054	x	*Nymphalidae*	*Coenonympha*	*dorus*	Muela de Cortes, Cortes de Pallas	Valencia	Comunidad Valenciana	Spain	
KP870381	FBLMX209-11	BC ZSM Lep 50420		*Nymphalidae*	*Coenonympha*	*gardetta*	Schoenau, Jenner/Stahlhaus	Oberbayern	Bavaria	Germany	1750
HM910010	GWORT464-10	BC ZSM Lep 32150		*Nymphalidae*	*Coenonympha*	*glycerion*	Garching-Hochbrueck	Oberbayern	Bavaria	Germany	487
HM910012	GWORT467-10	BC ZSM Lep 32153		*Nymphalidae*	*Coenonympha*	*glycerion*	Lenggries, Isarauen	Oberbayern	Bavaria	Germany	680
GU707146	FBLMU382-09	BC ZSM Lep 27032		*Nymphalidae*	*Coenonympha*	*glycerion*	Sinzing, Alpiner Steig	Oberpfalz	Bavaria	Germany	405
HM391830	FBLMU488-09	BC ZSM Lep 27138		*Nymphalidae*	*Coenonympha*	*glycerion*	Nittendorf	Oberpfalz	Bavaria	Germany	400
GU707297	FBLMV676-09	BC ZSM Lep 28656		*Nymphalidae*	*Coenonympha*	*glycerion*	Regenstauf, Kreuth	Oberpfalz	Bavaria	Germany	510
GU676550	EZSPN595-09	RVcoll.08-J183	x	*Nymphalidae*	*Coenonympha*	*glycerion*	Serra do Larouco (turfeira N), Padornelos, Montalegre		Tras-os-Montes	Portugal	1360
GU676551	EZSPN596-09	RVcoll.08-J184	x	*Nymphalidae*	*Coenonympha*	*glycerion*	Serra do Larouco (turfeira N), Padornelos, Montalegre		Tras-os-Montes	Portugal	1360
HQ004227	EZROM867-08	RVcoll.08-M217		*Nymphalidae*	*Coenonympha*	*glycerion*	3 Km SW of Lunca Corbului	Arges	Muntenia	Romania	235
HQ004223	EZROM871-08	RVcoll.08-M381		*Nymphalidae*	*Coenonympha*	*glycerion*	Buciumeni forest (Radacinesti)	Bacau	Moldavia	Romania	180
HQ004220	EZROM108-08	RV-07-C955		*Nymphalidae*	*Coenonympha*	*glycerion*	Baile Herculane (Pecinisca)	Caras-Severin	Banat	Romania	220
HQ004222	EZROM106-08	RV-06-K649		*Nymphalidae*	*Coenonympha*	*glycerion*	Baciu forest (Cluj-Napoca)	Cluj	Transylvania	Romania	530
HQ004221	EZROM107-08	RV-07-D191		*Nymphalidae*	*Coenonympha*	*glycerion*	Canaraua Fetei (Baneasa)	Constanta	Dobrogea	Romania	15
HQ004225	EZROM868-08	RVcoll.08-M221		*Nymphalidae*	*Coenonympha*	*glycerion*	2 Km E of Moreni	Dambovita	Muntenia	Romania	290
HQ004224	EZROM870-08	RVcoll.08-M277		*Nymphalidae*	*Coenonympha*	*glycerion*	3 Km NE of Targu Jiu (around Balanesti)	Gorj	Oltenia	Romania	295
HQ004226	EZROM869-08	RVcoll.08-M240		*Nymphalidae*	*Coenonympha*	*glycerion*	7 Km E of Strehaia	Mehedinti	Oltenia	Romania	125
HQ004219	EZROM616-08	RV-07-D906		*Nymphalidae*	*Coenonympha*	*glycerion*	valea Otasaului, Barzesti, Buila-Vanturarita	Valcea	Oltenia	Romania	515

GenBank accession number	BOLD Process ID	Sample ID	Used in the Iberian dataset	Family	Genus	Species	Exact Site	Region	State/Province	Country	Alt. (m)
HM901626	EZSPC771-10	RVcoll.08-L769	x	*Nymphalidae*	*Coenonympha*	*glycerion*	Puerto de Pozazal		Cantabria	Spain	1030
HM901627	EZSPC772-10	RVcoll.08-L770	x	*Nymphalidae*	*Coenonympha*	*glycerion*	Puerto de Pozazal		Cantabria	Spain	1030
GU676284	EZSPN871-09	RVcoll.08-L755	x	*Nymphalidae*	*Coenonympha*	*glycerion*	Oteruelo del Valle		Comunidad de Madrid	Spain	1148
GU676324	EZSPN824-09	RVcoll.08-L633	x	*Nymphalidae*	*Coenonympha*	*glycerion*	Hoyocasero, Venta Rasquilla	Avila	Castilla y Leon	Spain	1224
HM901645	EZSPC794-10	RVcoll.08-P218	x	*Nymphalidae*	*Coenonympha*	*glycerion*	La Molina	Barcelona	Catalonia	Spain	1622.8
GU676501	EZSPN650-09	RVcoll.08-J792	x	*Nymphalidae*	*Coenonympha*	*glycerion*	Acceso Penyagolosa, Vistabella del Maestrazgo	Castellon	Comunidad Valenciana	Spain	1560
HM901767	EZSPC930-10	RVcoll.09-X004	x	*Nymphalidae*	*Coenonympha*	*glycerion*	Vallsabollera, La Cerdanya	Girona	Catalonia	Spain	1560
HM901769	EZSPC932-10	RVcoll.09-X010	x	*Nymphalidae*	*Coenonympha*	*glycerion*	Vall de Campcardos, La Cerdanya	Girona	Catalonia	Spain	1700
HM901564	EZSPC707-10	RVcoll.07-W146	x	*Nymphalidae*	*Coenonympha*	*glycerion*	Finca Matidero, Laguarta	Huesca	Aragon	Spain	1178
HM901565	EZSPC708-10	RVcoll.07-W147	x	*Nymphalidae*	*Coenonympha*	*glycerion*	Finca Matidero, Laguarta	Huesca	Aragon	Spain	1178
GU676973	EZSPN251-09	RVcoll.07-W148	x	*Nymphalidae*	*Coenonympha*	*glycerion*	Finca Matidero, Laguarta	Huesca	Aragon	Spain	1178
GU675925	EZSPM308-09	RVcoll.08-J857	x	*Nymphalidae*	*Coenonympha*	*glycerion*	Bercianos del Real Camino	Leon	Castilla y Leon	Spain	
GU675862	EZSPM377-09	RVcoll.08-J867	x	*Nymphalidae*	*Coenonympha*	*glycerion*	Carande	Leon	Castilla y Leon	Spain	
GU668042	EZSPC352-09	RVcoll.07-C497	x	*Nymphalidae*	*Coenonympha*	*glycerion*	Meranges	Lleida	Catalonia	Spain	1555
GU668043	EZSPC353-09	RVcoll.07-C682	x	*Nymphalidae*	*Coenonympha*	*glycerion*	Meranges	Lleida	Catalonia	Spain	1555
HM901634	EZSPC779-10	RVcoll.08-L859	x	*Nymphalidae*	*Coenonympha*	*glycerion*	Abejar	Soria	Castilla y Leon	Spain	1155
GU676508	EZSPN638-09	RVcoll.08-J762	x	*Nymphalidae*	*Coenonympha*	*glycerion*	El Masegar, Moscardon	Teruel	Aragon	Spain	1531
GU655021	FBLMT911-09	BC ZSM Lep 25471		*Nymphalidae*	*Coenonympha*	*hero*	Jexhof, Hackenwiesen	Oberbayern	Bavaria	Germany	570
JF415692	GWORK516-09	BC ZSM Lep 21846		*Nymphalidae*	*Coenonympha*	*hero*	Diessen	Oberbayern	Bavaria	Germany	600
HM910009	GWORT463-10	BC ZSM Lep 32149		*Nymphalidae*	*Coenonympha*	*hero*	Isarauen, Fischerhaeuser	Oberbayern	Bavaria	Germany	482
HM909901	GWORT466-10	BC ZSM Lep 32152		*Nymphalidae*	*Coenonympha*	*hero*	Zellwies	Oberbayern	Bavaria	Germany	600
HQ004228	EZROM872-08	RVcoll.08-M245		*Nymphalidae*	*Coenonympha*	*leander*	Baile Herculane, Pecinisca	Caras-Severin	Banat	Romania	270
HQ004229	EZROM873-08	RVcoll.08-M252		*Nymphalidae*	*Coenonympha*	*leander*	Baile Herculane, Pecinisca	Caras-Severin	Banat	Romania	270
HQ004230	EZROM874-08	RVcoll.08-M253		*Nymphalidae*	*Coenonympha*	*leander*	Baile Herculane, Pecinisca	Caras-Severin	Banat	Romania	270
HQ004231	EZROM875-08	RVcoll.08-M254		*Nymphalidae*	*Coenonympha*	*leander*	Baile Herculane, Pecinisca	Caras-Severin	Banat	Romania	270
HQ004232	EZROM876-08	RVcoll.08-M255		*Nymphalidae*	*Coenonympha*	*leander*	Baile Herculane, Pecinisca	Caras-Severin	Banat	Romania	270
GU707147	FBLMU190-09	BC ZSM Lep 25700		*Nymphalidae*	*Coenonympha*	*oedippus*	Surroundings of Munich	Oberbayern	Bavaria	Germany	730
HM391828	FBLMU483-09	BC ZSM Lep 27133		*Nymphalidae*	*Coenonympha*	*pamphilus*	Saulburg	Niederbayern	Bavaria	Germany	360
GU655020	FBLMT883-09	BC ZSM Lep 25443		*Nymphalidae*	*Coenonympha*	*pamphilus*	Muenchen, Obermenzing	Oberbayern	Bavaria	Germany	520
GU707174	FBLMU484-09	BC ZSM Lep 27134		*Nymphalidae*	*Coenonympha*	*pamphilus*	Sinzing, Alpiner Steig	Oberpfalz	Bavaria	Germany	405
JF859943	PHLAC365-10	TLMF Lep 02400		*Nymphalidae*	*Coenonympha*	*pamphilus*	Ritten/ Obergruenwald		South Tyrol	Italy	1750
GU676581	EZSPN566-09	RVcoll.08-J107	x	*Nymphalidae*	*Coenonympha*	*pamphilus*	Ribeira de Varzea (EN311), Varzea Cova, Fafe		Minho	Portugal	500
HQ004241	EZROM879-08	RVcoll.08-M365		*Nymphalidae*	*Coenonympha*	*pamphilus*	Buciumeni forest (Radacinesti)	Bacau	Moldavia	Romania	180
HQ004233	EZROM574-08	RV-07-C311		*Nymphalidae*	*Coenonympha*	*pamphilus*	Oraseni Deal	Botosani	Moldavia	Romania	150
HQ004237	EZROM112-08	RV-07-D208		*Nymphalidae*	*Coenonympha*	*pamphilus*	Dealul Istrita, Breaza	Buzau	Muntenia	Romania	540
HQ004238	EZROM113-08	RV-07-D212		*Nymphalidae*	*Coenonympha*	*pamphilus*	Dealul Istrita, Breaza	Buzau	Muntenia	Romania	540
HQ004234	EZROM109-08	RV-06-K637		*Nymphalidae*	*Coenonympha*	*pamphilus*	Baciu forest (Cluj-Napoca)	Cluj	Transylvania	Romania	530
HQ004235	EZROM110-08	RV-06-M836		*Nymphalidae*	*Coenonympha*	*pamphilus*	Cluj-Napoca valea paraului Micus	Cluj	Transylvania	Romania	700
HQ004236	EZROM111-08	RV-07-D118		*Nymphalidae*	*Coenonympha*	*pamphilus*	Dumbraveni forest	Constanta	Dobrogea	Romania	80
HQ004239	EZROM877-08	RVcoll.08-M228		*Nymphalidae*	*Coenonympha*	*pamphilus*	2 Km E of Moreni	Dambovita	Muntenia	Romania	290
HQ004240	EZROM878-08	RVcoll.08-M348		*Nymphalidae*	*Coenonympha*	*pamphilus*	1 Km E of Gheorgheni	Harghita	Transylvania	Romania	900
GU675709	EZSPM102-09	RVcoll.08-P434	x	*Nymphalidae*	*Coenonympha*	*pamphilus*	Sonabia		Cantabria	Spain	16
GU676660	EZSPN378-09	RVcoll.08-H422	x	*Nymphalidae*	*Coenonympha*	*pamphilus*	Cantoblanco		Comunidad de Madrid	Spain	720
KP870702	WMB044-11	RVcoll.LD-1687	x	*Nymphalidae*	*Coenonympha*	*pamphilus*	Mallorca		Balearic Islands	Spain	100
HM901698	EZSPC857-10	RVcoll.08-R457	x	*Nymphalidae*	*Coenonympha*	*pamphilus*	Padron, Picarana-Rio Tinto	A Coruna	Galicia	Spain	30
GU675848	EZSPM244-09	RVcoll.08-R455	x	*Nymphalidae*	*Coenonympha*	*pamphilus*	Negreira, Ponte Maceira, Rio Tambre	A Coruna	Galicia	Spain	150
GU668038	EZSPC354-09	RVcoll.06-G663	x	*Nymphalidae*	*Coenonympha*	*pamphilus*	La Trinitat, El Brull, Osona	Barcelona	Catalonia	Spain	880
GU668039	EZSPC355-09	RVcoll.08-M716	x	*Nymphalidae*	*Coenonympha*	*pamphilus*	Delta de Llobregat	Barcelona	Catalonia	Spain	2
KP870849	EZSPM1014-12	Rvcoll.12-M763	x	*Nymphalidae*	*Coenonympha*	*pamphilus*	4 Km N to Fresnedoso de Ibor	Caceres	Extremadura	Spain	429
KP870430	EZSPM1021-12	Rvcoll.12-M770	x	*Nymphalidae*	*Coenonympha*	*pamphilus*	7 km SE Navalvillar de Ibor	Caceres	Extremadura	Spain	729
KP870349	EZSPM1035-12	Rvcoll.12-M786	x	*Nymphalidae*	*Coenonympha*	*pamphilus*	Merendero de los Horcones, Navatrasierra	Caceres	Extremadura	Spain	664
GU676603	EZSPN444-09	RVcoll.08-H630	x	*Nymphalidae*	*Coenonympha*	*pamphilus*	El Gastor	Cadiz	Andalusia	Spain	660
KP870663	EZSPM681-12	RVcoll.12-L282	x	*Nymphalidae*	*Coenonympha*	*pamphilus*	Los Cortijos	Ciudad Real	Castilla-La Mancha	Spain	

GenBank accession number	BOLD Process ID	Sample ID	Used in the Iberian dataset	Family	Genus	Species	Exact Site	Region	State/Province	Country	Alt. (m)
KP870216	EZSPM682-12	RVcoll.12-L283	x	*Nymphalidae*	*Coenonympha*	*pamphilus*	Los Cortijos	Ciudad Real	Castilla-La Mancha	Spain	
KP870328	EZSPM920-12	Rvcoll.12-M642	x	*Nymphalidae*	*Coenonympha*	*pamphilus*	Lagunas de Ruidera, Ossa de Montiel	Ciudad Real	Castilla-La Mancha	Spain	795
KP870705	EZSPM949-12	Rvcoll.12-M688	x	*Nymphalidae*	*Coenonympha*	*pamphilus*	Rio Jandula, Mestanza	Ciudad Real	Castilla-La Mancha	Spain	407
KP871084	EZSPM969-12	Rvcoll.12-M723	x	*Nymphalidae*	*Coenonympha*	*pamphilus*	Alrededores San Lorenzo de Calatrava	Ciudad Real	Castilla-La Mancha	Spain	749
KP871141	EZSPM972-12	Rvcoll.12-M730	x	*Nymphalidae*	*Coenonympha*	*pamphilus*	Arroyo de los Molinillos, Viso del Marques	Ciudad Real	Castilla-La Mancha	Spain	742
JN827878	EZROM715-08	RV-07-C622	x	*Nymphalidae*	*Coenonympha*	*pamphilus*	Cantallops	Girona	Catalonia	Spain	160
GU668041	EZSPC357-09	RVcoll.07-C669	x	*Nymphalidae*	*Coenonympha*	*pamphilus*	Sant Marti de Puigcerda	Girona	Catalonia	Spain	1145
HM901744	EZSPC905-10	RVcoll.09-V595	x	*Nymphalidae*	*Coenonympha*	*pamphilus*	Seminario (Sierra Nevada)	Granada	Andalusia	Spain	1520
GU676439	EZSPN704-09	RVcoll.08-L050	x	*Nymphalidae*	*Coenonympha*	*pamphilus*	Sierra Elvira, Albolote	Granada	Andalusia	Spain	819
GU675914	EZSPM324-09	RVcoll.08-J898	x	*Nymphalidae*	*Coenonympha*	*pamphilus*	Vega de Espinareda	Leon	Castilla y Leon	Spain	
GU668040	EZSPC356-09	RVcoll.06-G527	x	*Nymphalidae*	*Coenonympha*	*pamphilus*	Coll de Comiols, Pallars Jussa	Lleida	Catalonia	Spain	1130
GU676034	EZSPM044-09	RVcoll.08-P257	x	*Nymphalidae*	*Coenonympha*	*pamphilus*	Santa Maria	Mallorca	Balearic Islands	Spain	181
GU676031	EZSPM067-09	RVcoll.08-P300	x	*Nymphalidae*	*Coenonympha*	*pamphilus*	Genova	Mallorca	Balearic Islands	Spain	123
GU676033	EZSPM068-09	RVcoll.08-P304	x	*Nymphalidae*	*Coenonympha*	*pamphilus*	Sant Elm, Andratx	Mallorca	Balearic Islands	Spain	31
HM901569	EZSPC713-10	RVcoll.08-H112	x	*Nymphalidae*	*Coenonympha*	*pamphilus*	Na Vermella, Mao	Menorca	Balearic Islands	Spain	60
KP870628	EZSPM1002-12	Rvcoll.12-L888	x	*Nymphalidae*	*Coenonympha*	*pamphilus*	Turmaden des Capita, Alaior, Menorca	Menorca	Balearic Islands	Spain	
GU676731	EZSPN303-09	RVcoll.08-H108	x	*Nymphalidae*	*Coenonympha*	*pamphilus*	Na Vermella, Mao	Menorca	Balearic Islands	Spain	
GU676732	EZSPN304-09	RVcoll.08-H109	x	*Nymphalidae*	*Coenonympha*	*pamphilus*	Na Vermella, Mao	Menorca	Balearic Islands	Spain	
GU675845	EZSPM245-09	RVcoll.08-R456	x	*Nymphalidae*	*Coenonympha*	*pamphilus*	Tui, Rio Penedo	Pontevedra	Galicia	Spain	30
HM901594	EZSPC739-10	RVcoll.08-J717	x	*Nymphalidae*	*Coenonympha*	*pamphilus*	Rincon del Sardo, Castielfabib	Valencia	Comunidad Valenciana	Spain	1381
GU676945	EZSPN541-09	RVcoll.08-J033	x	*Nymphalidae*	*Coenonympha*	*pamphilus*	Sinarcas, Sinarcas	Valencia	Comunidad Valenciana	Spain	
KP870596	EULEP128-14	10-A395		*Nymphalidae*	*Coenonympha*	*rhodopensis*	Road to Kom peak (Stara Planina)		Berkovitsa	Bulgaria	1540
KP870770	EULEP129-14	10-B415		*Nymphalidae*	*Coenonympha*	*rhodopensis*	Orelek (Pirin Mts.)		Sandanski	Bulgaria	1800
KP870883	EULEP130-14	10-B420		*Nymphalidae*	*Coenonympha*	*rhodopensis*	Road to Belmeken (Rila Mts.)		Yakoruda	Bulgaria	2000-2400
HQ004242	EZROM880-08	RVcoll.08-M619		*Nymphalidae*	*Coenonympha*	*rhodopensis*	Scorota (Retezat Mts.)	Hunedoara	Transylvania	Romania	1560
HQ004243	EZROM881-08	RVcoll.08-M620		*Nymphalidae*	*Coenonympha*	*rhodopensis*	Scorota (Retezat Mts.)	Hunedoara	Transylvania	Romania	1560
HQ004244	EZROM882-08	RVcoll.08-M621		*Nymphalidae*	*Coenonympha*	*rhodopensis*	Scorota (Retezat Mts.)	Hunedoara	Transylvania	Romania	1560
HQ004245	EZROM883-08	RVcoll.08-M622		*Nymphalidae*	*Coenonympha*	*rhodopensis*	Scorota (Retezat Mts.)	Hunedoara	Transylvania	Romania	1560
HQ004246	EZROM884-08	RVcoll.08-M638		*Nymphalidae*	*Coenonympha*	*rhodopensis*	Scorota (Retezat Mts.)	Hunedoara	Transylvania	Romania	1560
GU707176	FBLMU482-09	BC ZSM Lep 27132		*Nymphalidae*	*Coenonympha*	*tullia*	Kammer, Kammerer Filz	Oberbayern	Bavaria	Germany	600
JF415693	GWORK321-09	BC ZSM Lep 21556		*Nymphalidae*	*Coenonympha*	*tullia*	Vielassmoos	Oberbayern	Bavaria	Germany	426
HM910011	GWORT465-10	BC ZSM Lep 32151		*Nymphalidae*	*Coenonympha*	*tullia*	Lindenrain, Geretsried	Oberbayern	Bavaria	Germany	610
HQ004250	EZRMN359-08	RVcoll.08-M726		*Nymphalidae*	*Coenonympha*	*tullia*	Oriental Carpathianss, Depresiunea Ciucului, Tusnad Sat.	Harghita	Transylvania	Romania	700
HQ004249	EZRMN360-08	RVcoll.08-M727		*Nymphalidae*	*Coenonympha*	*tullia*	Oriental Carpathians, Depresiunea Ciucului, Halta Vrabia	Harghita	Transylvania	Romania	650
HQ004248	EZRMN361-08	RVcoll.08-M728		*Nymphalidae*	*Coenonympha*	*tullia*	Oriental Carpathians, Depresiunea Ciucului, Halta Vrabia	Harghita	Transylvania	Romania	650
HQ004247	EZRMN362-08	RVcoll.08-M729		*Nymphalidae*	*Coenonympha*	*tullia*	Oriental Carpathians, Depresiunea Ciucului, Miercurea Ciuc.	Harghita	Transylvania	Romania	700
HQ004251	EZROM114-08	RV-06-N023		*Nymphalidae*	*Coenonympha*	*tullia*	Gheorgheni, Valea Belchia	Harghita	Transylvania	Romania	1010
JF415694	FBLMU462-09	BC ZSM Lep 27112		*Pieridae*	*Colias*	*alfacariensis*	Kelheim, Ihrlerstein, \`Brannt\`	Niederbayern	Bavaria	Germany	450
GU707335	FBLMV298-09	BC ZSM Lep 28278		*Pieridae*	*Colias*	*alfacariensis*	Kelheim, Lehnberg	Niederbayern	Bavaria	Germany	395
HM393177	GWORA2441-09	BC ZSM Lep 30653		*Pieridae*	*Colias*	*alfacariensis*	Arzmoos	Oberbayern	Bavaria	Germany	1200
HM909999	GWORT453-10	BC ZSM Lep 32139		*Pieridae*	*Colias*	*alfacariensis*	Zellwies, Koenigsdorf	Oberbayern	Bavaria	Germany	600
HM910000	GWORT454-10	BC ZSM Lep 32140		*Pieridae*	*Colias*	*alfacariensis*	Garching-Hochbrueck	Oberbayern	Bavaria	Germany	487
HM910001	GWORT455-10	BC ZSM Lep 32141		*Pieridae*	*Colias*	*alfacariensis*	Brannenburg, Wendelstein	Oberbayern	Bavaria	Germany	1700
HM910002	GWORT456-10	BC ZSM Lep 32142		*Pieridae*	*Colias*	*alfacariensis*	Riem	Oberbayern	Bavaria	Germany	530
HQ004257	EZROM620-08	RV-07-D363		*Pieridae*	*Colias*	*alfacariensis*	Racos	Brasov	Transylvania	Romania	460
HQ004265	EZROM116-08	RV-07-D232		*Pieridae*	*Colias*	*alfacariensis*	Dealul Istrita, Breaza	Buzau	Muntenia	Romania	540
HQ004262	EZROM128-08	RV-07-D093		*Pieridae*	*Colias*	*alfacariensis*	Dealul Istrita, Breaza	Buzau	Muntenia	Romania	540
HQ004261	EZROM569-08	RV-07-C996		*Pieridae*	*Colias*	*alfacariensis*	Baile Herculane (Pecinisca)	Caras-Severin	Banat	Romania	220
HQ004260	EZROM621-08	RV-07-C974		*Pieridae*	*Colias*	*alfacariensis*	Baile Herculane (Pecinisca)	Caras-Severin	Banat	Romania	220
HQ004254	EZRMN324-08	RVcoll.08-H004		*Pieridae*	*Colias*	*alfacariensis*	Cluj-Napoca, dealul Bogomaia, Fanatele Clujului	Cluj	Transylvania	Romania	450

GenBank accession number	BOLD Process ID	Sample ID	Used in the Iberian dataset	Family	Genus	Species	Exact Site	Region	State/Province	Country	Alt. (m)
HQ004253	EZRMN328-08	RVcoll.08-H034		*Pieridae*	*Colias*	*alfacariensis*	Dealul Sf. Pavel (Cluj-Napoca)	Cluj	Transylvania	Romania	450
HQ004252	EZRMN329-08	RVcoll.08-H035		*Pieridae*	*Colias*	*alfacariensis*	Dealul Sf. Pavel (Cluj-Napoca)	Cluj	Transylvania	Romania	450
HQ004264	EZROM117-08	RV-06-K644		*Pieridae*	*Colias*	*alfacariensis*	Baciu forest (Cluj-Napoca)	Cluj	Transylvania	Romania	530
HQ004266	EZROM118-08	RV-06-K654		*Pieridae*	*Colias*	*alfacariensis*	Baciu forest (Cluj-Napoca)	Cluj	Transylvania	Romania	530
HQ004263	EZROM129-08	RV-07-C105		*Pieridae*	*Colias*	*alfacariensis*	Cluj-Napoca, Hoia forest	Cluj	Transylvania	Romania	450
HQ004258	EZROM618-08	RV-07-C114		*Pieridae*	*Colias*	*alfacariensis*	Cluj-Napoca dealul Bogomaia Fanatele Clujului	Cluj	Transylvania	Romania	480
HQ004268	EZROM886-08	RVcoll.08-M500		*Pieridae*	*Colias*	*alfacariensis*	Badeni	Cluj	Transylvania	Romania	490
HQ004267	EZROM115-08	RV-07-D012		*Pieridae*	*Colias*	*alfacariensis*	Canaraua Fetei (Baneasa)	Constanta	Dobrogea	Romania	15
HQ004256	EZRMN399-09	RVcoll.08-L359		*Pieridae*	*Colias*	*alfacariensis*	Cheile Bicazului	Harghita	Transylvania	Romania	940
HQ004255	EZRMN375-08	RVcoll.08-M116		*Pieridae*	*Colias*	*alfacariensis*	Valea Lupului, Rezervatia Valea lui David	Iasi	Moldavia	Romania	75
HQ004269	EZROM887-08	RVcoll.08-M236		*Pieridae*	*Colias*	*alfacariensis*	7 Km E of Strehaia	Mehedinti	Oltenia	Romania	125
HQ004259	EZROM619-08	RV-07-C119		*Pieridae*	*Colias*	*alfacariensis*	Stana	Salaj	Transylvania	Romania	500
GU676691	EZSPN344-09	RVcoll.08-H307	x	*Pieridae*	*Colias*	*alfacariensis*	La Maranosa, San Martin de la Vega		Comunidad de Madrid	Spain	
GU675912	EZSPM326-09	RVcoll.08-R051	x	*Pieridae*	*Colias*	*alfacariensis*	Barrancada	Alicante	Comunidad Valenciana	Spain	817
KP870298	EZROM716-08	RV-06-G437	x	*Pieridae*	*Colias*	*alfacariensis*	El Sola, El Brull	Barcelona	Catalonia	Spain	800
GU668034	EZSPC358-09	RVcoll.08-L336	x	*Pieridae*	*Colias*	*alfacariensis*	Vall d`Horta, Valles Occidental	Barcelona	Catalonia	Spain	520
GU676419	EZSPN724-09	RVcoll.08-L119	x	*Pieridae*	*Colias*	*alfacariensis*	Riu Bergantes, Morella	Castellon	Comunidad Valenciana	Spain	744
GU668035	EZSPC359-09	RVcoll.07-Z078	x	*Pieridae*	*Colias*	*alfacariensis*	Gorg de l`Olla, Campdevanol, Ripolles	Girona	Catalonia	Spain	840
GU676713	EZSPN326-09	RVcoll.08-H200	x	*Pieridae*	*Colias*	*alfacariensis*	Setcases, El Ripolles	Girona	Catalonia	Spain	1321
GU676096	EZSPM438-09	Rvcoll.09-V947	x	*Pieridae*	*Colias*	*alfacariensis*	Collados de la Sagra	Granada	Andalusia	Spain	1475
GU676097	EZSPM439-09	Rvcoll.09-V934	x	*Pieridae*	*Colias*	*alfacariensis*	Sierra de la Sagra	Granada	Andalusia	Spain	1745
GU676632	EZSPN411-09	RVcoll.08-H521	x	*Pieridae*	*Colias*	*alfacariensis*	Duron, La Alcarria	Guadalajara	Castilla-La Mancha	Spain	725
GU676794	EZSPN139-09	RVcoll.06-G498	x	*Pieridae*	*Colias*	*alfacariensis*	Barranco de Valcuerna, Candasnos	Huesca	Aragon	Spain	210
GU675684	EZSPM161-09	RVcoll.08-P689	x	*Pieridae*	*Colias*	*alfacariensis*	Villadangos de Arbas	Leon	Castilla y Leon	Spain	1311
GU675928	EZSPM307-09	RVcoll.08-J855	x	*Pieridae*	*Colias*	*alfacariensis*	Bercianos del Real Camino	Leon	Castilla y Leon	Spain	
GU668036	EZSPC360-09	RVcoll.08-L335	x	*Pieridae*	*Colias*	*alfacariensis*	Alinya, Alt Urgell	Lleida	Catalonia	Spain	1250
HM901552	EZSPC693-10	RVcoll.06-G523	x	*Pieridae*	*Colias*	*alfacariensis*	Coll de Comiols, Pallars Jussa	Lleida	Catalonia	Spain	1130
HM901557	EZSPC700-10	RVcoll.06-V779	x	*Pieridae*	*Colias*	*alfacariensis*	Coll de Boixols, Abella de la Conca, Pallars Jussa	Lleida	Catalonia	Spain	1215
HM901615	EZSPC760-10	RVcoll.08-L424	x	*Pieridae*	*Colias*	*alfacariensis*	Rius de Cerdanya	Lleida	Catalonia	Spain	1273.9
HM901616	EZSPC761-10	RVcoll.08-L425	x	*Pieridae*	*Colias*	*alfacariensis*	Rius de Cerdanya	Lleida	Catalonia	Spain	1273.9
GU675962	EZSPM025-09	RVcoll.08-P097	x	*Pieridae*	*Colias*	*alfacariensis*	Espot, Pallars Sobira	Lleida	Catalonia	Spain	
GU668037	EZSPC361-09	RVcoll.08-H290	x	*Pieridae*	*Colias*	*alfacariensis*	Els Motllats, Vilaplana, Baix Camp	Tarragona	Catalonia	Spain	980
GU675909	EZSPM327-09	RVcoll.08-R064	x	*Pieridae*	*Colias*	*alfacariensis*	La Cerollera	Teruel	Aragon	Spain	781
GU676384	EZSPN758-09	RVcoll.08-L236	x	*Pieridae*	*Colias*	*alfacariensis*	Ruta de la Sima, El Vallecillo	Teruel	Aragon	Spain	1531
HQ004270	EZROM119-08	RV-06-V681		*Pieridae*	*Colias*	*chrysotheme*	Suatu	Cluj	Transylvania	Romania	380
HQ004271	EZROM120-08	RV-07-D438		*Pieridae*	*Colias*	*chrysotheme*	Suatu	Cluj	Transylvania	Romania	380
HQ004272	EZROM669-08	RV-06-V682		*Pieridae*	*Colias*	*chrysotheme*	Suatu	Cluj	Transylvania	Romania	380
HQ004273	EZROM888-08	RVcoll.07-D424		*Pieridae*	*Colias*	*chrysotheme*	Suatu	Cluj	Transylvania	Romania	380
GU688507	GWORO783-09	BC ZSM Lep 30475		*Pieridae*	*Colias*	*croceus*	Vorderriss TOEL		Bavaria	Germany	760
HM393176	GWORA2439-09	BC ZSM Lep 30651		*Pieridae*	*Colias*	*croceus*	Atting	Niederbayern	Bavaria	Germany	330
KP870480	EZSPM683-12	RVcoll.12-L545	x	*Pieridae*	*Colias*	*croceus*	Porches, Lagoa		Algarve	Portugal	
KP870473	EZSPM684-12	RVcoll.12-L567	x	*Pieridae*	*Colias*	*croceus*	Espiche, Lagos		Algarve	Portugal	
GU676558	EZSPN579-09	RVcoll.08-J129	x	*Pieridae*	*Colias*	*croceus*	Seculca, Beca, Boticas		Tras-os-Montes	Portugal	790
HQ004279	EZROM125-08	RV-06-V784		*Pieridae*	*Colias*	*croceus*	Dealul Istrita, Breaza	Buzau	Muntenia	Romania	600
HQ004278	EZROM123-08	RV-07-D588		*Pieridae*	*Colias*	*croceus*	Teregova	Caras-Severin	Banat	Romania	385
HQ004282	EZROM889-08	RVcoll.07-C950		*Pieridae*	*Colias*	*croceus*	Baile Herculane	Caras-Severin	Banat	Romania	220
HQ004281	EZROM890-08	RVcoll.07-E364		*Pieridae*	*Colias*	*croceus*	Cerna Sat	Caras-Severin	Banat	Romania	525
HQ004274	EZRMN395-09	RVcoll.08-H036		*Pieridae*	*Colias*	*croceus*	Dealul Sf. Pavel (Cluj-Napoca)	Cluj	Transylvania	Romania	450
HQ004275	EZROM317-08	RV-07-E679		*Pieridae*	*Colias*	*croceus*	Cluj-Napoca, dealul Sfantul Pavel	Cluj	Transylvania	Romania	450
HQ004280	EZROM121-08	RV-07-D169		*Pieridae*	*Colias*	*croceus*	Sipote	Constanta	Dobrogea	Romania	90
HQ004277	EZROM122-08	RV-07-D199		*Pieridae*	*Colias*	*croceus*	Canaraua Fetei (Baneasa)	Constanta	Dobrogea	Romania	15

GenBank accession number	BOLD Process ID	Sample ID	Used in the Iberian dataset	Family	Genus	Species	Exact Site	Region	State/Province	Country	Alt. (m)
FJ663414	LOWA288-06	2005-LOWA-288		*Pieridae*	*Colias*	*croceus*	Kislovodsk	Stavropol Region	Stavropol	Russia	
JN827879	EZROM717-08	RV-06-H846	x	*Pieridae*	*Colias*	*croceus*	Vallforners, Canoves	Barcelona	Catalonia	Spain	650
GU676893	EZSPN488-09	RVcoll.08-H925	x	*Pieridae*	*Colias*	*croceus*	Hervas, Carretera Hervas-La Garganta	Caceres	Extremadura	Spain	940
GU676892	EZSPN489-09	RVcoll.08-H926	x	*Pieridae*	*Colias*	*croceus*	Hervas, Carretera Hervas-La Garganta	Caceres	Extremadura	Spain	940
GU675852	EZSPM278-09	SMcoll.08-J601	x	*Pieridae*	*Colias*	*croceus*	El Bujeo, Tarifa	Cadiz	Andalusia	Spain	
GU676596	EZSPN449-09	RVcoll.08-H641	x	*Pieridae*	*Colias*	*croceus*	La Linea de la Concepcion, playa de Santa Margarita	Cadiz	Andalusia	Spain	
KP871017	EZSPM981-12	Rvcoll.12-M744	x	*Pieridae*	*Colias*	*croceus*	Illa Grossa, Columbretes, Castellon de la Plana	Castellon	Comunidad Valenciana	Spain	26.6
KP870482	EZSPM986-12	Rvcoll.12-M755	x	*Pieridae*	*Colias*	*croceus*	Illa Grossa, Columbretes, Castellon de la Plana	Castellon	Comunidad Valenciana	Spain	26.6
KP870543	EZSPM987-12	Rvcoll.12-M756	x	*Pieridae*	*Colias*	*croceus*	Illa Grossa, Columbretes, Castellon de la Plana	Castellon	Comunidad Valenciana	Spain	26.6
GU676500	EZSPN649-09	RVcoll.08-J790	x	*Pieridae*	*Colias*	*croceus*	Acceso Penyagolosa, Vistabella del Maestrazgo	Castellon	Comunidad Valenciana	Spain	1560
KP870697	EZSPM935-12	Rvcoll.12-M669	x	*Pieridae*	*Colias*	*croceus*	Rio Jandula, Mestanza	Ciudad Real	Castilla-La Mancha	Spain	407
KP870968	EZSPM959-12	Rvcoll.12-M710	x	*Pieridae*	*Colias*	*croceus*	Alrededores San Lorenzo de Calatrava	Ciudad Real	Castilla-La Mancha	Spain	749
GU668032	EZSPC365-09	RVcoll.08-P073	x	*Pieridae*	*Colias*	*croceus*	Torrent de Ridolaina, Cerdanya	Girona	Catalonia	Spain	1060
GU675677	EZSPC363-09	RVcoll.08-L490	x	*Pieridae*	*Colias*	*croceus*	Bassa d`Arres, Vall d`Aran	Lleida	Catalonia	Spain	1501
GU675960	EZSPM035-09	RVcoll.08-P236	x	*Pieridae*	*Colias*	*croceus*	Puig d`en Galileu	Mallorca	Balearic Islands	Spain	1071
GU675957	EZSPM052-09	RVcoll.08-P270	x	*Pieridae*	*Colias*	*croceus*	S`Albufera	Mallorca	Balearic Islands	Spain	4
GU676734	EZSPN306-09	RVcoll.08-H111	x	*Pieridae*	*Colias*	*croceus*	Torrellafuda, Ciutadella	Menorca	Balearic Islands	Spain	
GU676716	EZSPN320-09	RVcoll.08-H181	x	*Pieridae*	*Colias*	*croceus*	Llimpa, Mao	Menorca	Balearic Islands	Spain	
GU675851	EZSPM238-09	RVcoll.08-R439	x	*Pieridae*	*Colias*	*croceus*	Tui, Rio Penedo	Pontevedra	Galicia	Spain	30
GU668031	EZSPC364-09	RVcoll.08-M687	x	*Pieridae*	*Colias*	*croceus*	Farena, Alt Camp	Tarragona	Catalonia	Spain	580
FJ663413	LOWA529-06	2005-LOWA-529		*Pieridae*	*Colias*	*croceus*	Karadag		Crimea	Ukraine	
FJ663412	LOWA530-06	2005-LOWA-530		*Pieridae*	*Colias*	*croceus*	Karadag		Crimea	Ukraine	
HQ004287	EZROM126-08	RV-06-V718		*Pieridae*	*Colias*	*erate*	Dealul Istrita, Breaza	Buzau	Muntenia	Romania	600
HQ004288	EZROM127-08	RV-06-V722		*Pieridae*	*Colias*	*erate*	Dealul Istrita, Breaza	Buzau	Muntenia	Romania	600
HQ004286	EZROM161-08	RV-06-V721		*Pieridae*	*Colias*	*erate*	Dealul Istrita, Breaza	Buzau	Muntenia	Romania	600
HQ004289	EZROM187-08	RV-06-V720		*Pieridae*	*Colias*	*erate*	Dealul Istrita, Breaza	Buzau	Muntenia	Romania	600
HQ004292	EZROM617-08	RV-06-V785		*Pieridae*	*Colias*	*erate*	Dealul Istrita, Breaza	Buzau	Muntenia	Romania	600
HQ004295	EZROM893-08	RVcoll.08-M659		*Pieridae*	*Colias*	*erate*	Berca, vulcanii noroiosi Paclele Mici	Buzau	Muntenia	Romania	220
HQ004294	EZROM892-08	RVcoll.08-M451		*Pieridae*	*Colias*	*erate*	Canaraua Fetei (Baneasa)	Constanta	Dobrogea	Romania	20
HQ004284	EZRMN397-09	RVcoll.08-M114		*Pieridae*	*Colias*	*erate*	Valea Lupului, Rezervatia `Valea lui David`	Iasi	Moldavia	Romania	75
HQ004283	EZRMN398-09	RVcoll.08-M115		*Pieridae*	*Colias*	*erate*	Valea Lupului, Rezervatia `Valea lui David`	Iasi	Moldavia	Romania	75
HQ004291	EZROM576-08	RV-07-C375		*Pieridae*	*Colias*	*erate*	rezervatia Valea lui David	Iasi	Moldavia	Romania	100
HQ004285	EZRMN396-09	RVcoll.07-F556		*Pieridae*	*Colias*	*erate*	2 km N of Cilieni	Olt	Oltenia	Romania	50
HQ004300	EZROM885-08	RVcoll.07-E541		*Pieridae*	*Colias*	*hyale*	3 km S of Fundata	Arges	Muntenia	Romania	1190
HQ004299	EZROM130-08	RV-07-C104		*Pieridae*	*Colias*	*hyale*	Cluj-Napoca, Hoia forest	Cluj	Transylvania	Romania	450
HQ004298	EZRMN353-08	RVcoll.08-M720		*Pieridae*	*Colias*	*hyale*	Oriental Carpathians, Depresiunea Trei Scaune, Rezervatia `Mestecanisul de la Reci`	Covasna	Transylvania	Romania	520
HQ004297	EZRMN354-08	RVcoll.08-M721		*Pieridae*	*Colias*	*hyale*	Oriental Carpathians, Depresiunea Ciucului, Halta Vrabia	Harghita	Transylvania	Romania	650
HQ004301	EZROM895-08	RVcoll.08-M334		*Pieridae*	*Colias*	*hyale*	Gheorgheni	Harghita	Transylvania	Romania	850
HQ004302	EZROM896-08	RVcoll.08-M335		*Pieridae*	*Colias*	*hyale*	Gheorgheni	Harghita	Transylvania	Romania	850
FJ663418	LOWA782-06	2005-LOWA-782		*Pieridae*	*Colias*	*hyale*	Osyno	Sebezh distr.	Pskov	Russia	150
HQ004276	EZROM124-08	RV-06-V783		*Pieridae*	*Colias*	*hybrid?*	Dealul Istrita, Breaza	Buzau	Muntenia	Romania	600
HQ004296	EZROM894-08	RVcoll.08-M453		*Pieridae*	*Colias*	*hybrid?*	Canaraua Fetei (Baneasa)	Constanta	Dobrogea	Romania	20
HQ004290	EZROM575-08	RV-07-C376		*Pieridae*	*Colias*	*hybrid?*	rezervatia Valea lui David	Iasi	Moldavia	Romania	100
HQ004293	EZROM891-08	RVcoll.08-M411		*Pieridae*	*Colias*	*hybrid?*	Dealul Consul	Tulcea	Dobrogea	Romania	80
HQ004303	EZROM131-08	RV-06-V650		*Pieridae*	*Colias*	*myrmidone*	Faget forest (Cluj-Napoca)	Cluj	Transylvania	Romania	730
HQ004304	EZROM132-08	RV-06-V651		*Pieridae*	*Colias*	*myrmidone*	Faget forest (Cluj-Napoca)	Cluj	Transylvania	Romania	730
HQ004305	EZROM670-08	RV-06-M835		*Pieridae*	*Colias*	*myrmidone*	Cluj-Napoca, valea paraului Micus	Cluj	Transylvania	Romania	700
HQ004306	EZROM897-08	RVcoll.07-C136		*Pieridae*	*Colias*	*myrmidone*	Salicea (Ratu Penii), Cluj-Napoca	Cluj	Transylvania	Romania	750

GenBank accession number	BOLD Process ID	Sample ID	Used in the Iberian dataset	Family	Genus	Species	Exact Site	Region	State/Province	Country	Alt. (m)
GU688510	GWORO782-09	BC ZSM Lep 30474		*Pieridae*	*Colias*	*palaeno*	Schoenrain Koenigsdorf / TOEL		Bavaria	Germany	
HQ565464	GWOSA727-10	BC ZSM Lep 35738		*Pieridae*	*Colias*	*palaeno*	Todtenau, Dornerau	Niederbayern	Bavaria	Germany	
HM391817	FBLMU459-09	BC ZSM Lep 27109		*Pieridae*	*Colias*	*palaeno*	Ruhpolding, NSG Roethelmoos	Oberbayern	Bavaria	Germany	850
GU675959	EZSPM010-09	RVcoll.08-M990	x	*Pieridae*	*Colias*	*phicomone*	Grau Roig			Andorra	2114
HM901817	EZSPC982-10	RVcoll.07-C019	x	*Pieridae*	*Colias*	*phicomone*	Puigmal	Languedoc-Roussillon		France	2200
HM901818	EZSPC983-10	RVcoll.07-C020	x	*Pieridae*	*Colias*	*phicomone*	Puigmal	Languedoc-Roussillon		France	2200
HM901819	EZSPC984-10	RVcoll.07-C028	x	*Pieridae*	*Colias*	*phicomone*	Puigmal	Languedoc-Roussillon		France	2200
HM393178	GWORA2442-09	BC ZSM Lep 30654		*Pieridae*	*Colias*	*phicomone*	Giessenbach	Oberbayern	Bavaria	Germany	
KP870284	EZSPM832-12	RVcoll.140711ZG16	x	*Pieridae*	*Colias*	*phicomone*	Ibon de Asnos, Panticosa	Huesca	Aragon	Spain	2060
HM901717	EZSPC877-10	RVcoll.09-T128	x	*Pieridae*	*Colias*	*phicomone*	Cercanias del Cueto de Arbas, Villablino	Leon	Castilla y Leon	Spain	1930
HM901718	EZSPC878-10	RVcoll.09-T129	x	*Pieridae*	*Colias*	*phicomone*	Cercanias del Cueto de Arbas, Villablino	Leon	Castilla y Leon	Spain	1930
HM901566	EZSPC709-10	RVcoll.07-W195	x	*Pieridae*	*Colias*	*phicomone*	Tuc de la Cigalera, Vall d`Aran	Lleida	Catalonia	Spain	2330
HM901687	EZSPC846-10	RVcoll.08-R219	x	*Pieridae*	*Colias*	*phicomone*	Port de la Bonaigua, Pallars Sobira	Lleida	Catalonia	Spain	2338
HM901688	EZSPC847-10	RVcoll.08-R222	x	*Pieridae*	*Colias*	*phicomone*	Port de la Bonaigua, Pallars Sobira	Lleida	Catalonia	Spain	2338
HM901702	EZSPC861-10	RVcoll.09-T063	x	*Pieridae*	*Colias*	*phicomone*	Tuc de la Cigalera, Vall d`Aran	Lleida	Catalonia	Spain	2330
HM901703	EZSPC862-10	RVcoll.09-T065	x	*Pieridae*	*Colias*	*phicomone*	Tuc de la Cigalera, Vall d`Aran	Lleida	Catalonia	Spain	2330
GU675849	EZSPM191-09	RVcoll.08-R218	x	*Pieridae*	*Colias*	*phicomone*	Port de la Bonaigua, Pallars Sobira	Lleida	Catalonia	Spain	2338
GU675850	EZSPM192-09	RVcoll.08-R220	x	*Pieridae*	*Colias*	*phicomone*	Port de la Bonaigua, Pallars Sobira	Lleida	Catalonia	Spain	2338
GU676764	EZSPN272-09	RVcoll.07-W194	x	*Pieridae*	*Colias*	*phicomone*	Tuc de la Cigalera, Vall d`Aran	Lleida	Catalonia	Spain	2330
GU676765	EZSPN273-09	RVcoll.07-W196	x	*Pieridae*	*Colias*	*phicomone*	Tuc de la Cigalera, Vall d`Aran	Lleida	Catalonia	Spain	2330
HQ004308	EZRMN407-09	RVcoll.07-D210		*Lycaenidae*	*Cupido*	*alcetas*	Breaza, dealul Istrita	Buzau	Muntenia	Romania	400
HQ004309	EZRMN406-09	RVcoll.07-E526		*Lycaenidae*	*Cupido*	*alcetas*	2 km N of Cilieni	Olt	Oltenia	Romania	50
HQ004307	EZRMN409-09	RVcoll.07-E458		*Lycaenidae*	*Cupido*	*alcetas*	2 km E of Smardan	Tulcea	Dobrogea	Romania	5
GU669646	EZROM1063-09	RVcoll.07-F559		*Lycaenidae*	*Cupido*	*alcetas*	Nifon (Macin Mts.)	Tulcea	Dobrogea	Romania	120
HM901409	EZSPC1210-10	RVcoll.09-V500	x	*Lycaenidae*	*Cupido*	*alcetas*	Viladrau, Montseny, Osona	Barcelona	Catalonia	Spain	715
GU669826	EZSPC407-09	RVcoll.07-E644	x	*Lycaenidae*	*Cupido*	*alcetas*	Viladrau, Camp Gran	Barcelona	Catalonia	Spain	730
GU669829	EZSPC410-09	RVcoll.07-E639	x	*Lycaenidae*	*Cupido*	*alcetas*	road Gombren a Castellar de n`Hug	Barcelona	Catalonia	Spain	1250
GU677035	EZSPN178-09	RVcoll.06-V729	x	*Lycaenidae*	*Cupido*	*alcetas*	Greixer, El Bergueda	Barcelona	Catalonia	Spain	1050
HM901444	EZSPC1249-10	RVcoll.09-V824	x	*Lycaenidae*	*Cupido*	*alcetas*	Vilallonga de Ter, Ripolles	Girona	Catalonia	Spain	1143.6
GU669827	EZSPC408-09	RVcoll.08-R286	x	*Lycaenidae*	*Cupido*	*alcetas*	Masia del Plans, Vidra, La Garrotxa	Girona	Catalonia	Spain	1200
GU669828	EZSPC409-09	RVcoll.08-R305	x	*Lycaenidae*	*Cupido*	*alcetas*	Tapis, Macanet de Cabrenys, Alt Emporda	Girona	Catalonia	Spain	575
GU669822	EZSPC411-09	RVcoll.08-P058	x	*Lycaenidae*	*Cupido*	*alcetas*	Estoll, Fontanals de Cerdanya, Cerdanya	Girona	Catalonia	Spain	1085
GU676090	EZSPM407-09	Rvcoll.09-V823	x	*Lycaenidae*	*Cupido*	*alcetas*	Vilallonga de Ter, Ripolles	Girona	Catalonia	Spain	
GU675942	EZSPM292-09	RVcoll.08-J823	x	*Lycaenidae*	*Cupido*	*alcetas*	Toreno	Leon	Castilla y Leon	Spain	
GU675873	EZSPM363-09	RVcoll.08-J824	x	*Lycaenidae*	*Cupido*	*alcetas*	Santa Colomba de la Vega	Leon	Castilla y Leon	Spain	
HQ004310	EZROM190-08	RV-06-M930		*Lycaenidae*	*Cupido*	*argiades*	Dealul Istrita, Breaza	Buzau	Muntenia	Romania	600
HQ004312	EZROM192-08	RV-07-C951		*Lycaenidae*	*Cupido*	*argiades*	Baile Herculane (Pecinisca)	Caras-Severin	Banat	Romania	220
HQ004314	EZROM188-08	RV-06-K543		*Lycaenidae*	*Cupido*	*argiades*	Badeni	Cluj	Transylvania	Romania	440
HQ004311	EZROM189-08	RV-06-K545		*Lycaenidae*	*Cupido*	*argiades*	Badeni	Cluj	Transylvania	Romania	440
HQ004318	EZROM999-08	RVcoll.08-M447		*Lycaenidae*	*Cupido*	*argiades*	Canaraua Fetei (Baneasa)	Constanta	Dobrogea	Romania	20
HQ004313	EZROM191-08	RV-07-D957		*Lycaenidae*	*Cupido*	*argiades*	Valea Mare	Dambovita	Muntenia	Romania	225
HQ004317	EZROM998-08	RVcoll.07-D902		*Lycaenidae*	*Cupido*	*argiades*	Ciupercenii de Oltet	Gorj	Oltenia	Romania	500
HQ004316	EZROM997-08	RVcoll.07-D580		*Lycaenidae*	*Cupido*	*argiades*	Spini	Hunedoara	Transylvania	Romania	240
HQ004315	EZROM582-08	RV-07-C335		*Lycaenidae*	*Cupido*	*argiades*	Radauti	Suceava	Moldavia	Romania	375
GU675743	EZSPM104-09	RVcoll.08-P438	x	*Lycaenidae*	*Cupido*	*argiades*	Sonabia		Cantabria	Spain	16
GU675742	EZSPM132-09	RVcoll.08-P641	x	*Lycaenidae*	*Cupido*	*argiades*	Los Fontanares, Pena Munoz, Lena		Asturias	Spain	1331
GU675744	EZSPM134-09	RVcoll.08-P644	x	*Lycaenidae*	*Cupido*	*argiades*	Valle (Valle-Zurea), Lena		Asturias	Spain	662
JN276902	EZSPN1088-11	Rvcoll.09-X569	x	*Lycaenidae*	*Cupido*	*argiades*	Sorauren		Navarra	Spain	530
GU676928	EZSPN526-09	RVcoll.08-H996	x	*Lycaenidae*	*Cupido*	*argiades*	Quiros, Fresnedo-Subida al Collaon		Asturias	Spain	850
GU675800	EZSPM250-09	RVcoll.08-R462	x	*Lycaenidae*	*Cupido*	*argiades*	Santiso, Quintelas-Bajada Ferry	A Coruna	Galicia	Spain	266
KP870442	EZROM364-08	07-E629	x	*Lycaenidae*	*Cupido*	*argiades*	Castellar de n`Hug	Barcelona	Catalonia	Spain	1250
GU669823	EZSPC412-09	RVcoll.08-M707	x	*Lycaenidae*	*Cupido*	*argiades*	Masia del Plans, Vidra, La Garrotxa	Girona	Catalonia	Spain	1200
GU669824	EZSPC413-09	RVcoll.08-R270	x	*Lycaenidae*	*Cupido*	*argiades*	Masia del Plans, Vidra, La Garrotxa	Girona	Catalonia	Spain	1200
GU669825	EZSPC414-09	RVcoll.08-R271	x	*Lycaenidae*	*Cupido*	*argiades*	Masia del Plans, Vidra, La Garrotxa	Girona	Catalonia	Spain	1200

GenBank accession number	BOLD Process ID	Sample ID	Used in the Iberian dataset	Family	Genus	Species	Exact Site	Region	State/Province	Country	Alt. (m)
GU669818	EZSPC415-09	RVcoll.08-R272	x	*Lycaenidae*	*Cupido*	*argiades*	Masia del Plans, Vidra, La Garrotxa	Girona	Catalonia	Spain	1200
GU669819	EZSPC416-09	RVcoll.08-R291	x	*Lycaenidae*	*Cupido*	*argiades*	Masia del Plans, Vidra, La Garrotxa	Girona	Catalonia	Spain	1200
GU676091	EZSPM402-09	Rvcoll.09-V803	x	*Lycaenidae*	*Cupido*	*argiades*	Estanys de la Jonquera, La Jonquera, Alt Emporda	Girona	Catalonia	Spain	
GU676088	EZSPM409-09	Rvcoll.09-V826	x	*Lycaenidae*	*Cupido*	*argiades*	Vilallonga de Ter, Ripolles	Girona	Catalonia	Spain	
HQ004320	EZROM194-08	RV-07-D308		*Lycaenidae*	*Cupido*	*decolorata*	Racos	Brasov	Transylvania	Romania	460
HQ004327	EZROM1000-08	RVcoll.08-M565		*Lycaenidae*	*Cupido*	*decolorata*	Breaza, dealul Istrita	Buzau	Muntenia	Romania	600
GU675626	EZROM1045-09	RVcoll.07-D109.1		*Lycaenidae*	*Cupido*	*decolorata*	Nenciulesti	Buzau	Muntenia	Romania	360
GU675627	EZROM1072-09	RVcoll.08-L364		*Lycaenidae*	*Cupido*	*decolorata*	Breaza, dealul Istrita	Buzau	Muntenia	Romania	400
HQ004324	EZROM994-08	RVcoll.08-M574		*Lycaenidae*	*Cupido*	*decolorata*	Breaza, dealul Istrita	Buzau	Muntenia	Romania	600
HQ004325	EZROM995-08	RVcoll.08-M575		*Lycaenidae*	*Cupido*	*decolorata*	Breaza, dealul Istrita	Buzau	Muntenia	Romania	600
HQ004326	EZROM996-08	RVcoll.08-M655		*Lycaenidae*	*Cupido*	*decolorata*	Berca, vulcanii noroiosi Paclele Mici	Buzau	Muntenia	Romania	220
HQ004319	EZROM193-08	RV-06-K610		*Lycaenidae*	*Cupido*	*decolorata*	Badeni	Cluj	Transylvania	Romania	440
HQ004322	EZROM329-08	RV-08-A007		*Lycaenidae*	*Cupido*	*decolorata*	Cluj-Napoca, dealul Feleacului, Gheorgheni forest	Cluj	Transylvania	Romania	600
HQ004323	EZROM343-08	RV-08-A021		*Lycaenidae*	*Cupido*	*decolorata*	Badeni	Cluj	Transylvania	Romania	440
HQ004321	EZROM195-08	RV-07-C123		*Lycaenidae*	*Cupido*	*decolorata*	Stana	Salaj	Transylvania	Romania	500
HM901592	EZSPC737-10	RVcoll.08-J206	x	*Lycaenidae*	*Cupido*	*lorquinii*	Barranco de las Ermitas, Sierra Almijara, Albunuelas	Granada	Andalusia	Spain	1145
HM901598	EZSPC743-10	RVcoll.08-L035	x	*Lycaenidae*	*Cupido*	*lorquinii*	Casillas de Rojas, Gueejar Sierra	Granada	Andalusia	Spain	1733
HM901599	EZSPC744-10	RVcoll.08-L036	x	*Lycaenidae*	*Cupido*	*lorquinii*	Casillas de Rojas, Gueejar Sierra	Granada	Andalusia	Spain	1733
HM901600	EZSPC745-10	RVcoll.08-L037	x	*Lycaenidae*	*Cupido*	*lorquinii*	Casillas de Rojas, Gueejar Sierra	Granada	Andalusia	Spain	1733
HM901621	EZSPC766-10	RVcoll.08-L674	x	*Lycaenidae*	*Cupido*	*lorquinii*	Iznalloz, Sierra Arana	Granada	Andalusia	Spain	1306
HM901669	EZSPC821-10	RVcoll.08-P712	x	*Lycaenidae*	*Cupido*	*lorquinii*	Cueva de la Higuera, Sierra Nevada	Granada	Andalusia	Spain	1142
KP870431	EZSPM606-12	RVcoll.11-D612	x	*Lycaenidae*	*Cupido*	*lorquinii*	Canada Real de la Zubia, Monachil	Granada	Andalusia	Spain	1350
KP870965	EZSPM610-12	RVcoll.11-D682	x	*Lycaenidae*	*Cupido*	*lorquinii*	La Zubia, Barranco de Corvales	Granada	Andalusia	Spain	970
GU676459	EZSPN684-09	RVcoll.08-L009	x	*Lycaenidae*	*Cupido*	*lorquinii*	Cercanias El Purche, Gueejar Sierra	Granada	Andalusia	Spain	1494
GU676451	EZSPN692-09	RVcoll.08-L023	x	*Lycaenidae*	*Cupido*	*lorquinii*	Casillas de Rojas, Gueejar Sierra	Granada	Andalusia	Spain	1733
GU676416	EZSPN697-09	RVcoll.08-L034	x	*Lycaenidae*	*Cupido*	*lorquinii*	Casillas de Rojas, Gueejar Sierra	Granada	Andalusia	Spain	1733
GU676356	EZSPN793-09	RVcoll.08-L352	x	*Lycaenidae*	*Cupido*	*lorquinii*	Sierra Prieta	Granada	Andalusia	Spain	
GU676311	EZSPN842-09	RVcoll.08-L673	x	*Lycaenidae*	*Cupido*	*lorquinii*	Iznalloz, Sierra Arana	Granada	Andalusia	Spain	1306
KP871083	WMB006-11	RVcoll.11-D831	x	*Lycaenidae*	*Cupido*	*lorquinii*	Canada Real de la Zubia, Monachil	Granada	Andalusia	Spain	1350
HM901582	EZSPC727-10	RVcoll.08-H621.1	x	*Lycaenidae*	*Cupido*	*lorquinii*	Benadalid	Malaga	Andalusia	Spain	790
HM901583	EZSPC728-10	RVcoll.08-H623	x	*Lycaenidae*	*Cupido*	*lorquinii*	Benadalid	Malaga	Andalusia	Spain	790
GU676599	EZSPN440-09	RVcoll.08-H622	x	*Lycaenidae*	*Cupido*	*lorquinii*	Benadalid	Malaga	Andalusia	Spain	790
HM393190	GWORA2462-09	BC ZSM Lep 30674		*Lycaenidae*	*Cupido*	*minimus*	Zahmer Kaiser, Aschinger Alm		Tirol	Austria	1000
GU688441	GWORO792-09	BC ZSM Lep 30484		*Lycaenidae*	*Cupido*	*minimus*	Garching-Hochbrueck MuenchenL		Bavaria	Germany	486
GU707085	FBLMU452-09	BC ZSM Lep 27102		*Lycaenidae*	*Cupido*	*minimus*	Schoengeising	Oberbayern	Bavaria	Germany	545
GU686862	GWORL438-09	BC ZSM Lep 22340		*Lycaenidae*	*Cupido*	*minimus*	Oberschleissheim, Flughafen-Ost	Oberbayern	Bavaria	Germany	480
GU655019	FBLMT889-09	BC ZSM Lep 25449		*Lycaenidae*	*Cupido*	*minimus*	Nittendorf	Oberpfalz	Bavaria	Germany	400
HQ004334	EZROM133-08	RV-06-N004		*Lycaenidae*	*Cupido*	*minimus*	Apuseni Mts., Rimetea (Piatra Secuiului)	Alba	Transylvania	Romania	750
HQ004331	EZROM320-08	RV-07-E682		*Lycaenidae*	*Cupido*	*minimus*	Rimetea, Piatra Secuiului	Alba	Transylvania	Romania	750
HQ004330	EZROM345-08	RV-08-A023		*Lycaenidae*	*Cupido*	*minimus*	Dealul Istrita, Breaza	Buzau	Muntenia	Romania	525
HQ004329	EZROM347-08	RV-08-A025		*Lycaenidae*	*Cupido*	*minimus*	Dealul Istrita, Breaza	Buzau	Muntenia	Romania	525
HQ004337	EZROM135-08	RV-07-C941		*Lycaenidae*	*Cupido*	*minimus*	Baile Herculane (Pecinisca)	Caras-Severin	Banat	Romania	220
HQ004332	EZROM321-08	RV-07-E683		*Lycaenidae*	*Cupido*	*minimus*	Baisoara	Cluj	Transylvania	Romania	700
HQ004335	EZROM134-08	RV-07-D920		*Lycaenidae*	*Cupido*	*minimus*	Ciupercenii de Oltet	Gorj	Oltenia	Romania	500
HQ004336	EZROM898-08	RVcoll.08-M349		*Lycaenidae*	*Cupido*	*minimus*	1 Km E of Gheorgheni	Harghita	Transylvania	Romania	900
HQ004328	EZROM622-08	RV-07-D521		*Lycaenidae*	*Cupido*	*minimus*	Rohia	Maramures	Maramures	Romania	360
HQ004333	EZROM136-08	RV-07-E525		*Lycaenidae*	*Cupido*	*minimus*	2 km N of Cilieni	Olt	Oltenia	Romania	50
GQ128947		AD-00-P540		*Lycaenidae*	*Cupido*	*minimus*	Tatinki		Tula	Russia	
KP871039	EZSPM599-12	RVcoll.11-D670	x	*Lycaenidae*	*Cupido*	*minimus*	Font de l`Arbre, Serra d`Aitana	Alicante	Comunidad Valenciana	Spain	1209
KP870446	EZSPM600-12	RVcoll.11-D671	x	*Lycaenidae*	*Cupido*	*minimus*	Font de l`Arbre, Serra d`Aitana	Alicante	Comunidad Valenciana	Spain	1209
KP870679	EZSPM608-12	RVcoll.11-D675	x	*Lycaenidae*	*Cupido*	*minimus*	Font de l`Arbre, Serra d`Aitana	Alicante	Comunidad Valenciana	Spain	1209

GenBank accession number	BOLD Process ID	Sample ID	Used in the Iberian dataset	Family	Genus	Species	Exact Site	Region	State/Province	Country	Alt. (m)
KP870792	EZSPM765-12	RVcoll.040611HZ26	x	*Lycaenidae*	*Cupido*	*minimus*	El Montgo _ Nivel Alto, Xavia	Alicante	Comunidad Valenciana	Spain	629
KP871010	EZSPM782-12	RVcoll.070111GN93	x	*Lycaenidae*	*Cupido*	*minimus*	El Montgo _ Nivel Medio, Xavia	Alicante	Comunidad Valenciana	Spain	161
KP870564	EZSPM809-12	RVcoll.130211WX11	x	*Lycaenidae*	*Cupido*	*minimus*	Nevero de la Font Roja, Alcoi	Alicante	Comunidad Valenciana	Spain	1298
KP871105	WMB004-11	RVcoll.11-D667	x	*Lycaenidae*	*Cupido*	*minimus*	Font de l`Arbre, Serra d`Aitana	Alicante	Comunidad Valenciana	Spain	1209
KP871007	WMB005-11	RVcoll.11-D668	x	*Lycaenidae*	*Cupido*	*minimus*	Font de l`Arbre, Serra d`Aitana	Alicante	Comunidad Valenciana	Spain	1209
KP870315	EZROM718-08	RV-06-G445	x	*Lycaenidae*	*Cupido*	*minimus*	El Sola, El Brull	Barcelona	Catalonia	Spain	800
KP870569	EZSPC278-09	RVcoll.06-G465	x	*Lycaenidae*	*Cupido*	*minimus*	Can Rovira, El Brull, Osona	Barcelona	Catalonia	Spain	830
KP870524	EZSPM810-12	RVcoll.130211WX60	x	*Lycaenidae*	*Cupido*	*minimus*	Acceso Penyagolosa, Villahermosa del Rio	Castellon	Comunidad Valenciana	Spain	1810
GU676472	EZSPN648-09	RVcoll.08-J789	x	*Lycaenidae*	*Cupido*	*minimus*	Acceso Penyagolosa, Vistabella del Maestrazgo	Castellon	Comunidad Valenciana	Spain	1560
GU676622	EZSPN425-09	RVcoll.08-H591	x	*Lycaenidae*	*Cupido*	*minimus*	Gualda, La Alcarria	Guadalajara	Castilla-La Mancha	Spain	742
GU677039	EZSPN257-09	RVcoll.07-W160	x	*Lycaenidae*	*Cupido*	*minimus*	Vallibierna	Huesca	Aragon	Spain	1800
GU668033	EZSPC366-09	RVcoll.07-C630	x	*Lycaenidae*	*Cupido*	*minimus*	Meranges	Lleida	Catalonia	Spain	1555
GU669864	EZSPC367-09	RVcoll.08-M911	x	*Lycaenidae*	*Cupido*	*minimus*	Bassa d`Arres, Vall d`Aran	Lleida	Catalonia	Spain	1500
GU668027	EZSPC368-09	RVcoll.08-R113	x	*Lycaenidae*	*Cupido*	*minimus*	Salt de Pitx, Vall d`Aran	Lleida	Catalonia	Spain	1570
HM901699	EZSPC858-10	RVcoll.08-R490	x	*Lycaenidae*	*Cupido*	*minimus*	Pedrafita do Cebreiro, Serra do Caurel-Hospital	Lugo	Galicia	Spain	1230
HM901700	EZSPC859-10	RVcoll.08-R491	x	*Lycaenidae*	*Cupido*	*minimus*	Folgoso do Caurel, Alto de Visuna	Lugo	Galicia	Spain	1330
GU675797	EZSPM272-09	RVcoll.08-R489	x	*Lycaenidae*	*Cupido*	*minimus*	Folgoso do Caurel, Alto de Visuna	Lugo	Galicia	Spain	1330
HM901667	EZSPC819-10	RVcoll.08-P706	x	*Lycaenidae*	*Cupido*	*minimus*	Mata del Quintanar	Segovia	Castilla y Leon	Spain	960
HM901668	EZSPC820-10	RVcoll.08-P707	x	*Lycaenidae*	*Cupido*	*minimus*	Mata del Quintanar	Segovia	Castilla y Leon	Spain	960
GU668028	EZSPC369-09	RVcoll.08-H291	x	*Lycaenidae*	*Cupido*	*minimus*	Els Motllats, Vilaplana, Baix Camp	Tarragona	Catalonia	Spain	980
KP870334	EZSPM785-12	RVcoll.070211PL52	x	*Lycaenidae*	*Cupido*	*minimus*	Acceso Javalambre, Puebla de Valverde	Teruel	Aragon	Spain	1817
GU676506	EZSPN636-09	RVcoll.08-J758	x	*Lycaenidae*	*Cupido*	*minimus*	El Masegar, Moscardon	Teruel	Aragon	Spain	1531
GU676938	EZSPN536-09	RVcoll.08-J018	x	*Lycaenidae*	*Cupido*	*minimus*	Sinarcas, Sinarcas	Valencia	Comunidad Valenciana	Spain	
GU676520	EZSPN626-09	RVcoll.08-J716	x	*Lycaenidae*	*Cupido*	*minimus*	Rincon del Sardo, Castielfabib	Valencia	Comunidad Valenciana	Spain	1381
KP870606	EZSPC830-10	RVcoll.08-R094	x	*Lycaenidae*	*Cupido*	*minimus (carswelli)*	Sierra Espuna		Murcia	Spain	1300
KP870803	EZSPC831-10	RVcoll.08-R095	x	*Lycaenidae*	*Cupido*	*minimus (carswelli)*	Sierra Espuna		Murcia	Spain	1300
KP870556	EZSPC832-10	RVcoll.08-R096	x	*Lycaenidae*	*Cupido*	*minimus (carswelli)*	Sierra Espuna		Murcia	Spain	1300
KP870887	EZSPC833-10	RVcoll.08-R097	x	*Lycaenidae*	*Cupido*	*minimus (carswelli)*	Sierra Espuna		Murcia	Spain	1300
KP870815	EULEP098-14	09-V159	x	*Lycaenidae*	*Cupido*	*minimus (carswelli)*	Sierra María	Almería	Andalusia	Spain	1250
KP870825	EULEP099-14	09-V160	x	*Lycaenidae*	*Cupido*	*minimus (carswelli)*	Sierra María	Almería	Andalusia	Spain	1250
KP870888	EULEP100-14	09-V161	x	*Lycaenidae*	*Cupido*	*minimus (carswelli)*	E. Sierra de Orce	Granada	Andalusia	Spain	1300
KP871171	EULEP101-14	09-V162	x	*Lycaenidae*	*Cupido*	*minimus (carswelli)*	E. Sierra de Orce	Granada	Andalusia	Spain	1300
KP871077	EZSPC1405-10	RVcoll.09-V116	x	*Lycaenidae*	*Cupido*	*minimus (carswelli)*	Sierra Maria	Granada	Andalusia	Spain	1600
HM901532	EZSPC1407-10	RVcoll.09-V118	x	*Lycaenidae*	*Cupido*	*minimus (carswelli)*	Cullar	Granada	Andalusia	Spain	1350
HM901546	EZSPC1446-10	RVcoll.09-V158	x	*Lycaenidae*	*Cupido*	*minimus (carswelli)*	E. Sierra de Orce	Granada	Andalusia	Spain	1300

GenBank accession number	BOLD Process ID	Sample ID	Used in the Iberian dataset	Family	Genus	Species	Exact Site	Region	State/Province	Country	Alt. (m)
GU676357	EZSPN794-09	RVcoll.08-L353	x	*Lycaenidae*	*Cupido*	*minimus* (*carswelli*)	Sierra de Orce, Orce	Granada	Andalusia	Spain	
GU676358	EZSPN795-09	RVcoll.08-L354	x	*Lycaenidae*	*Cupido*	*minimus* (*carswelli*)	Sierra de Orce, Orce	Granada	Andalusia	Spain	
HQ004338	EZRMN383-09	RVcoll.08-L360		*Lycaenidae*	*Cupido*	*osiris*	Dealul Istrita, Breaza	Buzau	Muntenia	Romania	400
HQ004344	EZRMN425-09	RVcoll.09-V674		*Lycaenidae*	*Cupido*	*osiris*	Breaza, dealul Istrita	Buzau	Muntenia	Romania	350
HQ004341	EZROM900-08	RVcoll.08-M567		*Lycaenidae*	*Cupido*	*osiris*	Breaza, dealul Istrita	Buzau	Muntenia	Romania	600
HQ004343	EZROM901-08	RVcoll.08-M672		*Lycaenidae*	*Cupido*	*osiris*	Berca, vulcanii noroiosi Paclele Mici	Buzau	Muntenia	Romania	220
KP871023	EULEP089-14	08-P975		*Lycaenidae*	*Cupido*	*osiris*	Cluj-Napoca, Dealul Feleacului	Cluj	Transylvania	Romania	
HQ004340	EZROM137-08	RV-06-K599		*Lycaenidae*	*Cupido*	*osiris*	Suatu	Cluj	Transylvania	Romania	380
HQ004339	EZROM312-08	RV-07-E674		*Lycaenidae*	*Cupido*	*osiris*	Cluj-Napoca, dealul Bogomaia, Fanatele Clujului	Cluj	Transylvania	Romania	480
HQ004342	EZROM899-08	RVcoll.08-M304		*Lycaenidae*	*Cupido*	*osiris*	Suatu	Cluj	Transylvania	Romania	400
JN276893	EZSPN1064-11	Rvcoll.08-P738	x	*Lycaenidae*	*Cupido*	*osiris*	Puerto de Azaceta	Alava	Basque Country	Spain	1036
KP870476	EZSPM801-12	RVcoll.090211SD04	x	*Lycaenidae*	*Cupido*	*osiris*	Font del Arbre, Confrides	Alicante	Comunidad Valenciana	Spain	795
KP870325	EZSPM802-12	RVcoll.090211SD10	x	*Lycaenidae*	*Cupido*	*osiris*	Font del Arbre, Confrides	Alicante	Comunidad Valenciana	Spain	795
GU676786	EZSPN148-09	RVcoll.08-R049	x	*Lycaenidae*	*Cupido*	*osiris*	Barrancada	Alicante	Comunidad Valenciana	Spain	817
KP870465	EZROM719-08	RV-07-C482	x	*Lycaenidae*	*Cupido*	*osiris*	El Brull, Osona	Barcelona	Catalonia	Spain	835
GU668029	EZSPC370-09	RVcoll.07-C481	x	*Lycaenidae*	*Cupido*	*osiris*	El Brull, Osona	Barcelona	Catalonia	Spain	850
GU669860	EZSPC371-09	RVcoll.08-H293	x	*Lycaenidae*	*Cupido*	*osiris*	Borreda, Bergueda	Barcelona	Catalonia	Spain	850
GU676432	EZSPN713-09	RVcoll.08-L074	x	*Lycaenidae*	*Cupido*	*osiris*	Rambla de las Truchas, Vilafranca	Castellon	Comunidad Valenciana	Spain	1150
HM901533	EZSPC1410-10	RVcoll.09-V121	x	*Lycaenidae*	*Cupido*	*osiris*	Cullar	Granada	Andalusia	Spain	1350
HM901534	EZSPC1411-10	RVcoll.09-V122	x	*Lycaenidae*	*Cupido*	*osiris*	Cullar	Granada	Andalusia	Spain	1350
HM901535	EZSPC1412-10	RVcoll.09-V123	x	*Lycaenidae*	*Cupido*	*osiris*	Cullar	Granada	Andalusia	Spain	1350
HM901536	EZSPC1413-10	RVcoll.09-V124	x	*Lycaenidae*	*Cupido*	*osiris*	Cullar	Granada	Andalusia	Spain	1350
HM901312	EZSPC1111-10	RVcoll.08-M939	x	*Lycaenidae*	*Cupido*	*osiris*	Sobrecastells	Huesca	Aragon	Spain	686
HM901313	EZSPC1112-10	RVcoll.08-M940	x	*Lycaenidae*	*Cupido*	*osiris*	Sobrecastells	Huesca	Aragon	Spain	686
JF848008	EZSPN065-09	RVcoll.09-V719	x	*Lycaenidae*	*Cupido*	*osiris*	Yesero	Huesca	Aragon	Spain	1160
GU676153	EZSPN1015-09	RVcoll.08-M938	x	*Lycaenidae*	*Cupido*	*osiris*	Sobrecastells	Huesca	Aragon	Spain	686
GU676929	EZSPN525-09	RVcoll.08-H995	x	*Lycaenidae*	*Cupido*	*osiris*	Olleros de Alba	Leon	Castilla y Leon	Spain	1050
JF848000	EZSPN057-09	RVcoll.09-V352	x	*Lycaenidae*	*Cupido*	*osiris*	2 km S Prades	Tarragona	Catalonia	Spain	890
JN276892	EZSPN1055-11	Rvcoll.08-M804	x	*Lycaenidae*	*Cupido*	*osiris*	Talavera, Conca de Barbera	Tarragona	Catalonia	Spain	700
GU676509	EZSPN639-09	RVcoll.08-J767	x	*Lycaenidae*	*Cupido*	*osiris*	El Masegar, Moscardon	Teruel	Aragon	Spain	1531
KP870268	EZSPM778-12	RVcoll.050211XV70	x	*Lycaenidae*	*Cupido*	*osiris*	Alrededores, Siete Aguas	Valencia	Comunidad Valenciana	Spain	736
GU676936	EZSPN534-09	RVcoll.08-J010	x	*Lycaenidae*	*Cupido*	*osiris*	Sinarcas, Sinarcas	Valencia	Comunidad Valenciana	Spain	
GU676942	EZSPN540-09	RVcoll.08-J032	x	*Lycaenidae*	*Cupido*	*osiris*	Sinarcas, Sinarcas	Valencia	Comunidad Valenciana	Spain	
FJ663445	LOWA535-06	2005-LOWA-535		*Lycaenidae*	*Cupido*	*osiris*	Karadag		Crimea	Ukraine	
GU669631	EZSPC653-09	RVcoll.07-C654	x	*Lycaenidae*	*Cyaniris*	*semiargus*	Col de Puymorens	Languedoc-Roussillon		France	1825
GU688448	GWORO804-09	BC ZSM Lep 30496		*Lycaenidae*	*Cyaniris*	*semiargus*	Samerberg (RO) Zellboden		Bavaria	Germany	950
HQ563552	FBLMW312-10	BC ZSM Lep 37413		*Lycaenidae*	*Cyaniris*	*semiargus*	Doehlau, Quellitzgrund	Oberfranken	Bavaria	Germany	505
GU686987	GWORL258-09	BC ZSM Lep 18664		*Lycaenidae*	*Cyaniris*	*semiargus*	Grafenwoehr, Grafenwoehr	Oberpfalz	Bavaria	Germany	410
KP870306	FBLMX195-11	BC ZSM Lep 50406		*Lycaenidae*	*Cyaniris*	*semiargus*	Oberstdorf, Oytal	Schwaben	Bavaria	Germany	1025
GU686989	GWORL261-09	BC ZSM Lep 18667		*Lycaenidae*	*Cyaniris*	*semiargus*	Johannesberg, Oberafferbach (Bauersberg)	Unterfranken	Bavaria	Germany	340
HQ004348	EZROM138-08	RV-07-D334		*Lycaenidae*	*Cyaniris*	*semiargus*	Racos	Brasov	Transylvania	Romania	460
HQ004347	EZROM139-08	RV-07-C985		*Lycaenidae*	*Cyaniris*	*semiargus*	Baile Herculane (Pecinisca)	Caras-Severin	Banat	Romania	220
HQ004345	EZROM623-08	RV-07-C925		*Lycaenidae*	*Cyaniris*	*semiargus*	Teregova	Caras-Severin	Banat	Romania	385
HQ004350	EZROM902-08	RVcoll.08-M251		*Lycaenidae*	*Cyaniris*	*semiargus*	Baile Herculane, Pecinisca	Caras-Severin	Banat	Romania	270
HQ004349	EZROM903-08	RVcoll.08-M347		*Lycaenidae*	*Cyaniris*	*semiargus*	1 Km E of Gheorgheni	Harghita	Transylvania	Romania	900
HQ004351	EZROM904-08	RVcoll.08-M599		*Lycaenidae*	*Cyaniris*	*semiargus*	Gheorgheni, Valea Belchia	Harghita	Transylvania	Romania	1000

GenBank accession number	BOLD Process ID	Sample ID	Used in the Iberian dataset	Family	Genus	Species	Exact Site	Region	State/Province	Country	Alt. (m)
HQ004352	EZROM905-08	RVcoll.08-M643		*Lycaenidae*	*Cyaniris*	*semiargus*	Scorota (Retezat Mts.)	Hunedoara	Transylvania	Romania	1560
HQ004346	EZROM568-08	RV-07-D530		*Lycaenidae*	*Cyaniris*	*semiargus*	sat Rohia	Maramures	Maramures	Romania	360
HM901298	EZSPC1097-10	RVcoll.08-L940.1	x	*Lycaenidae*	*Cyaniris*	*semiargus*	Rascafria		Comunidad de Madrid	Spain	1150
HM901299	EZSPC1098-10	RVcoll.08-L941	x	*Lycaenidae*	*Cyaniris*	*semiargus*	Rascafria		Comunidad de Madrid	Spain	1150
HM901628	EZSPC773-10	RVcoll.08-L786.1	x	*Lycaenidae*	*Cyaniris*	*semiargus*	San Andres		Cantabria	Spain	972
HM901631	EZSPC776-10	RVcoll.08-L807	x	*Lycaenidae*	*Cyaniris*	*semiargus*	Hormiguera		Cantabria	Spain	968
GU676199	EZSPN963-09	RVcoll.08-L970	x	*Lycaenidae*	*Cyaniris*	*semiargus*	Oteruelo, Rascafria		Comunidad de Madrid	Spain	1150
GU676329	EZSPN829-09	RVcoll.08-L644	x	*Lycaenidae*	*Cyaniris*	*semiargus*	Hoyocasero, Pinar de Hoyocasero	Avila	Castilla y Leon	Spain	1254
GU669861	EZSPC372-09	RVcoll.07-C473	x	*Lycaenidae*	*Cyaniris*	*semiargus*	El Brull, Osona	Barcelona	Catalonia	Spain	800
GU676466	EZSPN652-09	RVcoll.08-J795	x	*Lycaenidae*	*Cyaniris*	*semiargus*	Acceso Penyagolosa, Vistabella del Maestrazgo	Castellon	Comunidad Valenciana	Spain	1560
HM901742	EZSPC903-10	RVcoll.09-V540	x	*Lycaenidae*	*Cyaniris*	*semiargus*	Loma de San Francisco (Sierra Nevada)	Granada	Andalusia	Spain	2315
HM901743	EZSPC904-10	RVcoll.09-V550	x	*Lycaenidae*	*Cyaniris*	*semiargus*	Loma de San Francisco (Sierra Nevada)	Granada	Andalusia	Spain	2315
GU676080	EZSPM455-09	Rvcoll.09-V585	x	*Lycaenidae*	*Cyaniris*	*semiargus*	Dornajo (Sierra Nevada)	Granada	Andalusia	Spain	2060
GU676081	EZSPM456-09	Rvcoll.09-V535	x	*Lycaenidae*	*Cyaniris*	*semiargus*	Loma de San Francisco (Sierra Nevada)	Granada	Andalusia	Spain	2315
GU676177	EZSPN989-09	RVcoll.08-M033	x	*Lycaenidae*	*Cyaniris*	*semiargus*	Gualda, La Alcarria	Guadalajara	Castilla-La Mancha	Spain	730
GU676083	EZSPM431-09	Rvcoll.09-X266	x	*Lycaenidae*	*Cyaniris*	*semiargus*	Donana, Almonte	Huelva	Andalusia	Spain	
GU676766	EZSPN170-09	RVcoll.08-R060	x	*Lycaenidae*	*Cyaniris*	*semiargus*	K2 Carretera Control	Huelva	Andalusia	Spain	
GU676965	EZSPN261-09	RVcoll.07-W168	x	*Lycaenidae*	*Cyaniris*	*semiargus*	Vallibierna	Huesca	Aragon	Spain	1800
GU669862	EZSPC373-09	RVcoll.08-L498	x	*Lycaenidae*	*Cyaniris*	*semiargus*	Bassa d`Arres, Vall d`Aran	Lleida	Catalonia	Spain	1501
GU669863	EZSPC374-09	RVcoll.08-R121	x	*Lycaenidae*	*Cyaniris*	*semiargus*	Salt de Pitx, Vall d`Aran	Lleida	Catalonia	Spain	1570
GU669856	EZSPC375-09	RVcoll.08-R122	x	*Lycaenidae*	*Cyaniris*	*semiargus*	Salt de Pitx, Vall d`Aran	Lleida	Catalonia	Spain	1570
GU676901	EZSPN496-09	RVcoll.08-H940	x	*Lycaenidae*	*Cyaniris*	*semiargus*	Candelario, Sierra de Candelario	Salamanca	Castilla y Leon	Spain	1300
HM901526	EZSPC1392-10	RVcoll.09-X594	x	*Nymphalidae*	*Danaus*	*chrysippus*	Desembocadura del Fluvia, Sant Pere Pescador, Alt Emporda	Girona	Catalonia	Spain	2
HM901527	EZSPC1393-10	RVcoll.09-X595	x	*Nymphalidae*	*Danaus*	*chrysippus*	Desembocadura del Fluvia, Sant Pere Pescador, Alt Emporda	Girona	Catalonia	Spain	2
HM901528	EZSPC1394-10	RVcoll.09-X596	x	*Nymphalidae*	*Danaus*	*chrysippus*	Desembocadura del Fluvia, Sant Pere Pescador, Alt Emporda	Girona	Catalonia	Spain	2
HM901395	EZSPC1195-10	RVcoll.09-V386	x	*Nymphalidae*	*Danaus*	*chrysippus*	Delta de l`Ebre, La Tancada, Montsia	Tarragona	Catalonia	Spain	1
HM901396	EZSPC1196-10	RVcoll.09-V387	x	*Nymphalidae*	*Danaus*	*chrysippus*	Delta de l`Ebre, La Tancada, Montsia	Tarragona	Catalonia	Spain	1
HM901397	EZSPC1197-10	RVcoll.09-V388	x	*Nymphalidae*	*Danaus*	*chrysippus*	Delta de l`Ebre, La Tancada, Montsia	Tarragona	Catalonia	Spain	1
HM901398	EZSPC1198-10	RVcoll.09-V389	x	*Nymphalidae*	*Danaus*	*chrysippus*	Delta de l`Ebre, La Tancada, Montsia	Tarragona	Catalonia	Spain	1
GU676371	EZSPN783-09	RVcoll.08-L326	x	*Nymphalidae*	*Danaus*	*chrysippus*	Ullalls de Baltasar, Delta de L`Ebre, Montsia	Tarragona	Catalonia	Spain	
GU676360	EZSPN784-09	RVcoll.08-L327	x	*Nymphalidae*	*Danaus*	*chrysippus*	Ullalls de Baltasar, Delta de L`Ebre, Montsia	Tarragona	Catalonia	Spain	
GU675765	EZSPM284-09	SMcoll.140907FY83	x	*Nymphalidae*	*Danaus*	*chrysippus*	El Perellonet, Sueca	Valencia	Comunidad Valenciana	Spain	-1
GU675948	EZSPM286-09	SMcoll.140907FY95	x	*Nymphalidae*	*Danaus*	*chrysippus*	El Perellonet, Sueca	Valencia	Comunidad Valenciana	Spain	-1
JN266597	EZSPC1347-10	RVcoll.130209KL61	x	*Nymphalidae*	*Danaus*	*plexippus*	Terra Mitica,Benidorm	Alicante	Comunidad Valenciana	Spain	
GU675768	EZSPM277-09	SMcoll.08-J600	x	*Nymphalidae*	*Danaus*	*plexippus*	Algeciras	Cadiz	Andalusia	Spain	
KP871034	EZSPM685-12	RVcoll.07-C576	x	*Nymphalidae*	*Danaus*	*plexippus*	Algeciras	Cadiz	Andalusia	Spain	
KP870908	EZSPM686-12	RVcoll.07-C577	x	*Nymphalidae*	*Danaus*	*plexippus*	Algeciras	Cadiz	Andalusia	Spain	
HM391826	FBLMU479-09	BC ZSM Lep 27129		*Nymphalidae*	*Erebia*	*aethiops*	Nationalpark BGL, Wimbachschloss	Oberbayern	Bavaria	Germany	995
JF415695	GWORR684-10	BC ZSM Lep 32180		*Nymphalidae*	*Erebia*	*aethiops*	Jachenau	Oberbayern	Bavaria	Germany	790
HQ563606	FBLMW375-10	BC ZSM Lep 37476		*Nymphalidae*	*Erebia*	*aethiops*	Schmidmuehlen, Vilshof	Oberpfalz	Bavaria	Germany	395
HQ004355	EZROM145-08	RV-07-E597		*Nymphalidae*	*Erebia*	*aethiops*	cabana Babarunca (Muntele Tesla - Muntii Ciucas)	Brasov	Transylvania	Romania	1300
HQ004358	EZROM906-08	RVcoll.06-V649		*Nymphalidae*	*Erebia*	*aethiops*	Racos	Brasov	Transylvania	Romania	500
HQ004354	EZROM144-08	RV-07-E365		*Nymphalidae*	*Erebia*	*aethiops*	Cerna Sat	Caras-Severin	Banat	Romania	525
HQ004353	EZROM143-08	RV-06-M983		*Nymphalidae*	*Erebia*	*aethiops*	Apuseni Mts., Baisoara	Cluj	Transylvania	Romania	590

GenBank accession number	BOLD Process ID	Sample ID	Used in the Iberian dataset	Family	Genus	Species	Exact Site	Region	State/Province	Country	Alt. (m)
HQ004356	EZROM146-08	RV-07-C153		*Nymphalidae*	*Erebia*	*aethiops*	Faget forest (Cluj-Napoca)	Cluj	Transylvania	Romania	680
HQ004361	EZROM909-08	RVcoll.07-E488		*Nymphalidae*	*Erebia*	*aethiops*	Scorota (Retezat Mts.)	Hunedoara	Transylvania	Romania	1600
HQ004357	EZROM577-08	RV-07-C349		*Nymphalidae*	*Erebia*	*aethiops*	Sunatori, valea Colbul	Suceava	Moldavia	Romania	800
HQ004359	EZROM907-08	RVcoll.07-C355		*Nymphalidae*	*Erebia*	*aethiops*	Valea Bistritei, Zugreni chei	Suceava	Moldavia	Romania	750
HQ004360	EZROM908-08	RVcoll.07-C358		*Nymphalidae*	*Erebia*	*aethiops*	Sadova, pas Pascanu	Suceava	Moldavia	Romania	1100
HQ004362	EZROM147-08	RV-06-V703		*Nymphalidae*	*Erebia*	*cassioides*	Retezat Mts., (between Varful Scorota and Plaiul Mic)	Hunedoara	Transylvania	Romania	1925
HQ004363	EZROM148-08	RV-07-E497		*Nymphalidae*	*Erebia*	*cassioides*	Muntele Papusa (Retezat Mts.)	Hunedoara	Transylvania	Romania	1880
HQ004364	EZROM671-08	RV-07-E493		*Nymphalidae*	*Erebia*	*cassioides*	Saua Dragsanului (Retezat Mts.)	Hunedoara	Transylvania	Romania	1780
HQ004365	EZROM910-08	RVcoll.06-V701		*Nymphalidae*	*Erebia*	*cassioides*	Saua Dragsanului (Retezat Mts.)	Hunedoara	Transylvania	Romania	1765
HQ004366	EZROM911-08	RVcoll.07-E494		*Nymphalidae*	*Erebia*	*cassioides*	Saua Dragsanului (Retezat Mts.)	Hunedoara	Transylvania	Romania	1780
HQ004367	EZROM912-08	RVcoll.07-E498		*Nymphalidae*	*Erebia*	*cassioides*	Mt. Papusa (Retezat Mts.)	Hunedoara	Transylvania	Romania	1880
HQ004368	EZROM913-08	RVcoll.08-M635		*Nymphalidae*	*Erebia*	*cassioides*	Scorota (Retezat Mts.)	Hunedoara	Transylvania	Romania	1560
GU676020	EZSPM093-09	RVcoll.08-P420	x	*Nymphalidae*	*Erebia*	*cassioides*	Puerto de San Glorio		Cantabria	Spain	1713
GU676017	EZSPM094-09	RVcoll.08-P421	x	*Nymphalidae*	*Erebia*	*cassioides*	Puerto de San Glorio		Cantabria	Spain	1713
GU675712	EZSPM096-09	RVcoll.08-P423	x	*Nymphalidae*	*Erebia*	*cassioides*	Puerto de San Glorio		Cantabria	Spain	1713
GU675825	EZSPM217-09	RVcoll.08-R404	x	*Nymphalidae*	*Erebia*	*cassioides*	Teverga, Vega Socellares		Asturias	Spain	1800
GU675826	EZSPM218-09	RVcoll.08-R405	x	*Nymphalidae*	*Erebia*	*cassioides*	Teverga, Vega Socellares		Asturias	Spain	1800
HM901728	EZSPC889-10	RVcoll.09-T171	x	*Nymphalidae*	*Erebia*	*cassioides*	Valverde de la Sierra	Leon	Castilla y Leon	Spain	1322
GU675938	EZSPM296-09	RVcoll.08-J838	x	*Nymphalidae*	*Erebia*	*cassioides*	Puerto de San Glorio	Leon	Castilla y Leon	Spain	
HM901777	EZSPC940-10	RVcoll.09-X052	x	*Nymphalidae*	*Erebia*	*cassioides*	Tossa Plana de Lles, La Cerdanya	Lleida	Catalonia	Spain	2750
HM401009	EZSPM557-10	RVcoll.09-X053	x	*Nymphalidae*	*Erebia*	*cassioides*	Tossa Plana de Lles, La Cerdanya	Lleida	Catalonia	Spain	2751
HM401010	EZSPM558-10	RVcoll.09-X054	x	*Nymphalidae*	*Erebia*	*cassioides*	Pla de les Someres, La Cerdanya	Lleida	Catalonia	Spain	2350
HM401011	EZSPM559-10	RVcoll.09-X027	x	*Nymphalidae*	*Erebia*	*cassioides*	Engorgs, La Cerdanya	Lleida	Catalonia	Spain	2310
HM401012	EZSPM560-10	RVcoll.09-X026	x	*Nymphalidae*	*Erebia*	*cassioides*	Engorgs, La Cerdanya	Lleida	Catalonia	Spain	2310
HM901837	EZSPM563-10	RVcoll.09-X024	x	*Nymphalidae*	*Erebia*	*cassioides*	Pleta d`Engorgs, La Cerdanya	Lleida	Catalonia	Spain	2100
HM901838	EZSPM564-10	RVcoll.09-X025	x	*Nymphalidae*	*Erebia*	*cassioides*	Pleta d`Engorgs, La Cerdanya	Lleida	Catalonia	Spain	2100
HM901314	EZSPC1113-10	RVcoll.08-M994	x	*Nymphalidae*	*Erebia*	*epiphron*	Grau Roig			Andorra	2114
HM901315	EZSPC1114-10	RVcoll.08-M995	x	*Nymphalidae*	*Erebia*	*epiphron*	Grau Roig			Andorra	2114
KP870916	EULEP261-14	12-R462		*Nymphalidae*	*Erebia*	*epiphron*	Honister Pass		Cumbria	England	682
KP870577	EULEP262-14	12-R463		*Nymphalidae*	*Erebia*	*epiphron*	Honister Pass		Cumbria	England	682
KP870587	EULEP263-14	12-R464		*Nymphalidae*	*Erebia*	*epiphron*	Honister Pass		Cumbria	England	682
KP870931	EULEP264-14	12-R465		*Nymphalidae*	*Erebia*	*epiphron*	Honister Pass		Cumbria	England	682
KP870625	EULEP170-14	11-I916		*Nymphalidae*	*Erebia*	*epiphron*	Pas de l'Aiguille, Vercors		Isère	France	1648
HQ004377	EZROM917-08	RVcoll.08-M607		*Nymphalidae*	*Erebia*	*epiphron*	3 km above cabana Capra (Fagaras Mts.)	Arges	Muntenia	Romania	1830
HQ004376	EZROM918-08	RVcoll.08-M614		*Nymphalidae*	*Erebia*	*epiphron*	Cabana Capra (Fagaras Mts.)	Arges	Muntenia	Romania	1630
GU669667	EZROM1037-09	RVcoll.06-V706		*Nymphalidae*	*Erebia*	*epiphron*	Retezat Mts., (between Varful Scorota and Plaiul Mic)	Hunedoara	Transylvania	Romania	1900
HQ004372	EZROM150-08	RV-06-V683		*Nymphalidae*	*Erebia*	*epiphron*	Retezat Mts., (between Varful Scorota and Plaiul Mic)	Hunedoara	Transylvania	Romania	1925
HQ004375	EZROM916-08	RVcoll.07-E495		*Nymphalidae*	*Erebia*	*epiphron*	Saua Dragsanului (Retezat Mts.)	Hunedoara	Transylvania	Romania	1780
HQ004371	EZROM149-08	RV-06-M974		*Nymphalidae*	*Erebia*	*epiphron*	Ciucas Mts. (towards Vf. Ciucas)	Prahova	Muntenia	Romania	1650
HQ004370	EZROM151-08	RV-07-E456		*Nymphalidae*	*Erebia*	*epiphron*	Babele (Bucegi Mts.)	Prahova	Muntenia	Romania	2140
HQ004369	EZROM672-08	RV-06-M985		*Nymphalidae*	*Erebia*	*epiphron*	Ciucas Mts. (towards Vf. Ciucas)	Prahova	Muntenia	Romania	1700
HQ004373	EZROM914-08	RVcoll.06-M987		*Nymphalidae*	*Erebia*	*epiphron*	Ciucas Mts. (towards Vf. Ciucas)	Prahova	Muntenia	Romania	1700
HQ004374	EZROM915-08	RVcoll.07-D631		*Nymphalidae*	*Erebia*	*epiphron*	Bucegi mountains	Prahova	Muntenia	Romania	1700
KP870980	EULEP265-14	12-R468		*Nymphalidae*	*Erebia*	*epiphron*	Kenknock		Stirling	Scotland	604
KP870580	EULEP266-14	12-R469		*Nymphalidae*	*Erebia*	*epiphron*	Kenknock		Stirling	Scotland	604
KP870616	EULEP267-14	12-R471		*Nymphalidae*	*Erebia*	*epiphron*	Kenknock		Stirling	Scotland	604
GU669853	EZSPC380-09	RVcoll.08-R174	x	*Nymphalidae*	*Erebia*	*epiphron*	Vallter 2000, Setcases, Ripolles	Girona	Catalonia	Spain	2537
GU675818	EZSPM221-09	RVcoll.08-R410	x	*Nymphalidae*	*Erebia*	*epiphron*	Villamanin, Busgondo	Leon	Castilla y Leon	Spain	1250
GU675815	EZSPM223-09	RVcoll.08-R413	x	*Nymphalidae*	*Erebia*	*epiphron*	Caso, Puerto de Tarna	Leon	Castilla y Leon	Spain	1400
HM901357	EZSPC1157-10	RVcoll.08-R257	x	*Nymphalidae*	*Erebia*	*epiphron*	Port de la Bonaigua, Pallars Sobira	Lleida	Catalonia	Spain	2338
HM901499	EZSPC1365-10	RVcoll.09-X029	x	*Nymphalidae*	*Erebia*	*epiphron*	Pleta d`Engorgs, La Cerdanya	Lleida	Catalonia	Spain	2100
GU669854	EZSPC381-09	RVcoll.07-W121	x	*Nymphalidae*	*Erebia*	*epiphron*	Val d`Hurno, Vall d`Aran	Lleida	Catalonia	Spain	1720
GU669855	EZSPC382-09	RVcoll.08-R259	x	*Nymphalidae*	*Erebia*	*epiphron*	Port de la Bonaigua, Pallars Sobira	Lleida	Catalonia	Spain	2338
GU669848	EZSPC383-09	RVcoll.08-R260	x	*Nymphalidae*	*Erebia*	*epiphron*	Port de la Bonaigua, Pallars Sobira	Lleida	Catalonia	Spain	2338

GenBank accession number	BOLD Process ID	Sample ID	Used in the Iberian dataset	Family	Genus	Species	Exact Site	Region	State/Province	Country	Alt. (m)
GU669849	EZSPC384-09	RVcoll.08-R261	x	*Nymphalidae*	*Erebia*	*epiphron*	Port de la Bonaigua, Pallars Sobira	Lleida	Catalonia	Spain	2338
JF847985	EZSPN036-09	RVcoll.09-T080	x	*Nymphalidae*	*Erebia*	*epiphron*	Val d`Hurno, Vall d`Aran	Lleida	Catalonia	Spain	1800
KP870445	EULEP183-14	11-J460		*Nymphalidae*	*Erebia*	*epiphron*	Albula Hospiz		Graubanden	Switzerland	2363
KP871050	EZSPM808-12	RVcoll.130211WX03	x	*Nymphalidae*	*Erebia*	*epistygne*	Acceso Penyagolosa, Villahermosa del Rio	Castellon	Comunidad Valenciana	Spain	1810
KP870517	EZSPM812-12	RVcoll.130406FP1	x	*Nymphalidae*	*Erebia*	*epistygne*	Fredes, La Pobla de Benifassa	Castellon	Comunidad Valenciana	Spain	1142
HM901243	EZSPC1040-10	RVcoll.08-J340	x	*Nymphalidae*	*Erebia*	*epistygne*	Coll de Comiols, Pallars Jussa	Lleida	Catalonia	Spain	1130
HM901245	EZSPC1042-10	RVcoll.08-J342	x	*Nymphalidae*	*Erebia*	*epistygne*	Coll de Comiols, Pallars Jussa	Lleida	Catalonia	Spain	1130
GU676792	EZSPN145-09	RVcoll.06-G515	x	*Nymphalidae*	*Erebia*	*epistygne*	Coll de Comiols, Pallars Jussa	Lleida	Catalonia	Spain	
GU676530	EZSPN612-09	RVcoll.08-J339	x	*Nymphalidae*	*Erebia*	*epistygne*	Coll de Comiols, Pallars Jussa	Lleida	Catalonia	Spain	1130
HM901839	EZSPN080-09	RVcoll.08-J652	x	*Nymphalidae*	*Erebia*	*epistygne*	Barranco del Toro	Teruel	Aragon	Spain	1443
HM901354	EZSPC1154-10	RVcoll.08-R047	x	*Nymphalidae*	*Erebia*	*epistygne*	Alto del Viso, Alpuente	Valencia	Comunidad Valenciana	Spain	1480
HM901355	EZSPC1155-10	RVcoll.08-R048	x	*Nymphalidae*	*Erebia*	*epistygne*	Alto del Viso, Alpuente	Valencia	Comunidad Valenciana	Spain	1480
GU675832	EZSPM280-09	SMcoll.150308PP52	x	*Nymphalidae*	*Erebia*	*epistygne*	Alto del Viso, Alpuente	Valencia	Comunidad Valenciana	Spain	1401
GU675829	EZSPM282-09	SMcoll.150308PP73	x	*Nymphalidae*	*Erebia*	*epistygne*	Alto del Viso, Alpuente	Valencia	Comunidad Valenciana	Spain	1401
GU669846	EZSPC389-09	RVcoll.08-P089	x	*Nymphalidae*	*Erebia*	*euryale*	Pla de l`Ingla, Vall de Madriu, Grau Roig			Andorra	2150
HM902070	FBLMU380-09	BC ZSM Lep 27030		*Nymphalidae*	*Erebia*	*euryale*	Marquartstein, Hochplatte	Oberbayern	Bavaria	Germany	1400
JF415696	GWORR683-10	BC ZSM Lep 32179		*Nymphalidae*	*Erebia*	*euryale*	Samerberg, Frasdorf	Oberbayern	Bavaria	Germany	1100
JF415697	GWORR687-10	BC ZSM Lep 32183		*Nymphalidae*	*Erebia*	*euryale*	Vorderriss	Oberbayern	Bavaria	Germany	760
HQ004383	EZROM925-08	RVcoll.07-E482		*Nymphalidae*	*Erebia*	*euryale*	2 km S of Cabana Capra (Fagaras Mts.)	Arges	Muntenia	Romania	1350
HQ004388	EZROM933-08	RVcoll.08-M613		*Nymphalidae*	*Erebia*	*euryale*	3 km below cabana Capra (Fagaras Mts.)	Arges	Muntenia	Romania	1350
HQ004382	EZROM155-08	RV-07-C149		*Nymphalidae*	*Erebia*	*euryale*	Corongis (Rodnei Mts.)	Bistrita-Nasaud	Transylvania	Romania	1400
GU669666	EZROM1036-09	RVcoll.06-V690		*Nymphalidae*	*Erebia*	*euryale*	Retezat Mts., (between Varful Scorota and Plaiul Mic)	Hunedoara	Transylvania	Romania	1900
HQ004378	EZROM152-08	RV-06-V671		*Nymphalidae*	*Erebia*	*euryale*	Retezat Mts., (between Varful Scorota and Plaiul Mic)	Hunedoara	Transylvania	Romania	1925
HQ004384	EZROM926-08	RVcoll.07-E490		*Nymphalidae*	*Erebia*	*euryale*	Scorota (Retezat Mts.)	Hunedoara	Transylvania	Romania	1600
GU669656	EZROM1053-09	RVcoll.07-D630		*Nymphalidae*	*Erebia*	*euryale*	Bucegi mountains	Prahova	Muntenia	Romania	1600
GU669651	EZROM1062-09	RVcoll.07-E599		*Nymphalidae*	*Erebia*	*euryale*	towards Varful Ciucas (Ciucas Mts.)	Prahova	Muntenia	Romania	1790
HQ004380	EZROM154-08	RV-07-E581		*Nymphalidae*	*Erebia*	*euryale*	Jepii Mici (Bucegi Mts.)	Prahova	Muntenia	Romania	1800
HQ004391	EZROM919-08	RVcoll.06-N021		*Nymphalidae*	*Erebia*	*euryale*	Ciucas Mts. (towards Vf. Ciucas)	Prahova	Muntenia	Romania	1700
HQ004386	EZROM924-08	RVcoll.07-D670		*Nymphalidae*	*Erebia*	*euryale*	Bucegi mountains, Cota 1600	Prahova	Muntenia	Romania	1600
HQ004385	EZROM927-08	RVcoll.08-M495		*Nymphalidae*	*Erebia*	*euryale*	Cota 1400 m (Bucegi Mts.), Sinaia	Prahova	Muntenia	Romania	1385
GU669664	EZROM1040-09	RVcoll.07-C319		*Nymphalidae*	*Erebia*	*euryale*	Rarau Mts., cabana Rarau, (Cimpulung Moldovenesc)	Suceava	Moldavia	Romania	1550
HQ004381	EZROM578-08	RV-07-C318		*Nymphalidae*	*Erebia*	*euryale*	Rarau Mts., cabana Rarau, (Cimpulung Moldovenesc)	Suceava	Moldavia	Romania	1550
HQ004390	EZROM920-08	RVcoll.07-C322		*Nymphalidae*	*Erebia*	*euryale*	Mts. Rarau, cabana Rarau, (Cimpulung Moldovenesc)	Suceava	Moldavia	Romania	1550
HQ004389	EZROM921-08	RVcoll.07-C325		*Nymphalidae*	*Erebia*	*euryale*	Calimani Mts., Poiana Negrii, Valea Pintii	Suceava	Moldavia	Romania	1100
HQ004387	EZROM922-08	RVcoll.07-C340		*Nymphalidae*	*Erebia*	*euryale*	Mts. Calimani, Gura Haitii, stancile `12 Apostoli`	Suceava	Moldavia	Romania	1750
HQ004379	EZROM153-08	RV-07-E487		*Nymphalidae*	*Erebia*	*euryale*	Buila-Vanturarita (Vanturarita Mare)	Valcea	Oltenia	Romania	1810
GU676013	EZSPM092-09	RVcoll.08-P419	x	*Nymphalidae*	*Erebia*	*euryale*	Puerto de San Glorio		Cantabria	Spain	1710
GU675715	EZSPM097-09	RVcoll.08-P424	x	*Nymphalidae*	*Erebia*	*euryale*	Puerto de San Glorio		Cantabria	Spain	1710
HM901445	EZSPC1250-10	RVcoll.09-V838	x	*Nymphalidae*	*Erebia*	*euryale*	Refugi d`Ulldeter, Ripolles	Girona	Catalonia	Spain	2263.1
HM901497	EZSPC1362-10	RVcoll.09-X008	x	*Nymphalidae*	*Erebia*	*euryale*	Estacio esqui nordic Guils-Fontanals, La Cerdanya	Girona	Catalonia	Spain	1800
GU676102	EZSPM417-09	Rvcoll.09-V837	x	*Nymphalidae*	*Erebia*	*euryale*	Refugi d`Ulldeter, Ripolles	Girona	Catalonia	Spain	
GU675822	EZSPM225-09	RVcoll.08-R417	x	*Nymphalidae*	*Erebia*	*euryale*	Valdeon, Caldevilla	Leon	Castilla y Leon	Spain	1100
GU676134	EZSPM226-09	RVcoll.08-R418	x	*Nymphalidae*	*Erebia*	*euryale*	Valdeon, Caldevilla	Leon	Castilla y Leon	Spain	1100
GU675937	EZSPM295-09	RVcoll.08-J836	x	*Nymphalidae*	*Erebia*	*euryale*	La Una	Leon	Castilla y Leon	Spain	

GenBank accession number	BOLD Process ID	Sample ID	Used in the Iberian dataset	Family	Genus	Species	Exact Site	Region	State/Province	Country	Alt. (m)
KP870652	EZSPM585-12	RVcoll.08-P943	x	*Nymphalidae*	*Erebia*	*euryale*	Puerto de Tarna	Leon	Castilla y Leon	Spain	1229
KP871033	EZSPM586-12	RVcoll.08-P944	x	*Nymphalidae*	*Erebia*	*euryale*	Puerto de Tarna	Leon	Castilla y Leon	Spain	1229
HM901449	EZSPC1255-10	RVcoll.09-V879	x	*Nymphalidae*	*Erebia*	*euryale*	Colomers, Vall d`Aran	Lleida	Catalonia	Spain	
HM901450	EZSPC1256-10	RVcoll.09-V880	x	*Nymphalidae*	*Erebia*	*euryale*	Colomers, Vall d`Aran	Lleida	Catalonia	Spain	
GU669850	EZSPC385-09	RVcoll.08-L477	x	*Nymphalidae*	*Erebia*	*euryale*	Bassa d`Arres, Vall d`Aran	Lleida	Catalonia	Spain	1501
GU669851	EZSPC386-09	RVcoll.08-L478	x	*Nymphalidae*	*Erebia*	*euryale*	Bassa d`Arres, Vall d`Aran	Lleida	Catalonia	Spain	1501
GU669844	EZSPC387-09	RVcoll.08-L479	x	*Nymphalidae*	*Erebia*	*euryale*	Bassa d`Arres, Vall d`Aran	Lleida	Catalonia	Spain	1501
GU669845	EZSPC388-09	RVcoll.08-P393	x	*Nymphalidae*	*Erebia*	*euryale*	Salt de Pitx, Vall d`Aran	Lleida	Catalonia	Spain	1570
GU669847	EZSPC390-09	RVcoll.08-P086	x	*Nymphalidae*	*Erebia*	*gorge*	Pic dels Pessons			Andorra	2860
JF415698	GWOSA781-10	BC ZSM Lep 36647		*Nymphalidae*	*Erebia*	*gorge*	Karwendelgebirge, Soiernspitze	Oberbayern	Bavaria	Germany	2100
HQ565494	GWOSA780-10	BC ZSM Lep 36646		*Nymphalidae*	*Erebia*	*gorge*	Allgaeuer Alpen, Geisshorn	Schwaben	Bavaria	Germany	2100
HQ004392	EZRMN356-08	RVcoll.08-M723		*Nymphalidae*	*Erebia*	*gorge*	Meridional Carpathians, Fagaras Mts., Balea lake	Arges	Muntenia	Romania	2100
HQ004394	EZROM326-08	RV-08-A004		*Nymphalidae*	*Erebia*	*gorge*	Fagaras Mts., Balea Lac	Arges	Muntenia	Romania	2100
HM901656	EZSPC806-10	RVcoll.09-X325	x	*Nymphalidae*	*Erebia*	*gorge*	Camaleno, Liebana		Cantabria	Spain	
HM901657	EZSPC807-10	RVcoll.09-X326	x	*Nymphalidae*	*Erebia*	*gorge*	Vega Redonda		Asturias	Spain	1700
HM901658	EZSPC808-10	RVcoll.09-X327	x	*Nymphalidae*	*Erebia*	*gorge*	Macizo de Andara, Cillorigo-Castro, Liebana		Cantabria	Spain	
HM901659	EZSPC809-10	RVcoll.09-X328	x	*Nymphalidae*	*Erebia*	*gorge*	Macizo de Andara, Cillorigo-Castro, Liebana		Cantabria	Spain	
KP870391	EULEP207-14	12-L025	x	*Nymphalidae*	*Erebia*	*gorge*	Llano de los Pozos, Cangas de Onis, Picos de Europa	Asturias		Spain	1900
KP870333	EULEP208-14	12-L026	x	*Nymphalidae*	*Erebia*	*gorge*	Llano de los Pozos, Cangas de Onis, Picos de Europa	Asturias		Spain	1900
HM901774	EZSPC937-10	RVcoll.09-X030	x	*Nymphalidae*	*Erebia*	*gorge*	Puigpedros, La Cerdanya	Girona	Catalonia	Spain	2900
JF847977	EZSPN028-09	RVcoll.09-T055	x	*Nymphalidae*	*Erebia*	*gorge*	Tuc de la Cigalera, Vall d`Aran	Lleida	Catalonia	Spain	2400
JN278865	EZSPC1331-10	RVcoll.130209KL46	x	*Nymphalidae*	*Erebia*	*gorgone*	Pto. de Sahun,Sahun	Huesca	Aragon	Spain	
KP871019	EZSPM838-12	RVcoll.140711ZG44	x	*Nymphalidae*	*Erebia*	*gorgone*	Ibon de Asnos, Panticosa	Huesca	Aragon	Spain	2060
KP871128	EZSPM842-12	RVcoll.140711ZG95	x	*Nymphalidae*	*Erebia*	*gorgone*	Ibon de Asnos, Panticosa	Huesca	Aragon	Spain	2060
HM901692	EZSPC851-10	RVcoll.08-R253	x	*Nymphalidae*	*Erebia*	*gorgone*	Port de la Bonaigua, Pallars Sobira	Lleida	Catalonia	Spain	2338
HM901693	EZSPC852-10	RVcoll.08-R255	x	*Nymphalidae*	*Erebia*	*gorgone*	Port de la Bonaigua, Pallars Sobira	Lleida	Catalonia	Spain	2338
HM901694	EZSPC853-10	RVcoll.08-R256	x	*Nymphalidae*	*Erebia*	*gorgone*	Port de la Bonaigua, Pallars Sobira	Lleida	Catalonia	Spain	2338
HM901695	EZSPC854-10	RVcoll.08-R258	x	*Nymphalidae*	*Erebia*	*gorgone*	Port de la Bonaigua, Pallars Sobira	Lleida	Catalonia	Spain	2338
HM901755	EZSPC917-10	RVcoll.09-V878	x	*Nymphalidae*	*Erebia*	*gorgone*	Colomers, Vall d`Aran	Lleida	Catalonia	Spain	
GU675819	EZSPM197-09	RVcoll.08-R248	x	*Nymphalidae*	*Erebia*	*gorgone*	Port de la Bonaigua, Pallars Sobira	Lleida	Catalonia	Spain	2338
GU675820	EZSPM198-09	RVcoll.08-R250	x	*Nymphalidae*	*Erebia*	*gorgone*	Port de la Bonaigua, Pallars Sobira	Lleida	Catalonia	Spain	2338
GU675817	EZSPM199-09	RVcoll.08-R251	x	*Nymphalidae*	*Erebia*	*gorgone*	Port de la Bonaigua, Pallars Sobira	Lleida	Catalonia	Spain	2338
KP870440	EZSPM649-12	RVcoll.11-I466	x	*Nymphalidae*	*Erebia*	*hispania*	Road to Veleta (Sierra Nevada)	Granada	Andalusia	Spain	2725
KP870845	EZSPM650-12	RVcoll.11-I468	x	*Nymphalidae*	*Erebia*	*hispania*	Road to Veleta (Sierra Nevada)	Granada	Andalusia	Spain	2725
JN278885	EZSPN1080-11	Rvcoll.09-X192	x	*Nymphalidae*	*Erebia*	*hispania*	Road to Veleta (Sierra Nevada)	Granada	Andalusia	Spain	2640
JN278886	EZSPN1081-11	Rvcoll.09-X193	x	*Nymphalidae*	*Erebia*	*hispania*	Road to Veleta (Sierra Nevada)	Granada	Andalusia	Spain	2640
JN278887	EZSPN1082-11	Rvcoll.09-X194	x	*Nymphalidae*	*Erebia*	*hispania*	Road to Veleta (Sierra Nevada)	Granada	Andalusia	Spain	2640
GU676473	EZSPN672-09	RVcoll.08-J978	x	*Nymphalidae*	*Erebia*	*hispania*	Acceso Veleta, Monachil	Granada	Andalusia	Spain	2737
GU676474	EZSPN674-09	RVcoll.08-J985	x	*Nymphalidae*	*Erebia*	*hispania*	Acceso Veleta, Monachil	Granada	Andalusia	Spain	2737
GU676476	EZSPN675-09	RVcoll.08-J987	x	*Nymphalidae*	*Erebia*	*hispania*	Acceso Veleta, Monachil	Granada	Andalusia	Spain	2737
GU676012	EZSPM022-09	RVcoll.08-P087	x	*Nymphalidae*	*Erebia*	*lefebvrei*	Pic dels Pessans, Escamps			Andorra	2860
HM901371	EZSPC1171-10	RVcoll.09-T119	x	*Nymphalidae*	*Erebia*	*lefebvrei*	Cercanias del Penon Ameno-Vega Socellares, Teverga		Asturias	Spain	1920
HM901372	EZSPC1172-10	RVcoll.09-T120	x	*Nymphalidae*	*Erebia*	*lefebvrei*	Cercanias del Penon Ameno-Vega Socellares, Teverga		Asturias	Spain	1920
GU675718	EZSPM183-09	RVcoll.08-R172	x	*Nymphalidae*	*Erebia*	*lefebvrei*	Vallter 2000, Setcases, Ripolles	Girona	Catalonia	Spain	2538
GU675717	EZSPM184-09	RVcoll.08-R173	x	*Nymphalidae*	*Erebia*	*lefebvrei*	Vallter 2000, Setcases, Ripolles	Girona	Catalonia	Spain	2538
GU675720	EZSPM186-09	RVcoll.08-R182	x	*Nymphalidae*	*Erebia*	*lefebvrei*	Vallter 2000, Setcases, Ripolles	Girona	Catalonia	Spain	2538
KP870347	EZSPM830-12	RVcoll.140711ZG05	x	*Nymphalidae*	*Erebia*	*lefebvrei*	Ibon de Asnos, Panticosa	Huesca	Aragon	Spain	2060
KP870948	EZSPM837-12	RVcoll.140711ZG41	x	*Nymphalidae*	*Erebia*	*lefebvrei*	Ibon de Asnos, Panticosa	Huesca	Aragon	Spain	2060
KP871158	EZSPM841-12	RVcoll.140711ZG84	x	*Nymphalidae*	*Erebia*	*lefebvrei*	Ibon de Asnos, Panticosa	Huesca	Aragon	Spain	2060
HM901356	EZSPC1156-10	RVcoll.08-R225	x	*Nymphalidae*	*Erebia*	*lefebvrei*	Port de la Bonaigua, Pallars Sobira	Lleida	Catalonia	Spain	2338

GenBank accession number	BOLD Process ID	Sample ID	Used in the Iberian dataset	Family	Genus	Species	Exact Site	Region	State/Province	Country	Alt. (m)
HM901365	EZSPC1165-10	RVcoll.09-T057	x	*Nymphalidae*	*Erebia*	*lefebvrei*	Tuc de la Cigalera, Vall d`Aran	Lleida	Catalonia	Spain	2400
GU675719	EZSPM187-09	RVcoll.08-R200	x	*Nymphalidae*	*Erebia*	*lefebvrei*	Port de la Bonaigua, Pallars Sobira	Lleida	Catalonia	Spain	2338
GU675816	EZSPM193-09	RVcoll.08-R223	x	*Nymphalidae*	*Erebia*	*lefebvrei*	Port de la Bonaigua, Pallars Sobira	Lleida	Catalonia	Spain	2338
GU675813	EZSPM194-09	RVcoll.08-R224	x	*Nymphalidae*	*Erebia*	*lefebvrei*	Port de la Bonaigua, Pallars Sobira	Lleida	Catalonia	Spain	2338
GU707099	FBLMU438-09	BC ZSM Lep 27088		*Nymphalidae*	*Erebia*	*ligea*	Diessen, Huebschenried	Oberbayern	Bavaria	Germany	650
JF415699	GWORR688-10	BC ZSM Lep 32184		*Nymphalidae*	*Erebia*	*ligea*	Vorderriss	Oberbayern	Bavaria	Germany	760
HM902069	FBLMU379-09	BC ZSM Lep 27029		*Nymphalidae*	*Erebia*	*ligea*	Schmidmuehlen, Vilshof	Oberpfalz	Bavaria	Germany	395
HQ004398	EZROM928-08	RVcoll.08-M532		*Nymphalidae*	*Erebia*	*ligea*	Valea Feregarilor - Poiana Musuroaie	Caras-Severin	Banat	Romania	350
HQ004399	EZROM929-08	RVcoll.08-M533		*Nymphalidae*	*Erebia*	*ligea*	Valea Feregarilor - Poiana Musuroaie	Caras-Severin	Banat	Romania	350
HQ004400	EZROM930-08	RVcoll.08-M534		*Nymphalidae*	*Erebia*	*ligea*	Valea Feregarilor - Poiana Musuroaie	Caras-Severin	Banat	Romania	350
HQ004401	EZROM931-08	RVcoll.08-M535		*Nymphalidae*	*Erebia*	*ligea*	Valea Feregarilor - Poiana Musuroaie	Caras-Severin	Banat	Romania	350
GU675635	EZROM1075-09	RVcoll.08-L379		*Nymphalidae*	*Erebia*	*ligea*	Gheorgheni, Valea Belchia	Harghita	Transylvania	Romania	1000
HQ004402	EZROM932-08	RVcoll.08-M593		*Nymphalidae*	*Erebia*	*ligea*	Gheorgheni, Valea Belchia	Harghita	Transylvania	Romania	1000
GU675634	EZROM1074-09	RVcoll.08-L377		*Nymphalidae*	*Erebia*	*ligea*	Scorota (Retezat Mts.)	Hunedoara	Transylvania	Romania	1250
HQ004403	EZROM934-08	RVcoll.08-M632		*Nymphalidae*	*Erebia*	*ligea*	Scorota (Retezat Mts.)	Hunedoara	Transylvania	Romania	1560
HQ004404	EZROM935-08	RVcoll.08-M633		*Nymphalidae*	*Erebia*	*ligea*	Scorota (Retezat Mts.)	Hunedoara	Transylvania	Romania	1560
HQ004395	EZROM579-08	RV-07-C316		*Nymphalidae*	*Erebia*	*ligea*	Rosu, Vatra Dornei	Suceava	Moldavia	Romania	1000
HQ004396	EZROM580-08	RV-07-C317		*Nymphalidae*	*Erebia*	*ligea*	Rosu, Vatra Dornei	Suceava	Moldavia	Romania	1000
HQ004397	EZROM923-08	RVcoll.07-C352		*Nymphalidae*	*Erebia*	*ligea*	Sunatori, valea Colbul	Suceava	Moldavia	Romania	800
GU669649	EZROM1060-09	RVcoll.07-E476		*Nymphalidae*	*Erebia*	*manto*	Cabana Capra (Fagaras Mts.)	Arges	Muntenia	Romania	1630
HQ004408	EZROM156-08	RV-07-E470		*Nymphalidae*	*Erebia*	*manto*	Cabana Capra (Fagaras Mts.)	Arges	Muntenia	Romania	1630
HQ004413	EZROM937-08	RVcoll.07-E471		*Nymphalidae*	*Erebia*	*manto*	Cabana Capra (Fagaras Mts.)	Arges	Muntenia	Romania	1630
HQ004410	EZROM938-08	RVcoll.07-E472		*Nymphalidae*	*Erebia*	*manto*	Cabana Capra (Fagaras Mts.)	Arges	Muntenia	Romania	1630
HQ004411	EZROM939-08	RVcoll.07-E475		*Nymphalidae*	*Erebia*	*manto*	Cabana Capra (Fagaras Mts.)	Arges	Muntenia	Romania	1630
GU669650	EZROM1061-09	RVcoll.07-E583		*Nymphalidae*	*Erebia*	*manto*	Jepii Mici (Bucegi Mts.)	Prahova	Muntenia	Romania	1800
HQ004406	EZROM157-08	RV-07-E585		*Nymphalidae*	*Erebia*	*manto*	Jepii Mici (Bucegi Mts.)	Prahova	Muntenia	Romania	1800
HQ004409	EZROM940-08	RVcoll.07-E586		*Nymphalidae*	*Erebia*	*manto*	Jepii Mici (Bucegi Mts.)	Prahova	Muntenia	Romania	1800
HQ004407	EZROM158-08	RV-07-C208		*Nymphalidae*	*Erebia*	*manto*	towards Varful Vanturarita Mare	Valcea	Oltenia	Romania	1545
HQ004405	EZROM625-08	RV-07-C209		*Nymphalidae*	*Erebia*	*manto*	Buila-Vanturarita (Vanturarita Mare)	Valcea	Oltenia	Romania	1700
HQ004412	EZROM936-08	RVcoll.07-C206		*Nymphalidae*	*Erebia*	*manto*	towards Vf. Vanturarita Mare, Buila-Vanturarita	Valcea	Oltenia	Romania	1600
HM901318	EZSPC1117-10	RVcoll.08-P390	x	*Nymphalidae*	*Erebia*	*manto*	Salt de Pitx, Vall d`Aran	Lleida	Catalonia	Spain	1570
GU669840	EZSPC391-09	RVcoll.07-W203	x	*Nymphalidae*	*Erebia*	*manto*	Repetidor pista a Col de Vilac, Vall d`Aran	Lleida	Catalonia	Spain	1700
GU669841	EZSPC392-09	RVcoll.08-P391	x	*Nymphalidae*	*Erebia*	*manto*	Salt de Pitx, Vall d`Aran	Lleida	Catalonia	Spain	1570
GU669842	EZSPC393-09	RVcoll.08-P392	x	*Nymphalidae*	*Erebia*	*manto*	Salt de Pitx, Vall d`Aran	Lleida	Catalonia	Spain	1570
GU669843	EZSPC394-09	RVcoll.08-R108	x	*Nymphalidae*	*Erebia*	*manto*	Salt de Pitx, Vall d`Aran	Lleida	Catalonia	Spain	1570
GU669838	EZSPC395-09	RVcoll.08-R109	x	*Nymphalidae*	*Erebia*	*manto*	Salt de Pitx, Vall d`Aran	Lleida	Catalonia	Spain	1570
KP870586	EZSPM640-12	RVcoll.11-H050	x	*Nymphalidae*	*Erebia*	*manto*	Banhs de Tredos, Vall d`Aran	Lleida	Catalonia	Spain	1800
KP870407	EZSPM644-12	RVcoll.11-H250	x	*Nymphalidae*	*Erebia*	*manto*	Banhs de Tredos, Vall d`Aran	Lleida	Catalonia	Spain	1875
HM393204	GWORA2483-09	BC ZSM Lep 30695		*Nymphalidae*	*Erebia*	*medusa*	Bayer. Wald, Gandsberg	Niederbayern	Bavaria	Germany	850
GU655018	FBLMT878-09	BC ZSM Lep 25438		*Nymphalidae*	*Erebia*	*medusa*	Jexhof, Hackenwiesen	Oberbayern	Bavaria	Germany	570
JF415701	GWORR681-10	BC ZSM Lep 32177		*Nymphalidae*	*Erebia*	*medusa*	Lindenrain, Geretsried	Oberbayern	Bavaria	Germany	610
JF415700	GWORR685-10	BC ZSM Lep 32181		*Nymphalidae*	*Erebia*	*medusa*	Isarauen, Fischerhaeuser	Oberbayern	Bavaria	Germany	482
JX034611	FBLMX271-11	BC ZSM Lep 50482		*Nymphalidae*	*Erebia*	*medusa*	Nittendorf	Oberpfalz	Bavaria	Germany	400
HM393205	GWORA2484-09	BC ZSM Lep 30696		*Nymphalidae*	*Erebia*	*medusa*	Rott a. Lech	Schwaben	Bavaria	Germany	640
JF859942	PHLAC363-10	TLMF Lep 02398		*Nymphalidae*	*Erebia*	*medusa*	Ritten/ Obergruenwald		South Tyrol	Italy	1750
FJ938191	EZROM947-08	RVcoll.08-M615		*Nymphalidae*	*Erebia*	*medusa*	Cabana Capra (Fagaras Mts.)	Arges	Muntenia	Romania	1630
FJ938190	EZROM946-08	RVcoll.08-M522		*Nymphalidae*	*Erebia*	*medusa*	Molhasurile de la Izbuce, Apuseni Mts.	Bihor	Transylvania	Romania	1200
FJ938179	EZRMN339-08	RVcoll.08-H025		*Nymphalidae*	*Erebia*	*medusa*	Corongis (Rodnei Mts.)	Bistrita-Nasaud	Transylvania	Romania	1500
FJ938184	EZROM627-08	RV-07-C150		*Nymphalidae*	*Erebia*	*medusa*	Corongis (Rodnei Mts.)	Bistrita-Nasaud	Transylvania	Romania	1600
FJ938182	EZROM162-08	RV-07-D282		*Nymphalidae*	*Erebia*	*medusa*	Dumbrava Vadului, Vad	Brasov	Transylvania	Romania	495
FJ938181	EZROM159-08	RV-06-K618		*Nymphalidae*	*Erebia*	*medusa*	Baciu forest (Cluj-Napoca)	Cluj	Transylvania	Romania	530
FJ938186	EZROM942-08	RVcoll.06-K622		*Nymphalidae*	*Erebia*	*medusa*	Baciu forest (Cluj-Napoca)	Cluj	Transylvania	Romania	530
FJ938188	EZROM944-08	RVcoll.07-C112		*Nymphalidae*	*Erebia*	*medusa*	Cluj-Napoca, Gheorgheni, dealul Feleacului	Cluj	Transylvania	Romania	600
FJ938189	EZROM945-08	RVcoll.08-M341		*Nymphalidae*	*Erebia*	*medusa*	1 Km E of Gheorgheni	Harghita	Transylvania	Romania	900
FJ938180	EZRMN346-08	RVcoll.08-M625		*Nymphalidae*	*Erebia*	*medusa*	Scorota (Retezat Mts.)	Hunedoara	Transylvania	Romania	1560
FJ938183	EZROM626-08	RV-06-N015		*Nymphalidae*	*Erebia*	*medusa*	Ciucas Mts. (towards Vf. Ciucas)	Prahova	Muntenia	Romania	1560

GenBank accession number	BOLD Process ID	Sample ID	Used in the Iberian dataset	Family	Genus	Species	Exact Site	Region	State/Province	Country	Alt. (m)
FJ938187	EZROM943-08	RVcoll.06-N019		*Nymphalidae*	*Erebia*	*medusa*	Ciucas Mts. (towards Vf. Ciucas)	Prahova	Muntenia	Romania	1700
FJ938185	EZROM581-08	RV-07-C306		*Nymphalidae*	*Erebia*	*medusa*	Radauti	Suceava	Moldavia	Romania	450
HQ968478	PHLAB315-10	TLMF Lep 01115		*Nymphalidae*	*Erebia*	*medusa*	Waltensburg/Vuorz, Spinatsch		Graubunden	Switzerland	1450
HQ968511	PHLAB351-10	TLMF Lep 01151		*Nymphalidae*	*Erebia*	*medusa*	Chur, Pagig		Graubunden	Switzerland	1400
HQ004417	EZROM164-08	RV-06-M981		*Nymphalidae*	*Erebia*	*melas*	Apuseni Mts., Rimetea (Piatra Secuiului)	Alba	Transylvania	Romania	750
HQ004421	EZROM564-08	RV-06-M994		*Nymphalidae*	*Erebia*	*melas*	Apuseni Mts., Rimetea (Piatra Secuiului)	Alba	Transylvania	Romania	750
HQ004422	EZROM948-08	RVcoll.06-M970		*Nymphalidae*	*Erebia*	*melas*	Apuseni Mts., Rimetea (Piatra Secuiului)	Alba	Transylvania	Romania	850
HQ004423	EZROM949-08	RVcoll.06-M992		*Nymphalidae*	*Erebia*	*melas*	Apuseni Mts., Rimetea (Piatra Secuiului)	Alba	Transylvania	Romania	850
HQ004424	EZROM950-08	RVcoll.06-M998		*Nymphalidae*	*Erebia*	*melas*	Apuseni Mts., Rimetea (Piatra Secuiului)	Alba	Transylvania	Romania	850
HQ004425	EZROM951-08	RVcoll.06-N025		*Nymphalidae*	*Erebia*	*melas*	Apuseni Mts., Rimetea (Piatra Secuiului)	Alba	Transylvania	Romania	850
GU675625	EZROM1055-09	RVcoll.07-E315		*Nymphalidae*	*Erebia*	*melas*	Baile Herculane (Pecinisca)	Caras-Severin	Banat	Romania	250
GU675628	EZROM1057-09	RVcoll.07-E389		*Nymphalidae*	*Erebia*	*melas*	Arjana Mountain	Caras-Severin	Banat	Romania	1400
HQ004418	EZROM165-08	RV-07-E309		*Nymphalidae*	*Erebia*	*melas*	Baile Herculane (Pecinisca)	Caras-Severin	Banat	Romania	270
HQ004419	EZROM166-08	RV-07-E387		*Nymphalidae*	*Erebia*	*melas*	Arjana Mountain	Caras-Severin	Banat	Romania	1410
HQ004426	EZROM952-08	RVcoll.07-E311		*Nymphalidae*	*Erebia*	*melas*	Baile Herculane (Pecinisca)	Caras-Severin	Banat	Romania	300
HQ004427	EZROM953-08	RVcoll.07-E314		*Nymphalidae*	*Erebia*	*melas*	Baile Herculane (Pecinisca)	Caras-Severin	Banat	Romania	300
HQ004428	EZROM954-08	RVcoll.07-E319		*Nymphalidae*	*Erebia*	*melas*	Baile Herculane (Pecinisca)	Caras-Severin	Banat	Romania	300
HQ004429	EZROM955-08	RVcoll.07-E388		*Nymphalidae*	*Erebia*	*melas*	Arjana Mountain	Caras-Severin	Banat	Romania	1400
HQ004430	EZROM956-08	RVcoll.07-E390		*Nymphalidae*	*Erebia*	*melas*	Arjana Mountain	Caras-Severin	Banat	Romania	1400
HQ004416	EZRMN351-08	RVcoll.07-D698		*Nymphalidae*	*Erebia*	*melas*	Mt. Hasmasul Mare	Harghita	Transylvania	Romania	1600
HQ004415	EZRMN366-08	RVcoll.08-M733		*Nymphalidae*	*Erebia*	*melas*	Oriental Carpathians, Hasmasul Mare	Harghita	Transylvania	Romania	1700
HQ004414	EZRMN367-08	RVcoll.08-M734		*Nymphalidae*	*Erebia*	*melas*	Oriental Carpathians, Hasmasul Mare	Harghita	Transylvania	Romania	1700
HQ004420	EZROM167-08	RV-07-E492		*Nymphalidae*	*Erebia*	*melas*	Saua Dragsanului (Retezat Mts.)	Hunedoara	Transylvania	Romania	1780
GU676016	EZSPM095-09	RVcoll.08-P422	x	*Nymphalidae*	*Erebia*	*meolans*	Puerto de San Glorio		Cantabria	Spain	1713
GU675711	EZSPM152-09	RVcoll.08-P672	x	*Nymphalidae*	*Erebia*	*meolans*	Gamoniteiru, Sierra de Aramo, Quiros		Asturias	Spain	1712
GU675714	EZSPM153-09	RVcoll.08-P673	x	*Nymphalidae*	*Erebia*	*meolans*	Gamoniteiru, Sierra de Aramo, Quiros		Asturias	Spain	1712
KP870812	EZSPM774-12	RVcoll.050111LP49	x	*Nymphalidae*	*Erebia*	*meolans*	PR-23 de Fuente De, Camaleno		Asturias	Spain	1093
KP870234	EZSPM847-12	RVcoll.150511SD33	x	*Nymphalidae*	*Erebia*	*meolans*	PR-23 de Fuente De, Camaleno		Asturias	Spain	1093
KP870969	EZSPM848-12	RVcoll.150511SD34	x	*Nymphalidae*	*Erebia*	*meolans*	PR-23 de Fuente De, Camaleno		Asturias	Spain	1093
GU675713	EZSPM179-09	RVcoll.08-R144	x	*Nymphalidae*	*Erebia*	*meolans*	Turo de L`Home, Montseny	Barcelona	Catalonia	Spain	1644
JN278861	EZSPC1264-10	RVcoll.090702PE5	x	*Nymphalidae*	*Erebia*	*meolans*	Penyagolosa,Vistabella del Maestrazgo	Castellon	Comunidad Valenciana	Spain	
HM901247	EZSPC1044-10	RVcoll.08-J409	x	*Nymphalidae*	*Erebia*	*meolans*	Coll de la Pal, Serra Cavallera, El Ripolles	Girona	Catalonia	Spain	1500
HM901436	EZSPC1240-10	RVcoll.09-V752	x	*Nymphalidae*	*Erebia*	*meolans*	Valle del Tena, Camino embalse Respomuso, Sallent de Gallego	Huesca	Aragon	Spain	1650
HM901437	EZSPC1241-10	RVcoll.09-V753	x	*Nymphalidae*	*Erebia*	*meolans*	Valle del Tena, Camino embalse Respomuso, Sallent de Gallego	Huesca	Aragon	Spain	1650
KP871055	EZSPM836-12	RVcoll.140711ZG33	x	*Nymphalidae*	*Erebia*	*meolans*	Ibon de Asnos, Panticosa	Huesca	Aragon	Spain	2060
HM901520	EZSPC1386-10	RVcoll.09-X544	x	*Nymphalidae*	*Erebia*	*meolans*	Puerto de San Isidro	Leon	Castilla y Leon	Spain	1215
HM901278	EZSPC1075-10	RVcoll.08-L480	x	*Nymphalidae*	*Erebia*	*meolans*	Bassa d`Arres, Vall d`Aran	Lleida	Catalonia	Spain	1501
HM901448	EZSPC1254-10	RVcoll.09-V877	x	*Nymphalidae*	*Erebia*	*meolans*	Port de la Bonaigua, Pallars Sobira	Lleida	Catalonia	Spain	2162.4
HM901303	EZSPC1102-10	RVcoll.08-L975	x	*Nymphalidae*	*Erebia*	*meolans*	Puerto de Navafria	Segovia	Castilla y Leon	Spain	1782
HM901304	EZSPC1103-10	RVcoll.08-L978	x	*Nymphalidae*	*Erebia*	*meolans*	Navafria, El Nevero	Segovia	Castilla y Leon	Spain	2130
GU676201	EZSPN965-09	RVcoll.08-L976	x	*Nymphalidae*	*Erebia*	*meolans*	Puerto de Navafria	Segovia	Castilla y Leon	Spain	1782
HM901812	EZSPC977-10	RVcoll.06-V748.1	x	*Nymphalidae*	*Erebia*	*neoridas*	Coll de Torn, Saldes, Bergueda	Barcelona	Catalonia	Spain	1910
HM901813	EZSPC978-10	RVcoll.06-V753	x	*Nymphalidae*	*Erebia*	*neoridas*	Coll de la Bena, Gisclareny, Bergueda	Barcelona	Catalonia	Spain	1150
HM901814	EZSPC979-10	RVcoll.06-V754	x	*Nymphalidae*	*Erebia*	*neoridas*	Coll de la Bena, Gisclareny, Bergueda	Barcelona	Catalonia	Spain	1150
GU677034	EZSPN180-09	RVcoll.06-V745	x	*Nymphalidae*	*Erebia*	*neoridas*	Serra Pedregosa, Cadi, Saldes, Bergueda	Barcelona	Catalonia	Spain	2025
GU677032	EZSPN182-09	RVcoll.06-V752	x	*Nymphalidae*	*Erebia*	*neoridas*	Coll de la Bena, Gisclareny, Bergueda	Barcelona	Catalonia	Spain	1150
HM901503	EZSPC1369-10	RVcoll.09-X062	x	*Nymphalidae*	*Erebia*	*neoridas*	Barranc de Montmalus, La Cerdanya	Girona	Catalonia	Spain	1340
HM901504	EZSPC1370-10	RVcoll.09-X063	x	*Nymphalidae*	*Erebia*	*neoridas*	Barranc de Montmalus, La Cerdanya	Girona	Catalonia	Spain	1340
GU676014	EZSPM016-09	RVcoll.08-P013	x	*Nymphalidae*	*Erebia*	*neoridas*	Torre del Riu, Ribera d`Alp, Cerdanya	Girona	Catalonia	Spain	1180
GU676011	EZSPM020-09	RVcoll.08-P043	x	*Nymphalidae*	*Erebia*	*neoridas*	La Valira (near Urus), Cerdanya	Girona	Catalonia	Spain	1300
HM901554	EZSPC695-10	RVcoll.06-V730	x	*Nymphalidae*	*Erebia*	*neoridas*	Prats d`Aguilo, Montella i Martinet, La Cerdanya	Lleida	Catalonia	Spain	2140
HM901555	EZSPC696-10	RVcoll.06-V731	x	*Nymphalidae*	*Erebia*	*neoridas*	Prats d`Aguilo, Montella i Martinet, La Cerdanya	Lleida	Catalonia	Spain	2140

GenBank accession number	BOLD Process ID	Sample ID	Used in the Iberian dataset	Family	Genus	Species	Exact Site	Region	State/Province	Country	Alt. (m)
HM901815	EZSPC980-10	RVcoll.06-V775	x	*Nymphalidae*	*Erebia*	*neoridas*	Boixols, Abella de la Conca, Pallars Jussa	Lleida	Catalonia	Spain	1290
HM901816	EZSPC981-10	RVcoll.06-V777	x	*Nymphalidae*	*Erebia*	*neoridas*	Boixols, Abella de la Conca, Pallars Jussa	Lleida	Catalonia	Spain	1290
GU676015	EZSPM012-09	RVcoll.08-M992	x	*Nymphalidae*	*Erebia*	*oeme*	Grau Roig			Andorra	2114
GU676018	EZSPM003-09	RVcoll.08-M973	x	*Nymphalidae*	*Erebia*	*oeme*	Col de Puymorens	Languedoc-Roussillon		France	1825
JF415703	GWORK563-09	BC ZSM Lep 21893		*Nymphalidae*	*Erebia*	*oeme*	Karwendelgebirge, Mittenwald, Vereinsalm	Oberbayern	Bavaria	Germany	1600
JF415702	GWORR686-10	BC ZSM Lep 32182		*Nymphalidae*	*Erebia*	*oeme*	Samerberg, Lederstuben	Oberbayern	Bavaria	Germany	
FJ938193	EZRMN320-08	RVcoll.08-M623		*Nymphalidae*	*Erebia*	*oeme*	Scorota (Retezat Mts.)	Hunedoara	Transylvania	Romania	1560
FJ938194	EZRMN321-08	RVcoll.08-M624		*Nymphalidae*	*Erebia*	*oeme*	Scorota (Retezat Mts.)	Hunedoara	Transylvania	Romania	1560
FJ938195	EZRMN322-08	RVcoll.08-M626		*Nymphalidae*	*Erebia*	*oeme*	Scorota (Retezat Mts.)	Hunedoara	Transylvania	Romania	1560
FJ938196	EZRMN323-08	RVcoll.08-M631		*Nymphalidae*	*Erebia*	*oeme*	Scorota (Retezat Mts.)	Hunedoara	Transylvania	Romania	1560
FJ938192	EZRMN403-09	RVcoll.06-V691		*Nymphalidae*	*Erebia*	*oeme*	Scorota (Retezat Mts.)	Hunedoara	Transylvania	Romania	1560
KP870780	EZSPC1361-10	RVcoll.09-X007	x	*Nymphalidae*	*Erebia*	*oeme*	Vallsabollera, La Cerdanya	Girona	Catalonia	Spain	1560
JN827881	EZSPC071-09	RVcoll.07-W126	x	*Nymphalidae*	*Erebia*	*oeme*	Bassa d`Arres, Vall d`Aran	Lleida	Catalonia	Spain	1568
JN827880	EZSPC072-09	RVcoll.07-W127	x	*Nymphalidae*	*Erebia*	*oeme*	Bassa d`Arres, Vall d`Aran	Lleida	Catalonia	Spain	1568
JN827882	EZSPC073-09	RVcoll.07-W128	x	*Nymphalidae*	*Erebia*	*oeme*	Bassa d`Arres, Vall d`Aran	Lleida	Catalonia	Spain	1568
JN827884	EZSPC074-09	RVcoll.07-W129	x	*Nymphalidae*	*Erebia*	*oeme*	Bassa d`Arres, Vall d`Aran	Lleida	Catalonia	Spain	1568
JN827883	EZSPC075-09	RVcoll.07-W130	x	*Nymphalidae*	*Erebia*	*oeme*	Bassa d`Arres, Vall d`Aran	Lleida	Catalonia	Spain	1568
HM901505	EZSPC1371-10	RVcoll.09-X137	x	*Nymphalidae*	*Erebia*	*oeme*	Castell Renau, Planes de Son, Pallars Sobira	Lleida	Catalonia	Spain	
HM901829	EZSPC994-10	RVcoll.07-W131	x	*Nymphalidae*	*Erebia*	*oeme*	Bassa d`Arres, Vall d`Aran	Lleida	Catalonia	Spain	1568
HM901830	EZSPC995-10	RVcoll.07-W132	x	*Nymphalidae*	*Erebia*	*oeme*	Bassa d`Arres, Vall d`Aran	Lleida	Catalonia	Spain	1568
GU675823	EZSPM215-09	RVcoll.08-R400	x	*Nymphalidae*	*Erebia*	*palarica*	Ibias, Puerto de El Connio		Asturias	Spain	1300
GU675821	EZSPM228-09	RVcoll.08-R423	x	*Nymphalidae*	*Erebia*	*palarica*	Grandas de Salime, Puerto de El Acebo		Asturias	Spain	1030
KP870766	EZSPM772-12	RVcoll.050111LP32	x	*Nymphalidae*	*Erebia*	*palarica*	Reserva de Muniellos, zona de ribera, Cangas del Narcea		Asturias	Spain	670
KP870523	EZSPM776-12	RVcoll.050111LP78	x	*Nymphalidae*	*Erebia*	*palarica*	Lago de la Isla, Cangas del Narcea		Asturias	Spain	1122
GU676254	EZSPN887-09	RVcoll.08-L797	x	*Nymphalidae*	*Erebia*	*palarica*	Brana Vieja		Cantabria	Spain	1408
GU676269	EZSPN888-09	RVcoll.08-L799	x	*Nymphalidae*	*Erebia*	*palarica*	Brana Vieja		Cantabria	Spain	1408
GU675716	EZSPM155-09	RVcoll.08-P680	x	*Nymphalidae*	*Erebia*	*palarica*	Villadangos de Arbas	Leon	Castilla y Leon	Spain	1311
GU675824	EZSPM222-09	RVcoll.08-R411	x	*Nymphalidae*	*Erebia*	*palarica*	Carmenes, Piedrafita	Leon	Castilla y Leon	Spain	1400
GU675934	EZSPM300-09	RVcoll.08-J847	x	*Nymphalidae*	*Erebia*	*palarica*	Puerto de San Glorio	Leon	Castilla y Leon	Spain	
GU676185	EZSPN973-09	RVcoll.08-L998	x	*Nymphalidae*	*Erebia*	*palarica*	Puerto de Piedrasluengas	Palencia	Castilla y Leon	Spain	1400
GU675661	EZSPC396-09	RVcoll.08-P084	x	*Nymphalidae*	*Erebia*	*pandrose*	Coll de Pessons			Andorra	2790
GU675662	EZSPC397-09	RVcoll.08-P085	x	*Nymphalidae*	*Erebia*	*pandrose*	Coll de Pessons			Andorra	2790
KP871159	EULEP114-14	09-X338	x	*Nymphalidae*	*Erebia*	*pandrose*	Engaït, Cerdanya			Andorra	2350
KP870681	EULEP115-14	09-X339	x	*Nymphalidae*	*Erebia*	*pandrose*	Collada del Pessons			Andorra	2775
HM396370	LEFIA020-10	MM00091		*Nymphalidae*	*Erebia*	*pandrose*	Enontekioe		Lapland	Finland	
HM873189	LEFID392-10	MM06305		*Nymphalidae*	*Erebia*	*pandrose*	Inari	Lapponia inarensis		Finland	
GU686974	GWORL287-09	BC ZSM Lep 21999		*Nymphalidae*	*Erebia*	*pandrose*	Wettersteingebirge, Kreuzeck-Stuibensee	Oberbayern	Bavaria	Germany	1600
JX034616	ODOPE688-11	BC ZSM Lep 50329		*Nymphalidae*	*Erebia*	*pandrose*	Oberstdorf, Nebelhorn/Koblat	Schwaben, Oberallgaeu	Bavaria	Germany	2000
HM425836	PHLAA304-09	TLMF Lep 00344		*Nymphalidae*	*Erebia*	*pandrose*	Passo di Valparola E	Belluno		Italy	2200
HM425891	PHLAA367-09	TLMF Lep 00407		*Nymphalidae*	*Erebia*	*pandrose*	Colle Fauniera Umgebung/ Demonte NW	Cuneo		Italy	2450
JX034634	PHLAF349-11	TLMF Lep 05519		*Nymphalidae*	*Erebia*	*pandrose*	Mavrovo NP, Korab, summit ridge			Macedonia	2700
JX034650	PHLAF350-11	TLMF Lep 05520		*Nymphalidae*	*Erebia*	*pandrose*	Mavrovo NP, Korab, summit ridge			Macedonia	2700
HQ004431	EZROM322-08	RV-08-A000		*Nymphalidae*	*Erebia*	*pandrose*	Fagaras Mts., above cabana Capra	Arges	Muntenia	Romania	1700
HQ004434	EZROM964-08	RVcoll.08-M662		*Nymphalidae*	*Erebia*	*pandrose*	Saua Cobasel, Rodnei Mts.	Bistrita-Nasaud	Transylvania	Romania	1835
HQ004433	EZROM963-08	RVcoll.08-M652		*Nymphalidae*	*Erebia*	*pandrose*	Curmatura Pietrosului, Rodnei Mts.	Maramures	Maramures	Romania	2230
KP870341	EULEP184-14	11-J851		*Nymphalidae*	*Erebia*	*pandrose*	M-ţii Bucegi, Cota 2000m	Prahova	Muntenia	Romania	
HQ004432	EZROM962-08	RVcoll.08-M490		*Nymphalidae*	*Erebia*	*pandrose*	Cota 2000 m (Bucegi Mts.), cabana Miorita	Prahova	Muntenia	Romania	2000
HQ968299	PHLAB1036-10	TLMF Lep 01836		*Nymphalidae*	*Erebia*	*pandrose*	Val Bever, Alp Suvretta		Graubunden	Switzerland	2100
HQ968528	PHLAB372-10	TLMF Lep 01172		*Nymphalidae*	*Erebia*	*pandrose*	Val Bever, Alp Suvretta		Graubunden	Switzerland	2100
HQ004435	EZROM168-08	RV-07-C145		*Nymphalidae*	*Erebia*	*pharte*	Corongis (Rodnei Mts.)	Bistrita-Nasaud	Transylvania	Romania	1650
HQ004436	EZROM169-08	RV-07-C148		*Nymphalidae*	*Erebia*	*pharte*	Corongis (Rodnei Mts.)	Bistrita-Nasaud	Transylvania	Romania	1650

GenBank accession number	BOLD Process ID	Sample ID	Used in the Iberian dataset	Family	Genus	Species	Exact Site	Region	State/Province	Country	Alt. (m)
HQ570388	LEFIF127-10	MM10583		*Nymphalidae*	*Erebia*	*polaris*	Utsjoki	Lapponia inarensis		Finland	
HQ570389	LEFIF128-10	MM10584		*Nymphalidae*	*Erebia*	*polaris*	Utsjoki	Lapponia inarensis		Finland	
JF853668	LEFIJ553-10	MM17178		*Nymphalidae*	*Erebia*	*polaris*	Utsjoki	Lapponia inarensis		Finland	
HM393206	GWORA2485-09	BC ZSM Lep 30697		*Nymphalidae*	*Erebia*	*pronoe*	Sudelfeld, Arzmoos	Oberbayern	Bavaria	Germany	1200
GU686947	GWORL320-09	BC ZSM Lep 22032		*Nymphalidae*	*Erebia*	*pronoe*	Karwendelgebirge, Mittenwald, Vereinsalm	Oberbayern	Bavaria	Germany	1600
JF415705	GWORR682-10	BC ZSM Lep 32178		*Nymphalidae*	*Erebia*	*pronoe*	Brannenburg, Wendelstein	Oberbayern	Bavaria	Germany	1700
JF415704	GWORR689-10	BC ZSM Lep 32185		*Nymphalidae*	*Erebia*	*pronoe*	Brannenburg, Wendelstein	Oberbayern	Bavaria	Germany	1260
HQ004437	EZROM170-08	RV-07-E449		*Nymphalidae*	*Erebia*	*pronoe*	Babele (Bucegi Mts.)	Prahova	Muntenia	Romania	2140
HQ004438	EZROM171-08	RV-07-E550		*Nymphalidae*	*Erebia*	*pronoe*	Saua Dichiului (Bucegi Mts.)	Prahova	Muntenia	Romania	1520
HQ004439	EZROM628-08	RV-07-E588		*Nymphalidae*	*Erebia*	*pronoe*	Jepii Mici (Bucegi Mts.)	Prahova	Muntenia	Romania	1800
HQ004440	EZROM965-08	RVcoll.07-E451		*Nymphalidae*	*Erebia*	*pronoe*	Babele (Bucegi Mts.)	Prahova	Muntenia	Romania	2140
HQ004441	EZROM966-08	RVcoll.07-E452		*Nymphalidae*	*Erebia*	*pronoe*	Babele (Bucegi Mts.)	Prahova	Muntenia	Romania	2140
HQ004442	EZROM967-08	RVcoll.07-E568		*Nymphalidae*	*Erebia*	*pronoe*	Saua Dichiului (Bucegi Mts.)	Prahova	Muntenia	Romania	1550
HQ004443	EZROM968-08	RVcoll.07-E572		*Nymphalidae*	*Erebia*	*pronoe*	Saua Dichiului (Bucegi Mts.)	Prahova	Muntenia	Romania	1550
HQ004444	EZROM969-08	RVcoll.07-E590		*Nymphalidae*	*Erebia*	*pronoe*	Jepii Mici (Bucegi Mts.)	Prahova	Muntenia	Romania	1800
HM901710	EZSPC870-10	RVcoll.09-T121	x	*Nymphalidae*	*Erebia*	*pronoe*	La Raya-Rio Duje, Cabrales		Asturias	Spain	1250
HM901711	EZSPC871-10	RVcoll.09-T122	x	*Nymphalidae*	*Erebia*	*pronoe*	La Raya-Rio Duje, Cabrales		Asturias	Spain	1250
KP871164	EZSPM583-12	RVcoll.08-P941	x	*Nymphalidae*	*Erebia*	*pronoe*	Vega de Sotres		Asturias	Spain	1271
KP870650	EZSPM584-12	RVcoll.08-P942	x	*Nymphalidae*	*Erebia*	*pronoe*	Vega de Sotres		Asturias	Spain	1271
HM901244	EZSPC1041-10	RVcoll.08-M805	x	*Nymphalidae*	*Erebia*	*pronoe*	Banhs de Tredos, Cabana d`Aigues Tortes, Val d`Aran	Lleida	Catalonia	Spain	1820
HM901254	EZSPC1051-10	RVcoll.08-M806	x	*Nymphalidae*	*Erebia*	*pronoe*	Banhs de Tredos, Cabana d`Aigues Tortes, Val d`Aran	Lleida	Catalonia	Spain	1820
HM901258	EZSPC1055-10	RVcoll.08-M807	x	*Nymphalidae*	*Erebia*	*pronoe*	Banhs de Tredos, Cabana d`Aigues Tortes, Val d`Aran	Lleida	Catalonia	Spain	1820
HM901260	EZSPC1057-10	RVcoll.08-M808	x	*Nymphalidae*	*Erebia*	*pronoe*	Banhs de Tredos, Cabana d`Aigues Tortes, Val d`Aran	Lleida	Catalonia	Spain	1820
HM901267	EZSPC1064-10	RVcoll.08-M809	x	*Nymphalidae*	*Erebia*	*pronoe*	Banhs de Tredos, Cabana d`Aigues Tortes, Val d`Aran	Lleida	Catalonia	Spain	1820
HM901783	EZSPC946-10	RVcoll.09-X302	x	*Nymphalidae*	*Erebia*	*pronoe*	Cabana dera Montanheta, Salardu, Naut Aran, VALL D`ARAN	Lleida	Catalonia	Spain	1920
HM901784	EZSPC947-10	RVcoll.09-X303	x	*Nymphalidae*	*Erebia*	*pronoe*	Cabana dera Montanheta, Salardu, Naut Aran, VALL D`ARAN	Lleida	Catalonia	Spain	1920
HM901785	EZSPC949-10	RVcoll.09-X305	x	*Nymphalidae*	*Erebia*	*pronoe*	Pletiu dera Montanheta,Salardu, Naut Aran, VALL D`ARAN	Lleida	Catalonia	Spain	1850
HM901786	EZSPC950-10	RVcoll.09-X306	x	*Nymphalidae*	*Erebia*	*pronoe*	Pletiu dera Montanheta,Salardu, Naut Aran, VALL D`ARAN	Lleida	Catalonia	Spain	1850
HM901787	EZSPC951-10	RVcoll.09-X307	x	*Nymphalidae*	*Erebia*	*pronoe*	Pletiu dera Montanheta,Salardu, Naut Aran, VALL D`ARAN	Lleida	Catalonia	Spain	1850
HM401014	EZSPM565-10	RVcoll.07-C031	x	*Nymphalidae*	*Erebia*	*rondoui*	Puigmal	Languedoc-Roussillon		France	2200
HM401015	EZSPM566-10	RVcoll.07-C034	x	*Nymphalidae*	*Erebia*	*rondoui*	Puigmal	Languedoc-Roussillon		France	2200
HM901556	EZSPC697-10	RVcoll.06-V733	x	*Nymphalidae*	*Erebia*	*rondoui*	Serra Pedregosa, Cadi, Saldes, Bergueda	Barcelona	Catalonia	Spain	2250
KP870785	EZSPC837-10	RVcoll.08-R184	x	*Nymphalidae*	*Erebia*	*rondoui*	Vallter 2000, Setcases, Ripolles	Girona	Catalonia	Spain	2537.7
HM901680	EZSPC838-10	RVcoll.08-R187	x	*Nymphalidae*	*Erebia*	*rondoui*	Vallter 2000, Setcases, Ripolles	Girona	Catalonia	Spain	2537.7
HM901681	EZSPC839-10	RVcoll.08-R188	x	*Nymphalidae*	*Erebia*	*rondoui*	Vallter 2000, Setcases, Ripolles	Girona	Catalonia	Spain	2537.7
HM901752	EZSPC914-10	RVcoll.09-V829	x	*Nymphalidae*	*Erebia*	*rondoui*	Refugi d`Ulldeter, Ripolles	Girona	Catalonia	Spain	2263.1
HM901753	EZSPC915-10	RVcoll.09-V830	x	*Nymphalidae*	*Erebia*	*rondoui*	Refugi d`Ulldeter, Ripolles	Girona	Catalonia	Spain	2263.1
HM401016	EZSPM567-10	RVcoll.08-R175	x	*Nymphalidae*	*Erebia*	*rondoui*	Vallter 2000, Setcases, Ripolles	Girona	Catalonia	Spain	2537.7
HM401017	EZSPM568-10	RVcoll.08-R183	x	*Nymphalidae*	*Erebia*	*rondoui*	Vallter 2000, Setcases, Ripolles	Girona	Catalonia	Spain	2537.7
GU676523	EZSPN617-09	RVcoll.08-J410	x	*Nymphalidae*	*Erebia*	*rondoui*	Coll de la Pal, Serra Cavallera, El Ripolles	Girona	Catalonia	Spain	
JN278866	EZSPC1335-10	RVcoll.130209KL76	x	*Nymphalidae*	*Erebia*	*rondoui*	Ibon de Plan,Saravillo	Huesca	Aragon	Spain	
HM901690	EZSPC849-10	RVcoll.08-R245	x	*Nymphalidae*	*Erebia*	*rondoui*	Port de la Bonaigua, Pallars Sobira	Lleida	Catalonia	Spain	2338

GenBank accession number	BOLD Process ID	Sample ID	Used in the Iberian dataset	Family	Genus	Species	Exact Site	Region	State/Province	Country	Alt. (m)
HM901691	EZSPC850-10	RVcoll.08-R246	x	*Nymphalidae*	*Erebia*	*rondoui*	Port de la Bonaigua, Pallars Sobira	Lleida	Catalonia	Spain	2338
KP871131	EZSPM790-12	RVcoll.070611MH49	x	*Nymphalidae*	*Erebia*	*sthennyo*	Ibon de Asnos, Panticosa	Huesca	Aragon	Spain	2060
KP870308	EZSPM829-12	RVcoll.140711ZG04	x	*Nymphalidae*	*Erebia*	*sthennyo*	Ibon de Asnos, Panticosa	Huesca	Aragon	Spain	2060
KP870954	EZSPM835-12	RVcoll.140711ZG24	x	*Nymphalidae*	*Erebia*	*sthennyo*	Ibon de Asnos, Panticosa	Huesca	Aragon	Spain	2060
KP870655	EZSPC812-10	RVcoll.07-W111	x	*Nymphalidae*	*Erebia*	*sthennyo*	Tuc de la Cigalera, Vall d`Aran	Lleida	Catalonia	Spain	2330
HM901662	EZSPC813-10	RVcoll.07-W115	x	*Nymphalidae*	*Erebia*	*sthennyo*	Val d`Hurno, Vall d`Aran	Lleida	Catalonia	Spain	1720
HM901678	EZSPC835-10	RVcoll.07-W122	x	*Nymphalidae*	*Erebia*	*sthennyo*	Val d`Hurno, Vall d`Aran	Lleida	Catalonia	Spain	1720
HM901679	EZSPC836-10	RVcoll.09-T058	x	*Nymphalidae*	*Erebia*	*sthennyo*	Tuc de la Cigalera, Vall d`Aran	Lleida	Catalonia	Spain	2330
HM901684	EZSPC842-10	RVcoll.09-T059	x	*Nymphalidae*	*Erebia*	*sthennyo*	Tuc de la Cigalera, Vall d`Aran	Lleida	Catalonia	Spain	2330
HM901685	EZSPC843-10	RVcoll.09-T060	x	*Nymphalidae*	*Erebia*	*sthennyo*	Tuc de la Cigalera, Vall d`Aran	Lleida	Catalonia	Spain	2330
HM401013	EZSPM561-10	RVcoll.07-W106	x	*Nymphalidae*	*Erebia*	*sthennyo*	Arriu dera pincela, Vall d`Aran	Lleida	Catalonia	Spain	2167
HM901836	EZSPM562-10	RVcoll.07-W107	x	*Nymphalidae*	*Erebia*	*sthennyo*	Arriu dera pincela, Vall d`Aran	Lleida	Catalonia	Spain	2167
HQ004449	EZROM172-08	RV-07-E473		*Nymphalidae*	*Erebia*	*sudetica*	Cabana Capra (Fagaras Mts.)	Arges	Muntenia	Romania	1630
HQ004450	EZROM173-08	RV-07-E474		*Nymphalidae*	*Erebia*	*sudetica*	Cabana Capra (Fagaras Mts.)	Arges	Muntenia	Romania	1630
HQ004453	EZROM970-08	RVcoll.08-M608		*Nymphalidae*	*Erebia*	*sudetica*	3 km below cabana Capra (Fagaras Mts.)	Arges	Muntenia	Romania	1350
HQ004454	EZROM971-08	RVcoll.08-M617		*Nymphalidae*	*Erebia*	*sudetica*	Cabana Capra (Fagaras Mts.)	Arges	Muntenia	Romania	1630
HQ004455	EZROM972-08	RVcoll.08-M618		*Nymphalidae*	*Erebia*	*sudetica*	Cabana Capra (Fagaras Mts.)	Arges	Muntenia	Romania	1630
HQ004448	EZRMN305-08	RVcoll.08-M789		*Nymphalidae*	*Erebia*	*sudetica*	Dealul Negru (Rodnei Mts.)	Bistrita-Nasaud	Transylvania	Romania	1500
HQ004447	EZRMN313-08	RVcoll.08-M797		*Nymphalidae*	*Erebia*	*sudetica*	Muntele Gojei (Mtii. Rodnei)	Bistrita-Nasaud	Transylvania	Romania	1500
HQ004446	EZRMN325-08	RVcoll.08-H023		*Nymphalidae*	*Erebia*	*sudetica*	Corongis (Rodnei Mts.)	Bistrita-Nasaud	Transylvania	Romania	1500
HQ004445	EZRMN326-08	RVcoll.08-H026		*Nymphalidae*	*Erebia*	*sudetica*	Corongis (Rodnei Mts.)	Bistrita-Nasaud	Transylvania	Romania	1500
HQ004451	EZROM174-08	RV-07-C146		*Nymphalidae*	*Erebia*	*sudetica*	Corongis (Rodnei Mts.)	Bistrita-Nasaud	Transylvania	Romania	1600
HQ004452	EZROM175-08	RV-07-C147		*Nymphalidae*	*Erebia*	*sudetica*	Corongis (Rodnei Mts.)	Bistrita-Nasaud	Transylvania	Romania	1250
GU676019	EZSPM013-09	RVcoll.08-M993	x	*Nymphalidae*	*Erebia*	*triaria*	Grau Roig			Andorra	2114
GU675827	EZSPM224-09	RVcoll.08-R415	x	*Nymphalidae*	*Erebia*	*triaria*	Allande, Puerto de El Palo		Asturias	Spain	1140
GU676261	EZSPN865-09	RVcoll.08-L737	x	*Nymphalidae*	*Erebia*	*triaria*	Puerto de la Morcuera		Comunidad de Madrid	Spain	1604
GU676270	EZSPN889-09	RVcoll.08-L800	x	*Nymphalidae*	*Erebia*	*triaria*	Brana Vieja		Cantabria	Spain	1408
GU676295	EZSPN859-09	RVcoll.08-L720	x	*Nymphalidae*	*Erebia*	*triaria*	Peguerinos	Avila	Castilla y Leon	Spain	1498
HM901263	EZSPC1060-10	RVcoll.08-L073	x	*Nymphalidae*	*Erebia*	*triaria*	Rambla de las Truchas, Vilafranca	Castellon	Comunidad Valenciana	Spain	1150
KP870778	EZSPM811-12	RVcoll.130211WX96	x	*Nymphalidae*	*Erebia*	*triaria*	Acceso Penyagolosa, Villahermosa del Rio	Castellon	Comunidad Valenciana	Spain	1810
GU676497	EZSPN653-09	RVcoll.08-J796	x	*Nymphalidae*	*Erebia*	*triaria*	Acceso Penyagolosa, Vistabella del Maestrazgo	Castellon	Comunidad Valenciana	Spain	1560
HM901379	EZSPC1179-10	RVcoll.09-T151	x	*Nymphalidae*	*Erebia*	*triaria*	Puerto del Cunillo	Cuenca	Castilla-La Mancha	Spain	1620
HM901496	EZSPC1360-10	RVcoll.09-X001	x	*Nymphalidae*	*Erebia*	*triaria*	Vall de Campcardos, La Cerdanya	Girona	Catalonia	Spain	1700
HM901438	EZSPC1242-10	RVcoll.09-V754	x	*Nymphalidae*	*Erebia*	*triaria*	Valle del Tena, Camino embalse Respomuso, Sallent de Gallego	Huesca	Aragon	Spain	1650
GU675830	EZSPM220-09	RVcoll.08-R408	x	*Nymphalidae*	*Erebia*	*triaria*	Carrocera, Piedrasecha	Leon	Castilla y Leon	Spain	1300
HM901369	EZSPC1169-10	RVcoll.09-T079	x	*Nymphalidae*	*Erebia*	*triaria*	Vall d`Hurno, Vall d`Aran	Lleida	Catalonia	Spain	1800
HM901506	EZSPC1372-10	RVcoll.09-X139	x	*Nymphalidae*	*Erebia*	*triaria*	Castell Renau, Planes de Son, Pallars Sobira	Lleida	Catalonia	Spain	
GU675828	EZSPM258-09	RVcoll.08-R471	x	*Nymphalidae*	*Erebia*	*triaria*	Folgoso do Caurel, Alto de Visuna	Lugo	Galicia	Spain	1330
GU676337	EZSPN811-09	RVcoll.08-L524	x	*Nymphalidae*	*Erebia*	*triaria*	Tossal d`Engrillo, Horta de Sant Joan, Terra Alta	Tarragona	Catalonia	Spain	950
GU676338	EZSPN812-09	RVcoll.08-L526	x	*Nymphalidae*	*Erebia*	*triaria*	Tossal d`Engrillo, Horta de Sant Joan, Terra Alta	Tarragona	Catalonia	Spain	950
HM901255	EZSPC1052-10	RVcoll.08-J782	x	*Nymphalidae*	*Erebia*	*triaria*	El Masegar	Teruel	Aragon	Spain	1531
GU676760	EZSPN276-09	RVcoll.08-J655	x	*Nymphalidae*	*Erebia*	*triaria*	Barranco del Toro	Teruel	Aragon	Spain	1443
GU676493	EZSPN644-09	RVcoll.08-J781	x	*Nymphalidae*	*Erebia*	*triaria*	El Masegar, Moscardon	Teruel	Aragon	Spain	1531
HQ565495	GWOSA785-10	BC ZSM Lep 36651		*Nymphalidae*	*Erebia*	*tyndarus*	Allgaeuer Alpen, Kemptner Kopf	Schwaben	Bavaria	Germany	2000
KP870224	EZSPC1266-10	RVcoll.290909LP51	x	*Nymphalidae*	*Erebia*	*zapateri*	Font d`Horta,Vilafranca	Castellon	Comunidad Valenciana	Spain	1049
KP870645	EZSPM542-10	RVcoll.08-P447	x	*Nymphalidae*	*Erebia*	*zapateri*	Villanueva de Alcoron, Alto Tajo	Guadalajara	Castilla-La Mancha	Spain	1345
KP870534	EZSPM543-10	RVcoll.08-P448.1	x	*Nymphalidae*	*Erebia*	*zapateri*	Villanueva de Alcoron, Alto Tajo	Guadalajara	Castilla-La Mancha	Spain	1345
KP870988	EZSPM544-10	RVcoll.08-P456	x	*Nymphalidae*	*Erebia*	*zapateri*	Villanueva de Alcoron, Alto Tajo	Guadalajara	Castilla-La Mancha	Spain	1345
KP871060	EZSPM546-10	RVcoll.08-P458	x	*Nymphalidae*	*Erebia*	*zapateri*	Zaorejas, Alto Tajo	Guadalajara	Castilla-La Mancha	Spain	1100

GenBank accession number	BOLD Process ID	Sample ID	Used in the Iberian dataset	Family	Genus	Species	Exact Site	Region	State/Province	Country	Alt. (m)
KP870514	EZSPM540-10	RVcoll.08-L244.1	x	*Nymphalidae*	*Erebia*	*zapateri*	Ruta de la Sima, El Vallecillo	Teruel	Aragon	Spain	1531
KP871117	EZSPM541-10	RVcoll.08-L259	x	*Nymphalidae*	*Erebia*	*zapateri*	Ruta de la Sima, El Vallecillo	Teruel	Aragon	Spain	1531
HM391840	FBLMU509-09	BC ZSM Lep 27159		*Hesperiidae*	*Erynnis*	*tages*	Neustadt/Donau, Plattenberg	Niederbayern	Bavaria	Germany	370
KP870527	FBLMX127-11	BC ZSM Lep 37608		*Hesperiidae*	*Erynnis*	*tages*	Dollnstein	Oberbayern	Bavaria	Germany	450
JN277566	FBLMX129-11	BC ZSM Lep 37610		*Hesperiidae*	*Erynnis*	*tages*	Schoenau, Jenner/Stahlhaus	Oberbayern	Bavaria	Germany	1750
HQ004462	EZROM973-08	RVcoll.07-D303		*Hesperiidae*	*Erynnis*	*tages*	Dumbrava Vadului, Vad	Brasov	Transylvania	Romania	495
HQ004456	EZROM179-08	RV-07-D240		*Hesperiidae*	*Erynnis*	*tages*	Dealul Istrita, Breaza	Buzau	Muntenia	Romania	540
HQ004463	EZROM974-08	RVcoll.08-M242		*Hesperiidae*	*Erynnis*	*tages*	Baile Herculane, Pecinisca	Caras-Severin	Banat	Romania	270
HQ004457	EZROM176-08	RV-06-K547		*Hesperiidae*	*Erynnis*	*tages*	Badeni	Cluj	Transylvania	Romania	440
HQ004458	EZROM177-08	RV-06-K549		*Hesperiidae*	*Erynnis*	*tages*	Badeni	Cluj	Transylvania	Romania	440
HQ004461	EZROM975-08	RVcoll.08-M309		*Hesperiidae*	*Erynnis*	*tages*	Suatu	Cluj	Transylvania	Romania	400
HQ004460	EZROM976-08	RVcoll.08-M350		*Hesperiidae*	*Erynnis*	*tages*	1 Km E of Gheorgheni	Harghita	Transylvania	Romania	900
HQ004459	EZROM178-08	RV-07-D061		*Hesperiidae*	*Erynnis*	*tages*	Babadag forest	Tulcea	Dobrogea	Romania	115
GU676993	EZSPN230-09	RVcoll.07-W034	x	*Hesperiidae*	*Erynnis*	*tages*	Armada, Lena		Asturias	Spain	
HM901339	EZSPC1139-10	RVcoll.08-P728	x	*Hesperiidae*	*Erynnis*	*tages*	Puerto de Herrera	Alava	Basque Country	Spain	1077
JN277555	EZSPC1284-10	RVcoll.030908SF85	x	*Hesperiidae*	*Erynnis*	*tages*	Puerto de Biar,Biar	Alicante	Comunidad Valenciana	Spain	814
KP870932	EZROM720-08	RV-06-G448	x	*Hesperiidae*	*Erynnis*	*tages*	Can Rovira, El Brull	Barcelona	Catalonia	Spain	830
GU669839	EZSPC398-09	RVcoll.08-J365	x	*Hesperiidae*	*Erynnis*	*tages*	Vallgrassa, Parc Natural del Garraf	Barcelona	Catalonia	Spain	285
GU669834	EZSPC399-09	RVcoll.06-G586	x	*Hesperiidae*	*Erynnis*	*tages*	Sta. Maria del Mont, La Garrotxa	Girona	Catalonia	Spain	425
HM901335	EZSPC1135-10	RVcoll.08-P718	x	*Hesperiidae*	*Erynnis*	*tages*	Alquife	Granada	Andalusia	Spain	1282
GU676618	EZSPN421-09	RVcoll.08-H576	x	*Hesperiidae*	*Erynnis*	*tages*	Gualda, La Alcarria	Guadalajara	Castilla-La Mancha	Spain	742
GU669835	EZSPC400-09	RVcoll.08-M905	x	*Hesperiidae*	*Erynnis*	*tages*	Bassa d`Arres, Vall d`Aran	Lleida	Catalonia	Spain	1500
KP871137	EZSPM771-12	RVcoll.050111LP29	x	*Hesperiidae*	*Erynnis*	*tages*	Ruta de los Molinos, Viveiro	Lugo	Galicia	Spain	32
GU676822	EZSPN111-09	RVcoll.06-A263	x	*Hesperiidae*	*Erynnis*	*tages*	Sierra de Ayllon, San Esteban de Gormaz	Soria	Castilla y Leon	Spain	
GU669836	EZSPC401-09	RVcoll.08-P315	x	*Hesperiidae*	*Erynnis*	*tages*	Farena, Alt Camp	Tarragona	Catalonia	Spain	580
GU675884	EZSPM354-09	RVcoll.08-R067	x	*Hesperiidae*	*Erynnis*	*tages*	La Cerollera	Teruel	Aragon	Spain	781
GU676475	EZSPN641-09	RVcoll.08-J773	x	*Hesperiidae*	*Erynnis*	*tages*	Ruta de la Sima, El Vallecillo	Teruel	Aragon	Spain	1531
GU676940	EZSPN538-09	RVcoll.08-J024	x	*Hesperiidae*	*Erynnis*	*tages*	Muela de Cortes, Cortes de Pallas	Valencia	Comunidad Valenciana	Spain	
GU676705	EZSPN334-09	RVcoll.08-H261	x	*Hesperiidae*	*Erynnis*	*tages*	Mequinenza	Zaragoza	Aragon	Spain	129
HQ004464	EZROM180-08	RV-07-D013		*Pieridae*	*Euchloe*	*ausonia*	Canaraua Fetei (Baneasa)	Constanta	Dobrogea	Romania	15
HQ004465	EZROM977-08	RVcoll.08-M415		*Pieridae*	*Euchloe*	*ausonia*	1 Km NE of Gura Dobrogei reserve	Constanta	Dobrogea	Romania	40
HQ004466	EZROM978-08	RVcoll.08-M416		*Pieridae*	*Euchloe*	*ausonia*	1 Km NE of Gura Dobrogei reserve	Constanta	Dobrogea	Romania	40
HQ004467	EZROM979-08	RVcoll.08-M434		*Pieridae*	*Euchloe*	*ausonia*	1 Km NE of Gura Dobrogei reserve	Constanta	Dobrogea	Romania	40
HQ004468	EZROM980-08	RVcoll.08-M435		*Pieridae*	*Euchloe*	*ausonia*	1 Km NE of Gura Dobrogei reserve	Constanta	Dobrogea	Romania	40
HQ004469	EZROM981-08	RVcoll.08-M455		*Pieridae*	*Euchloe*	*ausonia*	Esechioi forest	Constanta	Dobrogea	Romania	135
GU676773	EZSPN161-09	RVcoll.06-K683	x	*Pieridae*	*Euchloe*	*bazae*	Hermanillas, Depresion de Baza, Baza	Granada	Andalusia	Spain	850
GU676774	EZSPN162-09	RVcoll.06-K684	x	*Pieridae*	*Euchloe*	*bazae*	Hermanillas, Depresion de Baza, Baza	Granada	Andalusia	Spain	850
GU676775	EZSPN163-09	RVcoll.06-K685	x	*Pieridae*	*Euchloe*	*bazae*	Hermanillas, Depresion de Baza, Baza	Granada	Andalusia	Spain	850
GU676776	EZSPN164-09	RVcoll.06-K686	x	*Pieridae*	*Euchloe*	*bazae*	Hermanillas, Depresion de Baza, Baza	Granada	Andalusia	Spain	850
GU676545	EZSPN602-09	RVcoll.08-J300	x	*Pieridae*	*Euchloe*	*bazae*	Barranco de Valcuerna, Candasnos	Huesca	Aragon	Spain	210
KP870796	EZSPN606-09	RVcoll.08-J313	x	*Pieridae*	*Euchloe*	*bazae*	Barranco de Valcuerna, Candasnos	Huesca	Aragon	Spain	210
KP871081	EULEP108-14	09-V797	x	*Pieridae*	*Euchloe*	*bazae*	ca. 6 Km SE of Caspe	Zaragoza	Aragón	Spain	175
KP870846	EULEP122-14	10-A200	x	*Pieridae*	*Euchloe*	*bazae*	ca. 6 km SE of Caspe	Zaragoza	Aragón	Spain	155-215
KP870994	EULEP123-14	10-A201	x	*Pieridae*	*Euchloe*	*bazae*	ca. 6 km SE of Caspe	Zaragoza	Aragón	Spain	155-215
KP871161	EULEP124-14	10-A203	x	*Pieridae*	*Euchloe*	*bazae*	ca. 6 km SE of Caspe	Zaragoza	Aragón	Spain	155-215
KP870967	EULEP125-14	10-A204	x	*Pieridae*	*Euchloe*	*bazae*	ca. 6 km SE of Caspe	Zaragoza	Aragón	Spain	155-215
KP870305	EULEP126-14	10-A205	x	*Pieridae*	*Euchloe*	*bazae*	ca. 6 km SE of Caspe	Zaragoza	Aragón	Spain	155-215
KP870855	EZSPM687-12	RVcoll.12-L592	x	*Pieridae*	*Euchloe*	*belemia*	Nave, Monchique		Algarve	Portugal	
KP870709	EZSPM688-12	RVcoll.12-L593	x	*Pieridae*	*Euchloe*	*belemia*	Nave, Monchique		Algarve	Portugal	
GU676698	EZSPN342-09	RVcoll.08-H305	x	*Pieridae*	*Euchloe*	*belemia*	Aranjuez, Aranjuez		Comunidad de Madrid	Spain	
GU676722	EZSPN318-09	RVcoll.08-R038	x	*Pieridae*	*Euchloe*	*belemia*	Arenal de Almorxo	Alicante	Comunidad Valenciana	Spain	477
JN276232	EZSPC1311-10	RVcoll.130209KL19	x	*Pieridae*	*Euchloe*	*belemia*	Bolonia,Tarifa	Cadiz	Andalusia	Spain	
KP870468	EZSPM626-12	RVcoll.11-D959	x	*Pieridae*	*Euchloe*	*belemia*	Arroyo de Prior, Algeciras	Cadiz	Andalusia	Spain	5

GenBank accession number	BOLD Process ID	Sample ID	Used in the Iberian dataset	Family	Genus	Species	Exact Site	Region	State/Province	Country	Alt. (m)
KP870272	EZSPM627-12	RVcoll.11-D960	x	*Pieridae*	*Euchloe*	*belemia*	Arroyo de Prior, Algeciras	Cadiz	Andalusia	Spain	5
GU675919	EZSPM315-09	RVcoll.08-J871	x	*Pieridae*	*Euchloe*	*belemia*	Villamarco	Leon	Castilla y Leon	Spain	
GU675920	EZSPM316-09	RVcoll.08-J876	x	*Pieridae*	*Euchloe*	*belemia*	Villamoratiel de las Matas	Leon	Castilla y Leon	Spain	
KP871073	EZSPM607-12	RVcoll.11-D641	x	*Pieridae*	*Euchloe*	*belemia*	Colmenar	Malaga	Andalusia	Spain	
KP870588	EZSPM624-12	RVcoll.11-D924	x	*Pieridae*	*Euchloe*	*belemia*	Colmenar	Malaga	Andalusia	Spain	
KP870546	EZSPM625-12	RVcoll.11-D925	x	*Pieridae*	*Euchloe*	*belemia*	Colmenar	Malaga	Andalusia	Spain	
KP870237	EZSPM690-12	RVcoll.12-L551	x	*Pieridae*	*Euchloe*	*crameri*	Porches, Lagoa		Algarve	Portugal	
GU676670	EZSPN376-09	RVcoll.08-H407	x	*Pieridae*	*Euchloe*	*crameri*	Cantoblanco		Comunidad de Madrid	Spain	720
GU676658	EZSPN389-09	RVcoll.08-H442	x	*Pieridae*	*Euchloe*	*crameri*	Puerto de Pozazal		Cantabria	Spain	1026
GU676802	EZSPN130-09	RVcoll.06-G471	x	*Pieridae*	*Euchloe*	*crameri*	La Morera, El Brull, Osona	Barcelona	Catalonia	Spain	880
GU676783	EZSPN155-09	RVcoll.06-H840	x	*Pieridae*	*Euchloe*	*crameri*	Campus UAB, Bellaterra, Valles Occidental	Barcelona	Catalonia	Spain	170
GU676925	EZSPN521-09	RVcoll.08-H987	x	*Pieridae*	*Euchloe*	*crameri*	Plasencia	Caceres	Extremadura	Spain	400
GU676607	EZSPN435-09	RVcoll.08-H616	x	*Pieridae*	*Euchloe*	*crameri*	El Gastor	Cadiz	Andalusia	Spain	660
GU676836	EZSPN099-09	RVcoll.06-A038	x	*Pieridae*	*Euchloe*	*crameri*	Barranco de los Lagartos, Cadiar	Granada	Andalusia	Spain	860
GU676462	EZSPN687-09	RVcoll.08-L015	x	*Pieridae*	*Euchloe*	*crameri*	Ascenso al Veleta, Gueejar Sierra	Granada	Andalusia	Spain	2270
KP870638	EZSPM689-12	RVcoll.12-L543	x	*Pieridae*	*Euchloe*	*crameri*	Campos de cultivo, Cartaya	Huelva	Andalusia	Spain	
KP870549	EZSPN138-09	RVcoll.06-G497	x	*Pieridae*	*Euchloe*	*crameri*	Barranco de Valcuerna, Candasnos	Huesca	Aragon	Spain	210
GU676538	EZSPN608-09	RVcoll.08-J324	x	*Pieridae*	*Euchloe*	*crameri*	Barranco de Valcuerna, Candasnos	Huesca	Aragon	Spain	210
GU675859	EZSPM378-09	RVcoll.08-J870	x	*Pieridae*	*Euchloe*	*crameri*	Villamarco	Leon	Castilla y Leon	Spain	
GU675840	EZSPM274-09	SMcoll.150308PP31	x	*Pieridae*	*Euchloe*	*crameri*	Alto del Viso, Alpuente	Valencia	Comunidad Valenciana	Spain	1401
GU675955	EZSPM007-09	RVcoll.08-M981	x	*Pieridae*	*Euchloe*	*simplonia*	Pas d`Envalira			Andorra	2271
GU675956	EZSPM008-09	RVcoll.08-M982	x	*Pieridae*	*Euchloe*	*simplonia*	Pas d`Envalira			Andorra	2271
HM901723	EZSPC883-10	RVcoll.09-T144	x	*Pieridae*	*Euchloe*	*simplonia*	Laguna de las Verdes, Cabrillanes	Leon	Castilla y Leon	Spain	1750
JF847983	EZSPN034-09	RVcoll.09-T075	x	*Pieridae*	*Euchloe*	*simplonia*	Val d`Hurno, Vall d`Aran	Lleida	Catalonia	Spain	1800
HM901211	EZSPC1007-10	RVcoll.08-H429	x	*Pieridae*	*Euchloe*	*tagis*	Campo Real		Comunidad de Madrid	Spain	731
KP870370	EZSPM691-12	RVcoll.12-L046	x	*Pieridae*	*Euchloe*	*tagis*	Cellorigo, La Rioja		La Rioja	Spain	840
KP870997	EZSPM692-12	RVcoll.12-L047	x	*Pieridae*	*Euchloe*	*tagis*	Cellorigo, La Rioja		La Rioja	Spain	840
GU676690	EZSPN343-09	RVcoll.08-H306	x	*Pieridae*	*Euchloe*	*tagis*	Aranjuez, Aranjuez		Comunidad de Madrid	Spain	
GU676662	EZSPN380-09	RVcoll.08-H427	x	*Pieridae*	*Euchloe*	*tagis*	Campo Real		Comunidad de Madrid	Spain	731
HM901216	EZSPC1012-10	RVcoll.08-H542	x	*Pieridae*	*Euchloe*	*tagis*	Valdeconcha, La Alcarria	Guadalajara	Castilla-La Mancha	Spain	747
GU676625	EZSPN416-09	RVcoll.08-H541	x	*Pieridae*	*Euchloe*	*tagis*	Pastrana, La Alcarria	Guadalajara	Castilla-La Mancha	Spain	702
HM901241	EZSPC1038-10	RVcoll.08-J315	x	*Pieridae*	*Euchloe*	*tagis*	Barranco de Valcuerna, Candasnos	Huesca	Aragon	Spain	210
HM901242	EZSPC1039-10	RVcoll.08-J316	x	*Pieridae*	*Euchloe*	*tagis*	Barranco de Valcuerna, Candasnos	Huesca	Aragon	Spain	210
GU676537	EZSPN607-09	RVcoll.08-J317	x	*Pieridae*	*Euchloe*	*tagis*	Barranco de Valcuerna, Candasnos	Huesca	Aragon	Spain	210
JN276245	EZSPN1106-11	Rvcoll.10-B498	x	*Pieridae*	*Euchloe*	*tagis*	Sant Jordi d`Alfama, Baix Ebre	Tarragona	Catalonia	Spain	100
HM901226	EZSPC1022-10	RVcoll.08-J003	x	*Pieridae*	*Euchloe*	*tagis*	Ayora (Palomera), Ayora	Valencia	Comunidad Valenciana	Spain	900
GU676934	EZSPN532-09	RVcoll.08-J008	x	*Pieridae*	*Euchloe*	*tagis*	Cortes de Pallas (Sierra Martes), Cortes de Pallas	Valencia	Comunidad Valenciana	Spain	
KP871125	EULEP087-14	08-P973		*Lycaenidae*	*Eumedonia*	*eumedon*	Cluj-Napoca, colonia Becaș	Cluj	Transylvania	Romania	
HQ004472	EZROM051-08	RV-06-K670		*Lycaenidae*	*Eumedonia*	*eumedon*	Valea Calda (Cluj-Napoca)	Cluj	Transylvania	Romania	380
HQ004473	EZROM052-08	RV-06-K675		*Lycaenidae*	*Eumedonia*	*eumedon*	Cluj-Napoca dealul Bogomaia Fanatele Clujului	Cluj	Transylvania	Romania	480
HQ004471	EZROM604-08	RV-06-M828		*Lycaenidae*	*Eumedonia*	*eumedon*	Cluj-Napoca, dealul Feleacului, Gheorgheni forest	Cluj	Transylvania	Romania	600
HQ004470	EZROM662-08	RV-06-K674		*Lycaenidae*	*Eumedonia*	*eumedon*	Cluj-Napoca, dealul Bogomaia, Fanatele Clujului	Cluj	Transylvania	Romania	480
KP870291	EULEP150-14	10-C490		*Lycaenidae*	*Eumedonia*	*eumedon*	Cluj-Napoca, Fânațe	Cluj-Napoca	Transylvania	Romania	
KP870232	EZSPC1436-10	RVcoll.09-V147	x	*Lycaenidae*	*Eumedonia*	*eumedon*	Sierra Maria	Almeria	Andalusia	Spain	1700
GU676326	EZSPN826-09	RVcoll.08-L640	x	*Lycaenidae*	*Eumedonia*	*eumedon*	Hoyocasero, Pinar de Hoyocasero	Avila	Castilla y Leon	Spain	1254
GU676327	EZSPN827-09	RVcoll.08-L641	x	*Lycaenidae*	*Eumedonia*	*eumedon*	Hoyocasero, Pinar de Hoyocasero	Avila	Castilla y Leon	Spain	1254
HM901424	EZSPC1228-10	RVcoll.09-V688	x	*Lycaenidae*	*Eumedonia*	*eumedon*	Meranges, La Cerdanya	Girona	Catalonia	Spain	1744

GenBank accession number	BOLD Process ID	Sample ID	Used in the Iberian dataset	Family	Genus	Species	Exact Site	Region	State/Province	Country	Alt. (m)
HM901425	EZSPC1229-10	RVcoll.09-V689	x	*Lycaenidae*	*Eumedonia*	*eumedon*	Meranges, La Cerdanya	Girona	Catalonia	Spain	1744
JF848004	EZSPN061-09	RVcoll.09-V686	x	*Lycaenidae*	*Eumedonia*	*eumedon*	Meranges	Girona	Catalonia	Spain	1750
GU676359	EZSPN796-09	RVcoll.08-L356	x	*Lycaenidae*	*Eumedonia*	*eumedon*	Sierra Tejeda	Granada	Andalusia	Spain	
GU676348	EZSPN797-09	RVcoll.08-L357	x	*Lycaenidae*	*Eumedonia*	*eumedon*	Sierra Tejeda	Granada	Andalusia	Spain	
HM901441	EZSPC1245-10	RVcoll.09-V779	x	*Lycaenidae*	*Eumedonia*	*eumedon*	Valle del Tena, Camino embalse Respomuso, Sallent de Gallego	Huesca	Aragon	Spain	1650
HM901442	EZSPC1247-10	RVcoll.09-V786	x	*Lycaenidae*	*Eumedonia*	*eumedon*	Valle del Tena, Camino embalse Respomuso, Sallent de Gallego	Huesca	Aragon	Spain	1650
HM901377	EZSPC1177-10	RVcoll.09-T145	x	*Lycaenidae*	*Eumedonia*	*eumedon*	Valle de las Partidas, San Emiliano	Leon	Castilla y Leon	Spain	1670
JN827869	EZSPC273-09	RVcoll.08-R229	x	*Lycaenidae*	*Eumedonia*	*eumedon*	Port de la Bonaigua, Pallars Sobira	Lleida	Catalonia	Spain	2338
JN827868	EZSPC274-09	RVcoll.08-R234	x	*Lycaenidae*	*Eumedonia*	*eumedon*	Port de la Bonaigua, Pallars Sobira	Lleida	Catalonia	Spain	2338
JN827867	EZSPC275-09	RVcoll.08-R235	x	*Lycaenidae*	*Eumedonia*	*eumedon*	Port de la Bonaigua, Pallars Sobira	Lleida	Catalonia	Spain	2338
JN827866	EZSPC276-09	RVcoll.08-R236	x	*Lycaenidae*	*Eumedonia*	*eumedon*	Port de la Bonaigua, Pallars Sobira	Lleida	Catalonia	Spain	2338
GU676004	EZSPM004-09	RVcoll.08-M978	x	*Nymphalidae*	*Euphydryas*	*aurinia*	Pas d`Envalira			Andorra	2271
GU676003	EZSPM005-09	RVcoll.08-M979	x	*Nymphalidae*	*Euphydryas*	*aurinia*	Pas d`Envalira			Andorra	2271
GU676001	EZSPM006-09	RVcoll.08-M980	x	*Nymphalidae*	*Euphydryas*	*aurinia*	Pas d`Envalira			Andorra	2271
GU669627	EZSPC657-09	RVcoll.07-C678	x	*Nymphalidae*	*Euphydryas*	*aurinia*	Col de Puymorens	Languedoc-Roussillon		France	1825
JF847994	EZSPN051-09	RVcoll.09-V305	x	*Nymphalidae*	*Euphydryas*	*aurinia*	Porte Puymorens	Languedoc-Roussillon		France	1825
HM391825	FBLMU477-09	BC ZSM Lep 27127		*Nymphalidae*	*Euphydryas*	*aurinia*	Melleck, Ristfeucht	Oberbayern	Bavaria	Germany	660
JF415706	GWORR676-10	BC ZSM Lep 32172		*Nymphalidae*	*Euphydryas*	*aurinia*	Starnberg, Machtlfing	Oberbayern	Bavaria	Germany	715
HQ565491	GWOSA775-10	BC ZSM Lep 36641		*Nymphalidae*	*Euphydryas*	*aurinia*	Murnau, Nordwestufer d. Staffelsee	Oberbayern	Bavaria	Germany	650
KP870901	FBLMX203-11	BC ZSM Lep 50414		*Nymphalidae*	*Euphydryas*	*aurinia*	Oberstdorf, Nebelhorn/Koblat	Schwaben	Bavaria	Germany	2000
GU676571	EZSPN568-09	RVcoll.08-J109	x	*Nymphalidae*	*Euphydryas*	*aurinia*	Ribeira de Varzea (EN311), Varzea Cova, Fafe		Minho	Portugal	500
GU676572	EZSPN569-09	RVcoll.08-J110	x	*Nymphalidae*	*Euphydryas*	*aurinia*	Ribeira de Varzea (EN311), Varzea Cova, Fafe		Minho	Portugal	500
HQ004476	EZROM181-08	RV-07-D246		*Nymphalidae*	*Euphydryas*	*aurinia*	Dumbrava Vadului, Vad	Brasov	Transylvania	Romania	495
HQ004474	EZROM182-08	RV-07-D348		*Nymphalidae*	*Euphydryas*	*aurinia*	Racos	Brasov	Transylvania	Romania	460
HQ004478	EZROM983-08	RVcoll.07-D248		*Nymphalidae*	*Euphydryas*	*aurinia*	Dumbrava Vadului, Vad	Brasov	Transylvania	Romania	495
HQ004479	EZROM984-08	RVcoll.07-D254		*Nymphalidae*	*Euphydryas*	*aurinia*	Dumbrava Vadului, Vad	Brasov	Transylvania	Romania	495
HQ004477	EZROM985-08	RVcoll.07-D270		*Nymphalidae*	*Euphydryas*	*aurinia*	Dumbrava Vadului, Vad	Brasov	Transylvania	Romania	495
HQ004480	EZROM986-08	RVcoll.07-D327		*Nymphalidae*	*Euphydryas*	*aurinia*	Racos	Brasov	Transylvania	Romania	460
HQ004475	EZROM183-08	RV-07-C115		*Nymphalidae*	*Euphydryas*	*aurinia*	Baciu forest (Poiana Santivan) Cluj-Napoca	Cluj	Transylvania	Romania	530
HQ004481	EZROM982-08	RVcoll.07-C116		*Nymphalidae*	*Euphydryas*	*aurinia*	Baciu forest (Poiana Santivan) Cluj-Napoca	Cluj	Transylvania	Romania	530
GU677048	EZSPN357-09	RVcoll.08-H334	x	*Nymphalidae*	*Euphydryas*	*aurinia*	Puerto de Morcuera, Miraflores		Comunidad de Madrid	Spain	
GU676299	EZSPN855-09	RVcoll.08-L706	x	*Nymphalidae*	*Euphydryas*	*aurinia*	Soto del Real		Comunidad de Madrid	Spain	1230
GU669830	EZSPC403-09	RVcoll.06-H892	x	*Nymphalidae*	*Euphydryas*	*aurinia*	Vallforners, Canoves, Valles Oriental	Barcelona	Catalonia	Spain	650
GU669831	EZSPC404-09	RVcoll.08-L404	x	*Nymphalidae*	*Euphydryas*	*aurinia*	Sallent, Bages	Barcelona	Catalonia	Spain	381
GU669832	EZSPC405-09	RVcoll.08-L405	x	*Nymphalidae*	*Euphydryas*	*aurinia*	Sallent, Bages	Barcelona	Catalonia	Spain	381
GU669833	EZSPC406-09	RVcoll.08-L406	x	*Nymphalidae*	*Euphydryas*	*aurinia*	Sallent, Bages	Barcelona	Catalonia	Spain	381
GU676881	EZSPN474-09	RVcoll.08-H907	x	*Nymphalidae*	*Euphydryas*	*aurinia*	Hervas, Carretera Hervas-La Garganta	Caceres	Extremadura	Spain	1007
GU676886	EZSPN482-09	RVcoll.08-H919	x	*Nymphalidae*	*Euphydryas*	*aurinia*	Hervas, Carretera Hervas-La Garganta	Caceres	Extremadura	Spain	774
HM901256	EZSPC1053-10	RVcoll.08-J797	x	*Nymphalidae*	*Euphydryas*	*aurinia*	Acceso Penyagolosa, Vistabella del Maestrazgo	Castellon	Comunidad Valenciana	Spain	1560
HM901405	EZSPC1206-10	RVcoll.09-V474.1	x	*Nymphalidae*	*Euphydryas*	*aurinia*	Sierra de la Sagra	Granada	Andalusia	Spain	1745
GU676118	EZSPM424-09	Rvcoll.09-V599	x	*Nymphalidae*	*Euphydryas*	*aurinia*	Seminario (Sierra Nevada)	Granada	Andalusia	Spain	1520
GU676436	EZSPN709-09	RVcoll.08-L060	x	*Nymphalidae*	*Euphydryas*	*aurinia*	Venta del Molinillo, Huetor de Santillan	Granada	Andalusia	Spain	1236
GU675947	EZSPM322-09	RVcoll.08-J894	x	*Nymphalidae*	*Euphydryas*	*aurinia*	Vega de Espinareda	Leon	Castilla y Leon	Spain	
JN274549	EZSPN1111-11	Rvcoll.10-B599	x	*Nymphalidae*	*Euphydryas*	*aurinia*	Castelo Carbedo, Moreda, Folgoso do Courel	Lugo	Galicia	Spain	690
HM901471	EZSPC1299-10	RVcoll.210306RV7	x	*Nymphalidae*	*Euphydryas*	*aurinia*	Rio Genal,Juzcar	Malaga	Andalusia	Spain	
GU675769	EZSPM242-09	RVcoll.08-R449	x	*Nymphalidae*	*Euphydryas*	*aurinia*	Oia, As Marinas-Mougas	Pontevedra	Galicia	Spain	20
JF848001	EZSPN058-09	RVcoll.09-V353	x	*Nymphalidae*	*Euphydryas*	*aurinia*	2 km S Prades	Tarragona	Catalonia	Spain	890

GenBank accession number	BOLD Process ID	Sample ID	Used in the Iberian dataset	Family	Genus	Species	Exact Site	Region	State/Province	Country	Alt. (m)
GU676511	EZSPN629-09	RVcoll.08-J726	x	*Nymphalidae*	*Euphydryas*	*aurinia*	Rincon del Sardo, Castielfabib	Valencia	Comunidad Valenciana	Spain	1381
HM393211	GWORA2492-09	BC ZSM Lep 30704		*Nymphalidae*	*Euphydryas*	*cynthia*	Sudelfeld, Arzmoos	Oberbayern	Bavaria	Germany	1200
JN274548	EZSPC1310-10	RVcoll.130209KL99	x	*Nymphalidae*	*Euphydryas*	*desfontainii*	Camporreal,Camporreal		Comunidad de Madrid	Spain	
HM901275	EZSPC1072-10	RVcoll.08-L402	x	*Nymphalidae*	*Euphydryas*	*desfontainii*	Sallent, Bages	Barcelona	Catalonia	Spain	381
HM901276	EZSPC1073-10	RVcoll.08-L403	x	*Nymphalidae*	*Euphydryas*	*desfontainii*	Sallent, Bages	Barcelona	Catalonia	Spain	381
GU676349	EZSPN798-09	RVcoll.08-L401	x	*Nymphalidae*	*Euphydryas*	*desfontainii*	Sallent, Bages	Barcelona	Catalonia	Spain	381
HM901306	EZSPC1105-10	RVcoll.08-M002	x	*Nymphalidae*	*Euphydryas*	*desfontainii*	La Puerta, La Alcarria	Guadalajara	Castilla-La Mancha	Spain	809
HM901307	EZSPC1106-10	RVcoll.08-M004	x	*Nymphalidae*	*Euphydryas*	*desfontainii*	La Puerta, La Alcarria	Guadalajara	Castilla-La Mancha	Spain	809
HM901308	EZSPC1107-10	RVcoll.08-M005	x	*Nymphalidae*	*Euphydryas*	*desfontainii*	La Puerta, La Alcarria	Guadalajara	Castilla-La Mancha	Spain	809
GU676188	EZSPN976-09	RVcoll.08-M003	x	*Nymphalidae*	*Euphydryas*	*desfontainii*	La Puerta, La Alcarria	Guadalajara	Castilla-La Mancha	Spain	809
GU676790	EZSPN143-09	RVcoll.06-G511	x	*Nymphalidae*	*Euphydryas*	*desfontainii*	Coll de Comiols, Pallars Jussa	Lleida	Catalonia	Spain	
GU676502	EZSPN640-09	RVcoll.08-J772	x	*Nymphalidae*	*Euphydryas*	*desfontainii*	El Masegar, Moscardon	Teruel	Aragon	Spain	1531
HM901479	EZSPC1319-10	RVcoll.130209KL32	x	*Nymphalidae*	*Euphydryas*	*desfontainii*	Pico de MariSancho,Utiel	Valencia	Comunidad Valenciana	Spain	
KP870335	EZSPM851-12	RVcoll.201210GK02	x	*Nymphalidae*	*Euphydryas*	*desfontainii*	Alrededores, Siete Aguas	Valencia	Comunidad Valenciana	Spain	736
GU676949	EZSPN547-09	RVcoll.08-J049	x	*Nymphalidae*	*Euphydryas*	*desfontainii*	Utiel, Utiel	Valencia	Comunidad Valenciana	Spain	
KP870955	FBLMX202-11	BC ZSM Lep 50413		*Nymphalidae*	*Euphydryas*	*maturna*	Bad Reichenhall, Kirchholz b. Reichenhall	Oberbayern	Bavaria	Germany	550
HQ004487	EZROM990-08	RVcoll.08-M249		*Nymphalidae*	*Euphydryas*	*maturna*	Baile Herculane, Pecinisca	Caras-Severin	Banat	Romania	270
HQ004488	EZROM991-08	RVcoll.08-M250		*Nymphalidae*	*Euphydryas*	*maturna*	Baile Herculane, Pecinisca	Caras-Severin	Banat	Romania	270
HQ004482	EZROM184-08	RV-06-K677		*Nymphalidae*	*Euphydryas*	*maturna*	Badeni	Cluj	Transylvania	Romania	440
HQ004483	EZROM185-08	RV-06-M841		*Nymphalidae*	*Euphydryas*	*maturna*	Cluj-Napoca valea paraului Micus	Cluj	Transylvania	Romania	700
HQ004484	EZROM987-08	RVcoll.06-K678		*Nymphalidae*	*Euphydryas*	*maturna*	Badeni	Cluj	Transylvania	Romania	440
HQ004485	EZROM988-08	RVcoll.06-K680		*Nymphalidae*	*Euphydryas*	*maturna*	Badeni	Cluj	Transylvania	Romania	440
HQ004486	EZROM989-08	RVcoll.06-K681		*Nymphalidae*	*Euphydryas*	*maturna*	Badeni	Cluj	Transylvania	Romania	440
HQ004489	EZROM992-08	RVcoll.08-M363		*Nymphalidae*	*Euphydryas*	*maturna*	Bujoreni monastery (9 Km E of Zorleni)	Vaslui	Moldavia	Romania	240
HM393193	GWORA2470-09	BC ZSM Lep 30682		*Lycaenidae*	*Favonius*	*quercus*	Neuroth	Niederbayern	Bavaria	Germany	
HM393194	GWORA2471-09	BC ZSM Lep 30683		*Lycaenidae*	*Favonius*	*quercus*	Hausmetting	Niederbayern	Bavaria	Germany	370
HM393195	GWORA2472-09	BC ZSM Lep 30684		*Lycaenidae*	*Favonius*	*quercus*	Leak near Regensburg, Thiergarten	Oberpfalz	Bavaria	Germany	330
HQ004836	EZRMN134-08	RVcoll.08-M650		*Lycaenidae*	*Favonius*	*quercus*	Cluj-Napoca, Gheorgheni forest	Cluj	Transylvania	Romania	600
HQ004839	EZRMN307-08	RVcoll.08-M791		*Lycaenidae*	*Favonius*	*quercus*	Dealul Sf. Pavel (Cluj-Napoca)	Cluj	Transylvania	Romania	450
HQ004838	EZRMN327-08	RVcoll.08-H033		*Lycaenidae*	*Favonius*	*quercus*	Hoia forest (Cluj-Napoca)	Cluj	Transylvania	Romania	450
HQ004837	EZRMN352-08	RVcoll.07-D699		*Lycaenidae*	*Favonius*	*quercus*	Pasarea forest (Bucuresti)	Ilfov	Muntenia	Romania	77
KP870907	EZROM741-08	RV-06-G541	x	*Lycaenidae*	*Favonius*	*quercus*	Can Rovira, El Brull	Barcelona	Catalonia	Spain	830
GU669702	EZSPC563-09	RVcoll.08-R294	x	*Lycaenidae*	*Favonius*	*quercus*	Masia del Plans, Vidra, La Garrotxa	Barcelona	Catalonia	Spain	1200
GU669703	EZSPC564-09	RVcoll.08-R320	x	*Lycaenidae*	*Favonius*	*quercus*	Els Refugis del Montseny, St. Pere de Vilamajor, Valles Oriental	Barcelona	Catalonia	Spain	630
HQ562784	EZSPC1253-10	RVcoll.09-V847	x	*Lycaenidae*	*Favonius*	*quercus*	Coll de Banyuls, Alt Emporda	Girona	Catalonia	Spain	393.1
GU669698	EZSPC565-09	RVcoll.07-F332	x	*Lycaenidae*	*Favonius*	*quercus*	El Mont, Albanya, Alt Emporda	Girona	Catalonia	Spain	860
HM901423	EZSPC1227-10	RVcoll.09-V594	x	*Lycaenidae*	*Favonius*	*quercus*	Seminario (Sierra Nevada)	Granada	Andalusia	Spain	1520
GU676094	EZSPM445-09	Rvcoll.09-V593	x	*Lycaenidae*	*Favonius*	*quercus*	Seminario (Sierra Nevada)	Granada	Andalusia	Spain	1520
GU676095	EZSPM446-09	Rvcoll.09-V502	x	*Lycaenidae*	*Favonius*	*quercus*	Sierra de Alfacar	Granada	Andalusia	Spain	
HM901295	EZSPC1094-10	RVcoll.08-L923	x	*Lycaenidae*	*Favonius*	*quercus*	Duron	Guadalajara	Castilla-La Mancha	Spain	720
HM901296	EZSPC1095-10	RVcoll.08-L926	x	*Lycaenidae*	*Favonius*	*quercus*	Duron	Guadalajara	Castilla-La Mancha	Spain	720
KP870725	EZSPN945-09	RVcoll.08-L924	x	*Lycaenidae*	*Favonius*	*quercus*	Duron	Guadalajara	Castilla-La Mancha	Spain	720
GU675944	EZSPM290-09	RVcoll.08-J821	x	*Lycaenidae*	*Favonius*	*quercus*	Columbrianos	Leon	Castilla y Leon	Spain	
GU669699	EZSPC566-09	RVcoll.08-P328	x	*Lycaenidae*	*Favonius*	*quercus*	Perves, Pallars Jussa	Lleida	Catalonia	Spain	1071
HM901234	EZSPC1030-10	RVcoll.08-J086	x	*Lycaenidae*	*Favonius*	*quercus*	Venta del Moro, Venta del Moro	Valencia	Comunidad Valenciana	Spain	
GU676583	EZSPN555-09	RVcoll.08-J068	x	*Lycaenidae*	*Favonius*	*quercus*	Ayora, Ayora	Valencia	Comunidad Valenciana	Spain	
FJ663573	LOWA531-06	2005-LOWA-531		*Lycaenidae*	*Favonius*	*quercus*	Karadag		Crimea	Ukraine	
FJ663572	LOWA765-06	2005-LOWA-765		*Lycaenidae*	*Favonius*	*quercus*	Karadag Reserve		Crimea	Ukraine	

GenBank accession number	BOLD Process ID	Sample ID	Used in the Iberian dataset	Family	Genus	Species	Exact Site	Region	State/Province	Country	Alt. (m)
GU676828	EZSPN109-09	RVcoll.06-A259	x	*Hesperiidae*	*Gegenes*	*nostrodamus*	Laguna de San Juan, Chinchon		Comunidad de Madrid	Spain	
GU676810	EZSPN123-09	RVcoll.06-A310	x	*Hesperiidae*	*Gegenes*	*nostrodamus*	Laguna de San Juan, Chinchon		Comunidad de Madrid	Spain	
GU676812	EZSPN124-09	RVcoll.06-A311	x	*Hesperiidae*	*Gegenes*	*nostrodamus*	Laguna de San Juan, Chinchon		Comunidad de Madrid	Spain	
GU676813	EZSPN125-09	RVcoll.06-A312	x	*Hesperiidae*	*Gegenes*	*nostrodamus*	Laguna de San Juan, Chinchon		Comunidad de Madrid	Spain	
GU676181	EZSPN981-09	RVcoll.08-M021	x	*Hesperiidae*	*Gegenes*	*nostrodamus*	Alicante	Alicante	Comunidad Valenciana	Spain	11
GU676182	EZSPN982-09	RVcoll.08-M022	x	*Hesperiidae*	*Gegenes*	*nostrodamus*	Alicante	Alicante	Comunidad Valenciana	Spain	11
HM901311	EZSPC1110-10	RVcoll.08-M712.1	x	*Hesperiidae*	*Gegenes*	*nostrodamus*	UAB campus, BMS transect no. 9	Barcelona	Catalonia	Spain	170
KP870724	EZSPM590-12	RVcoll.10-A016	x	*Hesperiidae*	*Gegenes*	*nostrodamus*	Algeciras, Arroyo del Prior	Cadiz	Andalusia	Spain	50
KP871169	EULEP230-14	12-L082	x	*Hesperiidae*	*Gegenes*	*nostrodamus*	Benalup	Cádiz	Andalusia	Spain	10
KP870525	EULEP231-14	12-L083	x	*Hesperiidae*	*Gegenes*	*nostrodamus*	Benalup	Cádiz	Andalusia	Spain	10
KP870507	EULEP232-14	12-L084	x	*Hesperiidae*	*Gegenes*	*nostrodamus*	Benalup	Cádiz	Andalusia	Spain	10
KP870386	EULEP233-14	12-L085	x	*Hesperiidae*	*Gegenes*	*nostrodamus*	Benalup	Cádiz	Andalusia	Spain	10
KP870361	EZSPM1040-12	Rvcoll.12-L450	x	*Hesperiidae*	*Gegenes*	*nostrodamus*	Bollullos del Condado	Huelva	Andalusia	Spain	104
GU676353	EZSPN803-09	RVcoll.08-L456	x	*Hesperiidae*	*Gegenes*	*nostrodamus*	Ivars de Noguera	Lleida	Catalonia	Spain	374
HM901486	EZSPC1341-10	RVcoll.130209KL14	x	*Hesperiidae*	*Gegenes*	*nostrodamus*	Tamayo,Venta del Moro	Valencia	Comunidad Valenciana	Spain	
KP870240	EZSPM1041-12	Rvcoll.12-M791	x	*Hesperiidae*	*Gegenes*	*pumilio*	Palmanyola, Mallorca	Mallorca	Balearic Islands	Spain	79.4
KP870350	EZSPM1042-12	Rvcoll.12-M906	x	*Hesperiidae*	*Gegenes*	*pumilio*	Sa Pobla, Mallorca	Mallorca	Balearic Islands	Spain	28.2
GU655017	FBLMT890-09	BC ZSM Lep 25450		*Lycaenidae*	*Glaucopsyche*	*alexis*	Nittendorf	Oberpfalz	Bavaria	Germany	400
HQ004495	EZROM1007-08	RVcoll.08-M518		*Lycaenidae*	*Glaucopsyche*	*alexis*	Apuseni Mts., Rimetea (Piatra Secuiului)	Alba	Transylvania	Romania	850
HQ004497	EZROM1004-08	RVcoll.08-M219		*Lycaenidae*	*Glaucopsyche*	*alexis*	3 Km SW of Lunca Corbului	Arges	Muntenia	Romania	235
HQ004498	EZROM1005-08	RVcoll.08-M375		*Lycaenidae*	*Glaucopsyche*	*alexis*	Buciumeni forest (Radacinesti)	Bacau	Moldavia	Romania	180
HQ004490	EZROM200-08	RV-07-D225		*Lycaenidae*	*Glaucopsyche*	*alexis*	Dealul Istrita, Breaza	Buzau	Muntenia	Romania	540
HQ004491	EZROM201-08	RV-07-D228		*Lycaenidae*	*Glaucopsyche*	*alexis*	Dealul Istrita, Breaza	Buzau	Muntenia	Romania	540
HQ004499	EZROM1001-08	RVcoll.06-K577		*Lycaenidae*	*Glaucopsyche*	*alexis*	Badeni	Cluj	Transylvania	Romania	440
HQ004500	EZROM1002-08	RVcoll.06-K631		*Lycaenidae*	*Glaucopsyche*	*alexis*	Baciu forest (Cluj-Napoca)	Cluj	Transylvania	Romania	530
HQ004493	EZROM197-08	RV-06-K592		*Lycaenidae*	*Glaucopsyche*	*alexis*	Caianu Mic	Cluj	Transylvania	Romania	500
HQ004494	EZROM198-08	RV-06-K595		*Lycaenidae*	*Glaucopsyche*	*alexis*	Suatu	Cluj	Transylvania	Romania	380
HQ004501	EZROM1003-08	RVcoll.07-D001		*Lycaenidae*	*Glaucopsyche*	*alexis*	Canaraua Fetei (Baneasa)	Constanta	Dobrogea	Romania	15
HQ004496	EZROM1006-08	RVcoll.08-M405		*Lycaenidae*	*Glaucopsyche*	*alexis*	Macin Mts. (3 Km S of Jijila)	Tulcea	Dobrogea	Romania	230
HQ004492	EZROM199-08	RV-07-D030		*Lycaenidae*	*Glaucopsyche*	*alexis*	Babadag forest	Tulcea	Dobrogea	Romania	115
GU676298	EZSPN854-09	RVcoll.08-L704	x	*Lycaenidae*	*Glaucopsyche*	*alexis*	Manzanares el Real		Comunidad de Madrid	Spain	908
GU675783	EZSPM248-09	RVcoll.08-R460	x	*Lycaenidae*	*Glaucopsyche*	*alexis*	Santiso, Quintelas-Bajada Ferry	A Coruna	Galicia	Spain	266
KP870342	EZROM721-08	RV-06-G434	x	*Lycaenidae*	*Glaucopsyche*	*alexis*	Can Rovira, El Brull	Barcelona	Catalonia	Spain	830
GU669820	EZSPC417-09	RVcoll.08-J371	x	*Lycaenidae*	*Glaucopsyche*	*alexis*	Vallgrassa, Parc Natural del Garraf	Barcelona	Catalonia	Spain	285
GU669821	EZSPC418-09	RVcoll.08-J372	x	*Lycaenidae*	*Glaucopsyche*	*alexis*	Vallgrassa, Parc Natural del Garraf	Barcelona	Catalonia	Spain	285
GU669814	EZSPC419-09	RVcoll.08-L410	x	*Lycaenidae*	*Glaucopsyche*	*alexis*	Sallent, Bages	Barcelona	Catalonia	Spain	381
HM901266	EZSPC1063-10	RVcoll.08-L112	x	*Lycaenidae*	*Glaucopsyche*	*alexis*	Puerto de las Cabrillas, Portell de Morella	Castellon	Comunidad Valenciana	Spain	1301
GU676431	EZSPN712-09	RVcoll.08-L066	x	*Lycaenidae*	*Glaucopsyche*	*alexis*	Rambla de las Truchas, Vilafranca	Castellon	Comunidad Valenciana	Spain	1150
GU669815	EZSPC420-09	RVcoll.08-L434	x	*Lycaenidae*	*Glaucopsyche*	*alexis*	Baltarga, La Cerdenya	Girona	Catalonia	Spain	1121
HM901239	EZSPC1035-10	RVcoll.08-J210	x	*Lycaenidae*	*Glaucopsyche*	*alexis*	Sierra Pelada, Parapanda, Illora	Granada	Andalusia	Spain	1000
GU676621	EZSPN424-09	RVcoll.08-H587	x	*Lycaenidae*	*Glaucopsyche*	*alexis*	Gualda, La Alcarria	Guadalajara	Castilla-La Mancha	Spain	742
GU675784	EZSPM268-09	RVcoll.08-R485	x	*Lycaenidae*	*Glaucopsyche*	*alexis*	Pedrafita do Cebreiro, Serra do Caurel-Hospital	Lugo	Galicia	Spain	1230
HM901248	EZSPC1045-10	RVcoll.08-J661	x	*Lycaenidae*	*Glaucopsyche*	*alexis*	Barranco del Toro	Teruel	Aragon	Spain	1443
GU676510	EZSPN628-09	RVcoll.08-J721	x	*Lycaenidae*	*Glaucopsyche*	*alexis*	Rincon del Sardo, Castielfabib	Valencia	Comunidad Valenciana	Spain	1381

GenBank accession number	BOLD Process ID	Sample ID	Used in the Iberian dataset	Family	Genus	Species	Exact Site	Region	State/Province	Country	Alt. (m)
GU676574	EZSPN571-09	RVcoll.08-J118	x	*Lycaenidae*	*Glaucopsyche*	*melanops*	Ribeira de Varzea (EN311), Varzea Cova, Fafe		Minho	Portugal	500
KP870921	EZSPM694-12	RVcoll.12-L546	x	*Lycaenidae*	*Glaucopsyche*	*melanops*	Porches, Lagoa		Algarve	Portugal	
GU675785	EZSPM251-09	RVcoll.08-R463	x	*Lycaenidae*	*Glaucopsyche*	*melanops*	Santiso, Quintelas-Bajada Ferry	A Coruna	Galicia	Spain	266
GU676668	EZSPN374-09	RVcoll.08-H400	x	*Lycaenidae*	*Glaucopsyche*	*melanops*	Puebla de la Reina	Badajoz	Extremadura	Spain	365
JN276887	EZSPN1032-11	Rvcoll.06-G443	x	*Lycaenidae*	*Glaucopsyche*	*melanops*	Can Rovira, El Brull, Osona	Barcelona	Catalonia	Spain	830
JN276888	EZSPN1035-11	Rvcoll.06-G516	x	*Lycaenidae*	*Glaucopsyche*	*melanops*	Santuari de Puiglagulla, Vilalleons, Osona	Barcelona	Catalonia	Spain	720
JN276890	EZSPN1043-11	Rvcoll.08-J399	x	*Lycaenidae*	*Glaucopsyche*	*melanops*	Sallent, Bages	Barcelona	Catalonia	Spain	381
GU676592	EZSPN457-09	RVcoll.08-H663	x	*Lycaenidae*	*Glaucopsyche*	*melanops*	Ubrique, Puerto de Galis	Cadiz	Andalusia	Spain	579
GU676846	EZSPN086-09	RVcoll.06-A005	x	*Lycaenidae*	*Glaucopsyche*	*melanops*	Road from Ferreira to Puerto de La Ragua	Granada	Andalusia	Spain	1600
GU676838	EZSPN094-09	RVcoll.06-A024	x	*Lycaenidae*	*Glaucopsyche*	*melanops*	Mecina Bombaron, Alpujarras	Granada	Andalusia	Spain	1150
GU676458	EZSPN691-09	RVcoll.08-L022	x	*Lycaenidae*	*Glaucopsyche*	*melanops*	Casillas de Rojas, Gueejar Sierra	Granada	Andalusia	Spain	1733
GU676597	EZSPN450-09	RVcoll.08-H645	x	*Lycaenidae*	*Glaucopsyche*	*melanops*	Benadalid	Malaga	Andalusia	Spain	790
GU675786	EZSPM253-09	RVcoll.08-R465	x	*Lycaenidae*	*Glaucopsyche*	*melanops*	Tui, Rio Penedo	Pontevedra	Galicia	Spain	30
GU676306	EZSPN845-09	RVcoll.08-L682	x	*Lycaenidae*	*Glaucopsyche*	*melanops*	Candelario, Sierra de Candelario	Salamanca	Castilla y Leon	Spain	1300
GU676666	EZSPN372-09	RVcoll.08-H397	x	*Lycaenidae*	*Glaucopsyche*	*melanops*	Serra, Serra	Valencia	Comunidad Valenciana	Spain	
GU676701	EZSPN338-09	RVcoll.08-H270	x	*Pieridae*	*Gonepteryx*	*cleopatra*	Vallforners, Canoves	Barcelona	Catalonia	Spain	610
GU675890	EZSPM348-09	RVcoll.08-R043	x	*Pieridae*	*Gonepteryx*	*cleopatra*	Espadilla	Castellon	Comunidad Valenciana	Spain	395
KP870288	EZSPM914-12	Rvcoll.12-M636	x	*Pieridae*	*Gonepteryx*	*cleopatra*	Lagunas de Ruidera, Ossa de Montiel	Ciudad Real	Castilla-La Mancha	Spain	795
JN265784	EZSPN1045-11	Rvcoll.08-L041	x	*Pieridae*	*Gonepteryx*	*cleopatra*	Sierra Elvira, Albolote	Granada	Andalusia	Spain	819
GU676630	EZSPN409-09	RVcoll.08-H510	x	*Pieridae*	*Gonepteryx*	*cleopatra*	Duron, La Alcarria	Guadalajara	Castilla-La Mancha	Spain	725
GU675950	EZSPM370-09	RVcoll.08-J841	x	*Pieridae*	*Gonepteryx*	*cleopatra*	Posada de Valdeon	Leon	Castilla y Leon	Spain	
KP871111	EZSPM628-12	RVcoll.11-D973	x	*Pieridae*	*Gonepteryx*	*cleopatra*	Istan	Malaga	Andalusia	Spain	283
GU675993	EZSPM034-09	RVcoll.08-P235	x	*Pieridae*	*Gonepteryx*	*cleopatra*	Puig d`en Galileu	Mallorca	Balearic Islands	Spain	1071
GU675994	EZSPM048-09	RVcoll.08-P266	x	*Pieridae*	*Gonepteryx*	*cleopatra*	S`Albufera	Mallorca	Balearic Islands	Spain	4
GU676060	EZSPM434-09	Rvcoll.09-X101	x	*Pieridae*	*Gonepteryx*	*cleopatra*	Parque Albufera des Grau, Menorca	Menorca	Balearic Islands	Spain	
GU676730	EZSPN310-09	RVcoll.08-H117	x	*Pieridae*	*Gonepteryx*	*cleopatra*	Na Vermella, Mao	Menorca	Balearic Islands	Spain	
GU676725	EZSPN313-09	RVcoll.08-H171	x	*Pieridae*	*Gonepteryx*	*cleopatra*	Llimpa, Mao	Menorca	Balearic Islands	Spain	
GU676156	EZSPN1004-09	RVcoll.08-M680	x	*Pieridae*	*Gonepteryx*	*cleopatra*	Serra Major, Morera de Montsant, Priorat	Tarragona	Catalonia	Spain	1090
HM901348	EZSPC1148-10	RVcoll.08-P900	x	*Pieridae*	*Gonepteryx*	*cleopatra*	Jarafuel, Canada de Abajo	Valencia	Comunidad Valenciana	Spain	650
GU676702	EZSPN331-09	RVcoll.08-H250	x	*Pieridae*	*Gonepteryx*	*cleopatra*	Mequinenza	Zaragoza	Aragon	Spain	129
KP870253	FBLMX237-11	BC ZSM Lep 50448		*Pieridae*	*Gonepteryx*	*rhamni*	Eching, Obere Isarau	Niederbayern	Bavaria	Germany	400
HQ957209	GWORA2451-09	BC ZSM Lep 30663		*Pieridae*	*Gonepteryx*	*rhamni*	Ruderting	Niederbayern	Bavaria	Germany	430
HM393184	GWORA2452-09	BC ZSM Lep 30664		*Pieridae*	*Gonepteryx*	*rhamni*	Laberveinting, Hart	Niederbayern	Bavaria	Germany	360
GU655016	FBLMT874-09	BC ZSM Lep 25434		*Pieridae*	*Gonepteryx*	*rhamni*	Inning am Ammersee, Arzla (Auwald bei)	Oberbayern	Bavaria	Germany	550
KP871004	EZSPM695-12	RVcoll.12-L598	x	*Pieridae*	*Gonepteryx*	*rhamni*	Caldas de Monchique, Monchique		Algarve	Portugal	
GU676559	EZSPN580-09	RVcoll.08-J135	x	*Pieridae*	*Gonepteryx*	*rhamni*	Curalha (Rio Tamega), Chaves		Tras-os-Montes	Portugal	350
HQ004506	EZROM583-08	RV-07-C368		*Pieridae*	*Gonepteryx*	*rhamni*	Schit-Oraseni	Botosani	Moldavia	Romania	200
HQ004507	EZROM1008-08	RVcoll.07-D304		*Pieridae*	*Gonepteryx*	*rhamni*	Dumbrava Vadului, Vad	Brasov	Transylvania	Romania	495
HQ004502	EZROM204-08	RV-07-D300		*Pieridae*	*Gonepteryx*	*rhamni*	Dumbrava Vadului, Vad	Brasov	Transylvania	Romania	495
HQ004505	EZROM290-08	RV-07-E652		*Pieridae*	*Gonepteryx*	*rhamni*	Dealul Istrita, Breaza	Buzau	Muntenia	Romania	525
KP870591	EULEP091-14	08-P978		*Pieridae*	*Gonepteryx*	*rhamni*	Gheorgheni, Dealul Feleacului, Cluj-Napoca	Cluj	Transylvania	Romania	
KP870690	EULEP188-14	11-J886		*Pieridae*	*Gonepteryx*	*rhamni*	Gheorgheni, Dealul Feleacului, Cluj-Napoca	Cluj	Transylvania	Romania	
HQ004504	EZROM202-08	RV-06-K609		*Pieridae*	*Gonepteryx*	*rhamni*	Badeni	Cluj	Transylvania	Romania	440
HQ004503	EZROM203-08	RV-06-K634		*Pieridae*	*Gonepteryx*	*rhamni*	Baciu forest (Cluj-Napoca)	Cluj	Transylvania	Romania	530
FJ663595	LOWA165-06	2005-LOWA-165		*Pieridae*	*Gonepteryx*	*rhamni*	Osyno vill.	Sebezh distr	Pskov	Russia	150
FJ663594	LOWA166-06	2005-LOWA-166		*Pieridae*	*Gonepteryx*	*rhamni*	Osyno vill.	Sebezh distr	Pskov	Russia	150
KP871063	EZSPM813-12	RVcoll.130711PX13	x	*Pieridae*	*Gonepteryx*	*rhamni*	Fondos de Vega, Degana		Asturias	Spain	1079
GU676305	EZSPN852-09	RVcoll.08-L700	x	*Pieridae*	*Gonepteryx*	*rhamni*	Soto del Real		Comunidad de Madrid	Spain	1230
GU675801	EZSPM239-09	RVcoll.08-R442	x	*Pieridae*	*Gonepteryx*	*rhamni*	Ames, Novais	A Coruna	Galicia	Spain	160
HM901384	EZSPC1184-10	RVcoll.09-T163	x	*Pieridae*	*Gonepteryx*	*rhamni*	La Serrota, Valdetoro	Avila	Castilla y Leon	Spain	1800
HM901392	EZSPC1192-10	RVcoll.09-V340	x	*Pieridae*	*Gonepteryx*	*rhamni*	Viladrau, Montseny, Osona	Barcelona	Catalonia	Spain	715
GU669816	EZSPC421-09	RVcoll.08-H221	x	*Pieridae*	*Gonepteryx*	*rhamni*	Vallforners, Canoves	Barcelona	Catalonia	Spain	610

GenBank accession number	BOLD Process ID	Sample ID	Used in the Iberian dataset	Family	Genus	Species	Exact Site	Region	State/Province	Country	Alt. (m)
GU669817	EZSPC422-09	RVcoll.08-H231	x	*Pieridae*	*Gonepteryx*	*rhamni*	Vallforners, Canoves	Barcelona	Catalonia	Spain	610
GU669810	EZSPC423-09	RVcoll.08-M706	x	*Pieridae*	*Gonepteryx*	*rhamni*	Riu Ges, Vidra, La Garrotxa	Barcelona	Catalonia	Spain	930
GU676870	EZSPN464-09	RVcoll.08-H678	x	*Pieridae*	*Gonepteryx*	*rhamni*	Tragacete, Herreria de los Chorros	Cuenca	Castilla-La Mancha	Spain	1270
HM901426	EZSPC1230-10	RVcoll.09-V694	x	*Pieridae*	*Gonepteryx*	*rhamni*	Meranges, La Cerdanya	Girona	Catalonia	Spain	1744
GU669811	EZSPC425-09	RVcoll.08-P070	x	*Pieridae*	*Gonepteryx*	*rhamni*	Torrent de Ridolaina, Cerdanya	Girona	Catalonia	Spain	1060
GU676627	EZSPN418-09	RVcoll.08-H552	x	*Pieridae*	*Gonepteryx*	*rhamni*	Duron, La Alcarria	Guadalajara	Castilla-La Mancha	Spain	726
HM901367	EZSPC1167-10	RVcoll.09-T071	x	*Pieridae*	*Gonepteryx*	*rhamni*	Sant Joan de Toran, Vall d`Aran	Lleida	Catalonia	Spain	980
HQ957502	GWORO794-09	BC ZSM Lep 30486		*Riodinidae*	*Hamearis*	*lucina*	Machtlfing STA		Bavaria	Germany	
HQ004515	EZROM1011-08	RVcoll.08-M209		*Riodinidae*	*Hamearis*	*lucina*	3 Km SW of Lunca Corbului	Arges	Muntenia	Romania	235
HQ004508	EZROM205-08	RV-07-D106		*Riodinidae*	*Hamearis*	*lucina*	Ciolanu	Buzau	Muntenia	Romania	255
HQ004509	EZROM206-08	RV-07-D205		*Riodinidae*	*Hamearis*	*lucina*	Dealul Istrita, Breaza	Buzau	Muntenia	Romania	730
HQ004512	EZROM315-08	RV-07-E677		*Riodinidae*	*Hamearis*	*lucina*	Cluj-Napoca, dealul Bogomaia, Fanatele Clujului	Cluj	Transylvania	Romania	480
HQ004511	EZROM316-08	RV-07-E678		*Riodinidae*	*Hamearis*	*lucina*	Cluj-Napoca, dealul Bogomaia, Fanatele Clujului	Cluj	Transylvania	Romania	480
HQ004510	EZROM318-08	RV-07-E680		*Riodinidae*	*Hamearis*	*lucina*	Cluj-Napoca, dealul Sfantul Gheorghe	Cluj	Transylvania	Romania	450
HQ004513	EZROM1009-08	RVcoll.08-M225		*Riodinidae*	*Hamearis*	*lucina*	2 Km E of Moreni	Dambovita	Muntenia	Romania	290
HQ004514	EZROM1010-08	RVcoll.08-M356		*Riodinidae*	*Hamearis*	*lucina*	Valea Belchia (Gheorgheni)	Harghita	Transylvania	Romania	1010
KP870444	EULEP141-14	10-C415		*Riodinidae*	*Hamearis*	*lucina*	Lăpuș-Prislop	Maramureș	Maramureş	Romania	
HM901376	EZSPC1176-10	RVcoll.09-T136	x	*Riodinidae*	*Hamearis*	*lucina*	San Andres de Parana, Lena		Asturias	Spain	755
KP870213	EZROM722-08	RV-06-G509	x	*Riodinidae*	*Hamearis*	*lucina*	El Boix, El Brull	Barcelona	Catalonia	Spain	805
KP870505	EZROM365-08	07-E616	x	*Riodinidae*	*Hamearis*	*lucina*	Sansor, Cerdanya	Girona	Catalonia	Spain	1125
HM901364	EZSPC1164-10	RVcoll.09-T048	x	*Riodinidae*	*Hamearis*	*lucina*	Saltant d`AigueesToretes, Salardu, Vall d`Aran	Lleida	Catalonia	Spain	1800
GU669812	EZSPC426-09	RVcoll.07-W105	x	*Riodinidae*	*Hamearis*	*lucina*	Arriu dera pincela, Vall d`Aran	Lleida	Catalonia	Spain	2167
GU669813	EZSPC427-09	RVcoll.07-W137	x	*Riodinidae*	*Hamearis*	*lucina*	Bassa d`Arres, Vall d`Aran	Lleida	Catalonia	Spain	1568
JF847986	EZSPN037-09	RVcoll.09-T083	x	*Riodinidae*	*Hamearis*	*lucina*	Val d`Hurno, Vall d`Aran	Lleida	Catalonia	Spain	1800
GU676286	EZSPN873-09	RVcoll.08-L763	x	*Riodinidae*	*Hamearis*	*lucina*	Valsain	Segovia	Castilla y Leon	Spain	1400
GU676521	EZSPN627-09	RVcoll.08-J719	x	*Riodinidae*	*Hamearis*	*lucina*	Rincon del Sardo, Castielfabib	Valencia	Comunidad Valenciana	Spain	1381
HM393208	GWORA2488-09	BC ZSM Lep 30700		*Hesperiidae*	*Hesperia*	*comma*	Sudelfeld, Arzmoos	Oberbayern	Bavaria	Germany	1200
JF415708	GWORR701-10	BC ZSM Lep 32197		*Hesperiidae*	*Hesperia*	*comma*	Garching-Hochbrueck	Oberbayern	Bavaria	Germany	487
JF415707	GWORR702-10	BC ZSM Lep 32198		*Hesperiidae*	*Hesperia*	*comma*	Samerberg, Frasdorf	Oberbayern	Bavaria	Germany	1300
GU707172	FBLMU513-09	BC ZSM Lep 27163		*Hesperiidae*	*Hesperia*	*comma*	Sinzing, Alpiner Steig	Oberpfalz	Bavaria	Germany	405
HQ004517	EZROM296-08	RV-07-E658		*Hesperiidae*	*Hesperia*	*comma*	Dealul Istrita, Breaza	Buzau	Muntenia	Romania	700
HQ004516	EZROM207-08	RV-06-V656		*Hesperiidae*	*Hesperia*	*comma*	Faget forest, Ratu Penii	Cluj	Transylvania	Romania	680
KP870499	EULEP136-14	10-C382		*Hesperiidae*	*Hesperia*	*comma*	Filpea-Gălăuţaș	Harghita	Transylvania	Romania	
KP870383	EULEP016-14	07-C396		*Hesperiidae*	*Hesperia*	*comma*	Cloașterf	Mures	Transylvania	Romania	
KP870323	EULEP190-14	11-J949		*Hesperiidae*	*Hesperia*	*comma*	M-ții. Rarău, pajiste	Suceava	Moldavia	Romania	1200
KP871064	EULEP191-14	11-J950		*Hesperiidae*	*Hesperia*	*comma*	M-ții. Rarău, pajiste	Suceava	Moldavia	Romania	1200
KP870302	EULEP192-14	11-J951		*Hesperiidae*	*Hesperia*	*comma*	M-ții. Rarău, pajiste	Suceava	Moldavia	Romania	1200
KP870653	EZROM723-08	RV-06-G555	x	*Hesperiidae*	*Hesperia*	*comma*	Can Rovira, El Brull	Barcelona	Catalonia	Spain	830
GU675657	EZSPC428-09	RVcoll.08-R153	x	*Hesperiidae*	*Hesperia*	*comma*	Montvi de Baix, Moianes	Barcelona	Catalonia	Spain	800
GU676193	EZSPN969-09	RVcoll.08-L989	x	*Hesperiidae*	*Hesperia*	*comma*	Una	Cuenca	Castilla-La Mancha	Spain	1201
GU675658	EZSPC429-09	RVcoll.08-R170	x	*Hesperiidae*	*Hesperia*	*comma*	Vallter 2000, Setcases, Ripolles	Girona	Catalonia	Spain	2537
GU676099	EZSPM461-09	Rvcoll.09-V932	x	*Hesperiidae*	*Hesperia*	*comma*	Sierra de la Sagra	Granada	Andalusia	Spain	1745
GU676133	EZSPM462-09	Rvcoll.09-V541	x	*Hesperiidae*	*Hesperia*	*comma*	Loma de San Francisco (Sierra Nevada)	Granada	Andalusia	Spain	2315
GU675739	EZSPM107-09	RVcoll.08-P446	x	*Hesperiidae*	*Hesperia*	*comma*	Penalen, Alto Tajo	Guadalajara	Castilla-La Mancha	Spain	1371
GU676823	EZSPN112-09	RVcoll.06-A269	x	*Hesperiidae*	*Hesperia*	*comma*	Laguarta, Rio Alcanadre	Huesca	Aragon	Spain	
GU676811	EZSPN119-09	RVcoll.06-A292	x	*Hesperiidae*	*Hesperia*	*comma*	Laguarta, Rio Alcanadre	Huesca	Aragon	Spain	1216
GU675659	EZSPC430-09	RVcoll.08-P366	x	*Hesperiidae*	*Hesperia*	*comma*	Pas de Perves	Lleida	Catalonia	Spain	1246
GU675660	EZSPC431-09	RVcoll.08-R100	x	*Hesperiidae*	*Hesperia*	*comma*	Salt de Pitx, Vall d`Aran	Lleida	Catalonia	Spain	1570
HQ004520	EZROM1012-08	RVcoll.08-M520		*Hesperiidae*	*Heteropterus*	*morpheus*	Apuseni Mts., Rimetea (Piatra Secuiului)	Alba	Transylvania	Romania	850
HQ004518	EZROM208-08	RV-07-C139		*Hesperiidae*	*Heteropterus*	*morpheus*	Stana	Salaj	Transylvania	Romania	500
HQ004519	EZROM209-08	RV-07-C140		*Hesperiidae*	*Heteropterus*	*morpheus*	Stana	Salaj	Transylvania	Romania	500
GU676963	EZSPN263-09	RVcoll.07-W175	x	*Hesperiidae*	*Heteropterus*	*morpheus*	Las Xanas, Santo Adriano		Asturias	Spain	300
GU676960	EZSPN264-09	RVcoll.07-W176	x	*Hesperiidae*	*Heteropterus*	*morpheus*	La Mortera, Ribera de Arriba		Asturias	Spain	416

GenBank accession number	BOLD Process ID	Sample ID	Used in the Iberian dataset	Family	Genus	Species	Exact Site	Region	State/Province	Country	Alt. (m)
GU676763	EZSPN271-09	RVcoll.07-W193	x	*Hesperiidae*	*Heteropterus*	*morpheus*	Oyambre		Cantabria	Spain	
KP870839	EULEP219-14	12-L044	x	*Hesperiidae*	*Heteropterus*	*morpheus*	Delika	Álava	Basque Country	Spain	
KP871049	EULEP218-14	12-L043	x	*Hesperiidae*	*Heteropterus*	*morpheus*	Orduña	Vizcaya	Basque Country	Spain	836
KP870571	EULEP204-14	12-L017	x	*Hesperiidae*	*Heteropterus*	*morpheus*	Cercanías El Mazuco, Llanes		Asturias	Spain	
KP870611	EULEP205-14	12-L018	x	*Hesperiidae*	*Heteropterus*	*morpheus*	Cercanías El Mazuco, Llanes		Asturias	Spain	
HQ004523	EZROM310-08	RV-07-E672		*Nymphalidae*	*Hipparchia*	*fagi*	Cheile Runcului	Alba	Transylvania	Romania	545
HQ004525	EZROM210-08	RV-06-V696		*Nymphalidae*	*Hipparchia*	*fagi*	Padurea Craiului Mts., Suncuius	Bihor	Transylvania	Romania	370
HQ004526	EZROM211-08	RV-06-V700		*Nymphalidae*	*Hipparchia*	*fagi*	Padurea Craiului Mts., Suncuius	Bihor	Transylvania	Romania	370
HQ004524	EZROM1017-08	RVcoll.08-M570		*Nymphalidae*	*Hipparchia*	*fagi*	Breaza, dealul Istrita	Buzau	Muntenia	Romania	600
HQ004522	EZROM319-08	RV-07-E681		*Nymphalidae*	*Hipparchia*	*fagi*	Dealul Istrita, Breaza	Buzau	Muntenia	Romania	525
HQ004521	EZROM629-08	RV-07-E383		*Nymphalidae*	*Hipparchia*	*fagi*	Dobraia (surroundings)	Caras-Severin	Banat	Romania	640
HQ004527	EZRMN421-09	RVcoll.09-V622		*Nymphalidae*	*Hipparchia*	*fagi*	4 Km W of Drobeta Turnu-Severin	Mehedinti	Oltenia	Romania	75
KP870750	EZROM724-08	RV-06-G561	x	*Nymphalidae*	*Hipparchia*	*fagi*	El Sola, El Brull	Barcelona	Catalonia	Spain	800
GU669806	EZSPC432-09	RVcoll.08-R152	x	*Nymphalidae*	*Hipparchia*	*fagi*	Montvi de Baix, Moianes	Barcelona	Catalonia	Spain	800
GU675946	EZSPM288-09	SMcoll.220708XF16	x	*Nymphalidae*	*Hipparchia*	*fagi*	Mas de Cap del Pla a Mas del Pont, Vistabella del Maestrazgo	Castellon	Comunidad Valenciana	Spain	1145
KP870429	EZSPM824-12	RVcoll.140611ZG25	x	*Nymphalidae*	*Hipparchia*	*fagi*	Moli de Joaquim, La Pobla de Benifassa	Castellon	Comunidad Valenciana	Spain	1025
GU669807	EZSPC433-09	RVcoll.08-R297	x	*Nymphalidae*	*Hipparchia*	*fagi*	Masia del Plans, Vidra, La Garrotxa	Girona	Catalonia	Spain	1200
KP870258	EULEP258-14	12-Q529	x	*Nymphalidae*	*Hipparchia*	*fagi*	Cellers, Pantà Terradets, Pallars Jussà	Lleida	Catalonia	Spain	391
GU669808	EZSPC434-09	RVcoll.06-V772	x	*Nymphalidae*	*Hipparchia*	*fagi*	Coll de Boixols, Abella de la Conca, Pallars Jussa	Lleida	Catalonia	Spain	1365
GU669809	EZSPC435-09	RVcoll.08-P317	x	*Nymphalidae*	*Hipparchia*	*fagi*	Farena, Alt Camp	Tarragona	Catalonia	Spain	580
JN278874	EZSPN1044-11	Rvcoll.08-J677	x	*Nymphalidae*	*Hipparchia*	*fagi*	Monte Caro	Tarragona	Catalonia	Spain	
GU676375	EZSPN775-09	RVcoll.08-L285	x	*Nymphalidae*	*Hipparchia*	*fidia*	Los Collados - Benimaurell, Vall de Laguart	Alicante	Comunidad Valenciana	Spain	675
HM901271	EZSPC1068-10	RVcoll.08-L206	x	*Nymphalidae*	*Hipparchia*	*fidia*	La Vega, Vilafranca	Castellon	Comunidad Valenciana	Spain	1124
GU676108	EZSPM422-09	Rvcoll.09-V846	x	*Nymphalidae*	*Hipparchia*	*fidia*	Coll de Banyuls, Alt Emporda	Girona	Catalonia	Spain	
HM901327	EZSPC1127-10	RVcoll.08-P637	x	*Nymphalidae*	*Hipparchia*	*fidia*	Hoz Angosta, Duron	Guadalajara	Castilla-La Mancha	Spain	789
GU675725	EZSPM129-09	RVcoll.08-P633	x	*Nymphalidae*	*Hipparchia*	*fidia*	Hoz Angosta, Duron	Guadalajara	Castilla-La Mancha	Spain	789
GU675727	EZSPM182-09	RVcoll.08-J816	x	*Nymphalidae*	*Hipparchia*	*fidia*	Compludo	Leon	Castilla y Leon	Spain	
GU676035	EZSPM032-09	RVcoll.08-P232	x	*Nymphalidae*	*Hipparchia*	*fidia*	Puig d`en Galileu	Mallorca	Balearic Islands	Spain	1071
GU676038	EZSPM042-09	RVcoll.08-P250	x	*Nymphalidae*	*Hipparchia*	*fidia*	Santa Maria	Mallorca	Balearic Islands	Spain	181
GU676036	EZSPM062-09	RVcoll.08-P289	x	*Nymphalidae*	*Hipparchia*	*fidia*	Genova	Mallorca	Balearic Islands	Spain	123
GU676157	EZSPN1005-09	RVcoll.08-M685	x	*Nymphalidae*	*Hipparchia*	*fidia*	Farena, Alt Camp	Tarragona	Catalonia	Spain	580
HM901324	EZSPC1124-10	RVcoll.08-P616	x	*Nymphalidae*	*Hipparchia*	*fidia*	Albarracin	Teruel	Aragon	Spain	1250
GU676370	EZSPN782-09	RVcoll.08-L299	x	*Nymphalidae*	*Hipparchia*	*fidia*	Paraisos Altos, Manzanera	Teruel	Aragon	Spain	1200
HM901233	EZSPC1029-10	RVcoll.08-J084	x	*Nymphalidae*	*Hipparchia*	*fidia*	Requena, Requena	Valencia	Comunidad Valenciana	Spain	700
KP870917	EULEP167-14	11-I657		*Nymphalidae*	*Hipparchia*	*hermione*	Mont Ventoux		Vaucluse	France	1320
KP870772	EULEP168-14	11-I780		*Nymphalidae*	*Hipparchia*	*hermione*	Montauban-sur-l'Ouvèze		Drôme	France	761
KP870817	EULEP169-14	11-I885		*Nymphalidae*	*Hipparchia*	*hermione*	Rossas		Hautes Alpes	France	929
KP870371	EULEP248-14	12-P047		*Nymphalidae*	*Hipparchia*	*hermione*	Châtillon-en-Diois		Drôme	France	600-750
GU676548	EZSPN593-09	RVcoll.08-J178	x	*Nymphalidae*	*Hipparchia*	*hermione*	Serra do Larouco (topo), Padornelos, Montalegre		Tras-os-Montes	Portugal	1520
HM901224	EZSPC1020-10	RVcoll.08-P678	x	*Nymphalidae*	*Hipparchia*	*hermione*	Mina carbon, Puerto de Ventana		Asturias	Spain	1660
KP870942	EZSPM775-12	RVcoll.050111LP62	x	*Nymphalidae*	*Hipparchia*	*hermione*	Fuente De, Camaleno		Asturias	Spain	1093
JN278879	EZSPN1061-11	Rvcoll.08-P677.1	x	*Nymphalidae*	*Hipparchia*	*hermione*	Mina carbon, Puerto de Ventana		Asturias	Spain	1660
GU676259	EZSPN902-09	RVcoll.08-L819	x	*Nymphalidae*	*Hipparchia*	*hermione*	Miraflores de la Sierra		Comunidad de Madrid	Spain	1588
KP870520	EZSPC1316-10	RVcoll.130209KL75	x	*Nymphalidae*	*Hipparchia*	*hermione*	Sierra de Salinas,Salinas	Alicante	Comunidad Valenciana	Spain	
KP871130	EZSPN151-09	RVcoll.06-G649	x	*Nymphalidae*	*Hipparchia*	*hermione*	Font de l`Aulet, El Brull, Osona	Barcelona	Catalonia	Spain	1070
KP870941	EZSPM1015-12	Rvcoll.12-M764	x	*Nymphalidae*	*Hipparchia*	*hermione*	7 km SE Navalvillar de Ibor	Caceres	Extremadura	Spain	729
KP870277	EZSPM1016-12	Rvcoll.12-M765	x	*Nymphalidae*	*Hipparchia*	*hermione*	7 km SE Navalvillar de Ibor	Caceres	Extremadura	Spain	729
KP870999	EZSPM1027-12	Rvcoll.12-M777	x	*Nymphalidae*	*Hipparchia*	*hermione*	Hospital del Obispo, Navalvillar de Ibor	Caceres	Extremadura	Spain	1010

GenBank accession number	BOLD Process ID	Sample ID	Used in the Iberian dataset	Family	Genus	Species	Exact Site	Region	State/Province	Country	Alt. (m)
HM901529	EZSPC1395-10	SMcoll.190608FT66	x	*Nymphalidae*	*Hipparchia*	*hermione*	Font del Regatxal, Vilafranca	Castellon	Comunidad Valenciana	Spain	1361
HM901613	EZSPC758-10	RVcoll.08-L267	x	*Nymphalidae*	*Hipparchia*	*hermione*	Font d`Horta, Vilafranca	Castellon	Comunidad Valenciana	Spain	1049
KP870436	EZSPM845-12	RVcoll.150310ZX49	x	*Nymphalidae*	*Hipparchia*	*hermione*	Limite con Teruel, Vistavella	Castellon	Comunidad Valenciana	Spain	1156
HM901225	EZSPC1021-10	RVcoll.08-P044	x	*Nymphalidae*	*Hipparchia*	*hermione*	La Valira (near Urus), Cerdanya	Girona	Catalonia	Spain	1300
HM901677	EZSPC834-10	RVcoll.08-R165	x	*Nymphalidae*	*Hipparchia*	*hermione*	Llanars, Ripolles	Girona	Catalonia	Spain	1119.8
HM901734	EZSPC895-10	RVcoll.09-V431	x	*Nymphalidae*	*Hipparchia*	*hermione*	Loma de Lastonares (Sierra Nevada)	Granada	Andalusia	Spain	2050
HM901795	EZSPC960-10	RVcoll.100608DS72	x	*Nymphalidae*	*Hipparchia*	*hermione*	Casillas de Rojas, Gueejar Sierra	Granada	Andalusia	Spain	1733
GU675726	EZSPM164-09	RVcoll.08-P695	x	*Nymphalidae*	*Hipparchia*	*hermione*	Arbeteta	Guadalajara	Castilla-La Mancha	Spain	1136
GU676908	EZSPN505-09	RVcoll.08-H962	x	*Nymphalidae*	*Hipparchia*	*hermione*	Laguarta, Rio Alcanadre	Huesca	Aragon	Spain	1216
GU675929	EZSPM303-09	RVcoll.08-J850	x	*Nymphalidae*	*Hipparchia*	*hermione*	Posada de Valdeon	Leon	Castilla y Leon	Spain	
HM901560	EZSPC703-10	RVcoll.07-F087	x	*Nymphalidae*	*Hipparchia*	*hermione*	Coll de Boixols, Pallars Jussa	Lleida	Catalonia	Spain	1300
KP870649	EULEP246-14	12-N741	x	*Nymphalidae*	*Hipparchia*	*hermione*	El Valecillo	Teruel	Aragón	Spain	1526.7
KP870360	EZSPM796-12	RVcoll.080611XP73	x	*Nymphalidae*	*Hipparchia*	*hermione*	Fuente del Tajo, Nogueruelas	Teruel	Aragon	Spain	1727
GU676950	EZSPN551-09	RVcoll.08-J057	x	*Nymphalidae*	*Hipparchia*	*hermione*	Muela de Cortes, Cortes de Pallas	Valencia	Comunidad Valenciana	Spain	
KP870869	EULEP171-14	11-J138		*Nymphalidae*	*Hipparchia*	*hermione*	Birgisch		Valais	Switzerland	1480
KP870500	EULEP014-14	07-C295		*Nymphalidae*	*Hipparchia*	*semele*	Koksijde		West Vlaanderen	Belgium	0
KP870672	EULEP132-14	10-B727		*Nymphalidae*	*Hipparchia*	*semele*	Le Chaffaut-St-Jurson		Alpes-de-Haute-Provence	France	580
HQ004533	EZROM215-08	RV-06-V719		*Nymphalidae*	*Hipparchia*	*semele*	Dealul Istrita, Breaza	Buzau	Muntenia	Romania	600
HQ004535	EZROM196-08	RV-07-E379		*Nymphalidae*	*Hipparchia*	*semele*	Dobraia (surroundings)	Caras-Severin	Banat	Romania	640
HQ004532	EZROM214-08	RV-07-E377		*Nymphalidae*	*Hipparchia*	*semele*	Dobraia (surroundings)	Caras-Severin	Banat	Romania	640
HQ004530	EZROM630-08	RV-07-E378		*Nymphalidae*	*Hipparchia*	*semele*	Dobraia (surroundings)	Caras-Severin	Banat	Romania	640
HQ004528	EZRMN338-08	RVcoll.08-H018		*Nymphalidae*	*Hipparchia*	*semele*	Dealul Sf. Pavel (Cluj-Napoca)	Cluj	Transylvania	Romania	450
HQ004529	EZRMN389-09	RVcoll.08-H032		*Nymphalidae*	*Hipparchia*	*semele*	Hoia forest (Cluj-Napoca)	Cluj	Transylvania	Romania	450
GU669647	EZROM1065-09	RVcoll.08-H037		*Nymphalidae*	*Hipparchia*	*semele*	Dealul Sf. Pavel (Cluj-Napoca)	Cluj	Transylvania	Romania	450
HQ004534	EZROM213-08	RV-07-D409		*Nymphalidae*	*Hipparchia*	*semele*	Suatu	Cluj	Transylvania	Romania	380
HQ004536	EZRMN420-09	RVcoll.09-V621		*Nymphalidae*	*Hipparchia*	*semele*	4 Km W of Drobeta Turnu-Severin	Mehedinti	Oltenia	Romania	75
HQ004531	EZROM631-08	RV-07-C138		*Nymphalidae*	*Hipparchia*	*semele*	Stana	Salaj	Transylvania	Romania	500
GU675723	EZSPM151-09	RVcoll.08-P666	x	*Nymphalidae*	*Hipparchia*	*semele*	Gamoniteiru, Sierra de Aramo, Quiros		Asturias	Spain	1712
GU676204	EZSPN956-09	RVcoll.08-L953	x	*Nymphalidae*	*Hipparchia*	*semele*	Puerto de Pozazal		Cantabria	Spain	1110
KP871018	EZSPM906-12	Rvcoll.12-M628	x	*Nymphalidae*	*Hipparchia*	*semele*	Sotuelamos, El Bonillo	Albacete	Castilla-La Mancha	Spain	890
KP870485	EZROM367-08	07-E624	x	*Nymphalidae*	*Hipparchia*	*semele*	Vallforners, Canoves	Barcelona	Catalonia	Spain	650
GU676206	EZSPN958-09	RVcoll.08-L955	x	*Nymphalidae*	*Hipparchia*	*semele*	Corralejo	Burgos	Castilla y Leon	Spain	983
GU675887	EZSPM349-09	RVcoll.08-R058	x	*Nymphalidae*	*Hipparchia*	*semele*	La Cambreta	Castellon	Comunidad Valenciana	Spain	1520
GU676381	EZSPN769-09	RVcoll.08-L273	x	*Nymphalidae*	*Hipparchia*	*semele*	Mas de Cap del Pla a Mas del Pont, Vistabella del Maestrazgo	Castellon	Comunidad Valenciana	Spain	1145
KP870598	EZSPM917-12	Rvcoll.12-M639	x	*Nymphalidae*	*Hipparchia*	*semele*	Lagunas de Ruidera, Ossa de Montiel	Ciudad Real	Castilla-La Mancha	Spain	795
GU676871	EZSPN463-09	RVcoll.08-H677	x	*Nymphalidae*	*Hipparchia*	*semele*	Tragacete, Herreria de los Chorros	Cuenca	Castilla-La Mancha	Spain	1270
GU669802	EZSPC436-09	RVcoll.08-R274	x	*Nymphalidae*	*Hipparchia*	*semele*	Masia del Plans, Vidra, La Garrotxa	Girona	Catalonia	Spain	1200
GU669805	EZSPC439-09	RVcoll.08-P027	x	*Nymphalidae*	*Hipparchia*	*semele*	Talltendre, Cerdanya	Girona	Catalonia	Spain	1571
JN278880	EZSPN1062-11	Rvcoll.08-P717	x	*Nymphalidae*	*Hipparchia*	*semele*	Alquife	Granada	Andalusia	Spain	1282
JN278883	EZSPN1074-11	Rvcoll.09-V478	x	*Nymphalidae*	*Hipparchia*	*semele*	Sierra de la Sagra	Granada	Andalusia	Spain	1745
GU676407	EZSPN737-09	RVcoll.08-L147	x	*Nymphalidae*	*Hipparchia*	*semele*	Laguna Seca, Hueneja	Granada	Andalusia	Spain	2276
GU676155	EZSPN1003-09	RVcoll.08-M095	x	*Nymphalidae*	*Hipparchia*	*semele*	San Andres del Rey, La Alcarria	Guadalajara	Castilla-La Mancha	Spain	1018
GU676139	EZSPN1027-09	RVcoll.08-M961	x	*Nymphalidae*	*Hipparchia*	*semele*	Saganta	Huesca	Aragon	Spain	767
GU669803	EZSPC437-09	RVcoll.08-P359	x	*Nymphalidae*	*Hipparchia*	*semele*	Perves, Pallars Jussa	Lleida	Catalonia	Spain	1071
GU669804	EZSPC438-09	RVcoll.08-M673	x	*Nymphalidae*	*Hipparchia*	*semele*	Serra Major, Morera de Montsant, Priorat	Tarragona	Catalonia	Spain	1090
KP870535	EZSPM825-12	RVcoll.140611ZG43	x	*Nymphalidae*	*Hipparchia*	*semele*	Puerto de Sant Just, Escucha	Teruel	Aragon	Spain	1421
KP870629	EULEP049-14	08-H804		*Nymphalidae*	*Hipparchia*	*semele*	Öja		Gotland	Sweden	
KP870578	EULEP050-14	08-H815		*Nymphalidae*	*Hipparchia*	*semele*	Sandhammaren-		Skåne	Sweden	
GU676544	EZSPN601-09	RVcoll.08-J193	x	*Nymphalidae*	*Hipparchia*	*statilinus*	Seculca, Beca, Boticas		Tras-os-Montes	Portugal	790
HQ004538	EZROM1020-08	RVcoll.08-M582		*Nymphalidae*	*Hipparchia*	*statilinus*	Periprava, Danube Delta	Tulcea	Dobrogea	Romania	1

GenBank accession number	BOLD Process ID	Sample ID	Used in the Iberian dataset	Family	Genus	Species	Exact Site	Region	State/Province	Country	Alt. (m)
HQ004537	EZROM603-08	RV-07-F536		*Nymphalidae*	*Hipparchia*	*statilinus*	Delta Dunarii, Periprava	Tulcea	Dobrogea	Romania	3
GU676376	EZSPN776-09	RVcoll.08-L286	x	*Nymphalidae*	*Hipparchia*	*statilinus*	Los Collados - Benimaurell, Vall de Laguart	Alicante	Comunidad Valenciana	Spain	675
KP870934	EZSPM116-09	RVcoll.08-P475	x	*Nymphalidae*	*Hipparchia*	*statilinus*	Candeleda	Avila	Castilla y Leon	Spain	450
KP870265	EZROM740-08	RV-06-G531	x	*Nymphalidae*	*Hipparchia*	*statilinus*	Urb. El Muntanya	Barcelona	Catalonia	Spain	720
HM901500	EZSPC1366-10	RVcoll.09-X055	x	*Nymphalidae*	*Hipparchia*	*statilinus*	Barranc de Montmalus, La Cerdanya	Girona	Catalonia	Spain	1340
HM901501	EZSPC1367-10	RVcoll.09-X056	x	*Nymphalidae*	*Hipparchia*	*statilinus*	Barranc de Montmalus, La Cerdanya	Girona	Catalonia	Spain	1340
GU676862	EZSPC561-09	RVcoll.08-P052	x	*Nymphalidae*	*Hipparchia*	*statilinus*	Estoll, Fontanals de Cerdanya, Cerdanya	Girona	Catalonia	Spain	1085
GU676863	EZSPC562-09	RVcoll.08-P053	x	*Nymphalidae*	*Hipparchia*	*statilinus*	Estoll, Fontanals de Cerdanya, Cerdanya	Girona	Catalonia	Spain	1085
GU676109	EZSPM403-09	Rvcoll.09-V805	x	*Nymphalidae*	*Hipparchia*	*statilinus*	Estanys de la Jonquera, La Jonquera, Alt Emporda	Girona	Catalonia	Spain	
HM901412	EZSPC1213-10	RVcoll.09-V506	x	*Nymphalidae*	*Hipparchia*	*statilinus*	Sierra de Alfacar	Granada	Andalusia	Spain	1275
GU676106	EZSPM453-09	Rvcoll.09-V589	x	*Nymphalidae*	*Hipparchia*	*statilinus*	Seminario (Sierra Nevada)	Granada	Andalusia	Spain	1520
HM901328	EZSPC1128-10	RVcoll.08-P694	x	*Nymphalidae*	*Hipparchia*	*statilinus*	Arbeteta	Guadalajara	Castilla-La Mancha	Spain	1136
GU675729	EZSPM165-09	RVcoll.08-P696	x	*Nymphalidae*	*Hipparchia*	*statilinus*	Villanueva de Alcoron	Guadalajara	Castilla-La Mancha	Spain	1307
JN278890	EZSPN1112-11	Rvcoll.10-C309	x	*Nymphalidae*	*Hipparchia*	*statilinus*	Duron	Guadalajara	Castilla-La Mancha	Spain	730
JN278891	EZSPN1113-11	Rvcoll.10-C310	x	*Nymphalidae*	*Hipparchia*	*statilinus*	Duron	Guadalajara	Castilla-La Mancha	Spain	730
GU675941	EZSPM291-09	RVcoll.08-J822	x	*Nymphalidae*	*Hipparchia*	*statilinus*	Vega de Espinareda	Leon	Castilla y Leon	Spain	
KP870622	EZSPN767-09	RVcoll.08-L270	x	*Nymphalidae*	*Hipparchia*	*statilinus*	Paraisos Altos, Manzanera	Teruel	Aragon	Spain	1200
HM901231	EZSPC1027-10	RVcoll.08-J075	x	*Nymphalidae*	*Hipparchia*	*statilinus*	Jarafuel, Jarafuel	Valencia	Comunidad Valenciana	Spain	
HQ004542	EZRMN316-08	RVcoll.08-M465		*Nymphalidae*	*Hipparchia*	*syriaca*	Babadag forest (10 Km S of Babadag)	Tulcea	Dobrogea	Romania	115
HQ004541	EZRMN317-08	RVcoll.08-M484		*Nymphalidae*	*Hipparchia*	*syriaca*	Macin Mts., Greci (Esitul Sufletului)	Tulcea	Dobrogea	Romania	180
HQ004540	EZRMN318-08	RVcoll.08-M486		*Nymphalidae*	*Hipparchia*	*syriaca*	Macin Mts., Greci (Esitul Sufletului)	Tulcea	Dobrogea	Romania	180
HQ004539	EZRMN319-08	RVcoll.08-M487		*Nymphalidae*	*Hipparchia*	*syriaca*	Macin Mts., Greci (Esitul Sufletului)	Tulcea	Dobrogea	Romania	180
HQ004543	EZROM1013-08	RVcoll.08-M463		*Nymphalidae*	*Hipparchia*	*syriaca*	Babadag forest (10 Km S of Babadag)	Tulcea	Dobrogea	Romania	115
HQ004544	EZROM1014-08	RVcoll.08-M464		*Nymphalidae*	*Hipparchia*	*syriaca*	Babadag forest (10 Km S of Babadag)	Tulcea	Dobrogea	Romania	115
HQ004545	EZROM1015-08	RVcoll.08-M480		*Nymphalidae*	*Hipparchia*	*syriaca*	Macin Mts., Greci (Esitul Sufletului)	Tulcea	Dobrogea	Romania	200
HQ004546	EZROM1016-08	RVcoll.08-M481		*Nymphalidae*	*Hipparchia*	*syriaca*	Macin Mts., Greci (Esitul Sufletului)	Tulcea	Dobrogea	Romania	200
GU675620	EZROM1076-09	RVcoll.08-M472		*Nymphalidae*	*Hipparchia*	*syriaca*	Babadag forest (10 Km S of Babadag)	Tulcea	Dobrogea	Romania	115
GU675621	EZROM1077-09	RVcoll.08-M476		*Nymphalidae*	*Hipparchia*	*syriaca*	Babadag forest (3 Km E of Slava Rusa)	Tulcea	Dobrogea	Romania	130
GU675619	EZROM1078-09	RVcoll.08-M479		*Nymphalidae*	*Hipparchia*	*syriaca*	Macin Mts., Greci (Esitul Sufletului)	Tulcea	Dobrogea	Romania	250
HQ004547	EZROM216-08	RV-07-D072		*Nymphalidae*	*Hipparchia*	*volgensis*	Greci massif, izvorul Esitul Sufletului	Tulcea	Dobrogea	Romania	145
GU676180	EZSPN980-09	RVcoll.08-M019	x	*Nymphalidae*	*Hyponephele*	*lupinus*	San Martin de la Vega		Comunidad de Madrid	Spain	616
KP871143	EZSPM910-12	Rvcoll.12-M632	x	*Nymphalidae*	*Hyponephele*	*lupinus*	Sotuelamos, El Bonillo	Albacete	Castilla-La Mancha	Spain	890
KP870843	EZSPM911-12	Rvcoll.12-M633	x	*Nymphalidae*	*Hyponephele*	*lupinus*	Sotuelamos, El Bonillo	Albacete	Castilla-La Mancha	Spain	890
JN278862	EZSPC1279-10	RVcoll.290909HG13	x	*Nymphalidae*	*Hyponephele*	*lupinus*	Masia Gaspar,Olocau del Rey	Castellon	Comunidad Valenciana	Spain	1006
JN278875	EZSPN1048-11	Rvcoll.08-L268	x	*Nymphalidae*	*Hyponephele*	*lupinus*	Mas de Cap del Pla a Mas del Pont, Vistabella del Maestrazgo	Castellon	Comunidad Valenciana	Spain	1145
HM901411	EZSPC1212-10	RVcoll.09-V504	x	*Nymphalidae*	*Hyponephele*	*lupinus*	Sierra de Alfacar	Granada	Andalusia	Spain	1275
KP870656	EZSPM661-12	RVcoll.11-I530	x	*Nymphalidae*	*Hyponephele*	*lupinus*	SE of Cumbres Verdes (La Zubia)	Granada	Andalusia	Spain	1400
KP870552	EZSPM662-12	RVcoll.11-I531	x	*Nymphalidae*	*Hyponephele*	*lupinus*	SE of Cumbres Verdes (La Zubia)	Granada	Andalusia	Spain	1400
HM901294	EZSPC1093-10	RVcoll.08-L920.1	x	*Nymphalidae*	*Hyponephele*	*lupinus*	Escamilla	Guadalajara	Castilla-La Mancha	Spain	1040
HM901309	EZSPC1108-10	RVcoll.08-M046	x	*Nymphalidae*	*Hyponephele*	*lupinus*	Gualda, La Alcarria	Guadalajara	Castilla-La Mancha	Spain	730
HM901310	EZSPC1109-10	RVcoll.08-M064	x	*Nymphalidae*	*Hyponephele*	*lupinus*	Budia, La Alcarria	Guadalajara	Castilla-La Mancha	Spain	1006
JN278877	EZSPN1053-11	Rvcoll.08-L921	x	*Nymphalidae*	*Hyponephele*	*lupinus*	Escamilla	Guadalajara	Castilla-La Mancha	Spain	1040
GU676166	EZSPN990-09	RVcoll.08-M045	x	*Nymphalidae*	*Hyponephele*	*lupinus*	Gualda, La Alcarria	Guadalajara	Castilla-La Mancha	Spain	730
GU676948	EZSPN548-09	RVcoll.08-J052	x	*Nymphalidae*	*Hyponephele*	*lupinus*	Muela de Cortes, Cortes de Pallas	Valencia	Comunidad Valenciana	Spain	
KP870594	EULEP189-14	11-J887		*Nymphalidae*	*Hyponephele*	*lycaon*	M-ții Trascăului, Rimetea, Piatra Secuiului	Alba	Transylvania	Romania	
HQ004548	EZROM341-08	RV-08-A019		*Nymphalidae*	*Hyponephele*	*lycaon*	Apuseni Mts., Rimetea, medieval mines	Alba	Transylvania	Romania	650
HQ004549	EZROM1023-08	RVcoll.08-M564		*Nymphalidae*	*Hyponephele*	*lycaon*	Breaza, dealul Istrita	Buzau	Muntenia	Romania	600
HQ004550	EZROM1024-08	RVcoll.08-M573		*Nymphalidae*	*Hyponephele*	*lycaon*	Breaza, dealul Istrita	Buzau	Muntenia	Romania	600
GU676196	EZSPN960-09	RVcoll.08-L963	x	*Nymphalidae*	*Hyponephele*	*lycaon*	La Morcuera, Rascafria		Comunidad de Madrid	Spain	1766

GenBank accession number	BOLD Process ID	Sample ID	Used in the Iberian dataset	Family	Genus	Species	Exact Site	Region	State/Province	Country	Alt. (m)
GU669801	EZSPC443-09	RVcoll.07-E640	x	*Nymphalidae*	*Hyponephele*	*lycaon*	Mont Caro, Ports Tortosa-Besseit	Barcelona	Catalonia	Spain	1350
GU676397	EZSPN745-09	RVcoll.08-L173	x	*Nymphalidae*	*Hyponephele*	*lycaon*	Font del Regatxal, Vilafranca	Castellon	Comunidad Valenciana	Spain	1361
GU676390	EZSPN751-09	RVcoll.08-L215	x	*Nymphalidae*	*Hyponephele*	*lycaon*	Font del Regatxal, Vilafranca	Castellon	Comunidad Valenciana	Spain	1361
HM901223	EZSPC1019-10	RVcoll.08-H695	x	*Nymphalidae*	*Hyponephele*	*lycaon*	Huelamo, Rio Jucar	Cuenca	Castilla-La Mancha	Spain	1237
GU676877	EZSPN469-09	RVcoll.08-H694	x	*Nymphalidae*	*Hyponephele*	*lycaon*	Huelamo, Rio Jucar	Cuenca	Castilla-La Mancha	Spain	1237
GU669798	EZSPC440-09	RVcoll.08-P023	x	*Nymphalidae*	*Hyponephele*	*lycaon*	Talltendre, Cerdanya	Girona	Catalonia	Spain	1571
GU669799	EZSPC441-09	RVcoll.08-P024	x	*Nymphalidae*	*Hyponephele*	*lycaon*	Talltendre, Cerdanya	Girona	Catalonia	Spain	1571
GU669800	EZSPC442-09	RVcoll.08-P025	x	*Nymphalidae*	*Hyponephele*	*lycaon*	Talltendre, Cerdanya	Girona	Catalonia	Spain	1571
HM901415	EZSPC1216-10	RVcoll.09-V524	x	*Nymphalidae*	*Hyponephele*	*lycaon*	Loma de San Francisco (Sierra Nevada)	Granada	Andalusia	Spain	2315
HM901416	EZSPC1217-10	RVcoll.09-V525	x	*Nymphalidae*	*Hyponephele*	*lycaon*	Loma de San Francisco (Sierra Nevada)	Granada	Andalusia	Spain	2315
HM901418	EZSPC1219-10	RVcoll.09-V539	x	*Nymphalidae*	*Hyponephele*	*lycaon*	Loma de San Francisco (Sierra Nevada)	Granada	Andalusia	Spain	2315
HM901422	EZSPC1223-10	RVcoll.09-V557	x	*Nymphalidae*	*Hyponephele*	*lycaon*	Collado de las Sabinas (Sierra Nevada)	Granada	Andalusia	Spain	2175
GU676113	EZSPM463-09	Rvcoll.09-V538	x	*Nymphalidae*	*Hyponephele*	*lycaon*	Loma de San Francisco (Sierra Nevada)	Granada	Andalusia	Spain	2315
KP870978	EZSPM464-09	Rvcoll.09-V437	x	*Nymphalidae*	*Hyponephele*	*lycaon*	Loma de Lastonares (Sierra Nevada)	Granada	Andalusia	Spain	2050
GU676218	EZSPN942-09	RVcoll.08-L918	x	*Nymphalidae*	*Hyponephele*	*lycaon*	Escamilla	Guadalajara	Castilla-La Mancha	Spain	1040
GU676912	EZSPN509-09	RVcoll.08-H967	x	*Nymphalidae*	*Hyponephele*	*lycaon*	Laguarta, Rio Alcanadre	Huesca	Aragon	Spain	1216
GU675939	EZSPM293-09	RVcoll.08-J831	x	*Nymphalidae*	*Hyponephele*	*lycaon*	Puerto de Monteviejo	Leon	Castilla y Leon	Spain	
HM901515	EZSPC1381-10	RVcoll.09-X517	x	*Nymphalidae*	*Hyponephele*	*lycaon*	2 km NW El Vallecillo	Teruel	Aragon	Spain	1450
GU676391	EZSPN752-09	RVcoll.08-L219	x	*Nymphalidae*	*Hyponephele*	*lycaon*	Ruta de la Sima, El Vallecillo	Teruel	Aragon	Spain	1531
KP870858	EULEP007-14	06-G517	x	*Lycaenidae*	*Iolana*	*debilitata*	Santuari de Puiglagulla, Vilalleons, Osona	Barcelona	Catalonia	Spain	720
KP870314	EULEP008-14	06-G518	x	*Lycaenidae*	*Iolana*	*debilitata*	Santuari de Puiglagulla, Vilalleons, Osona	Barcelona	Catalonia	Spain	720
KP870539	EULEP009-14	06-G519	x	*Lycaenidae*	*Iolana*	*debilitata*	Santuari de Puiglagulla, Vilalleons, Osona	Barcelona	Catalonia	Spain	720
KP871116	EULEP010-14	06-G520	x	*Lycaenidae*	*Iolana*	*debilitata*	Santuari de Puiglagulla, Vilalleons, Osona	Barcelona	Catalonia	Spain	720
KP871085	EULEP038-14	07-Z062	x	*Lycaenidae*	*Iolana*	*debilitata*	Oristrell, Pont de Vilomara, Bages	Barcelona	Catalonia	Spain	283
KP870974	EULEP039-14	07-Z063	x	*Lycaenidae*	*Iolana*	*debilitata*	Oristrell, Pont de Vilomara, Bages	Barcelona	Catalonia	Spain	283
KP870925	EULEP064-14	08-J700	x	*Lycaenidae*	*Iolana*	*debilitata*	Alrededores de Almedíjar, Almedíjar	Castellón	Comunidad Valenciana	Spain	
KP870863	EULEP065-14	08-J701	x	*Lycaenidae*	*Iolana*	*debilitata*	Alrededores de Almedíjar, Almedíjar	Castellón	Comunidad Valenciana	Spain	
KP870322	EULEP066-14	08-J704	x	*Lycaenidae*	*Iolana*	*debilitata*	Alrededores de Almedíjar, Almedíjar	Castellón	Comunidad Valenciana	Spain	
KP870905	EULEP067-14	08-J706	x	*Lycaenidae*	*Iolana*	*debilitata*	Alrededores de Almedíjar, Almedíjar	Castellón	Comunidad Valenciana	Spain	
KP871120	EULEP073-14	08-L348	x	*Lycaenidae*	*Iolana*	*debilitata*	Sierra de Huétor	Granada	Andalusia	Spain	1000
KP871104	EULEP074-14	08-L349	x	*Lycaenidae*	*Iolana*	*debilitata*	Sierra de Huétor	Granada	Andalusia	Spain	1000
KP870508	EULEP037-14	07-W202	x	*Lycaenidae*	*Iolana*	*debilitata*	Santibañez de Ayllón	Segovia	Castilla y León	Spain	1200
KP870756	EULEP068-14	08-J783	x	*Lycaenidae*	*Iolana*	*debilitata*	Fuente, El Vallecillo	Teruel	Aragón	Spain	1432
KP870489	EULEP036-14	07-W087	x	*Lycaenidae*	*Iolana*	*debilitata*	Carretera M219, Loeches		Comunidad de Madrid	Spain	
KP851160	EULEP002-14	RVcoll.12-P222		*Papilionidae*	*Iphiclides*	*podalirius*	Caves		Aude	France	65
KP851159	EULEP003-14	RVcoll.12-P223		*Papilionidae*	*Iphiclides*	*podalirius*	Caves		Aude	France	65
HM393171	GWORA2430-09	BC ZSM Lep 30642		*Papilionidae*	*Iphiclides*	*podalirius*	Regensburg - Kallmuenz	Oberpfalz	Bavaria	Germany	300
GU676576	EZSPN561-09	RVcoll.08-J100	x	*Papilionidae*	*Iphiclides*	*podalirius*	Lanhoso (Castelo), Lanhoso, Povoa de Lanhoso		Minho	Portugal	380
HQ004554	EZROM220-08	RV-07-D235		*Papilionidae*	*Iphiclides*	*podalirius*	Dealul Istrita, Breaza	Buzau	Muntenia	Romania	540
HQ004552	EZROM351-08	RV-08-A029		*Papilionidae*	*Iphiclides*	*podalirius*	Dealul Istrita, Breaza	Buzau	Muntenia	Romania	525
HQ004551	EZRMN332-08	RVcoll.08-H001		*Papilionidae*	*Iphiclides*	*podalirius*	Cluj-Napoca, Hoia forest	Cluj	Transylvania	Romania	450
HQ004555	EZROM1029-08	RVcoll.08-M306		*Papilionidae*	*Iphiclides*	*podalirius*	Suatu	Cluj	Transylvania	Romania	400
HQ004553	EZROM219-08	RV-06-K576		*Papilionidae*	*Iphiclides*	*podalirius*	Badeni	Cluj	Transylvania	Romania	440
KP851163	EULEP001-14	10-C413		*Papilionidae*	*Iphiclides*	*podalirius*	Lăpuș-Prislop	Maramureș	Maramureş	Romania	
HQ004557	EZROM1028-08	RVcoll.08-M270		*Papilionidae*	*Iphiclides*	*podalirius*	4 Km SW of Cazanele Mari (around Plavisevita)	Mehedinti	Oltenia	Romania	110
HQ004556	EZROM1030-08	RVcoll.08-M475		*Papilionidae*	*Iphiclides*	*podalirius*	Babadag forest (10 Km S of Babadag)	Tulcea	Dobrogea	Romania	115
GU676064	EZSPM449-09	Rvcoll.09-X581	x	*Papilionidae*	*Iphiclides*	*podalirius*	2 km E Navascues		Navarra	Spain	680
KP851157	EZSPM803-12	RVcoll.090211SD11	x	*Papilionidae*	*Iphiclides*	*podalirius*	Sierra Espuna, Totana		Murcia	Spain	1411

GenBank accession number	BOLD Process ID	Sample ID	Used in the Iberian dataset	Family	Genus	Species	Exact Site	Region	State/Province	Country	Alt. (m)
KP851162	EZSPM629-12	RVcoll.11-E018	x	*Papilionidae*	*Iphiclides*	*podalirius*	Font de l`Arbre, Serra d`Aitana	Alicante	Comunidad Valenciana	Spain	1209
KP851156	EZROM725-08	RV-06-G438	x	*Papilionidae*	*Iphiclides*	*podalirius*	El Sola, El Brull	Barcelona	Catalonia	Spain	800
GU669796	EZSPC449-09	RVcoll.08-J383	x	*Papilionidae*	*Iphiclides*	*podalirius*	Turo del Fumet, La Floresta, Valles Occidental	Barcelona	Catalonia	Spain	315
GU676883	EZSPN477-09	RVcoll.08-H912	x	*Papilionidae*	*Iphiclides*	*podalirius*	Hervas, Carretera Hervas-La Garganta	Caceres	Extremadura	Spain	1007
GU675805	EZSPM275-09	SMcoll.08-J611	x	*Papilionidae*	*Iphiclides*	*podalirius*	Puerto de Santa Maria	Cadiz	Andalusia	Spain	
GU669797	EZSPC450-09	RVcoll.07-C075	x	*Papilionidae*	*Iphiclides*	*podalirius*	Puig Neulos, Alt Emporda	Girona	Catalonia	Spain	1110
GU676069	EZSPM419-09	Rvcoll.09-V842	x	*Papilionidae*	*Iphiclides*	*podalirius*	Coll de Banyuls, Alt Emporda	Girona	Catalonia	Spain	
GU676131	EZSPM447-09	Rvcoll.09-V592	x	*Papilionidae*	*Iphiclides*	*podalirius*	Seminario (Sierra Nevada)	Granada	Andalusia	Spain	1520
GU676067	EZSPM450-09	Rvcoll.09-V588	x	*Papilionidae*	*Iphiclides*	*podalirius*	Seminario (Sierra Nevada)	Granada	Andalusia	Spain	1520
KP851164	EZSPM611-12	RVcoll.11-D685	x	*Papilionidae*	*Iphiclides*	*podalirius*	La Zubia, Barranco de Corvales	Granada	Andalusia	Spain	970
GU676636	EZSPN403-09	RVcoll.08-H477	x	*Papilionidae*	*Iphiclides*	*podalirius*	Duron, La Alcarria	Guadalajara	Castilla-La Mancha	Spain	750
GU675876	EZSPM362-09	RVcoll.08-J820	x	*Papilionidae*	*Iphiclides*	*podalirius*	Columbrianos	Leon	Castilla y Leon	Spain	
GU669790	EZSPC451-09	RVcoll.06-G512	x	*Papilionidae*	*Iphiclides*	*podalirius*	Coll de Comiols, Pallars Jussa	Lleida	Catalonia	Spain	1050
GU669791	EZSPC452-09	RVcoll.08-R262	x	*Papilionidae*	*Iphiclides*	*podalirius*	Vilamos, Vall d`Aran	Lleida	Catalonia	Spain	1387
GU675734	EZSPM099-09	RVcoll.08-P429	x	*Papilionidae*	*Iphiclides*	*podalirius*	Candelario	Salamanca	Castilla y Leon	Spain	1132
GU676709	EZSPN330-09	RVcoll.08-H248	x	*Papilionidae*	*Iphiclides*	*podalirius*	Mequinenza	Zaragoza	Aragon	Spain	129
HM393217	GWORA2501-09	BC ZSM Lep 30713		*Nymphalidae*	*Issoria*	*lathonia*	Falkenfels, Hirschberg	Niederbayern	Bavaria	Germany	
KP871178	FBLMX196-11	BC ZSM Lep 50407		*Nymphalidae*	*Issoria*	*lathonia*	Haiming (Aoe)	Oberbayern	Bavaria	Germany	350
HQ563608	FBLMW377-10	BC ZSM Lep 37478		*Nymphalidae*	*Issoria*	*lathonia*	Regenstauf, Kreuth	Oberpfalz	Bavaria	Germany	520
HQ004566	EZROM1031-08	RVcoll.08-M211		*Nymphalidae*	*Issoria*	*lathonia*	3 Km SW of Lunca Corbului	Arges	Muntenia	Romania	235
HQ004567	EZROM1033-08	RVcoll.08-M366		*Nymphalidae*	*Issoria*	*lathonia*	Buciumeni forest (Radacinesti)	Bacau	Moldavia	Romania	180
HQ004559	EZROM224-08	RV-07-D237		*Nymphalidae*	*Issoria*	*lathonia*	Dealul Istrita, Breaza	Buzau	Muntenia	Romania	540
HQ004563	EZROM332-08	RV-08-A010		*Nymphalidae*	*Issoria*	*lathonia*	Dealul Istrita, Breaza	Buzau	Muntenia	Romania	525
HQ004564	EZROM334-08	RV-08-A012		*Nymphalidae*	*Issoria*	*lathonia*	Dealul Istrita, Breaza	Buzau	Muntenia	Romania	525
HQ004561	EZROM221-08	RV-06-M895		*Nymphalidae*	*Issoria*	*lathonia*	Cluj-Napoca, dealul Feleacului, Gheorgheni forest	Cluj	Transylvania	Romania	600
HQ004562	EZROM222-08	RV-06-M914		*Nymphalidae*	*Issoria*	*lathonia*	Cluj-Napoca dealul Bogomaia (northern slope)	Cluj	Transylvania	Romania	550
HQ004560	EZROM223-08	RV-07-D165		*Nymphalidae*	*Issoria*	*lathonia*	Dumbraveni forest	Constanta	Dobrogea	Romania	80
HQ004558	EZROM632-08	RV-07-D953		*Nymphalidae*	*Issoria*	*lathonia*	11 km south of Ranca	Gorj	Oltenia	Romania	940
HQ004565	EZROM1032-08	RVcoll.08-M239		*Nymphalidae*	*Issoria*	*lathonia*	7 Km E of Strehaia	Mehedinti	Oltenia	Romania	125
FJ663686	LOWA287-06	2005-LOWA-287		*Nymphalidae*	*Issoria*	*lathonia*	Kislovodsk	Stavropol Region	Stavropol	Russia	
GU675708	EZSPM131-09	RVcoll.08-P640	x	*Nymphalidae*	*Issoria*	*lathonia*	Los Fontanares, Pena Munoz, Lena		Asturias	Spain	1331
GU676642	EZSPN397-09	RVcoll.08-H462	x	*Nymphalidae*	*Issoria*	*lathonia*	Campo Real		Comunidad de Madrid	Spain	727
GU676310	EZSPN841-09	RVcoll.08-L670	x	*Nymphalidae*	*Issoria*	*lathonia*	Candeleda, Cuesta de la Joya	Avila	Castilla y Leon	Spain	415
KP870312	EZROM726-08	RV-06-G427	x	*Nymphalidae*	*Issoria*	*lathonia*	El Burgues, Coll Formic, Montseny	Barcelona	Catalonia	Spain	1000
KP870695	EZSPM958-12	Rvcoll.12-M709	x	*Nymphalidae*	*Issoria*	*lathonia*	Alrededores San Lorenzo de Calatrava	Ciudad Real	Castilla-La Mancha	Spain	749
GU669792	EZSPC453-09	RVcoll.08-H210	x	*Nymphalidae*	*Issoria*	*lathonia*	Setcases, El Ripolles	Girona	Catalonia	Spain	1321
GU669787	EZSPC456-09	RVcoll.08-P009	x	*Nymphalidae*	*Issoria*	*lathonia*	Torre de Riu, Ribera d`Alp, Cerdanya	Girona	Catalonia	Spain	1180
GU676837	EZSPN100-09	RVcoll.06-A039	x	*Nymphalidae*	*Issoria*	*lathonia*	Barranco de los Lagartos, Cadiar	Granada	Andalusia	Spain	860
GU676412	EZSPN734-09	RVcoll.08-L142	x	*Nymphalidae*	*Issoria*	*lathonia*	Laguna Seca, Hueneja	Granada	Andalusia	Spain	2276
GU676040	EZSPM085-09	RVcoll.08-P408	x	*Nymphalidae*	*Issoria*	*lathonia*	Buron	Leon	Castilla y Leon	Spain	1116
GU669793	EZSPC454-09	RVcoll.08-M903	x	*Nymphalidae*	*Issoria*	*lathonia*	Bassa d`Arres, Vall d`Aran	Lleida	Catalonia	Spain	1500
GU669786	EZSPC455-09	RVcoll.08-P389	x	*Nymphalidae*	*Issoria*	*lathonia*	Arros, Vall d`Aran	Lleida	Catalonia	Spain	906
GU676673	EZSPN367-09	RVcoll.08-H392	x	*Nymphalidae*	*Issoria*	*lathonia*	La Canada, Paterna	Valencia	Comunidad Valenciana	Spain	
GU676704	EZSPN333-09	RVcoll.08-H258	x	*Nymphalidae*	*Issoria*	*lathonia*	Mequinenza	Zaragoza	Aragon	Spain	129
HQ004570	EZROM225-08	RV-07-C959		*Nymphalidae*	*Kirinia*	*roxelana*	Baile Herculane (Pecinisca)	Caras-Severin	Banat	Romania	220
HQ004569	EZRMN001-08	RVcoll.08-M665		*Nymphalidae*	*Kirinia*	*roxelana*	Esechioi forest	Constanta	Dobrogea	Romania	135
HQ004568	EZRMN002-08	RVcoll.08-M666		*Nymphalidae*	*Kirinia*	*roxelana*	Esechioi forest	Constanta	Dobrogea	Romania	135
HQ004571	EZROM1034-08	RVcoll.08-M664		*Nymphalidae*	*Kirinia*	*roxelana*	Esechioi forest	Constanta	Dobrogea	Romania	135
HM901212	EZSPC1008-10	RVcoll.08-H466	x	*Lycaenidae*	*Kretania*	*hesperica*	Campo Real		Comunidad de Madrid	Spain	686

GenBank accession number	BOLD Process ID	Sample ID	Used in the Iberian dataset	Family	Genus	Species	Exact Site	Region	State/Province	Country	Alt. (m)
HM901332	EZSPC1132-10	RVcoll.08-P710	x	*Lycaenidae*	*Kretania*	*hesperica*	Campo Real		Comunidad de Madrid	Spain	693
GU677049	EZSPN355-09	RVcoll.08-H330	x	*Lycaenidae*	*Kretania*	*hesperica*	Campo Real, Campo Real		Comunidad de Madrid	Spain	
GU676644	EZSPN399-09	RVcoll.08-H465	x	*Lycaenidae*	*Kretania*	*hesperica*	Campo Real		Comunidad de Madrid	Spain	686
GU676770	EZSPN165-09	RVcoll.06-K689	x	*Lycaenidae*	*Kretania*	*hesperica*	Sierra de Orce, Orce	Granada	Andalusia	Spain	925
GU676771	EZSPN166-09	RVcoll.06-K690	x	*Lycaenidae*	*Kretania*	*hesperica*	Sierra de Orce, Orce	Granada	Andalusia	Spain	925
GU676450	EZSPN699-09	RVcoll.08-L042	x	*Lycaenidae*	*Kretania*	*hesperica*	Sierra Elvira, Albolote	Granada	Andalusia	Spain	819
GU676443	EZSPN707-09	RVcoll.08-L056	x	*Lycaenidae*	*Kretania*	*hesperica*	Sierra Elvira, Albolote	Granada	Andalusia	Spain	819
GU676435	EZSPN708-09	RVcoll.08-L057	x	*Lycaenidae*	*Kretania*	*hesperica*	Sierra Elvira, Albolote	Granada	Andalusia	Spain	819
GU676434	EZSPN715-09	RVcoll.08-L091	x	*Lycaenidae*	*Kretania*	*hesperica*	Ruta de la Sima, El Vallecillo	Teruel	Aragon	Spain	1531
GU676422	EZSPN717-09	RVcoll.08-L095	x	*Lycaenidae*	*Kretania*	*hesperica*	Ruta de la Sima, El Vallecillo	Teruel	Aragon	Spain	1531
GQ128990		AD-00-P066		*Lycaenidae*	*Kretania*	*pylaon*	Kamyshinsky		Volgograd	Russia	
HQ004998	EZRMN195-08	RVcoll.08-M284		*Lycaenidae*	*Kretania*	*sephirus*	Suatu	Cluj	Transylvania	Romania	400
HQ004997	EZRMN196-08	RVcoll.08-M285		*Lycaenidae*	*Kretania*	*sephirus*	Suatu	Cluj	Transylvania	Romania	400
HQ004996	EZRMN197-08	RVcoll.08-M286		*Lycaenidae*	*Kretania*	*sephirus*	Suatu	Cluj	Transylvania	Romania	400
HQ004995	EZRMN198-08	RVcoll.08-M287		*Lycaenidae*	*Kretania*	*sephirus*	Suatu	Cluj	Transylvania	Romania	400
HQ004994	EZRMN199-08	RVcoll.08-M288		*Lycaenidae*	*Kretania*	*sephirus*	Suatu	Cluj	Transylvania	Romania	400
HQ004999	EZROM160-08	RV-07-D404		*Lycaenidae*	*Kretania*	*sephirus*	Suatu	Cluj	Transylvania	Romania	380
HQ005000	EZROM490-08	RV-07-D428		*Lycaenidae*	*Kretania*	*sephirus*	Suatu	Cluj	Transylvania	Romania	380
HQ005001	EZROM491-08	RV-07-D436		*Lycaenidae*	*Kretania*	*sephirus*	Suatu	Cluj	Transylvania	Romania	380
HM901289	EZSPC1088-10	RVcoll.08-L850	x	*Lycaenidae*	*Laeosopis*	*roboris*	Pinilla del Valle		Comunidad de Madrid	Spain	1100
GU676289	EZSPN864-09	RVcoll.08-L735	x	*Lycaenidae*	*Laeosopis*	*roboris*	Manzanares el Real		Comunidad de Madrid	Spain	934
GU676243	EZSPN912-09	RVcoll.08-L849	x	*Lycaenidae*	*Laeosopis*	*roboris*	Pinilla del Valle		Comunidad de Madrid	Spain	1100
HM901391	EZSPC1191-10	RVcoll.09-V339	x	*Lycaenidae*	*Laeosopis*	*roboris*	Viladrau, Montseny, Osona	Barcelona	Catalonia	Spain	715
KP870677	EZSPM1033-12	Rvcoll.12-M783	x	*Lycaenidae*	*Laeosopis*	*roboris*	Merendero de los Horcones, Navatrasierra	Caceres	Extremadura	Spain	664
KP870612	EZSPM1034-12	Rvcoll.12-M784	x	*Lycaenidae*	*Laeosopis*	*roboris*	Merendero de los Horcones, Navatrasierra	Caceres	Extremadura	Spain	664
GU676882	EZSPN478-09	RVcoll.08-H913	x	*Lycaenidae*	*Laeosopis*	*roboris*	Hervas, Carretera Hervas-La Garganta	Caceres	Extremadura	Spain	774
KP871011	EZSPM931-12	Rvcoll.12-M663	x	*Lycaenidae*	*Laeosopis*	*roboris*	Mina de los Pontones, Mestanza	Ciudad Real	Castilla-La Mancha	Spain	450
KP870365	EZSPM951-12	Rvcoll.12-M696	x	*Lycaenidae*	*Laeosopis*	*roboris*	Rio Jandula, Mestanza	Ciudad Real	Castilla-La Mancha	Spain	407
KP870450	EZSPM980-12	Rvcoll.12-M742	x	*Lycaenidae*	*Laeosopis*	*roboris*	Arroyo de los Molinillos, Viso del Marques	Ciudad Real	Castilla-La Mancha	Spain	742
KP870746	EULEP247-14	12-N763	x	*Lycaenidae*	*Laeosopis*	*roboris*	Huélamo	Cuenca	Castilla-La Mancha	Spain	1237.3
GU676192	EZSPN968-09	RVcoll.08-L985	x	*Lycaenidae*	*Laeosopis*	*roboris*	Una	Cuenca	Castilla-La Mancha	Spain	1201
GU675972	EZSPM024-09	RVcoll.08-P091	x	*Lycaenidae*	*Laeosopis*	*roboris*	Estoll, Fontanals de Cerdanya, Cerdanya	Girona	Catalonia	Spain	1100
KP870890	EZSPM793-12	RVcoll.070611MH72	x	*Lycaenidae*	*Laeosopis*	*roboris*	El Pueyo de Jaca, Panticosa	Huesca	Aragon	Spain	1779
KP870859	EZSPM834-12	RVcoll.140711ZG21	x	*Lycaenidae*	*Laeosopis*	*roboris*	Balneario de Panticosa	Huesca	Aragon	Spain	1642
KP870336	EZSPM843-12	RVcoll.140711ZG98	x	*Lycaenidae*	*Laeosopis*	*roboris*	Balneario de Panticosa	Huesca	Aragon	Spain	1642
GU675921	EZSPM313-09	RVcoll.08-J868	x	*Lycaenidae*	*Laeosopis*	*roboris*	Posada de Valdeon	Leon	Castilla y Leon	Spain	
HM901498	EZSPC1364-10	RVcoll.09-X023	x	*Lycaenidae*	*Laeosopis*	*roboris*	La Farga Vella, La Cerdanya	Lleida	Catalonia	Spain	1100
GU675969	EZSPM079-09	RVcoll.08-P362	x	*Lycaenidae*	*Laeosopis*	*roboris*	Perves, Pallars Jussa	Lleida	Catalonia	Spain	1072
KP871042	EZSPM699-12	RVcoll.12-L601	x	*Lycaenidae*	*Lampides*	*boeticus*	Caldas de Monchique, Monchique		Algarve	Portugal	
GU676541	EZSPN598-09	RVcoll.08-J188	x	*Lycaenidae*	*Lampides*	*boeticus*	Serra do Larouco (turfeira N), Padornelos, Montalegre		Tras-os-Montes	Portugal	1360
HQ004573	EZROM226-08	RV-07-E423		*Lycaenidae*	*Lampides*	*boeticus*	Nifon (Macin Mts.)	Tulcea	Dobrogea	Romania	120
HQ004574	EZROM227-08	RV-07-E443		*Lycaenidae*	*Lampides*	*boeticus*	2 km E of Smardan	Tulcea	Dobrogea	Romania	5
HQ004572	EZROM673-08	RV-07-E424		*Lycaenidae*	*Lampides*	*boeticus*	Nifon (Macin Mts.)	Tulcea	Dobrogea	Romania	120
GU675748	EZSPM103-09	RVcoll.08-P435	x	*Lycaenidae*	*Lampides*	*boeticus*	Sonabia		Cantabria	Spain	16
KP870735	EZROM728-08	RV-06-G522	x	*Lycaenidae*	*Lampides*	*boeticus*	Santuari de Puiglagulla, Vilalleons	Barcelona	Catalonia	Spain	720
GU676885	EZSPN479-09	RVcoll.08-H914	x	*Lycaenidae*	*Lampides*	*boeticus*	Hervas, Carretera Hervas-La Garganta	Caceres	Extremadura	Spain	774
GU676589	EZSPN454-09	RVcoll.08-H659	x	*Lycaenidae*	*Lampides*	*boeticus*	Ubrique, Puerto de la Vibora	Cadiz	Andalusia	Spain	405
GU676517	EZSPN623-09	RVcoll.08-J707	x	*Lycaenidae*	*Lampides*	*boeticus*	Alrededores de Almedijar, Almedijar	Castellon	Comunidad Valenciana	Spain	
KP870421	EZSPM960-12	Rvcoll.12-M711	x	*Lycaenidae*	*Lampides*	*boeticus*	Alrededores San Lorenzo de Calatrava	Ciudad Real	Castilla-La Mancha	Spain	749

GenBank accession number	BOLD Process ID	Sample ID	Used in the Iberian dataset	Family	Genus	Species	Exact Site	Region	State/Province	Country	Alt. (m)
KP870810	EZSPM970-12	Rvcoll.12-M728	x	*Lycaenidae*	*Lampides*	*boeticus*	Arroyo de los Molinillos, Viso del Marques	Ciudad Real	Castilla-La Mancha	Spain	742
GU669788	EZSPC457-09	RVcoll.07-C491	x	*Lycaenidae*	*Lampides*	*boeticus*	Meranges	Girona	Catalonia	Spain	1555
GU669783	EZSPC460-09	RVcoll.08-P005	x	*Lycaenidae*	*Lampides*	*boeticus*	Torre de Riu, Ribera d`Alp, Cerdanya	Girona	Catalonia	Spain	1180
GU676833	EZSPN097-09	RVcoll.06-A033	x	*Lycaenidae*	*Lampides*	*boeticus*	Barranco de los Lagartos, Cadiar	Granada	Andalusia	Spain	860
GU676444	EZSPN700-09	RVcoll.08-L043	x	*Lycaenidae*	*Lampides*	*boeticus*	Sierra Elvira, Albolote	Granada	Andalusia	Spain	819
GU676141	EZSPN1028-09	RVcoll.08-M963	x	*Lycaenidae*	*Lampides*	*boeticus*	Saganta	Huesca	Aragon	Spain	767
GU675867	EZSPM369-09	RVcoll.08-J839	x	*Lycaenidae*	*Lampides*	*boeticus*	Puerto de San Glorio	Leon	Castilla y Leon	Spain	
GU669789	EZSPC458-09	RVcoll.08-P329	x	*Lycaenidae*	*Lampides*	*boeticus*	Perves, Pallars Jussa	Lleida	Catalonia	Spain	1071
GU675963	EZSPM037-09	RVcoll.08-P240	x	*Lycaenidae*	*Lampides*	*boeticus*	Puig d`en Galileu	Mallorca	Balearic Islands	Spain	1071
KP870264	EZSPM1006-12	Rvcoll.12-L895	x	*Lycaenidae*	*Lampides*	*boeticus*	Turdonell de Dalt, Mao, Menorca	Menorca	Balearic Islands	Spain	
GU669782	EZSPC459-09	RVcoll.08-M688	x	*Lycaenidae*	*Lampides*	*boeticus*	Farena, Alt Camp	Tarragona	Catalonia	Spain	580
KP871149	EZSPM900-12	Rvcoll.12-M622	x	*Lycaenidae*	*Lampides*	*boeticus*	Tudela de Duero	Valladolid	Castilla y Leon	Spain	713
GU707102	FBLMU437-09	BC ZSM Lep 27087		*Nymphalidae*	*Lasiommata*	*maera*	Diessen, Huebschenried	Oberbayern	Bavaria	Germany	650
JF415709	GWORK564-09	BC ZSM Lep 21894		*Nymphalidae*	*Lasiommata*	*maera*	Karwendelgebirge, Mittenwald, Vereinsalm	Oberbayern	Bavaria	Germany	1600
HM910006	GWORT460-10	BC ZSM Lep 32146		*Nymphalidae*	*Lasiommata*	*maera*	Starnberg, Machtlfing	Oberbayern	Bavaria	Germany	715
HQ004580	EZRMN003-08	RVcoll.06-M986		*Nymphalidae*	*Lasiommata*	*maera*	Apuseni Mts., Rimetea (Piatra Secuiului)	Alba	Transylvania	Romania	750
HQ004577	EZROM230-08	RV-07-D092		*Nymphalidae*	*Lasiommata*	*maera*	Dealul Istrita, Breaza	Buzau	Muntenia	Romania	540
HQ004579	EZROM232-08	RV-07-D219		*Nymphalidae*	*Lasiommata*	*maera*	Dealul Istrita, Breaza	Buzau	Muntenia	Romania	540
GU669652	EZROM1056-09	RVcoll.07-E341		*Nymphalidae*	*Lasiommata*	*maera*	Baile Herculane (Pecinisca)	Caras-Severin	Banat	Romania	250
HQ004575	EZROM228-08	RV-06-K626		*Nymphalidae*	*Lasiommata*	*maera*	Baciu forest (Cluj-Napoca)	Cluj	Transylvania	Romania	530
HQ004576	EZROM229-08	RV-06-M844		*Nymphalidae*	*Lasiommata*	*maera*	Cluj-Napoca valea paraului Micus	Cluj	Transylvania	Romania	700
GU669659	EZROM1048-09	RVcoll.07-D183		*Nymphalidae*	*Lasiommata*	*maera*	Sipote	Constanta	Dobrogea	Romania	90
HQ004578	EZROM231-08	RV-07-D153		*Nymphalidae*	*Lasiommata*	*maera*	Dumbraveni forest	Constanta	Dobrogea	Romania	80
HQ004582	EZRMN005-08	RVcoll.08-M594		*Nymphalidae*	*Lasiommata*	*maera*	Gheorgheni, Valea Belchia	Harghita	Transylvania	Romania	1000
GU669655	EZROM1052-09	RVcoll.07-D495		*Nymphalidae*	*Lasiommata*	*maera*	Cheile Babei	Maramures	Maramures	Romania	265
HQ004581	EZRMN004-08	RVcoll.08-M494		*Nymphalidae*	*Lasiommata*	*maera*	Cota 1400 m (Bucegi Mts.), Sinaia	Prahova	Muntenia	Romania	1385
GU669662	EZROM1043-09	RVcoll.07-D062		*Nymphalidae*	*Lasiommata*	*maera*	Horia	Tulcea	Dobrogea	Romania	140
GU675702	EZSPM145-09	RVcoll.08-P658	x	*Nymphalidae*	*Lasiommata*	*maera*	Puerto de la Cubilla		Asturias	Spain	1689
GU676197	EZSPN961-09	RVcoll.08-L966	x	*Nymphalidae*	*Lasiommata*	*maera*	La Morcuera, Rascafria		Comunidad de Madrid	Spain	1766
GU676186	EZSPN974-09	RVcoll.08-L999	x	*Nymphalidae*	*Lasiommata*	*maera*	Cotillos		Cantabria	Spain	1105
KP870998	EZSPM766-12	RVcoll.040611HZ35	x	*Nymphalidae*	*Lasiommata*	*maera*	El Montgo _ Nivel Alto, Xavia	Alicante	Comunidad Valenciana	Spain	629
KP870568	EZSPM779-12	RVcoll.070111GN29	x	*Nymphalidae*	*Lasiommata*	*maera*	El Montgo _ Nivel Medio, Xavia	Alicante	Comunidad Valenciana	Spain	161
KP870316	WMB010-11	RVcoll.11-E019	x	*Nymphalidae*	*Lasiommata*	*maera*	Font de l`Arbre, Serra d`Aitana	Alicante	Comunidad Valenciana	Spain	1209
KP870566	EZROM368-08	07-E617	x	*Nymphalidae*	*Lasiommata*	*maera*	Vallforners, Canoves	Barcelona	Catalonia	Spain	650
KP870318	EZROM369-08	07-E618	x	*Nymphalidae*	*Lasiommata*	*maera*	Can Prat, Gualba	Barcelona	Catalonia	Spain	530
HM901430	EZSPC1234-10	RVcoll.09-V705	x	*Nymphalidae*	*Lasiommata*	*maera*	Coll Formic, El Brull, Osona	Barcelona	Catalonia	Spain	1170
GU676115	EZSPM406-09	Rvcoll.09-V818	x	*Nymphalidae*	*Lasiommata*	*maera*	Vilallonga de Ter, Ripolles	Girona	Catalonia	Spain	
HM901434	EZSPC1238-10	RVcoll.09-V746	x	*Nymphalidae*	*Lasiommata*	*maera*	Valle del Tena, Camino embalse Respomuso, Sallent de Gallego	Huesca	Aragon	Spain	1650
HM901518	EZSPC1384-10	RVcoll.09-X531	x	*Nymphalidae*	*Lasiommata*	*maera*	Buron	Leon	Castilla y Leon	Spain	1120
GU675869	EZSPM367-09	RVcoll.08-J832	x	*Nymphalidae*	*Lasiommata*	*maera*	Puerto de Monteviejo	Leon	Castilla y Leon	Spain	
GU669784	EZSPC461-09	RVcoll.08-M907	x	*Nymphalidae*	*Lasiommata*	*maera*	Bassa d`Arres, Vall d`Aran	Lleida	Catalonia	Spain	1500
GU669785	EZSPC462-09	RVcoll.08-M926	x	*Nymphalidae*	*Lasiommata*	*maera*	Vilamos, Vall d`Aran	Lleida	Catalonia	Spain	1178
GU669778	EZSPC463-09	RVcoll.08-R110	x	*Nymphalidae*	*Lasiommata*	*maera*	Salt de Pitx, Vall d`Aran	Lleida	Catalonia	Spain	1570
GU675812	EZSPM264-09	RVcoll.08-R479	x	*Nymphalidae*	*Lasiommata*	*maera*	Pedrafita do Cebreiro, Serra do Caurel-Hospital	Lugo	Galicia	Spain	1230
KP870683	WMB009-11	RVcoll.11-D967	x	*Nymphalidae*	*Lasiommata*	*maera*	Istan	Malaga	Andalusia	Spain	283
GU676184	EZSPN972-09	RVcoll.08-L997	x	*Nymphalidae*	*Lasiommata*	*maera*	Puerto de Piedrasluengas	Palencia	Castilla y Leon	Spain	1400
GU676190	EZSPN966-09	RVcoll.08-L980	x	*Nymphalidae*	*Lasiommata*	*maera*	Navafria, El Nevero	Segovia	Castilla y Leon	Spain	2130
GU655015	FBLMT903-09	BC ZSM Lep 25463		*Nymphalidae*	*Lasiommata*	*megera*	Kallmuenz, Kirchenberg	Oberpfalz	Bavaria	Germany	390
GU676543	EZSPN600-09	RVcoll.08-J191	x	*Nymphalidae*	*Lasiommata*	*megera*	Seculca, Beca, Boticas		Tras-os-Montes	Portugal	790
HQ004587	EZROM235-08	RV-07-D216		*Nymphalidae*	*Lasiommata*	*megera*	Dealul Istrita, Breaza	Buzau	Muntenia	Romania	540
KP870644	EULEP181-14	11-J413		*Nymphalidae*	*Lasiommata*	*megera*	Breaza, Dealul Istrița	Buzău	Muntenia	Romania	400-720

GenBank accession number	BOLD Process ID	Sample ID	Used in the Iberian dataset	Family	Genus	Species	Exact Site	Region	State/Province	Country	Alt. (m)
HQ004584	EZROM236-08	RV-07-C960		*Nymphalidae*	*Lasiommata*	*megera*	Baile Herculane (Pecinisca)	Caras-Severin	Banat	Romania	220
HQ004585	EZROM233-08	RV-06-K633		*Nymphalidae*	*Lasiommata*	*megera*	Baciu forest (Cluj-Napoca)	Cluj	Transylvania	Romania	530
HQ004586	EZROM234-08	RV-06-V655		*Nymphalidae*	*Lasiommata*	*megera*	Faget forest, Ratu Penii	Cluj	Transylvania	Romania	680
HQ004583	EZROM633-08	RV-07-D159		*Nymphalidae*	*Lasiommata*	*megera*	Dumbraveni forest	Constanta	Dobrogea	Romania	80
HQ004588	EZRMN006-08	RVcoll.07-D067		*Nymphalidae*	*Lasiommata*	*megera*	Greci massif, Macin Mts.	Tulcea	Dobrogea	Romania	200
HQ004589	EZRMN007-08	RVcoll.08-M403		*Nymphalidae*	*Lasiommata*	*megera*	Macin Mts. (3 Km S of Jijila)	Tulcea	Dobrogea	Romania	230
GU676296	EZSPN860-09	RVcoll.08-L723	x	*Nymphalidae*	*Lasiommata*	*megera*	Arganda del Rey		Comunidad de Madrid	Spain	689
GU677051	EZSPN353-09	RVcoll.08-H324	x	*Nymphalidae*	*Lasiommata*	*megera*	Cebreros, Cebreros	Avila	Castilla y Leon	Spain	
KP870491	EZROM729-08	RV-07-C005	x	*Nymphalidae*	*Lasiommata*	*megera*	Vallforners, Canoves	Barcelona	Catalonia	Spain	650
GU669779	EZSPC464-09	RVcoll.08-J366	x	*Nymphalidae*	*Lasiommata*	*megera*	Vallgrassa, Parc Natural del Garraf	Barcelona	Catalonia	Spain	285
KP870668	EZSPM1019-12	Rvcoll.12-M768	x	*Nymphalidae*	*Lasiommata*	*megera*	7 km SE Navalvillar de Ibor	Caceres	Extremadura	Spain	729
GU676330	EZSPN816-09	RVcoll.08-L621	x	*Nymphalidae*	*Lasiommata*	*megera*	Algeciras, Arroyo del Prior	Cadiz	Andalusia	Spain	50
GU676433	EZSPN714-09	RVcoll.08-L080	x	*Nymphalidae*	*Lasiommata*	*megera*	Rambla de las Truchas, Vilafranca	Castellon	Comunidad Valenciana	Spain	1150
KP870726	EZSPM925-12	Rvcoll.12-M648	x	*Nymphalidae*	*Lasiommata*	*megera*	Lagunas de Ruidera, Ossa de Montiel	Ciudad Real	Castilla-La Mancha	Spain	795
KP870753	EZSPM926-12	Rvcoll.12-M649	x	*Nymphalidae*	*Lasiommata*	*megera*	Lagunas de Ruidera, Ossa de Montiel	Ciudad Real	Castilla-La Mancha	Spain	795
GU669780	EZSPC465-09	RVcoll.08-J348	x	*Nymphalidae*	*Lasiommata*	*megera*	Cantallops, Alt Emporda	Girona	Catalonia	Spain	225
GU676845	EZSPN085-09	RVcoll.06-A004	x	*Nymphalidae*	*Lasiommata*	*megera*	Road from Ferreira to Puerto de La Ragua	Granada	Andalusia	Spain	1600
GU676528	EZSPN610-09	RVcoll.08-J330	x	*Nymphalidae*	*Lasiommata*	*megera*	Barranco de Valcuerna, Candasnos	Huesca	Aragon	Spain	210
GU669781	EZSPC466-09	RVcoll.08-J343	x	*Nymphalidae*	*Lasiommata*	*megera*	Coll de Comiols, Pallars Jussa	Lleida	Catalonia	Spain	1130
GU669776	EZSPC467-09	RVcoll.08-L427	x	*Nymphalidae*	*Lasiommata*	*megera*	Rius de Cerdanya	Lleida	Catalonia	Spain	1273
GU676007	EZSPM036-09	RVcoll.08-P239	x	*Nymphalidae*	*Lasiommata*	*megera*	Puig d`en Galileu	Mallorca	Balearic Islands	Spain	1071
GU676005	EZSPM041-09	RVcoll.08-P249	x	*Nymphalidae*	*Lasiommata*	*megera*	Santa Maria	Mallorca	Balearic Islands	Spain	181
GU676008	EZSPM065-09	RVcoll.08-P298	x	*Nymphalidae*	*Lasiommata*	*megera*	Genova	Mallorca	Balearic Islands	Spain	123
GU675809	EZSPM208-09	RVcoll.08-R326	x	*Nymphalidae*	*Lasiommata*	*megera*	Pic Tomir	Mallorca	Balearic Islands	Spain	1048
GU675810	EZSPM211-09	RVcoll.08-R332	x	*Nymphalidae*	*Lasiommata*	*megera*	Coanegra, Sta. Maria del cami	Mallorca	Balearic Islands	Spain	125
KP870461	EZSPM1000-12	Rvcoll.12-L886	x	*Nymphalidae*	*Lasiommata*	*megera*	Sant Antoni de Ruma, Ferreries, Menorca	Menorca	Balearic Islands	Spain	
GU676112	EZSPM433-09	Rvcoll.09-X118	x	*Nymphalidae*	*Lasiommata*	*megera*	Parque Albufera des Grau, Menorca	Menorca	Balearic Islands	Spain	
KP870492	EZSPN302-09	RVcoll.08-H107	x	*Nymphalidae*	*Lasiommata*	*megera*	Na Vermella, Mao	Menorca	Balearic Islands	Spain	
GU676902	EZSPN499-09	RVcoll.08-H948	x	*Nymphalidae*	*Lasiommata*	*megera*	Candelario, Sierra de Candelario	Salamanca	Castilla y Leon	Spain	1300
JN278896	EZSPN1122-11	Rvcoll.10-C352	x	*Nymphalidae*	*Lasiommata*	*megera*	Sant Jordi d`Alfama, Baix Ebre	Tarragona	Catalonia	Spain	145
GU676674	EZSPN368-09	RVcoll.08-H393	x	*Nymphalidae*	*Lasiommata*	*megera*	Lliria, Lliria	Valencia	Comunidad Valenciana	Spain	
HM391833	FBLMU491-09	BC ZSM Lep 27141		*Nymphalidae*	*Lasiommata*	*petropolitana*	Melleck	Oberbayern	Bavaria	Germany	550
HM393202	GWORA2480-09	BC ZSM Lep 30692		*Nymphalidae*	*Lasiommata*	*petropolitana*	Arzmoos	Oberbayern	Bavaria	Germany	1200
HM901705	EZSPC864-10	RVcoll.09-T078	x	*Nymphalidae*	*Lasiommata*	*petropolitana*	Vall d`Hurno, Vall d`Aran	Lleida	Catalonia	Spain	1800
KP870736	EZSPN1038-11	Rvcoll.07-W120	x	*Nymphalidae*	*Lasiommata*	*petropolitana*	Vall d`Hurno, Vall d`Aran	Lleida	Catalonia	Spain	1720
GU677043	EZSPN243-09	RVcoll.07-W114	x	*Nymphalidae*	*Lasiommata*	*petropolitana*	Val d`Hurno, Vall d`Aran	Lleida	Catalonia	Spain	1720
GU676978	EZSPN244-09	RVcoll.07-W116	x	*Nymphalidae*	*Lasiommata*	*petropolitana*	Val d`Hurno, Vall d`Aran	Lleida	Catalonia	Spain	1720
GU676979	EZSPN245-09	RVcoll.07-W118	x	*Nymphalidae*	*Lasiommata*	*petropolitana*	Val d`Hurno, Vall d`Aran	Lleida	Catalonia	Spain	1720
GU676976	EZSPN246-09	RVcoll.07-W119	x	*Nymphalidae*	*Lasiommata*	*petropolitana*	Val d`Hurno, Vall d`Aran	Lleida	Catalonia	Spain	1720
JF512571		RVcoll.10-B373		*Pieridae*	*Leptidea*	*duponcheli*	Paril village		Hadjidimovo	Bulgaria	800
JF512569		RVcoll.09-V207		*Pieridae*	*Leptidea*	*duponcheli*	Oraison		Alpes-de-Haute-Provence	France	500
JF512570		RVcoll.10-A612		*Pieridae*	*Leptidea*	*duponcheli*	Digne les Bains		Alpes-de-Haute-Provence	France	710
JF512645		RVcoll.10-C244		*Pieridae*	*Leptidea*	*juvernica*	Príbram		Bohemia	CzechRepublic	500
JF512650		RVcoll.09-X268		*Pieridae*	*Leptidea*	*juvernica*	St. Etienne		Loire	France	550
JF512651		RVcoll.10-A259		*Pieridae*	*Leptidea*	*juvernica*	Gresse-en-Vercors		Isère	France	1260
JF512652		RVcoll.10-A262		*Pieridae*	*Leptidea*	*juvernica*	Gresse-en-Vercors		Isère	France	1340
JF512653		RVcoll.10-A263		*Pieridae*	*Leptidea*	*juvernica*	Gresse-en-Vercors		Isère	France	1340
JF512654		RVcoll.10-A264		*Pieridae*	*Leptidea*	*juvernica*	Gresse-en-Vercors		Isère	France	1340
JF512655		RVcoll.10-A269		*Pieridae*	*Leptidea*	*juvernica*	Gresse-en-Vercors		Isère	France	1340
GU655014	FBLMT876-09	BC ZSM Lep 25436		*Pieridae*	*Leptidea*	*juvernica*	Neustadt/Donau, Plattenberg	Niederbayern	Bavaria	Germany	370
GU707187	FBLMU118-09	BC ZSM Lep 25628		*Pieridae*	*Leptidea*	*juvernica*	Kallmuenz, Kirchenberg	Oberpfalz	Bavaria	Germany	390

GenBank accession number	BOLD Process ID	Sample ID	Used in the Iberian dataset	Family	Genus	Species	Exact Site	Region	State/Province	Country	Alt. (m)
GU707144	FBLMU383-09	BC ZSM Lep 27033		*Pieridae*	*Leptidea*	*juvernica*	Sinzing, Alpiner Steig	Oberpfalz	Bavaria	Germany	405
JF512713		RVcoll.09-X176		*Pieridae*	*Leptidea*	*juvernica*	Murlough		Down	Ireland	3
JF512714		RVcoll.09-X178		*Pieridae*	*Leptidea*	*juvernica*	Milltown		Roscommon	Ireland	60
JF512715		RVcoll.09-X181		*Pieridae*	*Leptidea*	*juvernica*	Kilternan		Dublin	Ireland	130
JF512716		RVcoll.09-X183		*Pieridae*	*Leptidea*	*juvernica*	Gortmore Point, Lough Derg		Tipperary	Ireland	40
HQ004601	EZROM703-08	RV-07-E553		*Pieridae*	*Leptidea*	*juvernica*	Tohanul Nou	Brasov	Transylvania	Romania	700
HQ004600	EZRMN011-08	RVcoll.08-M310		*Pieridae*	*Leptidea*	*juvernica*	Gheorgheni	Harghita	Transylvania	Romania	850
HQ004599	EZRMN012-08	RVcoll.08-M311		*Pieridae*	*Leptidea*	*juvernica*	Gheorgheni	Harghita	Transylvania	Romania	850
HQ004595	EZRMN013-08	RVcoll.08-M312		*Pieridae*	*Leptidea*	*juvernica*	Gheorgheni	Harghita	Transylvania	Romania	850
HQ004596	EZRMN014-08	RVcoll.08-M322		*Pieridae*	*Leptidea*	*juvernica*	Valea Belchia (Gheorgheni)	Harghita	Transylvania	Romania	1010
HQ004594	EZRMN015-08	RVcoll.08-M323		*Pieridae*	*Leptidea*	*juvernica*	Valea Belchia (Gheorgheni)	Harghita	Transylvania	Romania	1010
HQ004597	EZRMN016-08	RVcoll.08-M324		*Pieridae*	*Leptidea*	*juvernica*	Valea Belchia (Gheorgheni)	Harghita	Transylvania	Romania	1010
HQ004598	EZRMN017-08	RVcoll.08-M331		*Pieridae*	*Leptidea*	*juvernica*	1 Km E of Gheorgheni	Harghita	Transylvania	Romania	900
JF512573		RVcoll.08-M325		*Pieridae*	*Leptidea*	*juvernica*	Valea Belchia	Harghita	Transylvania	Romania	1010
JF512628		RVcoll.08-M313		*Pieridae*	*Leptidea*	*juvernica*	Gheorgheni	Harghita	Transylvania	Romania	850
JF512635		RVcoll.09-V633		*Pieridae*	*Leptidea*	*juvernica*	Gheorgheni, Valea Belchia	Harghita	Transylvania	Romania	1000
JF512636		RVcoll.09-V637		*Pieridae*	*Leptidea*	*juvernica*	Gheorgheni, Valea Belchia	Harghita	Transylvania	Romania	1000
JF512637		RVcoll.09-V638		*Pieridae*	*Leptidea*	*juvernica*	Gheorgheni, Valea Belchia	Harghita	Transylvania	Romania	1000
JF512638		RVcoll.09-V639		*Pieridae*	*Leptidea*	*juvernica*	Gheorgheni, Valea Belchia	Harghita	Transylvania	Romania	1000
JF512639		RVcoll.09-V641		*Pieridae*	*Leptidea*	*juvernica*	Gheorgheni, Valea Belchia	Harghita	Transylvania	Romania	1000
JF512640		RVcoll.09-V644		*Pieridae*	*Leptidea*	*juvernica*	Gheorgheni, Valea Belchia	Harghita	Transylvania	Romania	1000
JF512641		RVcoll.09-V648		*Pieridae*	*Leptidea*	*juvernica*	Gheorgheni, Valea Belchia	Harghita	Transylvania	Romania	1000
JF512642		RVcoll.09-V652		*Pieridae*	*Leptidea*	*juvernica*	Gheorgheni, Valea Belchia	Harghita	Transylvania	Romania	1000
JF512643		RVcoll.09-V653		*Pieridae*	*Leptidea*	*juvernica*	Gheorgheni, Valea Belchia	Harghita	Transylvania	Romania	1000
JF512644		RVcoll.09-V655		*Pieridae*	*Leptidea*	*juvernica*	Gheorgheni, Valea Belchia	Harghita	Transylvania	Romania	1000
JF512574		RVcoll.08-Y003		*Pieridae*	*Leptidea*	*juvernica*	St. Petersburg		St. Petersburg	Russia	
JF512575		RVcoll.08-Y007		*Pieridae*	*Leptidea*	*juvernica*	Peterhof		St.Petersburg	Russia	
JF512576		RVcoll.08-Y010		*Pieridae*	*Leptidea*	*juvernica*	Peterhof		St.Petersburg	Russia	
JF512577		RVcoll.08-Y012		*Pieridae*	*Leptidea*	*juvernica*	Peterhof		St.Petersburg	Russia	
JF512578		RVcoll.08-Y008		*Pieridae*	*Leptidea*	*juvernica*	Peterhof		St.Petersburg	Russia	
JF512629		RVcoll.08-Y001		*Pieridae*	*Leptidea*	*juvernica*	St. Petersburg		St. Petersburg	Russia	
JF512630		RVcoll.08-Y002		*Pieridae*	*Leptidea*	*juvernica*	St. Petersburg		St. Petersburg	Russia	
JF512631		RVcoll.08-Y005		*Pieridae*	*Leptidea*	*juvernica*	St. Petersburg		St. Petersburg	Russia	
JF512632		RVcoll.08-Y006		*Pieridae*	*Leptidea*	*juvernica*	St. Petersburg		St. Petersburg	Russia	
JF512633		RVcoll.08-Y009		*Pieridae*	*Leptidea*	*juvernica*	St. Petersburg		St. Petersburg	Russia	
JF512634		RVcoll.08-Y011		*Pieridae*	*Leptidea*	*juvernica*	St. Petersburg		St. Petersburg	Russia	
JF512649		RVcoll.08-Y004		*Pieridae*	*Leptidea*	*juvernica*	St. Petersburg		St. Petersburg	Russia	
JF512646		RVcoll.LR08-D680		*Pieridae*	*Leptidea*	*juvernica*	Jagnjenica		Radeče	Slovenia	280
HQ004591	EZRMN008-08	RVcoll.08-M498		*Pieridae*	*Leptidea*	*morsei*	Badeni	Cluj	Transylvania	Romania	490
HQ004590	EZRMN009-08	RVcoll.08-M499		*Pieridae*	*Leptidea*	*morsei*	Badeni	Cluj	Transylvania	Romania	490
HQ004592	EZROM237-08	RV-07-C176		*Pieridae*	*Leptidea*	*morsei*	Hoia forest (Cluj-Napoca)	Cluj	Transylvania	Romania	450
HQ004593	EZROM348-08	RV-08-A026		*Pieridae*	*Leptidea*	*morsei*	Badeni	Cluj	Transylvania	Romania	440
JF512604		RVcoll.09-T246		*Pieridae*	*Leptidea*	*reali*	Flassan		Provence	France	890
JF512605		RVcoll.09-T247		*Pieridae*	*Leptidea*	*reali*	Flassan		Provence	France	890
JF512606		RVcoll.09-T249		*Pieridae*	*Leptidea*	*reali*	Flassan		Provence	France	890
JF512607		RVcoll.10-A411		*Pieridae*	*Leptidea*	*reali*	Digne les Bains		Alpes-de-Haute-Provence	France	710
JF512608		RVcoll.10-A412		*Pieridae*	*Leptidea*	*reali*	Digne les Bains		Alpes-de-Haute-Provence	France	710
JF512671		RVcoll.09-T244		*Pieridae*	*Leptidea*	*reali*	Flassan		Provence	France	890
JF512672		RVcoll.09-T245		*Pieridae*	*Leptidea*	*reali*	Flassan		Provence	France	890
JF512673		RVcoll.09-T250		*Pieridae*	*Leptidea*	*reali*	Flassan		Provence	France	890
JF512674		RVcoll.09-T251		*Pieridae*	*Leptidea*	*reali*	Flassan		Provence	France	890
JF512675		RVcoll.09-V231		*Pieridae*	*Leptidea*	*reali*	Flassan		Provence	France	400
JF512609		RVcoll.10-C258		*Pieridae*	*Leptidea*	*reali*	Sant'Anatolia di Narco		Perugia	Italy	350
JF512610		RVcoll.10-C259		*Pieridae*	*Leptidea*	*reali*	Sant'Anatolia di Narco		Perugia	Italy	350
JF512611		RVcoll.10-C263		*Pieridae*	*Leptidea*	*reali*	Sant'Anatolia di Narco		Perugia	Italy	350

GenBank accession number	BOLD Process ID	Sample ID	Used in the Iberian dataset	Family	Genus	Species	Exact Site	Region	State/Province	Country	Alt. (m)
JF512612		RVcoll.10-C268		*Pieridae*	*Leptidea*	*reali*	Cascia		Perugia	Italy	650
JF512613		RVcoll.10-C271		*Pieridae*	*Leptidea*	*reali*	Sant'Anatolia di Narco		Perugia	Italy	350
JF512614		RVcoll.10-C272		*Pieridae*	*Leptidea*	*reali*	Sant'Anatolia di Narco		Perugia	Italy	350
JF512615		RVcoll.10-C264		*Pieridae*	*Leptidea*	*reali*	Cascia		Perugia	Italy	650
JF512616		RVcoll.10-C269		*Pieridae*	*Leptidea*	*reali*	Cascia		Perugia	Italy	650
JF512658		RVcoll.07-E080		*Pieridae*	*Leptidea*	*reali*	Rocca Pia		L'Aquila	Italy	1215
JF512659		RVcoll.07-E081		*Pieridae*	*Leptidea*	*reali*	Roccaraso		L'Aquila	Italy	1180
JF512660		RVcoll.07-E082		*Pieridae*	*Leptidea*	*reali*	Roccaraso		L'Aquila	Italy	1180
JF512704		RVcoll.10-C270		*Pieridae*	*Leptidea*	*reali*	Sibillini Mountains		Umbria	Italy	
JF512712		RVcoll.07-E083		*Pieridae*	*Leptidea*	*reali*	Roccaraso		L'Aquila	Italy	1180
GU676645	EZSPN400-09	RVcoll.08-H468	x	*Pieridae*	*Leptidea*	*reali*	Hormiguera		Cantabria	Spain	970
GU675656	EZSPC468-09	RVcoll.08-J391	x	*Pieridae*	*Leptidea*	*reali*	Viladrau, Osona	Barcelona	Catalonia	Spain	715
JF512603		RVcoll.08-J396	x	*Pieridae*	*Leptidea*	*reali*	Viladrau	Barcelona	Catalonia	Spain	720
JF512617		RV-03-H535	x	*Pieridae*	*Leptidea*	*reali*	Saldes, Berguedà	Barcelona	Catalonia	Spain	1198
JF512656		RVcoll.07-C279	x	*Pieridae*	*Leptidea*	*reali*	Viladrau	Barcelona	Catalonia	Spain	715
JF512657		RVcoll.07-C613	x	*Pieridae*	*Leptidea*	*reali*	La Llavina	Barcelona	Catalonia	Spain	970
JF512679		09-V344	x	*Pieridae*	*Leptidea*	*reali*	Viladrau, Montseny, Osona	Barcelona	Catalonia	Spain	715
JF512680		RVcoll.09-V347	x	*Pieridae*	*Leptidea*	*reali*	Viladrau	Barcelona	Catalonia	Spain	720
KP870654	EZSPM870-12	Rvcoll.120711LF04	x	*Pieridae*	*Leptidea*	*reali*	Pista Pobla Tornesa, Benicassim	Castellon	Comunidad Valenciana	Spain	300
GU676782	EZSPN154-09	RVcoll.08-R059	x	*Pieridae*	*Leptidea*	*reali*	La Cambreta	Castellon	Comunidad Valenciana	Spain	1520
JF512669		08-L078	x	*Pieridae*	*Leptidea*	*reali*	Rambla de las Truchas, Vilafranca	Castellón	Comunidad Valenciana	Spain	1150
GU675654	EZSPC474-09	RVcoll.07-C636	x	*Pieridae*	*Leptidea*	*reali*	Meranges	Girona	Catalonia	Spain	1555
JF512676		09-V320	x	*Pieridae*	*Leptidea*	*reali*	Meranges, La Cerdanya	Girona	Catalonia	Spain	1744
JF512677		09-V321	x	*Pieridae*	*Leptidea*	*reali*	Meranges, La Cerdanya	Girona	Catalonia	Spain	1744
JF512678		RVcoll.09-V325	x	*Pieridae*	*Leptidea*	*reali*	Meranges	Girona	Catalonia	Spain	1744
JF512686		09-V815	x	*Pieridae*	*Leptidea*	*reali*	Vilallonga de Ter, Ripollès	Girona	Catalonia	Spain	1143.6
GU676968	EZSPN254-09	RVcoll.07-W154	x	*Pieridae*	*Leptidea*	*reali*	Vallibierna	Huesca	Aragon	Spain	1800
JF512681		09-V713	x	*Pieridae*	*Leptidea*	*reali*	Yésero	Huesca	Aragón	Spain	1160
JF512682		RVcoll.09-V714	x	*Pieridae*	*Leptidea*	*reali*	Yésero	Huesca	Aragón	Spain	1160
JF512683		09-V733	x	*Pieridae*	*Leptidea*	*reali*	Valle del Tena, Camino embalse Respomuso, Sallent de Gállego	Huesca	Aragón	Spain	1650
JF512684		RVcoll.09-V734	x	*Pieridae*	*Leptidea*	*reali*	Valle del Tena	Huesca	Aragón	Spain	1650
JF512685		RVcoll.09-V743	x	*Pieridae*	*Leptidea*	*reali*	Valle del Tena	Huesca	Aragón	Spain	1650
KP870722	EULEP093-14	09-T221	x	*Pieridae*	*Leptidea*	*reali*	Fonts del Cardener, La Coma, Solsonés	Lleida	Catalonia	Spain	1100
JF512661		RVcoll.07-W134	x	*Pieridae*	*Leptidea*	*reali*	Bassa d´Arres	Lleida		Spain	1568
JF512720		09-T203	x	*Pieridae*	*Leptidea*	*reali*	Fonts del Cardener, La Coma, Solsonés	Lleida	Catalonia	Spain	1100
KF049419		07-W133	x	*Pieridae*	*Leptidea*	*reali*	Bassa d'Arrès, Vall d'Aran	Lleida	Catalonia	Spain	1568
KP871136	EZSPM703-12	RVcoll.12-L116	x	*Pieridae*	*Leptidea*	*reali*	Navafria	Segovia	Castilla y Leon	Spain	1420
JF512670		08-L090	x	*Pieridae*	*Leptidea*	*reali*	Ruta de la Sima, El Vallecillo	Teruel	Aragón	Spain	1531
HM393183	GWORA2450-09	BC ZSM Lep 30662		*Pieridae*	*Leptidea*	*sinapis*	Zahmer Kaiser, Aschinger Alm		Tirol	Austria	1000
JF512693		RVcoll.10-B385		*Pieridae*	*Leptidea*	*sinapis*	Paril village		Hadjidimovo	Bulgaria	800
JF512721		RVcoll.LR08-D577		*Pieridae*	*Leptidea*	*sinapis*	Hvoyna		Chepelare	Bulgaria	800
JF512694		RVcoll.10-B453		*Pieridae*	*Leptidea*	*sinapis*	Žuljana, Ston		Peninsula Peljesac	Croatia	6
JF512695		RVcoll.10-B457		*Pieridae*	*Leptidea*	*sinapis*	Pećani		Lika-Senj	Croatia	600
JF512697		RVcoll.10-C245		*Pieridae*	*Leptidea*	*sinapis*	Príbram		Bohemia	CzechRepublic	
JF512698		RVcoll.10-C248		*Pieridae*	*Leptidea*	*sinapis*	Príbram		Bohemia	CzechRepublic	500
JF512692		RVcoll.10-A742		*Pieridae*	*Leptidea*	*sinapis*	Piusa		Polva	Estonia	60
JF512585		RVcoll.07-E249		*Pieridae*	*Leptidea*	*sinapis*	Col de la Chaudière		Drôme	France	1025
JF512586		RVcoll.07-E252		*Pieridae*	*Leptidea*	*sinapis*	NE. Bézaudun-sur-Bine		Drôme	France	735
JF512587		RVcoll.07-E253		*Pieridae*	*Leptidea*	*sinapis*	NE. Bézaudun-sur-Bine		Drôme	France	735
JF512598		RVcoll.07-E254		*Pieridae*	*Leptidea*	*sinapis*	NE. Bézaudun-sur-Bine		Drôme	France	735
JF512599		RVcoll.07-E255		*Pieridae*	*Leptidea*	*sinapis*	NE. Bézaudun-sur-Bine		Drôme	France	735

GenBank accession number	BOLD Process ID	Sample ID	Used in the Iberian dataset	Family	Genus	Species	Exact Site	Region	State/Province	Country	Alt. (m)
JF512600		RVcoll.07-E256		*Pieridae*	*Leptidea*	*sinapis*	NE. Bézaudun-sur-Bine		Drôme	France	735
GU688533	GWORO781-09	BC ZSM Lep 30473		*Pieridae*	*Leptidea*	*sinapis*	Lenggries Isarauen		Bavaria	Germany	
GU688515	GWORO852-09	BC ZSM Lep 30544		*Pieridae*	*Leptidea*	*sinapis*	Ruhpolding		Bavaria	Germany	
JF512708		RVcoll.LR08-D094		*Pieridae*	*Leptidea*	*sinapis*	Kalavryta, Mt. Chelmos		Peloponessos	Greece	810
JF512709		RVcoll.LR08-D203		*Pieridae*	*Leptidea*	*sinapis*	Eptalofos, Parnassus Mt.		Parnassus Mt.	Greece	1150
JF512687		RVcoll.09-X168		*Pieridae*	*Leptidea*	*sinapis*	Bunnanagat South		Clare	Ireland	35
JF512688		RVcoll.09-X171		*Pieridae*	*Leptidea*	*sinapis*	Gortnahoon		Galway	Ireland	30
JF512689		RVcoll.09-X173		*Pieridae*	*Leptidea*	*sinapis*	Oakfield		Galway	Ireland	25
JF512690		RVcoll.09-X174		*Pieridae*	*Leptidea*	*sinapis*	Rockhill		Galway	Ireland	25
JF512691		RVcoll.09-X180		*Pieridae*	*Leptidea*	*sinapis*	Dromore Nature Reserve		Clare	Ireland	25
JF512591		RVcoll.10-C278		*Pieridae*	*Leptidea*	*sinapis*	Sant'Anatolia di Narco		Perugia	Italy	350
JF512593		RVcoll.07-E140		*Pieridae*	*Leptidea*	*sinapis*	Borgo Val di Taro		Parma	Italy	620
JF512595		RVcoll.07-E174		*Pieridae*	*Leptidea*	*sinapis*	Ozein-Visyes		Aosta	Italy	1000
JF512596		RVcoll.07-E217		*Pieridae*	*Leptidea*	*sinapis*	Mompantero Vecchio		Torino	Italy	720
JF512597		RVcoll.07-E237		*Pieridae*	*Leptidea*	*sinapis*	Novalesa-Moncenisio		Torino	Italy	1155
JF512665		RVcoll.07-E215		*Pieridae*	*Leptidea*	*sinapis*	Mompantero Vecchio		Torino	Italy	1340
JF512666		RVcoll.07-E216		*Pieridae*	*Leptidea*	*sinapis*	Mompantero Vecchio		Torino	Italy	1340
JF512699		RVcoll.10-C251		*Pieridae*	*Leptidea*	*sinapis*	Monte di Malo		Veneto	Italy	350
JF512700		RVcoll.10-C252		*Pieridae*	*Leptidea*	*sinapis*	Monte di Malo		Veneto	Italy	350
JF512701		RVcoll.10-C253		*Pieridae*	*Leptidea*	*sinapis*	Monte di Malo		Veneto	Italy	350
JF512702		RVcoll.10-C254		*Pieridae*	*Leptidea*	*sinapis*	Monte di Malo		Veneto	Italy	350
JF512703		RVcoll.10-C255		*Pieridae*	*Leptidea*	*sinapis*	Monte di Malo		Veneto	Italy	350
JF512705		RVcoll.10-C276		*Pieridae*	*Leptidea*	*sinapis*	Norcia		Perugia	Italy	700
JF512706		RVcoll.10-C277		*Pieridae*	*Leptidea*	*sinapis*	Norcia		Perugia	Italy	700
HQ004613	EZRMN018-08	RVcoll.08-M201		*Pieridae*	*Leptidea*	*sinapis*	3 Km SW of Lunca Corbului	Arges	Muntenia	Romania	235
HQ004603	EZROM240-08	RV-07-D080		*Pieridae*	*Leptidea*	*sinapis*	Dealul Istrita, Breaza	Buzau	Muntenia	Romania	540
HQ004606	EZROM241-08	RV-07-D081		*Pieridae*	*Leptidea*	*sinapis*	Dealul Istrita, Breaza	Buzau	Muntenia	Romania	540
HQ004607	EZROM287-08	RV-07-E649		*Pieridae*	*Leptidea*	*sinapis*	Dealul Istrita, Breaza	Buzau	Muntenia	Romania	525
GU669641	EZROM1079-09	RVcoll.08-M555		*Pieridae*	*Leptidea*	*sinapis*	Valea Feregarilor - Poiana Musuroaie	Caras-Severin	Banat	Romania	600
HQ004602	EZROM242-08	RV-07-C990		*Pieridae*	*Leptidea*	*sinapis*	Baile Herculane (Pecinisca)	Caras-Severin	Banat	Romania	220
JF512582		RVcoll.07-E362		*Pieridae*	*Leptidea*	*sinapis*	Pecinișca	Caraș-Severin	Banat	Romania	270
HQ004604	EZROM238-08	RV-06-K556		*Pieridae*	*Leptidea*	*sinapis*	Badeni	Cluj	Transylvania	Romania	440
HQ004605	EZROM239-08	RV-06-K554		*Pieridae*	*Leptidea*	*sinapis*	Badeni	Cluj	Transylvania	Romania	440
JF512580		RVcoll.06-K559		*Pieridae*	*Leptidea*	*sinapis*	Bădeni	Cluj	Transylvania	Romania	450
JF512581		RVcoll.07-D962		*Pieridae*	*Leptidea*	*sinapis*	Valea Mare	Dâmbovița	Muntenia	Romania	225
HQ004614	EZRMN010-08	RVcoll.08-M528		*Pieridae*	*Leptidea*	*sinapis*	Tismana monastery	Gorj	Oltenia	Romania	230
JF512662		RVcoll.07-D940		*Pieridae*	*Leptidea*	*sinapis*	Ciupercenii de Olteț	Gorj	Oltenia	Romania	500
HQ004610	EZRMN019-08	RVcoll.08-M315		*Pieridae*	*Leptidea*	*sinapis*	Cheile Bicazului	Harghita	Transylvania	Romania	940
HQ004611	EZRMN020-08	RVcoll.08-M316		*Pieridae*	*Leptidea*	*sinapis*	Cheile Bicazului	Harghita	Transylvania	Romania	940
HQ004612	EZRMN021-08	RVcoll.08-M326		*Pieridae*	*Leptidea*	*sinapis*	Valea Belchia (Gheorgheni)	Harghita	Transylvania	Romania	1010
HQ004609	EZRMN022-08	RVcoll.08-M329		*Pieridae*	*Leptidea*	*sinapis*	1 Km E of Gheorgheni	Harghita	Transylvania	Romania	900
JF512584		RVcoll.07-D500		*Pieridae*	*Leptidea*	*sinapis*	Cheile Babei	Maramureș	Maramures	Romania	265
HQ004608	EZROM584-08	RV-07-C307		*Pieridae*	*Leptidea*	*sinapis*	Radauti	Suceava	Moldavia	Romania	450
JF512592		RVcoll.07-C210		*Pieridae*	*Leptidea*	*sinapis*	Schitul Pahomie	Vâlcea	Oltenia	Romania	800
JF512710		RVcoll.LR08-D679		*Pieridae*	*Leptidea*	*sinapis*	Jagnjenica		Radeče	Slovenia	280
JF512711		RVcoll.LR08-D756		*Pieridae*	*Leptidea*	*sinapis*	Račice		Ilistra Bistrica	Slovenia	630
GU675693	EZSPM140-09	RVcoll.08-P652	x	*Pieridae*	*Leptidea*	*sinapis*	Valle (Valle-Zurea), Lena		Asturias	Spain	662
KP870821	EZSPM814-12	RVcoll.130711PX23	x	*Pieridae*	*Leptidea*	*sinapis*	Rio Muniellos, Cangas del Narcea		Asturias	Spain	594
KP871038	EZSPM815-12	RVcoll.130711PX77	x	*Pieridae*	*Leptidea*	*sinapis*	Casa de la Luz, Degana		Asturias	Spain	800
GU675857	EZSPM236-09	RVcoll.08-R436	x	*Pieridae*	*Leptidea*	*sinapis*	Ames, Novais	A Coruna	Galicia	Spain	160
KP870734	EZSPM705-12	RVcoll.12-L215	x	*Pieridae*	*Leptidea*	*sinapis*	Penacerrada, Tolono	Alava	Basque Country	Spain	1150
KP871088	EZSPM701-12	RVcoll.12-L113	x	*Pieridae*	*Leptidea*	*sinapis*	Santa Lucia de la Sierra	Avila	Castilla y Leon	Spain	1250
GU676686	EZSPN347-09	RVcoll.08-H312	x	*Pieridae*	*Leptidea*	*sinapis*	Casillas, Casillas	Avila	Castilla y Leon	Spain	
GU669777	EZSPC469-09	RVcoll.08-J393	x	*Pieridae*	*Leptidea*	*sinapis*	Viladrau, Osona	Barcelona	Catalonia	Spain	715
KP871000	EZSPC470-09	RVcoll.07-F504	x	*Pieridae*	*Leptidea*	*sinapis*	Viladrau	Barcelona	Catalonia	Spain	730
KP870893	EZSPC471-09	RVcoll.06-G558	x	*Pieridae*	*Leptidea*	*sinapis*	El Sola, El Brull, Osona	Barcelona	Catalonia	Spain	800

GenBank accession number	BOLD Process ID	Sample ID	Used in the Iberian dataset	Family	Genus	Species	Exact Site	Region	State/Province	Country	Alt. (m)
GU669774	EZSPC473-09	RVcoll.06-V761	x	*Pieridae*	*Leptidea*	*sinapis*	Torrent del Remogent, Canoves, Valles Oriental	Barcelona	Catalonia	Spain	480
GU669775	EZSPC475-09	RVcoll.08-J352	x	*Pieridae*	*Leptidea*	*sinapis*	Vallgrassa, Parc Natural del Garraf	Barcelona	Catalonia	Spain	285
JF512589		RVcoll.08-H275	x	*Pieridae*	*Leptidea*	*sinapis*	Viladrau	Barcelona	Catalonia	Spain	715
JF512590		RVcoll.09-V326	x	*Pieridae*	*Leptidea*	*sinapis*	Cànoves	Barcelona	Catalonia	Spain	650
JF512594		RVcoll.08-H281	x	*Pieridae*	*Leptidea*	*sinapis*	Viladrau	Barcelona	Catalonia	Spain	715
JF512623		RVcoll.07-C470	x	*Pieridae*	*Leptidea*	*sinapis*	Llinars del Vallès	Barcelona	Catalonia	Spain	200
JF512663		RVcoll.07-C466	x	*Pieridae*	*Leptidea*	*sinapis*	Cànoves	Barcelona	Catalonia	Spain	650
JF512667		RVcoll.08-H278	x	*Pieridae*	*Leptidea*	*sinapis*	Viladrau	Barcelona	Catalonia	Spain	715
KP870412	EZSPM700-12	RVcoll.12-L108	x	*Pieridae*	*Leptidea*	*sinapis*	Huerta del Rey	Burgos	Castilla y Leon	Spain	1150
KP870515	EZSPM1010-12	Rvcoll.12-L056	x	*Pieridae*	*Leptidea*	*sinapis*	Merendero de los Horcones, Navatrasierra, Caceres	Caceres	Extremadura	Spain	664
GU676879	EZSPN472-09	RVcoll.08-H902	x	*Pieridae*	*Leptidea*	*sinapis*	Hervas, Carretera Hervas-La Garganta	Caceres	Extremadura	Spain	1007
JF512668		RVcoll.08-H903	x	*Pieridae*	*Leptidea*	*sinapis*	La Garganta	Cáceres	Extremadura	Spain	1010
GU676793	EZSPN146-09	RVcoll.08-R045	x	*Pieridae*	*Leptidea*	*sinapis*	Barranco Truchelles	Castellon	Comunidad Valenciana	Spain	279
GU676785	EZSPN147-09	RVcoll.08-R046	x	*Pieridae*	*Leptidea*	*sinapis*	Barranco Truchelles	Castellon	Comunidad Valenciana	Spain	279
GU676340	EZSPN814-09	RVcoll.08-L617	x	*Pieridae*	*Leptidea*	*sinapis*	Huelamo, Rio Jucar	Cuenca	Castilla-La Mancha	Spain	1237
GU676132	EZSPM401-09	Rvcoll.09-V802	x	*Pieridae*	*Leptidea*	*sinapis*	Estanys de la Jonquera, La Jonquera, Alt Emporda	Girona	Catalonia	Spain	
GU676633	EZSPN412-09	RVcoll.08-H527	x	*Pieridae*	*Leptidea*	*sinapis*	Gualda, La Alcarria	Guadalajara	Castilla-La Mancha	Spain	730
KC866081		09-X553	x	*Pieridae*	*Leptidea*	*sinapis*	2 km SW Uribarri	Guipúzcoa	País Vasco	Spain	500
GU675913	EZSPM323-09	RVcoll.08-J897	x	*Pieridae*	*Leptidea*	*sinapis*	Vega de Espinareda	Leon	Castilla y Leon	Spain	
KP871053	EZSPM704-12	RVcoll.12-L123	x	*Pieridae*	*Leptidea*	*sinapis*	Villaescusa de las Torres, Las Tuerces	Palencia	Castilla y Leon	Spain	960
GU675855	EZSPM237-09	RVcoll.08-R438	x	*Pieridae*	*Leptidea*	*sinapis*	Tui, Rio Penedo	Pontevedra	Galicia	Spain	30
KP870351	EZSPM702-12	RVcoll.12-L115	x	*Pieridae*	*Leptidea*	*sinapis*	Navafria	Segovia	Castilla y Leon	Spain	1350
GU676739	EZSPN294-09	RVcoll.08-J660	x	*Pieridae*	*Leptidea*	*sinapis*	Barranco del Toro	Teruel	Aragon	Spain	1443
GU675910	EZSPM328-09	RVcoll.08-R034	x	*Pieridae*	*Leptidea*	*sinapis*	Casas de Barchell, Chelva	Valencia	Comunidad Valenciana	Spain	
GU676665	EZSPN371-09	RVcoll.08-H396	x	*Pieridae*	*Leptidea*	*sinapis*	Serra, Serra	Valencia	Comunidad Valenciana	Spain	
JF512707		RVcoll.160410QT77		*Pieridae*	*Leptidea*	*sinapis*	Gampel Bratsch, Martigny		Valais	Switzerland	630
JF512696		RVcoll.10-C197		*Pieridae*	*Leptidea*	*sinapis*	Novaya Ilienko station, Derkulriver		Luhansk	Ukraine	40
GU676554	EZSPN587-09	RVcoll.08-J149	x	*Lycaenidae*	*Leptotes*	*pirithous*	Ponte Pedrinha, Beca, Boticas		Tras-os-Montes	Portugal	730
HQ004615	EZRMN023-08	RVcoll.08-M581		*Lycaenidae*	*Leptotes*	*pirithous*	Canaraua Fetei (Baneasa)	Constanta	Dobrogea	Romania	15
HQ004616	EZROM243-08	RV-07-E448		*Lycaenidae*	*Leptotes*	*pirithous*	2 km E of Smardan	Tulcea	Dobrogea	Romania	5
KP870684	EZSPM882-12	Rvcoll.12-M600	x	*Lycaenidae*	*Leptotes*	*pirithous*	Medellin	Badajoz	Extremadura	Spain	240
GU675655	EZSPC476-09	RVcoll.08-M715	x	*Lycaenidae*	*Leptotes*	*pirithous*	Delta de Llobregat	Barcelona	Catalonia	Spain	3
GU675650	EZSPC477-09	RVcoll.08-R158	x	*Lycaenidae*	*Leptotes*	*pirithous*	Montvi de Baix, Moianes	Barcelona	Catalonia	Spain	800
GU676332	EZSPN818-09	RVcoll.08-L626	x	*Lycaenidae*	*Leptotes*	*pirithous*	Algeciras, Arroyo del Prior	Cadiz	Andalusia	Spain	50
GU677005	EZSPN214-09	RVcoll.07-F072	x	*Lycaenidae*	*Leptotes*	*pirithous*	10 km S. of Morella	Castellon	Comunidad Valenciana	Spain	900
GU675651	EZSPC478-09	RVcoll.07-C072	x	*Lycaenidae*	*Leptotes*	*pirithous*	Puig Neulos, Alt Emporda	Girona	Catalonia	Spain	1110
GU675652	EZSPC479-09	RVcoll.08-R164	x	*Lycaenidae*	*Leptotes*	*pirithous*	Llanars, Ripolles	Girona	Catalonia	Spain	1119
GU676835	EZSPN093-09	RVcoll.06-A022	x	*Lycaenidae*	*Leptotes*	*pirithous*	Mecina Bombaron, Alpujarras	Granada	Andalusia	Spain	1150
GU675874	EZSPM364-09	RVcoll.08-J825	x	*Lycaenidae*	*Leptotes*	*pirithous*	Santa Colomba de la Vega	Leon	Castilla y Leon	Spain	
GU675970	EZSPM056-09	RVcoll.08-P281	x	*Lycaenidae*	*Leptotes*	*pirithous*	S`Albufera	Mallorca	Balearic Islands	Spain	4
GU675967	EZSPM061-09	RVcoll.08-P287	x	*Lycaenidae*	*Leptotes*	*pirithous*	Genova	Mallorca	Balearic Islands	Spain	123
GU675799	EZSPM212-09	RVcoll.08-R333	x	*Lycaenidae*	*Leptotes*	*pirithous*	Palma de Mallorca	Mallorca	Balearic Islands	Spain	88
GU676715	EZSPN322-09	RVcoll.08-H183	x	*Lycaenidae*	*Leptotes*	*pirithous*	Llimpa, Mao	Menorca	Balearic Islands	Spain	
GU676307	EZSPN846-09	RVcoll.08-L684	x	*Lycaenidae*	*Leptotes*	*pirithous*	Candelario, Sierra de Candelario	Salamanca	Castilla y Leon	Spain	1300
GU676285	EZSPN872-09	RVcoll.08-L762	x	*Lycaenidae*	*Leptotes*	*pirithous*	Valsain	Segovia	Castilla y Leon	Spain	1437
GU675653	EZSPC480-09	RVcoll.08-M698	x	*Lycaenidae*	*Leptotes*	*pirithous*	Farena, Alt Camp	Tarragona	Catalonia	Spain	580
HQ004618	EZRMN024-08	RVcoll.08-M259		*Nymphalidae*	*Libythea*	*celtis*	road E70, (Gura Vaii) Portile de Fier	Mehedinti	Oltenia	Romania	50
HQ004620	EZRMN025-08	RVcoll.08-M262		*Nymphalidae*	*Libythea*	*celtis*	road E70, (Gura Vaii) Portile de Fier	Mehedinti	Oltenia	Romania	50
HQ004617	EZRMN026-08	RVcoll.08-M263		*Nymphalidae*	*Libythea*	*celtis*	road E70, (Gura Vaii) Portile de Fier	Mehedinti	Oltenia	Romania	50

GenBank accession number	BOLD Process ID	Sample ID	Used in the Iberian dataset	Family	Genus	Species	Exact Site	Region	State/Province	Country	Alt. (m)
HQ004619	EZRMN027-08	RVcoll.08-M264		*Nymphalidae*	*Libythea*	*celtis*	road E70, (Gura Vaii) Portile de Fier	Mehedinti	Oltenia	Romania	50
HQ004621	EZRMN028-08	RVcoll.08-M278		*Nymphalidae*	*Libythea*	*celtis*	road E70, (Gura Vaii) Portile de Fier	Mehedinti	Oltenia	Romania	50
HM901219	EZSPC1015-10	RVcoll.08-H606	x	*Nymphalidae*	*Libythea*	*celtis*	Candeleda, La Lagunilla	Avila	Castilla y Leon	Spain	380
GU676612	EZSPN428-09	RVcoll.08-H603	x	*Nymphalidae*	*Libythea*	*celtis*	Candeleda, La Lagunilla	Avila	Castilla y Leon	Spain	380
GU669770	EZSPC481-09	RVcoll.08-J378	x	*Nymphalidae*	*Libythea*	*celtis*	Turo del Fumet, La Floresta, Valles Occidental	Barcelona	Catalonia	Spain	315
GU669771	EZSPC482-09	RVcoll.08-J379	x	*Nymphalidae*	*Libythea*	*celtis*	Turo del Fumet, La Floresta, Valles Occidental	Barcelona	Catalonia	Spain	315
GU669772	EZSPC483-09	RVcoll.08-L446	x	*Nymphalidae*	*Libythea*	*celtis*	La Llavina, Montseny, Valles Oriental	Barcelona	Catalonia	Spain	500
GU669644	EZSPC639-09	RVcoll.08-L374	x	*Nymphalidae*	*Libythea*	*celtis*	Montseny d`Amunt, Can Codina	Barcelona	Catalonia	Spain	885
GU669773	EZSPC484-09	RVcoll.08-J349	x	*Nymphalidae*	*Libythea*	*celtis*	Cantallops, Alt Emporda	Girona	Catalonia	Spain	225
KP870434	EZSPM617-12	RVcoll.11-D839	x	*Nymphalidae*	*Libythea*	*celtis*	Canada Real de la Zubia, Monachil	Granada	Andalusia	Spain	1106
GU676953	EZSPN552-09	RVcoll.08-J059	x	*Nymphalidae*	*Libythea*	*celtis*	Yatova, Yatova	Valencia	Comunidad Valenciana	Spain	
HQ957503	GWORO809-09	BC ZSM Lep 30501		*Nymphalidae*	*Limenitis*	*camilla*	Fischerhaeuser Ismaning / MUC		Bavaria	Germany	482
HQ957216	GWORA2516-09	BC ZSM Lep 30728		*Nymphalidae*	*Limenitis*	*camilla*	Obernzell	Niederbayern	Bavaria	Germany	
GU707095	FBLMU442-09	BC ZSM Lep 27092		*Nymphalidae*	*Limenitis*	*camilla*	Schoengeising	Oberbayern	Bavaria	Germany	545
HQ957217	GWORA2517-09	BC ZSM Lep 30729		*Nymphalidae*	*Limenitis*	*camilla*	Oberaudorf, Muehlau	Oberbayern	Bavaria	Germany	500
HQ004626	EZRMN032-08	RVcoll.08-M540		*Nymphalidae*	*Limenitis*	*camilla*	Valea Feregarilor - Poiana Musuroaie	Caras-Severin	Banat	Romania	350
KP871099	EULEP090-14	08-P977		*Nymphalidae*	*Limenitis*	*camilla*	Cluj-Napoca, Dealul Feleacului	Cluj	Transylvania	Romania	
HQ004627	EZRMN029-08	RVcoll.06-M856		*Nymphalidae*	*Limenitis*	*camilla*	Cluj-Napoca, dealul Feleacului, Gheorgheni forest	Cluj	Transylvania	Romania	600
HQ004624	EZRMN030-08	RVcoll.08-M504		*Nymphalidae*	*Limenitis*	*camilla*	Badeni	Cluj	Transylvania	Romania	490
HQ004625	EZRMN031-08	RVcoll.08-M505		*Nymphalidae*	*Limenitis*	*camilla*	Badeni	Cluj	Transylvania	Romania	490
HQ004622	EZROM244-08	RV-06-M853		*Nymphalidae*	*Limenitis*	*camilla*	Cluj-Napoca, dealul Feleacului, Gheorgheni forest	Cluj	Transylvania	Romania	600
HQ004623	EZROM245-08	RV-06-M875		*Nymphalidae*	*Limenitis*	*camilla*	Cluj-Napoca, dealul Feleacului, Gheorgheni forest	Cluj	Transylvania	Romania	600
KP870376	EULEP146-14	10-C432		*Nymphalidae*	*Limenitis*	*camilla*	Cheile Bicazului	Neamt	Moldavia	Romania	
FJ663725	LOWA856-06	2005-LOWA-856		*Nymphalidae*	*Limenitis*	*camilla*			Belgorod	Russia	150
FJ663724	LOWA857-06	2005-LOWA-857		*Nymphalidae*	*Limenitis*	*camilla*			Belgorod	Russia	150
KP870495	EZROM730-08	RV-07-F310	x	*Nymphalidae*	*Limenitis*	*camilla*	Viladrau	Barcelona	Catalonia	Spain	720
HM901620	EZSPC765-10	RVcoll.08-L534	x	*Nymphalidae*	*Limenitis*	*camilla*	Torrent del Reguissol, Sant Esteve de Palautordera, Valles Oriental	Barcelona	Catalonia	Spain	300
HM901749	EZSPC911-10	RVcoll.09-V808	x	*Nymphalidae*	*Limenitis*	*camilla*	Vilallonga de Ter, Ripolles	Girona	Catalonia	Spain	1143.6
HM901751	EZSPC913-10	RVcoll.09-V822	x	*Nymphalidae*	*Limenitis*	*camilla*	Vilallonga de Ter, Ripolles	Girona	Catalonia	Spain	1143.6
GU676754	EZSPN278-09	RVcoll.07-W210	x	*Nymphalidae*	*Limenitis*	*camilla*	Bisaurri, Bisaurri	Huesca	Aragon	Spain	1350
GU669768	EZSPC486-09	RVcoll.08-M917	x	*Nymphalidae*	*Limenitis*	*camilla*	Arros, Vall d`Aran	Lleida	Catalonia	Spain	906
HM901701	EZSPC860-10	RVcoll.09-T052	x	*Nymphalidae*	*Limenitis*	*camilla*	Garos, Vall d`Aran	Lleida	Catalonia	Spain	1100
JN280062	EZSPN1059-11	Rvcoll.08-P348.1	x	*Nymphalidae*	*Limenitis*	*camilla*	Perves, Pallars Jussa	Lleida	Catalonia	Spain	1071.7
KP870985	EZSPM777-12	RVcoll.050111LP98	x	*Nymphalidae*	*Limenitis*	*camilla*	Ruta de los Molinos, Viveiro	Lugo	Galicia	Spain	32
GU675807	EZSPM265-09	RVcoll.08-R480	x	*Nymphalidae*	*Limenitis*	*camilla*	Tui, Rio Penedo	Pontevedra	Galicia	Spain	40
GU707193	FBLMU110-09	BC ZSM Lep 25620		*Nymphalidae*	*Limenitis*	*populi*	Kirchberg/Lkr. Regen, Amthofwald	Niederbayern	Bavaria	Germany	730
HQ565467	GWOSA734-10	BC ZSM Lep 35745		*Nymphalidae*	*Limenitis*	*populi*	Ruselgebiet, Dattinger Haenge	Niederbayern	Bavaria	Germany	700
HQ004629	EZROM247-08	RV-07-C965		*Nymphalidae*	*Limenitis*	*populi*	west of Obarsia Closani	Caras-Severin	Banat	Romania	380
HQ004628	EZROM246-08	RV-06-N008		*Nymphalidae*	*Limenitis*	*populi*	Gheorgheni, Valea Belchia	Harghita	Transylvania	Romania	1010
HQ004630	EZRMN034-08	RVcoll.08-M554		*Nymphalidae*	*Limenitis*	*reducta*	Valea Feregarilor - Poiana Musuroaie	Caras-Severin	Banat	Romania	350
HM901525	EZSPC1391-10	RVcoll.09-X582	x	*Nymphalidae*	*Limenitis*	*reducta*	2 km E Navascues		Navarra	Spain	680
GU676866	EZSPC489-09	RVcoll.08-H272	x	*Nymphalidae*	*Limenitis*	*reducta*	Vallforners, Canoves	Barcelona	Catalonia	Spain	610
GU676864	EZSPC490-09	RVcoll.08-R316	x	*Nymphalidae*	*Limenitis*	*reducta*	Els Refugis del Montseny, St. Pere de Vilamajor, Valles Oriental	Barcelona	Catalonia	Spain	630
GU676865	EZSPC491-09	RVcoll.08-P068	x	*Nymphalidae*	*Limenitis*	*reducta*	Torrent de Ridolaina, Cerdanya	Girona	Catalonia	Spain	1060
GU676171	EZSPN995-09	RVcoll.08-M066	x	*Nymphalidae*	*Limenitis*	*reducta*	San Andres del Rey, La Alcarria	Guadalajara	Castilla-La Mancha	Spain	1018
GU676303	EZSPN850-09	RVcoll.08-L695	x	*Nymphalidae*	*Limenitis*	*reducta*	Candelario	Salamanca	Castilla y Leon	Spain	1300
GU676230	EZSPN924-09	RVcoll.08-L871	x	*Nymphalidae*	*Limenitis*	*reducta*	Abejar	Soria	Castilla y Leon	Spain	1097
HM901393	EZSPC1193-10	RVcoll.09-V348	x	*Nymphalidae*	*Limenitis*	*reducta*	Barranc de Tillar, Riudabella, Poblet	Tarragona	Catalonia	Spain	
HM901394	EZSPC1194-10	RVcoll.09-V350	x	*Nymphalidae*	*Limenitis*	*reducta*	2 Km S of Prades	Tarragona	Catalonia	Spain	900

GenBank accession number	BOLD Process ID	Sample ID	Used in the Iberian dataset	Family	Genus	Species	Exact Site	Region	State/Province	Country	Alt. (m)
HM901516	EZSPC1382-10	RVcoll.09-X518	x	*Nymphalidae*	*Limenitis*	*reducta*	2 km NW El Vallecillo	Teruel	Aragon	Spain	1450
GU676515	EZSPN633-09	RVcoll.08-J734	x	*Nymphalidae*	*Limenitis*	*reducta*	Piscifactoria de La Tosquilla, Titaguas	Valencia	Comunidad Valenciana	Spain	586
JF415710	GWORK515-09	BC ZSM Lep 21845		*Nymphalidae*	*Lopinga*	*achine*	Diessen	Oberbayern	Bavaria	Germany	600
HM910005	GWORT459-10	BC ZSM Lep 32145		*Nymphalidae*	*Lopinga*	*achine*	Vorderriss	Oberbayern	Bavaria	Germany	760
HQ004632	EZRMN035-08	RVcoll.08-M667		*Nymphalidae*	*Lopinga*	*achine*	Gheorgheni forest, Cluj-Napoca	Cluj	Transylvania	Romania	600
HQ004631	EZRMN036-08	RVcoll.08-M671		*Nymphalidae*	*Lopinga*	*achine*	Paraul Micus, Salicea	Cluj	Transylvania	Romania	700
HQ004633	EZROM248-08	RV-06-M908		*Nymphalidae*	*Lopinga*	*achine*	Stana	Salaj	Transylvania	Romania	500
HQ004634	EZROM249-08	RV-06-M909		*Nymphalidae*	*Lopinga*	*achine*	Stana	Salaj	Transylvania	Romania	500
FJ663733	LOWA858-06	2005-LOWA-858		*Nymphalidae*	*Lopinga*	*achine*			Belgorod	Russia	150
KP870281	EULEP250-14	12-Q444	x	*Nymphalidae*	*Lopinga*	*achine*	Puerto Peña Orduña	Álava	País Vasco	Spain	650
KP870912	EZSPN822-09	RVcoll.08-L631	x	*Nymphalidae*	*Lopinga*	*achine*	Posada de Valden, Las Vegas	Leon	Castilla y Leon	Spain	550
KP871080	EZSPN823-09	RVcoll.08-L632	x	*Nymphalidae*	*Lopinga*	*achine*	Posada de Valden, Las Vegas	Leon	Castilla y Leon	Spain	550
KP871153	EULEP196-14	12-L006	x	*Nymphalidae*	*Lopinga*	*achine*	Angón, Amieva		Asturias	Spain	
KP870458	EULEP197-14	12-L007	x	*Nymphalidae*	*Lopinga*	*achine*	Angón, Amieva		Asturias	Spain	
KP871174	EULEP209-14	12-L027	x	*Nymphalidae*	*Lopinga*	*achine*	Foz de Andamios, Ponga		Asturias	Spain	609
HQ004641	EZRMN038-08	RVcoll.07-D358		*Lycaenidae*	*Lycaena*	*alciphron*	Racos	Brasov	Transylvania	Romania	460
HQ004640	EZRMN037-08	RVcoll.07-C922		*Lycaenidae*	*Lycaena*	*alciphron*	Teregova	Caras-Severin	Banat	Romania	385
HQ004636	EZROM252-08	RV-07-C958		*Lycaenidae*	*Lycaena*	*alciphron*	Baile Herculane (Pecinisca)	Caras-Severin	Banat	Romania	220
HQ004638	EZROM250-08	RV-06-M840		*Lycaenidae*	*Lycaena*	*alciphron*	Cluj-Napoca valea paraului Micus	Cluj	Transylvania	Romania	700
HQ004639	EZRMN040-08	RVcoll.08-M531		*Lycaenidae*	*Lycaena*	*alciphron*	Tismana monastery	Gorj	Oltenia	Romania	230
HQ004635	EZROM634-08	RV-07-D536		*Lycaenidae*	*Lycaena*	*alciphron*	Rohia	Maramures	Maramures	Romania	360
HQ004642	EZRMN039-08	RVcoll.08-M271		*Lycaenidae*	*Lycaena*	*alciphron*	road E70, 6 Km W of Turnu-Severin	Mehedinti	Oltenia	Romania	45
HQ004637	EZROM251-08	RV-07-D918		*Lycaenidae*	*Lycaena*	*alciphron*	valea Otasaului, Barzesti, Buila-Vanturarita	Valcea	Oltenia	Romania	515
HM901285	EZSPC1084-10	RVcoll.08-L738	x	*Lycaenidae*	*Lycaena*	*alciphron*	Puerto de la Morcuera		Comunidad de Madrid	Spain	1438
GU676300	EZSPN856-09	RVcoll.08-L712	x	*Lycaenidae*	*Lycaena*	*alciphron*	Soto del Real		Comunidad de Madrid	Spain	1200
HM901279	EZSPC1078-10	RVcoll.08-L638	x	*Lycaenidae*	*Lycaena*	*alciphron*	Hoyocasero, Venta Rasquilla	Avila	Castilla y Leon	Spain	1224
GU669766	EZSPC492-09	RVcoll.08-R147	x	*Lycaenidae*	*Lycaena*	*alciphron*	Turo de L`Home, Montseny	Barcelona	Catalonia	Spain	1643
GU669767	EZSPC493-09	RVcoll.08-R191	x	*Lycaenidae*	*Lycaena*	*alciphron*	Turo de L`Home, Montseny	Barcelona	Catalonia	Spain	1643
KP870592	EZROM731-08	RV-07-C073	x	*Lycaenidae*	*Lycaena*	*alciphron*	Puig Neulos	Girona	Catalonia	Spain	1110
GU669763	EZSPC495-09	RVcoll.08-P031	x	*Lycaenidae*	*Lycaena*	*alciphron*	La Valira (near Urus), Cerdanya	Girona	Catalonia	Spain	1300
HM901491	EZSPC1355-10	RVcoll.09-V901	x	*Lycaenidae*	*Lycaena*	*alciphron*	Sierra de la Sagra	Granada	Andalusia	Spain	1745
GU676483	EZSPN664-09	RVcoll.08-J967	x	*Lycaenidae*	*Lycaena*	*alciphron*	Acceso Veleta, Monachil	Granada	Andalusia	Spain	2737
GU676418	EZSPN731-09	RVcoll.08-L139	x	*Lycaenidae*	*Lycaena*	*alciphron*	Laguna Seca, Hueneja	Granada	Andalusia	Spain	2276
GU676146	EZSPN1020-09	RVcoll.08-M952	x	*Lycaenidae*	*Lycaena*	*alciphron*	Forcat	Huesca	Aragon	Spain	1039
GU669762	EZSPC494-09	RVcoll.08-R190	x	*Lycaenidae*	*Lycaena*	*alciphron*	Salt de Pitx, Vall d`Aran	Lleida	Catalonia	Spain	1570
GU676093	EZSPM430-09	Rvcoll.09-X503	x	*Lycaenidae*	*Lycaena*	*alciphron*	2 km NW El Vallecillo	Teruel	Aragon	Spain	1450
GU676944	EZSPN542-09	RVcoll.08-J037	x	*Lycaenidae*	*Lycaena*	*alciphron*	Cortes de Pallas (Sierra Martes), Cortes de Pallas	Valencia	Comunidad Valenciana	Spain	
HM901625	EZSPC770-10	RVcoll.08-L718	x	*Lycaenidae*	*Lycaena*	*bleusei*	Soto del Real		Comunidad de Madrid	Spain	1230
GU676301	EZSPN857-09	RVcoll.08-L717	x	*Lycaenidae*	*Lycaena*	*bleusei*	Soto del Real		Comunidad de Madrid	Spain	1230
GU676294	EZSPN858-09	RVcoll.08-L719	x	*Lycaenidae*	*Lycaena*	*bleusei*	Soto del Real		Comunidad de Madrid	Spain	1230
HM901587	EZSPC732-10	RVcoll.08-H900	x	*Lycaenidae*	*Lycaena*	*bleusei*	Hoyocasero, Pinar de Hoyocasero	Avila	Castilla y Leon	Spain	1254
GU676682	EZSPN351-09	RVcoll.08-H322	x	*Lycaenidae*	*Lycaena*	*bleusei*	Cebreros, Cebreros	Avila	Castilla y Leon	Spain	
GU676318	EZSPN830-09	RVcoll.08-L646	x	*Lycaenidae*	*Lycaena*	*bleusei*	Hoyocasero, Pinar de Hoyocasero	Avila	Castilla y Leon	Spain	1254
HM901590	EZSPC735-10	RVcoll.08-H937	x	*Lycaenidae*	*Lycaena*	*bleusei*	Candelario, Sierra de Candelario	Salamanca	Castilla y Leon	Spain	1300
HM901653	EZSPC803-10	RVcoll.08-P432	x	*Lycaenidae*	*Lycaena*	*bleusei*	Candelario	Salamanca	Castilla y Leon	Spain	1126
GU675688	EZSPM101-09	RVcoll.08-P433	x	*Lycaenidae*	*Lycaena*	*bleusei*	Candelario	Salamanca	Castilla y Leon	Spain	1126
GU676898	EZSPN495-09	RVcoll.08-H938	x	*Lycaenidae*	*Lycaena*	*bleusei*	Candelario, Sierra de Candelario	Salamanca	Castilla y Leon	Spain	1300
HM901758	EZSPC920-10	RVcoll.09-V898	x	*Lycaenidae*	*Lycaena*	*bleusei*	Los Angeles de San Rafael	Segovia	Castilla y Leon	Spain	1215
HQ004644	EZRMN044-08	RVcoll.08-M210		*Lycaenidae*	*Lycaena*	*dispar*	3 Km SW of Lunca Corbului	Arges	Muntenia	Romania	235
HQ004643	EZRMN045-08	RVcoll.08-M372		*Lycaenidae*	*Lycaena*	*dispar*	Buciumeni forest (Radacinesti)	Bacau	Moldavia	Romania	180

GenBank accession number	BOLD Process ID	Sample ID	Used in the Iberian dataset	Family	Genus	Species	Exact Site	Region	State/Province	Country	Alt. (m)
HQ004646	EZRMN042-08	RVcoll.07-D269		*Lycaenidae*	*Lycaena*	*dispar*	Dumbrava Vadului, Vad	Brasov	Transylvania	Romania	495
HQ004649	EZROM254-08	RV-07-D099		*Lycaenidae*	*Lycaena*	*dispar*	Dealul Istrita, Breaza	Buzau	Muntenia	Romania	540
HQ004651	EZROM635-08	RV-07-C935		*Lycaenidae*	*Lycaena*	*dispar*	Teregova	Caras-Severin	Banat	Romania	385
HQ004648	EZROM253-08	RV-06-M827		*Lycaenidae*	*Lycaena*	*dispar*	Cluj-Napoca, dealul Feleacului, Gheorgheni forest	Cluj	Transylvania	Romania	600
HQ004647	EZRMN041-08	RVcoll.07-D006		*Lycaenidae*	*Lycaena*	*dispar*	Canaraua Fetei (Baneasa)	Constanta	Dobrogea	Romania	15
HQ004650	EZROM255-08	RV-07-D194		*Lycaenidae*	*Lycaena*	*dispar*	Canaraua Fetei (Baneasa)	Constanta	Dobrogea	Romania	15
HQ004645	EZRMN043-08	RVcoll.07-D555		*Lycaenidae*	*Lycaena*	*dispar*	Rohia	Maramures	Maramures	Romania	360
JF847995	EZSPN052-09	RVcoll.09-V309	x	*Lycaenidae*	*Lycaena*	*helle*	Porte Puymorens	Languedoc-Roussillon		France	1825
JF847996	EZSPN053-09	RVcoll.09-V310	x	*Lycaenidae*	*Lycaena*	*helle*	Porte Puymorens	Languedoc-Roussillon		France	1825
JF847997	EZSPN054-09	RVcoll.09-V311	x	*Lycaenidae*	*Lycaena*	*helle*	Porte Puymorens	Languedoc-Roussillon		France	1825
JF847998	EZSPN055-09	RVcoll.09-V312	x	*Lycaenidae*	*Lycaena*	*helle*	Porte Puymorens	Languedoc-Roussillon		France	1825
JF847999	EZSPN056-09	RVcoll.09-V313	x	*Lycaenidae*	*Lycaena*	*helle*	Porte Puymorens	Languedoc-Roussillon		France	1825
GU677023	EZSPN193-09	RVcoll.07-C487	x	*Lycaenidae*	*Lycaena*	*helle*	Col de Puymorens	Languedoc-Roussillon		France	1825
GU688466	GWORO761-09	BC ZSM Lep 30453		*Lycaenidae*	*Lycaena*	*helle*	Geretsried		Bavaria	Germany	
GU688463	GWORO790-09	BC ZSM Lep 30482		*Lycaenidae*	*Lycaena*	*helle*	Zellwies TOEL		Bavaria	Germany	
HQ004656	EZRMN046-08	RVcoll.07-D262		*Lycaenidae*	*Lycaena*	*helle*	Dumbrava Vadului, Vad	Brasov	Transylvania	Romania	495
HQ004655	EZRMN047-08	RVcoll.07-D299		*Lycaenidae*	*Lycaena*	*helle*	Dumbrava Vadului, Vad	Brasov	Transylvania	Romania	495
HQ004657	EZRMN048-08	RVcoll.07-D305		*Lycaenidae*	*Lycaena*	*helle*	Dumbrava Vadului, Vad	Brasov	Transylvania	Romania	495
HQ004654	EZROM256-08	RV-07-D251		*Lycaenidae*	*Lycaena*	*helle*	Dumbrava Vadului, Vad	Brasov	Transylvania	Romania	495
HQ004653	EZROM257-08	RV-07-D253		*Lycaenidae*	*Lycaena*	*helle*	Dumbrava Vadului, Vad	Brasov	Transylvania	Romania	495
HQ004652	EZROM674-08	RV-07-D284		*Lycaenidae*	*Lycaena*	*helle*	Dumbrava Vadului, Vad	Brasov	Transylvania	Romania	495
KP870511	EULEP137-14	10-C402		*Lycaenidae*	*Lycaena*	*helle*	Baia Mare	Maramureş	Maramureş	Romania	
KP870723	EULEP138-14	10-C403		*Lycaenidae*	*Lycaena*	*helle*	Baia Mare	Maramureş	Maramureş	Romania	
GU669626	EZSPC655-09	RVcoll.07-C662	x	*Lycaenidae*	*Lycaena*	*hippothoe*	Col de Puymorens	Languedoc-Roussillon		France	1825
GU688462	GWORO788-09	BC ZSM Lep 30480		*Lycaenidae*	*Lycaena*	*hippothoe*	Jachenau / TOEL		Bavaria	Germany	790
KP870557	EULEP151-14	10-C491		*Lycaenidae*	*Lycaena*	*hippothoe*	Gheorgheni, Valea Belchia	Harghita	Transylvania	Romania	
HQ004659	EZRMN049-08	RVcoll.06-N020		*Lycaenidae*	*Lycaena*	*hippothoe*	Gheorgheni, Valea Belchia	Harghita	Transylvania	Romania	1010
HQ004658	EZRMN330-08	RVcoll.08-M783		*Lycaenidae*	*Lycaena*	*hippothoe*	Gheorgheni, Valea Belchia	Harghita	Transylvania	Romania	1000
HQ004661	EZROM258-08	RV-06-M977		*Lycaenidae*	*Lycaena*	*hippothoe*	Gheorgheni, Valea Belchia	Harghita	Transylvania	Romania	1010
HQ004662	EZROM259-08	RV-06-N003		*Lycaenidae*	*Lycaena*	*hippothoe*	Gheorgheni, Valea Belchia	Harghita	Transylvania	Romania	1010
HQ004660	EZROM675-08	RV-06-N011		*Lycaenidae*	*Lycaena*	*hippothoe*	Gheorgheni, Valea Belchia	Harghita	Transylvania	Romania	1010
GU676274	EZSPN885-09	RVcoll.08-L795	x	*Lycaenidae*	*Lycaena*	*hippothoe*	Brana Vieja		Cantabria	Spain	1575
GU676275	EZSPN886-09	RVcoll.08-L796	x	*Lycaenidae*	*Lycaena*	*hippothoe*	Brana Vieja		Cantabria	Spain	1575
GU669764	EZSPC496-09	RVcoll.07-C633	x	*Lycaenidae*	*Lycaena*	*hippothoe*	Meranges	Girona	Catalonia	Spain	1555
GU669765	EZSPC497-09	RVcoll.07-C665	x	*Lycaenidae*	*Lycaena*	*hippothoe*	Meranges	Girona	Catalonia	Spain	1555
GU676092	EZSPM418-09	Rvcoll.09-V841	x	*Lycaenidae*	*Lycaena*	*hippothoe*	Refugi d`Ulldeter, Ripolles	Girona	Catalonia	Spain	
HM901322	EZSPC1122-10	RVcoll.08-P479	x	*Lycaenidae*	*Lycaena*	*hippothoe*	Llanaves de la Reina	Leon	Castilla y Leon	Spain	1443
GU675924	EZSPM312-09	RVcoll.08-J866	x	*Lycaenidae*	*Lycaena*	*hippothoe*	Carande	Leon	Castilla y Leon	Spain	
HM901488	EZSPC1352-10	RVcoll.09-V881	x	*Lycaenidae*	*Lycaena*	*hippothoe*	Banys de Tredos, Vall d`Aran	Lleida	Catalonia	Spain	
HM901489	EZSPC1353-10	RVcoll.09-V882	x	*Lycaenidae*	*Lycaena*	*hippothoe*	Banys de Tredos, Vall d`Aran	Lleida	Catalonia	Spain	
JN827885	EZSPC498-09	RVcoll.07-W214	x	*Lycaenidae*	*Lycaena*	*hippothoe*	Val de Molieres, Vall d`Aran	Lleida	Catalonia	Spain	1650
GU675679	EZSPC499-09	RVcoll.08-M910	x	*Lycaenidae*	*Lycaena*	*hippothoe*	Bassa d`Arres, Vall d`Aran	Lleida	Catalonia	Spain	1500
KP870692	EULEP220-14	12-L051	x	*Lycaenidae*	*Lycaena*	*hippothoe*	Estación de Esqui de Valdezcaray, Ezcaray		La Rioja	Spain	1663
GU688465	GWORO789-09	BC ZSM Lep 30481		*Lycaenidae*	*Lycaena*	*phlaeas*	Riem Muenchen		Bavaria	Germany	
GU707089	FBLMU448-09	BC ZSM Lep 27098		*Lycaenidae*	*Lycaena*	*phlaeas*	Fuerstenfeldbruck-West	Oberbayern	Bavaria	Germany	530
HM391834	FBLMU494-09	BC ZSM Lep 27144		*Lycaenidae*	*Lycaena*	*phlaeas*	Woerth/D., Gmuender Au	Oberpfalz	Bavaria	Germany	320
GU707272	FBLMV674-09	BC ZSM Lep 28654		*Lycaenidae*	*Lycaena*	*phlaeas*	Laaber, Endorfmuehle	Oberpfalz	Bavaria	Germany	420
KP870660	EZSPM706-12	RVcoll.12-L565	x	*Lycaenidae*	*Lycaena*	*phlaeas*	Espiche, Lagos		Algarve	Portugal	
KP870214	EZSPM707-12	RVcoll.12-L578	x	*Lycaenidae*	*Lycaena*	*phlaeas*	Sagres, Vila do Bispo		Algarve	Portugal	

GenBank accession number	BOLD Process ID	Sample ID	Used in the Iberian dataset	Family	Genus	Species	Exact Site	Region	State/Province	Country	Alt. (m)
KP870567	EZSPM708-12	RVcoll.12-L596	x	*Lycaenidae*	*Lycaena*	*phlaeas*	Caldas de Monchique, Monchique		Algarve	Portugal	
KP870304	EZSPM709-12	RVcoll.12-L611	x	*Lycaenidae*	*Lycaena*	*phlaeas*	Rasmalho, Portimao		Algarve	Portugal	
GU676547	EZSPN592-09	RVcoll.08-J175	x	*Lycaenidae*	*Lycaena*	*phlaeas*	Pedras Negras (Sa Larouco), Padornelos, Montalegre		Tras-os-Montes	Portugal	1170
HQ004664	EZRMN050-08	RVcoll.06-M916		*Lycaenidae*	*Lycaena*	*phlaeas*	Cluj-Napoca, dealul Bogomaia, Fanatele Clujului	Cluj	Transylvania	Romania	450
HQ004671	EZROM260-08	RV-06-K651		*Lycaenidae*	*Lycaena*	*phlaeas*	Baciu forest (Cluj-Napoca)	Cluj	Transylvania	Romania	530
HQ004670	EZROM261-08	RV-06-K658		*Lycaenidae*	*Lycaena*	*phlaeas*	Baciu forest (Cluj-Napoca)	Cluj	Transylvania	Romania	530
HQ004666	EZRMN054-08	RVcoll.08-M459		*Lycaenidae*	*Lycaena*	*phlaeas*	Esechioi forest (eastern side)	Constanta	Dobrogea	Romania	135
HQ004669	EZROM262-08	RV-07-D134		*Lycaenidae*	*Lycaena*	*phlaeas*	Dumbraveni forest	Constanta	Dobrogea	Romania	80
HQ004668	EZROM263-08	RV-07-D960		*Lycaenidae*	*Lycaena*	*phlaeas*	Valea Mare	Dambovita	Muntenia	Romania	225
HQ004667	EZRMN052-08	RVcoll.08-M351		*Lycaenidae*	*Lycaena*	*phlaeas*	1 Km E of Gheorgheni	Harghita	Transylvania	Romania	900
HQ004665	EZRMN051-08	RVcoll.08-M235		*Lycaenidae*	*Lycaena*	*phlaeas*	7 Km E of Strehaia	Mehedinti	Oltenia	Romania	125
HQ004663	EZRMN053-08	RVcoll.08-M400		*Lycaenidae*	*Lycaena*	*phlaeas*	Macin Mts. (3 Km S of Jijila)	Tulcea	Dobrogea	Romania	230
FJ663739	LOWA776-06	2005-LOWA-776		*Lycaenidae*	*Lycaena*	*phlaeas*	Osyno	Sebezh distr.	Pskov	Russia	150
FJ663738	LOWA777-06	2005-LOWA-777		*Lycaenidae*	*Lycaena*	*phlaeas*	Osyno	Sebezh distr.	Pskov	Russia	150
GU676669	EZSPN375-09	RVcoll.08-H402	x	*Lycaenidae*	*Lycaena*	*phlaeas*	Cantoblanco		Comunidad de Madrid	Spain	720
GU676687	EZSPN348-09	RVcoll.08-H314	x	*Lycaenidae*	*Lycaena*	*phlaeas*	Casillas, Casillas	Avila	Castilla y Leon	Spain	
KP870536	EZROM732-08	RV-06-H854	x	*Lycaenidae*	*Lycaena*	*phlaeas*	Vallforners, Canoves	Barcelona	Catalonia	Spain	650
GU669759	EZSPC500-09	RVcoll.07-C406	x	*Lycaenidae*	*Lycaena*	*phlaeas*	Viladrau, Osona	Barcelona	Catalonia	Spain	700
GU669760	EZSPC501-09	RVcoll.08-H234	x	*Lycaenidae*	*Lycaena*	*phlaeas*	UAB, Bellaterra	Barcelona	Catalonia	Spain	184
KP870252	EZSPM1038-12	Rvcoll.12-M789	x	*Lycaenidae*	*Lycaena*	*phlaeas*	Carrascalejo	Caceres	Extremadura	Spain	636
GU676604	EZSPN445-09	RVcoll.08-H631	x	*Lycaenidae*	*Lycaena*	*phlaeas*	El Gastor	Cadiz	Andalusia	Spain	660
GU676427	EZSPN723-09	RVcoll.08-L114	x	*Lycaenidae*	*Lycaena*	*phlaeas*	Puerto de las Cabrillas, Portell de Morella	Castellon	Comunidad Valenciana	Spain	1301
KP870960	EZSPM930-12	Rvcoll.12-M660	x	*Lycaenidae*	*Lycaena*	*phlaeas*	Mina de los Pontones, Mestanza	Ciudad Real	Castilla-La Mancha	Spain	450
KP871079	EZSPM976-12	Rvcoll.12-M734	x	*Lycaenidae*	*Lycaena*	*phlaeas*	Arroyo de los Molinillos, Viso del Marques	Ciudad Real	Castilla-La Mancha	Spain	742
GU676847	EZSPN087-09	RVcoll.06-A011	x	*Lycaenidae*	*Lycaena*	*phlaeas*	Road from Ferreira to Puerto de La Ragua	Granada	Andalusia	Spain	1600
GU676455	EZSPN688-09	RVcoll.08-L017	x	*Lycaenidae*	*Lycaena*	*phlaeas*	Ascenso al Veleta, Gueejar Sierra	Granada	Andalusia	Spain	2270
GU669761	EZSPC502-09	RVcoll.08-L437	x	*Lycaenidae*	*Lycaena*	*phlaeas*	Baltarga, La Cerdenya	Lleida	Catalonia	Spain	1121
GU669755	EZSPC503-09	RVcoll.08-M906	x	*Lycaenidae*	*Lycaena*	*phlaeas*	Bassa d`Arres, Vall d`Aran	Lleida	Catalonia	Spain	1500
GU675984	EZSPM046-09	RVcoll.08-P261	x	*Lycaenidae*	*Lycaena*	*phlaeas*	Santa Maria	Mallorca	Balearic Islands	Spain	181
GU675981	EZSPM051-09	RVcoll.08-P269	x	*Lycaenidae*	*Lycaena*	*phlaeas*	S`Albufera	Mallorca	Balearic Islands	Spain	4
GU675983	EZSPM071-09	RVcoll.08-P312	x	*Lycaenidae*	*Lycaena*	*phlaeas*	Castell de Bellver, Palma, Mallorca	Mallorca	Balearic Islands	Spain	
KP870228	EZSPM998-12	Rvcoll.12-L884	x	*Lycaenidae*	*Lycaena*	*phlaeas*	Santa Cecilia, Ferreries, Menorca	Menorca	Balearic Islands	Spain	
GU676727	EZSPN307-09	RVcoll.08-H113	x	*Lycaenidae*	*Lycaena*	*phlaeas*	Na Vermella, Mao	Menorca	Balearic Islands	Spain	
GU676717	EZSPN321-09	RVcoll.08-H182	x	*Lycaenidae*	*Lycaena*	*phlaeas*	Llimpa, Mao	Menorca	Balearic Islands	Spain	
GU675860	EZSPM379-09	RVcoll.08-J873	x	*Lycaenidae*	*Lycaena*	*phlaeas*	El Vallecillo	Teruel	Aragon	Spain	
HQ004673	EZRMN056-08	RVcoll.07-E445		*Lycaenidae*	*Lycaena*	*thersamon*	2 km E of Smardan	Braila	Muntenia	Romania	5
HQ004676	EZROM265-08	RV-07-E468		*Lycaenidae*	*Lycaena*	*thersamon*	Dumbrava Vadului, Vad	Brasov	Transylvania	Romania	495
HQ004674	EZRMN055-08	RVcoll.07-C143		*Lycaenidae*	*Lycaena*	*thersamon*	Popesti (Cluj-Napoca)	Cluj	Transylvania	Romania	450
HQ004675	EZROM264-08	RV-06-K665		*Lycaenidae*	*Lycaena*	*thersamon*	Valea Calda (Cluj-Napoca)	Cluj	Transylvania	Romania	380
HQ004677	EZROM266-08	RV-07-D007		*Lycaenidae*	*Lycaena*	*thersamon*	Canaraua Fetei (Baneasa)	Constanta	Dobrogea	Romania	15
HQ004672	EZRMN057-08	RVcoll.08-M396		*Lycaenidae*	*Lycaena*	*thersamon*	Macin Mts. (3 Km S of Jijila)	Tulcea	Dobrogea	Romania	230
HQ004678	EZROM676-08	RV-07-E444		*Lycaenidae*	*Lycaena*	*thersamon*	2 km E of Smardan	Tulcea	Dobrogea	Romania	5
GU688459	GWORO791-09	BC ZSM Lep 30483		*Lycaenidae*	*Lycaena*	*tityrus*	Zellwies TOEL		Bavaria	Germany	
HM391835	FBLMU496-09	BC ZSM Lep 27146		*Lycaenidae*	*Lycaena*	*tityrus*	Saulburg	Niederbayern	Bavaria	Germany	360
HM393192	GWORA2468-09	BC ZSM Lep 30680		*Lycaenidae*	*Lycaena*	*tityrus*	Grandsberg	Niederbayern	Bavaria	Germany	850
JF415711	GWORK519-09	BC ZSM Lep 21849		*Lycaenidae*	*Lycaena*	*tityrus*	Diessen	Oberbayern	Bavaria	Germany	600
GU676573	EZSPN570-09	RVcoll.08-J114	x	*Lycaenidae*	*Lycaena*	*tityrus*	Ribeira de Varzea (EN311), Varzea Cova, Fafe		Minho	Portugal	500
HQ004679	EZRMN061-08	RVcoll.08-M612		*Lycaenidae*	*Lycaena*	*tityrus*	3 km below cabana Capra (Fagaras Mts.)	Arges	Muntenia	Romania	1350
HQ004684	EZROM270-08	RV-07-E479		*Lycaenidae*	*Lycaena*	*tityrus*	Cabana Capra (Fagaras Mts.)	Arges	Muntenia	Romania	1630
HQ004681	EZRMN058-08	RVcoll.07-D317		*Lycaenidae*	*Lycaena*	*tityrus*	Racos	Brasov	Transylvania	Romania	460
HQ004685	EZROM269-08	RV-07-C903		*Lycaenidae*	*Lycaena*	*tityrus*	Teregova	Caras-Severin	Banat	Romania	385
HQ004687	EZROM267-08	RV-06-K611		*Lycaenidae*	*Lycaena*	*tityrus*	Badeni	Cluj	Transylvania	Romania	440

GenBank accession number	BOLD Process ID	Sample ID	Used in the Iberian dataset	Family	Genus	Species	Exact Site	Region	State/Province	Country	Alt. (m)
HQ004682	EZRMN059-08	RVcoll.08-M460		*Lycaenidae*	*Lycaena*	*tityrus*	Esechioi forest (eastern side)	Constanta	Dobrogea	Romania	135
HQ004686	EZROM268-08	RV-06-M976		*Lycaenidae*	*Lycaena*	*tityrus*	Gheorgheni, Valea Belchia	Harghita	Transylvania	Romania	1010
HQ004680	EZRMN062-08	RVcoll.08-M639		*Lycaenidae*	*Lycaena*	*tityrus*	Scorota (Retezat Mts.)	Hunedoara	Transylvania	Romania	1560
HQ004683	EZRMN060-08	RVcoll.08-M559		*Lycaenidae*	*Lycaena*	*tityrus*	4 Km W of Drobeta Turnu-Severin	Mehedinti	Oltenia	Romania	75
FJ663596	LOWA289-06	2005-LOWA-289		*Lycaenidae*	*Lycaena*	*tityrus*	Osyno	Sebezh distr.	Pskov	Russia	
GU675689	EZSPM142-09	RVcoll.08-P654	x	*Lycaenidae*	*Lycaena*	*tityrus*	Valle (Valle-Zurea), Lena		Asturias	Spain	662
GU675690	EZSPM143-09	RVcoll.08-P655	x	*Lycaenidae*	*Lycaena*	*tityrus*	Valle (Valle-Zurea), Lena		Asturias	Spain	662
KP871012	EZSPM769-12	RVcoll.050111LP02	x	*Lycaenidae*	*Lycaena*	*tityrus*	Fondos de Vega, Degana		Asturias	Spain	1079
GU676648	EZSPN391-09	RVcoll.08-H447	x	*Lycaenidae*	*Lycaena*	*tityrus*	San Andres, Valdeprado del Rio		Cantabria	Spain	976
GU675802	EZSPM252-09	RVcoll.08-R464	x	*Lycaenidae*	*Lycaena*	*tityrus*	Padron, Picarana-Rio Tinto	A Coruna	Galicia	Spain	30
GU669756	EZSPC504-09	RVcoll.07-C624	x	*Lycaenidae*	*Lycaena*	*tityrus*	Meranges	Girona	Catalonia	Spain	1555
GU669758	EZSPC506-09	RVcoll.08-P003	x	*Lycaenidae*	*Lycaena*	*tityrus*	Torre de Riu, Ribera d`Alp, Cerdanya	Girona	Catalonia	Spain	1180
GU669751	EZSPC507-09	RVcoll.08-P004	x	*Lycaenidae*	*Lycaena*	*tityrus*	Torre de Riu, Ribera d`Alp, Cerdanya	Girona	Catalonia	Spain	1180
GU669752	EZSPC508-09	RVcoll.08-P033	x	*Lycaenidae*	*Lycaena*	*tityrus*	La Valira (near Urus), Cerdanya	Girona	Catalonia	Spain	1300
GU675935	EZSPM297-09	RVcoll.08-J842	x	*Lycaenidae*	*Lycaena*	*tityrus*	Posada de Valdeon	Leon	Castilla y Leon	Spain	
GU669757	EZSPC505-09	RVcoll.08-R309	x	*Lycaenidae*	*Lycaena*	*tityrus*	Planes de Son, Pallars Sobira	Lleida	Catalonia	Spain	1589
GU688460	GWORO760-09	BC ZSM Lep 30452		*Lycaenidae*	*Lycaena*	*virgaureae*	Anzing, Kreis Ebersberg		Bavaria	Germany	
HM393191	GWORA2465-09	BC ZSM Lep 30677		*Lycaenidae*	*Lycaena*	*virgaureae*	Leak near Regensburg, Thiergarten	Oberpfalz	Bavaria	Germany	330
HQ004692	EZROM677-08	RV-06-N002		*Lycaenidae*	*Lycaena*	*virgaureae*	Apuseni Mts., Rimetea (Piatra Secuiului)	Alba	Transylvania	Romania	750
HQ004691	EZRMN065-08	RVcoll.07-C907		*Lycaenidae*	*Lycaena*	*virgaureae*	Teregova	Caras-Severin	Banat	Romania	385
HQ004695	EZROM271-08	RV-06-M879		*Lycaenidae*	*Lycaena*	*virgaureae*	Cluj-Napoca, dealul Feleacului, Gheorgheni forest	Cluj	Transylvania	Romania	600
HQ004688	EZRMN066-08	RVcoll.08-M590		*Lycaenidae*	*Lycaena*	*virgaureae*	Gheorgheni, Valea Belchia	Harghita	Transylvania	Romania	1000
HQ004690	EZRMN064-08	RVcoll.06-V698		*Lycaenidae*	*Lycaena*	*virgaureae*	Retezat Mts., poiana Dragsanului	Hunedoara	Transylvania	Romania	1765
HQ004694	EZROM273-08	RV-07-E489		*Lycaenidae*	*Lycaena*	*virgaureae*	Saua Dragsanului (Retezat Mts.)	Hunedoara	Transylvania	Romania	1780
HQ004693	EZROM272-08	RV-07-D518		*Lycaenidae*	*Lycaena*	*virgaureae*	Rohia	Maramures	Maramures	Romania	360
HQ004689	EZRMN063-08	RVcoll.06-M904		*Lycaenidae*	*Lycaena*	*virgaureae*	Stana	Salaj	Transylvania	Romania	500
HM901301	EZSPC1100-10	RVcoll.08-L969	x	*Lycaenidae*	*Lycaena*	*virgaureae*	La Morcuera, Rascafria		Comunidad de Madrid	Spain	1766
KP870274	EZSPM849-12	RVcoll.150511SD71	x	*Lycaenidae*	*Lycaena*	*virgaureae*	Fondos de Vega, Degana		Asturias	Spain	1079
GU676208	EZSPN947-09	RVcoll.08-L936	x	*Lycaenidae*	*Lycaena*	*virgaureae*	Puerto de La Morcuera		Comunidad de Madrid	Spain	1766
GU676198	EZSPN962-09	RVcoll.08-L968	x	*Lycaenidae*	*Lycaena*	*virgaureae*	La Morcuera, Rascafria		Comunidad de Madrid	Spain	1766
HM901345	EZSPC1145-10	RVcoll.08-P762	x	*Lycaenidae*	*Lycaena*	*virgaureae*	La Serrota	Avila	Castilla y Leon	Spain	1835
GU669753	EZSPC509-09	RVcoll.08-R197	x	*Lycaenidae*	*Lycaena*	*virgaureae*	Setcases	Girona	Catalonia	Spain	1350
GU669749	EZSPC513-09	RVcoll.08-P006	x	*Lycaenidae*	*Lycaena*	*virgaureae*	Torre de Riu, Ribera d`Alp, Cerdanya	Girona	Catalonia	Spain	1180
HM901435	EZSPC1239-10	RVcoll.09-V750	x	*Lycaenidae*	*Lycaena*	*virgaureae*	Valle del Tena, Camino embalse Respomuso, Sallent de Gallego	Huesca	Aragon	Spain	1650
GU675986	EZSPM083-09	RVcoll.08-P403	x	*Lycaenidae*	*Lycaena*	*virgaureae*	Buron	Leon	Castilla y Leon	Spain	1120
KP870936	EZSPM163-09	RVcoll.08-P692	x	*Lycaenidae*	*Lycaena*	*virgaureae*	Villadangos de Arbas	Leon	Castilla y Leon	Spain	1311
GU675931	EZSPM301-09	RVcoll.08-J848	x	*Lycaenidae*	*Lycaena*	*virgaureae*	Puerto de San Glorio	Leon	Castilla y Leon	Spain	
GU669754	EZSPC510-09	RVcoll.08-M921	x	*Lycaenidae*	*Lycaena*	*virgaureae*	Arros, Vall d`Aran	Lleida	Catalonia	Spain	906
GU669747	EZSPC511-09	RVcoll.08-P386	x	*Lycaenidae*	*Lycaena*	*virgaureae*	Arros, Vall d`Aran	Lleida	Catalonia	Spain	906
GU669748	EZSPC512-09	RVcoll.08-R117	x	*Lycaenidae*	*Lycaena*	*virgaureae*	Salt de Pitx, Vall d`Aran	Lleida	Catalonia	Spain	1570
KP870453	EZSPM857-12	Rvcoll.040611HZ30	x	*Lycaenidae*	*Lysandra*	*albicans*	Moli de Joaquim, La Pobla de Benifassa	Castellon	Comunidad Valenciana	Spain	1025
GU676379	EZSPN766-09	RVcoll.08-L265	x	*Lycaenidae*	*Lysandra*	*albicans*	Font d`Horta, Vilafranca	Castellon	Comunidad Valenciana	Spain	1049
GU676079	EZSPM440-09	Rvcoll.09-V499	x	*Lycaenidae*	*Lysandra*	*albicans*	Sierra de la Sagra	Granada	Andalusia	Spain	1745
GU676076	EZSPM441-09	Rvcoll.09-V565	x	*Lycaenidae*	*Lysandra*	*albicans*	Dornajo (Sierra Nevada)	Granada	Andalusia	Spain	2060
JN276898	EZSPN1077-11	Rvcoll.09-V944	x	*Lycaenidae*	*Lysandra*	*albicans*	Collados de la Sagra	Granada	Andalusia	Spain	1475
GU676470	EZSPN679-09	RVcoll.08-J992	x	*Lycaenidae*	*Lysandra*	*albicans*	Casillas de Rojas, Gueejar Sierra	Granada	Andalusia	Spain	1733
KF834395		RV-03-H582	x	*Lycaenidae*	*Lysandra*	*albicans*	Puebla de Don Fadrique	Granada	Andalusia	Spain	1295
GU675756	EZSPM109-09	RVcoll.08-P449	x	*Lycaenidae*	*Lysandra*	*albicans*	Zaorejas, Alto Tajo	Guadalajara	Castilla-La Mancha	Spain	1284
GU677038	EZSPN177-09	RVcoll.06-K748	x	*Lycaenidae*	*Lysandra*	*albicans*	Duron	Guadalajara	Castilla-La Mancha	Spain	755
GU675978	EZSPM030-09	RVcoll.08-J813	x	*Lycaenidae*	*Lysandra*	*albicans*	Villavente	Leon	Castilla y Leon	Spain	

GenBank accession number	BOLD Process ID	Sample ID	Used in the Iberian dataset	Family	Genus	Species	Exact Site	Region	State/Province	Country	Alt. (m)
KP870300	EZSPM576-12	RVcoll.08-P933	x	*Lycaenidae*	*Lysandra*	*albicans*	Cistierna	Leon	Castilla y Leon	Spain	1100
KP871078	EZSPM578-12	RVcoll.08-P936	x	*Lycaenidae*	*Lysandra*	*albicans*	Cremenes	Leon	Castilla y Leon	Spain	1100
GU675757	EZSPM126-09	RVcoll.08-P618	x	*Lycaenidae*	*Lysandra*	*albicans*	Albarracin	Teruel	Aragon	Spain	1250
GU675896	EZSPM342-09	RVcoll.08-R006	x	*Lycaenidae*	*Lysandra*	*albicans*	Paraisos Altos, Manzanera	Teruel	Aragon	Spain	1200
KP871154	EZSPM859-12	Rvcoll.040611HZ81	x	*Lycaenidae*	*Lysandra*	*albicans*	Fuentes Calientes	Teruel	Aragon	Spain	1273
GU676389	EZSPN764-09	RVcoll.08-L249	x	*Lycaenidae*	*Lysandra*	*albicans*	Las Tajadas, Albarracin	Teruel	Aragon	Spain	1271
GU676368	EZSPN780-09	RVcoll.08-L292	x	*Lycaenidae*	*Lysandra*	*albicans*	Paraisos Altos, Manzanera	Teruel	Aragon	Spain	1200
KP870475	EZSPM580-12	RVcoll.08-P938	x	*Lycaenidae*	*Lysandra*	*albicans*	La Mata, CM 4053 Km 13	Toledo	Castilla-La Mancha	Spain	550
KP871133	EZSPM581-12	RVcoll.08-P939	x	*Lycaenidae*	*Lysandra*	*albicans*	La Mata, CM 4053 Km 13	Toledo	Castilla-La Mancha	Spain	550
GU676952	EZSPN553-09	RVcoll.08-J065	x	*Lycaenidae*	*Lysandra*	*albicans*	Ayora, Ayora	Valencia	Comunidad Valenciana	Spain	
GU688456	GWORO800-09	BC ZSM Lep 30492		*Lycaenidae*	*Lysandra*	*bellargus*	Garching-Hochbrueck MuenchenL		Bavaria	Germany	486
GU688453	GWORO801-09	BC ZSM Lep 30493		*Lycaenidae*	*Lysandra*	*bellargus*	Samerberg (RO) Lederstuben		Bavaria	Germany	750
GU655006	FBLMT406-09	BC ZSM Lep 24966		*Lycaenidae*	*Lysandra*	*bellargus*	Kelheim, Lehnberg	Niederbayern	Bavaria	Germany	395
GU707371	FBLMV428-09	BC ZSM Lep 28408		*Lycaenidae*	*Lysandra*	*bellargus*	Woerthsee, Etterschlag	Oberbayern	Bavaria	Germany	580
GU707036	FBLMU255-09	BC ZSM Lep 25765		*Lycaenidae*	*Lysandra*	*bellargus*	Kallmuenz, Kirchenberg	Oberpfalz	Bavaria	Germany	390
KP870691	EZSPM711-12	RVcoll.12-L597	x	*Lycaenidae*	*Lysandra*	*bellargus*	Caldas de Monchique, Monchique		Algarve	Portugal	
HQ005013	EZRMN070-08	RVcoll.08-M563		*Lycaenidae*	*Lysandra*	*bellargus*	Breaza, dealul Istrita	Buzau	Muntenia	Romania	600
HQ005017	EZROM496-08	RV-06-M933		*Lycaenidae*	*Lysandra*	*bellargus*	Dealul Istrita, Breaza	Buzau	Muntenia	Romania	600
HQ005016	EZROM497-08	RV-06-M938		*Lycaenidae*	*Lysandra*	*bellargus*	Dealul Istrita, Breaza	Buzau	Muntenia	Romania	600
HQ005019	EZROM275-08	RV-07-D435		*Lycaenidae*	*Lysandra*	*bellargus*	Suatu	Cluj	Transylvania	Romania	380
HQ005018	EZROM274-08	RV-07-D149		*Lycaenidae*	*Lysandra*	*bellargus*	Dumbraveni forest	Constanta	Dobrogea	Romania	80
HQ005011	EZRMN068-08	RVcoll.08-M388		*Lycaenidae*	*Lysandra*	*bellargus*	Garboavele forest	Galati	Moldavia	Romania	90
HQ005015	EZROM678-08	RV-07-D948		*Lycaenidae*	*Lysandra*	*bellargus*	Pestera Muierii, Baia de Fier	Gorj	Oltenia	Romania	640
HQ005010	EZRMN067-08	RVcoll.07-D497		*Lycaenidae*	*Lysandra*	*bellargus*	Cheile Babei	Maramures	Maramures	Romania	265
HQ005014	EZROM636-08	RV-07-D529		*Lycaenidae*	*Lysandra*	*bellargus*	Rohia	Maramures	Maramures	Romania	360
HQ005012	EZRMN069-08	RVcoll.08-M560		*Lycaenidae*	*Lysandra*	*bellargus*	4 Km W of Drobeta Turnu-Severin	Mehedinti	Oltenia	Romania	75
GU677050	EZSPN356-09	RVcoll.08-H331	x	*Lycaenidae*	*Lysandra*	*bellargus*	La Maranosa, San Martin de la Vega		Comunidad de Madrid	Spain	
GU676647	EZSPN390-09	RVcoll.08-H444	x	*Lycaenidae*	*Lysandra*	*bellargus*	San Andres, Valdeprado del Rio		Cantabria	Spain	950
KP870673	EZROM733-08	RV-06-G452	x	*Lycaenidae*	*Lysandra*	*bellargus*	Can Rovira, El Brull	Barcelona	Catalonia	Spain	830
GU669750	EZSPC514-09	RVcoll.06-G560	x	*Lycaenidae*	*Lysandra*	*bellargus*	El Sola, El Brull, Osona	Barcelona	Catalonia	Spain	800
GU669743	EZSPC515-09	RVcoll.08-R282	x	*Lycaenidae*	*Lysandra*	*bellargus*	Masia del Plans, Vidra, La Garrotxa	Barcelona	Catalonia	Spain	1200
HM901585	EZSPC730-10	RVcoll.08-H666	x	*Lycaenidae*	*Lysandra*	*bellargus*	Grazalema, Puerto de los Alamillos	Cadiz	Andalusia	Spain	887
GU676582	EZSPN458-09	RVcoll.08-H665	x	*Lycaenidae*	*Lysandra*	*bellargus*	Grazalema, Puerto de los Alamillos	Cadiz	Andalusia	Spain	887
HM901603	EZSPC748-10	RVcoll.08-L071	x	*Lycaenidae*	*Lysandra*	*bellargus*	Rambla de las Truchas, Vilafranca	Castellon	Comunidad Valenciana	Spain	1150
KP870914	EZSPM922-12	Rvcoll.12-M644	x	*Lycaenidae*	*Lysandra*	*bellargus*	Lagunas de Ruidera, Ossa de Montiel	Ciudad Real	Castilla-La Mancha	Spain	795
HM901670	EZSPC822-10	RVcoll.08-P722	x	*Lycaenidae*	*Lysandra*	*bellargus*	La Calahorra	Granada	Andalusia	Spain	1255
GU676456	EZSPN689-09	RVcoll.08-L019	x	*Lycaenidae*	*Lysandra*	*bellargus*	Casillas de Rojas, Gueejar Sierra	Granada	Andalusia	Spain	1733
HM901581	EZSPC726-10	RVcoll.08-H581	x	*Lycaenidae*	*Lysandra*	*bellargus*	Gualda, La Alcarria	Guadalajara	Castilla-La Mancha	Spain	742
GU676798	EZSPN134-09	RVcoll.06-G483	x	*Lycaenidae*	*Lysandra*	*bellargus*	Barranco de Valcuerna, Candasnos	Huesca	Aragon	Spain	210
HM901790	EZSPC954-10	RVcoll.09-X532	x	*Lycaenidae*	*Lysandra*	*bellargus*	Buron	Leon	Castilla y Leon	Spain	1120
GU669744	EZSPC516-09	RVcoll.08-L439	x	*Lycaenidae*	*Lysandra*	*bellargus*	Baltarga, La Cerdenya	Lleida	Catalonia	Spain	1121
GU669745	EZSPC517-09	RVcoll.08-M925	x	*Lycaenidae*	*Lysandra*	*bellargus*	Vilamos, Vall d`Aran	Lleida	Catalonia	Spain	1178
GU675798	EZSPM269-09	RVcoll.08-R486	x	*Lycaenidae*	*Lysandra*	*bellargus*	Folgoso do Caurel, Alto de Visuna	Lugo	Galicia	Spain	1330
GU676507	EZSPN637-09	RVcoll.08-J760	x	*Lycaenidae*	*Lysandra*	*bellargus*	El Masegar, Moscardon	Teruel	Aragon	Spain	1531
GU675752	EZSPM173-09	RVcoll.08-R036	x	*Lycaenidae*	*Lysandra*	*bellargus*	Casas de Barchell, Chelva	Valencia	Comunidad Valenciana	Spain	
FJ663748	LOWA762-06	2005-LOWA-762		*Lycaenidae*	*Lysandra*	*bellargus*	Karadag Reserve		Crimea	Ukraine	
FJ663747	LOWA763-06	2005-LOWA-763		*Lycaenidae*	*Lysandra*	*bellargus*	Karadag Reserve		Crimea	Ukraine	
FJ663746	LOWA764-06	2005-LOWA-764		*Lycaenidae*	*Lysandra*	*bellargus*	Karadag Reserve		Crimea	Ukraine	
GU675754	EZSPM118-09	RVcoll.08-P601	x	*Lycaenidae*	*Lysandra*	*caelestissima*	Piscifactoria, Laguna de Una, Una	Cuenca	Castilla-La Mancha	Spain	1219
GU676753	EZSPN285-09	RVcoll.07-W230	x	*Lycaenidae*	*Lysandra*	*caelestissima*	Sierra de los Barrancos, Cuenca	Cuenca	Castilla-La Mancha	Spain	1447
GU675753	EZSPM110-09	RVcoll.08-P450	x	*Lycaenidae*	*Lysandra*	*caelestissima*	Zaorejas, Alto Tajo	Guadalajara	Castilla-La Mancha	Spain	1284
JN276905	EZSPN1109-11	Rvcoll.10-B528	x	*Lycaenidae*	*Lysandra*	*caelestissima*	El Valecillo	Teruel	Aragon	Spain	1490
GU676387	EZSPN761-09	RVcoll.08-L242	x	*Lycaenidae*	*Lysandra*	*caelestissima*	Ruta de la Sima, El Vallecillo	Teruel	Aragon	Spain	1531

GenBank accession number	BOLD Process ID	Sample ID	Used in the Iberian dataset	Family	Genus	Species	Exact Site	Region	State/Province	Country	Alt. (m)
GU676378	EZSPN765-09	RVcoll.08-L254	x	*Lycaenidae*	*Lysandra*	*caelestissima*	Ruta de la Sima, El Vallecillo	Teruel	Aragon	Spain	1531
JN276881	EZSPC1274-10	RVcoll.070207MC50	x	*Lycaenidae*	*Lysandra*	*caelestissima* x *albicans* (*caerulescens*)?	Pino Monumental, Moscardon	Teruel	Aragon	Spain	1415
GU676194	EZSPN970-09	RVcoll.08-L990	x	*Lycaenidae*	*Lysandra*	*caelestissima* x *albicans* (*caerulescens*)?	Uña	Cuenca	Castilla-La Mancha	Spain	1201
GU676826	EZSPN106-09	RVcoll.06-A185	x	*Lycaenidae*	*Lysandra*	*coridon*	Comapedrosa			Andorra	1800
GU688454	GWORO802-09	BC ZSM Lep 30494		*Lycaenidae*	*Lysandra*	*coridon*	Vorderriss TOEL		Bavaria	Germany	760
HM393185	GWORA2453-09	BC ZSM Lep 30665		*Lycaenidae*	*Lysandra*	*coridon*	Arzmoos	Oberbayern	Bavaria	Germany	1200
HQ563553	FBLMW313-10	BC ZSM Lep 37414		*Lycaenidae*	*Lysandra*	*coridon*	Wonsees, Schloetzmuehle	Oberfranken	Bavaria	Germany	407
JF415725	GWORK302-09	BC ZSM Lep 21537		*Lycaenidae*	*Lysandra*	*coridon*	Moernsheim, Steinbruch 2 km w	Oberpfalz	Bavaria	Germany	430
HQ563554	FBLMW314-10	BC ZSM Lep 37415		*Lycaenidae*	*Lysandra*	*coridon*	Schwebheim	Unterfranken	Bavaria	Germany	210
HQ005023	EZRMN074-08	RVcoll.07-E563		*Lycaenidae*	*Lysandra*	*coridon*	3 km S of Fundata	Arges	Muntenia	Romania	1190
HQ005028	EZROM280-08	RV-07-E559		*Lycaenidae*	*Lysandra*	*coridon*	3 km S of Fundata	Arges	Muntenia	Romania	1190
HQ005020	EZRMN071-08	RVcoll.06-V713		*Lycaenidae*	*Lysandra*	*coridon*	Padurea Craiului Mts., Suncuius	Bihor	Transylvania	Romania	370
HQ005029	EZROM278-08	RV-07-E439		*Lycaenidae*	*Lysandra*	*coridon*	Dealul Istrita, Breaza	Buzau	Muntenia	Romania	450
HQ005030	EZROM279-08	RV-07-E440		*Lycaenidae*	*Lysandra*	*coridon*	Dealul Istrita, Breaza	Buzau	Muntenia	Romania	450
HQ005021	EZRMN072-08	RVcoll.07-E373		*Lycaenidae*	*Lysandra*	*coridon*	Cerna Sat	Caras-Severin	Banat	Romania	525
HQ005024	EZROM276-08	RV-07-E305		*Lycaenidae*	*Lysandra*	*coridon*	Baile Herculane (Pecinisca)	Caras-Severin	Banat	Romania	270
HQ005027	EZROM281-08	RV-07-C174		*Lycaenidae*	*Lysandra*	*coridon*	Popesti (Cluj-Napoca)	Cluj	Transylvania	Romania	530
HQ005026	EZROM282-08	RV-07-C175		*Lycaenidae*	*Lysandra*	*coridon*	Popesti (Cluj-Napoca)	Cluj	Transylvania	Romania	530
HQ005022	EZRMN073-08	RVcoll.07-E407		*Lycaenidae*	*Lysandra*	*coridon*	3 km E of Slava Rusa (Babadag forest)	Tulcea	Dobrogea	Romania	130
HQ005025	EZROM277-08	RV-07-E402		*Lycaenidae*	*Lysandra*	*coridon*	8 Km S of Babadag (Babadag forest)	Tulcea	Dobrogea	Romania	115
FJ663750	LOWA283-06	2005-LOWA-283		*Lycaenidae*	*Lysandra*	*coridon*	Kamyshinka	Don River	Volgograd	Russia	
FJ663749	LOWA284-06	2005-LOWA-284		*Lycaenidae*	*Lysandra*	*coridon*	Kamyshinka	Don River	Volgograd	Russia	
GU675758	EZSPM148-09	RVcoll.08-P662	x	*Lycaenidae*	*Lysandra*	*coridon*	Gamoniteiru, Sierra de Aramo, Quiros		Asturias	Spain	1712
KP871069	EZSPM881-12	Rvcoll.150511SD05	x	*Lycaenidae*	*Lysandra*	*coridon*	PR-23 de Fuente De, Camaleno		Asturias	Spain	1093
HM901660	EZSPC810-10	RVcoll.08-P557	x	*Lycaenidae*	*Lysandra*	*coridon*	Cedeira, Serra da Capelada	A Coruna	Galicia	Spain	30
HM901661	EZSPC811-10	RVcoll.08-P558	x	*Lycaenidae*	*Lysandra*	*coridon*	Cedeira, Serra da Capelada	A Coruna	Galicia	Spain	30
GU677027	EZSPN189-09	RVcoll.07-C272	x	*Lycaenidae*	*Lysandra*	*coridon*	Cedeira, Serra da Capelada	A Coruna	Galicia	Spain	30
GU677024	EZSPN190-09	RVcoll.07-C273	x	*Lycaenidae*	*Lysandra*	*coridon*	Cedeira, Serra da Capelada	A Coruna	Galicia	Spain	30
GU669746	EZSPC518-09	RVcoll.06-V740	x	*Lycaenidae*	*Lysandra*	*coridon*	Serra Pedregosa, Cadi, Saldes, Bergueda	Barcelona	Catalonia	Spain	2250
GU669739	EZSPC519-09	RVcoll.06-V744	x	*Lycaenidae*	*Lysandra*	*coridon*	Serra Pedregosa, Cadi, Saldes, Bergueda	Barcelona	Catalonia	Spain	2025
JX678094		RV-03-H454	x	*Lycaenidae*	*Lysandra*	*coridon*	El Brull, Osona	Barcelona	Catalonia	Spain	830
GU676831	EZSPN103-09	RVcoll.06-A165	x	*Lycaenidae*	*Lysandra*	*coridon*	Pto. de la Pez, Pluviometro	Huesca	Aragon	Spain	1800
GU676832	EZSPN104-09	RVcoll.06-A166	x	*Lycaenidae*	*Lysandra*	*coridon*	Val de Barbarisa, Pleta d`es Prats	Huesca	Aragon	Spain	1800
GU676825	EZSPN105-09	RVcoll.06-A169	x	*Lycaenidae*	*Lysandra*	*coridon*	Pto. de la Pez, Pluviometro	Huesca	Aragon	Spain	1800
GU676910	EZSPN507-09	RVcoll.08-H965	x	*Lycaenidae*	*Lysandra*	*coridon*	Laguarta, Rio Alcanadre	Huesca	Aragon	Spain	1216
HM901791	EZSPC955-10	RVcoll.09-X545	x	*Lycaenidae*	*Lysandra*	*coridon*	Puerto de San Isidro	Leon	Castilla y Leon	Spain	1215
GU675975	EZSPM087-09	RVcoll.08-P410	x	*Lycaenidae*	*Lysandra*	*coridon*	Buron	Leon	Castilla y Leon	Spain	1116
GU675976	EZSPM089-09	RVcoll.08-P413	x	*Lycaenidae*	*Lysandra*	*coridon*	Buron	Leon	Castilla y Leon	Spain	1116
GU675759	EZSPM162-09	RVcoll.08-P690	x	*Lycaenidae*	*Lysandra*	*coridon*	Villadangos de Arbas	Leon	Castilla y Leon	Spain	1311
GU675936	EZSPM298-09	RVcoll.08-J845	x	*Lycaenidae*	*Lysandra*	*coridon*	Posada de Valdeon	Leon	Castilla y Leon	Spain	
KP870401	EZSPN788-09	RVcoll.08-L347	x	*Lycaenidae*	*Lysandra*	*coridon*	Jou Santu (Pena Santa de Castilla), Picos de Europa, Macizo Occidental	Leon	Castilla y Leon	Spain	
GU669740	EZSPC520-09	RVcoll.06-A127	x	*Lycaenidae*	*Lysandra*	*coridon*	Conangles,Vall d`Aran	Lleida	Catalonia	Spain	2000
GU669741	EZSPC521-09	RVcoll.06-A147	x	*Lycaenidae*	*Lysandra*	*coridon*	Prats d`Aguilo, Montella i Martinet, La Cerdanya	Lleida	Catalonia	Spain	2200
GU669742	EZSPC522-09	RVcoll.08-R210	x	*Lycaenidae*	*Lysandra*	*coridon*	Port de la Bonaigua, Pallars Sobira	Lleida	Catalonia	Spain	2338
GU676362	EZSPN786-09	RVcoll.08-L344	x	*Lycaenidae*	*Lysandra*	*coridon*	Courel, Devesa da Rogueira	Lugo	Galicia	Spain	
GU676363	EZSPN787-09	RVcoll.08-L345	x	*Lycaenidae*	*Lysandra*	*coridon*	Courel, Devesa da Rogueira	Lugo	Galicia	Spain	
GU676383	EZSPN771-09	RVcoll.08-L280	x	*Lycaenidae*	*Lysandra*	*hispana*	Los Collados - Benimaurell, Vall de Laguart	Alicante	Comunidad Valenciana	Spain	675
GU676373	EZSPN773-09	RVcoll.08-L282	x	*Lycaenidae*	*Lysandra*	*hispana*	Alrededores del Somo, Castell de Castells	Alicante	Comunidad Valenciana	Spain	766

GenBank accession number	BOLD Process ID	Sample ID	Used in the Iberian dataset	Family	Genus	Species	Exact Site	Region	State/Province	Country	Alt. (m)
GU676377	EZSPN777-09	RVcoll.08-L287	x	*Lycaenidae*	*Lysandra*	*hispana*	Alrededores del Somo, Castell de Castells	Alicante	Comunidad Valenciana	Spain	766
GU677010	EZSPN206-09	RVcoll.07-F050	x	*Lycaenidae*	*Lysandra*	*hispana*	10 km S. of Morella	Castellon	Comunidad Valenciana	Spain	900
GU677011	EZSPN207-09	RVcoll.07-F056	x	*Lycaenidae*	*Lysandra*	*hispana*	10 km S. of Morella	Castellon	Comunidad Valenciana	Spain	900
KF834369		RV-07-F312	x	*Lycaenidae*	*Lysandra*	*hispana*	El Mont, Albanyà, Alt Empordà	Girona	Catalonia	Spain	860
GU676970	EZSPN252-09	RVcoll.07-W150	x	*Lycaenidae*	*Lysandra*	*hispana*	Finca Matidero, Laguarta	Huesca	Aragon	Spain	1178
HM391827	FBLMU481-09	BC ZSM Lep 27131		*Nymphalidae*	*Maniola*	*jurtina*	Oberfecking, NSG Sippenauer Moor	Niederbayern	Bavaria	Germany	350
KP870952	FBLMX211-11	BC ZSM Lep 50422		*Nymphalidae*	*Maniola*	*jurtina*	Schneizelreuth	Oberbayern	Bavaria	Germany	540
KJ994253	EZSPM713-12	RVcoll.12-L548	x	*Nymphalidae*	*Maniola*	*jurtina*	Porches, Lagoa		Algarve	Portugal	
KJ994246	EZSPM714-12	RVcoll.12-L569	x	*Nymphalidae*	*Maniola*	*jurtina*	Espiche, Lagos		Algarve	Portugal	
KJ994252	EZSPM871-12	Rvcoll.12-L620	x	*Nymphalidae*	*Maniola*	*jurtina*	Salinas, Vila Real de Santo Antonio		Algarve	Portugal	12
GU676552	EZSPN585-09	RVcoll.08-J146	x	*Nymphalidae*	*Maniola*	*jurtina*	Ponte Pedrinha, Beca, Boticas		Tras-os-Montes	Portugal	730
HQ004732	EZRMN092-08	RVcoll.08-M368		*Nymphalidae*	*Maniola*	*jurtina*	Buciumeni forest (Radacinesti)	Bacau	Moldavia	Romania	180
HQ004739	EZROM391-08	RV-06-M951		*Nymphalidae*	*Maniola*	*jurtina*	Dealul Istrita, Breaza	Buzau	Muntenia	Romania	600
HQ004737	EZROM393-08	RV-07-C987		*Nymphalidae*	*Maniola*	*jurtina*	Baile Herculane (Pecinisca)	Caras-Severin	Banat	Romania	220
HQ004734	EZROM331-08	RV-08-A009		*Nymphalidae*	*Maniola*	*jurtina*	Cluj-Napoca, dealul Feleacului, Gheorgheni forest	Cluj	Transylvania	Romania	600
HQ004738	EZROM390-08	RV-06-M859		*Nymphalidae*	*Maniola*	*jurtina*	Cluj-Napoca, dealul Feleacului, Gheorgheni forest	Cluj	Transylvania	Romania	600
HQ004736	EZROM392-08	RV-07-D474		*Nymphalidae*	*Maniola*	*jurtina*	Catcau	Cluj	Transylvania	Romania	255
HQ004733	EZRMN093-08	RVcoll.08-M456		*Nymphalidae*	*Maniola*	*jurtina*	Esechioi forest	Constanta	Dobrogea	Romania	135
HQ004731	EZRMN090-08	RVcoll.08-M226		*Nymphalidae*	*Maniola*	*jurtina*	2 Km E of Moreni	Dambovita	Muntenia	Romania	290
HQ004730	EZRMN091-08	RVcoll.08-M276		*Nymphalidae*	*Maniola*	*jurtina*	3 Km NE of Targu Jiu (around Balanesti)	Gorj	Oltenia	Romania	295
HQ004735	EZROM587-08	RV-07-C362		*Nymphalidae*	*Maniola*	*jurtina*	Sadova, pas Pascanu	Suceava	Moldavia	Romania	1100
FJ663760	LOWA864-06	2005-LOWA-864		*Nymphalidae*	*Maniola*	*jurtina*			Belgorod	Russia	150
FJ663759	LOWA865-06	2005-LOWA-865		*Nymphalidae*	*Maniola*	*jurtina*			Belgorod	Russia	150
FJ663758	LOWA866-06	2005-LOWA-866		*Nymphalidae*	*Maniola*	*jurtina*			Belgorod	Russia	150
FJ663757	LOWA867-06	2005-LOWA-867		*Nymphalidae*	*Maniola*	*jurtina*			Belgorod	Russia	150
GU676024	EZSPM081-09	RVcoll.08-P400	x	*Nymphalidae*	*Maniola*	*jurtina*	Cotillos		Cantabria	Spain	1105
GU675695	EZSPM137-09	RVcoll.08-P647	x	*Nymphalidae*	*Maniola*	*jurtina*	Valle (Valle-Zurea), Lena		Asturias	Spain	662
GU676651	EZSPN394-09	RVcoll.08-H452	x	*Nymphalidae*	*Maniola*	*jurtina*	Campo Real		Comunidad de Madrid	Spain	739
KJ994242	EZSPM905-12	Rvcoll.12-M627	x	*Nymphalidae*	*Maniola*	*jurtina*	Sotuelamos, El Bonillo	Albacete	Castilla-La Mancha	Spain	890
GU676374	EZSPN774-09	RVcoll.08-L284	x	*Nymphalidae*	*Maniola*	*jurtina*	Plano de Petracos, Castell de Castells	Alicante	Comunidad Valenciana	Spain	497
KJ994247	EZROM372-08	07-E621	x	*Nymphalidae*	*Maniola*	*jurtina*	Vallforners, Canoves	Barcelona	Catalonia	Spain	650
GU669731	EZSPC527-09	RVcoll.08-J414	x	*Nymphalidae*	*Maniola*	*jurtina*	Can Liro, St. Pere de Vilamajor, Valles Oriental	Barcelona	Catalonia	Spain	329
GU669732	EZSPC528-09	RVcoll.08-M719	x	*Nymphalidae*	*Maniola*	*jurtina*	Pobla de Lillet (bosc de Falgars)	Barcelona	Catalonia	Spain	1330
KJ994241	EZSPM1037-12	Rvcoll.12-M788	x	*Nymphalidae*	*Maniola*	*jurtina*	Carrascalejo	Caceres	Extremadura	Spain	636
GU676341	EZSPN815-09	RVcoll.08-L620	x	*Nymphalidae*	*Maniola*	*jurtina*	Algeciras, Arroyo del Prior	Cadiz	Andalusia	Spain	50
GU675885	EZSPM351-09	RVcoll.08-R093	x	*Nymphalidae*	*Maniola*	*jurtina*	Rio Tornos	Castellon	Comunidad Valenciana	Spain	845
KJ994243	EZSPM937-12	Rvcoll.12-M671	x	*Nymphalidae*	*Maniola*	*jurtina*	Rio Jandula, Mestanza	Ciudad Real	Castilla-La Mancha	Spain	407
KJ994245	EZSPM938-12	Rvcoll.12-M672	x	*Nymphalidae*	*Maniola*	*jurtina*	Rio Jandula, Mestanza	Ciudad Real	Castilla-La Mancha	Spain	407
KJ994249	EZSPM968-12	Rvcoll.12-M722	x	*Nymphalidae*	*Maniola*	*jurtina*	Alrededores San Lorenzo de Calatrava	Ciudad Real	Castilla-La Mancha	Spain	749
KJ994240	EZSPM979-12	Rvcoll.12-M741	x	*Nymphalidae*	*Maniola*	*jurtina*	Arroyo de los Molinillos, Viso del Marques	Ciudad Real	Castilla-La Mancha	Spain	742
GU676463	EZSPN682-09	RVcoll.08-L004	x	*Nymphalidae*	*Maniola*	*jurtina*	Carretera al Veleta, Gueejar Sierra	Granada	Andalusia	Spain	1266
KJ994239	EZSPM712-12	RVcoll.12-L542	x	*Nymphalidae*	*Maniola*	*jurtina*	Alrededores de Villarrasa, Villarrasa	Huelva	Andalusia	Spain	
GU676142	EZSPN1022-09	RVcoll.08-M954	x	*Nymphalidae*	*Maniola*	*jurtina*	Forcat	Huesca	Aragon	Spain	1039
KJ994248	EZSPM1044-12	Rvcoll.12-M966	x	*Nymphalidae*	*Maniola*	*jurtina*	Santa Eularia des Riu, Ibiza	Ibiza	Balearic Islands	Spain	48.2
GU675858	EZSPM380-09	RVcoll.08-J874	x	*Nymphalidae*	*Maniola*	*jurtina*	Villamoratiel de las Matas	Leon	Castilla y Leon	Spain	
KJ994244	EZROM373-08	07-E622	x	*Nymphalidae*	*Maniola*	*jurtina*	Planes del Son, Son	Lleida	Catalonia	Spain	1440
GU669733	EZSPC529-09	RVcoll.08-R264	x	*Nymphalidae*	*Maniola*	*jurtina*	Vilamos, Vall d`Aran	Lleida	Catalonia	Spain	1387
KJ994250	EZSPM770-12	RVcoll.050111LP05	x	*Nymphalidae*	*Maniola*	*jurtina*	Ruta de los Molinos, Viveiro	Lugo	Galicia	Spain	32

GenBank accession number	BOLD Process ID	Sample ID	Used in the Iberian dataset	Family	Genus	Species	Exact Site	Region	State/Province	Country	Alt. (m)
GU676103	EZSPM435-09	Rvcoll.09-X117	x	*Nymphalidae*	*Maniola*	*jurtina*	Parque Albufera des Grau, Menorca	Menorca	Balearic Islands	Spain	
GU676714	EZSPN319-09	RVcoll.08-H180	x	*Nymphalidae*	*Maniola*	*jurtina*	Llimpa, Mao	Menorca	Balearic Islands	Spain	
GU669734	EZSPC530-09	RVcoll.08-P322	x	*Nymphalidae*	*Maniola*	*jurtina*	La Riba, Alt Camp	Tarragona	Catalonia	Spain	335
GU676504	EZSPN634-09	RVcoll.08-J740	x	*Nymphalidae*	*Maniola*	*jurtina*	Casa de Camineros, Tuejar	Valencia	Comunidad Valenciana	Spain	581
KJ994251	EZSPM901-12	Rvcoll.12-M623	x	*Nymphalidae*	*Maniola*	*jurtina*	Tudela de Duero	Valladolid	Castilla y Leon	Spain	713
FJ663756	LOWA528-06	2005-LOWA-528		*Nymphalidae*	*Maniola*	*jurtina*	Karadag		Crimea	Ukraine	
HM393196	GWORA2473-09	BC ZSM Lep 30685		*Nymphalidae*	*Melanargia*	*galathea*	Neukirchen	Niederbayern	Bavaria	Germany	370
GU707177	FBLMU478-09	BC ZSM Lep 27128		*Nymphalidae*	*Melanargia*	*galathea*	Duggendorf, Girnitztal	Oberpfalz	Bavaria	Germany	375
HQ004745	EZROM395-08	RV-06-M927		*Nymphalidae*	*Melanargia*	*galathea*	Dealul Istrita, Breaza	Buzau	Muntenia	Romania	600
HQ004747	EZROM396-08	RV-06-M935		*Nymphalidae*	*Melanargia*	*galathea*	Dealul Istrita, Breaza	Buzau	Muntenia	Romania	600
HQ004744	EZROM398-08	RV-07-C971		*Nymphalidae*	*Melanargia*	*galathea*	Baile Herculane (Pecinisca)	Caras-Severin	Banat	Romania	220
HQ004746	EZROM394-08	RV-06-M861		*Nymphalidae*	*Melanargia*	*galathea*	Cluj-Napoca, dealul Feleacului, Gheorgheni forest	Cluj	Transylvania	Romania	600
HQ004743	EZROM397-08	RV-07-D440		*Nymphalidae*	*Melanargia*	*galathea*	Suatu	Cluj	Transylvania	Romania	380
HQ004741	EZRMN096-08	RVcoll.08-M421		*Nymphalidae*	*Melanargia*	*galathea*	1 Km NE of Gura Dobrogei reserve	Constanta	Dobrogea	Romania	40
HQ004742	EZRMN094-08	RVcoll.07-C141		*Nymphalidae*	*Melanargia*	*galathea*	Stana	Salaj	Transylvania	Romania	500
HQ004740	EZRMN095-08	RVcoll.08-M394		*Nymphalidae*	*Melanargia*	*galathea*	Macin Mts. (3 Km S of Jijila)	Tulcea	Dobrogea	Romania	230
KP871135	EZSPM816-12	RVcoll.130711PX78	x	*Nymphalidae*	*Melanargia*	*galathea*	Fondos de Vega, Degana		Asturias	Spain	1079
KP870648	EZSPM846-12	RVcoll.150511SD04	x	*Nymphalidae*	*Melanargia*	*galathea*	Fondos de Vega, Degana		Asturias	Spain	1079
GU676263	EZSPN897-09	RVcoll.08-L809	x	*Nymphalidae*	*Melanargia*	*galathea*	Hormiguera		Cantabria	Spain	968
GU676264	EZSPN898-09	RVcoll.08-L810	x	*Nymphalidae*	*Melanargia*	*galathea*	Hormiguera		Cantabria	Spain	968
GU676255	EZSPN899-09	RVcoll.08-L811	x	*Nymphalidae*	*Melanargia*	*galathea*	Hormiguera		Cantabria	Spain	968
HM901440	EZSPC1244-10	RVcoll.09-V771	x	*Nymphalidae*	*Melanargia*	*galathea*	Embalse La Sarra, Sallent de Gallego	Huesca	Aragon	Spain	1410
HM901443	EZSPC1248-10	RVcoll.09-V793	x	*Nymphalidae*	*Melanargia*	*galathea*	Pueto de Monrepos	Huesca	Aragon	Spain	1280
GU676145	EZSPN1019-09	RVcoll.08-M951.1	x	*Nymphalidae*	*Melanargia*	*galathea*	Forcat	Huesca	Aragon	Spain	1039
GU675922	EZSPM314-09	RVcoll.08-J869	x	*Nymphalidae*	*Melanargia*	*galathea*	Posada de Valdeon	Leon	Castilla y Leon	Spain	
HM901368	EZSPC1168-10	RVcoll.09-T073	x	*Nymphalidae*	*Melanargia*	*galathea*	Sant Joan de Toran, Vall d`Aran	Lleida	Catalonia	Spain	980
KP870229	EZSPC531-09	RVcoll.08-L485	x	*Nymphalidae*	*Melanargia*	*galathea*	Bassa d`Arres, Vall d`Aran	Lleida	Catalonia	Spain	1501
KP870833	EZSPC532-09	RVcoll.08-L491	x	*Nymphalidae*	*Melanargia*	*galathea*	Bassa d`Arres, Vall d`Aran	Lleida	Catalonia	Spain	1501
KP870287	EZSPC533-09	RVcoll.08-P373	x	*Nymphalidae*	*Melanargia*	*galathea*	Arros, Vall d`Aran	Lleida	Catalonia	Spain	906
GU669728	EZSPC534-09	RVcoll.08-P374	x	*Nymphalidae*	*Melanargia*	*galathea*	Arros, Vall d`Aran	Lleida	Catalonia	Spain	906
FJ663762	LOWA526-06	2005-LOWA-526		*Nymphalidae*	*Melanargia*	*galathea*	Karadag		Crimea	Ukraine	
FJ663761	LOWA527-06	2005-LOWA-527		*Nymphalidae*	*Melanargia*	*galathea*	Karadag		Crimea	Ukraine	
KP870674	EZSPM715-12	RVcoll.12-L559	x	*Nymphalidae*	*Melanargia*	*ines*	Porches, Lagoa		Algarve	Portugal	
KP870800	EZSPM716-12	RVcoll.12-L560	x	*Nymphalidae*	*Melanargia*	*ines*	Porches, Lagoa		Algarve	Portugal	
KP871030	EZSPM717-12	RVcoll.12-L561	x	*Nymphalidae*	*Melanargia*	*ines*	Porches, Lagoa		Algarve	Portugal	
KP870865	EZSPM609-12	RVcoll.11-D680	x	*Nymphalidae*	*Melanargia*	*ines*	El Campello	Alicante	Comunidad Valenciana	Spain	100
KP870467	EZSPM780-12	RVcoll.070111GN66	x	*Nymphalidae*	*Melanargia*	*ines*	El Montgo _ Nivel Medio, Xavia	Alicante	Comunidad Valenciana	Spain	161
HM901329	EZSPC1129-10	RVcoll.08-P700	x	*Nymphalidae*	*Melanargia*	*ines*	Almaraz	Caceres	Extremadura	Spain	290
HM901330	EZSPC1130-10	RVcoll.08-P704	x	*Nymphalidae*	*Melanargia*	*ines*	Romangordo	Caceres	Extremadura	Spain	395
HM901331	EZSPC1131-10	RVcoll.08-P705	x	*Nymphalidae*	*Melanargia*	*ines*	Romangordo	Caceres	Extremadura	Spain	395
GU676927	EZSPN523-09	RVcoll.08-H993	x	*Nymphalidae*	*Melanargia*	*ines*	Plasencia	Caceres	Extremadura	Spain	400
GU676610	EZSPN438-09	RVcoll.08-H620	x	*Nymphalidae*	*Melanargia*	*ines*	El Gastor	Cadiz	Andalusia	Spain	660
GU676588	EZSPN453-09	RVcoll.08-H657	x	*Nymphalidae*	*Melanargia*	*ines*	Ubrique, Puerto de la Vibora	Cadiz	Andalusia	Spain	405
GU676526	EZSPN620-09	RVcoll.08-J702	x	*Nymphalidae*	*Melanargia*	*ines*	Alrededores de Almedijar, Almedijar	Castellon	Comunidad Valenciana	Spain	
KP870795	EZSPM975-12	Rvcoll.12-M733	x	*Nymphalidae*	*Melanargia*	*ines*	Arroyo de los Molinillos, Viso del Marques	Ciudad Real	Castilla-La Mancha	Spain	742
HM901338	EZSPC1138-10	RVcoll.08-P727	x	*Nymphalidae*	*Melanargia*	*ines*	Aldeire	Granada	Andalusia	Spain	1335
HM901807	EZSPC972-10	RVcoll.06-A002	x	*Nymphalidae*	*Melanargia*	*ines*	Road from Ferreira to Puerto de La Ragua	Granada	Andalusia	Spain	1600
GU676437	EZSPN710-09	RVcoll.08-L062	x	*Nymphalidae*	*Melanargia*	*ines*	Venta del Molinillo, Huetor de Santillan	Granada	Andalusia	Spain	1236
HM901333	EZSPC1133-10	RVcoll.08-P711	x	*Nymphalidae*	*Melanargia*	*ines*	La Mata	Toledo	Castilla-La Mancha	Spain	524
GU676564	EZSPN573-09	RVcoll.08-J120	x	*Nymphalidae*	*Melanargia*	*lachesis*	Quinta de Pentieiros, Bertiandos, Ponte de Lima		Minho	Portugal	20

GenBank accession number	BOLD Process ID	Sample ID	Used in the Iberian dataset	Family	Genus	Species	Exact Site	Region	State/Province	Country	Alt. (m)
GU676546	EZSPN591-09	RVcoll.08-J168	x	*Nymphalidae*	*Melanargia*	*lachesis*	Pedras Negras (Sa Larouco), Padornelos, Montalegre		Tras-os-Montes	Portugal	1340
JN278873	EZSPN1039-11	Rvcoll.08-H335.1	x	*Nymphalidae*	*Melanargia*	*lachesis*	Puerto de Morcuera, Miraflores		Comunidad de Madrid	Spain	
GU676317	EZSPN840-09	RVcoll.08-L668	x	*Nymphalidae*	*Melanargia*	*lachesis*	Candeleda, Cuesta de la Joya	Avila	Castilla y Leon	Spain	415
GU676789	EZSPN150-09	RVcoll.06-G570	x	*Nymphalidae*	*Melanargia*	*lachesis*	La Sala, El Brull, Osona	Barcelona	Catalonia	Spain	800
KP871123	EZSPM1020-12	Rvcoll.12-M769	x	*Nymphalidae*	*Melanargia*	*lachesis*	7 km SE Navalvillar de Ibor	Caceres	Extremadura	Spain	729
KP870844	EZSPM1026-12	Rvcoll.12-M776	x	*Nymphalidae*	*Melanargia*	*lachesis*	Hospital del Obispo, Navalvillar de Ibor	Caceres	Extremadura	Spain	1010
KP871022	EZSPM1031-12	Rvcoll.12-M781	x	*Nymphalidae*	*Melanargia*	*lachesis*	Merendero de los Horcones, Navatrasierra	Caceres	Extremadura	Spain	664
GU676894	EZSPN491-09	RVcoll.08-H928	x	*Nymphalidae*	*Melanargia*	*lachesis*	Hervas, Carretera Hervas-La Garganta	Caceres	Extremadura	Spain	940
GU676100	EZSPM443-09	Rvcoll.09-V470	x	*Nymphalidae*	*Melanargia*	*lachesis*	Puerto de la Ragua (Sierra Nevada)	Granada	Andalusia	Spain	2090
GU676101	EZSPM444-09	Rvcoll.09-V935	x	*Nymphalidae*	*Melanargia*	*lachesis*	Sierra de la Sagra	Granada	Andalusia	Spain	1745
GU676420	EZSPN725-09	RVcoll.08-L131	x	*Nymphalidae*	*Melanargia*	*lachesis*	Casillas de Rojas, Gueejar Sierra	Granada	Andalusia	Spain	1733
GU676189	EZSPN977-09	RVcoll.08-M009	x	*Nymphalidae*	*Melanargia*	*lachesis*	Gualda, La Alcarria	Guadalajara	Castilla-La Mancha	Spain	730
GU676143	EZSPN1017-09	RVcoll.08-M942	x	*Nymphalidae*	*Melanargia*	*lachesis*	Sobrecastells	Huesca	Aragon	Spain	686
HM901519	EZSPC1385-10	RVcoll.09-X535	x	*Nymphalidae*	*Melanargia*	*lachesis*	Buron	Leon	Castilla y Leon	Spain	1120
GU675733	EZSPM156-09	RVcoll.08-P682	x	*Nymphalidae*	*Melanargia*	*lachesis*	Villadangos de Arbas	Leon	Castilla y Leon	Spain	1311
GU675917	EZSPM317-09	RVcoll.08-J878	x	*Nymphalidae*	*Melanargia*	*lachesis*	Villanueva de las Manzanas	Leon	Castilla y Leon	Spain	
GU675831	EZSPM256-09	RVcoll.08-R469	x	*Nymphalidae*	*Melanargia*	*lachesis*	Celanova, Vieiro de Abaixo	Ourense	Galicia	Spain	817
GU675834	EZSPM255-09	RVcoll.08-R468	x	*Nymphalidae*	*Melanargia*	*lachesis*	Tui, Rio Penedo	Pontevedra	Galicia	Spain	40
GU676302	EZSPN849-09	RVcoll.08-L693	x	*Nymphalidae*	*Melanargia*	*lachesis*	Candelario	Salamanca	Castilla y Leon	Spain	1300
JN278889	EZSPN1101-11	Rvcoll.10-A840	x	*Nymphalidae*	*Melanargia*	*lachesis*	Roquetes-Mont Caro, Ports de Tortosa	Tarragona	Catalonia	Spain	720
GU676678	EZSPN362-09	RVcoll.08-H344	x	*Nymphalidae*	*Melanargia*	*lachesis*	Sierra de Javalambre, Alpuente	Valencia	Comunidad Valenciana	Spain	
KP870481	EZSPM902-12	Rvcoll.12-M624	x	*Nymphalidae*	*Melanargia*	*lachesis*	Tudela de Duero	Valladolid	Castilla y Leon	Spain	713
KP870729	EZSPM903-12	Rvcoll.12-M625	x	*Nymphalidae*	*Melanargia*	*lachesis*	Tudela de Duero	Valladolid	Castilla y Leon	Spain	713
HM901213	EZSPC1009-10	RVcoll.08-H486	x	*Nymphalidae*	*Melanargia*	*occitanica*	Colmenar Viejo		Comunidad de Madrid	Spain	847
JN278867	EZSPC1342-10	RVcoll.130209KL47	x	*Nymphalidae*	*Melanargia*	*occitanica*	Fortuna,Fortuna		Murcia	Spain	
GU676650	EZSPN393-09	RVcoll.08-H451	x	*Nymphalidae*	*Melanargia*	*occitanica*	Campo Real		Comunidad de Madrid	Spain	736
GU676288	EZSPN867-09	RVcoll.08-L742	x	*Nymphalidae*	*Melanargia*	*occitanica*	Puerto de la Morcuera		Comunidad de Madrid	Spain	1438
GU676518	EZSPN624-09	RVcoll.08-J708	x	*Nymphalidae*	*Melanargia*	*occitanica*	Alrededores de Almedijar, Almedijar	Castellon	Comunidad Valenciana	Spain	
KP870609	EZSPM974-12	Rvcoll.12-M732	x	*Nymphalidae*	*Melanargia*	*occitanica*	Arroyo de los Molinillos, Viso del Marques	Ciudad Real	Castilla-La Mancha	Spain	742
HM901336	EZSPC1136-10	RVcoll.08-P723	x	*Nymphalidae*	*Melanargia*	*occitanica*	La Calahorra	Granada	Andalusia	Spain	1255
GU676804	EZSPN132-09	RVcoll.06-G480	x	*Nymphalidae*	*Melanargia*	*occitanica*	Barranco de Valcuerna, Candasnos	Huesca	Aragon	Spain	210
GU675918	EZSPM318-09	RVcoll.08-J883	x	*Nymphalidae*	*Melanargia*	*occitanica*	El Burgo Ranero	Leon	Castilla y Leon	Spain	
GU675836	EZSPM257-09	RVcoll.08-R470	x	*Nymphalidae*	*Melanargia*	*occitanica*	Lobios, Camino de Sa a Portela de Homen	Ourense	Galicia	Spain	1100
GU677015	EZSPN202-09	RVcoll.07-C695	x	*Nymphalidae*	*Melanargia*	*occitanica*	Collet de la Tina, La Mussara, Prades	Tarragona	Catalonia	Spain	
GU676695	EZSPN339-09	RVcoll.08-H288	x	*Nymphalidae*	*Melanargia*	*occitanica*	Els Motllats, Vilaplana, Baix Camp	Tarragona	Catalonia	Spain	980
GU676677	EZSPN361-09	RVcoll.08-H343	x	*Nymphalidae*	*Melanargia*	*occitanica*	Sierra de Javalambre, Alpuente	Valencia	Comunidad Valenciana	Spain	
HM901387	EZSPC1187-10	RVcoll.09-T206	x	*Nymphalidae*	*Melanargia*	*occitanica*	Barranco de Valcuerna, Candasnos	Zaragoza	Aragon	Spain	210
HM901237	EZSPC1033-10	RVcoll.08-J176	x	*Nymphalidae*	*Melanargia*	*russiae*	Serra do Larouco (topo), Padornelos, Montalegre		Tras-os-Montes	Portugal	1520
GU676566	EZSPN575-09	RVcoll.08-J122	x	*Nymphalidae*	*Melanargia*	*russiae*	Leiranco, Bobadela, Boticas		Tras-os-Montes	Portugal	1150
HM901286	EZSPC1085-10	RVcoll.08-L751	x	*Nymphalidae*	*Melanargia*	*russiae*	Oteruelo del Valle		Comunidad de Madrid	Spain	1138
HM901383	EZSPC1183-10	RVcoll.09-T162	x	*Nymphalidae*	*Melanargia*	*russiae*	La Hiruela		Comunidad de Madrid	Spain	1312
GU676287	EZSPN866-09	RVcoll.08-L740	x	*Nymphalidae*	*Melanargia*	*russiae*	Puerto de la Morcuera		Comunidad de Madrid	Spain	1438
GU676280	EZSPN875-09	RVcoll.08-L768	x	*Nymphalidae*	*Melanargia*	*russiae*	Puerto de Pozazal		Cantabria	Spain	1030
GU676262	EZSPN896-09	RVcoll.08-L808	x	*Nymphalidae*	*Melanargia*	*russiae*	Hormiguera		Cantabria	Spain	966
GU677036	EZSPN179-09	RVcoll.06-V743	x	*Nymphalidae*	*Melanargia*	*russiae*	Serra Pedregosa, Cadi, Saldes, Bergueda	Barcelona	Catalonia	Spain	2025

GenBank accession number	BOLD Process ID	Sample ID	Used in the Iberian dataset	Family	Genus	Species	Exact Site	Region	State/Province	Country	Alt. (m)
GU676398	EZSPN747-09	RVcoll.08-L185	x	*Nymphalidae*	*Melanargia*	*russiae*	Font del Regatxal, Vilafranca	Castellon	Comunidad Valenciana	Spain	1361
HM901316	EZSPC1115-10	RVcoll.08-P022	x	*Nymphalidae*	*Melanargia*	*russiae*	Talltendre, Cerdanya	Girona	Catalonia	Spain	1571
GU676037	EZSPM019-09	RVcoll.08-P042	x	*Nymphalidae*	*Melanargia*	*russiae*	La Valira (near Urus), Cerdanya	Girona	Catalonia	Spain	1300
HM901346	EZSPC1146-10	RVcoll.08-P764	x	*Nymphalidae*	*Melanargia*	*russiae*	Acevedo	Leon	Castilla y Leon	Spain	1364
GU675736	EZSPM157-09	RVcoll.08-P683	x	*Nymphalidae*	*Melanargia*	*russiae*	Villadangos de Arbas	Leon	Castilla y Leon	Spain	1311
GU675932	EZSPM302-09	RVcoll.08-J849	x	*Nymphalidae*	*Melanargia*	*russiae*	Cremenes	Leon	Castilla y Leon	Spain	
HM901358	EZSPC1158-10	RVcoll.08-R310	x	*Nymphalidae*	*Melanargia*	*russiae*	Planes de Son, Pallars Sobira	Lleida	Catalonia	Spain	1589.7
GU675833	EZSPM203-09	RVcoll.08-R311	x	*Nymphalidae*	*Melanargia*	*russiae*	Planes de Son, Pallars Sobira	Lleida	Catalonia	Spain	1590
KP871014	EULEP242-14	12-N702	x	*Nymphalidae*	*Melanargia*	*russiae*	El Valecillo	Teruel	Aragón	Spain	1526.7
GU676598	EZSPN451-09	RVcoll.08-H653	x	*Nymphalidae*	*Melitaea*	*aetherie*	Ubrique, Puerto de la Vibora	Cadiz	Andalusia	Spain	405
GU676587	EZSPN452-09	RVcoll.08-H654	x	*Nymphalidae*	*Melitaea*	*aetherie*	Ubrique, Puerto de la Vibora	Cadiz	Andalusia	Spain	405
KP870738	EZSPM718-12	RVcoll.12-L022	x	*Nymphalidae*	*Melitaea*	*aetherie*	La Ribera, Huelva	Huelva	Andalusia	Spain	7
KP870463	EZSPM719-12	RVcoll.12-L023	x	*Nymphalidae*	*Melitaea*	*aetherie*	La Ribera, Huelva	Huelva	Andalusia	Spain	7
KP871087	EZSPM720-12	RVcoll.12-L024	x	*Nymphalidae*	*Melitaea*	*aetherie*	La Ribera, Huelva	Huelva	Andalusia	Spain	7
KP871043	EZSPM721-12	RVcoll.12-L048	x	*Nymphalidae*	*Melitaea*	*aetherie*	La Ribera, Huelva	Huelva	Andalusia	Spain	7
GU676006	EZSPM027-09	RVcoll.08-P214	x	*Nymphalidae*	*Melitaea*	*athalia*	Courbassil	Languedoc-Roussillon		France	1390
GU655013	FBLMT898-09	BC ZSM Lep 25458		*Nymphalidae*	*Melitaea*	*athalia*	Nittendorf	Oberpfalz	Bavaria	Germany	400
GU707188	FBLMU119-09	BC ZSM Lep 25629		*Nymphalidae*	*Melitaea*	*athalia*	Kallmuenz, Kirchenberg	Oberpfalz	Bavaria	Germany	390
KP870791	FBLMX265-11	BC ZSM Lep 50476		*Nymphalidae*	*Melitaea*	*athalia*	Bodenwoehr, Hetschenlach	Oberpfalz	Bavaria	Germany	380
JF860020	PHLAC444-10	TLMF Lep 02479		*Nymphalidae*	*Melitaea*	*athalia*	Ritten/ Obergruenwald		South Tyrol	Italy	1750
GU676562	EZSPN583-09	RVcoll.08-J143	x	*Nymphalidae*	*Melitaea*	*athalia*	Ponte Pedrinha, Beca, Boticas		Tras-os-Montes	Portugal	730
HQ004751	EZRMN372-08	RVcoll.08-M103		*Nymphalidae*	*Melitaea*	*athalia*	Oraseni Vale (forest)	Botosani	Moldavia	Romania	160
HQ004752	EZRMN373-08	RVcoll.08-M104		*Nymphalidae*	*Melitaea*	*athalia*	Oraseni Vale (forest)	Botosani	Moldavia	Romania	160
HQ004753	EZRMN374-08	RVcoll.08-M105		*Nymphalidae*	*Melitaea*	*athalia*	Oraseni Vale (forest)	Botosani	Moldavia	Romania	160
HQ004754	EZROM693-08	RV-07-C312		*Nymphalidae*	*Melitaea*	*athalia*	Oraseni Vale	Botosani	Moldavia	Romania	150
HQ004755	EZROM694-08	RV-07-C314		*Nymphalidae*	*Melitaea*	*athalia*	Oraseni Vale	Botosani	Moldavia	Romania	150
HQ004759	EZROM404-08	RV-07-D295		*Nymphalidae*	*Melitaea*	*athalia*	Dumbrava Vadului, Vad	Brasov	Transylvania	Romania	495
HQ004757	EZROM402-08	RV-06-K602		*Nymphalidae*	*Melitaea*	*athalia*	Suatu	Cluj	Transylvania	Romania	380
HQ004758	EZROM403-08	RV-06-M839		*Nymphalidae*	*Melitaea*	*athalia*	Cluj-Napoca, valea paraului Micus	Cluj	Transylvania	Romania	700
HQ004748	EZRMN099-08	RVcoll.08-M222		*Nymphalidae*	*Melitaea*	*athalia*	2 Km E of Moreni	Dambovita	Muntenia	Romania	290
HQ004760	EZROM405-08	RV-07-D976		*Nymphalidae*	*Melitaea*	*athalia*	4 km west of I.L. Caragiale	Dambovita	Muntenia	Romania	270
HQ004756	EZROM406-08	RV-07-E394		*Nymphalidae*	*Melitaea*	*athalia*	Picior de Munte	Dambovita	Muntenia	Romania	260
HQ004749	EZRMN100-08	RVcoll.08-M346		*Nymphalidae*	*Melitaea*	*athalia*	1 Km E of Gheorgheni	Harghita	Transylvania	Romania	900
HQ004750	EZRMN101-08	RVcoll.08-M597		*Nymphalidae*	*Melitaea*	*athalia*	Gheorgheni, Valea Belchia	Harghita	Transylvania	Romania	1000
FJ663813	LOWA290-06	2005-LOWA-290		*Nymphalidae*	*Melitaea*	*athalia*	Osyno	Sebezh distr.	Pskov	Russia	
FJ663812	LOWA291-06	2005-LOWA-291		*Nymphalidae*	*Melitaea*	*athalia*	Osyno	Sebezh distr.	Pskov	Russia	
GU676244	EZSPN913-09	RVcoll.08-L852	x	*Nymphalidae*	*Melitaea*	*athalia*	Puerto de la Morcuera		Comunidad de Madrid	Spain	1420
KP870439	EZSPC1423-10	RVcoll.09-V134	x	*Nymphalidae*	*Melitaea*	*athalia*	Sierra de Gador (Fondon)	Almeria	Andalusia	Spain	1950
GU676878	EZSPN473-09	RVcoll.08-H904	x	*Nymphalidae*	*Melitaea*	*athalia*	Hervas, Carretera Hervas-La Garganta	Caceres	Extremadura	Spain	1007
GU676899	EZSPN494-09	RVcoll.08-H935	x	*Nymphalidae*	*Melitaea*	*athalia*	Hervas, Carretera Hervas-La Garganta	Caceres	Extremadura	Spain	1007
HM901454	EZSPC1269-10	RVcoll.210907WR65	x	*Nymphalidae*	*Melitaea*	*athalia*	Moli de Joaquim,La Pobla de Benifassa	Castellon	Comunidad Valenciana	Spain	971
KP870776	EZROM734-08	RV-07-C071	x	*Nymphalidae*	*Melitaea*	*athalia*	Puig Neulos	Girona	Catalonia	Spain	1110
GU669726	EZSPC539-09	RVcoll.07-C635	x	*Nymphalidae*	*Melitaea*	*athalia*	Meranges	Girona	Catalonia	Spain	1555
GU669727	EZSPC540-09	RVcoll.07-Z072	x	*Nymphalidae*	*Melitaea*	*athalia*	Viladrau, Osona	Girona	Catalonia	Spain	750
GU669707	EZSPC560-09	RVcoll.07-C680	x	*Nymphalidae*	*Melitaea*	*athalia*	Meranges	Girona	Catalonia	Spain	1555
HM901259	EZSPC1056-10	RVcoll.08-J954	x	*Nymphalidae*	*Melitaea*	*athalia*	Fuente Albergue de San Francisco, Gueejar Sierra	Granada	Andalusia	Spain	2221
HM901538	EZSPC1424-10	RVcoll.09-V135	x	*Nymphalidae*	*Melitaea*	*athalia*	Trevelez, N. Alpujarras, Sierra Nevada	Granada	Andalusia	Spain	1900
HM901539	EZSPC1425-10	RVcoll.09-V136	x	*Nymphalidae*	*Melitaea*	*athalia*	Trevelez, N. Alpujarras, Sierra Nevada	Granada	Andalusia	Spain	1900
HM901540	EZSPC1426-10	RVcoll.09-V137	x	*Nymphalidae*	*Melitaea*	*athalia*	Trevelez, N. Alpujarras, Sierra Nevada	Granada	Andalusia	Spain	1900
KP871044	EZSPM601-12	RVcoll.11-I433	x	*Nymphalidae*	*Melitaea*	*athalia*	3 km S Gueejar Sierra (road to Veleta, Sierra Nevada)	Granada	Andalusia	Spain	1565
KP870246	EZSPM660-12	RVcoll.11-I507	x	*Nymphalidae*	*Melitaea*	*athalia*	SE of Cumbres Verdes (La Zubia)	Granada	Andalusia	Spain	1400

GenBank accession number	BOLD Process ID	Sample ID	Used in the Iberian dataset	Family	Genus	Species	Exact Site	Region	State/Province	Country	Alt. (m)
GU676161	EZSPN997-09	RVcoll.08-M074	x	*Nymphalidae*	*Melitaea*	*athalia*	San Andres del Rey, La Alcarria	Guadalajara	Castilla-La Mancha	Spain	1018
GU675930	EZSPM304-09	RVcoll.08-J851	x	*Nymphalidae*	*Melitaea*	*athalia*	Posada de Valdeon	Leon	Castilla y Leon	Spain	
KP870866	EZSPC207-09	RVcoll.08-P394	x	*Nymphalidae*	*Melitaea*	*athalia*	Salt de Pitx, Vall d`Aran	Lleida	Catalonia	Spain	1570
KP870367	EZSPC257-09	RVcoll.08-P395	x	*Nymphalidae*	*Melitaea*	*athalia*	Salt de Pitx, Vall d`Aran	Lleida	Catalonia	Spain	1570
GU676148	EZSPN1010-09	RVcoll.08-M915	x	*Nymphalidae*	*Melitaea*	*athalia*	Bassa d`Arres, Vall d`Aran	Lleida	Catalonia	Spain	1500
JN274545	EZSPC1273-10	RVcoll.220602AM3	x	*Nymphalidae*	*Melitaea*	*athalia*	Abejuela,Abejuela	Teruel	Aragon	Spain	
HM901470	EZSPC1298-10	RVcoll.100908AW26	x	*Nymphalidae*	*Melitaea*	*athalia*	Barranco del Regajo,Castielfabib	Valencia	Comunidad Valenciana	Spain	1250
HQ004763	EZRMN105-08	RVcoll.08-M280		*Nymphalidae*	*Melitaea*	*aurelia*	3 Km W of Maierus	Brasov	Transylvania	Romania	550
HQ004768	EZROM408-08	RV-06-M931		*Nymphalidae*	*Melitaea*	*aurelia*	Dealul Istrita, Breaza	Buzau	Muntenia	Romania	600
HQ004769	EZROM409-08	RV-06-M961		*Nymphalidae*	*Melitaea*	*aurelia*	Dealul Istrita, Breaza	Buzau	Muntenia	Romania	600
HQ004771	EZROM411-08	RV-07-C980		*Nymphalidae*	*Melitaea*	*aurelia*	Baile Herculane (Pecinisca)	Caras-Severin	Banat	Romania	220
HQ004766	EZRMN102-08	RVcoll.06-M825		*Nymphalidae*	*Melitaea*	*aurelia*	Cluj-Napoca, dealul Feleacului, Gheorgheni forest	Cluj	Transylvania	Romania	600
HQ004764	EZRMN104-08	RVcoll.07-D411		*Nymphalidae*	*Melitaea*	*aurelia*	Suatu	Cluj	Transylvania	Romania	380
HQ004762	EZRMN333-08	RVcoll.08-H005		*Nymphalidae*	*Melitaea*	*aurelia*	Cluj-Napoca, dealul Bogomaia, Fanatele Clujului	Cluj	Transylvania	Romania	450
HQ004761	EZRMN334-08	RVcoll.08-H006		*Nymphalidae*	*Melitaea*	*aurelia*	Cluj-Napoca, dealul Bogomaia, Fanatele Clujului	Cluj	Transylvania	Romania	450
HQ004767	EZROM407-08	RV-06-K676		*Nymphalidae*	*Melitaea*	*aurelia*	Valea Calda (Cluj-Napoca)	Cluj	Transylvania	Romania	380
HQ004770	EZROM410-08	RV-07-D421		*Nymphalidae*	*Melitaea*	*aurelia*	Suatu	Cluj	Transylvania	Romania	380
HQ004765	EZRMN103-08	RVcoll.07-D387		*Nymphalidae*	*Melitaea*	*aurelia*	Iernut	Mures	Transylvania	Romania	355
GU707041	FBLMU265-09	BC ZSM Lep 25775		*Nymphalidae*	*Melitaea*	*britomartis*	Bad Abbach, Oberndorf	Niederbayern	Bavaria	Germany	380
HQ565472	GWOSA741-10	BC ZSM Lep 35752		*Nymphalidae*	*Melitaea*	*britomartis*	Regensburg - Kallmuenz	Oberpfalz	Bavaria	Germany	
HQ004772	EZRMN370-08	RVcoll.08-M100		*Nymphalidae*	*Melitaea*	*britomartis*	Oraseni Vale (forest)	Botosani	Moldavia	Romania	160
HQ004773	EZROM588-08	RV-07-C313		*Nymphalidae*	*Melitaea*	*britomartis*	Oraseni Vale	Botosani	Moldavia	Romania	150
KP870522	EULEP088-14	08-P974		*Nymphalidae*	*Melitaea*	*britomartis*	Gheorgheni, Dealul Feleacului, Cluj-Napoca	Cluj	Transylvania	Romania	
JF415713	GWORK317-09	BC ZSM Lep 21552		*Nymphalidae*	*Melitaea*	*cinxia*	Koenigsdorf, Weitfilz	Oberbayern	Bavaria	Germany	600
JF415714	GWORR677-10	BC ZSM Lep 32173		*Nymphalidae*	*Melitaea*	*cinxia*	Lindenrain, Geretsried	Oberbayern	Bavaria	Germany	610
GU655011	FBLMT871-09	BC ZSM Lep 25431		*Nymphalidae*	*Melitaea*	*cinxia*	Duggendorf, Girnitztal	Oberpfalz	Bavaria	Germany	375
HQ004775	EZRMN107-08	RVcoll.07-D288		*Nymphalidae*	*Melitaea*	*cinxia*	Dumbrava Vadului, Vad	Brasov	Transylvania	Romania	495
HQ004778	EZROM639-08	RV-07-D319		*Nymphalidae*	*Melitaea*	*cinxia*	Dumbrava Vadului, Vad	Brasov	Transylvania	Romania	495
HQ004779	EZROM163-08	RV-07-C981		*Nymphalidae*	*Melitaea*	*cinxia*	Baile Herculane (Pecinisca)	Caras-Severin	Banat	Romania	270
KP870805	EULEP040-14	08-H003		*Nymphalidae*	*Melitaea*	*cinxia*	Cluj-Napoca, Fânațe	Cluj	Transylvania	Romania	
HQ004776	EZRMN108-08	RVcoll.08-M308		*Nymphalidae*	*Melitaea*	*cinxia*	Suatu	Cluj	Transylvania	Romania	400
HQ004781	EZROM413-08	RV-07-D473		*Nymphalidae*	*Melitaea*	*cinxia*	Catcau	Cluj	Transylvania	Romania	255
HQ004780	EZROM412-08	RV-07-D164		*Nymphalidae*	*Melitaea*	*cinxia*	Dumbraveni forest	Constanta	Dobrogea	Romania	80
HQ004777	EZRMN109-08	RVcoll.08-M359		*Nymphalidae*	*Melitaea*	*cinxia*	Cheile Bicazului	Harghita	Transylvania	Romania	940
HQ004774	EZRMN106-08	RVcoll.07-D040		*Nymphalidae*	*Melitaea*	*cinxia*	8 Km S of Babadag (Babadag forest)	Tulcea	Dobrogea	Romania	115
KP870506	EZSPM804-12	RVcoll.090211SD72	x	*Nymphalidae*	*Melitaea*	*cinxia*	Sierra Espuna, Totana		Murcia	Spain	1411
GU676283	EZSPN870-09	RVcoll.08-L753	x	*Nymphalidae*	*Melitaea*	*cinxia*	Oteruelo del Valle		Comunidad de Madrid	Spain	1152
GU676273	EZSPN884-09	RVcoll.08-L793	x	*Nymphalidae*	*Melitaea*	*cinxia*	Brana Vieja		Cantabria	Spain	1408
JN274546	EZSPC1308-10	RVcoll.130209KL31	x	*Nymphalidae*	*Melitaea*	*cinxia*	Pto. de las Crucetillas,Riopar	Albacete	Castilla-La Mancha	Spain	
HM901487	EZSPC1344-10	RVcoll.130209KL22	x	*Nymphalidae*	*Melitaea*	*cinxia*	Sa Maria	Almeria	Andalusia	Spain	
GU676321	EZSPN833-09	RVcoll.08-L654	x	*Nymphalidae*	*Melitaea*	*cinxia*	San Martin del Pimpollar, Piscifactoria Rasquilla	Avila	Castilla y Leon	Spain	1270
KP870841	EZROM735-08	RV-06-G454	x	*Nymphalidae*	*Melitaea*	*cinxia*	El Sola, El Brull	Barcelona	Catalonia	Spain	800
GU669720	EZSPC541-09	RVcoll.06-H880	x	*Nymphalidae*	*Melitaea*	*cinxia*	El Polell, P.N. del Montseny	Barcelona	Catalonia	Spain	780
GU669721	EZSPC542-09	RVcoll.07-C629	x	*Nymphalidae*	*Melitaea*	*cinxia*	Meranges	Girona	Catalonia	Spain	1555
GU669722	EZSPC543-09	RVcoll.08-J388	x	*Nymphalidae*	*Melitaea*	*cinxia*	Estanys de la Jonquera, La Jonquera, Alt Emporda	Girona	Catalonia	Spain	180
GU676178	EZSPN978-09	RVcoll.08-M012	x	*Nymphalidae*	*Melitaea*	*cinxia*	Gualda, La Alcarria	Guadalajara	Castilla-La Mancha	Spain	730
GU669723	EZSPC544-09	RVcoll.08-L497	x	*Nymphalidae*	*Melitaea*	*cinxia*	Bassa d`Arres, Vall d`Aran	Lleida	Catalonia	Spain	1501
GU676904	EZSPN501-09	RVcoll.08-H951	x	*Nymphalidae*	*Melitaea*	*cinxia*	Candelario, Sierra de Candelario	Salamanca	Castilla y Leon	Spain	1300
GU676932	EZSPN530-09	RVcoll.08-J000	x	*Nymphalidae*	*Melitaea*	*cinxia*	Requena, Requena	Valencia	Comunidad Valenciana	Spain	

GenBank accession number	BOLD Process ID	Sample ID	Used in the Iberian dataset	Family	Genus	Species	Exact Site	Region	State/Province	Country	Alt. (m)
GU675908	EZSPM330-09	RVcoll.08-R055	x	*Nymphalidae*	*Melitaea*	*deione*	Barrancada	Alicante	Comunidad Valenciana	Spain	817
GU676803	EZSPN131-09	RVcoll.06-G472	x	*Nymphalidae*	*Melitaea*	*deione*	La Trinitat, El Brull, Osona	Barcelona	Catalonia	Spain	880
HM901455	EZSPC1270-10	RVcoll.290208WQ35	x	*Nymphalidae*	*Melitaea*	*deione*	L`Aldua,Lucena del Cid	Castellon	Comunidad Valenciana	Spain	1014
HM901456	EZSPC1271-10	RVcoll.290208WQ20	x	*Nymphalidae*	*Melitaea*	*deione*	Pobla del Bellestar,Vilafranca	Castellon	Comunidad Valenciana	Spain	1146
HM901464	EZSPC1291-10	RVcoll.140602MU8	x	*Nymphalidae*	*Melitaea*	*deione*	Coratxa,La Pobla de Benifassa	Castellon	Comunidad Valenciana	Spain	1200
GU675704	EZSPM120-09	RVcoll.08-P604	x	*Nymphalidae*	*Melitaea*	*deione*	Piscifactoria, Laguna de Una, Una	Cuenca	Castilla-La Mancha	Spain	1219
HM901281	EZSPC1080-10	RVcoll.08-L680	x	*Nymphalidae*	*Melitaea*	*deione*	Iznalloz, Sierra Arana	Granada	Andalusia	Spain	1306
HM901282	EZSPC1081-10	RVcoll.08-L681	x	*Nymphalidae*	*Melitaea*	*deione*	Iznalloz, Sierra Arana	Granada	Andalusia	Spain	1306
KP870762	EZSPC1427-10	RVcoll.09-V138	x	*Nymphalidae*	*Melitaea*	*deione*	E. Pradollano, Sierra Nevada	Granada	Andalusia	Spain	2000
KP870944	EZSPM613-12	RVcoll.11-D820	x	*Nymphalidae*	*Melitaea*	*deione*	Canada Real de la Zubia, Monachil	Granada	Andalusia	Spain	1684
KP870953	EZSPM614-12	RVcoll.11-D821	x	*Nymphalidae*	*Melitaea*	*deione*	Canada Real de la Zubia, Monachil	Granada	Andalusia	Spain	1684
GU676829	EZSPN101-09	RVcoll.06-A041	x	*Nymphalidae*	*Melitaea*	*deione*	Barranco de los Lagartos, Cadiar	Granada	Andalusia	Spain	860
GU676313	EZSPN844-09	RVcoll.08-L679	x	*Nymphalidae*	*Melitaea*	*deione*	Iznalloz, Sierra Arana	Granada	Andalusia	Spain	1306
GU676150	EZSPN1012-09	RVcoll.08-M932	x	*Nymphalidae*	*Melitaea*	*deione*	Vilamos, Vall d`Aran	Lleida	Catalonia	Spain	1178
GU675770	EZSPM261-09	RVcoll.08-R475	x	*Nymphalidae*	*Melitaea*	*deione*	Lobios, Camino de Sa a Portela de Homen	Ourense	Galicia	Spain	1100
GU676159	EZSPN1007-09	RVcoll.08-M699	x	*Nymphalidae*	*Melitaea*	*deione*	Farena, Alt Camp	Tarragona	Catalonia	Spain	580
GU676849	EZSPN081-09	RVcoll.08-J650	x	*Nymphalidae*	*Melitaea*	*deione*	Barranco del Toro	Teruel	Aragon	Spain	1443
GU676723	EZSPN311-09	RVcoll.08-J663	x	*Nymphalidae*	*Melitaea*	*deione*	Barranco del Toro	Teruel	Aragon	Spain	1443
JF415715	GWORK313-09	BC ZSM Lep 21548		*Nymphalidae*	*Melitaea*	*diamina*	Koenigsdorf, Weitfilz	Oberbayern	Bavaria	Germany	600
JF415716	GWORR678-10	BC ZSM Lep 32174		*Nymphalidae*	*Melitaea*	*diamina*	Lindenrain, Geretsried	Oberbayern	Bavaria	Germany	610
HM393216	GWORA2500-09	BC ZSM Lep 30712		*Nymphalidae*	*Melitaea*	*diamina*	Muehlau	Oberpfalz	Bavaria	Germany	380
HQ004782	EZRMN110-08	RVcoll.06-M864		*Nymphalidae*	*Melitaea*	*diamina*	Cluj-Napoca, dealul Feleacului, Gheorgheni forest	Cluj	Transylvania	Romania	600
HQ004783	EZRMN111-08	RVcoll.06-M880		*Nymphalidae*	*Melitaea*	*diamina*	Cluj-Napoca, dealul Feleacului, Gheorgheni forest	Cluj	Transylvania	Romania	600
HQ004787	EZROM414-08	RV-06-M863		*Nymphalidae*	*Melitaea*	*diamina*	Cluj-Napoca, dealul Feleacului, Gheorgheni forest	Cluj	Transylvania	Romania	600
HQ004785	EZROM680-08	RV-06-M866		*Nymphalidae*	*Melitaea*	*diamina*	Cluj-Napoca, dealul Feleacului, Gheorgheni forest	Cluj	Transylvania	Romania	600
HQ004784	EZRMN112-08	RVcoll.08-M592		*Nymphalidae*	*Melitaea*	*diamina*	Gheorgheni, Valea Belchia	Harghita	Transylvania	Romania	1000
HQ004788	EZROM415-08	RV-07-D542		*Nymphalidae*	*Melitaea*	*diamina*	Rohia	Maramures	Maramures	Romania	360
HQ004786	EZROM681-08	RV-07-D528		*Nymphalidae*	*Melitaea*	*diamina*	Rohia	Maramures	Maramures	Romania	360
HM901373	EZSPC1173-10	RVcoll.09-T132	x	*Nymphalidae*	*Melitaea*	*diamina*	Laguna del Cueto de Arbas, Cangas del Narcea		Asturias	Spain	1730
HM901374	EZSPC1174-10	RVcoll.09-T133	x	*Nymphalidae*	*Melitaea*	*diamina*	Laguna del Cueto de Arbas, Cangas del Narcea		Asturias	Spain	1730
GU669716	EZSPC545-09	RVcoll.08-P219	x	*Nymphalidae*	*Melitaea*	*diamina*	La Molina	Barcelona	Catalonia	Spain	1622
HM901452	EZSPC1258-10	RVcoll.280808DF35	x	*Nymphalidae*	*Melitaea*	*diamina*	Moli de Joaquim, La Pobla de Benifassa	Castellon	Comunidad Valenciana	Spain	971
KP870477	EZROM736-08	RV-07-C676	x	*Nymphalidae*	*Melitaea*	*diamina*	Sant Marti de Puigcerda	Girona	Catalonia	Spain	1145
HM901389	EZSPC1189-10	RVcoll.09-V317	x	*Nymphalidae*	*Melitaea*	*diamina*	Collada de Toses	Girona	Catalonia	Spain	1660
GU669717	EZSPC546-09	RVcoll.07-C681	x	*Nymphalidae*	*Melitaea*	*diamina*	Meranges	Girona	Catalonia	Spain	1555
HM901821	EZSPC986-10	RVcoll.07-C671	x	*Nymphalidae*	*Melitaea*	*diamina*	Sant Marti de Puigcerda	Girona	Catalonia	Spain	1145
HM901439	EZSPC1243-10	RVcoll.09-V756	x	*Nymphalidae*	*Melitaea*	*diamina*	Valle del Tena, Camino embalse Respomuso, Sallent de Gallego	Huesca	Aragon	Spain	1650
KP871163	EZSPM787-12	RVcoll.070611MH11	x	*Nymphalidae*	*Melitaea*	*diamina*	El Pueyo de Jaca, Panticosa	Huesca	Aragon	Spain	1779
GU669718	EZSPC547-09	RVcoll.07-W140	x	*Nymphalidae*	*Melitaea*	*diamina*	Val d`Hurno, Vall d`Aran	Lleida	Catalonia	Spain	1720
GU669719	EZSPC548-09	RVcoll.08-R115	x	*Nymphalidae*	*Melitaea*	*diamina*	Salt de Pitx, Vall d`Aran	Lleida	Catalonia	Spain	1570
JF415717	GWORK312-09	BC ZSM Lep 21547		*Nymphalidae*	*Melitaea*	*didyma*	Moernsheim, Steinbruch 2 km w	Oberpfalz	Bavaria	Germany	430
HQ004791	EZROM418-08	RV-06-M995		*Nymphalidae*	*Melitaea*	*didyma*	Apuseni Mts., Rimetea (Piatra Secuiului)	Alba	Transylvania	Romania	750
HQ004792	EZROM416-08	RV-06-M962		*Nymphalidae*	*Melitaea*	*didyma*	Racos	Brasov	Transylvania	Romania	460
KP870749	EULEP187-14	11-J885		*Nymphalidae*	*Melitaea*	*didyma*	Pădurea Hoia	Cluj	Transylvania	Romania	
HQ004790	EZRMN114-08	RVcoll.08-M300		*Nymphalidae*	*Melitaea*	*didyma*	Suatu	Cluj	Transylvania	Romania	400

GenBank accession number	BOLD Process ID	Sample ID	Used in the Iberian dataset	Family	Genus	Species	Exact Site	Region	State/Province	Country	Alt. (m)
HQ004793	EZROM417-08	RV-06-M973		*Nymphalidae*	*Melitaea*	*didyma*	Izvorul Muresului	Harghita	Transylvania	Romania	870
KP870704	EULEP143-14	10-C421		*Nymphalidae*	*Melitaea*	*didyma*	Lăpuș-Prislop	Maramureș	Maramureş	Romania	
HQ004789	EZRMN113-08	RVcoll.08-M265		*Nymphalidae*	*Melitaea*	*didyma*	4 Km W of Drobeta Turnu-Severin	Mehedinti	Oltenia	Romania	75
HM901283	EZSPC1082-10	RVcoll.08-L728	x	*Nymphalidae*	*Melitaea*	*didyma*	Arganda del Rey		Comunidad de Madrid	Spain	689
HM901300	EZSPC1099-10	RVcoll.08-L951	x	*Nymphalidae*	*Melitaea*	*didyma*	Puerto de Pozazal		Cantabria	Spain	1003
GU676256	EZSPN900-09	RVcoll.08-L812	x	*Nymphalidae*	*Melitaea*	*didyma*	San Andres		Cantabria	Spain	972
GU676247	EZSPN908-09	RVcoll.08-L832	x	*Nymphalidae*	*Melitaea*	*didyma*	Oteruelo del Valle		Comunidad de Madrid	Spain	1152
KP870836	EZROM737-08	RV-06-G478	x	*Nymphalidae*	*Melitaea*	*didyma*	La Trinitat, El Brull	Barcelona	Catalonia	Spain	850
GU669712	EZSPC549-09	RVcoll.06-H891	x	*Nymphalidae*	*Melitaea*	*didyma*	El Polell, P.N. del Montseny	Barcelona	Catalonia	Spain	780
GU669713	EZSPC550-09	RVcoll.08-L440	x	*Nymphalidae*	*Melitaea*	*didyma*	Balsareny, Bages	Barcelona	Catalonia	Spain	363
HM901249	EZSPC1046-10	RVcoll.08-J709	x	*Nymphalidae*	*Melitaea*	*didyma*	Alrededores de Almedijar, Almedijar	Castellon	Comunidad Valenciana	Spain	
HM901270	EZSPC1067-10	RVcoll.08-L204	x	*Nymphalidae*	*Melitaea*	*didyma*	La Vega, Vilafranca	Castellon	Comunidad Valenciana	Spain	1124
HM901825	EZSPC990-10	RVcoll.07-F075	x	*Nymphalidae*	*Melitaea*	*didyma*	10 km S. of Morella	Castellon	Comunidad Valenciana	Spain	900
HM901221	EZSPC1017-10	RVcoll.08-H692	x	*Nymphalidae*	*Melitaea*	*didyma*	Huelamo, Rio Jucar	Cuenca	Castilla-La Mancha	Spain	1237
GU669715	EZSPC552-09	RVcoll.08-P048	x	*Nymphalidae*	*Melitaea*	*didyma*	La Valira (near Urus), Cerdanya	Girona	Catalonia	Spain	1300
HQ562781	EZSPC1224-10	RVcoll.09-V572	x	*Nymphalidae*	*Melitaea*	*didyma*	Dornajo (Sierra Nevada)	Granada	Andalusia	Spain	2060
HQ562782	EZSPC1225-10	RVcoll.09-V583	x	*Nymphalidae*	*Melitaea*	*didyma*	Dornajo (Sierra Nevada)	Granada	Andalusia	Spain	2060
HQ562783	EZSPC1226-10	RVcoll.09-V584	x	*Nymphalidae*	*Melitaea*	*didyma*	Dornajo (Sierra Nevada)	Granada	Andalusia	Spain	2060
KP870593	EZSPM604-12	RVcoll.11-D602	x	*Nymphalidae*	*Melitaea*	*didyma*	Canada Real de la Zubia, Monachil	Granada	Andalusia	Spain	1684
KP870659	EZSPM605-12	RVcoll.11-D609	x	*Nymphalidae*	*Melitaea*	*didyma*	Canada Real de la Zubia, Monachil	Granada	Andalusia	Spain	1684
GU676464	EZSPN680-09	RVcoll.08-J993	x	*Nymphalidae*	*Melitaea*	*didyma*	Casillas de Rojas, Gueejar Sierra	Granada	Andalusia	Spain	1733
HM901292	EZSPC1091-10	RVcoll.08-L914	x	*Nymphalidae*	*Melitaea*	*didyma*	Escamilla	Guadalajara	Castilla-La Mancha	Spain	1040
HM901293	EZSPC1092-10	RVcoll.08-L915.1	x	*Nymphalidae*	*Melitaea*	*didyma*	Escamilla	Guadalajara	Castilla-La Mancha	Spain	1040
KP870744	EZSPM797-12	RVcoll.090111XZ00	x	*Nymphalidae*	*Melitaea*	*didyma*	Balneario de Panticosa	Huesca	Aragon	Spain	1642
GU676138	EZSPN1026-09	RVcoll.08-M960	x	*Nymphalidae*	*Melitaea*	*didyma*	Saganta	Huesca	Aragon	Spain	767
GU669714	EZSPC551-09	RVcoll.08-P370	x	*Nymphalidae*	*Melitaea*	*didyma*	Perves, Pallars Jussa	Lleida	Catalonia	Spain	1071
GU675881	EZSPM355-09	RVcoll.08-R068	x	*Nymphalidae*	*Melitaea*	*didyma*	La Cerollera	Teruel	Aragon	Spain	781
GU676395	EZSPN757-09	RVcoll.08-L234	x	*Nymphalidae*	*Melitaea*	*didyma*	Ruta de la Sima, El Vallecillo	Teruel	Aragon	Spain	1531
HM901250	EZSPC1047-10	RVcoll.08-J735	x	*Nymphalidae*	*Melitaea*	*didyma*	Piscifactoria de La Tosquilla, Titaguas	Valencia	Comunidad Valenciana	Spain	586
HM901251	EZSPC1048-10	RVcoll.08-J737	x	*Nymphalidae*	*Melitaea*	*didyma*	Casa de Camineros, Tuejar	Valencia	Comunidad Valenciana	Spain	581
HM901349	EZSPC1149-10	RVcoll.08-P929	x	*Nymphalidae*	*Melitaea*	*didyma*	Yatova, Sierra Martes	Valencia	Comunidad Valenciana	Spain	900
GU676943	EZSPN539-09	RVcoll.08-J030	x	*Nymphalidae*	*Melitaea*	*didyma*	Teresa de Cofrentes, arroyo Cangrejos, Cofrentes	Valencia	Comunidad Valenciana	Spain	
HM901809	EZSPC974-10	RVcoll.06-G485	x	*Nymphalidae*	*Melitaea*	*didyma*	Barranco de Valcuerna, Candasnos	Zaragoza	Aragon	Spain	210
HM901810	EZSPC975-10	RVcoll.06-G491	x	*Nymphalidae*	*Melitaea*	*didyma*	Barranco de Valcuerna, Candasnos	Zaragoza	Aragon	Spain	210
HM901235	EZSPC1031-10	RVcoll.08-J112	x	*Nymphalidae*	*Melitaea*	*parthenoides*	Ribeira de Varzea (EN311), Varzea Cova, Fafe		Minho	Portugal	500
HM901236	EZSPC1032-10	RVcoll.08-J115	x	*Nymphalidae*	*Melitaea*	*parthenoides*	Ribeira de Varzea (EN311), Varzea Cova, Fafe		Minho	Portugal	500
GU676570	EZSPN567-09	RVcoll.08-J108	x	*Nymphalidae*	*Melitaea*	*parthenoides*	Ribeira de Varzea (EN311), Varzea Cova, Fafe		Minho	Portugal	500
HM901375	EZSPC1175-10	RVcoll.09-T134	x	*Nymphalidae*	*Melitaea*	*parthenoides*	Laguna de Arbas, Cangas del Narcea		Asturias	Spain	1730
GU675706	EZSPM149-09	RVcoll.08-P664	x	*Nymphalidae*	*Melitaea*	*parthenoides*	Gamoniteiru, Sierra de Aramo, Quiros		Asturias	Spain	1712
GU676164	EZSPN1000-09	RVcoll.08-M084	x	*Nymphalidae*	*Melitaea*	*parthenoides*	Oteruelo del Valle		Comunidad de Madrid	Spain	1148
GU676271	EZSPN890-09	RVcoll.08-L801	x	*Nymphalidae*	*Melitaea*	*parthenoides*	Hormiguera		Cantabria	Spain	966
GU676320	EZSPN832-09	RVcoll.08-L650	x	*Nymphalidae*	*Melitaea*	*parthenoides*	Hoyocasero, Pinar de Hoyocasero	Avila	Castilla y Leon	Spain	1254
HM901820	EZSPC985-10	RVcoll.07-C483	x	*Nymphalidae*	*Melitaea*	*parthenoides*	El Brull, Osona	Barcelona	Catalonia	Spain	830
HM901834	EZSPC999-10	RVcoll.07-Z074	x	*Nymphalidae*	*Melitaea*	*parthenoides*	La Nou de Bergueda, BERGUEDA	Barcelona	Catalonia	Spain	990

GenBank accession number	BOLD Process ID	Sample ID	Used in the Iberian dataset	Family	Genus	Species	Exact Site	Region	State/Province	Country	Alt. (m)
GU676741	EZSPN296-09	RVcoll.07-Z073	x	*Nymphalidae*	*Melitaea*	*parthenoides*	La Nou de Bergueda, Bergueda	Barcelona	Catalonia	Spain	990
HM901269	EZSPC1066-10	RVcoll.08-L182	x	*Nymphalidae*	*Melitaea*	*parthenoides*	Font del Regatxal, Vilafranca	Castellon	Comunidad Valenciana	Spain	1361
HM901451	EZSPC1257-10	RVcoll.280808DF25	x	*Nymphalidae*	*Melitaea*	*parthenoides*	Moli de Joaquim,La Pobla de Benifassa	Castellon	Comunidad Valenciana	Spain	971
HM901475	EZSPC1303-10	RVcoll.140602MU13	x	*Nymphalidae*	*Melitaea*	*parthenoides*	Coratxa,La Pobla de Benifassa	Castellon	Comunidad Valenciana	Spain	1200
GU676404	EZSPN742-09	RVcoll.08-L166	x	*Nymphalidae*	*Melitaea*	*parthenoides*	Font del Regatxal, Vilafranca	Castellon	Comunidad Valenciana	Spain	1361
GU676117	EZSPM474-09	Rvcoll.09-V435	x	*Nymphalidae*	*Melitaea*	*parthenoides*	San Juan (Sierra Nevada)	Granada	Andalusia	Spain	2760
GU676114	EZSPM475-09	Rvcoll.09-V443	x	*Nymphalidae*	*Melitaea*	*parthenoides*	San Juan (Sierra Nevada)	Granada	Andalusia	Spain	2760
KP870760	EZSPM647-12	RVcoll.11-I423	x	*Nymphalidae*	*Melitaea*	*parthenoides*	Puerto de la Ragua (Sierra Nevada)	Granada	Andalusia	Spain	2090
GU676492	EZSPN656-09	RVcoll.08-J952	x	*Nymphalidae*	*Melitaea*	*parthenoides*	Fuente Albergue de San Francisco, Guejar Sierra	Granada	Andalusia	Spain	2221
GU676494	EZSPN657-09	RVcoll.08-J955	x	*Nymphalidae*	*Melitaea*	*parthenoides*	Fuente Albergue de San Francisco, Guejar Sierra	Granada	Andalusia	Spain	2221
GU676116	EZSPM427-09	Rvcoll.09-X546	x	*Nymphalidae*	*Melitaea*	*parthenoides*	Camino de Wamba, Puerto de San Isidro	Leon	Castilla y Leon	Spain	1215
HM901463	EZSPC1288-10	100908AW5	x	*Nymphalidae*	*Melitaea*	*parthenoides*	Muela del Royo, Castielfabib	Valencia	Comunidad Valenciana	Spain	1450
KP870548	FBLMX204-11	BC ZSM Lep 50415		*Nymphalidae*	*Melitaea*	*phoebe*	Ruhpolding, Seehauser Hochkienberg	Oberbayern	Bavaria	Germany	770
GU655012	FBLMT870-09	BC ZSM Lep 25430		*Nymphalidae*	*Melitaea*	*phoebe*	Sinzing, Alpiner Steig	Oberpfalz	Bavaria	Germany	405
KP870686	FBLMX205-11	BC ZSM Lep 50416		*Nymphalidae*	*Melitaea*	*phoebe*	Oberstdorf, Oytal	Schwaben	Bavaria	Germany	1025
HQ004798	EZRMN115-08	RVcoll.08-M214		*Nymphalidae*	*Melitaea*	*phoebe*	3 Km SW of Lunca Corbului	Arges	Muntenia	Romania	235
HQ004796	EZRMN117-08	RVcoll.08-M371		*Nymphalidae*	*Melitaea*	*phoebe*	Buciumeni forest (Radacinesti)	Bacau	Moldavia	Romania	180
HQ004803	EZROM589-08	RV-07-C300		*Nymphalidae*	*Melitaea*	*phoebe*	Rediu forest, Botosani	Botosani	Moldavia	Romania	160
GU669661	EZROM1050-09	RVcoll.07-D325		*Nymphalidae*	*Melitaea*	*phoebe*	Racos	Brasov	Transylvania	Romania	460
HQ004802	EZROM641-08	RV-07-D316		*Nymphalidae*	*Melitaea*	*phoebe*	Dumbrava Vadului, Vad	Brasov	Transylvania	Romania	495
HQ004794	EZRMN121-08	RVcoll.08-M572		*Nymphalidae*	*Melitaea*	*phoebe*	Breaza, dealul Istrita	Buzau	Muntenia	Romania	600
HQ004807	EZROM421-08	RV-07-D111		*Nymphalidae*	*Melitaea*	*phoebe*	Nenciulesti	Buzau	Muntenia	Romania	360
HQ004808	EZROM422-08	RV-07-D116		*Nymphalidae*	*Melitaea*	*phoebe*	Nenciulesti	Buzau	Muntenia	Romania	360
HQ004795	EZRMN120-08	RVcoll.08-M551		*Nymphalidae*	*Melitaea*	*phoebe*	Valea Feregarilor - Poiana Musuroaie	Caras-Severin	Banat	Romania	350
HQ004809	EZROM423-08	RV-07-C127		*Nymphalidae*	*Melitaea*	*phoebe*	Fanatele Clujului (dealul Bogomaia), Cluj-Napoca	Cluj	Transylvania	Romania	480
HQ004805	EZROM424-08	RV-06-V689		*Nymphalidae*	*Melitaea*	*phoebe*	Suatu	Cluj	Transylvania	Romania	380
HQ004799	EZRMN119-08	RVcoll.08-M418		*Nymphalidae*	*Melitaea*	*phoebe*	1 Km NE of Gura Dobrogei reserve	Constanta	Dobrogea	Romania	40
GU669658	EZROM1047-09	RVcoll.07-D182		*Nymphalidae*	*Melitaea*	*phoebe*	Sipote	Constanta	Dobrogea	Romania	90
HQ004810	EZROM419-08	RV-07-D004		*Nymphalidae*	*Melitaea*	*phoebe*	Canaraua Fetei (Baneasa)	Constanta	Dobrogea	Romania	15
HQ004801	EZROM640-08	RV-07-D123		*Nymphalidae*	*Melitaea*	*phoebe*	Dumbraveni forest	Constanta	Dobrogea	Romania	80
HQ004800	EZRMN116-08	RVcoll.08-M224		*Nymphalidae*	*Melitaea*	*phoebe*	2 Km E of Moreni	Dambovita	Muntenia	Romania	290
GU669653	EZROM1058-09	RVcoll.07-E393		*Nymphalidae*	*Melitaea*	*phoebe*	Picior de Munte	Dambovita	Muntenia	Romania	260
GU669654	EZROM1051-09	RVcoll.07-D477		*Nymphalidae*	*Melitaea*	*phoebe*	Cheile Babei	Maramures	Maramures	Romania	265
HQ004797	EZRMN118-08	RVcoll.08-M406		*Nymphalidae*	*Melitaea*	*phoebe*	Macin Mts. (3 Km S of Jijila)	Tulcea	Dobrogea	Romania	230
HQ004806	EZROM420-08	RV-07-D048		*Nymphalidae*	*Melitaea*	*phoebe*	Babadag forest	Tulcea	Dobrogea	Romania	115
HQ004804	EZROM425-08	RV-07-E422		*Nymphalidae*	*Melitaea*	*phoebe*	Nifon (Macin Mts.)	Tulcea	Dobrogea	Romania	120
GU676637	EZSPN404-09	RVcoll.08-H479	x	*Nymphalidae*	*Melitaea*	*phoebe*	Colmenar Viejo		Comunidad de Madrid	Spain	848
KP870947	EZSPM631-12	RVcoll.11-E050	x	*Nymphalidae*	*Melitaea*	*phoebe*	Font de l`Arbre, Serra d`Aitana	Alicante	Comunidad Valenciana	Spain	1209
KP870767	EZROM738-08	RV-06-H893	x	*Nymphalidae*	*Melitaea*	*phoebe*	Vallforners, Canoves	Barcelona	Catalonia	Spain	650
GU669708	EZSPC553-09	RVcoll.08-L412	x	*Nymphalidae*	*Melitaea*	*phoebe*	Sallent, Bages	Barcelona	Catalonia	Spain	381
GU676890	EZSPN487-09	RVcoll.08-H924	x	*Nymphalidae*	*Melitaea*	*phoebe*	Hervas, Carretera Hervas-La Garganta	Caceres	Extremadura	Spain	940
GU669709	EZSPC554-09	RVcoll.07-C628	x	*Nymphalidae*	*Melitaea*	*phoebe*	Meranges	Girona	Catalonia	Spain	1555
GU676852	EZSPN084-09	RVcoll.06-A003	x	*Nymphalidae*	*Melitaea*	*phoebe*	Road from Ferreira to Puerto de La Ragua	Granada	Andalusia	Spain	1600
GU676454	EZSPN695-09	RVcoll.08-L027	x	*Nymphalidae*	*Melitaea*	*phoebe*	Casillas de Rojas, Gueejar Sierra	Granada	Andalusia	Spain	1733
GU669710	EZSPC555-09	RVcoll.08-L464	x	*Nymphalidae*	*Melitaea*	*phoebe*	Ivars de Noguera	Lleida	Catalonia	Spain	373
GU675767	EZSPM263-09	RVcoll.08-R478	x	*Nymphalidae*	*Melitaea*	*phoebe*	Pedrafita do Cebreiro, Serra do Caurel-Hospital	Lugo	Galicia	Spain	1230

GenBank accession number	BOLD Process ID	Sample ID	Used in the Iberian dataset	Family	Genus	Species	Exact Site	Region	State/Province	Country	Alt. (m)
KP870493	EZSPM623-12	RVcoll.11-D910	x	*Nymphalidae*	*Melitaea*	*phoebe*	Puerto del Leon	Malaga	Andalusia	Spain	617
GU676907	EZSPN502-09	RVcoll.08-H956	x	*Nymphalidae*	*Melitaea*	*phoebe*	Candelario, Sierra de Candelario	Salamanca	Castilla y Leon	Spain	1300
GU669711	EZSPC556-09	RVcoll.08-M684	x	*Nymphalidae*	*Melitaea*	*phoebe*	Farena, Alt Camp	Tarragona	Catalonia	Spain	580
KP870343	EZSPM895-12	Rvcoll.12-M617	x	*Nymphalidae*	*Melitaea*	*phoebe*	Medina del Campo	Valladolid	Castilla y Leon	Spain	739
GU676707	EZSPN328-09	RVcoll.08-H236	x	*Nymphalidae*	*Melitaea*	*phoebe*	Mequinenza	Zaragoza	Aragon	Spain	129
HQ004817	EZROM426-08	RV-06-M925		*Nymphalidae*	*Melitaea*	*trivia*	Dealul Istrita, Breaza	Buzau	Muntenia	Romania	600
HQ004818	EZROM427-08	RV-07-D218		*Nymphalidae*	*Melitaea*	*trivia*	Dealul Istrita, Breaza	Buzau	Muntenia	Romania	540
HQ004814	EZRMN122-08	RVcoll.07-D591		*Nymphalidae*	*Melitaea*	*trivia*	Teregova	Caras-Severin	Banat	Romania	385
HQ004813	EZRMN123-08	RVcoll.07-E301		*Nymphalidae*	*Melitaea*	*trivia*	Baile Herculane (Pecinisca)	Caras-Severin	Banat	Romania	250
HQ004816	EZROM428-08	RV-07-E353		*Nymphalidae*	*Melitaea*	*trivia*	Baile Herculane (Pecinisca)	Caras-Severin	Banat	Romania	270
HQ004815	EZROM642-08	RV-07-D481		*Nymphalidae*	*Melitaea*	*trivia*	Cheile Babei	Maramures	Maramures	Romania	265
KP870797	EULEP021-14	07-D058		*Nymphalidae*	*Melitaea*	*trivia*	Babadag forest	Tulcea	Dobrogea	Romania	115
HQ004812	EZRMN124-08	RVcoll.08-M407		*Nymphalidae*	*Melitaea*	*trivia*	Macin Mts. (3 Km S of Jijila)	Tulcea	Dobrogea	Romania	230
HQ004811	EZRMN125-08	RVcoll.08-M420		*Nymphalidae*	*Melitaea*	*trivia*	3 km E of Slava Rusa (Babadag forest)	Tulcea	Dobrogea	Romania	130
GU676290	EZSPN868-09	RVcoll.08-L746	x	*Nymphalidae*	*Melitaea*	*trivia*	Oteruelo del Valle		Comunidad de Madrid	Spain	1267
GU676322	EZSPN834-09	RVcoll.08-L655	x	*Nymphalidae*	*Melitaea*	*trivia*	San Martin del Pimpollar, Piscifactoria Rasquilla	Avila	Castilla y Leon	Spain	1270
KP870802	EZROM739-08	RV-06-G474	x	*Nymphalidae*	*Melitaea*	*trivia*	La Trinitat, El Brull, Osona	Barcelona	Catalonia	Spain	850
GU669704	EZSPC557-09	RVcoll.06-G475	x	*Nymphalidae*	*Melitaea*	*trivia*	La Trinitat, El Brull, Osona	Barcelona	Catalonia	Spain	880
GU669705	EZSPC558-09	RVcoll.06-G476	x	*Nymphalidae*	*Melitaea*	*trivia*	La Trinitat, El Brull, Osona	Barcelona	Catalonia	Spain	880
GU669706	EZSPC559-09	RVcoll.06-G477	x	*Nymphalidae*	*Melitaea*	*trivia*	La Trinitat, El Brull, Osona	Barcelona	Catalonia	Spain	880
GU669640	EZSPC643-09	RVcoll.08-L373	x	*Nymphalidae*	*Melitaea*	*trivia*	Viladrau, Osona	Barcelona	Catalonia	Spain	810
GU676896	EZSPN493-09	RVcoll.08-H931	x	*Nymphalidae*	*Melitaea*	*trivia*	Hervas, Carretera Hervas-La Garganta	Caceres	Extremadura	Spain	1007
HM901460	EZSPC1283-10	RVcoll.270808DT38	x	*Nymphalidae*	*Melitaea*	*trivia*	Puerto de Ares,Ares del Maestre	Castellon	Comunidad Valenciana	Spain	872
HM901461	EZSPC1285-10	RVcoll.150707PK18	x	*Nymphalidae*	*Melitaea*	*trivia*	Alrededores de Cati,Cati	Castellon	Comunidad Valenciana	Spain	709
GU677040	EZSPN175-09	RVcoll.06-K711	x	*Nymphalidae*	*Melitaea*	*trivia*	Prados de Otero,Sierra Nevada, GUeEJAR SIERRA	Granada	Andalusia	Spain	2226
GU677037	EZSPN176-09	RVcoll.06-K712	x	*Nymphalidae*	*Melitaea*	*trivia*	Prados de Otero,Sierra Nevada, GUeEJAR SIERRA	Granada	Andalusia	Spain	2227
GU675694	EZSPM100-09	RVcoll.08-P430	x	*Nymphalidae*	*Melitaea*	*trivia*	Candelario	Salamanca	Castilla y Leon	Spain	1126
HM902067	FBLMU377-09	BC ZSM Lep 27027		*Nymphalidae*	*Minois*	*dryas*	Bad Abbach, Oberndorfer Haenge	Niederbayern	Bavaria	Germany	370
GU707298	FBLMV721-09	BC ZSM Lep 28701		*Nymphalidae*	*Minois*	*dryas*	Diessen, Alte Ammer	Oberbayern	Bavaria	Germany	535
HM910014	GWORT469-10	BC ZSM Lep 32155		*Nymphalidae*	*Minois*	*dryas*	Zellwies, Koenigsdorf	Oberbayern	Bavaria	Germany	600
HM910015	GWORT470-10	BC ZSM Lep 32156		*Nymphalidae*	*Minois*	*dryas*	Ismaning, Fischerhaeuser	Oberbayern	Bavaria	Germany	482
HQ004820	EZRMN127-08	RVcoll.08-M571		*Nymphalidae*	*Minois*	*dryas*	Breaza, dealul Istrita	Buzau	Muntenia	Romania	600
HQ004819	EZRMN128-08	RVcoll.08-M658		*Nymphalidae*	*Minois*	*dryas*	Berca, vulcanii noroiosi Paclele Mici	Buzau	Muntenia	Romania	220
HQ004821	EZRMN126-08	RVcoll.07-E385		*Nymphalidae*	*Minois*	*dryas*	Motel Dumbrava (Cerna Valley)	Caras-Severin	Banat	Romania	260
HQ004822	EZROM429-08	RV-06-M982		*Nymphalidae*	*Minois*	*dryas*	Apuseni Mts., Baisoara	Cluj	Transylvania	Romania	590
HQ004823	EZROM430-08	RV-07-E398		*Nymphalidae*	*Minois*	*dryas*	Picior de Munte	Dambovita	Muntenia	Romania	260
HQ004824	EZROM431-08	RV-07-E430		*Nymphalidae*	*Minois*	*dryas*	3 km E of Slava Rusa (Babadag forest)	Tulcea	Dobrogea	Romania	130
HQ004825	EZROM643-08	RV-07-C207		*Nymphalidae*	*Minois*	*dryas*	Buila-Vanturarita (Vanturarita Mare)	Valcea	Oltenia	Romania	1830
KP870743	EZSPM722-12	RVcoll.08-J477	x	*Nymphalidae*	*Minois*	*dryas*	Cruz de Priena, Covadonga, PN Picos de Europa		Asturias	Spain	641
KP871008	EZSPM723-12	RVcoll.08-J478	x	*Nymphalidae*	*Minois*	*dryas*	Cruz de Priena, Covadonga, PN Picos de Europa		Asturias	Spain	641
KP870995	EZSPM724-12	RVcoll.08-J479	x	*Nymphalidae*	*Minois*	*dryas*	Cruz de Priena, Covadonga, PN Picos de Europa		Asturias	Spain	641
KP870759	EZSPM729-12	RVcoll.12-L041	x	*Nymphalidae*	*Minois*	*dryas*	Gorliz	Biscay	Basque Country	Spain	70
KP870708	EZSPM730-12	RVcoll.12-L042	x	*Nymphalidae*	*Minois*	*dryas*	Gorliz	Biscay	Basque Country	Spain	70
KP870728	EULEP251-14	12-Q445	x	*Nymphalidae*	*Minois*	*dryas*	Valle de Mena	Burgos	Castilla y León	Spain	410
KP870718	EULEP252-14	12-Q446	x	*Nymphalidae*	*Minois*	*dryas*	Valle de Mena	Burgos	Castilla y León	Spain	410
KP870420	EULEP053-14	08-J480	x	*Nymphalidae*	*Minois*	*dryas*	Cruz de Priena, Covadonga, Picos de Europa		Asturias	Spain	641

GenBank accession number	BOLD Process ID	Sample ID	Used in the Iberian dataset	Family	Genus	Species	Exact Site	Region	State/Province	Country	Alt. (m)
KP870317	EULEP054-14	08-J481	x	*Nymphalidae*	*Minois*	*dryas*	Cruz de Priena, Covadonga, Picos de Europa		Asturias	Spain	641
KP870368	EULEP055-14	08-J482	x	*Nymphalidae*	*Minois*	*dryas*	Cruz de Priena, Covadonga, Picos de Europa		Asturias	Spain	641
KP870992	EULEP056-14	08-J483	x	*Nymphalidae*	*Minois*	*dryas*	Cruz de Priena, Covadonga, Picos de Europa		Asturias	Spain	641
KP870676	EULEP194-14	12-L000	x	*Nymphalidae*	*Minois*	*dryas*	Pico Priena, Cangas de Onís		Asturias	Spain	
KP870443	EULEP195-14	12-L003	x	*Nymphalidae*	*Minois*	*dryas*	Pico Priena, Cangas de Onís		Asturias	Spain	
KP870829	EULEP215-14	12-L038	x	*Nymphalidae*	*Minois*	*dryas*	Gorliz		Basque Country	Spain	70
KP870340	EULEP216-14	12-L039	x	*Nymphalidae*	*Minois*	*dryas*	Gorliz		Basque Country	Spain	70
KP870293	EULEP217-14	12-L040	x	*Nymphalidae*	*Minois*	*dryas*	Gorliz		Basque Country	Spain	70
HQ004829	EZRMN129-08	RVcoll.08-M296		*Hesperiidae*	*Muschampia*	*cribrellum*	Suatu	Cluj	Transylvania	Romania	400
HQ004828	EZRMN130-08	RVcoll.08-M297		*Hesperiidae*	*Muschampia*	*cribrellum*	Suatu	Cluj	Transylvania	Romania	400
HQ004827	EZRMN131-08	RVcoll.08-M298		*Hesperiidae*	*Muschampia*	*cribrellum*	Suatu	Cluj	Transylvania	Romania	400
HQ004826	EZRMN132-08	RVcoll.08-M299		*Hesperiidae*	*Muschampia*	*cribrellum*	Suatu	Cluj	Transylvania	Romania	400
HQ004830	EZROM432-08	RV-07-D401		*Hesperiidae*	*Muschampia*	*cribrellum*	Suatu	Cluj	Transylvania	Romania	380
HQ004831	EZROM433-08	RV-07-D402		*Hesperiidae*	*Muschampia*	*cribrellum*	Suatu	Cluj	Transylvania	Romania	380
KP871132	EULEP154-14	11-D634	x	*Hesperiidae*	*Muschampia*	*proto*	Porches, Lagoa		Algarve	Portugal	
KP871062	EULEP156-14	11-D636	x	*Hesperiidae*	*Muschampia*	*proto*	Bensafrim, Lagos		Algarve	Portugal	
KP870239	EZSPM731-12	RVcoll.12-L587	x	*Hesperiidae*	*Muschampia*	*proto*	Sagres, Vila do Bispo		Algarve	Portugal	
GU676745	EZSPN293-09	RVcoll.07-W265	x	*Hesperiidae*	*Muschampia*	*proto*	P.K. 1,9 M-219, Loeches		Comunidad de Madrid	Spain	
GU677002	EZSPN215-09	RVcoll.07-F074	x	*Hesperiidae*	*Muschampia*	*proto*	10 km S. of Morella	Castellon	Comunidad Valenciana	Spain	900
KP871108	EZSPM940-12	Rvcoll.12-M674	x	*Hesperiidae*	*Muschampia*	*proto*	Rio Jandula, Mestanza	Ciudad Real	Castilla-La Mancha	Spain	407
KP870731	EZSPM965-12	Rvcoll.12-M717	x	*Hesperiidae*	*Muschampia*	*proto*	Alrededores San Lorenzo de Calatrava	Ciudad Real	Castilla-La Mancha	Spain	749
KP870975	EZSPM966-12	Rvcoll.12-M718	x	*Hesperiidae*	*Muschampia*	*proto*	Alrededores San Lorenzo de Calatrava	Ciudad Real	Castilla-La Mancha	Spain	749
HM901808	EZSPC973-10	RVcoll.06-A320	x	*Hesperiidae*	*Muschampia*	*proto*	Sierra de los Barrancos, Cuenca	Cuenca	Castilla-La Mancha	Spain	1447
GU676807	EZSPN127-09	RVcoll.06-A318	x	*Hesperiidae*	*Muschampia*	*proto*	Casa de la Herreria, Huelamo	Cuenca	Castilla-La Mancha	Spain	1270
HM901222	EZSPC1018-10	RVcoll.09-V505	x	*Hesperiidae*	*Muschampia*	*proto*	Sierra de Alfacar	Granada	Andalusia	Spain	1275
HM901262	EZSPC1059-10	RVcoll.08-L046	x	*Hesperiidae*	*Muschampia*	*proto*	Sierra Elvira, Albolote	Granada	Andalusia	Spain	819
HM901337	EZSPC1137-10	RVcoll.08-P726	x	*Hesperiidae*	*Muschampia*	*proto*	Aldeire	Granada	Andalusia	Spain	1335
HM901413	EZSPC1214-10	RVcoll.09-V510	x	*Hesperiidae*	*Muschampia*	*proto*	Sierra de Alfacar	Granada	Andalusia	Spain	1275
GU675730	EZSPM111-09	RVcoll.08-P453	x	*Hesperiidae*	*Muschampia*	*proto*	Zaorejas, Alto Tajo	Guadalajara	Castilla-La Mancha	Spain	1284
KP870418	EULEP155-14	11-D635	x	*Hesperiidae*	*Muschampia*	*proto*	Alrededores de Villarrasa, Villarrasa	Huelva	Andalusia	Spain	
GU677031	EZSPN185-09	RVcoll.07-C087	x	*Hesperiidae*	*Muschampia*	*proto*	Boixols, Pallars Jussa	Lleida	Catalonia	Spain	1155
GU676983	EZSPN240-09	RVcoll.07-W094	x	*Hesperiidae*	*Muschampia*	*proto*	Sierra de Ayllon, Languilla	Segovia	Castilla y Leon	Spain	
HM901823	EZSPC988-10	RVcoll.07-C699	x	*Hesperiidae*	*Muschampia*	*proto*	Barranc de Vinarroig, la Febro	Tarragona	Catalonia	Spain	
HM901351	EZSPC1151-10	RVcoll.08-R007	x	*Hesperiidae*	*Muschampia*	*proto*	Paraisos Altos, Manzanera	Teruel	Aragon	Spain	1200
GU676955	EZSPN543-09	RVcoll.08-J039	x	*Hesperiidae*	*Muschampia*	*proto*	Cortes de Pallas (Sierra Martes), Cortes de Pallas	Valencia	Comunidad Valenciana	Spain	
HQ004833	EZRMN133-08	RVcoll.08-M668		*Hesperiidae*	*Muschampia*	*tessellum*	Fanatele Clujului (dealul Bogomaia), Cluj-Napoca	Cluj	Transylvania	Romania	450
HQ004832	EZRMN335-08	RVcoll.08-H007		*Hesperiidae*	*Muschampia*	*tessellum*	Cluj-Napoca, dealul Bogomaia, Fanatele Clujului	Cluj	Transylvania	Romania	450
HQ004834	EZROM434-08	RV-07-C125		*Hesperiidae*	*Muschampia*	*tessellum*	dealul Bogomaia (Valea Calda), Cluj-Napoca	Cluj	Transylvania	Romania	380
HQ004835	EZROM435-08	RV-07-C126		*Hesperiidae*	*Muschampia*	*tessellum*	dealul Bogomaia (Valea Calda), Cluj-Napoca	Cluj	Transylvania	Romania	380
HQ004843	EZRMN136-08	RVcoll.08-M509		*Nymphalidae*	*Neptis*	*rivularis*	Apuseni Mts., Rimetea (Piatra Secuiului)	Alba	Transylvania	Romania	850
HQ004845	EZROM436-08	RV-06-N007		*Nymphalidae*	*Neptis*	*rivularis*	Apuseni Mts., Rimetea (Piatra Secuiului)	Alba	Transylvania	Romania	750
HQ004847	EZROM682-08	RV-06-N017		*Nymphalidae*	*Neptis*	*rivularis*	Apuseni Mts., Rimetea (Piatra Secuiului)	Alba	Transylvania	Romania	650
HQ004842	EZRMN137-08	RVcoll.08-M542		*Nymphalidae*	*Neptis*	*rivularis*	Valea Feregarilor - Poiana Musuroaie	Caras-Severin	Banat	Romania	350
HQ004844	EZRMN135-08	RVcoll.08-M507		*Nymphalidae*	*Neptis*	*rivularis*	Badeni	Cluj	Transylvania	Romania	490
HQ004841	EZRMN138-08	RVcoll.08-M600		*Nymphalidae*	*Neptis*	*rivularis*	Gheorgheni, Valea Belchia	Harghita	Transylvania	Romania	1000
HQ004840	EZRMN139-08	RVcoll.08-M644		*Nymphalidae*	*Neptis*	*rivularis*	Scorota (Retezat Mts.)	Hunedoara	Transylvania	Romania	1560
HQ004846	EZROM437-08	RV-07-D485		*Nymphalidae*	*Neptis*	*rivularis*	Cheile Babei	Maramures	Maramures	Romania	265

GenBank accession number	BOLD Process ID	Sample ID	Used in the Iberian dataset	Family	Genus	Species	Exact Site	Region	State/Province	Country	Alt. (m)
HQ004855	EZROM644-08	RV-06-M984		*Nymphalidae*	*Neptis*	*sappho*	Apuseni Mts., Rimetea (Piatra Secuiului)	Alba	Transylvania	Romania	750
HQ004853	EZROM439-08	RV-07-E334		*Nymphalidae*	*Neptis*	*sappho*	Baile Herculane (Pecinisca)	Caras-Severin	Banat	Romania	270
HQ004851	EZRMN140-08	RVcoll.06-K604		*Nymphalidae*	*Neptis*	*sappho*	Badeni	Cluj	Transylvania	Romania	440
HQ004850	EZRMN141-08	RVcoll.06-M860		*Nymphalidae*	*Neptis*	*sappho*	Cluj-Napoca, dealul Feleacului, Gheorgheni forest	Cluj	Transylvania	Romania	600
HQ004848	EZRMN143-08	RVcoll.08-M506		*Nymphalidae*	*Neptis*	*sappho*	Badeni	Cluj	Transylvania	Romania	490
HQ004852	EZROM438-08	RV-06-K648		*Nymphalidae*	*Neptis*	*sappho*	Baciu forest (Cluj-Napoca)	Cluj	Transylvania	Romania	530
HQ004854	EZROM440-08	RV-07-E522		*Nymphalidae*	*Neptis*	*sappho*	2 km N of Cilieni	Olt	Oltenia	Romania	50
HQ004849	EZRMN142-08	RVcoll.06-M900		*Nymphalidae*	*Neptis*	*sappho*	Stana	Salaj	Transylvania	Romania	500
HM393222	GWORA2508-09	BC ZSM Lep 30720		*Nymphalidae*	*Nymphalis*	*antiopa*	Oberaudorf, Muehlau	Oberbayern	Bavaria	Germany	500
HM393223	GWORA2509-09	BC ZSM Lep 30721		*Nymphalidae*	*Nymphalis*	*antiopa*	Roding	Oberpfalz	Bavaria	Germany	
KP870666	EULEP015-14	07-C393		*Nymphalidae*	*Nymphalis*	*antiopa*	Gheorgheni, Dealul Feleacului, Cluj-Napoca	Cluj	Transylvania	Romania	
HQ004857	EZRMN145-08	RVcoll.08-M669		*Nymphalidae*	*Nymphalis*	*antiopa*	Gheorgheni forest, Cluj-Napoca	Cluj	Transylvania	Romania	600
HQ004858	EZROM441-08	RV-06-M842		*Nymphalidae*	*Nymphalis*	*antiopa*	Cluj-Napoca, valea paraului Micus	Cluj	Transylvania	Romania	700
HQ004859	EZRMN423-09	RVcoll.09-V660		*Nymphalidae*	*Nymphalis*	*antiopa*	Cheile Bicazului	Neamt	Moldavia	Romania	960
HQ004856	EZRMN144-08	RVcoll.08-M663		*Nymphalidae*	*Nymphalis*	*antiopa*	Balea Cascada, Fagaras Mts.	Sibiu	Transylvania	Romania	1300
KP871106	EZROM742-08	RV-07-C000	x	*Nymphalidae*	*Nymphalis*	*antiopa*	Vallforners, Canoves	Barcelona	Catalonia	Spain	650
GU669700	EZSPC567-09	RVcoll.08-H218	x	*Nymphalidae*	*Nymphalis*	*antiopa*	Vallforners, Canoves	Barcelona	Catalonia	Spain	610
GU669701	EZSPC568-09	RVcoll.08-H219	x	*Nymphalidae*	*Nymphalis*	*antiopa*	Vallforners, Canoves	Barcelona	Catalonia	Spain	610
GU669694	EZSPC569-09	RVcoll.08-H226	x	*Nymphalidae*	*Nymphalis*	*antiopa*	Vallforners, Canoves	Barcelona	Catalonia	Spain	610
GU669695	EZSPC570-09	RVcoll.08-H227	x	*Nymphalidae*	*Nymphalis*	*antiopa*	Vallforners, Canoves	Barcelona	Catalonia	Spain	610
KP870828	EZSPM798-12	RVcoll.090111XZ40	x	*Nymphalidae*	*Nymphalis*	*antiopa*	Ibon de Plan, Saravillo	Huesca	Aragon	Spain	1942
KP870721	EZSPM799-12	RVcoll.090111XZ43	x	*Nymphalidae*	*Nymphalis*	*antiopa*	Ibon de Plan, Saravillo	Huesca	Aragon	Spain	1942
KP870850	EZSPM800-12	RVcoll.090111XZ44	x	*Nymphalidae*	*Nymphalis*	*antiopa*	Ibon de Plan, Saravillo	Huesca	Aragon	Spain	1942
KP870651	EZSPM732-12	RVcoll.08-J461	x	*Nymphalidae*	*Nymphalis*	*antiopa*	Iglesia, Puerto el Ponton	Leon	Castilla y Leon	Spain	1282
KP870835	EZSPM733-12	RVcoll.08-J462	x	*Nymphalidae*	*Nymphalis*	*antiopa*	Iglesia, Puerto el Ponton	Leon	Castilla y Leon	Spain	1282
KP870403	EZSPM734-12	RVcoll.08-J463	x	*Nymphalidae*	*Nymphalis*	*antiopa*	Iglesia, Puerto el Ponton	Leon	Castilla y Leon	Spain	1282
KP871102	EULEP200-14	12-L011	x	*Nymphalidae*	*Nymphalis*	*antiopa*	Camino a Braña Campo Las Corrada, Degaña		Asturias	Spain	
HQ957215	GWORA2507-09	BC ZSM Lep 30719		*Nymphalidae*	*Nymphalis*	*polychloros*	Auenzell near Saulburg	Niederbayern	Bavaria	Germany	440
KP871057	FBLMX201-11	BC ZSM Lep 50412		*Nymphalidae*	*Nymphalis*	*polychloros*	Teisendorf, Waschau	Oberbayern	Bavaria	Germany	520
HM393221	GWORA2506-09	BC ZSM Lep 30718		*Nymphalidae*	*Nymphalis*	*polychloros*	Leak near Regensburg, Thiergarten	Oberpfalz	Bavaria	Germany	330
JF415718	GWOR3922-09	BC ZSM Lep 21206		*Nymphalidae*	*Nymphalis*	*polychloros*	Dachau Hebertshausen, Schiessanlage	Upper Bavaria	Bavaria	Germany	475
HQ004867	EZROM294-08	RV-07-E656		*Nymphalidae*	*Nymphalis*	*polychloros*	Dealul Istrita, Breaza	Buzau	Muntenia	Romania	525
HQ004865	EZROM303-08	RV-07-E665		*Nymphalidae*	*Nymphalis*	*polychloros*	Dealul Istrita, Breaza	Buzau	Muntenia	Romania	525
HQ004868	EZROM285-08	RV-07-E647		*Nymphalidae*	*Nymphalis*	*polychloros*	Cluj-Napoca, valea Morii	Cluj	Transylvania	Romania	650
HQ004866	EZROM302-08	RV-07-E664		*Nymphalidae*	*Nymphalis*	*polychloros*	Cluj-Napoca, valea Garbaului	Cluj	Transylvania	Romania	400
HQ004864	EZROM314-08	RV-07-E676		*Nymphalidae*	*Nymphalis*	*polychloros*	Cluj-Napoca, dealul Feleacului, Gheorgheni forest	Cluj	Transylvania	Romania	600
HQ004862	EZROM352-08	RV-08-A030		*Nymphalidae*	*Nymphalis*	*polychloros*	Cluj-Napoca, dealul Feleacului, Gheorgheni forest	Cluj	Transylvania	Romania	600
HQ004863	EZROM353-08	RV-08-A031		*Nymphalidae*	*Nymphalis*	*polychloros*	Cluj-Napoca, dealul Feleacului, Gheorgheni forest	Cluj	Transylvania	Romania	600
HQ004861	EZRMN146-08	RVcoll.08-M477		*Nymphalidae*	*Nymphalis*	*polychloros*	Babadag forest (10 Km S of Babadag)	Tulcea	Dobrogea	Romania	115
GU676692	EZSPN345-09	RVcoll.08-H310	x	*Nymphalidae*	*Nymphalis*	*polychloros*	Casillas, Casillas	Avila	Castilla y Leon	Spain	
JN827886	EZROM743-08	RV-06-H867	x	*Nymphalidae*	*Nymphalis*	*polychloros*	Vallforners, Canoves	Barcelona	Catalonia	Spain	650
GU669696	EZSPC571-09	RVcoll.07-C007	x	*Nymphalidae*	*Nymphalis*	*polychloros*	Vallforners, Canoves, Valles Oriental	Barcelona	Catalonia	Spain	650
GU669697	EZSPC572-09	RVcoll.08-H223	x	*Nymphalidae*	*Nymphalis*	*polychloros*	Vallforners, Canoves	Barcelona	Catalonia	Spain	610
GU669690	EZSPC573-09	RVcoll.08-H228	x	*Nymphalidae*	*Nymphalis*	*polychloros*	Vallforners, Canoves	Barcelona	Catalonia	Spain	610
KP870976	EZSPM735-12	RVcoll.12-L621	x	*Nymphalidae*	*Nymphalis*	*polychloros*	El Espadanal, Navalmoral de la Mata	Caceres	Extremadura	Spain	
KP870369	EZSPM736-12	RVcoll.12-L622	x	*Nymphalidae*	*Nymphalis*	*polychloros*	El Espadanal, Navalmoral de la Mata	Caceres	Extremadura	Spain	
KP870742	EZSPM737-12	RVcoll.12-L623	x	*Nymphalidae*	*Nymphalis*	*polychloros*	El Espadanal, Navalmoral de la Mata	Caceres	Extremadura	Spain	
GU676449	EZSPN698-09	RVcoll.08-L040	x	*Nymphalidae*	*Nymphalis*	*polychloros*	Sierra Elvira, Albolote	Granada	Andalusia	Spain	819
HM901580	EZSPC725-10	RVcoll.08-H553	x	*Nymphalidae*	*Nymphalis*	*polychloros*	Cifuentes, La Alcarria	Guadalajara	Castilla-La Mancha	Spain	801
GU676620	EZSPN423-09	RVcoll.08-H579	x	*Nymphalidae*	*Nymphalis*	*polychloros*	Gualda, La Alcarria	Guadalajara	Castilla-La Mancha	Spain	742
JF847981	EZSPN032-09	RVcoll.09-T067	x	*Nymphalidae*	*Nymphalis*	*polychloros*	Sant Joan de Toran, Vall d`Aran	Lleida	Catalonia	Spain	990

GenBank accession number	BOLD Process ID	Sample ID	Used in the Iberian dataset	Family	Genus	Species	Exact Site	Region	State/Province	Country	Alt. (m)
HM901480	EZSPC1320-10	RVcoll.130209KL80	x	*Nymphalidae*	*Nymphalis*	*polychloros*	El Regajo,Sinarcas	Valencia	Comunidad Valenciana	Spain	
GU676513	EZSPN631-09	RVcoll.08-J730	x	*Nymphalidae*	*Nymphalis*	*polychloros*	Piscifactoria de La Tosquilla, Titaguas	Valencia	Comunidad Valenciana	Spain	586
HQ004860	EZRMN419-09	RVcoll.07-D628		*Nymphalidae*	*Nymphalis*	*vaualbum*	Capalnas	Arad	Banat	Romania	180
HQ004873	EZROM442-08	RV-07-C961		*Nymphalidae*	*Nymphalis*	*xanthomelas*	Dumbrava Vadului, Vad	Brasov	Transylvania	Romania	495
HQ004869	EZRMN147-08	RVcoll.08-M670		*Nymphalidae*	*Nymphalis*	*xanthomelas*	Gheorgheni forest, Cluj-Napoca	Cluj	Transylvania	Romania	600
HQ004870	EZROM284-08	RV-07-E646		*Nymphalidae*	*Nymphalis*	*xanthomelas*	Cluj-Napoca, valea Garbaului	Cluj	Transylvania	Romania	400
HQ004871	EZROM299-08	RV-07-E661		*Nymphalidae*	*Nymphalis*	*xanthomelas*	Martinesti	Cluj	Transylvania	Romania	550
HQ004872	EZROM313-08	RV-07-E675		*Nymphalidae*	*Nymphalis*	*xanthomelas*	Cluj-Napoca, dealul Feleacului, Gheorgheni forest	Cluj	Transylvania	Romania	600
GU655010	FBLMT906-09	BC ZSM Lep 25466		*Hesperiidae*	*Ochlodes*	*sylvanus*	Diessen am Ammersee, Alte Ammer	Oberbayern	Bavaria	Germany	535
HM391838	FBLMU507-09	BC ZSM Lep 27157		*Hesperiidae*	*Ochlodes*	*sylvanus*	Laufen, Schoenramer Filz	Oberbayern	Bavaria	Germany	440
JF415719	GWORR703-10	BC ZSM Lep 32199		*Hesperiidae*	*Ochlodes*	*sylvanus*	Lindenrain, Geretsried	Oberbayern	Bavaria	Germany	610
GU707038	FBLMU257-09	BC ZSM Lep 25767		*Hesperiidae*	*Ochlodes*	*sylvanus*	Kallmuenz, Kirchenberg	Oberpfalz	Bavaria	Germany	390
GU676557	EZSPN590-09	RVcoll.08-J160	x	*Hesperiidae*	*Ochlodes*	*sylvanus*	Ponte Pedrinha, Beca, Boticas		Tras-os-Montes	Portugal	730
HQ004877	EZRMN149-08	RVcoll.08-M377		*Hesperiidae*	*Ochlodes*	*sylvanus*	Buciumeni forest (Radacinesti)	Bacau	Moldavia	Romania	180
HQ004881	EZROM443-08	RV-07-D115		*Hesperiidae*	*Ochlodes*	*sylvanus*	Nenciulesti	Buzau	Muntenia	Romania	360
HQ004880	EZROM446-08	RV-06-M958		*Hesperiidae*	*Ochlodes*	*sylvanus*	Dealul Istrita, Breaza	Buzau	Muntenia	Romania	600
HQ004882	EZROM444-08	RV-07-C934		*Hesperiidae*	*Ochlodes*	*sylvanus*	Teregova	Caras-Severin	Banat	Romania	385
HQ004878	EZRMN336-08	RVcoll.08-H008		*Hesperiidae*	*Ochlodes*	*sylvanus*	Cluj-Napoca, dealul Feleacului, Gheorgheni forest	Cluj	Transylvania	Romania	600
HQ004879	EZROM339-08	RV-08-A017		*Hesperiidae*	*Ochlodes*	*sylvanus*	Cluj-Napoca, dealul Bogomaia, Fanatele Clujului	Cluj	Transylvania	Romania	480
HQ004883	EZROM445-08	RV-06-M847		*Hesperiidae*	*Ochlodes*	*sylvanus*	Cluj-Napoca, valea paraului Micus	Cluj	Transylvania	Romania	700
HQ004875	EZRMN151-08	RVcoll.08-M458		*Hesperiidae*	*Ochlodes*	*sylvanus*	Esechioi forest	Constanta	Dobrogea	Romania	135
HQ004876	EZRMN148-08	RVcoll.08-M227		*Hesperiidae*	*Ochlodes*	*sylvanus*	2 Km E of Moreni	Dambovita	Muntenia	Romania	290
HQ004874	EZRMN150-08	RVcoll.08-M399		*Hesperiidae*	*Ochlodes*	*sylvanus*	Macin Mts. (3 Km S of Jijila)	Tulcea	Dobrogea	Romania	230
GU676154	EZSPN1002-09	RVcoll.08-M088	x	*Hesperiidae*	*Ochlodes*	*sylvanus*	Oteruelo del Valle		Comunidad de Madrid	Spain	1148
GU676961	EZSPN265-09	RVcoll.07-W177	x	*Hesperiidae*	*Ochlodes*	*sylvanus*	La Mortera, Ribera de Arriba		Asturias	Spain	416
GU676958	EZSPN266-09	RVcoll.07-W180	x	*Hesperiidae*	*Ochlodes*	*sylvanus*	La Mortera, Ribera de Arriba		Asturias	Spain	416
GU669691	EZSPC574-09	RVcoll.08-L509	x	*Hesperiidae*	*Ochlodes*	*sylvanus*	El Cafe, pla de la Calma, El Brull, Osona	Barcelona	Catalonia	Spain	1192
GU669692	EZSPC575-09	RVcoll.08-M713	x	*Hesperiidae*	*Ochlodes*	*sylvanus*	Delta de Llobregat	Barcelona	Catalonia	Spain	2
KP870483	EZSPM915-12	Rvcoll.12-M637	x	*Hesperiidae*	*Ochlodes*	*sylvanus*	Lagunas de Ruidera, Ossa de Montiel	Ciudad Real	Castilla-La Mancha	Spain	795
GU669686	EZSPC577-09	RVcoll.08-P063	x	*Hesperiidae*	*Ochlodes*	*sylvanus*	PEIN, Riberes de l`Alt Segre, Cerdanya	Girona	Catalonia	Spain	1100
GU669687	EZSPC578-09	RVcoll.07-C666	x	*Hesperiidae*	*Ochlodes*	*sylvanus*	Sant Marti de Puigcerda	Girona	Catalonia	Spain	1145
GU676175	EZSPN987-09	RVcoll.08-M030	x	*Hesperiidae*	*Ochlodes*	*sylvanus*	Gualda, La Alcarria	Guadalajara	Castilla-La Mancha	Spain	730
GU676824	EZSPN113-09	RVcoll.06-A272	x	*Hesperiidae*	*Ochlodes*	*sylvanus*	Laguarta, Rio Alcanadre	Huesca	Aragon	Spain	
GU675864	EZSPM375-09	RVcoll.08-J862	x	*Hesperiidae*	*Ochlodes*	*sylvanus*	Fresno de la Vega	Leon	Castilla y Leon	Spain	
GU669693	EZSPC576-09	RVcoll.07-W104	x	*Hesperiidae*	*Ochlodes*	*sylvanus*	Vilac, Vall d`Aran	Lleida	Catalonia	Spain	1024
GU676980	EZSPN241-09	RVcoll.07-W096	x	*Hesperiidae*	*Ochlodes*	*sylvanus*	Candelario	Salamanca	Castilla y Leon	Spain	1350
GU676947	EZSPN544-09	RVcoll.08-J044	x	*Hesperiidae*	*Ochlodes*	*sylvanus*	Cortes de Pallas (Sierra Martes), Cortes de Pallas	Valencia	Comunidad Valenciana	Spain	
HQ565496	GWOSA786-10	BC ZSM Lep 36652		*Nymphalidae*	*Oeneis*	*glacialis*	Karwendelgebirge, Hirzeneck	Oberbayern	Bavaria	Germany	1600
KP870752	FBLMX212-11	BC ZSM Lep 50423		*Nymphalidae*	*Oeneis*	*glacialis*	Oberstdorf, Edmund-Probst-Haus	Schwaben	Bavaria	Germany	1900
HM425911	PHLAA392-09	TLMF Lep 00432		*Nymphalidae*	*Oeneis*	*glacialis*	Gias Valcavera/ Demonte NW	Cuneo		Italy	2050
HQ968455	PHLAB287-10	TLMF Lep 01087		*Nymphalidae*	*Oeneis*	*glacialis*	Tiefencastel S/ Salouf, Got Grond		Graubunden	Switzerland	1650
HM386889	LEFIA745-10	MM04099		*Nymphalidae*	*Oeneis*	*norna*	Enontekioe		Lapland	Finland	
HQ570284	LEFIC093-10	MM03405		*Nymphalidae*	*Oeneis*	*norna*	Enontekioe	Lapponia enontekiensis	Lapland	Finland	
JF415720	GWORK513-09	BC ZSM Lep 21843		*Papilionidae*	*Papilio*	*machaon*	Diessen	Oberbayern	Bavaria	Germany	600
GU707119	FBLMU410-09	BC ZSM Lep 27060		*Papilionidae*	*Papilio*	*machaon*	Irlbach bei Wenzenbach	Oberpfalz	Bavaria	Germany	385
HQ004887	EZROM450-08	RV-07-D302		*Papilionidae*	*Papilio*	*machaon*	Dumbrava Vadului, Vad	Brasov	Transylvania	Romania	495
HQ004886	EZROM338-08	RV-08-A016		*Papilionidae*	*Papilio*	*machaon*	Dealul Istrita, Coama Istritei	Buzau	Muntenia	Romania	690
HQ004889	EZROM449-08	RV-06-M955		*Papilionidae*	*Papilio*	*machaon*	Dealul Istrita, Breaza	Buzau	Muntenia	Romania	600
KP870927	EULEP176-14	11-J350		*Papilionidae*	*Papilio*	*machaon*	Breaza, Dealul Istrița	Buzău	Muntenia	Romania	400-650

GenBank accession number	BOLD Process ID	Sample ID	Used in the Iberian dataset	Family	Genus	Species	Exact Site	Region	State/Province	Country	Alt. (m)
KP870574	EULEP179-14	11-J399		*Papilionidae*	*Papilio*	*machaon*	Breaza, Dealul Istrița	Buzău	Muntenia	Romania	400-720
HQ004888	EZROM447-08	RV-06-K527		*Papilionidae*	*Papilio*	*machaon*	Badeni	Cluj	Transylvania	Romania	440
HQ004890	EZROM448-08	RV-06-K638		*Papilionidae*	*Papilio*	*machaon*	Baciu forest (Cluj-Napoca)	Cluj	Transylvania	Romania	530
HQ004884	EZRMN152-08	RVcoll.08-M338		*Papilionidae*	*Papilio*	*machaon*	1 Km E of Gheorgheni	Harghita	Transylvania	Romania	900
HQ004885	EZRMN153-08	RVcoll.08-M392		*Papilionidae*	*Papilio*	*machaon*	Macin Mts. (3 Km S of Jijila)	Tulcea	Dobrogea	Romania	230
GU676652	EZSPN395-09	RVcoll.08-H454	x	*Papilionidae*	*Papilio*	*machaon*	Campo Real		Comunidad de Madrid	Spain	736
GU675943	EZSPM289-09	SMcoll.270708ZB68	x	*Papilionidae*	*Papilio*	*machaon*	Huelga-Utrera, Pontones	Albacete	Castilla-La Mancha	Spain	
GU669689	EZSPC582-09	RVcoll.08-J361	x	*Papilionidae*	*Papilio*	*machaon*	Vallgrassa, Parc Natural del Garraf	Barcelona	Catalonia	Spain	285
GU676601	EZSPN442-09	RVcoll.08-H627	x	*Papilionidae*	*Papilio*	*machaon*	El Gastor	Cadiz	Andalusia	Spain	660
HM901370	EZSPC1170-10	RVcoll.09-T105	x	*Papilionidae*	*Papilio*	*machaon*	Una	Cuenca	Castilla-La Mancha	Spain	1201
GU669682	EZSPC583-09	RVcoll.08-J346	x	*Papilionidae*	*Papilio*	*machaon*	Cantallops, Alt Emporda	Girona	Catalonia	Spain	225
HM901399	EZSPC1199-10	RVcoll.09-V419	x	*Papilionidae*	*Papilio*	*machaon*	San Juan (Sierra Nevada)	Granada	Andalusia	Spain	2760
GU676539	EZSPN609-09	RVcoll.08-J326	x	*Papilionidae*	*Papilio*	*machaon*	Barranco de Valcuerna, Candasnos	Huesca	Aragon	Spain	210
KP870541	EZSPM1043-12	Rvcoll.12-M963	x	*Papilionidae*	*Papilio*	*machaon*	Santa Eularia des Riu, Ibiza	Ibiza	Balearic Islands	Spain	48.2
GU675872	EZSPM366-09	RVcoll.08-J830	x	*Papilionidae*	*Papilio*	*machaon*	Mansilla de las Mulas	Leon	Castilla y Leon	Spain	
GU669683	EZSPC584-09	RVcoll.08-R265	x	*Papilionidae*	*Papilio*	*machaon*	Vilamos, Vall d`Aran	Lleida	Catalonia	Spain	1387
GU676002	EZSPM043-09	RVcoll.08-P251	x	*Papilionidae*	*Papilio*	*machaon*	Santa Maria	Mallorca	Balearic Islands	Spain	181
GU675999	EZSPM049-09	RVcoll.08-P267	x	*Papilionidae*	*Papilio*	*machaon*	S`Albufera	Mallorca	Balearic Islands	Spain	4
GU676000	EZSPM060-09	RVcoll.08-P286	x	*Papilionidae*	*Papilio*	*machaon*	Genova	Mallorca	Balearic Islands	Spain	123
GU675856	EZSPM207-09	RVcoll.08-R325	x	*Papilionidae*	*Papilio*	*machaon*	Pic Tomir	Mallorca	Balearic Islands	Spain	1048
KP870584	EZSPM1009-12	Rvcoll.12-L899	x	*Papilionidae*	*Papilio*	*machaon*	Son Gornes, Ferreries, Menorca	Menorca	Balearic Islands	Spain	
KP870235	EZSPM993-12	Rvcoll.12-L879	x	*Papilionidae*	*Papilio*	*machaon*	Rafael Vell, Mao, Menorca	Menorca	Balearic Islands	Spain	
KP870273	EZSPM996-12	Rvcoll.12-L882	x	*Papilionidae*	*Papilio*	*machaon*	Turdonell de Dalt, Mao, Menorca	Menorca	Balearic Islands	Spain	
HM901252	EZSPC1049-10	RVcoll.08-J745	x	*Papilionidae*	*Papilio*	*machaon*	Casa de Camineros, Tuejar	Valencia	Comunidad Valenciana	Spain	581
GU675945	EZSPM287-09	SMcoll.311007WR55	x	*Papilionidae*	*Papilio*	*machaon*	Montecanada, Paterna	Valencia	Comunidad Valenciana	Spain	89
GU655009	FBLMT891-09	BC ZSM Lep 25451		*Nymphalidae*	*Pararge*	*aegeria*	Neustadt/Donau, Plattenberg	Niederbayern	Bavaria	Germany	370
HM393197	GWORA2475-09	BC ZSM Lep 30687		*Nymphalidae*	*Pararge*	*aegeria*	Grandsberg	Niederbayern	Bavaria	Germany	850
GU655008	FBLMT873-09	BC ZSM Lep 25433		*Nymphalidae*	*Pararge*	*aegeria*	Jesenwang, Fuerstenfelder Wald	Oberbayern	Bavaria	Germany	590
KP870824	FBLMX207-11	BC ZSM Lep 50418		*Nymphalidae*	*Pararge*	*aegeria*	Teisendorf	Oberbayern	Bavaria	Germany	520
HM393198	GWORA2476-09	BC ZSM Lep 30688		*Nymphalidae*	*Pararge*	*aegeria*	Arzmoos	Oberbayern	Bavaria	Germany	1200
HM910007	GWORT461-10	BC ZSM Lep 32147		*Nymphalidae*	*Pararge*	*aegeria*	Isarauen, Fischerhaeuser	Oberbayern	Bavaria	Germany	482
HM391831	FBLMU489-09	BC ZSM Lep 27139		*Nymphalidae*	*Pararge*	*aegeria*	Nittendorf	Oberpfalz	Bavaria	Germany	400
HM391832	FBLMU490-09	BC ZSM Lep 27140		*Nymphalidae*	*Pararge*	*aegeria*	Bodenwoehr, Postloher Forst	Oberpfalz	Bavaria	Germany	380
JF859941	PHLAC361-10	TLMF Lep 02396		*Nymphalidae*	*Pararge*	*aegeria*	Ritten/ Obergruenwald		South Tyrol	Italy	1750
GU676575	EZSPN572-09	RVcoll.08-J119	x	*Nymphalidae*	*Pararge*	*aegeria*	Quinta de Pentieiros, Bertiandos, Ponte de Lima		Minho	Portugal	20
KP870498	EZSPM738-12	RVcoll.12-L607	x	*Nymphalidae*	*Pararge*	*aegeria*	Rasmalho, Portimao		Algarve	Portugal	
HQ004896	EZROM453-08	RV-07-D200		*Nymphalidae*	*Pararge*	*aegeria*	Dealul Istrita, Breaza	Buzau	Muntenia	Romania	540
HQ004895	EZROM454-08	RV-07-D220		*Nymphalidae*	*Pararge*	*aegeria*	Dealul Istrita, Breaza	Buzau	Muntenia	Romania	540
HQ004897	EZROM455-08	RV-07-C975		*Nymphalidae*	*Pararge*	*aegeria*	Baile Herculane (Pecinisca)	Caras-Severin	Banat	Romania	220
HQ004894	EZROM645-08	RV-07-D597		*Nymphalidae*	*Pararge*	*aegeria*	Teregova	Caras-Severin	Banat	Romania	385
HQ004898	EZROM451-08	RV-06-K523		*Nymphalidae*	*Pararge*	*aegeria*	Badeni	Cluj	Transylvania	Romania	440
HQ004899	EZROM452-08	RV-06-K524		*Nymphalidae*	*Pararge*	*aegeria*	Badeni	Cluj	Transylvania	Romania	440
HQ004891	EZRMN156-08	RVcoll.08-M445		*Nymphalidae*	*Pararge*	*aegeria*	Canaraua Fetei (Baneasa)	Constanta	Dobrogea	Romania	20
HQ004892	EZRMN154-08	RVcoll.08-M232		*Nymphalidae*	*Pararge*	*aegeria*	2 Km E of Moreni	Dambovita	Muntenia	Romania	290
HQ004893	EZRMN155-08	RVcoll.08-M358		*Nymphalidae*	*Pararge*	*aegeria*	Cheile Bicazului	Harghita	Transylvania	Romania	940
FJ663885	LOWA761-06	2005-LOWA-761		*Nymphalidae*	*Pararge*	*aegeria*	Bogorodizk		Tula	Russia	
HM901666	EZSPC817-10	RVcoll.08-P648	x	*Nymphalidae*	*Pararge*	*aegeria*	Valle (Valle-Zurea), Lena		Asturias	Spain	662
HM901697	EZSPC856-10	RVcoll.08-R452	x	*Nymphalidae*	*Pararge*	*aegeria*	Ames, Novais	A Coruna	Galicia	Spain	160
GU675814	EZSPM243-09	RVcoll.08-R453	x	*Nymphalidae*	*Pararge*	*aegeria*	Ames, Novais	A Coruna	Galicia	Spain	160
KP870562	EZROM745-08	RV-07-C003	x	*Nymphalidae*	*Pararge*	*aegeria*	Granja Viader, Cardedeu	Barcelona	Catalonia	Spain	185
GU669684	EZSPC585-09	RVcoll.08-H233	x	*Nymphalidae*	*Pararge*	*aegeria*	UAB, Bellaterra	Barcelona	Catalonia	Spain	184
GU675811	EZSPM276-09	SMcoll.08-J610	x	*Nymphalidae*	*Pararge*	*aegeria*	Cadiz	Cadiz	Andalusia	Spain	
GU676609	EZSPN437-09	RVcoll.08-H619	x	*Nymphalidae*	*Pararge*	*aegeria*	El Gastor	Cadiz	Andalusia	Spain	660

GenBank accession number	BOLD Process ID	Sample ID	Used in the Iberian dataset	Family	Genus	Species	Exact Site	Region	State/Province	Country	Alt. (m)
HM901607	EZSPC752-10	RVcoll.08-L197	x	*Nymphalidae*	*Pararge*	*aegeria*	La Vega, Vilafranca	Castellon	Comunidad Valenciana	Spain	1124
KP870966	EZSPM921-12	Rvcoll.12-M643	x	*Nymphalidae*	*Pararge*	*aegeria*	Lagunas de Ruidera, Ossa de Montiel	Ciudad Real	Castilla-La Mancha	Spain	795
GU669681	EZSPC588-09	RVcoll.08-P056	x	*Nymphalidae*	*Pararge*	*aegeria*	Estoll, Fontanals de Cerdanya, Cerdanya	Girona	Catalonia	Spain	1085
GU675868	EZSPM371-09	RVcoll.08-J844	x	*Nymphalidae*	*Pararge*	*aegeria*	Posada de Valdeon	Leon	Castilla y Leon	Spain	
GU669685	EZSPC586-09	RVcoll.08-M920	x	*Nymphalidae*	*Pararge*	*aegeria*	Arros, Vall d`Aran	Lleida	Catalonia	Spain	906
HM901646	EZSPC796-10	RVcoll.08-P262	x	*Nymphalidae*	*Pararge*	*aegeria*	Santa Maria	Mallorca	Balearic Islands	Spain	181.3
HM901648	EZSPC798-10	RVcoll.08-P307	x	*Nymphalidae*	*Pararge*	*aegeria*	Sant Elm, Andratx	Mallorca	Balearic Islands	Spain	30.9
GU676009	EZSPM053-09	RVcoll.08-P273	x	*Nymphalidae*	*Pararge*	*aegeria*	S`Albufera	Mallorca	Balearic Islands	Spain	4
GU676010	EZSPM064-09	RVcoll.08-P296	x	*Nymphalidae*	*Pararge*	*aegeria*	Genova	Mallorca	Balearic Islands	Spain	123
HM901780	EZSPC943-10	RVcoll.09-X110	x	*Nymphalidae*	*Pararge*	*aegeria*	Parque Albufera des Grau, Menorca	Menorca	Balearic Islands	Spain	15
GU676738	EZSPN301-09	RVcoll.08-H106	x	*Nymphalidae*	*Pararge*	*aegeria*	Na Vermella, Mao	Menorca	Balearic Islands	Spain	
GU676710	EZSPN323-09	RVcoll.08-H185	x	*Nymphalidae*	*Pararge*	*aegeria*	Llimpa, Mao	Menorca	Balearic Islands	Spain	
GU669680	EZSPC587-09	RVcoll.08-P324	x	*Nymphalidae*	*Pararge*	*aegeria*	La Riba, Alt Camp	Tarragona	Catalonia	Spain	335
GU676676	EZSPN370-09	RVcoll.08-H395	x	*Nymphalidae*	*Pararge*	*aegeria*	Serra, Serra	Valencia	Comunidad Valenciana	Spain	
GU675951	EZSPM014-09	RVcoll.08-M999	x	*Papilionidae*	*Parnassius*	*apollo*	Courbassil	Languedoc-Roussillon		France	1390
HM393172	GWORA2431-09	BC ZSM Lep 30643		*Papilionidae*	*Parnassius*	*apollo*	St- Bartholomae	Oberbayern	Bavaria	Germany	600
HM393173	GWORA2432-09	BC ZSM Lep 30644		*Papilionidae*	*Parnassius*	*apollo*	Sudelfeld, Arzmoos	Oberbayern	Bavaria	Germany	1200
HM393174	GWORA2435-09	BC ZSM Lep 30647		*Papilionidae*	*Parnassius*	*apollo*	Eichstaett, Wintershof	Oberpfalz	Bavaria	Germany	
JF415721	GWORK287-09	BC ZSM Lep 21522		*Papilionidae*	*Parnassius*	*apollo*	Moernsheim, Steinbruch 2 km w	Oberpfalz	Bavaria	Germany	430
HQ004901	EZRMN157-08	RVcoll.08-M577		*Papilionidae*	*Parnassius*	*apollo*	Valea Cupas (Hasmas Mts.)	Harghita	Transylvania	Romania	1100
HQ004900	EZRMN365-08	RVcoll.08-M732		*Papilionidae*	*Parnassius*	*apollo*	Oriental Carpathians, Cheile Bicazului	Harghita	Transylvania	Romania	1200
GU675952	EZSPM091-09	RVcoll.08-P417	x	*Papilionidae*	*Parnassius*	*apollo*	Puerto de San Glorio		Cantabria	Spain	1508
GU676684	EZSPN359-09	RVcoll.08-H337	x	*Papilionidae*	*Parnassius*	*apollo*	Puerto de Morcuera, Miraflores		Comunidad de Madrid	Spain	
GU676222	EZSPN933-09	RVcoll.08-L893	x	*Papilionidae*	*Parnassius*	*apollo*	Puerto de Pozazal		Cantabria	Spain	1080
HM901800	EZSPC965-10	RVcoll.190608FT56	x	*Papilionidae*	*Parnassius*	*apollo*	Font del Regatxal, Vilafranca	Castellon	Comunidad Valenciana	Spain	1361
GU676860	EZSPC589-09	RVcoll.07-C489	x	*Papilionidae*	*Parnassius*	*apollo*	Collada de Toses	Girona	Catalonia	Spain	1500
GU676861	EZSPC590-09	RVcoll.07-C492	x	*Papilionidae*	*Parnassius*	*apollo*	Meranges	Girona	Catalonia	Spain	1555
GU676857	EZSPC591-09	RVcoll.08-R195	x	*Papilionidae*	*Parnassius*	*apollo*	Setcases	Girona	Catalonia	Spain	1400
GU676858	EZSPC592-09	RVcoll.08-R196	x	*Papilionidae*	*Parnassius*	*apollo*	Setcases	Girona	Catalonia	Spain	1400
HM901730	EZSPC891-10	RVcoll.09-V401	x	*Papilionidae*	*Parnassius*	*apollo*	San Juan (Sierra Nevada)	Granada	Andalusia	Spain	2760
HM901794	EZSPC959-10	RVcoll.100608DS55	x	*Papilionidae*	*Parnassius*	*apollo*	Laguna Seca, Hueneja	Granada	Andalusia	Spain	2276
GU676063	EZSPM469-09	Rvcoll.09-V528	x	*Papilionidae*	*Parnassius*	*apollo*	Loma de San Francisco (Sierra Nevada)	Granada	Andalusia	Spain	2315
KP870873	EZSPC866-10	RVcoll.09-T088	x	*Papilionidae*	*Parnassius*	*apollo*	Pico del Oroel, Jaca	Huesca	Aragon	Spain	1660
HM901745	EZSPC907-10	RVcoll.09-V757	x	*Papilionidae*	*Parnassius*	*apollo*	Embalse La Sarra, Sallent de Gallego	Huesca	Aragon	Spain	1410
GU675933	EZSPM299-09	RVcoll.08-J846	x	*Papilionidae*	*Parnassius*	*apollo*	Cremenes	Leon	Castilla y Leon	Spain	
GU676062	EZSPM428-09	Rvcoll.09-X530	x	*Papilionidae*	*Parnassius*	*apollo*	Buron	Leon	Castilla y Leon	Spain	1120
GU676859	EZSPC593-09	RVcoll.08-R189	x	*Papilionidae*	*Parnassius*	*apollo*	Salt de Pitx, Vall d`Aran	Lleida	Catalonia	Spain	1570
HM901754	EZSPC916-10	RVcoll.09-V869	x	*Papilionidae*	*Parnassius*	*apollo*	Vall d`Aran	Lleida	Catalonia	Spain	1540
HM901803	EZSPC968-10	RVcoll.220708XF62	x	*Papilionidae*	*Parnassius*	*apollo*	Ruta de la Sima, El Vallecillo	Teruel	Aragon	Spain	1531
HM901796	EZSPC961-10	RVcoll.100608DS77	x	*Papilionidae*	*Parnassius*	*apollo*	Loma de Sancho, Alpuente	Valencia	Comunidad Valenciana	Spain	1492
GU676681	EZSPN364-09	RVcoll.08-H348	x	*Papilionidae*	*Parnassius*	*apollo*	Sierra de Javalambre, Alpuente	Valencia	Comunidad Valenciana	Spain	
KP870377	EULEP210-14	12-L028	x	*Papilionidae*	*Parnassius*	*apollo*	Puerto de Somiedo, Somiedo		Asturias	Spain	1485
GU669633	EZSPC645-09	RVcoll.07-C024	x	*Papilionidae*	*Parnassius*	*mnemosyne*	Puigmal	Languedoc-Roussillon		France	2050
HM910004	GWORT458-10	BC ZSM Lep 32144		*Papilionidae*	*Parnassius*	*mnemosyne*	Allgaeu	Schwaben	Bavaria	Germany	
HQ004906	EZRMN158-08	RVcoll.08-M246		*Papilionidae*	*Parnassius*	*mnemosyne*	Baile Herculane, Pecinisca	Caras-Severin	Banat	Romania	270
HQ004910	EZROM456-08	RV-06-K661		*Papilionidae*	*Parnassius*	*mnemosyne*	Cetatea Fetei (Cluj-Napoca)	Cluj	Transylvania	Romania	450
HQ004911	EZROM458-08	RV-07-C107		*Papilionidae*	*Parnassius*	*mnemosyne*	Cluj-Napoca, dealul Feleacului, Gheorgheni forest	Cluj	Transylvania	Romania	600
HQ004909	EZROM457-08	RV-07-D136		*Papilionidae*	*Parnassius*	*mnemosyne*	Dumbraveni forest	Constanta	Dobrogea	Romania	80

GenBank accession number	BOLD Process ID	Sample ID	Used in the Iberian dataset	Family	Genus	Species	Exact Site	Region	State/Province	Country	Alt. (m)
HQ004904	EZRMN162-08	RVcoll.08-M629		*Papilionidae*	*Parnassius*	*mnemosyne*	Scorota (Retezat Mts.)	Hunedoara	Transylvania	Romania	1560
HQ004905	EZRMN163-08	RVcoll.08-M642		*Papilionidae*	*Parnassius*	*mnemosyne*	Scorota (Retezat Mts.)	Hunedoara	Transylvania	Romania	1560
HQ004907	EZRMN159-08	RVcoll.08-M274		*Papilionidae*	*Parnassius*	*mnemosyne*	4 Km SW of Cazanele Mari (around Plavisevita)	Mehedinti	Oltenia	Romania	110
HQ004903	EZRMN161-08	RVcoll.08-M417		*Papilionidae*	*Parnassius*	*mnemosyne*	3 km E of Slava Rusa (Babadag forest)	Tulcea	Dobrogea	Romania	130
HQ004908	EZROM646-08	RV-07-D060		*Papilionidae*	*Parnassius*	*mnemosyne*	Horia	Tulcea	Dobrogea	Romania	140
HQ004902	EZRMN160-08	RVcoll.08-M361		*Papilionidae*	*Parnassius*	*mnemosyne*	Bujoreni monastery (9 Km E of Zorleni)	Vaslui	Moldavia	Romania	240
HM901765	EZSPC928-10	RVcoll.09-X000	x	*Papilionidae*	*Parnassius*	*mnemosyne*	Vall de Campcardos, La Cerdanya	Girona	Catalonia	Spain	1700
JF848002	EZSPN059-09	RVcoll.09-V683	x	*Papilionidae*	*Parnassius*	*mnemosyne*	Meranges	Girona	Catalonia	Spain	1750
JF848003	EZSPN060-09	RVcoll.09-V684	x	*Papilionidae*	*Parnassius*	*mnemosyne*	Meranges	Girona	Catalonia	Spain	1750
GU669679	EZSPC594-09	RVcoll.07-W141	x	*Papilionidae*	*Parnassius*	*mnemosyne*	Val d`Hurno, Vall d`Aran	Lleida	Catalonia	Spain	1720
JF847984	EZSPN035-09	RVcoll.09-T076	x	*Papilionidae*	*Parnassius*	*mnemosyne*	Val d`Hurno, Vall d`Aran	Lleida	Catalonia	Spain	1800
GU676615	EZSPN431-09	RVcoll.08-H608	x	*Papilionidae*	*Parnassius*	*mnemosyne*	Vielha, Valle de Hurno	Lleida	Catalonia	Spain	1720
GU676616	EZSPN432-09	RVcoll.08-H609	x	*Papilionidae*	*Parnassius*	*mnemosyne*	Vielha, Valle de Hurno	Lleida	Catalonia	Spain	1720
JF860278	PHLAC726-10	TLMF Lep 02761		*Papilionidae*	*Parnassius*	*phoebus*	Alp Rondadura/ Medel Luc		Graubunden	Switzerland	2200
JF860400	PHLAC932-10	TLMF Lep 02967		*Papilionidae*	*Parnassius*	*phoebus*	Juf/ Avers		Graubunden	Switzerland	2150
JF860401	PHLAC933-10	TLMF Lep 02968		*Papilionidae*	*Parnassius*	*phoebus*	Juf/ Avers		Graubunden	Switzerland	2150
GU707267	FBLMV714-09	BC ZSM Lep 28694		*Lycaenidae*	*Phengaris*	*alcon*	Duggendorf, Girnitztal	Oberpfalz	Bavaria	Germany	375
HQ004706	EZRMN078-08	RVcoll.08-M513		*Lycaenidae*	*Phengaris*	*alcon*	Apuseni Mts., Rimetea (Piatra Secuiului)	Alba	Transylvania	Romania	850
HQ004702	EZROM386-08	RV-06-N013		*Lycaenidae*	*Phengaris*	*alcon*	Apuseni Mts., Rimetea (Piatra Secuiului)	Alba	Transylvania	Romania	750
HQ004705	EZRMN077-08	RVcoll.07-E461		*Lycaenidae*	*Phengaris*	*alcon*	Dumbrava Vadului, Vad	Brasov	Transylvania	Romania	495
HQ004696	EZROM378-08	RV-07-E595		*Lycaenidae*	*Phengaris*	*alcon*	Purcareni (Sacele)	Brasov	Transylvania	Romania	725
HQ004701	EZROM340-08	RV-08-A018		*Lycaenidae*	*Phengaris*	*alcon*	Dealul Istrita, Breaza	Buzau	Muntenia	Romania	525
HQ004700	EZROM346-08	RV-08-A024		*Lycaenidae*	*Phengaris*	*alcon*	Dealul Istrita, Breaza	Buzau	Muntenia	Romania	525
GU675631	EZROM1042-09	RVcoll.07-C928		*Lycaenidae*	*Phengaris*	*alcon*	Baile Herculane	Caras-Severin	Banat	Romania	220
HQ004699	EZROM380-08	RV-07-C927		*Lycaenidae*	*Phengaris*	*alcon*	Baile Herculane (Pecinisca)	Caras-Severin	Banat	Romania	220
HQ004704	EZRMN075-08	RVcoll.06-M886		*Lycaenidae*	*Phengaris*	*alcon*	Cluj-Napoca, dealul Bogomaia	Cluj	Transylvania	Romania	550
HQ004703	EZRMN076-08	RVcoll.07-C158		*Lycaenidae*	*Phengaris*	*alcon*	Cluj-Napoca, Gheorgheni, dealul Feleacului	Cluj	Transylvania	Romania	600
HQ004698	EZROM377-08	RV-06-M892		*Lycaenidae*	*Phengaris*	*alcon*	Cluj-Napoca, dealul Feleacului, Gheorgheni forest	Cluj	Transylvania	Romania	600
HQ004697	EZROM379-08	RV-07-C156		*Lycaenidae*	*Phengaris*	*alcon*	Cluj-Napoca, dealul Feleacului, Gheorgheni forest	Cluj	Transylvania	Romania	600
GU676918	EZSPN515-09	RVcoll.08-H980	x	*Lycaenidae*	*Phengaris*	*alcon*	Carreno, Cabo de Penas		Asturias	Spain	50
GU676921	EZSPN516-09	RVcoll.08-H981	x	*Lycaenidae*	*Phengaris*	*alcon*	Carreno, Cabo de Penas		Asturias	Spain	50
GU676403	EZSPN741-09	RVcoll.08-L163	x	*Lycaenidae*	*Phengaris*	*alcon*	Font del Regatxal, Vilafranca	Castellon	Comunidad Valenciana	Spain	1361
KP870878	EZROM370-08	RVcoll.07-E619	x	*Lycaenidae*	*Phengaris*	*alcon*	Vall d`Ordesa, Osca	Huesca	Aragon	Spain	1275
KP870983	EZSPM791-12	RVcoll.070611MH65	x	*Lycaenidae*	*Phengaris*	*alcon*	Panticosa	Huesca	Aragon	Spain	1214
GU676996	EZSPN223-09	RVcoll.07-F532	x	*Lycaenidae*	*Phengaris*	*alcon*	Valle de Ordesa	Huesca	Aragon	Spain	1275
KP870716	EZSPM805-12	RVcoll.090611SZ07	x	*Lycaenidae*	*Phengaris*	*alcon*	Fuente del Pinarico, Valdelinares	Teruel	Aragon	Spain	1712
KP870289	EULEP059-14	08-J489	x	*Lycaenidae*	*Phengaris*	*alcon*	Cabo Peñas, Luanco		Asturias	Spain	98
KP870639	EULEP060-14	08-J490	x	*Lycaenidae*	*Phengaris*	*alcon*	Cabo Peñas, Luanco		Asturias	Spain	98
KP870946	EULEP061-14	08-J491	x	*Lycaenidae*	*Phengaris*	*alcon*	Cabo Peñas, Luanco		Asturias	Spain	98
KP870427	EULEP062-14	08-J492	x	*Lycaenidae*	*Phengaris*	*alcon*	Cabo Peñas, Luanco		Asturias	Spain	98
KP870501	EULEP063-14	08-J493	x	*Lycaenidae*	*Phengaris*	*alcon*	Cabo Peñas, Luanco		Asturias	Spain	98
KP870963	EULEP201-14	12-L013	x	*Lycaenidae*	*Phengaris*	*alcon*	Cabo Peñas, Gozón		Asturias	Spain	
KP870487	EULEP202-14	12-L014	x	*Lycaenidae*	*Phengaris*	*alcon*	Cabo Peñas, Gozón		Asturias	Spain	
GU688435	GWORO807-09	BC ZSM Lep 30499		*Lycaenidae*	*Phengaris*	*arion*	Samerberg (RO) Zellboden		Bavaria	Germany	950
HM393186	GWORA2456-09	BC ZSM Lep 30668		*Lycaenidae*	*Phengaris*	*arion*	Sudelfeld, Arzmoos	Oberbayern	Bavaria	Germany	1200
GU707270	FBLMV697-09	BC ZSM Lep 28677		*Lycaenidae*	*Phengaris*	*arion*	Duggendorf, Girnitztal	Oberpfalz	Bavaria	Germany	375
HQ004714	EZROM637-08	RV-06-M988		*Lycaenidae*	*Phengaris*	*arion*	Apuseni Mts., Rimetea (Piatra Secuiului)	Alba	Transylvania	Romania	750
HQ004709	EZRMN081-08	RVcoll.08-M566		*Lycaenidae*	*Phengaris*	*arion*	Breaza, dealul Istrita	Buzau	Muntenia	Romania	600
HQ004707	EZRMN079-08	RVcoll.08-M307		*Lycaenidae*	*Phengaris*	*arion*	Suatu	Cluj	Transylvania	Romania	400
HQ004711	EZROM381-08	RV-06-M887		*Lycaenidae*	*Phengaris*	*arion*	Cluj-Napoca, dealul Bogomaia	Cluj	Transylvania	Romania	550
HQ004713	EZROM383-08	RV-07-C135		*Lycaenidae*	*Phengaris*	*arion*	Suatu	Cluj	Transylvania	Romania	380
HQ004708	EZRMN080-08	RVcoll.08-M337		*Lycaenidae*	*Phengaris*	*arion*	1 Km E of Gheorgheni	Harghita	Transylvania	Romania	900
HQ004710	EZRMN082-08	RVcoll.08-M634		*Lycaenidae*	*Phengaris*	*arion*	Scorota (Retezat Mts.)	Hunedoara	Transylvania	Romania	1560

GenBank accession number	BOLD Process ID	Sample ID	Used in the Iberian dataset	Family	Genus	Species	Exact Site	Region	State/Province	Country	Alt. (m)
HQ004712	EZROM382-08	RV-07-D480		*Lycaenidae*	*Phengaris*	*arion*	Cheile Babei	Maramures	Maramures	Romania	265
KP870794	EULEP145-14	10-C430		*Lycaenidae*	*Phengaris*	*arion*	Cheile Bicazului	Neamt	Moldavia	Romania	
KP870769	EULEP185-14	11-J864		*Lycaenidae*	*Phengaris*	*arion*	M-ţii Vrancei, Vârful Ciuta	Vrancea	Moldavia	Romania	
GU676933	EZSPN529-09	RVcoll.08-H999	x	*Lycaenidae*	*Phengaris*	*arion*	Santo Adriano, Desfiladero de las Xanas		Asturias	Spain	300
GU676147	EZSPN1021-09	RVcoll.08-M953	x	*Lycaenidae*	*Phengaris*	*arion*	Forcat	Huesca	Aragon	Spain	1039
KP870851	EZROM371-08	07-E620	x	*Lycaenidae*	*Phengaris*	*arion*	Planes del Son, Son	Lleida	Catalonia	Spain	1440
GU669735	EZSPC523-09	RVcoll.08-R101	x	*Lycaenidae*	*Phengaris*	*arion*	Salt de Pitx, Vall d`Aran	Lleida	Catalonia	Spain	1570
GU669736	EZSPC524-09	RVcoll.08-R102	x	*Lycaenidae*	*Phengaris*	*arion*	Salt de Pitx, Vall d`Aran	Lleida	Catalonia	Spain	1570
GU669737	EZSPC525-09	RVcoll.08-R116	x	*Lycaenidae*	*Phengaris*	*arion*	Salt de Pitx, Vall d`Aran	Lleida	Catalonia	Spain	1570
GU669738	EZSPC526-09	RVcoll.08-R119	x	*Lycaenidae*	*Phengaris*	*arion*	Salt de Pitx, Vall d`Aran	Lleida	Catalonia	Spain	1570
JF847982	EZSPN033-09	RVcoll.09-T072	x	*Lycaenidae*	*Phengaris*	*arion*	Val d`Hurno, Vall d`Aran	Lleida	Catalonia	Spain	1430
GU688438	GWORO806-09	BC ZSM Lep 30498		*Lycaenidae*	*Phengaris*	*nausithous*	Zellwies Koenigsdorf TOEL		Bavaria	Germany	
HM391888	FBLMV483-09	BC ZSM Lep 28463		*Lycaenidae*	*Phengaris*	*nausithous*	Saulburg	Niederbayern	Bavaria	Germany	360
HQ004718	EZRMN083-08	RVcoll.06-M917		*Lycaenidae*	*Phengaris*	*nausithous*	Cluj-Napoca, dl. Bogomaia	Cluj	Transylvania	Romania	550
HQ004717	EZRMN084-08	RVcoll.07-C164		*Lycaenidae*	*Phengaris*	*nausithous*	Fanatele Clujului (dealul Bogomaia), Cluj-Napoca	Cluj	Transylvania	Romania	480
HQ004716	EZRMN085-08	RVcoll.07-C165		*Lycaenidae*	*Phengaris*	*nausithous*	Fanatele Clujului (dealul Bogomaia), Cluj-Napoca	Cluj	Transylvania	Romania	480
GU675624	EZROM1035-09	RVcoll.06-M890		*Lycaenidae*	*Phengaris*	*nausithous*	Cluj-Napoca, dealul Bogomaia	Cluj	Transylvania	Romania	550
GU675623	EZROM1038-09	RVcoll.07-C161		*Lycaenidae*	*Phengaris*	*nausithous*	Fanatele Clujului (dealul Bogomaia), Cluj-Napoca	Cluj	Transylvania	Romania	480
GU675622	EZROM1039-09	RVcoll.07-C167		*Lycaenidae*	*Phengaris*	*nausithous*	Fanatele Clujului (dealul Bogomaia), Cluj-Napoca	Cluj	Transylvania	Romania	480
HQ004719	EZROM384-08	RV-06-M889		*Lycaenidae*	*Phengaris*	*nausithous*	Cluj-Napoca, dealul Bogomaia	Cluj	Transylvania	Romania	550
HQ004720	EZROM385-08	RV-07-C166		*Lycaenidae*	*Phengaris*	*nausithous*	Fanatele Clujului (dealul Bogomaia), Cluj-Napoca	Cluj	Transylvania	Romania	480
HQ004715	EZRMN086-08	RVcoll.07-C334		*Lycaenidae*	*Phengaris*	*nausithous*	Radauti	Suceava	Moldavia	Romania	375
HQ004721	EZROM585-08	RV-07-C331		*Lycaenidae*	*Phengaris*	*nausithous*	Radauti	Suceava	Moldavia	Romania	375
HQ004722	EZROM586-08	RV-07-C332		*Lycaenidae*	*Phengaris*	*nausithous*	Radauti	Suceava	Moldavia	Romania	375
HM901302	EZSPC1101-10	RVcoll.08-L971	x	*Lycaenidae*	*Phengaris*	*nausithous*	Oteruelo, Rascafria		Comunidad de Madrid	Spain	1150
HM901347	EZSPC1147-10	RVcoll.08-P775	x	*Lycaenidae*	*Phengaris*	*nausithous*	Besande	Leon	Castilla y Leon	Spain	1237
HM901385	EZSPC1185-10	RVcoll.09-T172	x	*Lycaenidae*	*Phengaris*	*nausithous*	Valverde de la Sierra	Leon	Castilla y Leon	Spain	1322
GU675940	EZSPM294-09	RVcoll.08-J834	x	*Lycaenidae*	*Phengaris*	*nausithous*	La Una	Leon	Castilla y Leon	Spain	
GU676914	EZSPN511-09	RVcoll.08-H971	x	*Lycaenidae*	*Phengaris*	*nausithous*	Matabuena	Segovia	Castilla y Leon	Spain	1200
GU676917	EZSPN512-09	RVcoll.08-H972	x	*Lycaenidae*	*Phengaris*	*nausithous*	Matabuena	Segovia	Castilla y Leon	Spain	1200
GU676226	EZSPN928-09	RVcoll.08-L883	x	*Lycaenidae*	*Phengaris*	*nausithous*	Abejar	Soria	Castilla y Leon	Spain	1110
GU676227	EZSPN929-09	RVcoll.08-L884	x	*Lycaenidae*	*Phengaris*	*nausithous*	Abejar	Soria	Castilla y Leon	Spain	1110
GU688436	GWORO808-09	BC ZSM Lep 30500		*Lycaenidae*	*Phengaris*	*teleius*	Obertraubing Tutzing STA		Bavaria	Germany	
HM391889	FBLMV484-09	BC ZSM Lep 28464		*Lycaenidae*	*Phengaris*	*teleius*	Saulburg	Niederbayern	Bavaria	Germany	360
HQ957211	GWORA2457-09	BC ZSM Lep 30669		*Lycaenidae*	*Phengaris*	*teleius*	Passau, near Egling	Niederbayern	Bavaria	Germany	
JF415712	GWORK517-09	BC ZSM Lep 21847		*Lycaenidae*	*Phengaris*	*teleius*	Diessen	Oberbayern	Bavaria	Germany	600
HM910003	GWORT457-10	BC ZSM Lep 32143		*Lycaenidae*	*Phengaris*	*teleius*	Starnberg, Machtlfing	Oberbayern	Bavaria	Germany	715
HQ004727	EZROM388-08	RV-06-M891		*Lycaenidae*	*Phengaris*	*teleius*	Padurea Craiului Mts., Suncuius	Bihor	Transylvania	Romania	370
HQ004728	EZROM389-08	RV-07-E460		*Lycaenidae*	*Phengaris*	*teleius*	Dumbrava Vadului, Vad	Brasov	Transylvania	Romania	495
HQ004729	EZROM679-08	RV-06-M964		*Lycaenidae*	*Phengaris*	*teleius*	Racos	Brasov	Transylvania	Romania	500
HQ004725	EZRMN087-08	RVcoll.07-C163		*Lycaenidae*	*Phengaris*	*teleius*	Fanatele Clujului (dealul Bogomaia), Cluj-Napoca	Cluj	Transylvania	Romania	480
HQ004724	EZRMN088-08	RVcoll.07-C168		*Lycaenidae*	*Phengaris*	*teleius*	Fanatele Clujului (dealul Bogomaia), Cluj-Napoca	Cluj	Transylvania	Romania	480
HQ004723	EZRMN089-08	RVcoll.06-M885		*Lycaenidae*	*Phengaris*	*teleius*	Cluj-Napoca, dealul Bogomaia	Cluj	Transylvania	Romania	550
HQ004726	EZROM387-08	RV-07-C162		*Lycaenidae*	*Phengaris*	*teleius*	Fanatele Clujului (dealul Bogomaia), Cluj-Napoca	Cluj	Transylvania	Romania	480
HM393181	GWORA2448-09	BC ZSM Lep 30660		*Pieridae*	*Pieris*	*brassicae*	Ascha	Niederbayern	Bavaria	Germany	340
HM393182	GWORA2449-09	BC ZSM Lep 30661		*Pieridae*	*Pieris*	*brassicae*	Hausmetting	Niederbayern	Bavaria	Germany	370
JF415722	GWORK291-09	BC ZSM Lep 21526		*Pieridae*	*Pieris*	*brassicae*	Gaden, Isarauen	Oberbayern	Bavaria	Germany	428
GU707083	FBLMU454-09	BC ZSM Lep 27104		*Pieridae*	*Pieris*	*brassicae*	Duggendorf, Girnitztal	Oberpfalz	Bavaria	Germany	375

GenBank accession number	BOLD Process ID	Sample ID	Used in the Iberian dataset	Family	Genus	Species	Exact Site	Region	State/Province	Country	Alt. (m)
KP870372	EZSPM739-12	RVcoll.12-L588	x	*Pieridae*	*Pieris*	*brassicae*	Hotel Don Tenorio, Vila do Bispo		Algarve	Portugal	
KP870572	EZSPM740-12	RVcoll.12-L589	x	*Pieridae*	*Pieris*	*brassicae*	Hotel Don Tenorio, Vila do Bispo		Algarve	Portugal	
GU676568	EZSPN577-09	RVcoll.08-J124	x	*Pieridae*	*Pieris*	*brassicae*	Seculca, Beca, Boticas		Tras-os-Montes	Portugal	790
HQ004917	EZROM647-08	RV-07-D357		*Pieridae*	*Pieris*	*brassicae*	Racos	Brasov	Transylvania	Romania	460
HQ004914	EZROM293-08	RV-07-E655		*Pieridae*	*Pieris*	*brassicae*	Dealul Istrita, Breaza	Buzau	Muntenia	Romania	525
HQ004918	EZROM307-08	RV-07-E669		*Pieridae*	*Pieris*	*brassicae*	Dealul Istrita, Breaza	Buzau	Muntenia	Romania	525
HQ004916	EZROM460-08	RV-07-C969		*Pieridae*	*Pieris*	*brassicae*	Baile Herculane (Pecinisca)	Caras-Severin	Banat	Romania	220
KP870244	EULEP022-14	07-D470		*Pieridae*	*Pieris*	*brassicae*	Câțcău	Cluj	Transylvania	Romania	255
HQ004915	EZROM459-08	RV-06-M876		*Pieridae*	*Pieris*	*brassicae*	Cluj-Napoca, dealul Feleacului, Gheorgheni forest	Cluj	Transylvania	Romania	600
HQ004912	EZRMN164-08	RVcoll.08-M393		*Pieridae*	*Pieris*	*brassicae*	Macin Mts. (3 Km S of Jijila)	Tulcea	Dobrogea	Romania	230
HQ004913	EZRMN165-08	RVcoll.08-M422		*Pieridae*	*Pieris*	*brassicae*	3 km E of Slava Rusa (Babadag forest)	Tulcea	Dobrogea	Romania	130
FJ663921	LOWA754-06	2005-LOWA-754		*Pieridae*	*Pieris*	*brassicae*	Osyno	Sebezh distr.	Pskov	Russia	
FJ663920	LOWA755-06	2005-LOWA-755		*Pieridae*	*Pieris*	*brassicae*	Osyno	Sebezh distr.	Pskov	Russia	
GU675843	EZSPM279-09	SMcoll.150308PP65	x	*Pieridae*	*Pieris*	*brassicae*	La Serrella - Aitana, Confrides	Alicante	Comunidad Valenciana	Spain	1178
GU676316	EZSPN839-09	RVcoll.08-L667	x	*Pieridae*	*Pieris*	*brassicae*	Candeleda, Cuesta de la Joya	Avila	Castilla y Leon	Spain	415
JN827887	EZROM374-08	07-E602	x	*Pieridae*	*Pieris*	*brassicae*	Vallforners, Canoves	Barcelona	Catalonia	Spain	650
GU669675	EZSPC595-09	RVcoll.08-L513	x	*Pieridae*	*Pieris*	*brassicae*	El Cafe, pla de la Calma, El Brull, Osona	Barcelona	Catalonia	Spain	1192
GU669676	EZSPC596-09	RVcoll.08-M703	x	*Pieridae*	*Pieris*	*brassicae*	Masia del Plans, Vidra, La Garrotxa	Barcelona	Catalonia	Spain	1370
GU669678	EZSPC598-09	RVcoll.08-P071	x	*Pieridae*	*Pieris*	*brassicae*	Torrent de Ridolaina, Cerdanya	Girona	Catalonia	Spain	1060
GU676136	EZSPN1024-09	RVcoll.08-M956	x	*Pieridae*	*Pieris*	*brassicae*	Forcat	Huesca	Aragon	Spain	1039
GU669677	EZSPC597-09	RVcoll.08-J345	x	*Pieridae*	*Pieris*	*brassicae*	Coll de Comiols, Pallars Jussa	Lleida	Catalonia	Spain	1130
KP870390	EZSPM619-12	RVcoll.11-D877	x	*Pieridae*	*Pieris*	*brassicae*	Frigiliana	Malaga	Andalusia	Spain	433
KP870280	EZSPM1003-12	Rvcoll.12-L889	x	*Pieridae*	*Pieris*	*brassicae*	Turdonell de Dalt, Mao, Menorca	Menorca	Balearic Islands	Spain	
KP870553	EZSPM1007-12	Rvcoll.12-L896	x	*Pieridae*	*Pieris*	*brassicae*	San Bernat, Alaior, Menorca	Menorca	Balearic Islands	Spain	
KP870474	EZSPM999-12	Rvcoll.12-L885	x	*Pieridae*	*Pieris*	*brassicae*	Sant Antoni de Ruma, Ferreries, Menorca	Menorca	Balearic Islands	Spain	
GU676737	EZSPN300-09	RVcoll.08-H104	x	*Pieridae*	*Pieris*	*brassicae*	Na Vermella, Mao	Menorca	Balearic Islands	Spain	
GU675846	EZSPM232-09	RVcoll.08-R428	x	*Pieridae*	*Pieris*	*brassicae*	Oia, As Marinas-Mougas	Pontevedra	Galicia	Spain	20
GU676667	EZSPN373-09	RVcoll.08-H399	x	*Pieridae*	*Pieris*	*brassicae*	Serra, Serra	Valencia	Comunidad Valenciana	Spain	
GU707090	FBLMU449-09	BC ZSM Lep 27099		*Pieridae*	*Pieris*	*bryoniae*	Inning am Ammersee, Martinsberg b. Inning	Oberbayern	Bavaria	Germany	585
GU686975	GWORL288-09	BC ZSM Lep 22000		*Pieridae*	*Pieris*	*bryoniae*	Wettersteingebirge, Kreuzeck-Stuibensee	Oberbayern	Bavaria	Germany	1600
HQ004920	EZRMN171-08	RVcoll.08-M609		*Pieridae*	*Pieris*	*bryoniae*	3 km below cabana Capra (Fagaras Mts.)	Arges	Muntenia	Romania	1350
HQ004919	EZRMN172-08	RVcoll.08-M616		*Pieridae*	*Pieris*	*bryoniae*	Cabana Capra (Fagaras Mts.)	Arges	Muntenia	Romania	1630
HQ004925	EZRMN166-08	RVcoll.06-M968		*Pieridae*	*Pieris*	*bryoniae*	Ciucas Mts. (towards Vf. Ciucas)	Prahova	Muntenia	Romania	1700
HQ004924	EZRMN167-08	RVcoll.06-M969		*Pieridae*	*Pieris*	*bryoniae*	Ciucas Mts. (towards Vf. Ciucas)	Prahova	Muntenia	Romania	1700
HQ004923	EZRMN168-08	RVcoll.06-M991		*Pieridae*	*Pieris*	*bryoniae*	Ciucas Mts. (towards Vf. Ciucas)	Prahova	Muntenia	Romania	1700
HQ004922	EZRMN169-08	RVcoll.06-N014		*Pieridae*	*Pieris*	*bryoniae*	Ciucas Mts. (towards Vf. Ciucas)	Prahova	Muntenia	Romania	1700
HQ004921	EZRMN170-08	RVcoll.08-M496		*Pieridae*	*Pieris*	*bryoniae*	Cota 1400 m (Bucegi Mts.), Sinaia	Prahova	Muntenia	Romania	1385
HQ004926	EZROM461-08	RV-06-M972		*Pieridae*	*Pieris*	*bryoniae*	Ciucas Mts. (towards Vf. Ciucas)	Prahova	Muntenia	Romania	1650
HQ004927	EZROM462-08	RV-06-M997		*Pieridae*	*Pieris*	*bryoniae*	Ciucas Mts. (towards Vf. Ciucas)	Prahova	Muntenia	Romania	1650
HQ004928	EZROM683-08	RV-06-M978		*Pieridae*	*Pieris*	*bryoniae*	Ciucas Mts. (towards Vf. Ciucas)	Prahova	Muntenia	Romania	1700
JF847987	EZSPN038-09	RVcoll.09-T095	x	*Pieridae*	*Pieris*	*ergane*	Pico Oroel, Jaca	Huesca	Aragon	Spain	1660
JF847988	EZSPN039-09	RVcoll.09-T096	x	*Pieridae*	*Pieris*	*ergane*	Pico Oroel, Jaca	Huesca	Aragon	Spain	1660
GU676854	EZSPC599-09	RVcoll.08-L528	x	*Pieridae*	*Pieris*	*ergane*	Barranc d`Enseu, Gerri de la Sal, Pallars Sobira	Lleida	Catalonia	Spain	605
GU676855	EZSPC600-09	RVcoll.08-L529	x	*Pieridae*	*Pieris*	*ergane*	Barranc d`Enseu, Gerri de la Sal, Pallars Sobira	Lleida	Catalonia	Spain	605
GU676856	EZSPC601-09	RVcoll.08-L530	x	*Pieridae*	*Pieris*	*ergane*	Barranc d`Enseu, Gerri de la Sal, Pallars Sobira	Lleida	Catalonia	Spain	605
JN276230	EZSPC1260-10	RVcoll.220602AT3	x	*Pieridae*	*Pieris*	*ergane*	Abejuela,Abejuela	Teruel	Aragon	Spain	
KP870437	EZSPM821-12	RVcoll.140410KV39	x	*Pieridae*	*Pieris*	*ergane*	Abejuela	Teruel	Aragon	Spain	1121
GU676850	EZSPN082-09	RVcoll.08-J653	x	*Pieridae*	*Pieris*	*ergane*	Barranco del Toro	Teruel	Aragon	Spain	1443
HQ004931	EZRMN173-08	RVcoll.08-M248		*Pieridae*	*Pieris*	*mannii*	Baile Herculane, Pecinisca	Caras-Severin	Banat	Romania	270
HQ004933	EZRMN176-08	RVcoll.08-M544		*Pieridae*	*Pieris*	*mannii*	Valea Feregarilor - Poiana Musuroaie	Caras-Severin	Banat	Romania	350
HQ004934	EZRMN177-08	RVcoll.08-M547		*Pieridae*	*Pieris*	*mannii*	Valea Feregarilor - Poiana Musuroaie	Caras-Severin	Banat	Romania	350

GenBank accession number	BOLD Process ID	Sample ID	Used in the Iberian dataset	Family	Genus	Species	Exact Site	Region	State/Province	Country	Alt. (m)
HQ004937	EZROM463-08	RV-07-C956		*Pieridae*	*Pieris*	*mannii*	Baile Herculane (Pecinisca)	Caras-Severin	Banat	Romania	220
HQ004938	EZROM464-08	RV-07-C963		*Pieridae*	*Pieris*	*mannii*	Baile Herculane (Pecinisca)	Caras-Severin	Banat	Romania	220
HQ004936	EZROM648-08	RV-07-E356		*Pieridae*	*Pieris*	*mannii*	Baile Herculane (Pecinisca)	Caras-Severin	Banat	Romania	270
HQ004935	EZROM699-08	RV-07-F563		*Pieridae*	*Pieris*	*mannii*	Baile Herculane (Pecinisca)	Caras-Severin	Banat	Romania	270
HQ004930	EZRMN175-08	RVcoll.08-M529		*Pieridae*	*Pieris*	*mannii*	Tismana monastery	Gorj	Oltenia	Romania	230
HQ004929	EZRMN178-08	RVcoll.08-M647		*Pieridae*	*Pieris*	*mannii*	Valea Mielului (Retezat Mts.)	Hunedoara	Transylvania	Romania	1050
HQ004932	EZRMN174-08	RVcoll.08-M272		*Pieridae*	*Pieris*	*mannii*	road E70, 6 Km W of Turnu-Severin	Mehedinti	Oltenia	Romania	45
HM901797	EZSPC962-10	RVcoll.150308PP11	x	*Pieridae*	*Pieris*	*mannii*	La Serrella - Aitana, Confrides	Alicante	Comunidad Valenciana	Spain	1178
HM901798	EZSPC963-10	RVcoll.150308PP15	x	*Pieridae*	*Pieris*	*mannii*	La Serrella - Aitana, Confrides	Alicante	Comunidad Valenciana	Spain	1178
GU675879	EZSPM357-09	RVcoll.08-R053	x	*Pieridae*	*Pieris*	*mannii*	Barrancada	Alicante	Comunidad Valenciana	Spain	817
KP870793	EZROM746-08	RV-06-V759	x	*Pieridae*	*Pieris*	*mannii*	Cal Cerdanyola, Gisclareny	Barcelona	Catalonia	Spain	940
GU669674	EZSPC602-09	RVcoll.07-Z067	x	*Pieridae*	*Pieris*	*mannii*	Montjoi, Roses, ALT EMPORDA	Girona	Catalonia	Spain	70
HM901748	EZSPC910-10	RVcoll.09-V801	x	*Pieridae*	*Pieris*	*mannii*	Cantallops, Alt Emporda	Girona	Catalonia	Spain	225
HM901776	EZSPC939-10	RVcoll.09-X050	x	*Pieridae*	*Pieris*	*mannii*	Fontllebrera, La Cerdanya	Girona	Catalonia	Spain	1530
HM901622	EZSPC767-10	RVcoll.08-L677	x	*Pieridae*	*Pieris*	*mannii*	Iznalloz, Sierra Arana	Granada	Andalusia	Spain	1306
HM901623	EZSPC768-10	RVcoll.08-L678	x	*Pieridae*	*Pieris*	*mannii*	Iznalloz, Sierra Arana	Granada	Andalusia	Spain	1306
GU676312	EZSPN843-09	RVcoll.08-L676	x	*Pieridae*	*Pieris*	*mannii*	Iznalloz, Sierra Arana	Granada	Andalusia	Spain	1306
KP870775	EZSPN066-09	RVcoll.09-V744	x	*Pieridae*	*Pieris*	*mannii*	path to Embalse Respomuso, Sallent de Gallego	Huesca	Aragon	Spain	1650
JN276234	EZSPN067-09	RVcoll.09-V745	x	*Pieridae*	*Pieris*	*mannii*	path to Embalse Respomuso, Sallent de Gallego	Huesca	Aragon	Spain	1650
JN276235	EZSPN068-09	RVcoll.09-V748	x	*Pieridae*	*Pieris*	*mannii*	path to Embalse Respomuso, Sallent de Gallego	Huesca	Aragon	Spain	1650
KP870867	EZSPM762-12	RVcoll.030111FB42	x	*Pieridae*	*Pieris*	*mannii*	Pista Abejuela-Manzanera	Teruel	Aragon	Spain	1121
GU676369	EZSPN781-09	RVcoll.08-L294	x	*Pieridae*	*Pieris*	*mannii*	Paraisos Altos, Manzanera	Teruel	Aragon	Spain	1200
JN276231	EZSPC1263-10	RVcoll.060604EM1	x	*Pieridae*	*Pieris*	*mannii*	El Moragete,Jalance	Valencia	Comunidad Valenciana	Spain	
JN276233	EZSPC1324-10	RVcoll.130209KL97	x	*Pieridae*	*Pieris*	*mannii*	Muela de Cortes,Cortes de Pallas	Valencia	Comunidad Valenciana	Spain	
KP870494	EZSPM852-12	RVcoll.201210GK94	x	*Pieridae*	*Pieris*	*mannii*	Rio Cantaban, Jalance	Valencia	Comunidad Valenciana	Spain	374
GU676935	EZSPN531-09	RVcoll.08-J006	x	*Pieridae*	*Pieris*	*mannii*	Cortes de Pallas (Sierra Martes), Cortes de Pallas	Valencia	Comunidad Valenciana	Spain	
GU688503	GWORO778-09	BC ZSM Lep 30470		*Pieridae*	*Pieris*	*napi*	Traunstein		Bavaria	Germany	
GU676577	EZSPN562-09	RVcoll.08-J101	x	*Pieridae*	*Pieris*	*napi*	Moinho do Moleiro (Angeiras), Lavra, Matosinhos		Douro Litoral	Portugal	10
HQ004952	EZRMN179-08	RVcoll.08-M212		*Pieridae*	*Pieris*	*napi*	3 Km SW of Lunca Corbului	Arges	Muntenia	Romania	235
HQ004942	EZROM590-08	RV-07-C315		*Pieridae*	*Pieris*	*napi*	Oraseni Vale	Botosani	Moldavia	Romania	150
HQ004946	EZROM468-08	RV-07-D261		*Pieridae*	*Pieris*	*napi*	Dumbrava Vadului, Vad	Brasov	Transylvania	Romania	495
HQ004948	EZROM469-08	RV-07-D091		*Pieridae*	*Pieris*	*napi*	Dealul Istrita, Breaza	Buzau	Muntenia	Romania	540
HQ004943	EZROM471-08	RV-07-D101		*Pieridae*	*Pieris*	*napi*	Ciolanu	Buzau	Muntenia	Romania	255
HQ004951	EZRMN180-08	RVcoll.08-M243		*Pieridae*	*Pieris*	*napi*	Baile Herculane, Pecinisca	Caras-Severin	Banat	Romania	270
HQ004947	EZROM465-08	RV-07-C989		*Pieridae*	*Pieris*	*napi*	Baile Herculane (Pecinisca)	Caras-Severin	Banat	Romania	220
HQ004939	EZROM700-08	RV-07-F564		*Pieridae*	*Pieris*	*napi*	Baile Herculane (Pecinisca)	Caras-Severin	Banat	Romania	270
HQ004944	EZROM466-08	RV-06-K568		*Pieridae*	*Pieris*	*napi*	Badeni	Cluj	Transylvania	Romania	440
HQ004945	EZROM467-08	RV-06-K569		*Pieridae*	*Pieris*	*napi*	Badeni	Cluj	Transylvania	Romania	440
HQ004949	EZROM470-08	RV-07-D027		*Pieridae*	*Pieris*	*napi*	Canaraua Fetei (Baneasa)	Constanta	Dobrogea	Romania	15
GU669657	EZROM1054-09	RVcoll.07-D979		*Pieridae*	*Pieris*	*napi*	4 km west of I. L. Caragiale	Dambovita	Muntenia	Romania	270
HQ004941	EZROM650-08	RV-07-D929		*Pieridae*	*Pieris*	*napi*	Pestera Muierii, Baia de Fier	Gorj	Oltenia	Romania	640
HQ004940	EZROM649-08	RV-07-D544		*Pieridae*	*Pieris*	*napi*	Rohia	Maramures	Maramures	Romania	360
GU669645	EZROM1071-09	RVcoll.08-L363		*Pieridae*	*Pieris*	*napi*	4 Km W of Drobeta Turnu-Severin	Mehedinti	Oltenia	Romania	75
HQ004950	EZROM472-08	RV-07-E530		*Pieridae*	*Pieris*	*napi*	Slaveni	Olt	Oltenia	Romania	65
GU669665	EZROM1041-09	RVcoll.07-C327		*Pieridae*	*Pieris*	*napi*	Muntii Calimani, Poiana Negrii, Valea Pintii	Suceava	Moldavia	Romania	1100
GU669663	EZROM1044-09	RVcoll.07-D065		*Pieridae*	*Pieris*	*napi*	Greci massif, Macin Mts.	Tulcea	Dobrogea	Romania	220

GenBank accession number	BOLD Process ID	Sample ID	Used in the Iberian dataset	Family	Genus	Species	Exact Site	Region	State/Province	Country	Alt. (m)
GU669648	EZROM1059-09	RVcoll.07-E442		*Pieridae*	*Pieris*	*napi*	2 km E of Smardan	Tulcea	Dobrogea	Romania	5
FJ663932	LOWA759-06	2005-LOWA-759		*Pieridae*	*Pieris*	*napi*	Bogorodizk		Tula	Russia	
FJ663931	LOWA760-06	2005-LOWA-760		*Pieridae*	*Pieris*	*napi*	Bogorodizk		Tula	Russia	
GU675699	EZSPM146-09	RVcoll.08-P659	x	*Pieridae*	*Pieris*	*napi*	Valle (Valle-Zurea), Lena		Asturias	Spain	662
KP870449	EZSPM875-12	Rvcoll.130711PX61	x	*Pieridae*	*Pieris*	*napi*	Alrededores de Tineo		Asturias	Spain	252
KP870441	EZSPM877-12	Rvcoll.130711PX87	x	*Pieridae*	*Pieris*	*napi*	Rio Muniellos, Cangas del Narcea		Asturias	Spain	594
GU676297	EZSPN861-09	RVcoll.08-L729	x	*Pieridae*	*Pieris*	*napi*	Campo Real		Comunidad de Madrid	Spain	729
GU676272	EZSPN883-09	RVcoll.08-L792	x	*Pieridae*	*Pieris*	*napi*	Brana Vieja		Cantabria	Spain	1408
GU675842	EZSPM233-09	RVcoll.08-R430	x	*Pieridae*	*Pieris*	*napi*	Ames, Novais	A Coruna	Galicia	Spain	160
GU676613	EZSPN429-09	RVcoll.08-H605	x	*Pieridae*	*Pieris*	*napi*	Candeleda, La Lagunilla	Avila	Castilla y Leon	Spain	380
KP871037	EZROM747-08	RV-07-F503	x	*Pieridae*	*Pieris*	*napi*	Viladrau	Barcelona	Catalonia	Spain	740
GU669671	EZSPC604-09	RVcoll.06-H857	x	*Pieridae*	*Pieris*	*napi*	Vallforners, Canoves, Valles Oriental	Barcelona	Catalonia	Spain	650
GU676889	EZSPN484-09	RVcoll.08-H921	x	*Pieridae*	*Pieris*	*napi*	Hervas, Carretera Hervas-La Garganta	Caceres	Extremadura	Spain	774
GU676888	EZSPN485-09	RVcoll.08-H922	x	*Pieridae*	*Pieris*	*napi*	Hervas, Carretera Hervas-La Garganta	Caceres	Extremadura	Spain	774
GU669673	EZSPC606-09	RVcoll.07-C673	x	*Pieridae*	*Pieris*	*napi*	Sant Marti de Puigcerda	Girona	Catalonia	Spain	1145
GU669668	EZSPC607-09	RVcoll.08-P007	x	*Pieridae*	*Pieris*	*napi*	Torre de Riu, Ribera d`Alp, Cerdanya	Girona	Catalonia	Spain	1180
GU676098	EZSPM442-09	Rvcoll.09-V598	x	*Pieridae*	*Pieris*	*napi*	Seminario (Sierra Nevada)	Granada	Andalusia	Spain	1520
GU675863	EZSPM374-09	RVcoll.08-J861	x	*Pieridae*	*Pieris*	*napi*	Fresno de la Vega	Leon	Castilla y Leon	Spain	
GU669672	EZSPC605-09	RVcoll.08-P376	x	*Pieridae*	*Pieris*	*napi*	Arros, Vall d`Aran	Lleida	Catalonia	Spain	906
KP870286	EZSPM861-12	Rvcoll.050111LP92	x	*Pieridae*	*Pieris*	*napi*	Ruta de los Molinos, Viveiro	Lugo	Galicia	Spain	32
GU675839	EZSPM234-09	RVcoll.08-R432	x	*Pieridae*	*Pieris*	*napi*	Oia, As Marinas-Mougas	Pontevedra	Galicia	Spain	20
GU675701	EZSPM166-09	RVcoll.08-R001	x	*Pieridae*	*Pieris*	*napi*	Paraisos Altos, Manzanera	Teruel	Aragon	Spain	1200
GU675893	EZSPM343-09	RVcoll.08-R004	x	*Pieridae*	*Pieris*	*napi*	Paraisos Altos, Manzanera	Teruel	Aragon	Spain	1200
GU675703	EZSPM174-09	RVcoll.08-R037	x	*Pieridae*	*Pieris*	*napi*	Casas de Barchell, Chelva	Valencia	Comunidad Valenciana	Spain	
FJ663941	LOWA285-06	2005-LOWA-285		*Pieridae*	*Pieris*	*pseudorapae*	Kislovodsk	Stavropol Region	Stavropol	Russia	
FJ663940	LOWA286-06	2005-LOWA-286		*Pieridae*	*Pieris*	*pseudorapae*	Kislovodsk	Stavropol Region	Stavropol	Russia	
HQ957208	GWORA2446-09	BC ZSM Lep 30658		*Pieridae*	*Pieris*	*rapae*	Bergstorf	Niederbayern	Bavaria	Germany	330
JF415723	GWORK293-09	BC ZSM Lep 21528		*Pieridae*	*Pieris*	*rapae*	Bahndamm zwischen Markt Schwaben und Poing	Oberbayern	Bavaria	Germany	520
KP871016	EZSPM741-12	RVcoll.12-L595	x	*Pieridae*	*Pieris*	*rapae*	Caldas de Monchique, Monchique		Algarve	Portugal	
KP871086	EZSPM742-12	RVcoll.12-L549	x	*Pieridae*	*Pieris*	*rapae*	Porches, Lagoa		Algarve	Portugal	
KP870939	EZSPM743-12	RVcoll.12-L550	x	*Pieridae*	*Pieris*	*rapae*	Porches, Lagoa		Algarve	Portugal	
KP870868	EZSPM744-12	RVcoll.12-L612	x	*Pieridae*	*Pieris*	*rapae*	Rasmalho, Portimao		Algarve	Portugal	
GU676578	EZSPN563-09	RVcoll.08-J103	x	*Pieridae*	*Pieris*	*rapae*	Moinho do Moleiro (Angeiras), Lavra, Matosinhos		Douro Litoral	Portugal	10
GU676560	EZSPN581-09	RVcoll.08-J138	x	*Pieridae*	*Pieris*	*rapae*	Curalha (Rio Tamega), Chaves		Tras-os-Montes	Portugal	350
HQ004955	EZROM475-08	RV-07-D108		*Pieridae*	*Pieris*	*rapae*	Nenciulesti	Buzau	Muntenia	Romania	360
HQ004957	EZROM476-08	RV-07-D209		*Pieridae*	*Pieris*	*rapae*	Dealul Istrita, Breaza	Buzau	Muntenia	Romania	540
HQ004958	EZROM477-08	RV-07-C973		*Pieridae*	*Pieris*	*rapae*	Baile Herculane (Pecinisca)	Caras-Severin	Banat	Romania	220
HQ004956	EZROM473-08	RV-06-K542		*Pieridae*	*Pieris*	*rapae*	Badeni	Cluj	Transylvania	Romania	440
HQ004954	EZROM474-08	RV-06-K650		*Pieridae*	*Pieris*	*rapae*	Baciu forest (Cluj-Napoca)	Cluj	Transylvania	Romania	530
HQ004960	EZRMN182-08	RVcoll.08-M336		*Pieridae*	*Pieris*	*rapae*	1 Km E of Gheorgheni	Harghita	Transylvania	Romania	900
HQ004962	EZRMN184-08	RVcoll.08-M636		*Pieridae*	*Pieris*	*rapae*	Scorota (Retezat Mts.)	Hunedoara	Transylvania	Romania	1560
HQ004959	EZRMN181-08	RVcoll.08-M241		*Pieridae*	*Pieris*	*rapae*	7 Km E of Strehaia	Mehedinti	Oltenia	Romania	125
HQ004953	EZROM591-08	RV-07-C356		*Pieridae*	*Pieris*	*rapae*	Sucevita	Suceava	Moldavia	Romania	900
HQ004961	EZRMN183-08	RVcoll.08-M412		*Pieridae*	*Pieris*	*rapae*	Dealul Consul	Tulcea	Dobrogea	Romania	80
FJ663945	LOWA160-06	2005-LOWA-160		*Pieridae*	*Pieris*	*rapae*	Osyno vill.	Sebezh distr	Pskov	Russia	150
FJ663944	LOWA161-06	2005-LOWA-161		*Pieridae*	*Pieris*	*rapae*	Osyno vill.	Sebezh distr	Pskov	Russia	150
HM901724	EZSPC884-10	RVcoll.09-T147	x	*Pieridae*	*Pieris*	*rapae*	Valdelaguna		Comunidad de Madrid	Spain	710
HM901725	EZSPC885-10	RVcoll.09-T148	x	*Pieridae*	*Pieris*	*rapae*	Valdelaguna		Comunidad de Madrid	Spain	710
GU675686	EZSPM136-09	RVcoll.08-P646	x	*Pieridae*	*Pieris*	*rapae*	Valle (Valle-Zurea), Lena		Asturias	Spain	662

GenBank accession number	BOLD Process ID	Sample ID	Used in the Iberian dataset	Family	Genus	Species	Exact Site	Region	State/Province	Country	Alt. (m)
GU676659	EZSPN377-09	RVcoll.08-H414	x	*Pieridae*	*Pieris*	*rapae*	Cantoblanco		Comunidad de Madrid	Spain	720
KP870909	EZSPM885-12	Rvcoll.12-M603	x	*Pieridae*	*Pieris*	*rapae*	Medellin	Badajoz	Extremadura	Spain	240
KP870897	EZSPM886-12	Rvcoll.12-M604	x	*Pieridae*	*Pieris*	*rapae*	Medellin	Badajoz	Extremadura	Spain	240
JN827888	EZROM748-08	RV-06-H864	x	*Pieridae*	*Pieris*	*rapae*	Vallforners, Canoves	Barcelona	Catalonia	Spain	650
GU669670	EZSPC603-09	RVcoll.08-L455	x	*Pieridae*	*Pieris*	*rapae*	La Llavina, Montseny, Valles Oriental	Barcelona	Catalonia	Spain	500
GU669669	EZSPC608-09	RVcoll.06-G428	x	*Pieridae*	*Pieris*	*rapae*	El Burgues, Coll Formic, Montseny	Barcelona	Catalonia	Spain	1000
GU669619	EZSPC663-09	RVcoll.08-J362	x	*Pieridae*	*Pieris*	*rapae*	Vallgrassa, Parc Natural del Garraf	Barcelona	Catalonia	Spain	285
GU669620	EZSPC664-09	RVcoll.08-J377	x	*Pieridae*	*Pieris*	*rapae*	Turo del Fumet, La Floresta, Valles Occidental	Barcelona	Catalonia	Spain	315
GU676602	EZSPN443-09	RVcoll.08-H629	x	*Pieridae*	*Pieris*	*rapae*	El Gastor	Cadiz	Andalusia	Spain	660
KP870276	EZSPM982-12	Rvcoll.12-M745	x	*Pieridae*	*Pieris*	*rapae*	Illa Grossa, Columbretes, Castellon de la Plana	Castellon	Comunidad Valenciana	Spain	26.6
GU676400	EZSPN749-09	RVcoll.08-L201	x	*Pieridae*	*Pieris*	*rapae*	La Vega, Vilafranca	Castellon	Comunidad Valenciana	Spain	1124
KP870579	EZSPM919-12	Rvcoll.12-M641	x	*Pieridae*	*Pieris*	*rapae*	Lagunas de Ruidera, Ossa de Montiel	Ciudad Real	Castilla-La Mancha	Spain	795
KP870788	EZSPM977-12	Rvcoll.12-M737	x	*Pieridae*	*Pieris*	*rapae*	Arroyo de los Molinillos, Viso del Marques	Ciudad Real	Castilla-La Mancha	Spain	742
HM901602	EZSPC747-10	RVcoll.08-L052	x	*Pieridae*	*Pieris*	*rapae*	Sierra Elvira, Albolote	Granada	Andalusia	Spain	819
GU676848	EZSPN088-09	RVcoll.06-A017	x	*Pieridae*	*Pieris*	*rapae*	Mecina Bombaron, Alpujarras	Granada	Andalusia	Spain	1150
GU676461	EZSPN686-09	RVcoll.08-L014	x	*Pieridae*	*Pieris*	*rapae*	Ascenso al Veleta, Gueejar Sierra	Granada	Andalusia	Spain	2270
GU676452	EZSPN693-09	RVcoll.08-L025	x	*Pieridae*	*Pieris*	*rapae*	Casillas de Rojas, Gueejar Sierra	Granada	Andalusia	Spain	1733
GU676446	EZSPN702-09	RVcoll.08-L045	x	*Pieridae*	*Pieris*	*rapae*	Sierra Elvira, Albolote	Granada	Andalusia	Spain	819
GU676534	EZSPN603-09	RVcoll.08-J306	x	*Pieridae*	*Pieris*	*rapae*	Barranco de Valcuerna, Candasnos	Huesca	Aragon	Spain	210
GU669615	EZSPC667-09	RVcoll.08-L474	x	*Pieridae*	*Pieris*	*rapae*	Ivars de Noguera	Lleida	Catalonia	Spain	373
GU675995	EZSPM028-09	RVcoll.08-P225	x	*Pieridae*	*Pieris*	*rapae*	Puig d`en Galileu	Mallorca	Balearic Islands	Spain	1071
GU675996	EZSPM047-09	RVcoll.08-P264	x	*Pieridae*	*Pieris*	*rapae*	Santa Maria	Mallorca	Balearic Islands	Spain	181
GU675998	EZSPM063-09	RVcoll.08-P292	x	*Pieridae*	*Pieris*	*rapae*	Genova	Mallorca	Balearic Islands	Spain	123
GU675844	EZSPM213-09	RVcoll.08-R334	x	*Pieridae*	*Pieris*	*rapae*	Palma de Mallorca	Mallorca	Balearic Islands	Spain	88
KP870597	EZSPM992-12	Rvcoll.12-L878	x	*Pieridae*	*Pieris*	*rapae*	Turdonell de Dalt, Mao, Menorca	Menorca	Balearic Islands	Spain	
GU676736	EZSPN299-09	RVcoll.08-H103	x	*Pieridae*	*Pieris*	*rapae*	Na Vermella, Mao	Menorca	Balearic Islands	Spain	
GU676719	EZSPN315-09	RVcoll.08-H173	x	*Pieridae*	*Pieris*	*rapae*	Llimpa, Mao	Menorca	Balearic Islands	Spain	
GU675841	EZSPM235-09	RVcoll.08-R435	x	*Pieridae*	*Pieris*	*rapae*	Oia, As Marinas-Mougas	Pontevedra	Galicia	Spain	20
KP870279	EZSPM894-12	Rvcoll.12-M616	x	*Pieridae*	*Pieris*	*rapae*	Montuenga	Segovia	Castilla y Leon	Spain	890
HM901729	EZSPC890-10	RVcoll.09-V394	x	*Pieridae*	*Pieris*	*rapae*	Delta de l`Ebre, La Tancada, Montsia	Tarragona	Catalonia	Spain	1
HM901789	EZSPC953-10	RVcoll.09-X505	x	*Pieridae*	*Pieris*	*rapae*	2 km NW El Vallecillo	Teruel	Aragon	Spain	1450
GU675894	EZSPM344-09	RVcoll.08-R005	x	*Pieridae*	*Pieris*	*rapae*	Paraisos Altos, Manzanera	Teruel	Aragon	Spain	1200
GU676386	EZSPN760-09	RVcoll.08-L239	x	*Pieridae*	*Pieris*	*rapae*	Ruta de la Sima, El Vallecillo	Teruel	Aragon	Spain	1531
KP871138	EZSPM897-12	Rvcoll.12-M619	x	*Pieridae*	*Pieris*	*rapae*	Medina del Campo	Valladolid	Castilla y Leon	Spain	739
GU688445	GWORO795-09	BC ZSM Lep 30487		*Lycaenidae*	*Plebejus*	*argus*	Nantesbuch Bd. Heilbrunn / TOEL		Bavaria	Germany	
HM391836	FBLMU498-09	BC ZSM Lep 27148		*Lycaenidae*	*Plebejus*	*argus*	Traunstein, Pechschnait	Oberbayern	Bavaria	Germany	695
HM391837	FBLMU499-09	BC ZSM Lep 27149		*Lycaenidae*	*Plebejus*	*argus*	Traunstein, Pechschnait	Oberbayern	Bavaria	Germany	670
GU707265	FBLMV692-09	BC ZSM Lep 28672		*Lycaenidae*	*Plebejus*	*argus*	Duggendorf, Girnitztal	Oberpfalz	Bavaria	Germany	375
GU676542	EZSPN599-09	RVcoll.08-J189	x	*Lycaenidae*	*Plebejus*	*argus*	Serra do Larouco (turfeira N), Padornelos, Montalegre		Tras-os-Montes	Portugal	1360
HQ004976	EZRMN186-08	RVcoll.08-M216		*Lycaenidae*	*Plebejus*	*argus*	3 Km SW of Lunca Corbului	Arges	Muntenia	Romania	235
HQ004975	EZRMN185-08	RVcoll.08-M380		*Lycaenidae*	*Plebejus*	*argus*	Buciumeni forest (Radacinesti)	Bacau	Moldavia	Romania	180
HQ004966	EZROM592-08	RV-07-C302		*Lycaenidae*	*Plebejus*	*argus*	Rediu forest, Botosani	Botosani	Moldavia	Romania	160
HQ004971	EZRMN188-08	RVcoll.08-M281		*Lycaenidae*	*Plebejus*	*argus*	3 Km W of Maierus	Brasov	Transylvania	Romania	550
HQ004969	EZROM479-08	RV-07-D097		*Lycaenidae*	*Plebejus*	*argus*	Dealul Istrita, Breaza	Buzau	Muntenia	Romania	540
HQ004973	EZRMN392-09	RVcoll.07-E355		*Lycaenidae*	*Plebejus*	*argus*	Baile Herculane, Pecinisca	Caras-Severin	Banat	Romania	260
HQ004967	EZROM481-08	RV-07-D442		*Lycaenidae*	*Plebejus*	*argus*	Suatu	Cluj	Transylvania	Romania	380
HQ004968	EZROM482-08	RV-07-C109		*Lycaenidae*	*Plebejus*	*argus*	Cluj-Napoca, dealul Feleacului, Gheorgheni forest	Cluj	Transylvania	Romania	600
HQ004970	EZROM478-08	RV-07-D020		*Lycaenidae*	*Plebejus*	*argus*	Canaraua Fetei (Baneasa)	Constanta	Dobrogea	Romania	15
HQ004972	EZRMN393-09	RVcoll.07-E395		*Lycaenidae*	*Plebejus*	*argus*	Picior de Munte	Dambovita	Muntenia	Romania	260
HQ004965	EZROM651-08	RV-07-D545		*Lycaenidae*	*Plebejus*	*argus*	Rohia	Maramures	Maramures	Romania	360
HQ004974	EZRMN187-08	RVcoll.08-M269		*Lycaenidae*	*Plebejus*	*argus*	4 Km W of Drobeta Turnu-Severin	Mehedinti	Oltenia	Romania	75

GenBank accession number	BOLD Process ID	Sample ID	Used in the Iberian dataset	Family	Genus	Species	Exact Site	Region	State/Province	Country	Alt. (m)
HQ004963	EZROM658-08	RV-07-E516		*Lycaenidae*	*Plebejus*	*argus*	2 km N of Cilieni	Olt	Oltenia	Romania	50
HQ004964	EZROM652-08	RV-07-E436		*Lycaenidae*	*Plebejus*	*argus*	3 km E of Slava Rusa (Babadag forest)	Tulcea	Dobrogea	Romania	130
KP870694	EZSPM876-12	Rvcoll.130711PX72	x	*Lycaenidae*	*Plebejus*	*argus*	Fondos de Vega, Degana		Asturias	Spain	1079
JN276891	EZSPN1054-11	Rvcoll.08-L962	x	*Lycaenidae*	*Plebejus*	*argus*	Puerto de Pozazal		Cantabria	Spain	1040
GU676291	EZSPN869-09	RVcoll.08-L750	x	*Lycaenidae*	*Plebejus*	*argus*	Oteruelo del Valle		Comunidad de Madrid	Spain	1148
GU676282	EZSPN877-09	RVcoll.08-L774	x	*Lycaenidae*	*Plebejus*	*argus*	San Andres		Cantabria	Spain	972
KP870516	EZROM749-08	RV-07-C485	x	*Lycaenidae*	*Plebejus*	*argus*	El Brull, Osona	Barcelona	Catalonia	Spain	835
HM901465	EZSPC1292-10	RVcoll.121109PY13	x	*Lycaenidae*	*Plebejus*	*argus*	Pico Cruces,Cortes de Arenoso	Castellon	Comunidad Valenciana	Spain	1697
JN276884	EZSPC1293-10	RVcoll.050208ZX5	x	*Lycaenidae*	*Plebejus*	*argus*	Moli de Joaquim,La Pobla de Benifassa	Castellon	Comunidad Valenciana	Spain	971
KP870874	EZSPM872-12	Rvcoll.130410FG44	x	*Lycaenidae*	*Plebejus*	*argus*	Fredes, La Pobla de Benifassa	Castellon	Comunidad Valenciana	Spain	1079
GU676429	EZSPN718-09	RVcoll.08-L104	x	*Lycaenidae*	*Plebejus*	*argus*	Puerto de las Cabrillas, Portell de Morella	Castellon	Comunidad Valenciana	Spain	1301
GU669629	EZSPC649-09	RVcoll.07-C493	x	*Lycaenidae*	*Plebejus*	*argus*	Meranges	Girona	Catalonia	Spain	1555
GU676089	EZSPM451-09	Rvcoll.09-V591	x	*Lycaenidae*	*Plebejus*	*argus*	Dornajo (Sierra Nevada)	Granada	Andalusia	Spain	2060
GU676086	EZSPM452-09	Rvcoll.09-V925	x	*Lycaenidae*	*Plebejus*	*argus*	Sierra de la Sagra	Granada	Andalusia	Spain	1745
GU676421	EZSPN726-09	RVcoll.08-L133	x	*Lycaenidae*	*Plebejus*	*argus*	Casillas de Rojas, Guejar Sierra	Granada	Andalusia	Spain	1733
GU676409	EZSPN739-09	RVcoll.08-L152	x	*Lycaenidae*	*Plebejus*	*argus*	Laguna Seca, Hueneja	Granada	Andalusia	Spain	2276
GU676174	EZSPN986-09	RVcoll.08-M028	x	*Lycaenidae*	*Plebejus*	*argus*	Gualda, La Alcarria	Guadalajara	Castilla-La Mancha	Spain	730
GU676087	EZSPM432-09	Rvcoll.09-X267	x	*Lycaenidae*	*Plebejus*	*argus*	Donana, Almonte	Huelva	Andalusia	Spain	
GU675927	EZSPM305-09	RVcoll.08-J852	x	*Lycaenidae*	*Plebejus*	*argus*	Posada de Valdeon	Leon	Castilla y Leon	Spain	
JN276901	EZSPN1087-11	Rvcoll.09-X536	x	*Lycaenidae*	*Plebejus*	*argus*	Buron, Picos de Europa	Leon	Castilla y Leon	Spain	1120
GU669621	EZSPC665-09	RVcoll.08-L468	x	*Lycaenidae*	*Plebejus*	*argus*	Ivars de Noguera	Lleida	Catalonia	Spain	373
GU669618	EZSPC670-09	RVcoll.08-M904	x	*Lycaenidae*	*Plebejus*	*argus*	Bassa d`Arres, Vall d`Aran	Lleida	Catalonia	Spain	1500
GU669611	EZSPC671-09	RVcoll.08-M912	x	*Lycaenidae*	*Plebejus*	*argus*	Bassa d`Arres, Vall d`Aran	Lleida	Catalonia	Spain	1500
KP871005	EZROM375-08	07-E625	x	*Lycaenidae*	*Plebejus*	*argus*	Sierra de la Gritella, Prades	Tarragona	Catalonia	Spain	1000
JN276889	EZSPN1036-11	Rvcoll.07-C698	x	*Lycaenidae*	*Plebejus*	*argus*	Pla del Peiro, los Castillejos	Tarragona	Catalonia	Spain	
KP870231	EULEP245-14	12-N714	x	*Lycaenidae*	*Plebejus*	*argus*	El Valecillo	Teruel	Aragón	Spain	1526.7
GU676499	EZSPN647-09	RVcoll.08-J786	x	*Lycaenidae*	*Plebejus*	*argus*	Ruta de la Sima, El Vallecillo	Teruel	Aragon	Spain	1531
GU676680	EZSPN363-09	RVcoll.08-H347	x	*Lycaenidae*	*Plebejus*	*argus*	Sierra de Javalambre, Alpuente	Valencia	Comunidad Valenciana	Spain	
FJ663952	LOWA533-06	2005-LOWA-533		*Lycaenidae*	*Plebejus*	*argus*	Karadag		Crimea	Ukraine	
FJ663951	LOWA534-06	2005-LOWA-534		*Lycaenidae*	*Plebejus*	*argus*	Karadag		Crimea	Ukraine	
JX093468		NK-00-P135		*Lycaenidae*	*Plebejus*	*argus*	Ai-Petri Mt.		Crimea	Ukraine	
GU655007	FBLMT888-09	BC ZSM Lep 25448		*Lycaenidae*	*Plebejus*	*argyrognomon*	Nittendorf	Oberpfalz	Bavaria	Germany	400
GU707145	FBLMU384-09	BC ZSM Lep 27034		*Lycaenidae*	*Plebejus*	*argyrognomon*	Sinzing, Alpiner Steig	Oberpfalz	Bavaria	Germany	405
GU707134	FBLMU393-09	BC ZSM Lep 27043		*Lycaenidae*	*Plebejus*	*argyrognomon*	Kallmuenz, Kirchenberg	Oberpfalz	Bavaria	Germany	390
GU707266	FBLMV715-09	BC ZSM Lep 28695		*Lycaenidae*	*Plebejus*	*argyrognomon*	Duggendorf, Girnitztal	Oberpfalz	Bavaria	Germany	375
GU675633	EZROM1070-09	RVcoll.08-L362		*Lycaenidae*	*Plebejus*	*argyrognomon*	3 Km SW of Lunca Corbului	Arges	Muntenia	Romania	235
HQ004983	EZRMN190-08	RVcoll.08-M367		*Lycaenidae*	*Plebejus*	*argyrognomon*	Buciumeni forest (Radacinesti)	Bacau	Moldavia	Romania	180
HQ004991	EZROM653-08	RV-07-D310		*Lycaenidae*	*Plebejus*	*argyrognomon*	Racos	Brasov	Transylvania	Romania	460
HQ004980	EZRMN193-08	RVcoll.08-M656		*Lycaenidae*	*Plebejus*	*argyrognomon*	Berca, vulcanii noroiosi Paclele Mici	Buzau	Muntenia	Romania	220
HQ004977	EZRMN394-09	RVcoll.06-M953		*Lycaenidae*	*Plebejus*	*argyrognomon*	Dealul Istrita, Breaza	Buzau	Muntenia	Romania	400
HQ004987	EZROM486-08	RV-07-D214		*Lycaenidae*	*Plebejus*	*argyrognomon*	Dealul Istrita, Breaza	Buzau	Muntenia	Romania	540
HQ004988	EZROM487-08	RV-07-D233		*Lycaenidae*	*Plebejus*	*argyrognomon*	Dealul Istrita, Breaza	Buzau	Muntenia	Romania	540
HQ004989	EZROM488-08	RV-07-E357		*Lycaenidae*	*Plebejus*	*argyrognomon*	Baile Herculane (Pecinisca)	Caras-Severin	Banat	Romania	270
HQ004979	EZRMN390-09	RVcoll.07-C129		*Lycaenidae*	*Plebejus*	*argyrognomon*	Gheorgheni (Cluj-Napoca)	Cluj	Transylvania	Romania	600
HQ004984	EZROM483-08	RV-07-C159		*Lycaenidae*	*Plebejus*	*argyrognomon*	Faget forest (Cluj-Napoca)	Cluj	Transylvania	Romania	680
HQ004985	EZROM484-08	RV-07-D407		*Lycaenidae*	*Plebejus*	*argyrognomon*	Suatu	Cluj	Transylvania	Romania	380
HQ004982	EZRMN191-08	RVcoll.08-M440		*Lycaenidae*	*Plebejus*	*argyrognomon*	Canaraua Fetei (Baneasa)	Constanta	Dobrogea	Romania	20
HQ004986	EZROM485-08	RV-07-D975		*Lycaenidae*	*Plebejus*	*argyrognomon*	4 km west of I.L. Caragiale	Dambovita	Muntenia	Romania	270
HQ004981	EZRMN192-08	RVcoll.08-M238		*Lycaenidae*	*Plebejus*	*argyrognomon*	7 Km E of Strehaia	Mehedinti	Oltenia	Romania	125
HQ004978	EZRMN391-09	RVcoll.07-D382		*Lycaenidae*	*Plebejus*	*argyrognomon*	Iernut	Mures	Transylvania	Romania	355
HQ004990	EZROM489-08	RV-07-E506		*Lycaenidae*	*Plebejus*	*argyrognomon*	2 km N of Cilieni	Olt	Oltenia	Romania	50

GenBank accession number	BOLD Process ID	Sample ID	Used in the Iberian dataset	Family	Genus	Species	Exact Site	Region	State/Province	Country	Alt. (m)
FJ663959	LOWA859-06	2005-LOWA-859		*Lycaenidae*	*Plebejus*	*argyrognomon*			Belgorod	Russia	150
FJ663958	LOWA860-06	2005-LOWA-860		*Lycaenidae*	*Plebejus*	*argyrognomon*			Belgorod	Russia	150
JX093467		AD-00-P560		*Lycaenidae*	*Plebejus*	*argyrognomon*	Tatinki		Tula	Russia	
GU676041	EZSPM009-09	RVcoll.08-M989	x	*Lycaenidae*	*Plebejus*	*idas*	Grau Roig			Andorra	2114
GU675977	EZSPM011-09	RVcoll.08-M991	x	*Lycaenidae*	*Plebejus*	*idas*	Grau Roig			Andorra	2114
KP871092	EZROM376-08	07-E626	x	*Lycaenidae*	*Plebejus*	*idas*	road Sainte-Leocadie to Puigmal	Languedoc-Roussillon		France	1550
GU688446	GWORO796-09	BC ZSM Lep 30488		*Lycaenidae*	*Plebejus*	*idas*	Garching-Hochbrueck MuenchenL		Bavaria	Germany	486
GU688532	GWORO797-09	BC ZSM Lep 30489		*Lycaenidae*	*Plebejus*	*idas*	Garching-Hochbrueck MuenchenL		Bavaria	Germany	486
JF415724	GWOR3912-09	BC ZSM Lep 21196		*Lycaenidae*	*Plebejus*	*idas*	Oberschleissheim, Flughafen-Ost	Upper Bavaria	Bavaria	Germany	487
KP870344	EZRMN194-08	08-M557		*Lycaenidae*	*Plebejus*	*idas*	3 km SW of Lunca Corbului	Argeş	Muntenia	Romania	235
HQ004992	EZROM480-08	RV-07-D113		*Lycaenidae*	*Plebejus*	*idas*	Nenciulesti	Buzau	Muntenia	Romania	360
HQ004993	EZRMN422-09	RVcoll.09-V629		*Lycaenidae*	*Plebejus*	*idas*	4 km NE Praid	Harghita	Transylvania	Romania	590
GQ128974		NK-00-P165		*Lycaenidae*	*Plebejus*	*idas*	Luga		St. Petersburg	Russia	
HM901378	EZSPC1178-10	RVcoll.09-T146	x	*Lycaenidae*	*Plebejus*	*idas*	Teverga, Vega Socellares		Asturias	Spain	1780
HM901382	EZSPC1182-10	RVcoll.09-T161	x	*Lycaenidae*	*Plebejus*	*idas*	La Hiruela		Comunidad de Madrid	Spain	1312
JN276895	EZSPN1070-11	Rvcoll.09-T175	x	*Lycaenidae*	*Plebejus*	*idas*	Rio Duje, Sotres		Asturias	Spain	1216
HM901380	EZSPC1180-10	RVcoll.09-T153	x	*Lycaenidae*	*Plebejus*	*idas*	Puerto del Cunillo	Cuenca	Castilla-La Mancha	Spain	1620
GU675700	EZSPM119-09	RVcoll.08-P602	x	*Lycaenidae*	*Plebejus*	*idas*	Piscifactoria, Laguna de Una, Una	Cuenca	Castilla-La Mancha	Spain	1219
HM901419	EZSPC1220-10	RVcoll.09-V543	x	*Lycaenidae*	*Plebejus*	*idas*	Loma de San Francisco (Sierra Nevada)	Granada	Andalusia	Spain	2315
HM901420	EZSPC1221-10	RVcoll.09-V545	x	*Lycaenidae*	*Plebejus*	*idas*	Loma de San Francisco (Sierra Nevada)	Granada	Andalusia	Spain	2315
GU676490	EZSPN662-09	RVcoll.08-J964	x	*Lycaenidae*	*Plebejus*	*idas*	Fuente Albergue de San Francisco, Gueejar Sierra	Granada	Andalusia	Spain	2221
GU676408	EZSPN738-09	RVcoll.08-L151	x	*Lycaenidae*	*Plebejus*	*idas*	Laguna Seca, Hueneja	Granada	Andalusia	Spain	2276
JN276897	EZSPN1076-11	Rvcoll.09-V718.1	x	*Lycaenidae*	*Plebejus*	*idas*	Yesero	Huesca	Aragon	Spain	1160
GU669837	EZSPC402-09	RVcoll.08-R238	x	*Lycaenidae*	*Plebejus*	*idas*	Port de la Bonaigua, Pallars Sobira	Lleida	Catalonia	Spain	2338
GU669601	EZSPC683-09	RVcoll.08-R204	x	*Lycaenidae*	*Plebejus*	*idas*	Port de la Bonaigua, Pallars Sobira	Lleida	Catalonia	Spain	2338
GU669600	EZSPC684-09	RVcoll.08-R205	x	*Lycaenidae*	*Plebejus*	*idas*	Port de la Bonaigua, Pallars Sobira	Lleida	Catalonia	Spain	2338
GU669602	EZSPC685-09	RVcoll.08-R227	x	*Lycaenidae*	*Plebejus*	*idas*	Port de la Bonaigua, Pallars Sobira	Lleida	Catalonia	Spain	2338
GU669603	EZSPC686-09	RVcoll.08-R228	x	*Lycaenidae*	*Plebejus*	*idas*	Port de la Bonaigua, Pallars Sobira	Lleida	Catalonia	Spain	2338
GU676236	EZSPN921-09	RVcoll.08-L867	x	*Lycaenidae*	*Plebejus*	*idas*	Abejar	Soria	Castilla y Leon	Spain	1155
GU676228	EZSPN930-09	RVcoll.08-L885	x	*Lycaenidae*	*Plebejus*	*idas*	Abejar	Soria	Castilla y Leon	Spain	1110
HM901344	EZSPC1144-10	RVcoll.08-P749	x	*Lycaenidae*	*Plebejus*	*idas*	El Vallecillo	Teruel	Aragon	Spain	1529
GU707088	FBLMU451-09	BC ZSM Lep 27101		*Nymphalidae*	*Polygonia*	*c-album*	Schoengeising, Jaegereinfang b. Jexhof	Oberbayern	Bavaria	Germany	570
GU676556	EZSPN589-09	RVcoll.08-J152	x	*Nymphalidae*	*Polygonia*	*c-album*	Ponte Pedrinha, Beca, Boticas		Tras-os-Montes	Portugal	730
HQ005002	EZROM327-08	RV-08-A005		*Nymphalidae*	*Polygonia*	*c-album*	Dealul Istrita, Breaza	Buzau	Muntenia	Romania	525
HQ005003	EZROM492-08	RV-06-K608		*Nymphalidae*	*Polygonia*	*c-album*	Badeni	Cluj	Transylvania	Romania	440
HQ005004	EZROM493-08	RV-06-M874		*Nymphalidae*	*Polygonia*	*c-album*	Cluj-Napoca, dealul Feleacului, Gheorgheni forest	Cluj	Transylvania	Romania	600
HQ005008	EZRMN205-08	RVcoll.08-M441		*Nymphalidae*	*Polygonia*	*c-album*	Canaraua Fetei (Baneasa)	Constanta	Dobrogea	Romania	20
HQ005007	EZRMN204-08	RVcoll.07-D936		*Nymphalidae*	*Polygonia*	*c-album*	Ciupercenii de Oltet	Gorj	Oltenia	Romania	500
HQ005006	EZRMN203-08	RVcoll.07-D564		*Nymphalidae*	*Polygonia*	*c-album*	Spini	Hunedoara	Transylvania	Romania	240
KP870602	EULEP144-14	10-C427		*Nymphalidae*	*Polygonia*	*c-album*	Preluca	Neamt	Moldavia	Romania	
HQ005005	EZROM494-08	RV-07-D905		*Nymphalidae*	*Polygonia*	*c-album*	valea Otasaului, Barzesti, Buila-Vanturarita	Valcea	Oltenia	Romania	515
FJ663976	LOWA863-06	2005-LOWA-863		*Nymphalidae*	*Polygonia*	*c-album*			Belgorod	Russia	150
FJ663979	LOWA164-06	2005-LOWA-164		*Nymphalidae*	*Polygonia*	*c-album*	Osyno vill.	Sebezh distr	Pskov	Russia	150
FJ663978	LOWA785-06	2005-LOWA-785		*Nymphalidae*	*Polygonia*	*c-album*	Osyno	Sebezh distr.	Pskov	Russia	150
FJ663977	LOWA786-06	2005-LOWA-786		*Nymphalidae*	*Polygonia*	*c-album*	Osyno	Sebezh distr.	Pskov	Russia	150
GU675696	EZSPM147-09	RVcoll.08-P660	x	*Nymphalidae*	*Polygonia*	*c-album*	Valle (Valle-Zurea), Lena		Asturias	Spain	662
GU676209	EZSPN948-09	RVcoll.08-L938	x	*Nymphalidae*	*Polygonia*	*c-album*	Rascafria		Comunidad de Madrid	Spain	1150
KP870409	EZROM750-08	RV-06-G442	x	*Nymphalidae*	*Polygonia*	*c-album*	Revolt de les Serps, El Brull	Barcelona	Catalonia	Spain	800
GU669624	EZSPC661-09	RVcoll.08-H209	x	*Nymphalidae*	*Polygonia*	*c-album*	Setcases, El Ripolles	Girona	Catalonia	Spain	1321
GU669609	EZSPC677-09	RVcoll.08-P078	x	*Nymphalidae*	*Polygonia*	*c-album*	Estoll, Fontanals de Cerdanya, Cerdanya	Girona	Catalonia	Spain	1085
GU676169	EZSPN993-09	RVcoll.08-M062	x	*Nymphalidae*	*Polygonia*	*c-album*	Budia, La Alcarria	Guadalajara	Castilla-La Mancha	Spain	1006
HM901257	EZSPC1054-10	RVcoll.08-J817	x	*Nymphalidae*	*Polygonia*	*c-album*	Compludo	Leon	Castilla y Leon	Spain	

GenBank accession number	BOLD Process ID	Sample ID	Used in the Iberian dataset	Family	Genus	Species	Exact Site	Region	State/Province	Country	Alt. (m)
GU669616	EZSPC668-09	RVcoll.08-L494	x	*Nymphalidae*	*Polygonia*	*c-album*	Bassa d`Arres, Vall d`Aran	Lleida	Catalonia	Spain	1501
GU669610	EZSPC678-09	RVcoll.08-P336	x	*Nymphalidae*	*Polygonia*	*c-album*	Perves, Pallars Jussa	Lleida	Catalonia	Spain	1071
GU675774	EZSPM259-09	RVcoll.08-R473	x	*Nymphalidae*	*Polygonia*	*c-album*	Tui, Rio Louro-Magdalena	Pontevedra	Galicia	Spain	20
HM901290	EZSPC1089-10	RVcoll.08-L857	x	*Nymphalidae*	*Polygonia*	*c-album*	Abejar	Soria	Castilla y Leon	Spain	1155
KC581753		RVcoll.09-V962		*Lycaenidae*	*Polyommatus*	*admetus*	Paril - Nova Lovcha		Hadjidimovo	Bulgaria	750
KC581754		RVcoll.09-V963		*Lycaenidae*	*Polyommatus*	*admetus*	Paril - Nova Lovcha		Hadjidimovo	Bulgaria	750
HM393189	GWORA2460-09	BC ZSM Lep 30672		*Lycaenidae*	*Polyommatus*	*amandus*	Freischweibach	Mittelfranken	Bavaria	Germany	460
HQ563550	FBLMW310-10	BC ZSM Lep 37411		*Lycaenidae*	*Polyommatus*	*amandus*	Kirchenlamitz, Niederlamitz/Ofr.	Oberfranken	Bavaria	Germany	630
HQ005009	EZROM495-08	RV-07-D035		*Lycaenidae*	*Polyommatus*	*amandus*	Babadag forest	Tulcea	Dobrogea	Romania	115
JX093452		AD-00-P053		*Lycaenidae*	*Polyommatus*	*amandus*	Kamyshinsky		Volgograd	Russia	
GU676278	EZSPN880-09	RVcoll.08-L785	x	*Lycaenidae*	*Polyommatus*	*amandus*	San Andres		Cantabria	Spain	972
GU676260	EZSPN895-09	RVcoll.08-L806	x	*Lycaenidae*	*Polyommatus*	*amandus*	Hormiguera		Cantabria	Spain	968
GU676424	EZSPN720-09	RVcoll.08-L109	x	*Lycaenidae*	*Polyommatus*	*amandus*	Pobla del Bellestar, Vilafranca	Castellon	Comunidad Valenciana	Spain	1146
GU676402	EZSPN740-09	RVcoll.08-L162	x	*Lycaenidae*	*Polyommatus*	*amandus*	Font del Regatxal, Vilafranca	Castellon	Comunidad Valenciana	Spain	1361
GU675638	EZSPC656-09	RVcoll.07-C664	x	*Lycaenidae*	*Polyommatus*	*amandus*	Meranges	Girona	Catalonia	Spain	1555
HM901558	EZSPC701-10	RVcoll.07-C672	x	*Lycaenidae*	*Polyommatus*	*amandus*	Sant Marti de Puigcerda	Girona	Catalonia	Spain	1145
JX093453		MAT-99-Q840	x	*Lycaenidae*	*Polyommatus*	*amandus*	Urús	Girona	Catalonia	Spain	
KP870310	EZSPM663-12	RVcoll.11-I579	x	*Lycaenidae*	*Polyommatus*	*amandus*	ca. 8 km E of Cumbres Verdes (La Zubia), botanical garden	Granada	Andalusia	Spain	1700
KP870433	EZSPM664-12	RVcoll.11-I580	x	*Lycaenidae*	*Polyommatus*	*amandus*	ca. 8 km E of Cumbres Verdes (La Zubia), botanical garden	Granada	Andalusia	Spain	1700
HM901746	EZSPC908-10	RVcoll.09-V774	x	*Lycaenidae*	*Polyommatus*	*amandus*	Embalse La Sarra, Sallent de Gallego	Huesca	Aragon	Spain	1410
GU676969	EZSPN255-09	RVcoll.07-W155	x	*Lycaenidae*	*Polyommatus*	*amandus*	Vallibierna	Huesca	Aragon	Spain	1800
GU675637	EZSPC660-09	RVcoll.07-W117	x	*Lycaenidae*	*Polyommatus*	*amandus*	Val d`Hurno, Vall d`Aran	Lleida	Catalonia	Spain	1500
GU675636	EZSPC680-09	RVcoll.08-P371	x	*Lycaenidae*	*Polyommatus*	*amandus*	Perves, Pallars Jussa	Lleida	Catalonia	Spain	1071
KM459213	EULEP094-14	RVcoll.09-T513		*Lycaenidae*	*Polyommatus*	*celina*	Ficuzza, Bosco della Ficuzza		Sicily	Italy	
KM459217	EULEP096-14	RVcoll.09-T570		*Lycaenidae*	*Polyommatus*	*celina*	Gonnosfanadiga		Sardinia	Italy	
JN084663		RVcoll.09-T503		*Lycaenidae*	*Polyommatus*	*celina*	Gennargentu, env. Broncu Spina		Sardinia	Italy	
JN084665		RVcoll.09-T502		*Lycaenidae*	*Polyommatus*	*celina*	Gennargentu, env. Broncu Spina		Sardinia	Italy	
JN084676		RVcoll.186		*Lycaenidae*	*Polyommatus*	*celina*	Santa Lucia del Mela, Messina		Sicily	Italy	
JN084677		RVcoll.Lip1		*Lycaenidae*	*Polyommatus*	*celina*	Lipari island		Lipari	Italy	
JN084678		RVcoll.183		*Lycaenidae*	*Polyommatus*	*celina*	Santa Lucia del Mela, Messina		Sicily	Italy	
JN084679		RVcoll.Lip2		*Lycaenidae*	*Polyommatus*	*celina*	Lipari island		Lipari	Italy	
JN084681		RVcoll.09-T512		*Lycaenidae*	*Polyommatus*	*celina*	Ficuzza, Bosco della Ficuzza		Sicily	Italy	
KM517849	EZSPM869-12	Rvcoll.090211SD59	x	*Lycaenidae*	*Polyommatus*	*celina*	Sierra Espuna, Totana		Murcia	Spain	1411
GU676919	EZSPN514-09	RVcoll.08-H977	x	*Lycaenidae*	*Polyommatus*	*celina*	Hoyocasero, Venta Rasquilla	Avila	Castilla y Leon	Spain	1224
KM459288	EZSPM883-12	Rvcoll.12-M601	x	*Lycaenidae*	*Polyommatus*	*celina*	Medellin	Badajoz	Extremadura	Spain	240
KM517861	EZSPM884-12	Rvcoll.12-M602	x	*Lycaenidae*	*Polyommatus*	*celina*	Medellin	Badajoz	Extremadura	Spain	240
GU676593	EZSPN446-09	RVcoll.08-H637	x	*Lycaenidae*	*Polyommatus*	*celina*	La Linea de la Concepcion, playa de Santa Margarita	Cadiz	Andalusia	Spain	
GU676331	EZSPN817-09	RVcoll.08-L622	x	*Lycaenidae*	*Polyommatus*	*celina*	Algeciras, Arroyo del Prior	Cadiz	Andalusia	Spain	50
KM459289	EULEP241-14	12-M686	x	*Lycaenidae*	*Polyommatus*	*celina*	Río Jándula, Mestanza	Ciudad Real	Castilla-La Mancha	Spain	407
KM459290	EZSPM957-12	Rvcoll.12-M708	x	*Lycaenidae*	*Polyommatus*	*celina*	Alrededores San Lorenzo de Calatrava	Ciudad Real	Castilla-La Mancha	Spain	749
KM459291	EZSPM971-12	Rvcoll.12-M729	x	*Lycaenidae*	*Polyommatus*	*celina*	Arroyo de los Molinillos, Viso del Marques	Ciudad Real	Castilla-La Mancha	Spain	742
KM459232	EULEP153-14	11-D598	x	*Lycaenidae*	*Polyommatus*	*celina*	Parc Natural Ses Salines	Formentera	Balearic Islands	Spain	
KM459177	EULEP005-14	06-A042	x	*Lycaenidae*	*Polyommatus*	*celina*	Barranco de los Lagartos, Cádiar	Granada	Andalusia	Spain	860
KM459203	EULEP072-14	08-L130	x	*Lycaenidae*	*Polyommatus*	*celina*	Casillas de Rojas, Güejar Sierra	Granada	Andalusia	Spain	1733
KM459224	EULEP117-14	09-X369	x	*Lycaenidae*	*Polyommatus*	*celina*	Sierra de la Sagra	Granada	Andalusia	Spain	1670-1820
KM459285	EULEP163-14	11-I455	x	*Lycaenidae*	*Polyommatus*	*celina*	Road to Veleta (Sierra Nevada)	Granada	Andalusia	Spain	2550-2900
HM901553	EZSPC694-10	RVcoll.09-V472	x	*Lycaenidae*	*Polyommatus*	*celina*	Sierra de la Sagra	Granada	Andalusia	Spain	1745
GU676078	EZSPM466-09	Rvcoll.09-V900	x	*Lycaenidae*	*Polyommatus*	*celina*	Sierra de la Sagra	Granada	Andalusia	Spain	1745
KM517864	EZSPM756-12	RVcoll.12-L164	x	*Lycaenidae*	*Polyommatus*	*celina*	Sierra de la Sagra	Granada	Andalusia	Spain	
GU676772	EZSPN169-09	RVcoll.06-K695	x	*Lycaenidae*	*Polyommatus*	*celina*	Guillimona, La Sagra, HUESCAR	Granada	Andalusia	Spain	1750
GU676438	EZSPN711-09	RVcoll.08-L063	x	*Lycaenidae*	*Polyommatus*	*celina*	Venta del Molinillo, Huetor de Santillan	Granada	Andalusia	Spain	1236

GenBank accession number	BOLD Process ID	Sample ID	Used in the Iberian dataset	Family	Genus	Species	Exact Site	Region	State/Province	Country	Alt. (m)
JN084668		09-V503	x	*Lycaenidae*	*Polyommatus*	*celina*	Sierra de Alfacar	Granada	Andalusia	Spain	1275
JN084670		RVcoll.08-L002	x	*Lycaenidae*	*Polyommatus*	*celina*	Carretera al Veleta (Sierra Nevada)	Granada	Andalusia	Spain	
JN084675		RVcoll.09-V558	x	*Lycaenidae*	*Polyommatus*	*celina*	Collado de las Sabinas (Sierra Nevada)	Granada	Andalusia	Spain	
KM459227	EULEP148-14	10-C449	x	*Lycaenidae*	*Polyommatus*	*celina*	San Agustí	Ibiza	Balearic Islands	Spain	
HM901584	EZSPC729-10	RVcoll.08-H634	x	*Lycaenidae*	*Polyommatus*	*celina*	Benadalid	Malaga	Andalusia	Spain	790
HM901647	EZSPC797-10	RVcoll.08-P297	x	*Lycaenidae*	*Polyommatus*	*celina*	Genova	Mallorca	Balearic Islands	Spain	123.2
HM901649	EZSPC799-10	RVcoll.08-P310	x	*Lycaenidae*	*Polyommatus*	*celina*	Sant Elm, Andratx	Mallorca	Balearic Islands	Spain	30.9
GU675982	EZSPM039-09	RVcoll.08-P244	x	*Lycaenidae*	*Polyommatus*	*celina*	Puig d`en Galileu	Mallorca	Balearic Islands	Spain	1071
GU675979	EZSPM057-09	RVcoll.08-P282	x	*Lycaenidae*	*Polyommatus*	*celina*	S`Albufera	Mallorca	Balearic Islands	Spain	4
JN084664		RVcoll.08-P246	x	*Lycaenidae*	*Polyommatus*	*celina*	Puig d’en Galileu	Mallorca	Balearic Islands	Spain	
KM459181	EULEP046-14	RVcoll.08-H191	x	*Lycaenidae*	*Polyommatus*	*celina*	Llimpa, Maó	Menorca	Balearic Islands	Spain	
KM459182	EULEP047-14	RVcoll.08-H195	x	*Lycaenidae*	*Polyommatus*	*celina*	Llimpa, Maó	Menorca	Balearic Islands	Spain	
KM459219	EULEP110-14	RVcoll.09-X105	x	*Lycaenidae*	*Polyommatus*	*celina*	Cala de Tirant, Fornells	Menorca	Balearic Islands	Spain	
KM459220	EULEP111-14	RVcoll.09-X106	x	*Lycaenidae*	*Polyommatus*	*celina*	Cala de Tirant, Fornells	Menorca	Balearic Islands	Spain	
KM459221	EULEP112-14	RVcoll.09-X114	x	*Lycaenidae*	*Polyommatus*	*celina*	Parque Albufera des Grau	Menorca	Balearic Islands	Spain	
GU676726	EZSPN314-09	RVcoll.08-H172	x	*Lycaenidae*	*Polyommatus*	*celina*	Es Grau, Mao	Menorca	Balearic Islands	Spain	
GU676721	EZSPN317-09	RVcoll.08-H177	x	*Lycaenidae*	*Polyommatus*	*celina*	Llimpa, Mao	Menorca	Balearic Islands	Spain	
JN084662		RVcoll.08-H192	x	*Lycaenidae*	*Polyommatus*	*celina*	Llimpa, Maó	Menorca	Balearic Islands	Spain	
JF415726	GWORK306-09	BC ZSM Lep 21541		*Lycaenidae*	*Polyommatus*	*damon*	Moernsheim, Steinbruch 2 km w	Oberpfalz	Bavaria	Germany	430
GU676223	EZSPN934-09	RVcoll.08-L894	x	*Lycaenidae*	*Polyommatus*	*damon*	Hormiguera		Cantabria	Spain	968
GU676224	EZSPN935-09	RVcoll.08-L895	x	*Lycaenidae*	*Polyommatus*	*damon*	Hormiguera		Cantabria	Spain	968
GU676405	EZSPN743-09	RVcoll.08-L167	x	*Lycaenidae*	*Polyommatus*	*damon*	Font del Regatxal, Vilafranca	Castellon	Comunidad Valenciana	Spain	1361
HM901305	EZSPC1104-10	RVcoll.08-L994	x	*Lycaenidae*	*Polyommatus*	*damon*	Mogorrita, Tragacete	Cuenca	Castilla-La Mancha	Spain	1597
GU676752	EZSPN284-09	RVcoll.07-W228	x	*Lycaenidae*	*Polyommatus*	*damon*	Sierra de los Barrancos, Cuenca	Cuenca	Castilla-La Mancha	Spain	1447
GU675971	EZSPM015-09	RVcoll.08-P001	x	*Lycaenidae*	*Polyommatus*	*damon*	Torre del Riu, Ribera d`Alp, Cerdanya	Girona	Catalonia	Spain	1180
GU675795	EZSPM200-09	RVcoll.08-R289	x	*Lycaenidae*	*Polyommatus*	*damon*	Masia del Plans, Vidra, La Garrotxa	Girona	Catalonia	Spain	1200
AY496732.2		MAT-99-Q841	x	*Lycaenidae*	*Polyommatus*	*damon*	Urús	Girona	Catalonia	Spain	
HM901325	EZSPC1125-10	RVcoll.08-P629	x	*Lycaenidae*	*Polyommatus*	*damon*	Penalen	Guadalajara	Castilla-La Mancha	Spain	1288
HM901317	EZSPC1116-10	RVcoll.08-P326	x	*Lycaenidae*	*Polyommatus*	*damon*	Perves, Pallars Jussa	Lleida	Catalonia	Spain	1071.7
GU677042	EZSPN203-09	RVcoll.07-F023	x	*Lycaenidae*	*Polyommatus*	*damon*	Boixols, Pallars Jussa	Lleida	Catalonia	Spain	1240
HM901513	EZSPC1379-10	RVcoll.09-X513	x	*Lycaenidae*	*Polyommatus*	*damon*	2 km NW El Vallecillo	Teruel	Aragon	Spain	1450
GU676393	EZSPN754-09	RVcoll.08-L230	x	*Lycaenidae*	*Polyommatus*	*damon*	Ruta de la Sima, El Vallecillo	Teruel	Aragon	Spain	1531
GU688451	GWORO762-09	BC ZSM Lep 30454		*Lycaenidae*	*Polyommatus*	*daphnis*	Moernsheim	Oberbayern	Bavaria	Germany	550
GU707268	FBLMV713-09	BC ZSM Lep 28693		*Lycaenidae*	*Polyommatus*	*daphnis*	Duggendorf, Girnitztal	Oberpfalz	Bavaria	Germany	375
HQ005034	EZROM638-08	RV-06-N022		*Lycaenidae*	*Polyommatus*	*daphnis*	Racos	Brasov	Transylvania	Romania	460
KP870411	EULEP149-14	10-C471		*Lycaenidae*	*Polyommatus*	*daphnis*	Răscruci	Cluj	Transylvania	Romania	
KP870379	EULEP174-14	11-J335		*Lycaenidae*	*Polyommatus*	*daphnis*	Suatu	Cluj	Transylvania	Romania	350-400
HQ005032	EZRMN097-08	RVcoll.06-V686		*Lycaenidae*	*Polyommatus*	*daphnis*	Suatu	Cluj	Transylvania	Romania	380
HQ005037	EZROM399-08	RV-06-M883		*Lycaenidae*	*Polyommatus*	*daphnis*	Cluj-Napoca, dealul Bogomaia	Cluj	Transylvania	Romania	550
HQ005036	EZROM400-08	RV-06-M884		*Lycaenidae*	*Polyommatus*	*daphnis*	Cluj-Napoca, dealul Bogomaia	Cluj	Transylvania	Romania	550
HQ005033	EZRMN098-08	RVcoll.07-E413		*Lycaenidae*	*Polyommatus*	*daphnis*	8 Km S of Babadag (Babadag forest)	Tulcea	Dobrogea	Romania	115
HQ005035	EZROM401-08	RV-07-E425		*Lycaenidae*	*Polyommatus*	*daphnis*	3 km E of Slava Rusa (Babadag forest)	Tulcea	Dobrogea	Romania	130
GU669725	EZSPC538-09	RVcoll.08-P064	x	*Lycaenidae*	*Polyommatus*	*daphnis*	Torrent de Ridolaina, Cerdanya	Girona	Catalonia	Spain	1060
HM901326	EZSPC1126-10	RVcoll.08-P636	x	*Lycaenidae*	*Polyommatus*	*daphnis*	Hoz Angosta, Duron	Guadalajara	Castilla-La Mancha	Spain	789
GU675761	EZSPM130-09	RVcoll.08-P635	x	*Lycaenidae*	*Polyommatus*	*daphnis*	Hoz Angosta, Duron	Guadalajara	Castilla-La Mancha	Spain	789
GU669730	EZSPC536-09	RVcoll.07-F005	x	*Lycaenidae*	*Polyommatus*	*daphnis*	Valldarques, Coll de Nargo, Alt Urgell	Lleida	Catalonia	Spain	975
GU669729	EZSPC535-09	RVcoll.07-F533	x	*Lycaenidae*	*Polyommatus*	*daphnis*	Barranc de Vinarroig, Prades	Tarragona	Catalonia	Spain	920
GU669724	EZSPC537-09	RVcoll.08-M695	x	*Lycaenidae*	*Polyommatus*	*daphnis*	Farena, Alt Camp	Tarragona	Catalonia	Spain	580
GU669643	EZSPC638-09	RVcoll.08-L376	x	*Lycaenidae*	*Polyommatus*	*daphnis*	road T-704 between La Febro and La Mussara, km 27	Tarragona	Catalonia	Spain	845
GU676367	EZSPN779-09	RVcoll.08-L291	x	*Lycaenidae*	*Polyommatus*	*daphnis*	Paraisos Altos, Manzanera	Teruel	Aragon	Spain	1200
FJ663772	LOWA522-06	2005-LOWA-522		*Nymphalidae*	*Polyommatus*	*daphnis*	Karadag		Crimea	Ukraine	
FJ663771	LOWA523-06	2005-LOWA-523		*Nymphalidae*	*Polyommatus*	*daphnis*	Karadag		Crimea	Ukraine	
FJ663770	LOWA524-06	2005-LOWA-524		*Nymphalidae*	*Polyommatus*	*daphnis*	Karadag		Crimea	Ukraine	
JX093461		NK-00-P108		*Lycaenidae*	*Polyommatus*	*daphnis*	Kurortnoe		Crimea	Ukraine	
AY496740.2		MAT99-Q923		*Lycaenidae*	*Polyommatus*	*dolus*	Mende		Lozère	France	

GenBank accession number	BOLD Process ID	Sample ID	Used in the Iberian dataset	Family	Genus	Species	Exact Site	Region	State/Province	Country	Alt. (m)
HM210162		RE07-G106		*Lycaenidae*	*Polyommatus*	*dolus*	Rocca Pia		L´Aquila	Italy	1215
GU688452	GWORO803-09	BC ZSM Lep 30495		*Lycaenidae*	*Polyommatus*	*dorylas*	Samerberg (RO) Lederstuben		Bavaria	Germany	750
HQ005045	EZRMN200-08	RVcoll.08-M510		*Lycaenidae*	*Polyommatus*	*dorylas*	Apuseni Mts., Rimetea (Piatra Secuiului)	Alba	Transylvania	Romania	850
HQ005044	EZRMN201-08	RVcoll.08-M511		*Lycaenidae*	*Polyommatus*	*dorylas*	Apuseni Mts., Rimetea (Piatra Secuiului)	Alba	Transylvania	Romania	850
HQ005040	EZROM498-08	RV-06-M979		*Lycaenidae*	*Polyommatus*	*dorylas*	Apuseni Mts., Rimetea (Piatra Secuiului)	Alba	Transylvania	Romania	750
HQ005039	EZROM499-08	RV-06-N001		*Lycaenidae*	*Polyommatus*	*dorylas*	Apuseni Mts., Rimetea (Piatra Secuiului)	Alba	Transylvania	Romania	750
HQ005038	EZROM697-08	RV-07-F561		*Lycaenidae*	*Polyommatus*	*dorylas*	3 km S of Fundata	Arges	Muntenia	Romania	1190
HQ005042	EZRMN368-08	RVcoll.08-M735		*Lycaenidae*	*Polyommatus*	*dorylas*	Oriental Carpathians, Hasmasul Mare	Harghita	Transylvania	Romania	1700
HQ005041	EZRMN369-08	RVcoll.08-M736		*Lycaenidae*	*Polyommatus*	*dorylas*	Oriental Carpathians, Hasmasul Mare	Harghita	Transylvania	Romania	1700
HQ005043	EZRMN202-08	RVcoll.08-M641		*Lycaenidae*	*Polyommatus*	*dorylas*	Scorota (Retezat Mts.)	Hunedoara	Transylvania	Romania	1560
HM901524	EZSPC1390-10	RVcoll.09-X579	x	*Lycaenidae*	*Polyommatus*	*dorylas*	2 km W Nagore		Navarra	Spain	680
GU676276	EZSPN878-09	RVcoll.08-L779	x	*Lycaenidae*	*Polyommatus*	*dorylas*	San Andres		Cantabria	Spain	972
HM901268	EZSPC1065-10	RVcoll.08-L168	x	*Lycaenidae*	*Polyommatus*	*dorylas*	Font del Regatxal, Vilafranca	Castellon	Comunidad Valenciana	Spain	1361
GU675762	EZSPM117-09	RVcoll.08-P600	x	*Lycaenidae*	*Polyommatus*	*dorylas*	Piscifactoria, Laguna de Una, Una	Cuenca	Castilla-La Mancha	Spain	1219
HM901427	EZSPC1231-10	RVcoll.09-V696	x	*Lycaenidae*	*Polyommatus*	*dorylas*	Meranges, La Cerdanya	Girona	Catalonia	Spain	1744
GU675678	EZSPC650-09	RVcoll.07-C627	x	*Lycaenidae*	*Polyommatus*	*dorylas*	Meranges	Girona	Catalonia	Spain	1555
GU669606	EZSPC682-09	RVcoll.08-R162	x	*Lycaenidae*	*Polyommatus*	*dorylas*	Llanars, Ripolles	Girona	Catalonia	Spain	1119
HM901431	EZSPC1235-10	RVcoll.09-V715	x	*Lycaenidae*	*Polyommatus*	*dorylas*	Yesero	Huesca	Aragon	Spain	1160
HM901521	EZSPC1387-10	RVcoll.09-X550	x	*Lycaenidae*	*Polyommatus*	*dorylas*	Puerto de San Isidro	Leon	Castilla y Leon	Spain	1215
GU675763	EZSPM159-09	RVcoll.08-P685	x	*Lycaenidae*	*Polyommatus*	*dorylas*	Villadangos de Arbas	Leon	Castilla y Leon	Spain	1311
GU669623	EZSPC659-09	RVcoll.07-F024	x	*Lycaenidae*	*Polyommatus*	*dorylas*	Boixols, Pallars Jussa	Lleida	Catalonia	Spain	1240
GU669605	EZSPC681-09	RVcoll.08-R106	x	*Lycaenidae*	*Polyommatus*	*dorylas*	Salt de Pitx, Vall d`Aran	Lleida	Catalonia	Spain	1570
GU675796	EZSPM267-09	RVcoll.08-R483	x	*Lycaenidae*	*Polyommatus*	*dorylas*	Folgoso do Caurel, Estr. Ferramulin-Visuna Km. 18-20	Lugo	Galicia	Spain	850
HM901517	EZSPC1383-10	RVcoll.09-X522	x	*Lycaenidae*	*Polyommatus*	*dorylas*	2 km NW El Vallecillo	Teruel	Aragon	Spain	1450
JN084685		RVcoll.07-D815		*Lycaenidae*	*Polyommatus*	*eros*	Filettino, Monti Simbruini, sky resort		Frosinone	Italy	
HM901446	EZSPC1251-10	RVcoll.09-V839	x	*Lycaenidae*	*Polyommatus*	*eros*	Refugi d`Ulldeter, Ripolles	Girona	Catalonia	Spain	2263.1
GU675683	EZSPM185-09	RVcoll.08-R181	x	*Lycaenidae*	*Polyommatus*	*eros*	Vallter 2000, Setcases, Ripolles	Girona	Catalonia	Spain	2538
GU676070	EZSPM410-09	Rvcoll.09-V828	x	*Lycaenidae*	*Polyommatus*	*eros*	Refugi d`Ulldeter, Ripolles	Girona	Catalonia	Spain	
GU676967	EZSPN259-09	RVcoll.07-W163	x	*Lycaenidae*	*Polyommatus*	*eros*	Vallibierna	Huesca	Aragon	Spain	1800
GU676964	EZSPN260-09	RVcoll.07-W164	x	*Lycaenidae*	*Polyommatus*	*eros*	Vallibierna	Huesca	Aragon	Spain	1800
HM901366	EZSPC1166-10	RVcoll.09-T064	x	*Lycaenidae*	*Polyommatus*	*eros*	Tuc de la Cigalera, Vall d`Aran	Lleida	Catalonia	Spain	2400
GU675793	EZSPM195-09	RVcoll.08-R232	x	*Lycaenidae*	*Polyommatus*	*eros*	Port de la Bonaigua, Pallars Sobira	Lleida	Catalonia	Spain	2338
GU675794	EZSPM196-09	RVcoll.08-R233	x	*Lycaenidae*	*Polyommatus*	*eros*	Port de la Bonaigua, Pallars Sobira	Lleida	Catalonia	Spain	2338
GU676758	EZSPN274-09	RVcoll.07-W199	x	*Lycaenidae*	*Polyommatus*	*eros*	Tuc de la Cigalera, Vall d`Aran	Lleida	Catalonia	Spain	2330
GU676759	EZSPN275-09	RVcoll.07-W200	x	*Lycaenidae*	*Polyommatus*	*eros*	Tuc de la Cigalera, Vall d`Aran	Lleida	Catalonia	Spain	2330
JN084684		RVcoll.09-X293		*Lycaenidae*	*Polyommatus*	*eros*	Simplon Pass		Valais	Switzerland	
JN084682		RVcoll.07-D671		*Lycaenidae*	*Polyommatus*	*eros* (*eroides*)	Rila mountains, Rila/Samokov		Rila Mts.	Bulgaria	
JN084683		RVcoll.09-V961		*Lycaenidae*	*Polyommatus*	*eros* (*eroides*)	N Pirin Mts., Bunderitse nut-Vihren hut road		Pirin Mts.	Bulgaria	
JN084690		RVcoll.09-X075		*Lycaenidae*	*Polyommatus*	*eros* (*menelaos*)	Mt. Taygetos		Peloponessos	Greece	
HM901629	EZSPC774-10	RVcoll.08-L787	x	*Lycaenidae*	*Polyommatus*	*escheri*	San Andres		Cantabria	Spain	972
HM901630	EZSPC775-10	RVcoll.08-L791	x	*Lycaenidae*	*Polyommatus*	*escheri*	San Andres		Cantabria	Spain	972
KP870688	EZSPM745-12	RVcoll.12-L052	x	*Lycaenidae*	*Polyommatus*	*escheri*	Nieva de Cameros, La Rioja		La Rioja	Spain	850
GU676279	EZSPN882-09	RVcoll.08-L789	x	*Lycaenidae*	*Polyommatus*	*escheri*	San Andres		Cantabria	Spain	972
GU677022	EZSPN192-09	RVcoll.07-C477	x	*Lycaenidae*	*Polyommatus*	*escheri*	El Brull, Osona	Barcelona	Catalonia	Spain	
GU676430	EZSPN719-09	RVcoll.08-L105	x	*Lycaenidae*	*Polyommatus*	*escheri*	Riu Bergantes, Morella	Castellon	Comunidad Valenciana	Spain	744
GU676399	EZSPN748-09	RVcoll.08-L192	x	*Lycaenidae*	*Polyommatus*	*escheri*	La Vega, Vilafranca	Castellon	Comunidad Valenciana	Spain	1124
JX093465		MAT-99-Q838	x	*Lycaenidae*	*Polyommatus*	*escheri*	Urús	Girona	Catalonia	Spain	
HM901597	EZSPC742-10	RVcoll.08-J995	x	*Lycaenidae*	*Polyommatus*	*escheri*	Casillas de Rojas, Gueejar Sierra	Granada	Andalusia	Spain	1733
HM901740	EZSPC901-10	RVcoll.09-V518	x	*Lycaenidae*	*Polyommatus*	*escheri*	Road to Veleta (Sierra Nevada)	Granada	Andalusia	Spain	2640
HM901762	EZSPC925-10	RVcoll.09-V921	x	*Lycaenidae*	*Polyommatus*	*escheri*	Sierra de la Sagra	Granada	Andalusia	Spain	1745
HM901763	EZSPC926-10	RVcoll.09-V923	x	*Lycaenidae*	*Polyommatus*	*escheri*	Sierra de la Sagra	Granada	Andalusia	Spain	1745
KP871032	EZSPM755-12	RVcoll.12-L163	x	*Lycaenidae*	*Polyommatus*	*escheri*	Sierra de la Sagra	Granada	Andalusia	Spain	
GU676482	EZSPN663-09	RVcoll.08-J966	x	*Lycaenidae*	*Polyommatus*	*escheri*	Acceso Veleta, Monachil	Granada	Andalusia	Spain	2737

GenBank accession number	BOLD Process ID	Sample ID	Used in the Iberian dataset	Family	Genus	Species	Exact Site	Region	State/Province	Country	Alt. (m)
GU676477	EZSPN667-09	RVcoll.08-J971	x	*Lycaenidae*	*Polyommatus*	*escheri*	Acceso Veleta, Monachil	Granada	Andalusia	Spain	2737
GU676478	EZSPN668-09	RVcoll.08-J973	x	*Lycaenidae*	*Polyommatus*	*escheri*	Acceso Veleta, Monachil	Granada	Andalusia	Spain	2737
GU676479	EZSPN669-09	RVcoll.08-J975	x	*Lycaenidae*	*Polyommatus*	*escheri*	Acceso Veleta, Monachil	Granada	Andalusia	Spain	2737
HM901639	EZSPC786-10	RVcoll.08-M036	x	*Lycaenidae*	*Polyommatus*	*escheri*	Gualda, La Alcarria	Guadalajara	Castilla-La Mancha	Spain	730
HM901640	EZSPC787-10	RVcoll.08-M037	x	*Lycaenidae*	*Polyommatus*	*escheri*	Gualda, La Alcarria	Guadalajara	Castilla-La Mancha	Spain	730
GU676140	EZSPN1016-09	RVcoll.08-M941	x	*Lycaenidae*	*Polyommatus*	*escheri*	Sobrecastells	Huesca	Aragon	Spain	686
GU676149	EZSPN1011-09	RVcoll.08-M924	x	*Lycaenidae*	*Polyommatus*	*escheri*	Arros, Vall d`Aran	Lleida	Catalonia	Spain	906
GU675980	EZSPM075-09	RVcoll.08-P319	x	*Lycaenidae*	*Polyommatus*	*escheri*	Farena, Alt Camp	Tarragona	Catalonia	Spain	580
HM901481	EZSPC1326-10	RVcoll.130209KL20	x	*Lycaenidae*	*Polyommatus*	*escheri*	Albarracin,Albarracin	Teruel	Aragon	Spain	
GU676481	EZSPN642-09	RVcoll.08-J776	x	*Lycaenidae*	*Polyommatus*	*escheri*	Ruta de la Sima, El Vallecillo	Teruel	Aragon	Spain	1531
JN276880	EZSPC1272-10	RVcoll.280808DF13	x	*Lycaenidae*	*Polyommatus*	*escheri*	Arroyo Regajo,Sinarcas	Valencia	Comunidad Valenciana	Spain	753
JN276886	EZSPC1321-10	RVcoll.130209KL53	x	*Lycaenidae*	*Polyommatus*	*escheri*	El Regajo,Sinarcas	Valencia	Comunidad Valenciana	Spain	
KP870940	EZSPM746-12	RVcoll.12-L126	x	*Lycaenidae*	*Polyommatus*	*fabressei*	Munilla		La Rioja	Spain	840
HM901482	EZSPC1327-10	RVcoll.121109PY07	x	*Lycaenidae*	*Polyommatus*	*fabressei*	Pico Cruces,Cortes de Arenoso	Castellon	Comunidad Valenciana	Spain	1697
HM901609	EZSPC754-10	RVcoll.08-L212	x	*Lycaenidae*	*Polyommatus*	*fabressei*	Font del Regatxal, Vilafranca	Castellon	Comunidad Valenciana	Spain	1361
GU675905	EZSPM331-09	RVcoll.08-R069	x	*Lycaenidae*	*Polyommatus*	*fabressei*	La Pobla d`Alcolea	Castellon	Comunidad Valenciana	Spain	
GU675906	EZSPM332-09	RVcoll.08-R070	x	*Lycaenidae*	*Polyommatus*	*fabressei*	La Pobla d`Alcolea	Castellon	Comunidad Valenciana	Spain	
GU675903	EZSPM333-09	RVcoll.08-R071	x	*Lycaenidae*	*Polyommatus*	*fabressei*	La Pobla d`Alcolea	Castellon	Comunidad Valenciana	Spain	
GU675904	EZSPM334-09	RVcoll.08-R073	x	*Lycaenidae*	*Polyommatus*	*fabressei*	La Pobla d`Alcolea	Castellon	Comunidad Valenciana	Spain	
GU675901	EZSPM335-09	RVcoll.08-R083	x	*Lycaenidae*	*Polyommatus*	*fabressei*	Rio Tornos	Castellon	Comunidad Valenciana	Spain	845
GU675902	EZSPM336-09	RVcoll.08-R084	x	*Lycaenidae*	*Polyommatus*	*fabressei*	Rio Tornos	Castellon	Comunidad Valenciana	Spain	845
GU675899	EZSPM337-09	RVcoll.08-R085	x	*Lycaenidae*	*Polyommatus*	*fabressei*	Rio Tornos	Castellon	Comunidad Valenciana	Spain	845
GU675900	EZSPM338-09	RVcoll.08-R086	x	*Lycaenidae*	*Polyommatus*	*fabressei*	Rio Tornos	Castellon	Comunidad Valenciana	Spain	845
GU675886	EZSPM352-09	RVcoll.08-R090	x	*Lycaenidae*	*Polyommatus*	*fabressei*	Rio Tornos	Castellon	Comunidad Valenciana	Spain	845
GU675883	EZSPM353-09	RVcoll.08-R080	x	*Lycaenidae*	*Polyommatus*	*fabressei*	La Pobla d`Alcolea	Castellon	Comunidad Valenciana	Spain	
KP870758	EZSPM823-12	RVcoll.140611ZG01	x	*Lycaenidae*	*Polyommatus*	*fabressei*	Moli de Joaquim, La Pobla de Benifassa	Castellon	Comunidad Valenciana	Spain	1025
KP870804	EZSPM827-12	RVcoll.140611ZG90	x	*Lycaenidae*	*Polyommatus*	*fabressei*	Moli de Joaquim, La Pobla de Benifassa	Castellon	Comunidad Valenciana	Spain	1025
KP870754	EZSPM828-12	RVcoll.140611ZG95	x	*Lycaenidae*	*Polyommatus*	*fabressei*	Moli de Joaquim, La Pobla de Benifassa	Castellon	Comunidad Valenciana	Spain	1025
KP870374	EZSPM879-12	Rvcoll.150310ZX38	x	*Lycaenidae*	*Polyommatus*	*fabressei*	Limite con Teruel, Vistavella	Castellon	Comunidad Valenciana	Spain	1156
GU677001	EZSPN218-09	RVcoll.07-F079	x	*Lycaenidae*	*Polyommatus*	*fabressei*	Olocau del Rey	Castellon	Comunidad Valenciana	Spain	975
GU677041	EZSPN220-09	RVcoll.07-F212	x	*Lycaenidae*	*Polyommatus*	*fabressei*	10 km S. of Morella	Castellon	Comunidad Valenciana	Spain	900
KP870321	EZSPN746-09	RVcoll.08-L175	x	*Lycaenidae*	*Polyommatus*	*fabressei*	Font del Regatxal, Vilafranca	Castellon	Comunidad Valenciana	Spain	1361
EF104605.2		RV03-H596	x	*Lycaenidae*	*Polyommatus*	*fabressei*	Coll d´Ares	Castellón	Comunidad Valenciana	Spain	1148
GU676746	EZSPN286-09	RVcoll.07-W234	x	*Lycaenidae*	*Polyommatus*	*fabressei*	Sierra de los Barrancos, Cuenca	Cuenca	Castilla-La Mancha	Spain	1447
HM210165		MAT99-Q972	x	*Lycaenidae*	*Polyommatus*	*fabressei*	Uña	Cuenca	Castilla-La Mancha	Spain	970

GenBank accession number	BOLD Process ID	Sample ID	Used in the Iberian dataset	Family	Genus	Species	Exact Site	Region	State/Province	Country	Alt. (m)
KP870582	EZSPM749-12	RVcoll.12-L135	x	*Lycaenidae*	*Polyommatus*	*fabressei*	Fuentes de Magana	Soria	Castilla y Leon	Spain	1145
GU676234	EZSPN919-09	RVcoll.08-L864	x	*Lycaenidae*	*Polyommatus*	*fabressei*	Abejar	Soria	Castilla y Leon	Spain	1155
GU676235	EZSPN920-09	RVcoll.08-L865	x	*Lycaenidae*	*Polyommatus*	*fabressei*	Abejar	Soria	Castilla y Leon	Spain	1155
HM901610	EZSPC755-10	RVcoll.08-L241	x	*Lycaenidae*	*Polyommatus*	*fabressei*	Ruta de la Sima, El Vallecillo	Teruel	Aragon	Spain	1531
HM901663	EZSPC814-10	RVcoll.08-P613	x	*Lycaenidae*	*Polyommatus*	*fabressei*	Montes cerca Puerto Cubillo	Teruel	Aragon	Spain	1672
HM901788	EZSPC952-10	RVcoll.09-X504	x	*Lycaenidae*	*Polyommatus*	*fabressei*	2 km NW El Vallecillo	Teruel	Aragon	Spain	1450
KP871122	EZSPM866-12	Rvcoll.080611XP76	x	*Lycaenidae*	*Polyommatus*	*fabressei*	Solano de la Vega, Alcala de la Selva	Teruel	Aragon	Spain	1501
AY496744.2		MAT99-Q984	x	*Lycaenidae*	*Polyommatus*	*fabressei*	Puerto de la Losilla, Albarracín	Teruel	Aragón	Spain	
HM901458	EZSPC1280-10	RVcoll.121109PY21	x	*Lycaenidae*	*Polyommatus*	*fabressei*	Barranco del Regajo,Castielfabib	Valencia	Comunidad Valenciana	Spain	1250
HM901467	EZSPC1295-10	RVcoll.290808XZ3	x	*Lycaenidae*	*Polyommatus*	*fabressei*	Barranco del Covarron,Castielfabib	Valencia	Comunidad Valenciana	Spain	1300
HM901476	EZSPC1304-10	RVcoll.121109PY22	x	*Lycaenidae*	*Polyommatus*	*fabressei*	Barranco del Regajo,Castielfabib	Valencia	Comunidad Valenciana	Spain	1250
HM901483	EZSPC1328-10	RVcoll.130209KL89	x	*Lycaenidae*	*Polyommatus*	*fabressei*	Sierra del Negrete,Utiel	Valencia	Comunidad Valenciana	Spain	1110
HM901635	EZSPC780-10	RVcoll.08-L898	x	*Lycaenidae*	*Polyommatus*	*fulgens*	San Andres		Cantabria	Spain	976
GU676077	EZSPM425-09	Rvcoll.09-X583	x	*Lycaenidae*	*Polyommatus*	*fulgens*	2 km E Navascues		Navarra	Spain	680
GU676074	EZSPM426-09	Rvcoll.09-X565	x	*Lycaenidae*	*Polyommatus*	*fulgens*	Sorauren		Navarra	Spain	530
GU676214	EZSPN937-09	RVcoll.08-L899	x	*Lycaenidae*	*Polyommatus*	*fulgens*	San Andres		Cantabria	Spain	976
GU676212	EZSPN952-09	RVcoll.08-L944	x	*Lycaenidae*	*Polyommatus*	*fulgens*	San Andres		Cantabria	Spain	976
GU676202	EZSPN954-09	RVcoll.08-L947	x	*Lycaenidae*	*Polyommatus*	*fulgens*	San Andres		Cantabria	Spain	977
GU677013	EZSPN205-09	RVcoll.07-F036	x	*Lycaenidae*	*Polyommatus*	*fulgens*	El Brull, Osona	Barcelona	Catalonia	Spain	835
GU675973	EZSPM001-09	RVcoll.08-M964	x	*Lycaenidae*	*Polyommatus*	*fulgens*	Saganta	Huesca	Aragon	Spain	7675
JN276906	EZSPN1114-11	Rvcoll.10-C313	x	*Lycaenidae*	*Polyommatus*	*fulgens*	Acisa de las Arrimadas	Leon	Castilla y Leon	Spain	1080
GU675974	EZSPM077-09	RVcoll.08-P327	x	*Lycaenidae*	*Polyommatus*	*fulgens*	Perves, Pallars Jussa	Lleida	Catalonia	Spain	1072
GU677026	EZSPN188-09	RVcoll.07-C091	x	*Lycaenidae*	*Polyommatus*	*fulgens*	Valldarques, Alt Urgell	Lleida	Catalonia	Spain	975
AY496712.2		MAT-99-Q894	x	*Lycaenidae*	*Polyommatus*	*fulgens*	Tremp, Rúbies	Lleida	Catalonia	Spain	
AY496746.2		MAT99-Q910	x	*Lycaenidae*	*Polyommatus*	*fulgens*	Santa Coloma de Queralt	Tarragona	Catalonia	Spain	
HM901733	EZSPC894-10	RVcoll.09-V421	x	*Lycaenidae*	*Polyommatus*	*golgus*	San Juan (Sierra Nevada)	Granada	Andalusia	Spain	2760
HM901735	EZSPC896-10	RVcoll.09-V476	x	*Lycaenidae*	*Polyommatus*	*golgus*	Sierra de la Sagra	Granada	Andalusia	Spain	1745
HM901736	EZSPC897-10	RVcoll.09-V480	x	*Lycaenidae*	*Polyommatus*	*golgus*	Sierra de la Sagra	Granada	Andalusia	Spain	1745
HM901737	EZSPC898-10	RVcoll.09-V482	x	*Lycaenidae*	*Polyommatus*	*golgus*	Sierra de la Sagra	Granada	Andalusia	Spain	1745
HM901738	EZSPC899-10	RVcoll.09-V487	x	*Lycaenidae*	*Polyommatus*	*golgus*	Sierra de la Sagra	Granada	Andalusia	Spain	1745
HM901741	EZSPC902-10	RVcoll.09-V523	x	*Lycaenidae*	*Polyommatus*	*golgus*	Road to Veleta (Sierra Nevada)	Granada	Andalusia	Spain	2640
KP871048	EZSPM655-12	RVcoll.11-I480	x	*Lycaenidae*	*Polyommatus*	*golgus*	Road to Veleta (Sierra Nevada)	Granada	Andalusia	Spain	2725
KP870589	EZSPM656-12	RVcoll.11-I481	x	*Lycaenidae*	*Polyommatus*	*golgus*	Road to Veleta (Sierra Nevada)	Granada	Andalusia	Spain	2725
KP870856	EZSPM657-12	RVcoll.11-I482	x	*Lycaenidae*	*Polyommatus*	*golgus*	Road to Veleta (Sierra Nevada)	Granada	Andalusia	Spain	2725
GU676718	EZSPN167-09	RVcoll.06-K693	x	*Lycaenidae*	*Polyommatus*	*golgus*	Guillimona, La Sagra, HUESCAR	Granada	Andalusia	Spain	1750
HM901840	EZSPN168-09	RVcoll.06-K694	x	*Lycaenidae*	*Polyommatus*	*golgus*	Guillimona, La Sagra, HUESCAR	Granada	Andalusia	Spain	1750
GU677044	EZSPN174-09	RVcoll.06-K710	x	*Lycaenidae*	*Polyommatus*	*golgus*	Prados de Otero,Sierra Nevada, GUeEJAR SIERRA	Granada	Andalusia	Spain	2225
GU676485	EZSPN666-09	RVcoll.08-J970	x	*Lycaenidae*	*Polyommatus*	*golgus*	Acceso Veleta, Monachil	Granada	Andalusia	Spain	2737
GU676480	EZSPN670-09	RVcoll.08-J976	x	*Lycaenidae*	*Polyommatus*	*golgus*	Acceso Veleta, Monachil	Granada	Andalusia	Spain	2737
GU676471	EZSPN671-09	RVcoll.08-J977	x	*Lycaenidae*	*Polyommatus*	*golgus*	Acceso Veleta, Monachil	Granada	Andalusia	Spain	2737
GU677053	EZSPN673-09	RVcoll.08-J979	x	*Lycaenidae*	*Polyommatus*	*golgus*	Acceso Veleta, Monachil	Granada	Andalusia	Spain	2737
HM210169		RE07-G191		*Lycaenidae*	*Polyommatus*	*humedasae*	Val di Cogne, Ozein-Visyes		Aosta	Italy	1000
HM210170		RE07-G203		*Lycaenidae*	*Polyommatus*	*humedasae*	Val di Cogne, Ozein-Visyes		Aosta	Italy	1000
JN084695		RVcoll.08-L303		*Lycaenidae*	*Polyommatus*	*icarus*	Hammer Bakker		Aalborg	Denmark	
JN084694		RVcoll.07-C710		*Lycaenidae*	*Polyommatus*	*icarus*	Uusimaa Loviisa Harmaakallio		Uusima	Finland	
KM459359	EULEP102-14	RVcoll.09-V243		*Lycaenidae*	*Polyommatus*	*icarus*	Domaine de la chasse de Puits de Rians, Provence		Provence	France	
KM459367	EULEP113-14	RVcoll.09-X210		*Lycaenidae*	*Polyommatus*	*icarus*	Oraison, Provence		Provence	France	
JN084688		RVcoll.09-X222		*Lycaenidae*	*Polyommatus*	*icarus*	Sault		Provence	France	
JN084698		RVcoll.09-X260		*Lycaenidae*	*Polyommatus*	*icarus*	Bédoine		Provence	France	
JN084701		RVcoll.09-T515		*Lycaenidae*	*Polyommatus*	*icarus*	Bonifacio		Corsica	France	
JX678166		LD-385		*Lycaenidae*	*Polyommatus*	*icarus*	Corsica dint. Pietralba		Corsica	France	

GenBank accession number	BOLD Process ID	Sample ID	Used in the Iberian dataset	Family	Genus	Species	Exact Site	Region	State/Province	Country	Alt. (m)
GU688449	GWORO805-09	BC ZSM Lep 30497		*Lycaenidae*	*Polyommatus*	*icarus*	Koenigsdorf TOEL		Bavaria	Germany	
HM391783	FBLMT894-09	BC ZSM Lep 25454		*Lycaenidae*	*Polyommatus*	*icarus*	Wiesenfelden, Hoehenberg	Niederbayern	Bavaria	Germany	652
GU655005	FBLMT902-09	BC ZSM Lep 25462		*Lycaenidae*	*Polyommatus*	*icarus*	Muenchen, Obermenzing	Oberbayern	Bavaria	Germany	520
JF415727	GWORK562-09	BC ZSM Lep 21892		*Lycaenidae*	*Polyommatus*	*icarus*	Karwendelgebirge, Mittenwald, Vereinsalm	Oberbayern	Bavaria	Germany	1600
HQ563555	FBLMW315-10	BC ZSM Lep 37416		*Lycaenidae*	*Polyommatus*	*icarus*	Toepen, Fattigsmuehle	Oberfranken	Bavaria	Germany	482
JN084693		RVcoll.07-C552		*Lycaenidae*	*Polyommatus*	*icarus*	Steinheim, Hungen		Giessen	Germany	
KP871006	EULEP004-14	150308PP67		*Lycaenidae*	*Polyommatus*	*icarus*	Montes de Ornos		Crete	Greece	
JN084691		RVcoll.150308PP10		*Lycaenidae*	*Polyommatus*	*icarus*	Montes de Ornos		Crete	Greece	
JN084692		RVcoll.150308PP62		*Lycaenidae*	*Polyommatus*	*icarus*	Montes de Ornos		Crete	Greece	
JN084709		RVcoll.150308PP22		*Lycaenidae*	*Polyommatus*	*icarus*	Bahía de Almyros, Almyros		Crete	Greece	
KM459330	EULEP032-14	RVcoll.07-D864		*Lycaenidae*	*Polyommatus*	*icarus*	Filettino, Monti Simbruini		Frosinone	Italy	
KM459331	EULEP033-14	RVcoll.07-D899		*Lycaenidae*	*Polyommatus*	*icarus*	Serra del Prete, Mont Pollino		Calabria	Italy	
KM459332	EULEP034-14	RVcoll.07-E051		*Lycaenidae*	*Polyommatus*	*icarus*	P.N. La Sila, Calabria		Calabria	Italy	
KM459357	EULEP095-14	RVcoll.09-T560		*Lycaenidae*	*Polyommatus*	*icarus*	Capri island		Capri	Italy	
KM459358	EULEP097-14	RVcoll.09-T572		*Lycaenidae*	*Polyommatus*	*icarus*	Monte Perone Elba island		Elba	Italy	
KM459437	EULEP268-14	LD-292		*Lycaenidae*	*Polyommatus*	*icarus*	Aspromonte, Santuario Polsi		Calabria	Italy	
JN820118	PHLAC364-10	TLMF Lep 02399		*Lycaenidae*	*Polyommatus*	*icarus*	Ritten/ Obergruenwald		South Tyrol	Italy	1750
JN084700		RVcoll.07-D812		*Lycaenidae*	*Polyommatus*	*icarus*	Ostia Antica		Rome	Italy	
JN084702		RVcoll.07-E062		*Lycaenidae*	*Polyommatus*	*icarus*	Monte Sirino, Potenza		Basilicata	Italy	
JN084703		RVcoll.07-E207		*Lycaenidae*	*Polyommatus*	*icarus*	Ozein-Visyes, Cogne Valley		Aosta	Italy	
JN084705		RVcoll.07-D884		*Lycaenidae*	*Polyommatus*	*icarus*	Carpineto Romano, Monti Lepini		Rome	Italy	
JN084706		RVcoll.09-T559		*Lycaenidae*	*Polyommatus*	*icarus*	Salerno, Capri island		Capri	Italy	
KM459341	EULEP051-14	RVcoll.08-J150	x	*Lycaenidae*	*Polyommatus*	*icarus*	Ponte Pedrinha, Beça		Boticas	Portugal	
KM459342	EULEP052-14	RVcoll.08-J199	x	*Lycaenidae*	*Polyommatus*	*icarus*	Serra do Larouco, Padornelos		Montalegre	Portugal	
GU676569	EZSPN578-09	RVcoll.08-J127	x	*Lycaenidae*	*Polyommatus*	*icarus*	Seculca, Beca, Boticas		Tras-os-Montes	Portugal	790
HQ005053	EZRMN207-08	RVcoll.08-M374		*Lycaenidae*	*Polyommatus*	*icarus*	Buciumeni forest (Rădăcineşti)	Bacău	Moldavia	Romania	180
HQ005051	EZROM500-08	RV-06-M943		*Lycaenidae*	*Polyommatus*	*icarus*	Dealul Istriţa, Breaza	Buzău	Muntenia	Romania	600
HQ005048	EZROM502-08	RV-07-D104		*Lycaenidae*	*Polyommatus*	*icarus*	Ciolanu	Buzău	Muntenia	Romania	255
HQ005047	EZROM504-08	RV-07-C964		*Lycaenidae*	*Polyommatus*	*icarus*	Băile Herculane (Pecinisca)	Caraş-Severin	Banat	Romania	220
HQ005049	EZROM501-08	RV-06-V653		*Lycaenidae*	*Polyommatus*	*icarus*	Făget forest, Râtu Penii	Cluj	Transylvania	Romania	680
HQ005050	EZROM503-08	RV-07-D439		*Lycaenidae*	*Polyommatus*	*icarus*	Suatu	Cluj	Transylvania	Romania	380
HQ005054	EZRMN209-08	RVcoll.08-M425		*Lycaenidae*	*Polyommatus*	*icarus*	Gura Dobrogei reserve	Constanţa	Dobrogea	Romania	90
HQ005056	EZRMN206-08	RVcoll.08-M230		*Lycaenidae*	*Polyommatus*	*icarus*	2 Km E of Moreni	Dâmboviţa	Muntenia	Romania	290
HQ005052	EZRMN208-08	RVcoll.08-M630		*Lycaenidae*	*Polyommatus*	*icarus*	Scorota (Retezat Mts.)	Hunedoara	Transylvania	Romania	1560
HQ005046	EZROM593-08	RV-07-C366		*Lycaenidae*	*Polyommatus*	*icarus*	Sadova, pas Paşcanu	Suceava	Moldavia	Romania	1100
HQ005055	EZRMN210-08	RVcoll.07-D053		*Lycaenidae*	*Polyommatus*	*icarus*	4 Km SE of Enisala	Tulcea	Dobrogea	Romania	50
GU675755	EZSPM141-09	RVcoll.08-P653	x	*Lycaenidae*	*Polyommatus*	*icarus*	Valle (Valle-Zurea), Lena		Asturias	Spain	662
KM517856	EZSPM753-12	RVcoll.12-L153	x	*Lycaenidae*	*Polyommatus*	*icarus*	Valdevigas-Enciso		La Rioja	Spain	850
KM517862	EZSPM754-12	RVcoll.12-L159	x	*Lycaenidae*	*Polyommatus*	*icarus*	Sierra de Alcarama, Valdemadera		La Rioja	Spain	1190
KM517858	EZSPM873-12	Rvcoll.130711PX19	x	*Lycaenidae*	*Polyommatus*	*icarus*	Rio Muniellos, Cangas del Narcea		Asturias	Spain	594
KM517857	EZSPM874-12	Rvcoll.130711PX33	x	*Lycaenidae*	*Polyommatus*	*icarus*	Casa de la Luz, Degana		Asturias	Spain	800
GU676654	EZSPN384-09	RVcoll.08-H434	x	*Lycaenidae*	*Polyommatus*	*icarus*	Campo Real		Comunidad de Madrid	Spain	731
GU675791	EZSPM246-09	RVcoll.08-R458	x	*Lycaenidae*	*Polyommatus*	*icarus*	Padron, Picarana-Rio Tinto	A Coruña	Galicia	Spain	30
KM459387	EULEP160-14	11-E016	x	*Lycaenidae*	*Polyommatus*	*icarus*	Font de l'Arbre, Serra d'Aitana	Alicante	Comunidad Valenciana	Spain	1209
GU676372	EZSPN772-09	RVcoll.08-L281	x	*Lycaenidae*	*Polyommatus*	*icarus*	Los Collados - Benimaurell, Vall de Laguart	Alicante	Comunidad Valenciana	Spain	675
JN114417	EZROM751-08	RV-06-G433	x	*Lycaenidae*	*Polyommatus*	*icarus*	Can Rovira, El Brull	Barcelona	Catalonia	Spain	830
JN114415	EZSPC284-09	RVcoll.07-C463	x	*Lycaenidae*	*Polyommatus*	*icarus*	UAB campus, BMS transect, Bellaterra	Barcelona	Catalonia	Spain	158
GU669625	EZSPC662-09	RVcoll.08-H232	x	*Lycaenidae*	*Polyommatus*	*icarus*	UAB, Bellaterra	Barcelona	Catalonia	Spain	184
KM517859	EZSPM751-12	RVcoll.12-L141	x	*Lycaenidae*	*Polyommatus*	*icarus*	Huerta del Rey	Burgos	Castilla y Leon	Spain	1150
HM901614	EZSPC759-10	RVcoll.08-L276	x	*Lycaenidae*	*Polyommatus*	*icarus*	Mas de Cap del Pla a Mas del Pont, Vistabella del Maestrazgo	Castellon	Comunidad Valenciana	Spain	1145
KM459333	EULEP035-14	RVcoll.07-F062	x	*Lycaenidae*	*Polyommatus*	*icarus*	10 km S. of Morella	Castellón	Comunidad Valenciana	Spain	
KM517852	EZSPM948-12	Rvcoll.12-M687	x	*Lycaenidae*	*Polyommatus*	*icarus*	Rio Jandula, Mestanza	Ciudad Real	Castilla-La Mancha	Spain	407

GenBank accession number	BOLD Process ID	Sample ID	Used in the Iberian dataset	Family	Genus	Species	Exact Site	Region	State/Province	Country	Alt. (m)
JN114416	EZSPC283-09	RVcoll.08-P076	x	*Lycaenidae*	*Polyommatus*	*icarus*	Torrent de Ridolaina, Cerdanya	Girona	Catalonia	Spain	1060
JN114414	EZSPC285-09	RVcoll.07-C623	x	*Lycaenidae*	*Polyommatus*	*icarus*	Meranges	Girona	Catalonia	Spain	1555
KP870765	EULEP027-14	07-D768	x	*Lycaenidae*	*Polyommatus*	*icarus*	La Sagra	Granada	Andalusia	Spain	
KP870739	EULEP028-14	07-D769	x	*Lycaenidae*	*Polyommatus*	*icarus*	La Sagra	Granada	Andalusia	Spain	
KP870755	EULEP029-14	07-D778	x	*Lycaenidae*	*Polyommatus*	*icarus*	La Sagra	Granada	Andalusia	Spain	
KM459344	EULEP069-14	RVcoll.08-J962	x	*Lycaenidae*	*Polyommatus*	*icarus*	Fuente Albergue de San Francisco, Güejar Sierra	Granada	Andalusia	Spain	
KM459361	EULEP104-14	09-V446	x	*Lycaenidae*	*Polyommatus*	*icarus*	San Juan (Sierra Nevada)	Granada	Andalusia	Spain	2760
KM459410	EULEP162-14	11-I454	x	*Lycaenidae*	*Polyommatus*	*icarus*	Road to Veleta (Sierra Nevada)	Granada	Andalusia	Spain	2550-2900
KM459413	EULEP164-14	11-I458	x	*Lycaenidae*	*Polyommatus*	*icarus*	Road to Veleta (Sierra Nevada)	Granada	Andalusia	Spain	2550-2900
GU676489	EZSPN661-09	RVcoll.08-J960	x	*Lycaenidae*	*Polyommatus*	*icarus*	Fuente Albergue de San Francisco, Guejar Sierra	Granada	Andalusia	Spain	2221
GU676484	EZSPN665-09	RVcoll.08-J968	x	*Lycaenidae*	*Polyommatus*	*icarus*	Acceso Veleta, Monachil	Granada	Andalusia	Spain	2737
GU676611	EZSPN426-09	RVcoll.08-H592	x	*Lycaenidae*	*Polyommatus*	*icarus*	Gualda, La Alcarria	Guadalajara	Castilla-La Mancha	Spain	742
KM459335	EULEP048-14	RVcoll.08-H255	x	*Lycaenidae*	*Polyommatus*	*icarus*	Mequinenza	Huesca	Aragón	Spain	
GU676797	EZSPN142-09	RVcoll.06-G507	x	*Lycaenidae*	*Polyommatus*	*icarus*	Sierra de Alcubierre	Huesca	Aragon	Spain	600
GU675911	EZSPM325-09	RVcoll.08-J899	x	*Lycaenidae*	*Polyommatus*	*icarus*	Vega de Espinareda	Leon	Castilla y Leon	Spain	
GU669622	EZSPC666-09	RVcoll.08-L473	x	*Lycaenidae*	*Polyommatus*	*icarus*	Ivars de Noguera	Lleida	Catalonia	Spain	373
GU675792	EZSPM271-09	RVcoll.08-R488	x	*Lycaenidae*	*Polyommatus*	*icarus*	Tui, Rio Louro-Magdalena	Pontevedra	Galicia	Spain	20
KP870761	EULEP234-14	12-L149	x	*Lycaenidae*	*Polyommatus*	*icarus*	Torre Val de San Pedro, Río Cega	Segovia	Castilla y León	Spain	1090
KP870575	EULEP235-14	12-L152	x	*Lycaenidae*	*Polyommatus*	*icarus*	Santibañez de Ayllón	Segovia	Castilla y León	Spain	1180
KM517851	EZSPM752-12	RVcoll.12-L143	x	*Lycaenidae*	*Polyommatus*	*icarus*	Rio Piron, Sierra de Guadarrama	Segovia	Castilla y Leon	Spain	1390
KM459360	EULEP103-14	RVcoll.09-V396	x	*Lycaenidae*	*Polyommatus*	*icarus*	La Tancada, Delta de l'Ebre, Tarragona	Tarragona	Catalonia	Spain	
GU669617	EZSPC669-09	RVcoll.08-M674	x	*Lycaenidae*	*Polyommatus*	*icarus*	Serra Major, Morera de Montsant, Priorat	Tarragona	Catalonia	Spain	1090
KM459346	EULEP071-14	RVcoll.08-L092	x	*Lycaenidae*	*Polyommatus*	*icarus*	Ruta de la Sima, El Vallecillo	Teruel	Aragón	Spain	
KM459374	EULEP120-14	RVcoll.09-X521	x	*Lycaenidae*	*Polyommatus*	*icarus*	2 km NW El Vallecillo	Teruel	Aragón	Spain	
GU675891	EZSPM345-09	RVcoll.08-R025	x	*Lycaenidae*	*Polyommatus*	*icarus*	Sant Vicent, Lliria	Valencia	Comunidad Valenciana	Spain	
KP870585	EULEP236-14	12-L154	x	*Lycaenidae*	*Polyommatus*	*icarus*	Munilla		La Rioja	Spain	860
KP871101	EULEP237-14	12-L155	x	*Lycaenidae*	*Polyommatus*	*icarus*	Cabezón de Cameros		La Rioja	Spain	920
KP870886	EULEP238-14	12-L160	x	*Lycaenidae*	*Polyommatus*	*icarus*	Sierra de Cebollera, Villoslada de Cameros		La Rioja	Spain	1850
KM459362	EULEP106-14	RVcoll.09-V488	x	*Lycaenidae*	*Polyommatus*	*icarus (abdon)*	Sierra de la Sagra	Granada	Andalusia	Spain	
KM459363	EULEP109-14	RVcoll.09-V905	x	*Lycaenidae*	*Polyommatus*	*icarus (abdon)*	Sierra de la Sagra	Granada	Andalusia	Spain	
KM459368	EULEP116-14	09-X366	x	*Lycaenidae*	*Polyommatus*	*icarus (abdon)*	Sierra de la Sagra	Granada	Andalusia	Spain	1670-1820
KM459371	EULEP118-14	09-X372	x	*Lycaenidae*	*Polyommatus*	*icarus (abdon)*	Sierra de la Sagra	Granada	Andalusia	Spain	1670-1820
GU676071	EZSPM465-09	Rvcoll.09-V941	x	*Lycaenidae*	*Polyommatus*	*icarus (abdon)*	Sierra de la Sagra	Granada	Andalusia	Spain	1745
GU676068	EZSPM467-09	Rvcoll.09-V907	x	*Lycaenidae*	*Polyommatus*	*icarus (abdon)*	Sierra de la Sagra	Granada	Andalusia	Spain	1745
GU676767	EZSPN171-09	RVcoll.06-K697	x	*Lycaenidae*	*Polyommatus*	*icarus (abdon)*	Guillimona, La Sagra, Huescar	Granada	Andalusia	Spain	1750
GU676768	EZSPN172-09	RVcoll.06-K698	x	*Lycaenidae*	*Polyommatus*	*icarus (abdon)*	Guillimona, La Sagra, Huescar	Granada	Andalusia	Spain	1750
HM901841	EZSPN173-09	RVcoll.06-K699	x	*Lycaenidae*	*Polyommatus*	*icarus (abdon)*	Guillimona, La Sagra, Huescar	Granada	Andalusia	Spain	1750
JN084699		09-V902	x	*Lycaenidae*	*Polyommatus*	*icarus (abdon)*	Sierra de la Sagra	Granada	Andalusia	Spain	1670-1820
JN084707		RVcoll.09-V486	x	*Lycaenidae*	*Polyommatus*	*icarus (abdon)*	Sierra de la Sagra	Granada	Andalusia	Spain	
JN084697		RVcoll.09-V960		*Lycaenidae*	*Polyommatus*	*icarus (andronicus)*	N Pirin Mts., Banderitsa nut-Vihren hut road		Bansko	Bulgaria	
JN084708		RVcoll.09-V959		*Lycaenidae*	*Polyommatus*	*icarus (andronicus)*	N Pirin Mt., Banderitsa nut-vihren hut road		Bansko	Bulgaria	
KC581745		RVcoll.09-V964		*Lycaenidae*	*Polyommatus*	*nephohiptamenos*	Orelek, Popovi Livadi		Gotze Delchev	Bulgaria	1550
HM901707	EZSPC867-10	RVcoll.09-T101	x	*Lycaenidae*	*Polyommatus*	*nivescens*	Ciudad Encantada	Cuenca	Castilla-La Mancha	Spain	1200
HM901727	EZSPC887-10	RVcoll.09-T157	x	*Lycaenidae*	*Polyommatus*	*nivescens*	Puerto del Cunillo	Cuenca	Castilla-La Mancha	Spain	1620
HM901739	EZSPC900-10	RVcoll.09-V489	x	*Lycaenidae*	*Polyommatus*	*nivescens*	Sierra de la Sagra	Granada	Andalusia	Spain	1745
GU676072	EZSPM459-09	Rvcoll.09-V578	x	*Lycaenidae*	*Polyommatus*	*nivescens*	Dornajo (Sierra Nevada)	Granada	Andalusia	Spain	2060
GU676414	EZSPN728-09	RVcoll.08-L136	x	*Lycaenidae*	*Polyommatus*	*nivescens*	Casillas de Rojas, Gueejar Sierra	Granada	Andalusia	Spain	1733
HM901672	EZSPC824-10	RVcoll.08-P750	x	*Lycaenidae*	*Polyommatus*	*nivescens*	El Vallecillo	Teruel	Aragon	Spain	1529
HM901708	EZSPC868-10	RVcoll.09-T102	x	*Lycaenidae*	*Polyommatus*	*nivescens*	Cercanias de El Vallecillo, Albarracin	Teruel	Aragon	Spain	1527
GU676075	EZSPM429-09	Rvcoll.09-X523	x	*Lycaenidae*	*Polyommatus*	*nivescens*	2 km NW El Vallecillo	Teruel	Aragon	Spain	1450
HM901484	EZSPC1332-10	RVcoll.130209KL56	x	*Lycaenidae*	*Polyommatus*	*nivescens*	Marisancho, Utiel	Valencia	Comunidad Valenciana	Spain	

GenBank accession number	BOLD Process ID	Sample ID	Used in the Iberian dataset	Family	Genus	Species	Exact Site	Region	State/Province	Country	Alt. (m)
KC581746		RVcoll.10-A998		*Lycaenidae*	*Polyommatus*	*ripartii*	Hvoyna		Chepelare	Bulgaria	780
KC581747		RVcoll.11-J903		*Lycaenidae*	*Polyommatus*	*ripartii*	Mostovo, Plovdiv		Asenovgrad	Bulgaria	
KC581748		RVcoll.12-M011		*Lycaenidae*	*Polyommatus*	*ripartii*	Hvoyna		Chepelare	Bulgaria	780
KC581749		RVcoll.12-M012		*Lycaenidae*	*Polyommatus*	*ripartii*	Hvoyna		Chepelare	Bulgaria	780
KC581750		RVcoll.12-M014		*Lycaenidae*	*Polyommatus*	*ripartii*	Hvoyna		Chepelare	Bulgaria	780
KC581751		RVcoll.12-M016		*Lycaenidae*	*Polyommatus*	*ripartii*	Hvoyna		Chepelare	Bulgaria	780
KC581752		RVcoll.12-M017		*Lycaenidae*	*Polyommatus*	*ripartii*	Hvoyna		Chepelare	Bulgaria	780
KC581729		RVcoll.09-V363		*Lycaenidae*	*Polyommatus*	*ripartii*	Mosor Mountain		Dalmatia	Croatia	1100
KC581730		RVcoll.11-G188		*Lycaenidae*	*Polyommatus*	*ripartii*	Mosor Mountain		Dalmatia	Croatia	1000
KC581731		RVcoll.11-G191		*Lycaenidae*	*Polyommatus*	*ripartii*	Mosor Mountain		Dalmatia	Croatia	1000
KC581732		RVcoll.11-G193		*Lycaenidae*	*Polyommatus*	*ripartii*	Mosor Mountain		Dalmatia	Croatia	1000
KC581733		RVcoll.11-G198		*Lycaenidae*	*Polyommatus*	*ripartii*	Mosor Mountain		Dalmatia	Croatia	1000
KC581734		RVcoll.11-G190		*Lycaenidae*	*Polyommatus*	*ripartii*	Mosor Mountain		Dalmatia	Croatia	1000
KC581735		RVcoll.11-G196		*Lycaenidae*	*Polyommatus*	*ripartii*	Mosor Mountain		Dalmatia	Croatia	1000
KC581740		RVcoll.11-G195		*Lycaenidae*	*Polyommatus*	*ripartii*	Mosor Mountain		Dalmatia	Croatia	1000
KC581741		RVcoll.11-G189		*Lycaenidae*	*Polyommatus*	*ripartii*	Mosor Mountain		Dalmatia	Croatia	1000
KC581742		RVcoll.11-G194		*Lycaenidae*	*Polyommatus*	*ripartii*	Mosor Mountain		Dalmatia	Croatia	1000
KC581743		RVcoll.11-G197		*Lycaenidae*	*Polyommatus*	*ripartii*	Mosor Mountain		Dalmatia	Croatia	1000
KC581744		RVcoll.11-G192		*Lycaenidae*	*Polyommatus*	*ripartii*	Mosor Mountain		Dalmatia	Croatia	1000
HM210171		RE07-G266		*Lycaenidae*	*Polyommatus*	*ripartii*	Col de la Chaudière		Drôme	France	1025
KC581717		RVcoll.08-L390		*Lycaenidae*	*Polyommatus*	*ripartii*	Lorgues		Var	France	
KC581720		RVcoll.11-I888		*Lycaenidae*	*Polyommatus*	*ripartii*	Rossas		Drôme	France	930
KC581723		RVcoll.10-B712		*Lycaenidae*	*Polyommatus*	*ripartii*	Le Chaffaut-St-Jurson		Alpes-de-Haute-Provence	France	580
KC581724		RVcoll.11-I703		*Lycaenidae*	*Polyommatus*	*ripartii*	Le Pöet-en-Percip		Drôme	France	977
KC581725		RVcoll.11-I869		*Lycaenidae*	*Polyommatus*	*ripartii*	Marafit, Volvent		Drôme	France	770
KC581726		RVcoll.11-I851		*Lycaenidae*	*Polyommatus*	*ripartii*	Col des Roustans		Drôme	France	950
HM210163		RE07-G254		*Lycaenidae*	*Polyommatus*	*ripartii*	Novalesa-Moncenisio		Torino	Italy	1155
HM210164		RE07-G255		*Lycaenidae*	*Polyommatus*	*ripartii*	Novalesa-Moncenisio		Torino	Italy	1155
HM210167		RE07-G436		*Lycaenidae*	*Polyommatus*	*ripartii*	Serra del Prete, Monte Pollino		Calabria	Italy	1650
HM210168		RE07-G437		*Lycaenidae*	*Polyommatus*	*ripartii*	Serra del Prete, Monte Pollino		Calabria	Italy	1650
HM210172		RE07-G229		*Lycaenidae*	*Polyommatus*	*ripartii*	Urbiano, Mompantero		Torino	Italy	720
KC617794	EZSPC782-10	RVcoll.08-L946	x	*Lycaenidae*	*Polyommatus*	*ripartii*	San Andres		Cantabria	Spain	977
KC617793	EZSPC783-10	RVcoll.08-L948	x	*Lycaenidae*	*Polyommatus*	*ripartii*	San Andres		Cantabria	Spain	977
KC567885	EZSPM747-12	RVcoll.12-L129	x	*Lycaenidae*	*Polyommatus*	*ripartii*	Molinos eolicos, Larriba, Zarzosa		La Rioja	Spain	1460
KC567884	EZSPM750-12	RVcoll.12-L140	x	*Lycaenidae*	*Polyommatus*	*ripartii*	Larriba, Zarzosa		La Rioja	Spain	1420
GU676220	EZSPN932-09	RVcoll.08-L892	x	*Lycaenidae*	*Polyommatus*	*ripartii*	Puerto de Pozazal		Cantabria	Spain	1040
GU676213	EZSPN953-09	RVcoll.08-L945	x	*Lycaenidae*	*Polyommatus*	*ripartii*	San Andres		Cantabria	Spain	977
GU677012	EZSPN204-09	RVcoll.07-F035	x	*Lycaenidae*	*Polyommatus*	*ripartii*	El Brull, Osona	Barcelona	Catalonia	Spain	835
EF104603.2		RV03-H463	x	*Lycaenidae*	*Polyommatus*	*ripartii*	El Brull	Barcelona	Catalonia	Spain	830
HM901664	EZSPC815-10	RVcoll.08-P614.1	x	*Lycaenidae*	*Polyommatus*	*ripartii*	Pinilla de Molina	Guadalajara	Castilla-La Mancha	Spain	1234
GU676152	EZSPN1014-09	RVcoll.08-M935	x	*Lycaenidae*	*Polyommatus*	*ripartii*	Sobrecastells	Huesca	Aragon	Spain	686
KC581736		08-M934	x	*Lycaenidae*	*Polyommatus*	*ripartii*	Sobrecastells, 685 m	Huesca	Aragón	Spain	685
KC581737		09-V723	x	*Lycaenidae*	*Polyommatus*	*ripartii*	Boltaña	Huesca	Aragón	Spain	750
KC581738		09-V724	x	*Lycaenidae*	*Polyommatus*	*ripartii*	Boltaña	Huesca	Aragón	Spain	750
GU677029	EZSPN187-09	RVcoll.07-C089	x	*Lycaenidae*	*Polyommatus*	*ripartii*	Boixols, Pallars Jussa	Lleida	Catalonia	Spain	1240
KC581739		09-X021	x	*Lycaenidae*	*Polyommatus*	*ripartii*	La Farga Vella, 1100 m	Lleida	Catalonia	Spain	1100
KC567883	EZSPM748-12	RVcoll.12-L133	x	*Lycaenidae*	*Polyommatus*	*ripartii*	Tanine	Soria	Castilla y Leon	Spain	1230
HM901559	EZSPC702-10	RVcoll.07-F038	x	*Lycaenidae*	*Polyommatus*	*ripartii*	Barranc de Vinarroig, Prades Mt.	Tarragona	Catalonia	Spain	920
GU676039	EZSPM073-09	RVcoll.08-P316	x	*Lycaenidae*	*Polyommatus*	*ripartii*	Farena, Alt Camp	Tarragona	Catalonia	Spain	580
GU676158	EZSPN1006-09	RVcoll.08-M697	x	*Lycaenidae*	*Polyommatus*	*ripartii*	Farena, Alt Camp	Tarragona	Catalonia	Spain	580
JN276883	EZSPC1289-10	RVcoll.110806MU4	x	*Lycaenidae*	*Polyommatus*	*ripartii*	Albarracin,Albarracin	Teruel	Aragon	Spain	
HM901474	EZSPC1302-10	RVcoll.300107SM82	x	*Lycaenidae*	*Polyommatus*	*ripartii*	Albarracin,Albarracin	Teruel	Aragon	Spain	
GU675760	EZSPM125-09	RVcoll.08-P615	x	*Lycaenidae*	*Polyommatus*	*ripartii*	Albarracin	Teruel	Aragon	Spain	1250
KC581718		12-L130	x	*Lycaenidae*	*Polyommatus*	*ripartii*	Valdevigas		La Rioja	Spain	860
KC581719		12-L138	x	*Lycaenidae*	*Polyommatus*	*ripartii*	Arnedo		La Rioja	Spain	850
KC581721		12-L128	x	*Lycaenidae*	*Polyommatus*	*ripartii*	Enciso		La Rioja	Spain	815

GenBank accession number	BOLD Process ID	Sample ID	Used in the Iberian dataset	Family	Genus	Species	Exact Site	Region	State/Province	Country	Alt. (m)
KC581722		12-L137	x	*Lycaenidae*	*Polyommatus*	*ripartii*	Peña Isasa		La Rioja	Spain	1080
KC581727		12-Q453	x	*Lycaenidae*	*Polyommatus*	*ripartii*	Hornillos de Cameros		La Rioja	Spain	905
KC581728		12-Q455	x	*Lycaenidae*	*Polyommatus*	*ripartii*	Torre de Cameros		La Rioja	Spain	1150
JX678093		MAT-99-Q947		*Lycaenidae*	*Polyommatus*	*thersites*	Mende		Lozère	France	
HQ563548	FBLMW304-10	BC ZSM Lep 37405		*Lycaenidae*	*Polyommatus*	*thersites*	Koednitzer Weinleite	Oberfranken	Bavaria	Germany	385
HQ005063	EZRMN212-08	RVcoll.08-M370		*Lycaenidae*	*Polyommatus*	*thersites*	Buciumeni forest (Radacinesti)	Bacau	Moldavia	Romania	180
HQ005064	EZRMN214-08	RVcoll.08-M657		*Lycaenidae*	*Polyommatus*	*thersites*	Berca, vulcanii noroiosi Paclele Mici	Buzau	Muntenia	Romania	220
HQ005057	EZROM508-08	RV-07-D204		*Lycaenidae*	*Polyommatus*	*thersites*	Dealul Istrita, Breaza	Buzau	Muntenia	Romania	540
HQ005058	EZROM509-08	RV-07-D224		*Lycaenidae*	*Polyommatus*	*thersites*	Dealul Istrita, Breaza	Buzau	Muntenia	Romania	540
KP870798	EULEP180-14	11-J407		*Lycaenidae*	*Polyommatus*	*thersites*	Breaza, Dealul Istrița	Buzău	Muntenia	Romania	400-720
HQ005061	EZROM505-08	RV-06-M854		*Lycaenidae*	*Polyommatus*	*thersites*	Cluj-Napoca, dealul Feleacului, colonia Borhanci	Cluj	Transylvania	Romania	480
HQ005059	EZROM506-08	RV-06-V687		*Lycaenidae*	*Polyommatus*	*thersites*	Suatu	Cluj	Transylvania	Romania	380
HQ005062	EZRMN211-08	RVcoll.07-D142		*Lycaenidae*	*Polyommatus*	*thersites*	Dumbraveni forest	Constanta	Dobrogea	Romania	80
HQ005060	EZROM507-08	RV-07-D143		*Lycaenidae*	*Polyommatus*	*thersites*	Dumbraveni forest	Constanta	Dobrogea	Romania	80
GU676293	EZSPN863-09	RVcoll.08-L733	x	*Lycaenidae*	*Polyommatus*	*thersites*	Campo Real		Comunidad de Madrid	Spain	729
GU676225	EZSPN936-09	RVcoll.08-L896	x	*Lycaenidae*	*Polyommatus*	*thersites*	San Andres		Cantabria	Spain	976
KP870319	EZSPM630-12	RVcoll.11-E038	x	*Lycaenidae*	*Polyommatus*	*thersites*	Font de l`Arbre, Serra d`Aitana	Alicante	Comunidad Valenciana	Spain	1209
GU676787	EZSPN149-09	RVcoll.08-R052	x	*Lycaenidae*	*Polyommatus*	*thersites*	Barrancada	Alicante	Comunidad Valenciana	Spain	817
KP870885	EZROM752-08	RV-06-G453	x	*Lycaenidae*	*Polyommatus*	*thersites*	Can Rovira, El Brull	Barcelona	Catalonia	Spain	830
GU676401	EZSPN750-09	RVcoll.08-L203	x	*Lycaenidae*	*Polyommatus*	*thersites*	La Vega, Vilafranca	Castellon	Comunidad Valenciana	Spain	1124
GU676073	EZSPM454-09	Rvcoll.09-V908	x	*Lycaenidae*	*Polyommatus*	*thersites*	Sierra de la Sagra	Granada	Andalusia	Spain	1745
KP871175	EZSPM588-12	RVcoll.09-V912	x	*Lycaenidae*	*Polyommatus*	*thersites*	Sierra de la Sagra	Granada	Andalusia	Spain	1745
KP870880	EZSPC286-09	RVcoll.06-A221	x	*Lycaenidae*	*Polyommatus*	*thersites*	Gerri de la Sal, Pallars Sobira	Lleida	Catalonia	Spain	600
KP870404	EZSPC287-09	RVcoll.08-P349	x	*Lycaenidae*	*Polyommatus*	*thersites*	Perves, Pallars Jussa	Lleida	Catalonia	Spain	1071
GU676240	EZSPN917-09	RVcoll.08-L861	x	*Lycaenidae*	*Polyommatus*	*thersites*	Abejar	Soria	Castilla y Leon	Spain	1155
KP870935	EULEP244-14	12-N705	x	*Lycaenidae*	*Polyommatus*	*thersites*	El Valecillo	Teruel	Aragón	Spain	1526.7
GU676428	EZSPN716-09	RVcoll.08-L094	x	*Lycaenidae*	*Polyommatus*	*thersites*	Ruta de la Sima, El Vallecillo	Teruel	Aragon	Spain	1531
HM901759	EZSPC921-10	RVcoll.09-V906	x	*Lycaenidae*	*Polyommatus*	*violetae*	Sierra de la Sagra	Granada	Andalusia	Spain	1745
HM901760	EZSPC922-10	RVcoll.09-V909	x	*Lycaenidae*	*Polyommatus*	*violetae*	Sierra de la Sagra	Granada	Andalusia	Spain	1745
HM901761	EZSPC923-10	RVcoll.09-V910	x	*Lycaenidae*	*Polyommatus*	*violetae*	Sierra de la Sagra	Granada	Andalusia	Spain	1745
KP871068	EZSPC924-10	RVcoll.09-V913	x	*Lycaenidae*	*Polyommatus*	*violetae*	Sierra de la Sagra	Granada	Andalusia	Spain	1745
GU677016	EZSPN199-09	RVcoll.07-C686	x	*Lycaenidae*	*Polyommatus*	*violetae*	Sierra de Almijara	Granada	Andalusia	Spain	1700
GU677017	EZSPN200-09	RVcoll.07-C687	x	*Lycaenidae*	*Polyommatus*	*violetae*	Sierra de Almijara	Granada	Andalusia	Spain	1700
EF104604.2		RV03-H558	x	*Lycaenidae*	*Polyommatus*	*violetae*	Sierra de la Sagra	Granada	Andalusia	Spain	1702
HM210166		RV03-H555	x	*Lycaenidae*	*Polyommatus*	*violetae*	Sierra de la Sagra	Granada	Andalusia	Spain	1775
HM210173		FGT-05-J629	x	*Lycaenidae*	*Polyommatus*	*violetae*	Sierra de Almijara	Granada	Andalusia	Spain	
HM210174		FGT-05-J630	x	*Lycaenidae*	*Polyommatus*	*violetae*	Sierra de Almijara	Granada	Andalusia	Spain	
HM210175		08-H299	x	*Lycaenidae*	*Polyommatus*	*violetae*	Andalucía	Granada	Andalusia	Spain	1750
GU675997	EZSPM023-09	RVcoll.08-P088	x	*Pieridae*	*Pontia*	*callidice*	Pic dels Pessans, Escamps			Andorra	2860
KP870438	EZSPM637-12	RVcoll.11-H030	x	*Pieridae*	*Pontia*	*callidice*	Port de la Bonaigua, Tuc de la Cigalera	Lleida	Catalonia	Spain	2200
KP870892	EZSPM638-12	RVcoll.11-H031	x	*Pieridae*	*Pontia*	*callidice*	Port de la Bonaigua, Tuc de la Cigalera	Lleida	Catalonia	Spain	2200
KP871095	EZSPM639-12	RVcoll.11-H033	x	*Pieridae*	*Pontia*	*callidice*	Port de la Bonaigua, Tuc de la Cigalera	Lleida	Catalonia	Spain	2200
JF847978	EZSPN029-09	RVcoll.09-T056	x	*Pieridae*	*Pontia*	*callidice*	Tuc de la Cigalera, Vall d`Aran	Lleida	Catalonia	Spain	2400
JF847979	EZSPN030-09	RVcoll.09-T061	x	*Pieridae*	*Pontia*	*callidice*	Tuc de la Cigalera, Vall d`Aran	Lleida	Catalonia	Spain	2400
JN276243	EZSPN1069-11	Rvcoll.09-T054	x	*Pieridae*	*Pontia*	*callidice*	Tuc de la Cigalera, Vall d`Aran	Lleida	Catalonia	Spain	2400
GU676974	EZSPN248-09	RVcoll.07-W124	x	*Pieridae*	*Pontia*	*callidice*	Tuc de la Cigalera, Vall d`Aran	Lleida	Catalonia	Spain	2330
KP871118	EZSPM757-12	RVcoll.12-L541	x	*Pieridae*	*Pontia*	*daplidice*	Totana, Sierra Espuna		Murcia	Spain	
GU676641	EZSPN396-09	RVcoll.08-H456	x	*Pieridae*	*Pontia*	*daplidice*	Campo Real		Comunidad de Madrid	Spain	736
GU676366	EZSPN778-09	RVcoll.08-L288	x	*Pieridae*	*Pontia*	*daplidice*	Los Collados - Benimaurell, Vall de Laguart	Alicante	Comunidad Valenciana	Spain	675
GU675698	EZSPM115-09	RVcoll.08-P474	x	*Pieridae*	*Pontia*	*daplidice*	Candeleda	Avila	Castilla y Leon	Spain	450

GenBank accession number	BOLD Process ID	Sample ID	Used in the Iberian dataset	Family	Genus	Species	Exact Site	Region	State/Province	Country	Alt. (m)
KP870590	EZSPM889-12	Rvcoll.12-M607	x	*Pieridae*	*Pontia*	*daplidice*	Medellin	Badajoz	Extremadura	Spain	240
KP870292	EZSPM890-12	Rvcoll.12-M608	x	*Pieridae*	*Pontia*	*daplidice*	Medellin	Badajoz	Extremadura	Spain	240
JN276238	EZSPN1050-11	Rvcoll.08-L442	x	*Pieridae*	*Pontia*	*daplidice*	Balsareny, Bages	Barcelona	Catalonia	Spain	363.7
GU676922	EZSPN520-09	RVcoll.08-H985	x	*Pieridae*	*Pontia*	*daplidice*	Plasencia	Caceres	Extremadura	Spain	400
GU677000	EZSPN217-09	RVcoll.07-F078	x	*Pieridae*	*Pontia*	*daplidice*	10 km S. of Morella	Castellon	Comunidad Valenciana	Spain	900
KP870904	EZSPM913-12	Rvcoll.12-M635	x	*Pieridae*	*Pontia*	*daplidice*	Lagunas de Ruidera, Ossa de Montiel	Ciudad Real	Castilla-La Mancha	Spain	795
KP870698	EZSPM934-12	Rvcoll.12-M668	x	*Pieridae*	*Pontia*	*daplidice*	Rio Jandula, Mestanza	Ciudad Real	Castilla-La Mancha	Spain	407
KP871160	EZSPM978-12	Rvcoll.12-M738	x	*Pieridae*	*Pontia*	*daplidice*	Arroyo de los Molinillos, Viso del Marques	Ciudad Real	Castilla-La Mancha	Spain	742
KP870294	EZSPM603-12	RVcoll.11-D599	x	*Pieridae*	*Pontia*	*daplidice*	Parc Natural Ses Salines	Formentera	Balearic Islands	Spain	1
JN276240	EZSPN1056-11	Rvcoll.08-P079	x	*Pieridae*	*Pontia*	*daplidice*	Estoll, Fontanals de Cerdanya, Cerdanya	Girona	Catalonia	Spain	1100
GU676839	EZSPN095-09	RVcoll.06-A030	x	*Pieridae*	*Pontia*	*daplidice*	Barranco de los Lagartos, Cadiar	Granada	Andalusia	Spain	860
GU676488	EZSPN660-09	RVcoll.08-J959	x	*Pieridae*	*Pontia*	*daplidice*	Acceso Veleta, Monachil	Granada	Andalusia	Spain	2737
GU676799	EZSPN135-09	RVcoll.06-G487	x	*Pieridae*	*Pontia*	*daplidice*	Barranco de Valcuerna, Candasnos	Huesca	Aragon	Spain	210
GU676706	EZSPN327-09	RVcoll.08-H214	x	*Pieridae*	*Pontia*	*daplidice*	Monegros, Candasnos, Barranco de Valcuerna	Huesca	Aragon	Spain	208
GU675866	EZSPM373-09	RVcoll.08-J860	x	*Pieridae*	*Pontia*	*daplidice*	Fresno de la Vega	Leon	Castilla y Leon	Spain	
JN276239	EZSPN1052-11	Rvcoll.08-L476	x	*Pieridae*	*Pontia*	*daplidice*	Ivars de Noguera	Lleida	Catalonia	Spain	373.8
JN276241	EZSPN1057-11	Rvcoll.08-P337	x	*Pieridae*	*Pontia*	*daplidice*	Fontllonga-Montsec	Lleida	Catalonia	Spain	607.9
JN276242	EZSPN1065-11	Rvcoll.08-R105	x	*Pieridae*	*Pontia*	*daplidice*	Salt de Pitx, Vall d`Aran	Lleida	Catalonia	Spain	1570
GU675991	EZSPM033-09	RVcoll.08-P234	x	*Pieridae*	*Pontia*	*daplidice*	Puig d`en Galileu	Mallorca	Balearic Islands	Spain	1071
GU675992	EZSPM050-09	RVcoll.08-P268	x	*Pieridae*	*Pontia*	*daplidice*	S`Albufera	Mallorca	Balearic Islands	Spain	4
KP870717	EZSPM995-12	Rvcoll.12-L881	x	*Pieridae*	*Pontia*	*daplidice*	Sant Bernatell, Mao, Menorca	Menorca	Balearic Islands	Spain	
GU676728	EZSPN308-09	RVcoll.08-H114	x	*Pieridae*	*Pontia*	*daplidice*	Na Vermella, Mao	Menorca	Balearic Islands	Spain	
GU676711	EZSPN324-09	RVcoll.08-H188	x	*Pieridae*	*Pontia*	*daplidice*	Llimpa, Mao	Menorca	Balearic Islands	Spain	
GU675697	EZSPM176-09	RVcoll.08-R041	x	*Pieridae*	*Pontia*	*daplidice*	Pena Judia, Chulilla	Valencia	Comunidad Valenciana	Spain	288
HM393179	GWORA2445-09	BC ZSM Lep 30657		*Pieridae*	*Pontia*	*edusa*	Bergstorf	Niederbayern	Bavaria	Germany	330
HQ005072	EZROM514-08	RV-07-C942		*Pieridae*	*Pontia*	*edusa*	Baile Herculane (Pecinisca)	Caras-Severin	Banat	Romania	220
HQ005068	EZROM510-08	RV-06-M919		*Pieridae*	*Pontia*	*edusa*	Cluj-Napoca, dealul Bogomaia, Fanatele Clujului	Cluj	Transylvania	Romania	480
HQ005069	EZROM511-08	RV-06-V684		*Pieridae*	*Pontia*	*edusa*	Suatu	Cluj	Transylvania	Romania	380
HQ005066	EZRMN216-08	RVcoll.08-M234		*Pieridae*	*Pontia*	*edusa*	2 Km E of Moreni	Dambovita	Muntenia	Romania	290
HQ005070	EZROM512-08	RV-07-D955		*Pieridae*	*Pontia*	*edusa*	Valea Mare	Dambovita	Muntenia	Romania	225
HQ005071	EZROM513-08	RV-07-D970		*Pieridae*	*Pontia*	*edusa*	4 km west of I.L. Caragiale	Dambovita	Muntenia	Romania	270
HQ005073	EZROM594-08	RV-07-C378		*Pieridae*	*Pontia*	*edusa*	rezervatia Valea lui David	Iasi	Moldavia	Romania	100
HQ005067	EZRMN215-08	RVcoll.07-D901		*Pieridae*	*Pontia*	*edusa*	Otasau Valley, Buila-Vanturarita	Valcea	Oltenia	Romania	515
HQ005065	EZRMN217-08	RVcoll.08-M360		*Pieridae*	*Pontia*	*edusa*	Bujoreni monastery (9 Km E of Zorleni)	Vaslui	Moldavia	Romania	240
KP870513	EZSPC1429-10	RVcoll.09-V140	x	*Nymphalidae*	*Pseudochazara*	*hippolyte*	Sierra Maria	Almeria	Andalusia	Spain	1900
HM901541	EZSPC1430-10	RVcoll.09-V141	x	*Nymphalidae*	*Pseudochazara*	*hippolyte*	Sierra Maria	Almeria	Andalusia	Spain	1900
KP871067	EZSPC1431-10	RVcoll.09-V142	x	*Nymphalidae*	*Pseudochazara*	*hippolyte*	Centro Sierra de Gador	Almeria	Andalusia	Spain	2000
KP870243	EZSPC1435-10	RVcoll.09-V146	x	*Nymphalidae*	*Pseudochazara*	*hippolyte*	Los Filabres, Calar Alto	Almeria	Andalusia	Spain	2100
HM901545	EZSPC1443-10	RVcoll.09-V155	x	*Nymphalidae*	*Pseudochazara*	*hippolyte*	Centro Sierra de Gador	Almeria	Andalusia	Spain	2100
KP871026	EZSPC1444-10	RVcoll.09-V156	x	*Nymphalidae*	*Pseudochazara*	*hippolyte*	Centro Sierra de Gador	Almeria	Andalusia	Spain	2100
HM901542	EZSPC1433-10	RVcoll.09-V144	x	*Nymphalidae*	*Pseudochazara*	*hippolyte*	Sierra Guillimona: Norte de Sagra	Granada	Andalusia	Spain	2000
KP871066	EZSPC1440-10	RVcoll.09-V152	x	*Nymphalidae*	*Pseudochazara*	*hippolyte*	Sierra Guillimona, Norte Sierra Sagra	Granada	Andalusia	Spain	2100
GU676107	EZSPM470-09	Rvcoll.09-V432	x	*Nymphalidae*	*Pseudochazara*	*hippolyte*	San Juan (Sierra Nevada)	Granada	Andalusia	Spain	2760
GU676410	EZSPN732-09	RVcoll.08-L140	x	*Nymphalidae*	*Pseudochazara*	*hippolyte*	Laguna Seca, Hueneja	Granada	Andalusia	Spain	2276
GU676411	EZSPN733-09	RVcoll.08-L141	x	*Nymphalidae*	*Pseudochazara*	*hippolyte*	Laguna Seca, Hueneja	Granada	Andalusia	Spain	2276
GU676413	EZSPN735-09	RVcoll.08-L144	x	*Nymphalidae*	*Pseudochazara*	*hippolyte*	Laguna Seca, Hueneja	Granada	Andalusia	Spain	2276
GU676406	EZSPN736-09	RVcoll.08-L145	x	*Nymphalidae*	*Pseudochazara*	*hippolyte*	Laguna Seca, Hueneja	Granada	Andalusia	Spain	2276
GU676354	EZSPN791-09	RVcoll.08-L350	x	*Nymphalidae*	*Pseudochazara*	*hippolyte*	North-East Granada province	Granada	Andalusia	Spain	
KP870991	EZSPM758-12	RVcoll.12-L553	x	*Lycaenidae*	*Pseudophilotes*	*abencerragus*	Porches, Lagoa		Algarve	Portugal	
KP870680	EZSPM759-12	RVcoll.12-L554	x	*Lycaenidae*	*Pseudophilotes*	*abencerragus*	Porches, Lagoa		Algarve	Portugal	
KP870826	EZSPM760-12	RVcoll.12-L555	x	*Lycaenidae*	*Pseudophilotes*	*abencerragus*	Porches, Lagoa		Algarve	Portugal	
HM901570	EZSPC714-10	RVcoll.08-H315	x	*Lycaenidae*	*Pseudophilotes*	*abencerragus*	La Maranosa, San Martin de la Vega		Comunidad de Madrid	Spain	

GenBank accession number	BOLD Process ID	Sample ID	Used in the Iberian dataset	Family	Genus	Species	Exact Site	Region	State/Province	Country	Alt. (m)
HM901571	EZSPC715-10	RVcoll.08-H316	x	*Lycaenidae*	*Pseudophilotes*	*abencerragus*	La Maranosa, San Martin de la Vega		Comunidad de Madrid	Spain	
HM901575	EZSPC720-10	RVcoll.08-H435	x	*Lycaenidae*	*Pseudophilotes*	*abencerragus*	Campo Real		Comunidad de Madrid	Spain	765
HM901576	EZSPC721-10	RVcoll.08-H438.1	x	*Lycaenidae*	*Pseudophilotes*	*abencerragus*	Campo Real		Comunidad de Madrid	Spain	765
GU676655	EZSPN385-09	RVcoll.08-H436	x	*Lycaenidae*	*Pseudophilotes*	*abencerragus*	Campo Real		Comunidad de Madrid	Spain	765
GU676656	EZSPN386-09	RVcoll.08-H437	x	*Lycaenidae*	*Pseudophilotes*	*abencerragus*	Campo Real		Comunidad de Madrid	Spain	765
GU676657	EZSPN388-09	RVcoll.08-H439	x	*Lycaenidae*	*Pseudophilotes*	*abencerragus*	Campo Real		Comunidad de Madrid	Spain	765
HM901586	EZSPC731-10	RVcoll.08-H667.1	x	*Lycaenidae*	*Pseudophilotes*	*abencerragus*	Grazalema, Puerto de los Alamillos	Cadiz	Andalusia	Spain	887
HM401018	EZSPM569-10	RVcoll.09-X003	x	*Lycaenidae*	*Pseudophilotes*	*baton*	Olopte, La Cerdanya	Girona	Catalonia	Spain	1100
HM901721	EZSPC881-10	RVcoll.09-T142	x	*Lycaenidae*	*Pseudophilotes*	*baton*	Cercanias de La Vega de Robledo, Sena de Luna	Leon	Castilla y Leon	Spain	1120
KP871097	EULEP240-14	12-M018	x	*Lycaenidae*	*Pseudophilotes*	*baton*	Bassa d´Arres, Vall d'Aran	Lleida	Catalonia	Spain	1575
HM901617	EZSPC762-10	RVcoll.08-L431.1	x	*Lycaenidae*	*Pseudophilotes*	*baton*	Baltarga, La Cerdanya	Lleida	Catalonia	Spain	1121.3
HM401019	EZSPM570-10	RVcoll.09-X022	x	*Lycaenidae*	*Pseudophilotes*	*baton*	La Farga Vella, La Cerdanya	Lleida	Catalonia	Spain	1100
GU676351	EZSPN800-09	RVcoll.08-L417	x	*Lycaenidae*	*Pseudophilotes*	*baton*	Rius de Cerdanya	Lleida	Catalonia	Spain	1274
GU676352	EZSPN801-09	RVcoll.08-L430	x	*Lycaenidae*	*Pseudophilotes*	*baton*	Baltarga, La Cerdenya	Lleida	Catalonia	Spain	1121
HQ005078	EZRMN218-08	RVcoll.06-K584		*Lycaenidae*	*Pseudophilotes*	*bavius*	Caianu Mic	Cluj	Transylvania	Romania	500
HQ005077	EZRMN219-08	RVcoll.06-K585		*Lycaenidae*	*Pseudophilotes*	*bavius*	Caianu Mic	Cluj	Transylvania	Romania	500
HQ005076	EZRMN220-08	RVcoll.06-K586		*Lycaenidae*	*Pseudophilotes*	*bavius*	Caianu Mic	Cluj	Transylvania	Romania	500
HQ005075	EZRMN221-08	RVcoll.06-K587		*Lycaenidae*	*Pseudophilotes*	*bavius*	Caianu Mic	Cluj	Transylvania	Romania	500
HQ005074	EZRMN222-08	RVcoll.06-K589		*Lycaenidae*	*Pseudophilotes*	*bavius*	Caianu Mic	Cluj	Transylvania	Romania	500
HQ005079	EZROM515-08	RV-06-K583		*Lycaenidae*	*Pseudophilotes*	*bavius*	Caianu Mic	Cluj	Transylvania	Romania	500
HQ005081	EZROM565-08	RV-06-K588		*Lycaenidae*	*Pseudophilotes*	*bavius*	Caianu Mic	Cluj	Transylvania	Romania	500
HQ005080	EZROM516-08	RV-07-D117		*Lycaenidae*	*Pseudophilotes*	*bavius*	Dumbraveni forest	Constanta	Dobrogea	Romania	80
GU676328	EZSPN828-09	RVcoll.08-L643	x	*Lycaenidae*	*Pseudophilotes*	*panoptes*	Hoyocasero, Pinar de Hoyocasero	Avila	Castilla y Leon	Spain	1254
JN280738	EZSPN1042-11	Rvcoll.08-J369	x	*Lycaenidae*	*Pseudophilotes*	*panoptes*	Vallgrassa, Parc Natural del Garraf	Barcelona	Catalonia	Spain	285
GU676808	EZSPN128-09	RVcoll.06-G449	x	*Lycaenidae*	*Pseudophilotes*	*panoptes*	Can Rovira, El Brull, Osona	Barcelona	Catalonia	Spain	830
GU676784	EZSPN156-09	RVcoll.06-H852	x	*Lycaenidae*	*Pseudophilotes*	*panoptes*	Vallforners, Canoves, Valles Oriental	Barcelona	Catalonia	Spain	650
GU676498	EZSPN654-09	RVcoll.08-J798	x	*Lycaenidae*	*Pseudophilotes*	*panoptes*	Acceso Penyagolosa, Vistabella del Maestrazgo	Castellon	Comunidad Valenciana	Spain	1560
HM901548	EZSPC687-10	RVcoll.06-A015	x	*Lycaenidae*	*Pseudophilotes*	*panoptes*	Mecina Bombaron, Alpujarras	Granada	Andalusia	Spain	1150
HM901549	EZSPC689-10	RVcoll.06-A007	x	*Lycaenidae*	*Pseudophilotes*	*panoptes*	Road from Ferreira to Puerto de La Ragua	Granada	Andalusia	Spain	1600
HM901593	EZSPC738-10	RVcoll.08-J209	x	*Lycaenidae*	*Pseudophilotes*	*panoptes*	Barranco del Chorrillo, Sierra de Huetor, Huetor Santillan	Granada	Andalusia	Spain	1350
KP870799	EZSPM612-12	RVcoll.11-D802	x	*Lycaenidae*	*Pseudophilotes*	*panoptes*	La Zubia, Barranco de Corvales	Granada	Andalusia	Spain	970
KP870741	EZSPM616-12	RVcoll.11-D837	x	*Lycaenidae*	*Pseudophilotes*	*panoptes*	Canada Real de la Zubia, Monachil	Granada	Andalusia	Spain	1106
JN280737	EZSPN1029-11	Rvcoll.06-A009.1	x	*Lycaenidae*	*Pseudophilotes*	*panoptes*	Road from Ferreira to Puerto de La Ragua	Granada	Andalusia	Spain	1600
GU676442	EZSPN683-09	RVcoll.08-L007	x	*Lycaenidae*	*Pseudophilotes*	*panoptes*	Carretera al Veleta, Gueejar Sierra	Granada	Andalusia	Spain	1266
GU676460	EZSPN685-09	RVcoll.08-L013	x	*Lycaenidae*	*Pseudophilotes*	*panoptes*	Cercanias El Purche, Gueejar Sierra	Granada	Andalusia	Spain	1494
GU676631	EZSPN410-09	RVcoll.08-H513	x	*Lycaenidae*	*Pseudophilotes*	*panoptes*	Duron, La Alcarria	Guadalajara	Castilla-La Mancha	Spain	725
GU676795	EZSPN140-09	RVcoll.06-G504	x	*Lycaenidae*	*Pseudophilotes*	*panoptes*	Sierra de Alcubierre	Huesca	Aragon	Spain	600
GU676529	EZSPN611-09	RVcoll.08-J335	x	*Lycaenidae*	*Pseudophilotes*	*panoptes*	Barranco de Valcuerna, Candasnos	Huesca	Aragon	Spain	210
GU675916	EZSPM320-09	RVcoll.08-J888	x	*Lycaenidae*	*Pseudophilotes*	*panoptes*	Aviados	Leon	Castilla y Leon	Spain	1100
GU676926	EZSPN524-09	RVcoll.08-H994	x	*Lycaenidae*	*Pseudophilotes*	*panoptes*	Vega de Gordon	Leon	Castilla y Leon	Spain	1050
JN280739	EZSPN1097-11	Rvcoll.10-A206	x	*Lycaenidae*	*Pseudophilotes*	*panoptes*	1Km N. of Vall de Llebrera, La Nogurera	Lleida	Catalonia	Spain	405
GU675787	EZSPM283-09	SMcoll.150308PP69	x	*Lycaenidae*	*Pseudophilotes*	*panoptes*	Alto del Viso, Alpuente	Valencia	Comunidad Valenciana	Spain	1401
GU675788	EZSPM285-09	SMcoll.150308GV24	x	*Lycaenidae*	*Pseudophilotes*	*panoptes*	Laderas sobre el Turia, Domeno	Valencia	Comunidad Valenciana	Spain	443
HQ005082	EZRMN226-08	RVcoll.08-M369		*Lycaenidae*	*Pseudophilotes*	*vicrama*	Buciumeni forest (Radacinesti)	Bacau	Moldavia	Romania	180
HQ005088	EZROM519-08	RV-07-D112		*Lycaenidae*	*Pseudophilotes*	*vicrama*	Nenciulesti	Buzau	Muntenia	Romania	360
HQ005089	EZROM520-08	RV-07-D202		*Lycaenidae*	*Pseudophilotes*	*vicrama*	Dealul Istrita, Breaza	Buzau	Muntenia	Romania	540

GenBank accession number	BOLD Process ID	Sample ID	Used in the Iberian dataset	Family	Genus	Species	Exact Site	Region	State/Province	Country	Alt. (m)
HQ005083	EZRMN225-08	RVcoll.08-M258		*Lycaenidae*	*Pseudophilotes*	*vicrama*	Baile Herculane, Pecinisca	Caras-Severin	Banat	Romania	270
HQ005085	EZRMN223-08	RVcoll.06-V652		*Lycaenidae*	*Pseudophilotes*	*vicrama*	Faget forest, Ratu Penii	Cluj	Transylvania	Romania	680
HQ005086	EZROM517-08	RV-06-K590		*Lycaenidae*	*Pseudophilotes*	*vicrama*	Caianu Mic	Cluj	Transylvania	Romania	500
HQ005090	EZROM521-08	RV-07-C101		*Lycaenidae*	*Pseudophilotes*	*vicrama*	Cluj-Napoca, dealul Feleacului, Gheorgheni forest	Cluj	Transylvania	Romania	600
HQ005084	EZRMN224-08	RVcoll.07-D187		*Lycaenidae*	*Pseudophilotes*	*vicrama*	Sipote	Constanta	Dobrogea	Romania	90
HQ005087	EZROM518-08	RV-07-D054		*Lycaenidae*	*Pseudophilotes*	*vicrama*	Babadag forest	Tulcea	Dobrogea	Romania	115
GU687019	GWORL216-09	BC ZSM Lep 18622		*Hesperiidae*	*Pyrgus*	*alveus*	Postbauer-Heng, Pavelsbach	Oberpfalz	Bavaria	Germany	450
GU687015	GWORL220-09	BC ZSM Lep 18626		*Hesperiidae*	*Pyrgus*	*alveus*	Neumarkt/Opf., Laehr b. Neumarkt/Opf.	Oberpfalz	Bavaria	Germany	455
HQ005091	EZROM323-08	RV-08-A001		*Hesperiidae*	*Pyrgus*	*alveus*	Apuseni Mts., Rimetea (Piatra Secuiului)	Alba	Transylvania	Romania	750
KP870615	EULEP092-14	08-R371		*Hesperiidae*	*Pyrgus*	*alveus*	M-ții Bihorului., Padiș-Valea Izbuc	Bihor	Transylvania	Romania	
HQ005092	EZRMN227-08	RVcoll.08-M584		*Hesperiidae*	*Pyrgus*	*alveus*	Gheorgheni, Valea Belchia	Harghita	Transylvania	Romania	1000
HQ005093	EZRMN228-08	RVcoll.08-M585		*Hesperiidae*	*Pyrgus*	*alveus*	Gheorgheni, Valea Belchia	Harghita	Transylvania	Romania	1000
HQ005094	EZRMN229-08	RVcoll.08-M586		*Hesperiidae*	*Pyrgus*	*alveus*	Gheorgheni, Valea Belchia	Harghita	Transylvania	Romania	1000
HQ005095	EZRMN230-08	RVcoll.08-M587		*Hesperiidae*	*Pyrgus*	*alveus*	Gheorgheni, Valea Belchia	Harghita	Transylvania	Romania	1000
KP870631	EZSPC180-09	RVcoll.07-W089	x	*Hesperiidae*	*Pyrgus*	*alveus*	Laguna de los Veneros-Aramo, Quiros		Asturias	Spain	1300
KP870618	EZSPC181-09	RVcoll.07-W090	x	*Hesperiidae*	*Pyrgus*	*alveus*	Collada Granceno-Bosque de Pelono, Ponga		Asturias	Spain	1200
KP870993	EZSPC115-09	RVcoll.06-V723	x	*Hesperiidae*	*Pyrgus*	*alveus*	Collet de les Bigues, El Brull, Osona	Barcelona	Catalonia	Spain	1195
KP870727	EZSPC116-09	RVcoll.06-V749	x	*Hesperiidae*	*Pyrgus*	*alveus*	Coll de la Bena, Gisclareny, Bergueda	Barcelona	Catalonia	Spain	1150
KP870626	EZSPN017-09	RVcoll.09-T021	x	*Hesperiidae*	*Pyrgus*	*alveus*	Puerto de Castilla	Caceres	Extremadura	Spain	
KP870521	EZSPN018-09	RVcoll.09-T022	x	*Hesperiidae*	*Pyrgus*	*alveus*	Puerto de Castilla	Caceres	Extremadura	Spain	
KP871119	EZSPN019-09	RVcoll.09-T023	x	*Hesperiidae*	*Pyrgus*	*alveus*	Puerto de Castilla	Caceres	Extremadura	Spain	
KP871003	EZSPN020-09	RVcoll.09-T024	x	*Hesperiidae*	*Pyrgus*	*alveus*	Puerto de Castilla	Caceres	Extremadura	Spain	
KP870831	EZSPN021-09	RVcoll.09-T025	x	*Hesperiidae*	*Pyrgus*	*alveus*	Puerto de Castilla	Caceres	Extremadura	Spain	
KP870257	EZSPC042-09	RVcoll.08-L184	x	*Hesperiidae*	*Pyrgus*	*alveus*	Font del Regatxal, Vilafranca	Castellon	Comunidad Valenciana	Spain	1361
KP870864	EZSPC043-09	RVcoll.08-L207	x	*Hesperiidae*	*Pyrgus*	*alveus*	La Vega, Vilafranca	Castellon	Comunidad Valenciana	Spain	1124
GU676047	EZSPM382-09	Rvcoll.09-T108	x	*Hesperiidae*	*Pyrgus*	*alveus*	Serrania de Cuenca	Cuenca	Castilla-La Mancha	Spain	1300
KP871054	EZSPC117-09	RVcoll.07-C048	x	*Hesperiidae*	*Pyrgus*	*alveus*	Puig Neulos, Alt Emporda	Girona	Catalonia	Spain	1110
KP870972	EZSPC118-09	RVcoll.07-C049	x	*Hesperiidae*	*Pyrgus*	*alveus*	Puig Neulos, Alt Emporda	Girona	Catalonia	Spain	1110
JF848009	EZSPN071-09	RVcoll.09-V484	x	*Hesperiidae*	*Pyrgus*	*alveus*	Sierra de la Sagra	Granada	Andalusia	Spain	1750
JF848010	EZSPN072-09	RVcoll.09-V926	x	*Hesperiidae*	*Pyrgus*	*alveus*	Sierra de la Sagra	Granada	Andalusia	Spain	1750
JF848011	EZSPN073-09	RVcoll.09-V927	x	*Hesperiidae*	*Pyrgus*	*alveus*	Sierra de la Sagra	Granada	Andalusia	Spain	1750
KP870331	EZSPC001-09	RVcoll.07-W187	x	*Hesperiidae*	*Pyrgus*	*alveus*	Camino de Wamba, Puerto de San Isidro	Leon	Castilla y Leon	Spain	1700
KP870748	EZSPC003-09	RVcoll.07-W190	x	*Hesperiidae*	*Pyrgus*	*alveus*	Tuc de la Cigalera, Vall d`Aran	Lleida	Catalonia	Spain	2330
KP871046	EZSPC005-09	RVcoll.07-W192	x	*Hesperiidae*	*Pyrgus*	*alveus*	Val de Barrados (Subida Salt del Pitx), Vall d`Aran	Lleida	Catalonia	Spain	1300
KP870267	EZSPC061-09	RVcoll.08-P545	x	*Hesperiidae*	*Pyrgus*	*alveus*	Pas de Perves	Lleida	Catalonia	Spain	1246
KP870397	EZSPC188-09	RVcoll.07-W143	x	*Hesperiidae*	*Pyrgus*	*alveus*	Bassa d`Arres, Vall d`Aran	Lleida	Catalonia	Spain	1568
GU675740	EZSPM177-09	RVcoll.08-R120	x	*Hesperiidae*	*Pyrgus*	*alveus*	Salt de Pitx, Vall d`Aran	Lleida	Catalonia	Spain	1570
KP870634	EZSPC107-09	RVcoll.06-A314	x	*Hesperiidae*	*Pyrgus*	*alveus*	Sierra de Ayllon, Grado del Pico	Segovia	Castilla y Leon	Spain	
HM386892	LEFIA748-10	MM04102		*Hesperiidae*	*Pyrgus*	*andromedae*	Enontekioe		Lapland	Finland	
HQ570386	LEFIF116-10	MM10572		*Hesperiidae*	*Pyrgus*	*andromedae*	Enontekioe	Lapponia enontekiensis		Finland	
JN277845	LEFIJ483-10	MM17108		*Hesperiidae*	*Pyrgus*	*andromedae*	Enontekioe	Lapponia enontekiensis		Finland	
JN277846	LEFIJ484-10	MM17109		*Hesperiidae*	*Pyrgus*	*andromedae*	Enontekioe	Lapponia enontekiensis		Finland	
GU686972	GWORL284-09	BC ZSM Lep 21996		*Hesperiidae*	*Pyrgus*	*andromedae*	Wettersteingebirge, Kreuzeck-Stuibensee	Oberbayern	Bavaria	Germany	1600
KP870903	EULEP152-14	11-D597		*Hesperiidae*	*Pyrgus*	*andromedae*	M-ții Bucegi	Prahova	Muntenia	Romania	2000
HQ005098	EZRMN363-08	RVcoll.08-M730		*Hesperiidae*	*Pyrgus*	*andromedae*	Meridional Carpathians, Bucegi Mts., Vanturis	Prahova	Muntenia	Romania	1800
HQ005096	EZROM696-08	RV-07-F557		*Hesperiidae*	*Pyrgus*	*andromedae*	Bucegi mountains, Cota 2000	Prahova	Muntenia	Romania	2000
KP870712	EZSPC288-09	RVcoll.07-W109	x	*Hesperiidae*	*Pyrgus*	*andromedae*	Val d`Hurno, Vall d`Aran	Lleida	Catalonia	Spain	1720
KP870987	EZSPC289-09	RVcoll.07-W110	x	*Hesperiidae*	*Pyrgus*	*andromedae*	Val d`Hurno, Vall d`Aran	Lleida	Catalonia	Spain	1720

GenBank accession number	BOLD Process ID	Sample ID	Used in the Iberian dataset	Family	Genus	Species	Exact Site	Region	State/Province	Country	Alt. (m)
KP871001	EZSPC290-09	RVcoll.07-W125	x	*Hesperiidae*	*Pyrgus*	*andromedae*	Val d`Hurno, Vall d`Aran	Lleida	Catalonia	Spain	1720
KP870973	EZSPN1091-11	Rvcoll.10-A004	x	*Hesperiidae*	*Pyrgus*	*andromedae*	Tuc de la Cigalera, Vall d`Aran	Lleida	Catalonia	Spain	2330
KP870399	EZSPN1092-11	Rvcoll.10-A005	x	*Hesperiidae*	*Pyrgus*	*andromedae*	Tuc de la Cigalera, Vall d`Aran	Lleida	Catalonia	Spain	2330
KP870538	EZSPN1093-11	Rvcoll.10-A006	x	*Hesperiidae*	*Pyrgus*	*andromedae*	Tuc de la Cigalera, Vall d`Aran	Lleida	Catalonia	Spain	2330
HQ968473	PHLAB308-10	TLMF Lep 01108		*Hesperiidae*	*Pyrgus*	*andromedae*	Tiefencastel S/ Salouf, Got Grond		Graubunden	Switzerland	1650
JF860261	PHLAC706-10	TLMF Lep 02741		*Hesperiidae*	*Pyrgus*	*andromedae*	Riefawald/ Vals		Graubunden	Switzerland	1800
GU687002	GWORL248-09	BC ZSM Lep 18654		*Hesperiidae*	*Pyrgus*	*armoricanus*	Marktbergel, Petersberg b. Marktbergel	Mittelfranken	Bavaria	Germany	470
GU686993	GWORL256-09	BC ZSM Lep 18662		*Hesperiidae*	*Pyrgus*	*armoricanus*	Schweinfurt, Hoellental b. Schweinfurt	Unterfranken	Bavaria	Germany	240
HQ005099	EZROM654-08	RV-07-D247		*Hesperiidae*	*Pyrgus*	*armoricanus*	Dumbrava Vadului, Vad	Brasov	Transylvania	Romania	495
HQ005110	EZSPC131-09	RVcoll.07-D265		*Hesperiidae*	*Pyrgus*	*armoricanus*	Dumbrava Vadului, Vad	Brasov	Transylvania	Romania	495
HQ005114	EZRMN424-09	RVcoll.09-V673		*Hesperiidae*	*Pyrgus*	*armoricanus*	Breaza, dealul Istrita	Buzau	Muntenia	Romania	350
HQ005113	EZRMN426-09	RVcoll.09-V676		*Hesperiidae*	*Pyrgus*	*armoricanus*	Breaza, dealul Istrita	Buzau	Muntenia	Romania	350
HQ005105	EZRMN233-08	RVcoll.08-M256		*Hesperiidae*	*Pyrgus*	*armoricanus*	Baile Herculane, Pecinisca	Caras-Severin	Banat	Romania	270
HQ005108	EZSPC138-09	RVcoll.07-E360		*Hesperiidae*	*Pyrgus*	*armoricanus*	Baile Herculane (Pecinisca)	Caras-Severin	Banat	Romania	250
HQ005107	EZRMN231-08	RVcoll.07-D455		*Hesperiidae*	*Pyrgus*	*armoricanus*	Catcau	Cluj	Transylvania	Romania	255
HQ005102	EZROM523-08	RV-07-C110		*Hesperiidae*	*Pyrgus*	*armoricanus*	Cluj-Napoca, dealul Feleacului, Gheorgheni forest	Cluj	Transylvania	Romania	600
HQ005103	EZROM524-08	RV-07-C111		*Hesperiidae*	*Pyrgus*	*armoricanus*	Cluj-Napoca, dealul Feleacului, Gheorgheni forest	Cluj	Transylvania	Romania	600
HQ005106	EZRMN234-08	RVcoll.08-M419		*Hesperiidae*	*Pyrgus*	*armoricanus*	1 Km NE of Gura Dobrogei reserve	Constanta	Dobrogea	Romania	40
HQ005120	EZRMN413-09	RVcoll.07-D126		*Hesperiidae*	*Pyrgus*	*armoricanus*	Dumbraveni forest	Constanta	Dobrogea	Romania	80
HQ005119	EZRMN414-09	RVcoll.07-D128		*Hesperiidae*	*Pyrgus*	*armoricanus*	Dumbraveni forest	Constanta	Dobrogea	Romania	80
HQ005118	EZRMN415-09	RVcoll.07-D131		*Hesperiidae*	*Pyrgus*	*armoricanus*	Dumbraveni forest	Constanta	Dobrogea	Romania	80
HQ005117	EZRMN416-09	RVcoll.07-D140		*Hesperiidae*	*Pyrgus*	*armoricanus*	Dumbraveni forest	Constanta	Dobrogea	Romania	80
HQ005116	EZRMN417-09	RVcoll.07-D147		*Hesperiidae*	*Pyrgus*	*armoricanus*	Dumbraveni forest	Constanta	Dobrogea	Romania	80
HQ005115	EZRMN418-09	RVcoll.07-D174		*Hesperiidae*	*Pyrgus*	*armoricanus*	Sipote	Constanta	Dobrogea	Romania	90
HQ005101	EZROM522-08	RV-07-D144		*Hesperiidae*	*Pyrgus*	*armoricanus*	Dumbraveni forest	Constanta	Dobrogea	Romania	80
HQ005111	EZSPC126-09	RVcoll.07-D120		*Hesperiidae*	*Pyrgus*	*armoricanus*	Dumbraveni forest	Constanta	Dobrogea	Romania	80
HQ005112	EZSPC127-09	RVcoll.07-D127		*Hesperiidae*	*Pyrgus*	*armoricanus*	Dumbraveni forest	Constanta	Dobrogea	Romania	80
HQ005109	EZSPC130-09	RVcoll.07-D195		*Hesperiidae*	*Pyrgus*	*armoricanus*	Canaraua Fetei (Baneasa)	Constanta	Dobrogea	Romania	15
HQ005100	EZROM567-08	RV-07-D921		*Hesperiidae*	*Pyrgus*	*armoricanus*	Ciupercenii de Oltet	Gorj	Oltenia	Romania	500
HQ005104	EZRMN232-08	RVcoll.07-D534		*Hesperiidae*	*Pyrgus*	*armoricanus*	Rohia	Maramures	Maramures	Romania	360
HQ005121	EZRMN412-09	RVcoll.07-D034		*Hesperiidae*	*Pyrgus*	*armoricanus*	8 Km S of Babadag (Babadag forest)	Tulcea	Dobrogea	Romania	115
GU675674	EZSPC171-09	RVcoll.07-W033	x	*Hesperiidae*	*Pyrgus*	*armoricanus*	Armada, Lena		Asturias	Spain	
KP870529	EZSPC109-09	RVcoll.06-G571	x	*Hesperiidae*	*Pyrgus*	*armoricanus*	La Sala, El Brull, Osona	Barcelona	Catalonia	Spain	800
KP870595	EZSPC147-09	RVcoll.07-F300	x	*Hesperiidae*	*Pyrgus*	*armoricanus*	Viladrau, Osona	Barcelona	Catalonia	Spain	715
KP870819	EZSPC291-09	RVcoll.07-E638	x	*Hesperiidae*	*Pyrgus*	*armoricanus*	UAB campus	Barcelona	Catalonia	Spain	150
HM901360	EZSPC1160-10	RVcoll.09-T030	x	*Hesperiidae*	*Pyrgus*	*armoricanus*	Puerto de Castilla, Gata	Caceres	Extremadura	Spain	1200
HM901361	EZSPC1161-10	RVcoll.09-T031	x	*Hesperiidae*	*Pyrgus*	*armoricanus*	Puerto de Castilla, Gata	Caceres	Extremadura	Spain	1200
GU675907	EZSPM329-09	RVcoll.08-R062	x	*Hesperiidae*	*Pyrgus*	*armoricanus*	La Cambreta	Castellon	Comunidad Valenciana	Spain	1520
KP870848	EZSPC112-09	RVcoll.06-J012	x	*Hesperiidae*	*Pyrgus*	*armoricanus*	P.K. 43 CM-2106, Huelamo	Cuenca	Castilla-La Mancha	Spain	1237
KP870823	EZSPC292-09	RVcoll.06-A103	x	*Hesperiidae*	*Pyrgus*	*armoricanus*	La Corpella, Cantallops, Alt Emporda	Girona	Catalonia	Spain	150
KP871103	EZSPC293-09	RVcoll.08-J387	x	*Hesperiidae*	*Pyrgus*	*armoricanus*	Estanys de la Jonquera, La Jonquera, Alt Emporda	Girona	Catalonia	Spain	180
KP870984	EZSPC106-09	RVcoll.06-A298	x	*Hesperiidae*	*Pyrgus*	*armoricanus*	Sierra de Ayllon, Santo Tome del Puerto	Segovia	Castilla y Leon	Spain	
KP871155	EZSPC108-09	RVcoll.06-G535	x	*Hesperiidae*	*Pyrgus*	*bellieri*	Can Rovira, El Brull, Osona	Barcelona	Catalonia	Spain	830
KP871031	EZSPC139-09	RVcoll.07-F109	x	*Hesperiidae*	*Pyrgus*	*bellieri*	El Brull, Osona	Barcelona	Catalonia	Spain	835
KP870405	EZSPC141-09	RVcoll.07-F113	x	*Hesperiidae*	*Pyrgus*	*bellieri*	El Brull, Osona	Barcelona	Catalonia	Spain	835
KP870884	EZSPC142-09	RVcoll.07-F114	x	*Hesperiidae*	*Pyrgus*	*bellieri*	El Brull, Osona	Barcelona	Catalonia	Spain	835
GU675670	EZSPC143-09	RVcoll.07-F115	x	*Hesperiidae*	*Pyrgus*	*bellieri*	El Brull, Osona	Barcelona	Catalonia	Spain	835
KP871172	EZSPC006-09	RVcoll.07-W205	x	*Hesperiidae*	*Pyrgus*	*bellieri*	Finca Matidero, Laguarta	Huesca	Aragon	Spain	1178
KP870542	EZSPC007-09	RVcoll.07-W206	x	*Hesperiidae*	*Pyrgus*	*bellieri*	Finca Matidero, Laguarta	Huesca	Aragon	Spain	1178
KP870459	EZSPC008-09	RVcoll.07-W207	x	*Hesperiidae*	*Pyrgus*	*bellieri*	Finca Matidero, Laguarta	Huesca	Aragon	Spain	1178
KP870219	EZSPC101-09	RVcoll.06-A257	x	*Hesperiidae*	*Pyrgus*	*bellieri*	Rio Alcanadre, Laguarta	Huesca	Aragon	Spain	1216
KP870670	EZSPC103-09	RVcoll.06-A284	x	*Hesperiidae*	*Pyrgus*	*bellieri*	Rio Alcanadre, Laguarta	Huesca	Aragon	Spain	1216
KP870345	EZSPC104-09	RVcoll.06-A285	x	*Hesperiidae*	*Pyrgus*	*bellieri*	Rio Guarga, Laguarta	Huesca	Aragon	Spain	1140

GenBank accession number	BOLD Process ID	Sample ID	Used in the Iberian dataset	Family	Genus	Species	Exact Site	Region	State/Province	Country	Alt. (m)
KP870472	EZSPC105-09	RVcoll.06-A287	x	*Hesperiidae*	*Pyrgus*	*bellieri*	Rio Guarga, Laguarta	Huesca	Aragon	Spain	1140
KP870669	EZSPC062-09	RVcoll.08-P546	x	*Hesperiidae*	*Pyrgus*	*bellieri*	Pas de Perves	Lleida	Catalonia	Spain	1246
KP870324	EZSPC063-09	RVcoll.08-P547	x	*Hesperiidae*	*Pyrgus*	*bellieri*	Pas de Perves	Lleida	Catalonia	Spain	1246
KP870764	EZSPC047-09	RVcoll.08-M678	x	*Hesperiidae*	*Pyrgus*	*bellieri*	Serra Major, Morera de Montsant, Priorat	Tarragona	Catalonia	Spain	1090
HQ005122	EZROM336-08	RV-08-A014		*Hesperiidae*	*Pyrgus*	*cacaliae*	Fagaras Mts., Balea Lac	Arges	Muntenia	Romania	2100
KP870484	EULEP165-14	11-I596		*Hesperiidae*	*Pyrgus*	*cacaliae*	M-ţii Bucegi, Cota 2000 m	Prahova	Muntenia	Romania	2000
HQ005124	EZRMN235-08	RVcoll.08-M576		*Hesperiidae*	*Pyrgus*	*cacaliae*	Babele (Bucegi Mts.)	Prahova	Muntenia	Romania	2200
HQ005123	EZROM306-08	RV-07-E668		*Hesperiidae*	*Pyrgus*	*cacaliae*	Bucegi Mts., Babele	Prahova	Muntenia	Romania	2150
KP870610	EZSPC294-09	RVcoll.07-W246	x	*Hesperiidae*	*Pyrgus*	*cacaliae*	Tuc de la Cigalera, Vall d`Aran	Lleida	Catalonia	Spain	2330
KP870737	EZSPC295-09	RVcoll.07-W247	x	*Hesperiidae*	*Pyrgus*	*cacaliae*	Tuc de la Cigalera, Vall d`Aran	Lleida	Catalonia	Spain	2330
KP870986	EZSPC296-09	RVcoll.07-W249	x	*Hesperiidae*	*Pyrgus*	*cacaliae*	Tuc de la Cigalera, Vall d`Aran	Lleida	Catalonia	Spain	2330
KP871090	EZSPC297-09	RVcoll.07-W291	x	*Hesperiidae*	*Pyrgus*	*cacaliae*	Col de Vilac, Vall d`Aran	Lleida	Catalonia	Spain	2324
KP870283	EZSPC298-09	RVcoll.07-W292	x	*Hesperiidae*	*Pyrgus*	*cacaliae*	Col de Vilac, Vall d`Aran	Lleida	Catalonia	Spain	2324
KP870531	EZSPM592-12	RVcoll.10-A053	x	*Hesperiidae*	*Pyrgus*	*cacaliae*	Tuc de la Cigalera, Vall d`Aran	Lleida	Catalonia	Spain	2330
KP870460	EZSPM593-12	RVcoll.10-A055	x	*Hesperiidae*	*Pyrgus*	*cacaliae*	Col de Vilac, Vall d`Aran	Lleida	Catalonia	Spain	2324
KP870832	EZSPM594-12	RVcoll.10-A056	x	*Hesperiidae*	*Pyrgus*	*cacaliae*	Col de Vilac, Vall d`Aran	Lleida	Catalonia	Spain	2324
GU676743	EZSPN290-09	RVcoll.07-W248	x	*Hesperiidae*	*Pyrgus*	*cacaliae*	Col de Vilac, Vall d`Aran	Lleida	Catalonia	Spain	2324
HQ563566	FBLMW326-10	BC ZSM Lep 37427		*Hesperiidae*	*Pyrgus*	*carthami*	Eussenheim, Aschfeld	Unterfranken	Bavaria	Germany	225
KP870613	EULEP012-14	06-K671		*Hesperiidae*	*Pyrgus*	*carthami*	Valea Caldă (Cluj-Napoca)	Cluj	Transylvania	Romania	380
HQ005129	EZRMN236-08	RVcoll.06-M912		*Hesperiidae*	*Pyrgus*	*carthami*	Suatu	Cluj	Transylvania	Romania	380
HQ005130	EZRMN237-08	RVcoll.07-D400		*Hesperiidae*	*Pyrgus*	*carthami*	Suatu	Cluj	Transylvania	Romania	380
HQ005131	EZRMN238-08	RVcoll.08-M501		*Hesperiidae*	*Pyrgus*	*carthami*	Badeni	Cluj	Transylvania	Romania	490
HQ005128	EZROM525-08	RV-06-K667		*Hesperiidae*	*Pyrgus*	*carthami*	Cluj-Napoca, dealul Bogomaia, Fanatele Clujului	Cluj	Transylvania	Romania	480
HQ005127	EZROM527-08	RV-07-D422		*Hesperiidae*	*Pyrgus*	*carthami*	Suatu	Cluj	Transylvania	Romania	380
HQ005126	EZROM526-08	RV-07-D390		*Hesperiidae*	*Pyrgus*	*carthami*	Iernut	Mures	Transylvania	Romania	355
HQ005125	EZROM655-08	RV-06-M903		*Hesperiidae*	*Pyrgus*	*carthami*	Stana	Salaj	Transylvania	Romania	500
GU676762	EZSPN270-09	RVcoll.07-W189	x	*Hesperiidae*	*Pyrgus*	*carthami*	La Una-Ruta de Arcenorio, Ponga		Asturias	Spain	1250
GU676986	EZSPN235-09	RVcoll.07-W084	x	*Hesperiidae*	*Pyrgus*	*carthami*	Piscifactoria Rasquilla, San Martin del Pimpollar	Avila	Castilla y Leon	Spain	1270
KP871127	EZSPC301-09	RVcoll.07-E641	x	*Hesperiidae*	*Pyrgus*	*carthami*	Castellar de n`Hug, Bergueda	Barcelona	Catalonia	Spain	1250
GU669638	EZSPC641-09	RVcoll.08-L378	x	*Hesperiidae*	*Pyrgus*	*carthami*	Vallforners, Canoves, Valles Oriental	Barcelona	Catalonia	Spain	650
GU675888	EZSPM350-09	RVcoll.08-R063	x	*Hesperiidae*	*Pyrgus*	*carthami*	La Cambreta	Castellon	Comunidad Valenciana	Spain	1520
GU675882	EZSPM356-09	RVcoll.08-R061	x	*Hesperiidae*	*Pyrgus*	*carthami*	La Cambreta	Castellon	Comunidad Valenciana	Spain	1520
GU675738	EZSPM122-09	RVcoll.08-P611	x	*Hesperiidae*	*Pyrgus*	*carthami*	La Mogorrita	Cuenca	Castilla-La Mancha	Spain	1748
JF283325		09-V526	x	*Hesperiidae*	*Pyrgus*	*carthami*	Loma de San Francisco (Sierra Nevada)	Granada	Andalusia	Spain	2315
GU669614	EZSPC674-09	RVcoll.08-M959	x	*Hesperiidae*	*Pyrgus*	*carthami*	Forcat	Huesca	Aragon	Spain	1035
JF848007	EZSPN064-09	RVcoll.09-V717	x	*Hesperiidae*	*Pyrgus*	*carthami*	Yesero	Huesca	Aragon	Spain	1160
GU676971	EZSPN253-09	RVcoll.07-W152	x	*Hesperiidae*	*Pyrgus*	*carthami*	Castejon de Sos, Valle de Benasque	Huesca	Aragon	Spain	1100
KP870862	EZSPC299-09	RVcoll.08-L433	x	*Hesperiidae*	*Pyrgus*	*carthami*	Baltarga, La Cerdanya	Lleida	Catalonia	Spain	1121
KP870915	EZSPC300-09	RVcoll.08-P342	x	*Hesperiidae*	*Pyrgus*	*carthami*	Pas de Perves	Lleida	Catalonia	Spain	1246
GU676817	EZSPN114-09	RVcoll.06-A278	x	*Hesperiidae*	*Pyrgus*	*carthami*	El Vallecillo, Albarracin	Teruel	Aragon	Spain	
GU675645		07-C539		*Hesperiidae*	*Pyrgus*	*cinarae*	Vatochori		Florina	Greece	
GU675646		07-C540		*Hesperiidae*	*Pyrgus*	*cinarae*	Vatochori		Florina	Greece	
GU675647		07-C541		*Hesperiidae*	*Pyrgus*	*cinarae*	Vatochori		Florina	Greece	
GU675649		07-C538		*Hesperiidae*	*Pyrgus*	*cinarae*	Vatochori		Florina	Greece	
GU675668		LR-08-D411		*Hesperiidae*	*Pyrgus*	*cinarae*	Smolikas Mts, Pades		Ipiros	Greece	1497
KP871076		LR-08-D412.1		*Hesperiidae*	*Pyrgus*	*cinarae*	Smolikas Mts, Pades		Ipiros	Greece	1497
KP870671	EZSPM591-12	RVcoll.10-A037	x	*Hesperiidae*	*Pyrgus*	*cinarae*	Sierra de Avila	Avila	Castilla y Leon	Spain	1400
HM400941		09-X600	x	*Hesperiidae*	*Pyrgus*	*cinarae*	Sierra de Ávila	Ávila	Castilla y León	Spain	1450
GU676052		09-T112	x	*Hesperiidae*	*Pyrgus*	*cinarae*	Serranía de Cuenca	Cuenca	Castilla-La Mancha	Spain	1300
GU676054		09-T117	x	*Hesperiidae*	*Pyrgus*	*cinarae*	Serranía de Cuenca	Cuenca	Castilla-La Mancha	Spain	1300
GU676055		09-T109	x	*Hesperiidae*	*Pyrgus*	*cinarae*	Serranía de Cuenca	Cuenca	Castilla-La Mancha	Spain	1300
GU676056		09-T111	x	*Hesperiidae*	*Pyrgus*	*cinarae*	Serranía de Cuenca	Cuenca	Castilla-La Mancha	Spain	1300
GU676057		09-T116	x	*Hesperiidae*	*Pyrgus*	*cinarae*	Serranía de Cuenca	Cuenca	Castilla-La Mancha	Spain	1300

GenBank accession number	BOLD Process ID	Sample ID	Used in the Iberian dataset	Family	Genus	Species	Exact Site	Region	State/Province	Country	Alt. (m)
GU676058		09-T107	x	*Hesperiidae*	*Pyrgus*	*cinarae*	Serranía de Cuenca	Cuenca	Castilla-La Mancha	Spain	1300
GU676059		09-T110	x	*Hesperiidae*	*Pyrgus*	*cinarae*	Serranía de Cuenca	Cuenca	Castilla-La Mancha	Spain	1300
GU676061		09-T113	x	*Hesperiidae*	*Pyrgus*	*cinarae*	Serranía de Cuenca	Cuenca	Castilla-La Mancha	Spain	1300
JF283331		07-W241	x	*Hesperiidae*	*Pyrgus*	*cinarae*	P.K. 43 CM-2106, Huélamo	Cuenca	Castilla-La Mancha	Spain	1237
JF283332		07-W220	x	*Hesperiidae*	*Pyrgus*	*cinarae*	P.K. 43 CM-2106, Huélamo	Cuenca	Castilla-La Mancha	Spain	1237
JF283343		09-T100	x	*Hesperiidae*	*Pyrgus*	*cinarae*	Huélamo, Río Júcar	Cuenca	Castilla-La Mancha	Spain	1237
HM400942		09-X601		*Hesperiidae*	*Pyrgus*	*cinarae*	Kara-Dag		Feodosia	Ukraine	
HM400943		09-X602		*Hesperiidae*	*Pyrgus*	*cinarae*	Kara-Dag		Feodosia	Ukraine	
HM901835		09-X603		*Hesperiidae*	*Pyrgus*	*cinarae*	Kara-Dag		Feodosia	Ukraine	
KP870296	EZSPC111-09	RVcoll.06-G650	x	*Hesperiidae*	*Pyrgus*	*cirsii*	Font de l`Aulet, El Brull, Osona	Barcelona	Catalonia	Spain	1070
GU677007	EZSPN212-09	RVcoll.07-F067	x	*Hesperiidae*	*Pyrgus*	*cirsii*	10 km S. of Morella	Castellon	Comunidad Valenciana	Spain	900
GU676046	EZSPM388-09	Rvcoll.09-T115	x	*Hesperiidae*	*Pyrgus*	*cirsii*	Serrania de Cuenca	Cuenca	Castilla-La Mancha	Spain	1300
GU676777	EZSPN157-09	RVcoll.06-J002	x	*Hesperiidae*	*Pyrgus*	*cirsii*	P.K. 44,5 CM-2106, Huelamo	Cuenca	Castilla-La Mancha	Spain	1250
GU675731	EZSPM181-09	RVcoll.08-R161	x	*Hesperiidae*	*Pyrgus*	*cirsii*	Llanars, Ripolles	Girona	Catalonia	Spain	1120
GU676998	EZSPN221-09	RVcoll.07-F304	x	*Hesperiidae*	*Pyrgus*	*cirsii*	Viladrau, Osona	Girona	Catalonia	Spain	
GU675732	EZSPM106-09	RVcoll.08-P443	x	*Hesperiidae*	*Pyrgus*	*cirsii*	Villanueva de Alcoron, ctra. CU2101 km 10, Alto Tajo	Guadalajara	Castilla-La Mancha	Spain	1358
GU676339	EZSPN813-09	RVcoll.08-L605	x	*Hesperiidae*	*Pyrgus*	*cirsii*	Laguarta, Rio Alcanadre	Huesca	Aragon	Spain	1216
GU675897	EZSPM339-09	RVcoll.08-R010	x	*Hesperiidae*	*Pyrgus*	*cirsii*	Paraisos Altos, Manzanera	Teruel	Aragon	Spain	1200
GU675898	EZSPM340-09	RVcoll.08-R012	x	*Hesperiidae*	*Pyrgus*	*cirsii*	Paraisos Altos, Manzanera	Teruel	Aragon	Spain	1200
GU675895	EZSPM341-09	RVcoll.08-R018	x	*Hesperiidae*	*Pyrgus*	*cirsii*	Paraisos Altos, Manzanera	Teruel	Aragon	Spain	1200
GU655003	FBLMT877-09	BC ZSM Lep 25437		*Hesperiidae*	*Pyrgus*	*malvae*	Neustadt/Donau, Plattenberg	Niederbayern	Bavaria	Germany	370
HM393210	GWORA2490-09	BC ZSM Lep 30702		*Hesperiidae*	*Pyrgus*	*malvae*	Puchhof	Niederbayern	Bavaria	Germany	320
GU655002	FBLMT879-09	BC ZSM Lep 25439		*Hesperiidae*	*Pyrgus*	*malvae*	Jexhof, Hackenwiesen	Oberbayern	Bavaria	Germany	570
GU707175	FBLMU505-09	BC ZSM Lep 27155		*Hesperiidae*	*Pyrgus*	*malvae*	Traunstein, Pechschnait	Oberbayern	Bavaria	Germany	695
JF415729	GWORR698-10	BC ZSM Lep 32194		*Hesperiidae*	*Pyrgus*	*malvae*	Garching-Hochbrueck	Oberbayern	Bavaria	Germany	487
JF415728	GWORR699-10	BC ZSM Lep 32195		*Hesperiidae*	*Pyrgus*	*malvae*	Geretsried, Babenstuben	Oberbayern	Bavaria	Germany	
HQ563565	FBLMW325-10	BC ZSM Lep 37426		*Hesperiidae*	*Pyrgus*	*malvae*	Rehau, Loewitz	Oberfranken	Bavaria	Germany	545
JF415730	GWOR3938-09	BC ZSM Lep 21222		*Hesperiidae*	*Pyrgus*	*malvae*	Dachau Hebertshausen, Schiessanlage	Upper Bavaria	Bavaria	Germany	475
HQ005133	EZRMN242-08	RVcoll.08-M376		*Hesperiidae*	*Pyrgus*	*malvae*	Buciumeni forest (Radacinesti)	Bacau	Moldavia	Romania	180
HQ005139	EZROM530-08	RV-07-D217		*Hesperiidae*	*Pyrgus*	*malvae*	Dealul Istrita, Breaza	Buzau	Muntenia	Romania	540
HQ005135	EZRMN240-08	RVcoll.08-M244		*Hesperiidae*	*Pyrgus*	*malvae*	Baile Herculane, Pecinisca	Caras-Severin	Banat	Romania	270
HQ005137	EZROM528-08	RV-06-K575		*Hesperiidae*	*Pyrgus*	*malvae*	Badeni	Cluj	Transylvania	Romania	440
HQ005138	EZROM529-08	RV-07-D133		*Hesperiidae*	*Pyrgus*	*malvae*	Dumbraveni forest	Constanta	Dobrogea	Romania	80
HQ005136	EZRMN239-08	RVcoll.08-M229		*Hesperiidae*	*Pyrgus*	*malvae*	2 Km E of Moreni	Dambovita	Muntenia	Romania	290
HQ005134	EZRMN241-08	RVcoll.08-M352		*Hesperiidae*	*Pyrgus*	*malvae*	Valea Belchia (Gheorgheni)	Harghita	Transylvania	Romania	1010
HQ005140	EZROM656-08	RV-07-D522		*Hesperiidae*	*Pyrgus*	*malvae*	Rohia	Maramures	Maramures	Romania	360
HQ005132	EZRMN243-08	RVcoll.08-M414		*Hesperiidae*	*Pyrgus*	*malvae*	3 km E of Slava Rusa (Babadag forest)	Tulcea	Dobrogea	Romania	130
JN277558	EZSPN1063-11	Rvcoll.08-P732	x	*Hesperiidae*	*Pyrgus*	*malvoides*	Puerto de Herrera	Alava	Basque Country	Spain	1077
KP870532	EZSPN1033-11	Rvcoll.06-G457	x	*Hesperiidae*	*Pyrgus*	*malvoides*	El Sola, El Brull, Osona	Barcelona	Catalonia	Spain	800
GU677004	EZSPN213-09	RVcoll.07-F068	x	*Hesperiidae*	*Pyrgus*	*malvoides*	10 km S. of Morella	Castellon	Comunidad Valenciana	Spain	900
GU676779	EZSPN159-09	RVcoll.06-J009	x	*Hesperiidae*	*Pyrgus*	*malvoides*	P.K. 32 CM-2105, Una	Cuenca	Castilla-La Mancha	Spain	
GU676617	EZSPN420-09	RVcoll.08-H567	x	*Hesperiidae*	*Pyrgus*	*malvoides*	Arbeteta	Guadalajara	Castilla-La Mancha	Spain	1158
GU676814	EZSPN118-09	RVcoll.06-A290	x	*Hesperiidae*	*Pyrgus*	*malvoides*	Rio Guarga, Laguarta	Huesca	Aragon	Spain	1140
JN277557	EZSPN1049-11	Rvcoll.08-L423	x	*Hesperiidae*	*Pyrgus*	*malvoides*	Rius de Cerdanya	Lleida	Catalonia	Spain	1273.9
GU676981	EZSPN242-09	RVcoll.07-W113	x	*Hesperiidae*	*Pyrgus*	*malvoides*	Val d`Hurno, Vall d`Aran	Lleida	Catalonia	Spain	1720
GU676816	EZSPN121-09	RVcoll.06-A295	x	*Hesperiidae*	*Pyrgus*	*malvoides*	Sierra de Ayllon, San Esteban de Gormaz	Soria	Castilla y Leon	Spain	
GU676694	EZSPN292-09	RVcoll.07-W264	x	*Hesperiidae*	*Pyrgus*	*onopordi*	Laguna de San Juan, Chinchon		Comunidad de Madrid	Spain	
HM901359	EZSPC1159-10	RVcoll.09-T028	x	*Hesperiidae*	*Pyrgus*	*onopordi*	Puerto de Castilla, Gata	Caceres	Extremadura	Spain	1200
GU677006	EZSPN211-09	RVcoll.07-F066	x	*Hesperiidae*	*Pyrgus*	*onopordi*	10 km S. of Morella	Castellon	Comunidad Valenciana	Spain	900
HM901208	EZSPC1004-10	RVcoll.09-V490	x	*Hesperiidae*	*Pyrgus*	*onopordi*	Sierra de la Sagra	Granada	Andalusia	Spain	1745
HM901407	EZSPC1208-10	RVcoll.09-V496	x	*Hesperiidae*	*Pyrgus*	*onopordi*	Sierra de la Sagra	Granada	Andalusia	Spain	1745
HM901408	EZSPC1209-10	RVcoll.09-V497	x	*Hesperiidae*	*Pyrgus*	*onopordi*	Sierra de la Sagra	Granada	Andalusia	Spain	1745

GenBank accession number	BOLD Process ID	Sample ID	Used in the Iberian dataset	Family	Genus	Species	Exact Site	Region	State/Province	Country	Alt. (m)
HM901421	EZSPC1222-10	RVcoll.09-V546	x	*Hesperiidae*	*Pyrgus*	*onopordi*	Loma de San Francisco (Sierra Nevada)	Granada	Andalusia	Spain	2315
GU676830	EZSPN102-09	RVcoll.06-A043	x	*Hesperiidae*	*Pyrgus*	*onopordi*	Barranco de los Lagartos, Cadiar	Granada	Andalusia	Spain	860
KP871110	EULEP260-14	12-Q551	x	*Hesperiidae*	*Pyrgus*	*onopordi*	15 km S. Benabarre	Huesca	Aragón	Spain	755
KP870675	EULEP259-14	12-Q531	x	*Hesperiidae*	*Pyrgus*	*onopordi*	Cellers, Pantà Terradets, Pallars Jussà	Lleida	Catalonia	Spain	391
GU676989	EZSPN234-09	RVcoll.07-W078	x	*Hesperiidae*	*Pyrgus*	*onopordi*	Sierra de Ayllo, Cerezo de Abajo	Segovia	Castilla y Leon	Spain	1036
HM901228	EZSPC1024-10	RVcoll.08-J020	x	*Hesperiidae*	*Pyrgus*	*onopordi*	Muela de Cortes, Cortes de Pallas	Valencia	Comunidad Valenciana	Spain	800
HM901229	EZSPC1025-10	RVcoll.08-J040	x	*Hesperiidae*	*Pyrgus*	*onopordi*	Cortes de Pallas, Cortes de Pallas	Valencia	Comunidad Valenciana	Spain	400
HM901230	EZSPC1026-10	RVcoll.08-J047	x	*Hesperiidae*	*Pyrgus*	*onopordi*	Utiel, Utiel	Valencia	Comunidad Valenciana	Spain	700
HM901350	EZSPC1150-10	RVcoll.08-P931	x	*Hesperiidae*	*Pyrgus*	*onopordi*	Teresa, El Caroig	Valencia	Comunidad Valenciana	Spain	700
GU676700	EZSPN336-09	RVcoll.08-H268	x	*Hesperiidae*	*Pyrgus*	*onopordi*	Mequinenza	Zaragoza	Aragon	Spain	129
GU677021	EZSPN196-09	RVcoll.07-C660	x	*Hesperiidae*	*Pyrgus*	*serratulae*	Col de Puymorens	Languedoc-Roussillon		France	1825
HM901805	EZSPC970-10	RVcoll.08-P743	x	*Hesperiidae*	*Pyrgus*	*serratulae*	Puerto de Azaceta	Alava	Basque Country	Spain	1036
HM901362	EZSPC1162-10	RVcoll.09-T035	x	*Hesperiidae*	*Pyrgus*	*serratulae*	Garganta Navarenas, Navarredonda de Gredos	Avila	Castilla y Leon	Spain	1200
HM901363	EZSPC1163-10	RVcoll.09-T038	x	*Hesperiidae*	*Pyrgus*	*serratulae*	Garganta Navarenas, Navarredonda de Gredos	Avila	Castilla y Leon	Spain	1200
GU676821	EZSPN110-09	RVcoll.06-A260	x	*Hesperiidae*	*Pyrgus*	*serratulae*	Garganta Navarenas, Sierra de Gredos	Avila	Castilla y Leon	Spain	
HM901264	EZSPC1061-10	RVcoll.08-L083	x	*Hesperiidae*	*Pyrgus*	*serratulae*	Font del Regatxal, Vilafranca	Castellon	Comunidad Valenciana	Spain	1403
HM901381	EZSPC1181-10	RVcoll.09-T154	x	*Hesperiidae*	*Pyrgus*	*serratulae*	Puerto del Cunillo	Cuenca	Castilla-La Mancha	Spain	1620
HM901806	EZSPC971-10	RVcoll.09-T155	x	*Hesperiidae*	*Pyrgus*	*serratulae*	Puerto del Cunillo	Cuenca	Castilla-La Mancha	Spain	1620
GU675737	EZSPM123-09	RVcoll.08-P612	x	*Hesperiidae*	*Pyrgus*	*serratulae*	La Mogorrita	Cuenca	Castilla-La Mancha	Spain	1748
HM901447	EZSPC1252-10	RVcoll.09-V840	x	*Hesperiidae*	*Pyrgus*	*serratulae*	Refugi d`Ulldeter, Ripolles	Girona	Catalonia	Spain	2263.1
HM901638	EZSPC785-10	RVcoll.08-R133	x	*Hesperiidae*	*Pyrgus*	*serratulae*	Port de la Bonaigua, Pallars Sobira	Lleida	Catalonia	Spain	2162.4
HM901643	EZSPC791-10	RVcoll.08-R134	x	*Hesperiidae*	*Pyrgus*	*serratulae*	Port de la Bonaigua, Pallars Sobira	Lleida	Catalonia	Spain	2162.4
GU676962	EZSPN262-09	RVcoll.07-W171	x	*Hesperiidae*	*Pyrgus*	*serratulae*	Barranco Muyeres, Vielha	Lleida	Catalonia	Spain	1600
GU676350	EZSPN799-09	RVcoll.08-L414	x	*Hesperiidae*	*Pyrgus*	*serratulae*	Baltarga, La Cerdenya	Lleida	Catalonia	Spain	1121
HM400985	EZSPM526-10	RVcoll.09-X675	x	*Hesperiidae*	*Pyrgus*	*serratulae*	Candelario	Salamanca	Castilla y Leon	Spain	1350
GU676308	EZSPN847-09	RVcoll.08-L691	x	*Hesperiidae*	*Pyrgus*	*serratulae*	Candelario	Salamanca	Castilla y Leon	Spain	1300
GU676988	EZSPN233-09	RVcoll.07-W074	x	*Hesperiidae*	*Pyrgus*	*serratulae*	Sierra de Ayllon, Montejo de Tiermes	Soria	Castilla y Leon	Spain	1325
GU676819	EZSPN116-09	RVcoll.06-A282	x	*Hesperiidae*	*Pyrgus*	*serratulae*	El Vallecillo, Albarracin	Teruel	Aragon	Spain	
GU676050		09-V372		*Hesperiidae*	*Pyrgus*	*sidae*	E Stara Planina, Sinite kamari N Sliven		Sliven	Bulgaria	400
GU676053		09-V371		*Hesperiidae*	*Pyrgus*	*sidae*	Mt. Pirin. Road between Paril and Nova Lovcha villages		Hadjidimovo	Bulgaria	750
JF283352		07-W052		*Hesperiidae*	*Pyrgus*	*sidae*	Domaine de la chasse de Puits de Rians		Var	France	
JF283353		07-W051		*Hesperiidae*	*Pyrgus*	*sidae*	P.K. 16 R-D23, Pourrieres		Var	France	
JF283354		07-W050		*Hesperiidae*	*Pyrgus*	*sidae*	Domaine de la chasse de Puits de Rians		Var	France	
JF283355		07-W049		*Hesperiidae*	*Pyrgus*	*sidae*	Domaine de la chasse de Puits de Rians		Var	France	
JF283356		07-W037		*Hesperiidae*	*Pyrgus*	*sidae*	Mazaugues, Var, R-D64		Provence	France	
JF283357		07-W036		*Hesperiidae*	*Pyrgus*	*sidae*	Mazaugues, Var, R-D64		Provence	France	
JF283389		09-V221		*Hesperiidae*	*Pyrgus*	*sidae*	Domaine de la chasse de Puits de Rians		Provence	France	450
JF283390		09-V220		*Hesperiidae*	*Pyrgus*	*sidae*	Domaine de la chasse de Puits de Rians		Provence	France	450
JF283391		09-V219		*Hesperiidae*	*Pyrgus*	*sidae*	Domaine de la chasse de Puits de Rians		Provence	France	450
JF283392		09-V218		*Hesperiidae*	*Pyrgus*	*sidae*	Domaine de la chasse de Puits de Rians		Provence	France	450
JF283393		09-V217		*Hesperiidae*	*Pyrgus*	*sidae*	Domaine de la chasse de Puits de Rians		Provence	France	450
GU675641		07-C561		*Hesperiidae*	*Pyrgus*	*sidae*	Kristallopigi, Triklario-Geb.		Florina	Greece	1100
GU675648		07-C514		*Hesperiidae*	*Pyrgus*	*sidae*	Kristallopigi, Triklario-Geb.		Florina	Greece	1100
JF283351		07-W297		*Hesperiidae*	*Pyrgus*	*sidae*	Monodendri		Ipiros	Greece	1250
HM400990		09-T191		*Hesperiidae*	*Pyrgus*	*sidae*	Cineto R.		Rome	Italy	800
JF283350		06-A275		*Hesperiidae*	*Pyrgus*	*sidae*	Colle Ponzone, Bric Berton		Liguria	Italy	700
HQ005147	EZROM657-08	RV-06-N024		*Hesperiidae*	*Pyrgus*	*sidae*	Apuseni Mts., Rimetea (Piatra Secuiului)	Alba	Transylvania	Romania	750
HQ005141	EZRMN246-08	RVcoll.07-D412		*Hesperiidae*	*Pyrgus*	*sidae*	Suatu	Cluj	Transylvania	Romania	380

GenBank accession number	BOLD Process ID	Sample ID	Used in the Iberian dataset	Family	Genus	Species	Exact Site	Region	State/Province	Country	Alt. (m)
HQ005144	EZROM531-08	RV-06-M918		*Hesperiidae*	*Pyrgus*	*sidae*	Cluj-Napoca, dealul Bogomaia, Fanatele Clujului	Cluj	Transylvania	Romania	480
HQ005143	EZRMN244-08	RVcoll.07-D166		*Hesperiidae*	*Pyrgus*	*sidae*	Sipote	Constanta	Dobrogea	Romania	90
HQ005142	EZRMN245-08	RVcoll.07-D189		*Hesperiidae*	*Pyrgus*	*sidae*	Canaraua Fetei (Baneasa)	Constanta	Dobrogea	Romania	15
HQ005145	EZROM532-08	RV-07-D024		*Hesperiidae*	*Pyrgus*	*sidae*	Canaraua Fetei (Baneasa)	Constanta	Dobrogea	Romania	20
HQ005146	EZROM533-08	RV-07-D039		*Hesperiidae*	*Pyrgus*	*sidae*	Babadag forest	Tulcea	Dobrogea	Romania	115
KP870658	EZSPN005-09	RVcoll.07-W092.1	x	*Hesperiidae*	*Pyrgus*	*sidae*	Candelario	Salamanca	Castilla y Leon	Spain	1350
KP870250	EZSPN006-09	RVcoll.07-W101.1	x	*Hesperiidae*	*Pyrgus*	*sidae*	Candelario	Salamanca	Castilla y Leon	Spain	1350
JF283375		07-W102	x	*Hesperiidae*	*Pyrgus*	*sidae*	Candelario	Salamanca	Castilla y León	Spain	1350
JF283376		07-W100	x	*Hesperiidae*	*Pyrgus*	*sidae*	Candelario	Salamanca	Castilla y León	Spain	1350
JF283377		07-W099	x	*Hesperiidae*	*Pyrgus*	*sidae*	Candelario	Salamanca	Castilla y León	Spain	1350
JF283379		07-W091	x	*Hesperiidae*	*Pyrgus*	*sidae*	Candelario	Salamanca	Castilla y León	Spain	1350
JF283382		09-T043	x	*Hesperiidae*	*Pyrgus*	*sidae*	Candelario	Salamanca	Castilla y León	Spain	1300
JF283383		09-T042	x	*Hesperiidae*	*Pyrgus*	*sidae*	Candelario	Salamanca	Castilla y León	Spain	1300
JF283384		09-T041	x	*Hesperiidae*	*Pyrgus*	*sidae*	Candelario	Salamanca	Castilla y León	Spain	1300
JF283385		09-T040	x	*Hesperiidae*	*Pyrgus*	*sidae*	Candelario	Salamanca	Castilla y León	Spain	1300
JF283386		09-T039	x	*Hesperiidae*	*Pyrgus*	*sidae*	Candelario	Salamanca	Castilla y León	Spain	1300
GU687046	GWORL194-09	BC ZSM Lep 18600		*Hesperiidae*	*Pyrgus*	*warrenensis*	Berchtesgadener Alpen, Stahl-Haus/Brett	Oberbayern	Bavaria	Germany	1800
GU687048	GWORL190-09	BC ZSM Lep 18596		*Hesperiidae*	*Pyrgus*	*warrenensis*	Oberstdorf, Gaisalpsee	Schwaben	Bavaria	Germany	1400
GU687045	GWORL193-09	BC ZSM Lep 18599		*Hesperiidae*	*Pyrgus*	*warrenensis*	Oberstdorf, Himmelhorn	Schwaben	Bavaria	Germany	2050
GU676643	EZSPN398-09	RVcoll.08-H463	x	*Nymphalidae*	*Pyronia*	*bathseba*	Campo Real		Comunidad de Madrid	Spain	769
KP870346	EZSPM907-12	Rvcoll.12-M629	x	*Nymphalidae*	*Pyronia*	*bathseba*	Sotuelamos, El Bonillo	Albacete	Castilla-La Mancha	Spain	890
GU675880	EZSPM358-09	RVcoll.08-R050	x	*Nymphalidae*	*Pyronia*	*bathseba*	Barrancada	Alicante	Comunidad Valenciana	Spain	817
GU676696	EZSPN340-09	RVcoll.08-H298	x	*Nymphalidae*	*Pyronia*	*bathseba*	Can Montells, Cardedeu	Barcelona	Catalonia	Spain	220
KP870875	EZSPM1017-12	Rvcoll.12-M766	x	*Nymphalidae*	*Pyronia*	*bathseba*	7 km SE Navalvillar de Ibor	Caceres	Extremadura	Spain	729
KP870425	EZSPM1018-12	Rvcoll.12-M767	x	*Nymphalidae*	*Pyronia*	*bathseba*	7 km SE Navalvillar de Ibor	Caceres	Extremadura	Spain	729
GU676887	EZSPN481-09	RVcoll.08-H918	x	*Nymphalidae*	*Pyronia*	*bathseba*	Hervas, Carretera Hervas-La Garganta	Caceres	Extremadura	Spain	774
GU676606	EZSPN434-09	RVcoll.08-H612	x	*Nymphalidae*	*Pyronia*	*bathseba*	El Gastor	Cadiz	Andalusia	Spain	660
KP870426	EZSPM927-12	Rvcoll.12-M650	x	*Nymphalidae*	*Pyronia*	*bathseba*	Lagunas de Ruidera, Ossa de Montiel	Ciudad Real	Castilla-La Mancha	Spain	795
KP871167	EZSPM941-12	Rvcoll.12-M675	x	*Nymphalidae*	*Pyronia*	*bathseba*	Rio Jandula, Mestanza	Ciudad Real	Castilla-La Mancha	Spain	407
KP870840	EZSPM964-12	Rvcoll.12-M716	x	*Nymphalidae*	*Pyronia*	*bathseba*	Alrededores San Lorenzo de Calatrava	Ciudad Real	Castilla-La Mancha	Spain	749
GU676851	EZSPN083-09	RVcoll.06-A000	x	*Nymphalidae*	*Pyronia*	*bathseba*	Road from Ferreira to Puerto de La Ragua	Granada	Andalusia	Spain	1600
GU676441	EZSPN706-09	RVcoll.08-L055	x	*Nymphalidae*	*Pyronia*	*bathseba*	Sierra Elvira, Albolote	Granada	Andalusia	Spain	819
GU676187	EZSPN975-09	RVcoll.08-M000	x	*Nymphalidae*	*Pyronia*	*bathseba*	La Puerta, La Alcarria	Guadalajara	Castilla-La Mancha	Spain	809
GU676805	EZSPN133-09	RVcoll.06-G482	x	*Nymphalidae*	*Pyronia*	*bathseba*	Barranco de Valcuerna, Candasnos	Huesca	Aragon	Spain	210
GU675923	EZSPM310-09	RVcoll.08-J863	x	*Nymphalidae*	*Pyronia*	*bathseba*	Villaobispo de las Regueras	Leon	Castilla y Leon	Spain	
GU676931	EZSPN527-09	RVcoll.08-H997	x	*Nymphalidae*	*Pyronia*	*bathseba*	Vega de Gordon	Leon	Castilla y Leon	Spain	1050
JN278876	EZSPN1051-11	Rvcoll.08-L462	x	*Nymphalidae*	*Pyronia*	*bathseba*	Ivars de Noguera	Lleida	Catalonia	Spain	373.8
JN278878	EZSPN1058-11	Rvcoll.08-P339	x	*Nymphalidae*	*Pyronia*	*bathseba*	Fontllonga-Montsec	Lleida	Catalonia	Spain	607.9
GU676514	EZSPN632-09	RVcoll.08-J732	x	*Nymphalidae*	*Pyronia*	*bathseba*	Casa de Camineros, Tuejar	Valencia	Comunidad Valenciana	Spain	581
HM901522	EZSPC1388-10	RVcoll.09-X560	x	*Nymphalidae*	*Pyronia*	*cecilia*	Sorauren		Navarra	Spain	530
GU676639	EZSPN406-09	RVcoll.08-H497	x	*Nymphalidae*	*Pyronia*	*cecilia*	San Martin de la Vega		Comunidad de Madrid	Spain	616
HM901280	EZSPC1079-10	RVcoll.08-L663	x	*Nymphalidae*	*Pyronia*	*cecilia*	Candeleda, Cuesta de la Joya	Avila	Castilla y Leon	Spain	415
KP870353	EZSPM887-12	Rvcoll.12-M605	x	*Nymphalidae*	*Pyronia*	*cecilia*	Medellin	Badajoz	Extremadura	Spain	240
KP870447	EZSPM888-12	Rvcoll.12-M606	x	*Nymphalidae*	*Pyronia*	*cecilia*	Medellin	Badajoz	Extremadura	Spain	240
KP870714	EZSPM1013-12	Rvcoll.12-M762	x	*Nymphalidae*	*Pyronia*	*cecilia*	4 Km N to Fresnedoso de Ibor	Caceres	Extremadura	Spain	429
KP870977	EZSPM1039-12	Rvcoll.12-M790	x	*Nymphalidae*	*Pyronia*	*cecilia*	Carrascalejo	Caceres	Extremadura	Spain	636
KP870614	EZSPM929-12	Rvcoll.12-M655	x	*Nymphalidae*	*Pyronia*	*cecilia*	Puerto de Calero, Solana del Pino	Ciudad Real	Castilla-La Mancha	Spain	960
KP870398	EZSPM942-12	Rvcoll.12-M676	x	*Nymphalidae*	*Pyronia*	*cecilia*	Rio Jandula, Mestanza	Ciudad Real	Castilla-La Mancha	Spain	407
KP870959	EZSPM963-12	Rvcoll.12-M714	x	*Nymphalidae*	*Pyronia*	*cecilia*	Alrededores San Lorenzo de Calatrava	Ciudad Real	Castilla-La Mancha	Spain	749
HM901414	EZSPC1215-10	RVcoll.09-V512	x	*Nymphalidae*	*Pyronia*	*cecilia*	Sierra de Alfacar	Granada	Andalusia	Spain	1275
HM901433	EZSPC1237-10	RVcoll.09-V721	x	*Nymphalidae*	*Pyronia*	*cecilia*	Boltana	Huesca	Aragon	Spain	750
GU675926	EZSPM309-09	RVcoll.08-J858	x	*Nymphalidae*	*Pyronia*	*cecilia*	Bercianos del Real Camino	Leon	Castilla y Leon	Spain	

GenBank accession number	BOLD Process ID	Sample ID	Used in the Iberian dataset	Family	Genus	Species	Exact Site	Region	State/Province	Country	Alt. (m)
GU676343	EZSPN805-09	RVcoll.08-L463	x	*Nymphalidae*	*Pyronia*	*cecilia*	Ivars de Noguera	Lleida	Catalonia	Spain	374
GU676028	EZSPM054-09	RVcoll.08-P275	x	*Nymphalidae*	*Pyronia*	*cecilia*	S`Albufera	Mallorca	Balearic Islands	Spain	4
GU676026	EZSPM066-09	RVcoll.08-P299	x	*Nymphalidae*	*Pyronia*	*cecilia*	Genova	Mallorca	Balearic Islands	Spain	123
GU676027	EZSPM069-09	RVcoll.08-P309	x	*Nymphalidae*	*Pyronia*	*cecilia*	Sant Elm, Andratx	Mallorca	Balearic Islands	Spain	31
GU676720	EZSPN316-09	RVcoll.08-H175	x	*Nymphalidae*	*Pyronia*	*cecilia*	Llimpa, Mao	Menorca	Balearic Islands	Spain	
GU676025	EZSPM076-09	RVcoll.08-P325	x	*Nymphalidae*	*Pyronia*	*cecilia*	La Riba, Alt Camp	Tarragona	Catalonia	Spain	3356
HM901232	EZSPC1028-10	RVcoll.08-J077	x	*Nymphalidae*	*Pyronia*	*cecilia*	Mugron, Ayora	Valencia	Comunidad Valenciana	Spain	770
GU676553	EZSPN586-09	RVcoll.08-J148	x	*Nymphalidae*	*Pyronia*	*tithonus*	Ponte Pedrinha, Beca, Boticas		Tras-os-Montes	Portugal	730
HQ005154	EZRMN247-08	RVcoll.07-E349		*Nymphalidae*	*Pyronia*	*tithonus*	Baile Herculane (Pecinisca)	Caras-Severin	Banat	Romania	250
HQ005155	EZRMN248-08	RVcoll.07-E354		*Nymphalidae*	*Pyronia*	*tithonus*	Baile Herculane (Pecinisca)	Caras-Severin	Banat	Romania	250
HQ005152	EZRMN249-08	RVcoll.07-E358		*Nymphalidae*	*Pyronia*	*tithonus*	Baile Herculane (Pecinisca)	Caras-Severin	Banat	Romania	250
HQ005151	EZROM534-08	RV-07-E308		*Nymphalidae*	*Pyronia*	*tithonus*	Baile Herculane (Pecinisca)	Caras-Severin	Banat	Romania	270
HQ005148	EZROM684-08	RV-07-E335		*Nymphalidae*	*Pyronia*	*tithonus*	Baile Herculane (Pecinisca)	Caras-Severin	Banat	Romania	270
HQ005153	EZRMN250-08	RVcoll.07-E392		*Nymphalidae*	*Pyronia*	*tithonus*	Picior de Munte	Dambovita	Muntenia	Romania	260
HQ005150	EZROM535-08	RV-07-E396		*Nymphalidae*	*Pyronia*	*tithonus*	Picior de Munte	Dambovita	Muntenia	Romania	260
HQ005149	EZROM685-08	RV-07-E397		*Nymphalidae*	*Pyronia*	*tithonus*	Picior de Munte	Dambovita	Muntenia	Romania	260
GU676249	EZSPN910-09	RVcoll.08-L846	x	*Nymphalidae*	*Pyronia*	*tithonus*	Pinilla del Valle		Comunidad de Madrid	Spain	1100
GU676315	EZSPN838-09	RVcoll.08-L664	x	*Nymphalidae*	*Pyronia*	*tithonus*	Candeleda, Cuesta de la Joya	Avila	Castilla y Leon	Spain	415
KP870733	EZSPC302-09	RVcoll.08-R156	x	*Nymphalidae*	*Pyronia*	*tithonus*	Montvi de Baix, Moianes	Barcelona	Catalonia	Spain	800
GU676380	EZSPN768-09	RVcoll.08-L272	x	*Nymphalidae*	*Pyronia*	*tithonus*	Mas d`Arago, Vistabella del Maestrazgo	Castellon	Comunidad Valenciana	Spain	1333
GU676875	EZSPN467-09	RVcoll.08-H685	x	*Nymphalidae*	*Pyronia*	*tithonus*	Huelamo, Rio Jucar	Cuenca	Castilla-La Mancha	Spain	1237
KP870881	EZSPC304-09	RVcoll.08-P040	x	*Nymphalidae*	*Pyronia*	*tithonus*	La Valira (near Urus), Cerdanya	Girona	Catalonia	Spain	1300
KP870685	EZSPC306-09	RVcoll.08-P021	x	*Nymphalidae*	*Pyronia*	*tithonus*	Talltendre, Cerdanya	Girona	Catalonia	Spain	1571
GU676110	EZSPM436-09	Rvcoll.09-V949	x	*Nymphalidae*	*Pyronia*	*tithonus*	Collados de la Sagra	Granada	Andalusia	Spain	1475
GU676111	EZSPM437-09	Rvcoll.09-V950	x	*Nymphalidae*	*Pyronia*	*tithonus*	Collados de la Sagra	Granada	Andalusia	Spain	1475
GU676913	EZSPN508-09	RVcoll.08-H966	x	*Nymphalidae*	*Pyronia*	*tithonus*	Laguarta, Rio Alcanadre	Huesca	Aragon	Spain	1216
GU676030	EZSPM084-09	RVcoll.08-P406	x	*Nymphalidae*	*Pyronia*	*tithonus*	Buron	Leon	Castilla y Leon	Spain	1116
GU675854	EZSPM216-09	RVcoll.08-R402	x	*Nymphalidae*	*Pyronia*	*tithonus*	Bonar	Leon	Castilla y Leon	Spain	980
KP871124	EZSPC303-09	RVcoll.08-P369	x	*Nymphalidae*	*Pyronia*	*tithonus*	Perves, Pallars Jussa	Lleida	Catalonia	Spain	1071
KP870248	EZSPC305-09	RVcoll.08-P320	x	*Nymphalidae*	*Pyronia*	*tithonus*	La Riba, Alt Camp	Tarragona	Catalonia	Spain	335
GU707035	FBLMU262-09	BC ZSM Lep 25772		*Lycaenidae*	*Satyrium*	*acaciae*	Kelheim, Ihrlerstein, `Brannt`	Niederbayern	Bavaria	Germany	450
HQ005161	EZRMN260-08	RVcoll.08-M208		*Lycaenidae*	*Satyrium*	*acaciae*	3 Km SW of Lunca Corbului	Arges	Muntenia	Romania	235
HQ005162	EZRMN261-08	RVcoll.08-M378		*Lycaenidae*	*Satyrium*	*acaciae*	Buciumeni forest (Radacinesti)	Bacau	Moldavia	Romania	180
HQ005156	EZROM540-08	RV-07-C972		*Lycaenidae*	*Satyrium*	*acaciae*	Baile Herculane (Pecinisca)	Caras-Severin	Banat	Romania	220
HQ005157	EZROM536-08	RV-06-M877		*Lycaenidae*	*Satyrium*	*acaciae*	Cluj-Napoca, dealul Feleacului, Gheorgheni forest	Cluj	Transylvania	Romania	600
HQ005158	EZROM537-08	RV-06-M913		*Lycaenidae*	*Satyrium*	*acaciae*	Suatu	Cluj	Transylvania	Romania	380
KP871052	EULEP127-14	10-A336		*Lycaenidae*	*Satyrium*	*acaciae*	2 km south Șipote	Constanța	Dobrogea	Romania	70
HQ005166	EZRMN251-08	RVcoll.07-C422		*Lycaenidae*	*Satyrium*	*acaciae*	Valea Mare	Dambovita	Muntenia	Romania	225
HQ005159	EZROM538-08	RV-07-D965		*Lycaenidae*	*Satyrium*	*acaciae*	Valea Mare	Dambovita	Muntenia	Romania	225
HQ005160	EZRMN262-08	RVcoll.08-M384		*Lycaenidae*	*Satyrium*	*acaciae*	Garboavele forest	Galati	Moldavia	Romania	90
HQ005163	EZRMN254-08	RVcoll.07-D928		*Lycaenidae*	*Satyrium*	*acaciae*	Pestera Muierii, Baia de Fier	Gorj	Oltenia	Romania	640
HQ005165	EZRMN253-08	RVcoll.07-D571		*Lycaenidae*	*Satyrium*	*acaciae*	Spini	Hunedoara	Transylvania	Romania	240
HQ005164	EZRMN252-08	RVcoll.07-D371		*Lycaenidae*	*Satyrium*	*acaciae*	Iernut	Mures	Transylvania	Romania	355
GU676165	EZSPN1001-09	RVcoll.08-M086	x	*Lycaenidae*	*Satyrium*	*acaciae*	Oteruelo del Valle		Comunidad de Madrid	Spain	1148
GU676281	EZSPN876-09	RVcoll.08-L771	x	*Lycaenidae*	*Satyrium*	*acaciae*	Puerto de Pozazal		Cantabria	Spain	1030
HM901466	EZSPC1294-10	RVcoll.210207TM50	x	*Lycaenidae*	*Satyrium*	*acaciae*	Pista Banyeres-Biar,Biar	Alicante	Comunidad Valenciana	Spain	938
KP870923	EZSPC307-09	RVcoll.07-C480	x	*Lycaenidae*	*Satyrium*	*acaciae*	El Brull, Osona	Barcelona	Catalonia	Spain	800
KP870364	EZSPC308-09	RVcoll.08-M701	x	*Lycaenidae*	*Satyrium*	*acaciae*	Masia del Plans, Vidra, La Garrotxa	Barcelona	Catalonia	Spain	1200
KP870363	EZSPC309-09	RVcoll.08-R280	x	*Lycaenidae*	*Satyrium*	*acaciae*	Masia del Plans, Vidra, La Garrotxa	Barcelona	Catalonia	Spain	1200
HM901472	EZSPC1300-10	RVcoll.090806MA6	x	*Lycaenidae*	*Satyrium*	*acaciae*	Penyagolosa,Vistabella del Maestrazgo	Castellon	Comunidad Valenciana	Spain	

GenBank accession number	BOLD Process ID	Sample ID	Used in the Iberian dataset	Family	Genus	Species	Exact Site	Region	State/Province	Country	Alt. (m)
HM901490	EZSPC1354-10	RVcoll.09-V888	x	*Lycaenidae*	*Satyrium*	*acaciae*	Banys de Tredos, Vall d`Aran	Lleida	Catalonia	Spain	
KP870929	EZSPC310-09	RVcoll.08-M927	x	*Lycaenidae*	*Satyrium*	*acaciae*	Vilamos, Vall d`Aran	Lleida	Catalonia	Spain	1178
KP870313	EZSPC311-09	RVcoll.08-P356	x	*Lycaenidae*	*Satyrium*	*acaciae*	Pas de Perves	Lleida	Catalonia	Spain	1246
GU676238	EZSPN915-09	RVcoll.08-L858	x	*Lycaenidae*	*Satyrium*	*acaciae*	Abejar	Soria	Castilla y Leon	Spain	1155
GU676232	EZSPN926-09	RVcoll.08-L877	x	*Lycaenidae*	*Satyrium*	*acaciae*	Abejar	Soria	Castilla y Leon	Spain	1111
GU676781	EZSPN152-09	RVcoll.08-R057	x	*Lycaenidae*	*Satyrium*	*acaciae*	El Vallecillo	Teruel	Aragon	Spain	1531
HM901469	EZSPC1297-10	RVcoll.030908SF61	x	*Lycaenidae*	*Satyrium*	*acaciae*	Barranco del Regajo,Castielfabib	Valencia	Comunidad Valenciana	Spain	1250
GU676292	EZSPN862-09	RVcoll.08-L730	x	*Lycaenidae*	*Satyrium*	*esculi*	Campo Real		Comunidad de Madrid	Spain	729
GU676250	EZSPN903-09	RVcoll.08-L822	x	*Lycaenidae*	*Satyrium*	*esculi*	Puerto de la Morcuera, Rascafria		Comunidad de Madrid	Spain	1438
KP870930	EZSPM908-12	Rvcoll.12-M630	x	*Lycaenidae*	*Satyrium*	*esculi*	Sotuelamos, El Bonillo	Albacete	Castilla-La Mancha	Spain	890
KP870486	EZSPM909-12	Rvcoll.12-M631	x	*Lycaenidae*	*Satyrium*	*esculi*	Sotuelamos, El Bonillo	Albacete	Castilla-La Mancha	Spain	890
HM901459	EZSPC1281-10	RVcoll.210907WR75	x	*Lycaenidae*	*Satyrium*	*esculi*	Pista Banyeres-Biar,Biar	Alicante	Comunidad Valenciana	Spain	938
HM901468	EZSPC1296-10	RVcoll.270808DT2	x	*Lycaenidae*	*Satyrium*	*esculi*	Pista Banyeres-Biar,Biar	Alicante	Comunidad Valenciana	Spain	938
HM901277	EZSPC1074-10	RVcoll.08-L454	x	*Lycaenidae*	*Satyrium*	*esculi*	La Llavina, Montseny, Valles Oriental	Barcelona	Catalonia	Spain	970
HM901766	EZSPC929-10	RVcoll.08-R160.1	x	*Lycaenidae*	*Satyrium*	*esculi*	Montvi de Baix, Moianes	Barcelona	Catalonia	Spain	
KP870894	EZSPM1032-12	Rvcoll.12-M782	x	*Lycaenidae*	*Satyrium*	*esculi*	Merendero de los Horcones, Navatrasierra	Caceres	Extremadura	Spain	664
GU676924	EZSPN522-09	RVcoll.08-H989	x	*Lycaenidae*	*Satyrium*	*esculi*	Plasencia	Caceres	Extremadura	Spain	400
KP870806	EZSPM916-12	Rvcoll.12-M638	x	*Lycaenidae*	*Satyrium*	*esculi*	Lagunas de Ruidera, Ossa de Montiel	Ciudad Real	Castilla-La Mancha	Spain	795
KP870827	EZSPM932-12	Rvcoll.12-M664	x	*Lycaenidae*	*Satyrium*	*esculi*	Mina de los Pontones, Mestanza	Ciudad Real	Castilla-La Mancha	Spain	450
KP870392	EZSPM952-12	Rvcoll.12-M702	x	*Lycaenidae*	*Satyrium*	*esculi*	Rio Jandula, Mestanza	Ciudad Real	Castilla-La Mancha	Spain	407
KP870779	EZSPM954-12	Rvcoll.12-M705	x	*Lycaenidae*	*Satyrium*	*esculi*	Alrededores San Lorenzo de Calatrava	Ciudad Real	Castilla-La Mancha	Spain	749
GU675779	EZSPM201-09	RVcoll.08-R295	x	*Lycaenidae*	*Satyrium*	*esculi*	Masia del Plans, Vidra, La Garrotxa	Girona	Catalonia	Spain	1200
HM901494	EZSPC1358-10	RVcoll.09-V940	x	*Lycaenidae*	*Satyrium*	*esculi*	Sierra de la Sagra	Granada	Andalusia	Spain	1745
GU676176	EZSPN988-09	RVcoll.08-M031	x	*Lycaenidae*	*Satyrium*	*esculi*	Gualda, La Alcarria	Guadalajara	Castilla-La Mancha	Spain	730
GU676144	EZSPN1018-09	RVcoll.08-M949	x	*Lycaenidae*	*Satyrium*	*esculi*	Tolva	Huesca	Aragon	Spain	730
GU675875	EZSPM361-09	RVcoll.08-J818	x	*Lycaenidae*	*Satyrium*	*esculi*	Compludo	Leon	Castilla y Leon	Spain	
GU676342	EZSPN804-09	RVcoll.08-L457	x	*Lycaenidae*	*Satyrium*	*esculi*	Ivars de Noguera	Lleida	Catalonia	Spain	374
GU675965	EZSPM072-09	RVcoll.08-P314	x	*Lycaenidae*	*Satyrium*	*esculi*	Farena, Alt Camp	Tarragona	Catalonia	Spain	580
HQ005169	EZRMN267-08	RVcoll.08-M448		*Lycaenidae*	*Satyrium*	*ilicis*	Canaraua Fetei (Baneasa)	Constanta	Dobrogea	Romania	20
HQ005167	EZROM539-08	RV-07-D974		*Lycaenidae*	*Satyrium*	*ilicis*	4 km west of I.L. Caragiale	Dambovita	Muntenia	Romania	270
HQ005170	EZRMN263-08	RVcoll.08-M383		*Lycaenidae*	*Satyrium*	*ilicis*	Garboavele forest	Galati	Moldavia	Romania	90
HQ005171	EZRMN264-08	RVcoll.08-M390		*Lycaenidae*	*Satyrium*	*ilicis*	Garboavele forest	Galati	Moldavia	Romania	90
HQ005168	EZROM541-08	RV-06-M907		*Lycaenidae*	*Satyrium*	*ilicis*	Stana	Salaj	Transylvania	Romania	500
HQ005172	EZRMN265-08	RVcoll.08-M429		*Lycaenidae*	*Satyrium*	*ilicis*	8 Km S of Babadag (Babadag forest)	Tulcea	Dobrogea	Romania	115
HQ005173	EZRMN266-08	RVcoll.08-M431		*Lycaenidae*	*Satyrium*	*ilicis*	8 Km S of Babadag (Babadag forest)	Tulcea	Dobrogea	Romania	115
GU675780	EZSPM229-09	RVcoll.08-R424	x	*Lycaenidae*	*Satyrium*	*ilicis*	Allande, Rio del Oro		Asturias	Spain	290
KP870329	EZSPC223-09	RVcoll.08-L453	x	*Lycaenidae*	*Satyrium*	*ilicis*	La Llavina, Montseny, Valles Oriental	Barcelona	Catalonia	Spain	500
KP870807	EZSPC312-09	RVcoll.08-M700	x	*Lycaenidae*	*Satyrium*	*ilicis*	Masia del Plans, Vidra, La Garrotxa	Barcelona	Catalonia	Spain	1200
KP870600	EZSPC313-09	RVcoll.08-R288	x	*Lycaenidae*	*Satyrium*	*ilicis*	Masia del Plans, Vidra, La Garrotxa	Barcelona	Catalonia	Spain	1200
KP870951	EZSPC316-09	RVcoll.07-C474	x	*Lycaenidae*	*Satyrium*	*ilicis*	El Brull, Osona	Barcelona	Catalonia	Spain	800
GU676207	EZSPN959-09	RVcoll.08-L958	x	*Lycaenidae*	*Satyrium*	*ilicis*	Corralejo	Burgos	Castilla y Leon	Spain	983
GU676135	EZSPN1023-09	RVcoll.08-M955.1	x	*Lycaenidae*	*Satyrium*	*ilicis*	Forcat	Huesca	Aragon	Spain	1039
GU675966	EZSPM090-09	RVcoll.08-P414	x	*Lycaenidae*	*Satyrium*	*ilicis*	Buron	Leon	Castilla y Leon	Spain	1116
KP870488	EZSPC314-09	RVcoll.08-M929	x	*Lycaenidae*	*Satyrium*	*ilicis*	Vilamos, Vall d`Aran	Lleida	Catalonia	Spain	1178
KP870583	EZSPC315-09	RVcoll.08-M930	x	*Lycaenidae*	*Satyrium*	*ilicis*	Vilamos, Vall d`Aran	Lleida	Catalonia	Spain	1178
GU676233	EZSPN927-09	RVcoll.08-L882	x	*Lycaenidae*	*Satyrium*	*ilicis*	Abejar	Soria	Castilla y Leon	Spain	1110
GU707037	FBLMU254-09	BC ZSM Lep 25764		*Lycaenidae*	*Satyrium*	*pruni*	Kallmuenz, Kirchenberg	Oberpfalz	Bavaria	Germany	390
GU707034	FBLMU259-09	BC ZSM Lep 25769		*Lycaenidae*	*Satyrium*	*pruni*	Regenstauf, Kreuth	Oberpfalz	Bavaria	Germany	520
HQ005177	EZRMN268-08	RVcoll.08-M207		*Lycaenidae*	*Satyrium*	*pruni*	3 Km SW of Lunca Corbului	Arges	Muntenia	Romania	235
HQ005176	EZRMN269-08	RVcoll.08-M218		*Lycaenidae*	*Satyrium*	*pruni*	3 Km SW of Lunca Corbului	Arges	Muntenia	Romania	235
HQ005174	EZRMN271-08	RVcoll.08-M364		*Lycaenidae*	*Satyrium*	*pruni*	Buciumeni forest (Radacinesti)	Bacau	Moldavia	Romania	180
HQ005179	EZROM543-08	RV-07-D307		*Lycaenidae*	*Satyrium*	*pruni*	Racos	Brasov	Transylvania	Romania	460

GenBank accession number	BOLD Process ID	Sample ID	Used in the Iberian dataset	Family	Genus	Species	Exact Site	Region	State/Province	Country	Alt. (m)
HQ005180	EZROM544-08	RV-07-C923		*Lycaenidae*	*Satyrium*	*pruni*	Teregova	Caras-Severin	Banat	Romania	385
HQ005181	EZROM686-08	RV-07-D594		*Lycaenidae*	*Satyrium*	*pruni*	Teregova	Caras-Severin	Banat	Romania	385
HQ005175	EZRMN270-08	RVcoll.08-M303		*Lycaenidae*	*Satyrium*	*pruni*	Suatu	Cluj	Transylvania	Romania	400
HQ005178	EZROM542-08	RV-06-M829		*Lycaenidae*	*Satyrium*	*pruni*	Cluj-Napoca, dealul Feleacului, Gheorgheni forest	Cluj	Transylvania	Romania	600
HM901340	EZSPC1140-10	RVcoll.08-P734	x	*Lycaenidae*	*Satyrium*	*pruni*	Puerto de Azaceta	Alava	Basque Country	Spain	1036
HM901341	EZSPC1141-10	RVcoll.08-P735	x	*Lycaenidae*	*Satyrium*	*pruni*	Puerto de Azaceta	Alava	Basque Country	Spain	1036
HM901342	EZSPC1142-10	RVcoll.08-P736	x	*Lycaenidae*	*Satyrium*	*pruni*	Puerto de Azaceta	Alava	Basque Country	Spain	1036
HM901343	EZSPC1143-10	RVcoll.08-P737	x	*Lycaenidae*	*Satyrium*	*pruni*	Puerto de Azaceta	Alava	Basque Country	Spain	1036
KP870225	EULEP212-14	12-L033	x	*Lycaenidae*	*Satyrium*	*pruni*	Puerto de Azaceta, Vitoria	Álava	Basque Country	Spain	960
GU688442	GWORO787-09	BC ZSM Lep 30479		*Lycaenidae*	*Satyrium*	*spini*	Fischerhaeuser Isarauen / MUC		Bavaria	Germany	482
HQ955418	FBLMV667-09	BC ZSM Lep 28647		*Lycaenidae*	*Satyrium*	*spini*	Laaber, Endorfmuehle	Oberpfalz	Bavaria	Germany	420
GU676563	EZSPN584-09	RVcoll.08-J144	x	*Lycaenidae*	*Satyrium*	*spini*	Ponte Pedrinha, Beca, Boticas		Tras-os-Montes	Portugal	730
HQ005185	EZROM308-08	RV-07-E670		*Lycaenidae*	*Satyrium*	*spini*	Rimetea, Piatra Secuiului	Alba	Transylvania	Romania	750
HQ005186	EZROM309-08	RV-07-E671		*Lycaenidae*	*Satyrium*	*spini*	Rimetea, Piatra Secuiului	Alba	Transylvania	Romania	750
HQ005189	EZROM342-08	RV-08-A020		*Lycaenidae*	*Satyrium*	*spini*	Apuseni Mts., Rimetea (Piatra Secuiului)	Alba	Transylvania	Romania	750
HQ005183	EZROM349-08	RV-08-A027		*Lycaenidae*	*Satyrium*	*spini*	Apuseni Mts., Rimetea (Piatra Secuiului)	Alba	Transylvania	Romania	750
HQ005182	EZROM350-08	RV-08-A028		*Lycaenidae*	*Satyrium*	*spini*	Apuseni Mts., Rimetea (Piatra Secuiului)	Alba	Transylvania	Romania	750
HQ005187	EZROM545-08	RV-06-M929		*Lycaenidae*	*Satyrium*	*spini*	Dealul Istrita, Breaza	Buzau	Muntenia	Romania	600
HQ005188	EZROM546-08	RV-06-M946		*Lycaenidae*	*Satyrium*	*spini*	Dealul Istrita, Breaza	Buzau	Muntenia	Romania	600
HQ005184	EZROM324-08	RV-08-A002		*Lycaenidae*	*Satyrium*	*spini*	Cluj-Napoca, Cetatea Fetei	Cluj	Transylvania	Romania	480
GU675687	EZSPM150-09	RVcoll.08-P665	x	*Lycaenidae*	*Satyrium*	*spini*	Gamoniteiru, Sierra de Aramo, Quiros		Asturias	Spain	1712
KP870919	EZSPM860-12	Rvcoll.050111LP84	x	*Lycaenidae*	*Satyrium*	*spini*	Fondos de Vega, Degana		Asturias	Spain	1079
GU676200	EZSPN964-09	RVcoll.08-L974	x	*Lycaenidae*	*Satyrium*	*spini*	Oteruelo, Rascafria		Comunidad de Madrid	Spain	1150
KP870271	EZSPM646-12	RVcoll.11-D629	x	*Lycaenidae*	*Satyrium*	*spini*	La Serrella d`Aitana	Alicante	Comunidad Valenciana	Spain	1446
KP870384	EZSPM856-12	Rvcoll.040611HZ11	x	*Lycaenidae*	*Satyrium*	*spini*	El Montgo _ Nivel Alto, Xavia	Alicante	Comunidad Valenciana	Spain	629
KP870870	EZSPC317-09	RVcoll.08-R159	x	*Lycaenidae*	*Satyrium*	*spini*	Montvi de Baix, Moianes	Barcelona	Catalonia	Spain	800
KP870853	EZSPM953-12	Rvcoll.12-M703	x	*Lycaenidae*	*Satyrium*	*spini*	Rio Jandula, Mestanza	Ciudad Real	Castilla-La Mancha	Spain	407
KP871036	EZSPC320-09	RVcoll.08-P029	x	*Lycaenidae*	*Satyrium*	*spini*	Talltendre, Cerdanya	Girona	Catalonia	Spain	1571
KP870842	EZSPC321-09	RVcoll.08-P035	x	*Lycaenidae*	*Satyrium*	*spini*	La Valira (near Urus), Cerdanya	Girona	Catalonia	Spain	1300
KP871002	EZSPM602-12	RVcoll.11-I540	x	*Lycaenidae*	*Satyrium*	*spini*	SE of Cumbres Verdes (La Zubia)	Granada	Andalusia	Spain	1400
GU676445	EZSPN701-09	RVcoll.08-L044	x	*Lycaenidae*	*Satyrium*	*spini*	Sierra Elvira, Albolote	Granada	Andalusia	Spain	819
GU676440	EZSPN705-09	RVcoll.08-L051	x	*Lycaenidae*	*Satyrium*	*spini*	Sierra Elvira, Albolote	Granada	Andalusia	Spain	819
GU676219	EZSPN944-09	RVcoll.08-L922	x	*Lycaenidae*	*Satyrium*	*spini*	Escamilla	Guadalajara	Castilla-La Mancha	Spain	1040
GU675968	EZSPM002-09	RVcoll.08-M965	x	*Lycaenidae*	*Satyrium*	*spini*	Saganta	Huesca	Aragon	Spain	7675
KP870396	EZSPM867-12	Rvcoll.090111XZ15	x	*Lycaenidae*	*Satyrium*	*spini*	Balneario de Panticosa	Huesca	Aragon	Spain	1642
GU676801	EZSPN137-09	RVcoll.06-G496	x	*Lycaenidae*	*Satyrium*	*spini*	Barranco de Valcuerna, Candasnos	Huesca	Aragon	Spain	210
GU675682	EZSPM158-09	RVcoll.08-P684	x	*Lycaenidae*	*Satyrium*	*spini*	Villadangos de Arbas	Leon	Castilla y Leon	Spain	1311
KP870624	EZSPC318-09	RVcoll.07-F092	x	*Lycaenidae*	*Satyrium*	*spini*	Boixols, Pallars Jussa	Lleida	Catalonia	Spain	1240
GU676239	EZSPN916-09	RVcoll.08-L860	x	*Lycaenidae*	*Satyrium*	*spini*	Abejar	Soria	Castilla y Leon	Spain	1155
KP870275	EZSPC319-09	RVcoll.07-C691	x	*Lycaenidae*	*Satyrium*	*spini*	Serra de la Gritella, Prades	Tarragona	Catalonia	Spain	1100
GU707098	FBLMU441-09	BC ZSM Lep 27091		*Lycaenidae*	*Satyrium*	*w-album*	Gilching, Mischenrieder Wald	Oberbayern	Bavaria	Germany	590
HQ005193	EZRMN256-08	RVcoll.08-M536		*Lycaenidae*	*Satyrium*	*w-album*	Valea Feregarilor - Poiana Musuroaie	Caras-Severin	Banat	Romania	350
HQ005190	EZRMN257-08	RVcoll.08-M537		*Lycaenidae*	*Satyrium*	*w-album*	Valea Feregarilor - Poiana Musuroaie	Caras-Severin	Banat	Romania	350
HQ005191	EZRMN258-08	RVcoll.08-M538		*Lycaenidae*	*Satyrium*	*w-album*	Valea Feregarilor - Poiana Musuroaie	Caras-Severin	Banat	Romania	350
HQ005192	EZRMN259-08	RVcoll.08-M543		*Lycaenidae*	*Satyrium*	*w-album*	Valea Feregarilor - Poiana Musuroaie	Caras-Severin	Banat	Romania	350
KP871173	EULEP083-14	08-P969		*Lycaenidae*	*Satyrium*	*w-album*	Valea Feregarilor - Poiana Mușuroaie	Caraș-Severin	Banat	Romania	230-700
KP870547	EULEP084-14	08-P970		*Lycaenidae*	*Satyrium*	*w-album*	Valea Feregarilor - Poiana Mușuroaie	Caraș-Severin	Banat	Romania	230-700
HQ005194	EZRMN255-08	RVcoll.08-M497		*Lycaenidae*	*Satyrium*	*w-album*	Cota 1400 m (Bucegi Mts.), Sinaia	Prahova	Muntenia	Romania	1385
KP871107	EZSPC322-09	RVcoll.07-C479	x	*Lycaenidae*	*Satyrium*	*w-album*	El Brull, Osona	Barcelona	Catalonia	Spain	800
KP870918	EZSPC323-09	RVcoll.07-C685	x	*Lycaenidae*	*Satyrium*	*w-album*	Bosc de Can Cucurella, Llinars del Valles	Barcelona	Catalonia	Spain	230
GU669642	EZSPC637-09	RVcoll.08-L382	x	*Lycaenidae*	*Satyrium*	*w-album*	Llinars del Valles, Bosc de Can Cucurella	Barcelona	Catalonia	Spain	230
KP871144	EZSPM863-12	Rvcoll.070611MH52	x	*Lycaenidae*	*Satyrium*	*w-album*	El Pueyo de Jaca, Panticosa	Huesca	Aragon	Spain	1779
KP870496	EZSPM865-12	Rvcoll.070611MH66	x	*Lycaenidae*	*Satyrium*	*w-album*	Panticosa	Huesca	Aragon	Spain	1214

GenBank accession number	BOLD Process ID	Sample ID	Used in the Iberian dataset	Family	Genus	Species	Exact Site	Region	State/Province	Country	Alt. (m)
KP871029	EULEP077-14	08-P961	x	*Lycaenidae*	*Satyrium*	*w-album*	Saut deth Pish, Vall d'Aran	Lleida	Catalonia	Spain	1500-1570
KP870359	EULEP078-14	08-P962	x	*Lycaenidae*	*Satyrium*	*w-album*	Saut deth Pish, Vall d'Aran	Lleida	Catalonia	Spain	1500-1570
KP870813	EULEP203-14	12-L015	x	*Lycaenidae*	*Satyrium*	*w-album*	Puerto de Ventaniella, Ponga		Asturias	Spain	
GU676382	EZSPN770-09	RVcoll.08-L274	x	*Nymphalidae*	*Satyrus*	*actaea*	Mas d`Arago, Vistabella del Maestrazgo	Castellon	Comunidad Valenciana	Spain	1333
GU676021	EZSPM017-09	RVcoll.08-P014	x	*Nymphalidae*	*Satyrus*	*actaea*	Torre del Riu, Ribera d`Alp, Cerdanya	Girona	Catalonia	Spain	1180
GU676104	EZSPM421-09	Rvcoll.09-V845	x	*Nymphalidae*	*Satyrus*	*actaea*	Coll de Banyuls, Alt Emporda	Girona	Catalonia	Spain	
GU676105	EZSPM460-09	Rvcoll.09-V933	x	*Nymphalidae*	*Satyrus*	*actaea*	Sierra de la Sagra	Granada	Andalusia	Spain	1745
GU676415	EZSPN729-09	RVcoll.08-L137	x	*Nymphalidae*	*Satyrus*	*actaea*	Laguna Seca, Hueneja	Granada	Andalusia	Spain	2276
GU675721	EZSPM128-09	RVcoll.08-P623	x	*Nymphalidae*	*Satyrus*	*actaea*	Escamilla	Guadalajara	Castilla-La Mancha	Spain	1028
GU676906	EZSPN503-09	RVcoll.08-H958	x	*Nymphalidae*	*Satyrus*	*actaea*	Laguarta, Rio Alcanadre	Huesca	Aragon	Spain	1216
GU676909	EZSPN504-09	RVcoll.08-H959	x	*Nymphalidae*	*Satyrus*	*actaea*	Laguarta, Rio Alcanadre	Huesca	Aragon	Spain	1216
GU677028	EZSPN186-09	RVcoll.07-C088	x	*Nymphalidae*	*Satyrus*	*actaea*	Boixols, Pallars Jussa	Lleida	Catalonia	Spain	1155
KP871145	EZSPM858-12	Rvcoll.040611HZ71	x	*Nymphalidae*	*Satyrus*	*actaea*	Fuentes Calientes	Teruel	Aragon	Spain	1273
GU676951	EZSPN550-09	RVcoll.08-J056	x	*Nymphalidae*	*Satyrus*	*actaea*	Muela de Cortes, Cortes de Pallas	Valencia	Comunidad Valenciana	Spain	
JN278884	EZSPN1079-11	Rvcoll.09-X037	x	*Nymphalidae*	*Satyrus*	*ferula*	Ribera d`Er, La Cerdanya	Girona	Catalonia	Spain	1600
KP870979	EZSPM862-12	Rvcoll.070611MH08	x	*Nymphalidae*	*Satyrus*	*ferula*	El Pueyo de Jaca, Panticosa	Huesca	Aragon	Spain	1779
KP870327	EZSPM864-12	Rvcoll.070611MH61	x	*Nymphalidae*	*Satyrus*	*ferula*	El Pueyo de Jaca, Panticosa	Huesca	Aragon	Spain	1779
GU676022	EZSPM078-09	RVcoll.08-P358	x	*Nymphalidae*	*Satyrus*	*ferula*	Perves, Pallars Jussa	Lleida	Catalonia	Spain	1072
GU675724	EZSPM178-09	RVcoll.08-R124	x	*Nymphalidae*	*Satyrus*	*ferula*	Vilamos, Vall d`Aran	Lleida	Catalonia	Spain	1181
GU676151	EZSPN1013-09	RVcoll.08-M933	x	*Nymphalidae*	*Satyrus*	*ferula*	Vilamos, Vall d`Aran	Lleida	Catalonia	Spain	1178
JN278882	EZSPN1068-11	Rvcoll.08-R263	x	*Nymphalidae*	*Satyrus*	*ferula*	Vilamos, Vall d`Aran	Lleida	Catalonia	Spain	1387.1
GU676975	EZSPN249-09	RVcoll.07-W144	x	*Nymphalidae*	*Satyrus*	*ferula*	Vilamos, Vall d`Aran	Lleida	Catalonia	Spain	1152
GU676972	EZSPN250-09	RVcoll.07-W145	x	*Nymphalidae*	*Satyrus*	*ferula*	Vilamos, Vall d`Aran	Lleida	Catalonia	Spain	1152
HM391781	FBLMT884-09	BC ZSM Lep 25444		*Lycaenidae*	*Scolitantides*	*orion*	Essing, Schulerloch	Niederbayern	Bavaria	Germany	365
HQ005195	EZRMN274-08	RVcoll.08-M508		*Lycaenidae*	*Scolitantides*	*orion*	Apuseni Mts., Rimetea (Piatra Secuiului)	Alba	Transylvania	Romania	850
HQ005196	EZRMN273-08	RVcoll.08-M257		*Lycaenidae*	*Scolitantides*	*orion*	Baile Herculane, Pecinisca	Caras-Severin	Banat	Romania	270
HQ005200	EZROM548-08	RV-07-E346		*Lycaenidae*	*Scolitantides*	*orion*	Baile Herculane (Pecinisca)	Caras-Severin	Banat	Romania	270
HQ005197	EZROM698-08	RV-07-F562		*Lycaenidae*	*Scolitantides*	*orion*	Baile Herculane (Pecinisca)	Caras-Severin	Banat	Romania	270
HQ005201	EZRMN272-08	RVcoll.07-D017		*Lycaenidae*	*Scolitantides*	*orion*	Canaraua Fetei (Baneasa)	Constanta	Dobrogea	Romania	15
HQ005199	EZROM547-08	RV-07-D002		*Lycaenidae*	*Scolitantides*	*orion*	Canaraua Fetei (Baneasa)	Constanta	Dobrogea	Romania	15
HQ005198	EZROM687-08	RV-07-D019		*Lycaenidae*	*Scolitantides*	*orion*	Canaraua Fetei (Baneasa)	Constanta	Dobrogea	Romania	15
KP870981	EULEP020-14	07-D018		*Lycaenidae*	*Scolitantides*	*orion*	Canaraua Fetei (Băneasa)	Constanța	Dobrogea	Romania	15
HM901246	EZSPC1043-10	RVcoll.08-J373	x	*Lycaenidae*	*Scolitantides*	*orion*	Vallgrassa, Parc Natural del Garraf	Barcelona	Catalonia	Spain	285
KP870781	EZSPC324-09	RVcoll.06-H848	x	*Lycaenidae*	*Scolitantides*	*orion*	Panta de Vallforners, Canoves, Valles Oriental	Barcelona	Catalonia	Spain	570
KP870455	EZSPC325-09	RVcoll.07-C619	x	*Lycaenidae*	*Scolitantides*	*orion*	La Llavina, Montseny, Valles Oriental	Barcelona	Catalonia	Spain	500
KP870623	EZSPC326-09	RVcoll.08-J359	x	*Lycaenidae*	*Scolitantides*	*orion*	Vallgrassa, Parc Natural del Garraf	Barcelona	Catalonia	Spain	285
HM901428	EZSPC1232-10	RVcoll.09-V698	x	*Lycaenidae*	*Scolitantides*	*orion*	Meranges, La Cerdanya	Girona	Catalonia	Spain	1744
HM901429	EZSPC1233-10	RVcoll.09-V699	x	*Lycaenidae*	*Scolitantides*	*orion*	Meranges, La Cerdanya	Girona	Catalonia	Spain	1744
HM901388	EZSPC1188-10	RVcoll.09-T227	x	*Lycaenidae*	*Scolitantides*	*orion*	Fonts del Cardener, La Coma, Solsones	Lleida	Catalonia	Spain	1100
KP870233	EZSPC327-09	RVcoll.08-L419	x	*Lycaenidae*	*Scolitantides*	*orion*	Rius de Cerdanya	Lleida	Catalonia	Spain	1273
KP870366	EZSPC328-09	RVcoll.08-L426	x	*Lycaenidae*	*Scolitantides*	*orion*	Rius de Cerdanya	Lleida	Catalonia	Spain	1273
GU676740	EZSPN295-09	RVcoll.08-J662	x	*Lycaenidae*	*Scolitantides*	*orion*	Barranco del Toro	Teruel	Aragon	Spain	1443
HQ005202	EZRMN427-09	RVcoll.09-V680		*Hesperiidae*	*Spialia*	*orbifer*	Breaza, dealul Istrita	Buzau	Muntenia	Romania	350
HQ005203	EZRMN411-09	RVcoll.07-E361		*Hesperiidae*	*Spialia*	*orbifer*	Baile Herculane, Pecinisca	Caras-Severin	Banat	Romania	250
HQ005205	EZRMN278-08	RVcoll.08-M439		*Hesperiidae*	*Spialia*	*orbifer*	Gura Dobrogei reserve	Constanta	Dobrogea	Romania	90
HQ005210	EZROM550-08	RV-07-D138		*Hesperiidae*	*Spialia*	*orbifer*	Dumbraveni forest	Constanta	Dobrogea	Romania	80
HQ005206	EZRMN277-08	RVcoll.08-M387		*Hesperiidae*	*Spialia*	*orbifer*	Garboavele forest	Galati	Moldavia	Romania	90
HQ005204	EZRMN279-08	RVcoll.08-M558		*Hesperiidae*	*Spialia*	*orbifer*	4 Km W of Drobeta Turnu-Severin	Mehedinti	Oltenia	Romania	75
HQ005208	EZRMN275-08	RVcoll.07-D038		*Hesperiidae*	*Spialia*	*orbifer*	8 Km S of Babadag (Babadag forest)	Tulcea	Dobrogea	Romania	115
HQ005207	EZRMN276-08	RVcoll.07-D056		*Hesperiidae*	*Spialia*	*orbifer*	8 Km S of Babadag (Babadag forest)	Tulcea	Dobrogea	Romania	115
HQ005209	EZROM549-08	RV-07-D046		*Hesperiidae*	*Spialia*	*orbifer*	Babadag forest	Tulcea	Dobrogea	Romania	115
HQ005211	EZROM688-08	RV-07-D036		*Hesperiidae*	*Spialia*	*orbifer*	8 Km S of Babadag (Babadag forest)	Tulcea	Dobrogea	Romania	115
HM391839	FBLMU508-09	BC ZSM Lep 27158		*Hesperiidae*	*Spialia*	*sertorius*	Rauschberg, Waicher Maisalm	Oberbayern	Bavaria	Germany	900
HQ563568	FBLMW328-10	BC ZSM Lep 37429		*Hesperiidae*	*Spialia*	*sertorius*	Koednitzer Weinleite	Oberfranken	Bavaria	Germany	385

GenBank accession number	BOLD Process ID	Sample ID	Used in the Iberian dataset	Family	Genus	Species	Exact Site	Region	State/Province	Country	Alt. (m)
HQ563567	FBLMW327-10	BC ZSM Lep 37428		*Hesperiidae*	*Spialia*	*sertorius*	Karlburg, NSG Maeusberg	Unterfranken	Bavaria	Germany	260
KP871100	EULEP239-14	12-L610	x	*Hesperiidae*	*Spialia*	*sertorius*	Rasmalho, Portimao		Algarve	Portugal	
KP870943	EZSPM868-12	Rvcoll.090211SD27	x	*Hesperiidae*	*Spialia*	*sertorius*	Sierra Espuna, Totana		Murcia	Spain	1411
JN277554	EZSPC1259-10	RVcoll.030908SF81	x	*Hesperiidae*	*Spialia*	*sertorius*	Puerto de Biar,Biar	Alicante	Comunidad Valenciana	Spain	814
KP870362	EULEP256-14	12-Q469	x	*Hesperiidae*	*Spialia*	*sertorius*	Aldeavieja, Santa María del Cubillo	Ávila	Castilla y León	Spain	1236
KP870852	EULEP249-14	12-Q418	x	*Hesperiidae*	*Spialia*	*sertorius*	La Molina	Barcelona	Catalonia	Spain	1402
GU677008	EZSPN208-09	RVcoll.07-F059	x	*Hesperiidae*	*Spialia*	*sertorius*	10 km S. of Morella	Castellon	Comunidad Valenciana	Spain	900
KP871025	EULEP011-14	06-J013	x	*Hesperiidae*	*Spialia*	*sertorius*	P.K. 43 CM-2106, Huélamo	Cuenca	Castilla-La Mancha	Spain	1237
GU676780	EZSPN160-09	RVcoll.06-J014	x	*Hesperiidae*	*Spialia*	*sertorius*	P.K. 32 CM-2105, Una	Cuenca	Castilla-La Mancha	Spain	
GU677020	EZSPN194-09	RVcoll.07-C499	x	*Hesperiidae*	*Spialia*	*sertorius*	Meranges	Girona	Catalonia	Spain	1555
KP870301	EULEP070-14	08-L065	x	*Hesperiidae*	*Spialia*	*sertorius*	Venta del Molinillo, Huétor de Santillán	Granada	Andalusia	Spain	1236
KP870454	EULEP105-14	09-V459	x	*Hesperiidae*	*Spialia*	*sertorius*	Loma de Lastonares, Sierra Nevada	Granada	Andalusia	Spain	1900-2200
KP870432	EULEP121-14	10-A097	x	*Hesperiidae*	*Spialia*	*sertorius*	Puerto de la Ragua (Sierra Nevada)	Granada	Andalusia	Spain	
KP870790	EULEP161-14	11-G130	x	*Hesperiidae*	*Spialia*	*sertorius*	Puerto de la Ragua (Sierra Nevada)	Granada	Andalusia	Spain	2090
KP870646	EULEP221-14	12-L073	x	*Hesperiidae*	*Spialia*	*sertorius*	Puerto de la Ragua (Sierra Nevada)	Granada	Andalusia	Spain	2090
GU676128	EZSPM468-09	Rvcoll.09-V519	x	*Hesperiidae*	*Spialia*	*sertorius*	Road to Veleta (Sierra Nevada)	Granada	Andalusia	Spain	2640
GU676619	EZSPN422-09	RVcoll.08-H578	x	*Hesperiidae*	*Spialia*	*sertorius*	Gualda, La Alcarria	Guadalajara	Castilla-La Mancha	Spain	742
GU675989	EZSPM029-09	RVcoll.08-J812	x	*Hesperiidae*	*Spialia*	*sertorius*	Compludo	Leon	Castilla y Leon	Spain	
GU675808	EZSPM273-09	RVcoll.08-R492	x	*Hesperiidae*	*Spialia*	*sertorius*	Folgoso do Caurel, Alto de Visuna	Lugo	Galicia	Spain	1330
KP870693	EULEP006-14	06-A264	x	*Hesperiidae*	*Spialia*	*sertorius*	Sierra de Ayllón, Riaza	Segovia	Castilla y León	Spain	
GU675990	EZSPM074-09	RVcoll.08-P318	x	*Hesperiidae*	*Spialia*	*sertorius*	Farena, Alt Camp	Tarragona	Catalonia	Spain	580
KP870847	EZSPM878-12	Rvcoll.140410KV16	x	*Hesperiidae*	*Spialia*	*sertorius*	Ascenso a Penarroya, Alcala de la Selva	Teruel	Aragon	Spain	2016
KP870502	EZSPM880-12	Rvcoll.150310ZX95	x	*Hesperiidae*	*Spialia*	*sertorius*	Fuente del Santo, Torrijas	Teruel	Aragon	Spain	1330
GU676818	EZSPN115-09	RVcoll.06-A281	x	*Hesperiidae*	*Spialia*	*sertorius*	El Vallecillo, Albarracin	Teruel	Aragon	Spain	
GU676699	EZSPN335-09	RVcoll.08-H263	x	*Hesperiidae*	*Spialia*	*sertorius*	Mequinenza	Zaragoza	Aragon	Spain	129
KP871056	EULEP255-14	12-Q459	x	*Hesperiidae*	*Spialia*	*sertorius*	Canales de la Sierra		La Rioja	Spain	1230
HM901352	EZSPC1152-10	RVcoll.08-R029.1	x	*Lycaenidae*	*Tarucus*	*theophrastus*	Rambla de La Torrecilla, Lorca		Murcia	Spain	379
GU675750	EZSPM167-09	RVcoll.08-R019	x	*Lycaenidae*	*Tarucus*	*theophrastus*	Rambla de La Torrecilla, Lorca		Murcia	Spain	379
GU675751	EZSPM169-09	RVcoll.08-R022	x	*Lycaenidae*	*Tarucus*	*theophrastus*	Rambla de La Torrecilla, Lorca		Murcia	Spain	379
GU675749	EZSPM170-09	RVcoll.08-R027	x	*Lycaenidae*	*Tarucus*	*theophrastus*	Rambla de La Torrecilla, Lorca		Murcia	Spain	379
GU688458	GWORO784-09	BC ZSM Lep 30476		*Lycaenidae*	*Thecla*	*betulae*	Fischerhaeuser Isarauen / MUC		Bavaria	Germany	482
KP870320	EULEP206-14	12-L019	x	*Lycaenidae*	*Thecla*	*betulae*	Serra da Malcata, Guarda		Guarda	Portugal	925
KP870503	EULEP131-14	10-B440		*Lycaenidae*	*Thecla*	*betulae*	Răscruci	Cluj	Transylvania	Romania	
HQ005212	EZRMN280-08	RVcoll.08-M651		*Lycaenidae*	*Thecla*	*betulae*	Badeni	Cluj	Transylvania	Romania	490
HQ005213	EZRMN281-08	RVcoll.08-M660		*Lycaenidae*	*Thecla*	*betulae*	Cluj-Napoca, Salicea (Faget forest)	Cluj	Transylvania	Romania	720
HQ005217	EZRMN308-08	RVcoll.08-M792		*Lycaenidae*	*Thecla*	*betulae*	Gheorgheni (Cluj-Napoca)	Cluj	Transylvania	Romania	600
HQ005216	EZRMN309-08	RVcoll.08-M793		*Lycaenidae*	*Thecla*	*betulae*	Gheorgheni (Cluj-Napoca)	Cluj	Transylvania	Romania	600
HQ005215	EZRMN344-08	RVcoll.08-H041		*Lycaenidae*	*Thecla*	*betulae*	Cluj-Napoca, dealul Feleacului, Gheorgheni forest	Cluj	Transylvania	Romania	600
HQ005214	EZRMN345-08	RVcoll.08-H042		*Lycaenidae*	*Thecla*	*betulae*	Cluj-Napoca, dealul Feleacului, Gheorgheni forest	Cluj	Transylvania	Romania	600
HQ005218	EZROM552-08	RV-06-V715		*Lycaenidae*	*Thecla*	*betulae*	Badeni	Cluj	Transylvania	Romania	440
FJ664047	LOWA780-06	2005-LOWA-780		*Lycaenidae*	*Thecla*	*betulae*	Osyno	Sebezh distr.	Pskov	Russia	150
HM901410	EZSPC1211-10	RVcoll.09-V501	x	*Lycaenidae*	*Thecla*	*betulae*	Viladrau, Montseny, Osona	Barcelona	Catalonia	Spain	715
KP871082	EZSPC329-09	RVcoll.06-V724	x	*Lycaenidae*	*Thecla*	*betulae*	Coll Formic, El Brull, Osona	Barcelona	Catalonia	Spain	1170
KP871035	EZSPC330-09	RVcoll.08-L512	x	*Lycaenidae*	*Thecla*	*betulae*	El Cafe, pla de la Calma, El Brull, Osona	Barcelona	Catalonia	Spain	1192
KP871139	EZSPC331-09	RVcoll.08-P081	x	*Lycaenidae*	*Thecla*	*betulae*	Estoll, Fontanals de Cerdanya, Cerdanya	Girona	Catalonia	Spain	1085
KP870732	EULEP213-14	12-L034	x	*Lycaenidae*	*Thecla*	*betulae*	Cervera de Pisuerga	Palencia	Castilla y León	Spain	1065
KP870601	EULEP211-14	12-L030	x	*Lycaenidae*	*Thecla*	*betulae*	Villasrubias, Sierra de Gata	Salamanca	Castilla y León	Spain	850
KP870561	EULEP057-14	08-J487	x	*Lycaenidae*	*Thecla*	*betulae*	Tornin, Cangas de Onís		Asturias	Spain	100
KP870422	EULEP058-14	08-J488	x	*Lycaenidae*	*Thecla*	*betulae*	Tornin, Cangas de Onís		Asturias	Spain	100
HQ563560	FBLMW320-10	BC ZSM Lep 37421		*Hesperiidae*	*Thymelicus*	*acteon*	Koednitzer Weinleite	Oberfranken	Bavaria	Germany	385
HM391841	FBLMU510-09	BC ZSM Lep 27160		*Hesperiidae*	*Thymelicus*	*acteon*	Duggendorf, Girnitztal	Oberpfalz	Bavaria	Germany	375
GU676565	EZSPN574-09	RVcoll.08-J121	x	*Hesperiidae*	*Thymelicus*	*acteon*	Leiranco, Bobadela, Boticas		Tras-os-Montes	Portugal	1150
HQ005220	EZRMN283-08	RVcoll.08-M561		*Hesperiidae*	*Thymelicus*	*acteon*	Breaza, dealul Istrita	Buzau	Muntenia	Romania	500

GenBank accession number	BOLD Process ID	Sample ID	Used in the Iberian dataset	Family	Genus	Species	Exact Site	Region	State/Province	Country	Alt. (m)
HQ005221	EZROM295-08	RV-07-E657		*Hesperiidae*	*Thymelicus*	*acteon*	Dealul Istrita, Breaza	Buzau	Muntenia	Romania	525
KP871151	EULEP075-14	08-M562.1		*Hesperiidae*	*Thymelicus*	*acteon*	Breaza, Dealul Istrița	Buzău	Muntenia	Romania	340-650
KP870956	EULEP076-14	08-M569.1		*Hesperiidae*	*Thymelicus*	*acteon*	Breaza, Dealul Istrița	Buzău	Muntenia	Romania	340-650
KP870326	EULEP080-14	08-P966		*Hesperiidae*	*Thymelicus*	*acteon*	Breaza, Dealul Istrița	Buzău	Muntenia	Romania	340-650
KP870665	EULEP081-14	08-P967		*Hesperiidae*	*Thymelicus*	*acteon*	Breaza, Dealul Istrița	Buzău	Muntenia	Romania	340-650
KP870218	EULEP082-14	08-P968		*Hesperiidae*	*Thymelicus*	*acteon*	Breaza, Dealul Istrița	Buzău	Muntenia	Romania	340-650
HQ005219	EZRMN282-08	RVcoll.08-M549		*Hesperiidae*	*Thymelicus*	*acteon*	Valea Feregarilor - Poiana Musuroaie	Caras-Severin	Banat	Romania	350
GU676959	EZSPN267-09	RVcoll.07-W181	x	*Hesperiidae*	*Thymelicus*	*acteon*	La Mortera, Ribera de Arriba		Asturias	Spain	416
GU676183	EZSPN983-09	RVcoll.08-M023	x	*Hesperiidae*	*Thymelicus*	*acteon*	Alicante	Alicante	Comunidad Valenciana	Spain	11
KP870620	EZSPC332-09	RVcoll.07-C616	x	*Hesperiidae*	*Thymelicus*	*acteon*	La Llavina, Montseny, Valles Oriental	Barcelona	Catalonia	Spain	500
KP870471	EZSPM1023-12	Rvcoll.12-M773	x	*Hesperiidae*	*Thymelicus*	*acteon*	7 km SE Navalvillar de Ibor	Caceres	Extremadura	Spain	729
KP870809	EZSPM1029-12	Rvcoll.12-M779	x	*Hesperiidae*	*Thymelicus*	*acteon*	Merendero de los Horcones, Navatrasierra	Caceres	Extremadura	Spain	664
KP870643	EZSPM923-12	Rvcoll.12-M645	x	*Hesperiidae*	*Thymelicus*	*acteon*	Lagunas de Ruidera, Ossa de Montiel	Ciudad Real	Castilla-La Mancha	Spain	795
KP870512	EZSPM950-12	Rvcoll.12-M691	x	*Hesperiidae*	*Thymelicus*	*acteon*	Rio Jandula, Mestanza	Ciudad Real	Castilla-La Mancha	Spain	407
KP870757	EZSPM962-12	Rvcoll.12-M713	x	*Hesperiidae*	*Thymelicus*	*acteon*	Alrededores San Lorenzo de Calatrava	Ciudad Real	Castilla-La Mancha	Spain	749
KP870451	EZSPM589-12	RVcoll.09-V937	x	*Hesperiidae*	*Thymelicus*	*acteon*	Sierra de la Sagra	Granada	Andalusia	Spain	1745
KP870227	EZSPM648-12	RVcoll.11-I432	x	*Hesperiidae*	*Thymelicus*	*acteon*	3 km S Gueejar Sierra (road to Veleta, Sierra Nevada)	Granada	Andalusia	Spain	1565
GU676841	EZSPN096-09	RVcoll.06-A031	x	*Hesperiidae*	*Thymelicus*	*acteon*	Barranco de los Lagartos, Cadiar	Granada	Andalusia	Spain	860
GU676815	EZSPN120-09	RVcoll.06-A293	x	*Hesperiidae*	*Thymelicus*	*acteon*	Finca Matidero, Laguarta	Huesca	Aragon	Spain	1178
GU675861	EZSPM376-09	RVcoll.08-J865	x	*Hesperiidae*	*Thymelicus*	*acteon*	Columbrianos	Leon	Castilla y Leon	Spain	
KP870906	EZSPC333-09	RVcoll.08-P332	x	*Hesperiidae*	*Thymelicus*	*acteon*	Perves, Pallars Jussa	Lleida	Catalonia	Spain	1071
KP871024	EZSPC334-09	RVcoll.08-P364	x	*Hesperiidae*	*Thymelicus*	*acteon*	Pas de Perves	Lleida	Catalonia	Spain	1246
KP870970	EZSPM622-12	RVcoll.11-D897	x	*Hesperiidae*	*Thymelicus*	*acteon*	Puerto del Leon	Malaga	Andalusia	Spain	664
KP870635	EZSPC335-09	RVcoll.08-M683	x	*Hesperiidae*	*Thymelicus*	*acteon*	Farena, Alt Camp	Tarragona	Catalonia	Spain	580
KP871093	EZSPC336-09	RVcoll.08-M693	x	*Hesperiidae*	*Thymelicus*	*acteon*	Farena, Alt Camp	Tarragona	Catalonia	Spain	580
GU707097	FBLMU440-09	BC ZSM Lep 27090		*Hesperiidae*	*Thymelicus*	*lineola*	Diessen, Huebschenried	Oberbayern	Bavaria	Germany	650
GU686843	GWORL458-09	BC ZSM Lep 22360		*Hesperiidae*	*Thymelicus*	*lineola*	Muenchen - Allach	Oberbayern	Bavaria	Germany	500
HM391842	FBLMU511-09	BC ZSM Lep 27161		*Hesperiidae*	*Thymelicus*	*lineola*	Kallmuenz	Oberpfalz	Bavaria	Germany	395
HQ005227	EZROM595-08	RV-07-C308		*Hesperiidae*	*Thymelicus*	*lineola*	Oraseni Deal	Botosani	Moldavia	Romania	150
KP870565	EULEP042-14	08-H019		*Hesperiidae*	*Thymelicus*	*lineola*	Poiana Santivan, Popesti (jud. Cluj)	Cluj	Transylvania	Romania	
HQ005225	EZROM553-08	RV-06-M868		*Hesperiidae*	*Thymelicus*	*lineola*	Cluj-Napoca, dealul Feleacului, Gheorgheni forest	Cluj	Transylvania	Romania	600
HQ005226	EZROM554-08	RV-06-M920		*Hesperiidae*	*Thymelicus*	*lineola*	Cluj-Napoca, dealul Bogomaia, Fanatele Clujului	Cluj	Transylvania	Romania	480
HQ005224	EZRMN287-08	RVcoll.08-M426		*Hesperiidae*	*Thymelicus*	*lineola*	3 km E of Slava Rusa (Babadag forest)	Tulcea	Dobrogea	Romania	130
HQ005223	EZRMN288-08	RVcoll.08-M428		*Hesperiidae*	*Thymelicus*	*lineola*	3 km E of Slava Rusa (Babadag forest)	Tulcea	Dobrogea	Romania	130
HQ005222	EZRMN289-08	RVcoll.08-M432		*Hesperiidae*	*Thymelicus*	*lineola*	8 Km S of Babadag (Babadag forest)	Tulcea	Dobrogea	Romania	115
HM901573	EZSPC718-10	RVcoll.08-H333	x	*Hesperiidae*	*Thymelicus*	*lineola*	La Maranosa, San Martin de la Vega		Comunidad de Madrid	Spain	
GU676985	EZSPN238-09	RVcoll.07-W088	x	*Hesperiidae*	*Thymelicus*	*lineola*	P.K. 1,9 M-219, Loeches		Comunidad de Madrid	Spain	
HM901561	EZSPC704-10	RVcoll.07-W098	x	*Hesperiidae*	*Thymelicus*	*lineola*	San Martin del Pimpollar, Piscifactoria Rasquilla	Avila	Castilla y Leon	Spain	1270
GU676987	EZSPN236-09	RVcoll.07-W086	x	*Hesperiidae*	*Thymelicus*	*lineola*	Sierra de Gredos, San Martin del Pimpollar	Avila	Castilla y Leon	Spain	1270
KP870554	EZSPC340-09	RVcoll.07-C667	x	*Hesperiidae*	*Thymelicus*	*lineola*	Sant Marti de Puigcerda	Girona	Catalonia	Spain	1145
KP870509	EZSPC341-09	RVcoll.07-C668	x	*Hesperiidae*	*Thymelicus*	*lineola*	Sant Marti de Puigcerda	Girona	Catalonia	Spain	1145
HM901495	EZSPC1359-10	RVcoll.09-V942	x	*Hesperiidae*	*Thymelicus*	*lineola*	Sierra de la Sagra	Granada	Andalusia	Spain	1745
HM901671	EZSPC823-10	RVcoll.08-P725	x	*Hesperiidae*	*Thymelicus*	*lineola*	Aldeire	Granada	Andalusia	Spain	1335
HM901642	EZSPC789-10	RVcoll.08-M050	x	*Hesperiidae*	*Thymelicus*	*lineola*	Gualda, La Alcarria	Guadalajara	Castilla-La Mancha	Spain	730
GU676167	EZSPN991-09	RVcoll.08-M051	x	*Hesperiidae*	*Thymelicus*	*lineola*	Gualda, La Alcarria	Guadalajara	Castilla-La Mancha	Spain	730
GU676168	EZSPN992-09	RVcoll.08-M052	x	*Hesperiidae*	*Thymelicus*	*lineola*	Gualda, La Alcarria	Guadalajara	Castilla-La Mancha	Spain	730
KP871109	EZSPC337-09	RVcoll.08-L487	x	*Hesperiidae*	*Thymelicus*	*lineola*	Bassa d`Arres, Vall d`Aran	Lleida	Catalonia	Spain	1501
KP870297	EZSPC338-09	RVcoll.08-L488	x	*Hesperiidae*	*Thymelicus*	*lineola*	Bassa d`Arres, Vall d`Aran	Lleida	Catalonia	Spain	1501
KP870630	EZSPC339-09	RVcoll.08-P388	x	*Hesperiidae*	*Thymelicus*	*lineola*	Arros, Vall d`Aran	Lleida	Catalonia	Spain	906
KP870339	EZSPM893-12	Rvcoll.12-M615	x	*Hesperiidae*	*Thymelicus*	*lineola*	Montuenga	Segovia	Castilla y Leon	Spain	890

GenBank accession number	BOLD Process ID	Sample ID	Used in the Iberian dataset	Family	Genus	Species	Exact Site	Region	State/Province	Country	Alt. (m)
GU676982	EZSPN239-09	RVcoll.07-W093	x	*Hesperiidae*	*Thymelicus*	*lineola*	Sierra de Ayllon, Santibanez de Ayllon	Segovia	Castilla y Leon	Spain	
HM901591	EZSPC736-10	RVcoll.08-J031	x	*Hesperiidae*	*Thymelicus*	*lineola*	Jarafuel, Jarafuel	Valencia	Comunidad Valenciana	Spain	
KP870607	EZSPM896-12	Rvcoll.12-M618	x	*Hesperiidae*	*Thymelicus*	*lineola*	Medina del Campo	Valladolid	Castilla y Leon	Spain	739
JF415731	GWORR704-10	BC ZSM Lep 32200		*Hesperiidae*	*Thymelicus*	*sylvestris*	Jachenau	Oberbayern	Bavaria	Germany	790
GU676540	EZSPN597-09	RVcoll.08-J187	x	*Hesperiidae*	*Thymelicus*	*sylvestris*	Serra do Larouco (topo), Padornelos, Montalegre		Tras-os-Montes	Portugal	1520
HQ005230	EZRMN295-08	RVcoll.08-M517		*Hesperiidae*	*Thymelicus*	*sylvestris*	Apuseni Mts., Rimetea (Piatra Secuiului)	Alba	Transylvania	Romania	800
HQ005237	EZROM692-08	RV-07-E469		*Hesperiidae*	*Thymelicus*	*sylvestris*	Dumbrava Vadului, Vad	Brasov	Transylvania	Romania	495
HQ005241	EZROM556-08	RV-06-M940		*Hesperiidae*	*Thymelicus*	*sylvestris*	Dealul Istrita, Breaza	Buzau	Muntenia	Romania	600
HQ005242	EZROM557-08	RV-06-M948		*Hesperiidae*	*Thymelicus*	*sylvestris*	Dealul Istrita, Breaza	Buzau	Muntenia	Romania	600
HQ005234	EZRMN290-08	RVcoll.06-V658		*Hesperiidae*	*Thymelicus*	*sylvestris*	Faget forest, Ratu Penii	Cluj	Transylvania	Romania	680
HQ005228	EZRMN294-08	RVcoll.08-M502		*Hesperiidae*	*Thymelicus*	*sylvestris*	Badeni	Cluj	Transylvania	Romania	490
HQ005239	EZROM337-08	RV-08-A015		*Hesperiidae*	*Thymelicus*	*sylvestris*	Cluj-Napoca, dealul Feleacului, Gheorgheni forest	Cluj	Transylvania	Romania	600
HQ005243	EZROM558-08	RV-06-V657		*Hesperiidae*	*Thymelicus*	*sylvestris*	Faget forest, Ratu Penii	Cluj	Transylvania	Romania	680
HQ005244	EZROM559-08	RV-07-D942		*Hesperiidae*	*Thymelicus*	*sylvestris*	Ciupercenii de Oltet	Gorj	Oltenia	Romania	500
HQ005236	EZROM691-08	RV-07-D904		*Hesperiidae*	*Thymelicus*	*sylvestris*	Ciupercenii de Oltet	Gorj	Oltenia	Romania	500
HQ005231	EZRMN296-08	RVcoll.08-M596		*Hesperiidae*	*Thymelicus*	*sylvestris*	Gheorgheni, Valea Belchia	Harghita	Transylvania	Romania	1000
HQ005235	EZRMN286-08	RVcoll.07-D561		*Hesperiidae*	*Thymelicus*	*sylvestris*	Spini	Hunedoara	Transylvania	Romania	240
HQ005240	EZROM555-08	RV-07-D570		*Hesperiidae*	*Thymelicus*	*sylvestris*	Spini	Hunedoara	Transylvania	Romania	240
HQ005238	EZROM690-08	RV-07-D567		*Hesperiidae*	*Thymelicus*	*sylvestris*	Spini	Hunedoara	Transylvania	Romania	240
HQ005233	EZRMN291-08	RVcoll.08-M397		*Hesperiidae*	*Thymelicus*	*sylvestris*	Macin Mts. (3 Km S of Jijila)	Tulcea	Dobrogea	Romania	230
HQ005232	EZRMN292-08	RVcoll.08-M409		*Hesperiidae*	*Thymelicus*	*sylvestris*	Macin Mts. (3 Km S of Jijila)	Tulcea	Dobrogea	Romania	230
HQ005229	EZRMN293-08	RVcoll.08-M474		*Hesperiidae*	*Thymelicus*	*sylvestris*	Babadag forest (10 Km S of Babadag)	Tulcea	Dobrogea	Romania	115
HM901574	EZSPC719-10	RVcoll.08-H340	x	*Hesperiidae*	*Thymelicus*	*sylvestris*	Puerto de Morcuera, Miraflores		Comunidad de Madrid	Spain	
KP870902	EZSPC342-09	RVcoll.08-L451	x	*Hesperiidae*	*Thymelicus*	*sylvestris*	La Llavina, Montseny, Valles Oriental	Barcelona	Catalonia	Spain	500
KP870358	EZSPC343-09	RVcoll.08-R284	x	*Hesperiidae*	*Thymelicus*	*sylvestris*	Masia del Plans, Vidra, La Garrotxa	Barcelona	Catalonia	Spain	1200
HM901773	EZSPC936-10	RVcoll.08-L506	x	*Hesperiidae*	*Thymelicus*	*sylvestris*	El Cafe, pla de la Calma, El Brull, Osona	Barcelona	Catalonia	Spain	1192
KP870245	EZSPM632-12	RVcoll.11-E182	x	*Hesperiidae*	*Thymelicus*	*sylvestris*	Figols, Bergueda	Barcelona	Catalonia	Spain	1111
KP870464	EZSPM1024-12	Rvcoll.12-M774	x	*Hesperiidae*	*Thymelicus*	*sylvestris*	7 km SE Navalvillar de Ibor	Caceres	Extremadura	Spain	729
KP871156	EZSPM1028-12	Rvcoll.12-M778	x	*Hesperiidae*	*Thymelicus*	*sylvestris*	Hospital del Obispo, Navalvillar de Ibor	Caceres	Extremadura	Spain	1010
KP870470	EZSPM924-12	Rvcoll.12-M646	x	*Hesperiidae*	*Thymelicus*	*sylvestris*	Lagunas de Ruidera, Ossa de Montiel	Ciudad Real	Castilla-La Mancha	Spain	795
KP870604	EZSPM928-12	Rvcoll.12-M652	x	*Hesperiidae*	*Thymelicus*	*sylvestris*	Puerto de Calero, Solana del Pino	Ciudad Real	Castilla-La Mancha	Spain	960
KP870777	EZSPM961-12	Rvcoll.12-M712	x	*Hesperiidae*	*Thymelicus*	*sylvestris*	Alrededores San Lorenzo de Calatrava	Ciudad Real	Castilla-La Mancha	Spain	749
KP870633	EZSPM973-12	Rvcoll.12-M731	x	*Hesperiidae*	*Thymelicus*	*sylvestris*	Arroyo de los Molinillos, Viso del Marques	Ciudad Real	Castilla-La Mancha	Spain	742
KP870573	EZSPC344-09	RVcoll.07-C634	x	*Hesperiidae*	*Thymelicus*	*sylvestris*	Meranges	Girona	Catalonia	Spain	1555
HM901261	EZSPC1058-10	RVcoll.08-L005	x	*Hesperiidae*	*Thymelicus*	*sylvestris*	Carretera al Veleta, Gueejar Sierra	Granada	Andalusia	Spain	1266
HM901403	EZSPC1203-10	RVcoll.09-V451	x	*Hesperiidae*	*Thymelicus*	*sylvestris*	Loma de Lastonares (Sierra Nevada)	Granada	Andalusia	Spain	2050
HM901404	EZSPC1204-10	RVcoll.09-V461	x	*Hesperiidae*	*Thymelicus*	*sylvestris*	Puerto del Lobo, Pista de las Alpujarras	Granada	Andalusia	Spain	1950
HM901833	EZSPC998-10	RVcoll.09-V464	x	*Hesperiidae*	*Thymelicus*	*sylvestris*	Puerto del Lobo, Pista de las Alpujarras	Granada	Andalusia	Spain	1950
KP870254	EZSPM633-12	RVcoll.11-G143	x	*Hesperiidae*	*Thymelicus*	*sylvestris*	Puerto de la Ragua (Sierra Nevada)	Granada	Andalusia	Spain	2090
KP870924	EZSPM645-12	RVcoll.11-I407	x	*Hesperiidae*	*Thymelicus*	*sylvestris*	Puerto de la Ragua (Sierra Nevada)	Granada	Andalusia	Spain	2090
KP870751	EZSPM659-12	RVcoll.11-I501	x	*Hesperiidae*	*Thymelicus*	*sylvestris*	SE of Cumbres Verdes (La Zubia)	Granada	Andalusia	Spain	1400
KP870937	EZSPM665-12	RVcoll.11-I581	x	*Hesperiidae*	*Thymelicus*	*sylvestris*	ca. 8 km E of Cumbres Verdes (La Zubia), botanical garden	Granada	Andalusia	Spain	1700
JN273363	EZSPN1073-11	Rvcoll.09-V460	x	*Hesperiidae*	*Thymelicus*	*sylvestris*	Puerto del Lobo, Pista de las Alpujarras	Granada	Andalusia	Spain	1950
GU676179	EZSPN979-09	RVcoll.08-M017	x	*Hesperiidae*	*Thymelicus*	*sylvestris*	Gualda, La Alcarria	Guadalajara	Castilla-La Mancha	Spain	730
KP870713	EZSPC345-09	RVcoll.08-P355	x	*Hesperiidae*	*Thymelicus*	*sylvestris*	Pas de Perves	Lleida	Catalonia	Spain	1246
KP871140	EZSPC346-09	RVcoll.08-L486	x	*Hesperiidae*	*Thymelicus*	*sylvestris*	Bassa d`Arres, Vall d`Aran	Lleida	Catalonia	Spain	1501
KP870957	EZSPM620-12	RVcoll.11-D879.1	x	*Hesperiidae*	*Thymelicus*	*sylvestris*	Frigiliana	Malaga	Andalusia	Spain	433
KP871170	EZSPM621-12	RVcoll.11-D880.1	x	*Hesperiidae*	*Thymelicus*	*sylvestris*	Frigiliana	Malaga	Andalusia	Spain	433
GU676744	EZSPN291-09	RVcoll.07-W252	x	*Hesperiidae*	*Thymelicus*	*sylvestris*	Candelario	Salamanca	Castilla y Leon	Spain	1300
HM901771	EZSPC934-10	RVcoll.06-A265	x	*Hesperiidae*	*Thymelicus*	*sylvestris*	Sierra de Ayllon, Grado del Pico	Segovia	Castilla y Leon	Spain	
GU676809	EZSPN122-09	RVcoll.06-A300	x	*Hesperiidae*	*Thymelicus*	*sylvestris*	Sierra de Ayllon, San Esteban de Gormaz	Soria	Castilla y Leon	Spain	
HM901253	EZSPC1050-10	RVcoll.08-J775	x	*Hesperiidae*	*Thymelicus*	*sylvestris*	Ruta de la Sima, El Vallecillo	Teruel	Aragon	Spain	1531

GenBank accession number	BOLD Process ID	Sample ID	Used in the Iberian dataset	Family	Genus	Species	Exact Site	Region	State/Province	Country	Alt. (m)
HM901210	EZSPC1006-10	RVcoll.08-H413	x	*Lycaenidae*	*Tomares*	*ballus*	Cantoblanco		Comunidad de Madrid	Spain	720
GU676697	EZSPN341-09	RVcoll.08-H300	x	*Lycaenidae*	*Tomares*	*ballus*	Villaverde, Madrid		Comunidad de Madrid	Spain	
GU676661	EZSPN379-09	RVcoll.08-H425	x	*Lycaenidae*	*Tomares*	*ballus*	Guadalix de la Sierra		Comunidad de Madrid	Spain	882
GU676788	EZSPN129-09	RVcoll.06-G460	x	*Lycaenidae*	*Tomares*	*ballus*	El Sola, El Brull, Osona	Barcelona	Catalonia	Spain	800
GU676531	EZSPN613-09	RVcoll.08-J357	x	*Lycaenidae*	*Tomares*	*ballus*	Vallgrassa, Parc Natural del Garraf	Barcelona	Catalonia	Spain	285
JN280793	EZSPC1323-10	RVcoll.130209KL35	x	*Lycaenidae*	*Tomares*	*ballus*	Sa Ubrique,Ubrique	Cadiz	Andalusia	Spain	
JN280792	EZSPC1275-10	RVcoll.130606MU13	x	*Lycaenidae*	*Tomares*	*ballus*	Pina de Montalgrao,Pina de Montalgrao	Castellon	Comunidad Valenciana	Spain	
HM901457	EZSPC1277-10	RVcoll.070207MC2	x	*Lycaenidae*	*Tomares*	*ballus*	Rio Maimona,Puebla de Arenoso	Castellon	Comunidad Valenciana	Spain	707
HM901214	EZSPC1010-10	RVcoll.08-H531	x	*Lycaenidae*	*Tomares*	*ballus*	Gualda, La Alcarria	Guadalajara	Castilla-La Mancha	Spain	730
HM901215	EZSPC1011-10	RVcoll.08-H540	x	*Lycaenidae*	*Tomares*	*ballus*	Pastrana, La Alcarria	Guadalajara	Castilla-La Mancha	Spain	702
GU676623	EZSPN414-09	RVcoll.08-H532	x	*Lycaenidae*	*Tomares*	*ballus*	Gualda, La Alcarria	Guadalajara	Castilla-La Mancha	Spain	730
GU676614	EZSPN430-09	RVcoll.08-H607	x	*Lycaenidae*	*Tomares*	*ballus*	Aldeanueva de Figueroa	Salamanca	Castilla y Leon	Spain	900
KP870811	FBLMX236-11	BC ZSM Lep 50447		*Nymphalidae*	*Vanessa*	*atalanta*	Landshut-West	Niederbayern	Bavaria	Germany	400
HM393224	GWORA2510-09	BC ZSM Lep 30722		*Nymphalidae*	*Vanessa*	*atalanta*	Hausmetting	Niederbayern	Bavaria	Germany	370
HM393225	GWORA2511-09	BC ZSM Lep 30723		*Nymphalidae*	*Vanessa*	*atalanta*	Bogen, Bogenberg	Niederbayern	Bavaria	Germany	400
HQ005254	EZROM561-08	RV-07-D077		*Nymphalidae*	*Vanessa*	*atalanta*	Dealul Istrita, Breaza	Buzau	Muntenia	Romania	540
HQ005255	EZROM562-08	RV-07-D226		*Nymphalidae*	*Vanessa*	*atalanta*	Dealul Istrita, Breaza	Buzau	Muntenia	Romania	540
HQ005253	EZROM563-08	RV-07-D596		*Nymphalidae*	*Vanessa*	*atalanta*	Teregova	Caras-Severin	Banat	Romania	385
HQ005250	EZROM328-08	RV-08-A006		*Nymphalidae*	*Vanessa*	*atalanta*	Cluj-Napoca, dealul Feleacului, Gheorgheni forest	Cluj	Transylvania	Romania	600
HQ005251	EZROM560-08	RV-06-M897		*Nymphalidae*	*Vanessa*	*atalanta*	Cluj-Napoca, dealul Feleacului, Gheorgheni forest	Cluj	Transylvania	Romania	600
HQ005247	EZRMN298-08	RVcoll.08-M423		*Nymphalidae*	*Vanessa*	*atalanta*	Gura Dobrogei reserve	Constanta	Dobrogea	Romania	90
HQ005248	EZRMN299-08	RVcoll.08-M443		*Nymphalidae*	*Vanessa*	*atalanta*	Canaraua Fetei (Baneasa)	Constanta	Dobrogea	Romania	20
HQ005252	EZROM596-08	RV-07-C360		*Nymphalidae*	*Vanessa*	*atalanta*	Sadova, pas Pascanu	Suceava	Moldavia	Romania	1100
HQ005249	EZRMN297-08	RVcoll.08-M395		*Nymphalidae*	*Vanessa*	*atalanta*	Macin Mts. (3 Km S of Jijila)	Tulcea	Dobrogea	Romania	230
FJ664080	LOWA162-06	2005-LOWA-162		*Nymphalidae*	*Vanessa*	*atalanta*	Osyno vill.	Sebezh distr	Pskov	Russia	150
FJ664079	LOWA163-06	2005-LOWA-163		*Nymphalidae*	*Vanessa*	*atalanta*	Osyno vill.	Sebezh distr	Pskov	Russia	150
FJ664078	LOWA784-06	2005-LOWA-784		*Nymphalidae*	*Vanessa*	*atalanta*	Osyno	Sebezh distr.	Pskov	Russia	150
JN827889	EZSPC347-09	RVcoll.08-H295	x	*Nymphalidae*	*Vanessa*	*atalanta*	Granja Viader, Cardedeu	Barcelona	Catalonia	Spain	190
JN827890	EZSPC348-09	RVcoll.08-R278	x	*Nymphalidae*	*Vanessa*	*atalanta*	Masia del Plans, Vidra, La Garrotxa	Barcelona	Catalonia	Spain	1200
GU676895	EZSPN490-09	RVcoll.08-H927	x	*Nymphalidae*	*Vanessa*	*atalanta*	Hervas, Carretera Hervas-La Garganta	Caceres	Extremadura	Spain	940
GU676595	EZSPN448-09	RVcoll.08-H640	x	*Nymphalidae*	*Vanessa*	*atalanta*	La Linea de la Concepcion, playa de Santa Margarita	Cadiz	Andalusia	Spain	
JN827893	EZSPC351-09	RVcoll.08-P077	x	*Nymphalidae*	*Vanessa*	*atalanta*	Estoll, Fontanals de Cerdanya, Cerdanya	Girona	Catalonia	Spain	1085
GU676638	EZSPN405-09	RVcoll.08-H491	x	*Nymphalidae*	*Vanessa*	*atalanta*	San Andres del Rey, La Alcarria	Guadalajara	Castilla-La Mancha	Spain	1018
GU675691	EZSPM160-09	RVcoll.08-P687	x	*Nymphalidae*	*Vanessa*	*atalanta*	Villadangos de Arbas	Leon	Castilla y Leon	Spain	1311
JN827892	EZSPC349-09	RVcoll.08-L489	x	*Nymphalidae*	*Vanessa*	*atalanta*	Bassa d`Arres, Vall d`Aran	Lleida	Catalonia	Spain	1501
JN827891	EZSPC350-09	RVcoll.08-R308	x	*Nymphalidae*	*Vanessa*	*atalanta*	Planes de Son, Pallars Sobira	Lleida	Catalonia	Spain	1589
GU675771	EZSPM205-09	RVcoll.08-R323	x	*Nymphalidae*	*Vanessa*	*atalanta*	Es Teix, Valldemossa	Mallorca	Balearic Islands	Spain	920
KP870311	EZSPM1004-12	Rvcoll.12-L890	x	*Nymphalidae*	*Vanessa*	*atalanta*	Torralbet, Ciutadella, Menorca	Menorca	Balearic Islands	Spain	
KP870540	EZSPM997-12	Rvcoll.12-L883	x	*Nymphalidae*	*Vanessa*	*atalanta*	Santa Cecilia, Ferreries, Menorca	Menorca	Balearic Islands	Spain	
GU676742	EZSPN297-09	RVcoll.08-H100	x	*Nymphalidae*	*Vanessa*	*atalanta*	Na Vermella, Mao	Menorca	Balearic Islands	Spain	
GU676735	EZSPN298-09	RVcoll.08-H101	x	*Nymphalidae*	*Vanessa*	*atalanta*	Na Vermella, Mao	Menorca	Balearic Islands	Spain	
GU676671	EZSPN365-09	RVcoll.08-H389	x	*Nymphalidae*	*Vanessa*	*atalanta*	La Canada, Paterna	Valencia	Comunidad Valenciana	Spain	
GU707087	FBLMU450-09	BC ZSM Lep 27100		*Nymphalidae*	*Vanessa*	*cardui*	Diessen, Huebschenried	Oberbayern	Bavaria	Germany	650
JF415732	GWORK320-09	BC ZSM Lep 21555		*Nymphalidae*	*Vanessa*	*cardui*	Bahndamm zwischen Markt Schwaben und Poing	Oberbayern	Bavaria	Germany	520
GU686854	GWORL445-09	BC ZSM Lep 22347		*Nymphalidae*	*Vanessa*	*cardui*	Muenchen - Allach	Oberbayern	Bavaria	Germany	500
HQ005261	EZROM624-08	RV-07-E382		*Nymphalidae*	*Vanessa*	*cardui*	Arjana Mountain	Caras-Severin	Banat	Romania	1365
HQ005263	EZROM141-08	RV-07-D416		*Nymphalidae*	*Vanessa*	*cardui*	Suatu	Cluj	Transylvania	Romania	380

GenBank accession number	BOLD Process ID	Sample ID	Used in the Iberian dataset	Family	Genus	Species	Exact Site	Region	State/Province	Country	Alt. (m)
HQ005264	EZROM142-08	RV-07-D426		*Nymphalidae*	*Vanessa*	*cardui*	Suatu	Cluj	Transylvania	Romania	380
HQ005256	EZRMN300-08	RVcoll.07-D934		*Nymphalidae*	*Vanessa*	*cardui*	11 km south of Ranca	Gorj	Oltenia	Romania	940
HQ005259	EZRMN303-08	RVcoll.08-M382		*Nymphalidae*	*Vanessa*	*cardui*	Garvan	Tulcea	Dobrogea	Romania	100
HQ005260	EZRMN304-08	RVcoll.08-M398		*Nymphalidae*	*Vanessa*	*cardui*	Macin Mts. (3 Km S of Jijila)	Tulcea	Dobrogea	Romania	230
HQ005262	EZROM140-08	RV-07-D063		*Nymphalidae*	*Vanessa*	*cardui*	Horia	Tulcea	Dobrogea	Romania	140
HQ005257	EZRMN301-08	RVcoll.07-E484		*Nymphalidae*	*Vanessa*	*cardui*	Buila-Vanturarita (Vanturarita Mare)	Valcea	Oltenia	Romania	1545
HQ005258	EZRMN302-08	RVcoll.08-M362		*Nymphalidae*	*Vanessa*	*cardui*	Bujoreni monastery (9 Km E of Zorleni)	Vaslui	Moldavia	Romania	240
GU676162	EZSPN998-09	RVcoll.08-M077	x	*Nymphalidae*	*Vanessa*	*cardui*	Aranjuez		Comunidad de Madrid	Spain	588
GU669857	EZSPC376-09	RVcoll.08-M717	x	*Nymphalidae*	*Vanessa*	*cardui*	Delta de Llobregat	Barcelona	Catalonia	Spain	2
GU676608	EZSPN436-09	RVcoll.08-H618	x	*Nymphalidae*	*Vanessa*	*cardui*	El Gastor	Cadiz	Andalusia	Spain	660
GU676594	EZSPN447-09	RVcoll.08-H639	x	*Nymphalidae*	*Vanessa*	*cardui*	La Linea de la Concepcion, playa de Santa Margarita	Cadiz	Andalusia	Spain	
KP871040	EZSPM983-12	Rvcoll.12-M751	x	*Nymphalidae*	*Vanessa*	*cardui*	Illa Grossa, Columbretes, Castellon de la Plana	Castellon	Comunidad Valenciana	Spain	26.6
KP870469	EZSPM984-12	Rvcoll.12-M752	x	*Nymphalidae*	*Vanessa*	*cardui*	Illa Grossa, Columbretes, Castellon de la Plana	Castellon	Comunidad Valenciana	Spain	26.6
KP870949	EZSPM985-12	Rvcoll.12-M753	x	*Nymphalidae*	*Vanessa*	*cardui*	Illa Grossa, Columbretes, Castellon de la Plana	Castellon	Comunidad Valenciana	Spain	26.6
GU669858	EZSPC377-09	RVcoll.08-R296	x	*Nymphalidae*	*Vanessa*	*cardui*	Masia del Plans, Vidra, La Garrotxa	Girona	Catalonia	Spain	1200
GU669859	EZSPC378-09	RVcoll.07-C077	x	*Nymphalidae*	*Vanessa*	*cardui*	Puig Neulos, Alt Emporda	Girona	Catalonia	Spain	1110
GU669852	EZSPC379-09	RVcoll.07-F334	x	*Nymphalidae*	*Vanessa*	*cardui*	El Mont, Albanya, Alt Emporda	Girona	Catalonia	Spain	860
GU676915	EZSPN510-09	RVcoll.08-H968	x	*Nymphalidae*	*Vanessa*	*cardui*	Laguarta, Rio Alcanadre	Huesca	Aragon	Spain	1216
GU676043	EZSPM031-09	RVcoll.08-P230	x	*Nymphalidae*	*Vanessa*	*cardui*	Puig d`en Galileu	Mallorca	Balearic Islands	Spain	1071
GU675772	EZSPM214-09	RVcoll.08-R335	x	*Nymphalidae*	*Vanessa*	*cardui*	Palma de Mallorca	Mallorca	Balearic Islands	Spain	88
KP871009	EZSPM1005-12	Rvcoll.12-L893	x	*Nymphalidae*	*Vanessa*	*cardui*	Torralbet, Ciutadella, Menorca	Menorca	Balearic Islands	Spain	
GU676733	EZSPN305-09	RVcoll.08-H110	x	*Nymphalidae*	*Vanessa*	*cardui*	Na Vermella, Mao	Menorca	Balearic Islands	Spain	
GU676724	EZSPN312-09	RVcoll.08-H121	x	*Nymphalidae*	*Vanessa*	*cardui*	Barranc d`Alguendar	Menorca	Balearic Islands	Spain	60
KP871112	EZSPN1089-11	Rvcoll.09-X679	x	*Nymphalidae*	*Vanessa*	*virginiensis*	Malpartida de Plasencia	Caceres	Extremadura	Spain	600
GU676868	EZSPN462-09	RVcoll.08-H675	x	*Nymphalidae*	*Vanessa*	*virginiensis*	Malpartida de Plasencia	Caceres	Extremadura	Spain	600
HM901218	EZSPC1014-10	RVcoll.08-H560	x	*Pieridae*	*Zegris*	*eupheme*	Campus Cantoblanco		Comunidad de Madrid	Spain	725
GU676688	EZSPN349-09	RVcoll.08-H318	x	*Pieridae*	*Zegris*	*eupheme*	La Maranosa, San Martin de la Vega		Comunidad de Madrid	Spain	
GU676628	EZSPN419-09	RVcoll.08-H559	x	*Pieridae*	*Zegris*	*eupheme*	Campus Cantoblanco		Comunidad de Madrid	Spain	725
HM901217	EZSPC1013-10	RVcoll.08-H544	x	*Pieridae*	*Zegris*	*eupheme*	Chillaron del Rey, La Alcarria	Guadalajara	Castilla-La Mancha	Spain	710
GU676626	EZSPN417-09	RVcoll.08-H543	x	*Pieridae*	*Zegris*	*eupheme*	Chillaron del Rey, La Alcarria	Guadalajara	Castilla-La Mancha	Spain	710
HM901204	EZSPC1000-10	RVcoll.08-H238	x	*Pieridae*	*Zegris*	*eupheme*	Mequinenza	Huesca	Aragon	Spain	129.2
HM901205	EZSPC1001-10	RVcoll.08-H242	x	*Pieridae*	*Zegris*	*eupheme*	Mequinenza	Huesca	Aragon	Spain	129.2
HM901206	EZSPC1002-10	RVcoll.08-H243	x	*Pieridae*	*Zegris*	*eupheme*	Mequinenza	Huesca	Aragon	Spain	129.2
GU675915	EZSPM319-09	RVcoll.08-J885	x	*Pieridae*	*Zegris*	*eupheme*	Villaobispo de las Regueras	Leon	Castilla y Leon	Spain	
GU676937	EZSPN533-09	RVcoll.08-J009	x	*Pieridae*	*Zegris*	*eupheme*	Jalance, 560 mtrs., Jalance	Valencia	Comunidad Valenciana	Spain	
GU676939	EZSPN535-09	RVcoll.08-J012	x	*Pieridae*	*Zegris*	*eupheme*	Sinarcas, Sinarcas	Valencia	Comunidad Valenciana	Spain	
GU676708	EZSPN329-09	RVcoll.08-H240	x	*Pieridae*	*Zegris*	*eupheme*	Mequinenza	Zaragoza	Aragon	Spain	129
HQ005266	EZRMN404-09	RVcoll.07-D396		*Papilionidae*	*Zerynthia*	*polyxena*	Nenciulesti	Buzau	Muntenia	Romania	360
HQ005267	EZROM566-08	RV-07-D395		*Papilionidae*	*Zerynthia*	*polyxena*	Nenciulesti	Buzau	Muntenia	Romania	360
HQ005265	EZRMN405-09	RVcoll.07-D023		*Papilionidae*	*Zerynthia*	*polyxena*	Canaraua Fetei (Baneasa)	Constanta	Dobrogea	Romania	20
GU675629	EZROM1069-09	RVcoll.08-L358		*Papilionidae*	*Zerynthia*	*polyxena*	Canaraua Fetei (Baneasa)	Constanta	Dobrogea	Romania	20
GU675632	EZROM1064-09	RVcoll.07-F560		*Papilionidae*	*Zerynthia*	*polyxena*	Calan	Hunedoara	Transylvania	Romania	230
HQ005268	EZROM695-08	RV-07-F537		*Papilionidae*	*Zerynthia*	*polyxena*	Calan	Hunedoara	Transylvania	Romania	230
GU676664	EZSPN382-09	RVcoll.08-H430	x	*Papilionidae*	*Zerynthia*	*rumina*	Campo Real		Comunidad de Madrid	Spain	731
GU676653	EZSPN383-09	RVcoll.08-H431	x	*Papilionidae*	*Zerynthia*	*rumina*	Campo Real		Comunidad de Madrid	Spain	731

GenBank accession number	BOLD Process ID	Sample ID	Used in the Iberian dataset	Family	Genus	Species	Exact Site	Region	State/Province	Country	Alt. (m)
GU676635	EZSPN402-09	RVcoll.08-H472	x	*Papilionidae*	*Zerynthia*	*rumina*	Soto del Real		Comunidad de Madrid	Spain	1190
GU676683	EZSPN352-09	RVcoll.08-H323	x	*Papilionidae*	*Zerynthia*	*rumina*	Cebreros, Cebreros	Avila	Castilla y Leon	Spain	
GU677025	EZSPN191-09	RVcoll.07-C402	x	*Papilionidae*	*Zerynthia*	*rumina*	Massis del Garraf, Viladellops	Barcelona	Catalonia	Spain	
GU676591	EZSPN456-09	RVcoll.08-H662	x	*Papilionidae*	*Zerynthia*	*rumina*	Ubrique, Puerto de Galis	Cadiz	Andalusia	Spain	579
GU676867	EZSPN460-09	RVcoll.08-H668	x	*Papilionidae*	*Zerynthia*	*rumina*	Grazalema, Puerto de los Alamillos	Cadiz	Andalusia	Spain	887
GU675892	EZSPM346-09	RVcoll.08-R042	x	*Papilionidae*	*Zerynthia*	*rumina*	Espadilla	Castellon	Comunidad Valenciana	Spain	395
JN275715	EZSPN1041-11	Rvcoll.08-J350	x	*Papilionidae*	*Zerynthia*	*rumina*	Cantallops, Alt Emporda	Girona	Catalonia	Spain	225
GU676842	EZSPN089-09	RVcoll.06-A018	x	*Papilionidae*	*Zerynthia*	*rumina*	Mecina Bombaron, Alpujarras	Granada	Andalusia	Spain	1150
GU676535	EZSPN604-09	RVcoll.08-J311	x	*Papilionidae*	*Zerynthia*	*rumina*	Barranco de Valcuerna, Candasnos	Huesca	Aragon	Spain	210
GU676903	EZSPN498-09	RVcoll.08-H947	x	*Papilionidae*	*Zerynthia*	*rumina*	Candelario, Sierra de Candelario	Salamanca	Castilla y Leon	Spain	1300
JN275716	EZSPN1123-11	Rvcoll.10-C355	x	*Papilionidae*	*Zerynthia*	*rumina*	Sant Jordi d`Alfama, Baix Ebre	Tarragona	Catalonia	Spain	145
GU676672	EZSPN366-09	RVcoll.08-H391	x	*Papilionidae*	*Zerynthia*	*rumina*	La Canada, Paterna	Valencia	Comunidad Valenciana	Spain	
HM901831	EZSPC996-10	RVcoll.07-W244	x	*Lycaenidae*	*Zizeeria*	*knysna*	La Lagunilla, Candeleda	Avila	Castilla y Leon	Spain	380
HM901832	EZSPC997-10	RVcoll.07-W245	x	*Lycaenidae*	*Zizeeria*	*knysna*	La Lagunilla, Candeleda	Avila	Castilla y Leon	Spain	380
GU676748	EZSPN288-09	RVcoll.07-W242	x	*Lycaenidae*	*Zizeeria*	*knysna*	Candeleda, La Lagunilla	Avila	Castilla y Leon	Spain	380
GU676749	EZSPN289-09	RVcoll.07-W243	x	*Lycaenidae*	*Zizeeria*	*knysna*	Candeleda, La Lagunilla	Avila	Castilla y Leon	Spain	380
GU676334	EZSPN820-09	RVcoll.08-L629	x	*Lycaenidae*	*Zizeeria*	*knysna*	Algeciras, Arroyo del Prior	Cadiz	Andalusia	Spain	50
GU676335	EZSPN821-09	RVcoll.08-L630	x	*Lycaenidae*	*Zizeeria*	*knysna*	Algeciras, Arroyo del Prior	Cadiz	Andalusia	Spain	50
KP871162	EZSPM618-12	RVcoll.11-D865	x	*Lycaenidae*	*Zizeeria*	*knysna*	Salobrena	Granada	Andalusia	Spain	3
HM901353	EZSPC1153-10	RVcoll.08-R032	x	*Lycaenidae*	*Zizeeria*	*knysna*	Sant Vicent, Lliria	Valencia	Comunidad Valenciana	Spain	190
GU675692	EZSPM168-09	RVcoll.08-R021	x	*Lycaenidae*	*Zizeeria*	*knysna*	Sant Vicent, Lliria	Valencia	Comunidad Valenciana	Spain	
GU675746	EZSPM171-09	RVcoll.08-R028	x	*Lycaenidae*	*Zizeeria*	*knysna*	Sant Vicent, Lliria	Valencia	Comunidad Valenciana	Spain	
GU675889	EZSPM347-09	RVcoll.08-R030	x	*Lycaenidae*	*Zizeeria*	*knysna*	Sant Vicent, Lliria	Valencia	Comunidad Valenciana	Spain	

Supplementary Table 2 | DNA barcoding performance assessed for the Iberian and the full dataset. Taxa are ordered by family, genus and species.

Family	Genus	species	Iberian dataset	Comments Iberian dataset	Full dataset	Comments full dataset
Hesperiidae	*Borbo*	*borbonica*	monophyletic		monophyletic	
Hesperiidae	*Carcharodus*	*alceae*	monophyletic		monophyletic	
Hesperiidae	*Carcharodus*	*baeticus*	monophyletic		monophyletic	
Hesperiidae	*Carcharodus*	*floccifera*	monophyletic		para-/polyphyletic	paraphyletic (C. orientalis); min. p. distance to C. orientalis is 0.5%
Hesperiidae	*Carcharodus*	*lavatherae*	monophyletic		monophyletic	
Hesperiidae	*Carcharodus*	*orientalis*	n/a		monophyletic	min. p. distance to C. floccifera is 0.5%
Hesperiidae	*Carcharodus*	*tripolina*	monophyletic		monophyletic	
Hesperiidae	*Carterocephalus*	*palaemon*	monophyletic		monophyletic	
Hesperiidae	*Erynnis*	*tages*	monophyletic		monophyletic	
Hesperiidae	*Gegenes*	*nostrodamus*	monophyletic		monophyletic	
Hesperiidae	*Gegenes*	*pumilio*	monophyletic		monophyletic	
Hesperiidae	*Hesperia*	*comma*	monophyletic		monophyletic	
Hesperiidae	*Heteropterus*	*morpheus*	monophyletic		monophyletic	
Hesperiidae	*Muschampia*	*cribrellum*	n/a		monophyletic	
Hesperiidae	*Muschampia*	*proto*	monophyletic		monophyletic	
Hesperiidae	*Muschampia*	*tessellum*	n/a		monophyletic	
Hesperiidae	*Ochlodes*	*sylvanus*	monophyletic		monophyletic	
Hesperiidae	*Pyrgus*	*alveus*	para-/polyphyletic	paraphyletic (P. bellieri); min. p-dist. to P. bellieri is 0.8%	para-/polyphyletic	paraphyletic (P. bellieri); min. p-dist. to P. bellieri is 0.8%
Hesperiidae	*Pyrgus*	*andromedae*	monophyletic		monophyletic	
Hesperiidae	*Pyrgus*	*armoricanus*	monophyletic		para-/polyphyletic	paraphyletic (P. onopordi)
Hesperiidae	*Pyrgus*	*bellieri*	monophyletic	min. p-dist. to P. alveus is 0.8%	monophyletic	min. p-dist. to P. alveus is 0.8%
Hesperiidae	*Pyrgus*	*cacaliae*	monophyletic		monophyletic	
Hesperiidae	*Pyrgus*	*carthami*	monophyletic		monophyletic	
Hesperiidae	*Pyrgus*	*cinarae*	monophyletic		monophyletic	
Hesperiidae	*Pyrgus*	*cirsii*	monophyletic		monophyletic	
Hesperiidae	*Pyrgus*	*malvae*	n/a		monophyletic	
Hesperiidae	*Pyrgus*	*malvoides*	monophyletic		monophyletic	
Hesperiidae	*Pyrgus*	*onopordi*	monophyletic		monophyletic	
Hesperiidae	*Pyrgus*	*serratulae*	monophyletic		monophyletic	

Family	Genus	species	Iberian dataset	Comments Iberian dataset	Full dataset	Comments full dataset
Hesperiidae	*Pyrgus*	*sidae*	monophyletic		monophyletic	
Hesperiidae	*Pyrgus*	*warrenensis*	n/a		monophyletic	
Hesperiidae	*Spialia*	*orbifer*	n/a		monophyletic	
Hesperiidae	*Spialia*	*sertorius*	monophyletic		para-/polyphyletic	paraphyletic (S. orbifer); min. p-dist. to S. orbifer is 1.1%
Hesperiidae	*Thymelicus*	*acteon*	monophyletic		monophyletic	
Hesperiidae	*Thymelicus*	*lineola*	monophyletic		monophyletic	
Hesperiidae	*Thymelicus*	*sylvestris*	monophyletic		monophyletic	
Lycaenidae	*Agriades*	*glandon*	monophyletic		monophyletic	
Lycaenidae	*Agriades*	*optilete*	n/a		monophyletic	
Lycaenidae	*Agriades*	*pyrenaicus*	monophyletic		monophyletic	
Lycaenidae	*Agriades*	*zullichi*	monophyletic		monophyletic	
Lycaenidae	*Aricia*	*agestis*	monophyletic		monophyletic	
Lycaenidae	*Aricia*	*anteros*	n/a		monophyletic	
Lycaenidae	*Aricia*	*artaxerxes*	n/a		monophyletic	
Lycaenidae	*Aricia*	*cramera*	monophyletic		monophyletic	
Lycaenidae	*Aricia*	*montensis*	monophyletic		monophyletic	
Lycaenidae	*Aricia*	*morronensis*	monophyletic		monophyletic	
Lycaenidae	*Aricia*	*nicias*	monophyletic		monophyletic	
Lycaenidae	*Azanus*	*jesous*	monophyletic	singleton	monophyletic	singleton
Lycaenidae	*Cacyreus*	*marshalli*	monophyletic		monophyletic	
Lycaenidae	*Callophrys*	*avis*	monophyletic		monophyletic	
Lycaenidae	*Callophrys*	*rubi*	monophyletic		monophyletic	
Lycaenidae	*Celastrina*	*argiolus*	monophyletic		monophyletic	
Lycaenidae	*Cupido*	*alcetas*	monophyletic		shared barcodes	paraphyletic (C. decolorata); min. p-dist. to C. decolorata is 0.2%
Lycaenidae	*Cupido*	*argiades*	monophyletic		monophyletic	
Lycaenidae	*Cupido*	*decolorata*	n/a		shared barcodes	paraphyletic (C. alcetas); min. p-dist. to C. alcetas is 0.2%
Lycaenidae	*Cupido*	*lorquinii*	shared barcodes	shared barcodes (C. minimus - taxon carswelli)	shared barcodes	shared barcodes (C. minimus taxon carswelli)
Lycaenidae	*Cupido*	*minimus*	shared barcodes	the taxon carswelli shares barcodes with C. lorquinii	shared barcodes	the taxon carswelli shares barcodes with C. lorquinii
Lycaenidae	*Cupido*	*osiris*	monophyletic		monophyletic	

Family	Genus	species	Iberian dataset	Comments Iberian dataset	Full dataset	Comments full dataset
Lycaenidae	*Cyaniris*	*semiargus*	monophyletic		monophyletic	
Lycaenidae	*Eumedonia*	*eumedon*	monophyletic		monophyletic	
Lycaenidae	*Favonius*	*quercus*	monophyletic		monophyletic	
Lycaenidae	*Glaucopsyche*	*alexis*	monophyletic		monophyletic	
Lycaenidae	*Glaucopsyche*	*melanops*	monophyletic		monophyletic	
Lycaenidae	*Iolana*	*debilitata*	monophyletic		monophyletic	
Lycaenidae	*Kretania*	*hesperica*	monophyletic		monophyletic	
Lycaenidae	*Kretania*	*pylaon*	n/a		monophyletic	singleton; min. p-dist. to K. sephirus is 0.3%
Lycaenidae	*Kretania*	*sephirus*	n/a		para-/polyphyletic	paraphyletic (K. pylaon); min. p-dist. to K. pylaon is 0.3%
Lycaenidae	*Laeosopis*	*roboris*	monophyletic		monophyletic	
Lycaenidae	*Lampides*	*boeticus*	monophyletic		monophyletic	
Lycaenidae	*Leptotes*	*pirithous*	monophyletic		monophyletic	
Lycaenidae	*Lycaena*	*alciphron*	monophyletic		monophyletic	
Lycaenidae	*Lycaena*	*bleusei*	monophyletic		monophyletic	
Lycaenidae	*Lycaena*	*dispar*	n/a		monophyletic	
Lycaenidae	*Lycaena*	*helle*	monophyletic		monophyletic	
Lycaenidae	*Lycaena*	*hippothoe*	monophyletic		monophyletic	
Lycaenidae	*Lycaena*	*phlaeas*	monophyletic		monophyletic	
Lycaenidae	*Lycaena*	*thersamon*	n/a		monophyletic	
Lycaenidae	*Lycaena*	*tityrus*	monophyletic		monophyletic	
Lycaenidae	*Lycaena*	*virgaureae*	monophyletic		monophyletic	
Lycaenidae	*Lysandra*	*albicans*	shared barcodes	shared barcodes (L. caelestissima); complex relationship with L. hispana and L. coridon; min. p-dist. under 1% with respect to all three taxa	shared barcodes	shared barcodes (L. caelestissima); complex relationship with L. hispana and L. coridon; min. p-dist. under 1% with respect to all three taxa
Lycaenidae	*Lysandra*	*bellargus*	monophyletic		para-/polyphyletic	paraphyletic with respect to L. coridon, L. albicans, L. hispana and L. caelestissima; min. p-dist. is 0.3%
Lycaenidae	*Lysandra*	*caelestissima*	shared barcodes	shared barcodes (L. albicans). The specimen sharing barcodes is a potential hybrid caelestissima x albicans	shared barcodes	shared barcodes (L. albicans). The specimen sharing barcodes is a potential hybrid caelestissima x albicans

Family	Genus	species	Iberian dataset	Comments Iberian dataset	Full dataset	Comments full dataset
Lycaenidae	*Lysandra*	*coridon*	para-/polyphyletic	complex relationship with L. hispana, L. albicans and L. caelestissima; under 1% divergence with respect to L. hispana and L. albicans	para-/polyphyletic	complex relationship with L. hispana, L. albicans and L. caelestissima; under 1% divergence with respect to L. hispana and L. albicans
Lycaenidae	*Lysandra*	*hispana*	para-/polyphyletic	complex relationship with L. albicans, L. coridon and L. caelestissima; under 1% divergence with respect to L. caelestissima and L. albicans	para-/polyphyletic	complex relationship with L. albicans, L. coridon and L. caelestissima; under 1% divergence with respect to L. caelestissima and L. albicans
Lycaenidae	*Phengaris*	*alcon*	monophyletic		monophyletic	
Lycaenidae	*Phengaris*	*arion*	monophyletic		monophyletic	
Lycaenidae	*Phengaris*	*nausithous*	monophyletic		monophyletic	
Lycaenidae	*Phengaris*	*teleius*	n/a		monophyletic	
Lycaenidae	*Plebejus*	*argus*	monophyletic		monophyletic	
Lycaenidae	*Plebejus*	*argyrognomon*	n/a		monophyletic	min. p-dist. to P. idas is 0.5%
Lycaenidae	*Plebejus*	*idas*	monophyletic		para-/polyphyletic	paraphyletic (P. argyrognomon); min. p-dist. to P. argyrognomon is 0.5%
Lycaenidae	*Polyommatus*	*admetus*	n/a		monophyletic	
Lycaenidae	*Polyommatus*	*amandus*	monophyletic		monophyletic	
Lycaenidae	*Polyommatus*	*celina*	monophyletic		monophyletic	
Lycaenidae	*Polyommatus*	*damon*	monophyletic		monophyletic	
Lycaenidae	*Polyommatus*	*daphnis*	monophyletic		monophyletic	
Lycaenidae	*Polyommatus*	*dolus*	n/a		monophyletic	min. p-dist. to P. fulgens is 0.6%
Lycaenidae	*Polyommatus*	*dorylas*	monophyletic		para-/polyphyletic	paraphyletic (P. golgus); min. p-dist. to P. golgus is 0.8%
Lycaenidae	*Polyommatus*	*eros*	monophyletic		monophyletic	
Lycaenidae	*Polyommatus*	*escheri*	monophyletic		monophyletic	
Lycaenidae	*Polyommatus*	*fabressei*	shared barcodes	shared barcodes (P. fulgens)	shared barcodes	shared barcodes (P. fulgens)
Lycaenidae	*Polyommatus*	*fulgens*	shared barcodes	shared barcodes (P. fabressei)	shared barcodes	shared barcodes (P. fabressei)
Lycaenidae	*Polyommatus*	*golgus*	monophyletic		monophyletic	min. p-dist. to P. dorylas is 0.8%
Lycaenidae	*Polyommatus*	*humedasae*	n/a		monophyletic	
Lycaenidae	*Polyommatus*	*icarus*	monophyletic		monophyletic	
Lycaenidae	*Polyommatus*	*nephohiptamenos*	n/a		monophyletic	singleton; min. p-dist. to P. ripartii is 0.8%

Family	Genus	species	Iberian dataset	Comments Iberian dataset	Full dataset	Comments full dataset
Lycaenidae	*Polyommatus*	*nivescens*	monophyletic		monophyletic	
Lycaenidae	*Polyommatus*	*ripartii*	monophyletic		para-/polyphyletic	paraphyletic (P. admetus, P. nephohiptamenos); min. p-dist. to P. nephohiptamenos is 0.8%
Lycaenidae	*Polyommatus*	*thersites*	monophyletic		monophyletic	
Lycaenidae	*Polyommatus*	*violetae*	monophyletic		monophyletic	
Lycaenidae	*Pseudophilotes*	*abencerragus*	monophyletic		monophyletic	
Lycaenidae	*Pseudophilotes*	*baton*	shared barcodes	shared barcodes (P. panoptes)	shared barcodes	shared barcodes (P. panoptes)
Lycaenidae	*Pseudophilotes*	*bavius*	n/a		monophyletic	
Lycaenidae	*Pseudophilotes*	*panoptes*	shared barcodes	shared barcodes (P. baton)	shared barcodes	shared barcodes (P. baton)
Lycaenidae	*Pseudophilotes*	*vicrama*	n/a		monophyletic	min. p-dist. to P. baton is 0.5% and to P. panoptes is 0.6%
Lycaenidae	*Satyrium*	*acaciae*	monophyletic		monophyletic	
Lycaenidae	*Satyrium*	*esculi*	monophyletic		monophyletic	
Lycaenidae	*Satyrium*	*ilicis*	monophyletic		monophyletic	
Lycaenidae	*Satyrium*	*pruni*	monophyletic		monophyletic	
Lycaenidae	*Satyrium*	*spini*	monophyletic		monophyletic	
Lycaenidae	*Satyrium*	*w-album*	monophyletic		monophyletic	
Lycaenidae	*Scolitantides*	*orion*	monophyletic		monophyletic	
Lycaenidae	*Tarucus*	*theophrastus*	monophyletic		monophyletic	
Lycaenidae	*Thecla*	*betulae*	monophyletic		monophyletic	
Lycaenidae	*Tomares*	*ballus*	monophyletic		monophyletic	
Lycaenidae	*Zizeeria*	*knysna*	monophyletic		monophyletic	
Nymphalidae	*Aglais*	*io*	monophyletic		monophyletic	
Nymphalidae	*Aglais*	*urticae*	monophyletic		monophyletic	
Nymphalidae	*Apatura*	*ilia*	monophyletic		para-/polyphyletic	paraphyletic (A. metis); min. p-dist. to A. metis is 0.2%
Nymphalidae	*Apatura*	*iris*	monophyletic		monophyletic	
Nymphalidae	*Apatura*	*metis*	n/a		monophyletic	min. p-dist. to A. ilia is 0.2%
Nymphalidae	*Aphantopus*	*hyperantus*	monophyletic		monophyletic	
Nymphalidae	*Araschnia*	*levana*	monophyletic		monophyletic	

Family	Genus	species	Iberian dataset	Comments Iberian dataset	Full dataset	Comments full dataset
Nymphalidae	*Arethusana*	*arethusa*	monophyletic		monophyletic	
Nymphalidae	*Argynnis*	*adippe*	monophyletic		monophyletic	
Nymphalidae	*Argynnis*	*aglaja*	monophyletic		monophyletic	
Nymphalidae	*Argynnis*	*laodice*	n/a		monophyletic	
Nymphalidae	*Argynnis*	*niobe*	monophyletic		monophyletic	
Nymphalidae	*Argynnis*	*pandora*	monophyletic		monophyletic	
Nymphalidae	*Argynnis*	*paphia*	monophyletic		monophyletic	
Nymphalidae	*Boloria*	*aquilonaris*	n/a		monophyletic	
Nymphalidae	*Boloria*	*dia*	monophyletic		monophyletic	
Nymphalidae	*Boloria*	*eunomia*	monophyletic		monophyletic	
Nymphalidae	*Boloria*	*euphrosyne*	monophyletic		monophyletic	
Nymphalidae	*Boloria*	*napaea*	n/a		para-/polyphyletic	paraphyletic (B. aquilonaris)
Nymphalidae	*Boloria*	*pales*	monophyletic		monophyletic	
Nymphalidae	*Boloria*	*selene*	monophyletic		monophyletic	
Nymphalidae	*Boloria*	*thore*	n/a		monophyletic	
Nymphalidae	*Boloria*	*titania*	n/a		monophyletic	
Nymphalidae	*Brenthis*	*daphne*	para-/polyphyletic	paraphyletic (B. ino); min. p-dist. to B. ino is 0.6%	para-/polyphyletic	paraphyletic (B. ino); min. p-dist. to B. ino is 0.6%
Nymphalidae	*Brenthis*	*hecate*	monophyletic		monophyletic	
Nymphalidae	*Brenthis*	*ino*	para-/polyphyletic	paraphyletic (B. daphne); min. p-dist. to B. daphne is 0.6%	para-/polyphyletic	paraphyletic (B. daphne); min. p-dist. to B. daphne is 0.6%
Nymphalidae	*Brintesia*	*circe*	monophyletic		monophyletic	
Nymphalidae	*Charaxes*	*jasius*	monophyletic		monophyletic	
Nymphalidae	*Chazara*	*briseis*	monophyletic		monophyletic	
Nymphalidae	*Chazara*	*prieuri*	monophyletic		monophyletic	
Nymphalidae	*Coenonympha*	*arcania*	monophyletic		para-/polyphyletic	paraphyletic (C. leander, C. gardetta)
Nymphalidae	*Coenonympha*	*dorus*	monophyletic		monophyletic	
Nymphalidae	*Coenonympha*	*gardetta*	n/a		monophyletic	singleton; min. p-dist. to C. arcania is 1.1%
Nymphalidae	*Coenonympha*	*glycerion*	monophyletic		monophyletic	
Nymphalidae	*Coenonympha*	*hero*	n/a		monophyletic	
Nymphalidae	*Coenonympha*	*leander*	n/a		monophyletic	

Family	Genus	species	Iberian dataset	Comments Iberian dataset	Full dataset	Comments full dataset
Nymphalidae	*Coenonympha*	*oedippus*	n/a		monophyletic	singleton
Nymphalidae	*Coenonympha*	*pamphilus*	monophyletic		monophyletic	
Nymphalidae	*Coenonympha*	*rhodopensis*	n/a		para-/polyphyletic	paraphyletic (C. tullia); min. p-dist. to C. tullia is 0.3%
Nymphalidae	*Coenonympha*	*tullia*	n/a		monophyletic	min. p-dist. to C. rhodopensis is 0.3%
Nymphalidae	*Danaus*	*chrysippus*	monophyletic		monophyletic	
Nymphalidae	*Danaus*	*plexippus*	monophyletic		monophyletic	
Nymphalidae	*Erebia*	*aethiops*	n/a		monophyletic	
Nymphalidae	*Erebia*	*cassioides*	monophyletic		shared barcodes	shared barcode with E. tyndarus
Nymphalidae	*Erebia*	*epiphron*	monophyletic		monophyletic	
Nymphalidae	*Erebia*	*epistygne*	monophyletic		monophyletic	
Nymphalidae	*Erebia*	*euryale*	monophyletic		monophyletic	min. p-dist. to E. ligea is 0.2%
Nymphalidae	*Erebia*	*gorge*	monophyletic		monophyletic	
Nymphalidae	*Erebia*	*gorgone*	monophyletic		monophyletic	
Nymphalidae	*Erebia*	*hispania*	monophyletic		monophyletic	
Nymphalidae	*Erebia*	*lefebvrei*	monophyletic		monophyletic	
Nymphalidae	*Erebia*	*ligea*	n/a		para-/polyphyletic	paraphyly (E. euryale); min. p-dist. to E. euryale is 0.2%
Nymphalidae	*Erebia*	*manto*	monophyletic		monophyletic	
Nymphalidae	*Erebia*	*medusa*	n/a		shared barcodes	shared barcodes (E. polaris)
Nymphalidae	*Erebia*	*melas*	n/a		monophyletic	
Nymphalidae	*Erebia*	*meolans*	monophyletic		monophyletic	
Nymphalidae	*Erebia*	*neoridas*	para-/polyphyletic	paraphyletic (E. zapateri); min. p-dist. to E. zapateri is 0.2%	para-/polyphyletic	paraphyletic (E. zapateri); min. p-dist. to E. zapateri is 0.2%
Nymphalidae	*Erebia*	*oeme*	monophyletic		monophyletic	
Nymphalidae	*Erebia*	*palarica*	monophyletic		monophyletic	
Nymphalidae	*Erebia*	*pandrose*	monophyletic		para-/polyphyletic	paraphyletic (E. sthennyo); min. p-dist. to E. sthennyo is 0.5%
Nymphalidae	*Erebia*	*pharte*	n/a		monophyletic	
Nymphalidae	*Erebia*	*polaris*	n/a		shared barcodes	shared barcodes (E. medusa)
Nymphalidae	*Erebia*	*pronoe*	monophyletic		para-/polyphyletic	paraphyletic (E. melas)

Family	Genus	species	Iberian dataset	Comments Iberian dataset	Full dataset	Comments full dataset
Nymphalidae	*Erebia*	*rondoui*	monophyletic		monophyletic	
Nymphalidae	*Erebia*	*sthennyo*	monophyletic		monophyletic	min. p-dist. to E. pandrose is 0.5%
Nymphalidae	*Erebia*	*sudetica*	n/a		monophyletic	
Nymphalidae	*Erebia*	*triaria*	monophyletic		monophyletic	
Nymphalidae	*Erebia*	*tyndarus*	n/a		shared barcodes	singleton; shared barcode with E. cassioides
Nymphalidae	*Erebia*	*zapateri*	monophyletic	min. p-dist. to E. neoridas is 0.2%	monophyletic	min. p-dist. to E. neoridas is 0.2%
Nymphalidae	*Euphydryas*	*aurinia*	monophyletic		monophyletic	
Nymphalidae	*Euphydryas*	*cynthia*	n/a		monophyletic	singleton
Nymphalidae	*Euphydryas*	*desfontainii*	monophyletic		monophyletic	
Nymphalidae	*Euphydryas*	*maturna*	n/a		monophyletic	
Nymphalidae	*Hipparchia*	*fagi*	monophyletic		para-/polyphyletic	paraphyletic (H. syriaca); min. p-dist. to H. syriaca is 0.2%
Nymphalidae	*Hipparchia*	*fidia*	monophyletic		monophyletic	
Nymphalidae	*Hipparchia*	*hermione*	monophyletic		para-/polyphyletic	paraphyletic (H. fagi, H. syriaca); min. p-dist. to H. syriaca is 0.6% and to H. fagi 0.8%
Nymphalidae	*Hipparchia*	*semele*	monophyletic		shared barcodes	shared barcode (H. volgensis)
Nymphalidae	*Hipparchia*	*statilinus*	monophyletic		monophyletic	
Nymphalidae	*Hipparchia*	*syriaca*	n/a		monophyletic	min. p-dist. to H. fagi is 0.2%
Nymphalidae	*Hipparchia*	*volgensis*	n/a		shared barcodes	singleton, shared barcode with H. semele
Nymphalidae	*Hyponephele*	*lupinus*	monophyletic		monophyletic	
Nymphalidae	*Hyponephele*	*lycaon*	monophyletic		monophyletic	
Nymphalidae	*Issoria*	*lathonia*	monophyletic		monophyletic	
Nymphalidae	*Kirinia*	*roxelana*	n/a		monophyletic	
Nymphalidae	*Lasiommata*	*maera*	monophyletic		monophyletic	
Nymphalidae	*Lasiommata*	*megera*	monophyletic		monophyletic	
Nymphalidae	*Lasiommata*	*petropolitana*	monophyletic		monophyletic	
Nymphalidae	*Libythea*	*celtis*	monophyletic		monophyletic	
Nymphalidae	*Limenitis*	*camilla*	monophyletic		monophyletic	
Nymphalidae	*Limenitis*	*populi*	n/a		monophyletic	
Nymphalidae	*Limenitis*	*reducta*	monophyletic		monophyletic	
Nymphalidae	*Lopinga*	*achine*	monophyletic		monophyletic	

Family	Genus	species	Iberian dataset	Comments Iberian dataset	Full dataset	Comments full dataset
Nymphalidae	*Maniola*	*jurtina*	monophyletic		monophyletic	
Nymphalidae	*Melanargia*	*galathea*	monophyletic		monophyletic	
Nymphalidae	*Melanargia*	*ines*	monophyletic		monophyletic	
Nymphalidae	*Melanargia*	*lachesis*	monophyletic		monophyletic	
Nymphalidae	*Melanargia*	*occitanica*	monophyletic		monophyletic	
Nymphalidae	*Melanargia*	*russiae*	monophyletic		monophyletic	
Nymphalidae	*Melitaea*	*aetherie*	monophyletic		monophyletic	
Nymphalidae	*Melitaea*	*athalia*	monophyletic		para-/polyphyletic	paraphyletic (M. britomartis)
Nymphalidae	*Melitaea*	*aurelia*	n/a		monophyletic	
Nymphalidae	*Melitaea*	*britomartis*	n/a		monophyletic	
Nymphalidae	*Melitaea*	*cinxia*	monophyletic		monophyletic	
Nymphalidae	*Melitaea*	*deione*	monophyletic		monophyletic	
Nymphalidae	*Melitaea*	*diamina*	monophyletic		monophyletic	
Nymphalidae	*Melitaea*	*didyma*	monophyletic		monophyletic	
Nymphalidae	*Melitaea*	*parthenoides*	monophyletic		monophyletic	
Nymphalidae	*Melitaea*	*phoebe*	monophyletic		monophyletic	
Nymphalidae	*Melitaea*	*trivia*	monophyletic		monophyletic	
Nymphalidae	*Minois*	*dryas*	monophyletic		monophyletic	
Nymphalidae	*Neptis*	*rivularis*	n/a		monophyletic	
Nymphalidae	*Neptis*	*sappho*	n/a		monophyletic	
Nymphalidae	*Nymphalis*	*antiopa*	monophyletic		monophyletic	
Nymphalidae	*Nymphalis*	*polychloros*	monophyletic		monophyletic	
Nymphalidae	*Nymphalis*	*vaualbum*	n/a		monophyletic	singleton
Nymphalidae	*Nymphalis*	*xanthomelas*	n/a		monophyletic	
Nymphalidae	*Oeneis*	*glacialis*	n/a		shared barcodes	shared barcodes (O. norna)
Nymphalidae	*Oeneis*	*norna*	n/a		shared barcodes	shared barcodes (O. glacialis)
Nymphalidae	*Pararge*	*aegeria*	monophyletic		monophyletic	
Nymphalidae	*Polygonia*	*c-album*	monophyletic		monophyletic	
Nymphalidae	*Pseudochazara*	*hippolyte*	monophyletic		monophyletic	

Family	Genus	species	Iberian dataset	Comments Iberian dataset	Full dataset	Comments full dataset
Nymphalidae	*Pyronia*	*bathseba*	monophyletic		monophyletic	
Nymphalidae	*Pyronia*	*cecilia*	monophyletic		monophyletic	
Nymphalidae	*Pyronia*	*tithonus*	monophyletic		monophyletic	
Nymphalidae	*Satyrus*	*actaea*	monophyletic		monophyletic	
Nymphalidae	*Satyrus*	*ferula*	monophyletic		monophyletic	
Nymphalidae	*Vanessa*	*atalanta*	monophyletic		monophyletic	
Nymphalidae	*Vanessa*	*cardui*	monophyletic		monophyletic	
Nymphalidae	*Vanessa*	*virginiensis*	monophyletic		monophyletic	
Papilionidae	*Allancastria*	*cerisyi*	n/a		monophyletic	singleton
Papilionidae	*Iphiclides*	*podalirius*	monophyletic		monophyletic	
Papilionidae	*Papilio*	*machaon*	monophyletic		monophyletic	
Papilionidae	*Parnassius*	*apollo*	monophyletic		monophyletic	
Papilionidae	*Parnassius*	*mnemosyne*	monophyletic		monophyletic	
Papilionidae	*Parnassius*	*phoebus*	n/a		monophyletic	
Papilionidae	*Zerynthia*	*polyxena*	n/a		monophyletic	
Papilionidae	*Zerynthia*	*rumina*	monophyletic		monophyletic	
Pieridae	*Anthocharis*	*cardamines*	monophyletic		monophyletic	
Pieridae	*Anthocharis*	*euphenoides*	monophyletic		monophyletic	
Pieridae	*Aporia*	*crataegi*	monophyletic		monophyletic	
Pieridae	*Colias*	*alfacariensis*	monophyletic		monophyletic	
Pieridae	*Colias*	*chrysotheme*	n/a		monophyletic	
Pieridae	*Colias*	*croceus*	monophyletic		shared barcodes	shared barcodes (C. erate)
Pieridae	*Colias*	*erate*	n/a		shared barcodes	shared barcodes (C. croceus)
Pieridae	*Colias*	*hyale*	n/a		monophyletic	
Pieridae	*Colias*	*myrmidone*	n/a		monophyletic	
Pieridae	*Colias*	*palaeno*	n/a		monophyletic	
Pieridae	*Colias*	*phicomone*	monophyletic		monophyletic	
Pieridae	*Euchloe*	*ausonia*	n/a		monophyletic	
Pieridae	*Euchloe*	*bazae*	monophyletic		monophyletic	
Pieridae	*Euchloe*	*belemia*	monophyletic		monophyletic	

Family	Genus	species	Iberian dataset	Comments Iberian dataset	Full dataset	Comments full dataset
Pieridae	*Euchloe*	*crameri*	monophyletic		monophyletic	
Pieridae	*Euchloe*	*simplonia*	monophyletic		monophyletic	
Pieridae	*Euchloe*	*tagis*	monophyletic		monophyletic	
Pieridae	*Gonepteryx*	*cleopatra*	monophyletic		monophyletic	
Pieridae	*Gonepteryx*	*rhamni*	monophyletic		monophyletic	
Pieridae	*Leptidea*	*duponcheli*	n/a		monophyletic	
Pieridae	*Leptidea*	*juvernica*	n/a		monophyletic	
Pieridae	*Leptidea*	*morsei*	n/a		monophyletic	
Pieridae	*Leptidea*	*reali*	monophyletic		monophyletic	
Pieridae	*Leptidea*	*sinapis*	monophyletic		monophyletic	
Pieridae	*Pieris*	*brassicae*	monophyletic		monophyletic	
Pieridae	*Pieris*	*bryoniae*	n/a		shared barcodes	shared barcodes (P. napi); min. p-dist. to P. napi is 0.2%
Pieridae	*Pieris*	*ergane*	monophyletic		monophyletic	
Pieridae	*Pieris*	*mannii*	monophyletic		monophyletic	
Pieridae	*Pieris*	*napi*	monophyletic		shared barcodes	shared barcodes (P. bryoniae)
Pieridae	*Pieris*	*pseudorapae*	n/a		monophyletic	min. p-dist. to P. napi is 0.2%
Pieridae	*Pieris*	*rapae*	monophyletic		monophyletic	
Pieridae	*Pontia*	*callidice*	monophyletic		monophyletic	
Pieridae	*Pontia*	*daplidice*	monophyletic		monophyletic	
Pieridae	*Pontia*	*edusa*	n/a		monophyletic	
Pieridae	*Zegris*	*eupheme*	monophyletic		monophyletic	
Riodinidae	*Hamearis*	*lucina*	monophyletic		monophyletic	

Supplementary Table 3 | ST-GMYC results and categories used for the 299 species in the dataset. The taxa are ordered according to their appearance in Supplementary Fig. 5.

Family	Genus	Species	Author & year	GMYC Category	GMYC vs. taxonomy match	GMYC entities	Entity name (matches Supplementary Fig. 5)	Comments
Pieridae	*Zegris*	*eupheme*	(Esper, 1804)	SE	yes	1	eupheme	
Pieridae	*Euchloe*	*tagis*	(Hübner, 1804)	ME	yes	2	tagis 1	
Pieridae							tagis 2	
Pieridae	*Euchloe*	*bazae*	Fabiano, 1993	SE	yes	1	bazae	
Pieridae	*Anthocharis*	*cardamines*	(Linnaeus, 1758)	SE	yes	1	cardamines	
Pieridae	*Anthocharis*	*euphenoides*	Staudinger, 1869	SE	yes	1	euphenoides	
Pieridae	*Euchloe*	*belemia*	(Esper, 1800)	ME	yes	2	belemia 1	
Pieridae							belemia 2	
Pieridae	*Euchloe*	*crameri*	Butler, 1869	SE	yes	1	crameri	
Pieridae	*Euchloe*	*simplonia*	(Freyer, 1829)	SE	yes	1	simplonia	
Pieridae	*Euchloe*	*ausonia*	(Hübner, 1804)	SE	yes	1	ausonia	
Pieridae	*Gonepteryx*	*cleopatra*	(Linnaeus, 1767)	SE	yes	1	cleopatra	
Pieridae	*Gonepteryx*	*rhamni*	(Linnaeus, 1758)	ME	yes	3	rhamni 1	
Pieridae							rhamni 2	
Pieridae							rhamni 3	
Pieridae	*Pontia*	*daplidice*	(Linnaeus, 1758)	SE	yes	1	daplidice	
Pieridae	*Pontia*	*edusa*	(Fabricius, 1777)	SE	yes	1	edusa	
Pieridae	*Pontia*	*callidice*	(Hübner, 1800)	SE	yes	1	callidice	
Pieridae	*Pieris*	*brassicae*	(Linnaeus, 1758)	SE	yes	1	brassicae	
Pieridae	*Pieris*	*napi*	(Linnaeus, 1758)	ME; lumped	no	2	bryoniae / napi	one entity clusters with *P. bryoniae*, the other with *P. pseudorapae*
Pieridae							napi / pseudorapae	
Pieridae	*Pieris*	*bryoniae*	(Hübner, 1806)	lumped	no			it clusters with some *P. napi*
Pieridae	*Pieris*	*pseudorapae*	(Vérity, 1908)	lumped	no			it clusters with *P. napi*
Pieridae	*Pieris*	*ergane*	(Geyer, 1828)	SE	yes	1	ergane	
Pieridae	*Pieris*	*mannii*	(Mayer, 1851)	ME	yes	2	mannii 1	
Pieridae							mannii 2	
Pieridae	*Pieris*	*rapae*	(Linnaeus, 1758)	ME	yes	2	rapae 1	
Pieridae							rapae 2	
Pieridae	*Aporia*	*crataegi*	(Linnaeus, 1758)	SE	yes	1	crataegi	
Hesperiidae	*Gegenes*	*nostrodamus*	(Fabricius, 1793)	SE	yes	1	nostrodamus	

Family	Genus	Species	Author & year	GMYC Category	GMYC vs. taxonomy match	GMYC entities	Entity name (matches Supplementary Fig. 5)	Comments
Hesperiidae	*Gegenes*	*pumilio*	(Hoffmansegg, 1804)	SE	yes	1	pumilio	
Hesperiidae	*Borbo*	*borbonica*	(Boisduval, 1833)	SE	yes	1	borbonica	
Hesperiidae	*Ochlodes*	*sylvanus*	(Esper, 1777)	SE	yes	1	sylvanus	
Hesperiidae	*Hesperia*	*comma*	(Linnaeus, 1758)	SE	yes	1	comma	
Hesperiidae	*Thymelicus*	*sylvestris*	(Poda, 1761)	ME	yes	4	sylvestris 1	
Hesperiidae							sylvestris 2	
Hesperiidae							sylvestris 3	
Hesperiidae							sylvestris 4	
Hesperiidae	*Thymelicus*	*lineola*	(Ochsenheimer, 1808)	ME	yes	4	lineola 1	
Hesperiidae							lineola 2	
Hesperiidae							lineola 3	
Hesperiidae							lineola 4	
Hesperiidae	*Thymelicus*	*acteon*	(Rottemburg, 1775)	ME	yes	2	acteon 1	
Hesperiidae							acteon 2	
Hesperiidae	*Carterocephalus*	*palaemon*	(Pallas, 1771)	SE	yes	1	palaemon	
Hesperiidae	*Heteropterus*	*morpheus*	(Pallas, 1771)	SE	yes	1	morpheus	
Hesperiidae	*Pyrgus*	*andromedae*	(Wallengren, 1853)	ME	yes	2	andromedae 1	
Hesperiidae							andromedae 2	
Hesperiidae	*Pyrgus*	*cacaliae*	(Rambur, 1839)	SE	yes	1	cacaliae	
Hesperiidae	*Pyrgus*	*sidae*	(Esper, 1784)	SE	yes	1	sidae	
Hesperiidae	*Pyrgus*	*carthami*	(Hübner, 1813)	SE	yes	1	carthami	
Hesperiidae	*Pyrgus*	*cinarae*	(Rambur, 1839)	ME	yes	2	cinarae1	
Hesperiidae							cinarae2	
Hesperiidae	*Pyrgus*	*onopordi*	(Rambur, 1839)	SE	yes	1	onopordi	
Hesperiidae	*Pyrgus*	*cirsii*	(Rambur, 1839)	SE	yes	1	cirsii	
Hesperiidae	*Pyrgus*	*alveus*	(Hübner, 1803)	ME; lumped	no	2	alveus	one entity clusters with *P. bellieri* and *P. warrenensis*
Hesperiidae							alveus / bellieri / warrenensis	
Hesperiidae	*Pyrgus*	*bellieri*	(Oberthür, 1910)	lumped	no			it clusters with *P. alveus* and *P. warrenensis*
Hesperiidae	*Pyrgus*	*warrenensis*	(Vérity, 1928)	lumped	no			it clusters with *P. alveus* and *P. bellieri*
Hesperiidae	*Pyrgus*	*serratulae*	(Rambur, 1839)	SE	yes	1	serratulae	

Family	Genus	Species	Author & year	GMYC Category	GMYC vs. taxonomy match	GMYC entities	Entity name (matches Supplementary Fig. 5)	Comments
Hesperiidae	*Pyrgus*	*armoricanus*	(Oberthür, 1910)	ME	yes	2	armoricanus 1	
Hesperiidae							armoricanus 2	
Hesperiidae	*Pyrgus*	*malvae*	(Linnaeus, 1758)	SE	yes	1	malvae	
Hesperiidae	*Pyrgus*	*malvoides*	(Elwes y Edwards, 1897)	SE	yes	1	malvoides	
Hesperiidae	*Erynnis*	*tages*	(Linnaeus, 1758)	SE	yes	1	tages	
Papilionidae	*Iphiclides*	*podalirius*	(Linnaeus, 1758)	SE	yes	1	podalirius	
Papilionidae	*Zerynthia*	*polyxena*	(Denis & Schiffermüller, 1775)	SE	yes	1	polyxena	
Papilionidae	*Zerynthia*	*rumina*	(Linnaeus, 1758)	ME	yes	2	rumina 1	
Papilionidae							rumina 2	
Papilionidae	*Allancastria*	*cerisyi*	(Godart, 1824)	SE	yes	1	cerisyi	
Papilionidae	*Papilio*	*machaon*	Linnaeus, 1758	ME	yes	2	machaon 1	
Papilionidae							machaon 2	
Papilionidae	*Parnassius*	*mnemosyne*	(Linnaeus, 1758)	ME	yes	2	mnemosyne 1	
Papilionidae							mnemosyne 2	
Papilionidae	*Parnassius*	*apollo*	(Linnaeus, 1758)	ME	yes	3	apollo 1	
Papilionidae							apollo 2	
Papilionidae							apollo 3	
Papilionidae	*Parnassius*	*phoebus*	(Fabricius, 1793)	SE	yes	1	phoebus	
Nymphalidae	*Lasiommata*	*maera*	(Linnaeus, 1758)	ME	yes	4	maera 1	
Nymphalidae							maera 2	
Nymphalidae							maera 3	
Nymphalidae							maera 4	
Nymphalidae	*Lasiommata*	*megera*	(Linnaeus, 1767)	SE	yes	1	megera	
Nymphalidae	*Lasiommata*	*petropolitana*	(Fabricius, 1787)	SE	yes	1	petropolitana	
Nymphalidae	*Pararge*	*aegeria*	(Linnaeus, 1758)	SE	yes	1	aegeria	
Nymphalidae	*Kirinia*	*roxelana*	(Cramer, 1777)	SE	yes	1	roxelana	
Nymphalidae	*Lopinga*	*achine*	(Scopoli, 1763)	SE	yes	1	achine	
Nymphalidae	*Erebia*	*pharte*	(Hübner, 1804)	SE	yes	1	pharte	
Nymphalidae	*Erebia*	*epiphron*	(Knoch, 1783)	SE	yes	1	epiphron	
Nymphalidae	*Erebia*	*ligea*	(Linnaeus, 1758)	lumped	no	1	euryale / ligea	it clusters together with *E. euryale*
Nymphalidae	*Erebia*	*euryale*	(Esper, 1805)	lumped	no			it clusters together with *E. ligea*
Nymphalidae	*Erebia*	*melas*	(Herbst, 1796)	SE	yes	1	melas	
Nymphalidae	*Erebia*	*pronoe*	(Esper, 1780)	ME	yes	2	pronoe 1	
Nymphalidae							pronoe 2	

Family	Genus	Species	Author & year	GMYC Category	GMYC vs. taxonomy match	GMYC entities	Entity name (matches Supplementary Fig. 5)	Comments
Nymphalidae	*Erebia*	*neoridas*	(Boisduval, 1828)	lumped	no	1	neoridas / zapateri	it clusters together with *E. zapateri*
Nymphalidae	*Erebia*	*zapateri*	Oberthür, 1875	lumped	no			it clusters together with *E. neoridas*
Nymphalidae	*Erebia*	*lefebvrei*	(Boisduval, 1828)	SE	yes	1	lefebvrei	
Nymphalidae	*Erebia*	*manto*	(Denis & Schiffermüller, 1775)	SE	yes	1	manto	
Nymphalidae	*Erebia*	*sudetica*	Staudinger, 1861	SE	yes	1	sudetica	
Nymphalidae	*Erebia*	*gorgone*	(Boisduval, 1833)	SE	yes	1	gorgone	
Nymphalidae	*Erebia*	*triaria*	(de Prunner, 1798)	SE	yes	1	triaria	
Nymphalidae	*Erebia*	*medusa*	(Denis & Schiffermüller, 1775)	lumped	no	1	medusa / polaris	it clusters together with *E. polaris*
Nymphalidae	*Erebia*	*polaris*	Staudinger, 1871	lumped	no			it clusters together with *E. medusa*
Nymphalidae	*Erebia*	*pandrose*	(Borkhausen, 1788)	lumped	no	1	pandrose / sthennyo	it clusters together with *E. sthennyo*
Nymphalidae	*Erebia*	*sthennyo*	Graslin, 1850	lumped	no			it clusters together with *E. pandrose*
Nymphalidae	*Erebia*	*gorge*	(Hübner, 1804)	SE	yes	1	gorge	
Nymphalidae	*Erebia*	*oeme*	(Hübner, 1804)	SE	yes	1	oeme	
Nymphalidae	*Erebia*	*rondoui*	Oberthür, 1908	lumped	no	1	rondoui / tyndarus / cassioides	it clusters together with *E. tyndarus* and *E. cassioides*
Nymphalidae	*Erebia*	*tyndarus*	(Esper, 1781)	lumped	no			it clusters together with *E. rondoui* and *E. cassioides*
Nymphalidae	*Erebia*	*cassioides*	(Reiner & Hochenwarth, 1792)	lumped	no			it clusters together with *E. tyndarus* and *E. rondoui*
Nymphalidae	*Erebia*	*hispania*	Butler, 1868	SE	yes	1	hispania	
Nymphalidae	*Erebia*	*aethiops*	(Esper, 1777)	SE	yes	1	aethiops	
Nymphalidae	*Erebia*	*epistygne*	(Hübner, 1819)	SE	yes	1	epistygne	
Nymphalidae	*Erebia*	*palarica*	Chapman, 1905	lumped	no	1	meolans / palarica	it clusters with *E. meolans*
Nymphalidae	*Erebia*	*meolans*	(de Prunner, 1798)	lumped	no			it clusters with *E. palarica*
Nymphalidae	*Hyponephele*	*lycaon*	(Kühn, 1774)	SE	yes	1	lycaon	
Nymphalidae	*Hyponephele*	*lupinus*	(O. Costa, 1836)	SE	yes	1	lupinus	
Nymphalidae	*Hipparchia*	*fidia*	(Linnaeus, 1767)	SE	yes	1	fidia	
Nymphalidae	*Hipparchia*	*statilinus*	(Hufnagel, 1766)	ME	yes	2	statilinus 1	
Nymphalidae							statilinus 2	
Nymphalidae	*Hipparchia*	*semele*	(Linnaeus, 1758)	ME; lumped	no	2	semele	one entity clusters together with *H. volgensis*
							semele / volgensis	
Nymphalidae	*Hipparchia*	*volgensis*	(Mazochin-Porshnjakov, 1952)	lumped	no			it clusters with some *H. semele*

Family	Genus	Species	Author & year	GMYC Category	GMYC vs. taxonomy match	GMYC entities	Entity name (matches Supplementary Fig. 5)	Comments
Nymphalidae	*Hipparchia*	*fagi*	(Scopoli, 1763)	lumped	no			it clusters with *H. syriaca* and some *H. hermione* (potential taxon *genava*)
Nymphalidae	*Hipparchia*	*syriaca*	(Staudinger, 1871)	lumped	no			it clusters with *H. fagi* and some *H. hermione* (potential taxon *genava*)
Nymphalidae	*Hipparchia*	*hermione*	(Linnaeus, 1764)	ME; lumped	no	2	hermione	one entity clusters with *H. fagi* and *H. syriaca*
							fagi / syriaca / hermione	
Nymphalidae	*Minois*	*dryas*	(Scopoli, 1763)	SE	yes	1	dryas	
Nymphalidae	*Arethusana*	*arethusa*	(Denis & Schiffermüller, 1775)	ME	yes	2	arethusa 1	
Nymphalidae							arethusa 2	
Nymphalidae	*Satyrus*	*actaea*	(Esper, 1781)	SE	yes	1	actaea	
Nymphalidae	*Satyrus*	*ferula*	(Fabricius, 1793)	SE	yes	1	ferula	
Nymphalidae	*Chazara*	*briseis*	(Linnaeus, 1764)	SE	yes	1	briseis	
Nymphalidae	*Chazara*	*prieuri*	(Pierret, 1837)	SE	yes	1	prieuri	
Nymphalidae	*Pseudochazara*	*hippolyte*	(Esper, 1783)	SE	yes	1	hippolyte	
Nymphalidae	*Brintesia*	*circe*	(Fabricius, 1775)	SE	yes	1	circe	
Nymphalidae	*Oeneis*	*glacialis*	(Moll, 1783)	lumped	no	1	glacialis / norna	it clusters together with *O. norna*
Nymphalidae	*Oeneis*	*norna*	(Thunberg, 1791)	lumped	no			it clusters together with *O. glacialis*
Nymphalidae	*Melanargia*	*occitanica*	(Esper, 1789)	SE	yes	1	occitanica	
Nymphalidae	*Melanargia*	*ines*	(Hoffmansegg, 1804)	SE	yes	1	ines	
Nymphalidae	*Melanargia*	*russiae*	(Esper, 1783)	SE	yes	1	russiae	
Nymphalidae	*Melanargia*	*galathea*	(Linnaeus, 1758)	lumped	no	1	galathea / lachesis	it clusters together with *M. lachesis*
Nymphalidae	*Melanargia*	*lachesis*	(Hübner, 1790)	lumped	no			it clusters together with *M. galathea*
Nymphalidae	*Pyronia*	*tithonus*	(Linnaeus, 1771)	SE	yes	1	tithonus	
Nymphalidae	*Aphantopus*	*hyperantus*	(Linnaeus, 1758)	ME	yes	2	hyperantus 1	
Nymphalidae							hyperantus 2	
Nymphalidae	*Pyronia*	*bathseba*	(Fabricius, 1793)	SE	yes	1	bathseba	
Nymphalidae	*Maniola*	*jurtina*	(Linnaeus, 1758)	ME	yes	2	jurtina 1	
Nymphalidae							jurtina 2	
Nymphalidae	*Pyronia*	*cecilia*	(Vallantin, 1894)	ME	yes	2	cecilia 1	
Nymphalidae							cecilia 2	
Nymphalidae	*Coenonympha*	*glycerion*	(Borkhausen, 1788)	SE	yes	1	glycerion	

**Family**	**Genus**	**Species**	**Author & year**	**GMYC Category**	**GMYC vs. taxonomy match**	**GMYC entities**	**Entity name (matches Supplementary Fig. 5)**	**Comments**
Nymphalidae	*Coenonympha*	*hero*	(Linnaeus, 1761)	SE	yes	1	hero	
Nymphalidae	*Coenonympha*	*gardetta*	(de Prunner, 1798)	lumped	no			it clusters together with *C. leander* and *C. arcania*
Nymphalidae	*Coenonympha*	*leander*	(Esper, 1784)	lumped	no			it clusters together with *C. gardetta* and *C. arcania*
Nymphalidae	*Coenonympha*	*arcania*	(Linnaeus, 1761)	ME; lumped	no	2	arcania	one entity clusters together with *C. leander* and *C. gardetta*
							arcania / leander / gardetta	
Nymphalidae	*Coenonympha*	*dorus*	(Esper, 1782)	ME	yes	2	dorus 1	
Nymphalidae							dorus 2	
Nymphalidae	*Coenonympha*	*tullia*	(Muller, 1764)	lumped	no	1	tullia / rhodopensis	it clusters together with *C. rhodopensis*
Nymphalidae	*Coenonympha*	*rhodopensis*	Elwes, 1900	lumped	no			it clusters together with *C. tullia*
Nymphalidae	*Coenonympha*	*oedippus*	(Fabricius, 1787)	SE	yes	1	oedippus	
Nymphalidae	*Coenonympha*	*pamphilus*	(Linnaeus, 1758)	ME	yes	2	pamphilus 1	
Nymphalidae							pamphilus 2	
Nymphalidae	*Libythea*	*celtis*	(Laicharting, 1782)	SE	yes	1	celtis	
Nymphalidae	*Charaxes*	*jasius*	(Linnaeus, 1767)	SE	yes	1	jasius	
Nymphalidae	*Neptis*	*sappho*	(Pallas, 1771)	SE	yes	1	sappho	
Nymphalidae	*Neptis*	*rivularis*	(Scopoli, 1763)	SE	yes	1	rivularis	
Nymphalidae	*Boloria*	*napaea*	(Denis & Schiffermüller, 1775)	ME	yes	2	napaea 1	
Nymphalidae							napaea 2	
Nymphalidae	*Boloria*	*pales*	(Hoffmansegg, 1804)	ME	yes	2	pales 1	
Nymphalidae							pales 2	
Nymphalidae	*Boloria*	*aquilonaris*	(Stichel, 1908)	SE	yes	1	aquilonaris	
Nymphalidae	*Boloria*	*eunomia*	(Esper, 1800)	SE	yes	1	eunomia	
Nymphalidae	*Boloria*	*selene*	(Denis & Schiffermüller, 1775)	SE	yes	1	selene	
Nymphalidae	*Boloria*	*thore*	(Hübner, 1803)	ME	yes	2	thore 1	
Nymphalidae							thore 2	
Nymphalidae	*Boloria*	*titania*	(Esper, 1793)	SE	yes	1	titania	
Nymphalidae	*Boloria*	*euphrosyne*	(Linnaeus, 1758)	ME	yes	3	euphrosyne 1	
Nymphalidae							euphrosyne 2	
Nymphalidae							euphrosyne 3	

Family	Genus	Species	Author & year	GMYC Category	GMYC vs. taxonomy match	GMYC entities	Entity name (matches Supplementary Fig. 5)	Comments
Nymphalidae	*Boloria*	*dia*	(Linnaeus, 1767)	SE	yes	1	dia	
Nymphalidae	*Argynnis*	*pandora*	(Denis & Schiffermüller, 1775)	ME	yes	2	pandora 1	
Nymphalidae							pandora 2	
Nymphalidae	*Argynnis*	*laodice*	(Pallas, 1771)	SE	yes	1	laodice	
Nymphalidae	*Argynnis*	*aglaja*	(Linnaeus, 1758)	ME	yes	2	aglaja 1	
Nymphalidae							aglaja 2	
Nymphalidae	*Argynnis*	*adippe*	(Denis & Schiffermüller, 1775)	ME	yes	2	adippe 1	
Nymphalidae							adippe 2	
Nymphalidae	*Argynnis*	*niobe*	(Linnaeus, 1758)	SE	yes	1	niobe	
Nymphalidae	*Argynnis*	*paphia*	(Linnaeus, 1758)	SE	yes	1	paphia	
Nymphalidae	*Issoria*	*lathonia*	(Linnaeus, 1758)	SE	yes	1	lathonia	
Nymphalidae	*Brenthis*	*daphne*	(Bergsträsser, 1780)	ME; lumped	no	3	daphne	
Nymphalidae							daphne / ino	one entity consists of both specimens of *B. daphne* and *B. ino*
Nymphalidae	*Brenthis*	*ino*	(Rottemburg, 1775)	ME; lumped	no		ino	
Nymphalidae	*Brenthis*	*hecate*	(Denis & Schiffermüller, 1775)	ME	yes	2	hecate 1	
Nymphalidae							hecate 2	
Nymphalidae	*Araschnia*	*levana*	(Linnaeus, 1758)	SE	yes	1	levana	
Nymphalidae	*Vanessa*	*atalanta*	(Linnaeus, 1758)	SE	yes	1	atalanta	
Nymphalidae	*Vanessa*	*virginiensis*	(Drury, 1773)	SE	yes	1	virginiensis	
Nymphalidae	*Vanessa*	*cardui*	(Linnaeus, 1758)	SE	yes	1	cardui	
Nymphalidae	*Polygonia*	*c-album*	(Linnaeus, 1758)	SE	yes	1	c-album	
Nymphalidae	*Aglais*	*io*	(Linnaeus, 1758)	SE	yes	1	io	
Nymphalidae	*Nymphalis*	*xanthomelas*	(Esper, 1781)	SE	yes	1	xanthomelas	
Nymphalidae	*Nymphalis*	*antiopa*	(Linnaeus, 1758)	SE	yes	1	antiopa	
Nymphalidae	*Nymphalis*	*polychloros*	(Linnaeus, 1758)	ME	yes	2	polychloros 1	
Nymphalidae							polychloros 2	
Nymphalidae	*Nymphalis*	*vaualbum*	(Denis & Schiffermüller, 1775)	SE	yes	1	vaualbum	
Nymphalidae	*Aglais*	*urticae*	(Linnaeus, 1758)	ME	yes	2	urticae 1	
Nymphalidae							urticae 2	
Nymphalidae	*Apatura*	*iris*	(Linnaeus, 1758)	SE	yes	1	iris	
Nymphalidae	*Apatura*	*ilia*	(Denis & Schiffermüller, 1775)	lumped	no	1	ilia / metis	it clusters with *A. metis*
Nymphalidae	*Apatura*	*metis*	Freyer, 1829	lumped	no			it clusters with *A. ilia*

Family	Genus	Species	Author & year	GMYC Category	GMYC vs. taxonomy match	GMYC entities	Entity name (matches Supplementary Fig. 5)	Comments
Nymphalidae	*Danaus*	*plexippus*	(Linnaeus, 1758)	SE	yes	1	plexippus	
Nymphalidae	*Danaus*	*chrysippus*	(Linnaeus, 1758)	SE	yes	1	chrysippus	
Nymphalidae	*Euphydryas*	*maturna*	(Linnaeus, 1758)	SE	yes	1	maturna	
Nymphalidae	*Euphydryas*	*cynthia*	(Denis & Schiffermüller, 1775)	SE	yes	1	cynthia	
Nymphalidae	*Euphydryas*	*desfontainii*	(Godart, 1819)	SE	yes	1	desfontainii	
Nymphalidae	*Euphydryas*	*aurinia*	(Rottemburg, 1775)	SE	yes	1	aurinia	
Nymphalidae	*Melitaea*	*cinxia*	(Linnaeus, 1758)	ME	yes	2	cinxia 1	
Nymphalidae							cinxia 2	
Nymphalidae	*Melitaea*	*phoebe*	(Denis & Schiffermüller, 1775)	SE	yes	1	phoebe	
Nymphalidae	*Melitaea*	*aetherie*	(Hübner, 1826)	SE	yes	1	aetherie	
Nymphalidae	*Melitaea*	*trivia*	(Denis & Schiffermüller, 1775)	ME	yes	2	trivia 1	
Nymphalidae							trivia 2	
Nymphalidae	*Melitaea*	*didyma*	(Esper, 1778)	ME	yes	4	didyma 1	
Nymphalidae							didyma 2	
Nymphalidae							didyma 3	
Nymphalidae							didyma 4	
Nymphalidae	*Melitaea*	*diamina*	(H.G. Lang, 1789)	ME	yes	2	diamina 1	
Nymphalidae							diamina 2	
Nymphalidae	*Melitaea*	*aurelia*	Nickerl, 1850	ME	yes	2	aurelia 1	
Nymphalidae							aurelia 2	
Nymphalidae	*Melitaea*	*parthenoides*	Keferstein, 1851	ME	yes	2	parthenoides 1	
Nymphalidae							parthenoides 2	
Nymphalidae	*Melitaea*	*deione*	(Geyer, 1832)	ME	yes	3	deione 1	
Nymphalidae							deione 2	
Nymphalidae							deione 3	
Nymphalidae	*Melitaea*	*britomartis*	Assmann, 1847	SE	yes	1	britomartis	
Nymphalidae	*Melitaea*	*athalia*	(Rottemburg, 1775)	ME	yes	3	athalia 1	
Nymphalidae							athalia 2	
Nymphalidae							athalia 3	
Nymphalidae	*Limenitis*	*reducta*	Staudinger, 1901	SE	yes	1	reducta	
Nymphalidae	*Limenitis*	*populi*	(Linnaeus, 1758)	SE	yes	1	populi	
Nymphalidae	*Limenitis*	*camilla*	(Linnaeus, 1764)	ME	yes	2	camilla 1	
Nymphalidae							camilla 2	
Lycaenidae	*Favonius*	*quercus*	(Linnaeus, 1758)	ME	yes	2	quercus 1	

Family	Genus	Species	Author & year	GMYC Category	GMYC vs. taxonomy match	GMYC entities	Entity name (matches Supplementary Fig. 5)	Comments
Lycaenidae							quercus 2	
Lycaenidae	*Thecla*	*betulae*	(Linnaeus, 1758)	SE	yes	1	betulae	
Lycaenidae	*Cupido*	*lorquinii*	(Herrich-Schäffer, 1850)	ME; lumped	no	3	lorquinii	
Lycaenidae							lorquinii / minimus	one entity lumps *C. minimus* (taxon *carswelli*) with specimens of *C. lorquinii*
Lycaenidae	*Cupido*	*minimus*	(Fuessly, 1775)	ME; lumped	no		minimus	
Lycaenidae	*Cupido*	*osiris*	(Meigen, 1829)	ME	yes	3	osiris 1	
Lycaenidae							osiris 2	
Lycaenidae							osiris 3	
Lycaenidae	*Cupido*	*argiades*	(Pallas, 1771)	SE	yes	1	argiades	
Lycaenidae	*Cupido*	*decolorata*	(Staudinger, 1886)	lumped	no		decolorata / alcetas	it clusters with some *C. alcetas*
Lycaenidae	*Cupido*	*alcetas*	(Hoffmansegg, 1804)	ME; lumped	no	2	alcetas	one entity clusters with *C. decolorata*
Lycaenidae	*Tomares*	*ballus*	(Fabricius, 1787)	SE	yes	1	ballus	
Lycaenidae	*Callophrys*	*avis*	Chapman, 1909	SE	yes	1	avis	
Lycaenidae	*Callophrys*	*rubi*	(Linnaeus, 1758)	SE	yes	1	rubi	
Lycaenidae	*Laeosopis*	*roboris*	(Esper, 1789)	SE	yes	1	roboris	
Lycaenidae	*Satyrium*	*pruni*	(Linnaeus, 1758)	SE	yes	1	pruni	
Lycaenidae	*Satyrium*	*spini*	(Fabricius, 1787)	ME	yes	3	spini 1	
Lycaenidae							spini 2	
Lycaenidae							spini 3	
Lycaenidae	*Satyrium*	*acaciae*	(Fabricius, 1787)	SE	yes	1	acaciae	
Lycaenidae	*Satyrium*	*w-album*	(Knoch, 1782)	SE	yes	1	w-album	
Lycaenidae	*Satyrium*	*esculi*	(Hübner, 1804)	SE	yes	1	esculi	
Lycaenidae	*Satyrium*	*ilicis*	(Esper, 1779)	SE	yes	1	ilicis	
Lycaenidae	*Tarucus*	*theophrastus*	(Fabricius, 1793)	SE	yes	1	theophrastus	
Lycaenidae	*Azanus*	*jesous*	(Guérin-Méneville, 1849)	SE	yes	1	jesous	
Lycaenidae	*Cacyreus*	*marshalli*	Butler, 1898	SE	yes	1	marshalli	
Lycaenidae	*Celastrina*	*argiolus*	(Linnaeus, 1758)	SE	yes	1	argiolus	
Lycaenidae	*Phengaris*	*arion*	(Linnaeus, 1758)	SE	yes	1	arion	
Lycaenidae	*Phengaris*	*teleius*	(Bergsträsser, 1779)	SE	yes	1	teleius	
Lycaenidae	*Phengaris*	*nausithous*	(Bergsträsser, 1779)	SE	yes	1	nausithous	
Lycaenidae	*Phengaris*	*alcon*	(Denis & Schiffermüller, 1775)	SE	yes	1	alcon	

Family	Genus	Species	Author & year	GMYC Category	GMYC vs. taxonomy match	GMYC entities	Entity name (matches Supplementary Fig. 5)	Comments
Lycaenidae	*Scolitantides*	*orion*	(Pallas, 1771)	ME	yes	2	orion 1	
Lycaenidae							orion 2	
Lycaenidae	*Iolana*	*debilitata*	(Schultz, 1905)	SE	yes	1	debilitata	
Lycaenidae	*Pseudophilotes*	*panoptes*	(Hübner, 1813)	ME; lumped	no	2	panoptes / vicrama	one entity clusters together with *P. vicrama* and the other with *P. baton*
Lycaenidae	*Pseudophilotes*	*vicrama*	(Moore, 1865)	lumped	no			it clusters together with some *P. panoptes*
Lycaenidae	*Pseudophilotes*	*baton*	(Bergsträsser, 1779)	lumped	no		baton / panoptes	it clusters together with some *P. panoptes*
Lycaenidae	*Pseudophilotes*	*abencerragus*	(Pierret, 1837)	SE	yes	1	abencerragus	
Lycaenidae	*Pseudophilotes*	*bavius*	(Eversmann, 1832)	SE	yes	1	bavius	
Lycaenidae	*Glaucopsyche*	*melanops*	(Boisduval, 1828)	SE	yes	1	melanops	
Lycaenidae	*Glaucopsyche*	*alexis*	(Poda, 1761)	ME	yes	2	alexis 1	
Lycaenidae							alexis 2	
Lycaenidae	*Lampides*	*boeticus*	(Linnaeus, 1767)	SE	yes	1	boeticus	
Lycaenidae	*Plebejus*	*argus*	(Linnaeus, 1758)	ME	yes	4	argus 1	
Lycaenidae							argus 2	
Lycaenidae							argus 3	
Lycaenidae							argus 4	
Lycaenidae	*Plebejus*	*argyrognomon*	(Bergsträsser, 1779)	lumped	no			it clusters together with some *P. idas*
Lycaenidae	*Plebejus*	*idas*	(Linnaeus, 1761)	ME; lumped	no	3	idas / argyrognomon	3 entities, one clusters together with *P. argyrognomon*
Lycaenidae							idas 1	
							idas 2	
Lycaenidae	*Polyommatus*	*amandus*	(Schneider, 1792)	ME	yes	2	amandus 1	
Lycaenidae							amandus 2	
Lycaenidae	*Polyommatus*	*celina*	(Austaut, 1879)	ME	yes	2	celina 1	
Lycaenidae							celina 2	
Lycaenidae	*Polyommatus*	*escheri*	(Hübner, 1823)	ME	yes	2	escheri 1	
Lycaenidae							escheri 2	
Lycaenidae	*Polyommatus*	*daphnis*	(Denis & Schiffermüller, 1775)	ME	yes	2	daphnis 1	
Lycaenidae							daphnis 2	

Family	Genus	Species	Author & year	GMYC Category	GMYC vs. taxonomy match	GMYC entities	Entity name (matches Supplementary Fig. 5)	Comments
Lycaenidae	*Polyommatus*	*thersites*	(Cantener, 1835)	ME	yes	2	thersites 1	
Lycaenidae							thersites 2	
Lycaenidae	*Polyommatus*	*violetae*	(Gómez Bustillo, Expósito & Martínez, 1979)	ME	yes	2	violetae 1	
Lycaenidae							violetae 2	
Lycaenidae	*Polyommatus*	*dolus*	(Hübner, 1823)	lumped	no	1	dolus / fabressei / fulgens	it clusters together with *P. fabressei* and *P. fulgens*
Lycaenidae	*Polyommatus*	*fabressei*	(Oberthür, 1910)	lumped	no			it clusters together with *P. dolus* and *P. fulgens*
Lycaenidae	*Polyommatus*	*fulgens*	(Sagarra, 1925)	lumped	no			it clusters together with *P. fabressei* and *P. dolus*
Lycaenidae	*Polyommatus*	*humedasae*	Toso & Balletto, 1976	SE	yes	1	humedasae	
Lycaenidae	*Polyommatus*	*golgus*	(Hübner, 1813)	lumped	no	1	dorylas / golgus	it clusters together with *P. dorylas*
Lycaenidae	*Polyommatus*	*dorylas*	(Denis & Schiffermüller, 1775)	lumped	no			it clusters together with *P. golgus*
Lycaenidae	*Polyommatus*	*nivescens*	(Keferstein, 1851)	SE	yes	1	nivescens	
Lycaenidae	*Polyommatus*	*damon*	(Denis & Schiffermüller, 1775)	ME	yes	2	damon 1	
Lycaenidae							damon 2	
Lycaenidae	*Polyommatus*	*ripartii*	(Freyer, 1830)	ME; lumped	no	2	ripartii / admetus / nephohiptamenos	one entity clusters together with *P. admetus* and *P. nephohiptamenos*
							ripartii	
Lycaenidae	*Polyommatus*	*admetus*	(Esper, 1783)	lumped	no			it clusters together with *P. ripartii* and *P. nephohiptamenos*
Lycaenidae	*Polyommatus*	*nephohiptamenos*	(Brown & Coutsis, 1978)	lumped	no			it clusters together with *P. ripartii* and *P. admetus*
Lycaenidae	*Polyommatus*	*icarus*	(Rottemburg, 1775)	ME	yes	3	icarus 1	
Lycaenidae							icarus 2	
Lycaenidae							icarus 3	
Lycaenidae	*Polyommatus*	*eros*	(Ochsenheimer, 1808)	SE	yes	1	eros	
Lycaenidae	*Lysandra*	*bellargus*	(Rottemburg, 1775)	ME; lumped	no	5	bellargus	2 entities, one clusters together with *L. coridon* and *L. albicans*
Lycaenidae	*Lysandra*	*coridon*	(Poda, 1761)	ME; lumped	no		coridon / bellargus / albicans	3 entities, one clusters together with some *L. bellargus* and *L. albicans*

Family	Genus	Species	Author & year	GMYC Category	GMYC vs. taxonomy match	GMYC entities	Entity name (matches Supplementary Fig. 5)	Comments
Lycaenidae							coridon / hispana / albicans 1	another one clusters together with some *L. hispana* and *L. albicans*
Lycaenidae							coridon / hispana / albicans 2	another one clusters together with some *L. hispana* and *L. albicans*
Lycaenidae	*Lysandra*	*albicans*	(Gerhard, 1851)	ME; lumped	no		albicans / hispana / caelestissima	4 entities, one clusters with some *L. coridon* and *L. bellargus*
Lycaenidae								one of them clusters with some *L. hispana* and *L. coridon*
Lycaenidae								one of them clusters with some *L. hispana* and *L. caelestissima*
Lycaenidae								one of them clusters with some *L. hispana* and *L. coridon*
Lycaenidae	*Lysandra*	*hispana*	(Herrich-Schäffer, 1851)	ME; lumped	no			3 entities, one clusters with some *L. albicans* and *L. coridon*
Lycaenidae								one clusters with some *L. albicans* and *L. caelestissima*
Lycaenidae								one clusters with some *L. albicans* and *L. coridon*
Lycaenidae	*Lysandra*	*caelestissima*	(Vérity, 1921)	lumped	no			it clusters with some *L. hispana* and *L. albicans*
Lycaenidae	*Aricia*	*morronensis*	(Ribbe, 1910)	ME	yes	2	morronensis 1	
Lycaenidae							morronensis 2	
Lycaenidae	*Aricia*	*anteros*	(Freyer, 1838)	SE	yes	1	anteros	
Lycaenidae	*Aricia*	*nicias*	(Meigen, 1830)	ME	yes	2	nicias 1	
Lycaenidae							nicias 2	
Lycaenidae	*Aricia*	*artaxerxes*	(Fabricius, 1793)	SE	yes	1	artaxerxes	
Lycaenidae	*Aricia*	*montensis*	Vérity, 1928	SE	yes	1	montensis	
Lycaenidae	*Aricia*	*agestis*	(Denis & Schiffermüller, 1775)	ME	yes	2	agestis 1	
Lycaenidae							agestis 2	
Lycaenidae	*Aricia*	*cramera*	(Eschscholtz, 1821)	SE	yes	1	cramera	
Lycaenidae	*Agriades*	*pyrenaicus*	(Boisduval, 1840)	SE	yes	1	pyrenaicus	
Lycaenidae	*Agriades*	*glandon*	(de Prunner, 1798)	SE	yes	1	glandon	
Lycaenidae	*Agriades*	*zullichi*	Hemming, 1933	SE	yes	1	zullichi	
Lycaenidae	*Agriades*	*optilete*	(Knoch, 1781)	SE	yes	1	optilete	

Family	Genus	Species	Author & year	GMYC Category	GMYC vs. taxonomy match	GMYC entities	Entity name (matches Supplementary Fig. 5)	Comments
Lycaenidae	*Cyaniris*	*semiargus*	(Rottemburg, 1775)	ME	yes	2	semiargus 1	
Lycaenidae							semiargus 2	
Lycaenidae	*Eumedonia*	*eumedon*	(Esper, 1780)	ME	yes	3	eumedon 1	
Lycaenidae							eumedon 2	
Lycaenidae							eumedon 3	
Lycaenidae	*Kretania*	*pylaon*	(Fischer von Waldheim, 1832)	lumped	no	1	pylaon / sephirus	it clusters together with *K. sephirus*
Lycaenidae	*Kretania*	*sephirus*	(Frivaldzky, 1835)	lumped	no			it clusters together with *K. pylaon*
Lycaenidae	*Kretania*	*hesperica*	(Rambur, 1839)	SE	yes	1	hesperica	
Lycaenidae	*Zizeeria*	*knysna*	(Trimen, 1862)	SE	yes	1	knysna	
Lycaenidae	*Leptotes*	*pirithous*	(Linnaeus, 1767)	SE	yes	1	pirithous	
Lycaenidae	*Lycaena*	*phlaeas*	(Linnaeus, 1761)	SE	yes	1	phlaeas	
Lycaenidae	*Lycaena*	*dispar*	(Haworth, 1802)	SE	yes	1	dispar	
Lycaenidae	*Lycaena*	*hippothoe*	(Linnaeus, 1761)	SE	yes	1	hippothoe	
Lycaenidae	*Lycaena*	*virgaureae*	(Linnaeus, 1758)	SE	yes	1	virgaureae	
Lycaenidae	*Lycaena*	*tityrus*	(Poda, 1761)	SE	yes	1	tityrus	
Lycaenidae	*Lycaena*	*bleusei*	(Oberthür, 1884)	SE	yes	1	bleusei	
Lycaenidae	*Lycaena*	*thersamon*	(Esper, 1784)	SE	yes	1	thersamon	
Lycaenidae	*Lycaena*	*alciphron*	(Rottemburg, 1775)	ME	yes	2	alciphron 1	
Lycaenidae							alciphron 2	
Lycaenidae	*Lycaena*	*helle*	(Denis & Schiffermüller, 1775)	SE	yes	1	helle	
Riodinidae	*Hamearis*	*lucina*	(Linnaeus, 1758)	SE	yes	1	lucina	
Hesperiidae	*Spialia*	*orbifer*	(Hübner, 1823)	lumped	no		sertorius / orbifer	it clusters together with some *S. sertorius*
Hesperiidae	*Spialia*	*sertorius*	(Hoffmansegg, 1804)	ME; lumped	no	2	sertorius	one entity clusters together with *S. orbifer*
Hesperiidae	*Carcharodus*	*alceae*	(Esper, 1780)	ME	yes	2	alceae 1	
Hesperiidae							alceae 2	
Hesperiidae	*Carcharodus*	*tripolina*	(Vérity, 1925)	SE	yes	1	tripolina	
Hesperiidae	*Carcharodus*	*lavatherae*	(Esper, 1783)	ME	yes	2	lavatherae 1	
							lavatherae 2	
Hesperiidae	*Carcharodus*	*baeticus*	(Rambur, 1839)	SE	yes	1	baeticus	
Hesperiidae	*Carcharodus*	*floccifera*	(Zeller, 1847)	lumped	no	1	floccifera / orientalis	it clusters together with *C. orientalis*
Hesperiidae	*Carcharodus*	*orientalis*	Reverdin, 1913	lumped	no			it clusters together with *C. floccifera*

Family	Genus	Species	Author & year	GMYC Category	GMYC vs. taxonomy match	GMYC entities	Entity name (matches Supplementary Fig. 5)	Comments
Hesperiidae	*Muschampia*	*tessellum*	(Hübner, 1803)	SE	yes	1	tessellum	
Hesperiidae	*Muschampia*	*proto*	(Ochsenheimer, 1808)	SE	yes	1	proto	
Hesperiidae	*Muschampia*	*cribrellum*	(Eversmann, 1841)	SE	yes	1	cribrellum	
Pieridae	*Leptidea*	*juvernica*	(Williams, 1946)	SE	yes	1	juvernica	
Pieridae	*Leptidea*	*reali*	Reissinger, 1990	SE	yes	1	reali	
Pieridae	*Leptidea*	*sinapis*	(Linnaeus, 1758)	SE	yes	1	sinapis	
Pieridae	*Leptidea*	*morsei*	(Fenton, 1882)	SE	yes	1	morsei	
Pieridae	*Leptidea*	*duponcheli*	(Staudinger, 1871)	SE	yes	1	duponcheli	
Pieridae	*Colias*	*phicomone*	(Esper, 1780)	SE	yes	1	phicomone	
Pieridae	*Colias*	*palaeno*	(Linnaeus, 1761)	SE	yes	1	palaeno	
Pieridae	*Colias*	*myrmidone*	(Esper, 1781)	SE	yes	1	myrmidone	
Pieridae	*Colias*	*croceus*	(Geoffroy, 1785)	lumped	no	1	croceus / erate	it clusters together with *C. erate*
Pieridae	*Colias*	*erate*	(Esper, 1805)	lumped	no			it clusters together with *C. croceus*
Pieridae	*Colias*	*chrysotheme*	(Esper, 1781)	SE	yes	1	chrysotheme	
Pieridae	*Colias*	*alfacariensis*	Ribbe, 1905	SE	yes	1	alfacariensis	
Pieridae	*Colias*	*hyale*	(Linnaeus, 1758)	SE	yes	1	hyale	

Supplementary Table 4 | Species involving multiple GMYC entities (ME), ST and MM-GMYC supports for entity differentiation, and minimum p-distance (%) among conspecific entities.

Family	Genus	species	Status according to the ST-GMYC analysis	No. of entities for each ME case	GMYC entities involved	ST-GMYC support for entity differentiation	MM-GMYC support for entity differentiation	Minimum p-distance (%) to the nearest conspecific entity or entity containing conspecifics
Pieridae	*Euchloe*	*tagis*	ME	2	tagis 1	0.98	0.97	1.2
Pieridae					tagis 2	0.98	0.97	1.2
Pieridae	*Euchloe*	*belemia*	ME	2	belemia 1	0.91	0.89	1.2
Pieridae					belemia 2	0.91	0.89	1.2
Pieridae	*Gonepteryx*	*rhamni*	ME	3	rhamni 1	0.51	0.46	0.6
Pieridae					rhamni 2	0.51	0.46	0.6
Pieridae					rhamni 3	0.97	0.96	0.6
Pieridae	*Pieris*	*napi*	ME; lumped	2	bryoniae/napi	1	1	1.8
Pieridae					napi/pseudorapae	1	1	1.8
Pieridae	*Pieris*	*mannii*	ME	2	mannii 1	1	1	1.7
Pieridae					mannii 2	1	1	1.7
Pieridae	*Pieris*	*rapae*	ME	2	rapae 1	0.83	0.81	0.7
Pieridae					rapae 2	0.83	0.81	0.7
Hesperiidae	*Thymelicus*	*sylvestris*	ME	4	sylvestris 1	1	1	2.4
Hesperiidae					sylvestris 2	1	1	2.3
Hesperiidae					sylvestris 3	1	1	1.7
Hesperiidae					sylvestris 4	1	1	1.7
Hesperiidae	*Thymelicus*	*lineola*	ME	4	lineola 1	1	1	3.8
Hesperiidae					lineola 2	1	1	2.6
Hesperiidae					lineola 3	1	1	2.4
Hesperiidae					lineola 4	1	1	2.4
Hesperiidae	*Thymelicus*	*acteon*	ME	2	acteon 1	1	1	1.1
Hesperiidae					acteon 2	1	1	1.1
Hesperiidae	*Pyrgus*	*andromedae*	ME	2	andromedae 1	1	0.99	0.6
Hesperiidae					andromedae 2	1	0.99	0.6
Hesperiidae	*Pyrgus*	*cinarae*	ME	2	cinarae1	1	1	3.1
Hesperiidae					cinarae2	1	1	3.1
Hesperiidae	*Pyrgus*	*alveus*	ME; lumped	2	alveus	0.33	0.33	0.5
Hesperiidae					alveus/bellieri/warrenensis	0.33	0.33	0.5

Family	Genus	species	Status according to the ST-GMYC analysis	No. of entities for each ME case	GMYC entities involved	ST-GMYC support for entity differentiation	MM-GMYC support for entity differentiation	Minimum p-distance (%) to the nearest conspecific entity or entity containing conspecifics
Hesperiidae	*Pyrgus*	*armoricanus*	ME	2	armoricanus 1	1	1	2.6
Hesperiidae					armoricanus 2	1	1	2.6
Papilionidae	*Zerynthia*	*rumina*	ME	2	rumina 1	0.46	0.46	1.2
Papilionidae					rumina 2	0.46	0.46	1.2
Papilionidae	*Papilio*	*machaon*	ME	2	machaon 1	1	1	0.8
Papilionidae					machaon 2	1	1	0.8
Papilionidae	*Parnassius*	*mnemosyne*	ME	2	mnemosyne 1	1	1	3.2
Papilionidae					mnemosyne 2	0.98	0.96	3.2
Papilionidae	*Parnassius*	*apollo*	ME	3	apollo 1	1	1	0.9
Papilionidae					apollo 2	1	1	0.9
Papilionidae					apollo 3	1	1	2.3
Nymphalidae	*Lasiommata*	*maera*	ME	4	maera 1	1	1	1.2
Nymphalidae					maera 2	0.49	0.41	1.2
Nymphalidae					maera 3	0.49	0.41	1.2
Nymphalidae					maera 4	0.94	0.92	1.2
Nymphalidae	*Erebia*	*pronoe*	ME	2	pronoe 1	1	0.98	2.6
Nymphalidae					pronoe 2	1	1	2.6
Nymphalidae	*Hipparchia*	*statilinus*	ME	2	statilinus 1	0.53	0.53	1.1
Nymphalidae					statilinus 2	0.53	0.53	1.1
Nymphalidae	*Hipparchia*	*semele*	ME; lumped	2	semele	1	1	3.3
Nymphalidae					semele/volgensis	1	1	3.3
Nymphalidae	*Hipparchia*	*alcyone*	ME; lumped		hermione	1	1	1.7
Nymphalidae					fagi/syriaca/hermione (taxon genava)	1	1	1.7
Nymphalidae	*Arethusana*	*arethusa*	ME	2	arethusa1	1	1	2.9
Nymphalidae					arethusa2	1	1	2.9
Nymphalidae	*Aphantopus*	*hyperantus*	ME	2	hyperantus 1	1	1	2.0
Nymphalidae					hyperantus 2	1	1	2.0
Nymphalidae	*Maniola*	*jurtina*	ME	2	jurtina 1	0.96	0.94	0.5
Nymphalidae					jurtina 2	0.96	0.94	0.5
Nymphalidae	*Pyronia*	*cecilia*	ME	2	cecilia 1	0.94	0.93	1.2
Nymphalidae					cecilia 2	0.94	0.93	1.2

Family	Genus	species	Status according to the ST-GMYC analysis	No. of entities for each ME case	GMYC entities involved	ST-GMYC support for entity differentiation	MM-GMYC support for entity differentiation	Minimum p-distance (%) to the nearest conspecific entity or entity containing conspecifics
Nymphalidae	*Coenonympha*	*arcania*	ME; lumped	2	arcania	1	1	2.1
Nymphalidae					arcania/leander/gardetta	0.99	0.97	2.1
Nymphalidae	*Coenonympha*	*dorus*	ME	2	dorus 1	0.67	0.66	1.4
Nymphalidae					dorus 2	0.67	0.66	1.4
Nymphalidae	*Coenonympha*	*pamphilus*	ME	2	pamphilus 1	1	1	1.8
Nymphalidae					pamphilus 2	1	1	1.8
Nymphalidae	*Boloria*	*napaea*	ME	2	napaea 1	1	1	1.5
Nymphalidae					napaea 2	0.52	0.52	1.5
Nymphalidae	*Boloria*	*pales*	ME	2	pales 1	1	1	1.2
Nymphalidae					pales 2	1	1	1.2
Nymphalidae	*Boloria*	*thore*	ME	2	thore 1	1	1	2.1
Nymphalidae					thore 2	1	1	2.1
Nymphalidae	*Boloria*	*euphrosyne*	ME	3	euphrosyne 1	1	1	4.0
Nymphalidae					euphrosyne 2	1	1	2.0
Nymphalidae					euphrosyne 3	0.86	0.85	2.0
Nymphalidae	*Argynnis*	*pandora*	ME	2	pandora 1	0.9	0.88	0.9
Nymphalidae					pandora 2	0.9	0.88	0.9
Nymphalidae	*Argynnis*	*aglaja*	ME	2	aglaja 1	1	1	1.1
Nymphalidae					aglaja 2	1	1	1.1
Nymphalidae	*Argynnis*	*adippe*	ME	2	adippe 1	1	1	1.6
Nymphalidae					adippe 2	1	1	1.6
Nymphalidae	*Brenthis*	*daphne*	ME; lumped	2	daphne	1	1	1.8
Nymphalidae					daphne/ino	1	1	1.8
Nymphalidae	*Brenthis*	*ino*	ME; lumped	2	daphne/ino	1	1	1.7
Nymphalidae					ino	1	0.99	1.7
Nymphalidae	*Brenthis*	*hecate*	ME	2	hecate 1	1	1	2.1
Nymphalidae					hecate 2	1	1	2.1
Nymphalidae	*Nymphalis*	*polychloros*	ME	2	polychloros 1	0.38	0.38	1.5
Nymphalidae					polychloros 2	0.38	0.38	1.5
Nymphalidae	*Aglais*	*urticae*	ME	2	urticae 1	0.89	0.87	1.4
Nymphalidae					urticae 2	0.89	0.87	1.4

Family	Genus	species	Status according to the ST-GMYC analysis	No. of entities for each ME case	GMYC entities involved	ST-GMYC support for entity differentiation	MM-GMYC support for entity differentiation	Minimum p-distance (%) to the nearest conspecific entity or entity containing conspecifics
Nymphalidae	*Melitaea*	*cinxia*	ME	2	cinxia 1	1	1	2.1
Nymphalidae					cinxia 2	1	0.99	2.1
Nymphalidae	*Melitaea*	*trivia*	ME	2	trivia 1	0.93	0.91	1.2
Nymphalidae					trivia 2	0.93	0.91	1.2
Nymphalidae	*Melitaea*	*didyma*	ME	4	didyma 1	0.97	0.95	2.6
Nymphalidae					didyma 2	1	1	2.6
Nymphalidae					didyma 3	1	0.98	2.6
Nymphalidae					didyma 4	1	1	2.6
Nymphalidae	*Melitaea*	*diamina*	ME	2	diamina 1	1	1	2.1
Nymphalidae					diamina 2	1	1	2.1
Nymphalidae	*Melitaea*	*aurelia*	ME	2	aurelia 1	0.93	0.9	1.2
Nymphalidae					aurelia 2	0.93	0.9	1.2
Nymphalidae	*Melitaea*	*parthenoides*	ME	2	parthenoides 1	1	0.99	0.9
Nymphalidae					parthenoides 2	1	0.99	0.9
Nymphalidae	*Melitaea*	*deione*	ME	3	deione 1	0.61	0.6	0.8
Nymphalidae					deione 2	0.61	0.6	0.8
Nymphalidae					deione 3	1	1	2.2
Nymphalidae	*Melitaea*	*athalia*	ME	3	athalia 1	0.85	0.83	0.3
Nymphalidae					athalia 2	0.85	0.83	0.3
Nymphalidae					athalia 3	1	0.98	2.9
Nymphalidae	*Limenitis*	*camilla*	ME	2	camilla 1	1	1	2.7
Nymphalidae					camilla 2	1	1	2.7
Lycaenidae	*Favonius*	*quercus*	ME	2	quercus 1	1	1	1.5
Lycaenidae					quercus 2	1	0.99	1.5
Lycaenidae	*Cupido*	*lorquinii*	ME; lumped	2	lorquinii	0.71	0.7	0.5
Lycaenidae					lorquinii/minimus (taxon carswelli)	0.71	0.7	0.5
Lycaenidae	*Cupido*	*minimus*	ME; lumped	2	lorquinii/minimus (taxon carswelli)	0.71	0.7	1.2
Lycaenidae					minimus	1	1	1.2
Lycaenidae	*Cupido*	*osiris*	ME	3	osiris 1	1	1	1.4
Lycaenidae					osiris 2	1	1	1.1
Lycaenidae					osiris 3	1	1	1.1

Family	Genus	species	Status according to the ST-GMYC analysis	No. of entities for each ME case	GMYC entities involved	ST-GMYC support for entity differentiation	MM-GMYC support for entity differentiation	Minimum p-distance (%) to the nearest conspecific entity or entity containing conspecifics
Lycaenidae	*Cupido*	*alcetas*	ME; lumped	2	decolorata/alcetas	0.79	0.77	1.1
Lycaenidae					alcetas	0.79	0.77	1.1
Lycaenidae	*Satyrium*	*spini*	ME	3	spini 1	1	0.99	2.9
Lycaenidae					spini 2	0.76	0.75	1.2
Lycaenidae					spini 3	0.76	0.75	1.2
Lycaenidae	*Scolitantides*	*orion*	ME	2	orion 1	0.93	0.91	1.2
Lycaenidae					orion 2	0.93	0.91	1.2
Lycaenidae	*Pseudophilotes*	*panoptes*	ME; lumped	2	panoptes/vicrama	0.74	0.72	0.5
Lycaenidae					baton/panoptes	0.74	0.72	0.5
Lycaenidae	*Glaucopsyche*	*alexis*	ME	2	alexis 1	1	1	1.2
Lycaenidae					alexis 2	1	1	1.2
Lycaenidae	*Plebejus*	*argus*	ME	4	argus 1	0.24	0.25	0.8
Lycaenidae					argus 2	0.24	0.25	0.8
Lycaenidae					argus 3	0.99	0.98	1.2
Lycaenidae					argus 4	0.99	0.98	1.1
Lycaenidae	*Plebejus*	*idas*	ME; lumped	3	idas/argyrognomon	1	1	2.0
Lycaenidae					idas 1	0.29	0.29	1.1
Lycaenidae					idas 2	0.29	0.29	1.1
Lycaenidae	*Polyommatus*	*amandus*	ME	2	amandus 1	1	1	0.9
Lycaenidae					amandus 2	1	1	0.9
Lycaenidae	*Polyommatus*	*celina*	ME	2	celina 1	0.57	0.57	0.5
Lycaenidae					celina 2	0.57	0.57	0.5
Lycaenidae	*Polyommatus*	*escheri*	ME	2	escheri 1	1	1	1.7
Lycaenidae					escheri 2	1	1	1.7
Lycaenidae	*Polyommatus*	*daphnis*	ME	2	daphnis 1	0.64	0.63	1.1
Lycaenidae					daphnis 2	0.64	0.63	1.1
Lycaenidae	*Polyommatus*	*thersites*	ME	2	thersites 1	0.81	0.79	0.9
Lycaenidae					thersites 2	0.81	0.79	0.9
Lycaenidae	*Polyommatus*	*violetae*	ME	2	violetae 1	0.42	0.42	1.1
Lycaenidae					violetae 2	0.42	0.42	1.1
Lycaenidae	*Polyommatus*	*damon*	ME	2	damon 1	0.97	0.96	1.2

Family	Genus	species	Status according to the ST-GMYC analysis	No. of entities for each ME case	GMYC entities involved	ST-GMYC support for entity differentiation	MM-GMYC support for entity differentiation	Minimum p-distance (%) to the nearest conspecific entity or entity containing conspecifics
Lycaenidae					damon 2	0.97	0.96	1.2
Lycaenidae	*Polyommatus*	*ripartii*	ME; lumped	2	ripartii/admetus/nephohiptamenos	0.96	0.95	1.2
Lycaenidae					ripartii	0.96	0.95	1.2
Lycaenidae	*Polyommatus*	*icarus*	ME	3	icarus 1	1	1	1.4
Lycaenidae					icarus 2	1	1	1.4
Lycaenidae					icarus 3	1	1	1.9
Lycaenidae	*Lysandra*	*bellargus*	ME; lumped	2	bellargus	1	1	2.3
Lycaenidae					coridon/bellargus/albicans	1	1	2.3
Lycaenidae	*Lysandra*	*coridon*	ME; lumped	3	coridon/bellargus/albicans	1	1	0.9
Lycaenidae					coridon/hispana/albicans 1	0.55	0.54	0.9
Lycaenidae					coridon/hispana/albicans 2	0.96	0.95	0.9
Lycaenidae	*Lysandra*	*albicans*	ME; lumped	4	coridon/bellargus/albicans	1	1	0.5
Lycaenidae					coridon/hispana/albicans 1	0.55	0.54	0.8
Lycaenidae					coridon/hispana/albicans 2	0.96	0.95	0.5
Lycaenidae					albicans/hispana/caelestissima	0.55	0.54	0.5
Lycaenidae	*Lysandra*	*hispana*	ME; lumped	3	coridon/hispana/albicans 1	0.55	0.54	0.8
Lycaenidae					coridon/hispana/albicans 2	0.96	0.95	0.5
Lycaenidae					albicans/hispana/caelestissima	0.55	0.54	0.5
Lycaenidae	*Aricia*	*morronensis*	ME	2	morronensis 1	1	0.99	1.4
Lycaenidae					morronensis 2	1	0.99	1.4
Lycaenidae	*Aricia*	*nicias*	ME	2	nicias 1	1	1	2.6
Lycaenidae					nicias 2	1	1	2.6
Lycaenidae	*Aricia*	*agestis*	ME	2	agestis 1	0.69	0.68	0.6
Lycaenidae					agestis 2	0.69	0.68	0.6
Lycaenidae	*Cyaniris*	*semiargus*	ME	2	semiargus 1	0.87	0.85	1.1
Lycaenidae					semiargus 2	0.87	0.85	1.1
Lycaenidae	*Eumedonia*	*eumedon*	ME	3	eumedon 1	1	1	1.2
Lycaenidae					eumedon 2	0.92	0.9	1.2
Lycaenidae					eumedon 3	0.92	0.9	1.2
Lycaenidae	*Lycaena*	*alciphron*	ME	2	alciphron 1	1	1	1.7
Lycaenidae					alciphron 2	1	1	1.7

**Family**	**Genus**	**species**	**Status according to the ST-GMYC analysis**	**No. of entities for each ME case**	**GMYC entities involved**	**ST-GMYC support for entity differentiation**	**MM-GMYC support for entity differentiation**	**Minimum p-distance (%) to the nearest conspecific entity or entity containing conspecifics**
Hesperiidae	*Spialia*	*sertorius*	ME; lumped	2	sertorius/orbifer	0.9	0.88	2.9
Hesperiidae					sertorius	1	1	2.9
Hesperiidae	*Carcharodus*	*alceae*	ME	2	alceae 1	1	1	1.5
Hesperiidae					alceae 2	1	1	1.5
Hesperiidae	*Carcharodus*	*lavatherae*	ME	2	lavatherae 1	1	1	1.5
Hesperiidae					lavatherae 2	1	1	1.5

Supplementary Table 5 | *Iphiclides* specimens and outgroup taxa used for DNA and/or morphological analyses.

Used in the GMYC analyses	Sample ID	COI GenBank accession number	ITS2 GenBank accession number	Morpho-metrics	Genus	Species	Subspecies	Locality	Province	Country
x	RVcoll.08-H001	HQ004551	KP851170	x	*Iphiclides*	*podalirius*		Cluj-Napoca, Hoia forest	Cluj	Romania
x	RV-07-D235	HQ004554	KP851168	x	*Iphiclides*	*podalirius*		Dealul Istrita, Breaza	Buzau	Romania
x	RVcoll.08-M475	HQ004556	KP851185	x	*Iphiclides*	*podalirius*		Babadag forest (10 Km S of Babadag)	Tulcea	Romania
x	RVcoll.08-M270	HQ004557	KP851191	x	*Iphiclides*	*podalirius*		4 Km SW of Cazanele Mari (around Plavisevita)	Mehedinti	Romania
x	RVcoll.10-C413		KP851183	x	*Iphiclides*	*podalirius*		Lăpuș-Prislop	Maramureș	Romania
	JC02001	AY556868	AY556551		*Iphiclides*	*podalirius*		Agia Marina	Spetses Island	Greece
x	RVcoll.12-P222		KP851178		*Iphiclides*	*podalirius*	*feisthamelii*	Caves	Aude	France
x	RVcoll.12-P223		KP851177		*Iphiclides*	*podalirius*	*feisthamelii*	Caves	Aude	France
x	RVcoll.08-H912	GU676883	KP851186		*Iphiclides*	*podalirius*	*feisthamelii*	Hervas, Carretera Hervas-La Garganta	Caceres	Spain
x	RVcoll.07-C075	GU669797	KP851179		*Iphiclides*	*podalirius*	*feisthamelii*	Puig Neulos, Alt Emporda	Girona	Spain
x	RVcoll.08-R262	GU669791	KP851167		*Iphiclides*	*podalirius*		Vilamos, Vall d`Aran	Lleida	Spain
x	RV-06-G438		KP851166		*Iphiclides*	*podalirius*	*feisthamelii*	El Sola, El Brull	Barcelona	Spain
x	RVcoll.08-H248	GU676709	KP851187		*Iphiclides*	*podalirius*	*feisthamelii*	Mequinenza	Zaragoza	Spain
x	RVcoll.06-G512	GU669790	KP851190	x	*Iphiclides*	*podalirius*	*feisthamelii*	Coll de Comiols, Pallars Jussa	Lleida	Spain
x	RVcoll.08-P429	GU675734	KP851169		*Iphiclides*	*podalirius*	*feisthamelii*	Candelario	Salamanca	Spain
x	RVcoll.09-V842	GU676069	KP851176	x	*Iphiclides*	*podalirius*	*feisthamelii*	Coll de Banyuls, Alt Emporda	Girona	Spain
x	RVcoll.11-E018		KP851182		*Iphiclides*	*podalirius*	*feisthamelii*	Font de l`Arbre, Serra d`Aitana	Alicante	Spain
x	RVcoll.11-D685		KP851188		*Iphiclides*	*podalirius*	*feisthamelii*	La Zubia, Barranco de Corvales	Granada	Spain
x	RVcoll.090211SD11		KP851173		*Iphiclides*	*podalirius*	*feisthamelii*	Sierra Espuna, Totana	Murcia	Spain
x	RVcoll.09-V592	GU676131	KP851172	x	*Iphiclides*	*podalirius*	*feisthamelii*	Seminario (Sierra Nevada)	Granada	Spain
x	SMcoll.08-J611	GU675805	KP851180	x	*Iphiclides*	*podalirius*	*feisthamelii*	Puerto de Santa Maria	Cadiz	Spain
x	RVcoll.08-J820	GU675876	KP851171	x	*Iphiclides*	*podalirius*	*feisthamelii*	Columbrianos	Leon	Spain
x	RVcoll.09-X581	GU676064	KP851184	x	*Iphiclides*	*podalirius*	*feisthamelii*	2 km E Navascues	Navarra	Spain
	MW01114	AY556964	GQ374235		*Iphiclides*	*podalirius*	*feisthamelii*	Taradell	Barcelona	Spain
x	RVcoll.08-J100	GU676576	KP851175	x	*Iphiclides*	*podalirius*	*feisthamelii*	Lanhoso (Castelo), Lanhoso, Povoa de Lanhoso	Minho	Portugal
	RVcoll.09-V106		KP851189	x	*Iphiclides*	*podalirius*	*feisthamelii*	Chefchaouen	Rif Mountains	Morocco
	RVcoll.11-F122		KP851174	x	*Iphiclides*	*podalirius*	*feisthamelii*	Zinat, 13 km S Tétouan	Rif Mountains	Morocco
	RVcoll.11-F292		KP851181	x	*Iphiclides*	*podalirius*	*feisthamelii*	Jbel Tissouka, Chefchaouen	Rif Mountains	Morocco

Used in the GMYC analyses	Sample ID	COI GenBank accession number	ITS2 GenBank accession number	Morpho-metrics	Genus	Species	Subspecies	Locality	Province	Country
		AF044001			*Papilio*	*phorcas*				
			EU153780		*Papilio*	*phorcas*				
x	RVcoll.07-E253	JF512587	JF512811		*Leptidea*	*sinapis*		NE Bézaudun-sur-Bine	Drôme	France

Supplementary Table 6 | Male genitalia measurement results for the *Iphiclides* specimens used for morphometrics.

Sample ID	Genus	Species	Subspecies	Country	Ostium phallus (OP)	Phallus width (PW)	Phallus length (PL)	OP/PL	PW/PL
RVcoll.09-V106	*Iphiclides*	*podalirius*	*feisthamelii*	Morocco	1.109	0.336	2.492	0.445	0.135
RVcoll.11-F122	*Iphiclides*	*podalirius*	*feisthamelii*	Morocco	1.055	0.339	2.670	0.395	0.127
RVcoll.11-F292	*Iphiclides*	*podalirius*	*feisthamelii*	Morocco	1.165	0.426	2.751	0.423	0.155
RVcoll.08-J100	*Iphiclides*	*podalirius*	*feisthamelii*	Portugal	1.208	0.357	3.010	0.401	0.119
RVcoll.06-G512	*Iphiclides*	*podalirius*	*feisthamelii*	Spain	1.084	0.322	2.570	0.422	0.125
RVcoll.09-V842	*Iphiclides*	*podalirius*	*feisthamelii*	Spain	0.992	0.347	2.652	0.374	0.131
RVcoll.09-V592	*Iphiclides*	*podalirius*	*feisthamelii*	Spain	1.140	0.391	2.834	0.402	0.138
SMcoll.08-J611	*Iphiclides*	*podalirius*	*feisthamelii*	Spain	1.020	0.360	2.528	0.404	0.143
RVcoll.08-J820	*Iphiclides*	*podalirius*	*feisthamelii*	Spain	1.139	0.384	2.753	0.414	0.139
RVcoll.09-X581	*Iphiclides*	*podalirius*	*feisthamelii*	Spain	1.074	0.359	2.588	0.415	0.139
RVcoll.08-H001	*Iphiclides*	*podalirius*		Romania	1.477	0.445	2.821	0.524	0.158
RV-07-D235	*Iphiclides*	*podalirius*		Romania	1.704	0.436	3.104	0.549	0.141
RVcoll.08-M475	*Iphiclides*	*podalirius*		Romania	1.603	0.394	2.972	0.539	0.133
RVcoll.08-M270	*Iphiclides*	*podalirius*		Romania	1.517	0.514	2.874	0.528	0.179
RVcoll.10-C413	*Iphiclides*	*podalirius*		Romania	1.382	0.337	2.621	0.527	0.129

Supplementary Annex 1

Discussion of taxa lumped or split into multiple entities by the GMYC model

We recognized 299 species based on the checklist of the Fauna Europaea Project[1] which represents a consensus of the taxonomic community working on these organisms. We supplemented this list with two species that have been thoroughly documented in recent studies (*Polyommatus celina*, *Leptidea juvernica*) (see comments for each below). One species (*Polyommatus galloi*) was eliminated based on recent evidence[2].

Our entire dataset of 5782 specimens (299 species) was subjected to a GMYC analysis. We then examined all the entities (i.e. putative species) recovered by this analysis and classified them in three main categories with respect to the current taxonomic list of 299 species (Supplementary Table 3).
1) **Single entity (SE)**: species that were recovered as a single entity, indicating correspondence with current taxonomy.
2) **Lumped**: specimens of two or more species that were recovered as a single GMYC entity. Example: the specimens of *Polyommatus golgus* (Hübner, 1813) and of *Polyommatus dorylas* (Denis & Schiffermüller, 1775) present in our dataset were recovered as a single GMYC entitity - thus, both *P. golgus* and *P. dorylas* were considered as "lumped".
3) **Multiple entities (ME)**: species that were split in two or more GMYC entities, which represent potential cryptic biodiversity.

There are 18 cases in which a species was split into two entities by the GMYC tree, but one of the entities actually included specimens of (at least) another species. In this case, for a given species, we would simultaneously have "ME" and "lumped". Example: *Pyrgus alveus* was split in two entities, but one of them includes *P. bellieri* and *P. warrenensis*. These are often rather complex cases, and their interpretation is not always straightforward without additional studies.

Each specific case present in our dataset is discussed below in accordance to the criteria mentioned above. It is worth noting that the references usually cover general works that likely represent widely accepted trends in European butterfly taxonomy. We used this approach for practical reasons, since it is almost impossible to cover all the existing literature for so many species in a single paper, not to mention that access to some of the publications is difficult. However, in several cases where relevant case studies were available, these have been cited as well.

We are also aware that some of the GMYC entities highlighted by our analysis seem to partially or fully match the distribution of previously described subspecies for various taxa. Only where relevant material was available, we made reference to potential matches with subspecies for a few Iberian taxa, where our sampling was more comprehensive. While this is interesting, our sampling is not sufficient to draw definitive conclusions on the detailed distributions of different genetic lineages.

It is important to keep in mind that the categories used here do not represent our conclusions based on DNA barcodes, but the description of the current taxonomic situation as reflected in the general works cited. Thus, this study should be seen as a general preview of the genetic diversity of a part of the European butterflies, providing general guidelines for future research

on case studies. Due to the nature of our dataset having the best sampling in Iberia, Romania and southern Germany, the interpretation of the genetic patterns detected is often based on these regions.

The taxonomy and nomenclature largely follows Fauna Europaea, with corrections where considered necessary based on the following taxonomic revisions:[3,4] for Pieridae;[5] for Nymphalidae;[6] for Lycaenidae;[7] for Hesperiidae. We are aware that several taxon names used in Fauna Europaea are subject to debate. Several such cases are briefly commented below, under the relevant taxa. GMYC entities were considered sympatric if they were separated by less than 50 kilometres. In order to facilitate the interpretation of the genetic patterns detected, for each taxon involving multiple entities (ME), the distribution of these entities has been figured on maps that are provided in Supplementary Annex 2.
Within each family, the species are listed in alphabetical order.

PAPILIONIDAE

***Papilio machaon* Linnaeus, 1758 (ME)**
This taxon was split into two entities in the GMYC tree. Both are widespread and occur in sympatry at least in north-western Romania, although their general distribution pattern suggests potential sympatry in central Europe and Iberia as well.

***Parnassius apollo* (Linnaeus, 1758) (ME)**
It was split into three allopatric entities in the GMYC tree. Their distribution is in accordance with the findings of[8]. A high number of subspecies has been described within *P. apollo.*

***Parnassius mnemosyne* (Linnaeus, 1758) (ME)**
It was split into two allopatric entities in the GMYC tree: one was detected in Iberia and southern Germany, and the other in Romania. This pattern mirrors the results of[9] and[10] who found that the populations of *P. mnemosyne* form two main groups in Europe (a western and an eastern one).

***Zerynthia rumina* (Linnaeus, 1758) (ME)**
It was split into two allopatric entities in the GMYC tree: one occurs in north-eastern Spain while the other populates the rest of the Iberian Peninsula.

PIERIDAE

***Colias croceus* (Geoffroy, 1785) (lumped) - *C. erate* (Esper, 1805) (lumped)**
These two species are lumped as a single entity in the GMYC analysis and they also share DNA barcodes. This was to be expected since similar phenomena have been recently reported by studies from which we are using data in the current analysis[11,12]. Natural hybrids between the two species have been recorded[13,14,15] and[12] reported specimens with intermediate morphology suspected to represent hybrids. Those specimens have been indicated as potential hybrids in our dataset. Fauna Europaea accepts both taxa as species.
Although in Fauna Europaea the specific name *croceus* (Fourcroy, 1785) is used, some authors consider that the name *crocea* should replace it[16].

***Euchloe belemia* (Esper, 1800) (ME)**
This taxon was split into two entities in the GMYC tree. One of the entities comprises a single specimen from Cádiz (Andalusia), found in sympatry with specimens belonging to the other

entity. It is possible that this single specimen represents a lineage occurring in North Africa, but additional molecular studies are needed to confirm this hypothesis.

***Euchloe tagis* (Hübner, 1804) (ME)**
This taxon was split into two entities in the GMYC tree. The distribution of the entities suggests that they are sympatric in the central and eastern parts of Iberia.

***Gonepteryx rhamni* (Linnaeus, 1758) (ME)**
This taxon was split in three entities in the GMYC tree. Current sampling suggests that one is confined to Iberia, one is widespread outside Iberia (it stops in north-eastern Spain), and the third one occurs both in north-eastern Iberia and Romania (being probably more widespread in between). The last two entities were detected in sympatry in north-eastern Spain and in Romania.

***Pieris mannii* (Mayer, 1851) (ME)**
This taxon was split into two entities in the GMYC tree. They are allopatric, one appearing to be restricted to southern and eastern Iberia, while the other was detected in the Pyrenees and south-western Romania.

***Pieris napi* (Linnaeus, 1758) (ME, lumped) - *P. bryoniae* (Hübner, 1806) (lumped)**
There has been a long debate whether these taxa represent different species after various analyses of hybridization patterns[12,13,17,18,19]. This resulted in some authors suggesting that *bryoniae* is a subspecies of *napi*[20,21,22]. However, most recent studies consider them as different species[13,15,23,24]. These two taxa can be generally considered as sympatric, although they usually display different ecological preferences (*P. bryoniae* is restricted to higher altitudes). Fauna Europaea accepts *bryoniae* as species.
Pieris napi was split into two entities in our GMYC analysis. One of these entities is actually shared with *P. bryoniae*, causing both taxa to be categorized as “lumped”. It should be noted that the two taxa are already known to hybridize rather frequently[13] and the observed genetic pattern is likely to be the consequence of introgression (e.g.[12]). Furthermore, the two taxa have identical DNA barcodes in the shared GMYC entity.
It is worth noting that one specimen of *P. napi* that clusters with *P. bryoniae* originates from north-eastern Spain (Catalonia), area far from the current range of *P. bryoniae*, although see [24] for comments on reports from the Pyrenees.

***Pieris pseudorapae* Verity, 1908 (lumped)**
This taxon is not listed in Fauna Europaea. However, the taxon *balcana* (Lorković, 1970) does appear as a species and Fauna Europaea comments that it has sometimes been considered as a subspecies of *P. pseudorapae*. We maintained the name *pseudorapae* as used in the original publication producing these DNA barcodes[11]. It should be noted that the taxonomic status, relationship and distribution of both *pseudorapae* and *balcana* with respect to *napi* are not fully understood and require further studies[24,25,26,27].
The two specimens present in our dataset originate from southern European Russia and cluster together with *P. napi*.

***Pieris rapae* (Linnaeus, 1758) (ME)**
It was split into two entities in the GMYC tree. They have been detected in sympatry in southern Portugal, north-eastern Spain, the Balearics (Mallorca) and Romania.

NYMPHALIDAE

***Aphantopus hyperantus* (Linnaeus, 1758) (ME)**
It was split into two entities in the GMYC tree. One of the entities consists of a single specimen found in sympatry with individuals of the other entity in the eastern Pyrenees (Spain).

***Arethusana arethusa* (Denis & Schiffermüller, 1775) (ME)**
This species is split into two allopatric entities in our GMYC analysis. One of the entities includes all the specimens attributable to the taxon *boabdil* (Rambur, 1839), considered to be restricted to southern Spain[15,20,24,28]. Interestingly, the same entity also includes specimens from north-western Spain, that were either considered to belong to the nominotypical subspecies[15] or to the subspecies *dentata* (Staudinger, 1871)[20,26,29].
Some studies[30,31] noted the morphological similarity between *dentata* and *boabdil* and proposed their recognition as a distinct taxon, *A. boabdil*[32]. Our data support this view.
Although it is considered in Fauna Europaea and Fauna Iberica[29] as a synonym of *arethusa*, the taxon *boabdil* has been raised to species rank by some authors[26,27,32,33].

***Coenonympha arcania* (Linnaeus, 1761) (ME & lumped) - *C. leander* (Esper, 1784) (lumped) - *C. gardetta* (de Prunner, 1798) (lumped)**
These taxa are part of a group with long debated taxonomy (taxa such as *darwiniana*, *macromma* and *orientalis* are also part of it)[24,34,35,36].
The species status of *C. leander* has not usually been questioned and the taxon is recognized as a species in Fauna Europaea. In the GMYC analysis, it is lumped with *C. gardetta* and the Romanian *C. arcania*. However, *C. leander* forms a distinct clade and is closely related to the Romanian *C. arcania* plus the single specimen of *C. gardetta* present in the dataset.
Coenonympha gardetta has been treated as a species in the synthesis works on European butterflies published beginning with 1970 and also by Fauna Europaea. The single specimen of *C. gardetta* present in our dataset was lumped together with *C. leander* and Romanian *C. arcania* in the GMYC analysis. Published data suggest that *C. arcania* and *C. gardetta* are parapatric, with a restricted hybrid zone in Lago Maggiore and Col de Larche[35].
Coenonympha arcania was split into two GMYC entities: one included all Iberian and some of the German specimens analysed; the other included all Romanian samples, one German specimen, as well as all *C. leander* and the single *C. gardetta* analysed. Therefore, it appears that the two *C. arcania* entities occur in close proximity in southern Germany.

***Coenonympha dorus* (Esper, 1782) (ME)**
This taxon was split into two entities in the GMYC tree. One was identified in two localities in north-eastern Spain, while the other appears to be widespread in Iberia. The 50 km distance threshold used by us suggests that they could occur in sympatry in north-eastern Spain.

***Coenonympha pamphilus* (Linnaeus, 1758) (ME)**
It was split into two allopatric entities in the GMYC tree. One of these entities only included specimens from the Balearic Islands. These have sometimes been attributed to the taxon *lyllus* (Esper, 1805), which has been raised to species rank by some authors[35,37,38]. However, it should be noted that the taxon *lyllus* was described from the southern part of the Iberian Peninsula and such specimens included in our analysis are recovered within the same entity with *C. pamphilus*. This can be interpreted in two ways:
1) *lyllus* may be conspecific with *pamphilus* and the Balearics populations might actually represent a distinct taxon requiring a new name;
2) *lyllus* may be a distinct species but, on mainland, there has been genetic introgression from *pamphilus* into *lyllus*, causing their similarity in mtDNA.

Certain morphological differences have also been reported between *pamphilus* and *lyllus*[37, 39]. Further studies correlating morphological and genetical data, including nuclear markers and extending the study area to North Africa as well, are needed in order to clarify the situation.
Fauna Europaea considers *lyllus* as a synonym of *C. pamphilus,* but mentions that some authors considered *lyllus* as subspecies of *pamphilus*[29] or as a distinct species.

***Coenonympha tullia* (Müller, 1764) (lumped) - *C. rhodopensis* Elwes, 1900 (lumped)**
The taxon *rhodopensis* has sometimes been treated as a subspecies of *C. tullia*[20,21]. More recently, it has usually been regarded as a distinct species[15,24,26,27,40]. Fauna Europaea accepts both taxa as distinct species.
In our GMYC analysis, both species are lumped as a single entity and *C. rhodopensis* appears as very little diverged (min. p-distance 0.3%) and paraphyletic with respect to *C. tullia*. Paraphyly involving these two species has recently been reported based on Romanian material[12].

***Erebia ligea* (Linnaeus, 1758) (lumped) - *E. euryale* (Esper, 1805) (lumped)**
Both are well-recognized species that, although morphologically similar, are readily separable based on morphological characters. They are recovered as a single entity in our GMYC analysis. A lack of clear separation based on DNA barcodes, suggesting incomplete lineage sorting or local introgression, has been recently reported by[12].

***Erebia medusa* (Denis & Schiffermüller, 1775) (lumped) - *E. polaris* Staudinger, 1871 (lumped)**
Since 1970, the two taxa have been most often regarded as different species, although (especially) older works considered *polaris* as a subspecies of *medusa*[41]. Fauna Europaea accepts *polaris* as a species, but comments that some authors consider it as a subspecies of *E. medusa*. In accordance with the results of[42], our analyses reveal that the two taxa share DNA barcodes and are lumped in a single entity in the GMYC analysis.

***Erebia meolans* (de Prunner, 1798) (lumped) - *E. palarica* Chapman, 1905 (lumped)**
Most authors, as well as Fauna Europaea, consider them as closely related, but distinct species. They are recovered as a single entity in our GMYC analysis, but form distinct clades (minimum genetic distance of 0.9%). These findings are in accordance with previous genetic analyses[43] showing that *E. meolans* and *E. palarica* are recently diverged sister species.

***Erebia neoridas* (Boisduval, 1828) (lumped) - *E. zapateri* Oberthür, 1875 (lumped)**
These morphologically similar taxa are usually accepted as distinct species, including by Fauna Europaea. In our GMYC analysis, they are lumped in a single entity, with *neoridas* paraphyletic with respect to *zapateri*. The issue requires further research, but the results suggest incomplete lineage sorting or introgression.

***Erebia pandrose* (Borkhausen, 1788) (lumped) - *E. sthennyo* Graslin, 1850 (lumped)**
These two morphologically similar sister species can be recognized on the basis of minor morphologic traits. They are recovered as a single entity in our GMYC analysis. The taxon *sthennyo* has been considered as a subspecies of *E. pandrose*[21,29,44], but also as a distinct species[15,20,24,26,27].
Although most authors consider that *sthennyo* is restricted to the Pyrenees, the results of[44] suggest that this taxon is more widespread (Pyrenees, south-eastern Alps, Durmitor in Montenegro and the Altai Mountains). According to this study, the taxa *pandrose* and *sthennyo* appear to be largely allopatric with some narrow contact zones. A population with

intermediate morphology has been identified in the south-eastern Alps, suggesting the potential for hybridization. Fauna Europaea accepts both taxa as species.

***Erebia pronoe* (Esper, 1780) (ME)**
It was split into two allopatric entities in the GMYC tree. One entity comprises specimens from southern Germany and Romania, while the other reunites specimens from the Pyrenees and the Cantabrian Mountains, which are attributable to subspecies *glottis* Fruhstorfer, 1920 (Pyrenees) and *martae* Mortera, 2001 (Cantabrian Mountains). Our analyses indicate that geographically-correlated genetic structure exists within the Iberian entity: the Cantabrian and Pyrenean specimens form distinct clades separated by a minimum genetic distance of 1.1%.

***E. rondoui* Oberthür, 1908 (lumped) - *E. tyndarus* (Esper, 1781) (lumped) - *E. cassioides* (Reiner & Hochenwarth, 1792) (lumped)**
These morphologically similar taxa belong to the so-called *Erebia tyndarus* species-group. This is a taxonomically challenging system and contrasting opinions have been expressed regarding species composition and their distribution[13,15,20,24,26,27,29,45], with *E. rondoui* sometimes being treated as a subspecies of *E. hispania* Butler, 1868. The names assigned to different taxa in our dataset follow the results of[45].
In our GMYC analysis, *tyndarus*, *rondoui* and *cassioides* are lumped in a single entity, with *rondoui* recovered as a clade, while the single *tyndarus* specimen present in our analysis shares its barcode with *E. cassioides*. *E. tyndarus* and *E. cassiodes* are known to be very closely related[45].

***Hipparchia fagi* (Scopoli, 1763) (lumped)**
In our GMYC analysis, this species is lumped with *H. syriaca* and five specimens of *H. hermione*. Similarity between *fagi* and *syriaca* DNA barcodes has been recently reported[12]. The *H. hermione* specimens clustering with *fagi* and *syriaca* are briefly discussed under the following case.

***Hipparchia hermione* (Linnaeus, 1764) (ME, lumped) - *H. syriaca* (Staudinger, 1871) (lumped)**
Certain studies[20,21] considered *syriaca* as a subspecies of *hermione*. Most subsequent works (including Fauna Europaea) treat *hermione* and *syriaca* as distinct species.
In our GMYC analysis, *H. hermione* was split into two allopatric entities. One of them includes most sequenced specimens, while the other includes five specimens (four from south-eastern France and one from Switzerland). These five specimens are lumped into a single entity together with *H. fagi* and *H. syriaca*, but form a distinct clade that is genetically closest to *H. syriaca* (minimum p-distance of 0.6%). Since all specimens were males, we examined the Jullien organ (Figs. S1-S5).
The appearance of the Jullien organ of the four French specimens (having 8 to 13 batons - see insertions points of two missing batons in the right part of the Jullien organ of sample 12-P047) corresponds to that of the taxon *genava* (Fruhstorfer, 1908) (Figs S1-S4), while the Swiss specimen, having over 25 batons, corresponds to *H. hermione*[46,47,48] (Fig. S5). The taxon *genava* has been raised to species rank by some authors[27,46,47,48,49], but several other works[15,21,24,40,50], as well as Fauna Europaea, consider it as a subspecies or synonym of *H. hermione*. The whole topic requires detailed studies from both a morphological and molecular point of view.

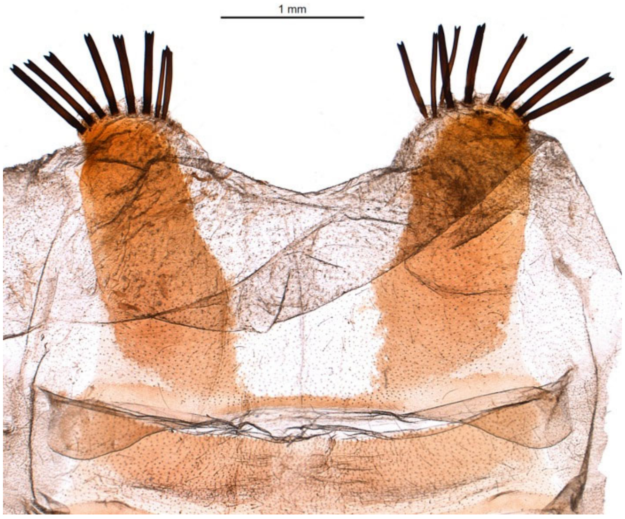

Fig. S1. Jullien organ of sample 11-I657, Mont Ventoux (Vaucluse), France. Prep. by S. Montagud.

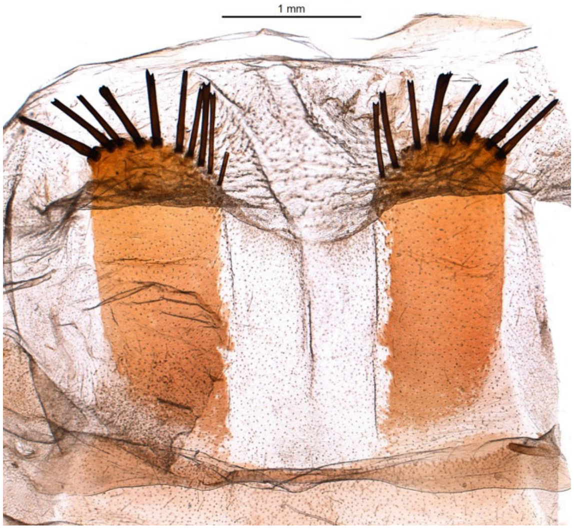

Fig. S2. Jullien organ of sample 11-I780, Montauban-sur-l'Ouvèze (Drôme), France. Prep. by S. Montagud.

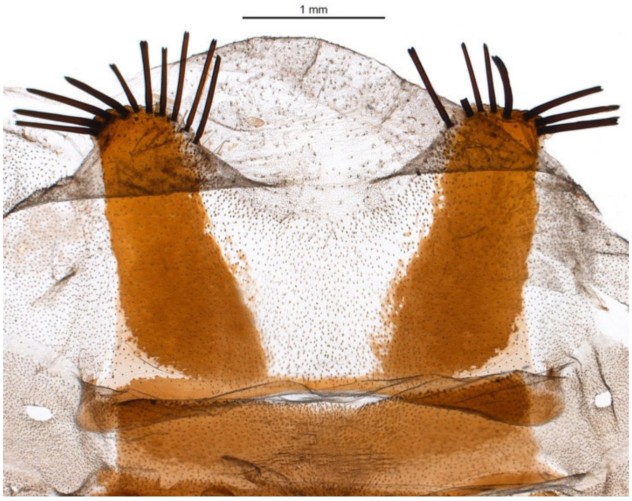

Fig. S3. Jullien organ of sample 11-I885, Rossas (Hautes Alpes), France. Prep. by S. Montagud.

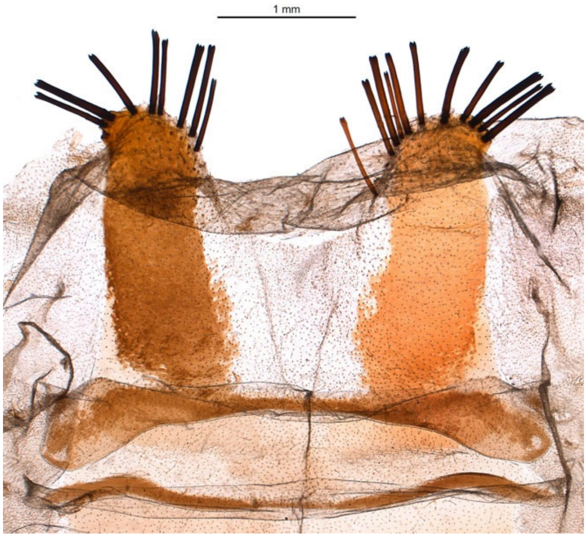

Fig. S4. Jullien organ of sample 12-P047, Châtillon-en-Diois (Drôme), France. Prep. by S. Montagud.

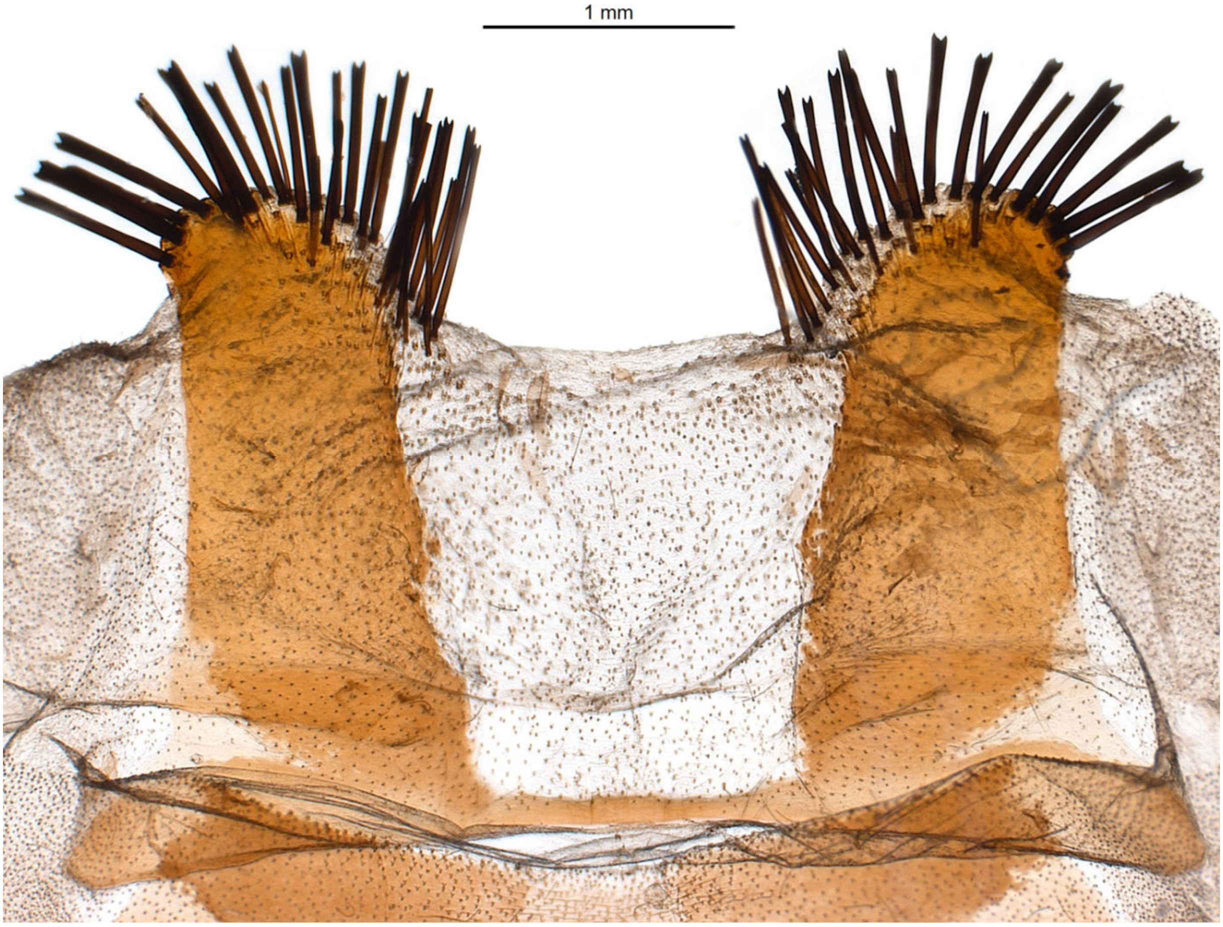

Fig. S5. Jullien organ of sample 11-J138, Birgisch (Valais), Switzerland. Prep. by S. Montagud.

It should be noted that Fauna Europaea considers *alcyone* (Denis & Schiffermüller, 1775) as a synonym of *hermione* (Linnaeus, 1764). Following other authors[16,29,50], we consider that the name *hermione* should be used.

***Hipparchia semele* (Linnaeus, 1758) (ME, lumped) - *H. volgensis* (Mazochin-Porshnjakov 1952) (lumped)**

The relationship and distribution of these two taxa in eastern and south-eastern Europe is poorly known due to their morphological similarity[24]. For further details about the single *volgensis* specimen included in our analyses which shares barcode with *H. semele*, see Annex 4 of the electronic supplementary material of [12].

It is worth noting that one Romanian specimen assigned to *H. semele* is well-diverged and recovered as a different entity that occurs in sympatry with the other entity in south-western Romania[12]. The whole group certainly requires further studies at least at an European scale.

***Hipparchia statilinus* (Hufnagel, 1766) (ME)**

It was split into two allopatric entities in the GMYC tree. One entity corresponds to Iberian specimens, while the other includes the single Romanian specimen analysed.

***Lasiommata maera* (Linnaeus, 1758) (ME)**

This taxon was split into four entities in the GMYC tree. Two of them were detected in sympatry in north-western Romania; two appear sympatric in the Alps; one of them was

identified only in Iberia. Note that one of the GMYC entities ("maera 2") is represented by a single specimen from north-western Romania.

***Maniola jurtina* (Linnaeus, 1758) (ME)**
It was split into two allopatric entities in the GMYC tree. The two GMYC entities correspond to a western lineage (taxon *jurtina* Linnaeus, 1758) and a central-eastern European one (taxon *janira* Linnaeus, 1758), in accordance with previous results[51,52,53].

***Melanargia galathea* (Linnaeus, 1758) (lumped) - *M. lachesis* (Hübner, 1790) (lumped)**
The taxon *lachesis* has been treated as a subspecies of *M. galathea* by[20] and[21], but was raised to species rank by most subsequent synthesis works on European butterflies. Recent molecular and morphological analyses seem to support the species status of *lachesis*[54]. Fauna Europaea and Fauna Iberica[29] accept both taxa as different species.
Although each of the two taxa forms distinct clades, they are closely related (minimum genetic distance 0.8%) and are recovered as a single GMYC entity.

***Oeneis norna* (Thunberg, 1791) (lumped) - *O. glacialis* (Moll, 1783) (lumped)**
Although recognized as being morphologically similar, the two taxa have usually been recognized as distinct allopatric species, including by Fauna Europaea. In accordance with the findings of[42], they are lumped together in our GMYC analysis and some even have identical DNA barcodes.

***Pyronia cecilia* (Vallantin, 1894) (ME)**
It was split into two entities in the GMYC tree. These entities were found in sympatry on the Iberian mainland, as well as on Mallorca (Balearics).

***Aglais urticae* (Linnaeus, 1758) (ME)**
It was split into two allopatric entities in the GMYC tree. One of the entities was found only in the Sierra Nevada Mountains in southern Spain. It is worth noting that previous studies using both allozyme and mitochondrial DNA (COI and control region) data did not report any clear correlation between geography and genetic patterns[55, 56].

***Apatura ilia* (Denis & Schiffermüller, 1775) (lumped) - *A. metis* Freyer, 1829 (lumped)**
The two taxa are lumped in a single entity in the GMYC analysis, *A. ilia* being paraphyletic with respect to *A. metis*, in accordance with recent findings by[12]. Especially in older literature[20,57], *A. metis* has sometimes been considered as a subspecies of *A. ilia*. *Apatura metis* is treated as a species by Fauna Europaea.

***Argynnis adippe* (Denis & Schiffermüller, 1775) (ME)**
It was split into two entities in the GMYC tree. One of these entities appears confined to Iberia, while the other ranges from the Pyrenees across southern Germany and to Romania. The two entities have been found in sympatry in the eastern Pyrenees.

***Argynnis aglaja* (Linnaeus, 1758) (ME)**
Argynnis aglaja was split into two entities in the GMYC tree. One of them consists of a single Romanian specimen occurring in sympatry with specimens of the other entity in north-western Romania (Transylvania).

***Argynnis pandora* (Denis & Schiffermüller, 1775) (ME)**

It was split into two entities in the GMYC tree. These entities were detected in sympatry in Romania and the current sampling suggests that they may be found in sympatry in Iberia as well.

***Boloria euphrosyne* (Linnaeus, 1758) (ME)**
This taxon was split into three entities in the GMYC tree. One was detected from Iberia to Romania; another was detected in the Pyrenees and the Alps and occurs in sympatry with the previous in the eastern Pyrenees; a third one is represented by a single specimen from south-western Romania. Deep genetic divergence within this taxon was recently reported based on Romanian material[12].

***Boloria napaea* (Hoffmannsegg, 1804) (ME)**
This taxon was split into two allopatric entities in the GMYC tree. One consists of a single German specimen. It is worth noting that the Alps host both entities while Scandinavia has only one of them. We have checked the trace files associated with the German specimen and their quality was good. Because of this single specimen, *B. napaea* appears as paraphyletic with respect to *B. aquilonaris* (Stichel, 1908).

***Boloria pales* (Denis & Schiffermüller, 1775) (ME)**
This taxon was split into two entities in the GMYC tree. One corresponds to Carpathian and Alpine populations, while the other consists exclusively of Pyrenean and Cantabrian specimens (taxon *pyrenesmiscens* Warren, 1944). The taxon *pyrenesmiscens* has usually been treated as a subspecies of *B. pales*. However,[58] considers *pyrenesmiscens* as a distinct species, a view adopted by other authors as well[59].
The presence of *B. napaea* has been reported in the eastern part of the Spanish Pyrenees[29], but we did not have any Spanish material that can be assigned to this taxon beyond doubt. As a matter of fact, one would need females with "typical" *napaea* appearance in order to be confident of the identification because of the morphological similarity between the males of *pales* and *napaea*, even with respect to their genitalia[21]. In our opinion, while it cannot be discounted, the presence of *B. napaea* in Spain requires further documentation. As a matter of fact, as suggested by the DNA barcodes, both *B. pales* and *B. napaea* require further studies at larger geographical scales.

***Boloria thore* (Hübner, 1803) (ME)**
It was split into two sympatric entities in the GMYC tree. Similar to *B. napaea*, it appears that the Alps host both entities in sympatry while Scandinavia has only one of them.

***Brenthis daphne* (Bergstässer, 1780) (ME & lumped) - *B. ino* (Rottemburg, 1775) (ME & lumped)**
Each of these species was split into two entities in the GMYC analysis but they actually share one entity that contains both specimens of *B. daphne* and *B. ino*. Within this shared entity, specimens of the two taxa form closely related (minimum genetic distance 0.5%), but distinct clades. It is possible that the observed pattern is due to introgression, but this will require further studies.

***Brenthis hecate* (Denis & Schiffermüller, 1775) (ME)**
It was split into two entities in the GMYC tree. One of the entities consists of a single specimen from Transylvania, Romania, sampled less than 40 km away from specimens of the other entity (considered sympatric according to our 50 km distance threshold).

***Limenitis camilla* (Linnaeus, 1764) (ME)**
It was split into two allopatric entities in the GMYC tree. The Romanian Carpathians represent a region where both entities have been detected: one in the Western Carpathians, the other in the Oriental Carpathians.

***Melitaea athalia* (Rottemburg, 1775) (ME)**
Melitaea athalia was split into three entities in the GMYC analysis. One of the entities includes exclusively specimens from western Europe (Iberia and northern Italy), another one was found only in Iberia while the third one appeared in central and eastern Europe.
It is interesting to note that the populations of *M. athalia* from south-western Europe have been considered as either a subspecies of *M. athalia*, or as a distinct species. The correct name of this taxon has been debated, with opinions ranging from *celadussa* Fruhstorfer, 1910, to *helvetica* Rühl, 1888 and *nevadensis* Oberthür, 1904[15,16,60]. Our results suggest that populations of *M. athalia* from south-western Europe can be attributed to the taxon *celadussa*, recently proposed as a distinct species[61]. The entity that seems to occur exclusively in Iberia could be associated to the taxon *nevadensis* (considered to be restricted to Sierra Nevada in southern Spain), although our results indicate that this lineage is not restricted to southern Spain. Actually, both the *celadussa* and *nevadensis* entities occur in north-eastern Spain and, although not detected in sympatry according to our 50 km threshold, they are separated by less than 100 km in our sampling.
Fauna Europaea treats *celadussa* and *nevadensis* as subspecies of *M. athalia*. The complex genetic structure identified in *M. athalia* indicates the need for deeper studies. Such studies should also clarify the taxonomic status of *celadussa* and *nevadensis* that are here tentatively assigned to the entities detected.

***Melitaea aurelia* Nickerl, 1850 (ME)**
It was split into two entities in the GMYC tree. Based on our sampling, the two entities appear to occur in sympatry in Romania.

***Melitaea cinxia* (Linnaeus, 1758) (ME)**
It was split into two allopatric entities in the GMYC tree, one appearing to be restricted to Iberia. This separation appears to be in agreement with the results of[62].

***Melitaea deione* (Geyer, 1832) (ME)**
It was split into three entities in the GMYC tree. Two of the entities were found only in the Sierra Nevada Mountains in southern Spain, and all three entities were detected in sympatry in these mountains.

***Melitaea diamina* (Lang, 1789) (ME)**
It was split into two allopatric entities in the GMYC tree. One of the entities consists of a single specimen originating from a population at the south-western distribution limit of the species (Castellón)[28,63]. The populations from Castellón and Tarragona (Ports de Tortosa) have been assigned to *M. diamina codinai* (de Sagarra, 1930).

***Melitaea didyma* (Esper, 1778) (ME)**
It was split into four entities in the GMYC tree: one in Romania; another from southern Germany as well as eastern and north-eastern Iberia; a third one found exclusively in Iberia (occurring in sympatry with the previous one), while a fourth one was detected only in the Sierra Nevada Mountains in southern Spain. A complex genetic structure for various populations of *M. didyma* has also been recently reported by[61].

***Melitaea parthenoides* Keferstein, 1851 (ME)**
It was split into two allopatric entities in the GMYC tree, one occurring in the extreme north-east of Spain, the other in the rest of Iberia.

***Melitaea trivia* (Denis & Schiffermüller, 1775) (ME)**
It was split into two allopatric entities in the GMYC tree. Each entity corresponds to the two geographical areas sampled (Iberia and Romania respectively). It should be noted that[60] treat the Iberian populations as a distinct species, *Melitaea ignasiti* de Sagarra, 1926, whose type locality lies in Montseny (Catalonia). Our dataset includes five specimens of *M. trivia* from the Montseny area (Supplementary Table 1). A more comprehensive sampling will be needed in order to better understand the genetic structure of *M. trivia*.

***Nymphalis polychloros* (Linnaeus, 1758) (ME)**
It was split into two entities in the GMYC tree. One of the entities consists of a single specimen from Valencia (Spain) that occurs in sympatry with specimens of the other entity.

LYCAENIDAE
***Aricia agestis* (Denis & Schiffermüller, 1775) (ME)**
It was split into two allopatric entities in the GMYC tree. One consists exclusively of two Sicilian specimens while the other appears widespread from north-eastern Spain, across central and eastern Europe, including the Balkans.

***Aricia morronensis* (Ribbe, 1910) (ME)**
This Iberian endemic was split into two allopatric entities in the GMYC tree, in agreement with the results of[64]. Up to six subspecies might exist for *A. morronensis* according to[29]. In the light of our results and while the subspecific status remains acceptable, it is reasonable to restrict the number of subspecies to two: the nominal one (*A. morronensis morronensis* Ribbe, 1910) occurring in the South and South-East of Spain (from Málaga to Alicante) of which *ramburi* (Verity, 1913) would be a synonym, and another one occupying the rest of the area, for which *A. morronensis chapmani* (Ribbe, 1910) would be the correct name.
The relationship between the several isolated populations of this species requires further study.

***Aricia nicias* (Meigen, 1829) (ME)**
It was split into two allopatric entities in the GMYC tree. One entity includes specimens from the Pyrenees, while the other includes specimens from the Alps and Scandinavia. Thus, opposite to what[29] have recently hypothesized, there might be a basis to reconsider *A. nicias* ssp. *judithi* (Gómez Bustillo, 1979) as a biologically meaningful taxon.

***Cupido alcetas* (Hoffmannsegg, 1804) (ME, lumped) - *C. decolorata* (Staudinger, 1886) (lumped)**
The two taxa are usually regarded as well-established species, including by fauna Europaea. Our GMYC analysis highlights an interesting pattern: the Romanian specimens of *C. alcetas* are recovered as a distinct entity, while the Iberian specimens are lumped with the Romanian *C. decolorata*. Within the lumped entity, the Iberian *alcetas* share DNA barcodes with the Romanian *decolorata*. The two species can be distinguished based on external morphology (especially in males)[15,20,27], and are reported to display certain differences in the male genitalia[21].

***Cupido lorquinii* (Herrich-Schäffer, 1850) (lumped)**

It was split into two entities in the GMYC tree. One includes specimens that can be assigned both to typical *C. lorquinii* and to the taxon *C. carswelli* Stempffer, 1927 (our dataset includes *carswelli* samples from the type locality, Sierra de Espuña). This is a rather unexpected result since the taxon *carswelli* was usually considered to be a distinct species[15,20,21,27,65] or was regarded as conspecific with *C. minimus*[29,66]. However,[49] considered *carswelli* as a subspecies of *C. lorquinii*.

Fauna Europaea considers *carswelli* as a subspecies of *C. minimus*.

***Cupido minimus* (Fuessly, 1775) (lumped)**

This species was split into two entities in our GMYC analysis. One of the entities consists of specimens belonging to the taxon *carswelli*, lumped together with several specimens of *C. lorquinii*. According to Fauna Europaea, *carswelli* is a subspecies of *C. minimus*, but *carswelli* was often treated as a distinct species (see text under *C. lorquinii*).

The taxa *minimus*, *carswelli* and *lorquinii* require further studies. The current DNA barcode data suggest that *carswelli* is distinct from *minimus* and more closely related to *lorquinii* than to *minimus*, despite obvious differences in males (blue upperside in *lorquinii* and dark brown in *carswelli*). The relationship among *minimus, lorquinii* and *carswelli* requires deeper molecular studies.

***Cupido osiris* (Meigen, 1829) (ME)**

Cupido osiris was split into three entities in the GMYC tree. Two occur in sympatry in Romania as well as in Iberia while a third entity was found only in Iberia where it occurs in relative proximity (but over 50 km) to sites occupied by the other two entities.

***Cyaniris semiargus* (Rottemburg, 1775) (ME)**

It was split into two allopatric entities in the GMYC tree. While one is widespread from Iberia to Romania, the other consists of two specimens from southern Spain (Huelva, Andalusia).

***Eumedonia eumedon* (Esper, 1780) (ME)**

It was split into three allopatric entities in the GMYC tree: one occurs in Iberia (excluding the Pyrenees), another appears restricted to the Pyrenees, and a third one was found in Romania.

***Favonius quercus* (Linnaeus, 1758) (ME)**

It was split into two entities in the GMYC tree. Based on our sampling, one of these entities occurs in central and southern Spain where it locally occurs in sympatry with the more widespread entity that ranges from western Iberia to Crimea.

***Glaucopsyche alexis* (Poda, 1761) (ME)**

It was split into two allopatric entities in the GMYC tree. One of these entities consists of a single specimen originating from south-eastern Romania (Dobrogea) while the other appears widespread in Europe, including south-eastern Romania.

***Kretania pylaon* (Fischer von Waldheim, 1832) (lumped) - *K. sephirus* (Frivaldzky 1835) (lumped)**

These two taxa are lumped in our GMYC analysis and *sephirus* is recovered as paraphyletic with respect to *pylaon*. However, it should be noted that our dataset includes only one specimen of *pylaon* from the study of[11] and the *sephirus* specimens originate exclusively from Romania. Further sampling is needed, but these results already indicate the close relationship between *sephirus* and *pylaon*.

The taxon *sephirus* has often been regarded as a subspecies of *K. pylaon*[15,20,21,24,25,27], but also as a distinct species[26,40,49]. The relationships between these two taxa, as well as two other [*hesperica* (Rambur, 1839) and *trappi* Verity, 1927] are still subject to debate. Fauna Europaea accepts *sephirus* as a species.

***Lycaena alciphron* (Rottemburg, 1775) (ME)**
It was split into two allopatric entities in the GMYC tree. One of these entities appears to correspond to the subspecies *gordius* (Sulzer, 1776) occurring in south-western Europe[29] while the other has currently been sampled only from Romania.

***Lysandra bellargus* (Rottemburg, 1775) (lumped)**
It was split into two entities in the GMYC tree, one being lumped together with some specimens of *L. coridon* and *L. albicans*. *Lysandra bellargus* is usually readily distinguishable from the other two species based on external morphology. All three species are known to produce natural hybrids[13]. A phenomenon suggesting potential introgression from *L. coridon* into *L. bellargus* was reported from Romania[12] and confirmed in a more recent study[67].

***Lysandra coridon* (Poda, 1761) (lumped) - *L. hispana* (Herrich-Schäffer, 1851) (lumped) - *L. albicans* (Gerhard, 1851) (lumped) - *L. caelestissima* (Verity, 1921) (lumped)**
Specimen identifications within this group (and especially those of *L. hispana* and *L. albicans*) are often difficult based on morphology and even when corroborated with taxon distribution (contrasting opinions have been expressed by various researchers). The identifications used here should be taken as tentative.
These taxa display a complex pattern in our GMYC analysis: *L. albicans* is split into four entities, *L. coridon* and *L. hispana* are split into three entities and *L. caelestissima* is lumped with some *L. hispana* and *L. albicans*. However, none of the entities mentioned above contained exclusively specimens attributable to a single taxon. The taxa *coridon*, *hispana* and *albicans* have most often been treated as distinct species[15,20,21,24,26,27]. Opinions vary more regarding *caelestissima*, with some authors considering it as a subspecies of *coridon*[15,21,24,26,29] while others regard it as a distinct species[20,27,40,49]. Due to their morphological similarity and variability, the boundaries between the geographic ranges of *hispana* and *albicans* are not well understood. Fauna Europaea accepts all four taxa as distinct species. All are notorious for producing natural hybrids in various combinations[13]. Actually, samples 070207MC50 (collected in the Teruel province) and RVcoll.08-L990 (collected in the Cuenca province) present in our dataset have external morphology reminding of potential hybrids between *L. caelestissima* and *L. albicans*. Such hybrids are usually attributed to the taxon *caerulescens* (Tutt, 1909) and are known to appear more or less regularly in the provinces of Cuenca, Guadalajara and Teruel[29].
DNA barcodes alone are not sufficient to disentangle the complex relationships and likely introgression phenomena occurring among all four taxa. For a multilocus analysis see[67].

***Pseudophilotes baton* (Bergsträsser, 1779) (lumped) - *P. panoptes* (Hübner, 1813) (ME, lumped) - *P. vicrama* (Moore, 1865) (lumped)**
These are taxa with disputed taxonomic status and similar morphology. Both *vicrama* and *panoptes*, have sometimes been treated as subspecies of *P. baton*[20,21,68]. Most recent authors consider all three taxa as separate species[15,24,26,27,29] and this position was adopted by Fauna Europaea.
In our GMYC analysis, none of the taxa is recovered as a distinct entity: *panoptes* is split into two entities, one clustering with *vicrama* and the other clustering with *baton*.

Although not recovered as a single GMYC entity, *P. vicrama* forms a monophyletic group sister to most specimens of *panoptes*. The monophyly of *panoptes* and *baton* is affected by three Iberian specimens identified as *panoptes* (from Valencia and Castilla y León provinces). These three specimens were identified based on morphology and taking into account the accepted distributions of *panoptes* and *baton*. It should, however, be noted that external morphology is not 100% reliable in separating these two taxa and the male genitalia are also reported to be extremely similar[21]. Actually, the exact Iberian distribution of *P. baton* and *P. panoptes* is not clearly known due to morphological similarities[29]. Some authors consider that *baton* and *panoptes* hybridize[68]. Thus, the topic requires further studies to better document the observed pattern and clarify if the distribution of *baton* is actually wider than previously thought, or if we deal with other phenomena such as genetic introgression.

***Plebejus argus* (Linnaeus, 1758) (ME)**
It was split into four entities in the GMYC tree. One of the entities seems to be widely distributed from Iberia to Crimea. The other three entities were identified only in Iberia: two appear sympatric and were detected only in the Iberian Mountain System in eastern Spain, while the other occurs in the Sierra Nevada Mountains in southern Spain.

***Plebejus idas* (Linnaeus, 1761) (ME, lumped) - *P. argyrognomon* (Bergsträsser, 1879) (lumped)**
The two taxa have usually been regarded as distinct species and they are treated accordingly in Fauna Europaea. In our GMYC analysis, *P. idas* is split into three entities, one of which is lumped with *P. argyrognomon*. One of the *P. idas* entities consists of a single specimen from Aragón, Spain.
It is worth noting that, within the lumped entity, *P. argyrognomon* and *P. idas* form different clades.

***Polyommatus amandus* (Schneider, 1792) (ME)**
It was split in two entities in the GMYC tree. They appear to have a disjoint western-eastern distribution and come into vicinity in southern Germany, where they are separated by ca. 90 km.

***Polyommatus celina* (Austaut, 1879) (ME)**
It was split into two entities in the GMYC tree. Until recently, the taxon *celina* was treated as a subspecies of *P. icarus* and distinguished on the basis of minor morphological differences in wing pattern. It has sometimes even been considered as a form of *P. icarus*[15]. Recent studies[69,70] showed that *P. celina* is a distinct species, a view accepted by Fauna Iberica[29]. Thus, although currently not present in Fauna Europaea, we consider *P. celina* as a species. *Polyommatus icarus* and *P. celina* are widely distributed and parapatric, with a contact zone in southern and central Iberian Peninsula.
The entities recovered by GMYC correspond to some of the main genetic lineages reported by[70].

***Polyommatus damon* (Denis & Schiffermüller, 1775) (ME)**
It was split into two allopatric entities in the GMYC tree. Interestingly, one entity includes specimens from the Cantabrian Mountains, as well as southern Germany (Bavaria), while the other entity appears in the Iberian Mountain System (eastern Spain) and the Pyrenees.

***Polyommatus daphnis* (Denis & Schiffermüller, 1775) (ME)**

It was split into two allopatric entities in the GMYC tree. Based on the available samples, one of the entities appears restricted to Iberia, while the other ranges from Germany to Crimea (Ukraine).

***Polyommatus dolus* (Hübner, 1823) (lumped) - *P. fulgens* (Sagarra, 1925) (lumped) - *P. fabressei* (Oberthür, 1910) (lumped)**
The taxon *fulgens* has often been regarded as conspecific with *P. dolus*[15,20,21,27]. However, recent molecular and karyological data support the species status of *fulgens*[2,71]. Fauna Europaea and Fauna Iberica accept it as a species.
Polyommatus dolus, P. fulgens and *P. fabressei* are recovered as a single entity in our GMYC analyses. Thus, the DNA barcodes appear to be insufficient to distinguish among these closely related taxa. However, when additional DNA data is included, the three taxa can be separated[2,73].

***Polyommatus dorylas* (Denis & Schiffermüller, 1775) (lumped) - *P. golgus* (Hübner, 1813) (lumped)**
The two taxa are lumped in a single entity in the GMYC analysis. They are closely related, *P. golgus* being monophyletic and *P. dorylas* being paraphyletic with respect to *P. golgus*. Both taxa are accepted as distinct species by most authors, as well as by Fauna Europaea and Fauna Iberica.

***Polyommatus escheri* (Hübner, 1823) (ME)**
This taxon was split into two entities in the GMYC tree. These entities appear to be sympatric at least in the Iberian Mountain System in eastern Spain and in the Cantabrian Mountains.

***Polyommatus icarus* (Rottemburg, 1775) (ME)**
It was split in three entities in the GMYC tree. The Iberian taxon *abdon* Aistleitner & Aistleitner, 1994 and the Balkanic taxon *andronicus* Coutsis & Ghavalás, 1995 have been separated from *P. icarus* and raised to species rank by some authors[73,74]. However, they are not treated as species by most synthesis works dealing with European butterflies, including Fauna Europaea which, although it briefly comments on their controversial status, considers them as synonyms of *P. icarus*. Our dataset includes putative specimens belonging to *abdon* and *andronicus*, but the GMYC entities lump them together with specimens of *P. icarus*. A recent study[70] documented the presence of four main genetic lineages of *P. icarus* that were named: Palaearctic + Iberia-Italy, Crete, Sierra Nevada, Alicante-Provence. These correspond to the GMYC entities recovered here, with the mention that the GMYC lumped together two of the lineages (“Sierra Nevada” and “Alicante-Provence”).

***Polyommatus ripartii* (Freyer, 1830) (ME, lumped) - *P. nephohiptamenos* (Brown & Coutsis, 1978) (lumped) - *P. admetus* (Esper, 1783) (lumped)**
All three taxa are accepted as species by Fauna Europaea. *Polyommatus ripartii* was split into two entities by the GMYC analysis. If compared to the three main genetic lineages (Eurasian, West-European and Balkan) recently documented by[75], it appears that one GMYC entity corresponds to the West-European lineage, while the other merges the Eurasian and Balkan lineages, as well as *P. admetus* and *P. nephohiptamenos*.
The taxonomic status of *P. nephohiptamenos* is debatable: several authors treated it as species[15,26,27] but other recent works suggest that it is conspecific with *P. ripartii*[24]. Fauna Europaea accepts *nephohiptamenos* as a species.

***Polyommatus thersites* (Cantener, 1835) (ME)**

It was split into two allopatric entities in the GMYC tree. Based on the available samples, one of the entities appears restricted to Iberia, while the other ranges from France (Massif Central) to eastern Romania.

***Polyommatus violetae* (Gómez Bustillo, Expósito & Martínez, 1979) (ME)**
The status of *P. violetae* has been subject to debate as it has been suspected to represent a subspecies of *P. ripartii* or *P. fabressei*[15,27]. A recent study[2] presented compelling evidence in favour of the species status of *violetae*. Fauna Europaea, as well as other authors[24,26,29,40,49] treat it as a distinct species.
P. violetae was split into two entities in the GMYC tree. One corresponds to the nominotypical subspecies, while the other (consisting of specimens from Sierra de la Sagra) belongs to subspecies *subbaeticus* initially described as a subspecies of *P. fabressei*[2,76].

***Satyrium spini* (Fabricius, 1787) (ME)**
This taxon was split into three geographically structured allopatric entities (Iberia, Germany, Romania) in the GMYC tree.

***Scolitantides orion* (Pallas, 1771) (ME)**
It was split into two allopatric entities in the GMYC tree. One entity was found in Spain, while the other occurs in Germany and Romania.

HESPERIIDAE
***Carcharodus alceae* (Esper, 1780) (ME)**
It was split into two allopatric entities in the GMYC tree. While one is widespread from Iberia to Romania, the other was restricted to a limited area in southern Romania.

***Carcharodus floccifera* (Zeller, 1847) (lumped) - *C. orientalis* Reverdin, 1913 (lumped)**
Due to their high similarity in external characters, the taxon *C. orientalis* has sometimes been regarded as a subspecies of *C. floccifera*[21], but more often as a different species[20,24,26,27,40]. Fauna Europaea treats this taxon as a valid species.
In our GMYC analysis, *C. floccifera* and *C. orientalis* are lumped in a single entity and we observed the same pattern that has been recently reported based exclusively on Romanian material[12]: *C. floccifera* appears as paraphyletic with respect to *C. orientalis* and, although not sharing barcodes, the minimum genetic distance between the two taxa is 0.46%.
Note that, according to[16], the correct name should be *Carcharodus floccifer* (Zeller, 1847).

***Carcharodus lavatherae* (Esper, 1783) (ME)**
It was split into two allopatric entities (disjoint distribution between Iberia and Romania) in the GMYC tree. If the two entities were to be assumed of interest for nomenclatural purposes, a comparison with specimens from France would be needed as the type of this species originated in southern France.

***Pyrgus alveus* (Hübner, 1803) (ME, lumped) - *P. bellieri* (Oberthür, 1910) (lumped) - *P. warrenensis* (Vérity, 1928) (lumped)**
The taxon *bellieri* was described by Oberthür in 1910, as a subspecies of *P. alveus*. However, most revisions after 1970 recognized these taxa as different species[15,20,21,27,23,24,26,49]. Differences in the genitalia and in chromosome number have been documented[77].
The taxon *warrenensis* has sometimes been considered as a subspecies or form of *P. alveus*[20,21]. Many reviews treat *warrenensis* as a distinct species despite its morphological similarity to *P. alveus*[15,24,26,27,78].

The three German specimens in our dataset are recovered by the GMYC analysis within the same entity as *P. bellieri* and several *P. alveus*. It should however be noted that they form a monophyletic group, but are closely related to *P. alveus* (minimum genetic distance of 0.9%). In our dataset, *P. alveus* was split into two GMYC entities: one comprised exclusively of Iberian specimens, while the other is lumped with *P. bellieri* and *P. warrenensis* and occurs in central and eastern Europe. Two infraspecific names have been proposed for the Iberian populations, i.e. ssp. *accretus* (Verity, 1925) (northern mountains) and ssp. *centralhispaniae* Warren, 1926 (central and southern Iberia). These distributions do not completely match the patterns derived from our analyses. Samples from additional areas (e.g. from France and Italy) are required to evaluate the taxonomic status of the Iberian populations.

***Pyrgus andromedae* (Wallengren, 1853) (ME)**
This taxon was split into two allopatric entities in the GMYC tree. One entity includes exclusively Pyrenean specimens, while the other unites Alpine, Carpathian and Scandinavian specimens.

***Pyrgus armoricanus* (Oberthür, 1910) (ME)**
This taxon was split into two entities in the GMYC tree. One seems to be widely distributed from Iberia to Romania, while the other was detected only in south-eastern Romania, in local sympatry with the previous one. This separation seems to correspond to the conclusions of[78] who considered that subspecies *persicus* Reverdin, 1913 occurs in south-eastern Europe (extending eastwards to the Caucasus and Iran). Although[78] based the separation of *persicus* on genitalic differences with respect to nominal *armoricanus,* this taxon has usually not been raised to species rank in the synthesis works dealing with the European butterflies.

***Pyrgus cinarae* (Rambur, 1839) (ME)**
This taxon was split into two allopatric entities in the GMYC tree. One of these entities was comprised exclusively of Iberian specimens corresponding to the geographically isolated taxon *clorinda* (Warren, 1927). Recent morphological and molecular studies treat this taxon as a subspecies of *P. cinarae* because of any lack of evidence for differentiation beyond high genetic divergence[79,80]. Fauna Europaea considers *clorinda* a synonym of *cinarae*.

***Spialia orbifer* (Hübner, 1823) (lumped) - *S. sertorius* (Hoffmannsegg, 1804) (ME, lumped)**
In older literature, *S. orbifer* was sometimes treated as a subspecies of *S. sertorius*[20,21]. More recently, it is usually raised to species rank[15,24,26,27,49]. *Spialia sertorius* and *S. orbifer* are parapatric, with a contact zone in central Europe. Fauna Europaea accepts *orbifer* as a species. Interestingly, in our GMYC analysis, *S. orbifer* was lumped with five specimens of *S. sertorius* originating from the Sierra Nevada Mountains (southern Spain). Although placed within the same entity as *S. orbifer*, these five specimens formed a rather well-differentiated clade (minimum genetic distance from *S. orbifer* is 1.1%) that is sister to *S. orbifer*. This unexpected pattern is requiring further studies.

***Thymelicus acteon* (Rottemburg, 1775) (ME)**
It was split into two entities in the GMYC tree. One was obtained from a restricted area in Spain, while the other appears from southern Iberia to central and eastern Europe. The two entities have been detected in sympatry in Spain.

***Thymelicus lineola* (Ochsenheimer, 1808) (ME)**

This taxon was split into four entities in the GMYC tree: one appears confined to Iberia, two were found only in the Pyrenees (where they occur in sympatry), while the fourth seems to be distributed across central and eastern Europe.

Thymelicus sylvestris **(Poda, 1761) (ME)**

This taxon was split into four entities in the GMYC tree: one appears restricted to Iberia (where it is widespread); another occurs in Romania, southern Germany and also in southern Spain (Sierra Nevada, Granada), where it occurs in sympatry with the Iberian lineage; a third entity was detected in south-eastern Romania (Dobrogea), while the fourth was found in other parts of Romania. The correct interpretation of this complex pattern requires further studies. A recent study[12] reported three well-differentiated lineages in Romania, two detected in sympatry.

Supplementary discussion on taxa with past or currently debated taxonomy not treated in the previous list

PAPILIONIDAE

Iphiclides podalirius **(Linnaeus, 1758) -** ***I. p. feisthamelii*** **(Duponchel, 1832)**

Iphiclides podalirius is a relatively common species widely distributed from North Africa and western Europe to western China. The populations from North Africa, Iberia and south-western France have often been assigned to the taxon *feisthamelii* that has a controversial status[69]. Based on molecular and morphological data, recent studies suggested that this taxon may represent a distinct species[69,81].

Our results indicate that *COI* cannot distinguish between European populations of the two taxa, but the nuclear marker analysed (*ITS2*), as well as the morphology of the male genitalia, highlighted the distinctness of the taxon *feisthamelii* (Fig. 6 in the main manuscript). The discordance between the mitochondrial and nuclear markers and the distribution of the sequenced samples (Fig. 6e) support the hypothesis of mitochondrial DNA introgression from *podalirius* into *feisthamelii* that is now apparently fixed in the Iberian Peninsula.

PIERIDAE

Euchloe crameri **Butler, 1869 -** ***E. simplonia*** **(Freyer, 1829)**

Both *E. crameri* and *E. simplonia* have sometimes been considered as conspecific with *E. ausonia*[20,21]. Recent reviews suggest that they represent distinct species[15,24,26,27,28] . Some authors have shown the distributions of *E. ausonia*, *E. crameri* and *E. simplonia* on the same map due to the challenge of morphology-based identifications[24]. On the other hand, other authors produced individual maps (not always congruent) for their distribution in Europe[15,26,49], or in Italy[23].

The taxa *crameri* and *simplonia* were recovered as distinct GMYC entities.

Leptidea sinapis **(Linnaeus, 1758) -** ***Leptidea reali*** **Reissinger, 1990 -** ***Leptidea juvernica*** **Williams, 1946**

Leptidea reali was first described[82] from the French Pyrenees in 1988, after 230 years of confusion with *L. sinapis*. Recent genetic and cytogenetic studies have revealed that the pair *L. sinapis* - *L. reali* actually involves a triplet of species. The third species (*L. juvernica*) is apparently synmorphic with *L. reali* and can be reliably distinguished based exclusively on DNA data[83]. Available genetic data indicate that: *L. sinapis* is widespread in the western

Palaearctic where it often occurs in sympatry with the other two species; *L. reali* is confined to south-western Europe (Spain, southern France and parts of Italy), while *L. juvernica* replaces it in the rest of the western Palaearctic. Potential contact zones between *L. juvernica* and *L. reali* may exist in south-eastern France and north-western Italy. Recent female mate acceptance studies support the existence of a triplet of cryptic species[84].
Fauna Europaea does not currently include *L. juvernica* among the species in Europe. However, the evidence in favour of the species status of this taxon is in our opinion strong. *Leptidea sinapis*, *L. reali* and *L. juvernica* were recovered as distinct GMYC entities.

***Pontia edusa* (Fabricius, 1777)**
The taxon *edusa* has sometimes been considered to be conspecific with *P. daplidice*, the two having virtually identical morphology[20,21,26]. However, several recent reviews, supported by molecular data[85,86,87] split them into two cryptic species with parapatric distribution[13,15,23,24,27,87]. A study based on allozyme markers suggests that the two taxa display a contact zone in central Europe (northern Italy), where introgression seems to occur[86], but hybrid offspring appear to suffer from low viability[88].
In our analysis, *daplidice* and *edusa* are recovered as different GMYC entities with a minimum genetic distance of 8.2%.

LYCAENIDAE
***Agriades zullichi* Hemming, 1933**
This taxon has sometimes been considered as a subspecies of *A. glandon*[15,20,21]. A study[89] provided compelling morphological and geographical evidence in favour of the species status of *zullichi*. Most recent works regard *zullichi* as a distinct species[24,26,27,40,49]. The GMYC model recovered *A. zullichi* as a distinct entity.

***Aricia artaxerxes* (Fabricius, 1793)**
A study[20] considered that Scottish populations of this taxon represent a subspecies of the morphologically very similar *A. agestis*, while other European populations have been regarded as a distinct species (*A. allous*). In subsequent publications, most authors treated *A. artaxerxes* as a species different from *A. agestis*, but different populations were often described as subspecies and occasionally raised to species rank[64]. The GMYC model recovered *A. artaxerxes* as a distinct entity.

***Aricia cramera* (Eschscholtz, 1821)**
It has often been regarded as a subspecies of *A. agestis*[15,21,26,27,29] but also as a distinct species[20,24,40]. Our results are consistent with the morphological differences between the male genitalia of these two taxa[21,90], as well as with previous molecular analyses[64], all of which support the species status of *A. cramera* as presently recognised in Fauna Europaea and Fauna Iberica. The GMYC model recovered *A. cramera* as a distinct entity.

***Aricia montensis* Verity, 1928**
This taxon has been considered either as a subspecies of *A. artaxerxes*[15,21,24,26], or as a different species[29]. Recent research suggests that *montensis* may represent a different species[64] and our results support this view. Fauna Europaea accepts the taxon *montensis* as species and our GMYC analysis recovered it as an entity.

***Iolana debilitata* (Schultz, 1905)**
Our sampling includes exclusively Iberian specimens of *Iolana*. Based on compelling evidence,[91] suggested that the Iberian populations of *Iolana iolas* (Ochsenheimer 1816) may

actually belong to a distinct species, *Iolana debilitata*. [29] also consider that the Iberian populations belong to *I. debilitata*. We are preparing a detailed study on this taxon and preliminary results support the view of [91]. Thus, we tentatively use the name *debilitata* for the Iberian specimens in our dataset. Fauna Europaea lists *I. iolas* as the only species on the continent.

Kretania hesperica **(Rambur, 1839)**
This Iberian endemic taxon was formerly considered as a subspecies of *K. pylaon* (e.g.[15,20,21,27]), but at present it is generally accepted as a distinct species[1,24,26,29,40,49], in agreement with our results that recovered it as an entity.

Lycaena bleusei **(Oberthür, 1884)**
This taxon has often been treated as a subspecies of *Lycaena tityrus* (Poda, 1761)[15,20,26,27,49], but also as a distinct species[24,28,29]. Fauna Europaea accepts *bleusei* as a species and our results, in line with the findings of[92], support this view.

HESPERIIDAE
Carcharodus tripolina **(Verity, 1925)**
This taxon is externally extremely similar to *C. alceae*. It was actually described in 1925 as a subspecies of *C. alceae* and, for over half a century, was considered as such by most authors. The taxon was raised to species rank by[93]. Most recent reviews treat it as a distinct species distinguished from *C. alceae* based on genitalic morphology[15,24,26,27,29]. They are parapatric taxa with a contact zone in southern Iberian Peninsula. Our GMYC analysis recovered the taxon *tripolina* as an entity.

Pyrgus cirsii **(Rambur, 1839)**
This taxon is externally similar to *P. carlinae*, but displays differences in the male and female genitalia[21,94]. *Pyrgus cirsii* was sometimes treated as a subspecies of *P. carlinae*[15,26], although many older and recent works consider them as different species[20,21,24,27,49,95]. They are parapatric taxa and, according to[24] their ranges slightly overlap in the south-western Alps. Fauna Europaea recognizes both taxa as species, but our dataset does not include *P. carlinae*.

Pyrgus malvoides **(Elwes & Edwards, 1897)**
The taxon *malvoides* has often been regarded as a subspecies of *P. malvae*[15,20,21,26,27], but also as a distinct species[29,40,49,95]. Fauna Europaea regards *malvoides* as distinct species, a decision supported by our analyses that recovered it as an entity.

References

1. Karsholt, O. & Van Nieukerken, E.J. (eds.). Lepidoptera, Papilionoidea. Fauna Europaea version 2.6, http://www.faunaeur.org (2013).

2. Vila, R., Lukhtanov, V.A., Talavera, G., Gil, F. & Pierce, N.E. How common are dot-like distributions? Taxonomical oversplitting in western European *Agrodiaetus* (Lepidoptera: Lycaenidae) revealed by chromosomal and molecular markers. *Biol. J. Linn. Soc.* **101,** 130-154 (2010).

3. Braby, M.F. Provisional checklist of genera of the Pieridae (Lepidoptera: Papilionidae). *Zootaxa* **832,** 1-16 (2005).

4. Braby, M.F., Vila, R. & Pierce, N.E. Molecular phylogeny and systematics of the Pieridae (Lepidoptera: Papilionoidea): higher classification and biogeography. *Zool. J. Linn. Soc.* **147,** 239-275 (2006).

5. Wahlberg, N. The higher classification of Nymphalidae. http://www.nymphalidae.net (visited in August 2014) (2014).

6. Talavera, G., Lukhtanov, V.A., Pierce, N.E. & Vila, R. Establishing criteria for higher level taxonomic classification using molecular data: an example with blue butterflies (Lepidoptera, Lycaenidae). *Cladistics* **29 (2),** 166-192 (2013).

7. Warren, A.D., Ogawac, J.R. & Brower, A.V.Z. Phylogenetic relationships of subfamilies and circumscription of tribes in the family Hesperiidae (Lepidoptera: Hesperioidea). *Cladistics* **24,** 1-35 (2008).

8. Todisco, V., Gratton, P., Cesaroni, D. & Sbordoni, V. Phylogeography of *Parnassius apollo*: hints on taxonomy and conservation of a vulnerable glacial butterfly invader. *Biol. J. Linnean. Soc.* **101 (1),** 169-183 (2010).

9. Gratton, P. Phylogeography and conservation genetics of *Parnassius mnemosyne* L., 1758 (Lepidoptera, Papilionidae). 1-93. (Doctoral thesis, Universita degli Studi di Roma "Tor Vergata", Rome, 2006).

10. Gratton, P., Konopinski, M.K. & Sbordoni, V. Pleistocene evolutionary history of the Clouded Apollo (*Parnassius mnemosyne*): genetic signatures of climate cycles and a 'time-dependent' mitochondrial substitution rate. *Mol. Ecol.* **17 (19),** 4248-4262 (2008).

11. Lukhtanov, V.A., Sourakov, A, Zakharov, E.V. & Hebert, P.D.N. DNA barcoding Central Asian butterflies: increasing geographical dimension does not significantly reduce the success of species identification. *Mol. Ecol. Resour.* **9 (5),** 1302-1310 (2009).

12. Dincă, V., Zakharov, E.V., Hebert, P.D.N. & Vila, R. Complete DNA barcode reference library for a country's butterfly fauna reveals high performance for temperate Europe. *Proc. R. Soc. B.* **278,** 347-355 (2011).

13. Descimon, H. & Mallet, J. *Bad species*. In Settele, J., Shreeve, T.G., Konvicka, M. & Van Dyck, H. eds. *Ecology of Butterflies in Europe* (Cambridge University Press, 2009).

14. Alberti, B. Zur Frage der Hybridisation zwischen *Colias erate* Esp., *hyale* L. und *edusa* F. und ueber Umgrenzung der 3 Arten. *Mitt. Münch. Entomol. Ges.* **33,** 606-625 (1943).

15. Tolman, T. & Lewington, R. *Collins Butterfly Guide*. (Harper-Collins Publishers, London, 2008).

16. Dupont, P., Luquet, G.Chr., Demerges, D. & Drouet, E. *Révision systématique, taxinomique et nomenclaturale des Rhopalocera et des Zygaenidae de France métropolitaine. Conséquences sur l'acquisition et la gestion des données d'inventaire.* Rapport MMNHN-SPN 2013-19, 201 p (2013).

17. Geiger, H.J. & Scholl, A. Systematics and evolution of holarctic Pierinae (Lepidoptera) - an enzyme electrophoretic approach. *Experientia* **41,** 24-29 (1985).

18. Geiger, H. & Shapiro, A.M. Genetics, systematics and evolution of holarctic *Pieris napi* species group populations (Lepidoptera, Pieridae). *J. Zoolog. Syst. Evol. Res.* **30,** 100-122 (1992).

19. Porter, A.H. The *Pieris napi bryoniae* hybrid zone at Pont de Nant, Switzerland: Broad overlap in the range of suitable host plants. *Ecol. Entomol.* **22,** 189-196 (1997).

20. Higgins, L.G. & Riley, N.D. *A field guide to the butterflies of Britain and Europe*. (1st American edn. Houghton Mifflin, Boston, 1970).

21. Higgins, L.G. *The classification of European butterflies*. (Collins, London, 1975).

22. Higgins, L., Hargreaves, B. & Lhonoré, J. *Guide complet des papillons d'Europe et d'Afrique du nord. Paris*. (Delachaux et Niestlé, 1991).

23. Balletto, E., Bonelli, S. & Cassulo, L. Insecta Lepidoptera Papilionoidea. In: *Checklist and distribution of the Italian fauna. 10,000 terrestrial and inland water species. Verona: Memorie del Museo Civico di Storia Naturale di Verona, Sez. Scienze della Vita, 2nd and revised edition.* (eds. Ruffo S & Stoch F, 2007).

24. Kudrna, O. et al. *Distribution atlas of butterflies in Europe*. (Gesellschaft für Schmetterlingsschutz, Halle, Germany, 2011).

25. Tshikolovets, V.V. *Butterflies of Eastern Europe, Urals and Caucasus*. (Tshikolovets Publications, 2003).

26. Tshikolovets, V.V. *Butterflies of Europe and the Mediterranean area*. (Tshikolovets Publications, 2011).

27. Lafranchis, T. *Butterflies of Europe*. (Diatheo, Paris, 2004).

28. García-Barros, E. et al. *Atlas of butterflies of the Iberian Peninsula and Balearic islands.* (Monografias S.E.A., 11, 2004).

29. García-Barros, E., Munguira, M.L., Stefanescu, C. & Vives Moreno, A. *Lepidoptera Papilionoidea*. En: *Fauna Ibérica*, vol. 37. (Museo Nacional de Ciencias Naturales, CSIC. Madrid, 2013).

30. Pintureau, B. Contribution à l'étude du genre *Arethusana* de Lesse (Lep. Satyridae) (suite). *Alexanor* **9 (7),** 317-324 (1976).

31. Pintureau, B. Contribution à l'étude du genre *Arethusana* De Lesse (Lep. Satyridae). Résumé des parties II et III et compléments. *Alexanor* **10 (3),** 98-104 (1977).

32. Pintureau, B. Etude des genitalia mâles d'*Arethusana* De Lesse et redescription d'*A. boabdil status nov*. (Lep. Satyridae). *Alexanor* **11 (3),** 105-110 (1979).

33. Olivares, J., Jutzeler, D. & Leestmans, R. *Arethusana boabdil* (Rambur, 1840) from the Province of Granada, Andalusia: a good species! (Lepidoptera: Nymphalidae, Satyrinae). *Linneana Belg.* **17 (4),** 135-154 (1999).

34. Coutsis, J. & Ghavalás, N. A recently discovered new locality for *Coenonympha leander* in Greece, and notes about the taxonomic position of the species-group taxon *Coenonympha orientalis* (Lepidoptera: Nymphalidae, Satyrinae). *Phegea* **33 (4),** 121-128 (2005).

35. Wiemers, M. Die Gattung *Coenonympha* Hübner, 1819, in Europa: Systematik, Ökologie und Schutz. *Oedippus* **25,** 1-42 (2007).

36. Schmitt, T. & Besold, J. Upslope movements and large scale expansions: the taxonomy and biogeography of the *Coenonympha arcania* - *C. darwiniana* - *C. gardetta* butterfly species complex. *Zool. J. Linn. Soc.* **159,** 890-904 (2010).

37. Boillat, H. *Coenonympha lyllus* Esper, 1805, spec. Rev. Une nouvelle approche taxinomique du complexe *pamphilus* (Lepidoptera Nymphalidae Satyrinae). *Alexanor* **22,** 243-309 (2003).

38. Dapporto, L., Bruschini, C., Dincă, V., Vila, R. & Dennis, R.L.H. Identifying zones of phenetic compression in West Mediterranean butterflies (Satyrinae): refugia, invasion and hybridization. *Divers. Distrib.* **18,** 1066-1076 (2012).

39. García-Barros, E. & Meneguz, M. Estructura de las escamas ventrales de las alas de Coenonympha Hübner, [1819], con especial referencia a *C. pamphilus* (L., 1758) y su morfotipo *lyllus* (Esper, 1805) (Lepidoptera: Nymphalidae). *SHILAP* **40,** 279-293 (2012).

40. Van Swaay, C. et al. *European Red List of Butterflies.* (Luxembourg: Publications Office of the European Union, 2010).

41. Warren, B.C.S. *Monograph of the genus* Erebia. (Trustees, Adlard and Son Ltd., London, 1936).

42. Mutanen, M. et al. Allopatry as a gordian knot for taxonomists: Patterns of DNA barcode divergence in Arctic-Alpine Lepidoptera. PLoS ONE **7 (10),** e47214 (2012).

43. Vila, M. & Björklund, M. The utility of the neglected mitochondrial control region for evolutionary studies in Lepidoptera Insecta. *J. Mol. Evol.* **58 (3),** 280-290 (2004).

44. Cupedo, F. Geographical variation and Pleistocene history of the *Erebia pandrose - sthennyo* complex (Nymphalidae; Satyrinae). *Nota lepid.* **30,** 329-353 (2007).

45. Albre, J., Gers, C. & Legal, L. Molecular phylogeny of the *Erebia tyndarus* (Lepidoptera, Rhopalocera Nymphalidae, Satyrinae) species group combining CoxII and ND5 mitochondrial genes: A case study of a recent radiation. *Mol. Phylogenet. Evol.* **47**, 196-210 (2008).

46. Leraut, P. Contribution à l´étude des Satyrinae de France (Lep. Nymphaldiae). *Ent. gall.* **2 (1),** 8-19 (1990).

47. Jutzeler, D. & Volpe, G. Confirmation de la dualité du “Petit Sylvandre” diagnostiquée par Leraut (1990). Part 2. *Linneana Belg.* **20 (5),** 193-206 (2006).

48. Gibeaux, C. Données sur la systématique et la répartition française présente et passée des *Hipparchia* du groupe *fagi. Alexanor* **24 (5),** 261-354 (2011).

49. Haahtela, T., Saarinen, K., Ojaleinen, P. & Aarnio, H. *Butterflies of Britain and Europe. A photographic guide.* (A&C Black Publishers Ltd, London, 2011).

50. Kudrna, O. *A revision of the genus* Hipparchia *Fabricius.* (E.W. Classey Ltd., Faringdon, Oxon, 1977).

51. Schmitt, T., Röber, S. & Seitz, A. Is the last glaciation the only relevant event for the present genetic population structure of the Medow Brown Butterfly *Maniola jurtina* (Lepidoptera: Nymphalidae)? *Biol. J. Linnean. Soc.* **85,** 419-431 (2005).

52. Dapporto, L. et al. Phylogeography and counter-intuitive inferences in island biogeography: evidence from morphometric markers in the mobile butterfly *Maniola jurtina* (Linnaeus) (Lepidoptera, Nymphalidae). *Biol. J. Linnean. Soc.* **98 (3),** 677-692 (2009).

53. Thomson, G. *The Meadow Brown butterflies, a study in genetics, morphology and evolution.* (Editions Thomson, Waterbeck, Scotland, 2011).

54. Nazari, V., Ten Hagen, W. & Bozano, G.C. Molecular systematics and phylogeny of the 'Marbled Whites' (Lepidoptera: Nymphalidae, Satyrinae, *Melanargia* Meigen). *Syst. Entomol.* **35,** 132-147 (2010).

55. Vandewoestijne, S., Nève, G. & Baguette, M. Spatial and temporal population genetic structure of the butterfly *Aglais urticae* L. (Lepidoptera, Nymphalidae). *Mol. Ecol.* **8,** 1539-1543 (1999).

56. Vandewoestijne, S., Baguette, M., Brakefield, P.M. & Saccheri, I.J. Phylogeography of *Aglais urticae* (Lepidoptera) based on DNA sequences of the mitochondrial COI gene and control region. *Mol. Phylogenet. Evol.* **31,** 630-646 (2004).

57. Niculescu, E.V. *Apatura ilia metis* Freyer et la notion de species in statu nascendi (Lepidoptera). *Bull. Soc. Entomol. Mulhouse,* Janvier-Mars, 7-14 (1980).

58. Tuzov, V.K. & Bozano, G.C. *Guide to the Butterflies of the Palaearctic Region. Nymphalidae part II. Tribe Argynnini: Boloria, Proclossiana, Clossiana.* (Omnes Artes, Milan, 2006).

59. Simonsen, T.J., Wahlberg, N., Warren, A.D. & Sperling, F.A.H. The evolutionary history of *Boloria* (Lepidoptera: Nymphalidae): phylogeny, zoogeography and larval-foodplant relationships. *Systematics and Biodiversity* **8,** 513-529 (2010).

60. Van Oorschot, H. & Coutsis, J.G. *The genus Melitaea Fabricius, 1807. Taxonomy and systematics with special reference to the male genitalia (Lepidoptera, Nymphalidae, Nymphalinae).* (Tshikolovets Publications, 2014).

61. Leneveu, J., Chichvarkhin, A. & Wahlberg, N. Varying rates of diversification in the genus *Melitaea* (Lepidoptera: Nymphalidae) during the past 20 million years. *Biol. J. Linnean. Soc.* **97,** 346-361 (2009).

62. Wahlberg, N. & Saccheri, I.S. The effects of Pleistocene glaciations on the phylogeography of *Melitaea cinxia* (Lepidoptera: Nymphalidae). *Eur. J. Entomol.* **104,** 675-684 (2007).

63. Montagud, S. & García-Alamá, J.A. *Mariposas diurnas de la Comunitat Valenciana (Papilionoidea & Hesperioidea)* (Generalitat Valenciana, Spain, 2010).

64. Sañudo-Restrepo, C.P., Dincă, V., Talavera, G. & Vila, R. Biogeography and systematics of *Aricia* butterflies (Lepidoptera, Lycaenidae). *Mol. Phylogenet. Evol.* **66,** 369-79 (2013).

65. Gil-T., F. *Cupido carswelli* (Stempffer, 1927): Morphology of its chrysalis and genitalia compared with those of *Cupido minimus* (Fuessly, 1775) and *Cupido lorquinii* (Herrich-Schäffer, 1847) (Lepidoptera, Lycaenidae). *Atalanta* **37 (1/2),** 150-160, 280-281 (2006).

66. Prieto, C.G., Munguira, M.L. & Romo, H. Morphometric analysis of genitalia and wing pattern elements in the genus *Cupido* (Lepidoptera, Lycaenidae): are *Cupido minimus* and *C. carswelli* different species? *Dtsch. Entomol. Z.* **56 (1),** 137-147 (2009).

67. Talavera, G., Lukthanov, V.A., Rieppel, L., Pierce, N.E. & Vila, R. In the shadow of phylogenetic uncertainty: the recent diversification of the *Lysandra* butterflies through chromosomal change. *Mol. Phylogenet. Evol.* **69 (3),** 469-478 (2013).

68. Mazel, R. *Pseudophilotes baton panoptes* Hübner, 1813 bona subspecies (Lepidoptera, Lycaenidae). *R.A.R.E.* **16 (2),** 38-47 (2007).

69. Wiemers, M. & Gottsberger, B. Discordant patterns of mitochondrial and nuclear differentiation in the Scarce Swallowtail *Iphiclides podalirius feisthamelii* (Duponchel, 1832) (Lepidoptera: Papilionidae). *Dtsch. entomol. Z.* **120 (3),** 111-115 (2010).

70. Dincă, V., Dapporto, L. & Vila, R. A combined genetic-morphometric analysis unravels the complex biogeographical history of *Polyommatus icarus* and *Polyommatus celina* Common Blue butterflies. *Mol. Ecol.* **20,** 3921-3935 (2011).

71. Lukhtanov, V.A., Vila, R. & Kandul, N.P. Rearrangement of the *Agrodiaetus dolus* species group (Lepidoptera, Lyeaenidae) using a new cytological approach and molecular data. *Insect. Syst. Evol.* **37,** 325-334 (2006).

72. Kandul, N.P. et al. Phylogeny of *Agrodiaetus* Hübner, 1822 (Lepidoptera: Lycaenidae) inferred from mtDNA sequences of COI and COII and nuclear sequences of EF1-a: karyotype diversification and species radiation. *Syst. Biol.* **53,** 278-298 (2004).

73. Kolev, Z. New data on the taxonomic status and distribution of *Polyommatus andronicus* Coutsis & Ghavalás, 1995 (Lycaenidae). *Nota lepid.* **28 (1),** 35-48 (2005).

74. Muñoz-Sariot, M.L. *Biología y ecología de los licénidos españoles.* (Published by the author, 2011).

75. Dincă, V., Runquist, M., Nilsson, M. & Vila, R. Dispersal, fragmentation and isolation shape the phylogeography of the European lineages of *Polyommatus* (*Agrodiaetus*) *ripartii* (Lepidoptera: Lycaenidae). *Biol. J. Linn. Soc.* **109,** 817-829 (2013).

76. Gil-T, F. & Gil-Uceda, T. *Agrodiaetus violetae* (Gómez-Bustillo, Expósito & Martínez, 1979): Morfología comparada y descripción de *Agrodiaetus fabressei subbaeticus* ssp. nov. del sureste de la Península Ibérica (Lepidoptera, Lycaenidae). *Boletín de la S.E.A.* **36,** 357-364 (2005).

77. Hernández-Roldán, J.L. & Munguira, M.L. Multivariate analysis techniques in the study of the male genitalia of *Pyrgus bellieri* (Oberthur 1910) and *P. alveus* (Hubner 1803) (Lepidoptera : Hesperiidae): species discrimination and distribution in the Iberian Peninsula. *Ann. Soc. Entomol. Fr.* **44,** 145-155 (2008).

78. De Jong, R. Systematics and geographic history of the genus *Pyrgus* in the Palaearctic region. *Tijdschr. entomol.* **115,** 1-121 (1972).

79. Hernández-Roldán, J.L. et al. Tracing the origin of disjunct distributions: a case of biogeographical convergence in *Pyrgus* butterflies. *J. Biogeography* **38 (10),** (2011).

80. Hernández-Roldán, J.L., Vicente, J.C. & Munguira, M.L. Natural history, immature stage morphology, and taxonomic status of the threatened skipper *Pyrgus cinarae* (Rambur, 1839) in the Iberian Peninsula (Lepidoptera: Hesperiidae). *Nota lepid.* **35 (1),** 3-18 (2012).

81. Coutsis, J. & van Oorschot, H. Differences in the male and female genitalia between *Iphiclides podalirius* and *Iphiclides feisthamelii,* further supporting species status for the latter (Lepidoptera: Papilionidae). *Phegea* **39 (1),** 12-22 (2011).

82. Réal, P. Lepidoptères noveaux principalement Jurassiens. *Mem Com Liaison Rech Ecofaun Jura* **4,** 17-24 (1988).

83. Dincă, V., Lukhtanov, V.A., Talavera, G. & Vila, R. Unexpected layers of cryptic diversity in wood white *Leptidea* butterflies. *Nat. Commun.* **2,** 324 (2011).

84. Dincă, V. et al. Reproductive isolation and patterns of genetic differentiation in a cryptic butterfly species complex. *J. Evol. Biol.* **26,** 2095-2106 (2013).

85. Geiger, H. & Scholl, A. *Pontia daplidice* (Lepidoptera, Pieridae) in Südeuropa – eine Gruppe von zwei Arten. *Mitt. Schweiz. entomol. Ges.* **55,** 107-114 (1982).

86. Porter, A.H., Wenger, R., Geiger, H., Scholl, A. & Shapiro, A.M. The *Pontia daplidice-edusa* hybrid zone in northwestern Italy. *Evolution* **51,** 1561-1573 (1997).

87. John, E., Wiemers, M., Makris, C. & Russell, P. The *Pontia daplidice* (Linnaeus, 1758) /*Pontia edusa* (Fabricius, 1777) complex (Lepidoptera: Pieridae): confirmation of the presence of *Pontia daplidice* in Cyprus, and of *Cleome iberica* DC. as a new host-plant for this species in the Levant. *Entomol. Gazette* **64,** 69-78 (2013).

88. Geiger, H., Descimon, H. & Scholl, A. Evidence for speciation within nominal *Pontia daplidice* (Linnaeus, 1758) in southern Europe (Lepidoptera: Pieridae). *Nota lepid.* **11,** 7-20 (1988).

89. Munguira, M.L. *Biología y biogeografía de los licénidos ibéricos en peligro de extinción (Lepidoptera, Lycaenidae)* (Doctoral thesis, Universidad Autónoma de Madrid, 1987).

90. Balletto, E., Toso, G. & Troiano, G. *Aricia cramera* (Erschscholtz, 1821) in Sardinia (Lycaenidae, Plebejinae). *Nota lepid.* **4,** 81-92 (1981).

91. Dumont, D. Révision du genre *Iolana* Bethune-Baker 1914 (Lepidoptera: Lycaenidae). *Linneana Belg.* **19 (8),** 332-358 (2004).

92. Casulo, L., Mensi, P. & Balletto, E. Taxonomy and evolution in *Lycaena* (subgenus *Heodes*) (Lycaenidae). *Nota lepid.*, Supplement **1,** 23-25 (1989).

93. De Jong, R. *Carcharodus tripolinus* Verity, stat. nov., une nouvelle espèce pour la faune d´Europe. Remarques au sujet de la notion d´espèce. *Linneana Belg.* **7,** 117-122 (1978).

94. Guillaumin, M. Détermination des femelles du genre *Pyrgus* (espèces françaises) d'après les caractéristiques de leurs genitalia. *Alexanor* **4,** 243-292 (1966).

95. Karsholt, O. & Razowski, J. *The Lepidoptera of Europe: A Distributional Checklist.* (Apollo Books Stenstrup, 1996).

Supplementary Annex 2

To facilitate the vizualization of spatial genetic patterns, we provide maps for the 83 species that were split into two to four entities by the GMYC model. The entities have also been indicated in Supplementary Fig. 5.

Note that the distance scale corresponds to longitudinally-oriented distances only.

The maps are listed alphabetically according to taxon name, and were generated with gvSIG 1.12.0 (http://www.gvsig.org), based on relief maps from Natural Earth (free vector and raster map data at www.naturalearthdata.com).

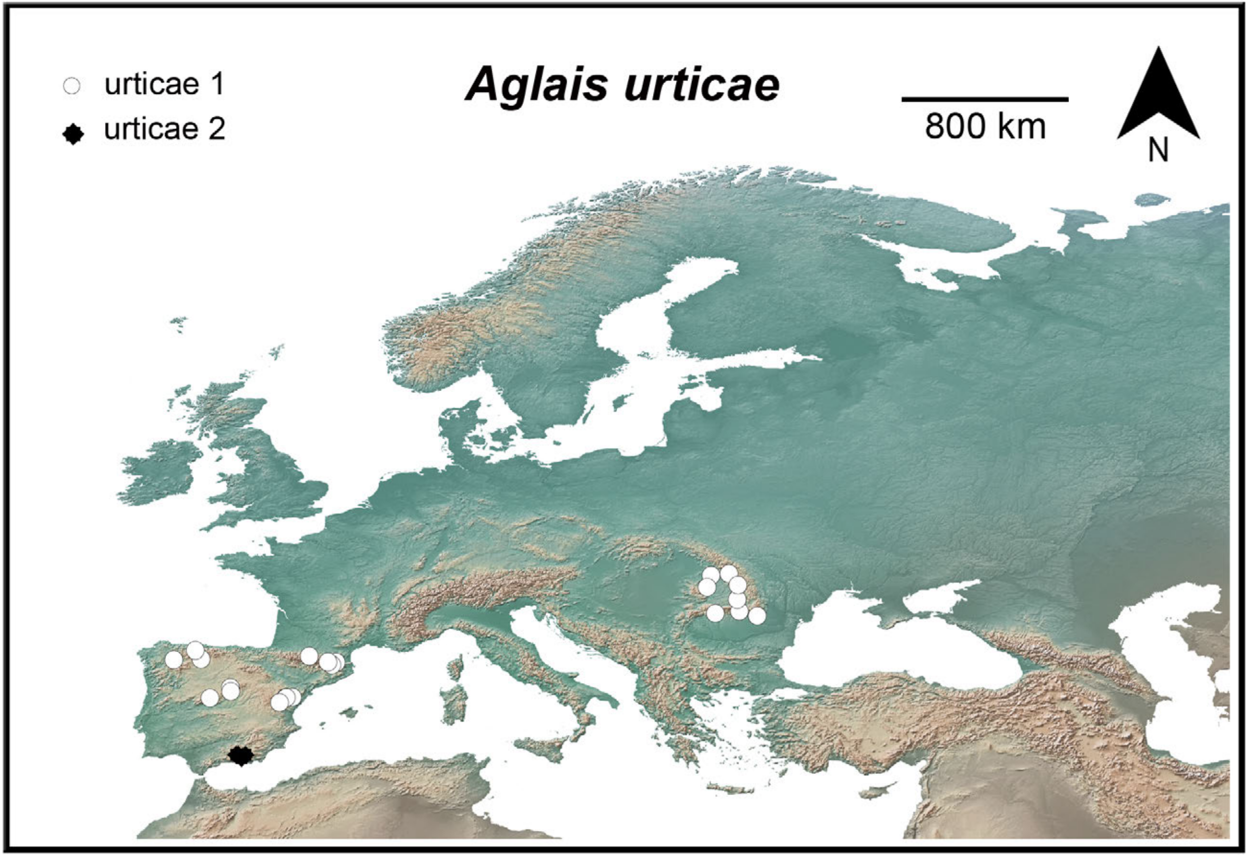

Aglais urticae
urticae 1
urticae 2
800 km
N

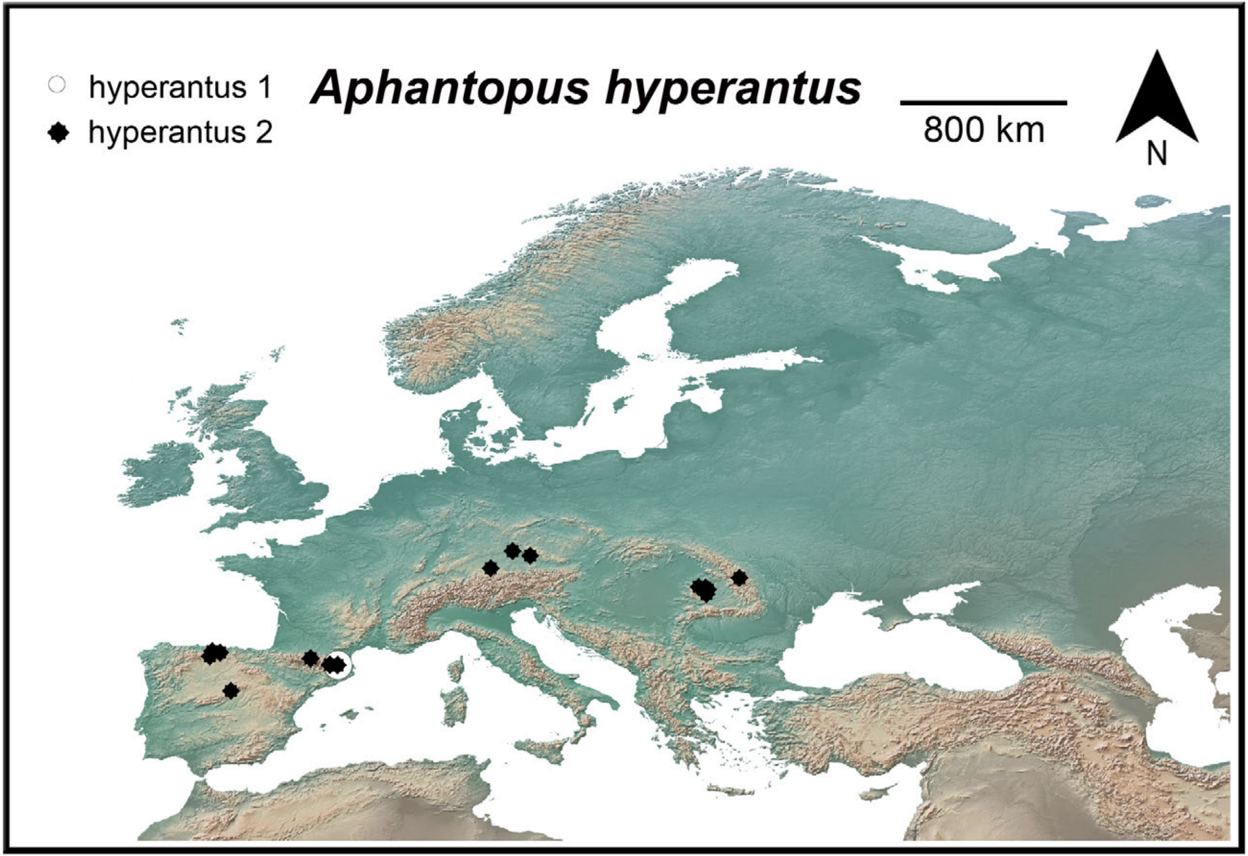
Aphantopus hyperantus
hyperantus 1
hyperantus 2
800 km
N

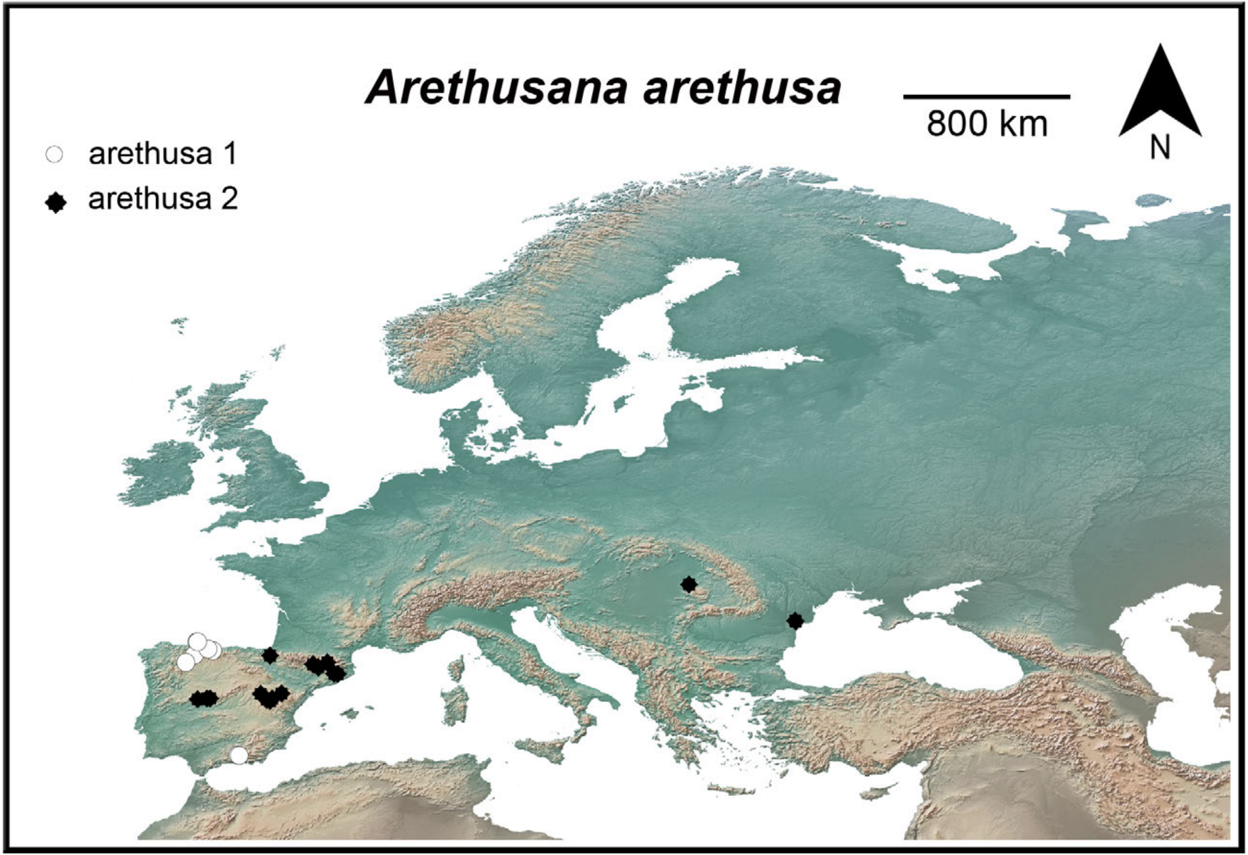
Arethusana arethusa
800 km
N
arethusa 1
arethusa 2

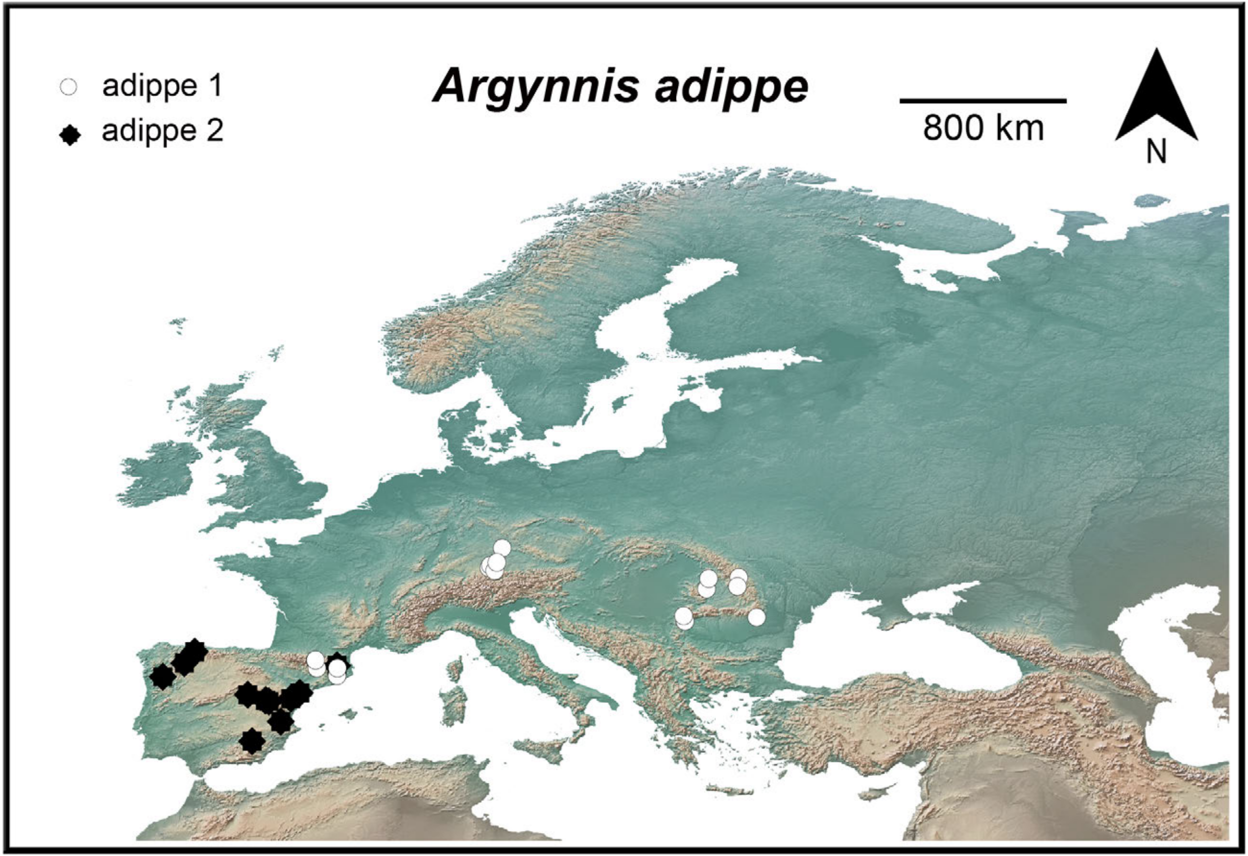
adippe 1
adippe 2
Argynnis adippe
800 km
N

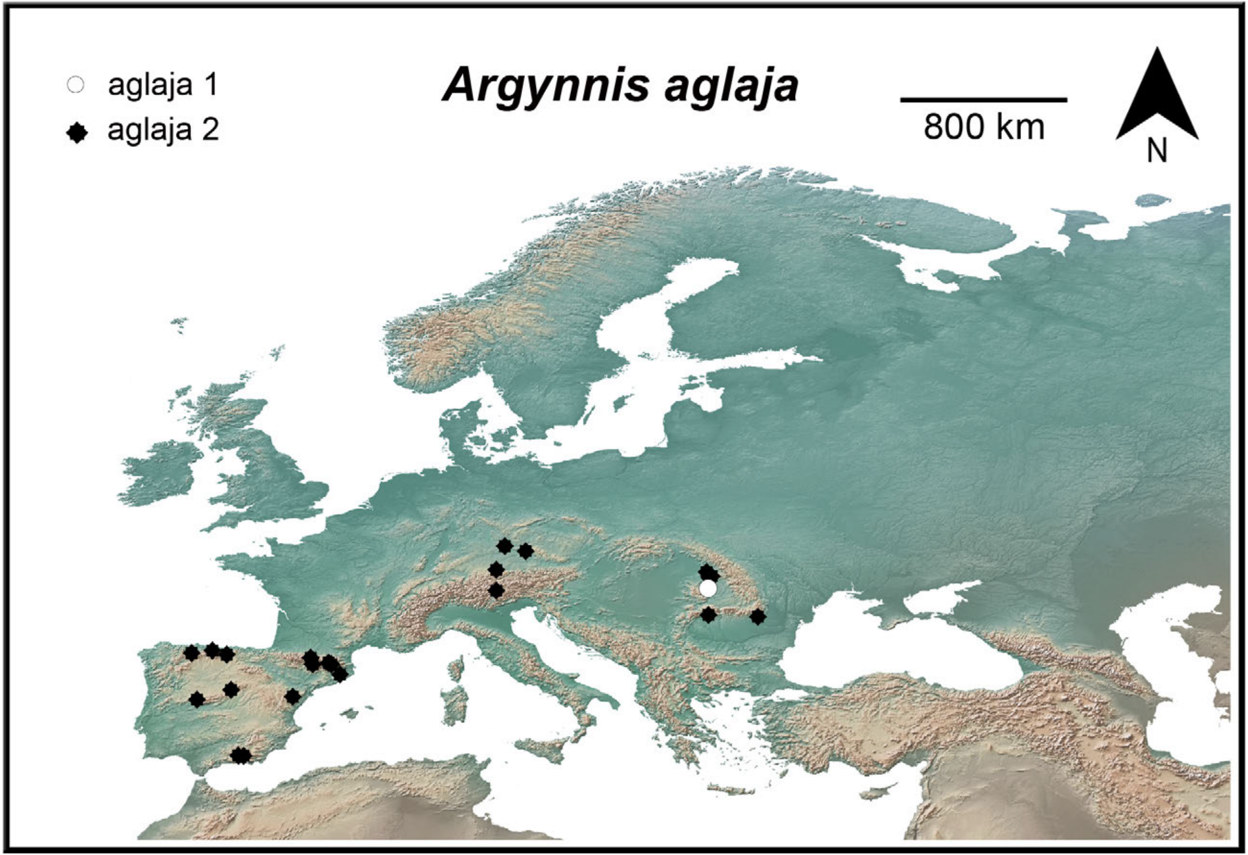
Argynnis aglaja
aglaja 1
aglaja 2
800 km
N

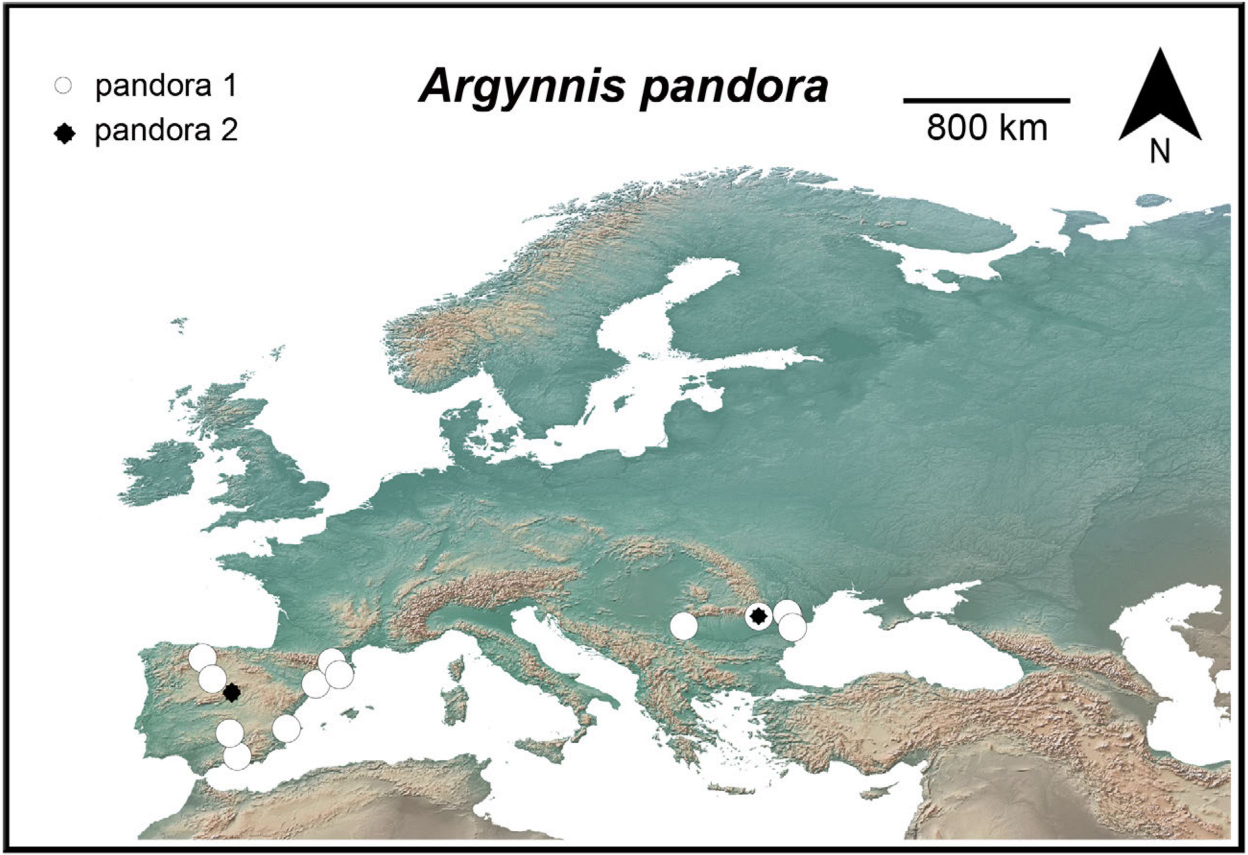
pandora 1
pandora 2
Argynnis pandora
800 km
N

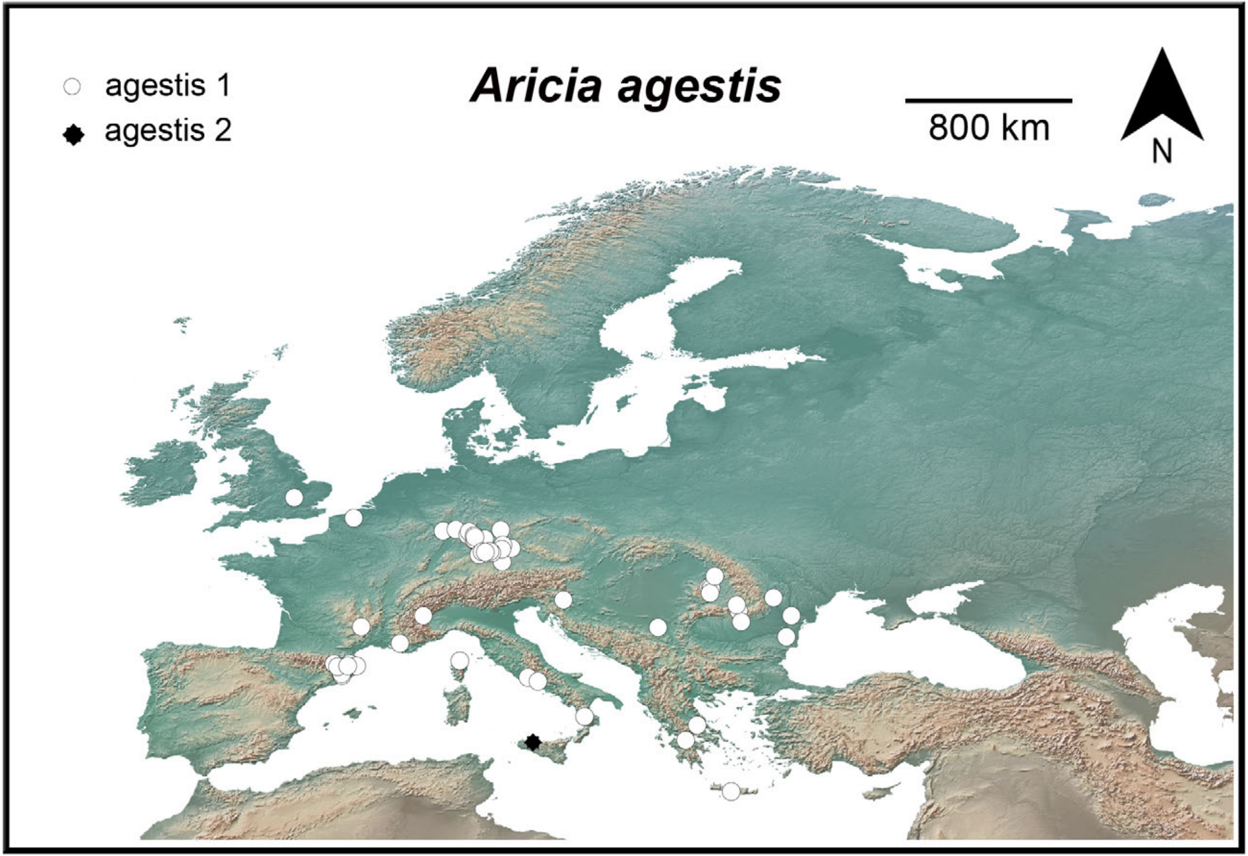
Aricia agestis
agestis 1
agestis 2
800 km
N

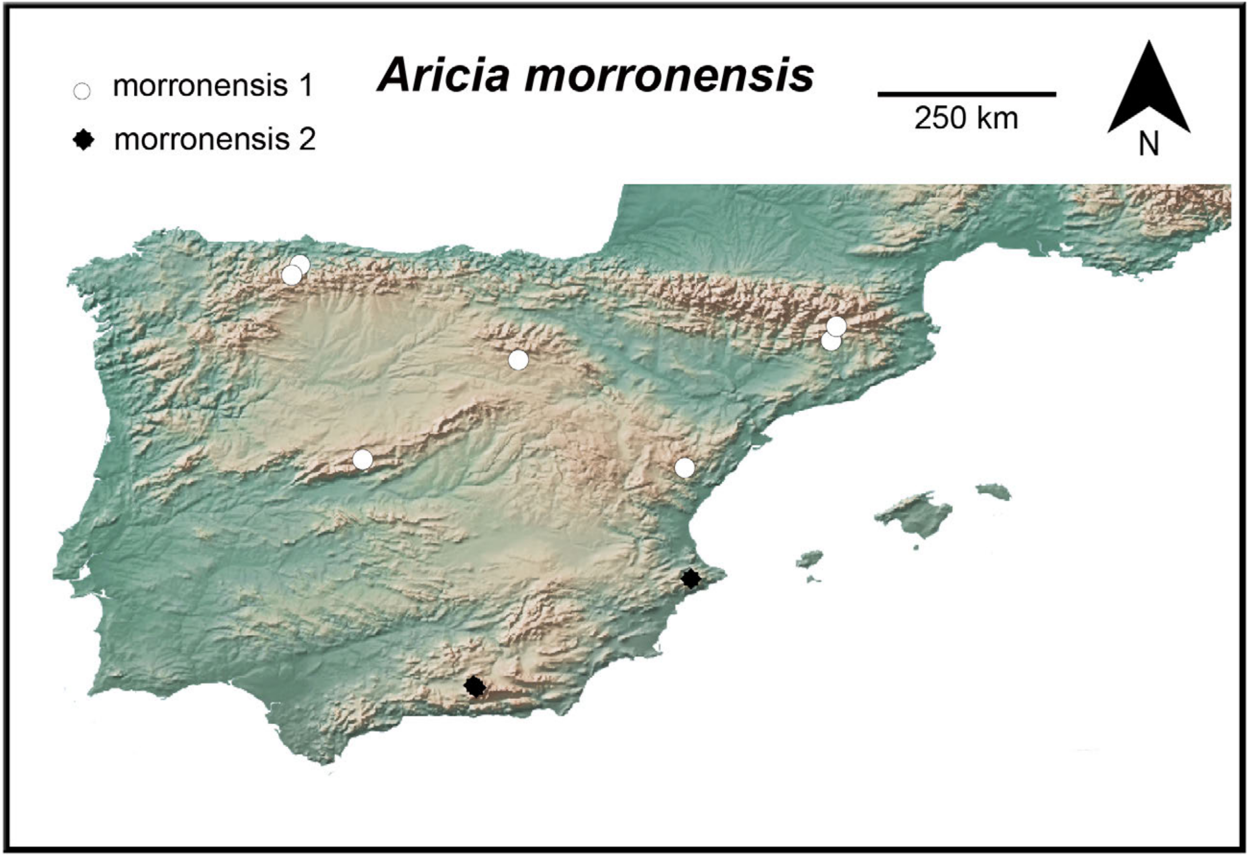
Aricia morronensis
morronensis 1
morronensis 2
250 km
N

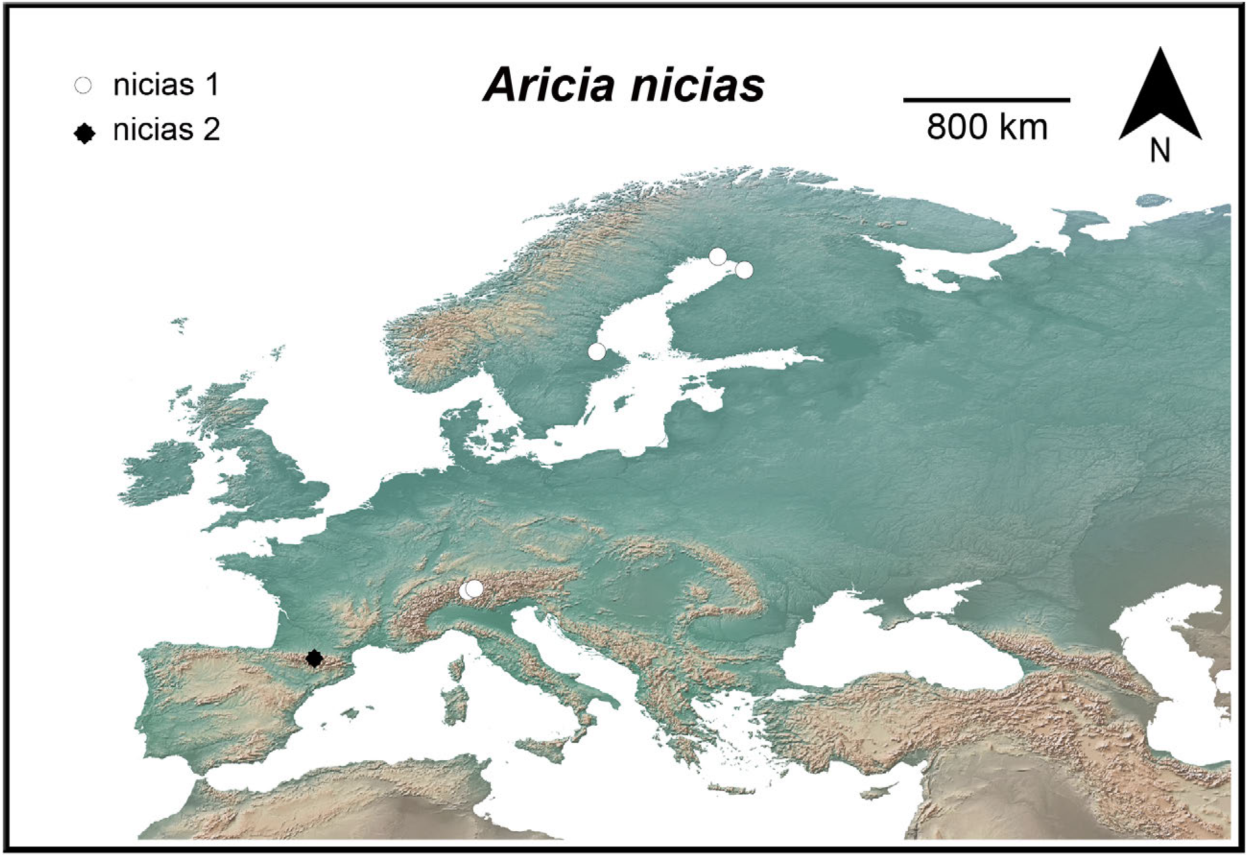
Aricia nicias
nicias 1
nicias 2
800 km
N

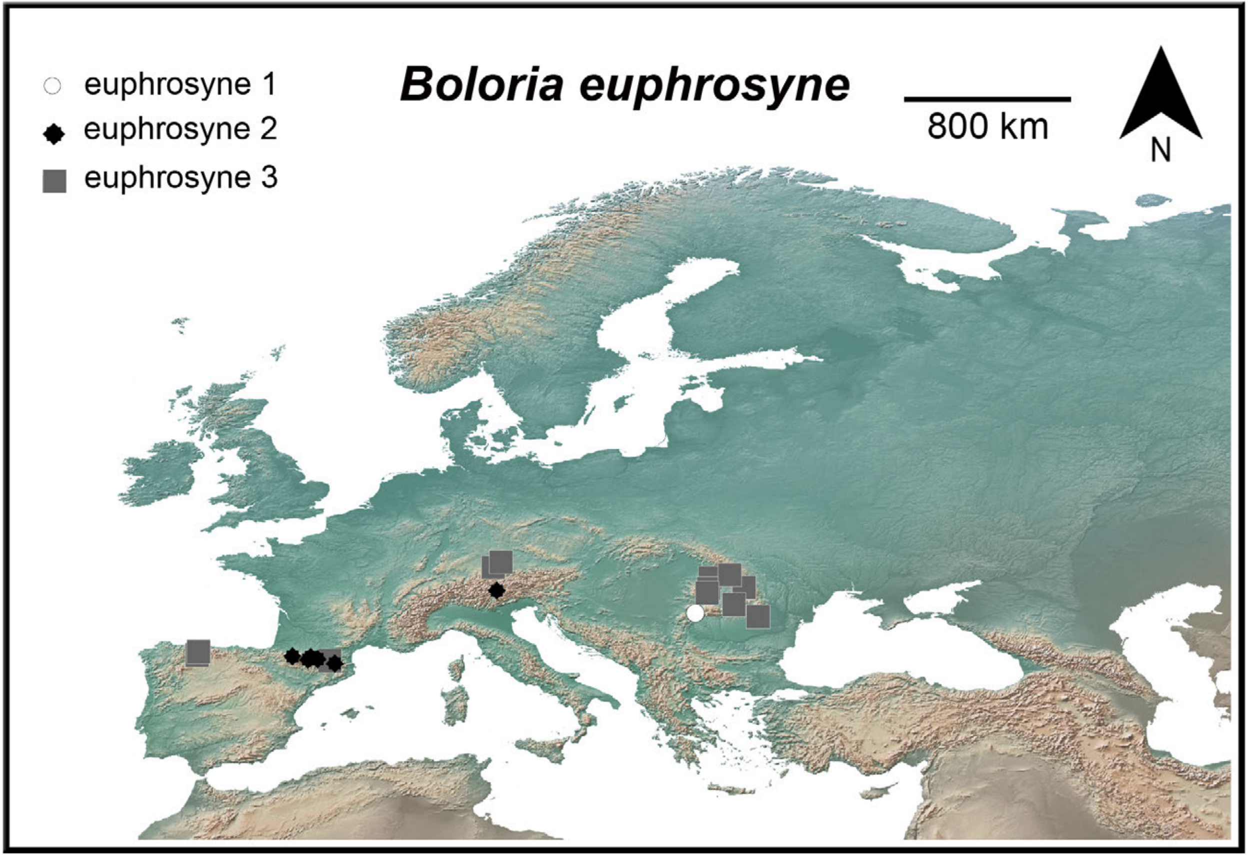
euphrosyne 1
euphrosyne 2
euphrosyne 3
Boloria euphrosyne
800 km
N

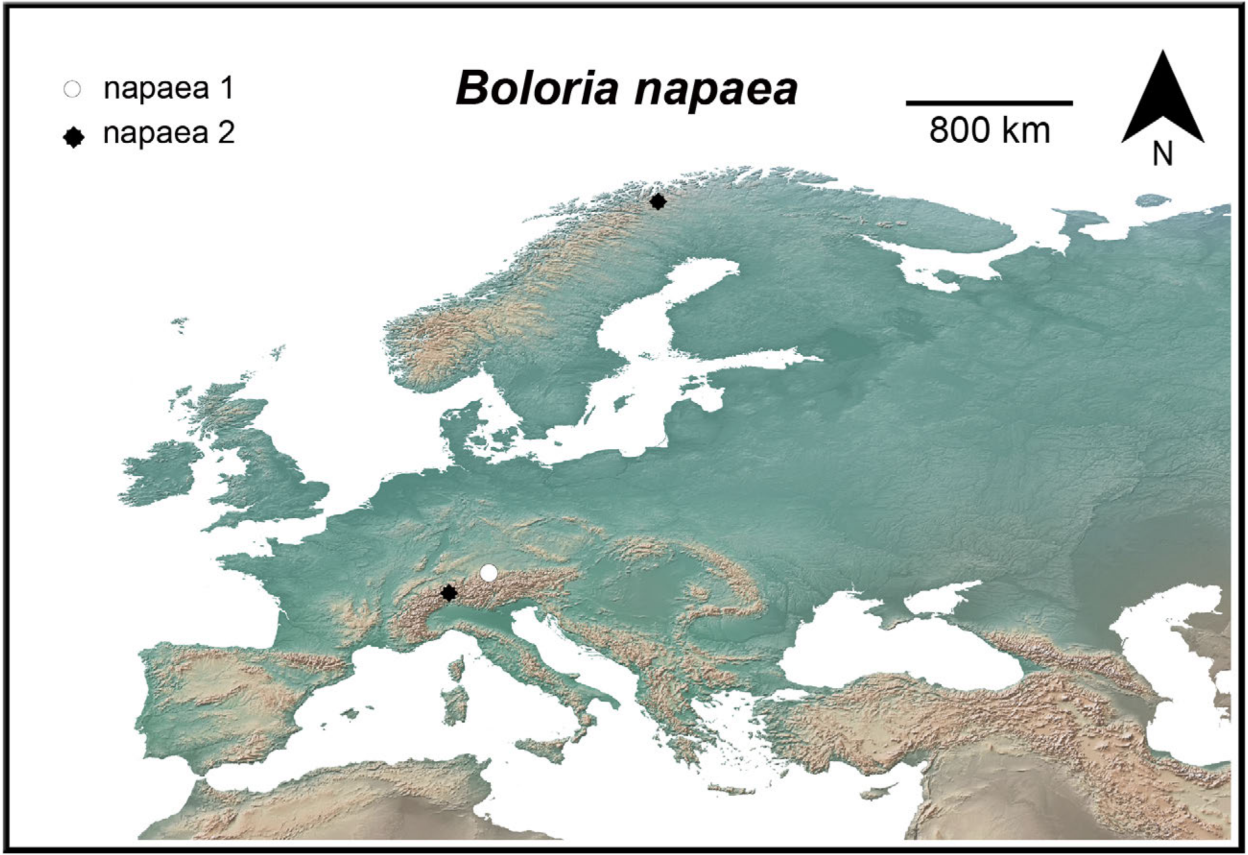

Boloria napaea
napaea 1
napaea 2
800 km
N

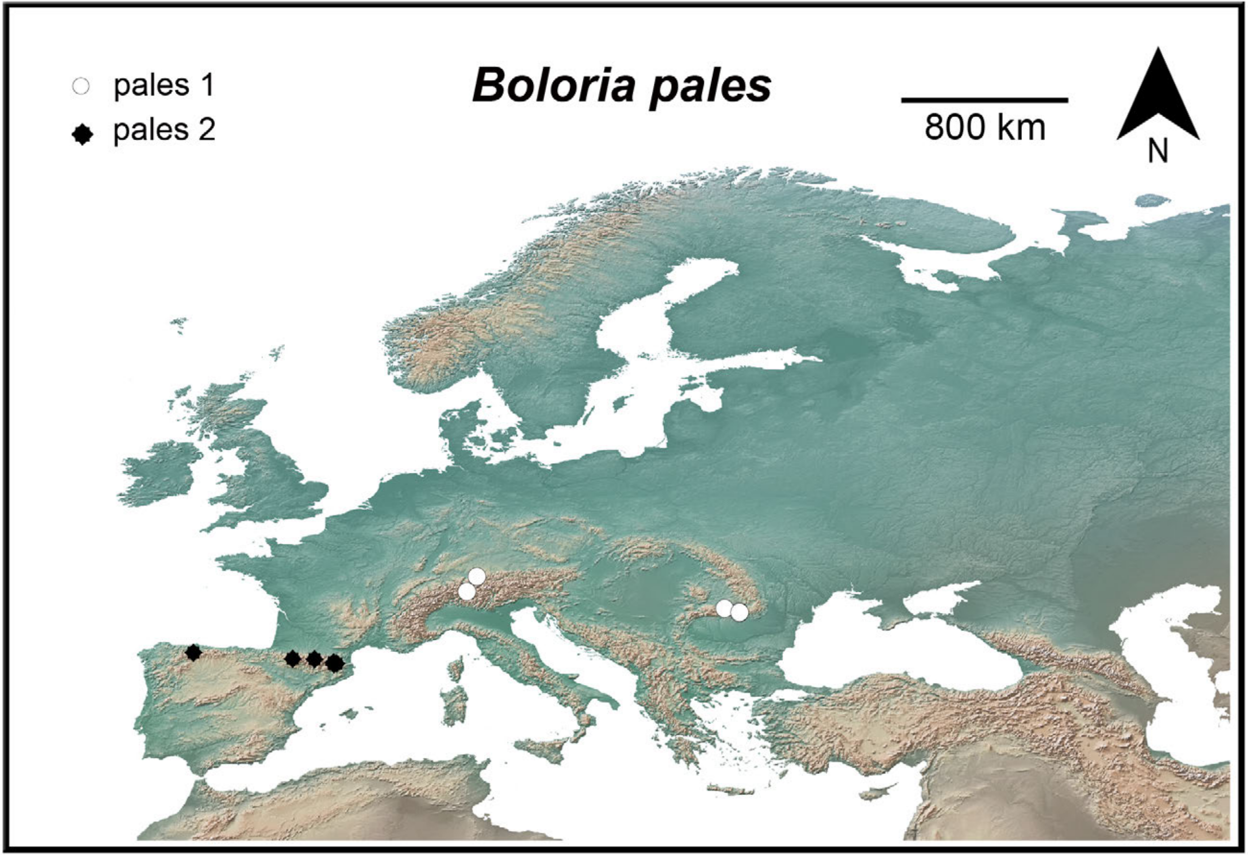

pales 1
pales 2
Boloria pales
800 km
N

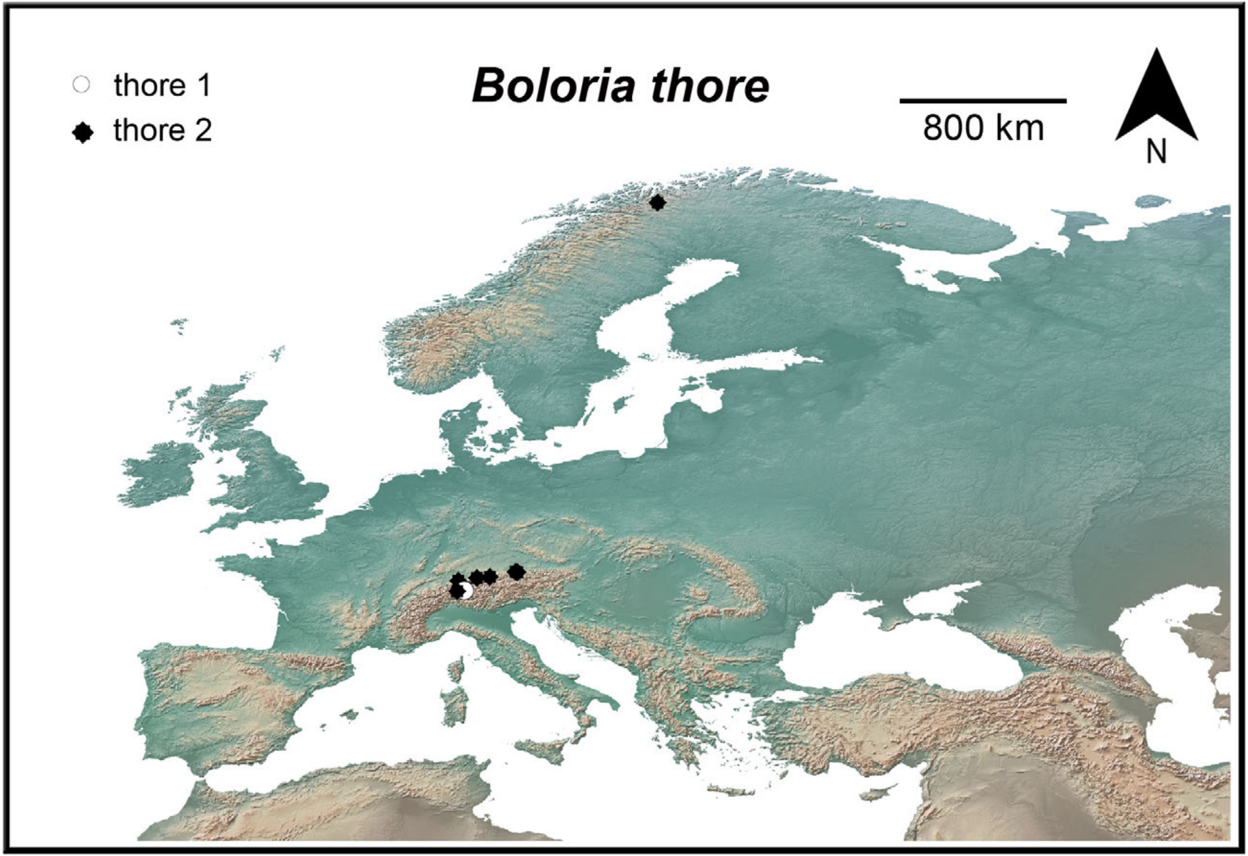
thore 1
thore 2
Boloria thore
800 km
N

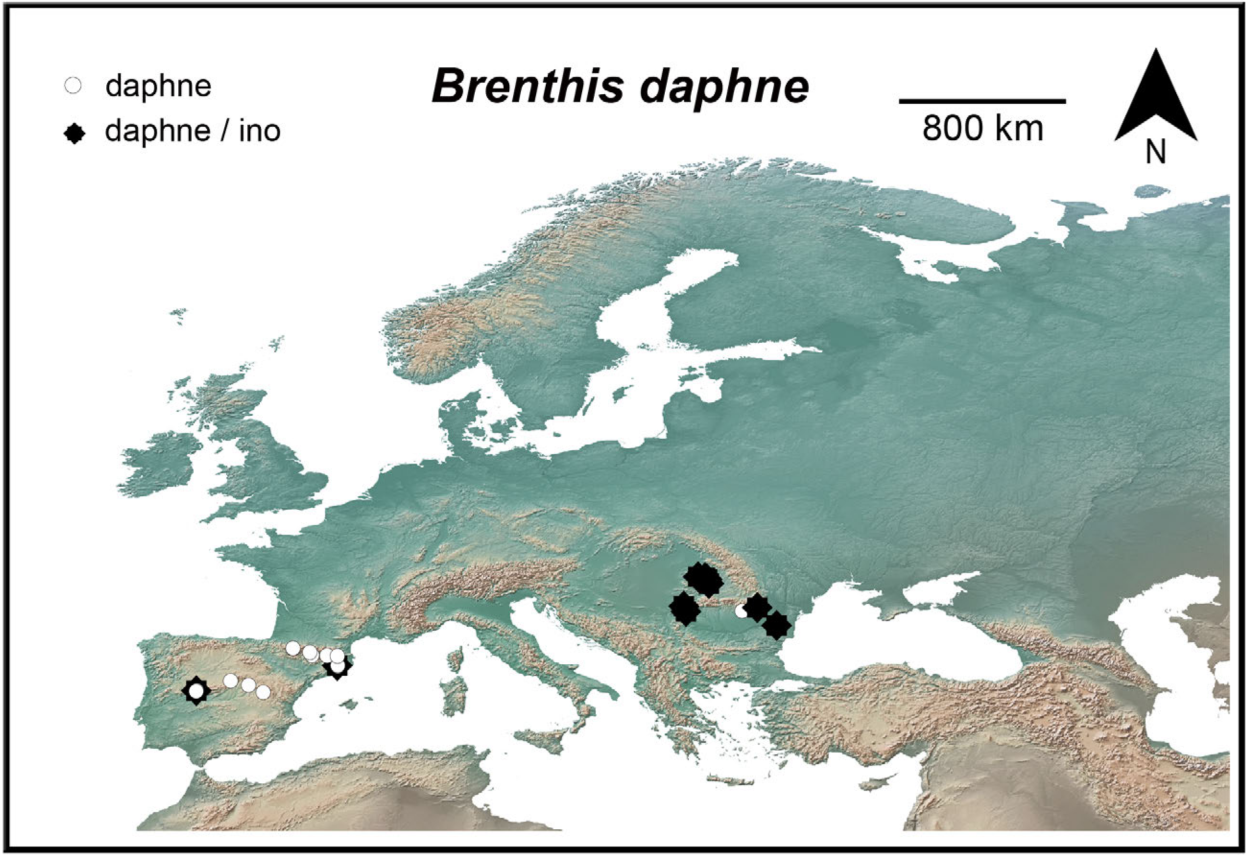
daphne
daphne / ino
Brenthis daphne
800 km
N

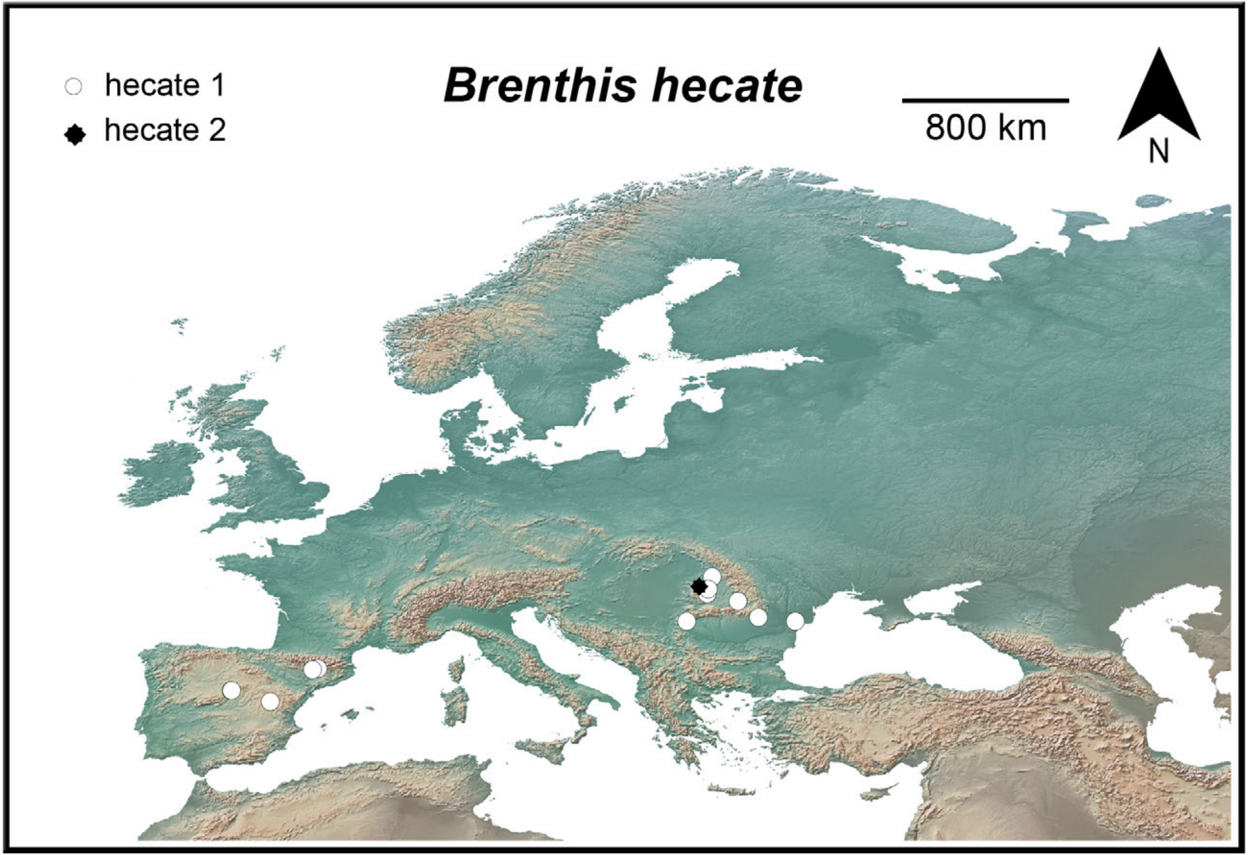
hecate 1
hecate 2
Brenthis hecate
800 km
N

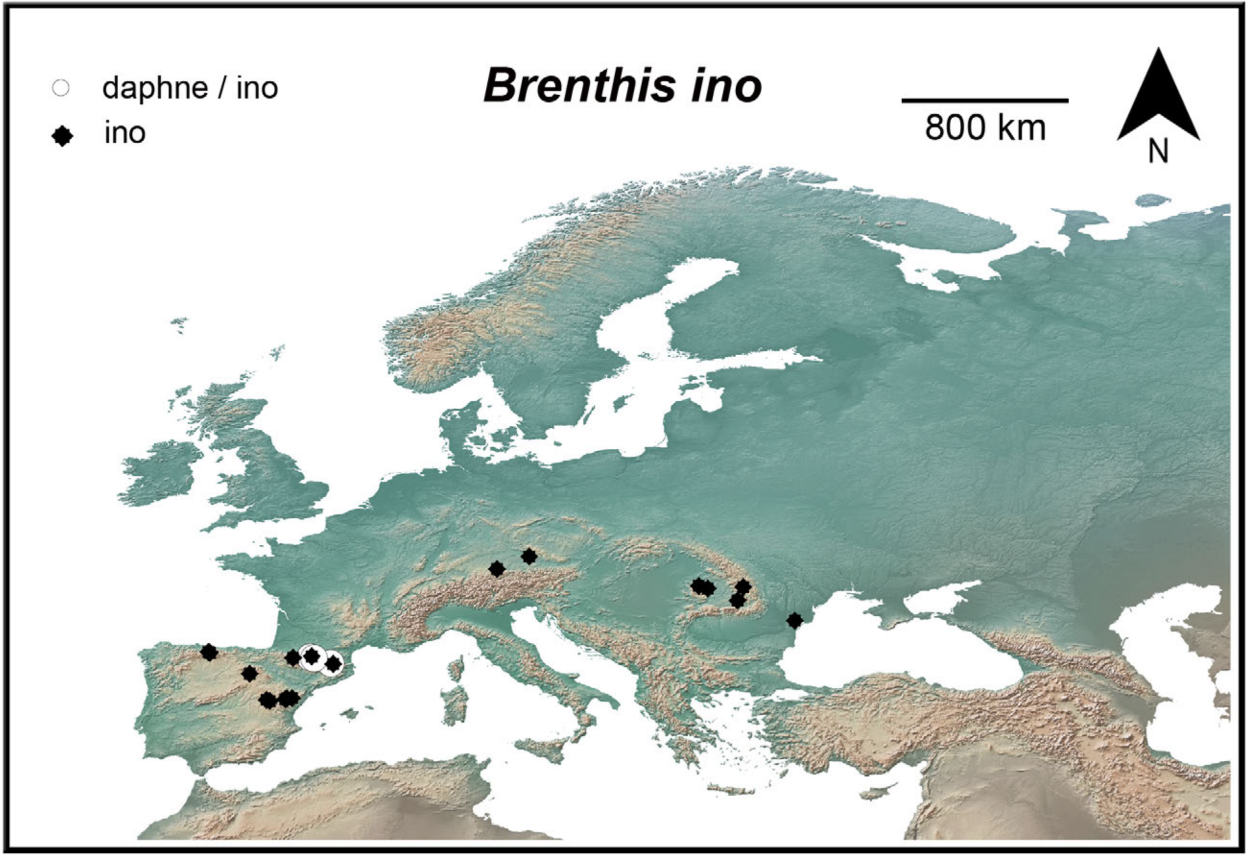

daphne / ino
ino
Brenthis ino
800 km
N

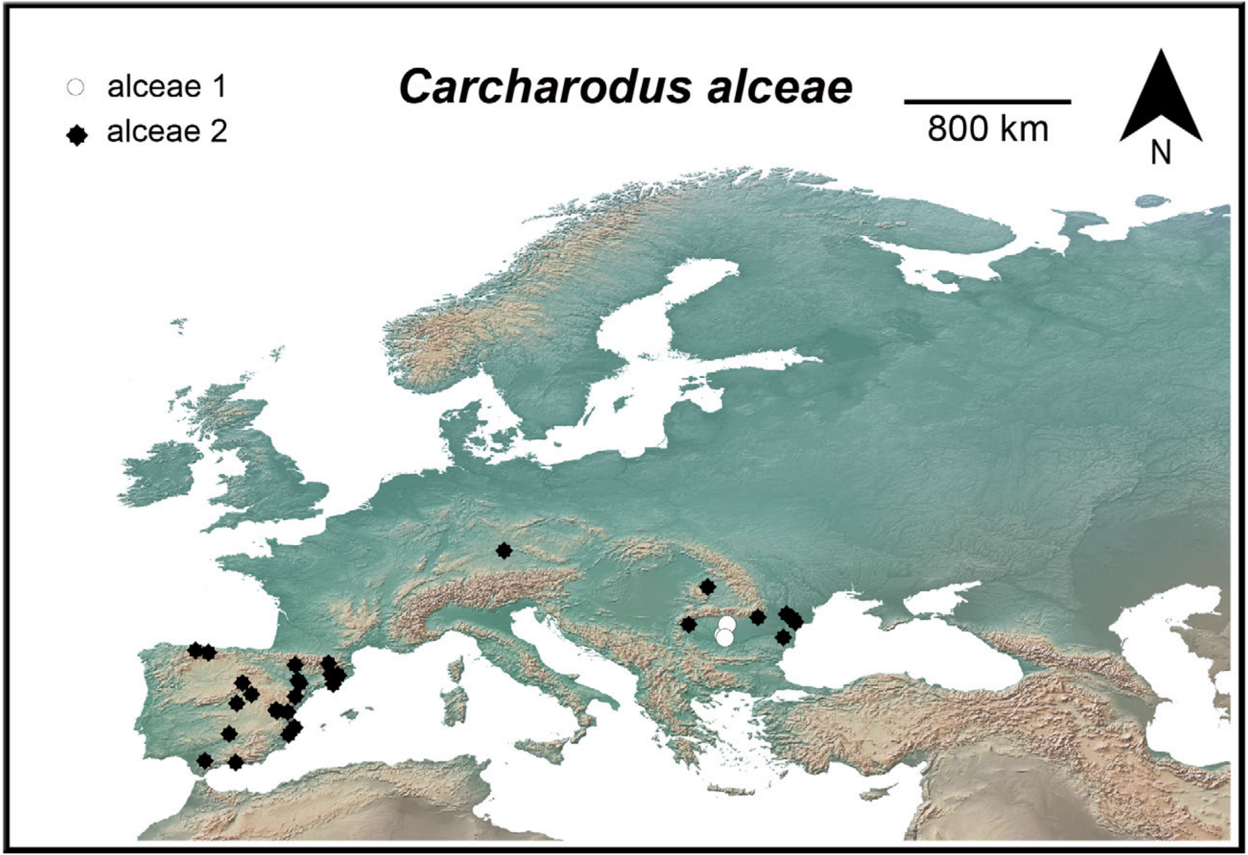
alceae 1
alceae 2
Carcharodus alceae
800 km
N

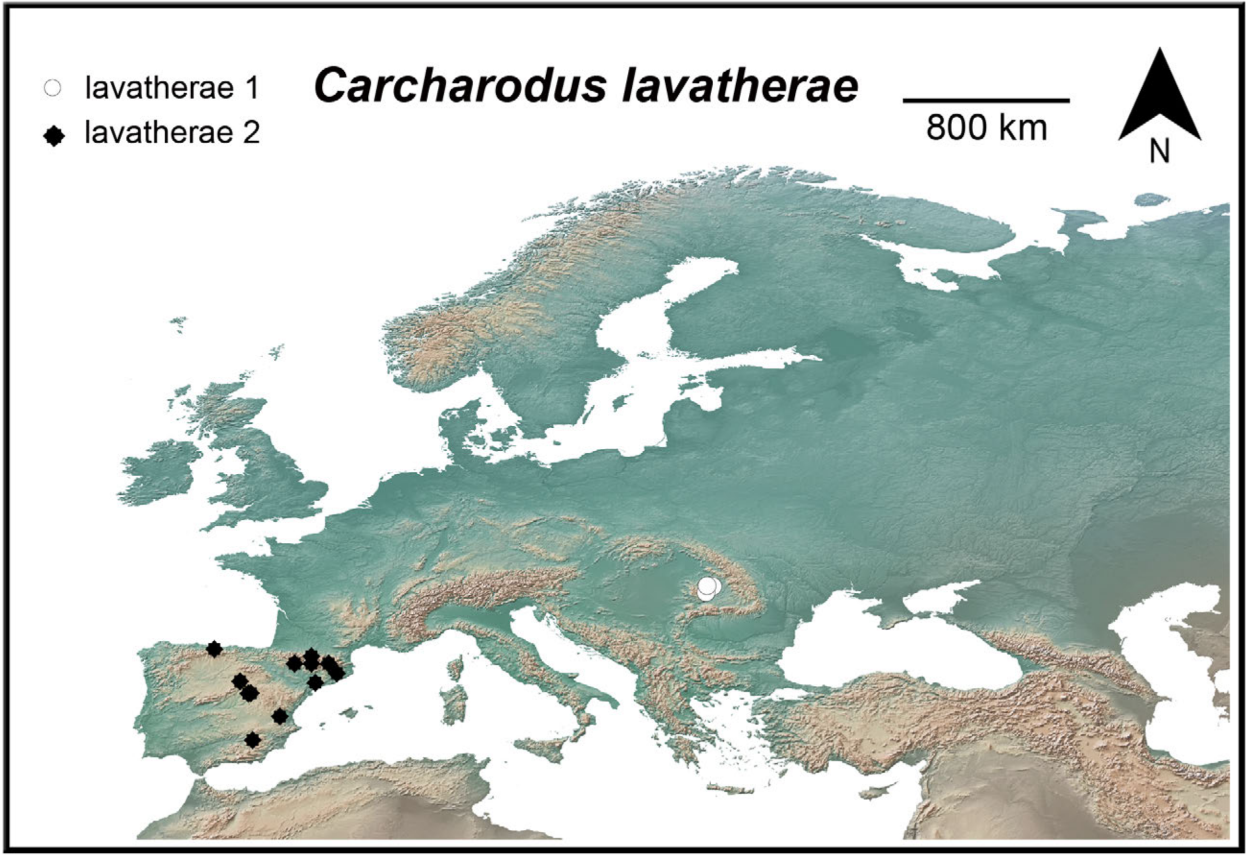
lavatherae 1
lavatherae 2
Carcharodus lavatherae
800 km
N

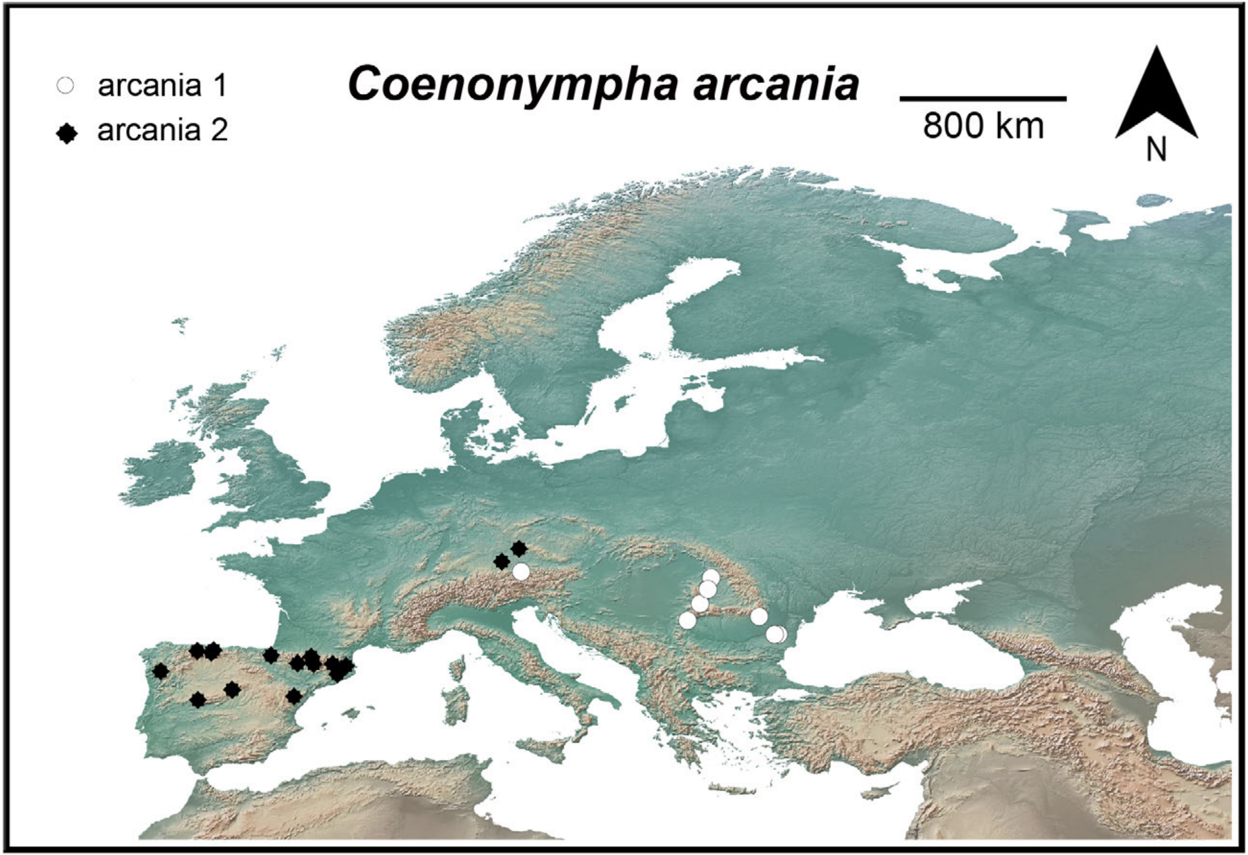
Coenonympha arcania
arcania 1
arcania 2
800 km
N

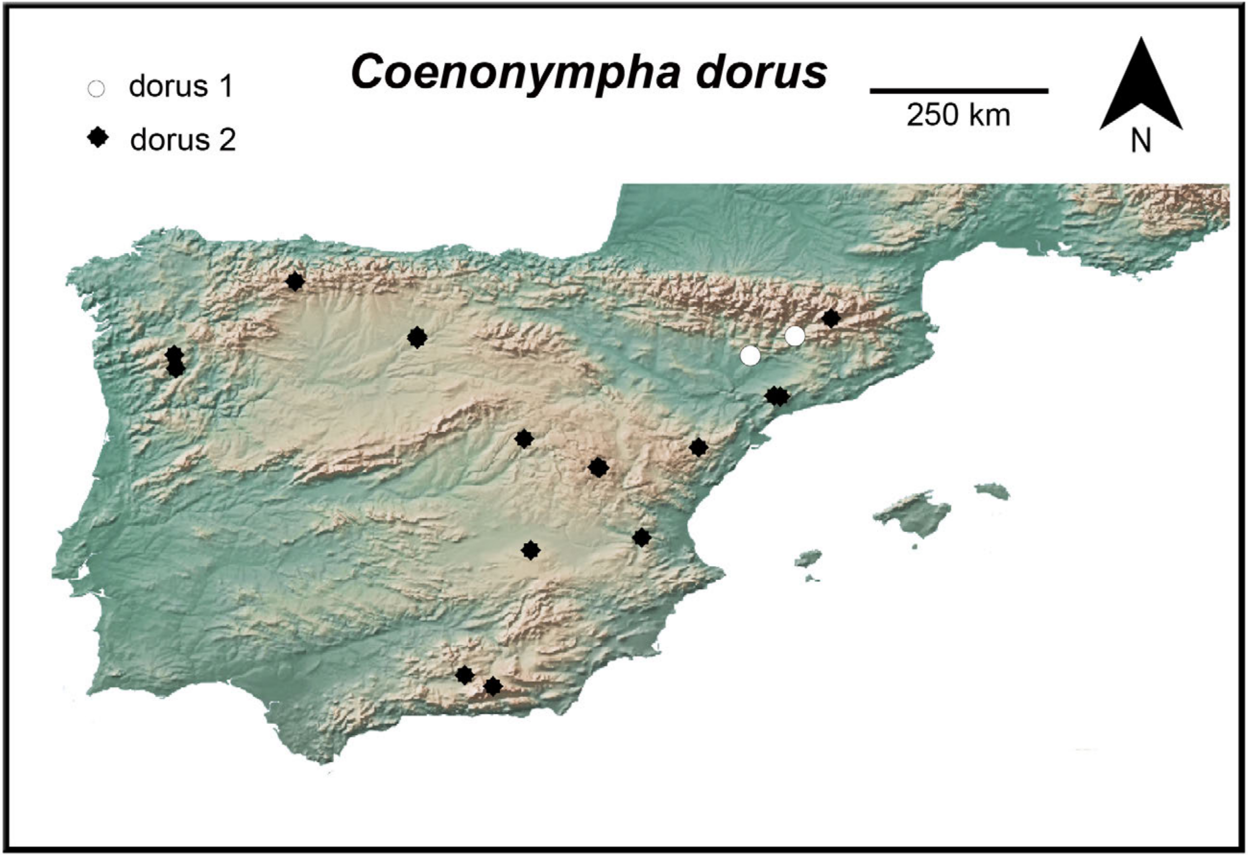

Coenonympha dorus
dorus 1
dorus 2
250 km
N

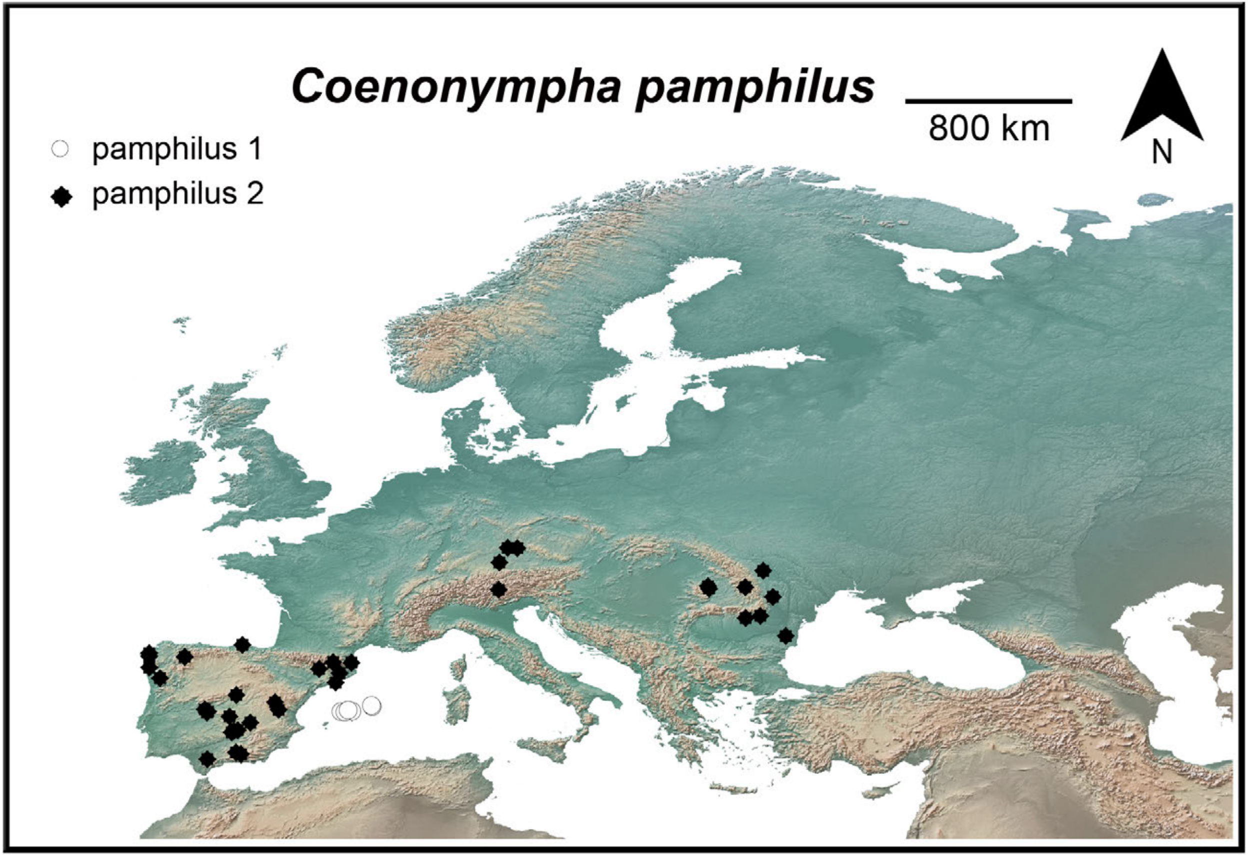

Coenonympha pamphilus
800 km
N
pamphilus 1
pamphilus 2

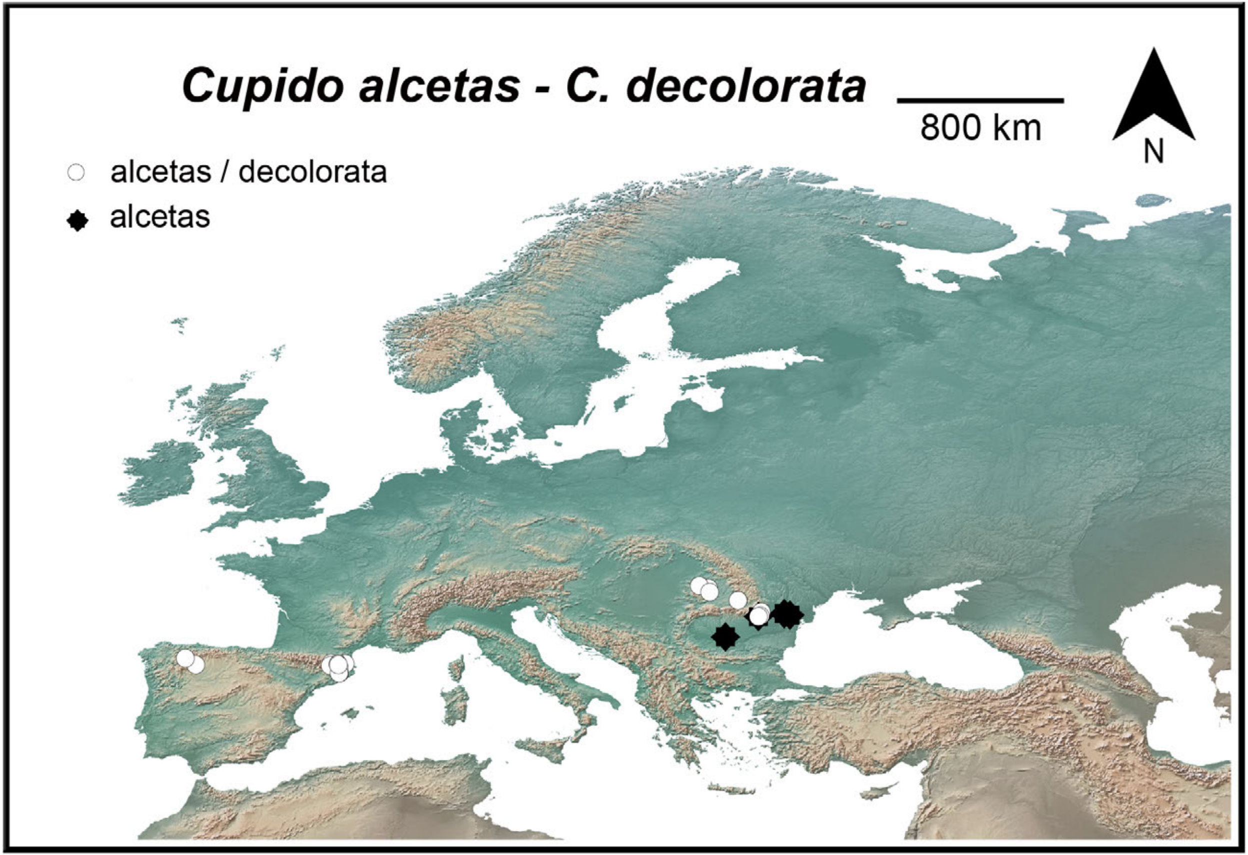

Cupido alcetas - C. decolorata
800 km
N
alcetas / decolorata
alcetas

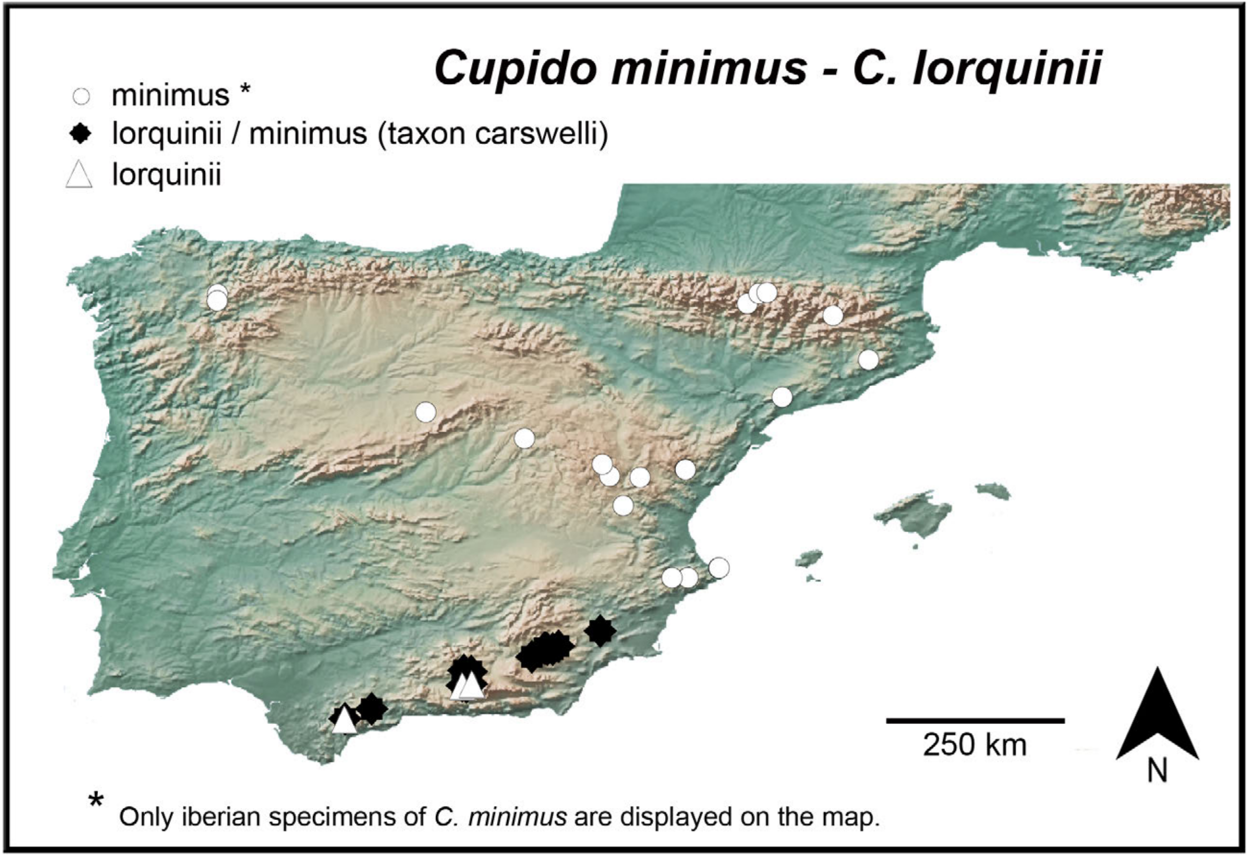
Cupido minimus - C. lorquinii
minimus *
lorquinii / minimus (taxon carswelli)
lorquinii
250 km
N
* Only iberian specimens of C. minimus are displayed on the map.

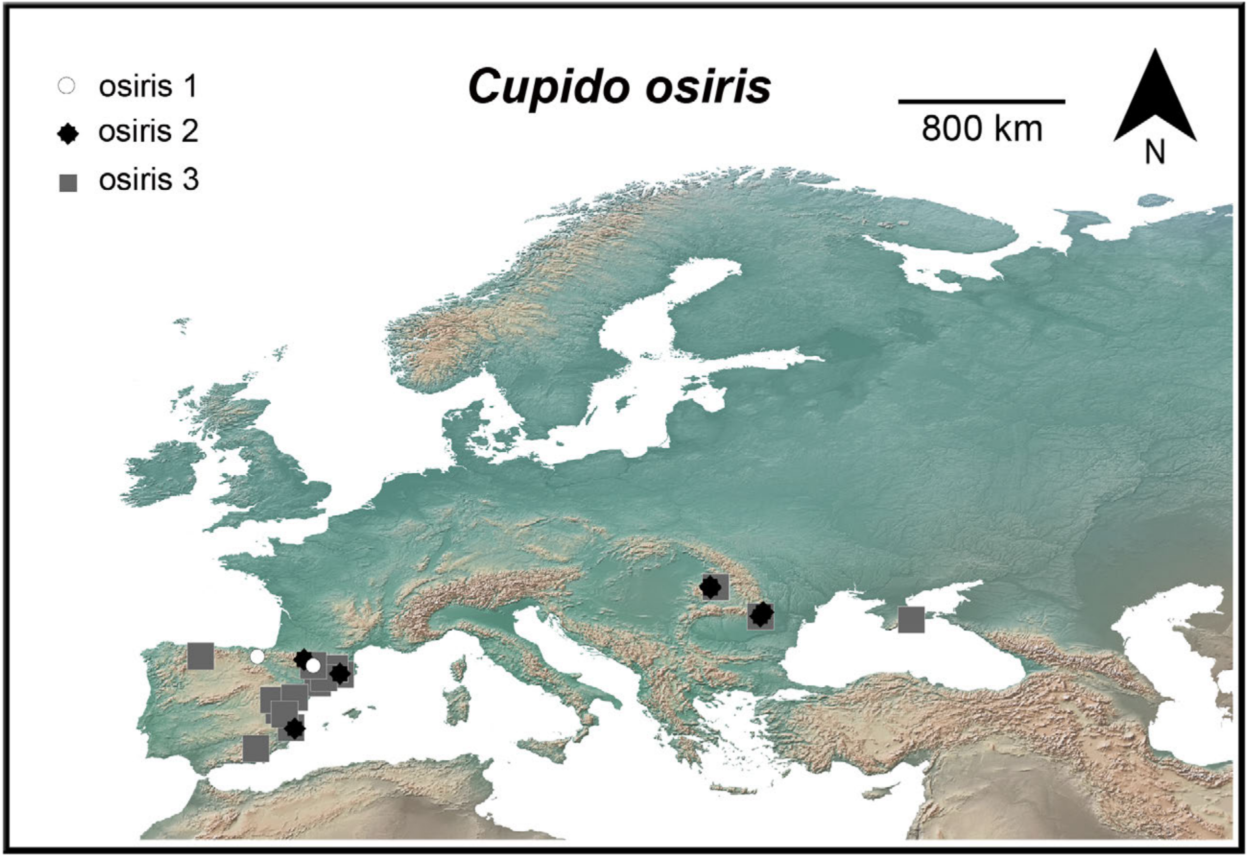

osiris 1
osiris 2
osiris 3
Cupido osiris
800 km
N

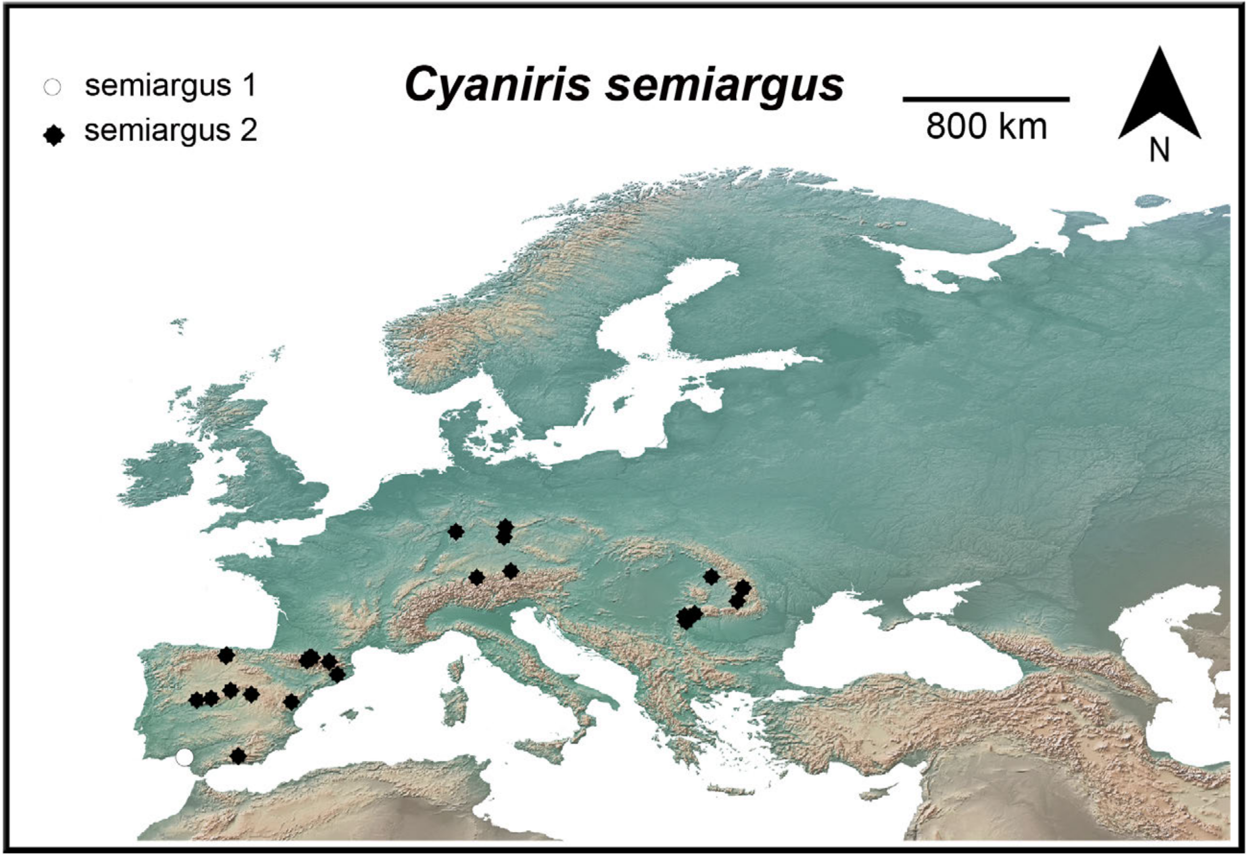
semiargus 1
semiargus 2
Cyaniris semiargus
800 km
N

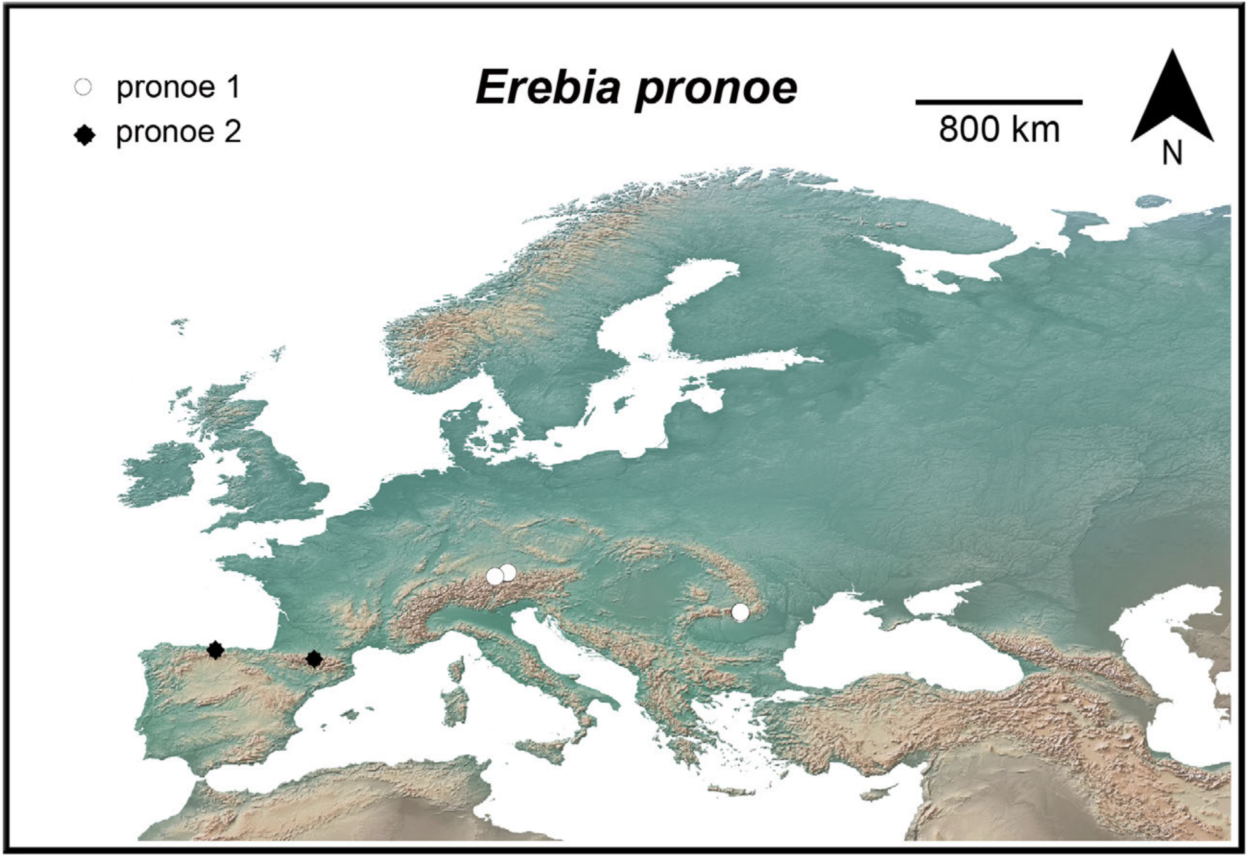
Erebia pronoe
pronoe 1
pronoe 2
800 km
N

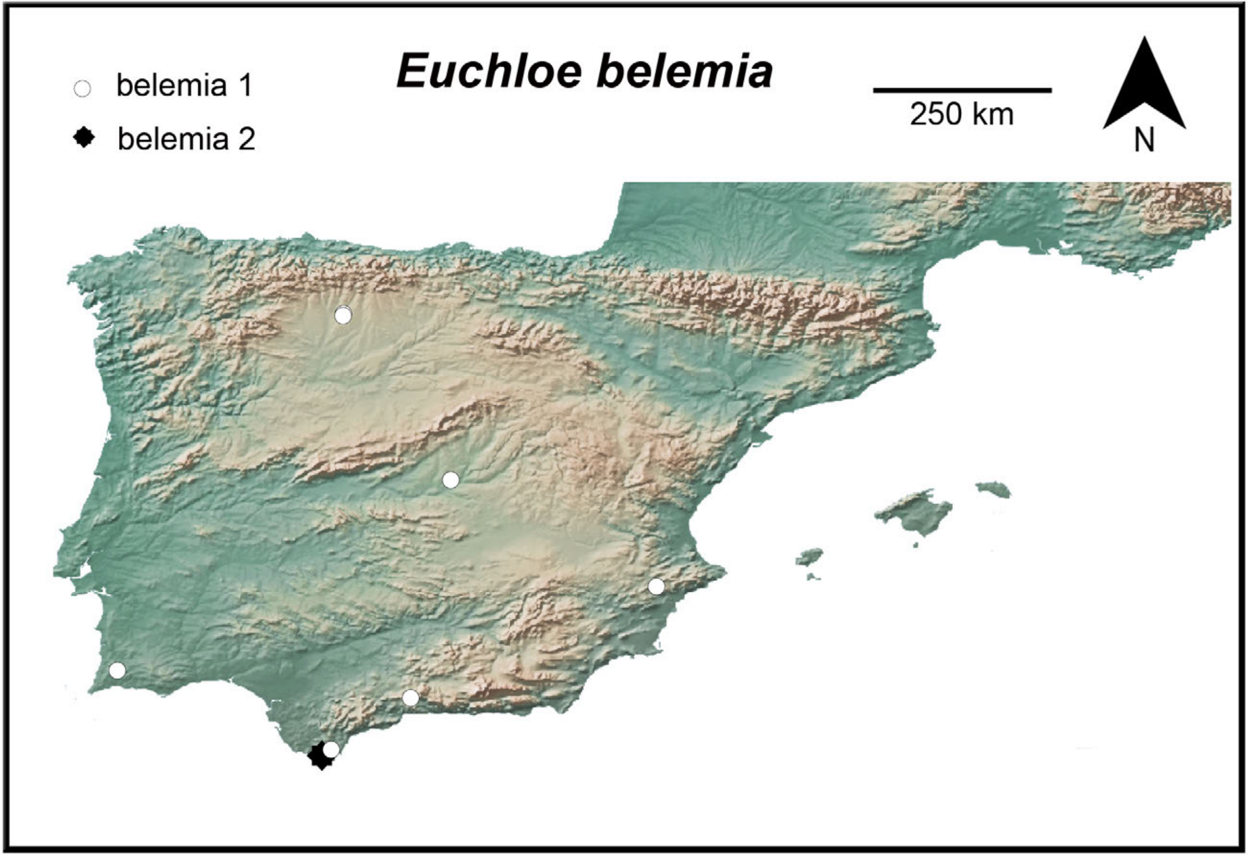
Euchloe belemia
belemia 1
belemia 2
250 km
N

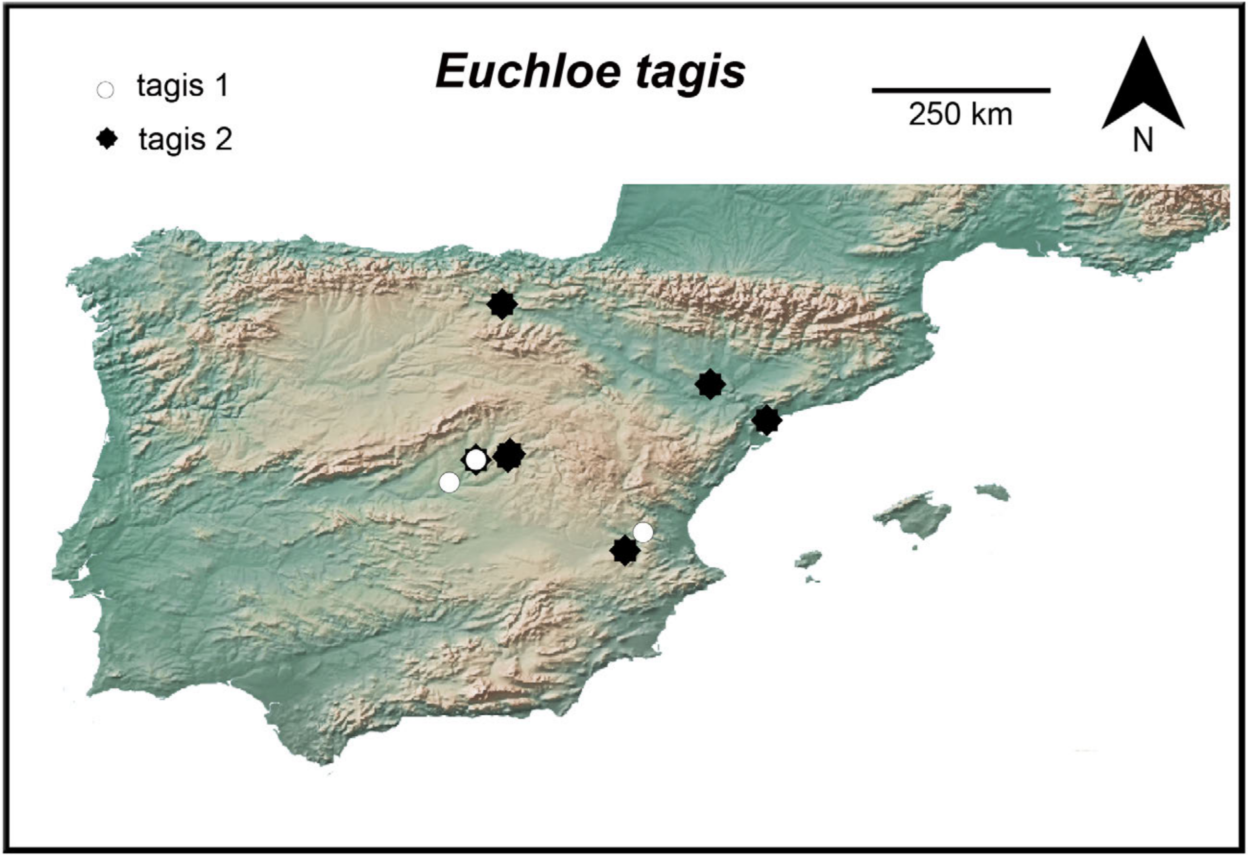
Euchloe tagis
tagis 1
tagis 2
250 km
N

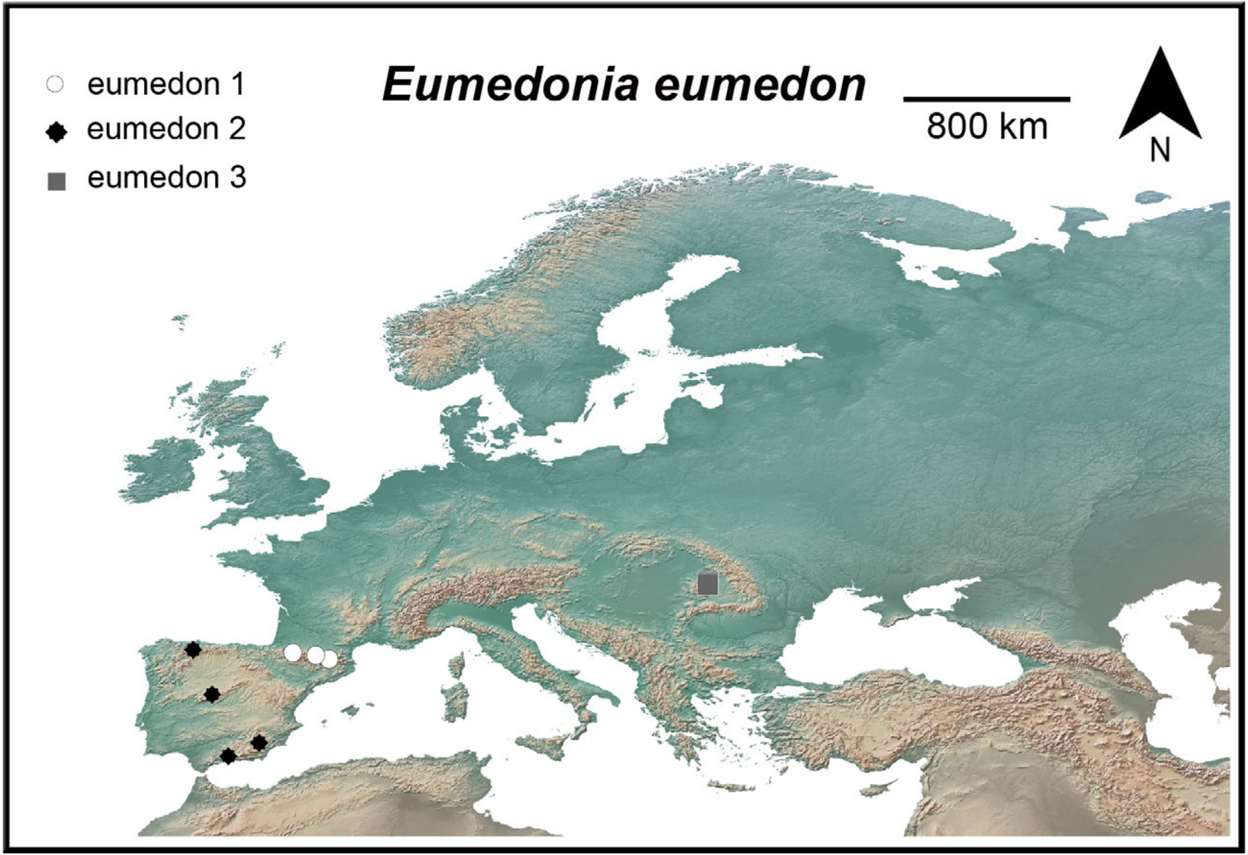
Eumedonia eumedon
eumedon 1
eumedon 2
eumedon 3
800 km
N

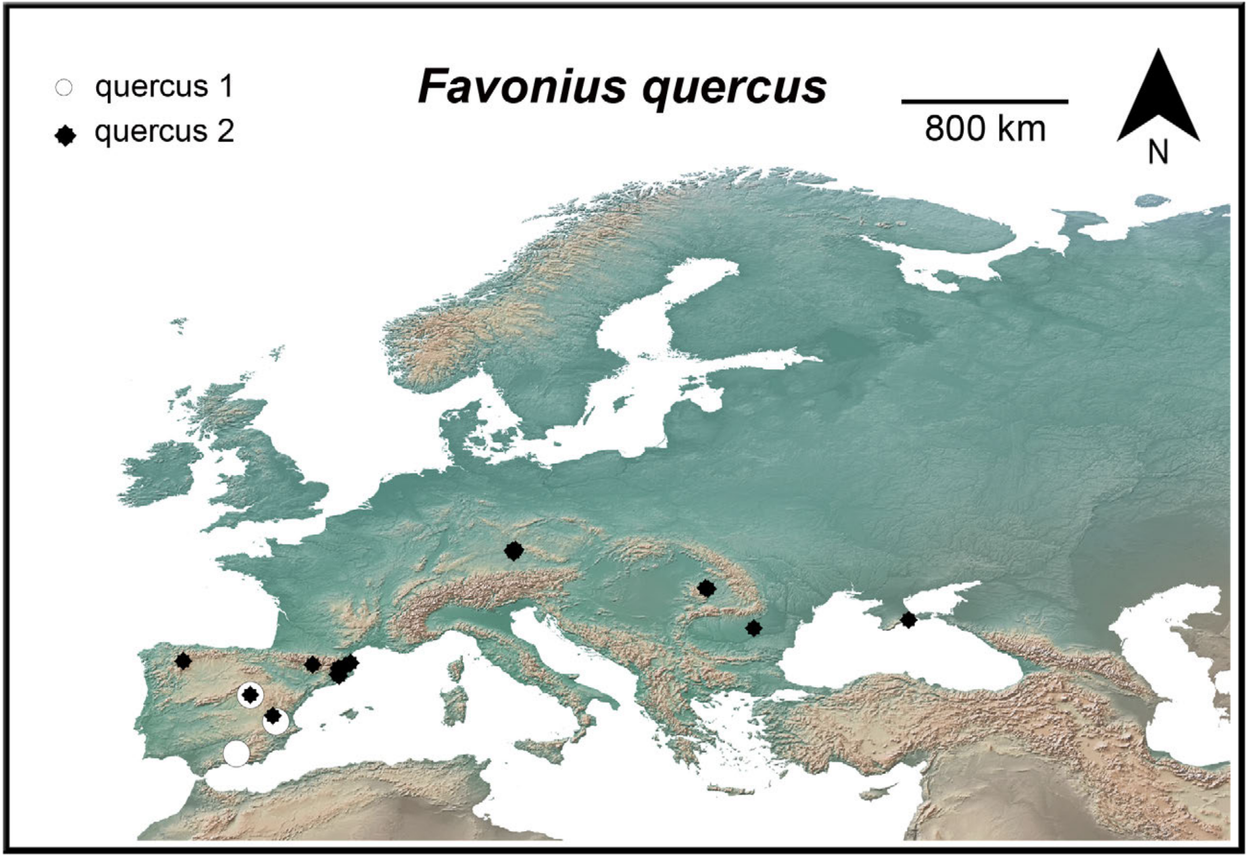

quercus 1
quercus 2
Favonius quercus
800 km
N

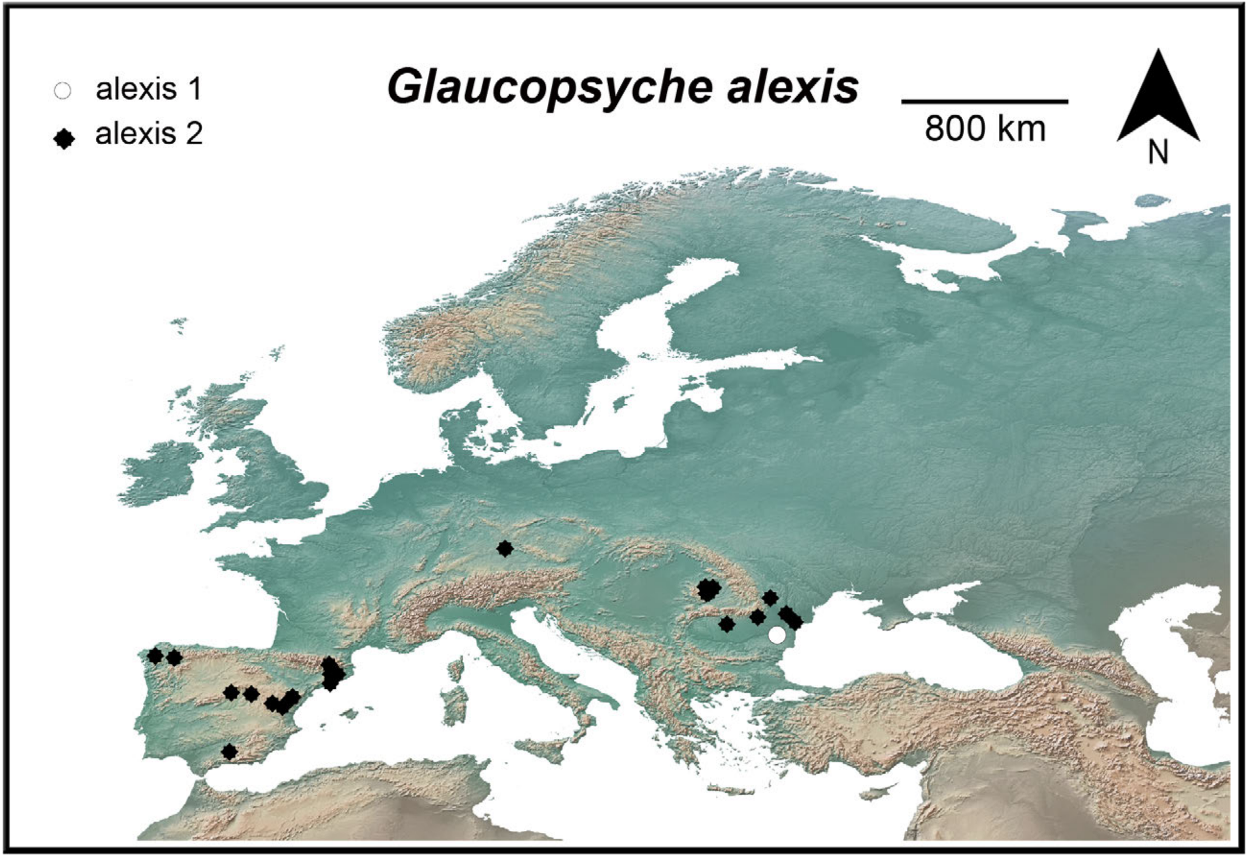
alexis 1
alexis 2
Glaucopsyche alexis
800 km
N

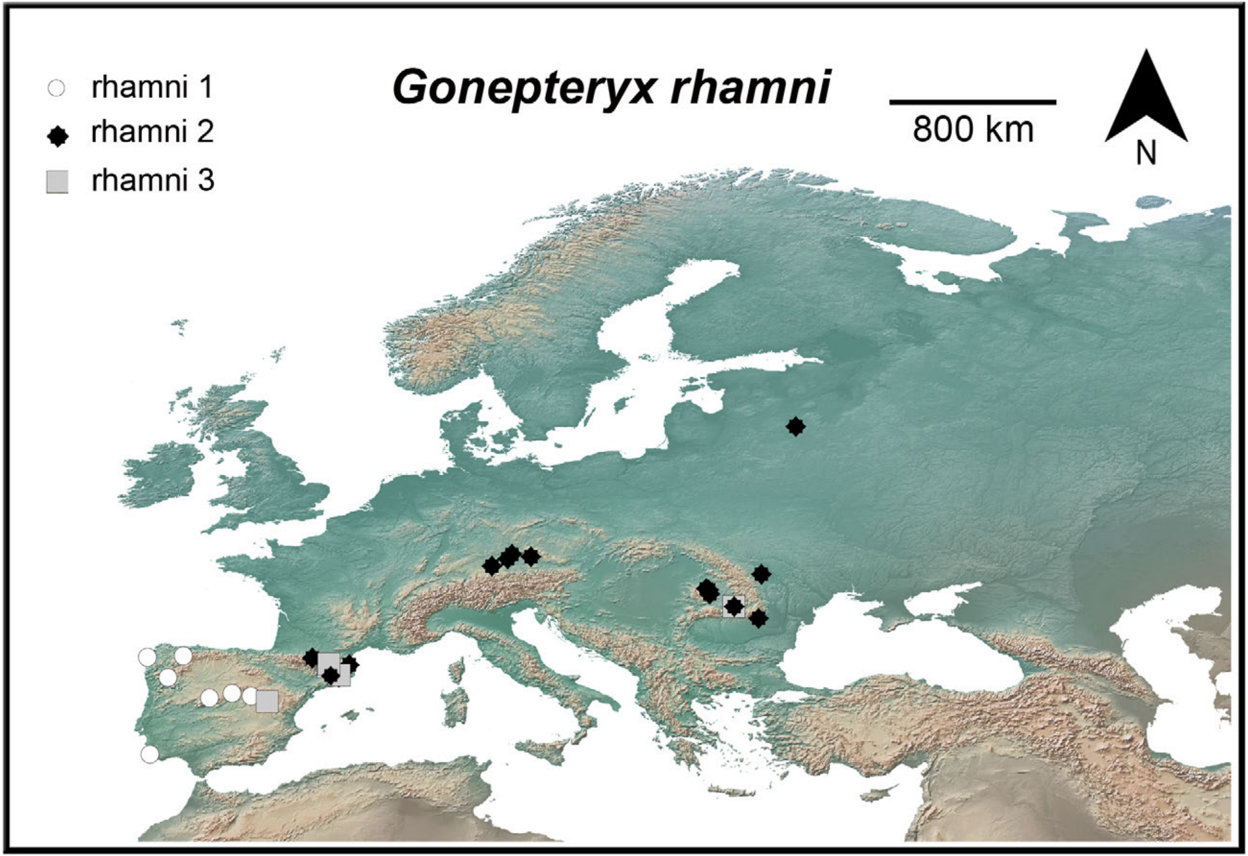
rhamni 1
rhamni 2
rhamni 3
Gonepteryx rhamni
800 km
N

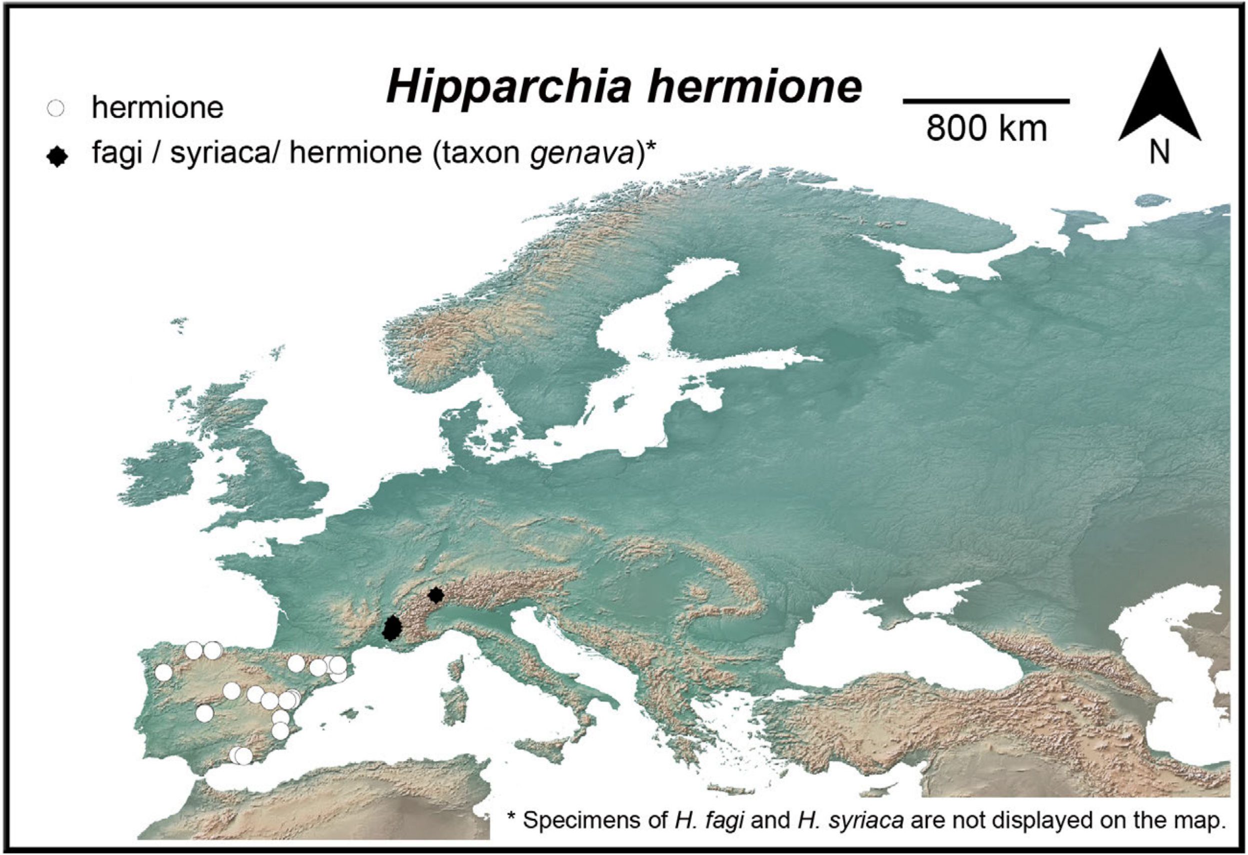

Hipparchia hermione
hermione
fagi / syriaca/ hermione (taxon genava)*
800 km
N
* Specimens of H. fagi and H. syriaca are not displayed on the map.

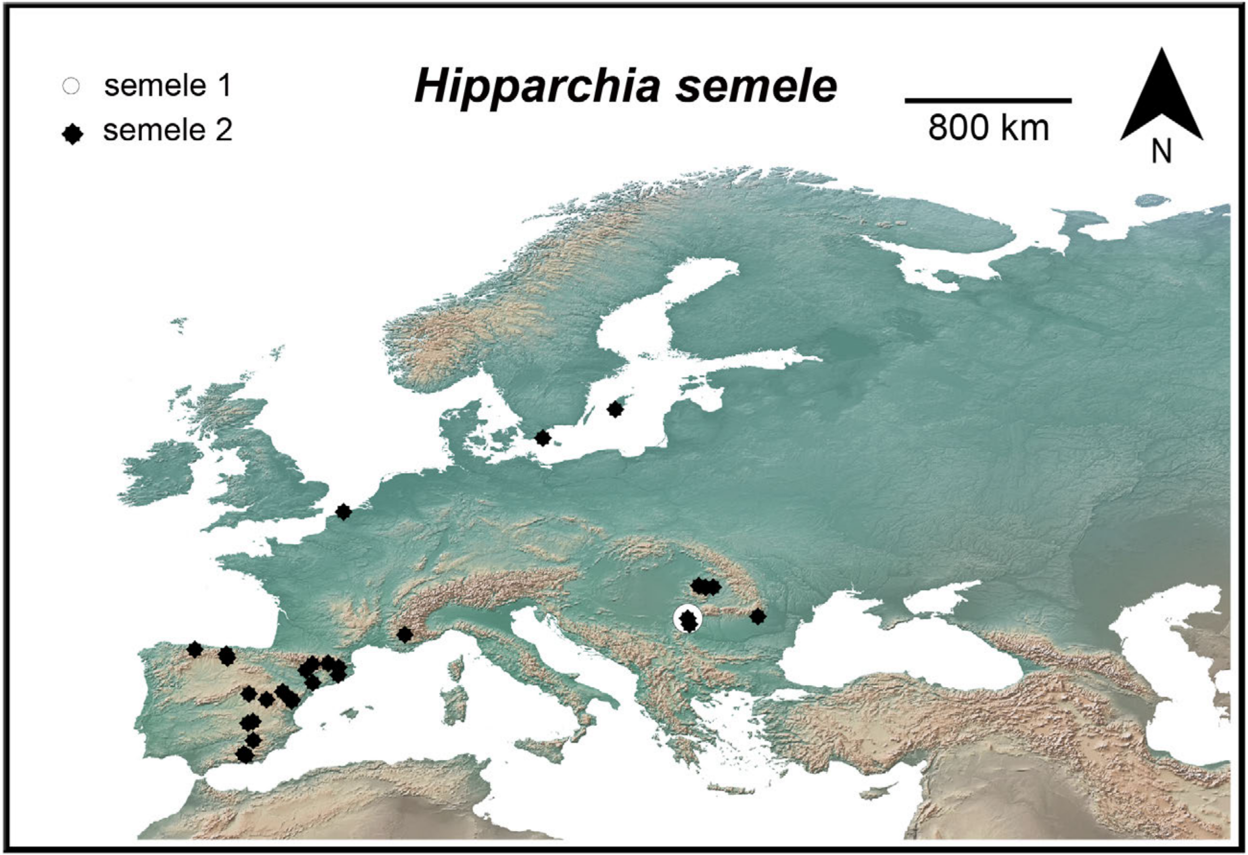
semele 1
semele 2
Hipparchia semele
800 km
N

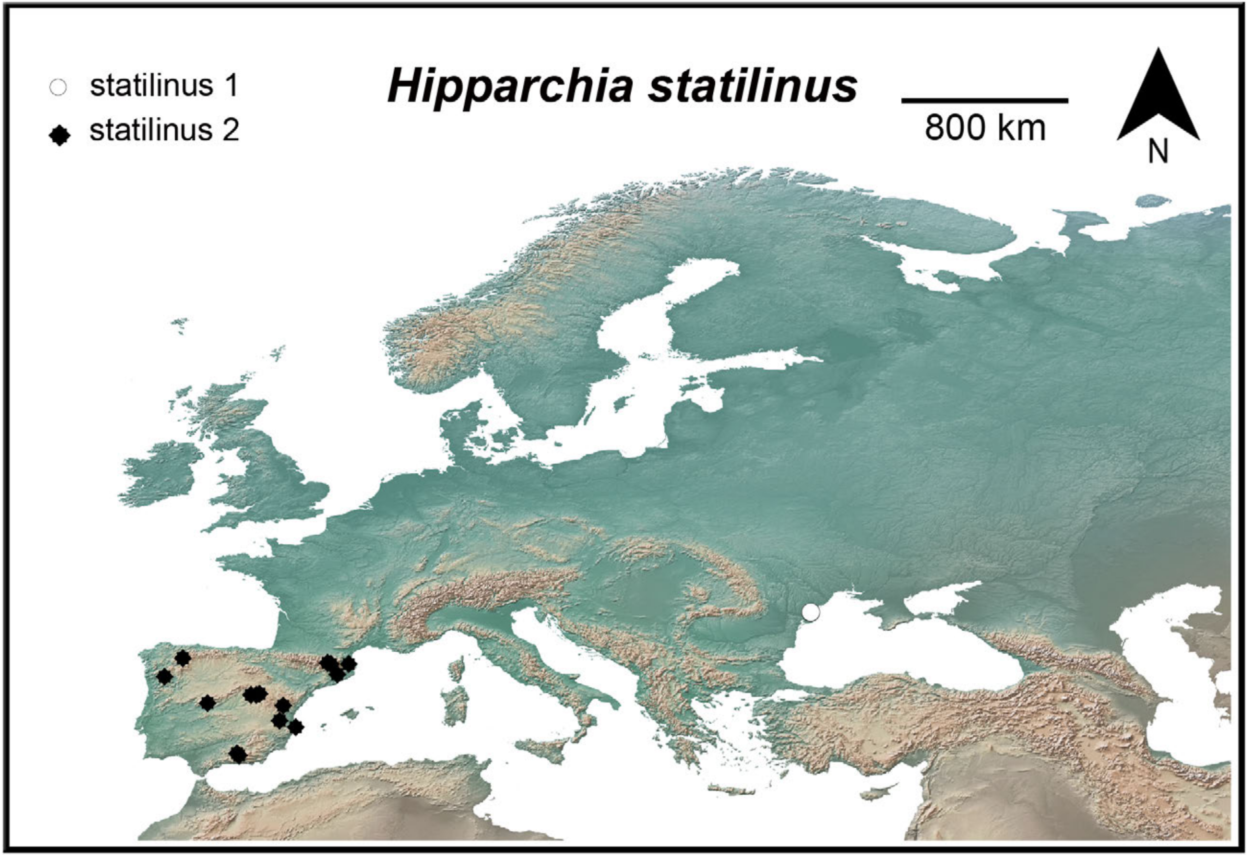
statilinus 1
statilinus 2
Hipparchia statilinus
800 km
N

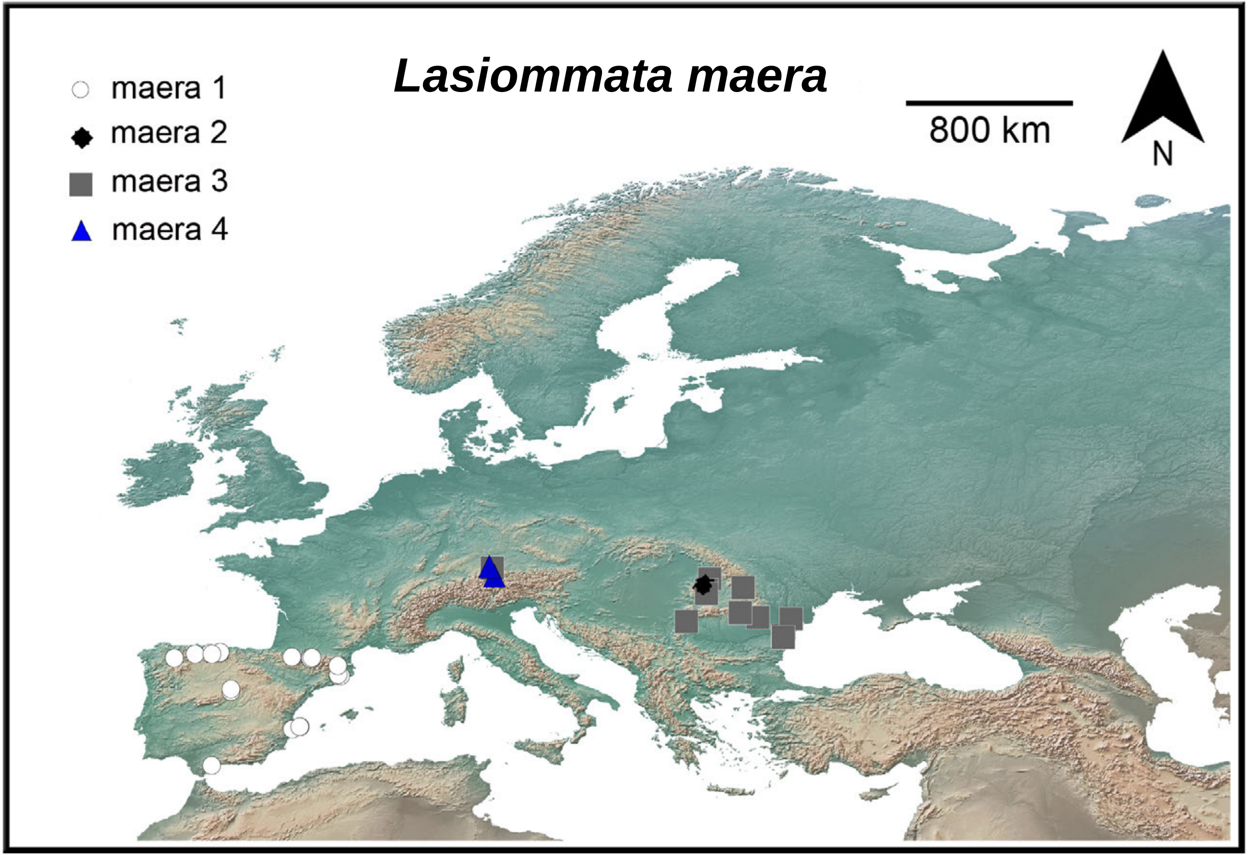
Lasiommata maera
maera 1
maera 2
maera 3
maera 4
800 km
N

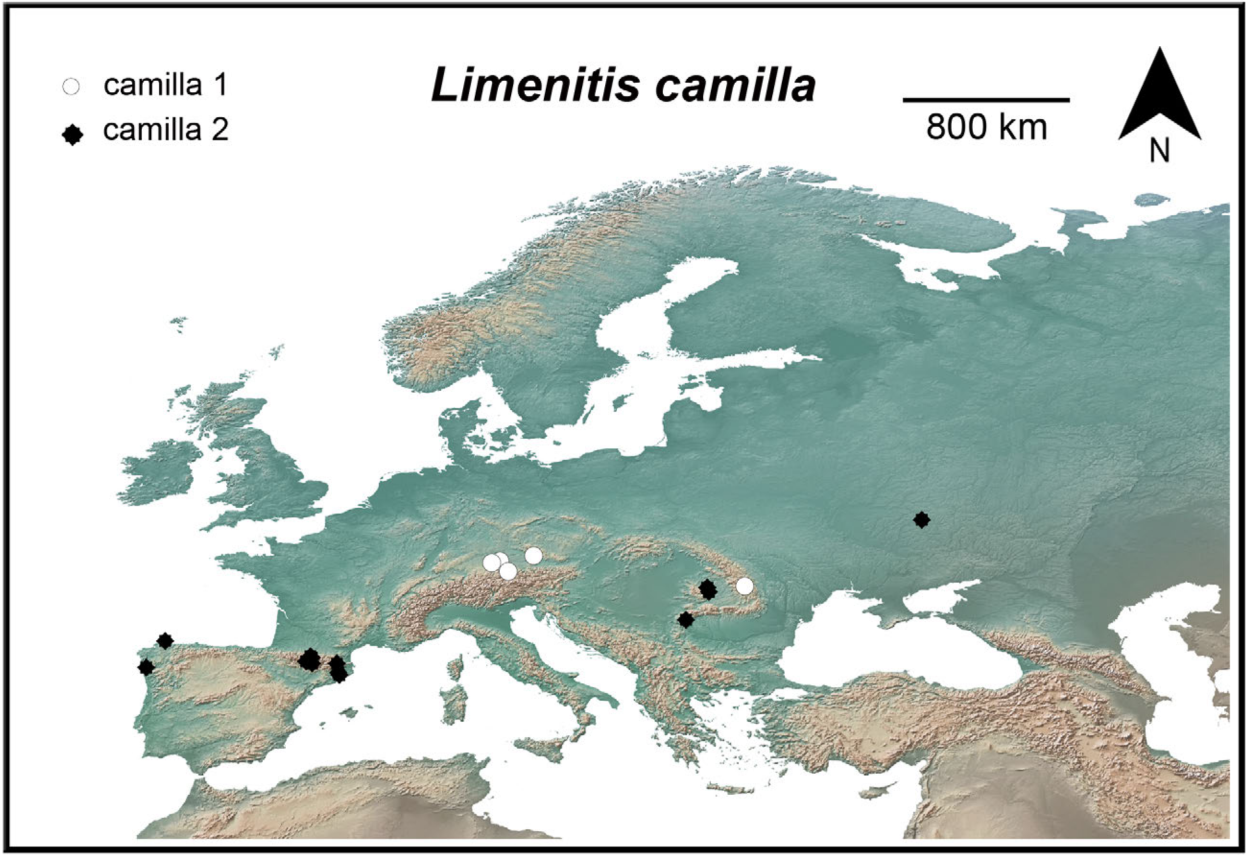
camilla 1
camilla 2
Limenitis camilla
800 km
N

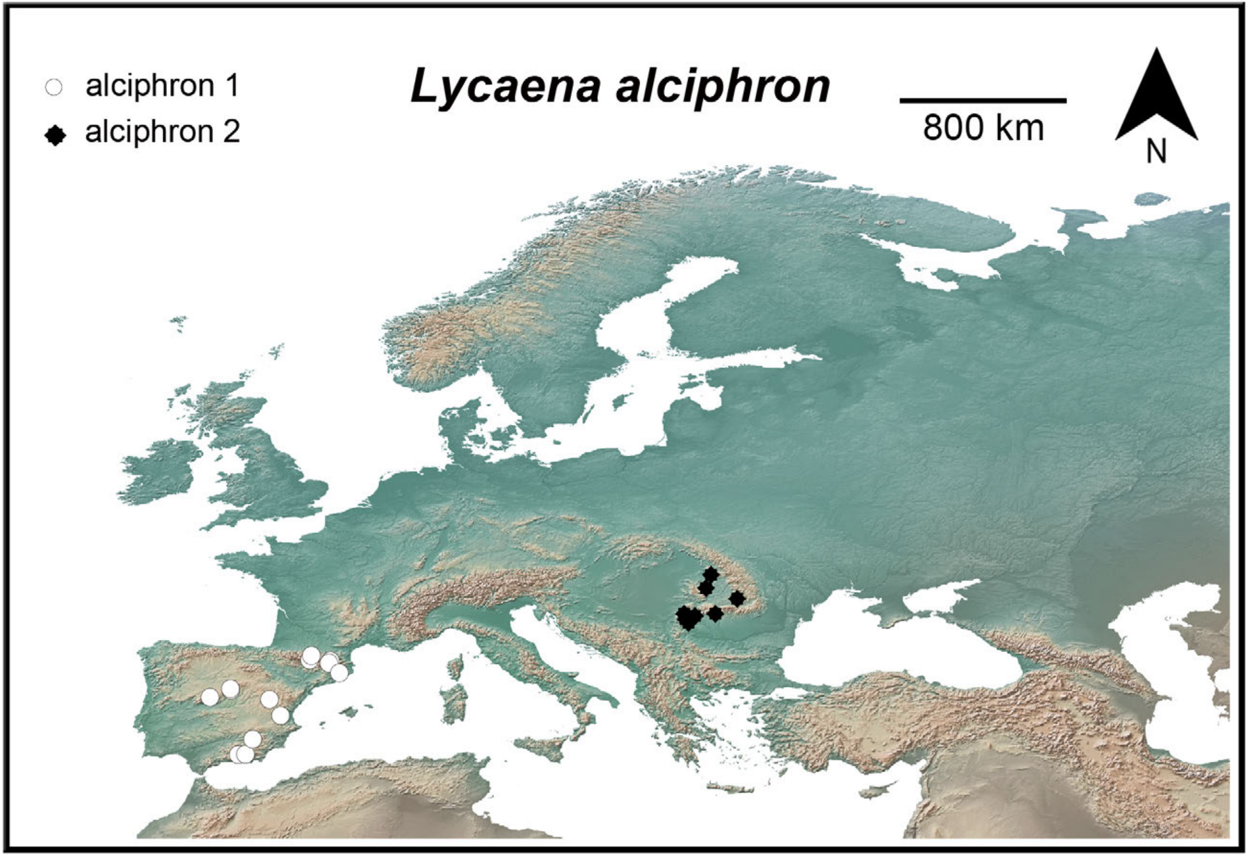

alciphron 1
alciphron 2
Lycaena alciphron
800 km
N

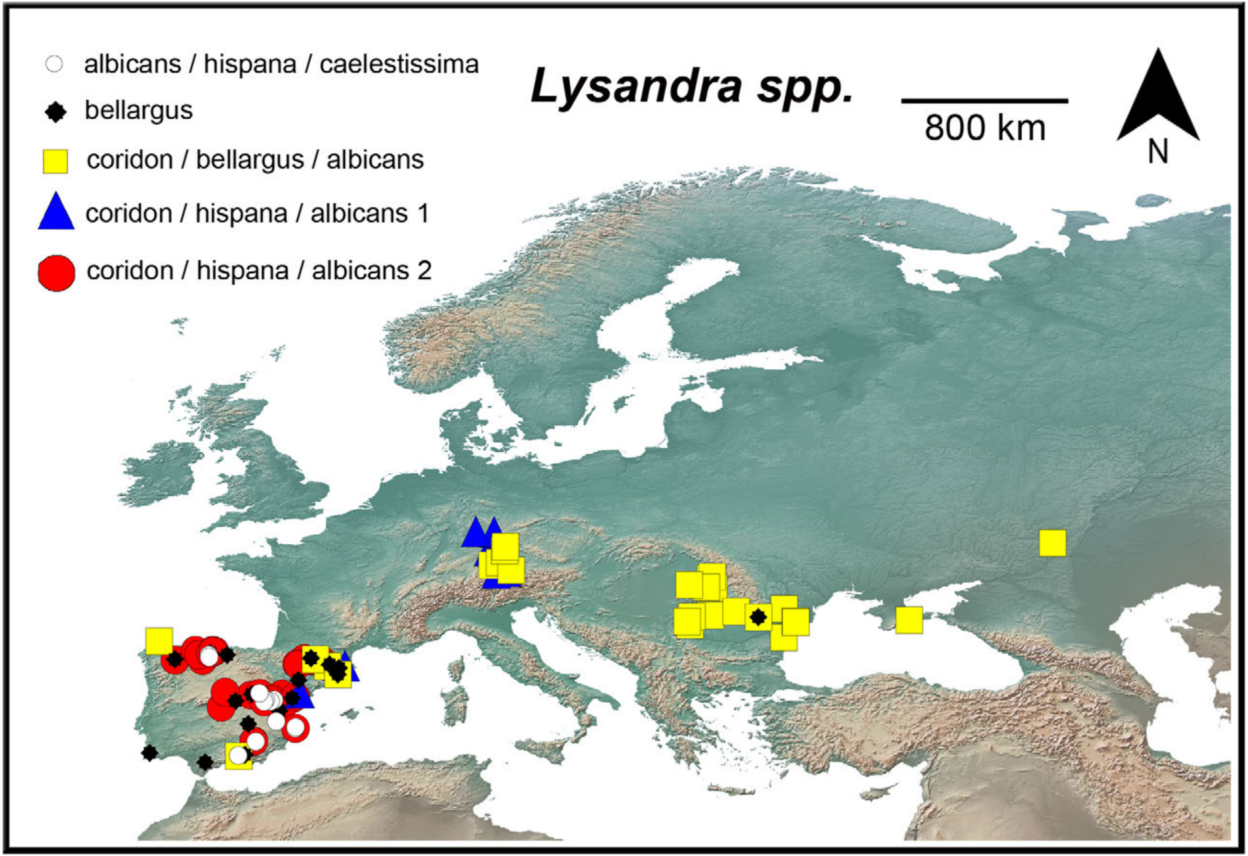
albicans / hispana / caelestissima
bellargus
coridon / bellargus / albicans
coridon / hispana / albicans 1
coridon / hispana / albicans 2
Lysandra spp.
800 km
N

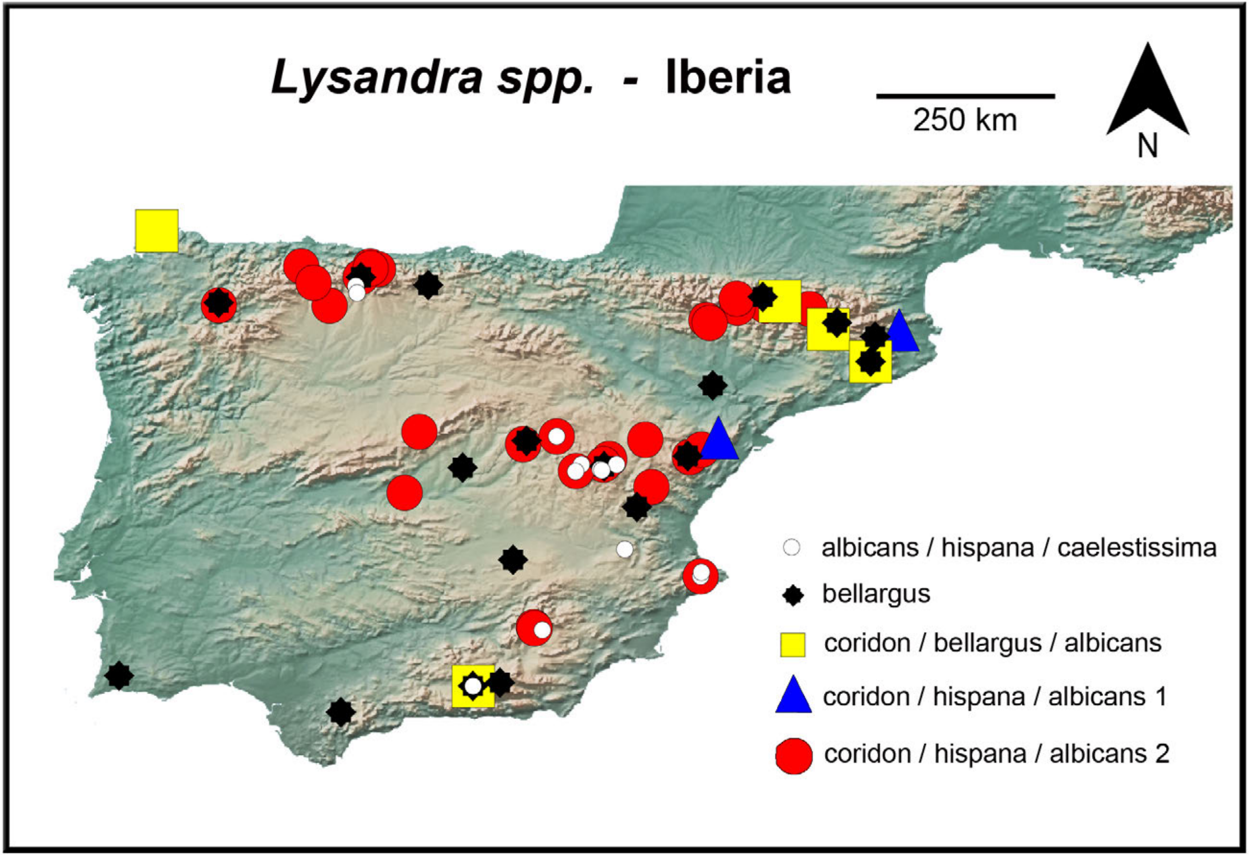
Lysandra spp. - Iberia
250 km
N
albicans / hispana / caelestissima
bellargus
coridon / bellargus / albicans
coridon / hispana / albicans 1
coridon / hispana / albicans 2

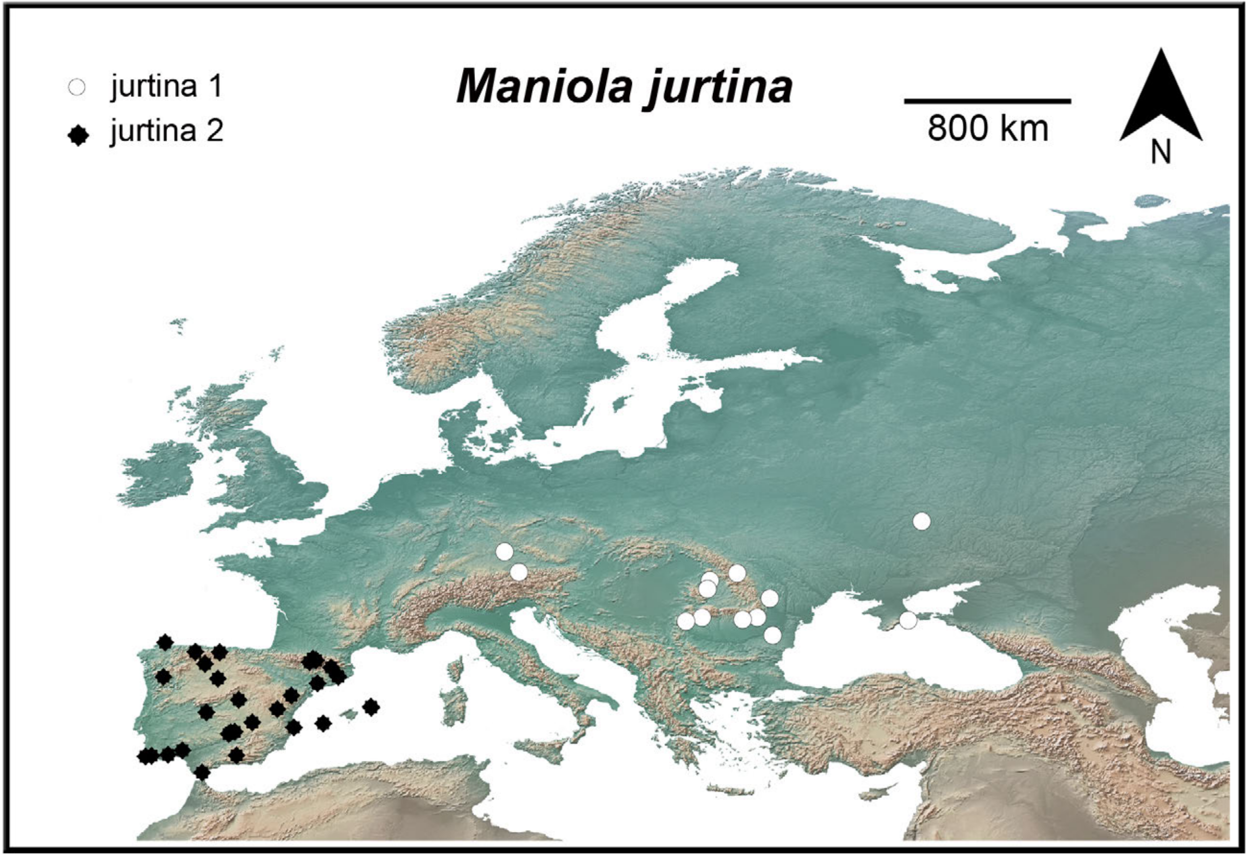
Maniola jurtina
jurtina 1
jurtina 2
800 km
N

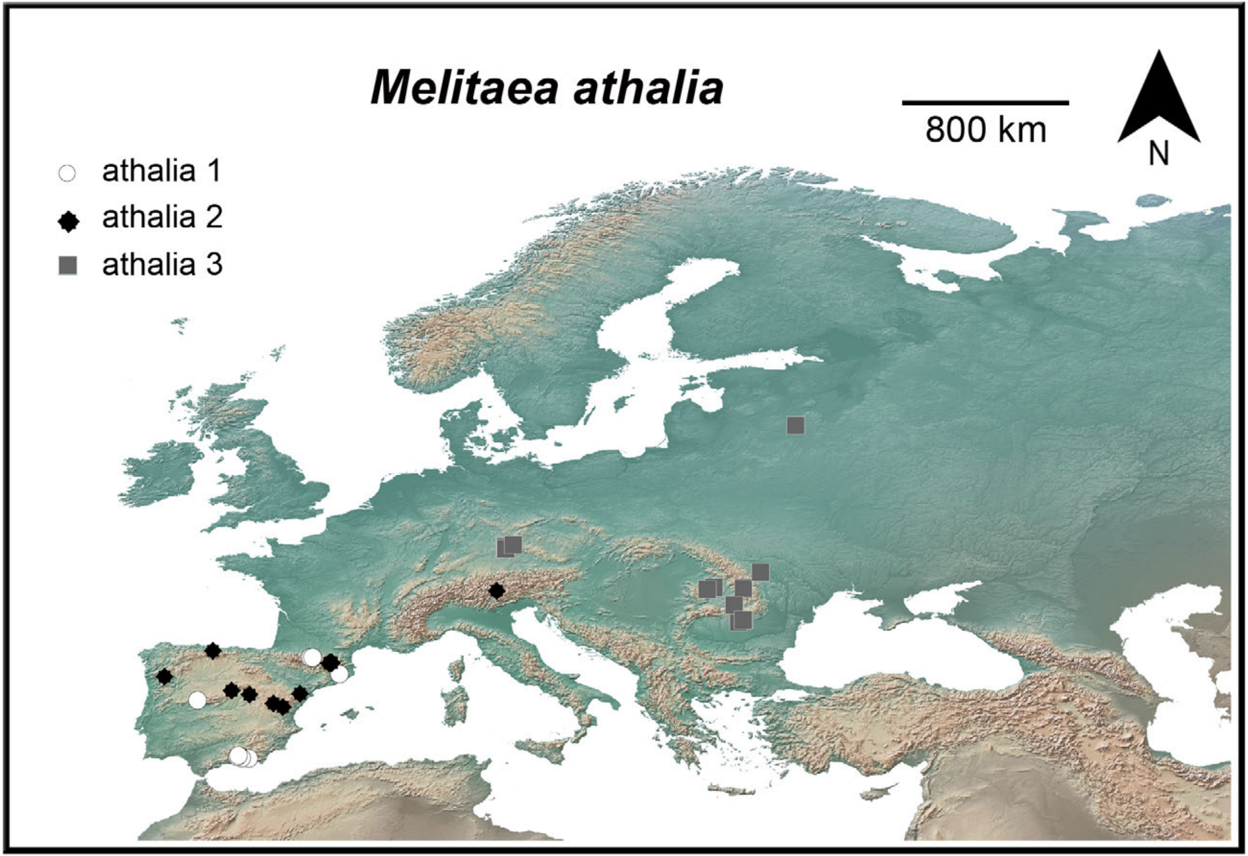

Melitaea athalia
800 km
N
athalia 1
athalia 2
athalia 3

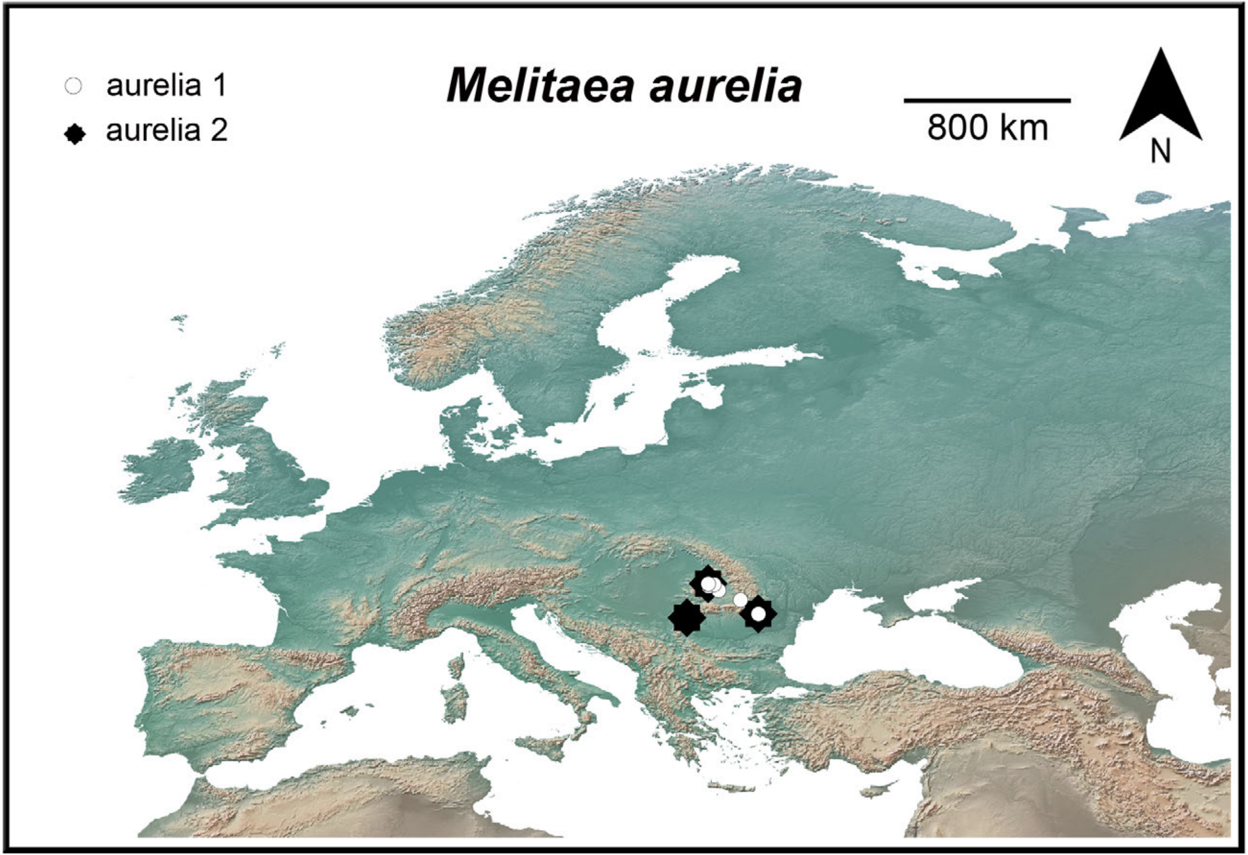
aurelia 1
aurelia 2
Melitaea aurelia
800 km
N

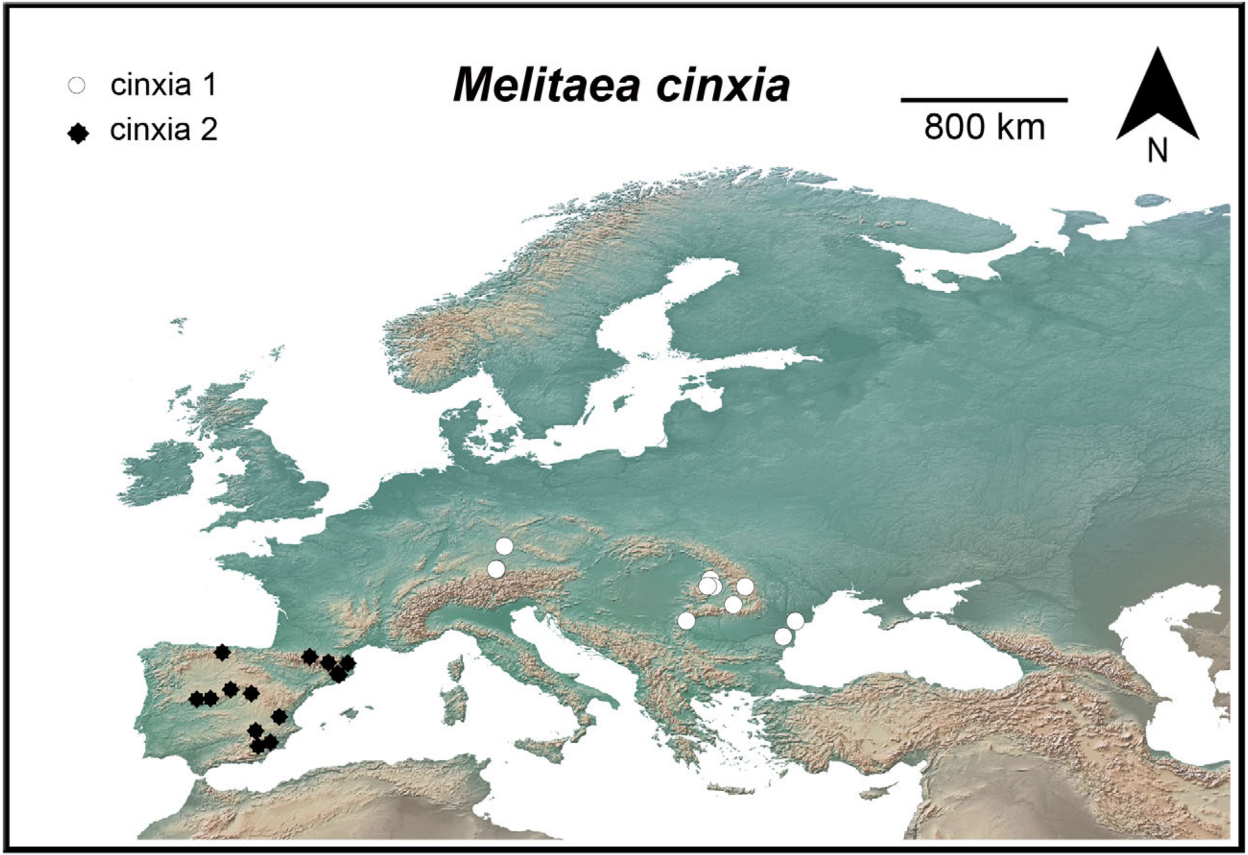
Melitaea cinxia
cinxia 1
cinxia 2
800 km
N

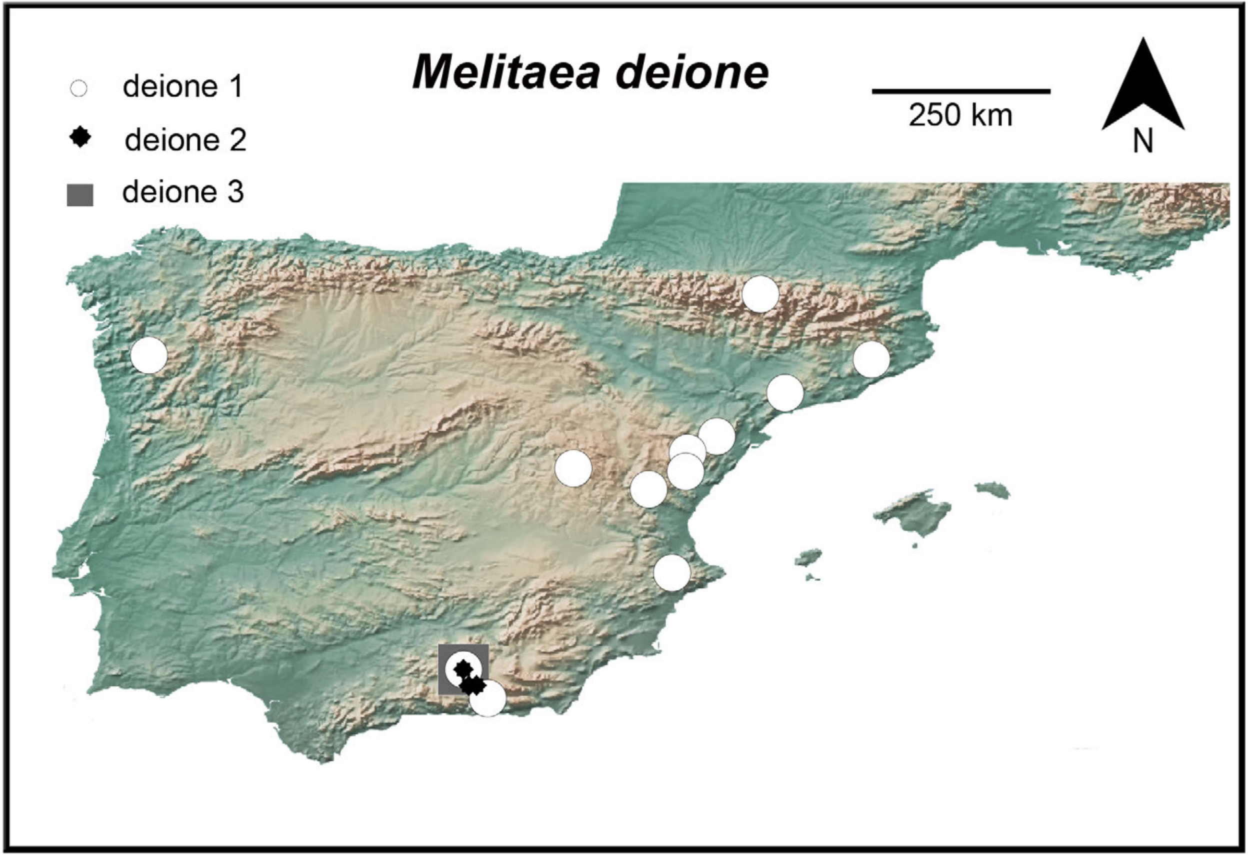
Melitaea deione
deione 1
deione 2
deione 3
250 km
N

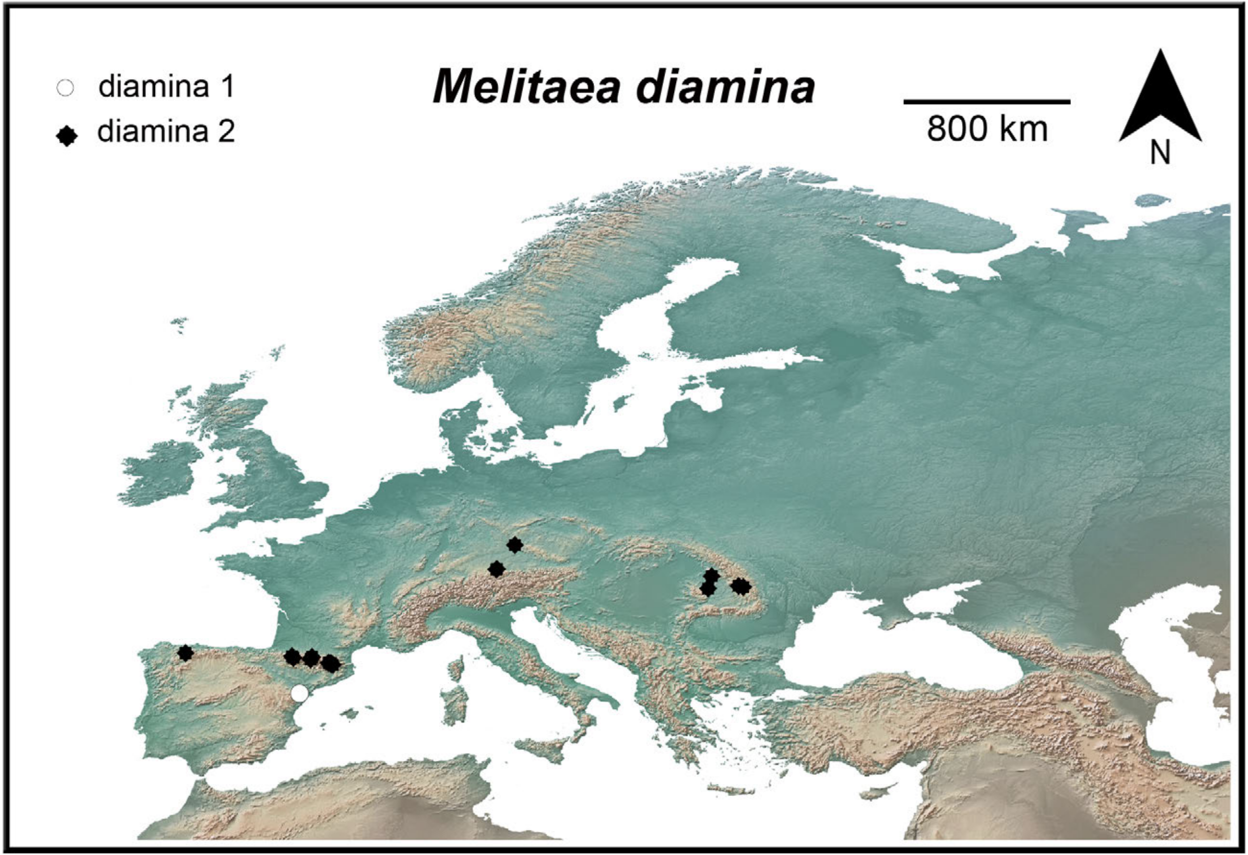
Melitaea diamina
diamina 1
diamina 2
800 km
N

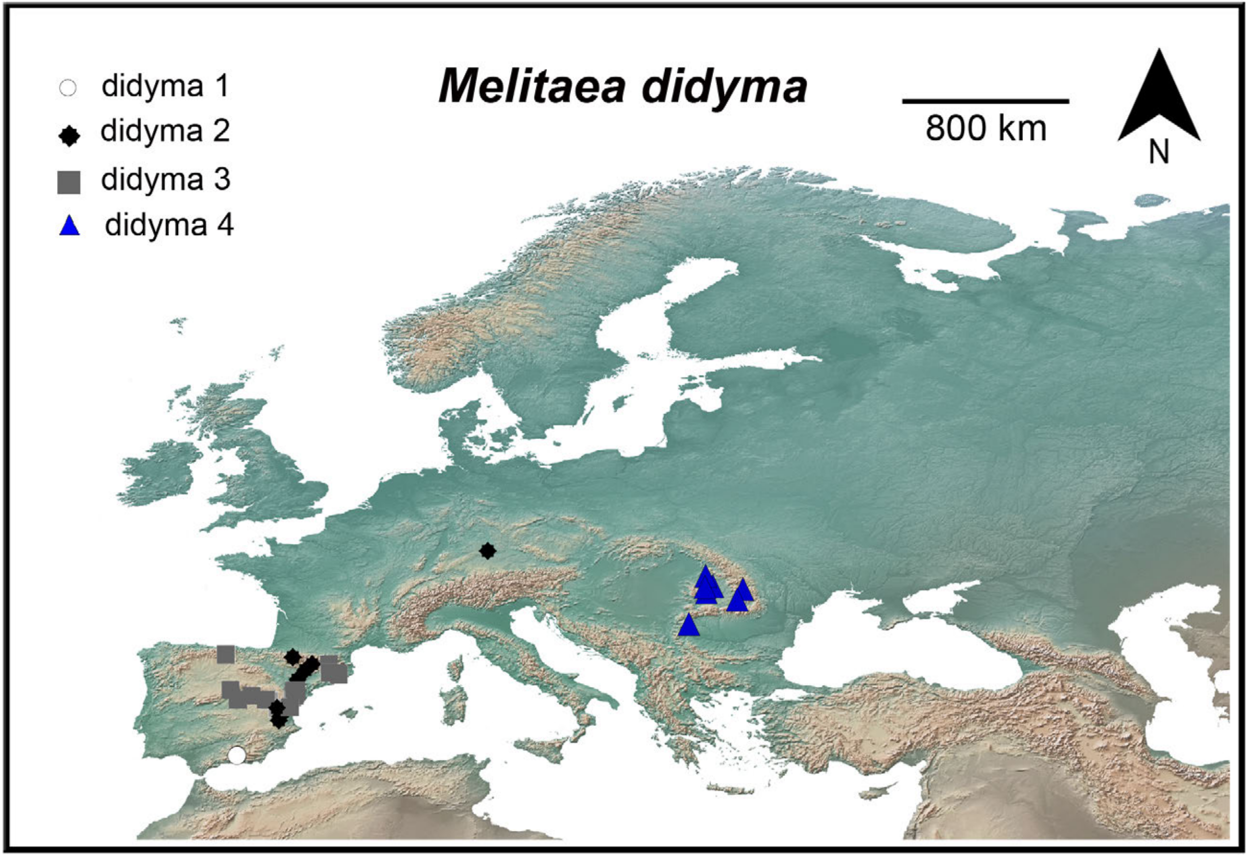
Melitaea didyma
didyma 1
didyma 2
didyma 3
didyma 4
800 km
N

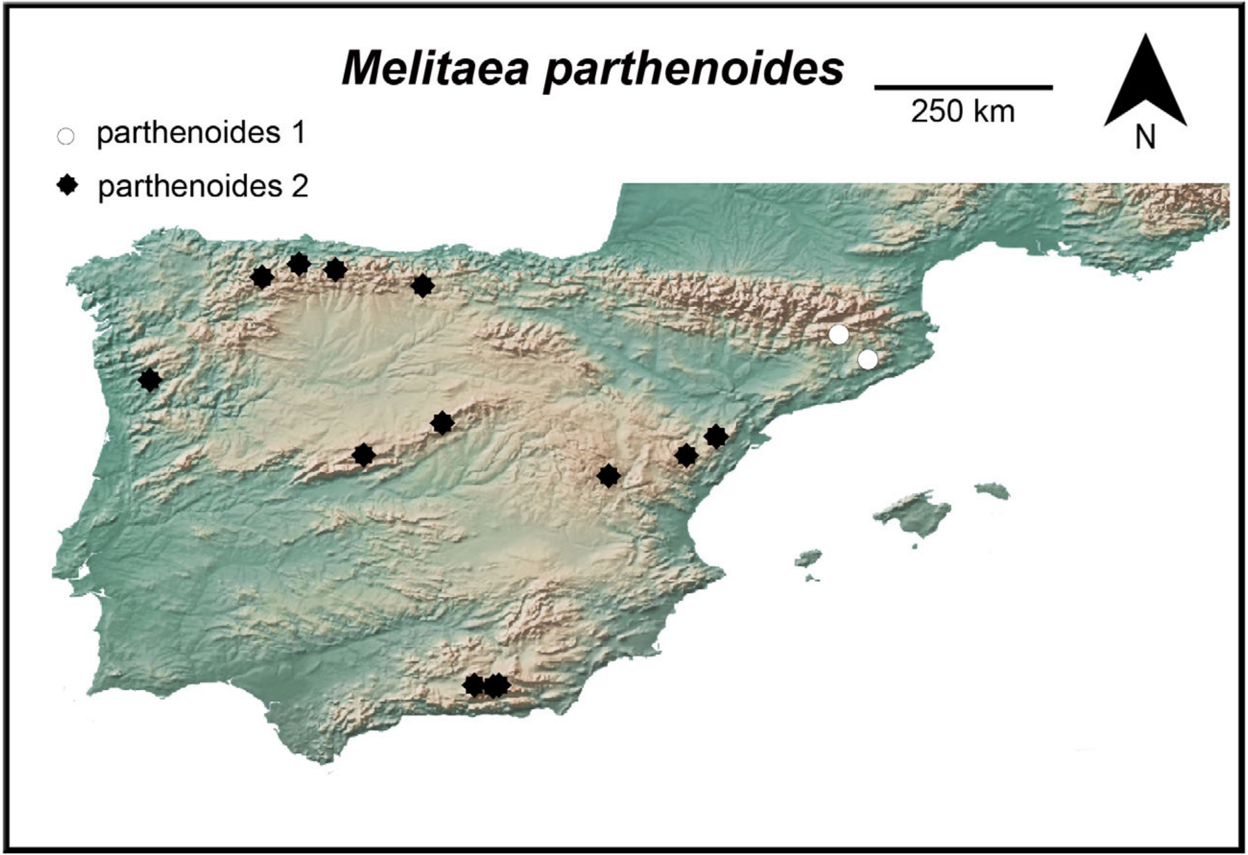

Melitaea parthenoides
250 km
N
parthenoides 1
parthenoides 2

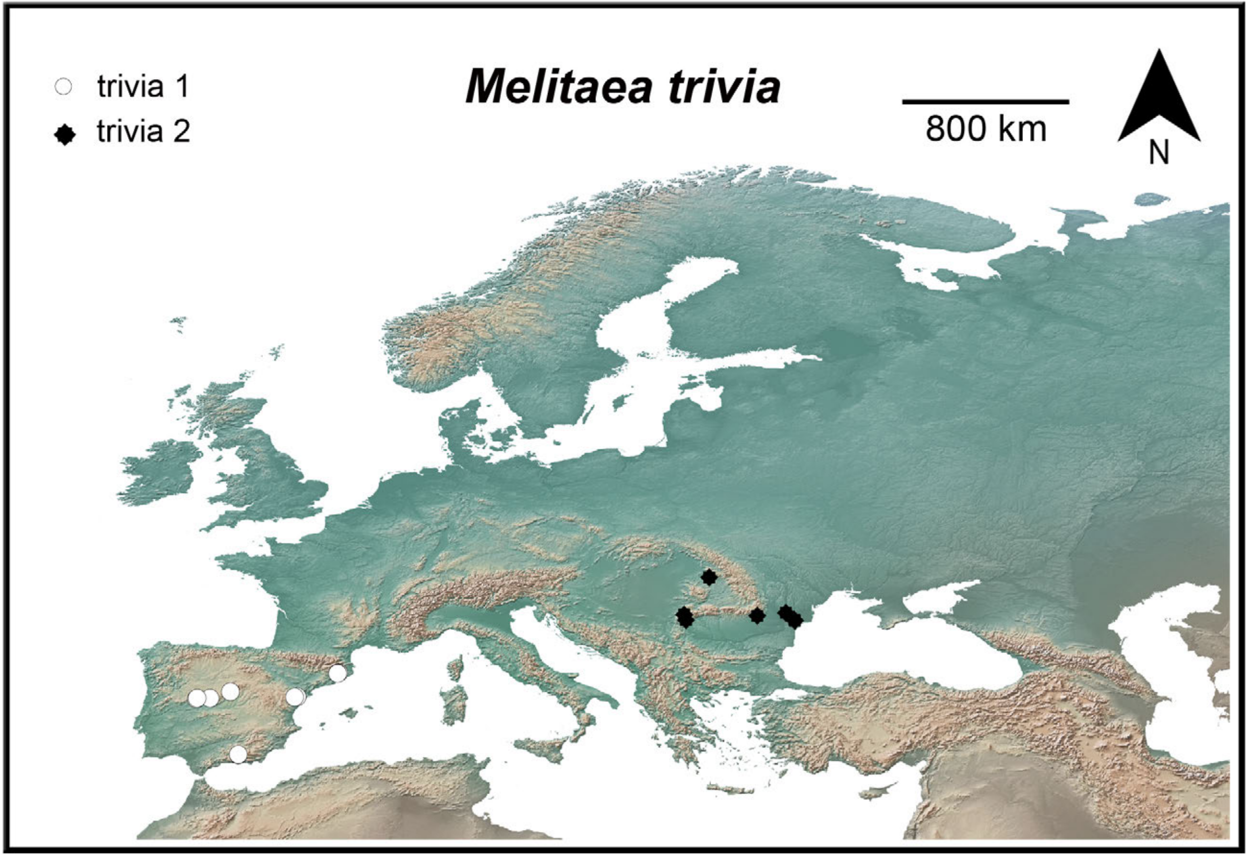
trivia 1
trivia 2
Melitaea trivia
800 km
N

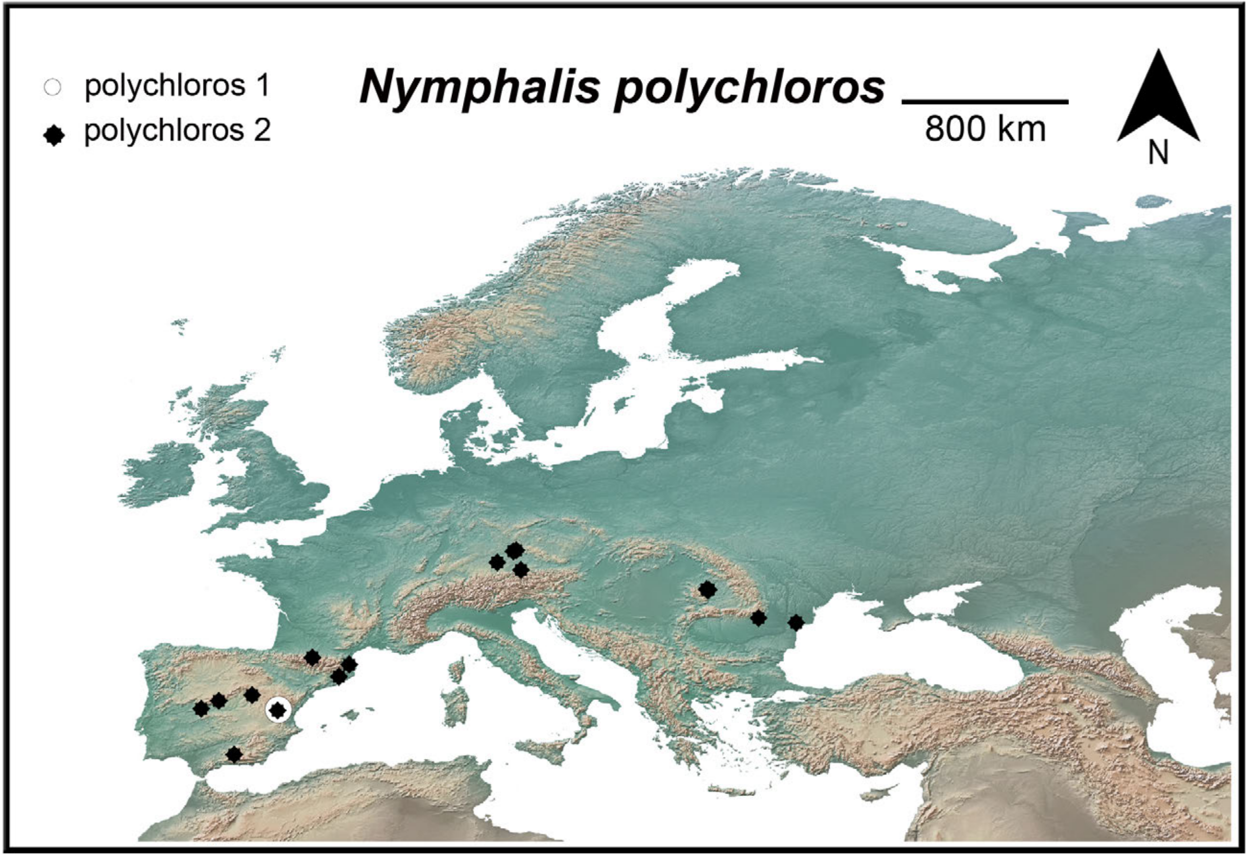
polychloros 1
polychloros 2
Nymphalis polychloros
800 km
N

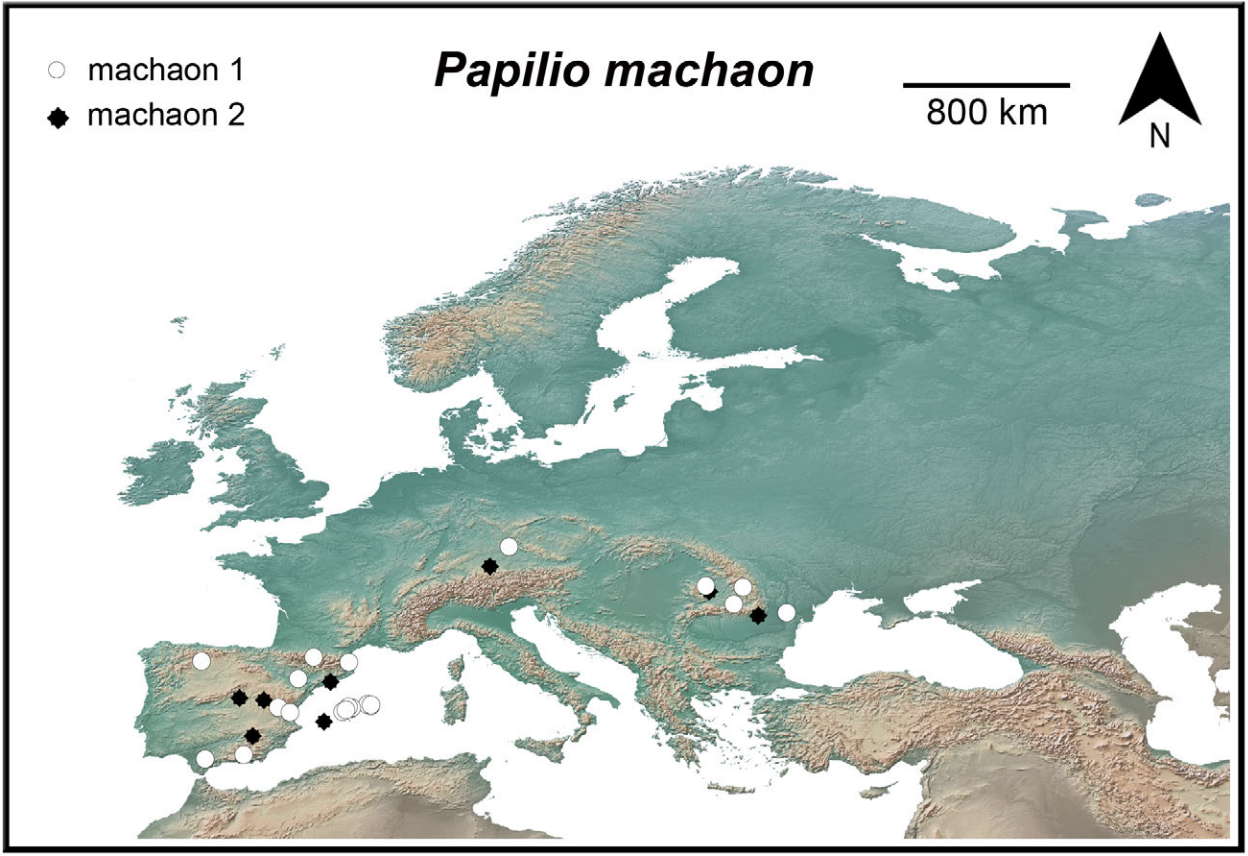
machaon 1
machaon 2
Papilio machaon
800 km
N

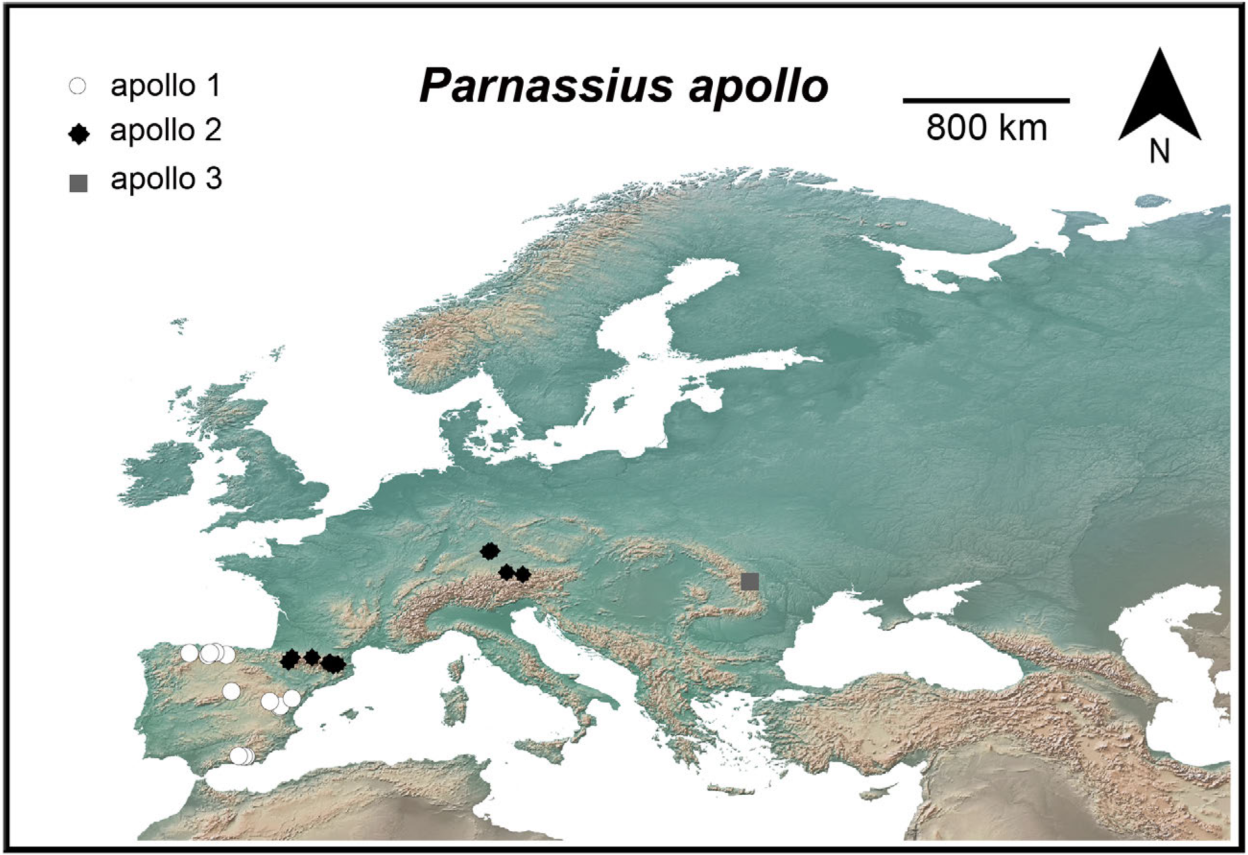
Parnassius apollo
apollo 1
apollo 2
apollo 3
800 km
N

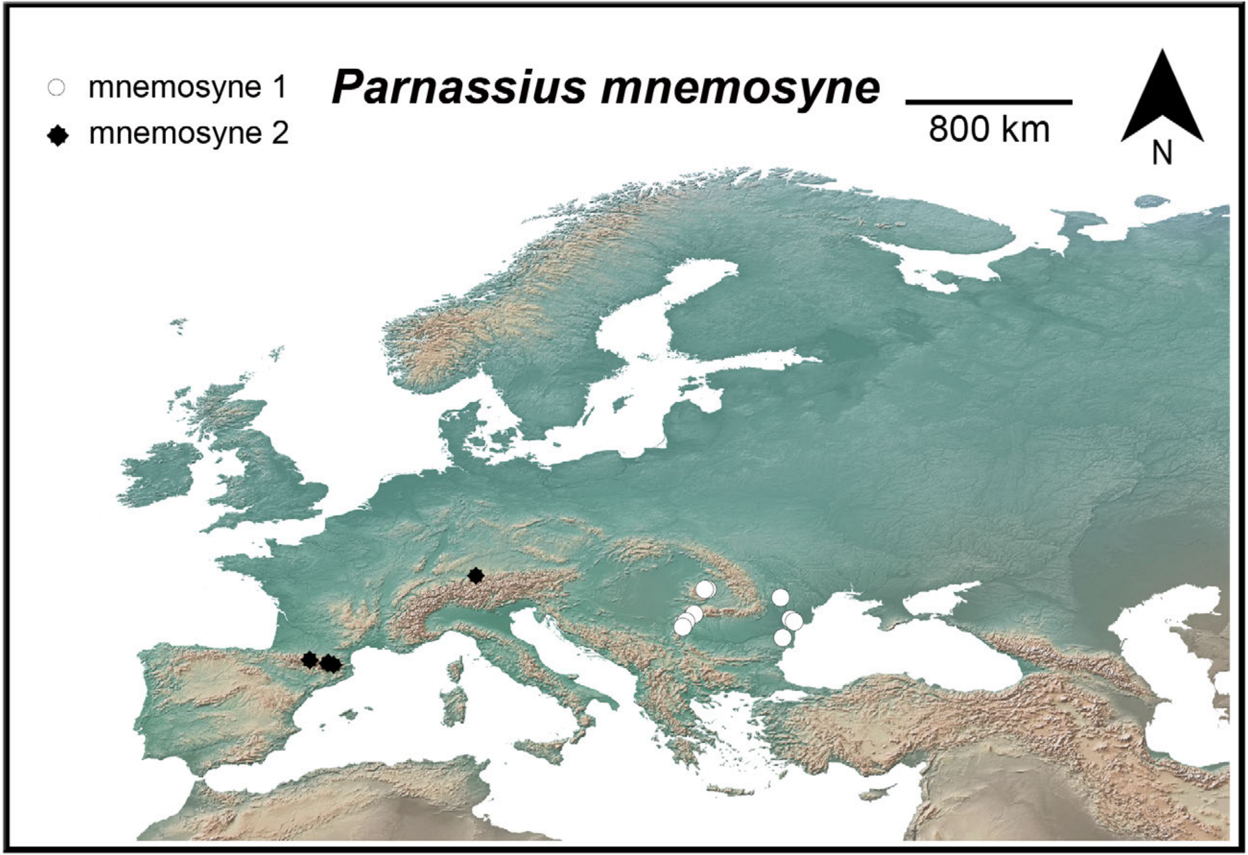
mnemosyne 1
mnemosyne 2
Parnassius mnemosyne
800 km
N

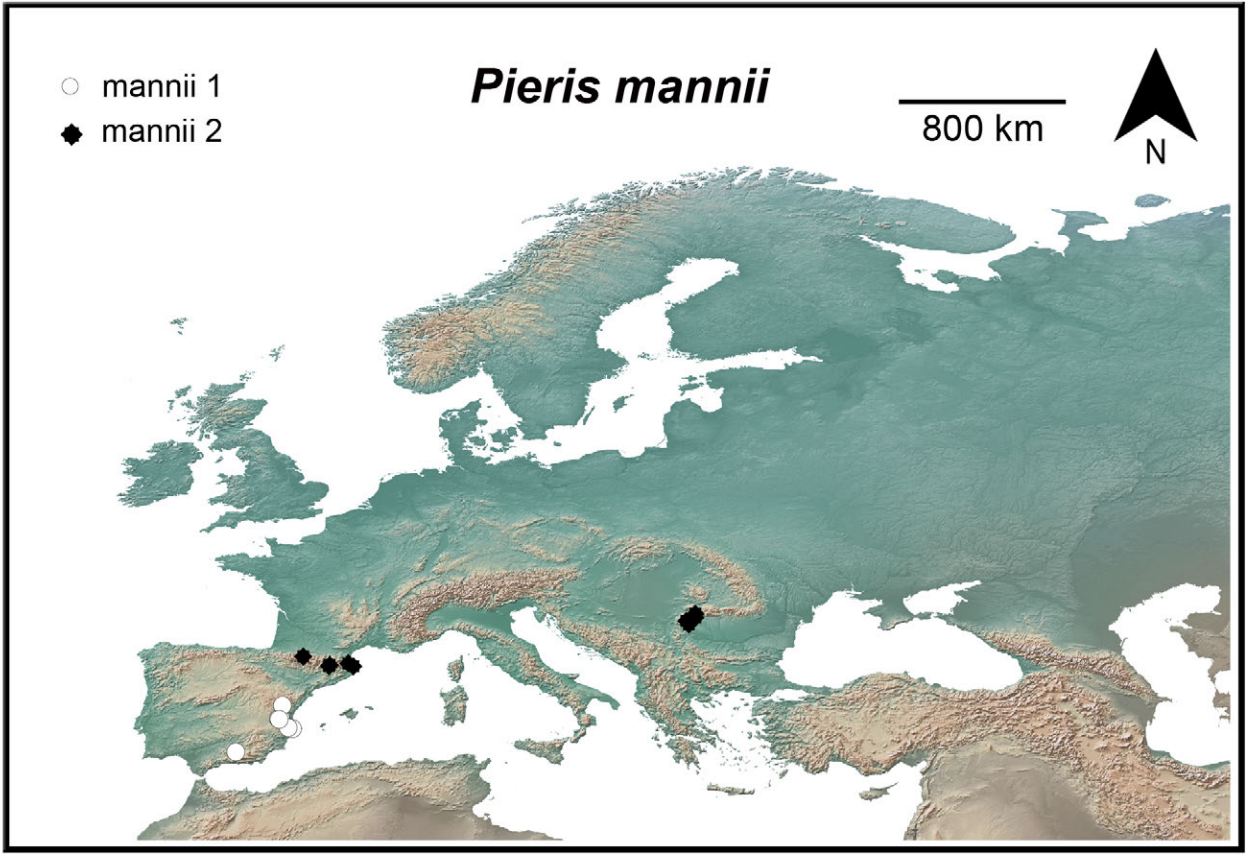
mannii 1
mannii 2
Pieris mannii
800 km
N

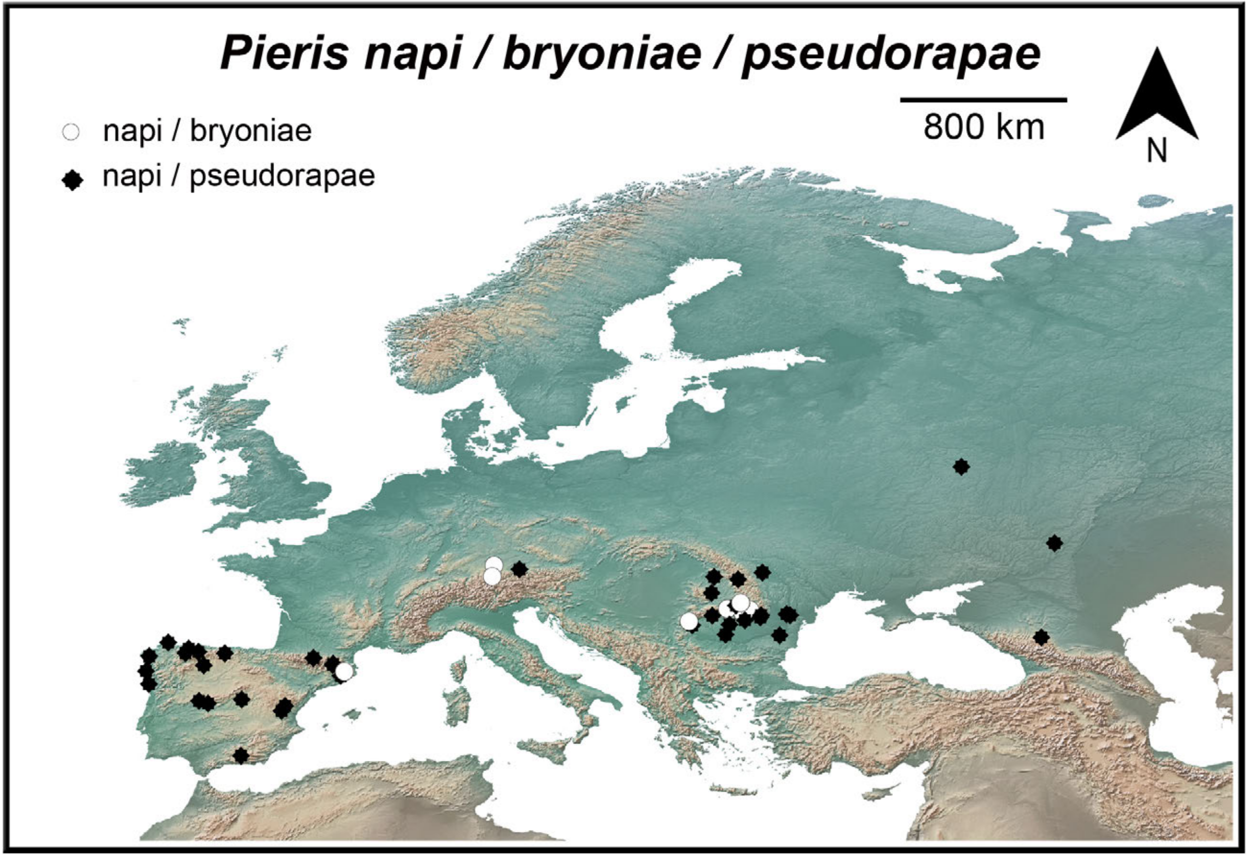
Pieris napi / bryoniae / pseudorapae
napi / bryoniae
napi / pseudorapae
800 km
N

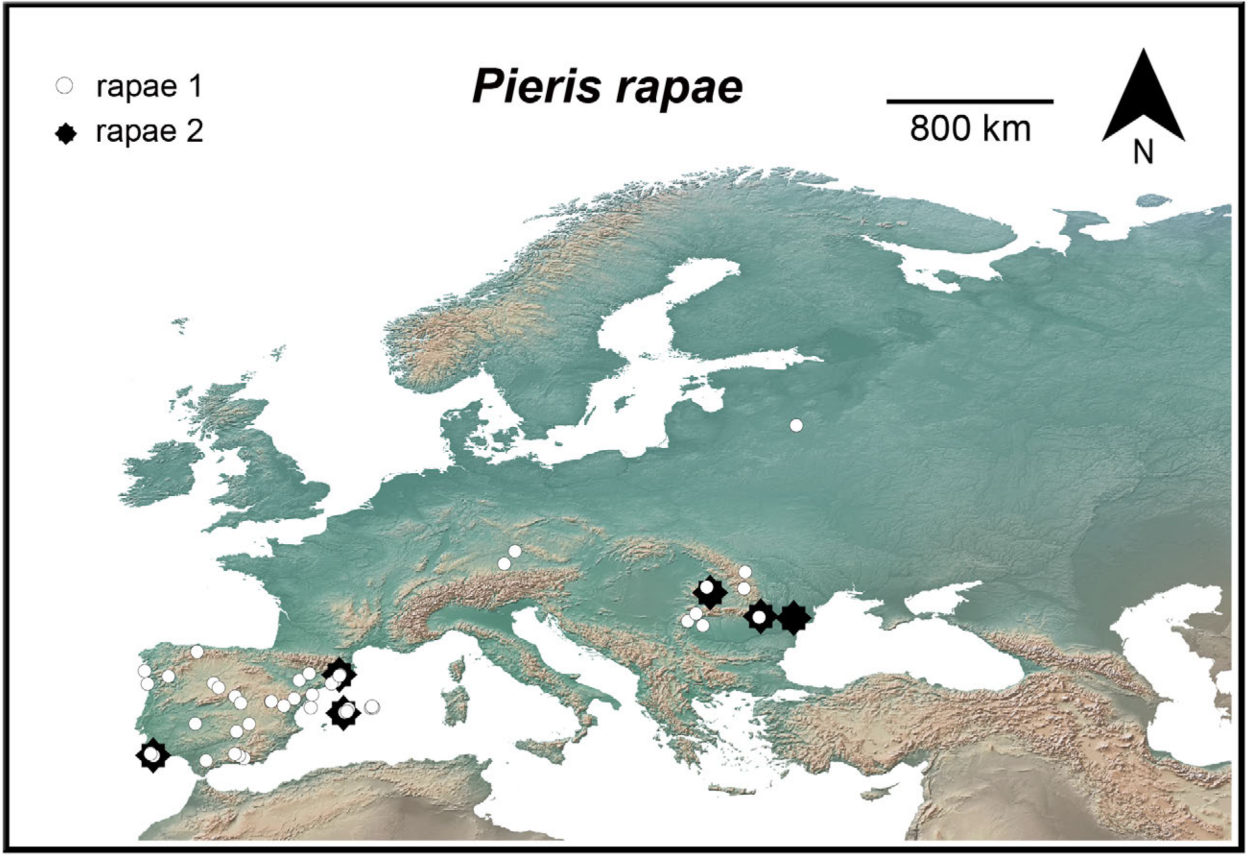
Pieris rapae
rapae 1
rapae 2
800 km
N

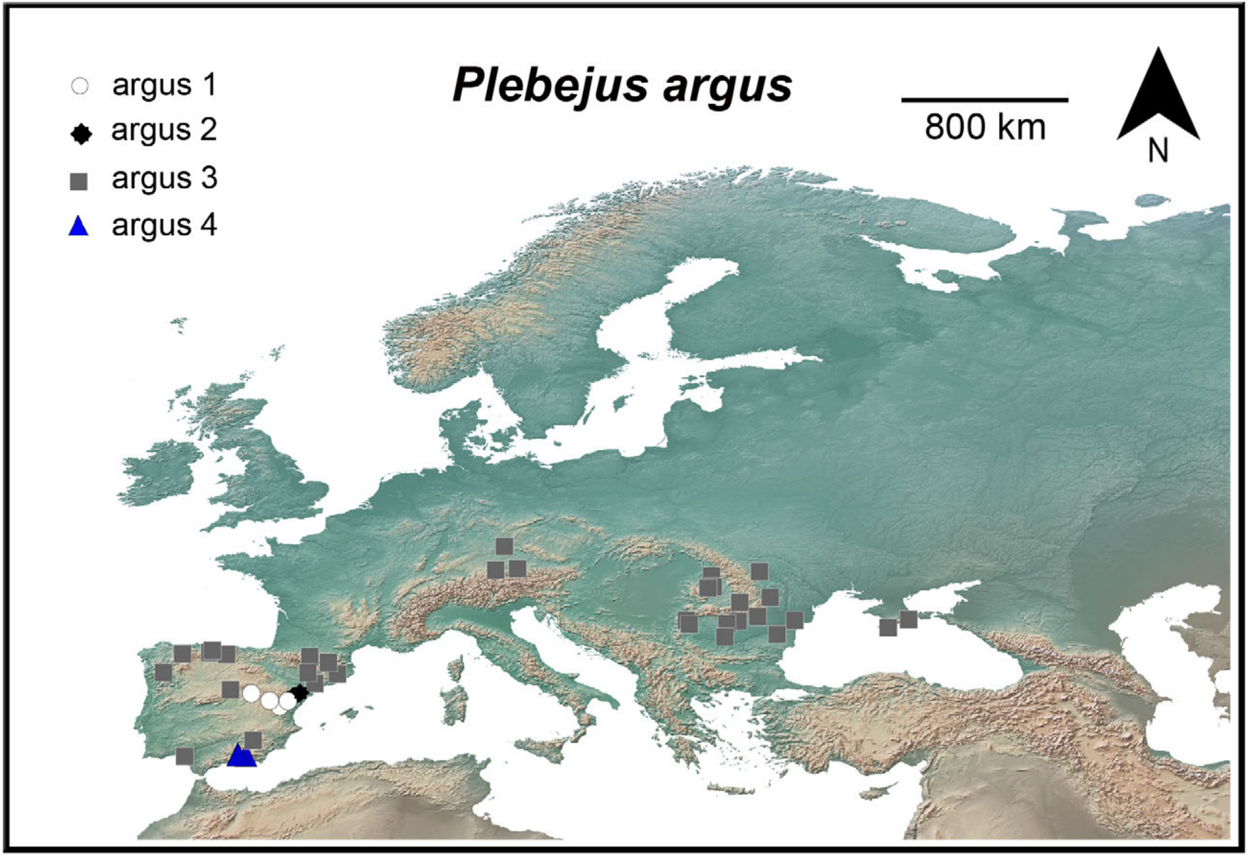
Plebejus argus
argus 1
argus 2
argus 3
argus 4
800 km
N

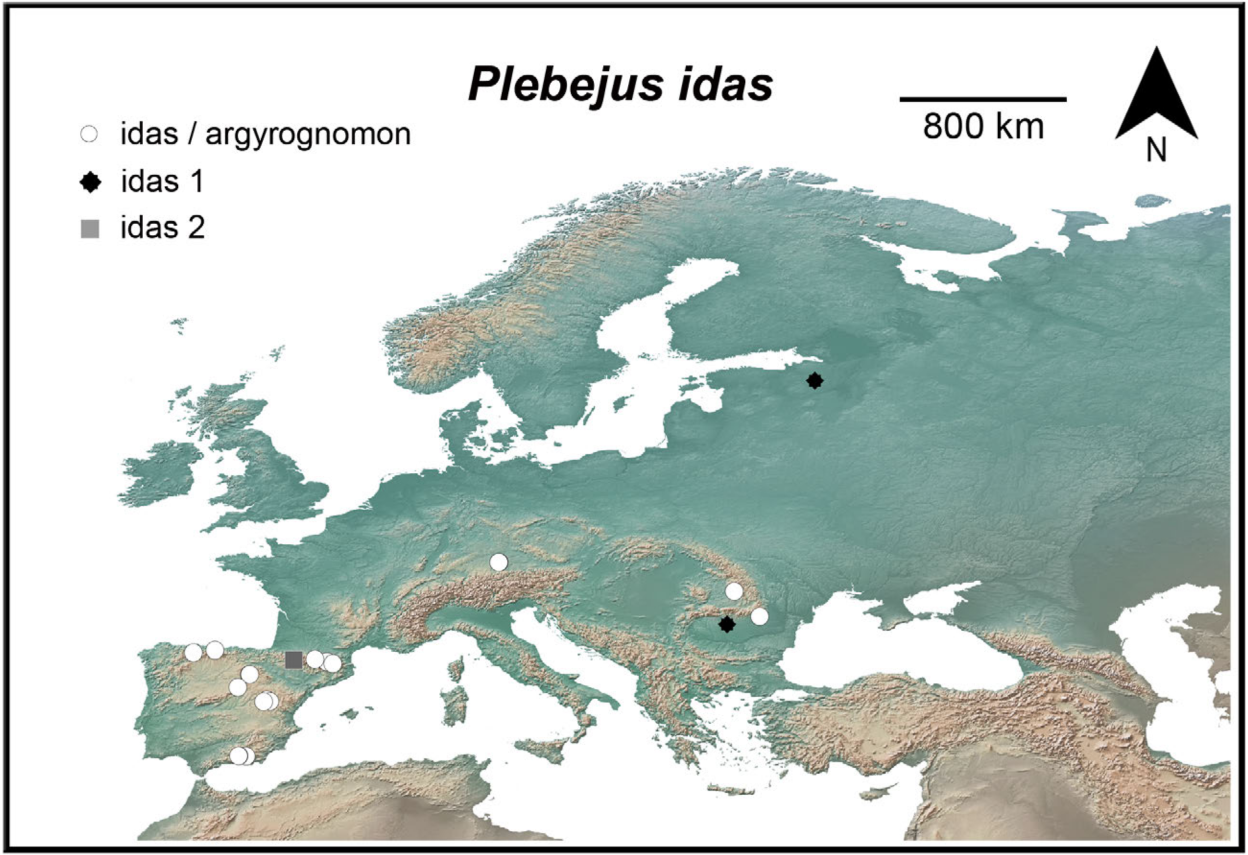

Plebejus idas
800 km
N
idas / argyrognomon
idas 1
idas 2

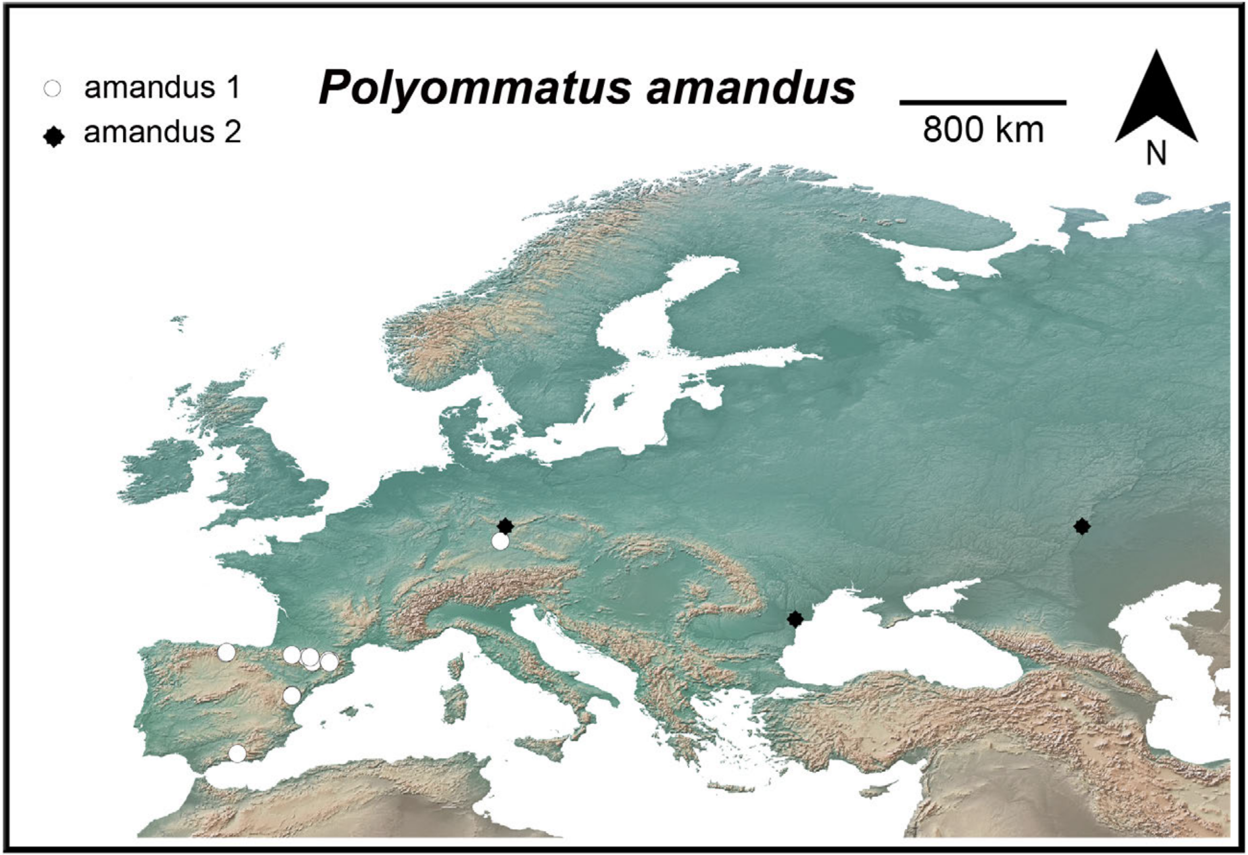
amandus 1
amandus 2
Polyommatus amandus
800 km
N

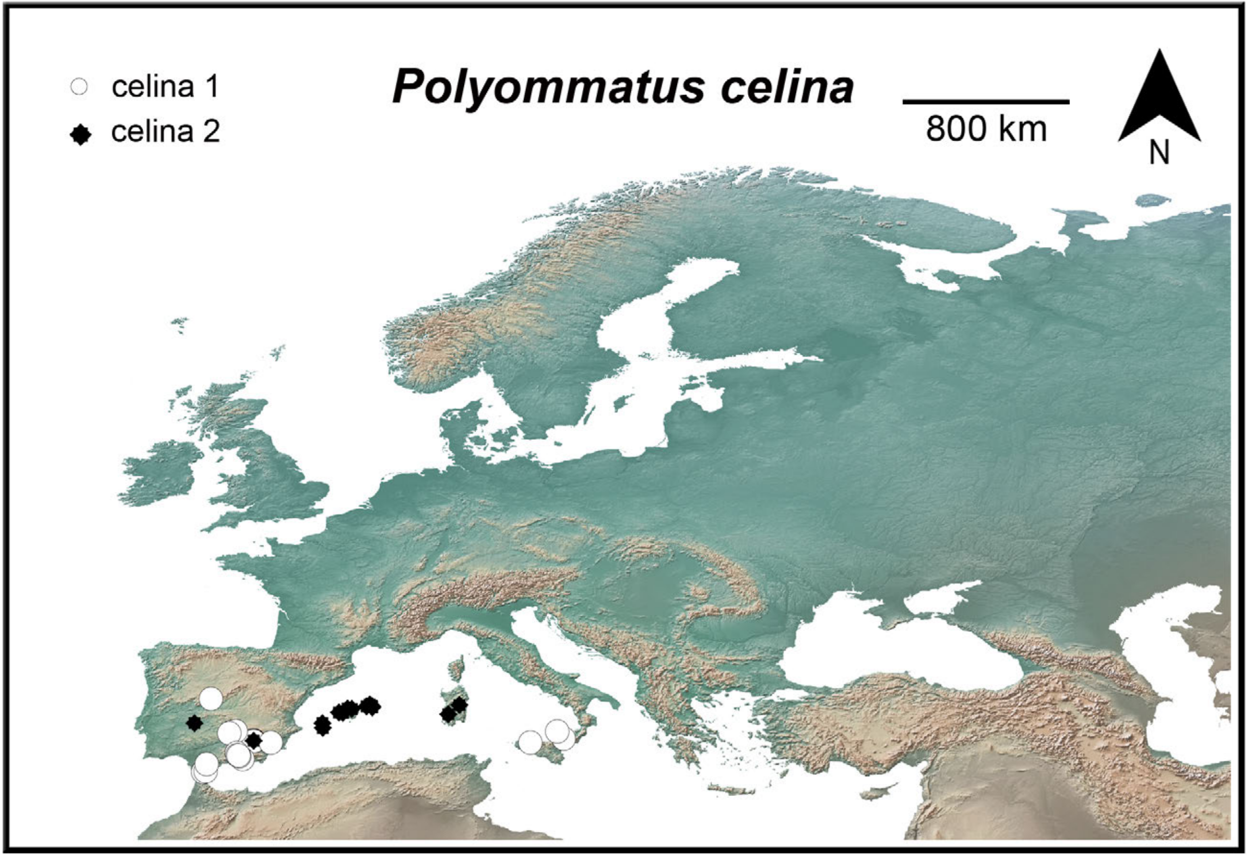
celina 1
celina 2
Polyommatus celina
800 km
N

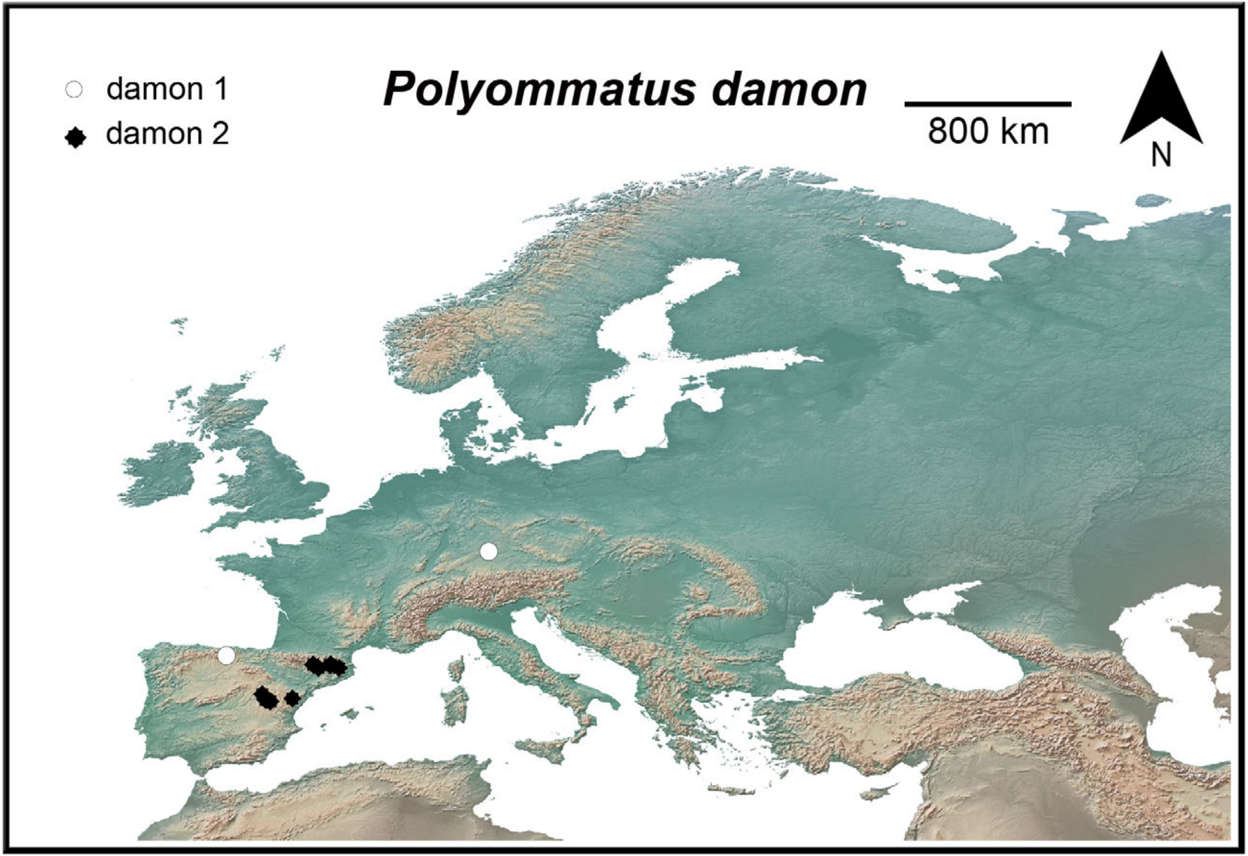
damon 1
damon 2
Polyommatus damon
800 km
N

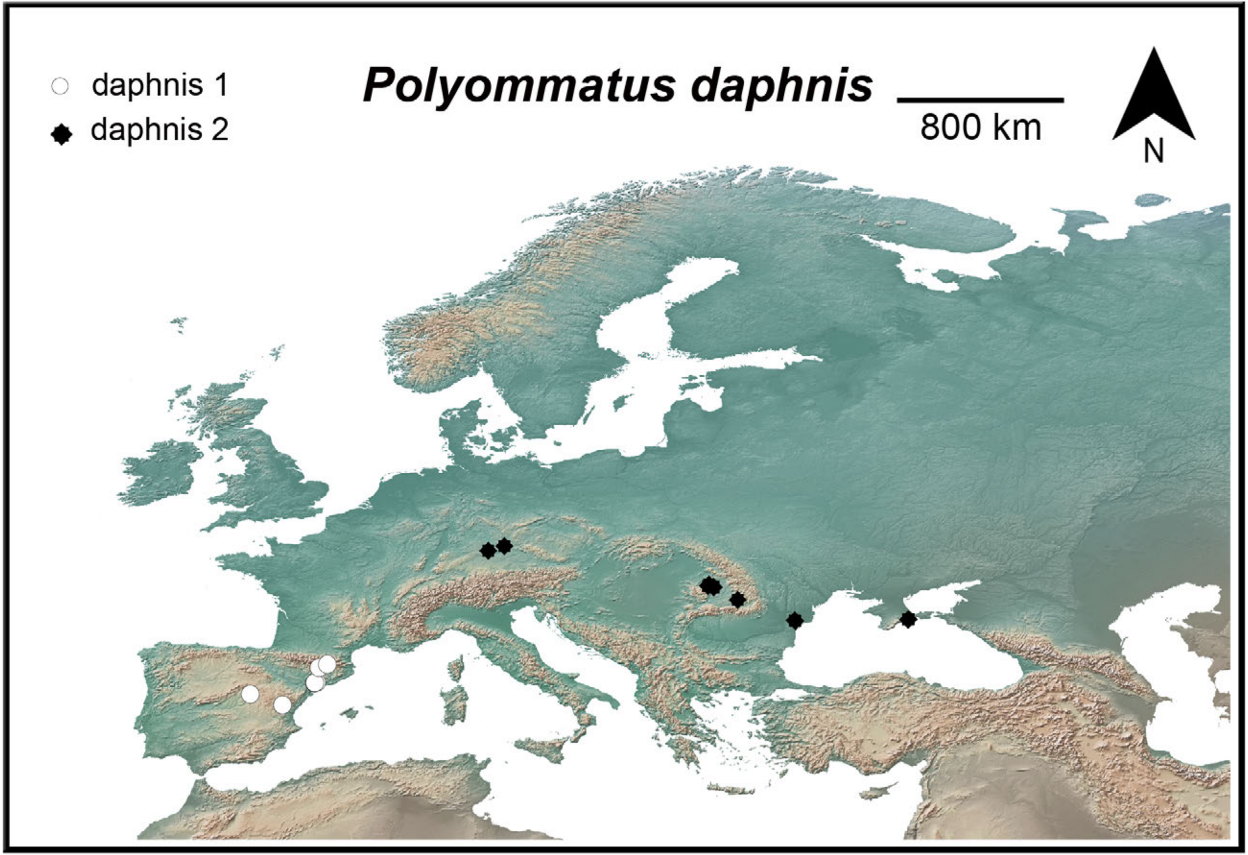
daphnis 1
daphnis 2
Polyommatus daphnis
800 km
N

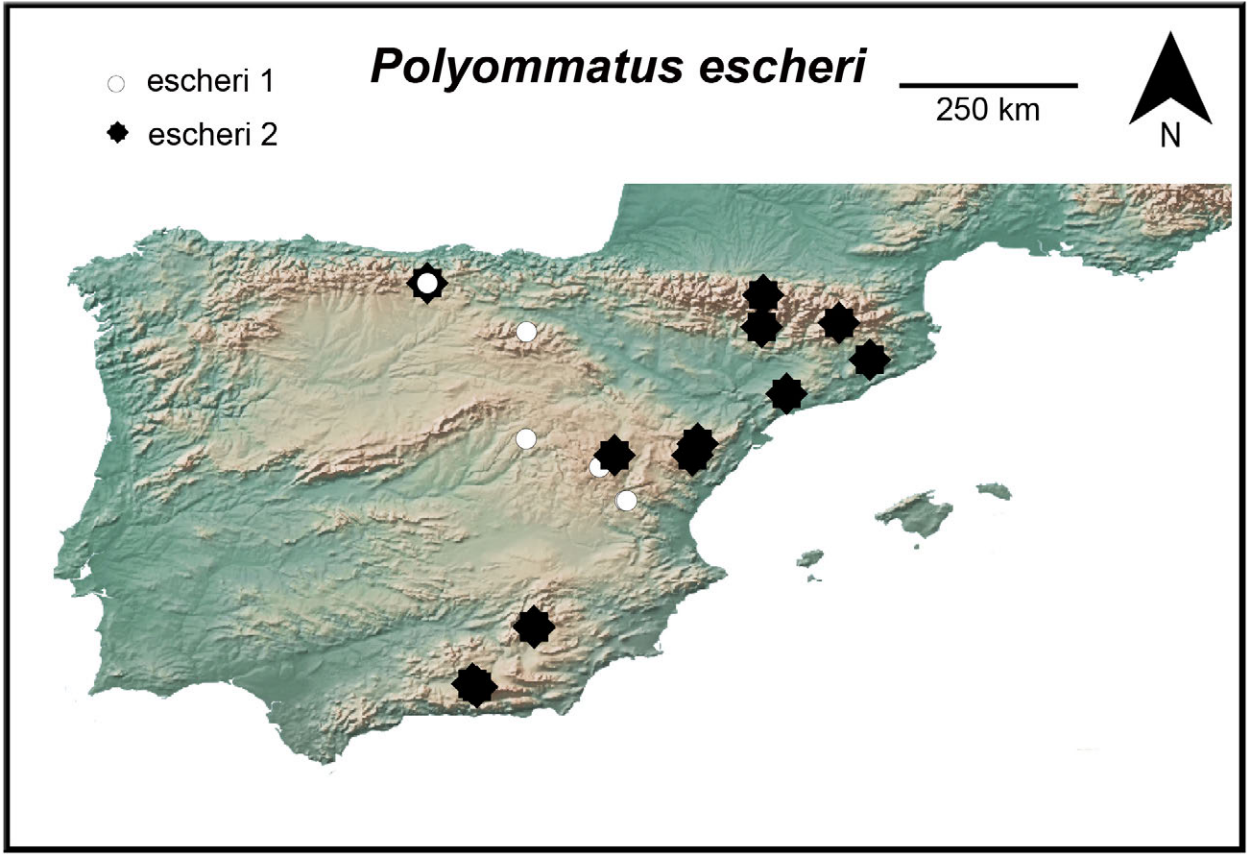
Polyommatus escheri
escheri 1
escheri 2
250 km
N

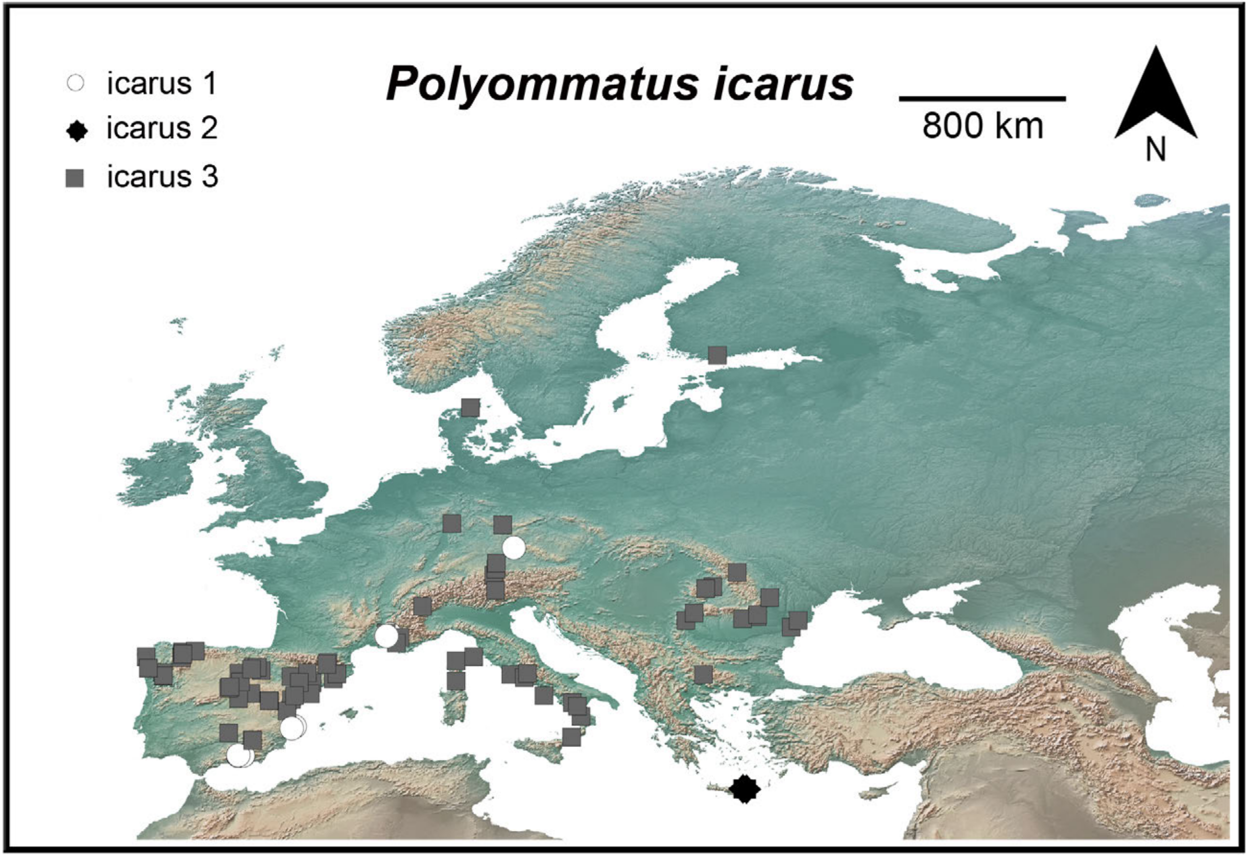
icarus 1
icarus 2
icarus 3
Polyommatus icarus
800 km
N

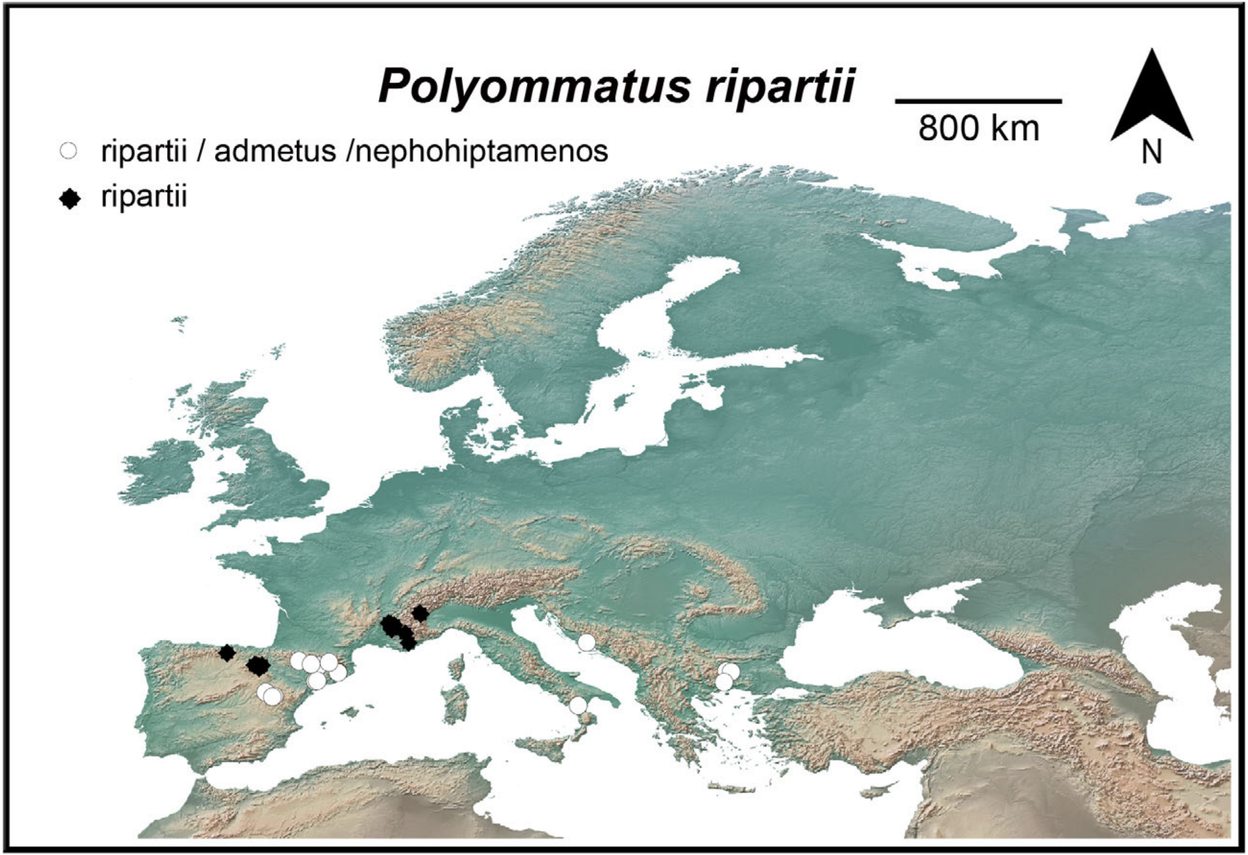

Polyommatus ripartii
800 km
N
ripartii / admetus /nephohiptamenos
ripartii

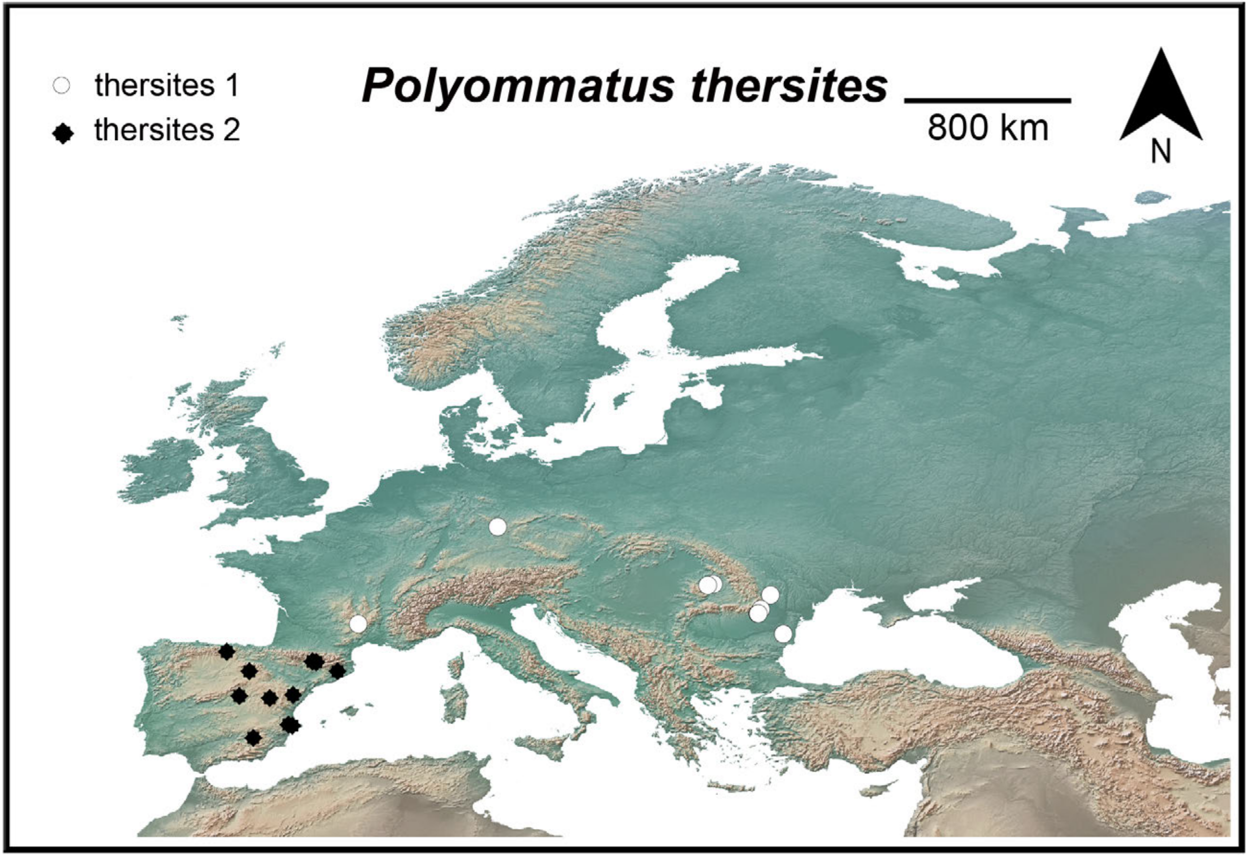
thersites 1
thersites 2
Polyommatus thersites
800 km
N

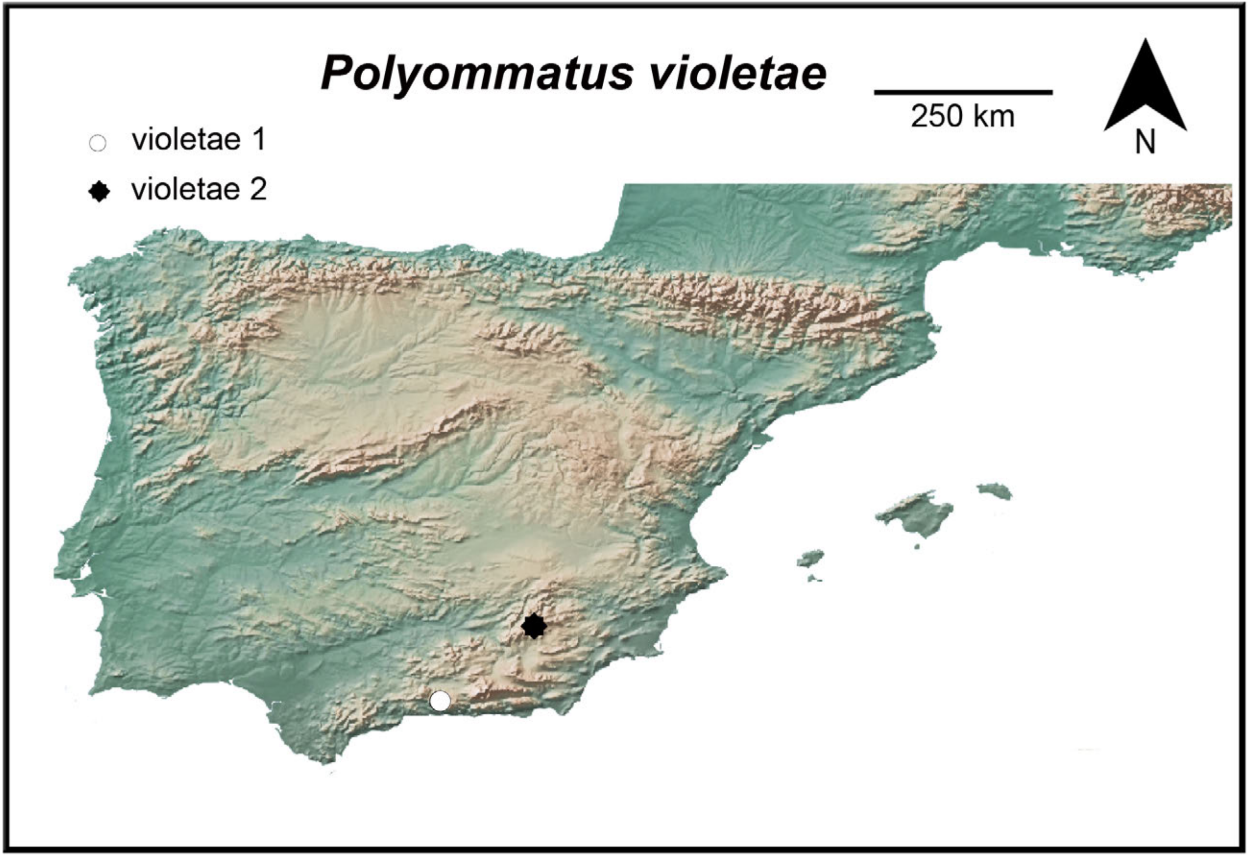
Polyommatus violetae
250 km
N
violetae 1
violetae 2

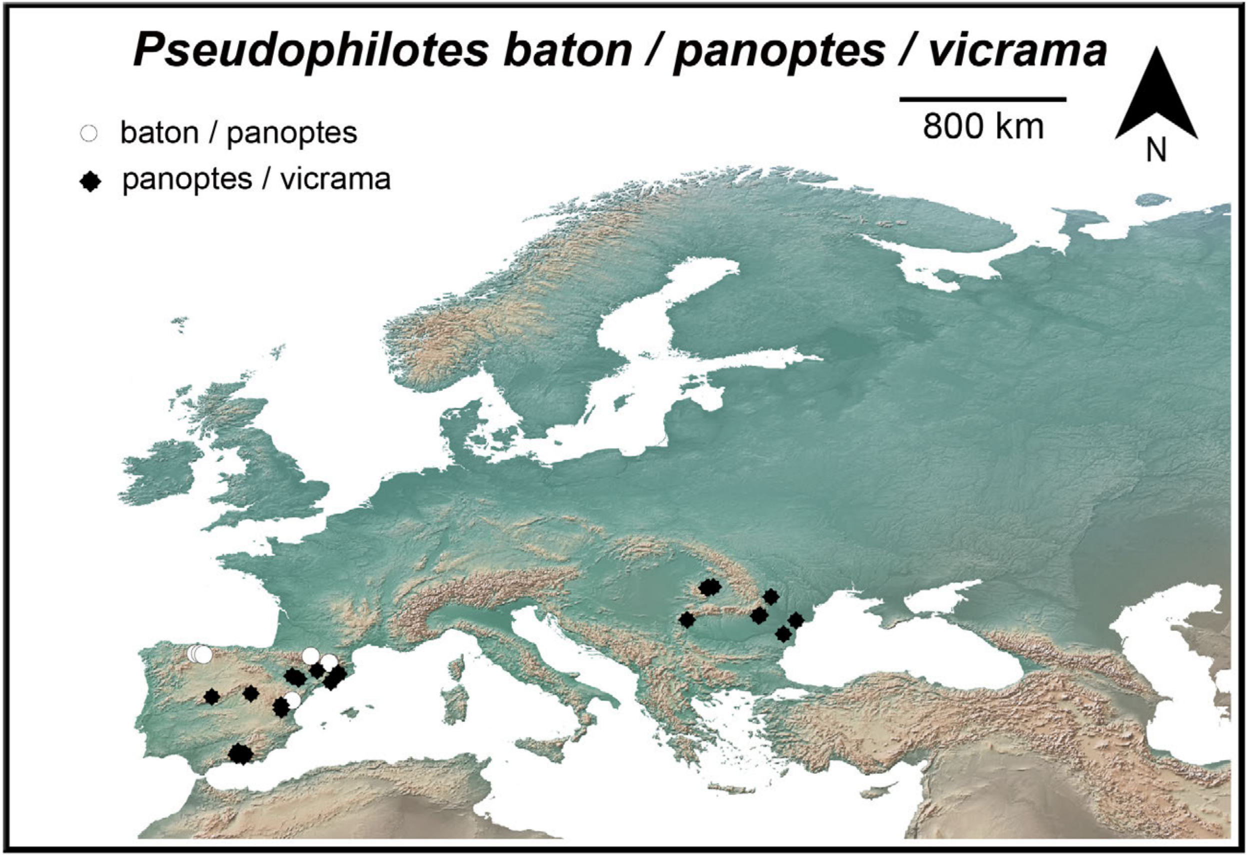
Pseudophilotes baton / panoptes / vicrama
800 km
N
baton / panoptes
panoptes / vicrama

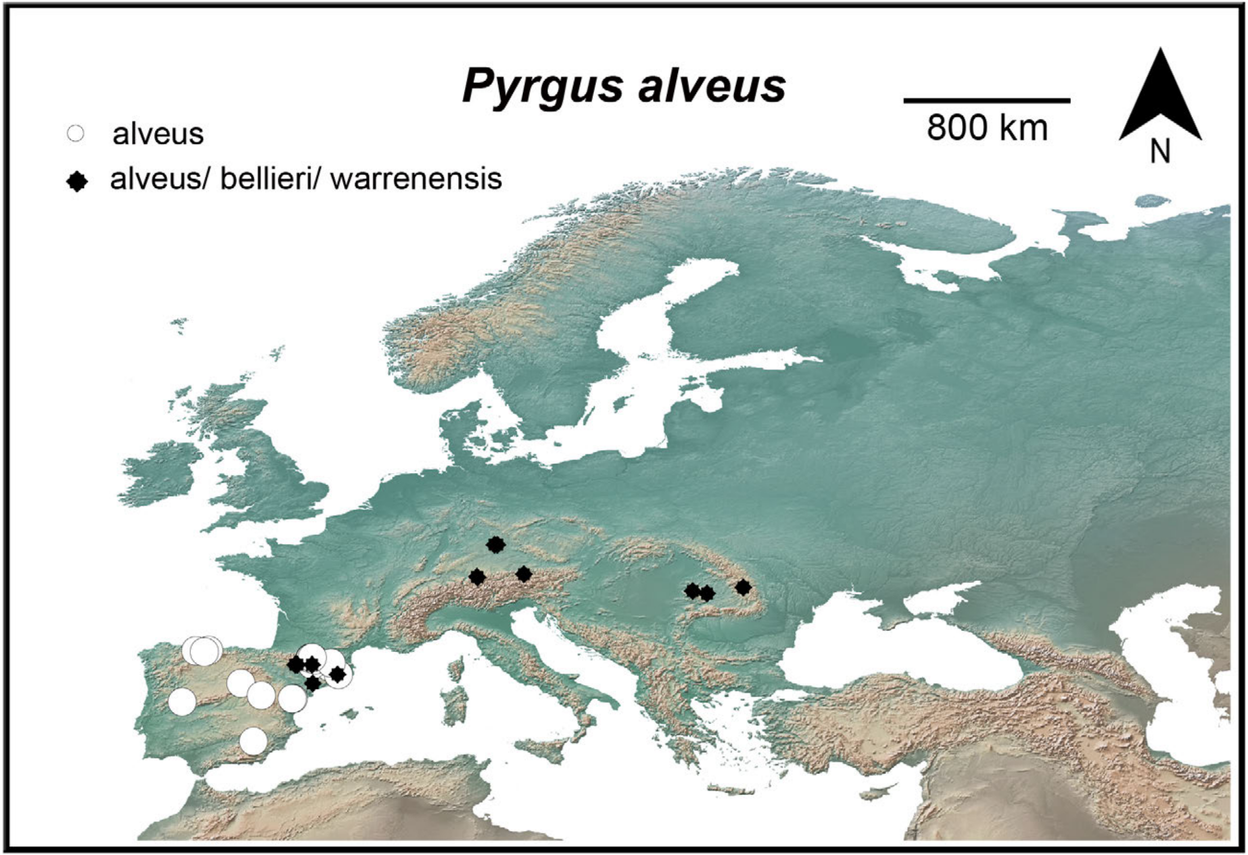
Pyrgus alveus
alveus
alveus/ bellieri/ warrenensis
800 km
N

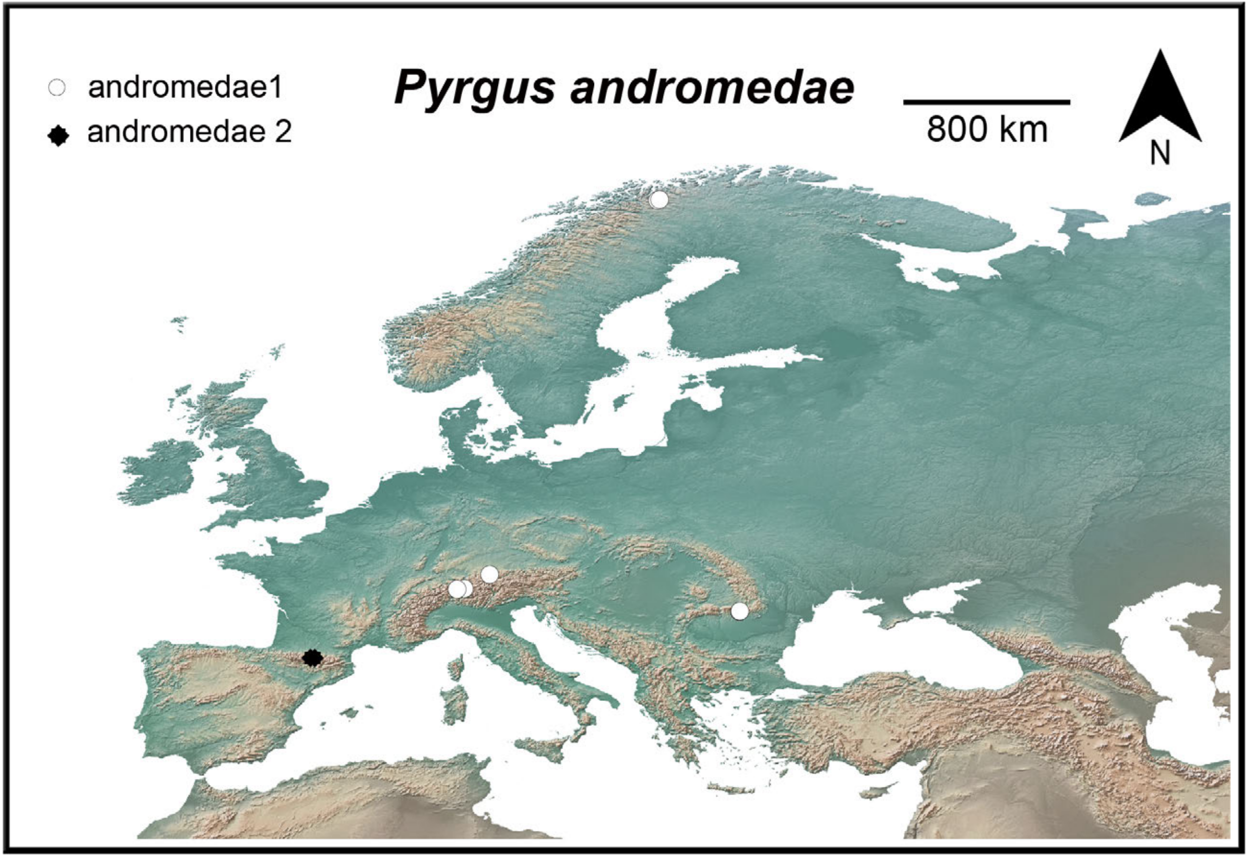
andromedae1
andromedae 2
Pyrgus andromedae
800 km
N

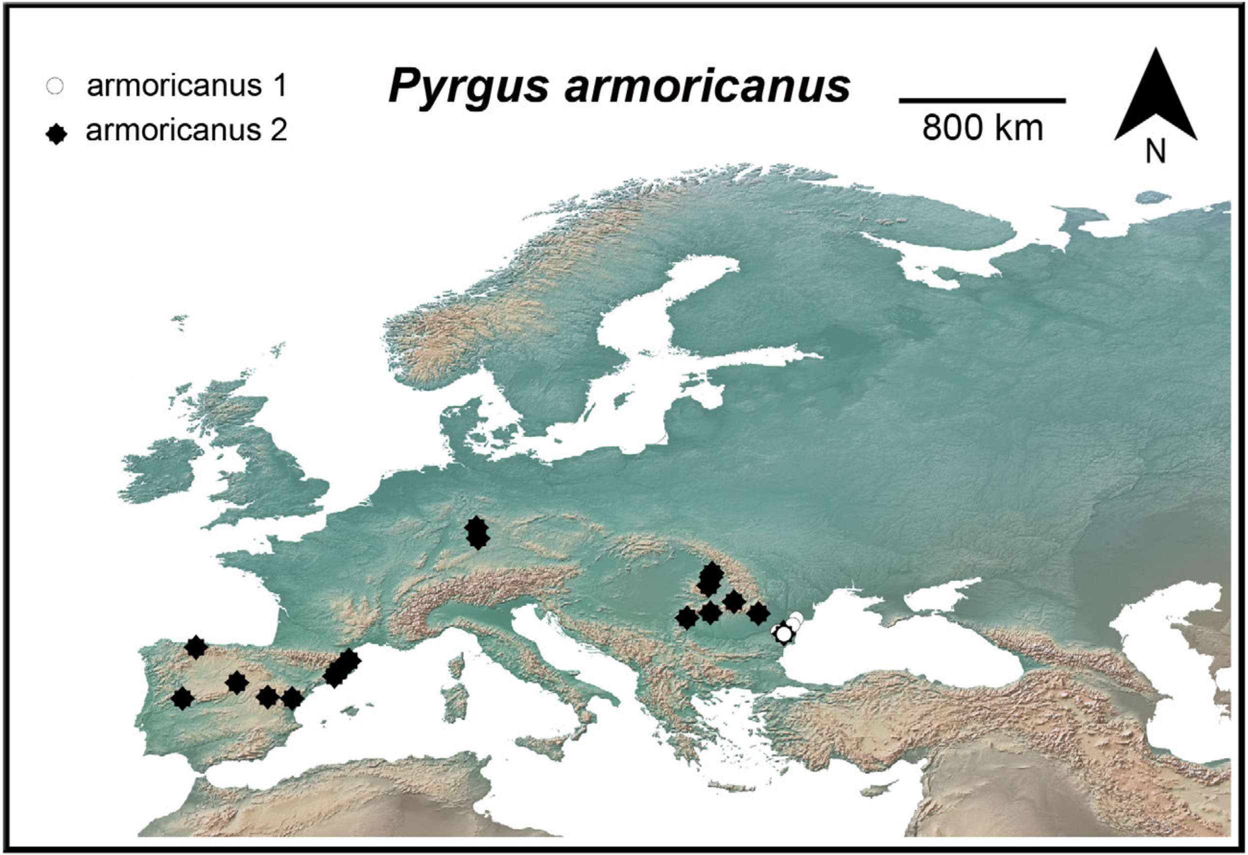
armoricanus 1
armoricanus 2
Pyrgus armoricanus
800 km
N

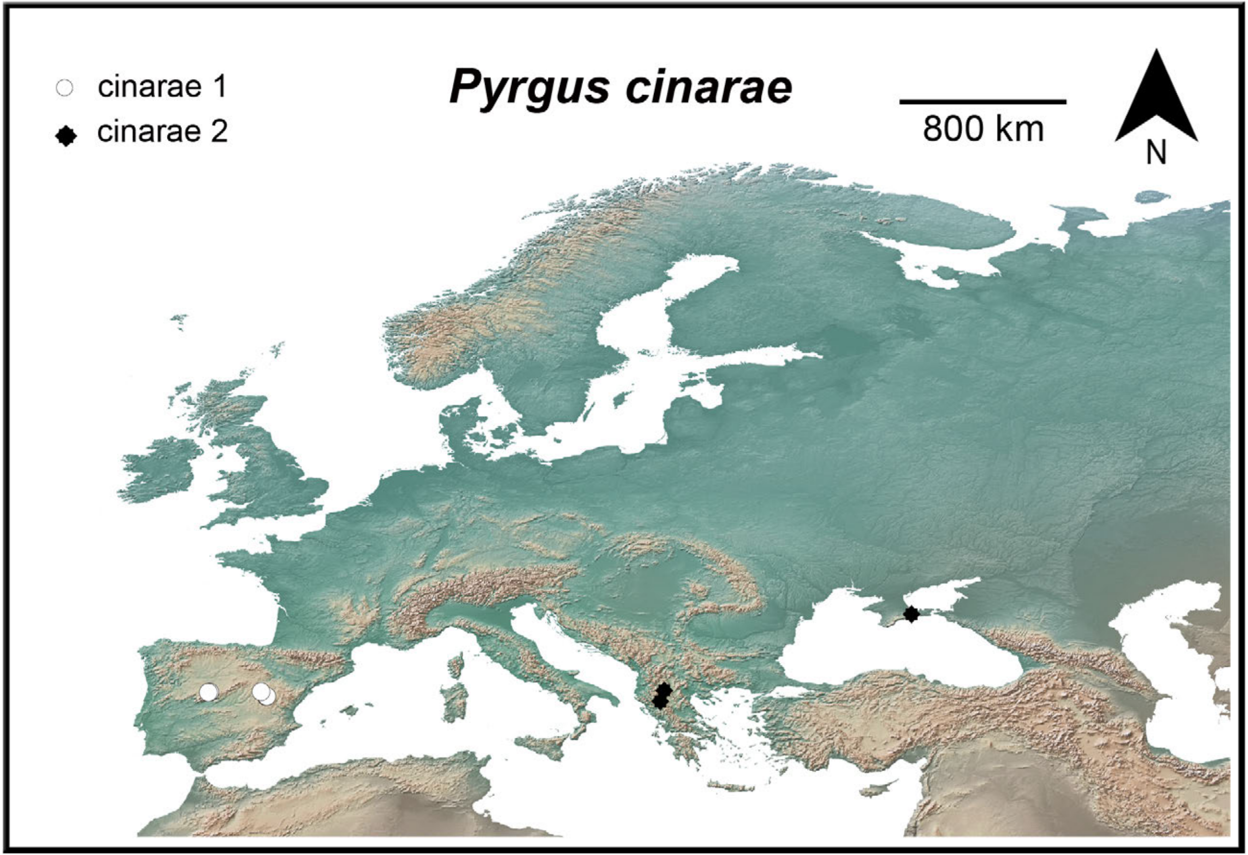

cinarae 1
cinarae 2
Pyrgus cinarae
800 km
N

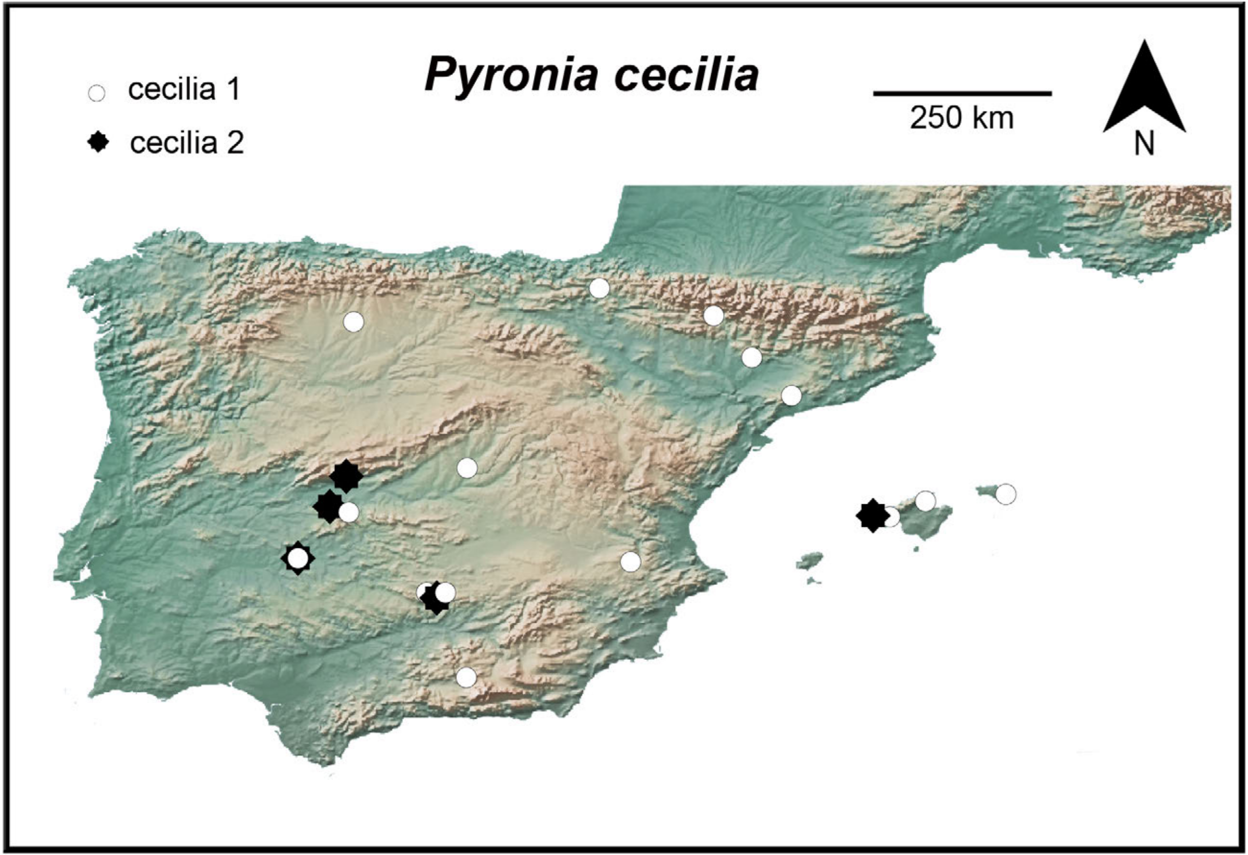
Pyronia cecilia
cecilia 1
cecilia 2
250 km
N

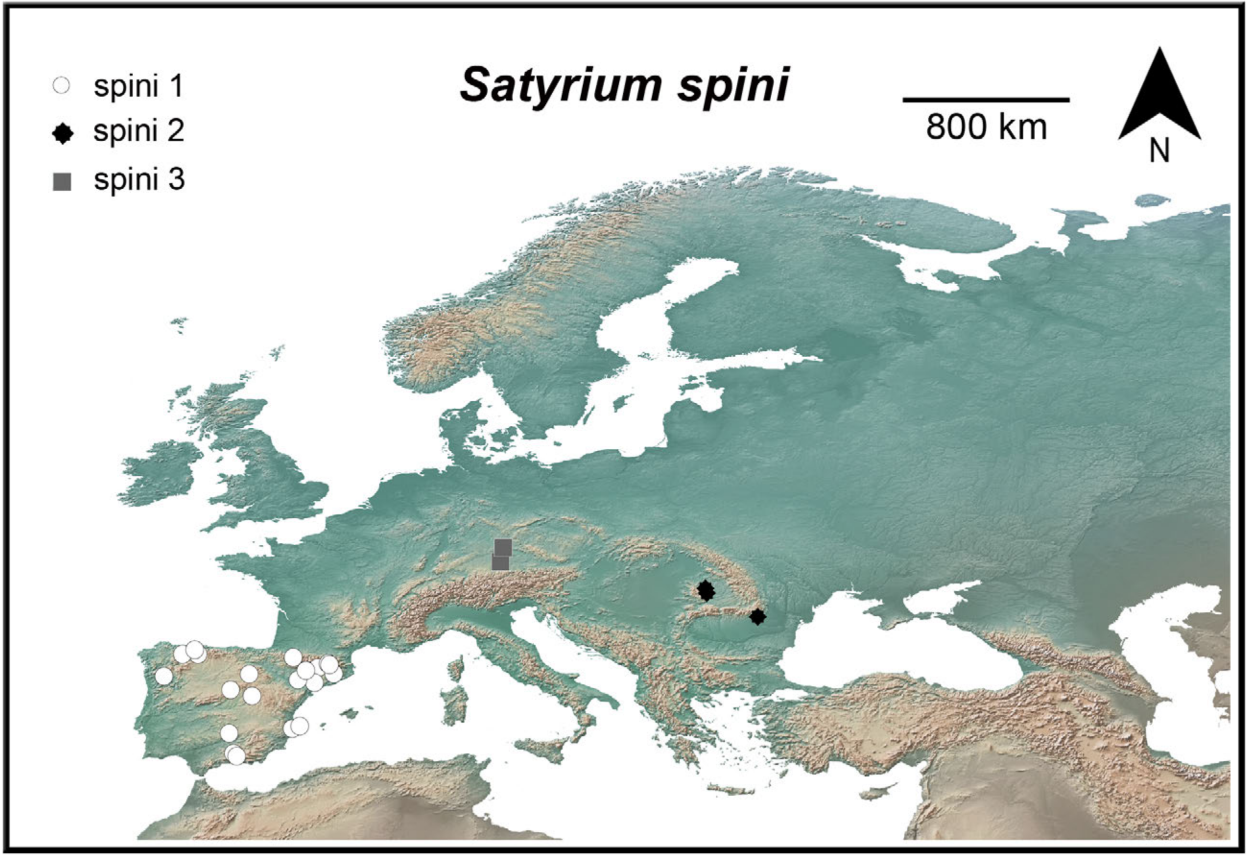
Satyrium spini
spini 1
spini 2
spini 3
800 km
N

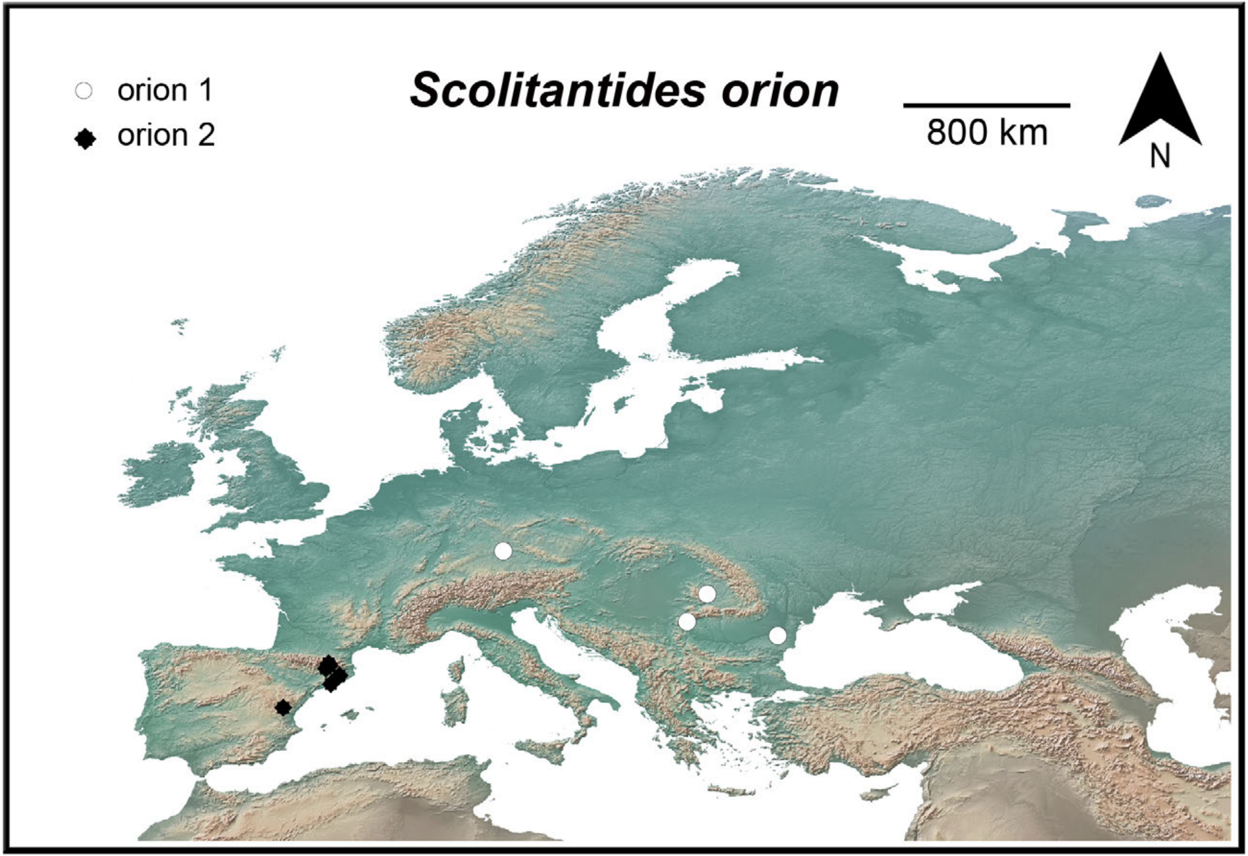
Scolitantides orion
orion 1
orion 2
800 km
N

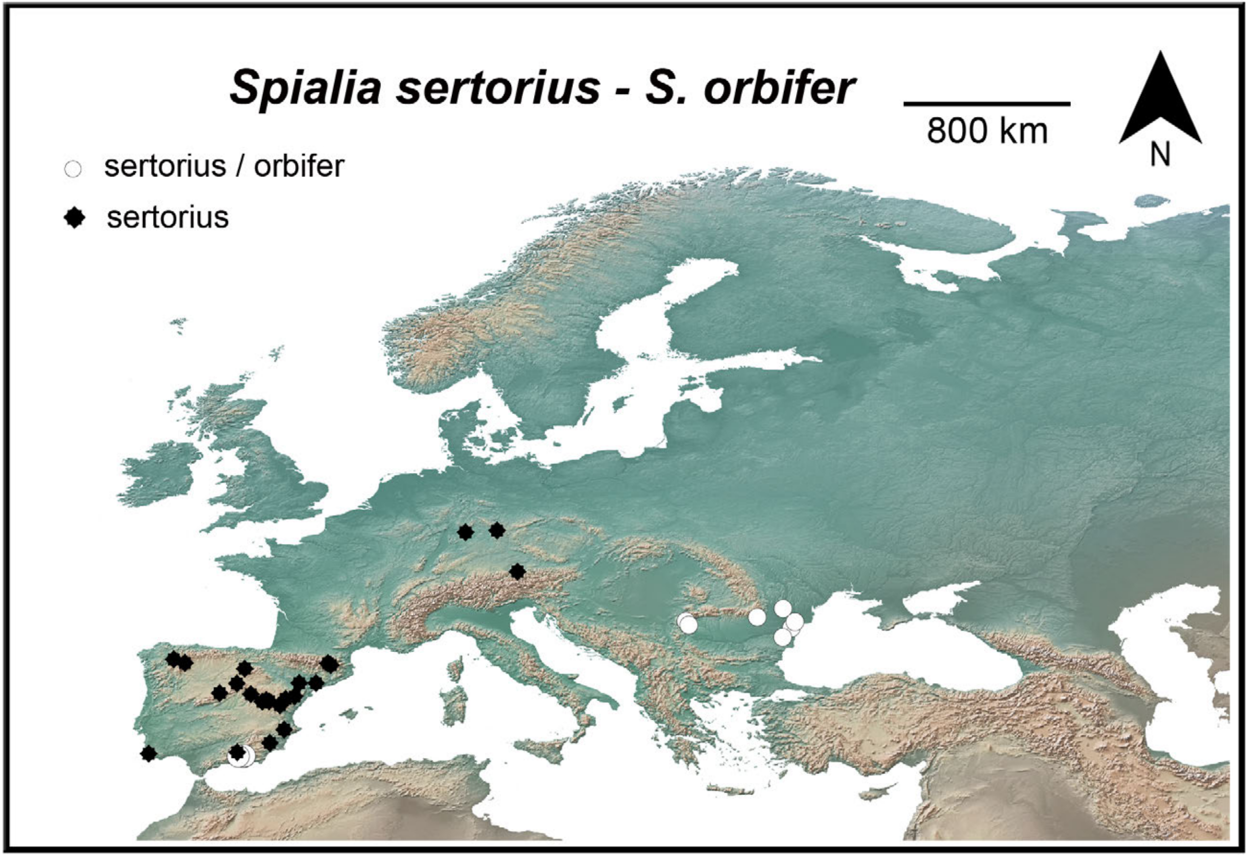
Spialia sertorius - S. orbifer
800 km
N
sertorius / orbifer
sertorius

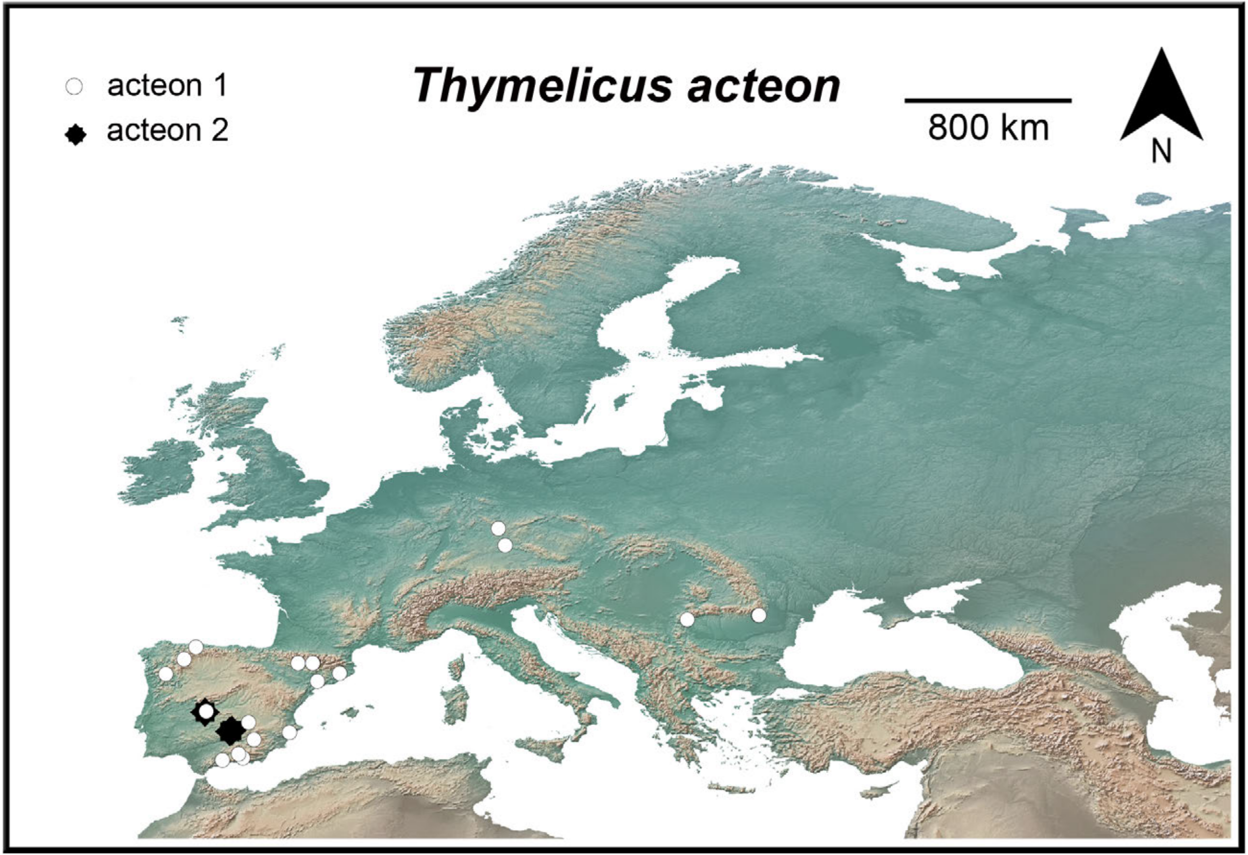

acteon 1
acteon 2
Thymelicus acteon
800 km
N

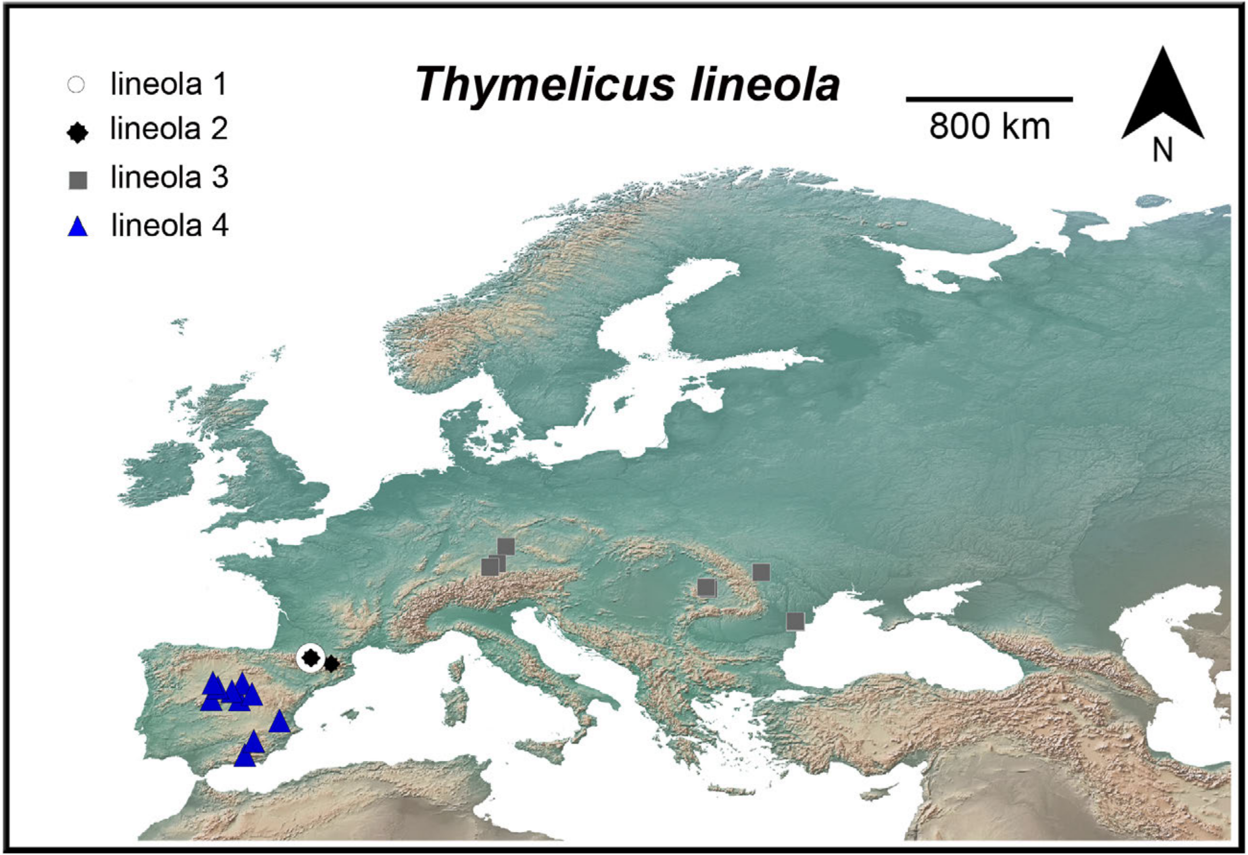
Thymelicus lineola
lineola 1
lineola 2
lineola 3
lineola 4
800 km
N

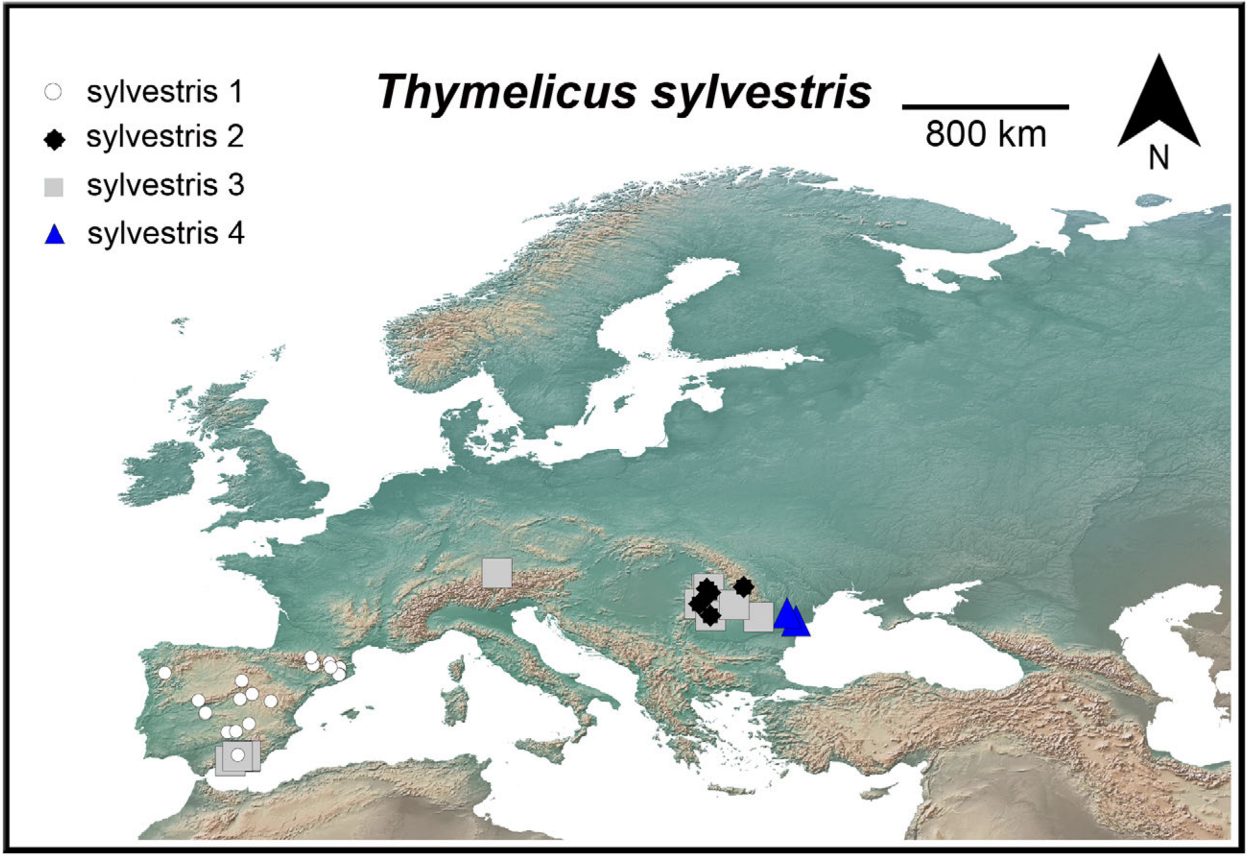

sylvestris 1
sylvestris 2
sylvestris 3
sylvestris 4
Thymelicus sylvestris
800 km
N

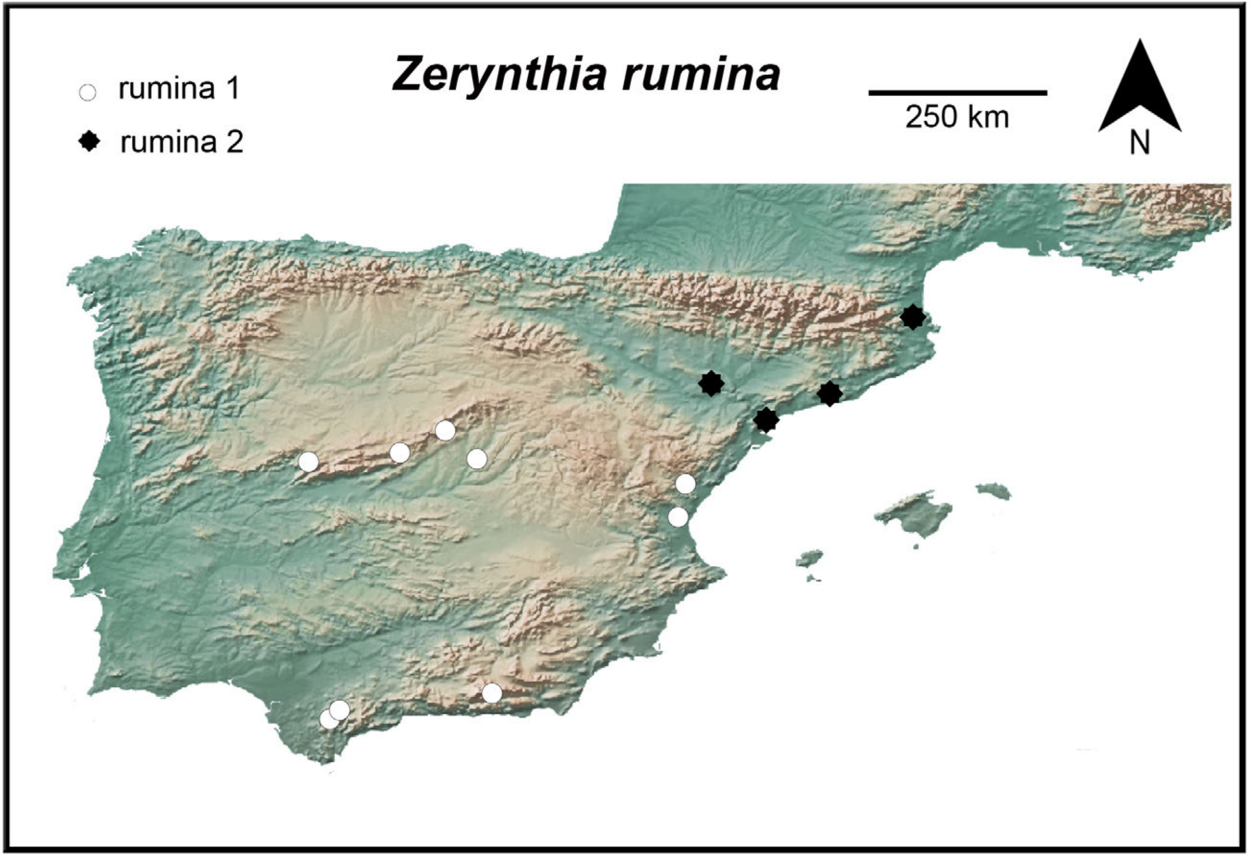
Zerynthia rumina
rumina 1
rumina 2
250 km
N
